# Tracking mutational semantics of SARS-CoV-2 genomes

**DOI:** 10.1038/s41598-022-20000-5

**Published:** 2022-09-20

**Authors:** Rohan Singh, Sunil Nagpal, Nishal K. Pinna, Sharmila S. Mande

**Affiliations:** 1 TCS Research, Tata Consultancy Services Ltd, Pune, 411013 India; 2 CSIR-Institute of Genomics and Integrative Biology (CSIR-IGIB), New Delhi, 110025 India; 3 Academy of Scientific and Innovative Research (AcSIR), Ghaziabad, 201002 India

**Keywords:** Data processing, Genome informatics, Machine learning

## Abstract

Natural language processing (NLP) algorithms process linguistic data in order to discover the associated word semantics and develop models that can describe or even predict the latent meanings of the data. The applications of NLP become multi-fold while dealing with dynamic or temporally evolving datasets (e.g., historical literature). Biological datasets of genome-sequences are interesting since they are sequential as well as dynamic. Here we describe how SARS-CoV-2 genomes and mutations thereof can be processed using fundamental algorithms in NLP to reveal the characteristics and evolution of the virus. We demonstrate applicability of NLP in not only probing the temporal mutational signatures through dynamic topic modelling, but also in tracing the mutation-associations through tracing of semantic drift in genomic mutation records. Our approach also yields promising results in unfolding the mutational relevance to patient health status, thereby identifying putative signatures linked to known/highly speculated mutations of concern.

## Introduction

Understanding genome sequences, an ordered collection of nucleotide bases constituting the genome, requires deciphering the rules governing the structure or positioning of the bases in the genome sequence. Human language has inherently been sequential in nature and is driven by the need for adding context (semantics) to the communication. The science of studying the rules of the human language, its grammar, semantics and more comes under the purview of linguistics^1^, and the use of Natural Language Processing (NLP) can automate this process by making computers explore, understand, learn, improve, generate, anticipate, and respond to this language^2^. It does so by leveraging the fields of computer science, machine learning/ artificial intelligence and mathematics towards the common goal of understanding the language of ordered datasets. Given the structural similarities between genomic and linguistic data records, it is pertinent to ask if NLP can be utilized to decipher the hidden meanings even from ‘Genome sequence data’.

The more we explore the depths of the biological world, the greater organization we witness in the perceived complexity of biological systems^2^. It is this ‘order’ or sequential nature of various biological data that has previously inspired the use of NLP in genomics^3^, metagenomics^4^, proteomics^5^ and more. In fact, DNA, the basic hereditary unit of life, is a classic example of a sequential dataset. The use of NLP to explore the latent information of genomes is therefore a rational proposition. It becomes further interesting when the biological data is dynamic in nature, like the evolving or mutating genomes. Dynamically changing biological datasets offer the opportunity to establish a parallel with another realm of computational linguistics, namely, dynamic topic modelling^6^ and diachronic analysis of literature^7,8^ or language corpora. In other words, an interesting question can be posed as to whether we can treat genomes as documents (especially temporally changing documents) in order to explore and understand the continuously evolving molecular signatures (equivalent to linguistic themes) in the vast corpus of biological features like genes or mutations (equivalent to linguistic words).

**Figure 1:**
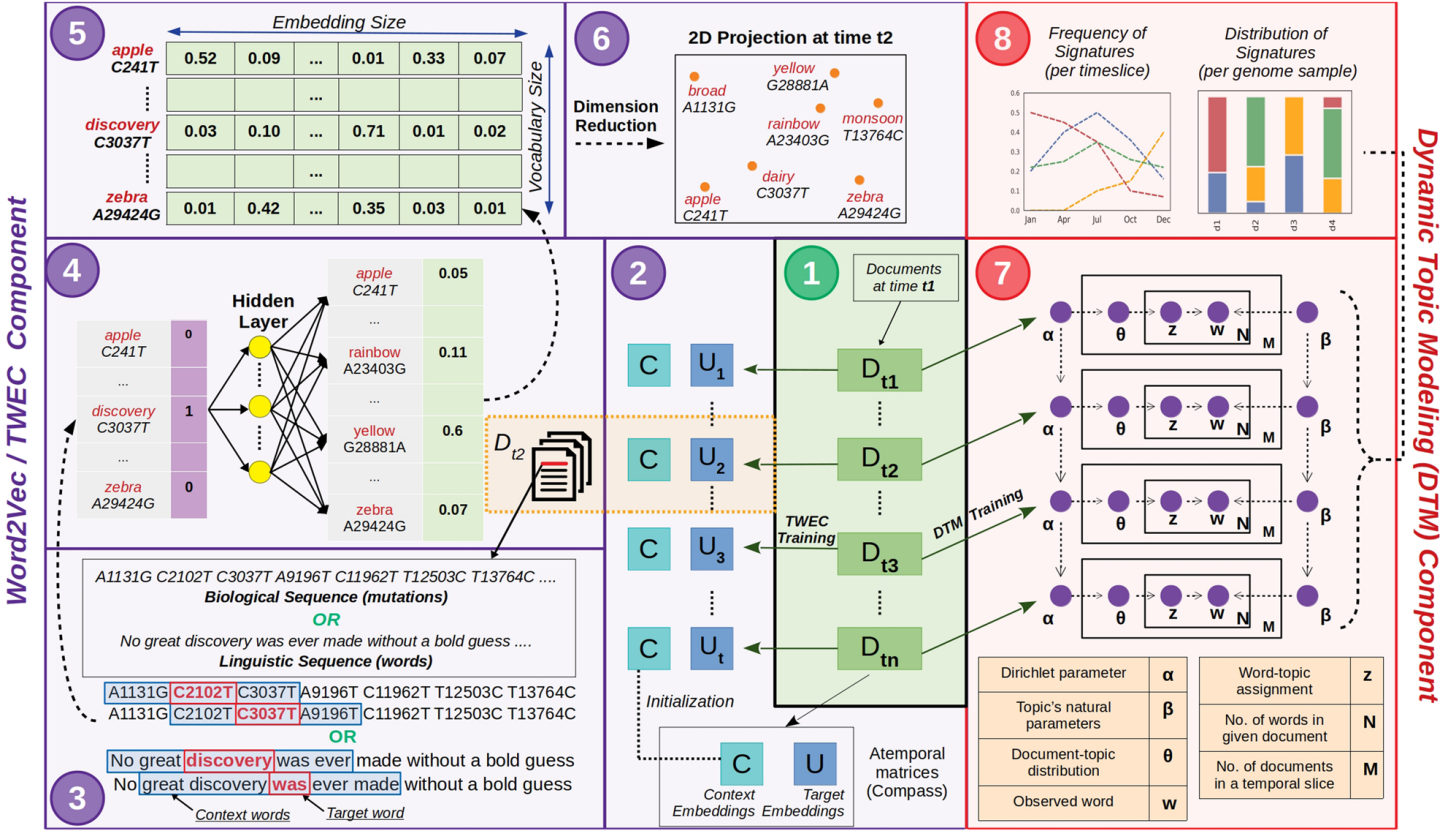
Graphical Summary of the entire NLP approach towards elucidating mutational signatures of genomic datasets (using SARS-CoV-2 genomes). Schematic representation of training procedure of both temporal natural language processing (NLP) approaches used in our study, i.e., Dynamic Topic Modeling (DTM) and Temporal Word Embeddings with a Compass (TWEC). Both techniques train on a collection of documents (corpus) that is split into respective time-slices. The panel description are as follows: (1) Splitting the corpus into respective time-slices: Documents (or genomes) at a particular time-slice, “tn”, is used to train DTM and TWEC models. (2) TWEC model training: Context embeddings are temporally fixed while the target embeddings are trained over each time-slice. (3) Words in each document/genome sample from time-slice t2 are iterated over sequentially. Window size (in blue) determines the span of neighbours for context consideration. (4) For each word present in time-slice t2, the embeddings are generated using Skipgram architecture of Word2vec. (5) Embedding matrix of the vocabulary (i.e., all words). (6) 2D projection of the embedding vectors generated for time-slice t2. (7) Dynamic Topic Modeling (DTM) training architecture. (8) Signature distributions are obtained for each time-slice and each document/sample.

Here, we highlight the applicability of NLP in capturing the temporal/diachronic trends in the evolution of genomic datasets. We demonstrate the same by perceiving SARS-CoV-2 genomes as documents and the associated mutations as the words of these documents. A large number of SARS-CoV-2 genome sequences are being deposited to public repositories like GISAID^9^ through an unprecedented spirit of scientific collaboration across the world. The high volume of raw data is expected to balloon further as the pandemic progresses. Each newly sequenced genome is a potential mutant/variant of the original reference genome, i.e., Wuhan/WIV04/2019 (EPI_ISL_402124). Understanding the diversity and evolution of these variants has been a subject of interest to a wide spectrum of researchers^10,11^. Various reports aimed at identification of clades or classification system(s) for these genomes have in fact been outcomes of the afore-mentioned problem statement^11^. Although conventional topic modelling has been utilized to obtain insights on COVID-19 from literature data, NLP can also be leveraged for understanding the signatures and diachronicity of SARS-CoV-2 mutational landscape. We demonstrate how the temporal word(mutation)-embeddings of SARS-CoV-2 nucleotide mutations can aid discovery of latent signatures (refer Supplementary File 1: Concepts of word embeddings, Word2Vec and DTM models, and Fig. 1 for graphical intuition of the same). The method enables the semantic characterization of the mutational landscape of SARS-CoV-2 and subsequent tracing of its progression with time. Such an approach could be helpful in the pathogenesis-tracking of COVID-19, especially when corroborated by leveraging the unsupervised classification of mutation vocabulary of the patient-status labelled SARS-CoV-2 genome corpora using *scattertext*^12^ (an NLP approach applied for learning severity linked mutations). This methodology could also be extended to capture meaningful biological information for not only SARS-CoV-2, but other biological datasets as well. Supplementary Table 1 provides a summary of the entire approach towards the design of this research study based on asking some pertinent questions through the perspective of natural language processing.

### Establishing parallels between documents and genomics data

In order to apply NLP routines for genomic data, we need to first relate the NLP terminologies that can be utilized for obtaining insights from genome sequence dataset (Table 1). A genome string containing mutational information in the form of position-specific nucleotide base-pair changes corresponding to an individual/organism is akin to a 'document', and each mutation is analogous to a 'word' in a document. The entire dataset derived from GISAID^9^ can be considered equivalent to the 'corpus', a complete set of documents. Unique nucleotide mutation set within the entire genome datasets can be considered equivalent to the vocabulary, which refers to the entire word set present in the corpus. Supplementary Fig. 1a displays the frequency of genome sample (i.e., document) and unique mutation counts observed in each time-slice (i.e., a month).

**Table 1:** Analogy between NLP terms used for documents and genomes.

S.No	Term	NLP Definition	Analogous Genomic Relation
1	Word/Term	A single element of a document	A nucleotide mutation
2	Document	A sequence of words	A genomic sample i.e., a list of nucleotide mutations from a sample
3	Corpus	Entire collection of documents	All genomes e.g., SARS-CoV-2 genomes from GISAID dataset (obtained till July 2021)
4	Topic	A set of words that relate to a subject/theme. A document may comprise of one or more topics	A signature (i.e., nucleotide mutation signature)
5	Context	The particular setting or pattern in which the word occurs, usually influencing its meaning or effect	The ordered genomic position of a nucleotide mutation
6	Vocabulary	Entire collection of unique words within the corpus	Unique nucleotide mutation set within the entire dataset

A nucleotide mutation signature is like a topic (comprised of several words) that carries a particular idea (mutations of concern). Technically, a topic is a probability distribution of its constitutive words, and as a result, documents with similar probabilities of such words are estimated to contain the topic(s) concerned. Like a document is composed of one or more topics, a genome sequence may contain more than one unique mutation signature, each having different probabilities. The inherent order in the occurrence/co-occurrence of genomic mutations is equivalent to the semantics/context of a document. A word’s meaning may differ with different texts/sentences as they are surrounded by different words (within a range of its neighbouring words called ‘window size’) and therefore influence semantics. Our study defined a ‘context’ as the genomic proximity of a mutation with other mutations, thereby establishing analogous relation between the conventional documents and genomes (Table 1).

## Results

### Discerning mutational signatures (topics) in the SARS-CoV-2 genomes (corpus)

**Figure 2:**
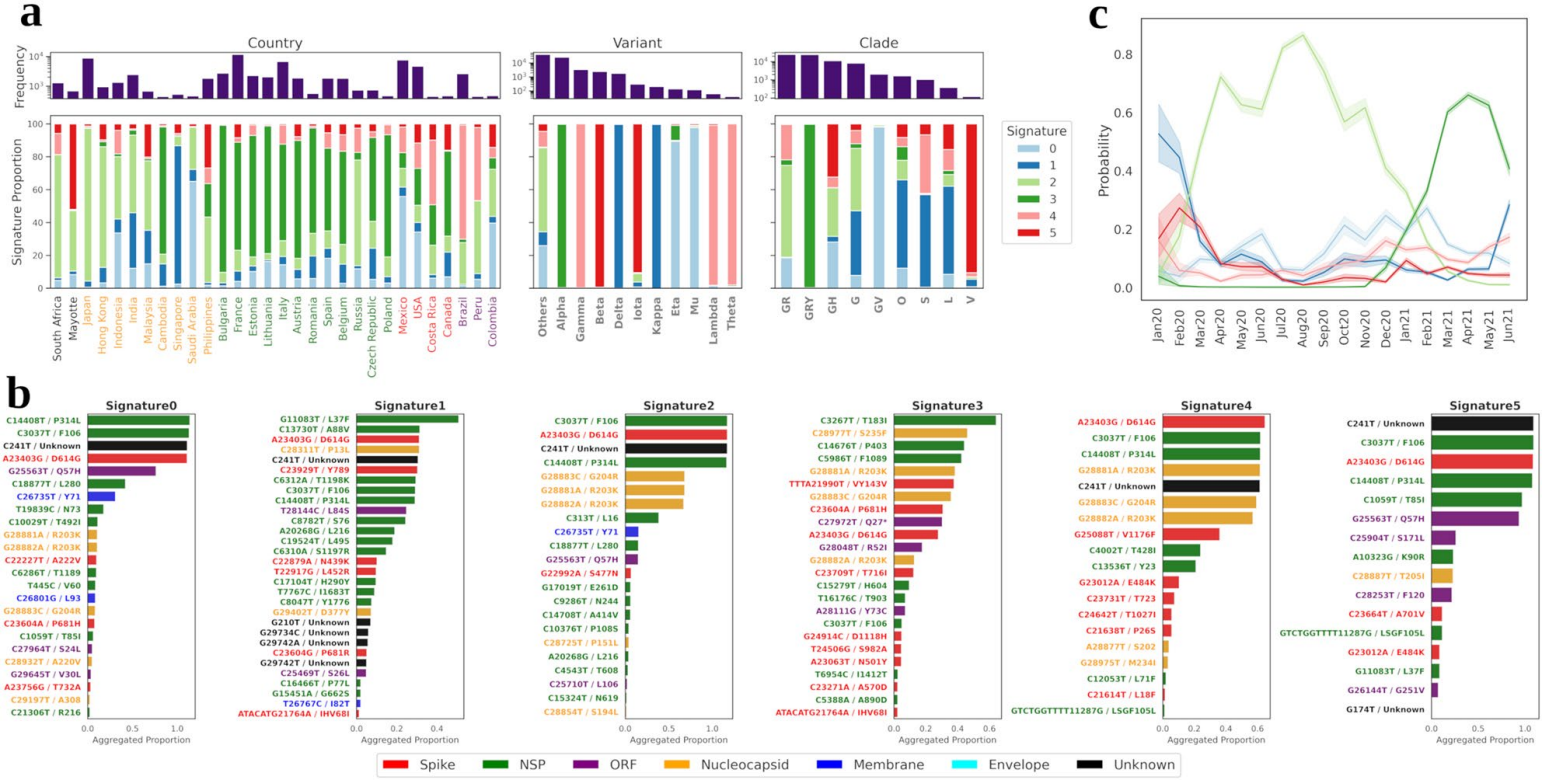
Distribution of mutational signature in SARS-CoV-2 genomes across geography (**a**) Stacked proportions of signatures (topics) in corpus segregated by 3 categories: country, SARS-Cov-2 variant and GISAID clade. The frequency of each constituent within each category are shown at the top of each stacked bar. Within the country subpanel, each country is differentially coloured according to respective continents. (**b**) Aggregated proportions of word probabilities in each signature across all time-slices. Nucleotide mutations coloured in black (as Unknown) are UTRs. (**c**) Temporal probabilities for each signature.

Six mutational signatures were inferred from the optimised model generated using Dynamic Topic Modeling (DTM) (Fig. 2). The geographical distribution of these signatures and their mapping to various SARS-CoV-2 variants or GISAID clades are represented in Fig. 2a. It was observed that the inferred signatures from DTM display strong coherence with the known ‘Variants of concern’ (VoC) and a moderate predilection towards geography. These included Signature0 that mapped to Eta and Mu variants; Signature1 that mapped to the Delta variant; Signature3 (Alpha); Signature4 (Gamma, Lambda, Theta) and Signature5 (Beta). Signature2 did not map to any known VoC. The composite mutations for each of these signatures are represented in Fig. 2b. Notably, Signature3 (Alpha) seemed to be topically dominant in majority of the genomes collected from European nations, and it is known that the Alpha variant had a widespread impact in European cohorts^12,13^.

Similarly, other observed geographical associations indicated Signature2 to have certain prominence among Asian countries, and Signature4 to be proportionally higher among South American countries. Certain countries showed single signature prominence. These included Signature2 for Japan, Signature3 for Cambodia, Bulgaria, Signature1 for Singapore, Signature0 for Saudi Arabia and Signature4 for Brazil. Several Southeast Asian countries were affected by different SARS-CoV-2 strains, and our DTM model displayed nearly similar VoC proportions for Cambodia, Indonesia and Malaysia, in line with that reported by Chookajorn et al. 2021^14^, using the GISAID dataset.

A temporal tracing of the inferred signatures is shown in Fig. 2c. The mean probabilities of different signatures for genomes corresponding to different time-slices are plotted with a confidence interval of 0.99 (highlights the shaded area around each line). While Signature2 showed the highest probability during 2020, Signature3 (Alpha) started to manifest towards the end of 2020. As described in the ‘Discussion’ section, these trends align well not only with geography specific observations reported elsewhere, but also with the phylogeny/clinical information linked variant classifications.

Furthermore, we probed the temporal progression of the top 20 most probable mutations within each signature (Supplementary Fig. 2). All these signatures comprised of mutations with very high initial probabilities that declined over time. Although two signatures, namely, Signature3 and Signature5, had no or very few abrupt losses among their topmost probable mutations, their corresponding mutation probabilities evened out over time. However, for the rest, new emergent mutations could be distinctly observed, which included Signatures 0,1 and 2, with few older mutations fading out with time. Not all signatures were found to have every mutation unique to itself. Certain mutations like those of codon G28881A, G28882A, G28883C could be seen to encompass several signatures, albeit with declining probabilities. The biological inferences/implications of DTM (and other) results are attempted in the ‘Discussion’ section.

### Temporal divergence of SARS-CoV-2 mutations

**Figure 3:**
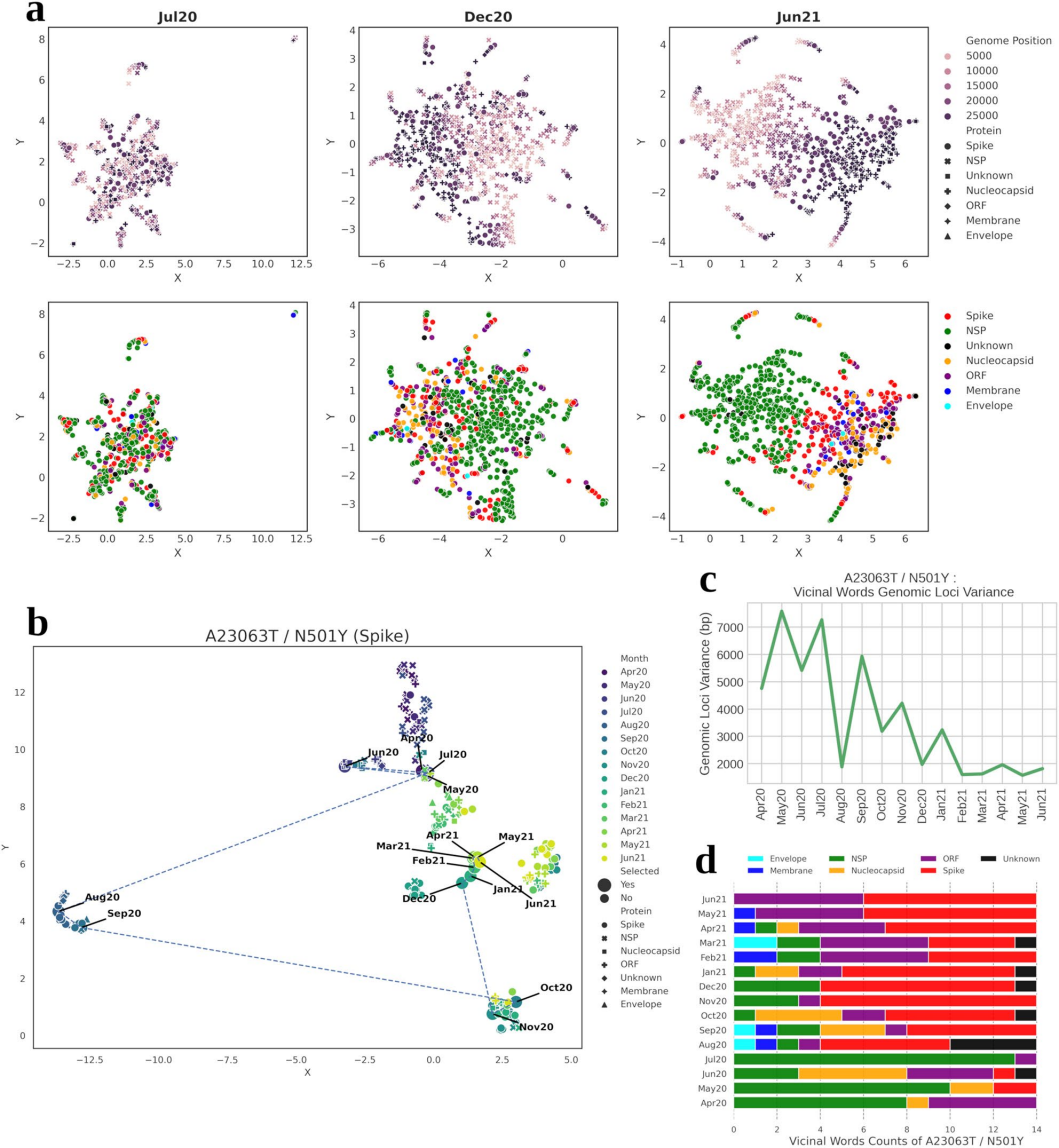
Semantic drift of mutation A23063T/N501Y (Spike). (**a**) UMAP projection of word embeddings of 1000 most frequent mutations in the corpus (i.e., GISAID dataset) in 3 time-slices. Most mutations tend to disperse by their genomic position. (**b**) Tracking semantic drifts of mutation of concern A23063T/N501Y from Apr2020 to Jun2021, represented by the dotted line. Other points refer to the 15 most semantically related mutations in each time-slice. (**c**) Tracking temporal shifts in the standard deviation of genomic loci of neighbouring mutations to A23063T in each time-slice (**d**) Protein classification of semantically closest words (neighbour) to A23063T in each time-slice.

To get a general overview of the collective divergence of SARS-CoV-2 mutations across time, we examined the embedding vectors (representing meaning of a word in dynamic/continuous vector space) in three time-slices with 6-month gaps in between them (Jul2020, Dec2020 and Jun2021). 2D Uniform Manifold Approximation and Projection (UMAP)^15^ of the embedding vectors corresponding to these time points were plotted for the top 1000 most frequent mutations in the corpus. These mutations appeared to converge with other mutations that shared genomic proximity, i.e., those in neighbouring genomic loci or mutations belonging to the same protein (Fig. 3a). In Jul2020 (Fig. 3a), the word embeddings of most frequent mutations (top 1000) were seen to be quite indistinct. This was expected as most of these mutations either had none or smaller frequencies at the beginning of 2020 and started to manifest as the pandemic spread globally. By Dec2020 (Fig. 3a), the embeddings evolved enough to gain contextual segregation which became more prominent by Jun2021 (Fig. 3a). For instance, from Dec2020 onwards, NSP (Non-structured Protein) related mutations were observed to cluster distinctly when their embeddings were projected.

### Semantic drift of individual mutations of concern (MoC)

To closely examine how the semantics of a single mutation shifted temporally, four mutations of concern (MoC) found in the spike protein (as reported in outbreak.info^16^), namely A23063T/N501Y, C23604A/P681H, C23604G/P681R and T22917G/L452R, were selected.

The initial embedding vectors of A23063T/N501Y mutation during the Apr2020 to Jul2020 period, remained unchanged and hence clustered closely (Fig. 3b). The embedding vectors in the months of 2021 remained relatively unchanged with high similarity of genomic context as seen by lower genomic loci variance (*see Methods for description*), shown in Fig. 3c, as compared to time period between Apr2020 to Jul2020, where the neighbouring words (mutations) genomic loci variance were observed to be higher. Roughly half of the neighbouring mutations pertained to spike protein, which appeared post Nov2020. However, from Feb2021, its neighbouring mutations also constituted some from ORF protein. Given that the genomic loci variance has been quite low (Fig. 3c) since Jan2021 and some neighbouring mutations originated from the ORF protein for these months, it showcases that the word embeddings were able to capture the adjacency of ORF protein mutations to this spike protein mutation). Figure 3d displays the neighbouring word composition of A23063T/N501Y for each time-slice. This MoC also displayed many non-contextual neighbours (i.e., top 15 most semantically related mutations), which were found to be mostly non-spike mutations in those initial months (as seen in Fig. 3d). But in the months of Aug2020 and Oct2020, the embeddings changed in meaning (i.e., they experienced semantic drift), which stabilised in Dec2020 thereafter (Fig. 3b). It is also evident that with the progression of COVID pandemic, the neighbouring mutations comprised fewer mutations on NSP proteins, which are genomically distant to spike mutations. Supplementary Fig. 3 shows 2D projection of word embeddings of neighbouring mutations of A23063T/N501Y in Jun2021.

As with A23063T mutation, the embedding vector of C23604A/P681H mutation only gained momentum after Jul20 (Supplementary Fig. 4a), after which its context steadily drifted till Dec2020, post which the drift momentum as well as genomic loci variance (Supplementary Fig. 4d) ebbed and became stabilised from Mar2021 onwards. Interestingly, its mutation neighbours from Feb2021 to Apr2021 (Supplementary Fig. 4b) lacked those occurring in spike protein but rather had mutations from majorly nucleocapsid and ORF protein and even envelope and membrane protein.

C23604G/P681R mutation and its counterpart mutation ‘C23604A’ shared a similar semantic transition (Supplementary Fig. 5a). Although, its semantics during initial periods remained unaltered till Jul2020, a small shift was observed in Aug2020, which again reverted to its previous quarter’s embedding cluster. The maximum drift in semantic change was observed in Nov2020 and Dec2020, post which embeddings were found to be stabilised. The genomic loci variance dropped drastically from 2021 onwards (Supplementary Fig. 5d), reaching the lowest at Apr2021, post which neighbouring mutations comprised majorly from ORF and spike proteins (Supplementary Fig. 5b).

T22917G/L452R mutation’s semantics made a sudden movement in Jun2020 but reverted close to the previous months’ embedding cluster (Supplementary Fig. 6a). Some drastic changes were seen in Sep2020, with neighbouring mutations being of non-spike origin, indicating non-contextual connotation (as seen by many cross markers surrounding it) (Supplementary Fig. 6b). Some shifts were also observed in Nov2020, wherein the neighbouring mutations were found to be related to spike protein. After Dec2020, the embeddings stabilised in neighbourhood of mutations occurring mostly in spike protein, thereby displaying lower positional variance (Supplementary Fig. 6d).

In all the above-mentioned mutations of concern, one could notice that with the progression of COVID epidemic, the neighbouring mutations comprised fewer mutations on NSP proteins, which are genomically distant to spike mutations.

### Identifying infection severity specific mutations

**Figure 4:**
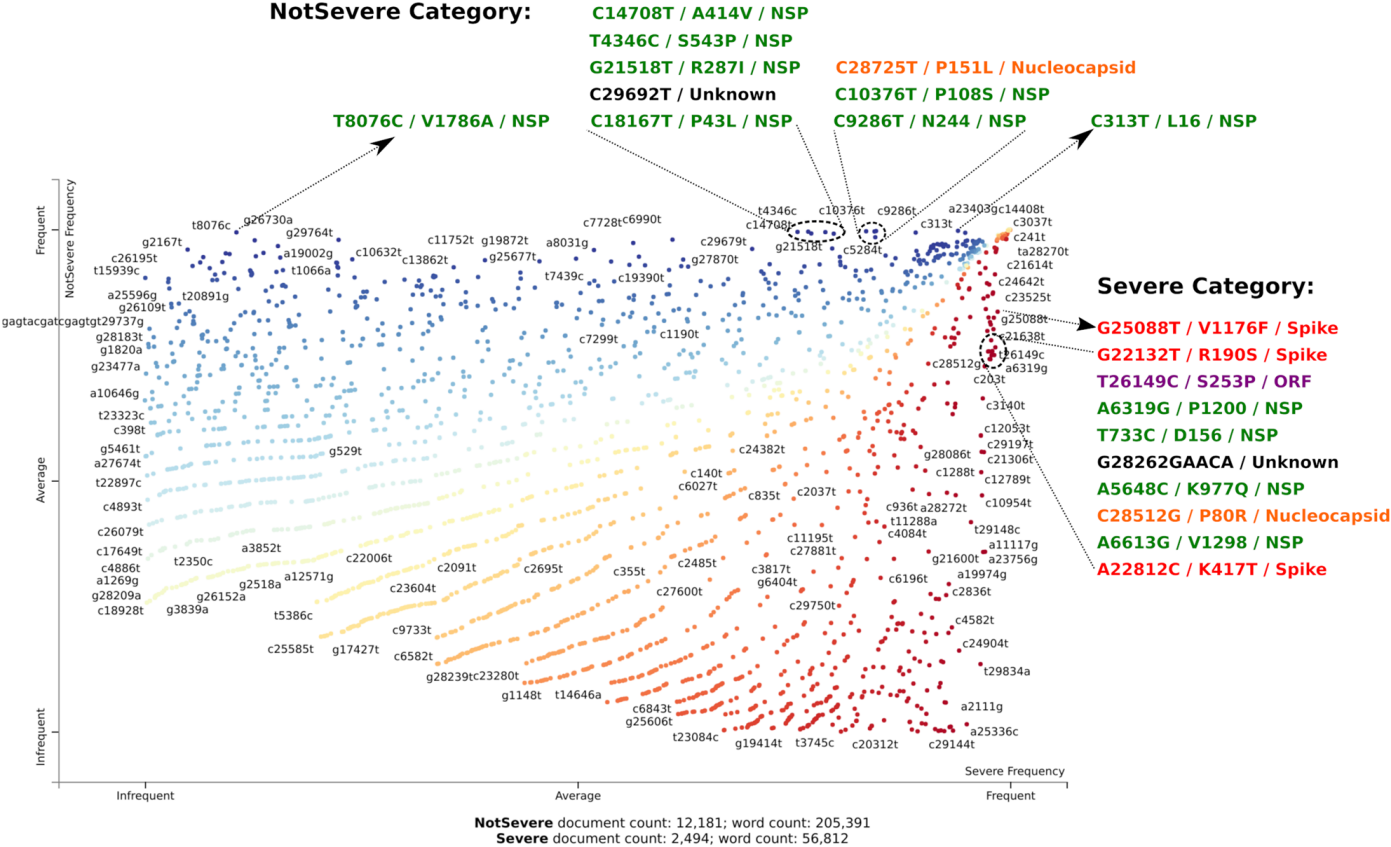
Visualising infection severity association of mutations. The figure shows the distribution of SARS-CoV-2 mutations in corpus along 'Severe' or 'NotSevere' frequency gradient in X and Y-axis, respectively. Points highlight mutations and are coloured according to binary classification based on F-scaled scores (see methods under Severity classification of Patient Health Status). Note that nucleotide mutations coloured in black (as Unknown) are on UTR.

NLP can also be employed on labelled datasets to classify text corpora and to identify driving words/topics of such labelled documents^3,17^. With the aim of identifying the severity specific mutations, a total of 12,182 non-severe (‘Asymptomatic’, ‘Mild’, ‘Moderate’) infection specific (labelled) genomes and 2,497 severe (‘Critical’, ’Severe’, ’Fatal’) infection causing genomes were employed for the classification of latent-mutation signatures using *scattertext*^12^. Figure 4 displays the scatterplot of frequency of ~ 2000 mutations within ‘Severe’ and ‘NotSevere’ categories. The highlighted mutations within the figure correspond to the top ten characteristic mutations, whose likelihood of occurrences were observed to be higher in one category (i.e. Severe) as compared to that in another category (i.e. NotSevere), as determined via F-scaled scores (*see methods for more details*). While most of the characteristic mutations in ‘NotSevere’ category belonged to NSP related mutations, the top ten characteristic mutations in ‘Severe’ category were found on several proteins. Among these, three corresponded to mutations on spike protein (G25088T/V1176F, G22132T/R190S, A22812C/K417T), one on nucleocapsid protein (C28512G/P80R), one on putatively non-genic mutation (G28262GAACA) and the remaining were found to be NSP linked mutations. Points in the top right corner in Fig. 4 represented the most frequent mutations (having high frequency) in both categories.

### Temporal clustering of infection severity specific mutations

**Figure 5:**
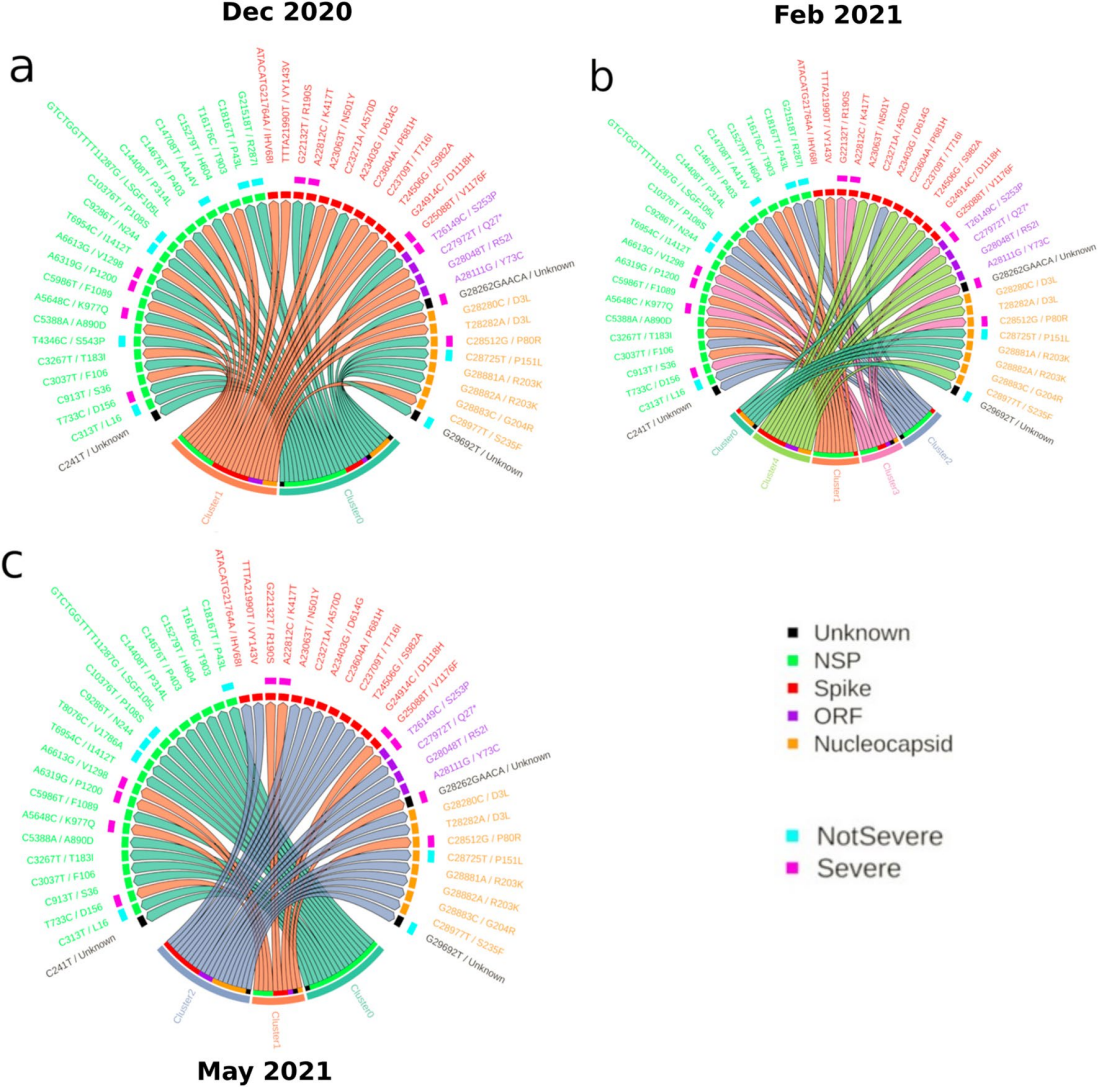
Semantic clustering of Severe, NotSevere and most frequent mutations. Panels (**a,b,c**) show chord diagrams of k-means clustering of 50 mutations (10 ‘Severe', 10 ‘NotSevere' and 30 most frequent mutations) in time-slices Dec2020, Feb2021 and May2021, respectively. Note that nucleotide mutations coloured in black (as Unknown) are on UTRs.

To illustrate differences in temporal contextual changes in mutations associated with a physiological label (i.e., Severe or NotSevere classification), ten characteristic mutations in both categories as well as top 30 most frequent mutations (as a neutral set) were selected for relative comparison. The mutations within the neutral set were observed to be relatively ubiquitous and non-characteristic to both infection severity categories and hence they were considered as non-markers. By semantically comparing to this neutral set, we intended to differentiate how the derived/putative characteristic mutations converge/diverge away from a neutral pool of mutations. K-means clustering was performed to observe the formation of mutation grouping in different time-slices. Cluster tracking of the afore-mentioned mutations (Fig. 5) indicated varied numbers of clusters in three time-slices, namely, Dec2020, Feb2021 and May2021. Since a few mutations from the NotSevere category were observed to be absent in some of these time-slices, their embeddings were ignored while plotting for the respective time-slices. Figure 5a–c depict chord diagrams highlighting mutations to the respective k-means cluster (refer Methods for further details). Supplementary Fig. 7a–c show 2D UMAP dimension reduction plots of the embedding vectors for the three time periods of these selected mutations.

In Dec2020 (Fig. 5a and Supplementary Fig. 7a), the characteristic mutations of both patient health status categories (Severe and NotSevere) were observed to cluster into one group, and the majority of the most frequent mutations were found to be clubbed into another cluster. This indicated that the semantic differences were not very prominent between mutations of both patient health status categories. In contrast, segregation of mutations was observed into numerous clusters in Feb2021 (Fig. 5b and Supplementary Fig. 7b), wherein k-means clustering differentiated both Severe/NotSevere associated mutations into their respective clusters. However, for ‘NotSevere’ category mutations, the cluster set was not fully exclusive. Furthermore, the segregation of most frequent mutations into their respective protein of origin were found especially for NSP, spike and nucleocapsid proteins. In May2021 (shown in Fig. 5c and Supplementary Fig. 7c), the trend was however found to be contrasting. Unlike the subclusters seen in the Feb2021 time-slice, fewer cluster numbers were observed. In this timepoint, the NSP mutations from NotSevere category had coalesced into a separate cluster that comprised of NSP mutations from the neutral set. Cluster 1 was solely comprised of severe-category mutations. These inferences suggest that the ‘Severe’ and ‘NotSevere’ mutations are semantically different as well. The biological implications of the observations have been further described in the ‘Discussion’ section.

### Productivity and Frequency of Mutations of Concern (MoC) and severity associated mutations

**Figure 6:**
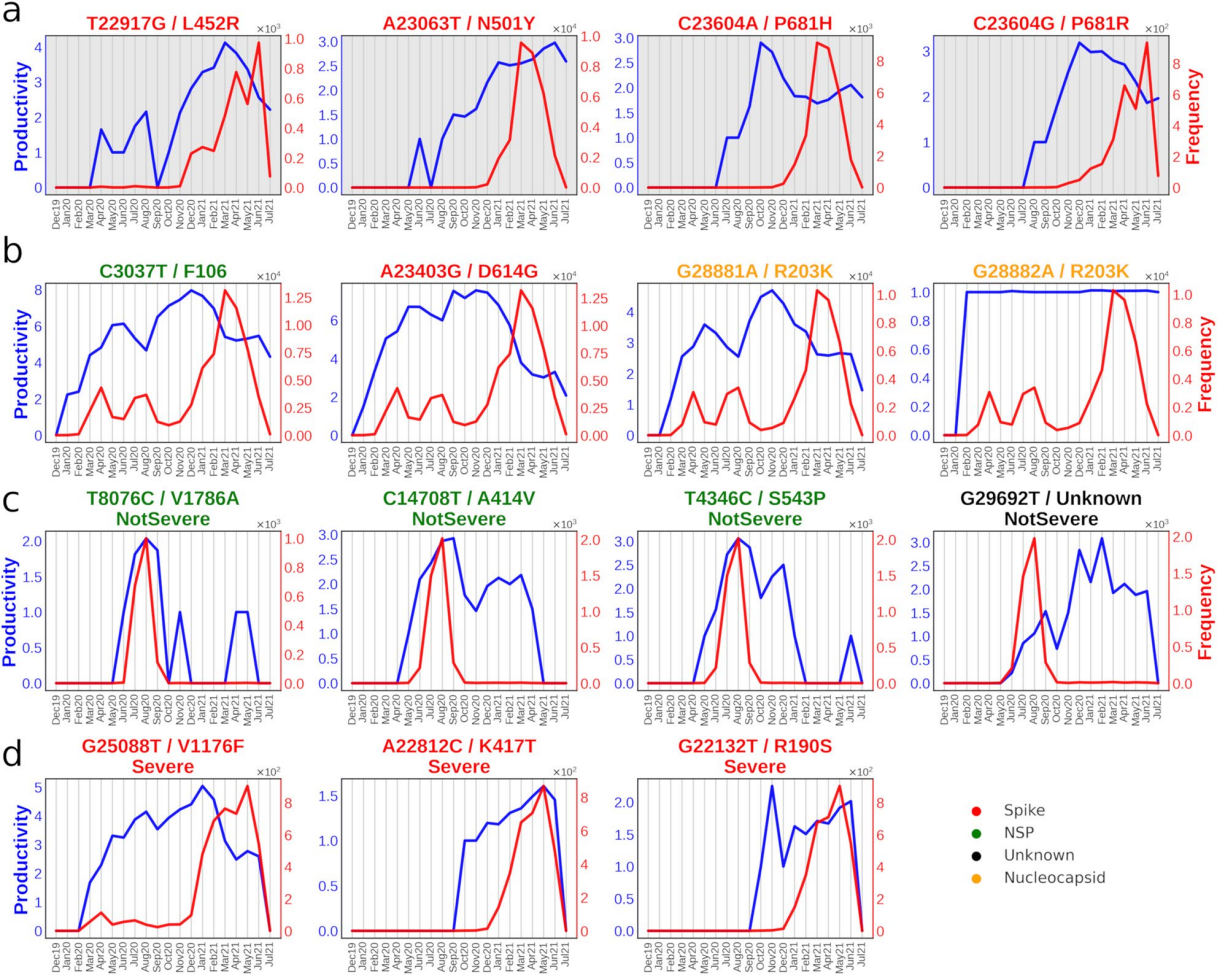
Temporal Frequency and Productivity of selected 15 mutations. Red and blue lines indicate frequency and productivity (tendency to form multi-word associations) of mutations. (**a)** comprises four mutations of concern (MoC). (**b)** comprises of four most frequent mutations in the corpus. (**c)** and (**d)** panels comprise of mutations of the 'NotSevere' and 'Severe' categories, respectively. Note that nucleotide mutations coloured in black (as Unknown) are on UTRs.

Figure 6 depicts the *productivity* (tendency to form multi-word associations) and *frequency* for the four mutations of concern (Fig. 6a), along with the top four most frequent mutations in the corpus being C3037T, A23403G, G28881A and G28882A (Fig. 6b).; top 4 characteristic mutations of ‘NotSevere’ patient health category (Fig. 6c); and top 3 characteristic spike mutations of ‘Severe’ category (Fig. 6d). C3037T is a prevalent synonymous mutation providing no known evolutionary advantage for the virus. A23403G/D614G has been speculated to increase viral infectivity and reduce spike shedding^18^. G28881A and G28882A are consecutive mutations that have previously been speculated to affect the molecular flexibility of N protein^18^.

While frequency represents the occurrence of mutations (terms) with time, productivity highlights the associability of the mutations (terms) to other mutations. Therefore, the productivity plot pattern of a term may be non-identical to its corresponding frequency plot, as a term might associate more/less to other terms irrespective of its own recurrence with time. A noticeable difference could be seen for ‘NotSevere’ mutations (Fig. 6c), wherein their frequencies were observed to drop drastically after their peak in summer 2020. Their productivity showed retention of peaks during late 2020, post which it dropped subsequently. These therefore belonged to ‘declining terms’ wherein both frequency and productivity had reduced.

Contrastingly, the frequency and productivity of ‘severe’ mutations were seen to attain a peak in 2021 itself. Frequency overlap could be observed between the top four most frequent mutations, which indicated how prevalent these mutations were during the COVID pandemic and were also native to most geographies and/or lineages. Interestingly, the productivity of G28882A/R203K mutation became stagnant, which indicated that it created no new associations but solely paired with its codon partners (G28881A/R203K).

Comparison of the productivity and frequency of the mutations of concern (MoC) to their corresponding semantic drifts (Fig. 3b–d and Supplementary Fig. 4–6) indicated that the frequency pattern didn’t relate to how the semantics of the mutations changed. However, the productivity provided following additional hints to its semantic progression.

1. *A23063T/ Spike-N501Y:* Just like in semantic drift, the productivity and frequency were found to be relatively low/unchanged during the initial months, while the productivity was then seen to steadily increase post Aug2020, the frequency had a sharp rise in Jan2021 (Fig. 6a) and then substantially dropped after Apr2021. This sudden drop in frequency along with sustained rise in productivity may indicate that the fewer cases that were reported for SARS-CoV-2 infections that contained A23063T/ Spike-N501Y mutation still acquired new mutations for A23063T to associate with.
2. *C23604A/ Spike-P681H:* Just like with other mutations of concern, this mutation’s productivity started around mid-2020 and kept a steady rise till productivity = 3 towards the end of the year, which approximately coincided with the drifting pattern displayed in semantic drift. But unlike A23063T, its productivity decreased from Nov2020 onwards to productivity = 2, just when its frequency started to pick up rapidly. This peculiar observation may be due to its consistency in finding the same set of partners to which it associates. Jan2021 was found to be the month when it increased sharply for unnormalized frequency.
3. *C23604G/ Spike-P681R:* Its productivity pattern was found to be similar to its counterpart mutation, C23604A. However, its decline in productivity was not seen to be as sharp and sudden when compared with C23604A. Also, C23604G lagged in frequency rise, wherein it started to make a sharp increment after Jan2021.
4. *T22917G/ Spike-L452R:* Here, the productivity pattern was observed to start much sooner than the rest of the mutations of concern. Despite low frequency during the time-slices of Mar2020 to Aug2020, it displayed a certain rise in productivity. A sudden collapse was observed in Sep2020, after which it garnered high productivity, which steadily rose to a value of four in Mar2021 and then steadily declined thereafter. Its frequency curve indicated a slight rise in Dec2020, but a much higher rise was after Mar2021.

### Acceleration of A23063T with associated mutations

**Figure 7:**
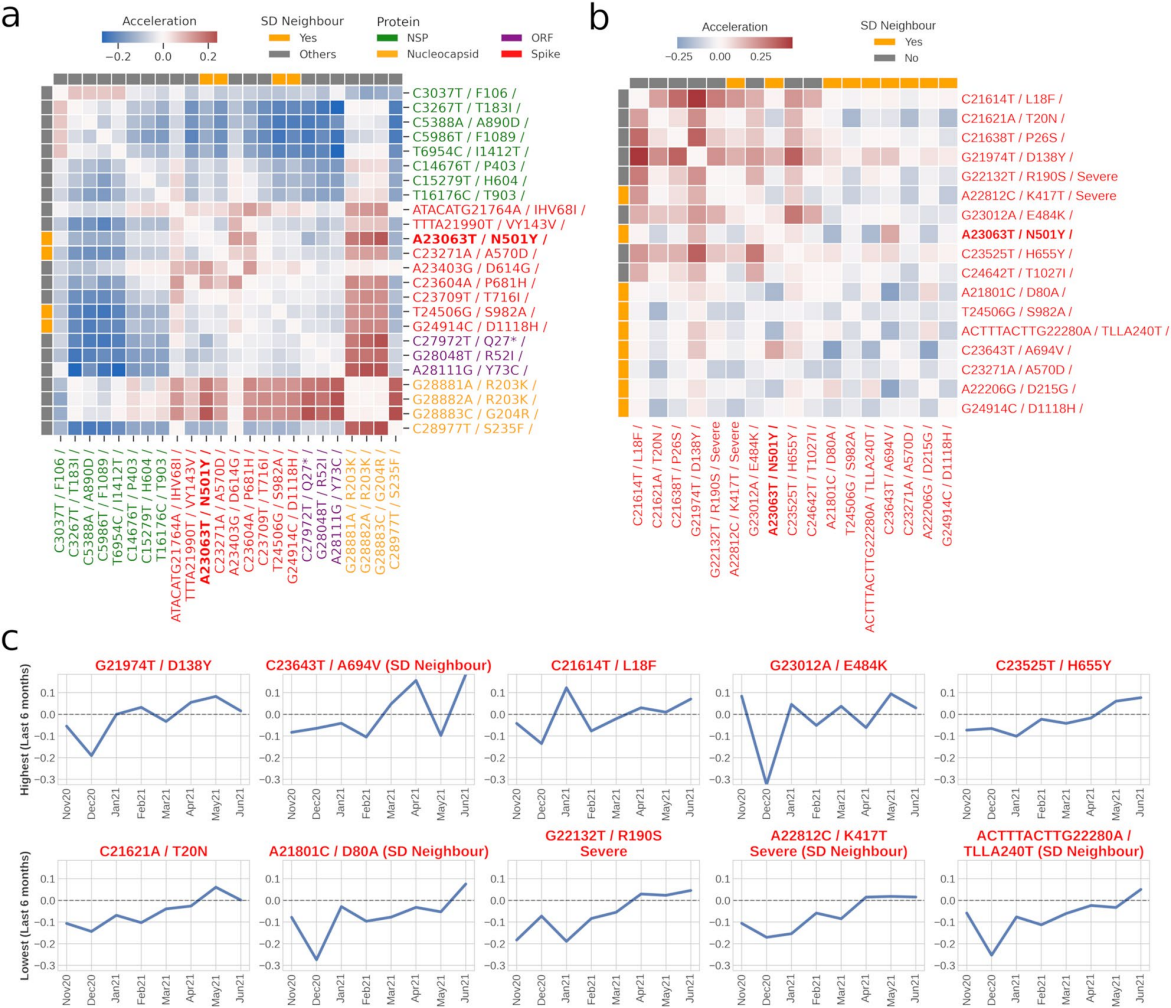
Semantic Acceleration of A23063T with associated mutations. (**a)** Heatmap of semantic accelerations between Jan2021 and Jun2021 among mutations identified from signature3 of the dynamic topic model. This signature consisted of mutation of concern A23063T. 'SD neighbour' refers to mutations that were most recurrent as neighbouring mutations of A23063T from Oct2020 to Jun2021. (**b)** Heatmap of semantic accelerations between Jan2021 and Jun2021 among key mutations of Lineage P.1^15^ and nine SD neighbours of A23063T. (**c)** Dynamic acceleration tracking of ten mutations from panel (**b**) against A23063T. The top row highlights five mutations with the highest sum of accelerations between Jan2021 to Jun2021. The bottom row highlights five mutations with the lowest sum of acceleration between Jan2021 to Jun2021.

Acceleration compares the similarity between two terms (mutations in our case) and determines their level of convergence or divergence between two-time points. To exemplify the temporal drifts occurring between mutations belonging to the same signature set (i.e., a DTM topic), the accelerations of Signature3 mutations between two time-slices (Jan2021 to Jun2021) were plotted (Fig. 7a). Interestingly, three mutations within this signature set were found to be semantic drift neighbours of A23063T/Spike-N501Y’ mutation (referred to as ‘SD neighbour’). These neighbours refer to mutations that were most recurrent as neighbouring mutations of A23063T from Oct2020 to Jun2021 (when productivity of A23063T started increasing rapidly).

Broadly, while the mutations of NSP protein (coloured in green) had diverged from mutations on other proteins, the mutations of nucleocapsid protein (coloured in yellow) displayed a certain level of positive acceleration with non-nucleocapsid mutations (Fig. 7a). Most of the mutations on the spike protein remained largely unchanged relative to each other. It was also observed that many mutations alongside their neighbours had zero acceleration (as shown by white patches) with respect to each other. This may highlight their strong affinity to remain clustered within that duration. These mutations included the consecutive (codon linked) mutations (G28881A/N-R203K, G28882A/N-R203K, G28883C/N-G204R), all of which did not diverge (i.e., zero acceleration), which also supported the observation of productivity = 1 for G28882A/R203K (Fig. 6b). Literature evidence also indicated that these mutations were rarely described separately^18^.

For demonstrating key mutations associated with one MoC, namely, A23063T/Spike-N501Y, we selected mutations of Lineage P.1^10^ (Gamma VoC) and the neighbouring mutations derived from A23063T’s semantic drift and plotted their pairwise acceleration among themselves from Jan2021 to Jul2021 (Fig. 7b). Almost zero acceleration for neighbouring mutations (in orange bars) derived from the semantic drift of A23063T were observed. In other words, they did not accelerate or had diverged from a semantic point of view. However, a stronger level of acceleration (seen as red cells in the heatmap) were observed among mutations of Gamma variant (highlighted in grey). These could be prominently observed between several spike mutations, such as C21614T/L18F and G21974T/D138Y. C21614T/L18F notably had previously been reported for its impact on neutralizing some antibodies^19^ and hence contributing to the immune escape of SARS-CoV-2 mutant. G21974T/D138Y is found to be located on the N-terminal domain of S1 subunit of spike protein and has however not been observed to be reported extensively in the literature for the evidence of its significance. Interestingly, mutations T24506G/S982A, C23271A/A570D and G24914C/D1118H, which were found to be semantic drift neighbours of the MoC A23063T/Spike-N501Y (as shown in Fig. 7b) were also first detected in Alpha variant (B.1.1.7 UK variant), same as that of the MoC^20^.

Figure 7c shows acceleration changes of ‘A23063T’ with the neighbouring mutations derived from A23063T’s semantic drift as well as mutations from the Lineage P.1^10^. Acceleration at each time-slice indicated a change in the word (mutation) embedding in comparison to the previous time-slice/month. The top row in Fig. 7c highlights mutations whose sum of acceleration over the previous six months was the highest (from Jan2021 to Jun2021). From these graphs, the time-slices where the mutation pair came closer or diverged semantically could be specifically identified. For example, while C23643T/A694V had accelerated positively in March and April 2021 and mutation C21614T/L18F had accelerated in Jan2021; mutation G23012A/E484K diverged largely in Dec2020. In contrast, the mutations with the lowest sum of accelerations (between Jan2021 to Jun2021) with MoC ‘A23063T’, were found initially to have a slightly diverging trend, but with time the trend approached zero acceleration (i.e., unchanging semantics).

## Discussion and conclusion

Dynamic topic modeling (DTM) approach employed in our study can potentially aid evolution informed viral classification. When associated with a parameter of a labelled dataset (such as disease severity and geography), it can assist in the identification of mutations and their temporal prominence as key drivers of epidemiological or biological events (Fig. 2). While static topic modeling is a resourceful unsupervised method for identifying signatures, it does not however provide diachronic insights into the frequency of any signatures. Hence, DTM provides additional depth by showcasing how a variant evolves and what mutations drive its evolution (Fig. 2).

The signatures, identified through an NLP approach like DTM, although may not necessarily have an apparent phylogenetic relationship, they hold significance from an underlying linguistic/semantic point of view. In other words, here the collective mutation corpus and the context thereof directs the classification rather than genomic variants branching from phylogenetic clustering. Thus, the DTM methodology is a biology agnostic model which potentially has the capability of finding biological signatures. For instance, the Alpha variant, which was initially documented in the United Kingdom in Sep2020, became an epidemiological concern with rising cases across Europe by late 2020^12,13^. The same has been captured quite effectively by the DTM model (Fig. 2a). Other geography-specific observations can be seen for the Gamma variant which was first documented in Brazil^21^. Similarly, the Delta variant, which was reported in India (discernible proportion) and Singapore, was also captured^21^. Capturing such trends, in an unsupervised manner, without prior clinical information, hints towards the potential suitability of such NLP-related approaches in assisting the discovery/tracing of VoCs and MoCs.

Mutation peculiarity across signatures/variants can also be tracked and ascertained via linguistic approaches. In our study, use of TWEC model offered a neoteric approach to infer not just the mutation-mutation relationship but also their evolution (Fig. 3 and Supplementary Figs. 4–6). To showcase its applicability, we focussed on semantic changes in mutations of concern (MoC) by considering them as words in a document. One prominent mutation of concern, namely A23063T/Spike-N501Y, has been purported to increase transmission by variants that harbour this mutation^22^. The mutations on this site have been implicated in conferring dynamic stability to spike protein^23^ It is predominantly higher in Alpha and Beta variants, both of which became major concern around Dec2020^22,24^. Figure 6 shows the frequency and productivity of a few key mutations analysed in our study. We could observe that the emergent high frequency of A23063T was clearly apparent from Dec2020 onwards. However, the increment in productivity predated that of frequency, indicating that it prevailed prior to becoming a part of concerning variants, where its productivity peak emerged from Aug2020 onwards. Our observations from Fig. 3d indicated that before Aug2020, the neighbouring mutations were mostly of non-spike origin, possibly owing to the initialised random word (mutation) vectors due to smaller mutation vocabulary in early time-slices.

Another aspect in our study pertains to utilising labelled patient status annotation of a document’s (i.e., genome sample) affiliation to a binary category, ‘Severe’ and ‘Non-Severe’. With the help of F-scaled scores computed using the *scattertext* package, we were able to classify certain mutations that affiliate to these two classes. The top 10 mutations for each class are depicted in Fig. 4. Examples of delineated ‘Severe’ mutation were G25088T (corresponding to V1176F of spike protein) and T26149C (corresponding to S253P of ORF3 protein), which have also been previously reported as mortality linked mutations^25^. Two other spike mutations of the ‘Severe’ category, namely G22132T (R190S) and A22812C (K417T), have been reported earlier to be key members of Lineage P.1^10^. Lysine417’s role in interaction with ACE2 receptor for infectivity obliquely substantiates our severity association, although further investigation would be needed to justify this linkage^10,26^. Hence, this category-linked linguistic method can potentially provide useful perspectives related to that class, which in our case related to disease severity and mutations, which can be corroborated further from a structural point of view. From viral evolution perspective, several mutations within SARS-CoV-2 appear to be positively selected^27^ . Some of the most prominent ones, A23403G/D614G, G28882A/N-R203K and G28883C/N-G204R, were also found to be high frequency nonsynonymous mutations (Fig. 6b) and appeared in multiple signatures of DTM model (Fig. 2b).

In the present study, we showcase NLP-driven, biologically unconventional methods for trailing the evolutions of variants of concern and the associated mutations of concern. We believe that using genomic datasets from a linguistic perspective could supplement the ongoing efforts in understanding associations between the mutations of SARS-CoV-2 genomes and beyond. The NLP-methodologies undertaken, namely DTM and TWEC, can provide temporal insights from a regional or global perspective that may assist in variant identification, classification or even bring forth structural connections for therapeutic targeting.

## Limitations

It is pertinent to note certain limitations and considerations associated with the application of temporal natural language processing concepts on genomic datasets. These are described below.

- **Countering short form text for topic modeling**
  It is well founded that topic modelling works best on large texts (or documents) and discovery of meaningful themes (signatures) can be challenging when document size is too small. Given that mutational profiles observed in viral genomes are sparse, the document equivalents in genomes are inherently small as well. The latter however are not prone to conventional trimming for stop-word or topic general word (TGW) removal, thereby avoiding the aggravating challenges of short-text pre-processing. The challenges of short-text are further lowered in temporal approaches like DTM and TWEC, wherein time-slice and global corpus-based document pooling takes place. Nevertheless, the size of genomic documents (mutation profile in each genome) remains small, and caution must be exercised in reporting the observed topics (signatures) without due validations. In this study, given the small document size and huge pool of genome corpora, we faced computational challenges in converging on window size (for DTM, TWEC), optimal topic numbers and associated hyper-parameter tuning. These points need to be considered while attempting NLP rooted in topic modelling for mutation-profiles in genomes, especially in absence of computational resources.
- **Time-slice selection for DTM and TWEC**
  In the present study, temporal binning (or time-slices) of the documents (i.e., the genome samples) was taken at a monthly level. Given the fixed size of time-slice, the number of sequences captured in each window cannot be controlled. Therefore, considering that the sample size in each slice may be different and is confounded by number of sequence depositions over the course of the pandemic, learning of topics and drift occurs across heterogenous time-slices. This holds true for conventional temporal NLP on evolving pieces of literary documents as well. Based on data availability across different time frames, it is, however, possible to choose a narrower bin to get finer temporal resolution of the semantic shifts from the changes in the word embeddings. One must consider two aspects here, Firstly, a very small window with insufficient data can lead to spurious detection of drift. Secondly, a very large window with too many documents (genomes in this case) can lead to loss in tracking the putative drift. A right balance in choosing the time slice is therefore needed. In case of SARS-CoV-2 genome sequences, the availability of sequenced genomes was limited in early phase of 2020, followed by exponential rise in genome submissions by end of 2020. While a weekly sampling strategy would not capture enough samples in the earlier phases, a quarterly or half yearly sampling was too large for later phases. Considering these points, we kept one-month window for the time-slices as well as for DTM and TWEC models to maintain comparable assessments.
- **Biological interpretation of signatures, drift and scattertext**
  The methodology adopted in this study for tracing topic (signature) evolution and mutational drift is agnostic of the underlying clinical confounders like co-morbidities, vaccination status, HLA type, gender, geography, etc. Consequently, caution must be exercised in associating signatures with clinical relevance. The identified signatures, in absence of experimental corroboration, should be treated as pieces of intelligence that may complement the existing in-silico methods of target discovery. Post-hoc associations with clinically labelled genomes, as attempted in this study, are indeed possible for validating the signatures or relevance of observed drift. For e.g., as presented in Supplementary Fig 8, the intelligence generated by the NLP approach (particularly semantic drift) can aid targeted exploration of the structural context of the mutations of interest (viz. mutations drifting with respect to a mutation of concern).
- **Considerations for clinically labelled data**
  It is pertinent to highlight the limitations of the current study pertaining to utilising associated metadata (corresponding to patient health status) along with the genome sequences deposited by the clinicians and researchers at GISAID. Our choice of labelled data was rooted in the goal of a post-hoc analysis/validation of signatures. However, a very limited number of clinically labelled genomes were available on GISAID. Notably, out of nearly ~2.6 million genome sequences deposited at GISAID (accessed July 2021), only ~77,000 genomes contained metadata specific to patient health status. Moreover, a majority of the labelled genomes had noisy annotations (with inconclusive severity indication). The disproportionate contribution of labelled genomes from different geographical regions across the world further suggested that the data size would be too small for considering analysing country/continent specific temporal analysis using NLP methods. To overcome this limitation, a pooled approach was adopted for global analysis of temporal topics and the mutational evolution/ drift. Nonetheless, given sufficient data availability, the proposed methodology and analytical metrics presented this study can be executed (i) at a regional/VoC level that has good quality clinically labelled data, for gaining region-specific or variant-specific mutational landscape changes; (ii) for choosing any set of mutations (either biologically or statistically inferred), which could then be tracked diachronically to determine their associated mutational partners.

## Methods

### Data preparation

SARS-CoV-2 genomes along with associated metadata were downloaded from GISAID, which at the moment of collection (Jul2021) contained ~ 2.6 million samples. All the samples without patient health status were filtered out. Among these 77,283 filtered samples, those for which the collection date was complete were selected. This led to a total of 75,084 samples comprised of ~ 30,000 mutations.

To profile the mutations, we used a previously adopted protocol^28^. Here, fasta files of individual genomes samples were downloaded and mapped with NCBI GenBank accession NC_045512 (GISAID ID EPI_ISL_402125/coronavirus-2 isolate Wuhan-Hu-1) as the reference using minimap2^29^ (with default parameters other than ‘-cs -cx asm5’). Subsequently, paftools.js was used for identifying nucleotide variations (.vcf file) with respect to reference. Amino acid changes corresponding to the identified nucleotide variations were predicted using BCFtools/csq program^30^. All genome samples were binned into different time-slices, where each time-slice corresponded to months from Dec2019 to Jul2021, bringing to a total of 18 time-slices.

### DTM training

DTM models were created with different ‘k’ values (no. of topics) from 4 to 15, while keeping the default values for the rest of the parameters. Unlike classical LDA, wherein the topic remains unchanged and choosing optimal ‘k’ value from coherence score is rather straightforward, the ‘k’ value for DTM implementation, was chosen based on how distinct each topic was in each time-slice. For this, a similarity metric was created that measured the difference in the top 20 most probable mutations between different topics at a given time-slice. This was measured using the Jaccard similarity metric. For a particular value of ‘k’, the Jaccard similarity between any two topics was summed across all time-slices and divided by the number of time-slices. This Paired Jaccard score was then summed for all pairs of topics possible for the value of ‘k’ and divided by the total no. of pairs.

$${P}_{\left(n,m\right)}=\frac{{\sum }_{i}^{T}{J}_{\left({t}_{n}^{i},{t}_{m}^{i}\right)}}{T}$$

$$OverallJaccardscore=\frac{\sum P}{k\left(k-1\right)/2}$$

k**:** total topics, i **:** one time point (month), no. of time-slices (T) = 18, t_n_, t_m_ **:** any two different topics for same k; J**:** Jaccard Similarity.

The total topic number was set to 6 since its inter-topic similarity was low (Supplementary Fig. 9a) as well as fewer topics make it more interpretable for tracking in drift analysis.

### DTM parameters

For DTM, each sample from the GISAID dataset (till the month of July 2021) was considered as a document (See Data preparation for further details). Nucleotide mutation lists from each GISAID genome sample were segregated according to their month of collection. These constituted the time-slices on which the DTM was performed with the optimal ‘k’ (no. of topics) = 6, lda_sequence_min_iter = 6, alpha = 0.01, rng_seed = 0. A python wrapper for Dynamic Topic Models (DTM) present in gensim^31^ python package (version 3.8) was used on top of compiled binaries of DTM from the original paper^6^ to execute DTM on our corpus. Signature distribution (Fig. 2a) were subsequently calculated by computing the signature distribution (i.e., probabilities of individual signature) for each document (i.e., genome sample) and grouping them into respective countries/variants and then calculating the overall proportion of signature probabilities for each. Aggregated probabilities of ‘most probable mutations’ in each signature (is shown in Fig. 2b) were computed by summing the probabilities in all time-slices, and only the top 20 mutations with the highest sum of probabilities were listed.

### DRIFT skip-gram training

To build the temporal TWEC^7^ model and perform subsequent analysis, we modified and utilized the DRIFT toolkit^8^. Hyperparameters of the Skip-gram architecture of a Word2Vec model like embedding size (i.e., vector dimensionality), word size (i.e., context window) and number of negative samplings, affect the quality of the model. In order to narrow down towards an optimal set of hyperparameters, an approach similar to that incorporated by Dridi *et al*^32^ was followed. The approach was to find overlap in the closest words to a target word with varying hyperparameters, yielding models that were trained on different training sets. The overlap (between models trained on two training sets) was measured via Jaccard similarity between the closest neighbouring words to a target word from each timeframe obtained from one training set vs the other. In our case, the top five nearest neighbours were chosen for stringent similarity testing, and the top 50 most frequent words were chosen as the set of target words for which the Jaccard similarity metric was computed.

$$W=\frac{{\sum }_{i}^{T}J\left({S}_{{h}_{i}},{S}_{{h}_{i}^{^{\prime}}}\right)}{T}$$

$${W}_{k50}=\frac{\sum W}{50}$$

${S}_{{h}_{i}},{S}_{{h}_{i}^{^{\prime}}}$= top 5 closest words in training set1 and set2, J = Jaccard Similarity Score; T = No. of time-slices (18), W = Overall Jaccard score for one word, W_k50_ = Combined Jaccard score for top 50 most frequent words.

On this basis, the training parameters were obtained, specifically for Embedding Size, Window Size, and Negative Sampling (Supplementary Fig. 9b–d). Each pair of training sets whose overlap was measured via the ‘Overall Jaccard score’ for a target word differed only by one hyperparameter value. The training parameters ranged from 50 to 400 for Embedding Size, 2–60 for Window Size, and 1–21 for Negative Sampling.

For embedding size, a window of 10 was first randomly fixed and different training parameters were utilized for tuning embedding size. For embedding size, the mean value of overall Jaccard score (W_k50_) increases rather sluggishly after embedding size of 100. Therefore, the embedding size was fixed at a value of 200 to lower the computational and time costs of training. Similarly, word size of 8 was chosen, as no significant change can be observed beyond that, and a smaller word size helps preserve the genomic context. A negative sampling of 14 was chosen as it showed a relative maximum to other values. Therefore, the final model selection had the parameters as Word Size = 8, Negative Sampling = 14, Embedding Size = 200.

### Individual semantic drift

In this study, four mutations of concerns as reported by outbreak.info^16^ were considered. These were A23063T, C23604A, C23604G and T22917G; all belonging to spike protein. UMAP was used for dimension reduction of individual mutation’s embedding vectors for each time-slice. Additionally, 15 closest similar mutations (as determined through cosine similarity) were chosen for each of those time-slices and were plotted as well. Distribution of neighbours (horizontal barplot) for each MoC was made by mapping which protein the neighbouring mutations (i.e., 15 closest semantically similar mutations) for each time-slice originate from and plotting them in Fig. 3d and Supplementary Figs. 4b, 5b, 6b.

### Genomic loci variance

Genomic loci variance showcases the deviation in genomic positional distance of the neighbouring words to the target (MoC) for each time-slice (Fig. 3c and Supplementary Fig. 4d, 5d, 6d). It is calculated by first extracting the genomic loci position of each neighbouring mutation in a time-slice and computing the standard deviation of the absolute differences between the genomic position of MoC with its neighbouring mutations.

$${\text{PV}}_{{\text{t}}} = {{\varvec{\upsigma}}} \, (\left| {{\text{GP}}_{{{\text{MoC}}}} - {\text{ GP}}_{{\text{Neighbouring Word}}} } \right|)$$

GP : genomic position, PV_t_ : positional variance of at a time-slice t.

### Productivity

To understand the diachronic development of a term/word, Schuman et al.^33^ described a method to model the life cycle of individual words with the help of a term’s frequency and productivity. ‘Term frequency’ signifies the frequency of occurrence of a given term in a given time-slice. In contrast, ‘Term productivity’ measures the ability of a single term to produce new, related multi-word terms. They have conceptualized term productivity by calculating the entropy of conditional probabilities of all *n* multi-word terms *m* that contains single-word term *t* for each timepoint *y*. This is represented in the equation below:

$${e}_{\left(t,y\right)}=-{\sum }_{i=1}^{n}{log}_{2}{(P}_{{m}_{i},y}).{P}_{{m}_{i},y}$$

$$where, {P}_{{m}_{i},y}=\frac{f\left(m\right)}{{\sum }_{i=1}^{n}f\left({m}_{i}\right)}$$

where f (m) denotes the absolute frequency of m.

### Acceleration

To highlight the nature of similarity and the extent of convergence/divergence between two words across two time periods, the word-pair ‘acceleration’ was used. By finding the difference in the cosine similarity of the embedding vectors for a pair of words between two time-slices, the acceleration with which the corresponding word-pair was getting closer (or not) was computed. In other words, it was examined whether they are appearing more frequently in similar contexts or separating out of that context. This metric has been formalised by Dridi *et al*^32^ and is described below.

All distances between two words word_i_ (w_i_) and word_j_ (w_j_) were calculated by the cosine similarity between embedding vectors ${u}_{{w}_{i}}$ and ${u}_{{w}_{j}}$.

$$sim \left({w}_{i},{w}_{j}\right)=cosine\left({u}_{{w}_{i}},{u}_{{w}_{j}}\right)=\frac{{u}_{{w}_{i}},{u}_{{w}_{j}}}{{||u}_{{w}_{i}}||\cdot ||{u}_{{w}_{j}}||}$$

$$acceleration\left({w}_{i},{w}_{j}\right)={\left({w}_{i},{w}_{j}\right)}^{t+1}-{\left({w}_{i},{w}_{j}\right)}^{t}$$

Dynamic acceleration plot Fig. 7c shows changes in acceleration between two words at every time-slice. However, only ten neighbouring words/mutations were selected to display in the graphs, five of them being the most positively accelerating mutations and the other five being the lowest accelerating mutations. The most accelerating neighbour wordset and lowest accelerating wordset were chosen by calculating the sum of accelerations in the past six months (i.e., Jan2021 to Jun2021) and choosing the top five and bottom five, respectively.

### Severity classification of patient health status

We had previously manually annotated the patient health outcomes (reported in GISAID database) into ‘Asymptomatic, Mild, Severe, Fatal’ classes^34^. These classes were grouped into ‘NotSevere’ and ‘Severe’ categories to run binary classification using Scattertext package. Scattertext package version 0.1.4 was used in python3 environment. It uses a criterion called ‘Scaled F-Score' to find how associated words are with two categories. Terms associated to a category must have high category-specific precision and category specific frequency (i.e., percentage of terms in the category that contain the term). F-score is thus the scaled harmonic mean of this precision and frequency. Scaled F-score ranges from − 1 to 1, wherein words closer to − 1 (red-coloured) or 1 (blue coloured) are more characteristic of category1 or category2, respectively. On the other hand, a word is plotted according to its frequency in both categories, i.e., its cartesian coordinates are its frequency (per 25,000) in category1 (i.e., the X-axis) and frequency (per 25,000) in category2 (i.e., the Y-axis). Points corresponding to terms were selectively labelled so that they didn’t overlap with other labels or points. The minimum words frequency for plotting was set to 5, resulting in 2271 words displayed in Fig. 4.

### Tracking clusters

Many words collectively form a context, i.e., they are similar in meaning. But as each of the words drift, so do the semantic cluster they were part of. Therefore, one can track the transition of several words (i.e., mutations) as to whether they consolidate in their semantic context or disunite with time (Fig. 5). Here for each time-slice, mutations were clustered by first reducing the dimensions of their word embeddings using UMAP, then clustering them using faiss’s library for k-means clustering algorithm. The optimal number of clusters for each time-slice was determined using *Silhouette Score* module from the sklearn package^35^. The resulting k-means cluster demarcation for a set of chosen mutations for a given month is represented using circlise package in R language^36^.

## Supplementary Information

Supplementary Information 1.

Supplementary Information 2.

## Data Availability

The datasets analysed during the current study are available in the GISAID repository (Global initiative on sharing all influenza data hosted at https://www.gisaid.org. The datasets are freely available from GISAID, access is however subject to free registration at https://gisaid.org/register/ and subsequent login at https://www.epicov.org/epi3/frontend. Supplementary File 2 provides details of the data contributors for the data employed in this research as per the data access policy of GISAID.

## Code availability

Supplementary Table 5 summarizes the links to the softwares which were utilized to generate the results described in this manuscript. Briefly, the integral core functionality of the algorithms used in the study were derived from the comprehensively documented and easy to follow Drift^8^, *scattertext*^37^ and DTM (https://radimrehurek.com/gensim_3.8.3/models/wrappers/dtmmodel.html) package. All the figures were generated by the authors using the data obtained from these software and plotted using matplotlib and seaborn packages.
