# Supplementary material for: Tracking mutational semantics of SARS-CoV-2 genomes
Source: Sci Rep. 2022 Sep 20;12:15704. doi: 10.1038/s41598-022-20000-5 (PMC9487856; doi:10.1038/s41598-022-20000-5)
Supplement: Supplementary file 1 — Supplementary Information 1. [file 41598_2022_20000_MOESM1_ESM.docx]

**Tracking mutational semantics of SARS-CoV-2 genomes**

Rohan Singh^1^, Sunil Nagpal^1,2,3*^, Nishal K. Pinna^1^, Sharmila S. Mande^1*^

^1^TCS Research, Tata Consultancy Services Ltd, Pune, India 411013

^2^CSIR-Institute of Genomics and Integrative Biology (CSIR-IGIB), New Delhi-110025, India

^3^Academy of Scientific and Innovative Research (AcSIR), Ghaziabad- 201002, India

* Corresponding authors: [sharmila.mande@tcs.com](mailto:sharmila.mande@tcs.com), [sunil.nagpal@tcs.com](mailto:sunil.nagpal@tcs.com)

**Supplementary File 1**

**Identifying mutation signatures (themes) from genomic documents**

In NLP, topic modeling is a type of statistical modeling approach used to describe the process of finding abstract ‘topics’ in a corpus. Latent Dirichlet Allocation^1^ is one popular method that learns a predefined number of topics from a corpus. Each document can be considered a probability distribution of one or more topics, and each topic is a representation of probability distribution over certain n-grams (words) associated with them. LDA has frequently been used in biological contexts for data exploration via clustering, classification or features extraction in an unsupervised fashion^2–4^.

Like LDA, Dynamic Topic Models^5^ (DTM) are stochastic models but can help in analysing the evolution of ’latent’ topics within a corpus over time. In a temporal dataset, the context is heavily dependent on the order of the data (i.e., the documents) that are grouped by time-slices (e.g., days, months, years). However, a (static) LDA model does not consider this ordering. In a DTM model, the words are considered exchangeable and so is the order of appearance of documents, where it is assumed that the documents of each time-slice are generated from the topics that were evolved from the previous time-slice.

DTM associates words to topics based on their statistical relationship of occurrences, then accumulatively assigning topic probabilities to each document. It assigns what topics comprise a document. *Given a biological problem such as tracking mutations of a species, wherein a genome of an organism (i.e., a document) is evolving with time, a use case of DTM could be to discern a set of mutational signatures across a species and investigate how they change in time, essentially, does a mutation differ in its importance as a signature with time, or do we see novel signatures with time.*

**Semantic (context) Drift and Topic (signature) evolution**

From a linguistic perspective, words may ‘drift’ from their semantics over time due to changing linguistic, social and cultural norms. Analogous to this, the mutational prevalence may also shift with respect to time, leading to new variants or subtyping of the disease or depicting a change in pathogenesis.

The solution to the problem of tracing the semantic change in an NLP setting is rooted in Word2Vec^6^. It works on the philosophy of deriving relations between words through their word vectors/embeddings, that forms a context to which words with similar vectors can be associated and thus, it becomes useful in either classifying innominate words or discovering contexts.

Temporal Word Embeddings with a Compass^7^ (TWEC), yields similar type of inferences but with a temporal level of detail. As new documents are generated for newer timepoint, we can trace shifts in the meaning of words or associations with other words via this approach. Therefore, taking the same biological problem (as mentioned in the above section), we can track changes in genomic context of mutation occurrence.

TWEC can be modeled around two Word2vec architectures: Continuous bag-of-words (CBOW) and Skip-gram. Skip-gram predicts the context word for a given target word. The target word (mutation) vectors/embeddings can be provided as input, which can then be used to predict context words occurring nearby to it within a fixed window size (**Fig 1**). Conversely, CBOW method can take the context (as vectors/embeddings) as input to predict the word (i.e. target) corresponding to the said context. While training a TWEC model, one of the two embeddings is fixed while the other is updated with each time-slice of the corpus. The fixed embedding acts as a compass to guide the training of the temporal embeddings to their respective time-slices. The semantic shift can inherently be computed from the TWEC embeddings for a word in each time-slice. By calculating the euclidean/cosine distances for the embeddings of words in two timeframes, we can get an estimate of a word's context over time.

| S.No. | NLP Question | Analogous Biological Question |
| --- | --- | --- |
| 1. | a) What is the vocabulary of a document?  b) What is the word counts in the corpus (entire documents)? | What are the mutations in a sample?  What is the mutation counts in GISAID dataset? |
| 2. | What are the topical themes of a document? | What signatures are present in a sample? |
| 3. | What is the distribution of topical themes in the corpus? | What is the distribution of signatures in the dataset? |
| 4. | How are the themes evolving with time? | How are the signatures evolving with time? |
| 5. | What is driving the evolution of a theme?  Or  Which words are changing (semantic change) that are causing the topics to evolve? | How are signatures evolving? |
| 6. | Is there a semantic context to the word evolutions?  Can we trace word evolution through semantic context?  Can semantic drift reveal theme evolution or emergent themes? | Is mutational progression associated to genomic position? |
| 7. | How are the words changing (semantic change) w.r.t context? | How are the mutations progressing? |
| 8. | How does semantic change of a word correspond with other words? | How does progression of a mutation correspond with other mutations? |
| 9. |  | a) What are the characteristics mutations of Severe and NotSevere classification?  b) How does the temporal clustering of these characteristic mutations look like? |

**Supplementary Table 1:** Summary of the questions examined towards study design for the current research

| **Set name** | **Negative Samples** | **Window Size** | **Embedding size** |
| --- | --- | --- | --- |
| E50 | 10 | 10 | 50 |
| E100 | 10 | 10 | 100 |
| E150 | 10 | 10 | 150 |
| E200 | 10 | 10 | 200 |
| E250 | 10 | 10 | 250 |
| E300 | 10 | 10 | 300 |
| E400 | 10 | 10 | 400 |

**Supplementary Table 2:** TWEC Training parameter for embedding-size tuning

| **Set name** | **Negative Samples** | **Window Size** | **Embedding size** |
| --- | --- | --- | --- |
| W2 | 10 | 2 | 200 |
| W3 | 10 | 3 | 200 |
| W4 | 10 | 4 | 200 |
| W5 | 10 | 5 | 200 |
| W6 | 10 | 6 | 200 |
| W7 | 10 | 7 | 200 |
| W8 | 10 | 8 | 200 |
| W9 | 10 | 9 | 200 |
| W10 | 10 | 10 | 200 |
| W11 | 10 | 11 | 200 |
| W12 | 10 | 12 | 200 |
| W13 | 10 | 13 | 200 |
| W14 | 10 | 14 | 200 |
| W15 | 10 | 15 | 200 |
| W17 | 10 | 17 | 200 |
| W20 | 10 | 20 | 200 |
| W23 | 10 | 23 | 200 |
| W25 | 10 | 25 | 200 |
| W27 | 10 | 27 | 200 |
| W30 | 10 | 30 | 200 |
| W35 | 10 | 35 | 200 |
| W40 | 10 | 40 | 200 |
| W45 | 10 | 45 | 200 |
| W50 | 10 | 50 | 200 |
| W60 | 10 | 60 | 200 |

**Supplementary Table 3:** TWEC Training parameter for word-size tuning

| **Set name** | **Negative Samples** | **Window Size** | **Embedding size** |
| --- | --- | --- | --- |
| NS1 | 1 | 8 | 200 |
| NS2 | 2 | 8 | 200 |
| NS3 | 3 | 8 | 200 |
| NS4 | 4 | 8 | 200 |
| NS5 | 5 | 8 | 200 |
| NS6 | 6 | 8 | 200 |
| NS7 | 7 | 8 | 200 |
| NS8 | 8 | 8 | 200 |
| NS9 | 9 | 8 | 200 |
| NS10 | 10 | 8 | 200 |
| NS11 | 11 | 8 | 200 |
| NS12 | 12 | 8 | 200 |
| NS13 | 13 | 8 | 200 |
| NS14 | 14 | 8 | 200 |
| NS15 | 15 | 8 | 200 |
| NS16 | 16 | 8 | 200 |
| NS17 | 17 | 8 | 200 |
| NS18 | 18 | 8 | 200 |
| NS19 | 19 | 8 | 200 |
| NS20 | 20 | 8 | 200 |
| NS21 | 21 | 8 | 200 |

**Supplementary Table 4:** TWEC Training parameter for negative samples tuning

| **Tool** | **Function** | **Code reference** |
| --- | --- | --- |
| DRIFT | To create TWEC models and word embedding analysis | https://github.com/rajaswa/DRIFT |
| DTM | A wrapper for genism package to create dynamic topic model in python | https://radimrehurek.com/gensim_3.8.3/models/wrappers/dtmmodel.html |
| Scattertext | To find and plot characteristic terms for binary categories in a corpus | https://github.com/JasonKessler/scattertext |
| EzMol | Protein structure visualization | http://www.sbg.bio.ic.ac.uk/ezmol/ |

**Supplementary Table 5:** List of software and code references used in the study.


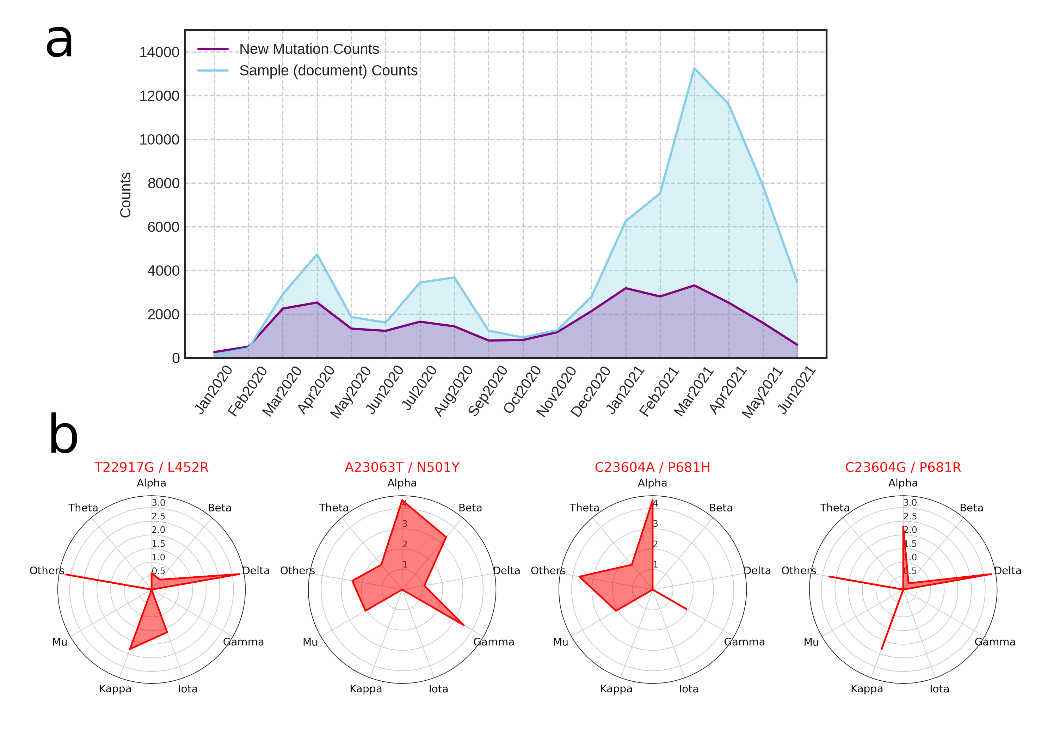


**Supplementary Fig 1: a)** Frequency of documents (i.e. genomic samples obtained from GISAID^8^) highlighted in blue, and frequency of new mutations highlighted in purple. **b)** Radial plot showing the distribution of four mutations of concern in samples classified according to the variant type. Inner contour lines correspond to frequency (scaled by log10).


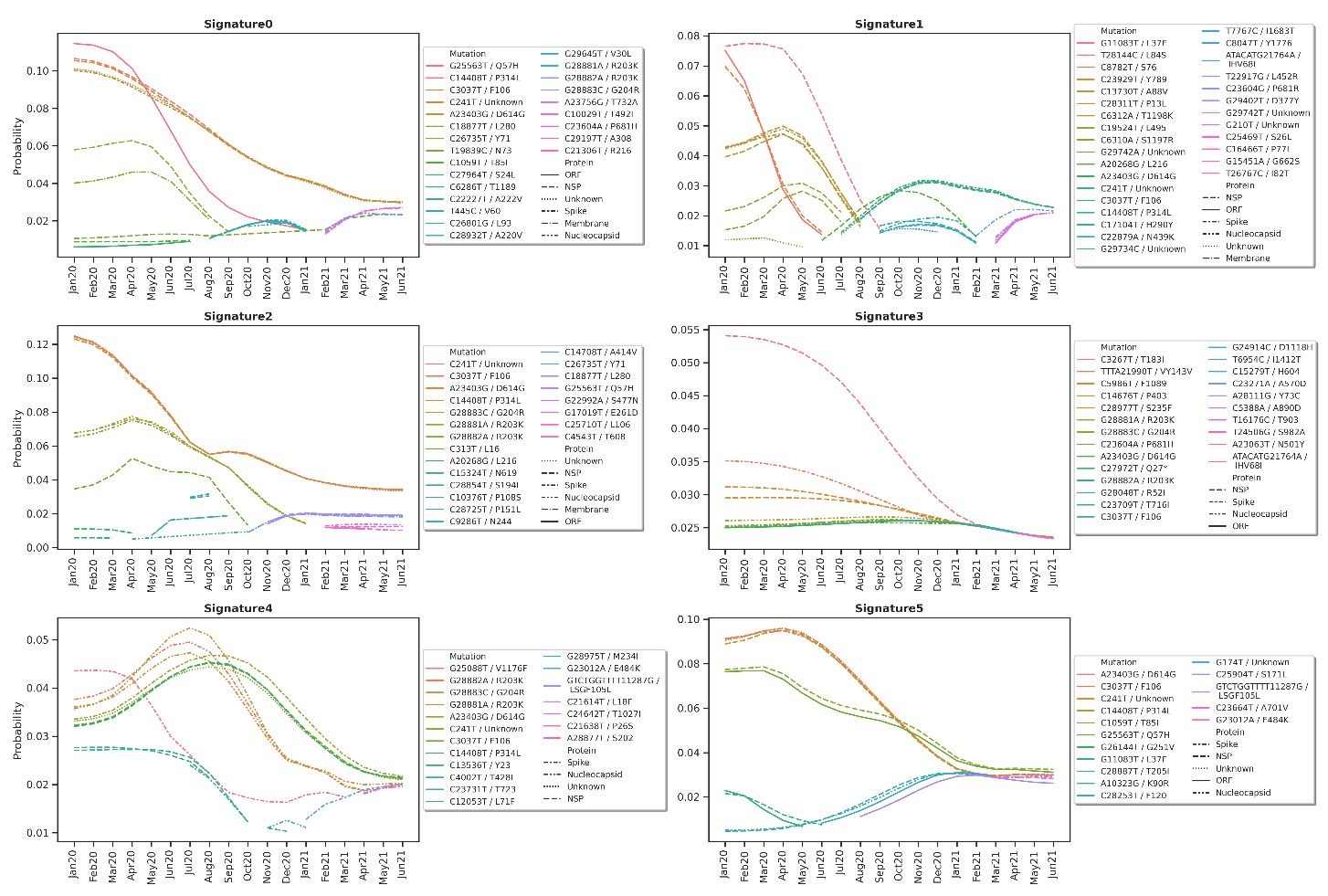
**Supplementary Fig 2:** Diachronic shifts of word probabilities in each topic/signature.


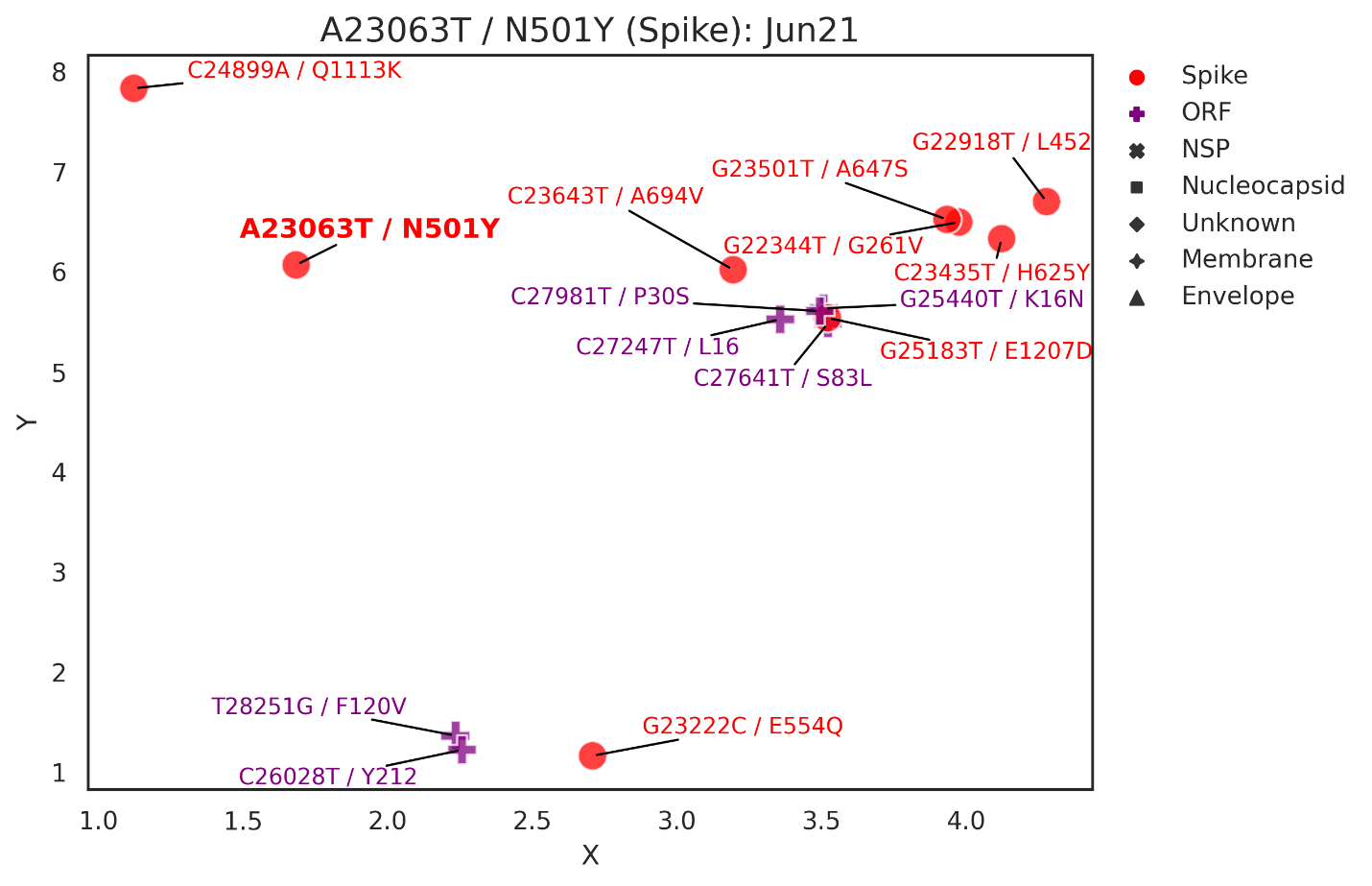


**Supplementary Fig 3:** Embedding UMAP projection of word embedding of mutation A23063T and 15 most similar words in the last time-slice (i.e., for the month of June 2021).


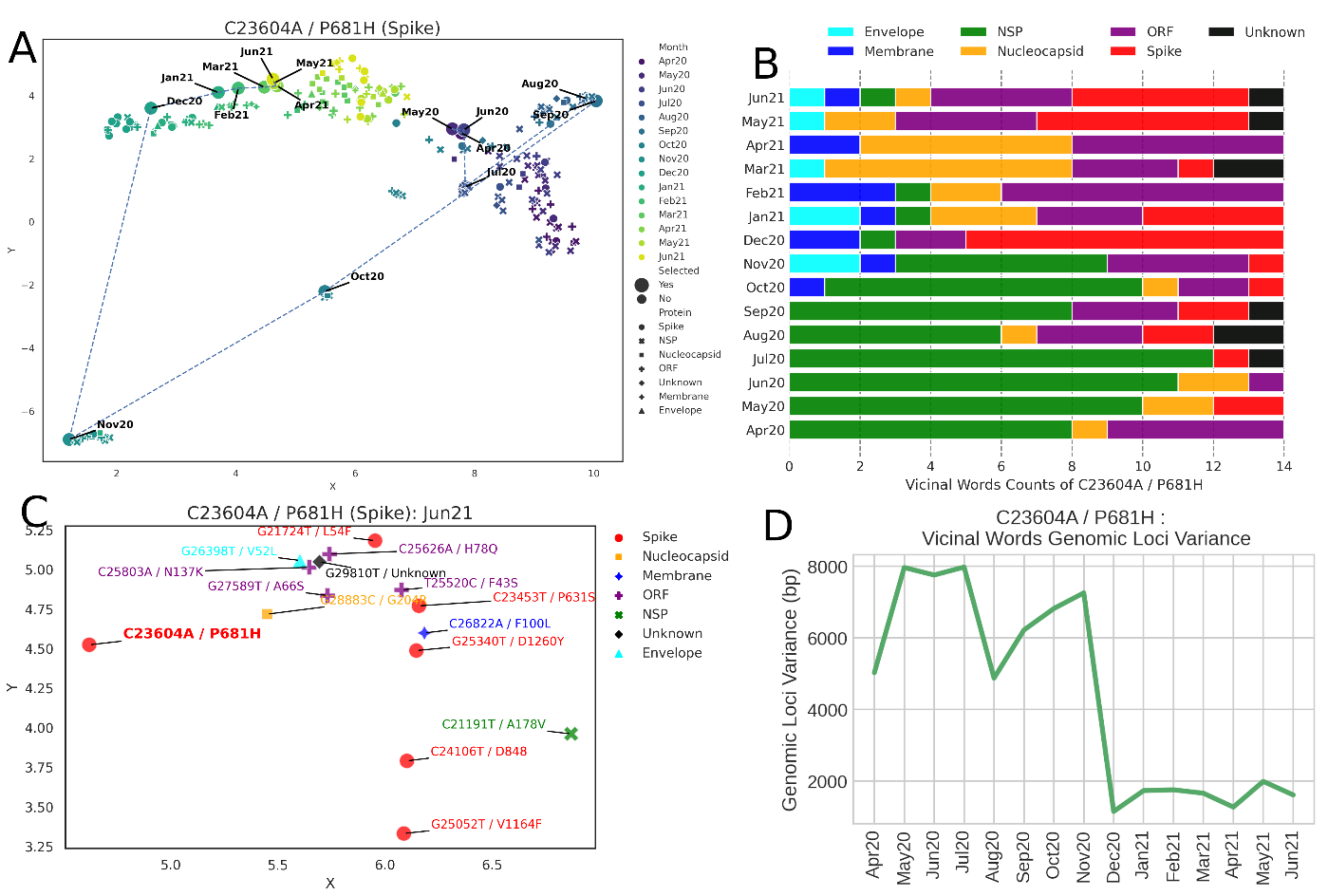


**Supplementary Fig 4:** Semantic Drift of mutation C23604A / P681H (Spike) **a)** UMAP projection of word embedding of mutation C23604A and 15 most similar words for each time-slice from Apr2020 to Jun2021. The dotted line links the word embedding projection point for the mutation of concern. **b)** Neighbouring word distribution based on protein mutation for each time-slice. **c)** UMAP^9^ projection of word embedding of mutation C23604A and 15 most similar words in the last time-slice. **d)** Standard deviations of genomic positions of neighbouring words of the mutation of concern.


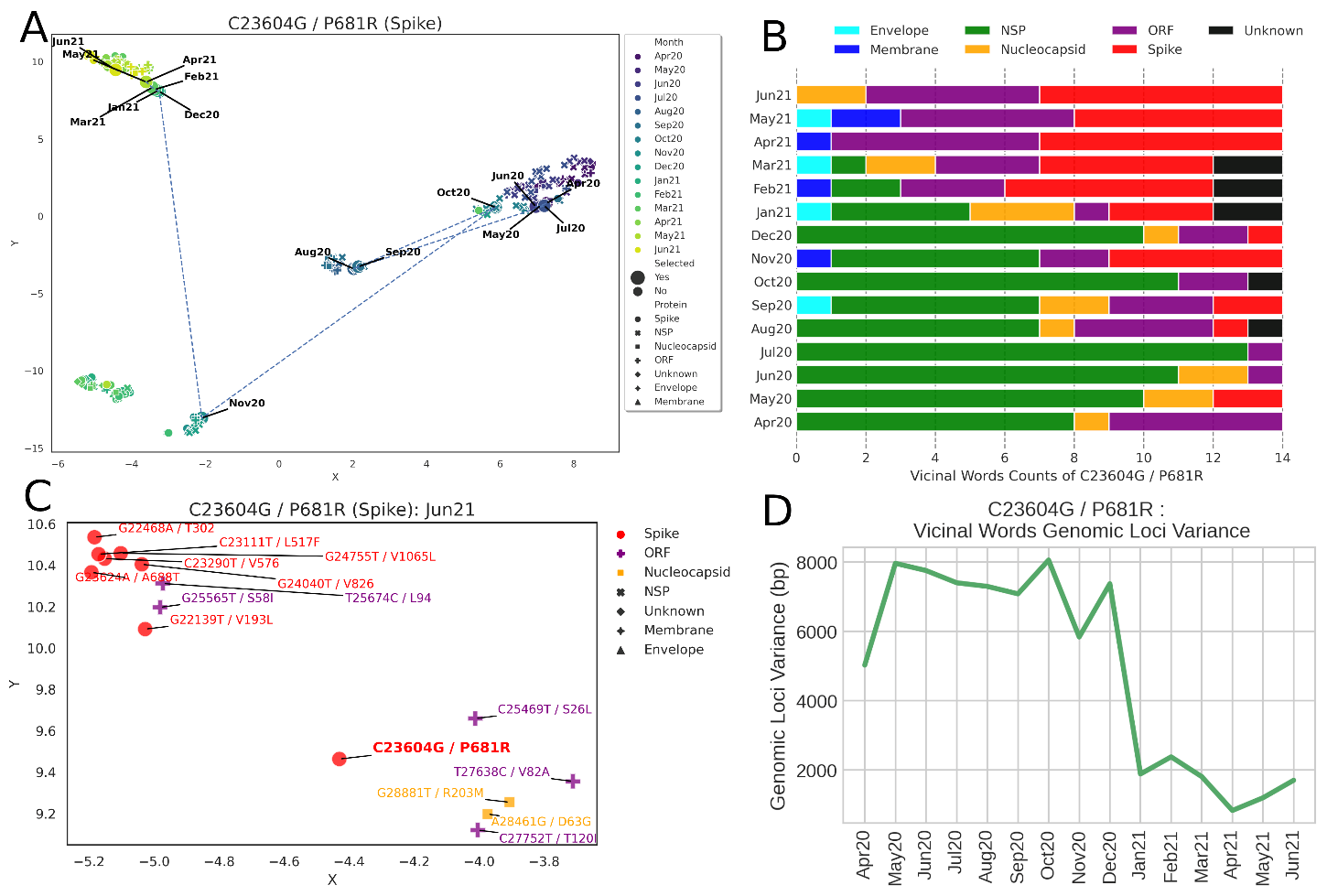
**Supplementary Fig 5:** Semantic Drift of mutation C23604G / P681R (Spike) **a)** UMAP projection of word embedding of mutation C23604G and 15 most similar words for each time-slice from Apr2020 to Jun2021. The dotted line links the word embedding projection point for the mutation of concern. **b)** Neighbouring word distribution based on protein mutation for each time-slice. **c)** UMAP projection of word embedding of mutation C23604G and 15 most similar words in the last time-slice. **d)** Standard deviations of genomic positions of neighbouring words of the mutation of concern.


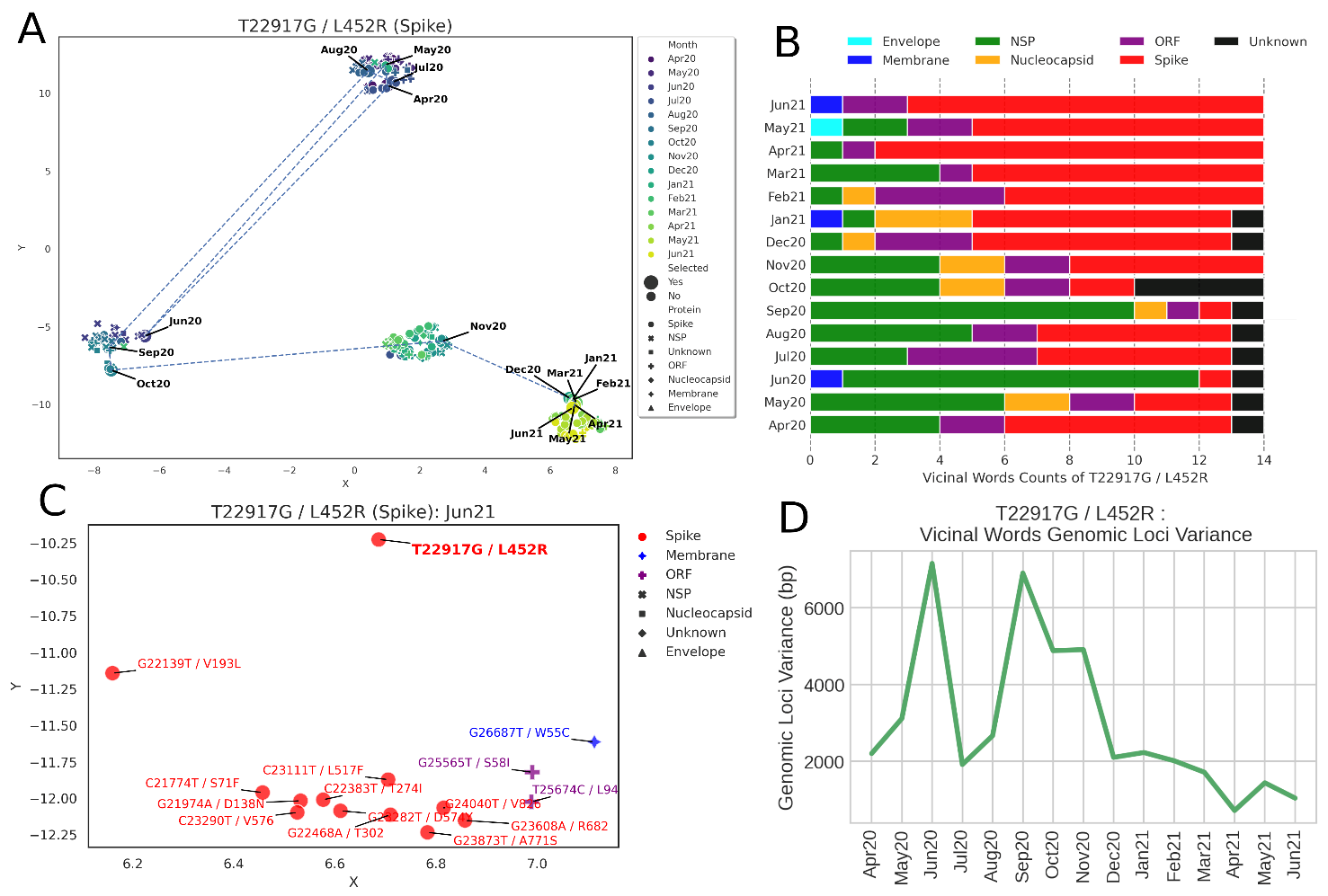
**Supplementary Fig 6:** Semantic Drift of mutation T22917G / L452R (Spike) **a)** UMAP projection of word embedding of mutation T22917G and 15 most similar words for each time-slice from Apr2020 to Jun2021. The dotted line links the word embedding projection point for the mutation of concern. **b)** Neighbouring word distribution based on protein mutation for each time-slice. **c)** UMAP projection of word embedding of mutation T22917G and 15 most similar words in the last time-slice. **d)** Standard deviations of genomic positions of neighbouring words of the mutation of concern.


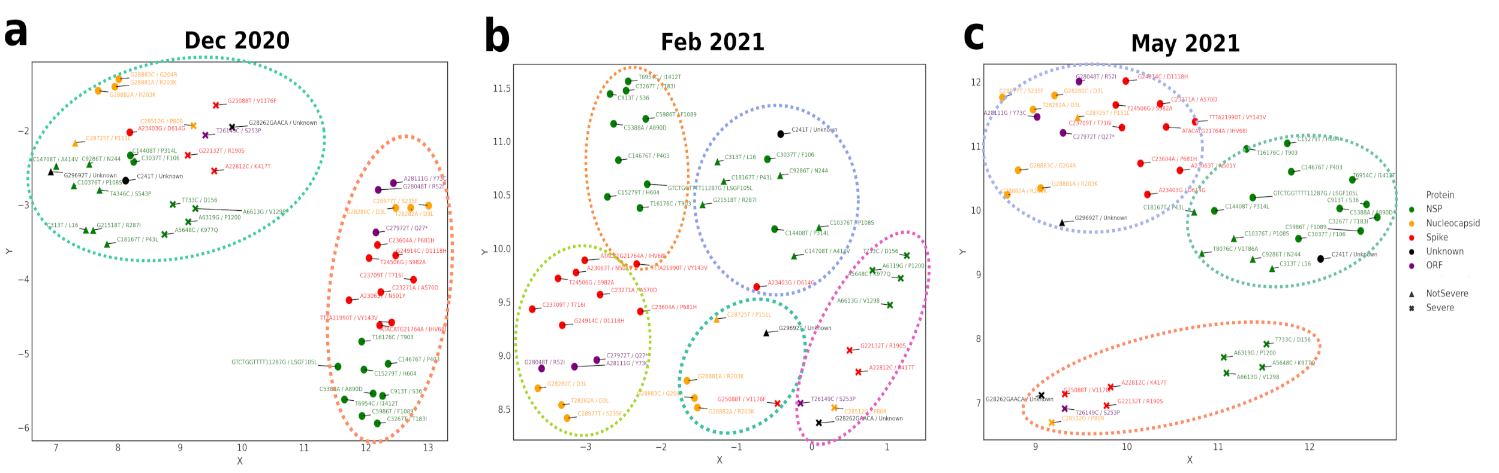


**Supplementary Fig 7: Semantic clustering of Severe, NotSevere and most frequent mutations.** Panels **a,b,c** show UMAP projection of the k-means clustered word embeddings of 50 mutations (10 'Severe', 10 'NotSevere' and 30 most frequent mutations) in time-slices Dec2020, Feb2021 and May2021, respectively. Mutations belonging to a cluster are encircled with dotted lines coloured according to clusters shown in panels **Fig 5a,b,c**. Note that nucleotide mutations coloured in black (as Unknown) are UTRs.


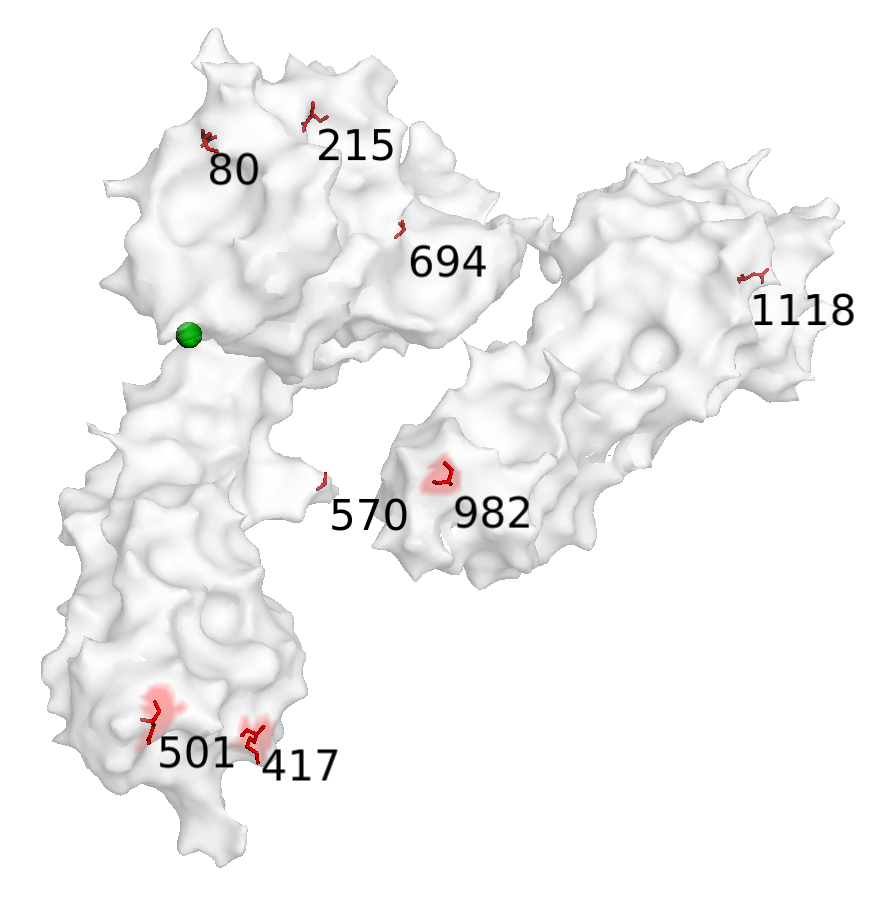


**Supplementary Fig 8: Highlighting residue positions of the drifting neighbours of N501Y mutation.** Semantic drift neighbours of A23063T/Spike-N501Y mutation (in red) are represented on the chain A of spike protein (pdb id: 6VXX^10^). All of these residues are on the exposed part of the spike protein chain. Protein structure visualization was performed using the EzMol webtool^11^.


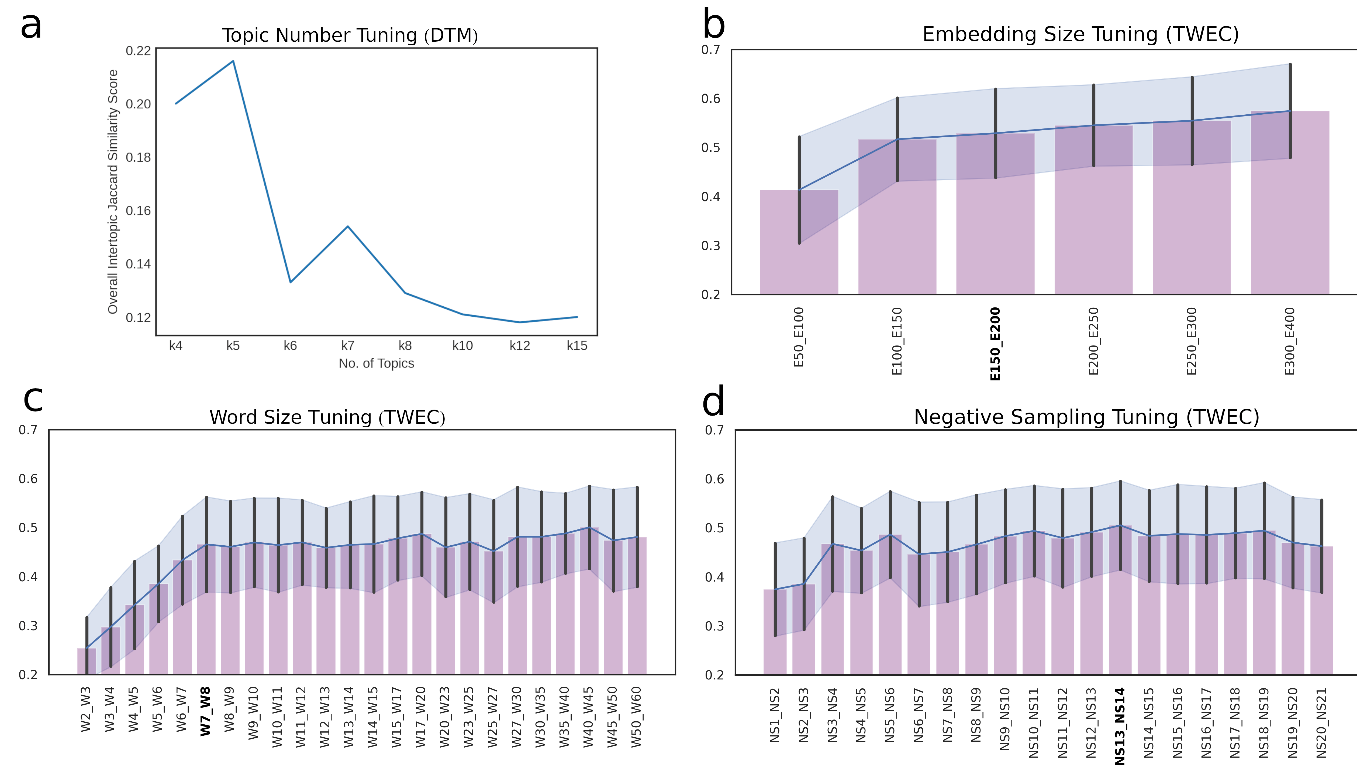


**Supplementary Fig 9:** Hyperparameter tuning of DTM and TWEC models. **a)** Topic number selection via Jaccard (dis)similarity between topics of models trained on different topic numbers. For our study, we chose six as the topic number. Panels **b,c,d** correspond to hyperparameter optimisation for embedding size, word size and negative sampling (for Skipgram architecture of Word2Vec). The selected hyperparameter value is highlighted in bold on the X-axis of each panel.

1. Blei, D. M., Ng, A. Y. & Jordan, M. I. Latent Dirichlet Allocation. *J. Mach. Learn. Res.* **3**, 993–1022 (2003).

2. Liu, L., Tang, L., Dong, W., Yao, S. & Zhou, W. An overview of topic modeling and its current applications in bioinformatics. *Springerplus* **5**, 1608 (2016).

3. Zhang, Y. *et al.* Systematic identification of latent disease-gene associations from PubMed articles. *PLoS ONE* **13**, (2018).

4. Backenroth, D. *et al.* FUN-LDA: A Latent Dirichlet Allocation Model for Predicting Tissue-Specific Functional Effects of Noncoding Variation: Methods and Applications. *American Journal of Human Genetics* **102**, (2018).

5. Blei, D. M. & Lafferty, J. D. Dynamic topic models. in *ACM International Conference Proceeding Series* vol. 148 (2006).

6. Mikolov, T., Chen, K., Corrado, G. & Dean, J. Efficient estimation of word representations in vector space. in *1st International Conference on Learning Representations, ICLR 2013 - Workshop Track Proceedings* (2013).

7. di Carlo, V., Bianchi, F. & Palmonari, M. Training temporal word embeddings with a compass. in *33rd AAAI Conference on Artificial Intelligence, AAAI 2019, 31st Innovative Applications of Artificial Intelligence Conference, IAAI 2019 and the 9th AAAI Symposium on Educational Advances in Artificial Intelligence, EAAI 2019* (2019). doi:10.1609/aaai.v33i01.33016326.

8. Shu, Y. & McCauley, J. GISAID: Global initiative on sharing all influenza data – from vision to reality. *Eurosurveillance* vol. 22 Preprint at https://doi.org/10.2807/1560-7917.ES.2017.22.13.30494 (2017).

9. McInnes, L., Healy, J., Saul, N. & Großberger, L. UMAP: Uniform Manifold Approximation and Projection. *Journal of Open Source Software* **3**, (2018).

10. Walls, A. C. *et al.* Structure, Function, and Antigenicity of the SARS-CoV-2 Spike Glycoprotein. *Cell* **181**, (2020).

11. Reynolds, C. R., Islam, S. A. & Sternberg, M. J. E. EzMol: A Web Server Wizard for the Rapid Visualization and Image Production of Protein and Nucleic Acid Structures. *Journal of Molecular Biology* **430**, (2018).
