# Supplementary material for: Tracking mutational semantics of SARS-CoV-2 genomes
Source: Sci Rep. 2022 Sep 20;12:15704. doi: 10.1038/s41598-022-20000-5 (PMC9487856; doi:10.1038/s41598-022-20000-5)
Supplement: Supplementary file 2 — Supplementary Information 2. [file 41598_2022_20000_MOESM2_ESM.pdf]

We gratefully acknowledge the following Authors from the Originating laboratories responsible for obtaining the specimens, as well as the Submitting laboratories where the genome data were generated and shared via GISAID, on which this research is based.

All Submitters of data may be contacted directly via www.gisaid.org

Authors are sorted alphabetically.

Accession ID	Originating Laboratory	Submitting Laboratory	Authors
EPI_ISL_1001044, EPI_ISL_1001052, EPI_ISL_1001059, EPI_ISL_1001062	Centre Pasteur du Cameroun	Institut Pasteur de Dakar	Njouom Richard
EPI_ISL_1007662, EPI_ISL_1007663, EPI_ISL_1007664, EPI_ISL_1007665, EPI_ISL_1007666, EPI_ISL_1007667, EPI_ISL_1007668, EPI_ISL_1007669, EPI_ISL_1007670, EPI_ISL_1007671	Department of Internal Medicine, College of Medicine, Chosun University	Department of Internal Medicine, College of Medicine, Chosun University	Dong-Min Kim
EPI_ISL_1014677, EPI_ISL_1014682	National Influenza Center, Virology Department	National Influenza Center	NZ Shafiei Jandaghi, V Salimi, A Nejati, K Sadeghi, J Yavarian, N Ghavvami,F Ajaminejad and T Mokhtari Azad
EPI_ISL_1014683	National Influenza Center, Virology Department	National Influenza Center	J Yavarian,K Sadeghi, NZ Shafiei Jandaghi, V Salimi, A Nejati, N Ghavvami,F Ajaminejad and T Mokhtari Azad
EPI_ISL_1014684	National Influenza Center, Virology Department	National Influenza Center	A Nejati, J Yavarian,K Sadeghi, NZ Shafiei Jandaghi, V Salimi, N Ghavvami,F Ajaminejad and T Mokhtari Azad
EPI_ISL_1020171, EPI_ISL_1020172, EPI_ISL_1020173, EPI_ISL_1020174	AZ Klina	AZ Klina	Dr. C. Vael
EPI_ISL_1027658, EPI_ISL_1027659, EPI_ISL_1027660, EPI_ISL_1027661, EPI_ISL_1027662, EPI_ISL_1027663, EPI_ISL_1027664, EPI_ISL_1027665, EPI_ISL_1027666	Mako Medical Laboratories	Mako Medical Laboratories	"Lauren Moon, Matthew Tugwell"
EPI_ISL_1029957, EPI_ISL_1029958, EPI_ISL_1029959, EPI_ISL_1029960, EPI_ISL_1029961, EPI_ISL_1029962, EPI_ISL_1029963, EPI_ISL_1029964, EPI_ISL_1029965, EPI_ISL_1029966, EPI_ISL_1029968, EPI_ISL_1029969, EPI_ISL_1029971, EPI_ISL_1029972, EPI_ISL_1029973			
see above	Cliniques universitaires Saint-Luc	UCLouvain/IREC/MBLG	Jean Ruelle, Lysa Pinsmaye, Benoit Kabamba Mukadi
EPI_ISL_1034305	Laboratorio de Ecologia de Doencas Transmissiveis na Amazonia, Instituto Leonidas e Maria Deane - Fiocruz Amazonia	Laboratorio de Ecologia de Doencas Transmissiveis na Amazonia, Instituto Leonidas e Maria Deane - Fiocruz Amazonia	Valdinete Nascimento, Victor Souza, André Corado, Fernanda Nascimento, George Silva, Ágatha Costa, Debora Duarte, Karina Pessoa, Matilde Mejía, Luciana Gonçalves, Maria Júlia Brandão, Michele Jesus, Felipe Naveca on behalf of the Fiocruz COVID-19 Genomic Surveillance Network
EPI_ISL_1039249	Virus Research and Diagnostic Laboratory, Medical College Level, Kasturba Hospital for Infectious Diseases	Indian Council of Medical Research-National Institute of Virology, Microbial Containment Complex	Pragya D. Yadav, Jayanti Shastri
EPI_ISL_1040031, EPI_ISL_1040033, EPI_ISL_1040034, EPI_ISL_1040036, EPI_ISL_1040037, EPI_ISL_1040040, EPI_ISL_1040041, EPI_ISL_1040042, EPI_ISL_1040043, EPI_ISL_1040045, EPI_ISL_1040046, EPI_ISL_1040047, EPI_ISL_1040048			
see above	PCR Laboratory, The First Affiliated Hospital of Zhengzhou University, Zhengzhou, Henan, China	PCR Laboratory, The First Affiliated Hospital of Zhengzhou University, Zhengzhou, Henan, China	Guanglin Cui, Shijie Zhang, Li Sun, Xianchun Meng, Ting Sun, Junhu Wan, Lijuan Rong, Xiangbo Huang, Lei Zheng, Hao Guo, Meijing Long
EPI_ISL_1055383	Virus Research and Diagnostic Laboratory, Medical College Level, Kasturba Hospital for Infectious Diseases	Indian Council of Medical Research-National Institute of Virology, Microbial Containment Complex	Pragya D. Yadav, Jayanti Shastri
EPI_ISL_1055384, EPI_ISL_1055768, EPI_ISL_1055770, EPI_ISL_1055771, EPI_ISL_1055785, EPI_ISL_1055788, EPI_ISL_1055789, EPI_ISL_1063400, EPI_ISL_1063401, EPI_ISL_1063494, EPI_ISL_1063526, EPI_ISL_1063527, EPI_ISL_1063529, EPI_ISL_1063531			
see above	Virus Research and Diagnostic Laboratory, Medical College Level, Kasturba Hospital for Infectious Diseases	National Institute of Virology-Microbial Containment Complex, Indian Council of Medical Research	Pragya D. Yadav, Jayanti Shastri
EPI_ISL_1068593	Microbiological Laboratory	Microbiological Laboratory,Lu`an Center for Disease Control and Prevention	Yang Wei, Gao Dawei, Chen Zhichao, Chang Hongwei, Zhang Feng, Zhang Limei, Chen Beilei, Zhang Qin, Li Zhaoyang, Zhu Rui
EPI_ISL_1068682	Micrbiological Laboratory	Micrbiological Laboratory,Lu`an Center for Disease Control and Prevention	Yang Wei,Gao Dawei, Chen Zhichao,Chang Hongwei,Zhang Feng,Zhang Limei,Chen Beilei,Zhang Qin,Li Zhaoyang,Zhu Rui
EPI_ISL_1069173	Micrbiological Laboratory,Lu`an Center for Disease Control and Prevention	Micrbiological Laboratory,Lu`an Center for Disease Control and Prevention	Yang Wei,Gao Dawei, Chen Zhichao,Chang Hongwei,Zhang Feng,Zhang Limei,Chen Beilei,Zhang Qin,Li Zhaoyang,Zhu Rui
EPI_ISL_1069192	Micrbiological Laboratory,Lu`an Center for Disease Control and Prevention	No.409,Gaocheng Middle Road,Lu`an City,Anhui Provice	Yang Wei,Gao Dawei, Chen Zhichao,Chang Hongwei,Zhang Feng,Zhang Limei,Chen Beilei,Zhang Qin,Li Zhaoyang,Zhu Rui
EPI_ISL_1069193	Micrbiology Laboratory,Lu`an Center for Disease Control and Prevention	Micrbiology Laboratory,Lu`an Center for Disease Control and Prevention	Yang Wei,Gao Dawei, Chen Zhichao,Chang Hongwei,Zhang Feng,Zhang Limei,Chen Beilei,Zhang Qin,Li Zhaoyang,Zhu Rui
EPI_ISL_1069198, EPI_ISL_1069199, EPI_ISL_1069200, EPI_ISL_1069201, EPI_ISL_1069203, EPI_ISL_1069205, EPI_ISL_1069206, EPI_ISL_1069207, EPI_ISL_1069208, EPI_ISL_1069210, EPI_ISL_1069211, EPI_ISL_1069213, EPI_ISL_1069215			
see above	Microbiology Laboratory,Lu`an Center for Disease Control and Prevention	Microbiology Laboratory,Lu`an Center for Disease Control and Prevention	Yang Wei,Gao Dawei, Chen Zhichao,Chang Hongwei,Zhang Feng,Zhang Limei,Chen Beilei,Zhang Qin,Li Zhaoyang,Zhu Rui
EPI_ISL_1069216	Microbiology Laboratory,Lu`an Center for Disease Control and Prevention	Microbiology Laboratory,Lu`an Center for Disease Control and Prevention	Microbiology Laboratory,Lu`an Center for Disease Control and Prevention
EPI_ISL_1069219, EPI_ISL_1081193, EPI_ISL_1081194, EPI_ISL_1081195, EPI_ISL_1081196, EPI_ISL_1081197, EPI_ISL_1081198, EPI_ISL_1081199, EPI_ISL_1081260, EPI_ISL_1081291, EPI_ISL_1081310, EPI_ISL_1081311, EPI_ISL_1081324, EPI_ISL_1081325, EPI_ISL_1081326, EPI_ISL_1081331, EPI_ISL_1081340			
see above	Microbiology Laboratory,Lu`an Center for Disease Control and Prevention	Microbiology Laboratory,Lu`an Center for Disease Control and Prevention	Yang Wei,Gao Dawei, Chen Zhichao,Chang Hongwei,Zhang Feng,Zhang Limei,Chen Beilei,Zhang Qin,Li Zhaoyang,Zhu Rui
EPI_ISL_1111075, EPI_ISL_1111076, EPI_ISL_1111077, EPI_ISL_1111078, EPI_ISL_1111079, EPI_ISL_1111080, EPI_ISL_1111081, EPI_ISL_1111086, EPI_ISL_1111087, EPI_ISL_1111088, EPI_ISL_1111089, EPI_ISL_1111090, EPI_ISL_1111091, EPI_ISL_1111092, EPI_ISL_1111093, EPI_ISL_1111094, EPI_ISL_1111095, EPI_ISL_1111096, EPI_ISL_1111097, EPI_ISL_1111098, EPI_ISL_1111100, EPI_ISL_1111101, EPI_ISL_1111102, EPI_ISL_1111103, EPI_ISL_1111104, EPI_ISL_1111105, EPI_ISL_1111106, EPI_ISL_1111107, EPI_ISL_1111108, EPI_ISL_1111109, EPI_ISL_1111110, EPI_ISL_1111111, EPI_ISL_1111112, EPI_ISL_1111113, EPI_ISL_1111114, EPI_ISL_1111115, EPI_ISL_1111116, EPI_ISL_1111117, EPI_ISL_1111118, EPI_ISL_1111119, EPI_ISL_1111120, EPI_ISL_1111122, EPI_ISL_1111123, EPI_ISL_1111124, EPI_ISL_1111125, EPI_ISL_1111126, EPI_ISL_1111162, EPI_ISL_1111163, EPI_ISL_1111164, EPI_ISL_1111169, EPI_ISL_1111170, EPI_ISL_1111171, EPI_ISL_1111172, EPI_ISL_1111174, EPI_ISL_1111175, EPI_ISL_1111176, EPI_ISL_1111177, EPI_ISL_1111178, EPI_ISL_1111179, EPI_ISL_1111180, EPI_ISL_1111181, EPI_ISL_1111182, EPI_ISL_1111183, EPI_ISL_1111184, EPI_ISL_1111185, EPI_ISL_1111186, EPI_ISL_1111187, EPI_ISL_1111188, EPI_ISL_1111189, EPI_ISL_1111190, EPI_ISL_1111192, EPI_ISL_1111193, EPI_ISL_1111194, EPI_ISL_1111195, EPI_ISL_1111196, EPI_ISL_1111197, EPI_ISL_1111198, EPI_ISL_1111199, EPI_ISL_1111200, EPI_ISL_1111201, EPI_ISL_1111202, EPI_ISL_1111203, EPI_ISL_1111204, EPI_ISL_1111205, EPI_ISL_1111206, EPI_ISL_1111207, EPI_ISL_1111208, EPI_ISL_1111209, EPI_ISL_1111210, EPI_ISL_1111211, EPI_ISL_1111212, EPI_ISL_1111213, EPI_ISL_1111214, EPI_ISL_1111215, EPI_ISL_1111216, EPI_ISL_1111217, EPI_ISL_1111218, EPI_ISL_1111219, EPI_ISL_1111220, EPI_ISL_1111221, EPI_ISL_1111222, EPI_ISL_1111223, EPI_ISL_1111224, EPI_ISL_1111225, EPI_ISL_1111226, EPI_ISL_1111227, EPI_ISL_1111228, EPI_ISL_1111229, EPI_ISL_1111230, EPI_ISL_1111231, EPI_ISL_1111232, EPI_ISL_1111233, EPI_ISL_1111234, EPI_ISL_1111235, EPI_ISL_1111236, EPI_ISL_1111237, EPI_ISL_1111238, EPI_ISL_1111239, EPI_ISL_1111240, EPI_ISL_1111267, EPI_ISL_1111268, EPI_ISL_1111269, EPI_ISL_1111270, EPI_ISL_1111271, EPI_ISL_1111272, EPI_ISL_1111273, EPI_ISL_1111274, EPI_ISL_1111275, EPI_ISL_1111276, EPI_ISL_1111277, EPI_ISL_1111278, EPI_ISL_1111279, EPI_ISL_1111280, EPI_ISL_1111313, EPI_ISL_1111333, EPI_ISL_1111342, EPI_ISL_1111352, EPI_ISL_1111356, EPI_ISL_1111358, EPI_ISL_1111366, EPI_ISL_1111367, EPI_ISL_1111368, EPI_ISL_1111369, EPI_ISL_1111370, EPI_ISL_1111371, EPI_ISL_1111372, EPI_ISL_1111373, EPI_ISL_1111374, EPI_ISL_1111375, EPI_ISL_1111376, EPI_ISL_1111377, EPI_ISL_1111378, EPI_ISL_1111379, EPI_ISL_1111380, EPI_ISL_1111381, EPI_ISL_1111382			
see above	Laboratorio de Referencia Nacional de Virus Respiratorio. Instituto Nacional de Salud Perú	Laboratorio de Referencia Nacional de Enteropatógenos. Instituto Nacional de Salud del Perú	Ronnie Gavilan Chavez, Junior Caro Castro, Willi Quino Sifuentes, Veronica Hurtado Vela, Iris Silva Molina, Fiorella Orellana Peralta

EPI_ISL_1112947, EPI_ISL_1112948, EPI_ISL_1112949, EPI_ISL_1112950	Baylor College of Medicine/ GCID	Baylor College of Medicine/ GCID	Vasanthi Avadhanula, Erin Nicholson, David Henke, Pedro Piedra, Harsha Doddapaneni, Donna Muzny, Qingchang Meng, Hsu Chao, Zeineen Momin, Hua Shen, George Weissenberger, Kavya Kottapalli, Yimiti Meiheerguli, Sejal Salvi, Ginger Metcalf, Vipin Menon, Sara J.J. Cregeen, Matthew C. Ross, Tulin Ayvaz, Richard Sucgang, Kristi L. Hoffman, Matthew Wong, Joseph F. Petrosino
EPI_ISL_1138989	Rajendra Institute of Medical Sciences, Ranchi, Jharkhand	Indian Council of Medical Research-National Institute of Virology, Microbial Containment Complex	Pragya D. Yadav, Manoj Kumar
EPI_ISL_1139056, EPI_ISL_1139057, EPI_ISL_1139058, EPI_ISL_1139060, EPI_ISL_1139061, EPI_ISL_1139062, EPI_ISL_1139063, EPI_ISL_1139064, EPI_ISL_1139065, EPI_ISL_1139066, EPI_ISL_1139067, EPI_ISL_1139068			
see above	LACEN do Mato Grosso do Sul	Instituto Adolfo Lutz, Interdiciplinary Procedures Center, Strategic Laboratory	Claudio Tavares Sacchi, Claudia Regina Gonçalves, Erica Valessa Ramos Gomes, Karoline Rodrigues Campos, Caio Vinicius Dias Lopes
EPI_ISL_1139114, EPI_ISL_1139115, EPI_ISL_1139116, EPI_ISL_1139117, EPI_ISL_1139118, EPI_ISL_1139119, EPI_ISL_1139120, EPI_ISL_1139121	ICMR-RMRC, Dibrugarh	Indian Council of Medical Research-National Institute of Virology, Microbial Containment Complex	Pragya D. Yadav, Biswajyoti Borkakoty
EPI_ISL_1163693, EPI_ISL_1163694, EPI_ISL_1163695, EPI_ISL_1163696, EPI_ISL_1163697, EPI_ISL_1163698, EPI_ISL_1163699, EPI_ISL_1163700	LABCOVID_HCPA	LABRESIS_HCPA	Martins AF, Wink PL, Volpato F, Rosset C, de Paris F, Monteiro F, Zavascki AP, Barth AL
EPI_ISL_1163896, EPI_ISL_1163897, EPI_ISL_1163898, EPI_ISL_1163899, EPI_ISL_1163900, EPI_ISL_1163901, EPI_ISL_1163902, EPI_ISL_1163903, EPI_ISL_1163904, EPI_ISL_1163905, EPI_ISL_1163906, EPI_ISL_1163907, EPI_ISL_1163908, EPI_ISL_1163909			
see above	IZSM	IZSM-U.O.C. Virologia	Maurizio Viscardi, Lorena Cardillo, Luigi Atripaldi,Giovanna Fusco, Esterina De Carlo, Antonio Limone
EPI_ISL_1164932, EPI_ISL_1164933	B.J. Medical College and Civil hospital	Indian Council of Medical Research-National Institute of Virology, Microbial Containment Complex	Pragya D. Yadav, Disha Patel
EPI_ISL_1165082, EPI_ISL_1165083	Medical Ain Shams Research Institute (MASRI), Ain Shams University	Medical Ain Shams Research Institute (MASRI), Ain Shams University	Hesham Elghazaly, Sara Hassan Agwa, Hala Hafez , Fatma Ebied, Manal Hamdy Elsaid, Mohamed Elhadidi, Aya Mohamed , Shimaa Moustafa, Reham Kassab, Iman Foda , Samia Abdou Girgis, Hoda Ezz Elarab, Hagar Elshora, Osama Mansour.
EPI_ISL_1165088, EPI_ISL_1165089, EPI_ISL_1165090, EPI_ISL_1165091, EPI_ISL_1165092	Government Medical College, Patiala, Punjab	Indian Council of Medical Research-National Institute of Virology, Microbial Containment Complex	Pragya D. Yadav, Rupinder Bakshi
EPI_ISL_1180182, EPI_ISL_1180183, EPI_ISL_1180184, EPI_ISL_1180185	Dept. of Microbiology, All India Institute of Medical Sciences, Jodhpur	Indian Council of Medical Research-National Institute of Virology, Microbial Containment Complex	Pragya D. Yadav, Vijaylakshmi Nag
EPI_ISL_1180188, EPI_ISL_1180189, EPI_ISL_1180190, EPI_ISL_1180191, EPI_ISL_1180192, EPI_ISL_1180193, EPI_ISL_1180194	VRDL, GMC, Haldwani , Nainital	Indian Council of Medical Research-National Institute of Virology, Microbial Containment Complex	Pragya D. Yadav, Vinita Rawat
EPI_ISL_1180935	LHUB-ULB	LHUB-ULB	Charlotte Michel, Olivier Vandenberg, Marie Hallin
EPI_ISL_1181614	Laboratorio Central de Saude Publica do Estado do Parana (LACEN-PR)	Laboratory of Respiratory Viruses and Measles, Oswaldo Cruz Institute, FIOCRUZ	Paola Resende, Luciana Appolinario, Fernando Motta, Ana Carolina Paixao, Ana Carolina Mendonca, Alice Sampaio Rocha, Renata Serrano Lopes, Maria do Carmo Debur, Irina Nastassja Riediger, Marilda Siqueira on behalf of the Fiocruz COVID-19 Genomic Surveillance Network
EPI_ISL_1182103, EPI_ISL_1182104	Adolfo Lutz Institute Santos Regional Center Brazil	Retrovirus Laboratory Adolfo Lutz Institute	Gabriela Bastos Cabral, Giselle I S Lopez-Lopes, Cintia Ahagon, Audrey Cilli, Paula Morena Guimaraes, Igor Mohamed Hussein, Luis Brigido
EPI_ISL_1190402	Institute of Life Sciences	Institute of Life Sciences	Sanchari Chatterjee, Eshna Laha, Ankita Datey, Saikat De, Supriya Suman Keshry, Soumyajit Ghosh, Arup Ghosh, Atimukta Jha, Safal Rajesh Walia, Sunil Raghav, Amol Suryawanshi, Rupesh Dash, Shantibhushan Senapati, Tushar K. Beuria, Rajeeb Swain, Gulam Hussain Syed, Punit Prasad, Soma Chattopadhyay ,Odisha COVID-19 Study Group, ILS COVID-19 Team and Ajay Parida.
EPI_ISL_1192100, EPI_ISL_1192114, EPI_ISL_1192115, EPI_ISL_1192116, EPI_ISL_1192117, EPI_ISL_1192118, EPI_ISL_1192119, EPI_ISL_1192120	Sri Venkateswara Institute of Medical Sciences SVIMS, Tirupati	Indian Council of Medical Research-National Institute of Virology, Microbial Containment Complex	Pragya D. Yadav, Usha Kalawat
EPI_ISL_1192149, EPI_ISL_1192150	Viral Research and Diagnostic Laboratory, Dr. R.P. Govt. Medical College,Kangra	Indian Council of Medical Research-National Institute of Virology, Microbial Containment Complex	Pragya D. Yadav, S.C. Jaryal
EPI_ISL_1196305	Institute of Life Sciences	Institute of Life Sciences	Sanchari Chatterjee, Eshna Laha, Ankita Datey, Saikat De, Supriya Suman Keshry, Soumyajit Ghosh, Arup Ghosh, Atimukta Jha, Safal Rajesh Walia, Sunil Raghav, Amol Suryawanshi, Rupesh Dash, Shantibhushan Senapati, Tushar K. Beuria, Rajeeb Swain, Gulam Hussain Syed, Punit Prasad, Soma Chattopadhyay ,Odisha COVID-19 Study Group, ILS COVID-19 Team and Ajay Parida.
EPI_ISL_1196930	Institute for Medical Research, Infectious Disease Research Centre, National Institutes of Health, Ministry of Health Malaysia	Institute for Medical Research, Infectious Disease Research Centre, National Institutes of Health, Ministry of Health Malaysia	Suppiah J, Kamel K, Mohd Zawawi Z, Ramly N, Robert F, Thayan R
EPI_ISL_1201883	LACEN do Mato Grosso do Sul	Instituto Adolfo Lutz, Interdiciplinary Procedures Center, Strategic Laboratory	Claudio Tavares Sacchi, Claudia Regina Gonçalves, Erica Valessa Ramos Gomes, Karoline Rodrigues Campos, Caio Vinicius Dias Lopes
EPI_ISL_1231311, EPI_ISL_1231312, EPI_ISL_1231313	Depratment of Microbiology, Viral Research and Diagnostic Laboratory	Indian Council of Medical Research-National Institute of Virology, Microbial Containment Complex	Pragya D. Yadav, Tapan Majumdar
EPI_ISL_1251267	Sri Venkateswara Institute of Medical Sciences SVIMS, Tirupati	Indian Council of Medical Research-National Institute of Virology, Microbial Containment Complex	Pragya D. Yadav, Usha Kalawat
EPI_ISL_1259400, EPI_ISL_1260834, EPI_ISL_1260836	BSL-3 Lab, National Institute for Viral Disease Control and Prevention, Chinese Center for Disease Control and Prevention	BSL-3 Lab, National Institute for Viral Disease Control and Prevention, Chinese Center for Disease Control and Prevention	Peipei Liu, Xiang Zhao, Shumei Zou, Jing Zhang, Zhixiao Chen, Yuchao Wu, Dayan Wang, Jun Han, George F. Gao, Guizhen Wu
EPI_ISL_1289435, EPI_ISL_1289436, EPI_ISL_1289437, EPI_ISL_1289438, EPI_ISL_1289439, EPI_ISL_1289440, EPI_ISL_1289441, EPI_ISL_1289442, EPI_ISL_1289443, EPI_ISL_1289444, EPI_ISL_1289445, EPI_ISL_1289446			
see above	Department of Health Technology and Informatics, The Hong Kong Polytechnic University	Department of Health Technology and Informatics, The Hong Kong Polytechnic University	Gilman Kit-Hang Siu, Lam-Kwong Lee, Kenneth Siu-Sing Leung, Jake Siu-Lun Leung, Timothy Ting-Leung Ng, Chloe Toi-Mei Chan, Kingsley King-Gee Tam, Hiu-Yin Lao, Denise Sze-Hang Wong, Alan Ka-Lun Wu, Miranda Chong-Yee Yau, Yvette Wai-Man Lai, Kitty Sau-Chun Fung, Sandy Ka-Yee Chau, Barry Kin-Chung Wong, Wing-Kin To, Kristine Luk, Alex Yat-Man Ho, Tak-Lun Que, Kam-Tong Yip, Wing Cheong Yam, David Ho-Keung Shum, Shea Ping Yip
EPI_ISL_1293351	Institut National d'Hygiène (INH)	Unité Mixte Internationale TransVIHMI (UMI 233 IRD - U1175 INSERM - Université de Montpellier)IRD (Institut de recherche pour le développement)	Mounerou SALOU, Christelle BUTEL, Wembo A. HALATOKO,Issaka Maman, Abla A. KONOU, Amivi EHLAN, Adodo SADJI, Kokou TEGUENI,Sidonie A.M.KAGNISSODE, Akoélé SILIADIN, Alassane OURO-MEDELI, Messanh DOUFFAN,Déléma MABA,Sika DOSSIM, Améyo DORKENOO, Mireille PRINCE-DAVID,Anoumou DAGNRA,Laetitia SERRANO,Ahidjo AYOUBA,Eric DELAPORTE, Martine PEETERS
EPI_ISL_1301550	Instituto Nacional de Enfermedades Respiratorias (INER)	Instituto de Biotecnología de la UNAM	Authors from IBT, IMSS, InDRE and INER (in alphabetical order): Carlos F. Arias, Santiago Ávila-Ríos, Gisela Barrera-Badillo, Eduardo Becerril-Vargas, Celia Boukadida, Natividad Cruz-Ortiz, Larissa Fernandes-Matano, Ricardo Grande, Lucia Hernandez-Rivas, Alejandra Hernández-Terán, Pavel Isa, Irma Lopez-Martinez, José Arturo Martínez-Orozco, Margarita Matías-Florentino, Fidencio Mejía-Nepomuceno, Edgar Mendieta-Condado, Mario Mújica-Sánchez, José Esteban Muñoz-Medina, Tatiana Nunez-Garcia, Luis Alberto Ochoa-Carrera, Hector Esteban Paz-Juárez, Francisco Pulido, José Ernesto Ramírez-González, Alma Rincón-Rubio, Teresita Rojas-Mendoza, Jorge Salas-Hernández, Alejandro Sanchez-Flores, Clara Esperanza Santacruz-Tinoco, Andrea Santos Coy-Arechavaleta, Blanca Taboada, Gloria Vazquez, Joel Armando Vázquez-Pérez, Jerome Jean Verleyen
EPI_ISL_1301552, EPI_ISL_1301553	Laboratorio Central de Epidemiología IMSS	Instituto de Biotecnología de la UNAM	Authors from IBT, IMSS, InDRE and INER (in alphabetical order): Carlos F. Arias, Santiago Ávila-Ríos, Gisela Barrera-Badillo, Eduardo Becerril-Vargas, Celia

			Boukadida, Natividad Cruz-Ortiz, Larissa Fernandes-Matano, Ricardo Grande, Lucia Hernandez-Rivas, Alejandra Hernández-Terán, Pavel Isa, Irma Lopez-Martinez, José Arturo Martínez-Orozco, Margarita Matías-Florentino, Fidencio Mejía-Nepomuceno, Edgar Mendieta-Condado, Mario Mújica-Sánchez, José Esteban Muñoz-Medina, Tatiana Nunez-Garcia, Luis Alberto Ochoa-Carrera, Hector Esteban Paz-Juárez, Francisco Pulido, José Ernesto Ramírez-González, Alma Rincón-Rubio, Teresita Rojas-Mendoza, Jorge Salas-Hernández, Alejandro Sanchez-Flores, Clara Esperanza Santacruz-Tinoco, Andrea Santos Coy-Arechavaleta, Blanca Taboada, Gloria Vazquez, Joel Armando Vázquez-Pérez, Jerome Jean Verleyen
EPI_ISL_1301575, EPI_ISL_1301576, EPI_ISL_1301577, EPI_ISL_1301578, EPI_ISL_1301579, EPI_ISL_1301580, EPI_ISL_1301581, EPI_ISL_1301582, EPI_ISL_1301583, EPI_ISL_1301584	Instituto de Diagnostico y Referencia Epidemiologicos InDRE_RNLSP	Instituto de Biotecnología de la UNAM	Authors from IBT, IMSS, InDRE and INER (in alphabetical order): Carlos F. Arias, Santiago Ávila-Ríos, Gisela Barrera-Badillo, Eduardo Becerril-Vargas, Celia Boukadida, Natividad Cruz-Ortiz, Larissa Fernandes-Matano, Ricardo Grande, Lucia Hernandez-Rivas, Alejandra Hernández-Terán, Pavel Isa, Irma Lopez-Martinez, José Arturo Martínez-Orozco, Margarita Matías-Florentino, Fidencio Mejía-Nepomuceno, Edgar Mendieta-Condado, Mario Mújica-Sánchez, José Esteban Muñoz-Medina, Tatiana Nunez-Garcia, Luis Alberto Ochoa-Carrera, Hector Esteban Paz-Juárez, Francisco Pulido, José Ernesto Ramírez-González, Alma Rincón-Rubio, Teresita Rojas-Mendoza, Jorge Salas-Hernández, Alejandro Sanchez-Flores, Clara Esperanza Santacruz-Tinoco, Andrea Santos Coy-Arechavaleta, Blanca Taboada, Gloria Vazquez, Joel Armando Vázquez-Pérez, Jerome Jean Verleyen
EPI_ISL_1301593, EPI_ISL_1301594, EPI_ISL_1301595, EPI_ISL_1301596	Laboratorio Central de Epidemiología IMSS	Instituto de Biotecnología de la UNAM	Authors from IBT, IMSS, InDRE and INER (in alphabetical order): Carlos F. Arias, Santiago Ávila-Ríos, Gisela Barrera-Badillo, Eduardo Becerril-Vargas, Celia Boukadida, Natividad Cruz-Ortiz, Larissa Fernandes-Matano, Ricardo Grande, Lucia Hernandez-Rivas, Alejandra Hernández-Terán, Pavel Isa, Irma Lopez-Martinez, José Arturo Martínez-Orozco, Margarita Matías-Florentino, Fidencio Mejía-Nepomuceno, Edgar Mendieta-Condado, Mario Mújica-Sánchez, José Esteban Muñoz-Medina, Tatiana Nunez-Garcia, Luis Alberto Ochoa-Carrera, Hector Esteban Paz-Juárez, Francisco Pulido, José Ernesto Ramírez-González, Alma Rincón-Rubio, Teresita Rojas-Mendoza, Jorge Salas-Hernández, Alejandro Sanchez-Flores, Clara Esperanza Santacruz-Tinoco, Andrea Santos Coy-Arechavaleta, Blanca Taboada, Gloria Vazquez, Joel Armando Vázquez-Pérez, Jerome Jean Verleyen
EPI_ISL_1301598, EPI_ISL_1301600, EPI_ISL_1301605, EPI_ISL_1301606, EPI_ISL_1301607, EPI_ISL_1301608, EPI_ISL_1301609, EPI_ISL_1301610, EPI_ISL_1301611, EPI_ISL_1301612, EPI_ISL_1301613, EPI_ISL_1301614, EPI_ISL_1301615, EPI_ISL_1301616, EPI_ISL_1301617			
see above	Instituto Nacional de Enfermedades Respiratorias (INER)	Instituto de Biotecnología de la UNAM	Authors from IBT, IMSS, InDRE and INER (in alphabetical order): Carlos F. Arias, Santiago Ávila-Ríos, Gisela Barrera-Badillo, Eduardo Becerril-Vargas, Celia Boukadida, Natividad Cruz-Ortiz, Larissa Fernandes-Matano, Ricardo Grande, Lucia Hernandez-Rivas, Alejandra Hernández-Terán, Pavel Isa, Irma Lopez-Martinez, José Arturo Martínez-Orozco, Margarita Matías-Florentino, Fidencio Mejía-Nepomuceno, Edgar Mendieta-Condado, Mario Mújica-Sánchez, José Esteban Muñoz-Medina, Tatiana Nunez-Garcia, Luis Alberto Ochoa-Carrera, Hector Esteban Paz-Juárez, Francisco Pulido, José Ernesto Ramírez-González, Alma Rincón-Rubio, Teresita Rojas-Mendoza, Jorge Salas-Hernández, Alejandro Sanchez-Flores, Clara Esperanza Santacruz-Tinoco, Andrea Santos Coy-Arechavaleta, Blanca Taboada, Gloria Vazquez, Joel Armando Vázquez-Pérez, Jerome Jean Verleyen
EPI_ISL_1301620, EPI_ISL_1301621, EPI_ISL_1301622, EPI_ISL_1301623, EPI_ISL_1301624, EPI_ISL_1301625, EPI_ISL_1301626, EPI_ISL_1301627, EPI_ISL_1301628, EPI_ISL_1301629, EPI_ISL_1301630, EPI_ISL_1301631, EPI_ISL_1301632, EPI_ISL_1301633, EPI_ISL_1301634, EPI_ISL_1301635, EPI_ISL_1301636, EPI_ISL_1301637, EPI_ISL_1301638, EPI_ISL_1301639, EPI_ISL_1301640, EPI_ISL_1301641, EPI_ISL_1301642, EPI_ISL_1301643, EPI_ISL_1301644, EPI_ISL_1301645, EPI_ISL_1301646, EPI_ISL_1301647, EPI_ISL_1301648, EPI_ISL_1301649, EPI_ISL_1301650, EPI_ISL_1301651, EPI_ISL_1301652, EPI_ISL_1301653, EPI_ISL_1301654, EPI_ISL_1301655, EPI_ISL_1301656, EPI_ISL_1301657, EPI_ISL_1301658, EPI_ISL_1301659, EPI_ISL_1301660, EPI_ISL_1301661, EPI_ISL_1301662, EPI_ISL_1301663, EPI_ISL_1301664, EPI_ISL_1301665, EPI_ISL_1301666, EPI_ISL_1301667, EPI_ISL_1301668, EPI_ISL_1301669, EPI_ISL_1301670, EPI_ISL_1301671			
see above	Laboratorio Central de Epidemiología IMSS	Instituto de Biotecnología de la UNAM	Authors from IBT, IMSS, InDRE and INER (in alphabetical order): Carlos F. Arias, Santiago Ávila-Ríos, Gisela Barrera-Badillo, Eduardo Becerril-Vargas, Celia Boukadida, Natividad Cruz-Ortiz, Larissa Fernandes-Matano, Ricardo Grande, Lucia Hernandez-Rivas, Alejandra Hernández-Terán, Pavel Isa, Irma Lopez-Martinez, José Arturo Martínez-Orozco, Margarita Matías-Florentino, Fidencio Mejía-Nepomuceno, Edgar Mendieta-Condado, Mario Mújica-Sánchez, José Esteban Muñoz-Medina, Tatiana Nunez-Garcia, Luis Alberto Ochoa-Carrera, Hector Esteban Paz-Juárez, Francisco Pulido, José Ernesto Ramírez-González, Alma Rincón-Rubio, Teresita Rojas-Mendoza, Jorge Salas-Hernández, Alejandro Sanchez-Flores, Clara Esperanza Santacruz-Tinoco, Andrea Santos Coy-Arechavaleta, Blanca Taboada, Gloria Vazquez, Joel Armando Vázquez-Pérez, Jerome Jean Verleyen
EPI_ISL_1301679, EPI_ISL_1301680, EPI_ISL_1301681, EPI_ISL_1301682, EPI_ISL_1301684, EPI_ISL_1301685, EPI_ISL_1301686, EPI_ISL_1301687, EPI_ISL_1301690, EPI_ISL_1301709	Instituto de Diagnostico y Referencia Epidemiologicos InDRE_RNLSP	Instituto de Biotecnología de la UNAM	Authors from IBT, IMSS, InDRE and INER (in alphabetical order): Carlos F. Arias, Santiago Ávila-Ríos, Gisela Barrera-Badillo, Eduardo Becerril-Vargas, Celia Boukadida, Natividad Cruz-Ortiz, Larissa Fernandes-Matano, Ricardo Grande, Lucia Hernandez-Rivas, Alejandra Hernández-Terán, Pavel Isa, Irma Lopez-Martinez, José Arturo Martínez-Orozco, Margarita Matías-Florentino, Fidencio Mejía-Nepomuceno, Edgar Mendieta-Condado, Mario Mújica-Sánchez, José Esteban Muñoz-Medina, Tatiana Nunez-Garcia, Luis Alberto Ochoa-Carrera, Hector Esteban Paz-Juárez, Francisco Pulido, José Ernesto Ramírez-González, Alma Rincón-Rubio, Teresita Rojas-Mendoza, Jorge Salas-Hernández, Alejandro Sanchez-Flores, Clara Esperanza Santacruz-Tinoco, Andrea Santos Coy-Arechavaleta, Blanca Taboada, Gloria Vazquez, Joel Armando Vázquez-Pérez, Jerome Jean Verleyen
EPI_ISL_1301714, EPI_ISL_1301715, EPI_ISL_1301716	Laboratorio Central de Epidemiología IMSS	Instituto de Biotecnología de la UNAM	Authors from IBT, IMSS, InDRE and INER (in alphabetical order): Carlos F. Arias, Santiago Ávila-Ríos, Gisela Barrera-Badillo, Eduardo Becerril-Vargas, Celia Boukadida, Natividad Cruz-Ortiz, Larissa Fernandes-Matano, Ricardo Grande, Lucia Hernandez-Rivas, Alejandra Hernández-Terán, Pavel Isa, Irma Lopez-Martinez, José Arturo Martínez-Orozco, Margarita Matías-Florentino, Fidencio Mejía-Nepomuceno, Edgar Mendieta-Condado, Mario Mújica-Sánchez, José Esteban Muñoz-Medina, Tatiana Nunez-Garcia, Luis Alberto Ochoa-Carrera, Hector Esteban Paz-Juárez, Francisco Pulido, José Ernesto Ramírez-González, Alma Rincón-Rubio, Teresita Rojas-Mendoza, Jorge Salas-Hernández, Alejandro Sanchez-Flores, Clara Esperanza Santacruz-Tinoco, Andrea Santos Coy-Arechavaleta, Blanca Taboada, Gloria Vazquez, Joel Armando Vázquez-Pérez, Jerome Jean Verleyen
EPI_ISL_1301717, EPI_ISL_1301718, EPI_ISL_1301719, EPI_ISL_1301720, EPI_ISL_1301721, EPI_ISL_1301722, EPI_ISL_1301723, EPI_ISL_1301724, EPI_ISL_1301731	Instituto Nacional de Enfermedades Respiratorias (INER)	Instituto de Biotecnología de la UNAM	Authors from IBT, IMSS, InDRE and INER (in alphabetical order): Carlos F. Arias, Santiago Ávila-Ríos, Gisela Barrera-Badillo, Eduardo Becerril-Vargas, Celia Boukadida, Natividad Cruz-Ortiz, Larissa Fernandes-Matano, Ricardo Grande, Lucia Hernandez-Rivas, Alejandra Hernández-Terán, Pavel Isa, Irma Lopez-Martinez, José Arturo Martínez-Orozco, Margarita Matías-Florentino, Fidencio Mejía-Nepomuceno, Edgar Mendieta-Condado, Mario Mújica-Sánchez, José Esteban Muñoz-Medina, Tatiana Nunez-Garcia, Luis Alberto Ochoa-Carrera, Hector Esteban Paz-Juárez, Francisco Pulido, José Ernesto Ramírez-González, Alma Rincón-Rubio, Teresita Rojas-Mendoza, Jorge Salas-Hernández, Alejandro Sanchez-Flores, Clara Esperanza Santacruz-Tinoco, Andrea Santos Coy-Arechavaleta, Blanca Taboada, Gloria Vazquez, Joel Armando Vázquez-Pérez, Jerome Jean Verleyen
EPI_ISL_1303031	Institut National d'Hygiène (INH)	Unité Mixte Internationale TransVIHMI (UMI 233 IRD - U1175 INSERM - Université de Montpellier)IRD (Institut de recherche pour le développement)	Mounerou SALOU, Christelle BUTEL, Wembo A. HALATOKO,Issaka Maman, Abla A. KONOU, Amivi EHLAN, Adodo SADJI, Kokou TEGUENI,Sidonie A.M.KAGNISSODE, Akoélé SILIADIN, Alassane OURO-MEDELI, Messanh DOUFFAN,Déléma MABA,Sika DOSSIM, Améyo DORKENOO, Mireille PRINCE-DAVID,Anoumou DAGNRA,Laetitia SERRANO,Ahidjo AYOUBA,Eric DELAPORTE, Martine PEETERS
EPI_ISL_1315064	Armed Forces Medical Research Laboratories and Blood Bank, Egypt	Department of Microbial Biotechnology, Genetic Engineering Division, National Research Centre,	Reham Dawood, Mohamed Khalifa, Ahmed Taha, Ayman Farghaly, Ahmed Elsayed, Mohamed Shemis
EPI_ISL_1315065	Armed Forces Medical Research Laboratories and Blood Bank, Egypt	Department of Microbial Biotechnology, Genetic Engineering Division, National Research Centre	Reham Dawood, Mohamed Khalifa, Ahmed Taha, Ayman Farghaly, Ahmed Elsayed, Mohamed Shemis
EPI_ISL_1321604	Institut National d'Hygiène (INH)	Unité Mixte Internationale TransVIHMI (UMI 233 IRD - U1175 INSERM - Université de Montpellier)IRD (Institut de recherche pour le développement)	Mounerou SALOU, Christelle BUTEL, Wembo A. HALATOKO,Issaka Maman, Abla A. KONOU, Amivi EHLAN, Adodo SADJI, Kokou TEGUENI,Sidonie A.M.KAGNISSODE, Akoélé SILIADIN, Alassane OURO-MEDELI, Messanh DOUFFAN,Déléma MABA,Sika DOSSIM, Améyo DORKENOO, Mireille PRINCE-DAVID,Anoumou DAGNRA,Laetitia SERRANO,Ahidjo AYOUBA,Eric DELAPORTE, Martine PEETERS
EPI_ISL_1340764	Departamento de Virologia, Laboratorio Central de Salud Pública, Avenida Venezuela y Teniente Escurra, Asunción, Paraguay	Laboratory of Respiratory Viruses and Measles, Oswaldo Cruz Institute, FIOCRUZ	Paola Resende, Cynthia Vazquez, Luciana Appolinario, Fernando Motta, Anna Carolina Paixao, Ana Carolina Mendonca, Alice Sampaio Rocha, Renata Serrano Lopes, Marilda Siqueira on behalf of the Fiocruz COVID-19 Genomic Surveillance Network
EPI_ISL_1379197	Department of Molecular and Translational Medicine, Section of Microbiology, University of Brescia, ASST Spedali Civili, Brescia	Department of Molecular and Translational Medicine, Section of Microbiology, University of Brescia, ASST Spedali Civili, Brescia	Francesca Caccuri, Alberto Zani, Serena Messali, Antonella Bugatti, Giovanni Campisi, Federica Filippini, Erika Scaltriti, Simona Fiorentini, Arnaldo Caruso
EPI_ISL_1398937	Emam Ali Hospital	Razi Vaccine and Serum Research Institute	Amir Kaffashi, Jiabin Huang, S Reza Banihashemi, Mohammad Hossein Fallah Mehrabadi, Mohsen Lotfi, Morteza Taghizadeh, Akbar Khorasani, Mohsen Bashashati, Sayed Hamidreza Mozhgan
EPI_ISL_1404615	Institut National d'Hygiène	Unité Mixte Internationale TransVIHMI (UMI 233 IRD - U1175 INSERM - Université de Montpellier) IRD (Institut de recherche pour le développement)	Mounerou SALOU, Christelle BUTEL, Wembo A. HALATOKO,Issaka Maman, Abla A. KONOU, Amivi EHLAN, Adodo SADJI, Kokou TEGUENI,Sidonie A.M.KAGNISSODE, Akoélé SILIADIN, Alassane OURO-MEDELI, Messanh DOUFFAN,Déléma MABA,Sika DOSSIM, Améyo DORKENOO, Mireille PRINCE-DAVID,Anoumou DAGNRA,Laetitia SERRANO,Ahidjo AYOUBA,Eric DELAPORTE, Martine PEETERS
EPI_ISL_1406144, EPI_ISL_1406177	Institut National d'Hygiène (INH)	Unité Mixte Internationale TransVIHMI (UMI 233 IRD - U1175 INSERM - Université de Montpellier)IRD (Institut de recherche pour le développement)	Mounerou SALOU, Christelle BUTEL, Wembo A. HALATOKO,Issaka Maman, Abla A. KONOU, Amivi EHLAN, Adodo SADJI, Kokou TEGUENI,Sidonie A.M.KAGNISSODE, Akoélé SILIADIN, Alassane OURO-MEDELI, Messanh DOUFFAN,Déléma MABA,Sika DOSSIM, Améyo DORKENOO, Mireille PRINCE-DAVID,Anoumou DAGNRA,Laetitia SERRANO,Ahidjo AYOUBA,Eric DELAPORTE, Martine PEETERS
EPI_ISL_1415293, EPI_ISL_1415425, EPI_ISL_1415428	Institut National d'hygiène	"Unité Mixte Internationale TransVIHMI (UMI 233 IRD - U1175 INSERM - Université de Montpellier) IRD (Institut de	Mounerou SALOU, Christelle BUTEL, Wembo A. HALATOKO,Issaka Maman, Abla A. KONOU, Amivi EHLAN, Adodo SADJI, Kokou TEGUENI,Sidonie A.M.KAGNISSODE, Akoélé SILIADIN, Alassane OURO-MEDELI, Messanh DOUFFAN,Déléma MABA,Sika DOSSIM, Améyo DORKENOO, Mireille

		recherche pour le développement)"	PRINCE-DAVID,Anoumou DAGNRA,Laetitia SERRANO,Ahidjo AYOUBA,Eric DELAPORTE, Martine PEETERS
EPI_ISL_1439647, EPI_ISL_1440879	Institut National d'Hygiène	Unité Mixte Internationale TransVIHMI (UMI 233 IRD - U1175 INSERM - Université de Montpellier) IRD (Institut de recherche pour le développement)	Mounerou SALOU, Christelle BUTEL, Wembo A. HALATOKO,Issaka Maman, Abla A. KONOU, Amivi EHLAN, Adodo SADJI, Kokou TEGUENI,Sidonie A.M.KAGNISSODE, Akoélé SILIADIN, Alassane OURO-MEDELI, Messanh DOUFFAN,Déléma MABA,Sika DOSSIM, Améyo DORKENOO, Mireille PRINCE-DAVID,Anoumou DAGNRA,Laetitia SERRANO,Ahidjo AYOUBA,Eric DELAPORTE, Martine PEETERS
EPI_ISL_1442993, EPI_ISL_1443000, EPI_ISL_1443001	Institut National d'hygiène	"Unité Mixte Internationale TransVIHMI (UMI 233 IRD - U1175 INSERM - Université de Montpellier) IRD (Institut de recherche pour le développement)"	Mounerou SALOU, Christelle BUTEL, Wembo A. HALATOKO,Issaka Maman, Abla A. KONOU, Amivi EHLAN, Adodo SADJI, Kokou TEGUENI,Sidonie A.M.KAGNISSODE, Akoélé SILIADIN, Alassane OURO-MEDELI, Messanh DOUFFAN,Déléma MABA,Sika DOSSIM, Améyo DORKENOO, Mireille PRINCE-DAVID,Anoumou DAGNRA,Laetitia SERRANO,Ahidjo AYOUBA,Eric DELAPORTE, Martine PEETERS
EPI_ISL_1447336	Diagnostic and Research Center of Infectious Diseases, Medical Faculty, Andalas University	Diagnostic and Research Center of Infectious Diseases, Medical Faculty, Andalas University	Andani Eka Putra, Syafrizayanti, Ikhwan R. Sudji, Linosefa, Dede Rahman Agustian, Ayu Novita Trisnawati, Siskalil Fahma, Nia Ayuni Putri, Sekar Asri Tresnaningtyas, SM Rezvi, Fauzul Azhim, Dessy Arisanty, Gestina Aliska, Desmawati, Nita Afriani ,Syandrez Prima Putra, Juane Plantika Menra, Yolani Syaputri, Mutia Lailani
EPI_ISL_1469668	Hospital Unimed Vale do Caí	Epiclin	Fernando Hayashi Sant'Anna, Ana Paula Muterle, Janira Prichula, Juliana Comerlato, Carolina Comerlato, Eliana Márcia Da Ros Wendland
EPI_ISL_1469752	Fundação Hospitalar de Sapucaia do Sul	Epiclin	Fernando Hayashi Sant'Anna, Ana Paula Muterle, Janira Prichula, Juliana Comerlato, Carolina Comerlato, Eliana Márcia Da Ros Wendland
EPI_ISL_1469799	Hospital Regina	Epiclin	Fernando Hayashi Sant'Anna, Ana Paula Muterle, Janira Prichula, Juliana Comerlato, Carolina Comerlato, Eliana Márcia Da Ros Wendland
EPI_ISL_1469959	CUB Hopital Erasme Laboratoire d'Anatomie Pathologique	CUB Hopital Erasme Laboratoire d'Anatomie Pathologique	Prof. Isabelle Salmon, Dr. Nicky D'Haene
EPI_ISL_1499504	Laboratorio di Microbiologia e Virologia, Università Vita-Salute San Raffaele, Milano	Laboratorio di Microbiologia e Virologia, Università Vita-Salute San Raffaele, Milano	Roberto Ferrarese, Roberta Antonia Diotti, Elena Criscuolo, Matteo Castelli, Nicasio Mancini, Massimo Clementi
EPI_ISL_1499572	Laboratorio di Microbiologia e Virologia, Università Vita-Salute San Raffaele, Milano	Laboratorio di Microbiologia e Virologia, Università Vita-Salute San Raffaele, Milano	Matteo Castelli, Elena Criscuolo, Roberta Antonia Diotti, Roberto Ferrarese, Nicasio Mancini, Massimo Clementi, Nicola Clementi on behalf of SARS-CoV-2 ITALIAN RESEARCH ENTERPRISE-(SCIRE) Collaborative Group
EPI_ISL_1499593	Laboratorio di Microbiologia e Virologia, Università Vita-Salute San Raffaele, Milan	Laboratorio di Microbiologia e Virologia, Università Vita-Salute San Raffaele, Milan	Elena Criscuolo, Roberta Antonia Diotti, Roberto Ferrarese, Matteo Castelli, Massimo Clementi, Nicola Clementi, Nicasio Mancini on behalf of SARS-CoV-2 ITALIAN RESEARCH ENTERPRISE-(SCIRE) Collaborative Group
EPI_ISL_1502818, EPI_ISL_1502819, EPI_ISL_1502820	Hospital Regional Anita Moreno Los Santos	Gorgas Memorial Laboratory of Health Studies	Gonzalez Claudia, Best Dayana, Espino Juliee, Leyda Abrego, Moreno Ambar, Oris Chavarria, Jessica Gondola, Marlenne Castillo, Ortiz Alma, Castillo Jorge, Moreno Brechla, Franco Danilo, Lopez-Verges Sandra, Martinez Alexander
EPI_ISL_1502821, EPI_ISL_1502822, EPI_ISL_1502823	Hospital Regional Dr. Luis "Chicho" Fábrega	Gorgas Memorial Laboratory of Health Studies	Gonzalez Claudia, Herrera Melissa, Herrera Genarino, Leyda Abrego, Moreno Ambar, Oris Chavarria, Jessica Gondola, Marlenne Castillo, Ortiz Alma, Castillo Jorge, Moreno Brechla, Franco Danilo, Lopez-Verges Sandra, Martinez Alexander
EPI_ISL_1502824	Hospital Regional Anita Moreno Los Santos	Gorgas Memorial Laboratory of Health Studies	Gonzalez Claudia, Best Dayana, Espino Juliee, Leyda Abrego, Moreno Ambar, Oris Chavarria, Jessica Gondola, Marlenne Castillo, Ortiz Alma, Castillo Jorge, Moreno Brechla, Franco Danilo, Lopez-Verges Sandra, Martinez Alexander
EPI_ISL_1502826, EPI_ISL_1502828, EPI_ISL_1502829, EPI_ISL_1502831, EPI_ISL_1502832, EPI_ISL_1502833, EPI_ISL_1502834	Hospital Regional Dr. Luis "Chicho" Fábrega	Gorgas Memorial Laboratory of Health Studies	Gonzalez Claudia, Herrera Melissa, Herrera Genarino, Leyda Abrego, Moreno Ambar, Oris Chavarria, Jessica Gondola, Marlenne Castillo, Ortiz Alma, Castillo Jorge, Moreno Brechla, Franco Danilo, Lopez-Verges Sandra, Martinez Alexander
EPI_ISL_1502836	Hospital Regional Anita Moreno Los Santos	Gorgas Memorial Laboratory of Health Studies	Gonzalez Claudia, Best Dayana, Espino Juliee, Leyda Abrego, Moreno Ambar, Oris Chavarria, Jessica Gondola, Marlenne Castillo, Ortiz Alma, Castillo Jorge, Moreno Brechla, Franco Danilo, Lopez-Verges Sandra, Martinez Alexander
EPI_ISL_1502837, EPI_ISL_1502838, EPI_ISL_1502839, EPI_ISL_1502840, EPI_ISL_1502841	Hospital Regional Dr. Luis "Chicho" Fábrega	Gorgas Memorial Laboratory of Health Studies	Gonzalez Claudia, Herrera Melissa, Herrera Genarino, Leyda Abrego, Moreno Ambar, Oris Chavarria, Jessica Gondola, Marlenne Castillo, Ortiz Alma, Castillo Jorge, Moreno Brechla, Franco Danilo, Lopez-Verges Sandra, Martinez Alexander
EPI_ISL_1502842, EPI_ISL_1502843, EPI_ISL_1502844, EPI_ISL_1502845, EPI_ISL_1502846, EPI_ISL_1502847	Gorgas Memorial Laboratory of Health Studies	Gorgas Memorial Laboratory of Health Studies	Gonzalez Claudia, Leyda Abrego, Moreno Ambar, Oris Chavarria, Jessica Gondola, Marlenne Castillo, Ortiz Alma, Castillo Jorge, Moreno Brechla, Franco Danilo, Lopez-Verges Sandra, Martinez Alexander
EPI_ISL_1510666	University of Rome Tor Vergata: Departm Experim Medicine Chair of Virology	University of Rome Tor Vergata: Departm Experim Medicine Chair of Virology	Maria Concetta Bellocchi
EPI_ISL_1511268	Medicina Genómica	Medicina Genómica	Ana Laura Sánchez-Sandoval, María Eligia Juárez-Torres, Mariana Villalba-De la Peña, Siddhartha Ríos-Zaragoza, Guadalupe Mercedes Lucía Guerrero-Avendaño, Eira Valeria Barrón-Palma
EPI_ISL_1511269, EPI_ISL_1511270	Hospital General de México, Medicina Genómica	Hospital General de México, Medicina Genómica	Ana Laura Sánchez-Sandoval, María Eligia Juárez-Torres, Mariana Villalba-De la Peña, Siddhartha Ríos-Zaragoza, Guadalupe Mercedes Lucía Guerrero-Avendaño, Eira Valeria Barrón-Palma
EPI_ISL_1511641	Laboratorio de Patologia Clinica - UNICAMP	Laboratorio de Estudos de Virus Emergentes	Mariene R. Amorim, William M. Souza, Antonio C. G. Carlos Jr, Daniel A. Toledo-Teixeira, Karina Bispo-dos-Santos, Camila L. Simeoni, Pierina L. Parise, Aline Vieira, Julia Forato, Ingra M. Claro, Luciana S. Mofatto, Natalia S. Brunetti, Emerson S.S. França, Gisele A. Pedroso, Barbara F. N. Carvalho, Tania R. Zaccariotto, Kamila C. S. Krywacz, André S. Vieira, Marcelo A. Mori, Alessandro S. Farias, Maria H. P. Pavan, Luís Felipe Bachur, Luís G. O. Cardoso, Fernando R. Spilki, Ester C. Sabino, Nuno R. Faria, Magnun N. N. Santos, Rodrigo Angerami, Patricia A. F. Leme, Angelica Schreiber, Maria L. Moretti, Fabiana Granja, José Luiz Proenca-Modena
EPI_ISL_1533610	Shiraz University	Shiraz University	Abozar Ghorbani, Hakimeh Ahmadi, Hamed Gouklani, Ebrahim Eftekhar
EPI_ISL_1550878	Instituto Oswaldo Cruz	Laboratorio de Virologia, Faculdade de Medicina, Universidade Federal de Mato Grosso, campus Cuiabá	Maria de Fatima Ferrerira, Renata Dezengrini Slhessarenko, Janeth Aracely Ramirez Pavon, Luciano Nakazato, Valeria Dutra , Rosane Hahn
EPI_ISL_1655937	State Key Laboratory of Emerging Infectious Diseases, The University of Hong Kong	State Key Laboratory of Emerging Infectious Diseases, The University of Hong Kong	Jia Wang, Lifeng Li, Yiu Man Cheung, Huachen Zhu, Yi Guan
EPI_ISL_1678584, EPI_ISL_1690551, EPI_ISL_1694627, EPI_ISL_1694628, EPI_ISL_1694631, EPI_ISL_1694632, EPI_ISL_1694634, EPI_ISL_1695909	Instituto Estadual do Cérebro Paulo Niemayer (IECPN)	Laboratório de Imunofarmacologia	Thiago Moreno Lopes Souza
EPI_ISL_1718321	SCIENSANO National Influenza Centre	PathoSense BV	Nick Vereecke
EPI_ISL_1752476, EPI_ISL_1752480, EPI_ISL_1752484, EPI_ISL_1752495, EPI_ISL_1752496, EPI_ISL_1752497, EPI_ISL_1752498, EPI_ISL_1752499, EPI_ISL_1752500, EPI_ISL_1752501, EPI_ISL_1752502, EPI_ISL_1752503, EPI_ISL_1752504, EPI_ISL_1752505, EPI_ISL_1752506, EPI_ISL_1752507, EPI_ISL_1752508, EPI_ISL_1752509			
see above	LHUB-ULB	LHUB-ULB	Charlotte Michel, Olivier Vandenberg, Marie Hallin
EPI_ISL_1797550, EPI_ISL_1797552, EPI_ISL_1797553, EPI_ISL_1797554, EPI_ISL_1797556, EPI_ISL_1797558, EPI_ISL_1797560, EPI_ISL_1797561, EPI_ISL_1797563, EPI_ISL_1797564, EPI_ISL_1797565, EPI_ISL_1797566, EPI_ISL_1797567, EPI_ISL_1797568, EPI_ISL_1797570, EPI_ISL_1797571, EPI_ISL_1797572, EPI_ISL_1797573, EPI_ISL_1797575, EPI_ISL_1797576, EPI_ISL_1797578, EPI_ISL_1797579, EPI_ISL_1797582, EPI_ISL_1797583, EPI_ISL_1797584, EPI_ISL_1797585, EPI_ISL_1797586, EPI_ISL_1797588, EPI_ISL_1797589, EPI_ISL_1797590, EPI_ISL_1797591, EPI_ISL_1797592, EPI_ISL_1797595, EPI_ISL_1797597, EPI_ISL_1797598, EPI_ISL_1797599, EPI_ISL_1797600, EPI_ISL_1797601			
see above	Medical Center, Aircraft Carrier	IRBA, 2MI	Franck de Laval, Hervé Chaudet, Constance Lacrosse, Joffrey Marchi, Aissata Dia, Gaëtan Texier, Olivier Gorgé, Bakridine Mmadi Mrenda, Vanessa Marbac, Flavie Letois, Anthony Levasseur, Jacques Cobola, Flora Nolent, Fabien Dutasta, Frédéric Janvier, Meynard Jean-Baptiste, Vincent Pommier de Santi
EPI_ISL_1805651	National Center for Communicable Diseases (NCCD) National Influenza Center	National Center for Communicable Diseases (NCCD) National Influenza Center	Naranzul Ts,Bayasgalan N,Khishigmunkh Ch,Seiichiro F,Hideka M,Mina N,Shinji W,Ankhbayar S,Tsogzolmaa G,Darmaa B,Battur L,Nymadawa P
EPI_ISL_1859756	City Clinical Hospital named after S.I. Spasokukotsky of Moscow Healthcare Department, Academic Consortium	FEDERAL RESEARCH AND CLINICAL CENTER OF PHYSICAL-CHEMICAL MEDICINE OF FEDERAL MEDICAL BIOLOGICAL AGENCY, Academic Consortium	Vladislav Babenko, Ramiz Bakhtyev, Vladimir Baklaushev, Larisa Balykova, Pavel Bashkirov, Anna Blagonravova, Daria Boldyreva, Dmitry Fedorov, Ilshat Gafurov, Raushaniya Gaifullina, Yulia Galeeva, Elena Galova, Vadim Govorun, Elena Ilina, Konstantin Ivanov, Daria Kharlampieva, Polina Khromova, Ksenia Klimina, Konstantin Kolontarev, Nadezhda Kolyshkina, Andrey Koritsky, Vyacheslav Kuropatkin, Vasily Lazarev, Alexander Manolov, Valentin Manuvera, Daria

			Matyushkina, Ekaterina Moskaleva, Varvara Musarova, Oleg Ogarkov, Elizaveta Orlova, Alexander Pavlenko, Alla Petrova, Natalia Pozhenko, Dmitry Pushkar, Alexander Rumyantsev, Sergey Rumyantsev, Vladimir Rumyantsev, Lyubov Rychkova, Alexander Samoilov, Irina Shirokova, Vyacheslav Sinkov, Svetlana Solovieva, Polina Tikhonova, Galina Trifonova, Alexander Troitsky, Alexander Tulichev, Yuri Udalov, Anna Varizhuk, Alexander Vasiliev, Vladimir Veselovsky, Alexey Volnukhin, Gaukhar Yusubalieva
EPI_ISL_1910396	Centre Hospitalier Universitaire Clermont-Ferrand	CHU Clermont-Ferrand, service de virologie	Bisseux Maxime, Mirand Audrey, Combes Patricia, Henquell Cécile
EPI_ISL_1972889, EPI_ISL_1972890, EPI_ISL_1972892, EPI_ISL_1972893, EPI_ISL_1972895	National Public Health Laboratory Malaysia	National Public Health Laboratory Malaysia	Noorliza MN, JL Tan, Chong CK, Norhayati R, Yukie C, Norazimah T Rehan SAB Selvanesan S, Hannah PYP, NurulAina MCA, W.NurAfiza WMA Zirwatul AA, Hani MH, Nurulsyahida I, Aziyati O, Ushananthiny R, Kamal HKZ, Mohd AY, YF Ngeow
EPI_ISL_2000551, EPI_ISL_2000552, EPI_ISL_2000553, EPI_ISL_2000554, EPI_ISL_2000555, EPI_ISL_2000556, EPI_ISL_2000557, EPI_ISL_2000558, EPI_ISL_2000559, EPI_ISL_2000560, EPI_ISL_2000561, EPI_ISL_2000562, EPI_ISL_2000564, EPI_ISL_2000565, EPI_ISL_2000566, EPI_ISL_2000567, EPI_ISL_2000570, EPI_ISL_2000571, EPI_ISL_2000572			
see above	Tropical Medicine Institute Pedro Kouri	Respiratory Infections Laboratory	Yahisel Tejero Suarez, Lissette Perez Santos, Yanet Pintos Saavedra, Daylin Mederos, Odalys Valdes, Mirtha Elisa Aguado, Vivian Kouri Cardella
EPI_ISL_2001114, EPI_ISL_2001115, EPI_ISL_2001213, EPI_ISL_2001429, EPI_ISL_2001430, EPI_ISL_2001643, EPI_ISL_2001644, EPI_ISL_2001655, EPI_ISL_2001656, EPI_ISL_2001659	National Public Health Laboratory Malaysia	National Public Health Laboratory Malaysia	Noorliza MN, JL Tan, Chong CK, Norhayati R, Yukie C, Norazimah T Rehan SAB Selvanesan S, Hannah PYP, NurulAina MCA, W.NurAfiza WMA Zirwatul AA, Hani MH, Nurulsyahida I, Aziyati O, Ushananthiny R, Kamal HKZ, Mohd AY, YF Ngeow
EPI_ISL_2009116	National Public Health Laboratory Malaysia	National Public Health Laboratory Malaysia	Noorliza MN, JL Tan, Chong CK, Norhayati R, Yukie C, Norazimah T, Rehan SAB, Selvanesan S, Hannah PYP, NurulAina MCA, W.NurAfiza WMA Zirwatul AA, Hani MH, Nurulsyahida I, Aziyati O, Ushananthiny R, Kamal HKZ, Mohd AY, YF Ngeow
EPI_ISL_2009170, EPI_ISL_2009171, EPI_ISL_2009172, EPI_ISL_2009173	MSK Microbiology Lab	MSK Microbiology Lab	Esther Babady, Tracy McMillen, Krupa Jani
EPI_ISL_2013323, EPI_ISL_2015112, EPI_ISL_2015113, EPI_ISL_2015114, EPI_ISL_2015118, EPI_ISL_2015119, EPI_ISL_2015120, EPI_ISL_2015123, EPI_ISL_2015127, EPI_ISL_2015128, EPI_ISL_2015129, EPI_ISL_2015130, EPI_ISL_2015131, EPI_ISL_2015132, EPI_ISL_2015133, EPI_ISL_2015134, EPI_ISL_2015135, EPI_ISL_2015136, EPI_ISL_2015137, EPI_ISL_2015138			
see above	IU-Cerrahpasa, Cerrahpasa School of Medicine, COVID-19 Lab	IU-Cerrahpasa, Cerrahpasa School of Medicine, COVID-19 Lab	Mert Kuskucu, Yesim Tuyji Tok, Kenan Midilli
EPI_ISL_2134584	Laboratorio de Virologia Molecular / UFRJ	Bioinformatics Laboratory / LNCC	Alice L. Herlinger, Fábio L. Monteiro, Mirela D'arc, Filipe R.R. Moreira, Harrison J. Westgarth, Rafael M. Galliez, Diana Mariani, Luciana J. da Costa, Luiz Almeida, Carolina M. Voloch, Covid19-UFRJ Workgroup, Adriana S.O. Melo, Renato S. Aguiar, André F.A. dos Santos, Terezinha Marta P. P. Castiñeiras, Ana Tereza R. Vasconcelos, Esaú C. João Filho, Claudia C. Escosteguy, Orlando C. Ferreira Jr, Amilcar Tanuri, Luiza M. Higa
EPI_ISL_2138923	Laboratorio HUB -Azienda Ospedaliero Universitaria - AOU - Cagliari	Laboratorio SPOKE Biologia Molecolare -Azienda Ospedaliero Universitaria - AOU - Cagliari	Germano Orrù, Alessandra Scano, Sara Fais, Valentina Medda, Miriam Loddo, Riccardo Cappai, Ferdinando Coghe
EPI_ISL_2138924	Laboratorio HUB -Azienda Ospedaliero Universitaria - AOU - Cagliari	Laboratorio SPOKE Biologia Molecolare -Azienda Ospedaliero Universitaria - AOU - Cagliari	Alessandra Scano, Germano Orrù, Sara Fais, Valentina Medda, Miriam Loddo, Riccardo Cappai, Ferdinando Coghe
EPI_ISL_2140172, EPI_ISL_2140173, EPI_ISL_2140188, EPI_ISL_2140189	Wits VIDA	Wits VIDA	Vicky Baillie, Shabir Madhi et al.
EPI_ISL_2156767	Valenzuela Hope Molecular Laboratory	Philippine Genome Center	Francis A. Tablizo, Kenneth M. Kim, Carlo M. Lapid, Marc Jerrone R. Castro, Maria Sofia L. Yangzon, Elcid Aaron R. Pangilinan, Benedict A. Maralit, Marc Edsel C. Ayes, Eva Maria Cutiongco-de la Paz, Alethea R. de Guzman, Jan Michael C. Yap, Jo-Hannah S. Llames, Sheila Mae M. Araiza, Kris P. Punayan, Irish Coleen A. Asin, Candice Francheska B. Tambaoan, Asia Louisa U. Chong, Karol Sophia Agape R. Padilla, Rianna Patricia S. Cruz, El King D. Morado, Joshua Gregor A. Dizon, Razel Nikka M. Hao, Arianne A. Zamora, Devon Ray Pacial, Juan Antonio R. Magalang, Marissa Alejandria, Celia Carlos, Anna Ong-Lim, Edsel Maurice Salvaña, John Q. Wong, Jaime C. Montoya, Maria Rosario Singh-Vergeire and Cynthia P. Saloma
EPI_ISL_2158598, EPI_ISL_2158600	Laboratório de Biologia Molecular Jean Piaget	MRCG at LSHTM, Genomics lab	Aladje Balde, Abdul Karim Sesay, Abdoulie Kanteh, Bakary Sanyang, Simão Tchuda Bióté, Rui Inndi, Adul Candé, Faatu Cassama, Milanca Agostinho Cá, Rei José Pereira, Erica Luis Maria Magalhães, Aicha Balde, Bubacar Delgado Pinto Embaló, Edmira Maria da Costa, Paulina Joãozinho da Costa Jarra Manneh, Mariama Kujabi, Dabiri Damilari, Sainabou Laye Ndure
EPI_ISL_2254725, EPI_ISL_2254726, EPI_ISL_2255181	Regional Authority of Public Health Banská Bystrica	Veterinary institute in Zvolen Slovakia	Dirbáková Z., Maarová L., Mancoš M., Strhársky J., Sujová S., Mokryšová S., Tinák M., Mojžiš M.
EPI_ISL_2271902, EPI_ISL_2271903, EPI_ISL_2271904	Hospital General de México, Medicina Genómica	Hospital General de México, Medicina Genómica	Ana Laura Sánchez-Sandoval, María Eligia Juárez-Torres, Mariana Villalba-De la Peña, Siddhartha Ríos-Zaragoza, Guadalupe Mercedes Lucía Guerrero-Avendaño, Erika Anabel Alvarado-Silva, Eira Valeria Barrón-Palma
EPI_ISL_2284873	National Hospital	Institute of Tropical Disease, Universitas Airlangga	Jezzy R Dewantari, Rima R Prasetya, Krisnoadi Rahardjo, Aldise M Nastri, Azwan H Lubis, Gatot Soegiarto, Laksmi Wulandari, Resti Yudhawati, Yasuko Mori, Soetjipto, Kazufumi Shimizu, Maria I Lusida
EPI_ISL_2285148, EPI_ISL_2285149, EPI_ISL_2285152	Microbiology Department. Complexo Hospitalario Universitario de Vigo	Microbiology Department. Complexo Hospitalario Universitario de Vigo	Alfaya N, Alonso I, Alvarez M, Cabrera JJ, Carballo R, Cores O, Cortizo S, del-Campo V, Martinez L, Mediero G, Perez S, Potel C, Regueiro B, Rey S, Vasallo FJ
EPI_ISL_2304304, EPI_ISL_2307338, EPI_ISL_2307339, EPI_ISL_2307340, EPI_ISL_2307341	Genome Analysis Center, Yamanashi Central Hospital	Genome Analysis Center, Yamanashi Central Hospital	Yosuke Hirotsu
EPI_ISL_2350785	CHU Purpan - Laboratoire de Virologie - Institut Fédératif de Biologie	Ecole Veterinaire de Toulouse UMR1225 IHAP Interactions hôtes-agents pathogènes	Thomas Figueroa, Charlotte Foret-Lucas, Pierre Bessière, Romain Volmer, Mathilda Walch, Guillaume Croville, Jean-Luc Guérin, Jacques Izopet
EPI_ISL_2350793, EPI_ISL_2350794, EPI_ISL_2350795, EPI_ISL_2350796, EPI_ISL_2350797, EPI_ISL_2350798, EPI_ISL_2350799, EPI_ISL_2350800, EPI_ISL_2350801, EPI_ISL_2350803, EPI_ISL_2350804, EPI_ISL_2350805, EPI_ISL_2350806, EPI_ISL_2350807, EPI_ISL_2350808			
see above	Molecular Lab, Evercare hospital Dhaka	International Institute for Zoonosis Control, Hokkaido university	Junya Yamagishi, Mizanur Rahman
EPI_ISL_2364506, EPI_ISL_2364507, EPI_ISL_2364508	Genome Analysis Center, Yamanashi Central Hospital	Genome Analysis Center, Yamanashi Central Hospital	Yosuke Hirotsu
EPI_ISL_2382417, EPI_ISL_2382418, EPI_ISL_2382419, EPI_ISL_2382420, EPI_ISL_2398291, EPI_ISL_2398293, EPI_ISL_2398294	Centre for Human Virology and Genomics, Microbiology Department, Nigerian Institute of Medical Research	Central Research Laboratory, Nigerian Institute of Medical Research	Chika Kingsley Onwuamah, Ayorinde Babatunde James, Joseph Ojonugwa Shaibu, Rahaman A. Ahmed, Azuka Patrick Okwuraiwe, Sola Ajibaye, Sharon Abimbola, Olufemi Samuel Amoo, Muinah Adenike Fowora, Phasha-Muchemenye Mmatshepho, Joy Ayoola, Nyam Itse Yusuf, Grace Oni, Yusuf Jimoh, Josiah Ayoola Isong, Uyi Emokpae, Ngozi Mirabel Otuonye, Rosemary Ajuma Audu, Bamidele Iwalokun, Babatunde Lawal Salako
EPI_ISL_2433280, EPI_ISL_2433281, EPI_ISL_2433282, EPI_ISL_2433283, EPI_ISL_2433284	Fourth affiliated hospital, college of medicine, zhejiang university	Fourth affiliated hospital, college of medicine, zhejiang university	yingping wu,shufa zheng, tian liu, xueke liu, huina tang, yutong he, wei xu, lele li, wenxu yu, ke xing,xiaoping xia
EPI_ISL_2443718, EPI_ISL_2443725, EPI_ISL_2443752, EPI_ISL_2443763, EPI_ISL_2443769	Orange County Public Health Laboratory	University of California, Irvine	Amanda N. Pinski, Ilhem Messaoudi
EPI_ISL_2484141	Mater Misericordiae University Hospital Dublin	University College Dublin, CFS, School of Public Health	Maureen Lynch, Guerrino Macori, Séamus Fanning, Edel O' Regan, Eoin Hunt, Dermot O'Callaghan, Brian McCullagh, Cormac Jennings, Anne Fortune
EPI_ISL_2484209, EPI_ISL_2484210, EPI_ISL_2484211, EPI_ISL_2484212, EPI_ISL_2484213, EPI_ISL_2484214	University of Brescia - Dept. of Molecular and Translational Medicine	Istituto Zooprofilattico Sperimentale della Lombardia e dell'Emilia Romagna (IZSLER), Risk Analysis and Genomic Epidemiology Unit	Caruso Arnaldo, Fiorentini Simona, Caccuri Francesca, Messali Serena, Zani Alberto, Marina Morganti, Erika Scaltriti, Stefano Pongolini

EPI_ISL_2501081, EPI_ISL_2501084, EPI_ISL_2501087	Centre for Human Virology and Genomics, Microbiology Department, Nigerian Institute of Medical Research	Central Research Laboratory, Nigerian Institute of Medical Research	Chika Kingsley Onwuamah, Ayorinde Babatunde James, Joseph Ojonugwa Shaibu, Rahaman A. Ahmed, Azuka Patrick Okwuraiwe, Sola Ajibaye, Sharon Abimbola, Olufemi Samuel Amoo, Muinah Adenike Fowora, Phasha-Muchemenye Mmatshepho, Joy Ayoola, Nyam Itse Yusuf, Grace Oni, Yusuf Jimoh, Josiah Ayoola Isong, Uyi Emokpae, Ngozi Mirabel Otuonye, Rosemary Ajuma Audu, Bamidele Iwalokun, Babatunde Lawal Salako
EPI_ISL_2501394, EPI_ISL_2501395	Microbiology Department. Complexo Hospitalario Universitario de Vigo	Microbiology Department. Complexo Hospitalario Universitario de Vigo	Alfaya N, Alonso I, Alvarez M, Cabrera JJ, Carballo R, Cores O, Cortizo S, del-Campo V, Martinez L, Mediero G, Perez S, Potel C, Regueiro B, Rey S, Vasallo FJ
EPI_ISL_2502015, EPI_ISL_2502016, EPI_ISL_2502017, EPI_ISL_2502023, EPI_ISL_2502024	Centre for Human Virology and Genomics, Microbiology Department, Nigerian Institute of Medical Research	Central Research Laboratory, Nigerian Institute of Medical Research	Chika Kingsley Onwuamah, Ayorinde Babatunde James, Joseph Ojonugwa Shaibu, Rahaman A. Ahmed, Azuka Patrick Okwuraiwe, Sola Ajibaye, Sharon Abimbola, Olufemi Samuel Amoo, Muinah Adenike Fowora, Phasha-Muchemenye Mmatshepho, Joy Ayoola, Nyam Itse Yusuf, Grace Oni, Yusuf Jimoh, Josiah Ayoola Isong, Uyi Emokpae, Ngozi Mirabel Otuonye, Rosemary Ajuma Audu, Bamidele Iwalokun, Babatunde Lawal Salako
EPI_ISL_2502632, EPI_ISL_2502633, EPI_ISL_2502634, EPI_ISL_2502637, EPI_ISL_2502640, EPI_ISL_2502643, EPI_ISL_2502644, EPI_ISL_2502647, EPI_ISL_2502657, EPI_ISL_2502658, EPI_ISL_2502659, EPI_ISL_2502660, EPI_ISL_2502662, EPI_ISL_2502663, EPI_ISL_2502664, EPI_ISL_2502665, EPI_ISL_2502666			
see above	All India Institute of Medical Sciences Delhi Hospital	Virology Laboratory, AIIMS Delhi	Aashish Choudhary, Sumedha Bagga, Lata Rani, Dibyabhaba Pradhan, Pooja Pandey, Jyoti Jethani, Shivram Dhakad, Nazneen Arif, Deepankar Srigyan, Megha Brijwal, Lalit Dar, Manish Soneja, Rakesh Lodha, Puneet Kaur, Ritu Gupta, Subrata Sinha, Chitra Sarkar, Randeep Guleria
EPI_ISL_2565184	SK-Roy Romanow Provincial Laboratory	National Microbiology Laboratory (NML)	Anna Majer, Shari Tyson, Grace Seo, Philip Mabon, Elsie Grudeski, Rhiannon Huzarewich, Russell Mandes, Anneliese Landgraff, Jennifer Tanner, Natalie Knox, Morag Graham, Gary Van Domselaar, Ryan McDonald, Amanda Lang, Rachel DePaulo, Jessica Minion, Nathalie Bastien, Yan Li, Timothy Booth, Darian Hole, Madison Chapel, Kirsten Biggar, CanCOGeN's metadata curation team, Public Health Agency of Canada CanCOGeN team
EPI_ISL_2603247, EPI_ISL_2603249, EPI_ISL_2603250	Microbiology Department. Complexo Hospitalario Universitario de Vigo	Microbiology Department. Complexo Hospitalario Universitario de Vigo	Alfaya N, Alonso I, Alvarez M, Cabrera JJ, Carballo R, Cores O, Cortizo S, del-Campo V, Martinez L, Mediero G, Perez S, Potel C, Regueiro B, Rey S, Vasallo FJ
EPI_ISL_2614387	Adolfo Lutz Institute Sao Paulo Virology Center	Retrovirus Laboratory Adolfo Lutz Institute	Gabriela Bastos Cabral, Giselle I S Lopez-Lopes, Cintia Ahagon, Audrey Cilli, Paula Morena Guimaraes, Igor Mohamed Hussein, Luis Brigido
EPI_ISL_2663160	Ochsner Health	Bioinfoexperts, LLC	Judy Crabtree, Amy Feehan, Rebecca Rose, David J. Nolan, Tessa LaFleur, Julia-Garcia-Diaz, Lucio Miele, Susanna L. Lamers
EPI_ISL_2671835	Juntendo University Hospital	Juntendo University Hospital	Koji Tuchiya, Yoshie Hosaka
EPI_ISL_2690150	Ochsner Health	Bioinfoexperts, LLC	Judy Crabtree, Amy Feehan, Rebecca Rose, David J. Nolan, Tessa LaFleur, Julia-Garcia-Diaz, Lucio Miele, Susanna L. Lamers
EPI_ISL_2691710	Department of pathology, school of medicine, Imam Hospital complex, Tehran university of medical sciences, Tehran, Iran	Genetics Research Center, University of Social Welfare and Rehabilitation Sciences	Zohreh Fattahi, Marzieh Mohseni, Khadijeh Jalalvand, Azam Ghaziasadi, Seyedeh elham Mortazavi, Azar Hadadi, Alireza Abdollahi, Ali Jafarpour, Azam Ghaziasad, Seyedeh elham Mortazavi, Saber Soltani, Reza Najafipour, Kimia Kahrizi, Seyed Mohammad Jazayeri, Hossein Najmabadi.
EPI_ISL_2692885, EPI_ISL_2692888	Pars Hospital Laboratory, Rasht, Iran.	Genetics Research Center, University of Social Welfare and Rehabilitation Sciences	Zohreh Fattahi, Marzieh Mohseni, Khadijeh Jalalvand, Issa Jahanzad ,Kimia Kahrizi, Hossein Najmabadi.
EPI_ISL_2693672	Department of Virology, School of Medicine, Ahvaz Jundishapur University of Medical Sciences, Ahvaz, Iran	Genetics Research Center, University of Social Welfare and Rehabilitation Sciences	Zohreh Fattahi, Marzieh Mohseni, Khadijeh Jalalvand, Azam Ghaziasadi, Seyedeh elham Mortazavi, Ali Jafarpour, Azarakhsh Azaran, Farid Yousefi, Gholam Abbas Kaydani, Kimia Kahrizi, Seyed Mohammad Jazayeri, Hossein Najmabadi.
EPI_ISL_2693673	Department of Virology, School of Medicine, Ahvaz Jundishapur University of Medical Sciences, Ahvaz, Iran	Genetics Research Center, University of Social Welfare and Rehabilitation Sciences	Zohreh Fattahi, Marzieh Mohseni, Khadijeh Jalalvand, Azam Ghaziasadi, Seyedeh elham Mortazavi, Ali Jafarpour, Azarakhsh Azaran, Farid Yousefi, Gholam Abbas Kaydani, Kimia Kahrizi, Seyed Mohammad Jazayeri, Hossein Najmabadi.
EPI_ISL_2693674	Department of Virology, School of Medicine, Ahvaz Jundishapur University of Medical Sciences, Ahvaz, Iran	Genetics Research Center, University of Social Welfare and Rehabilitation Sciences	Zohreh Fattahi, Marzieh Mohseni, Khadijeh Jalalvand, Azam Ghaziasadi, Seyedeh elham Mortazavi, Ali Jafarpour, Azarakhsh Azaran, Farid Yousefi, Gholam Abbas Kaydani, Kimia Kahrizi, Seyed Mohammad Jazayeri, Hossein Najmabadi.
EPI_ISL_2693675	Department of Molecular Diagnostic, Emam Ali Educational and Therapeutic Center, Alborz University of Medical Sciences, Karaj, Iran	Genetics Research Center, University of Social Welfare and Rehabilitation Sciences	Zohreh Fattahi, Marzieh Mohseni, Khadijeh Jalalvand, Azam Ghaziasadi, Seyedeh elham Mortazavi, Ali Jafarpour, Zakiye Mokhames Mokhames, Alireza Soleimani, Kimia Kahrizi, Seyed Mohammad Jazayeri, Hossein Najmabadi.
EPI_ISL_2693676	Department of Molecular Diagnostic, Emam Ali Educational and Therapeutic Center, Alborz University of Medical Sciences, Karaj, Iran	Genetics Research Center, University of Social Welfare and Rehabilitation Sciences	Zohreh Fattahi, Marzieh Mohseni, Khadijeh Jalalvand, Azam Ghaziasadi, Seyedeh elham Mortazavi, Ali Jafarpour, Zakiye Mokhames, Alireza Soleimani, Kimia Kahrizi, Seyed Mohammad Jazayeri, Hossein Najmabadi.
EPI_ISL_2693905, EPI_ISL_2693906	Infectious Disease and Tropical Medicine Research Center, Resistant Tuberculosis Institute, Zahedan Universiry of Medical Sciences, Zahedan, Iran	Genetics Research Center, University of Social Welfare and Rehabilitation Sciences	Zohreh Fattahi, Marzieh Mohseni, Khadijeh Jalalvand, Azam Ghaziasadi, Seyedeh elham Mortazavi, Ali Jafarpour, Ebrahim Kord, Seyed Mohammad Hashemi-Shahri, Kimia Kahrizi, Seyed Mohammad Jazayeri, Hossein Najmabadi.
EPI_ISL_2693907	Infectious Diseases Research Center, Birjand University of Medical Sciences, Birjand, Iran	Genetics Research Center, University of Social Welfare and Rehabilitation Sciences	Zohreh Fattahi, Marzieh Mohseni, Khadijeh Jalalvand, Azam Ghaziasadi, Seyedeh elham Mortazavi, Ali Jafarpour, Shokouh Ghafari, Masood Ziaee, Kimia Kahrizi, Seyed Mohammad Jazayeri, Hossein Najmabadi.
EPI_ISL_2693908	Department of Infectious Disease, School of Medicine, Sina Hospital, Tehran University of Medical Sciences	Genetics Research Center, University of Social Welfare and Rehabilitation Sciences	Zohreh Fattahi, Marzieh Mohseni, Khadijeh Jalalvand, Jila Yavarian, Kimia Kahrizi, Talat Mokhtari-Azad, Hossein Najmabadi.
EPI_ISL_2693972, EPI_ISL_2694195, EPI_ISL_2694587	Virology Centre, School of public health, Tehran University of Medical Sciences, Tehran, Iran	Genetics Research Center, University of Social Welfare and Rehabilitation Sciences	Zohreh Fattahi, Marzieh Mohseni, Khadijeh Jalalvand, Jila Yavarian, Kimia Kahrizi, Talat Mokhtari-Azad, Hossein Najmabadi.
EPI_ISL_2710021	Ochsner Health	Bioinfoexperts, LLC	Judy Crabtree, Amy Feehan, Rebecca Rose, David J. Nolan, Tessa LaFleur, Julia-Garcia-Diaz, Lucio Miele, Susanna L. Lamers
EPI_ISL_2710810, EPI_ISL_2710812, EPI_ISL_2710814, EPI_ISL_2710815, EPI_ISL_2710817, EPI_ISL_2710818, EPI_ISL_2710820, EPI_ISL_2710821, EPI_ISL_2710823, EPI_ISL_2710825, EPI_ISL_2710827, EPI_ISL_2710831, EPI_ISL_2710837, EPI_ISL_2710838, EPI_ISL_2710872, EPI_ISL_2710873, EPI_ISL_2710874, EPI_ISL_2710875, EPI_ISL_2710876, EPI_ISL_2710877, EPI_ISL_2710878, EPI_ISL_2710879, EPI_ISL_2710880, EPI_ISL_2710881, EPI_ISL_2710882, EPI_ISL_2713077, EPI_ISL_2713078			
see above	Department of Health Technology and Informatics, The Hong Kong Polytechnic University	Department of Health Technology and Informatics, The Hong Kong Polytechnic University	Gilman Kit-Hang Siu, Lam-Kwong Lee, Kenneth Siu-Sing Leung, Jake Siu-Lun Leung, Wing-Hei Lo, Timothy Ting-Leung Ng, Chloe Toi-Mei Chan, Hoi-Ching Jim, Ivan Tak-Fai Wong, Kingsley King-Gee Tam, Hiu-Yin Lao, Denise Sze-Hang Wong, Alan Ka-Lun Wu, Miranda Chong-Yee Yau, Yvette Wai-Man Lai, Kitty Sau-Chun Fung, Sandy Ka-Yee Chau, Barry Kin-Chung Wong, Wing-Kin To, Kristine Luk, Alex Yat-Man Ho, Tak-Lun Que, Kam-Tong Yip, Wing Cheong Yam, David Ho-Keung Shum, Shea Ping Yip
EPI_ISL_2727891, EPI_ISL_2727899	All India Institute of Medical Sciences Delhi Hospital	Virology Laboratory, AIIMS Delhi	Aashish Choudhary, Sumedha Bagga, Lata Rani, Dibyabhaba Pradhan, Pooja Pandey, Jyoti Jethani, Shivram Dhakad, Nazneen Arif, Deepankar Srigyan, Megha Brijwal, Lalit Dar, Manish Soneja, Rakesh Lodha, Puneet Kaur, Ritu Gupta, Subrata Sinha, Chitra Sarkar, Randeep Guleria
EPI_ISL_2732373	Diagnostic Laboratory, Virology Erlangen	Armin Ensser	Alexandra Herrmann, Armin Ensser
EPI_ISL_2779638	State Key Laboratory of Emerging Infectious Diseases, The University of Hong Kong	The University of Hong Kong	Lifeng Li, Xianfan Wu, Yiu Man Cheung, Huachen Zhu, Yi Guan
EPI_ISL_2790002, EPI_ISL_2790003	All India Institute of Medical Sciences Delhi Hospital	Virology Laboratory, AIIMS Delhi	Aashish Choudhary, Sumedha Bagga, Lata Rani, Dibyabhaba Pradhan, Pooja Pandey, Jyoti Jethani, Shivram Dhakad, Nazneen Arif, Deepankar Srigyan, Megha Brijwal, Lalit Dar, Manish Soneja, Rakesh Lodha, Puneet Kaur, Ritu Gupta, Subrata Sinha, Chitra Sarkar, Randeep Guleria
EPI_ISL_2790800, EPI_ISL_2790801, EPI_ISL_2790802, EPI_ISL_2790803	Armies	National Reference Center for Viruses of Respiratory Infections, Institut Pasteur, Paris	Marion Barbet, Sylvie Behillil, Méline Bizard, Angela Brisebarre, Camille Capel, Vincent Enouf, Louise Lefrançois, Frédéric Lemoine, Christophe Malabat, Corinne Maufrais, Emmanuelle Permal, Etienne Simon-Lorière, Maud Vanpeene, Sylvie Van der Werf ,Christine Bigaillon
EPI_ISL_2790804, EPI_ISL_2790805, EPI_ISL_2790806, EPI_ISL_2790807, EPI_ISL_2790808	Hospital	National Reference Center for Viruses of Respiratory Infections, Institut Pasteur, Paris	Marion Barbet, Sylvie Behillil, Méline Bizard, Angela Brisebarre, Camille Capel, Vincent Enouf, Louise Lefrançois, Frédéric Lemoine, Christophe Malabat, Corinne Maufrais, Emmanuelle Permal, Etienne Simon-Lorière, Maud Vanpeene, Sylvie Van der Werf ,Christine Lambert
EPI_ISL_2790809	Hospital	National Reference Center for Viruses of Respiratory Infections, Institut Pasteur, Paris	Marion Barbet, Sylvie Behillil, Méline Bizard, Angela Brisebarre, Camille Capel, Vincent Enouf, Louise Lefrançois, Frédéric Lemoine, Christophe Malabat, Corinne Maufrais, Emmanuelle Permal, Etienne Simon-Lorière, Maud Vanpeene, Sylvie Van der Werf
EPI_ISL_2790810, EPI_ISL_2790811, EPI_ISL_2790812, EPI_ISL_2790813, EPI_ISL_2790814	Hospital	National Reference Center for Viruses of Respiratory Infections, Institut Pasteur, Paris	Marion Barbet, Sylvie Behillil, Méline Bizard, Angela Brisebarre, Camille Capel, Vincent Enouf, Louise Lefrançois, Frédéric Lemoine, Christophe Malabat, Corinne Maufrais, Emmanuelle Permal, Etienne Simon-Lorière, Maud Vanpeene, Sylvie Van der Werf ,Olivia Raulin
EPI_ISL_2790815, EPI_ISL_2790816	Hospital	National Reference Center for Viruses of Respiratory Infections, Institut Pasteur, Paris	Marion Barbet, Sylvie Behillil, Méline Bizard, Angela Brisebarre, Camille Capel, Vincent Enouf, Louise Lefrançois, Frédéric Lemoine, Christophe Malabat, Corinne Maufrais, Emmanuelle Permal, Etienne Simon-Lorière, Maud Vanpeene, Sylvie Van der Werf ,Christine Lambert

EPI_ISL_2790817, EPI_ISL_2790818	Hospital	National Reference Center for Viruses of Respiratory Infections, Institut Pasteur, Paris	Marion Barbet, Sylvie Behillil, Méline Bizard, Angela Brisebarre, Camille Capel, Vincent Enouf, Louise Lefrançois, Frédéric Lemoine, Christophe Malabat, Corinne Maufrais, Emmanuelle Permal, Etienne Simon-Lorière, Maud Vanpeene, Sylvie Van der Werf
EPI_ISL_2790819, EPI_ISL_2790820, EPI_ISL_2790821, EPI_ISL_2790822, EPI_ISL_2790823, EPI_ISL_2790824, EPI_ISL_2790825, EPI_ISL_2790826	Hospital	National Reference Center for Viruses of Respiratory Infections, Institut Pasteur, Paris	Marion Barbet, Sylvie Behillil, Méline Bizard, Angela Brisebarre, Camille Capel, Vincent Enouf, Louise Lefrançois, Frédéric Lemoine, Christophe Malabat, Corinne Maufrais, Emmanuelle Permal, Etienne Simon-Lorière, Maud Vanpeene, Sylvie Van der Werf ,Olivia Raulin
EPI_ISL_2790827, EPI_ISL_2790828, EPI_ISL_2790829, EPI_ISL_2790830, EPI_ISL_2790831, EPI_ISL_2790832, EPI_ISL_2790833	Hospital	National Reference Center for Viruses of Respiratory Infections, Institut Pasteur, Paris	Marion Barbet, Sylvie Behillil, Méline Bizard, Angela Brisebarre, Camille Capel, Vincent Enouf, Louise Lefrançois, Frédéric Lemoine, Christophe Malabat, Corinne Maufrais, Emmanuelle Permal, Etienne Simon-Lorière, Maud Vanpeene, Sylvie Van der Werf
EPI_ISL_2790834, EPI_ISL_2790835, EPI_ISL_2790836, EPI_ISL_2790837	Hospital	National Reference Center for Viruses of Respiratory Infections, Institut Pasteur, Paris	Marion Barbet, Sylvie Behillil, Méline Bizard, Angela Brisebarre, Camille Capel, Vincent Enouf, Louise Lefrançois, Frédéric Lemoine, Christophe Malabat, Corinne Maufrais, Emmanuelle Permal, Etienne Simon-Lorière, Maud Vanpeene, Sylvie Van der Werf ,Olivia Raulin
EPI_ISL_2790838, EPI_ISL_2790839, EPI_ISL_2790840, EPI_ISL_2790841, EPI_ISL_2790842, EPI_ISL_2790843	Hospital	National Reference Center for Viruses of Respiratory Infections, Institut Pasteur, Paris	Marion Barbet, Sylvie Behillil, Méline Bizard, Angela Brisebarre, Camille Capel, Vincent Enouf, Louise Lefrançois, Frédéric Lemoine, Christophe Malabat, Corinne Maufrais, Emmanuelle Permal, Etienne Simon-Lorière, Maud Vanpeene, Sylvie Van der Werf
EPI_ISL_2790844	Hospital	National Reference Center for Viruses of Respiratory Infections, Institut Pasteur, Paris	Marion Barbet, Sylvie Behillil, Méline Bizard, Angela Brisebarre, Camille Capel, Vincent Enouf, Louise Lefrançois, Frédéric Lemoine, Christophe Malabat, Corinne Maufrais, Emmanuelle Permal, Etienne Simon-Lorière, Maud Vanpeene, Sylvie Van der Werf ,LéA Pilorge
EPI_ISL_2790845, EPI_ISL_2790846, EPI_ISL_2790847	Hospital	National Reference Center for Viruses of Respiratory Infections, Institut Pasteur, Paris	Marion Barbet, Sylvie Behillil, Méline Bizard, Angela Brisebarre, Camille Capel, Vincent Enouf, Louise Lefrançois, Frédéric Lemoine, Christophe Malabat, Corinne Maufrais, Emmanuelle Permal, Etienne Simon-Lorière, Maud Vanpeene, Sylvie Van der Werf ,Patricia Barbut
EPI_ISL_2790848	Hospital	National Reference Center for Viruses of Respiratory Infections, Institut Pasteur, Paris	Marion Barbet, Sylvie Behillil, Méline Bizard, Angela Brisebarre, Camille Capel, Vincent Enouf, Louise Lefrançois, Frédéric Lemoine, Christophe Malabat, Corinne Maufrais, Emmanuelle Permal, Etienne Simon-Lorière, Maud Vanpeene, Sylvie Van der Werf ,Vincent Esteve
EPI_ISL_2790849	Hospital	National Reference Center for Viruses of Respiratory Infections, Institut Pasteur, Paris	Marion Barbet, Sylvie Behillil, Méline Bizard, Angela Brisebarre, Camille Capel, Vincent Enouf, Louise Lefrançois, Frédéric Lemoine, Christophe Malabat, Corinne Maufrais, Emmanuelle Permal, Etienne Simon-Lorière, Maud Vanpeene, Sylvie Van der Werf ,Patricia Barbut
EPI_ISL_2790850, EPI_ISL_2790851	Hospital	National Reference Center for Viruses of Respiratory Infections, Institut Pasteur, Paris	Marion Barbet, Sylvie Behillil, Méline Bizard, Angela Brisebarre, Camille Capel, Vincent Enouf, Louise Lefrançois, Frédéric Lemoine, Christophe Malabat, Corinne Maufrais, Emmanuelle Permal, Etienne Simon-Lorière, Maud Vanpeene, Sylvie Van der Werf ,Olivia Raulin
EPI_ISL_2790852, EPI_ISL_2790853, EPI_ISL_2790854, EPI_ISL_2790855, EPI_ISL_2790856	Hospital	National Reference Center for Viruses of Respiratory Infections, Institut Pasteur, Paris	Marion Barbet, Sylvie Behillil, Méline Bizard, Angela Brisebarre, Camille Capel, Vincent Enouf, Louise Lefrançois, Frédéric Lemoine, Christophe Malabat, Corinne Maufrais, Emmanuelle Permal, Etienne Simon-Lorière, Maud Vanpeene, Sylvie Van der Werf ,Patricia Barbut
EPI_ISL_2790857, EPI_ISL_2790858	EHPAD_IRA	National Reference Center for Viruses of Respiratory Infections, Institut Pasteur, Paris	Marion Barbet, Sylvie Behillil, Méline Bizard, Angela Brisebarre, Camille Capel, Vincent Enouf, Louise Lefrançois, Frédéric Lemoine, Christophe Malabat, Corinne Maufrais, Emmanuelle Permal, Etienne Simon-Lorière, Maud Vanpeene, Sylvie Van der Werf ,La Villa Papyri
EPI_ISL_2790859	EHPAD_IRA	National Reference Center for Viruses of Respiratory Infections, Institut Pasteur, Paris	Marion Barbet, Sylvie Behillil, Méline Bizard, Angela Brisebarre, Camille Capel, Vincent Enouf, Louise Lefrançois, Frédéric Lemoine, Christophe Malabat, Corinne Maufrais, Emmanuelle Permal, Etienne Simon-Lorière, Maud Vanpeene, Sylvie Van der Werf ,Plaisance
EPI_ISL_2790904	Hospital	National Reference Center for Viruses of Respiratory Infections, Institut Pasteur, Paris	Marion Barbet, Sylvie Behillil, Méline Bizard, Angela Brisebarre, Camille Capel, Vincent Enouf, Louise Lefrançois, Frédéric Lemoine, Christophe Malabat, Corinne Maufrais, Emmanuelle Permal, Etienne Simon-Lorière, Maud Vanpeene, Sylvie Van der Werf ,StéPhanie Marque Juillet
EPI_ISL_2790992	Hospital	National Reference Center for Viruses of Respiratory Infections, Institut Pasteur, Paris	Marion Barbet, Sylvie Behillil, Méline Bizard, Angela Brisebarre, Camille Capel, Vincent Enouf, Louise Lefrançois, Frédéric Lemoine, Christophe Malabat, Corinne Maufrais, Emmanuelle Permal, Etienne Simon-Lorière, Maud Vanpeene, Sylvie Van der Werf ,Olivia Raulin
EPI_ISL_2790993	Hospital	National Reference Center for Viruses of Respiratory Infections, Institut Pasteur, Paris	Marion Barbet, Sylvie Behillil, Méline Bizard, Angela Brisebarre, Camille Capel, Vincent Enouf, Louise Lefrançois, Frédéric Lemoine, Christophe Malabat, Corinne Maufrais, Emmanuelle Permal, Etienne Simon-Lorière, Maud Vanpeene, Sylvie Van der Werf ,Laurent Andreoletti
EPI_ISL_2790994	Hospital	National Reference Center for Viruses of Respiratory Infections, Institut Pasteur, Paris	Marion Barbet, Sylvie Behillil, Méline Bizard, Angela Brisebarre, Camille Capel, Vincent Enouf, Louise Lefrançois, Frédéric Lemoine, Christophe Malabat, Corinne Maufrais, Emmanuelle Permal, Etienne Simon-Lorière, Maud Vanpeene, Sylvie Van der Werf ,JéRome Guinard
EPI_ISL_2790995, EPI_ISL_2790996, EPI_ISL_2790997	EHPAD_IRA	National Reference Center for Viruses of Respiratory Infections, Institut Pasteur, Paris	Marion Barbet, Sylvie Behillil, Méline Bizard, Angela Brisebarre, Camille Capel, Vincent Enouf, Louise Lefrançois, Frédéric Lemoine, Christophe Malabat, Corinne Maufrais, Emmanuelle Permal, Etienne Simon-Lorière, Maud Vanpeene, Sylvie Van der Werf ,Vill'Alizé
EPI_ISL_2790998, EPI_ISL_2790999, EPI_ISL_2791000, EPI_ISL_2791001, EPI_ISL_2791002	Hospital	National Reference Center for Viruses of Respiratory Infections, Institut Pasteur, Paris	Marion Barbet, Sylvie Behillil, Méline Bizard, Angela Brisebarre, Camille Capel, Vincent Enouf, Louise Lefrançois, Frédéric Lemoine, Christophe Malabat, Corinne Maufrais, Emmanuelle Permal, Etienne Simon-Lorière, Maud Vanpeene, Sylvie Van der Werf ,Olivia Raulin
EPI_ISL_2791003	Hospital	National Reference Center for Viruses of Respiratory Infections, Institut Pasteur, Paris	Marion Barbet, Sylvie Behillil, Méline Bizard, Angela Brisebarre, Camille Capel, Vincent Enouf, Louise Lefrançois, Frédéric Lemoine, Christophe Malabat, Corinne Maufrais, Emmanuelle Permal, Etienne Simon-Lorière, Maud Vanpeene, Sylvie Van der Werf ,JéRome Guinard
EPI_ISL_2791004, EPI_ISL_2791005, EPI_ISL_2791006, EPI_ISL_2791007	Hospital	National Reference Center for Viruses of Respiratory Infections, Institut Pasteur, Paris	Marion Barbet, Sylvie Behillil, Méline Bizard, Angela Brisebarre, Camille Capel, Vincent Enouf, Louise Lefrançois, Frédéric Lemoine, Christophe Malabat, Corinne Maufrais, Emmanuelle Permal, Etienne Simon-Lorière, Maud Vanpeene, Sylvie Van der Werf ,Olivia Raulin
EPI_ISL_2791008, EPI_ISL_2791009	Hospital	National Reference Center for Viruses of Respiratory Infections, Institut Pasteur, Paris	Marion Barbet, Sylvie Behillil, Méline Bizard, Angela Brisebarre, Camille Capel, Vincent Enouf, Louise Lefrançois, Frédéric Lemoine, Christophe Malabat, Corinne Maufrais, Emmanuelle Permal, Etienne Simon-Lorière, Maud Vanpeene, Sylvie Van der Werf ,Alice Cadennes
EPI_ISL_2791010	Hospital	National Reference Center for Viruses of Respiratory Infections, Institut Pasteur, Paris	Marion Barbet, Sylvie Behillil, Méline Bizard, Angela Brisebarre, Camille Capel, Vincent Enouf, Louise Lefrançois, Frédéric Lemoine, Christophe Malabat, Corinne Maufrais, Emmanuelle Permal, Etienne Simon-Lorière, Maud Vanpeene, Sylvie Van der Werf ,Christine Lambert
EPI_ISL_2791011, EPI_ISL_2791012, EPI_ISL_2791013, EPI_ISL_2791014	Hospital	National Reference Center for Viruses of Respiratory Infections, Institut Pasteur, Paris	Marion Barbet, Sylvie Behillil, Méline Bizard, Angela Brisebarre, Camille Capel, Vincent Enouf, Louise Lefrançois, Frédéric Lemoine, Christophe Malabat, Corinne Maufrais, Emmanuelle Permal, Etienne Simon-Lorière, Maud Vanpeene, Sylvie Van der Werf ,Olivia Raulin
EPI_ISL_2791015	Hospital	National Reference Center for Viruses of Respiratory Infections, Institut Pasteur, Paris	Marion Barbet, Sylvie Behillil, Méline Bizard, Angela Brisebarre, Camille Capel, Vincent Enouf, Louise Lefrançois, Frédéric Lemoine, Christophe Malabat, Corinne Maufrais, Emmanuelle Permal, Etienne Simon-Lorière, Maud Vanpeene, Sylvie Van der Werf ,JéRome Guinard
EPI_ISL_2791016, EPI_ISL_2791017, EPI_ISL_2791018, EPI_ISL_2791019, EPI_ISL_2791020	Hospital	National Reference Center for Viruses of Respiratory Infections, Institut Pasteur, Paris	Marion Barbet, Sylvie Behillil, Méline Bizard, Angela Brisebarre, Camille Capel, Vincent Enouf, Louise Lefrançois, Frédéric Lemoine, Christophe Malabat, Corinne Maufrais, Emmanuelle Permal, Etienne Simon-Lorière, Maud Vanpeene, Sylvie Van der Werf ,Olivia Raulin
EPI_ISL_2791021, EPI_ISL_2791022, EPI_ISL_2791023, EPI_ISL_2791024, EPI_ISL_2791025, EPI_ISL_2791026, EPI_ISL_2791027, EPI_ISL_2791028, EPI_ISL_2791029, EPI_ISL_2791030, EPI_ISL_2791031, EPI_ISL_2791032, EPI_ISL_2791033, EPI_ISL_2791034, EPI_ISL_2791035			
see above	Armies	National Reference Center for Viruses of Respiratory Infections, Institut Pasteur, Paris	Marion Barbet, Sylvie Behillil, Méline Bizard, Angela Brisebarre, Camille Capel, Vincent Enouf, Louise Lefrançois, Frédéric Lemoine, Christophe Malabat, Corinne Maufrais, Emmanuelle Permal, Etienne Simon-Lorière, Maud Vanpeene, Sylvie Van der Werf ,Christine Bigaillon
EPI_ISL_2811805	Jining Center for Disease Control and Prevention	Jining Center for Disease Control and Prevention	Jiao Bo-yan
EPI_ISL_2835273, EPI_ISL_2835274, EPI_ISL_2835275, EPI_ISL_2835276, EPI_ISL_2835277, EPI_ISL_2835278, EPI_ISL_2835279, EPI_ISL_2835280, EPI_ISL_2835281, EPI_ISL_2835282, EPI_ISL_2835283, EPI_ISL_2835284, EPI_ISL_2835285, EPI_ISL_2835286, EPI_ISL_2835287, EPI_ISL_2835288, EPI_ISL_2835289, EPI_ISL_2835290, EPI_ISL_2835291, EPI_ISL_2835292, EPI_ISL_2835293, EPI_ISL_2835294, EPI_ISL_2835295, EPI_ISL_2835296, EPI_ISL_2835297, EPI_ISL_2835298, EPI_ISL_2835299, EPI_ISL_2835300, EPI_ISL_2835301, EPI_ISL_2835302, EPI_ISL_2835303, EPI_ISL_2835304, EPI_ISL_2835305, EPI_ISL_2835306, EPI_ISL_2835307, EPI_ISL_2835308, EPI_ISL_2835309, EPI_ISL_2835310, EPI_ISL_2835311, EPI_ISL_2835312, EPI_ISL_2835313, EPI_ISL_2835314, EPI_ISL_2835315, EPI_ISL_2835316, EPI_ISL_2835317, EPI_ISL_2835318, EPI_ISL_2835319, EPI_ISL_2835320, EPI_ISL_2835321, EPI_ISL_2835322, EPI_ISL_2835323, EPI_ISL_2835324, EPI_ISL_2835325, EPI_ISL_2835326, EPI_ISL_2835327, EPI_ISL_2835328, EPI_ISL_2835329, EPI_ISL_2835330, EPI_ISL_2835331, EPI_ISL_2835332, EPI_ISL_2835333, EPI_ISL_2835334, EPI_ISL_2835335, EPI_ISL_2835336, EPI_ISL_2835337, EPI_ISL_2835338, EPI_ISL_2835339, EPI_ISL_2835340, EPI_ISL_2835341, EPI_ISL_2835342, EPI_ISL_2835343, EPI_ISL_2835344, EPI_ISL_2835345, EPI_ISL_2835346, EPI_ISL_2835347, EPI_ISL_2835348, EPI_ISL_2835349, EPI_ISL_2835351, EPI_ISL_2835352, EPI_ISL_2835353, EPI_ISL_2835354, EPI_ISL_2835355, EPI_ISL_2835356, EPI_ISL_2835357, EPI_ISL_2835358, EPI_ISL_2835359, EPI_ISL_2835360, EPI_ISL_2835361, EPI_ISL_2835362, EPI_ISL_2835363, EPI_ISL_2835365, EPI_ISL_2835366, EPI_ISL_2835371, EPI_ISL_2835372, EPI_ISL_2835373, EPI_ISL_2835375, EPI_ISL_2835376, EPI_ISL_2835377, EPI_ISL_2835378, EPI_ISL_2835379, EPI_ISL_2835406, EPI_ISL_2835418, EPI_ISL_2835419			
see above	Instituto de Diagnostico y Referencia Epidemiologicos (InDRE)	Andersen lab at Scripps Research	SEARCH Alliance with Gisela Barrera Badillo, Jose Ernesto Ramirez Gonzalez, Maria Natividad Cruz Ortiz, Nervain Benjamin Contreras Gonzalez, Tatiana Ernestina Nunez Garcia, Dayanira Sarith Arellano Suarez, Irma Lopez Martinez, Rita Flores Leon, Beatriz Olivares Flores

EPI_ISL_2835566	Sharp HealthCare Laboratory	Andersen lab at Scripps Research	SEARCH Alliance San Diego with Aaron Harding, Jacquelyn Berumen, Cathy Woerle, Liam McGinnis, Art Mendoza, Omid Bakhtar
EPI_ISL_2877292, EPI_ISL_2877293	Hospital	National Reference Center for Viruses of Respiratory Infections, Institut Pasteur, Paris	Marion Barbet, Sylvie Behillil, Méline Bizard, Angela Brisebarre, Camille Capel, Vincent Enouf, Louise Lefrançois, Frédéric Lemoine, Christophe Malabat, Corinne Maufrais, Etienne Simon-Lorière, Maud Vanpeene, Sylvie Van der Werf
EPI_ISL_2877294, EPI_ISL_2877295, EPI_ISL_2877296, EPI_ISL_2877297	Hospital	National Reference Center for Viruses of Respiratory Infections, Institut Pasteur, Paris	Marion Barbet, Sylvie Behillil, Méline Bizard, Angela Brisebarre, Camille Capel, Vincent Enouf, Louise Lefrançois, Frédéric Lemoine, Christophe Malabat, Corinne Maufrais, Etienne Simon-Lorière, Maud Vanpeene, Sylvie Van der Werf ,Olivia Raulin
EPI_ISL_2877298	Hospital	National Reference Center for Viruses of Respiratory Infections, Institut Pasteur, Paris	Marion Barbet, Sylvie Behillil, Méline Bizard, Angela Brisebarre, Camille Capel, Vincent Enouf, Louise Lefrançois, Frédéric Lemoine, Christophe Malabat, Corinne Maufrais, Etienne Simon-Lorière, Maud Vanpeene, Sylvie Van der Werf
EPI_ISL_2877299, EPI_ISL_2877300	EHPAD_IRA	National Reference Center for Viruses of Respiratory Infections, Institut Pasteur, Paris	Marion Barbet, Sylvie Behillil, Méline Bizard, Angela Brisebarre, Camille Capel, Vincent Enouf, Louise Lefrançois, Frédéric Lemoine, Christophe Malabat, Corinne Maufrais, Etienne Simon-Lorière, Maud Vanpeene, Sylvie Van der Werf
EPI_ISL_2877301, EPI_ISL_2877302, EPI_ISL_2877303	Hospital	National Reference Center for Viruses of Respiratory Infections, Institut Pasteur, Paris	Marion Barbet, Sylvie Behillil, Méline Bizard, Angela Brisebarre, Camille Capel, Vincent Enouf, Louise Lefrançois, Frédéric Lemoine, Christophe Malabat, Corinne Maufrais, Etienne Simon-Lorière, Maud Vanpeene, Sylvie Van der Werf
EPI_ISL_2877304, EPI_ISL_2877305, EPI_ISL_2877306, EPI_ISL_2877307, EPI_ISL_2877308, EPI_ISL_2877309, EPI_ISL_2877310, EPI_ISL_2877311, EPI_ISL_2877312	EHPAD_IRA	National Reference Center for Viruses of Respiratory Infections, Institut Pasteur, Paris	Marion Barbet, Sylvie Behillil, Méline Bizard, Angela Brisebarre, Camille Capel, Vincent Enouf, Louise Lefrançois, Frédéric Lemoine, Christophe Malabat, Corinne Maufrais, Etienne Simon-Lorière, Maud Vanpeene, Sylvie Van der Werf
EPI_ISL_2877313, EPI_ISL_2877314, EPI_ISL_2877315, EPI_ISL_2877316	Hospital	National Reference Center for Viruses of Respiratory Infections, Institut Pasteur, Paris	Marion Barbet, Sylvie Behillil, Méline Bizard, Angela Brisebarre, Camille Capel, Vincent Enouf, Louise Lefrançois, Frédéric Lemoine, Christophe Malabat, Corinne Maufrais, Etienne Simon-Lorière, Maud Vanpeene, Sylvie Van der Werf ,Pascale Martres
EPI_ISL_2877317, EPI_ISL_2877318, EPI_ISL_2877319	Hospital	National Reference Center for Viruses of Respiratory Infections, Institut Pasteur, Paris	Marion Barbet, Sylvie Behillil, Méline Bizard, Angela Brisebarre, Camille Capel, Vincent Enouf, Louise Lefrançois, Frédéric Lemoine, Christophe Malabat, Corinne Maufrais, Etienne Simon-Lorière, Maud Vanpeene, Sylvie Van der Werf ,Olivia Raulin
EPI_ISL_2877320, EPI_ISL_2877321, EPI_ISL_2877322, EPI_ISL_2877323, EPI_ISL_2877324, EPI_ISL_2877325, EPI_ISL_2877326, EPI_ISL_2877327, EPI_ISL_2877328, EPI_ISL_2877329, EPI_ISL_2877330, EPI_ISL_2877331			
see above	Hospital	National Reference Center for Viruses of Respiratory Infections, Institut Pasteur, Paris	Marion Barbet, Sylvie Behillil, Méline Bizard, Angela Brisebarre, Camille Capel, Vincent Enouf, Louise Lefrançois, Frédéric Lemoine, Christophe Malabat, Corinne Maufrais, Etienne Simon-Lorière, Maud Vanpeene, Sylvie Van der Werf
EPI_ISL_2877332	Hospital	National Reference Center for Viruses of Respiratory Infections, Institut Pasteur, Paris	Marion Barbet, Sylvie Behillil, Méline Bizard, Angela Brisebarre, Camille Capel, Vincent Enouf, Louise Lefrançois, Frédéric Lemoine, Christophe Malabat, Corinne Maufrais, Etienne Simon-Lorière, Maud Vanpeene, Sylvie Van der Werf ,S Kouhil
EPI_ISL_2877333, EPI_ISL_2877334, EPI_ISL_2877335, EPI_ISL_2877336, EPI_ISL_2877337, EPI_ISL_2877338, EPI_ISL_2877339, EPI_ISL_2877340	Hospital	National Reference Center for Viruses of Respiratory Infections, Institut Pasteur, Paris	Marion Barbet, Sylvie Behillil, Méline Bizard, Angela Brisebarre, Camille Capel, Vincent Enouf, Louise Lefrançois, Frédéric Lemoine, Christophe Malabat, Corinne Maufrais, Etienne Simon-Lorière, Maud Vanpeene, Sylvie Van der Werf ,Olivia Raulin
EPI_ISL_2877341, EPI_ISL_2877342, EPI_ISL_2877343, EPI_ISL_2877344, EPI_ISL_2877345, EPI_ISL_2877346, EPI_ISL_2877347	Hospital	National Reference Center for Viruses of Respiratory Infections, Institut Pasteur, Paris	Marion Barbet, Sylvie Behillil, Méline Bizard, Angela Brisebarre, Camille Capel, Vincent Enouf, Louise Lefrançois, Frédéric Lemoine, Christophe Malabat, Corinne Maufrais, Etienne Simon-Lorière, Maud Vanpeene, Sylvie Van der Werf
EPI_ISL_2877348, EPI_ISL_2877349, EPI_ISL_2877350, EPI_ISL_2877351, EPI_ISL_2877353	EHPAD_IRA	National Reference Center for Viruses of Respiratory Infections, Institut Pasteur, Paris	Marion Barbet, Sylvie Behillil, Méline Bizard, Angela Brisebarre, Camille Capel, Vincent Enouf, Louise Lefrançois, Frédéric Lemoine, Christophe Malabat, Corinne Maufrais, Etienne Simon-Lorière, Maud Vanpeene, Sylvie Van der Werf
EPI_ISL_2877355, EPI_ISL_2877357, EPI_ISL_2877359, EPI_ISL_2877361, EPI_ISL_2877363, EPI_ISL_2877364, EPI_ISL_2877366, EPI_ISL_2877368	Hospital	National Reference Center for Viruses of Respiratory Infections, Institut Pasteur, Paris	Marion Barbet, Sylvie Behillil, Méline Bizard, Angela Brisebarre, Camille Capel, Vincent Enouf, Louise Lefrançois, Frédéric Lemoine, Christophe Malabat, Corinne Maufrais, Etienne Simon-Lorière, Maud Vanpeene, Sylvie Van der Werf
EPI_ISL_2877370	EHPAD_IRA	National Reference Center for Viruses of Respiratory Infections, Institut Pasteur, Paris	Marion Barbet, Sylvie Behillil, Méline Bizard, Angela Brisebarre, Camille Capel, Vincent Enouf, Louise Lefrançois, Frédéric Lemoine, Christophe Malabat, Corinne Maufrais, Etienne Simon-Lorière, Maud Vanpeene, Sylvie Van der Werf
EPI_ISL_2877371, EPI_ISL_2877373, EPI_ISL_2877375, EPI_ISL_2877377, EPI_ISL_2877379, EPI_ISL_2877381, EPI_ISL_2877383	Hospital	National Reference Center for Viruses of Respiratory Infections, Institut Pasteur, Paris	Marion Barbet, Sylvie Behillil, Méline Bizard, Angela Brisebarre, Camille Capel, Vincent Enouf, Louise Lefrançois, Frédéric Lemoine, Christophe Malabat, Corinne Maufrais, Etienne Simon-Lorière, Maud Vanpeene, Sylvie Van der Werf ,Patricia Barbut
EPI_ISL_2877384, EPI_ISL_2877386, EPI_ISL_2877388	Hospital	National Reference Center for Viruses of Respiratory Infections, Institut Pasteur, Paris	Marion Barbet, Sylvie Behillil, Méline Bizard, Angela Brisebarre, Camille Capel, Vincent Enouf, Louise Lefrançois, Frédéric Lemoine, Christophe Malabat, Corinne Maufrais, Etienne Simon-Lorière, Maud Vanpeene, Sylvie Van der Werf ,Vincent Esteve
EPI_ISL_2877390, EPI_ISL_2877392, EPI_ISL_2877394, EPI_ISL_2877395, EPI_ISL_2877397, EPI_ISL_2877399, EPI_ISL_2877401, EPI_ISL_2877403, EPI_ISL_2877405, EPI_ISL_2877407, EPI_ISL_2877408, EPI_ISL_2877410, EPI_ISL_2877412, EPI_ISL_2877414, EPI_ISL_2877416, EPI_ISL_2877418, EPI_ISL_2877420, EPI_ISL_2877422			
see above	Hospital	National Reference Center for Viruses of Respiratory Infections, Institut Pasteur, Paris	Marion Barbet, Sylvie Behillil, Méline Bizard, Angela Brisebarre, Camille Capel, Vincent Enouf, Louise Lefrançois, Frédéric Lemoine, Christophe Malabat, Corinne Maufrais, Etienne Simon-Lorière, Maud Vanpeene, Sylvie Van der Werf ,Olivia Raulin
EPI_ISL_2877423, EPI_ISL_2877425, EPI_ISL_2877427, EPI_ISL_2877429	Hospital	National Reference Center for Viruses of Respiratory Infections, Institut Pasteur, Paris	Marion Barbet, Sylvie Behillil, Méline Bizard, Angela Brisebarre, Camille Capel, Vincent Enouf, Louise Lefrançois, Frédéric Lemoine, Christophe Malabat, Corinne Maufrais, Etienne Simon-Lorière, Maud Vanpeene, Sylvie Van der Werf ,Patricia Barbut
EPI_ISL_2877431, EPI_ISL_2877433, EPI_ISL_2877435, EPI_ISL_2877437, EPI_ISL_2877438	EHPAD_IRA	National Reference Center for Viruses of Respiratory Infections, Institut Pasteur, Paris	Marion Barbet, Sylvie Behillil, Méline Bizard, Angela Brisebarre, Camille Capel, Vincent Enouf, Louise Lefrançois, Frédéric Lemoine, Christophe Malabat, Corinne Maufrais, Etienne Simon-Lorière, Maud Vanpeene, Sylvie Van der Werf
EPI_ISL_2877440	Hospital	National Reference Center for Viruses of Respiratory Infections, Institut Pasteur, Paris	Marion Barbet, Sylvie Behillil, Méline Bizard, Angela Brisebarre, Camille Capel, Vincent Enouf, Louise Lefrançois, Frédéric Lemoine, Christophe Malabat, Corinne Maufrais, Etienne Simon-Lorière, Maud Vanpeene, Sylvie Van der Werf ,Patricia Barbut
EPI_ISL_2877442	Hospital	National Reference Center for Viruses of Respiratory Infections, Institut Pasteur, Paris	Marion Barbet, Sylvie Behillil, Méline Bizard, Angela Brisebarre, Camille Capel, Vincent Enouf, Louise Lefrançois, Frédéric Lemoine, Christophe Malabat, Corinne Maufrais, Etienne Simon-Lorière, Maud Vanpeene, Sylvie Van der Werf ,Olivia Raulin
EPI_ISL_2877444	Hospital	National Reference Center for Viruses of Respiratory Infections, Institut Pasteur, Paris	Marion Barbet, Sylvie Behillil, Méline Bizard, Angela Brisebarre, Camille Capel, Vincent Enouf, Louise Lefrançois, Frédéric Lemoine, Christophe Malabat, Corinne Maufrais, Etienne Simon-Lorière, Maud Vanpeene, Sylvie Van der Werf ,Vincent Esteve
EPI_ISL_2877446	Hospital	National Reference Center for Viruses of Respiratory Infections, Institut Pasteur, Paris	Marion Barbet, Sylvie Behillil, Méline Bizard, Angela Brisebarre, Camille Capel, Vincent Enouf, Louise Lefrançois, Frédéric Lemoine, Christophe Malabat, Corinne Maufrais, Etienne Simon-Lorière, Maud Vanpeene, Sylvie Van der Werf ,Olivia Raulin
EPI_ISL_2877448, EPI_ISL_2877450, EPI_ISL_2877451, EPI_ISL_2877452, EPI_ISL_2877453	Hospital	National Reference Center for Viruses of Respiratory Infections, Institut Pasteur, Paris	Marion Barbet, Sylvie Behillil, Méline Bizard, Angela Brisebarre, Camille Capel, Vincent Enouf, Louise Lefrançois, Frédéric Lemoine, Christophe Malabat, Corinne Maufrais, Etienne Simon-Lorière, Maud Vanpeene, Sylvie Van der Werf ,Patricia Barbut
EPI_ISL_2877454, EPI_ISL_2877455, EPI_ISL_2877456, EPI_ISL_2877457, EPI_ISL_2877458	Hospital	National Reference Center for Viruses of Respiratory Infections, Institut Pasteur, Paris	Marion Barbet, Sylvie Behillil, Méline Bizard, Angela Brisebarre, Camille Capel, Vincent Enouf, Louise Lefrançois, Frédéric Lemoine, Christophe Malabat, Corinne Maufrais, Etienne Simon-Lorière, Maud Vanpeene, Sylvie Van der Werf ,Olivia Raulin
EPI_ISL_2877459	EHPAD_IRA	National Reference Center for Viruses of Respiratory Infections, Institut Pasteur, Paris	Marion Barbet, Sylvie Behillil, Méline Bizard, Angela Brisebarre, Camille Capel, Vincent Enouf, Louise Lefrançois, Frédéric Lemoine, Christophe Malabat, Corinne Maufrais, Etienne Simon-Lorière, Maud Vanpeene, Sylvie Van der Werf

EPI_ISL_2877460	Hospital	National Reference Center for Viruses of Respiratory Infections, Institut Pasteur, Paris	Marion Barbet, Sylvie Behillil, Méline Bizard, Angela Brisebarre, Camille Capel, Vincent Enouf, Louise Lefrançois, Frédéric Lemoine, Christophe Malabat, Corinne Maufrais, Etienne Simon-Lorière, Maud Vanpeene, Sylvie Van der Werf ,S Kouhil
EPI_ISL_2877461	Hospital	National Reference Center for Viruses of Respiratory Infections, Institut Pasteur, Paris	Marion Barbet, Sylvie Behillil, Méline Bizard, Angela Brisebarre, Camille Capel, Vincent Enouf, Louise Lefrançois, Frédéric Lemoine, Christophe Malabat, Corinne Maufrais, Etienne Simon-Lorière, Maud Vanpeene, Sylvie Van der Werf ,Patricia Barbut
EPI_ISL_2877462, EPI_ISL_2877463, EPI_ISL_2877464	Hospital	National Reference Center for Viruses of Respiratory Infections, Institut Pasteur, Paris	Marion Barbet, Sylvie Behillil, Méline Bizard, Angela Brisebarre, Camille Capel, Vincent Enouf, Louise Lefrançois, Frédéric Lemoine, Christophe Malabat, Corinne Maufrais, Etienne Simon-Lorière, Maud Vanpeene, Sylvie Van der Werf ,Olivia Raulin
EPI_ISL_2877465, EPI_ISL_2877466	EHPAD_IRA	National Reference Center for Viruses of Respiratory Infections, Institut Pasteur, Paris	Marion Barbet, Sylvie Behillil, Méline Bizard, Angela Brisebarre, Camille Capel, Vincent Enouf, Louise Lefrançois, Frédéric Lemoine, Christophe Malabat, Corinne Maufrais, Etienne Simon-Lorière, Maud Vanpeene, Sylvie Van der Werf
EPI_ISL_2877467	Sentinelles Province	National Reference Center for Viruses of Respiratory Infections, Institut Pasteur, Paris	Marion Barbet, Sylvie Behillil, Méline Bizard, Angela Brisebarre, Camille Capel, Vincent Enouf, Louise Lefrançois, Frédéric Lemoine, Christophe Malabat, Corinne Maufrais, Etienne Simon-Lorière, Maud Vanpeene, Sylvie Van der Werf ,LaurèNe Millet-Malingrey
EPI_ISL_2877468	Sentinelles Province	National Reference Center for Viruses of Respiratory Infections, Institut Pasteur, Paris	Marion Barbet, Sylvie Behillil, Méline Bizard, Angela Brisebarre, Camille Capel, Vincent Enouf, Louise Lefrançois, Frédéric Lemoine, Christophe Malabat, Corinne Maufrais, Etienne Simon-Lorière, Maud Vanpeene, Sylvie Van der Werf ,Isabelle Husson
EPI_ISL_2877469, EPI_ISL_2877470, EPI_ISL_2877471	Hospital	National Reference Center for Viruses of Respiratory Infections, Institut Pasteur, Paris	Marion Barbet, Sylvie Behillil, Méline Bizard, Angela Brisebarre, Camille Capel, Vincent Enouf, Louise Lefrançois, Frédéric Lemoine, Christophe Malabat, Corinne Maufrais, Etienne Simon-Lorière, Maud Vanpeene, Sylvie Van der Werf ,Patricia Barbut
EPI_ISL_2877472	Hospital	National Reference Center for Viruses of Respiratory Infections, Institut Pasteur, Paris	Marion Barbet, Sylvie Behillil, Méline Bizard, Angela Brisebarre, Camille Capel, Vincent Enouf, Louise Lefrançois, Frédéric Lemoine, Christophe Malabat, Corinne Maufrais, Etienne Simon-Lorière, Maud Vanpeene, Sylvie Van der Werf ,Vincent Esteve
EPI_ISL_2877473, EPI_ISL_2877474, EPI_ISL_2877475, EPI_ISL_2877476, EPI_ISL_2877477, EPI_ISL_2877478, EPI_ISL_2877479, EPI_ISL_2877480, EPI_ISL_2877481, EPI_ISL_2877482, EPI_ISL_2877483, EPI_ISL_2877484, EPI_ISL_2877485, EPI_ISL_2877486			
see above	Hospital	National Reference Center for Viruses of Respiratory Infections, Institut Pasteur, Paris	Marion Barbet, Sylvie Behillil, Méline Bizard, Angela Brisebarre, Camille Capel, Vincent Enouf, Louise Lefrançois, Frédéric Lemoine, Christophe Malabat, Corinne Maufrais, Etienne Simon-Lorière, Maud Vanpeene, Sylvie Van der Werf ,Olivia Raulin
EPI_ISL_2877487	Sentinelles Idf	National Reference Center for Viruses of Respiratory Infections, Institut Pasteur, Paris	Marion Barbet, Sylvie Behillil, Méline Bizard, Angela Brisebarre, Camille Capel, Vincent Enouf, Louise Lefrançois, Frédéric Lemoine, Christophe Malabat, Corinne Maufrais, Etienne Simon-Lorière, Maud Vanpeene, Sylvie Van der Werf ,Christine Aubry
EPI_ISL_2877488, EPI_ISL_2877489, EPI_ISL_2877490, EPI_ISL_2877491, EPI_ISL_2877492, EPI_ISL_2877493, EPI_ISL_2877494, EPI_ISL_2877495, EPI_ISL_2877496, EPI_ISL_2877497	Hospital	National Reference Center for Viruses of Respiratory Infections, Institut Pasteur, Paris	Marion Barbet, Sylvie Behillil, Méline Bizard, Angela Brisebarre, Camille Capel, Vincent Enouf, Louise Lefrançois, Frédéric Lemoine, Christophe Malabat, Corinne Maufrais, Etienne Simon-Lorière, Maud Vanpeene, Sylvie Van der Werf ,Ilka Engelmann
EPI_ISL_402124	Wuhan Jinyintan Hospital	Wuhan Institute of Virology, Chinese Academy of Sciences	Peng Zhou, Xing-Lou Yang, Ding-Yu Zhang, Lei Zhang, Yan Zhu, Hao-Rui Si, Zhengli Shi
EPI_ISL_402126	Dept. of Virology III, National Institute of Infectious Diseases	Dept. of Virology III, National Institute of Infectious Diseases	Naganori Nao, Kazuya Shirato, Shutoku Matsuyama, Makoto Takeda
EPI_ISL_402127, EPI_ISL_402128, EPI_ISL_402129, EPI_ISL_402130	Wuhan Jinyintan Hospital	Wuhan Institute of Virology, Chinese Academy of Sciences	Peng Zhou, Xing-Lou Yang, Ding-Yu Zhang, Lei Zhang, Yan Zhu, Hao-Rui Si, Zhengli Shi
EPI_ISL_402132	Wuhan Jinyintan Hospital	Hubei Provincial Center for Disease Control and Prevention	Bin Fang, Xiang Li, Xiao Yu, Linlin Liu, Bo Yang, Faxian Zhan, Guojun Ye, Xixiang Huo, Junqiang Xu, Bo Yu, Kun Cai, Jing Li, Yongzhong Jiang.
EPI_ISL_403962, EPI_ISL_403963	Bamrasnaradura Hospital	1. Department of Medical Sciences, Ministry of Public Health, Thailand 2. Thai Red Cross Emerging Infectious Diseases - Health Science Centre 3. Department of Disease Control, Ministry of Public Health, Thailand	Pilailuk,Okada; Siripaporn,Phuygun; Thanutsapa,Thanadachakul; Supaporn,Wacharapluesadee; Sittiporn,Parnmen; Warawan,Wongboot; Sunthareeya,Waicharoen; Rome,Buathong; Malinee,Chittaganpitch; Nanthawan,Mekha
EPI_ISL_404227	Zhejiang Provincial Center for Disease Control and Prevention	Department of Microbiology, Zhejiang Provincial Center for Disease Control and Prevention	Yin Chen, Yanjun Zhang, Haiyan Mao, Junhang Pan, Xiuyu Lou, Yiyu Lu, Juying Yan, Hanping Zhu, Jian Gao, Yan Feng, Yi Sun, Hao Yan, Zhen Li, Yisheng Sun, Liming Gong, Qiong Ge, Wen Shi, Xinying Wang, Wenwu Yao, Zhangnv Yang, Fang Xu, Chen Chen, Enfu Chen, Zhen Wang, Zhiping Chen, Jianmin Jiang, Chonggao Hu
EPI_ISL_404228	Zhejiang Provincial Center for Disease Control and Prevention	Department of Microbiology, Zhejiang Provincial Center for Disease Control and Prevention	Yanjun Zhang, Yin Chen, Haiyan Mao, Junhang Pan, Xiuyu Lou, Yiyu Lu, Juying Yan, Hanping Zhu, Jian Gao, Yan Feng, Yi Sun, Hao Yan, Zhen Li, Yisheng Sun, Liming Gong, Qiong Ge, Wen Shi, Xinying Wang, Wenwu Yao, Zhangnv Yang, Fang Xu, Chen Chen, Enfu Chen, Zhen Wang, Zhiping Chen, Jianmin Jiang, Chonggao Hu
EPI_ISL_406592	Shenzhen Third People's Hospital	Shenzhen Key Laboratory of Pathogen and Immunity, National Clinical Research Center for Infectious Disease,Shenzhen Third People's Hospital	Yang Yang, Chenguang Shen, Li Xing, Zhixiang Xu, Haixia Zheng, Yingxia Liu
EPI_ISL_406593, EPI_ISL_406594, EPI_ISL_406595	Shenzhen Key Laboratory of Pathogen and Immunity, National Clinical Research Center for Infectious Disease, Shenzhen Third People's Hospital	Shenzhen Key Laboratory of Pathogen and Immunity, National Clinical Research Center for Infectious Disease, Shenzhen Third People's Hospital	Yang Yang, Chenguang Shen, Li Xing, Zhixiang Xu, Haixia Zheng, Yingxia Liu
EPI_ISL_406862	Charité Universitätsmedizin Berlin, Institute of Virology; Institut für Mikrobiologie der Bundeswehr, Munich	Charité Universitätsmedizin Berlin, Institute of Virology	Victor M Corman, Julia Schneider, Talitha Veith, Barbara Mühlemann, Markus Antwerpen, Christian Drosten, Roman Wölfel
EPI_ISL_406970	Hangzhou Center for Disease and Control Microbiology Lab	Hangzhou Center for Disease and Control Microbiology Lab	Yu Hua, Wang Haoqiu, Li Jun, Yu Xinfeng
EPI_ISL_407079	Lapland Central Hospital	Department of Virology, University of Helsinki and Helsinki University Hospital, Helsinki, Finland	Teemu Smura, Suvi Kuivanen, Hannimari Kallio-Kokko, Olli Vapalahti
EPI_ISL_407313	Hangzhou Center for Disease Control and Prevention	Hangzhou Center for Disease Control and Prevention	Jun Li, Haoqiu Wang, Hua Yu, Lingfeng Mao, Xinfen Yu, Zhou Sun, Qingxin Kong, Xin Qian, Shuchang Chen, Xuchu Wang
EPI_ISL_407893	Centre for Infectious Diseases and Microbiology Laboratory Services	NSW Health Pathology - Institute of Clinical Pathology and Medical Research; Westmead Hospital; University of Sydney	Eden J-S, Carter I, Rahman H, Holmes EC, Rockett R, O'Sullivan MV, Sintchenko V, Chen SC, Maddocks S, Kok J and Dwyer DE for the 2019-nCoV Study Group
EPI_ISL_407976	KU Leuven, Clinical and Epidemiological Virology	KU Leuven, Clinical and Epidemiological Virology	Bert Vanmechelen, Elke Wollants, Annabel Rector, Els Keyaerts, Lies Laenen, Marc Van Ranst, and Piet Maes
EPI_ISL_408068	Virology Laboratory National Institute for Infectious Diseases 'Lazzaro Spallanzani' IRCCS	Virology Laboratory National Institute for Infectious Diseases 'Lazzaro Spallanzani' IRCCS	Capobianchi,M.R., Carletti,F., Lalle,E., Bordi,L., Marsella,P.,Colavita,F., Matusali,G., Nicastri,E., Ippolito,G. and Castilletti,C.
EPI_ISL_408430	Department of Infectious and Tropical Diseases, Bichat Claude Bernard Hospital, Paris	National Reference Center for Viruses of Respiratory Infections, Institut Pasteur, Paris	Mélanie Albert, Marion Barbet, Sylvie Behillil, Méline Bizard, Angela Brisebarre, Flora Donati, Vincent Enouf, Maud Vanpeene, Sylvie van der Werf, Yazdan Yazdanpanah, Xavier Lescure
EPI_ISL_408431	Sorbonne Université, Inserm et Assistance Publique-Hôpitaux de Paris (Pitié Salpétrière)	National Reference Center for Viruses of Respiratory Infections, Institut Pasteur, Paris	Mélanie Albert, Marion Barbet, Sylvie Behillil, Méline Bizard, Angela Brisebarre, Flora Donati, Vincent Enouf, Maud Vanpeene, Sylvie van der Werf, Sonia Burrel, Anne-Geneviève Marcelin, Vincent Calvez, David Boutolleau, Elise Klément, Valérie Pourcher, Eric Caumes.
EPI_ISL_408480	National Institute for Viral Disease Control and Prevention, China CDC	National Institute for Viral Disease Control & Prevention, CCDC	Wenjie TanXiaoqing FuXiang ZhaoWenling Wang Peihua NiuRoujian Lu,Yanhong SunBaoying HuangLi ZhaoFei YeWenbo XuGeorge F. GaoGuizhen Wu
EPI_ISL_408481	National Institute for Viral Disease Control and Prevention, China CDC	National Institute for Viral Disease Control & Prevention, CCDC	Wenjie Tan, Hengqin Wang, Xiang Zhao, Wenling Wang, Peihua Niu, Roujian Lu, Sheng Ye, Baoying Huang, Li Zhao, Fei Ye, Wenbo Xu, George F. Gao, Guizhen Wu
EPI_ISL_408482	National Institute for Viral Disease Control and Prevention, China CDC	National Institute for Viral Disease Control & Prevention, CCDC	Wenjie Tan, Zhaoguo Wang, Xiang Zhao, Wenling Wang, Peihua Niu, Roujian Lu, Ti Liu, Baoying Huang, Li Zhao, Fei Ye, Wenbo Xu, George F. Gao, Guizhen Wu
EPI_ISL_408484	National Institute for Viral Disease Control and Prevention, China CDC	National Institute for Viral Disease Control & Prevention, CCDC	Wenjie Tan, Jianan Xu, Wenling Wang, Peihua Niu, Roujian Lu, Huiping Yang, Xiang Zhao, Baoying Huang, Li Zhao, Fei Ye, Wenbo Xu, George F. Gao, Guizhen Wu

EPI_ISL_408485	National Institute for Viral Disease Control and Prevention, China CDC	National Institute for Viral Disease Control & Prevention, CCDC	Wenjie Tan,Quanyi Wang,Wenling Wang, Peihua Niu,Roujian Lu,Yang Pan,Xiang Zhao,Baoying Huang,Li Zhao,Fei Ye,Wenbo Xu,George F. Gao,Guizhen Wu
EPI_ISL_408486	National Institute for Viral Disease Control and Prevention, China CDC	National Institute for Viral Disease Control & Prevention, CCDC	Wenjie Tan, Yong Shi, Wenling Wang, Peihua Niu, Roujian Lu, Jianxiong Li, Xiang Zhao, Baoying Huang, Li Zhao, Fei Ye, Wenbo Xu, George F. Gao, Guizhen Wu
EPI_ISL_408488	National Institute for Viral Disease Control and Prevention, China CDC	National Institute for Viral Disease Control & Prevention, CCDC	Wenjie Tan, Shenjiao Wang, Wenling Wang, Peihua Niu, Roujian Lu, Kangchen Zhao, Xiang Zhao, Baoying Huang, Li Zhao, Fei Ye, Wenbo Xu, George F. Gao, Guizhen Wu
EPI_ISL_408489	Department of Laboratory Medicine, National Taiwan University Hospital	Microbial Genomics Core Lab, National Taiwan University Centers of Genomic and Precision Medicine	Shiou-Hwei Yeh, You-Yu Lin, Ya-Yun Lai, Chiao-Ling Li, Shan-Chwen Chang, Pei-Jer Chen, Sui-Yuan Chang
EPI_ISL_408666, EPI_ISL_408669	Dept. of Virology III, National Institute of Infectious Diseases	Pathogen Genomics Center, National Institute of Infectious Diseases	Tsuyoshi Sekizuka, Shutoku Matsuyama, Naganori Nao, Kazuya Shirato, Makoto Takeda, Makoto Kuroda
EPI_ISL_408977	Serology, Virology and OTDS Laboratories (SAViD), NSW Health Pathology Randwick	NSW Health Pathology - Institute of Clinical Pathology and Medical Research; Centre for Infectious Diseases and Microbiology Laboratory Services; Westmead Hospital; University of Sydney	Eden J-S, Carter I, Rahman H, Rawlinson W, Holmes EC, Rockett R, O'Sullivan MV, Sintchenko V, Chen SC, Maddocks S, Kok J and Dwyer DE for the 2019-nCoV Study Group*
EPI_ISL_410218	Department of Laboratory Medicine, National Taiwan University Hospital	Microbial Genomics Core Lab, National Taiwan University Centers of Genomic and Precision Medicine	Shiou-Hwei Yeh, You-Yu Lin, Ya-Yun Lai, Chiao-Ling Li, Shan-Chwen Chang, Pei-Jer Chen, Sui-Yuan Chang
EPI_ISL_410301	National Influenza Centre, National Public Health Laboratory, Kathmandu, Nepal	The University of Hong Kong	Ranjit Sah , Runa Jha, Daniel Chu, Haogao Gu, Malik Peiris, Anup Bastola, Alfonso J. Rodriguez-Morales, Bibek Kumar Lal, Basu Dev Pandey, Leo Poon
EPI_ISL_410531, EPI_ISL_410532	Dept. of Pathology, National Institute of Infectious Diseases	Pathogen Genomics Center, National Institute of Infectious Diseases	Tsuyoshi Sekizuka, Harutaka Katano, Shutoku Matsuyama, Naganori Nao, Kazuya Shirato, Motoi Suzuki, Hideki Hasegawa, Takaji Wakita, Makoto Takeda, Tadaki Suzuki, Makoto Kuroda
EPI_ISL_410545	INMI Lazzaro Spallanzani IRCCS	Laboratory of Virology, INMI Lazzaro Spallanzani IRCCS	Maria R. Capobianchi, Cesare E. M. Gruber, Martina Rueca, Barbara Bartolini, Francesco Messina, Emanuela Giombini, Francesca Colavita, Concetta Castilletti, Eleonora Lalle, Fabrizio Carletti, Emanuele Nicastri, Giuseppe Ippolito.
EPI_ISL_410546	INMI Lazzaro Spallanzani IRCCS	Laboratory of Virology, INMI Lazzaro Spallanzani IRCCS	Maria R. Capobianchi, Cesare E. M. Gruber, Martina Rueca, Fabrizio Carletti, Barbara Bartolini, Francesco Messina, Emanuela Giombini, Francesca Colavita, Concetta Castilletti, Eleonora Lalle, Emanuele Nicastri, Giuseppe Ippolito.
EPI_ISL_410984	Department of Infectious and Tropical Diseases, Bichat Claude Bernard Hospital, Paris	National Reference Center for Viruses of Respiratory Infections, Institut Pasteur, Paris	Mélanie Albert, Marion Barbet, Sylvie Behillil, Méline Bizard, Angela Brisebarre, Flora Donati, Vincent Enouf, Maud Vanpeene, Sylvie van der Werf, Yazdan Yazdanpanah, Xavier Lescure
EPI_ISL_411218	Department of Infectious and Tropical Diseases, Bichat Claude Bernard Hospital, Paris	Laboratoire Virpath, CIRI U111, UCBL1, INSERM, CNRS, ENS Lyon	Olivier Terrier, Aurélien Traversier, Julien Fouret, Yazdan Yazdanpanah, Xavier Lescure, Catherine Legras-Lachuer, Alexandre Gaymard, Bruno Lina, Manuel Rosa-Calatrava
EPI_ISL_411219, EPI_ISL_411220	Department of Infectious and Tropical Diseases, Bichat Claude Bernard Hospital, Paris	Laboratoire Virpath, CIRI U111, UCBL1, INSERM, CNRS, ENS Lyon	Olivier Terrier, Aurélien Traversier, Julien Fouret, Yazdan Yazdanpanah, Xavier Lescure, Alexandre Gaymard, Bruno Lina, Manuel Rosa-Calatrava
EPI_ISL_411902	Virology Unit, Institut Pasteur du Cambodge.	Virology Unit, Institut Pasteur du Cambodge (Sequencing done by: Jessica E Manning/Jennifer A Bohl at Malaria and Vector Research Research Laboratory, National Institute of Allergy and Infectious Diseases and Vida Ahyong from Chan-Zuckerberg Biohub)	Erik A Karlsson, Jennifer A Bohl, Vida Ahyong, Veasna Duong, Philippe Dussart, Jessica E Manning.
EPI_ISL_412386	Beijing Ditan Hospital, Capital Medical University	National Institute for Communicable Disease Control and Prevention, Chinese Center for Disease Control and Prevention	Xinmin Xu, Xin Lu, Pan Xiang, Haijian Zhou, Biao Kan, Yajie Wang, Jingyuan Liu, Yanwen Xiong, Huizhu Wang, Ruihong Li, Fangfang Jin, Jie Gong, Xiaoping Chen, Lili Gao, Haofeng Xiong, Lin Pu, Chuansheng Li, Ming Zhang, Jianbo Tan, Yao Sun, Yufeng Liu, Hebing Guo, Jingjing Hao
EPI_ISL_412459	Jingzhou Center for Disease Control and Prevention	Hubei Provincial Center for Disease Control and Prevention	Bin Fang, Xiang Li, Xiao Yu, Linlin Liu, Bo Yang, Faxian Zhan, Guojun Ye, Xixiang Huo, Junqiang Xu, Bo Yu, Kun Cai, Jing Li, Maoyi Chen,Jie Hu, Chunlin Mao, Yongzhong Jiang.
EPI_ISL_412964	Hospital Israelita Albert Einstein	Instituto Adolfo Lutz Interdisciplinary Procedures Center Strategic Laboratory	Jaqueline Goes de Jesus, Claudio Tavares Sacchi, Daniela Bernardes Borges da Silva, Ingra Morales Claro, Flávia Cristina da Silva Sales, Claudia Regina Gonçalves, Joshua Quick, Maria do Carmo, Sampaio Tavares Timenetsky, Nicholas James Loman, Andrew Rambaut, Ester Cerdeira Sabino, Nuno Rodrigues Faria
EPI_ISL_412970	Washington State Department of Health	Seattle Flu Study	Helen Chu, Michael Boeckh, Janet Englund, Michael Famulare, Barry Lutz, Deborah Nickerson, Mark Rieder, Lea Starita, Matthew Thompson, Jay Shendure, and Trevor Bedford
EPI_ISL_412972	Instituto Nacional de Enfermedades Respiratorias	Instituto de Diagnostico y Referencia Epidemiologicos (INDRE)	Ramirez-Gonzalez Ernesto, Garces-Ayala Fabiola, Araiza-Rodriguez Adnan, Mendieta-Condado Edgar, Rodriguez-Maldonado Abril, Wong-Arambula Claudia, Vazquez-Perez Joel, Martinez Arturo, Boukadida Celia, Munoz-Medina Esteban, Sanchez Alejandro, Isa Pavel, Taboada Blanca, Lopez Susana, Arias Carlos, Barrera-Badillo Gisela, Hernandez-Rivas Lucia, Lopez-Martinez Irma
EPI_ISL_412975	Centre for Infectious Diseases and Microbiology Laboratory Services	NSW Health Pathology - Institute of Clinical Pathology and Medical Research; Westmead Hospital; University of Sydney	Eden J-S, Carter I, Rahman H, Holmes EC, Rockett R, O'Sullivan MV, Sintchenko V, Chen SC, Maddocks S, Kok J and Dwyer DE for the 2019-nCoV Study Group
EPI_ISL_412978	The Central Hospital Of Wuhan	Hubei Provincial Center for Disease Control and Prevention	Bin Fang, Xiang Li, Xiao Yu, Linlin Liu, Bo Yang, Faxian Zhan, Guojun Ye, Xixiang Huo, Junqiang Xu, Bo Yu, Kun Cai, Jing Li, Yongzhong Jiang.
EPI_ISL_412979, EPI_ISL_412980	Union Hospital of Tongji Medical College, Huazhong University of Science and Technology	Hubei Provincial Center for Disease Control and Prevention	Bin Fang, Xiang Li, Xiao Yu, Linlin Liu, Bo Yang, Faxian Zhan, Guojun Ye, Xixiang Huo, Junqiang Xu, Bo Yu, Kun Cai, Jing Li, Yongzhong Jiang.
EPI_ISL_412981	CR&WISCO GENERAL HOSPITAL	Hubei Provincial Center for Disease Control and Prevention	Bin Fang, Xiang Li, Xiao Yu, Linlin Liu, Bo Yang, Faxian Zhan, Guojun Ye, Xixiang Huo, Junqiang Xu, Bo Yu, Kun Cai, Jing Li, Yongzhong Jiang.
EPI_ISL_412982	Wuhan Lung Hospital	Hubei Provincial Center for Disease Control and Prevention	Bin Fang, Xiang Li, Xiao Yu, Linlin Liu, Bo Yang, Faxian Zhan, Guojun Ye, Xixiang Huo, Junqiang Xu, Bo Yu, Kun Cai, Jing Li, Yongzhong Jiang.
EPI_ISL_412983	Tianmen Center for Disease Control and Prevention	Hubei Provincial Center for Disease Control and Prevention	Bin Fang, Xiang Li, Xiao Yu, Linlin Liu, Bo Yang, Faxian Zhan, Guojun Ye, Xixiang Huo, Junqiang Xu, Bo Yu, Kun Cai, Jing Li, YiFa Zhu, Yangyang Tao,Xierong Li,Yongzhong Jiang.
EPI_ISL_413016	Hospital Israelita Albert Einstein	Instituto Adolfo Lutz, Interdisciplinary Procedures Center, Strategic Laboratory	Jaqueline Goes de Jesus, Claudio Tavares Sacchi, Fabiana Cristina Pereira dos Santos, Ingra Morales Claro, Flávia Cristina da Silva Sales, Claudia Regina Gonçalves, Joshua Quick, Maria do Carmo Sampaio Tavares Timenetsky, Nicholas James Loman, Andrew Rambaut, Ester Cerdeira Sabino, Nuno Rodrigues Faria
EPI_ISL_413213, EPI_ISL_413214	Centre for Infectious Diseases and Microbiology Laboratory Services	NSW Health Pathology - Institute of Clinical Pathology and Medical Research; Westmead Hospital; University of Sydney	Eden J-S, Carter I, Rahman H, Holmes EC, Rockett R, O'Sullivan MV, Sintchenko V, Chen SC, Maddocks S, Kok J and Dwyer DE for the 2019-nCoV Study Group*
EPI_ISL_413219	National Institute of Health Research and Development	National Institute of Health Research and Development	Setiawaty,V; Subangkit; Pawestri,HA; Puspa,KD; Ikawati,HD; Nugraha,AA; Hariastuti,NI; Ramadhany,R; Susilarini,NK; Pratiwi,E; Agustiningsih; Kurniawati,J; Siswanto
EPI_ISL_413459	Department of Pathology, Toshima Hospital	Pathogen Genomics Center, National Institute of Infectious Diseases	Tsuyoshi Sekizuka, Kentaro Itokawa, Takuya Adachi, Masahiro Sano, Jun Yamazaki, Ippei Miyamoto, Haruka Nishioka, Ja-Mun Chong, Noriko Nakajima, Yuko Sato, Minoru Tobiume, Harutaka Katano, Tadaki Suzuki, Makoto Kuroda
EPI_ISL_413490	Auckland Hospital	Institute of Environmental Science and Research (ESR)	Matt Storey, Xiaoyun Ren, Gary McAuliffe, Sally Roberts, Matthew Blakiston, Erasmus Smit, Lauren Jelly, Joep de Ligt
EPI_ISL_413522	Indian Council of Medical Research - National Institute of Virology	National Influenza Center, Indian Council of Medical Research - National Institute of Virology	Potdar V, Yadav PD, Choudhary ML, Shete-Aich A

EPI_ISL_413523	Indian Council of Medical Research-National Institute of Virology	National Influenza Center, Indian Council of Medical Research-National Institute of Virology	Potdar V, Yadav PD, Choudhary ML, Shete-Aich A
EPI_ISL_413553	Iran National Influenza Center	Iran National Influenza Center	Jila Yavarian, Nazanin Zahra Shafiei Jandaghi, Kaveh Sadeghi, Fatemeh Ajaminejad, Nastaran Ghavvami and Talat Mokhtari Azad
EPI_ISL_413554	Iran National Influenza Center	Iran National Influenza Center	Jila Yavarian, Nazanin Zahra Shafiei Jandaghi, Kaveh Sadeghi, Nastaran Ghavvami, Fatemeh Ajami Nejad, Fatemeh Saadatmand and Talat Mokhtari Azad
EPI_ISL_413594	Centre for Infectious Diseases and Microbiology Laboratory Services	NSW Health Pathology - Institute of Clinical Pathology and Medical Research; Westmead Hospital; University of Sydney	Rockett R, Eden J-S, Lam C, Gray K, Timms, V, Gall, M, Alicia, A, Carter I, Rahman H, Holmes EC, , O'Sullivan MV, Sintchenko V, Chen SC, Maddocks S, Kok J and Dwyer DE for the 2019-nCoV Study Group*
EPI_ISL_413595	Centre for Infectious Diseases and Microbiology Laboratory Services	NSW Health Pathology - Institute of Clinical Pathology and Medical Research; Westmead Hospital; University of Sydney	Rockett R, Eden J-S, Lam C, Gray K, Timms, V, Gall, M, Carter I, Rahman H, Holmes EC, O'Sullivan MV, Sintchenko V, Chen SC, Maddocks S, Kok J and Dwyer DE for the 2019-nCoV Study Group*
EPI_ISL_413596	Centre for Infectious Diseases and Microbiology - Public Health	NSW Health Pathology - Institute of Clinical Pathology and Medical Research; Westmead Hospital; University of Sydney	Rockett R, Eden J-S, Lam C, Gray K, Timms, V, Gall, M, Carter I, Rahman H, Holmes EC, O'Sullivan MV, Sintchenko V, Chen SC, Maddocks S, Kok J and Dwyer DE for the 2019-nCoV Study Group*
EPI_ISL_413597	Centre for Infectious Diseases and Microbiology- Public Health	NSW Health Pathology - Institute of Clinical Pathology and Medical Research; Westmead Hospital; University of Sydney	Lam C, Eden J-S, Rockett R, Gray K, Timms, V, Gall, M, Carter I, Rahman H, Holmes EC, O'Sullivan MV, Sintchenko V, Chen SC, Maddocks S, Kok J and Dwyer DE for the 2019-nCoV Study Group*
EPI_ISL_413598	Centre for Infectious Diseases and Microbiology - Public Health	NSW Health Pathology - Institute of Clinical Pathology and Medical Research; Westmead Hospital; University of Sydney	Gray K, Eden J-S, Lam C, Rockett R, Timms, V, Gall, M, Carter I, Rahman H, Holmes EC, O'Sullivan MV, Sintchenko V, Chen SC, Maddocks S, Kok J and Dwyer DE for the 2019-nCoV Study Group*
EPI_ISL_413599	Centre for Infectious Diseases and Microbiology - Public Health	NSW Health Pathology - Institute of Clinical Pathology and Medical Research; Westmead Hospital; University of Sydney	Timms, V, Eden J-S, Lam C, Gray K, Rockett R, Gall, M, Carter I, Rahman H, Holmes EC, O'Sullivan MV, Sintchenko V, Chen SC, Maddocks S, Kok J and Dwyer DE for the 2019-nCoV Study Group*
EPI_ISL_413600	Centre for Infectious Diseases and Microbiology - Public Health	NSW Health Pathology - Institute of Clinical Pathology and Medical Research; Westmead Hospital; University of Sydney	Gall, M, Eden J-S, Lam C, Gray K, Timms, V, Rockett R, Carter I, Rahman H, Holmes EC, O'Sullivan MV, Sintchenko V, Chen SC, Maddocks S, Kok J and Dwyer DE for the 2019-nCoV Study Group*
EPI_ISL_413647	Centro Hospital do Porto, E.P.E. - H. Geral de Santo Antonio	Instituto Nacional de Saude (INSA)	Raquel Guiomar, Inês Costa, Pedro Pechirra, Joana Mendonça, Luís Vieira, Helena Ramos, Joana Isidro, Vítor Borges, João Paulo Gomes
EPI_ISL_413648	Centro Hospitalar e Universitário de Sao Joao, Porto	Instituto Nacional de Saude (INSA)	Raquel Guiomar, Inês Costa, Pedro Pechirra, Joana Mendonça, Luís Vieira, João Tiago Guimarães, Joana Isidro, Vítor Borges, João Paulo Gomes
EPI_ISL_413904	Iran National Influenza Center	Iran National Influenza Center	Nazanin Zahra Shafiei Jandaghi,Jila Yavarian,Fatemeh AjamiNejad, Nastaran Ghavvami,Kaveh Sadeghi and Talat Mokhtari Azad
EPI_ISL_414014	Hospital Israelita Albert Einstein	Instituto Adolfo Lutz, Interdiciplinary Procedures Center, Strategic Laboratory	Claudio Tavares Sacchi, Claudia Regina Gonçalves, Katia Correia dos Santos, Carlos Henrique Camargo, Maria do Carmo Sampaito Tavares Timenetsky, Terezinha Maria de Paiva, Ester Cerdeira Sabino
EPI_ISL_414015	Hospital São Joaquim Beneficencia Portuguesa	Instituto Adolfo Lutz, Interdiciplinary Procedures Center, Strategic Laboratory	Claudio Tavares Sacchi, Claudia Regina Gonçalves, SimoneGuadagnucci Morillo, Carlos Henrique Camargo, Maria do Carmo Sampaito Tavares Timenetsky, Fabiana Cristina Pereira dos Santos Terezinha Maria de Paiva, Ester Cerdeira Sabino
EPI_ISL_414016	Hospital São Joaquim Beneficencia Portuguesa	Instituto Adolfo Lutz, Interdiciplinary Procedures Center, Strategic Laboratory	Claudio Tavares Sacchi, Claudia Regina Gonçalves, Audrey Cilli, Carlos Henrique Camargo, Maria do Carmo Sampaito Tavares Timenetsky, Daniela Bernardes Borges da Silva, Terezinha Maria de Paiva, Ester Cerdeira Sabino
EPI_ISL_414017	Hospital São Joaquim Beneficencia Portuguesa	Instituto Adolfo Lutz, Interdiciplinary Procedures Center, Strategic Laboratory	Claudio Tavares Sacchi, Claudia Regina Gonçalves, Fabiana Cristina Pereira dos Santos, Carlos Henrique Camargo, Maria do Carmo Sampaito Tavares Timenetsky, Daniela Bernardes Borges da Silva, Terezinha Maria de Paiva, Ester Cerdeira Sabino
EPI_ISL_414372	Iran National Influenza Center	Iran National Influenza Center	Jila Yavarian, Nazanin Zahra Shafiei Jandaghi, Kaveh Sadeghi, Fatemeh Ajaminejad, Fatemeh Saadatmand and Talat Mokhtari Azad
EPI_ISL_414373	Iran National Influenza Center	Iran National Influenza Center	Kaveh Sadeghi, Jila Yavarian, Nazanin Zahra Shafiei Jandaghi, Ahmad Nejati, Najmeh Parhizghari, Soad Ghabeshi and Talat Mokhtari Azad
EPI_ISL_414375, EPI_ISL_414376, EPI_ISL_414377	National Institute of Health Research and Development	National Institute of Health Research and Development	Setiawaty,V; Subangkit; Puspa,KD; Ikawati,HD; Nugraha, AA; Susilarini, NK; Agustiningsih; Ramadhany,R; Pratiwi,E; Hariastuti,NI; Kurniawati,J; Pawestri,HA; Siswanto
EPI_ISL_414477	The National Institute of Public Health Center for Epidemiology and Microbiology	State Veterinary Institute Prague	Alexander Nagy, Oldrich Bartos, Helena Jirincova, Klara Labska, Ludmila Novakova, Olga Storkanova, Dusan Trnka, Jaromira Vecerova
EPI_ISL_414495	Servicio Microbiología. Hospital Clínico Universitario. Valencia.	Sequencing and Bioinformatics Service. Molecular Epidemiology Laboratory. FISABIO-Public Health	David Navarro, Maria Alma Bracho, Giuseppe D'Auria, Griselda De Marco, Neris Garcia-Gonzalez, Fernando Gonzalez-Candelas
EPI_ISL_414496	Servicio Microbiologia. Hospital Clinico Universitario. Valencia.	Sequencing and Bioinformatics Service. Molecular Epidemiology Laboratory. FISABIO-Public Health	David Navarro, María Alma Bracho, Giuseppe D'Auria, Griselda De Marco, Neris Garcia-Gonzalez, Fernando Gonzalez-Candelas
EPI_ISL_414512	Dipartimento di Medicina e Chirurgia, University of Insubria and Ospedale di Circolo e Fondazione Macchi	Dipartimento di Medicina e Chirurgia, University of Insubria and Ospedale di Circolo e Fondazione Macchi	Genoni,A.P., Baj,A. and Rossi,A.
EPI_ISL_414520, EPI_ISL_414521	Bundeswehr Institute of Microbiology	Bundeswehr Institute of Microbiology	Mathias C Walter, Markus H Antwerpen and Roman Wölfel
EPI_ISL_414568	Pasteur Institute of Iran	Pasteur Institute of Iran	Arash Arashkia, Kayhan Azadmanesh, Mohammad Hassan Pouriayevali, Tahmineh Jalali, Zahra Ahmadi, Mohammad Sadegh Shams Nosrati, Ali Maleki, Zabihollah Shoja, Sanam Azad-Mazjiri, Neda Amin, Mehdi Rohani, Saber Esmaeili, Ahmad Ghasemi, Amir Hesam Nemati, Ahmad Mahmoudi, Zahra Fereydouni, Mahsa Tavakolirad, Tahereh Mohammadi, Sahar Khakifirouz, Mehdi Fazlalipour, Maryam Rostamtabar, Arezoo Parikhani, Hesam Karimi, Kazem Baesi, Seyed Dawood Mousavi Nasab, Mahmood Barati, Mohammad Reza Asadi Karam, Mehri Habibi, Fatemeh Fotouhi-Chahooki, Neda Afzali, Ali Torabi, Azita Eshratkhah mohammadnejad, Seyedeh Sahar Bathaeian, Mina Bahri, Mohamad Mahdi Mortazavipour, Seyedeh Atefe Hosseini, Farideh Niknam, Parastoo Yekta Sanati, Hadiseh Shokouhi, Azam Amirian, Afsaneh zokaei, Hajarossadat Ghaderi, Mahboobeh Rafigh, Elmira Vadaye kheiri, Akram Agharezaei, Akram Abouie Mehrizi, seyedeh Zahra Moravej, Mostafa Salehi-Vaziri
EPI_ISL_414577	Hospital de Talca, Chile	Instituto de Salud Publica de Chile	Andrés E. Castillo, Bárbara Parra, Paz Tapia, Alejandra Acevedo, Jaime Lagos, Winston Andrade, Loredana Arata, Gabriel Leal, Gisselle Barra, Carolina Tambley, Javier Tognarelli, Patricia Bustos, Soledad Ulloa, Rodrigo Fasce, Jorge Fernández.
EPI_ISL_414598	Servicio Microbiologia, Hospital Clinico Universitario, Valencia	Sequencing and Bioinformatics Service and Molecular Epidemiology Research Group. FISABIO-Public Health.	David Navarro, Maria Alma Bracho, Giuseppe D'Auria, Griselda De Marco, Neris Garcia-Gonzalez, Fernando Gonzalez-Candelas
EPI_ISL_414623	Laboratoire de Virologie Institut de Virologie - INSERM U 1109 Hôpitaux Universitaires de Strasbourg	National Reference Center for Viruses of Respiratory Infections, Institut Pasteur, Paris	Mélnie Albert, Marion Barbet, Sylvie Behillil, Méline Bizard, Angela Brisebarre, Flora Donati Vincent Enouf, Maud Vanpeene, Sylvie van der Werf, Samira Fafi-Kremer
EPI_ISL_414624	Centre Hositalier Universitaire de Rouen Laboratoire de Virologie	National Reference Center for Viruses of Respiratory Infections, Institut Pasteur, Paris	Mélnie Albert, Marion Barbet, Sylvie Behillil, Méline Bizard, Angela Brisebarre, Flora Donati Vincent Enouf, Maud Vanpeene, Sylvie van der Werf, Jean-Christophe Plantier
EPI_ISL_414625	Centre Hospitalier Régional Universitaire de Nantes Laboratoire de Virologie	National Reference Center for Viruses of Respiratory Infections, Institut Pasteur, Paris	Mélnie Albert, Marion Barbet, Sylvie Behillil, Méline Bizard, Angela Brisebarre, Flora Donati Vincent Enouf, Maud Vanpeene, Sylvie van der Werf, Marianne Coste-Burel
EPI_ISL_414626	unknown	National Reference Center for Viruses of Respiratory Infections, Institut Pasteur, Paris	Mélnie Albert, Marion Barbet, Sylvie Behillil, Méline Bizard, Angela Brisebarre, Flora Donati Vincent Enouf, Maud Vanpeene, Sylvie van der Werf
EPI_ISL_414627, EPI_ISL_414628, EPI_ISL_414629, EPI_ISL_414630	Centre Hospitalier Compiègne Laboratoire de Biologie	National Reference Center for Viruses of Respiratory Infections, Institut Pasteur, Paris	Mélnie Albert, Marion Barbet, Sylvie Behillil, Méline Bizard, Angela Brisebarre, Flora Donati Vincent Enouf, Maud Vanpeene, Sylvie van der Werf, Raulin Olivia
EPI_ISL_414631, EPI_ISL_414632	Hôpital Robert Debré Laboratoire de Virologie	National Reference Center for Viruses of Respiratory	Mélnie Albert, Marion Barbet, Sylvie Behillil, Méline Bizard, Angela Brisebarre, Flora Donati Vincent Enouf, Maud Vanpeene, Sylvie van der Werf, Laurent

		Infections, Institut Pasteur, Paris	Andreoletti
EPI_ISL_414633	Centre Hospitalier René Dubois Laboratoire de Microbiologie - Bât A	National Reference Center for Viruses of Respiratory Infections, Institut Pasteur, Paris	Mélnie Albert, Marion Barbet, Sylvie Behillil, Méline Bizard, Angela Brisebarre, Flora Donati Vincent Enouf, Maud Vanpeene, Sylvie van der Werf, Pascale Martres
EPI_ISL_414634, EPI_ISL_414635, EPI_ISL_414636, EPI_ISL_414637, EPI_ISL_414638	Centre Hospitalier Compiègne Laboratoire de Biologie	National Reference Center for Viruses of Respiratory Infections, Institut Pasteur, Paris	Mélnie Albert, Marion Barbet, Sylvie Behillil, Méline Bizard, Angela Brisebarre, Flora Donati Vincent Enouf, Maud Vanpeene, Sylvie van der Werf, Raulin Olivia
EPI_ISL_414663, EPI_ISL_414691	State Key Laboratory of Respiratory Disease, National Clinical Research Center for Respiratory Disease, Guangzhou Institute of Respiratory Health, the First Affiliated Hospital of Guangzhou Medical University	The First Affiliated Hospital of Guangzhou Medical University & BGI-Shenzhen	Zhao et al
EPI_ISL_414945	Iran National Influenza Center	Iran National Influenza Center	Nazanin Zahra Shafiei Jandaghi, Jila Yavarian,Kaveh Sadeghi, Vahid Salimi, Simin Abbasi, Saeedeh Mahfozi and Talat Mokhtari Azad
EPI_ISL_415153	KU Leuven, Clinical and Epidemiological Virology	KU Leuven, Clinical and Epidemiological Virology	Bert Vanmechelen, Joan Marti-Carreras, Tony Wawina, Marc Van Ranst, Piet Maes
EPI_ISL_415154	KU Leuven, Clincal and Epidemiological Virology	KU Leuven, Clincal and Epidemiological Virology	Bert Vanmechelen, Joan Marti-Careras, Tony Wawina, Marc Van Ranst, Piet Maes.
EPI_ISL_415155	KU Leuven, Clinical and Epidemiological Virology	KU Leuven, Clinical and Epidemiological Virology	Bert Vanmechelen, Joan Marti-Carreras, Tony Wawina, Marc Van Ranst, Piet Maes
EPI_ISL_415156, EPI_ISL_415157, EPI_ISL_415158, EPI_ISL_415159	KU Leuven, Clinical and Epidemiological Virology	KU Leuven, Clinical and Epidemiological Virology	Bert Vanmechelen, Joan Marti-Carreras, Tony Wawina, Piet Maes
EPI_ISL_415641, EPI_ISL_415642, EPI_ISL_415643, EPI_ISL_415644	R. G. Lugar Center for Public Health Research, National Center for Disease Control and Public Health (NCDC) of Georgia.	R. G. Lugar Center for Public Health Research, National Center for Disease Control and Public Health (NCDC) of Georgia.	Nato Kotaria, Marine Murtskhvaladze, Ann Machablishvili, Lela Sabadze, Mari Gavashelidze, Ana Papkiauri, Meri Pantsulaia, Gvantsa Brachveli, Tata Imnadze, Tamar Jashiashvili, Tea Tevdoradze, Ketevan Sidamonidze, Ekaterine Khmaladze, Ekaterine Zhghenti, Roena Sukhiashvili, Mariam Zakalashvili, Lela Urushadze, Magda Dgebuadze, Giorgi Tomashvili, Davit Tsaguria, Ekaterine Zangaladze, Nino Berishvili, Gvantsa Chanturia, Adam Kotorashvili, Maia Alkhazashvili, Irma Burjanadze, Anna Kasradze, Khatuna Zakhashvili, Paata Imnadze, Amiran Gamkrelidze.
EPI_ISL_415649	unknown	National Reference Center for Viruses of Respiratory Infections, Institut Pasteur, Paris	Mélnie Albert, Marion Barbet, Sylvie Behillil, Méline Bizard, Angela Brisebarre, Flora Donati Vincent Enouf, Maud Vanpeene, Sylvie van der Werf
EPI_ISL_415650	Hôpital Instruction des Armées - BEGIN	National Reference Center for Viruses of Respiratory Infections, Institut Pasteur, Paris	Mélnie Albert, Marion Barbet, Sylvie Behillil, Méline Bizard, Angela Brisebarre, Flora Donati Vincent Enouf, Maud Vanpeene, Sylvie van der Werf, Christine Bigaillon
EPI_ISL_415652	unknown	National Reference Center for Viruses of Respiratory Infections, Institut Pasteur, Paris	Mélnie Albert, Marion Barbet, Sylvie Behillil, Méline Bizard, Angela Brisebarre, Flora Donati Vincent Enouf, Maud Vanpeene, Sylvie van der Werf
EPI_ISL_415654	Centre Hospitalier Compiègne Laboratoire de Biologie	National Reference Center for Viruses of Respiratory Infections, Institut Pasteur, Paris	Mélnie Albert, Marion Barbet, Sylvie Behillil, Méline Bizard, Angela Brisebarre, Flora Donati Vincent Enouf, Maud Vanpeene, Sylvie van der Werf, Raulin Olivia
EPI_ISL_415709	State Key Laboratory for Diagnosis and Treatment of Infectious Diseases, National Clinical Research Center for Infectious Diseases, First Affiliated Hospital, Zhejiang University School of Medicine, Hangzhou, China. 310003	State Key Laboratory for Diagnosis and Treatment of Infectious Diseases, National Clinical Research Center for Infectious Diseases, First Affiliated Hospital, Zhejiang University School of Medicine, Hangzhou, China. 310003	Hangping Yao, Nanping Wu, Chao Jiang, Xiangyun Lu, Linfang Cheng, Fumin Liu, Zhigang Wu, Haibo Wu, Changzhong Jin, Min Zheng, Lanjuan Li
EPI_ISL_415710	WHO National Influenza Centre Russian Federation	WHO National Influenza Centre Russian Federation	Andrey Komissarov, Artem Fadeev, Anna Ivanova, Daria Danilenko
EPI_ISL_415711	State Key Laboratory for Diagnosis and Treatment of Infectious Diseases, National Clinical Research Center for Infectious Diseases, First Affiliated Hospital, Zhejiang University School of Medicine, Hangzhou, China. 310003	State Key Laboratory for Diagnosis and Treatment of Infectious Diseases, National Clinical Research Center for Infectious Diseases, First Affiliated Hospital, Zhejiang University School of Medicine, Hangzhou, China. 310003	Hangping Yao, Nanping Wu, Chao Jiang, Xiangyun Lu, Linfang Cheng, Fumin Liu, Zhigang Wu, Haibo Wu, Changzhong Jin, Min Zheng, Lanjuan Li
EPI_ISL_416028	National Influenza Center - Instituto Adolfo Lutz	Instituto Adolfo Lutz, Interdiciplinary Procedures Center, Strategic Laboratory	Claudio Tavares Sacchi, Claudia Regina Gonçalves, Carlos Henrique Camargo, Fabiana Cristina Pereira dos Santos, Daniela Bernardes Borges da Silva, Simone Guadagnucci Morillo, Adriano Abbud, Adriana Bugno, Maria do Carmo Sampaio Tavares Timenetsky, Terezinha Maria de Paiva
EPI_ISL_416029	Laboiratório Fleury	Instituto Adolfo Lutz, Interdiciplinary Procedures Center, Strategic Laboratory	Claudio Tavares Sacchi, Claudia Regina Gonçalves, Carlos Henrique Camargo, Fabiana Cristina Pereira dos Santos, Daniela Bernardes Borges da Silva, Simone Guadagnucci Morillo, Adriano Abbud, Adriana Bugno, Maria do Carmo Sampaio Tavares Timenetsky, Terezinha Maria de Paiva
EPI_ISL_416031, EPI_ISL_416032	National Influenza Center - Instituto Adolfo Lutz	Instituto Adolfo Lutz, Interdiciplinary Procedures Center, Strategic Laboratory	Claudio Tavares Sacchi, Claudia Regina Gonçalves, Carlos Henrique Camargo, Fabiana Cristina Pereira dos Santos, Daniela Bernardes Borges da Silva, Simone Guadagnucci Morillo, Adriano Abbud, Adriana Bugno, Maria do Carmo Sampaio Tavares Timenetsky, Terezinha Maria de Paiva
EPI_ISL_416033, EPI_ISL_416034	Hospital Israelita Albert Einstein	Instituto Adolfo Lutz, Interdiciplinary Procedures Center, Strategic Laboratory	Claudio Tavares Sacchi, Claudia Regina Gonçalves, Carlos Henrique Camargo, Erica Valessa Ramos Gomes, Fabiana Cristina Pereira dos Santos, Daniela Bernardes Borges da Silva, Simone Guadagnucci Morillo, Adriano Abbud, Adriana Bugno, Maria do Carmo Sampaio Tavares Timenetsky, Terezinha Maria de Paiva
EPI_ISL_416035, EPI_ISL_416036	National Influenza Center - Instituto Adolfo Lutz	Instituto Adolfo Lutz, Interdiciplinary Procedures Center, Strategic Laboratory	Claudio Tavares Sacchi, Claudia Regina Gonçalves, Carlos Henrique Camargo, Erica Valessa Ramos Gomes, Fabiana Cristina Pereira dos Santos, Daniela Bernardes Borges da Silva, Simone Guadagnucci Morillo, Adriano Abbud, Adriana Bugno, Maria do Carmo Sampaio Tavares Timenetsky, Terezinha Maria de Paiva
EPI_ISL_416042	State Key Laboratory for Diagnosis and Treatment of Infectious Diseases, National Clinical Research Center for Infectious Diseases, First Affiliated Hospital, Zhejiang University School of Medicine, Hangzhou, China. 310003	State Key Laboratory for Diagnosis and Treatment of Infectious Diseases, National Clinical Research Center for Infectious Diseases, First Affiliated Hospital, Zhejiang University School of Medicine, Hangzhou, China. 310003	Hangping Yao, Nanping Wu, Chao Jiang, Xiangyun Lu, Linfang Cheng, Fumin Liu, Zhigang Wu, Haibo Wu, Changzhong Jin, Min Zheng, Lanjuan Li
EPI_ISL_416044, EPI_ISL_416046, EPI_ISL_416047	State Key Laboratory for Diagnosis and Treatment of Infectious Diseases, National Clinical Research Center for Infectious Diseases, First Affiliated Hospital, Zhejiang University School of Medicine, Hangzhou, China 310003	State Key Laboratory for Diagnosis and Treatment of Infectious Diseases, National Clinical Research Center for Infectious Diseases, First Affiliated Hospital, Zhejiang University School of Medicine, Hangzhou, China 310003	Hangping Yao, Nanping Wu, Chao Jiang, Xiangyun Lu, Linfang Cheng, Fumin Liu, Zhigang Wu, Haibo Wu, Changzhong Jin, Min Zheng, Lanjuan Li
EPI_ISL_416142	Department of Virus and Microbiological Special diagnostics, Statens Serum Institut, Copenhagen, Denmark.	Statens Serum Institute	Morten Rasmussen, Maiken Worsoe Rosenstierne , Anders Fomsgaard
EPI_ISL_416143, EPI_ISL_416144	Department of Virus and Microbiological Special diagnostics, Statens Serum Institut, Copenhagen, Denmark.	ViFU	Morten Rasmussen, Maiken Worsoe Rosenstierne , Anders Fomsgaard
EPI_ISL_416425	State Key Laboratory for Diagnosis and Treatment of Infectious Diseases, National Clinical Research Center for Infectious Diseases, First Affiliated Hospital, Zhejiang University School of Medicine, Hangzhou, China 310003	State Key Laboratory for Diagnosis and Treatment of Infectious Diseases, National Clinical Research Center for Infectious Diseases, First Affiliated Hospital, Zhejiang University School of Medicine, Hangzhou, China 310003	Hangping Yao, Nanping Wu, Chao Jiang, Xiangyun Lu, Linfang Cheng, Fumin Liu, Zhigang Wu, Haibo Wu, Changzhong Jin, Min Zheng, Lanjuan Li
EPI_ISL_416426	Virological Research Group, Szentágothai Research Centre, University of Pécs	Bioinformatics Research Group, Szentágothai Research Centre, University of Pécs	Péter Urbán, Endre Gábor Tóth, Gábor Kemenesi, Róbert Herczeg, Attila Gyenesei, Ferenc Jakab
EPI_ISL_416458	Virology laboratory Ministry of Health Kuwait sequenced at Dasman Diabetes Institute	Dasman Diabetes Institute	Fahd Al-Mulla, Sumi John, Sara Alqabandi, Rasheeba iqbal, Motasem Melhem, Ebaa alOzairi, Qais Al-Duwairi
EPI_ISL_416469, EPI_ISL_416470, EPI_ISL_416471, EPI_ISL_416472	KU Leuven, Clinical and Epidemiological Virology	KU Leuven, Clinical and Epidemiological Virology	Bert Vanmechelen, Tony Wawina, Joan Marti-Carreras, Piet Maes
EPI_ISL_416473, EPI_ISL_416474	State Key Laboratory for Diagnosis and Treatment of Infectious Diseases, National Clinical Research Center for	State Key Laboratory for Diagnosis and Treatment of Infectious Diseases, National Clinical Research Center for	Hangping Yao, Nanping Wu, Chao Jiang, Xiangyun Lu, Linfang Cheng, Fumin Liu, Zhigang Wu, Haibo Wu, Changzhong Jin, Min Zheng, Lanjuan Li

	Infectious Diseases, First Affiliated Hospital, Zhejiang University School of Medicine, Hangzhou, China 310003	Infectious Diseases, First Affiliated Hospital, Zhejiang University School of Medicine, Hangzhou, China 310003	
EPI_ISL_416475	KU Leuven, Clinical and Epidemiological Virology	KU Leuven, Clinical and Epidemiological Virology	Bert Vanmechelen, Tony Wawina, Joan Marti-Carreras, Piet Maes
EPI_ISL_416485	Servicio de Microbiologia. Consorcio Hospital General Universitario de Valencia	Sequencing and Bioinformatics Service and Molecular Epidemiology Research Group. FISABIO-Public Health	Griselda De Marco, Neris Garcia-Gonzalez, Maria Alma Bracho, Maria Dolores Ocete, Concepcion Gimeno, Giuseppe D'Auria, Fernando Gonzalez-Candelas
EPI_ISL_416486	Servicio de Microbiología. Consorcio Hospital General Universitario de Valencia	Sequencing and Bioinformatics Service and Molecular Epidemiology Research Group. FISABIO-Public Health	Neris Garcia-Gonzalez, Maria Alma Bracho, Maria Dolores Ocete, Concepcion Gimeno, Giuseppe D'Auria, Griselda De Marco, Fernando Gonzalez-Candelas
EPI_ISL_416487	Servicio de Microbiología. Consorcio Hospital General Universitario de Valencia	Sequencing and Bioinformatics Service and Molecular Epidemiology Research Group. FISABIO-Public Health	Giuseppe D'Auria, Griselda De Marco, Neris Garcia-Gonzalez, Maria Alma Bracho, Maria Dolores Ocete, Concepcion Gimeno, Fernando Gonzalez-Candelas
EPI_ISL_416488	ViroGenetics - BSL3 Laboratory of Virology; Human Genome Variation Research Group & Genomics Centre MCB; Bioinformatics Research Group Department of Virology	ViroGenetics - BSL3 Laboratory of Virology; Human Genome Variation Research Group & Genomics Centre MCB; Bioinformatics Research Group Department of Virology	Aleksandra Milewska, Ewelina Popiech, Agata Jarosz, Adrianna Klajmon, Kamila Marszaek, Katarzyna Pancer, Magdalena Rzeczkowska, Tomasz Wokowicz, Katarzyna Zacharczuk, Agnieszka Koakowska-Kulesza, Natalia Wolaniuk, Ewelina Hallman-Szeliska, Pawe P abaj, Wojciech Branicki, Krzysztof Pyr
EPI_ISL_416493	CH Jean de Navarre Laboratoire de Biologie	National Reference Center for Viruses of Respiratory Infections, Institut Pasteur, Paris	Mélnie Albert, Marion Barbet, Sylvie Behillil, Méline Bizard, Angela Brisebarre, Flora Donati, Etienne Simon-Lorière, Vincent Enouf, Maud Vanpeene, Sylvie van der Werf
EPI_ISL_416494	Centre Hositalier Universitaire de Rouen Laboratoire de Virologie	National Reference Center for Viruses of Respiratory Infections, Institut Pasteur, Paris	Mélnie Albert, Marion Barbet, Sylvie Behillil, Méline Bizard, Angela Brisebarre, Flora Donati, Etienne Simon-Lorière, Vincent Enouf, Maud Vanpeene, Sylvie van der Werf, Jean-Christophe Plantier
EPI_ISL_416495, EPI_ISL_416496, EPI_ISL_416497	Centre Hospitalier Compiègne Laboratoire de Biologie	National Reference Center for Viruses of Respiratory Infections, Institut Pasteur, Paris	Mélnie Albert, Marion Barbet, Sylvie Behillil, Méline Bizard, Angela Brisebarre, Flora Donati, Etienne Simon-Lorière, Vincent Enouf, Maud Vanpeene, Sylvie van der Werf, Raulin Olivia
EPI_ISL_416498	Institut Médico légal- Hop R. Poincaré	National Reference Center for Viruses of Respiratory Infections, Institut Pasteur, Paris	Mélnie Albert, Marion Barbet, Sylvie Behillil, Méline Bizard, Angela Brisebarre, Flora Donati, Etienne Simon-Lorière, Vincent Enouf, Maud Vanpeene, Sylvie van der Werf
EPI_ISL_416499, EPI_ISL_416500	LABM GH nord Essonne	National Reference Center for Viruses of Respiratory Infections, Institut Pasteur, Paris	Mélnie Albert, Marion Barbet, Sylvie Behillil, Méline Bizard, Angela Brisebarre, Flora Donati, Etienne Simon-Lorière, Vincent Enouf, Maud Vanpeene, Sylvie van der Werf
EPI_ISL_416501	Hopital franco britannique - Service des Urgences	National Reference Center for Viruses of Respiratory Infections, Institut Pasteur, Paris	Mélnie Albert, Marion Barbet, Sylvie Behillil, Méline Bizard, Angela Brisebarre, Flora Donati, Etienne Simon-Lorière, Vincent Enouf, Maud Vanpeene, Sylvie van der Werf
EPI_ISL_416502, EPI_ISL_416503, EPI_ISL_416504, EPI_ISL_416505, EPI_ISL_416506, EPI_ISL_416507, EPI_ISL_416508, EPI_ISL_416509, EPI_ISL_416510, EPI_ISL_416511, EPI_ISL_416512, EPI_ISL_416513			
see above	CHRU Pontchaillou - Laboratoire de Virologie	National Reference Center for Viruses of Respiratory Infections, Institut Pasteur, Paris	Mélnie Albert, Marion Barbet, Sylvie Behillil, Méline Bizard, Angela Brisebarre, Flora Donati, Etienne Simon-Lorière, Vincent Enouf, Maud Vanpeene, Sylvie van der Werf, Gisèle Lagathu
EPI_ISL_416565, EPI_ISL_416566, EPI_ISL_416567, EPI_ISL_416569, EPI_ISL_416570, EPI_ISL_416571, EPI_ISL_416572, EPI_ISL_416573, EPI_ISL_416574, EPI_ISL_416575, EPI_ISL_416576, EPI_ISL_416577, EPI_ISL_416578, EPI_ISL_416579, EPI_ISL_416580, EPI_ISL_416581, EPI_ISL_416582, EPI_ISL_416583, EPI_ISL_416585, EPI_ISL_416586, EPI_ISL_416587, EPI_ISL_416588, EPI_ISL_416589, EPI_ISL_416590, EPI_ISL_416591, EPI_ISL_416592, EPI_ISL_416593, EPI_ISL_416594, EPI_ISL_416595, EPI_ISL_416596, EPI_ISL_416597, EPI_ISL_416598, EPI_ISL_416599, EPI_ISL_416600, EPI_ISL_416601, EPI_ISL_416602, EPI_ISL_416603, EPI_ISL_416604, EPI_ISL_416605, EPI_ISL_416606, EPI_ISL_416607, EPI_ISL_416608, EPI_ISL_416609, EPI_ISL_416610, EPI_ISL_416611, EPI_ISL_416612, EPI_ISL_416613, EPI_ISL_416614, EPI_ISL_416615, EPI_ISL_416617, EPI_ISL_416618, EPI_ISL_416619, EPI_ISL_416620, EPI_ISL_416621, EPI_ISL_416622, EPI_ISL_416624, EPI_ISL_416625, EPI_ISL_416626, EPI_ISL_416627, EPI_ISL_416628, EPI_ISL_416629, EPI_ISL_416630, EPI_ISL_416631, EPI_ISL_416632, EPI_ISL_416633, EPI_ISL_416634			
see above	Japanese Quarantine Stations	Pathogen Genomics Center, National Institute of Infectious Diseases	Tsuyoshi Sekizuka, Kentaro Itokawa, Rina Tanaka, Masanori Hashino, Tsutomu Kageyama, Shinji Saito, Ikuyo Takayama, Hideki Hasegawa, Takuri Takahashi, Hajime Kamiya, Takuya Yamagishi, Motoi Suzuki, Takaji Wakita, Makoto Kuroda
EPI_ISL_416744	Virological Research Group, Szentágothai Research Centre	Bioinformatics Research Group, Szentágothai Research Centre	Péter Urbán, Endre Gábor Tóth, Gábor Kemenesi, Róbert Herczeg, Attila Gyenesei, Ferenc Jakab
EPI_ISL_416829	National Public Health Laboratory	Malaysia Genome Institute	Mohd Noor Mat Isa, Irni Suhayu Sapian, Yusuf Muhammad Noor, Nurhezreen Md Iqbal, Mohd Faizal Abu Bakar, Enizza Kasim, Shamsidar Sopie, Siti Noraini Othman, Azrin Ahmad, Nor Azfa Johari, Norazimah Tajudin, Noorliza Mohamad Noordin, W Afiza W Mohd Arifin, Rehan Shuhada Abu Bakar, Yu Kie Chem, Selvanesan Sengol, Hani Mat Hussin, Shahrul Hisham Zainal Ariffin
EPI_ISL_416866	National Public Health Laboratory	Malaysia Genome Institute	Mohd Noor Mat Isa, Irni Suhayu Sapian, Yusuf Muhammad Noor, Nurhezreen Md Iqbal, Mohd Faizal Abu Bakar, Enizza Kasim, Shamsidar Sopie, Siti Noraini Othman, Azrin Ahmad, Nor Azfa Johari, Norazimah Tajudin, Noorliza Mohamad Noordin, W Afiza W Mohd Arifin, Rehan Shuhada Abu Bakar, Yu Kie Chem, Selvanesan Sengol, Hani Mat Hussin, Shahrul Hisham Zainal Ariffin
EPI_ISL_416885, EPI_ISL_416886, EPI_ISL_416907	National Public Health Laboratory	Malaysia Genome Institute	Mohd Noor Mat Isa, Irni Suhayu Sapian, Yusuf Muhammad Noor, Nurhezreen Md Iqbal, Mohd Faizal Abu Bakar, Enizza Kasim, Shamsidar Sopie, Siti Noraini Othman, Azrin Ahmad, Nor Azfa Johari, Norazimah Tajudin, Noorliza Mohamad Noordin, W Afiza W Mohd Arifin, Rehan Shuhada Abu Bakar, Yu Kie Chem, Selvanesan Sengol, Hani Mat Hussin, Shahrul Hisham Zainal Ariffin
EPI_ISL_416994	COMPLEJO ASISTENCIAL UNIVERSITARIO DE BURGOS	Instituto de Salud Carlos III	Iglesias-Caballero, M. Molinero Calamita, M. González-Esguevillas, M. Camarero S. Pozo F. Casas I. Jiménez P. Jiménez M. Zaballos A. Monzón, S. Varona, S. Juliá M. Cuesta I. Megias Lobón, G. Hospital: ------
EPI_ISL_417007	HOSPITAL SANTA MARIA NAI	Instituto de Salud Carlos III	Iglesias-Caballero, M. Molinero Calamita, M. González-Esguevillas, M. Camarero S. Pozo F. Casas I. Jiménez, P. Jiménez, M. Zaballos, A. Monzón, S. Varona, S. Juliá, M. Cuesta, I. García Costa, J.
EPI_ISL_417010	FUNDACION JIMENEZ DIAZ	Instituto de Salud Carlos III	Iglesias-Caballero, M. Molinero Calamita, M. González-Esguevillas, M. Camarero, S. Pozo, F. Casas, I. Jiménez, P. Jiménez, M. Zaballos, A. Monzón, S. Varona, S. Juliá, M. Cuesta, I. Fernández Roblas, R.
EPI_ISL_417015, EPI_ISL_417016, EPI_ISL_417018, EPI_ISL_417019	Department of Clinical Microbiology	GIGA Medical Genomics	Durkin Keith, Artesi Maria, Bontems Sébastien, Boreux Raphaël, Meex Cécile, Melin Pierrette, Hayette Marie-Pierre, Bours Vincent.
EPI_ISL_417030	Centre for Infectious Diseases and Microbiology Laboratory Services	NSW Health Pathology - Institute of Clinical Pathology and Medical Research; Westmead Hospital; University of Sydney	Eden J-S, Rockett R, Carter I, Rahman H, Holmes EC, O'Sullivan MV, Sintchenko V, Chen SC, Maddocks S, Kok J and Dwyer DE for the 2019-nCoV Study Group*
EPI_ISL_417034	Laboratorio de Ecologia de Doencas Transmissiveis na Amazonia, Instituto Leonidas e Maria Deane - Fiocruz Amazonia	Laboratorio de Ecologia de Doencas Transmissiveis na Amazonia, Instituto Leonidas e Maria Deane - Fiocruz Amazonia	Valdinete Nascimento, André Corado, Fernanda Nascimento, Ágatha Costa, Debora Duarte, Luciana Gonçalves, Michele Jesus, Sérgio Luz, Felipe Naveca on behalf of the Fiocruz COVID-19 Genomic Surveillance Network
EPI_ISL_417176	Department of Pathology, Princess Margaret Hospital	Department of Health Technology and Informatics, Faculty of Health and Social Science, The Hong Kong Polytechnic University	Kenneth Siu-Sing LEUNG, Timothy Ting-Leung NG, Alan Ka-Lun WU, Miranda Chong-Yee YAU, Hiu-Yin LAO, Ming-Pan CHOI, Kingsley King-Gee TAM, Lam-Kwong LEE, Barry Kin-Chung WONG, Alex Yat-Man HO, Kam-Tong Yip, Kwok-Cheung LUNG, Raymond Wai-To LIU, Eugene Yuk-Keung TSO, Wai-Shing LEUNG, Man-Chun CHAN, Yuk-Yung NG, Kit-Man SIN, Kitty Sau-Chun FUNG, Sandy Ka-Yee CHAU, Wing-Kin TO, Tak-Lun Que, David Ho-Keung SHUM, Shea Ping YIP, Wing Cheong YAM, Gilman Kit-Hang SIU
EPI_ISL_417181, EPI_ISL_417185	Department of Pathology, United Christian Hospital	Department of Health Technology and Informatics, Faculty of Health and Social Science, The Hong Kong Polytechnic University	Kenneth Siu-Sing LEUNG, Timothy Ting-Leung NG, Alan Ka-Lun WU, Miranda Chong-Yee YAU, Hiu-Yin LAO, Ming-Pan CHOI, Kingsley King-Gee TAM, Lam-Kwong LEE, Barry Kin-Chung WONG, Alex Yat-Man HO, Kam-Tong Yip, Kwok-Cheung LUNG, Raymond Wai-To LIU, Eugene Yuk-Keung TSO, Wai-Shing LEUNG, Man-Chun CHAN, Yuk-Yung NG, Kit-Man SIN, Kitty Sau-Chun FUNG, Sandy Ka-Yee CHAU, Wing-Kin TO, Tak-Lun Que, David Ho-Keung SHUM, Shea Ping YIP, Wing Cheong YAM, Gilman Kit-Hang SIU
EPI_ISL_417186	National Institute for Communicable Diseases of the National Health Laboratory Service	National Institute for Communicable Diseases of the National Health Laboratory Service	Allam M, Kwenda S, van Heusden P, Khumalo Z, Mohale T, Subramoney K, von Gottberg, A, Ismail A, Bhiman JN
EPI_ISL_417187, EPI_ISL_417188, EPI_ISL_417193	Department of Clinical Pathology, Pamela Youde Nethersole Eastern Hospital	Department of Health Technology and Informatics, Faculty of Health and Social Science, The Hong Kong Polytechnic University	Kenneth Siu-Sing LEUNG, Timothy Ting-Leung NG, Alan Ka-Lun WU, Miranda Chong-Yee YAU, Hiu-Yin LAO, Ming-Pan CHOI, Kingsley King-Gee TAM, Lam-Kwong LEE, Barry Kin-Chung WONG, Alex Yat-Man HO, Kam-Tong Yip, Kwok-Cheung LUNG, Raymond Wai-To LIU, Eugene Yuk-Keung TSO, Wai-Shing LEUNG, Man-Chun CHAN, Yuk-Yung NG, Kit-Man SIN, Kitty Sau-Chun FUNG, Sandy Ka-Yee CHAU, Wing-Kin TO, Tak-Lun Que, David Ho-Keung SHUM, Shea Ping YIP, Wing Cheong YAM, Gilman Kit-Hang SIU

EPI_ISL_417333, EPI_ISL_417334, EPI_ISL_417335, EPI_ISL_417336, EPI_ISL_417337, EPI_ISL_417339	Institut des Agents Infectieux (IAI), Hospices Civils de Lyon	CNR Virus des Infections Respiratoires - France SUD	Antonin Bal, Gregory Destras, Gwendolyne Burfin, Solenne Brun, Carine Moustaud, Raphaelle Lamy, Alexandre Gaymard, Maude Bouscambert-Duchamp, Florence Morfin-Sherpa, Martine Valette, Laurence Josset, Bruno Lina
EPI_ISL_417443	State Key Laboratory for Emerging Infectious Diseases Department of Microbiology Li Ka Shing Faculty of Medicine The University of Hong Kong	State Key Laboratory for Emerging Infectious Diseases Department of Microbiology Li Ka Shing Faculty of Medicine The University of Hong Kong	Pui Wang, Siu-Ying Lau, Shaofeng Deng, Bobo Wing-Yee Mok, Wenjun Song, Kwok-Yung Yuen, Honglin Chen
EPI_ISL_417491	Virology Laboratory, Department of Biomedical Sciences and Public Health, University Politecnica delle Marche	Virology and Legal Medicine Laboratories, Department of Biomedical Sciences and Public Health, University Politecnica delle Marche	Bagnarelli,P., Caucci,S., Di Sante,L., Menzo,S., Alessandrini,F., Onofri,V., Turchi,C., Tagliabracci,A.
EPI_ISL_417877, EPI_ISL_417878, EPI_ISL_417879, EPI_ISL_417880	Institute of Virology, Biomedical Research Center of the Slovak Academy of Sciences, Bratislava; Public Health Authority of the Slovak Republic, Bratislava	Institute of Virology, Biomedical Research Center of the Slovak Academy of Sciences, Bratislava; Comenius University Science Park, Bratislava	Monika Slávikova, Martina Liková, Sabina Fumaová Havlíková, Juraj Koi, Juraj Kopáek, Elena Tichá, Edita Staroová, Jaroslav Budiš, Werner Krampl, Miroslav Böhmer, Diana Rusáková, Tomáš Szemeš, Boris Klempa
EPI_ISL_417924	Secretaría de Salud Medellín	Instituto Nacional de Salud, Universidad Cooperativa de Colombia, Instituto Alexander von Humboldt, Imperial College-London, London School of Hygiene & Tropical Medicine	Marcela Mercado-Reyes, Katherine Laiton-Donato, Diego A. Álvarez-Díaz, Carlos Franco-Muñoz, Jose A. Usme-Ciro, Gloria Puerto, Nicolás D. Franco-Sierra, Mailyn A. Gonzalez, Zulma M. Cucunubá, Christian Julian VillabonaArenas, Liz Villabona-Arenas, Sussy Echeverria-Londoño, Astrid C. Flórez, Sergio Gomez Rangel, Luz Dary Rodriguez, Juliana Barbosa, Erika Ospitia, Diana Marcela Walteros-Acero, Martha Lucia Ospina Martinez
EPI_ISL_418183	Virological Research Group, Szentágothai Research Centre	Bioinformatics Research Group, Szentágothai Research Centre	Péter Urbán, Endre Gábor Tóth, Gábor Kemenesi, Róbert Herczeg, Attila Gyenesei, Ferenc Jakab
EPI_ISL_418206, EPI_ISL_418207, EPI_ISL_418208, EPI_ISL_418209, EPI_ISL_418210, EPI_ISL_418211	Institut Pasteur Dakar	Institut Pasteur de Dakar	Ndongo Dia, Ousmane Faye, Amadou Alpha Sall
EPI_ISL_418212	Institut Pasteur Dakar	Institut Pasteur de Dakar	Ndongo Dia, Ousmane Faye, Amadou Alpha sall
EPI_ISL_418213	Institut Pasteur Dakar	Institut Pasteur de Dakar	Ndongo Dia, Ousmane Faye, Amadou Alpha Sall
EPI_ISL_418215	Instirut Pasteur Dakar	Institut Pasteur de Dakar	Ndongo Dia, Ousmane Faye, Amadou Alpha Sall
EPI_ISL_418216, EPI_ISL_418217	Institut Pasteur Dakar	Institut Pasteur de Dakar	Ndongo Dia, Ousmane Faye, Amadou Alpha Sall
EPI_ISL_418218	Centre Hospitalier Compiègne Laboratoire de Biologie	National Reference Center for Viruses of Respiratory Infections, Institut Pasteur, Paris	Mélanie Albert, Marion Barbet, Sylvie Behillil, Méline Bizard, Angela Brisebarre, Flora Donati, Fabiana Gambaro, Etienne Simon-Lorière, Vincent Enouf, Maud Vanpeene, Sylvie van der Werf, Raulin Olivia
EPI_ISL_418219	CHU - Hôpital Cavale Blanche - Labo. de Virologie	National Reference Center for Viruses of Respiratory Infections, Institut Pasteur, Paris	Mélanie Albert, Marion Barbet, Sylvie Behillil, Méline Bizard, Angela Brisebarre, Flora Donati, Fabiana Gambaro, Etienne Simon-Lorière, Vincent Enouf, Maud Vanpeene, Sylvie van der Werf, Léa Pilorge
EPI_ISL_418220, EPI_ISL_418221	Centre Hospitalier Compiègne Laboratoire de Biologie	National Reference Center for Viruses of Respiratory Infections, Institut Pasteur, Paris	Mélanie Albert, Marion Barbet, Sylvie Behillil, Méline Bizard, Angela Brisebarre, Flora Donati, Fabiana Gambaro, Etienne Simon-Lorière, Vincent Enouf, Maud Vanpeene, Sylvie van der Werf, Raulin Olivia
EPI_ISL_418222	CHRU Bretonneau - Serv. Bacterio-Virol.	National Reference Center for Viruses of Respiratory Infections, Institut Pasteur, Paris	Mélanie Albert, Marion Barbet, Sylvie Behillil, Méline Bizard, Angela Brisebarre, Flora Donati, Fabiana Gambaro, Etienne Simon-Lorière, Vincent Enouf, Maud Vanpeene, Sylvie van der Werf, Julien Marlet
EPI_ISL_418223, EPI_ISL_418224, EPI_ISL_418225	Centre Hospitalier Compiègne Laboratoire de Biologie	National Reference Center for Viruses of Respiratory Infections, Institut Pasteur, Paris	Mélanie Albert, Marion Barbet, Sylvie Behillil, Méline Bizard, Angela Brisebarre, Flora Donati, Fabiana Gambaro, Etienne Simon-Lorière, Vincent Enouf, Maud Vanpeene, Sylvie van der Werf, Raulin Olivia
EPI_ISL_418226	EHPAD - Résidences les Cèdres	National Reference Center for Viruses of Respiratory Infections, Institut Pasteur, Paris	Mélanie Albert, Marion Barbet, Sylvie Behillil, Méline Bizard, Angela Brisebarre, Flora Donati, Etienne Simon-Lorière, Vincent Enouf, Maud Vanpeene, Sylvie van der Werf
EPI_ISL_418227, EPI_ISL_418228	Centre Hospitalier Compiègne Laboratoire de Biologie	National Reference Center for Viruses of Respiratory Infections, Institut Pasteur, Paris	Mélanie Albert, Marion Barbet, Sylvie Behillil, Méline Bizard, Angela Brisebarre, Flora Donati, Etienne Simon-Lorière, Vincent Enouf, Maud Vanpeene, Sylvie van der Werf, Raulin Olivia
EPI_ISL_418229	Hopital franco britannique - Laboratoire	National Reference Center for Viruses of Respiratory Infections, Institut Pasteur, Paris	Mélanie Albert, Marion Barbet, Sylvie Behillil, Méline Bizard, Angela Brisebarre, Flora Donati, Etienne Simon-Lorière, Vincent Enouf, Maud Vanpeene, Sylvie van der Werf, Marianne Asso Bonnet
EPI_ISL_418230	Clinique AVERAY LA BROUSTE, Med. Polyvalente	National Reference Center for Viruses of Respiratory Infections, Institut Pasteur, Paris	Mélanie Albert, Marion Barbet, Sylvie Behillil, Méline Bizard, Angela Brisebarre, Flora Donati, Etienne Simon-Lorière, Vincent Enouf, Maud Vanpeene, Sylvie van der Werf, Elsa Ngwem
EPI_ISL_418231	Centre Hospitalier Compiègne Laboratoire de Biologie	National Reference Center for Viruses of Respiratory Infections, Institut Pasteur, Paris	Mélanie Albert, Marion Barbet, Sylvie Behillil, Méline Bizard, Angela Brisebarre, Flora Donati, Etienne Simon-Lorière, Vincent Enouf, Maud Vanpeene, Sylvie van der Werf, Raulin Olivia
EPI_ISL_418232, EPI_ISL_418233	Service des Urgences	National Reference Center for Viruses of Respiratory Infections, Institut Pasteur, Paris	Mélanie Albert, Marion Barbet, Sylvie Behillil, Méline Bizard, Angela Brisebarre, Flora Donati, Etienne Simon-Lorière, Vincent Enouf, Maud Vanpeene, Sylvie van der Werf, Boubkeur
EPI_ISL_418234	LABM GH nord Essonne	National Reference Center for Viruses of Respiratory Infections, Institut Pasteur, Paris	Mélanie Albert, Marion Barbet, Sylvie Behillil, Méline Bizard, Angela Brisebarre, Flora Donati, Etienne Simon-Lorière, Vincent Enouf, Maud Vanpeene, Sylvie van der Werf, Christine Lambert
EPI_ISL_418236, EPI_ISL_418237, EPI_ISL_418238, EPI_ISL_418239	Centre Hospitalier Compiègne Laboratoire de Biologie	National Reference Center for Viruses of Respiratory Infections, Institut Pasteur, Paris	Mélanie Albert, Marion Barbet, Sylvie Behillil, Méline Bizard, Angela Brisebarre, Flora Donati, Etienne Simon-Lorière, Vincent Enouf, Maud Vanpeene, Sylvie van der Werf, Raulin Olivia
EPI_ISL_418240	LABM GH nord Essonne	National Reference Center for Viruses of Respiratory Infections, Institut Pasteur, Paris	Mélanie Albert, Marion Barbet, Sylvie Behillil, Méline Bizard, Angela Brisebarre, Flora Donati, Etienne Simon-Lorière, Vincent Enouf, Maud Vanpeene, Sylvie van der Werf, Christine Lambert
EPI_ISL_418241, EPI_ISL_418242	NIC Viral Respiratory Unit - Institut Pasteur of Algeria	National Reference Center for Viruses of Respiratory Infections, Institut Pasteur, Paris	Mélanie Albert, Marion Barbet, Sylvie Behillil, Méline Bizard, Angela Brisebarre, Flora Donati, Etienne Simon-Lorière, Vincent Enouf, Maud Vanpeene, Sylvie van der Werf, Fawzi Derrar
EPI_ISL_418243, EPI_ISL_418244	HOSPITAL UNIVERSITARIO VIRGEN DE LAS NIEVES	Instituto de Salud Carlos III	Iglesias-Caballero, M. Molinero Calamita, M. González-Esguevillas, M. Camarero, S. Pozo, F. Casas, I. Jiménez, P. Jiménez, M. Zaballos, A. Monzón, S. Varona, S. Juliá, M. Cuesta, I. Sanbonmatsu S.
EPI_ISL_418245, EPI_ISL_418246	Hospital General y Universitario de Guadalajara	Instituto de Salud Carlos III	Iglesias-Caballero, M. Molinero Calamita, M. González-Esguevillas, M. Camarero, S. Pozo, F. Casas, I. Jiménez, P. Jiménez, M. Zaballos, A. Monzón, S. Varona, S. Juliá, M. Cuesta, I. Gonzalez-Praetorius A.
EPI_ISL_418247	HOSPITAL GENERAL DE SEGOVIA	Instituto de Salud Carlos III	Iglesias-Caballero, M. Molinero Calamita, M. González-Esguevillas, M. Camarero, S. Pozo, F. Casas, I. Jiménez, P. Jiménez, M. Zaballos, A. Monzón, S. Varona, S. Juliá, M. Cuesta, I. Hernando-Real S.
EPI_ISL_418248, EPI_ISL_418249	COMPLEJO ASISTENCIAL UNIVERSITARIO DE BURGOS	Instituto de Salud Carlos III	Iglesias-Caballero, M. Molinero Calamita, M. González-Esguevillas, M. Camarero, S. Pozo, F. Casas, I. Jiménez, P. Jiménez, M. Zaballos, A. Monzón, S. Varona, S. Juliá, M. Cuesta, I. Megias-Lobon G.
EPI_ISL_418250	HOSPITAL CLINIC	Instituto de Salud Carlos III	Iglesias-Caballero, M. Molinero Calamita, M. González-Esguevillas, M. Camarero, S. Pozo, F. Casas, I. Jiménez, P. Jiménez, M. Zaballos, A. Monzón, S. Varona, S. Juliá, M. Cuesta, I. Marcos M.A
EPI_ISL_418251	HOSPITAL UNIVERSITARIO LA PAZ	Instituto de Salud Carlos III	Iglesias-Caballero, M. Molinero Calamita, M. González-Esguevillas, M. Camarero, S. Pozo, F. Casas, I. Jiménez, P. Jiménez, M. Zaballos, A. Monzón, S. Varona, S. Juliá, M. Cuesta, I. Romero P.
EPI_ISL_418252	FUNDACION JIMENEZ DIAZ	Instituto de Salud Carlos III	Iglesias-Caballero, M. Molinero Calamita, M. González-Esguevillas, M. Camarero, S. Pozo, F. Casas, I. Jiménez, P. Jiménez, M. Zaballos, A. Monzón, S. Varona, S. Juliá, M. Cuesta, I. Fernández Roblas, R.
EPI_ISL_418253	HOSPITAL TXAGORRITXU	Instituto de Salud Carlos III	Iglesias-Caballero, M. Molinero Calamita, M. González-Esguevillas, M. Camarero, S. Pozo, F. Casas, I. Jiménez, P. Jiménez, M. Zaballos, A. Monzón, S. Varona, S. Juliá, M. Cuesta, I. Gomez-Gonzalez C.

EPI_ISL_418256	Ospedale "San Liberatore" di Atri	Istituto Zooprofilattico Sperimentale dell'Abruzzo e Molise "G. Caporale"	Lorusso A, Marcacci M, Di Domenico M, Puglia I, Curini V, Ancora M, Di Pasquale A, Rinaldi A, Mangone I, Cammà C, Savini G.
EPI_ISL_418257	Ospedale Civile Giuseppe Mazzini, Teramo	Istituto Zooprofilattico Sperimentale dell'Abruzzo e Molise "G.Caporale"	Lorusso A, Marcacci M, Di Domenico M, Puglia I, Curini V, Ancora M, Di Pasquale A, Rinaldi A, Mangone I, Cammà C, Savini G.
EPI_ISL_418262	Instituto Nacional de Salud	Instituto Nacional de Salud Universidad Cooperativa de Colombia Instituto Alexander von Humboldt Imperial College-London London School of Hygiene & Tropical Medicine	Marcela Mercado-Reyes, Katherine Laiton-Donato, Diego A. Álvarez-Díaz, Carlos Franco-Muñoz, Jose A. Usme-Ciro, Gloria Puerto, Nicolas D. Franco-Sierra, Mailyn A.Gonzalez, Zulma M. Cucunubá, Christian Julian VillabonaArenas, Liz Villabona-Arenas, Sussy Echeverria, Astrid C. Flórez, Sergio Gomez Rangel, Luz Dary Rodriguez, Juliana Barbosa, Erika Ospitia, Diana Marcela Walteros-Acero, Nuno Rodrigues Faria, Martha Lucia Ospina Martinez
EPI_ISL_418624, EPI_ISL_418633, EPI_ISL_418645, EPI_ISL_418646, EPI_ISL_418648, EPI_ISL_418649, EPI_ISL_418650, EPI_ISL_418651, EPI_ISL_418652, EPI_ISL_418659, EPI_ISL_418660, EPI_ISL_418664			
see above	Department of Clinical Microbiology	GIGA Medical Genomics	Keith Durkin, Maria Artesi, Sébastien Bontems, Raphaël Boreux, Cécile Meex, Pierrette Melin, Marie-Pierre Hayette, Vincent Bours.
EPI_ISL_418800	KU Leuven, Clinical and Epidemiological Virology	KU Leuven, Clinical and Epidemiological Virology	Bert Vanmechelen, Joan Marti-Carreras, Tony Wawina, Piet Maes
EPI_ISL_418815	Department of Clinical Pathology, Pamela Youde Nethersole Eastern Hospital	Department of Health Technology and Informatics, Faculty of Health and Social Science, The Hong Kong Polytechnic University	Kenneth Siu-Sing LEUNG, Timothy Ting-Leung NG, Alan Ka-Lun WU, Miranda Chong-Yee YAU, Hiu-Yin LAO, Ming-Pan CHOI, Kingsley King-Gee TAM, Lam-Kwong LEE, Barry Kin-Chung WONG, Alex Yat-Man HO, Kam-Tong YIP, Kwok-Cheung LUNG, Raymond Wai-To LIU, Eugene Yuk-Keung TSO, Wai-Shing LEUNG, Man-Chun CHAN, Yuk-Yung NG, Kit-Man SIN, Kitty Sau-Chun FUNG, Sandy Ka-Yee CHAU, Wing-Kin TO, Tak-Lun QUE, David Ho-Keung SHUM, Shea Ping YIP, Wing Cheong YAM, Gilman Kit-Hang SIU
EPI_ISL_418990, EPI_ISL_418991	State Key Laboratory for Diagnosis and Treatment of Infectious Diseases, National Clinical Research Center for Infectious Diseases, First Affiliated Hospital, Zhejiang University School of Medicine, Hangzhou, China 310003	State Key Laboratory for Diagnosis and Treatment of Infectious Diseases, National Clinical Research Center for Infectious Diseases, First Affiliated Hospital, Zhejiang University School of Medicine, Hangzhou, China 310003	Hangping Yao, Nanping Wu, Chao Jiang, Xiangyun Lu, Linfang Cheng, Fumin Liu, Zhigang Wu, Haibo Wu, Changzhong Jin, Min Zheng, Lanjuan Li
EPI_ISL_419214, EPI_ISL_419215, EPI_ISL_419216	Department of Clinical Pathology, Pamela Youde Nethersole Eastern Hospital	Department of Health Technology and Informatics, Faculty of Health and Social Science, The Hong Kong Polytechnic University	Kenneth Siu-Sing LEUNG, Timothy Ting-Leung NG, Alan Ka-Lun WU, Miranda Chong-Yee YAU, Hiu-Yin LAO, Ming-Pan CHOI, Kingsley King-Gee TAM, Lam-Kwong LEE, Barry Kin-Chung WONG, Alex Yat-Man HO, Kam-Tong YIP, Kwok-Cheung LUNG, Raymond Wai-To LIU, Eugene Yuk-Keung TSO, Wai-Shing LEUNG, Man-Chun CHAN, Yuk-Yung NG, Kit-Man SIN, Kitty Sau-Chun FUNG, Sandy Ka-Yee CHAU, Wing-Kin TO, Tak-Lun QUE, David Ho-Keung SHUM, Shea Ping YIP, Wing Cheong YAM, Gilman Kit-Hang SIU
EPI_ISL_419217	Department of Pathology, Princess Margaret Hospital	Department of Health Technology and Informatics, Faculty of Health and Social Science, The Hong Kong Polytechnic University	Kenneth Siu-Sing LEUNG, Timothy Ting-Leung NG, Alan Ka-Lun WU, Miranda Chong-Yee YAU, Hiu-Yin LAO, Ming-Pan CHOI, Kingsley King-Gee TAM, Lam-Kwong LEE, Barry Kin-Chung WONG, Alex Yat-Man HO, Kam-Tong YIP, Kwok-Cheung LUNG, Raymond Wai-To LIU, Eugene Yuk-Keung TSO, Wai-Shing LEUNG, Man-Chun CHAN, Yuk-Yung NG, Kit-Man SIN, Kitty Sau-Chun FUNG, Sandy Ka-Yee CHAU, Wing-Kin TO, Tak-Lun QUE, David Ho-Keung SHUM, Shea Ping YIP, Wing Cheong YAM, Gilman Kit-Hang SIU
EPI_ISL_419219	Department of Clinical Pathology, Pamela Youde Nethersole Eastern Hospital	Department of Health Technology and Informatics, Faculty of Health and Social Science, The Hong Kong Polytechnic University	Kenneth Siu-Sing LEUNG, Timothy Ting-Leung NG, Alan Ka-Lun WU, Miranda Chong-Yee YAU, Hiu-Yin LAO, Ming-Pan CHOI, Kingsley King-Gee TAM, Lam-Kwong LEE, Barry Kin-Chung WONG, Alex Yat-Man HO, Kam-Tong YIP, Kwok-Cheung LUNG, Raymond Wai-To LIU, Eugene Yuk-Keung TSO, Wai-Shing LEUNG, Man-Chun CHAN, Yuk-Yung NG, Kit-Man SIN, Kitty Sau-Chun FUNG, Sandy Ka-Yee CHAU, Wing-Kin TO, Tak-Lun QUE, David Ho-Keung SHUM, Shea Ping YIP, Wing Cheong YAM, Gilman Kit-Hang SIU
EPI_ISL_419221	Department of Pathology, United Christian Hospital	Department of Health Technology and Informatics, Faculty of Health and Social Science, The Hong Kong Polytechnic University	Kenneth Siu-Sing LEUNG, Timothy Ting-Leung NG, Alan Ka-Lun WU, Miranda Chong-Yee YAU, Hiu-Yin LAO, Ming-Pan CHOI, Kingsley King-Gee TAM, Lam-Kwong LEE, Barry Kin-Chung WONG, Alex Yat-Man HO, Kam-Tong YIP, Kwok-Cheung LUNG, Raymond Wai-To LIU, Eugene Yuk-Keung TSO, Wai-Shing LEUNG, Man-Chun CHAN, Yuk-Yung NG, Kit-Man SIN, Kitty Sau-Chun FUNG, Sandy Ka-Yee CHAU, Wing-Kin TO, Tak-Lun QUE, David Ho-Keung SHUM, Shea Ping YIP, Wing Cheong YAM, Gilman Kit-Hang SIU
EPI_ISL_419222	Department of Pathology, Princess Margaret Hospital	Department of Health Technology and Informatics, Faculty of Health and Social Science, The Hong Kong Polytechnic University	Kenneth Siu-Sing LEUNG, Timothy Ting-Leung NG, Alan Ka-Lun WU, Miranda Chong-Yee YAU, Hiu-Yin LAO, Ming-Pan CHOI, Kingsley King-Gee TAM, Lam-Kwong LEE, Barry Kin-Chung WONG, Alex Yat-Man HO, Kam-Tong YIP, Kwok-Cheung LUNG, Raymond Wai-To LIU, Eugene Yuk-Keung TSO, Wai-Shing LEUNG, Man-Chun CHAN, Yuk-Yung NG, Kit-Man SIN, Kitty Sau-Chun FUNG, Sandy Ka-Yee CHAU, Wing-Kin TO, Tak-Lun QUE, David Ho-Keung SHUM, Shea Ping YIP, Wing Cheong YAM, Gilman Kit-Hang SIU
EPI_ISL_419224, EPI_ISL_419225, EPI_ISL_419226, EPI_ISL_419227, EPI_ISL_419228, EPI_ISL_419229	Department of Clinical Pathology, Pamela Youde Nethersole Eastern Hospital	Department of Health Technology and Informatics, Faculty of Health and Social Science, The Hong Kong Polytechnic University	Kenneth Siu-Sing LEUNG, Timothy Ting-Leung NG, Alan Ka-Lun WU, Miranda Chong-Yee YAU, Hiu-Yin LAO, Ming-Pan CHOI, Kingsley King-Gee TAM, Lam-Kwong LEE, Barry Kin-Chung WONG, Alex Yat-Man HO, Kam-Tong YIP, Kwok-Cheung LUNG, Raymond Wai-To LIU, Eugene Yuk-Keung TSO, Wai-Shing LEUNG, Man-Chun CHAN, Yuk-Yung NG, Kit-Man SIN, Kitty Sau-Chun FUNG, Sandy Ka-Yee CHAU, Wing-Kin TO, Tak-Lun QUE, David Ho-Keung SHUM, Shea Ping YIP, Wing Cheong YAM, Gilman Kit-Hang SIU
EPI_ISL_419230	Hospital Universitario Virgen de las Nieves	Instituto de Salud Carlos III	Iglesias-Caballero, M.; Molinero Calamita, M.; González-Esguevillas, M.; Camarero, S.; Pozo, F.; Casas, I.; Jiménez, P.; Jiménez, M.; Zaballos, A.; Monzón, S.; Varona, S.; Juliá, M.; Cuesta, I.; Sanbonmatsu, S.
EPI_ISL_419231	Department of Clinical Pathology, Tuen Mun Hospital, 23 Tsing Chung Koon Road, Tuen Mun, N.T.	Department of Health Technology and Informatics, Faculty of Health and Social Science, The Hong Kong Polytechnic University	Kenneth Siu-Sing LEUNG, Timothy Ting-Leung NG, Alan Ka-Lun WU, Miranda Chong-Yee YAU, Hiu-Yin LAO, Ming-Pan CHOI, Kingsley King-Gee TAM, Lam-Kwong LEE, Barry Kin-Chung WONG, Alex Yat-Man HO, Kam-Tong YIP, Kwok-Cheung LUNG, Raymond Wai-To LIU, Eugene Yuk-Keung TSO, Wai-Shing LEUNG, Man-Chun CHAN, Yuk-Yung NG, Kit-Man SIN, Kitty Sau-Chun FUNG, Sandy Ka-Yee CHAU, Wing-Kin TO, Tak-Lun QUE, David Ho-Keung SHUM, Shea Ping YIP, Wing Cheong YAM, Gilman Kit-Hang SIU
EPI_ISL_419232	Department of Clinical Pathology, Pamela Youde Nethersole Eastern Hospital	Department of Health Technology and Informatics, Faculty of Health and Social Science, The Hong Kong Polytechnic University	Kenneth Siu-Sing LEUNG, Timothy Ting-Leung NG, Alan Ka-Lun WU, Miranda Chong-Yee YAU, Hiu-Yin LAO, Ming-Pan CHOI, Kingsley King-Gee TAM, Lam-Kwong LEE, Barry Kin-Chung WONG, Alex Yat-Man HO, Kam-Tong YIP, Kwok-Cheung LUNG, Raymond Wai-To LIU, Eugene Yuk-Keung TSO, Wai-Shing LEUNG, Man-Chun CHAN, Yuk-Yung NG, Kit-Man SIN, Kitty Sau-Chun FUNG, Sandy Ka-Yee CHAU, Wing-Kin TO, Tak-Lun QUE, David Ho-Keung SHUM, Shea Ping YIP, Wing Cheong YAM, Gilman Kit-Hang SIU
EPI_ISL_419233	Hospital Universitario de Canarias	Instituto de Salud Carlos III	Iglesias-Caballero, M.; Molinero Calamita, M.; González-Esguevillas, M.; Camarero, S.; Pozo, F.; Casas, I.; Jiménez, P.; Jiménez, M.; Zaballos, A.; Monzón, S.; Varona, S.; Juliá, M.; Cuesta, I.; Castro, B.
EPI_ISL_419234	Hospital San Pedro	Instituto de Salud Carlos III	Iglesias-Caballero, M.; Molinero Calamita, M.; González-Esguevillas, M.; Camarero, S.; Pozo, F.; Casas, I.; Jiménez, P.; Jiménez, M.; Zaballos, A.; Monzón, S.; Varona, S.; Juliá, M.; Cuesta, I.; Alonso, C.
EPI_ISL_419235, EPI_ISL_419236, EPI_ISL_419237	Fundacion Jimenez Diaz	Instituto de Salud Carlos III	Iglesias-Caballero, M.; Molinero Calamita, M.; González-Esguevillas, M.; Camarero, S.; Pozo, F.; Casas, I.; Jiménez, P.; Jiménez, M.; Zaballos, A.; Monzón, S.; Varona, S.; Juliá, M.; Cuesta, I.; Fernández, R.
EPI_ISL_419238	HOSPITAL DE CRUCES.	Instituto de Salud Carlos III	Iglesias-Caballero, M. Molinero Calamita, M. González-Esguevillas, M. Camarero, S. Pozo, F. Casas, I. Jiménez, P. Jiménez, M. Zaballos, A. Monzón, S. Varona, S. Juliá, M. Cuesta, I. Aranzamendi, M.
EPI_ISL_419240	HOSPITAL TXAGORRITXU	Instituto de Salud Carlos III	Iglesias-Caballero, M. Molinero Calamita, M. González-Esguevillas, M. Camarero, S. Pozo, F. Casas, I. Jiménez, P. Jiménez, M. Zaballos, A. Monzón, S. Varona, S. Juliá, M. Cuesta, I. Gómez, C
EPI_ISL_419245, EPI_ISL_419247, EPI_ISL_419250, EPI_ISL_419252	Department of Clinical Pathology, Pamela Youde Nethersole Eastern Hospital	Department of Health Technology and Informatics, Faculty of Health and Social Science, The Hong Kong Polytechnic University	Kenneth Siu-Sing LEUNG, Timothy Ting-Leung NG, Alan Ka-Lun WU, Miranda Chong-Yee YAU, Hiu-Yin LAO, Ming-Pan CHOI, Kingsley King-Gee TAM, Lam-Kwong LEE, Barry Kin-Chung WONG, Alex Yat-Man HO, Kam-Tong YIP, Kwok-Cheung LUNG, Raymond Wai-To LIU, Eugene Yuk-Keung TSO, Wai-Shing LEUNG, Man-Chun CHAN, Yuk-Yung NG, Kit-Man SIN, Kitty Sau-Chun FUNG, Sandy Ka-Yee CHAU, Wing-Kin TO, Tak-Lun QUE, David Ho-Keung SHUM, Shea Ping YIP, Wing Cheong YAM, Gilman Kit-Hang SIU
EPI_ISL_419296	Kochi Prefectural Institute of Public Health	Pathogen Genomics Center, National Institute of Infectious Diseases	Tsuyoshi Sekizuka, Akihiko Tokaji, Kentaro Itokawa, Rina Tanaka, Masanori Hashino, Hajime Kamiya, Motoi Suzuki, Makoto Kuroda
EPI_ISL_419297, EPI_ISL_419298	Chiba Prefectural Institute of Public Health	Pathogen Genomics Center, National Institute of Infectious Diseases	Tsuyoshi Sekizuka, Masakatsu Taira, Yushi Hachisu, Kentaro Itokawa, Rina Tanaka, Masanori Hashino, Hajime Kamiya, Motoi Suzuki, Makoto Kuroda
EPI_ISL_419299, EPI_ISL_419300	Ishikawa Prefectural Institute of Public Health and Environmental Science	Pathogen Genomics Center, National Institute of Infectious Diseases	Tsuyoshi Sekizuka, Sanae Kuramoto, Eri Nariai, Kentaro Itokawa, Rina Tanaka, Masanori Hashino, Hajime Kamiya, Motoi Suzuki, Makoto Kuroda
EPI_ISL_419301, EPI_ISL_419302,	Saitama Prefectural Institute of Public Health	Pathogen Genomics Center, National Institute of Infectious	Tsuyoshi Sekizuka, Michiyo Shinohara, Tsuyoshi Kishimoto, Kentaro Itokawa, Rina Tanaka, Masanori Hashino, Hajime Kamiya, Motoi Suzuki, Makoto Kuroda

EPI_ISL_419303, EPI_ISL_419304, EPI_ISL_419305, EPI_ISL_419306, EPI_ISL_419307, EPI_ISL_419308		Diseases	
EPI_ISL_419309, EPI_ISL_419310, EPI_ISL_419311	Chiba Prefectural Institute of Public Health	Pathogen Genomics Center, National Institute of Infectious Diseases	Tsuyoshi Sekizuka, Masakatsu Taira, Yushi Hachisu, Kentaro Itokawa, Rina Tanaka, Masanori Hashino, Hajime Kamiya, Motoi Suzuki, Makoto Kuroda
EPI_ISL_419662, EPI_ISL_419664	Center for Virology, Medical University of Vienna	Bergthaler laboratory, CeMM Research Center for Molecular Medicine of the Austrian Academy of Sciences	Alexandra Popa, Benedikt Agerer, Henrique Colaco, Lukas Endler, Jakob-Wendelin Genger, Alexander Lercher, Mark Smyth, Thomas Penz, Michael Schuster, Judith Aberle, Stephan Aberle, Elisabeth Puchhammer-Stöckl, Christoph Bock, Andreas Bergthaler
EPI_ISL_419707	HOSPITAL CLINIC	Instituto de Salud Carlos III	Iglesias-Caballero, M. Molinero Calamita, M. González-Esguevillas, M. Camarero S. Pozo F. Casas I. Jiménez, P. Jiménez, M. Zaballos, A. Monzón, S. Varona, S. Juliá, M. Cuesta, I. Marcos, M.A
EPI_ISL_419709	HOSPITAL TXAGORRITXU	Instituto de Salud Carlos III	Iglesias-Caballero, M. Molinero Calamita, M. González-Esguevillas, M. Camarero S. Pozo F. Casas I. Jiménez, P. Jiménez, M. Zaballos, A. Monzón, S. Varona, S. Juliá, M. Cuesta, I. Gómez, C.
EPI_ISL_420037	NIC Viral Respiratory Unit - Institut Pasteur of Algeria	National Reference Center for Viruses of Respiratory Infections, Institut Pasteur, Paris	Mélanie Albert, Marion Barbet, Sylvie Behillil, Méline Bizard, Angela Brisebarre, Flora Donati, Etienne Simon-Lorière, Vincent Enouf, Maud Vanpeene, Sylvie van der Werf, Fawzi Derrar
EPI_ISL_420039, EPI_ISL_420040	L'Air du Temps	National Reference Center for Viruses of Respiratory Infections, Institut Pasteur, Paris	Mélanie Albert, Marion Barbet, Sylvie Behillil, Méline Bizard, Angela Brisebarre, Flora Donati, Etienne Simon-Lorière, Vincent Enouf, Maud Vanpeene, Sylvie van der Werf
EPI_ISL_420041	CH Compiègne Laboratoire de Biologie	National Reference Center for Viruses of Respiratory Infections, Institut Pasteur, Paris	Mélanie Albert, Marion Barbet, Sylvie Behillil, Méline Bizard, Angela Brisebarre, Flora Donati, Etienne Simon-Lorière, Vincent Enouf, Maud Vanpeene, Sylvie van der Werf, Raulin Olivia
EPI_ISL_420042	Service de Biologie clinique	National Reference Center for Viruses of Respiratory Infections, Institut Pasteur, Paris	Mélanie Albert, Marion Barbet, Sylvie Behillil, Méline Bizard, Angela Brisebarre, Flora Donati, Etienne Simon-Lorière, Vincent Enouf, Maud Vanpeene, Sylvie van der Werf
EPI_ISL_420044	CH Jean de Navarre Laboratoire de Biologie	National Reference Center for Viruses of Respiratory Infections, Institut Pasteur, Paris	Mélanie Albert, Marion Barbet, Sylvie Behillil, Méline Bizard, Angela Brisebarre, Flora Donati, Etienne Simon-Lorière, Vincent Enouf, Maud Vanpeene, Sylvie van der Werf
EPI_ISL_420046, EPI_ISL_420047	Résidence Villa Caroline	National Reference Center for Viruses of Respiratory Infections, Institut Pasteur, Paris	Mélanie Albert, Marion Barbet, Sylvie Behillil, Méline Bizard, Angela Brisebarre, Flora Donati, Etienne Simon-Lorière, Vincent Enouf, Maud Vanpeene, Sylvie van der Werf
EPI_ISL_420048	Service de Biologie Médicale - BP 125	National Reference Center for Viruses of Respiratory Infections, Institut Pasteur, Paris	Mélanie Albert, Marion Barbet, Sylvie Behillil, Méline Bizard, Angela Brisebarre, Flora Donati, Etienne Simon-Lorière, Vincent Enouf, Maud Vanpeene, Sylvie van der Werf, Christine Lambert
EPI_ISL_420049, EPI_ISL_420050	CH Compiègne Laboratoire de Biologie	National Reference Center for Viruses of Respiratory Infections, Institut Pasteur, Paris	Mélanie Albert, Marion Barbet, Sylvie Behillil, Méline Bizard, Angela Brisebarre, Flora Donati, Etienne Simon-Lorière, Vincent Enouf, Maud Vanpeene, Sylvie van der Werf, Raulin Olivia
EPI_ISL_420051	Résidence Eleusis	National Reference Center for Viruses of Respiratory Infections, Institut Pasteur, Paris	Mélanie Albert, Marion Barbet, Sylvie Behillil, Méline Bizard, Angela Brisebarre, Flora Donati, Etienne Simon-Lorière, Vincent Enouf, Maud Vanpeene, Sylvie van der Werf
EPI_ISL_420052	Résidence les Marines	National Reference Center for Viruses of Respiratory Infections, Institut Pasteur, Paris	Mélanie Albert, Marion Barbet, Sylvie Behillil, Méline Bizard, Angela Brisebarre, Flora Donati, Etienne Simon-Lorière, Vincent Enouf, Maud Vanpeene, Sylvie van der Werf
EPI_ISL_420053	CH Jean de Navarre Laboratoire de Biologie	National Reference Center for Viruses of Respiratory Infections, Institut Pasteur, Paris	Mélanie Albert, Marion Barbet, Sylvie Behillil, Méline Bizard, Angela Brisebarre, Flora Donati, Etienne Simon-Lorière, Vincent Enouf, Maud Vanpeene, Sylvie van der Werf
EPI_ISL_420056, EPI_ISL_420057	CH Compiègne Laboratoire de Biologie	National Reference Center for Viruses of Respiratory Infections, Institut Pasteur, Paris	Mélanie Albert, Marion Barbet, Sylvie Behillil, Méline Bizard, Angela Brisebarre, Flora Donati, Etienne Simon-Lorière, Vincent Enouf, Maud Vanpeene, Sylvie van der Werf, Raulin Olivia
EPI_ISL_420058, EPI_ISL_420059, EPI_ISL_420060	Service de Biologie Médicale - BP 125	National Reference Center for Viruses of Respiratory Infections, Institut Pasteur, Paris	Mélanie Albert, Marion Barbet, Sylvie Behillil, Méline Bizard, Angela Brisebarre, Flora Donati, Etienne Simon-Lorière, Vincent Enouf, Maud Vanpeene, Sylvie van der Werf, Christine Lambert
EPI_ISL_420063	Labo BM - Site de Juvisy - Hopital Général	National Reference Center for Viruses of Respiratory Infections, Institut Pasteur, Paris	Mélanie Albert, Marion Barbet, Sylvie Behillil, Méline Bizard, Angela Brisebarre, Flora Donati, Etienne Simon-Lorière, Vincent Enouf, Maud Vanpeene, Sylvie van der Werf
EPI_ISL_420064	Service de Biologie Médicale - BP 125	National Reference Center for Viruses of Respiratory Infections, Institut Pasteur, Paris	Mélanie Albert, Marion Barbet, Sylvie Behillil, Méline Bizard, Angela Brisebarre, Flora Donati, Etienne Simon-Lorière, Vincent Enouf, Maud Vanpeene, Sylvie van der Werf, Christine Lambert
EPI_ISL_420069, EPI_ISL_420070	Institut Pasteur Dakar	Institut Pasteur de Dakar	Ndongo Dia, Moussa Moise Diagne, Mamadou Diop, Ousmane Faye, Amadou Alpha Sall
EPI_ISL_420072, EPI_ISL_420073, EPI_ISL_420074	Institut Pasteur Dakar	Institut Pasteur de Dakar	Ndongo Dia, Moussa Moise Diagne, Mamadou Diop, Ousmane Faye , Amadou Alpha Sall
EPI_ISL_420076	Institut Pasteur Dakar	Institut Pasteur de Dakar	Ndongo Dia, Moussa Moise Diagne, Mamadou Diop, Ousmane Faye , Ndongo Dia
EPI_ISL_420077, EPI_ISL_420078	Institut Pasteur Dakar	Institut Pasteur de Dakar	Ndongo Dia, Moussa Moise Diagne, Mamadou Diop, Ousmane Faye , Amadou Alpha Sall
EPI_ISL_420080	WHO National Influenza Centre Russian Federation	WHO National Influenza Centre Russian Federation	Andrey Komissarov, Artem Fadeev, Anna Ivanova, Daria Danilenko
EPI_ISL_420081	WHO National Influenza Centre Russian Federation	WHO National Influenza Centre Russian Federation	Andrey Komissarov, Artem Fadeev, Mariia Sergeeva, Anna Ivanova, Daria Danilenko
EPI_ISL_421182, EPI_ISL_421183, EPI_ISL_421184, EPI_ISL_421185, EPI_ISL_421186, EPI_ISL_421187, EPI_ISL_421188, EPI_ISL_421189, EPI_ISL_421190, EPI_ISL_421191, EPI_ISL_421192, EPI_ISL_421193, EPI_ISL_421194, EPI_ISL_421195, EPI_ISL_421196			
see above	Department of Clinical Microbiology	GIGA Medical Genomics	Keith Durkin, Maria Artesi, Sébastien Bontems, Raphaël Boreux, Cécile Meex, Pierrette Melin, Marie-Pierre Hayette, Vincent Bours.
EPI_ISL_421221, EPI_ISL_421222, EPI_ISL_421224, EPI_ISL_421225, EPI_ISL_421226, EPI_ISL_421227, EPI_ISL_421228, EPI_ISL_421229, EPI_ISL_421230, EPI_ISL_421231, EPI_ISL_421232, EPI_ISL_421233, EPI_ISL_421234, EPI_ISL_421235, EPI_ISL_421236			
see above	Hangzhou Center for Diseases Control and Prevention	Hangzhou Center for Diseases Control and Prevention	Jun Li, Haoqiu Wang, Lingfeng Mao, Hua Yu, Xinfen Yu, Zhou Sun, Xin Qian, Shuchang Chen, Junfang Chen, Xuchu Wang
EPI_ISL_421275	Russian State Collection of Viruses	Pathogenic Microorganisms Variability Laboratory	Alexey Shchetinin, Maria Nikiforova, Nadezhda Kuznetsova, Ekaterina Aksenova, Marina Kunda, Natalia Ryzhova, Olga Voronina, Inna Dolzhikova, Daria Grousova, Andrey Botikov, Denis Logunov, Alexander Gintsburg, Vladimir Gushchin
EPI_ISL_421500	CH Compiègne Laboratoire de Biologie	National Reference Center for Viruses of Respiratory Infections, Institut Pasteur, Paris	Mélanie Albert, Marion Barbet, Sylvie Behillil, Méline Bizard, Angela Brisebarre, Flora Donati, Etienne Simon-Lorière, Vincent Enouf, Maud Vanpeene, Sylvie van der Werf, Raulin Olivia
EPI_ISL_421501	Service de Biologie Médicale - BP 125	National Reference Center for Viruses of Respiratory Infections, Institut Pasteur, Paris	Mélanie Albert, Marion Barbet, Sylvie Behillil, Méline Bizard, Angela Brisebarre, Flora Donati, Etienne Simon-Lorière, Vincent Enouf, Maud Vanpeene, Sylvie van der Werf, Christine Lambert
EPI_ISL_421502, EPI_ISL_421503	Parc des Dames	National Reference Center for Viruses of Respiratory Infections, Institut Pasteur, Paris	Mélanie Albert, Marion Barbet, Sylvie Behillil, Méline Bizard, Angela Brisebarre, Flora Donati, Etienne Simon-Lorière, Vincent Enouf, Maud Vanpeene, Sylvie van der Werf
EPI_ISL_421504, EPI_ISL_421505, EPI_ISL_421506	Service de Biologie Médicale - BP 125	National Reference Center for Viruses of Respiratory Infections, Institut Pasteur, Paris	Mélanie Albert, Marion Barbet, Sylvie Behillil, Méline Bizard, Angela Brisebarre, Flora Donati, Etienne Simon-Lorière, Vincent Enouf, Maud Vanpeene, Sylvie van der Werf, Christine Lambert
EPI_ISL_421507, EPI_ISL_421508	Le Château de Seine-Port	National Reference Center for Viruses of Respiratory Infections, Institut Pasteur, Paris	Mélanie Albert, Marion Barbet, Sylvie Behillil, Méline Bizard, Angela Brisebarre, Flora Donati, Etienne Simon-Lorière, Vincent Enouf, Maud Vanpeene, Sylvie van der Werf
EPI_ISL_421509, EPI_ISL_421510, EPI_ISL_421511	CH Compiègne Laboratoire de Biologie	National Reference Center for Viruses of Respiratory Infections, Institut Pasteur, Paris	Mélanie Albert, Marion Barbet, Sylvie Behillil, Méline Bizard, Angela Brisebarre, Flora Donati, Etienne Simon-Lorière, Vincent Enouf, Maud Vanpeene, Sylvie van der Werf, Raulin Olivia
EPI_ISL_421512	Service de Biologie Médicale - BP 125	National Reference Center for Viruses of Respiratory Infections, Institut Pasteur, Paris	Mélanie Albert, Marion Barbet, Sylvie Behillil, Méline Bizard, Angela Brisebarre, Flora Donati, Etienne Simon-Lorière, Vincent Enouf, Maud Vanpeene, Sylvie van der Werf, Christine Lambert
EPI_ISL_421513	Service de Biologie clinique	National Reference Center for Viruses of Respiratory	Mélanie Albert, Marion Barbet, Sylvie Behillil, Méline Bizard, Angela Brisebarre, Flora Donati, Etienne Simon-Lorière, Vincent Enouf, Maud Vanpeene, Sylvie van

		Infections, Institut Pasteur, Paris	der Werf, Christine Lambert
EPI_ISL_421574	Molecular Diagnostic Services	KRISP, KZN Research Innovation and Sequencing Platform	Giandhari J, Pillay S, Ngcapu S, Samsunder N, Lessells R, Chimukangara B, Deforche K, Tegally H, Wilkinson E, de Oliveira T
EPI_ISL_421652	Dasman Diabetes Institute	Dasman Diabetes Institute	Fahd Al-Mulla, Rasheeba Iqbal, Sumi John, Ebaa Al-Ozairi, Qais Al-Duwairi
EPI_ISL_422407, EPI_ISL_422408, EPI_ISL_422409, EPI_ISL_422410, EPI_ISL_422411, EPI_ISL_422412, EPI_ISL_422413, EPI_ISL_422414, EPI_ISL_422415, EPI_ISL_422416, EPI_ISL_422417, EPI_ISL_422418, EPI_ISL_422419, EPI_ISL_422420, EPI_ISL_422421, EPI_ISL_422422			
see above	Department of Laboratory Medicine, National Taiwan University Hospital	Microbial Genomics Core Lab, National Taiwan University Centers of Genomic and Precision Medicine	Shiou-Hwei Yeh, You-Yu Lin, Ya-Yun Lai, Chiao-Ling Li, Shan-Chwen Chang, Pei-Jer Chen, Sui-Yuan Chang
EPI_ISL_422424	Jaber Al Ahmad Al Sabah Hospital	Dasman diabetes Institute	Fahd Al-Mulla, Rasheeba Iqbal, Sumi John, Ebaa Al-Ozairi, Qais Al-Duwairi
EPI_ISL_422427	JABER AL AHMAD AL SABAH HOSPITAL - KUWAIT CITY	Dasman Diabetes Institute	Fahd Al-Mulla, Rasheeba Iqbal, Sumi John, Ebaa Al-Ozairi, Qais Al-Duwairi
EPI_ISL_422636	The National Institute of Public Health Center for Epidemiology and Microbiology	State Veterinary Institute Prague	Alexander Nagy, Helena Jirincova, Klara Labska, Ludmila Novakova, Olga Storkanova, Dusan Trnka, Jaromira Vecerova
EPI_ISL_424366	Vaccine Research, Development and Application Center, Erciyes University	Gen Era Diagnostics Inc.	Shaikh Terkis Islam Pavel, Hazel Yetiskin, Gunsu Aydin, Can Holyavkin, Muhammet Ali Uygut, Zehra B Dursun, Ihami Celik, Alper Iseri, Aykut Ozdarendeli
EPI_ISL_424629, EPI_ISL_424630, EPI_ISL_424631, EPI_ISL_424633, EPI_ISL_424636, EPI_ISL_424637, EPI_ISL_424640, EPI_ISL_424641, EPI_ISL_424642, EPI_ISL_424643, EPI_ISL_424644, EPI_ISL_424647, EPI_ISL_424649, EPI_ISL_424650, EPI_ISL_424652, EPI_ISL_424653, EPI_ISL_424654, EPI_ISL_424655, EPI_ISL_424657, EPI_ISL_424658, EPI_ISL_424660, EPI_ISL_424661, EPI_ISL_424664			
see above	Department of Clinical Microbiology	GIGA Medical Genomics	Keith Durkin, Maria Artesi, Sébastien Bontems, Raphaël Boreux, Cécile Meex, Pierrette Melin, Marie-Pierre Hayette, Vincent Bours.
EPI_ISL_426364	Instituto Nacional de Ciencias Medicas y Nutricion Salvador Zubiran	Instituto Nacional de Ciencias Medicas y Nutricion	Guillermo M. Ruiz-Palacios, Pilar Ramos Cervantes, Violeta Ibarra Gonzalez, Fernando Ledesma Barrientos, Luis Alberto García Andrade, Alfredo Ponce de León Garduño, Irma López Martínez, Lucia Hernández Rivas, Gisela Barrera Badillo, Edgar Mendieta Condado, Fabiola Garcés Ayala, Adnan Araiza Rodríguez, José Ernesto Ramírez González, Celia Boukadida, Santiago Avila Ríos, Mario Mújica Sánchez, José Arturo Martínez Orozco, Eduardo Becerril Vargas, Joel Armando Vázquez Pérez, Victor Hugo Borja Aburto, Concepción Grajales Muñiz, Cesar Raúl González Bonilla, Carolina González Torres, Francisco Javier Gaytán Cervantes, José Esteban Muñoz Medina, Blanca Taboada, Alejandro Sánchez, Pavel Isa, Ricardo Grande, Gloria Vázquez, Francisco Pulido, Carlos F. Arias
EPI_ISL_426414	Sir M P Shah Government Medical College	Gujarat Biotechnology Research Centre	Ramesh Pandit, Tejas Shah, Ankit Hinsu, Pritesh Sabara, Apurvasinh Puvar, Janvi Raval, Monika Gandhi, Pinal Trivedi, Maharshi Pandya, Amit Kanani, Akanksha Verma, Nitin Savaliya, Raghawendra Kumar, Dinesh Kumar, Zubair Saiyed, Dipa Kinariwala, Disha Patel, Binita Aring, Geeta Vaghela, Sonia Barve, Bhavesh Modi, Kairavi Joshi, Nidhi Sood, Pranay Shah, R D Dixit, Snehal Bagatharia, Madhvi Joshi, Chaitanya Joshi
EPI_ISL_426415	Sir M P Shah Government Medical College, Jamnagar	Gujarat Biotechnology Research Centre, Gandhinagar	Ramesh Pandit, Tejas Shah, Ankit Hinsu, Pritesh Sabara, Apurvasinh Puvar, Janvi Raval, Monika Gandhi, Pinal Trivedi, Maharshi Pandya, Amit Kanani, Akanksha Verma, Nitin Savaliya, Raghvendra Kumar, Dinesh Kumar, Zuber Saiyed, Dipa Kinariwala, Disha Patel, Binita Aring, Geeta Vaghela, Sonia Barve, Bhavesh Modi, Kairavi Joshi, Nidhi Sood, Pranay Shah, R D Dixit, Snehal Bagatharia, Madhvi Joshi, Chaitanya Joshi
EPI_ISL_426580	Instituto Sabin	Laboratory of Virology	Fernando L Melo,Gustavo Barra, Ticiane H Santa-Rita, Pedro G Mesquita, Ikaro A Andrade, Tatsuya Nagata, Bergmann M Ribeiro
EPI_ISL_427307, EPI_ISL_427308, EPI_ISL_427309, EPI_ISL_427310, EPI_ISL_427311, EPI_ISL_427312, EPI_ISL_427314, EPI_ISL_427315, EPI_ISL_427319, EPI_ISL_427320, EPI_ISL_427321, EPI_ISL_427322, EPI_ISL_427323, EPI_ISL_427337, EPI_ISL_427338, EPI_ISL_427339			
see above	WHO National Influenza Centre Russian Federation	WHO National Influenza Centre Russian Federation	Andrey Komissarov, Artem Fadeev, Mariia Sergeeva, Anna Ivanova, Daria Danilenko
EPI_ISL_427349, EPI_ISL_427350, EPI_ISL_427351, EPI_ISL_427352, EPI_ISL_427354, EPI_ISL_427357, EPI_ISL_427358, EPI_ISL_427359, EPI_ISL_427364, EPI_ISL_427366, EPI_ISL_427370, EPI_ISL_427371, EPI_ISL_427372, EPI_ISL_427379, EPI_ISL_427385, EPI_ISL_427388, EPI_ISL_427390			
see above	Department of Clinical Microbiology	GIGA Medical Genomics	Keith Durkin, Maria Artesi, Sébastien Bontems, Raphaël Boreux, Cécile Meex, Pierrette Melin, Marie-Pierre Hayette, Vincent Bours.
EPI_ISL_427391	Genomic Laboratory (GLAB) (Conjoint lab of Health Directorate of Istanbul and Istanbul Technical University)	Genomic Laboratory (GLAB), Istanbul Technical University	Ilker Karacan, Tugba Kizilboga Akgun, Bugra Agaoglu, Gizem Alkurt, Jale Yildiz, Betsi Köse, Elifnaz Çelik, Mehtap Aydn, Levent Doganay, Gizem Dinler Doganay
EPI_ISL_428207, EPI_ISL_428208	National Institute of Health Research and Development	National Institute of Health Research and Development	Setiawaty,V;Subangkit;Puspa,KD;Ikawati,HD;Nugraha,AA;Hariastuti,NI;Ramadhany,R;Susilarini,NK;Pratiwi,E;Agustiningsih;Kurniawati,J;Pawestri,HA;Siswanto
EPI_ISL_428209	Laboratory of Molecular Biology, Diagnostyka sp. z o.o.	Laboratory of Recombinant Vaccines	Lukasz Rabalski, Anna Piotrowska-Mietelska, Boguslaw Szewczyk, Krystyna Bienkowska-Szewczyk
EPI_ISL_428229	TSGH-CP molecular lab	TSGH-CP molecular lab	Cherng-Lih Perng, Ming-Jr Jian, Chih-Kai Chang, Jung-Chung Lin, Kuo-Ming Yeh, Chien-Wen Chen, Sheng-Kang Chiu, Hsing-Yi Chung, Shih-Hung Tsai, Kuo-Sheng Hung, Tien-Yao Chang, Feng-Yee Chang, Hung-Sheng Shang
EPI_ISL_428346	Genomic Laboratory (GLAB) (Conjoint lab of Health Directorate of Istanbul and Istanbul Technical University)	Genomic Laboratory (GLAB), Istanbul Technical University	Ilker Karacan, Tugba Kizilboga Akgun, Bugra Agaoglu, Gizem Alkurt, Jale Yildiz, Betsi Köse, Elifnaz Çelik, Arzu Irvem, Yasemin Kendir Demirkol, Ozlem Akgun Dogan, Mehtap Aydn, Levent Doganay, Gizem Dinler Doganay
EPI_ISL_428347, EPI_ISL_428349	Service de Biologie Médicale - BP 125	National Reference Center for Viruses of Respiratory Infections, Institut Pasteur, Paris	Mélanie Albert, Marion Barbet, Sylvie Behillil, Méline Bizard, Angela Brisebarre, Flora Donati, Etienne Simon-Lorière, Vincent Enouf, Maud Vanpeene, Sylvie van der Werf
EPI_ISL_428350	CH Jean de Navarre Laboratoire de Biologie	National Reference Center for Viruses of Respiratory Infections, Institut Pasteur, Paris	Mélanie Albert, Marion Barbet, Sylvie Behillil, Méline Bizard, Angela Brisebarre, Flora Donati, Etienne Simon-Lorière, Vincent Enouf, Maud Vanpeene, Sylvie van der Werf
EPI_ISL_428351, EPI_ISL_428352	GH Nord Essonne Service de Biologie clinique	National Reference Center for Viruses of Respiratory Infections, Institut Pasteur, Paris	Mélanie Albert, Marion Barbet, Sylvie Behillil, Méline Bizard, Angela Brisebarre, Flora Donati, Etienne Simon-Lorière, Vincent Enouf, Maud Vanpeene, Sylvie van der Werf
EPI_ISL_428353	CH Compiègne Laboratoire de Biologie	National Reference Center for Viruses of Respiratory Infections, Institut Pasteur, Paris	Mélanie Albert, Marion Barbet, Sylvie Behillil, Méline Bizard, Angela Brisebarre, Flora Donati, Etienne Simon-Lorière, Vincent Enouf, Maud Vanpeene, Sylvie van der Werf
EPI_ISL_428354	LABM GH nord Essonne de Longjumeau - BP 125	National Reference Center for Viruses of Respiratory Infections, Institut Pasteur, Paris	Mélanie Albert, Marion Barbet, Sylvie Behillil, Méline Bizard, Angela Brisebarre, Flora Donati, Etienne Simon-Lorière, Vincent Enouf, Maud Vanpeene, Sylvie van der Werf
EPI_ISL_428355, EPI_ISL_428356, EPI_ISL_428357	Institut Médico légal- Hop R. Poincaré	National Reference Center for Viruses of Respiratory Infections, Institut Pasteur, Paris	Mélanie Albert, Marion Barbet, Sylvie Behillil, Méline Bizard, Angela Brisebarre, Flora Donati, Etienne Simon-Lorière, Vincent Enouf, Maud Vanpeene, Sylvie van der Werf
EPI_ISL_428358	CH Jeanne de Navarre Laboratoire de Biologie	National Reference Center for Viruses of Respiratory Infections, Institut Pasteur, Paris	Mélanie Albert, Marion Barbet, Sylvie Behillil, Méline Bizard, Angela Brisebarre, Flora Donati, Etienne Simon-Lorière, Vincent Enouf, Maud Vanpeene, Sylvie van der Werf
EPI_ISL_428359, EPI_ISL_428360	CH Compiègne Laboratoire de Biologie	National Reference Center for Viruses of Respiratory Infections, Institut Pasteur, Paris	Mélanie Albert, Marion Barbet, Sylvie Behillil, Méline Bizard, Angela Brisebarre, Flora Donati, Etienne Simon-Lorière, Vincent Enouf, Maud Vanpeene, Sylvie van der Werf
EPI_ISL_428361	LABM GH nord Essonne de Longjumeau - BP 125	National Reference Center for Viruses of Respiratory Infections, Institut Pasteur, Paris	Mélanie Albert, Marion Barbet, Sylvie Behillil, Méline Bizard, Angela Brisebarre, Flora Donati, Etienne Simon-Lorière, Vincent Enouf, Maud Vanpeene, Sylvie van der Werf
EPI_ISL_428363	GH Nord Essonne Service de Biologie clinique	National Reference Center for Viruses of Respiratory Infections, Institut Pasteur, Paris	Mélanie Albert, Marion Barbet, Sylvie Behillil, Méline Bizard, Angela Brisebarre, Flora Donati, Etienne Simon-Lorière, Vincent Enouf, Maud Vanpeene, Sylvie van der Werf
EPI_ISL_428365	LABM GH nord Essonne de Longjumeau - BP 125	National Reference Center for Viruses of Respiratory Infections, Institut Pasteur, Paris	Mélanie Albert, Marion Barbet, Sylvie Behillil, Méline Bizard, Angela Brisebarre, Flora Donati, Etienne Simon-Lorière, Vincent Enouf, Maud Vanpeene, Sylvie van der Werf
EPI_ISL_428366	CH Jeanne de Navarre Laboratoire de Biologie	National Reference Center for Viruses of Respiratory Infections, Institut Pasteur, Paris	Mélanie Albert, Marion Barbet, Sylvie Behillil, Méline Bizard, Angela Brisebarre, Flora Donati, Etienne Simon-Lorière, Vincent Enouf, Maud Vanpeene, Sylvie van der Werf
EPI_ISL_428368	Genomic Laboratory (GLAB) (Conjoint lab of Health Directorate of Istanbul and Istanbul Technical University)	Genomic Laboratory (GLAB), Istanbul Technical University	Ilker Karacan, Tugba Kizilboga Akgun, Bugra Agaoglu, Gizem Alkurt, Jale Yildiz, Betsi Köse, Elifnaz Çelik, Arzu Irvem, Yasemin Kendir Demirkol, Ozlem Akgun Dogan, Mehtap Aydn, Levent Doganay, Gizem Dinler Doganay
EPI_ISL_428670	Centre for Dengue Research	Centre for Dengue Research	Chandima Jeewandara, Dinuka Ariyaratne, Laksiri Gomes, Deshni Jayathilaka, Ananda Wijewickrama, Eranga Narangoda, Damayanthi Idampitiya, Neelika Malaige
EPI_ISL_428671	Centre for Dengue Research	Centre for Dengue Research	Chandima Jeewandara, Dinuka Ariyatane, Laksiri Gomes, Deshni Jayathilaka, Diyanath Ranasinghe, Ananda Wijewickrama, Eranga Narangoda, Damayanthi

			Tdampitiya, Neelika Malavige
EPI_ISL_428672, EPI_ISL_428673	Centre for Dengue Research	Centre for Dengue Research	Chandima Jeewandara, Dinuka Ariyaratne, Laksiri Gomes, Deshni Jayathilaka, Diyanath Ranasinghe, Ananda Wijewickrama, Eranga Narangoda, Damayanthi Idampitiya, Neelika Malavige
EPI_ISL_428851, EPI_ISL_428852	FSBSI "Chumakov Federal Scientific Center for Research and Development of Immune-and-Biological Products of Russian Academy of Sciences"	FSBSI "Chumakov Federal Scientific Center for Research and Development of Immune-and-Biological Products of Russian Academy of Sciences" & NRC "Kurchatov institute"	Liubov Kozlovskaya, Anastasia Piniaeva, Georgy Ignatyev, Anna Shishova, Aydar Ishmukhametov, Mikhail Rychev, Egor Prokhorchuk, Denis Protsenko, Anastasia Berestovskaya
EPI_ISL_428855	MRCG at LSHTM Geomics lab	MRCG at LSHTM Genomics lab	Sesay et al
EPI_ISL_428856	MRCG at LSHTM Genomics Lab	MRCG at LSHTM Genomics lab	Sesay et al
EPI_ISL_429256	Health Sciences Technology Park, Avicena, 8, 18016 Granada. Spain	Sequencing and Bioinformatics Service FISABIO-Public Health	Joaquín Mendoza, Almudena Rojas, Pablo Mendoza
EPI_ISL_429852, EPI_ISL_429853, EPI_ISL_429854	Centers for Disease Control and Prevention of Lishui	Department of InspectionCenters for Disease Control and Prevention of Lishui	Wang Xiaoguang,Ji Qiaoying,Ji Jiansong,Ye Bifeng,Ye Ling
EPI_ISL_429968	Centre Hospitalier Compiègne Laboratoire de Biologie	National Reference Center for Viruses of Respiratory Infections, Institut Pasteur, Paris	Mélanie Albert, Marion Barbet, Sylvie Behillil, Méline Bizard, Angela Brisebarre, Flora Donati, Fabiana Gambaro, Etienne Simon-Lorière, Vincent Enouf, Maud Vanpeene, Sylvie van der Werf, Raulin Olivia
EPI_ISL_430067, EPI_ISL_430068, EPI_ISL_430069, EPI_ISL_430070, EPI_ISL_430071, EPI_ISL_430072, EPI_ISL_430073, EPI_ISL_430074, EPI_ISL_430075, EPI_ISL_430076, EPI_ISL_430077, EPI_ISL_430078, EPI_ISL_430079, EPI_ISL_430080, EPI_ISL_430081, EPI_ISL_430082, EPI_ISL_430083, EPI_ISL_430084, EPI_ISL_430085, EPI_ISL_430086, EPI_ISL_430087, EPI_ISL_430088, EPI_ISL_430089, EPI_ISL_430090, EPI_ISL_430100, EPI_ISL_430101, EPI_ISL_430102, EPI_ISL_430103, EPI_ISL_430104, EPI_ISL_430105, EPI_ISL_430106, EPI_ISL_430107, EPI_ISL_430108, EPI_ISL_430109, EPI_ISL_430110, EPI_ISL_430111, EPI_ISL_430112			
see above	WHO National Influenza Centre Russian Federation	WHO National Influenza Centre Russian Federation	Andrey Komissarov, Artem Fadeev, Mariia Sergeeva, Anna Ivanova, Daria Danilenko
EPI_ISL_430297	National Institute for Communicable Diseases of the National Health Laboratory Service	National Institute for Communicable Diseases of the National Health Laboratory Service	Allam M, Kwenda S, van Heusden P, Khumalo Z, Mohale T, Subramoney K, von Gottberg, A, Ismail A, Bhiman JN
EPI_ISL_430439	Institute for Medical Research, Infectious Disease Research Centre, National Institutes of Health, Ministry of Health Malaysia	Institute for Medical Research Infectious Disease Research Centre, National Institutes of Health, Ministry of Health Malaysia	Suppiah.J, Mohd-Zawawi.Z, Kalyanasundram.J, Azizan.M-A, Mat-Sharani.S, Hisham.H-A, Tan.L-P, Abdul-Wahid.M-Z, Mohd-Zain.R, Ahmad.N, Thayan.R
EPI_ISL_430440	Institute for Medical Research, Infectious Disease Research Centre, National Institutes of Health, Ministry of Health Malaysia	Institute for Medical Research, Infectious Disease Research Centre, National Institutes of Health, Ministry of Health Malaysia	Suppiah.J, Mohd-Zawawi.Z, Kalyanasundram.J, Azizan.M-A, Mat-Sharani.S, Hisham.H-A, Tan.L-P, Abdul-Wahid.M-Z, Tengku-Abd-Rashid.T-R, Mohd-Zain.R, Ahmad.N, Thayan.R
EPI_ISL_430441, EPI_ISL_430442, EPI_ISL_430443, EPI_ISL_430444	Institute for Medical Research, Infectious Disease Research Centre, National Institutes of Health, Ministry of Health Malaysia	Institute for Medical Research, Infectious Disease Research Centre, National Institutes of Health, Ministry of Health Malaysia	Suppiah.J, Mohd-Zawawi.Z, Kalyanasundram.J, Azizan.M-A, Mat-Sharani.S, Hisham.H-A, Tan.L-P, Abdul-Wahid.M-Z, Tengku-Rogayah.TAR, Mohd-Zain.R, Ahmad.N, Thayan.R
EPI_ISL_430469	Hellenic Pasteur Institute, Public Health Laboratories	Hellenic Pasteur Institute, Public Health Laboratories, Unit of Bioinformatics and Applied Genomics	Vasiliki Pogka, Timokratis Karamitros, Athanasios Kossyvakis, Antonios Kalliaropoulos, Horefti Elina, Evangelidou Maria, Androniki Voulgari-Kokota, Aspasia Kontou, Andreas Mentis
EPI_ISL_430722, EPI_ISL_430724, EPI_ISL_430725, EPI_ISL_430728, EPI_ISL_430729, EPI_ISL_430730, EPI_ISL_430731, EPI_ISL_430733, EPI_ISL_430734, EPI_ISL_430735, EPI_ISL_430736, EPI_ISL_430737, EPI_ISL_430738, EPI_ISL_430740, EPI_ISL_430741, EPI_ISL_430742			
see above	Chinese PLA Institute for Disease Control and Prevention	Chinese PLA Institute for Disease Control and Prevention	Peng LiJinhui Li, Lizhong Li
EPI_ISL_430819	Center of Scientific Excellence for Influenza Viruses,National Research Centre (NRC), Egypt.	Center of Scientific Excellence for Influenza Viruses,National Research Centre (NRC), Egypt.	Mohamed Ahmed Ali, Ahmed Kandeil, Ahmed Mostafa, Rabeh El-Shesheny, Mahmoud Shehata, Wael Roshdy, Shymaa Showky Ahmed , Amal Naguib, Nancy M. El Guindy, Mokhtar Gomaa, Ahmed El-Taweel, Ahmed E Kayed, Yassmin Moatasim, Omnia Kutkat, Sara Mahmoud, Mina Kamel, Abo Shama, M Noura, Mohamed El Sayes
EPI_ISL_430820	Center of Scientific Excellence for Influenza Viruses, National Research Centre (NRC), Egypt.	Center of Scientific Excellence for Influenza Viruses, National Research Centre (NRC), Egypt.	Mohamed Ahmed Ali, Ahmed Kandeil, Ahmed Mostafa, Rabeh El-Shesheny, Mahmoud Shehata, Wael Roshdy, Shymaa Showky Ahmed , Amal Naguib, Mokhtar Gomaa, Ahmed El-Taweel, Ahmed E Kayed, Yassmin Moatasim, Omnia Kutkat, Sara Mahmoud, Mina Kamel, Abo Shama, M Noura, Mohamed El Sayes, Nancy M. El Guindy
EPI_ISL_431101	Department of Microbiology,Gandhi Medical College and Hospital	Virus Research Laboratory, Department of Zoology, Osmania University,Hyderabad,India	Muttineni Radhakrishna, Nagamani K, Thrilok Chander B, Raja Rao M, Kalyani Putty, Ravikumar P, Sunitha P, Pankaj Singh D, Anand Kumar K, Amit A. Upadhyay Steven E. Bosinger, Rama Amara
EPI_ISL_431102	Department of MicroBiology,Gandhi Medical College and Hospital,Secendrabad,Hyderabad,India	Department of Microbiology, Gandhi Medical College and Hospital, Secendrabad, Hyderabad	Nagamani K, Muttineni Radhakrishna, Thrilok Chander B, Raja Rao M, Kalyani Putty, Ravikumar P, Sunitha P, Pankaj Singh D, Anand Kumar K, Amit A. Upadhyay, Steven E. Bosinger, Rama Amara
EPI_ISL_431103	Department of Microbiology, Gandhi Medical College and Hospital, Secendrabad, Hyderabad, India	Department of Microbiology, Gandhi Medical College and Hospital, Secendrabad, Hyderabad, India	Nagamani K, Muttineni Radhakrishna, Thrilok Chander B, Raja Rao M, Kalyani Putty, Ravikumar P, Sunitha P, Pankaj Singh D, Anand Kumar K, Amit A. Upadhyay, Steven E. Bosinger, Rama Amara
EPI_ISL_431117	Department of Microbiology, Gandhi Medical College and Hospital, Secendrabad, Hyderabad, India	Department of Microbiology, Gandhi Medical College and Hospital, Secendrabad, Hyderabad, India	Thrilok Chander B, Muttineni Radhakrishna, Nagamani K, Raja Rao M, Kalyani Putty, Ravikumar P, Sunitha P, Pankaj Singh D, Anand Kumar K, Amit A. Upadhyay, Steven E.Bosinger, Rama Amara
EPI_ISL_431118, EPI_ISL_431180, EPI_ISL_431240, EPI_ISL_431782, EPI_ISL_431783, EPI_ISL_431784, EPI_ISL_431785	Fujian Center for Disease Control and Prevention	Fujian Center for Disease Control and Prevention	Lin Qi, Huang Zhimiao, Zhang Yanhua, Weng Yuwei
EPI_ISL_434366, EPI_ISL_434369, EPI_ISL_434371	Hospital AZ Rivierenland	Institute of Tropical Medicine	Philippe Selhorst, Colin Anthony
EPI_ISL_434372, EPI_ISL_434373, EPI_ISL_434375, EPI_ISL_434376, EPI_ISL_434377, EPI_ISL_434378, EPI_ISL_434379, EPI_ISL_434380, EPI_ISL_434381, EPI_ISL_434383	Hospital AZ Rivierenland	Institute of Tropical Medicine	Philippe Selhorst, Colin Anthony,
EPI_ISL_434384	Hospital AZ Rivierenland	Institute of Tropical Medicine	Philippe Selhorst, Colin Anthony
EPI_ISL_434533	Area de Salud Alajuela Sur	Inciensa, Instituto Costarricense de Investigación y Enseñanza en Nutrición y Salud	Francisco Duarte, Hebleen Porras, Claudio Soto-Garita, Estela Cordero, Adriana Godinez & Melany Calderon
EPI_ISL_434534	National Institute for Viral Disease Control and Prevention, China CDC	National Institute for Viral Disease Control and Prevention, China CDC, Yunnan Provincial CDC	Wenjie Tan, Roujian Lu, Wenling Wang, Peihua Niu, Huijuan Wang, Baoying Huang, Li Zhao, Fei Ye, Guizhen Wu
EPI_ISL_434535	Area de Salud Alajuela Sur	Inciensa, Instituto Costarricense de Investigación y Enseñanza en Nutrición y Salud	Francisco Duarte, Hebleen Porras, Claudio Soto-Garita, Estela Cordero, Adriana Godinez & Melany Calderon
EPI_ISL_434538	COOPESAIN	Inciensa, Instituto Costarricense de Investigación y Enseñanza en Nutrición y Salud	Francisco Duarte, Hebleen Porras, Claudio Soto-Garita, Estela Cordero, Adriana Godinez & Melany Calderon
EPI_ISL_434539	Area de Salud Orotina	Inciensa, Instituto Costarricense de Investigación y Enseñanza en Nutrición y Salud	Francisco Duarte, Hebleen Porras, Claudio Soto-Garita, Estela Cordero, Adriana Godinez & Melany Calderon
EPI_ISL_434541, EPI_ISL_434543, EPI_ISL_434545, EPI_ISL_434546, EPI_ISL_434547, EPI_ISL_434548,	Puerto Rico Department of Health	Centers for Disease Control and Prevention, Dengue Branch	Gilberto A. Santiago, Glenda Gonzalez, Betzabel Flores, Keyla Charriez, Fabiola Cruz, Chaney Kalinich, Joseph Fauver, Jessica I. Falcon, Nathan Grubaugh, Jorge L. Munoz-Jordan

EPI_ISL_434549, EPI_ISL_434551			
EPI_ISL_434572	The National Institute of Public Health Center for Epidemiology and Microbiology	The National Institute of Public Health Center for Epidemiology and Microbiology	Alexander Nagy, Helena Jirincova, Ludmila Novakova, Dusan Trnka, Jaromira Vecerova
EPI_ISL_435045	Laboratory of Applied Genetics	RSE "National Center for Biotechnology"	Alexandr Shevtsov, Ilyas Akhmetollayev, Viktoriya Lutsay, Asylulan Amirgazin, Ruslan Kalendar, Yerlan Ramanculov
EPI_ISL_435049	B.J. Medical College and Civil hospital	Gujarat Biotechnology Research Centre	Pinal Trivedi, Maharshi Pandya, Amit Kanani, Akanksha Verma, Nitin Savaliya, Raghawendra Kumar, Dinesh Kumar, Zuber Saiyed, Dipa Kinariwala, Disha Patel, Binita Aring, Geeta Vaghela, Sonia Barve, Bhavesh Modi, Kairavi Joshi, Gaurishankar Shrimali, Nidhi Sood, Pranay Shah, R D Dixit, Snehal Bagatharia, Kamlesh J Upadhyay, Ramesh Pandit, Tejas Shah, Ankit Hinsu, Pritesh Sabara, Apurvasinh Puvar, Janvi Raval, Monika Gandhi, Neha Rajpara, Chaitanya Joshi, Madhvi Joshi
EPI_ISL_435050	B.J. Medical College and Civil hospital	Gujarat Biotechnology Research Centre	Ankit Hinsu, Pritesh Sabara, Apurvasinh Puvar, Janvi Raval, Monika Gandhi, Pinal Trivedi, Maharshi Pandya, Amit Kanani, Akanksha Verma, Nitin Savaliya, Raghawendra Kumar, Dinesh Kumar, Zuber Saiyed, Dipa Kinariwala, Disha Patel, Binita Aring, Geeta Vaghela, Sonia Barve, Bhavesh Modi, Kairavi Joshi, Gaurishankar Shrimali, Nidhi Sood, Pranay Shah, R D Dixit, Snehal Bagatharia, Kamlesh J Upadhyay, Ramesh Pandit, Tejas Shah, Dipeshwari Shewale, Chaitanya Joshi, Madhvi Joshi
EPI_ISL_435051	B.J. Medical College and Civil hospital	Gujarat Biotechnology Research Centre	Pritesh Sabara, Apurvasinh Puvar, Janvi Raval, Monika Gandhi, Pinal Trivedi, Maharshi Pandya, Amit Kanani, Akanksha Verma, Nitin Savaliya, Raghawendra Kumar, Dinesh Kumar, Zuber Saiyed, Dipa Kinariwala, Disha Patel, Binita Aring, Geeta Vaghela, Sonia Barve, Bhavesh Modi, Kairavi Joshi, Gaurishankar Shrimali, Nidhi Sood, Pranay Shah, R D Dixit, Snehal Bagatharia, Kamlesh J Upadhyay, Ramesh Pandit, Tejas Shah, Ankit Hinsu, Vasudha Sharma, Chaitanya Joshi, Madhvi Joshi
EPI_ISL_435052	B.J. Medical College and Civil hospital	Gujarat Biotechnology Research Centre	Apurvasinh Puvar, Janvi Raval, Monika Gandhi, Pinal Trivedi, Maharshi Pandya, Amit Kanani, Akanksha Verma, Nitin Savaliya, Raghawendra Kumar, Dinesh Kumar, Zuber Saiyed, Dipa Kinariwala, Disha Patel, Binita Aring, Geeta Vaghela, Sonia Barve, Bhavesh Modi, Kairavi Joshi, Gaurishankar Shrimali, Nidhi Sood, Pranay Shah, R D Dixit, Snehal Bagatharia, Kamlesh J Upadhyay, Ramesh Pandit, Tejas Shah, Ankit Hinsu, Pritesh Sabara, Pooja P Doshi, Chaitanya Joshi, Madhvi Joshi
EPI_ISL_435053	B.J. Medical College and Civil hospital	Gujarat Biotechnology Research Centre	Janvi Raval, Monika Gandhi, Pinal Trivedi, Maharshi Pandya, Amit Kanani, Akanksha Verma, Nitin Savaliya, Raghawendra Kumar, Dinesh Kumar, Zuber Saiyed, Dipa Kinariwala, Disha Patel, Binita Aring, Geeta Vaghela, Sonia Barve, Bhavesh Modi, Kairavi Joshi, Gaurishankar Shrimali, Nidhi Sood, Pranay Shah, R D Dixit, Snehal Bagatharia, Kamlesh J Upadhyay, Ramesh Pandit, Tejas Shah, Ankit Hinsu, Pritesh Sabara, Apurvasinh Puvar, Nidhi Patel, Chaitanya Joshi, Madhvi Joshi
EPI_ISL_435055	Gujarat Biotechnology Research Centre	Gujarat Biotechnology Research Centre	Tejas Shah, Ankit Hinsu, Pritesh Sabara, Apurvasinh Puvar, Janvi Raval, Monika Gandhi, Pinal Trivedi, Maharshi Pandya, Amit Kanani, Akanksha Verma, Nitin Savaliya, Raghawendra Kumar, Dinesh Kumar, Zuber Saiyed, Dipa Kinariwala, Disha Patel, Binita Aring, Geeta Vaghela, Sonia Barve, Bhavesh Modi, Kairavi Joshi, Gaurishankar Shrimali, Nidhi Sood, Pranay Shah, R D Dixit, Snehal Bagatharia, Kamlesh J Upadhyay, Ramesh Pandit, Anjali Rajwal, Chaitanya Joshi, Madhvi Joshi
EPI_ISL_435056	Gujarat Biotechnology Research Centre	Gujarat Biotechnology Research Centre	Maharshi Pandya, Amit Kanani, Akanksha Verma, Nitin Savaliya, Raghawendra Kumar, Dinesh Kumar, Zuber Saiyed, Dipa Kinariwala, Disha Patel, Binita Aring, Geeta Vaghela, Sonia Barve, Bhavesh Modi, Kairavi Joshi, Gaurishankar Shrimali, Nidhi Sood, Pranay Shah, R D Dixit, Snehal Bagatharia, Kamlesh J Upadhyay, Ramesh Pandit, Tejas Shah, Ankit Hinsu, Pritesh Sabara, Apurvasinh Puvar, Janvi Raval, Monika Gandhi, Pinal Trivedi, Afzal Ansari, Chaitanya Joshi, Madhvi Joshi
EPI_ISL_435145	Ospedale Civile Giuseppe Mazzini	Istituto Zooprofilattico Sperimentale dell'Abruzzo e Molise "G.Caporale"	Lorusso A, Marcacci M, Di Domenico M, Ancora M, Curini V, Mangone I, Rinaldi A, Di Pasquale A, Cammà C, Puglia I, Savini G
EPI_ISL_435146, EPI_ISL_435147	Villa Serena del Dr. Leonardo Petruzzi	Istituto Zooprofilattico Sperimentale dell'Abruzzo e Molise "G.Caporale"	Lorusso A, Marcacci M, Di Domenico M, Ancora M, Curini V, Mangone I, Rinaldi A, Di Pasquale A, Cammà C, Puglia I, Savini G
EPI_ISL_435148	Ospedale SS Annunziata	Istituto Zooprofilattico Sperimentale dell'Abruzzo e Molise "G.Caporale"	Lorusso A, Marcacci M, Di Domenico M, Ancora M, Curini V, Mangone I, Rinaldi A, Di Pasquale A, Cammà C, Puglia I, Savini G
EPI_ISL_435149	SERVIZIO DI IGIENE E SANITÀ PUBBLICA ASL Teramo	Istituto Zooprofilattico Sperimentale dell'Abruzzo e Molise "G.Caporale"	Lorusso A, Marcacci M, Di Domenico M, Ancora M, Curini V, Mangone I, Rinaldi A, Di Pasquale A, Cammà C, Puglia I, Savini G
EPI_ISL_435150, EPI_ISL_435151	Ospedale SS Annunziata	Istituto Zooprofilattico Sperimentale dell'Abruzzo e Molise "G.Caporale"	Lorusso A, Marcacci M, Di Domenico M, Ancora M, Curini V, Mangone I, Rinaldi A, Di Pasquale A, Cammà C, Puglia I, Savini G
EPI_ISL_435152	Servizio di Igiene, Epidemiologia e Sanità Pubblica (SIESP) Avezzano	Istituto Zooprofilattico Sperimentale dell'Abruzzo e Molise "G.Caporale"	Lorusso A, Marcacci M, Di Domenico M, Ancora M, Curini V, Mangone I, Rinaldi A, Di Pasquale A, Cammà C, Puglia I, Savini G
EPI_ISL_435153, EPI_ISL_435154, EPI_ISL_435155	SERVIZIO DI IGIENE E SANITÀ PUBBLICA ASL Teramo	Istituto Zooprofilattico Sperimentale dell'Abruzzo e Molise "G.Caporale"	Lorusso A, Marcacci M, Di Domenico M, Ancora M, Curini V, Mangone I, Rinaldi A, Di Pasquale A, Cammà C, Puglia I, Savini G
EPI_ISL_435281	Medistra Hospital Jakarta	Eijkman Institute for Molecular Biology, Ministry of Research and Technology/National Agency for Research and Innovation	Edison Johar, Frilasita A Yudhaputri, Hidayat Trimarsanto, David H Muljono, Safarina G Malik, Khin Saw Myint, Amin Soebandrio
EPI_ISL_435282, EPI_ISL_435283	RS Pondok Indah Hospital - Pondok Indah	Eijkman Institute for Molecular Biology, Ministry of Research and Technology/National Agency for Research and Innovation	Edison Johar, Frilasita A Yudhaputri, Hidayat Trimarsanto, David H Muljono, Safarina G Malik, Khin Saw Myint, Amin Soebandrio
EPI_ISL_435284	Central Virology Laboratory, Israel Ministry of Health	Central Virology Laboratory, Israel Ministry of Health	Neta Zuckerman, Efrat Bucris, Oran Erster, Danit Sofer, Orna Mor, Ella Mendelson, Michal Mandelboim
EPI_ISL_435286	Central Virology Laboratory, Israel Ministry of Health	Central Virology Laboratory, Israel Ministry of Health	eta Zuckerman, Efrat Bucris, Oran Erster, Orna Mor, Ella Mendelson, Michal Mandelboim, Danit Sofer
EPI_ISL_435289, EPI_ISL_435291	Central Virology Laboratory, Israel Ministry of Health	Central Virology Laboratory, Israel Ministry of Health	Neta Zuckerman, Efrat Bucris, Oran Erster, Danit Sofer, Orna Mor, Ella Mendelson, Michal Mandelboim
EPI_ISL_435292	Central Virology Laboratory, Israel Ministry of Health	Central Virology Laboratory, Israel Ministry of Health	Neta Zuckerman, Efrat Bucris, Oran Erster, Danit Sofer, Ella Mendelson, Michal Mandelboim, Orna Mor
EPI_ISL_435303	National Hospital of Tropical Diseases	Oxford University Clinical Research Unit, Hanoi, Vietnam	Nguyen Thi Tam, Van Dinh Trang, Nguyen Thu Trang, Nguyen Thi Ngoc Diep, Le Nguyen Minh Hoa, Pham Ngoc Thach, H.Rogier van Doorn, on behalf of the OUCRU COVID-19 research group
EPI_ISL_435305	National Hospital of Tropical Diseases	Oxford University Clinical Research Unit, Hanoi, Vietnam	Nguyen Thi Tam, Van Dinh Trang, Nguyen Thu Trang, Nguyen Thi Ngoc Diep, Le Nguyen Minh Hoa, Pham Ngoc Thach, H. Rogier van Doorn, on behalf of the OUCRU COVID-19 research group
EPI_ISL_435405, EPI_ISL_435407, EPI_ISL_435408, EPI_ISL_435410, EPI_ISL_435412, EPI_ISL_435414, EPI_ISL_435416, EPI_ISL_435419, EPI_ISL_435422, EPI_ISL_435423, EPI_ISL_435424, EPI_ISL_435425, EPI_ISL_435430, EPI_ISL_435431			
see above	Virological Research Group, Szentágothai Research Centre	Bioinformatics Research Group, Szentágothai Research Centre	Péter Urbán, Endre Gábor Tóth, Gábor Kemenesi, Róbert Herczeg, Attila Gyenesei, Ferenc Jakab
EPI_ISL_436099	TSGH-CP molecular lab	TSGH-CP molecular lab	Cherng-Lih Perng, Ming-Jr JIAN, Chih-Kai Chang, Jung-Chung Lin, Kuo-Ming Yeh, Chien-Wen Chen, Sheng-Kang Chiu, Hsing-Yi Chung, Shih-Hung Tsai, Kuo-Sheng Hung, Tien-Yao Chang, Feng-Yee Chang, Hung-Sheng Shang
EPI_ISL_436100	TSGH-CP molecular lab	TSGH-CP molecular lab	Cherng-Lih Perng, Ming-Jr Jian, Chih-Kai Chang, Jung-Chung Lin, Kuo-Ming Yeh, Chien-Wen Chen, Sheng-Kang Chiu, Hsing-Yi Chung, Shih-Hung Tsai, Kuo-Sheng Hung, Tien-Yao Chang, Feng-Yee Chang, Hung-Sheng Shang
EPI_ISL_436101, EPI_ISL_436102, EPI_ISL_436103, EPI_ISL_436104, EPI_ISL_436106, EPI_ISL_436107, EPI_ISL_436108	TSGH-CP molecular lab	TSGH-CP molecular lab	Cherng-Lih Perng, Ming-Jr JIAN, Chih-Kai Chang, Jung-Chung Lin, Kuo-Ming Yeh, Chien-Wen Chen, Sheng-Kang Chiu, Hsing-Yi Chung, Shih-Hung Tsai, Kuo-Sheng Hung, Tien-Yao Chang, Feng-Yee Chang, Hung-Sheng Shang
EPI_ISL_436157	District Surveillance Unit	Department of Neurovirology, National Institute of Mental Health and Neuroscience (NIMHANS)	Chitra Pattabiraman, Vijayalakshmi Reddy, Harsha PK, Risha Rasheed, Shafeeq S Hameed, Manjunatha Venkataswamy, Anita Desai, Ravi Vasanthapuram

EPI_ISL_436412	Viral Respiratory Lab, National Institute for Biomedical Research (INRB)	Pathogen Sequencing Lab, National Institute for Biomedical Research (INRB)	Placide Mbala-Kingebeni, Edith Nkwembe, Eddy Kinganda-Lusamaki, Amuri Aziza, Francisca Muyembe Mawete, Catherine Pratt, Matthias Pauthner, Josh Quick, Allison Black, James Hadfield, Trevor Bedford, Ian Goodfellow, Andrew Rambaut, Nick Loman, Kristian Andersen, Michael Wiley, Steve Ahuka-Mundeke, Jean-Jacques Muyembe Tamfum
EPI_ISL_436684, EPI_ISL_436686	KRISP, KZN Research Innovation and Sequencing Platform	KRISP, KZN Research Innovation and Sequencing Platform	Giandhari J, Pillay S, Lessells R, Chimukangara B, Deforche K, Tegally H, Wilkinson E, de Oliveira T
EPI_ISL_436715, EPI_ISL_436716, EPI_ISL_436717	Genomics and Computational Biology Lab, Scientific Research Institute of Physical-Chemical Medicine, FMBA of Russia	Genomics and Computational Biology Lab, Scientific Research Institute of Physical-Chemical Medicine, FMBA of Russia	A. Pavlenko, O. Guskova, K. Klimina, V. Veselovsky, A. Manolov, D. Fedorov, V. Govorun and E. Ilina
EPI_ISL_436718	Ospedale Regionale San Salvatore	Istituto Zooprofilattico Sperimentale dell'Abruzzo e Molise "G.Caporale"	Lorusso A, Marcacci M, Di Domenico M, Ancora M, Curini V, Mangone I, Rinaldi A, Di Pasquale A, Cammà C, Puglia I, Savini G
EPI_ISL_436719, EPI_ISL_436720, EPI_ISL_436721, EPI_ISL_436722	Ospedale Civile S. Liberatore di Atri	Istituto Zooprofilattico Sperimentale dell'Abruzzo e Molise "G.Caporale"	Lorusso A, Marcacci M, Di Domenico M, Ancora M, Curini V, Mangone I, Rinaldi A, Di Pasquale A, Cammà C, Puglia I, Savini G
EPI_ISL_436723	Ospedale Civile Giuseppe Mazzini	Istituto Zooprofilattico Sperimentale dell'Abruzzo e Molise "G.Caporale"	Lorusso A, Marcacci M, Di Domenico M, Ancora M, Curini V, Mangone I, Rinaldi A, Di Pasquale A, Cammà C, Puglia I, Savini G
EPI_ISL_436724	Ospedale Civile S. Liberatore di Atri	Istituto Zooprofilattico Sperimentale dell'Abruzzo e Molise "G.Caporale"	Lorusso A, Marcacci M, Di Domenico M, Ancora M, Curini V, Mangone I, Rinaldi A, Di Pasquale A, Cammà C, Puglia I, Savini G
EPI_ISL_436725	RSA/RP Villa San Giovanni - Gruppo Edos	Istituto Zooprofilattico Sperimentale dell'Abruzzo e Molise "G.Caporale"	Lorusso A, Marcacci M, Di Domenico M, Ancora M, Curini V, Mangone I, Rinaldi A, Di Pasquale A, Cammà C, Puglia I, Savini G
EPI_ISL_436726, EPI_ISL_436727, EPI_ISL_436729	SERVIZIO DI IGIENE E SANITÀ PUBBLICA ASL Teramo	Istituto Zooprofilattico Sperimentale dell'Abruzzo e Molise "G.Caporale"	Lorusso A, Marcacci M, Di Domenico M, Ancora M, Curini V, Mangone I, Rinaldi A, Di Pasquale A, Cammà C, Puglia I, Savini G
EPI_ISL_436730	Servizio di igiene epidemiologia e sanità pubblica (Siesp) Chieti	Istituto Zooprofilattico Sperimentale dell'Abruzzo e Molise "G.Caporale"	Lorusso A, Marcacci M, Di Domenico M, Ancora M, Curini V, Mangone I, Rinaldi A, Di Pasquale A, Cammà C, Puglia I, Savini G
EPI_ISL_436731, EPI_ISL_436732	Ospedale Civile S. Liberatore di Atri	Istituto Zooprofilattico Sperimentale dell'Abruzzo e Molise "G.Caporale"	Lorusso A, Marcacci M, Di Domenico M, Ancora M, Curini V, Mangone I, Rinaldi A, Di Pasquale A, Cammà C, Puglia I, Savini G
EPI_ISL_436939, EPI_ISL_436942, EPI_ISL_436943, EPI_ISL_436944, EPI_ISL_436945, EPI_ISL_436946, EPI_ISL_436947, EPI_ISL_436948, EPI_ISL_436949, EPI_ISL_436950, EPI_ISL_436952, EPI_ISL_436953, EPI_ISL_436954, EPI_ISL_436956, EPI_ISL_436957, EPI_ISL_436958, EPI_ISL_436959, EPI_ISL_436960, EPI_ISL_436961			
see above	Ochsner Health	Bioinfoexperts, LLC	Amy Feehan, David J. Nolan, Rebecca Rose, Sissy Cross, David Moraga Amador, Tong Yang, Luke Caruso, Wayra Navia, Lydia Von Borstel, Xiao Hui Zhou, Julia-Garcia-Diaz, Susanna L. Lamers
EPI_ISL_437187	Siloam Hospitals	Institute of Tropical Disease, Universitas Airlangga	Kazufumi Shimizu, Krisnoadi Rahardjo, Aldise M Nastri, Jezzy R Dewantari, Rima R Prasetya, Maria M Padmidewi, Gatot Soegiarto, Laksmi Wulandari, Retno A Setyoningrum, Resti Y Meliana, Yohko K Shimizu, Mitsuhiro Nishimura, Yasuko Mori, Soetjipto, Maria I Lusida
EPI_ISL_437188	RSUD Dr. Soetomo	Institute of Tropical Disease, Universitas Airlangga	Krisnoadi Rahardjo, Aldise M Nastri, Jezzy R Dewantari, Rima R Prasetya, Joni Wahyuhadi, Gatot Soegiarto, Laksmi Wulandari, Retno A Setyoningrum, Resti Y Meliana, Yohko K Shimizu, Mitsuhiro Nishimura, Yasuko Mori, Soetjipto, Kazufumi Shimizu, Maria I Lusida
EPI_ISL_437189	Pusat Pertamina Hospital	Eijkman Institute for Molecular Biology, Ministry of Research and Technology/National Agency for Research and Innovation	Edison Johar, Frilasita A Yudhaputri, Hidayat Trimarsanto, David H Muljono, Safarina G Malik, Khin Saw Myint, Amin Soebandrio
EPI_ISL_437190, EPI_ISL_437191	RS Pondok Indah Hospital - Pondok Indah	Eijkman Institute for Molecular Biology, Ministry of Research and Technology/National Agency for Research and Innovation	Edison Johar, Frilasita A Yudhaputri, Hidayat Trimarsanto, David H Muljono, Safarina G Malik, Khin Saw Myint, Amin Soebandrio
EPI_ISL_437192	Mitra Keluarga Kelapa Gading Hospital	Eijkman Institute for Molecular Biology, Ministry of Research and Technology/National Agency for Research and Innovation	Edison Johar, Frilasita A Yudhaputri, Hidayat Trimarsanto, David H Muljono, Safarina G Malik, Khin Saw Myint, Amin Soebandrio
EPI_ISL_437194	Viral Respiratory Lab, National Institute for Biomedical Research (INRB)	Pathogen Sequencing Lab, National Institute for Biomedical Research (INRB)	Placide Mbala-Kingebeni, Edith Nkwembe, Eddy Kinganda-Lusamaki, Amuri Aziza, Francisca Muyembe Mawete, Catherine Pratt, Matthias Pauthner, Josh Quick, Allison Black, James Hadfield, Trevor Bedford, Ian Goodfellow, Andrew Rambaut, Nick Loman, Kristian Andersen, Michael Wiley, Steve Ahuka-Mundeke, Jean-Jacques Muyembe Tamfum
EPI_ISL_437197, EPI_ISL_437198, EPI_ISL_437199, EPI_ISL_437200, EPI_ISL_437201, EPI_ISL_437202, EPI_ISL_437203, EPI_ISL_437298, EPI_ISL_437299, EPI_ISL_437300, EPI_ISL_437301, EPI_ISL_437302, EPI_ISL_437303			
see above	Diagnostic- and Research Institute of Pathology, Medical University of Graz	Diagnostic- and Research Institute of Pathology, Medical University of Graz	Karl Kashofer, Peter Regitnig, Martin Zacharias, Gregor Gorkiewicz
EPI_ISL_437339	Viral Respiratory Lab, National Institute for Biomedical Research (INRB)	Pathogen Sequencing Lab, National Institute for Biomedical Research (INRB)	Placide Mbala-Kingebeni, Edith Nkwembe, Eddy Kinganda-Lusamaki, Amuri Aziza, Francisca Muyembe Mawete, Catherine Pratt, Matthias Pauthner, Josh Quick, Allison Black, James Hadfield, Trevor Bedford, Ian Goodfellow, Andrew Rambaut, Nick Loman, Kristian Andersen, Michael Wiley, Steve Ahuka-Mundeke, Jean-Jacques Muyembe Tamfum
EPI_ISL_437433	Bozeman Health Deaconess Hospital	Wiedenheft lab, Montana State University	Artem Nemudryi, Anna Nemudraia, Kevin Surya, Tanner Wiegand, Murat Buyukyoruk, Royce Wilkinson, Blake Wiedenheft
EPI_ISL_437440	Department of MicroBiology, Government Medical College, Surat	Gujarat Biotechnology Research Centre	Nitin Savaliya, Raghawendra Kumar, Dinesh Kumar, Zuber Saiyed, Dipa Kinariwala, Disha Patel, Binita Aring, Neeta Khandelwal, Geeta Vaghela, Sonia Barve, Bhavesh Modi, Kairavi Joshi, Gaurishankar Shrimali, Nidhi Sood, Pranay Shah, R D Dixit, Snehal Bagatharia, Kamlesh J Upadhyay, Ramesh Pandit, Tejas Shah, Ankit Hinsu, Pritesh Sabara, Apurvasinh Puvar, Janvi Raval, Monika Gandhi, Pinal Trivedi, Maharshi Pandya, Amit Kanani, Akanksha Verma, Bhavya Jindal, Chaitanya Joshi, Madhvi Joshi
EPI_ISL_437442	Department of MicroBiology, Government Medical College, Surat	Gujarat Biotechnology Research Centre	Dinesh Kumar, Zuber Saiyed, Dipa Kinariwala, Disha Patel, Binita Aring, Neeta Khandelwal, Geeta Vaghela, Sonia Barve, Bhavesh Modi, Kairavi Joshi, Gaurishankar Shrimali, Nidhi Sood, Pranay Shah, R D Dixit, Snehal Bagatharia, Kamlesh J Upadhyay, Ramesh Pandit, Tejas Shah, Ankit Hinsu, Pritesh Sabara, Apurvasinh Puvar, Janvi Raval, Monika Gandhi, Pinal Trivedi, Maharshi Pandya, Amit Kanani, Akanksha Verma, Nitin Savaliya, Raghawendra Kumar, Dipeshwari Shewale, Chaitanya Joshi, Madhvi Joshi
EPI_ISL_437445	B.J. Medical College and Civil hospital	Gujarat Biotechnology Research Centre	Disha Patel, Binita Aring, Neeta Khandelwal, Geeta Vaghela, Sonia Barve, Bhavesh Modi, Kairavi Joshi, Gaurishankar Shrimali, Nidhi Sood, Pranay Shah, R D Dixit, Snehal Bagatharia, Kamlesh J Upadhyay, Ramesh Pandit, Tejas Shah, Ankit Hinsu, Pritesh Sabara, Apurvasinh Puvar, Janvi Raval, Monika Gandhi, Pinal Trivedi, Maharshi Pandya, Amit Kanani, Akanksha Verma, Nitin Savaliya, Raghawendra Kumar, Dinesh Kumar, Zuber Saiyed, Dipa Kinariwala, Nidhi Patel, Chaitanya Joshi, Madhvi Joshi
EPI_ISL_437446	B.J. Medical College and Civil hospital	Gujarat Biotechnology Research Centre	Binita Aring, Neeta Khandelwal, Geeta Vaghela, Sonia Barve, Bhavesh Modi, Kairavi Joshi, Gaurishankar Shrimali, Nidhi Sood, Pranay Shah, R D Dixit, Snehal Bagatharia, Kamlesh J Upadhyay, Ramesh Pandit, Tejas Shah, Ankit Hinsu, Pritesh Sabara, Apurvasinh Puvar, Janvi Raval, Monika Gandhi, Pinal Trivedi, Maharshi Pandya, Amit Kanani, Akanksha Verma, Nitin Savaliya, Raghawendra Kumar, Dinesh Kumar, Zuber Saiyed, Dipa Kinariwala, Disha Patel, Priti Pandita, Chaitanya Joshi, Madhvi Joshi
EPI_ISL_437447	B.J. Medical College and Civil hospital	Gujarat Biotechnology Research Centre	Neeta Khandelwal, Geeta Vaghela, Sonia Barve, Bhavesh Modi, Kairavi Joshi, Gaurishankar Shrimali, Nidhi Sood, Pranay Shah, R D Dixit, Snehal Bagatharia, Kamlesh J Upadhyay, Ramesh Pandit, Tejas Shah, Ankit Hinsu, Pritesh Sabara, Apurvasinh Puvar, Janvi Raval, Monika Gandhi, Pinal Trivedi, Maharshi Pandya, Amit Kanani, Akanksha Verma, Nitin Savaliya, Raghawendra Kumar, Dinesh Kumar, Zuber Saiyed, Dipa Kinariwala, Disha Patel, Binita Aring, Neha Rajpara, Chaitanya Joshi, Madhvi Joshi
EPI_ISL_437448	B.J. Medical College and Civil hospital	Gujarat Biotechnology Research Centre	Geeta Vaghela, Sonia Barve, Bhavesh Modi, Kairavi Joshi, Gaurishankar Shrimali, Nidhi Sood, Pranay Shah, R D Dixit, Snehal Bagatharia, Kamlesh J Upadhyay, Ramesh Pandit, Tejas Shah, Ankit Hinsu, Pritesh Sabara, Apurvasinh Puvar, Janvi Raval, Monika Gandhi, Pinal Trivedi, Maharshi Pandya, Amit Kanani, Akanksha Verma, Nitin Savaliya, Raghawendra Kumar, Dinesh Kumar, Zuber Saiyed, Dipa Kinariwala, Disha Patel, Binita Aring, Neeta Khandelwal,

			Afzal Ansari, Chaitanya Joshi, Madhvi Joshi
EPI_ISL_437449	B.J. Medical College and Civil hospital	Gujarat Biotechnology Research Centre	Sonia Barve, Bhavesh Modi, Kairavi Joshi, Gaurishankar Shrimali, Nidhi Sood, Pranay Shah, R D Dixit, Snehal Bagatharia, Kamlesh J Upadhyay, Ramesh Pandit, Tejas Shah, Ankit Hinsu, Pritesh Sabara, Apurvasinh Puvar, Janvi Raval, Monika Gandhi, Pinal Trivedi, Maharshi Pandya, Amit Kanani, Akanksha Verma, Nitin Savaliya, Raghawendra Kumar, Dinesh Kumar, Zuber Saiyed, Dipa Kinariwala, Disha Patel, Binita Aring, Neeta Khandelwal, Geeta Vaghela, Neelam Nathani, Chaitanya Joshi, Madhvi Joshi
EPI_ISL_437450	B.J. Medical College and Civil hospital	Gujarat Biotechnology Research Centre	Bhavesh Modi, Kairavi Joshi, Gaurishankar Shrimali, Nidhi Sood, Pranay Shah, R D Dixit, Snehal Bagatharia, Kamlesh J Upadhyay, Ramesh Pandit, Tejas Shah, Ankit Hinsu, Pritesh Sabara, Apurvasinh Puvar, Janvi Raval, Monika Gandhi, Pinal Trivedi, Maharshi Pandya, Amit Kanani, Akanksha Verma, Nitin Savaliya, Raghawendra Kumar, Dinesh Kumar, Zuber Saiyed, Dipa Kinariwala, Disha Patel, Binita Aring, Neeta Khandelwal, Geeta Vaghela, Sonia Barve, Armi Chaudhari, Chaitanya Joshi, Madhvi Joshi
EPI_ISL_437452	B.J. Medical College and Civil hospital	Gujarat Biotechnology Research Centre	Gaurishankar Shrimali, Nidhi Sood, Pranay Shah, R D Dixit, Snehal Bagatharia, Kamlesh J Upadhyay, Ramesh Pandit, Tejas Shah, Ankit Hinsu, Pritesh Sabara, Apurvasinh Puvar, Janvi Raval, Monika Gandhi, Pinal Trivedi, Maharshi Pandya, Amit Kanani, Akanksha Verma, Nitin Savaliya, Raghawendra Kumar, Dinesh Kumar, Zuber Saiyed, Dipa Kinariwala, Disha Patel, Binita Aring, Neeta Khandelwal, Geeta Vaghela, Sonia Barve, Bhavesh Modi, Kairavi Joshi, Chaitanya Joshi, Anjali Rajwar, Madhvi Joshi
EPI_ISL_437453	B.J. Medical College and Civil hospital	Gujarat Biotechnology Research Centre	Nidhi Sood, Pranay Shah, R D Dixit, Snehal Bagatharia, Kamlesh J Upadhyay, Ramesh Pandit, Tejas Shah, Ankit Hinsu, Pritesh Sabara, Apurvasinh Puvar, Janvi Raval, Monika Gandhi, Pinal Trivedi, Maharshi Pandya, Amit Kanani, Akanksha Verma, Nitin Savaliya, Raghawendra Kumar, Dinesh Kumar, Zuber Saiyed, Dipa Kinariwala, Disha Patel, Binita Aring, Neeta Khandelwal, Geeta Vaghela, Sonia Barve, Bhavesh Modi, Kairavi Joshi, Gaurishankar Shrimali, Chaitanya Joshi, Dipeshwari Shewale, Madhvi Joshi
EPI_ISL_437454	B.J. Medical College and Civil hospital	Gujarat Biotechnology Research Centre	Pranay Shah, R D Dixit, Snehal Bagatharia, Kamlesh J Upadhyay, Ramesh Pandit, Tejas Shah, Ankit Hinsu, Pritesh Sabara, Apurvasinh Puvar, Janvi Raval, Monika Gandhi, Pinal Trivedi, Maharshi Pandya, Amit Kanani, Akanksha Verma, Nitin Savaliya, Raghawendra Kumar, Dinesh Kumar, Zuber Saiyed, Dipa Kinariwala, Disha Patel, Binita Aring, Neeta Khandelwal, Geeta Vaghela, Sonia Barve, Bhavesh Modi, Kairavi Joshi, Gaurishankar Shrimali, Nidhi Sood, Chaitanya Joshi, Sharmistha Majumdar, Madhvi Joshi
EPI_ISL_437459, EPI_ISL_437460, EPI_ISL_437461, EPI_ISL_437462, EPI_ISL_437463, EPI_ISL_437464, EPI_ISL_437465, EPI_ISL_437466, EPI_ISL_437468, EPI_ISL_437469, EPI_ISL_437470, EPI_ISL_437471, EPI_ISL_437472, EPI_ISL_437473, EPI_ISL_437474, EPI_ISL_437475, EPI_ISL_437477, EPI_ISL_437479			
see above	Pathogen Genomics Lab King Abdullah University of Science and Technology(KAUST)	Pathogen Genomics Lab King Abdullah University of Science and Technology(KAUST)	Sharif Hala,Raeece Naeem,Sara Mfarrej,Arnab Pain
EPI_ISL_437481, EPI_ISL_437482, EPI_ISL_437483, EPI_ISL_437484, EPI_ISL_437485, EPI_ISL_437486, EPI_ISL_437487, EPI_ISL_437488, EPI_ISL_437489, EPI_ISL_437490, EPI_ISL_437491, EPI_ISL_437492, EPI_ISL_437493, EPI_ISL_437494, EPI_ISL_437495, EPI_ISL_437496, EPI_ISL_437497			
see above	Pathogen Genomics Lab King Abdullah University of Science and Technology(KAUST)	Pathogen Genomics Lab King Abdullah University of Science and Technology(KAUST)	Sara Mfarrej,Raeece Naeem,Sharif Hala,Amit Subudhi,Fathia Rached,Arnab Pain
EPI_ISL_437498, EPI_ISL_437499	Biotechnology Center for Advanced Technologies	Biotechnology Center for Advanced Technologies	Abdullaev,A., Abdurakhimov,A., Muminov,M., Nuriddinov,S., Dalimova,D., Tsoy,V., Tsay,E., Bozorov,S., Charishnikova,O., Dalimova,D. and Turdikulova,S.
EPI_ISL_437626	Department of Microbiology,Gandhi Medical College and Hospital	Department of Veterinary Biotechnalogy, College of Veterinary Science, Rajendranagar, PV Narsimha Rao Telengana Veterinary University	Kalyani Putty, Muttineni Radhakrishna, Nagamani K, Thrilok Chander B, Raja Rao M, Ravikumar P, Sunitha P, Pankaj Singh D, Anand Kumar K, Amit A. Upadhyay, Steven Bosinger, Rama Amara
EPI_ISL_437691, EPI_ISL_437692, EPI_ISL_437693, EPI_ISL_437694, EPI_ISL_437695, EPI_ISL_437696, EPI_ISL_437697, EPI_ISL_437698, EPI_ISL_437699, EPI_ISL_437700, EPI_ISL_437701, EPI_ISL_437702, EPI_ISL_437703, EPI_ISL_437704, EPI_ISL_437705, EPI_ISL_437706, EPI_ISL_437707, EPI_ISL_437708, EPI_ISL_437709, EPI_ISL_437710, EPI_ISL_437711, EPI_ISL_437712, EPI_ISL_437713, EPI_ISL_437714, EPI_ISL_437715, EPI_ISL_437716, EPI_ISL_437717, EPI_ISL_437718, EPI_ISL_437719, EPI_ISL_437720, EPI_ISL_437721, EPI_ISL_437722, EPI_ISL_437723, EPI_ISL_437724, EPI_ISL_437725, EPI_ISL_437726, EPI_ISL_437727, EPI_ISL_437728, EPI_ISL_437729, EPI_ISL_437730, EPI_ISL_437731, EPI_ISL_437732, EPI_ISL_437733, EPI_ISL_437734, EPI_ISL_437735, EPI_ISL_437736, EPI_ISL_437737, EPI_ISL_437738, EPI_ISL_437739, EPI_ISL_437740, EPI_ISL_437741, EPI_ISL_437742, EPI_ISL_437743, EPI_ISL_437744, EPI_ISL_437745, EPI_ISL_437746, EPI_ISL_437747, EPI_ISL_437748, EPI_ISL_437749, EPI_ISL_437750, EPI_ISL_437751, EPI_ISL_437752, EPI_ISL_437753, EPI_ISL_437754, EPI_ISL_437755, EPI_ISL_437756, EPI_ISL_437757, EPI_ISL_437758, EPI_ISL_437759, EPI_ISL_437760, EPI_ISL_437761, EPI_ISL_437762			
see above	Pathogen Genomics Lab King Abdullah University of Science and Technology(KAUST)	Pathogen Genomics Lab King Abdullah University of Science and Technology(KAUST)	Sharif Hala,Fadwa Alofi,Afrah Alsomali, Asim Khogeer, Sara Mfarrej, Khaled Algithami,Raeece Naeem, Amit Kumar Subudhi,Fathia Ben-Rached, Rahul Salunke, Anwar Hashem, Naif Almontashiri, Arnab Pain
EPI_ISL_438138	Department of Microbiology,Gandhi Medical College and Hospital	Department of Microbiology, Gandhi Medical College and Hospital Secendrabad, Hyderbad, India	Raja Rao Mesipogu, Muttineni Radhakrishna, Nagamani K, Thrilok Chander B, Kalyani Putty, Ravikumar P, Sunitha P, Pankaj Singh D, Anand Kumar K, Amit A. Upadhyay, Steven Bosinger, Rama Amara
EPI_ISL_438139	Department of Microbiology,Gandhi Medical College and Hospital,Hyderabad	Virus Research Laboratory, Department of Zoology, Osmania University, Hyderabad, India	Muttineni Radhakrishna, Nagamani K, Thrilok Chander B, Raja Rao M, Kalyani Putty, Ravikumar P, Sunitha P, Pankaj Singh D, Anand Kumar K, Amit A. Upadhyay, Steven Bosinger, Rama Amara
EPI_ISL_438546, EPI_ISL_438548	Siloam Hospital Lippo Village	Mochtar Riady Institute for Nanotechnology, Universitas Pelita Harapan	Aksar C Lages, David Rustandi, Febi Andriani, Ivet M Suriapranata, Riska N Taufik, Tri Shinta Kurniasih, Irawan Yusuf
EPI_ISL_442044	Kawsar Human Genetic Research Center	Kawsar Human Genetic Research Center	Mohammad Ali Khosravi, Maryam Abbasalipour Bashash, Sirous Zeinali, Solmaz Sabeghi, Yeganeh Keshvar, Fatemeh Hosseini, Yeganeh Haghdoost
EPI_ISL_442523	Pasteur Institute of Iran	Kawsar Human Genetic Research Company	Sirous Zeinali, Mohammad Ali Khosravi,Maryam Abbasalipour Bashash, Sanaz Mostafavi Jabbari, Maraym Firoozi, Sormeh Pourtavakoli, Elmira Khateri, Razieh Zeinali and Fahimeh Hoseini
EPI_ISL_443258, EPI_ISL_443259	Résidence Ornano	National Reference Center for Viruses of Respiratory Infections, Institut Pasteur, Paris	Mélanie Albert, Marion Barbet, Sylvie Behillil, Méline Bizard, Angela Brisebarre, Flora Donati, Etienne Simon-Lorière, Vincent Enouf, Maud Vanpeene, Sylvie van der Werf
EPI_ISL_443260	LABM GH nord Essonne de Longjumeau - BP 125	National Reference Center for Viruses of Respiratory Infections, Institut Pasteur, Paris	Mélanie Albert, Marion Barbet, Sylvie Behillil, Méline Bizard, Angela Brisebarre, Flora Donati, Etienne Simon-Lorière, Vincent Enouf, Maud Vanpeene, Sylvie van der Werf
EPI_ISL_443261, EPI_ISL_443262, EPI_ISL_443263, EPI_ISL_443264	CHU de Dijon - Laboratoire de Virologie	National Reference Center for Viruses of Respiratory Infections, Institut Pasteur, Paris	Mélanie Albert, Marion Barbet, Sylvie Behillil, Méline Bizard, Angela Brisebarre, Flora Donati, Etienne Simon-Lorière, Vincent Enouf, Maud Vanpeene, Sylvie van der Werf, Jean-Baptiste Bour
EPI_ISL_443265, EPI_ISL_443266, EPI_ISL_443267, EPI_ISL_443268, EPI_ISL_443269, EPI_ISL_443270, EPI_ISL_443271, EPI_ISL_443272, EPI_ISL_443273, EPI_ISL_443274, EPI_ISL_443275, EPI_ISL_443276, EPI_ISL_443277, EPI_ISL_443278, EPI_ISL_443279, EPI_ISL_443280, EPI_ISL_443281, EPI_ISL_443282, EPI_ISL_443283			
see above	CHU - Hôpital Cavale Blanche - Labo. de Virologie	National Reference Center for Viruses of Respiratory Infections, Institut Pasteur, Paris	Mélanie Albert, Marion Barbet, Sylvie Behillil, Méline Bizard, Angela Brisebarre, Flora Donati, Etienne Simon-Lorière, Vincent Enouf, Maud Vanpeene, Sylvie van der Werf, Léa Pilorge
EPI_ISL_443284, EPI_ISL_443285, EPI_ISL_443286, EPI_ISL_443287, EPI_ISL_443288	Laboratoire de Microbiologie - Bât A - CH René Dubois	National Reference Center for Viruses of Respiratory Infections, Institut Pasteur, Paris	Mélanie Albert, Marion Barbet, Sylvie Behillil, Méline Bizard, Angela Brisebarre, Flora Donati, Etienne Simon-Lorière, Vincent Enouf, Maud Vanpeene, Sylvie van der Werf, Pascale Martres
EPI_ISL_443289, EPI_ISL_443290, EPI_ISL_443291, EPI_ISL_443292, EPI_ISL_443293, EPI_ISL_443294	CHRU Pontchaillou - Laboratoire de Virologie	National Reference Center for Viruses of Respiratory Infections, Institut Pasteur, Paris	Mélanie Albert, Marion Barbet, Sylvie Behillil, Méline Bizard, Angela Brisebarre, Flora Donati, Etienne Simon-Lorière, Vincent Enouf, Maud Vanpeene, Sylvie van der Werf, Gisèle Lagathu
EPI_ISL_443295, EPI_ISL_443296, EPI_ISL_443298, EPI_ISL_443299	Hôpital Necker - Enfants - Malades Laboratoire de Virologie	National Reference Center for Viruses of Respiratory Infections, Institut Pasteur, Paris	Mélanie Albert, Marion Barbet, Sylvie Behillil, Méline Bizard, Angela Brisebarre, Flora Donati, Etienne Simon-Lorière, Vincent Enouf, Maud Vanpeene, Sylvie van der Werf, Marianne Leruez-Ville
EPI_ISL_443303	Résidence Les Marines	National Reference Center for Viruses of Respiratory Infections, Institut Pasteur, Paris	Mélanie Albert, Marion Barbet, Sylvie Behillil, Méline Bizard, Angela Brisebarre, Flora Donati, Etienne Simon-Lorière, Vincent Enouf, Maud Vanpeene, Sylvie van der Werf
EPI_ISL_443305	LABM GH nord Essonne de Longjumeau - BP 125	National Reference Center for Viruses of Respiratory Infections, Institut Pasteur, Paris	Mélanie Albert, Marion Barbet, Sylvie Behillil, Méline Bizard, Angela Brisebarre, Flora Donati, Etienne Simon-Lorière, Vincent Enouf, Maud Vanpeene, Sylvie van der Werf
EPI_ISL_443307	La Villa Papyri	National Reference Center for Viruses of Respiratory Infections, Institut Pasteur, Paris	Mélanie Albert, Marion Barbet, Sylvie Behillil, Méline Bizard, Angela Brisebarre, Flora Donati, Etienne Simon-Lorière, Vincent Enouf, Maud Vanpeene, Sylvie van der Werf
EPI_ISL_443308	Plaisance	National Reference Center for Viruses of Respiratory Infections, Institut Pasteur, Paris	Mélanie Albert, Marion Barbet, Sylvie Behillil, Méline Bizard, Angela Brisebarre, Flora Donati, Etienne Simon-Lorière, Vincent Enouf, Maud Vanpeene, Sylvie van der Werf

EPI_ISL_443309	CH Compiègne Laboratoire de Biologie	National Reference Center for Viruses of Respiratory Infections, Institut Pasteur, Paris	Mélanie Albert, Marion Barbet, Sylvie Behillil, Méline Bizard, Angela Brisebarre, Flora Donati, Etienne Simon-Lorière, Vincent Enouf, Maud Vanpeene, Sylvie van der Werf
EPI_ISL_443314	LABM GH nord Essonne de Longjumeau - BP 125	National Reference Center for Viruses of Respiratory Infections, Institut Pasteur, Paris	Mélanie Albert, Marion Barbet, Sylvie Behillil, Méline Bizard, Angela Brisebarre, Flora Donati, Etienne Simon-Lorière, Vincent Enouf, Maud Vanpeene, Sylvie van der Werf
EPI_ISL_443315	Château de la Source	National Reference Center for Viruses of Respiratory Infections, Institut Pasteur, Paris	Mélanie Albert, Marion Barbet, Sylvie Behillil, Méline Bizard, Angela Brisebarre, Flora Donati, Etienne Simon-Lorière, Vincent Enouf, Maud Vanpeene, Sylvie van der Werf
EPI_ISL_443316	CH Compiègne Laboratoire de Biologie	National Reference Center for Viruses of Respiratory Infections, Institut Pasteur, Paris	Mélanie Albert, Marion Barbet, Sylvie Behillil, Méline Bizard, Angela Brisebarre, Flora Donati, Etienne Simon-Lorière, Vincent Enouf, Maud Vanpeene, Sylvie van der Werf, Olivia Raulin
EPI_ISL_444022	Baylor College of Medicine	Baylor College of Medicine: HGSC	Vasanthi Avadhanula, Erin Nicholson, David Henke, Pedro Piedra, Harsha Doddapaneni, Donna Muzny, Qingchang Meng, Hsu Chao, Zeineen Momin, Hua Shen, George Weissenberger, Kavya Kottapalli, Yimiti Meiheerguli, Sejal Salvi, Ginger Metcalf, Vipin Menon, Sara J.J. Cregeen, Matthew C. Ross, Tulin Ayvaz, Richard Sucgang, Kristi L. Hoffman, Matthew Wong, Joseph F. Petrosino
EPI_ISL_444027	Pamela Youde Nethersole Eastern Hospital	Hong Kong Department of Health	Mak Gannon C.K., Cheng Peter K.C., Lam Edman T.K., Chan Rickjason C.W., Tsang Dominic N.C.
EPI_ISL_444028	Queen Elizabeth Hospital	Hong Kong Department of Health	Mak Gannon C.K., Cheng Peter K.C., Lam Edman T.K., Chan Rickjason C.W., Tsang Dominic N.C.
EPI_ISL_444030	United Christian Hospital	Hong Kong Department of Health	Mak Gannon C.K., Cheng Peter K.C., Lam Edman T.K., Chan Rickjason C.W., Tsang Dominic N.C.
EPI_ISL_444031	Queen Mary Hospital	Hong Kong Department of Health	Mak Gannon C.K., Cheng Peter K.C., Lam Edman T.K., Chan Rickjason C.W., Tsang Dominic N.C.
EPI_ISL_444032	North Lantau Hospital	Hong Kong Department of Health	Mak Gannon C.K., Cheng Peter K.C., Lam Edman T.K., Chan Rickjason C.W., Tsang Dominic N.C.
EPI_ISL_444033	Queen Mary Hospital	Hong Kong Department of Health	Mak Gannon C.K., Cheng Peter K.C., Lam Edman T.K., Chan Rickjason C.W., Tsang Dominic N.C.
EPI_ISL_444034	Prince of Wales Hospital	Hong Kong Department of Health	Mak Gannon C.K., Cheng Peter K.C., Lam Edman T.K., Chan Rickjason C.W., Tsang Dominic N.C.
EPI_ISL_444035	Princess Margaret Hospital	Hong Kong Department of Health	Mak Gannon C.K., Cheng Peter K.C., Lam Edman T.K., Chan Rickjason C.W., Tsang Dominic N.C.
EPI_ISL_444036	North Lantau Hospital	Hong Kong Department of Health	Mak Gannon C.K., Cheng Peter K.C., Lam Edman T.K., Chan Rickjason C.W., Tsang Dominic N.C.
EPI_ISL_444038	Princess Margaret Hospital	Hong Kong Department of Health	Mak Gannon C.K., Cheng Peter K.C., Lam Edman T.K., Chan Rickjason C.W., Tsang Dominic N.C.
EPI_ISL_444040	Queen Elizabeth Hospital	Hong Kong Department of Health	Mak Gannon C.K., Cheng Peter K.C., Lam Edman T.K., Chan Rickjason C.W., Tsang Dominic N.C.
EPI_ISL_444041	Tuen Mun Hospital	Hong Kong Department of Health	Mak Gannon C.K., Cheng Peter K.C., Lam Edman T.K., Chan Rickjason C.W., Tsang Dominic N.C.
EPI_ISL_444042, EPI_ISL_444043	Pamela Youde Nethersole Eastern Hospital	Hong Kong Department of Health	Mak Gannon C.K., Cheng Peter K.C., Lam Edman T.K., Chan Rickjason C.W., Tsang Dominic N.C.
EPI_ISL_444044	Yan Chai Hospital	Hong Kong Department of Health	Mak Gannon C.K., Cheng Peter K.C., Lam Edman T.K., Chan Rickjason C.W., Tsang Dominic N.C.
EPI_ISL_444045, EPI_ISL_444046	Queen Elizabeth Hospital	Hong Kong Department of Health	Mak Gannon C.K., Cheng Peter K.C., Lam Edman T.K., Chan Rickjason C.W., Tsang Dominic N.C.
EPI_ISL_444047	United Christian Hospital	Hong Kong Department of Health	Mak Gannon C.K., Cheng Peter K.C., Lam Edman T.K., Chan Rickjason C.W., Tsang Dominic N.C.
EPI_ISL_444048	Queen Elizabeth Hospital	Hong Kong Department of Health	Mak Gannon C.K., Cheng Peter K.C., Lam Edman T.K., Chan Rickjason C.W., Tsang Dominic N.C.
EPI_ISL_444049	North Lantau Hospital	Hong Kong Department of Health	Mak Gannon C.K., Cheng Peter K.C., Lam Edman T.K., Chan Rickjason C.W., Tsang Dominic N.C.
EPI_ISL_444050	Shek Wu Hui Jockey Club General Out-patient Clinic	Hong Kong Department of Health	Mak Gannon C.K., Cheng Peter K.C., Lam Edman T.K., Chan Rickjason C.W., Tsang Dominic N.C.
EPI_ISL_444456	B.J. Medical College and Civil hospital	Gujarat Biotechnology Research Centre	R D Dixit, Snehal Bagatharia, Kamlesh J Upadhyay, Ramesh Pandit, Tejas Shah, Ankit Hinsu, Pritesh Sabara, Apurvasinh Puvar, Janvi Raval, Monika Gandhi, Pinal Trivedi, Maharshi Pandya, Amit Kanani, Akanksha Verma, Nitin Savaliya, Raghawendra Kumar, Dinesh Kumar, Zuber Saiyed, Dipa Kinariwala, Disha Patel, Binita Aring, Neeta Khandelwal, Geeta Vaghela, Sonia Barve, Bhavesh Modi, Kairavi Joshi, Gaurishankar Shrimali, Nidhi Sood, Pranay Shah, Pooja P Doshi, Chaitanya Joshi, Madhvi Joshi
EPI_ISL_444457	B.J. Medical College and Civil hospital	Gujarat Biotechnology Research Centre	Snehal Bagatharia, Kamlesh J Upadhyay, Ramesh Pandit, Tejas Shah, Ankit Hinsu, Pritesh Sabara, Apurvasinh Puvar, Janvi Raval, Monika Gandhi, Pinal Trivedi, Maharshi Pandya, Amit Kanani, Akanksha Verma, Nitin Savaliya, Raghawendra Kumar, Dinesh Kumar, Zuber Saiyed, Dipa Kinariwala, Disha Patel, Binita Aring, Neeta Khandelwal, Geeta Vaghela, Sonia Barve, Bhavesh Modi, Kairavi Joshi, Gaurishankar Shrimali, Nidhi Sood, Pranay Shah, R D Dixit, Nidhi Patel, Chaitanya Joshi, Madhvi Joshi
EPI_ISL_444458	B.J. Medical College and Civil hospital	Gujarat Biotechnology Research Centre	Kamlesh J Upadhyay, Ramesh Pandit, Tejas Shah, Ankit Hinsu, Pritesh Sabara, Apurvasinh Puvar, Janvi Raval, Monika Gandhi, Pinal Trivedi, Maharshi Pandya, Amit Kanani, Akanksha Verma, Nitin Savaliya, Raghawendra Kumar, Dinesh Kumar, Zuber Saiyed, Dipa Kinariwala, Disha Patel, Binita Aring, Neeta Khandelwal, Geeta Vaghela, Sonia Barve, Bhavesh Modi, Kairavi Joshi, Gaurishankar Shrimali, Nidhi Sood, Pranay Shah, R D Dixit, Snehal Bagatharia, Priti Pandita, Chaitanya Joshi, Madhvi Joshi
EPI_ISL_444459	B.J. Medical College and Civil hospital	Gujarat Biotechnology Research Centre	Ramesh Pandit, Tejas Shah, Ankit Hinsu, Pritesh Sabara, Apurvasinh Puvar, Janvi Raval, Monika Gandhi, Pinal Trivedi, Maharshi Pandya, Amit Kanani, Akanksha Verma, Nitin Savaliya, Raghawendra Kumar, Dinesh Kumar, Zuber Saiyed, Dipa Kinariwala, Disha Patel, Binita Aring, Neeta Khandelwal, Geeta Vaghela, Sonia Barve, Bhavesh Modi, Kairavi Joshi, Gaurishankar Shrimali, Nidhi Sood, Pranay Shah, R D Dixit, Snehal Bagatharia, Kamlesh J Upadhyay, Neha Rajpara, Chaitanya Joshi, Madhvi Joshi
EPI_ISL_444460	B.J. Medical College and Civil hospital	Gujarat Biotechnology Research Centre	Tejas Shah, Ankit Hinsu, Pritesh Sabara, Apurvasinh Puvar, Janvi Raval, Monika Gandhi, Pinal Trivedi, Maharshi Pandya, Amit Kanani, Akanksha Verma, Nitin Savaliya, Raghawendra Kumar, Dinesh Kumar, Zuber Saiyed, Dipa Kinariwala, Disha Patel, Binita Aring, Neeta Khandelwal, Geeta Vaghela, Sonia Barve, Bhavesh Modi, Kairavi Joshi, Gaurishankar Shrimali, Nidhi Sood, Pranay Shah, R D Dixit, Snehal Bagatharia, Kamlesh J Upadhyay, Ramesh Pandit, Afzal Ansari, Chaitanya Joshi, Madhvi Joshi
EPI_ISL_444462	B.J. Medical College and Civil hospital	Gujarat Biotechnology Research Centre	Pritesh Sabara, Apurvasinh Puvar, Janvi Raval, Monika Gandhi, Pinal Trivedi, Maharshi Pandya, Amit Kanani, Akanksha Verma, Nitin Savaliya, Raghawendra Kumar, Dinesh Kumar, Zuber Saiyed, Dipa Kinariwala, Disha Patel, Binita Aring, Neeta Khandelwal, Geeta Vaghela, Sonia Barve, Bhavesh Modi, Kairavi Joshi, Gaurishankar Shrimali, Nidhi Sood, Pranay Shah, R D Dixit, Snehal Bagatharia, Kamlesh J Upadhyay, Ramesh Pandit, Tejas Shah, Ankit Hinsu, Armi Chaudhari, Chaitanya Joshi, Madhvi Joshi
EPI_ISL_444465	B.J. Medical College and Civil hospital	Gujarat Biotechnology Research Centre	Monika Gandhi, Pinal Trivedi, Maharshi Pandya, Amit Kanani, Akanksha Verma, Nitin Savaliya, Raghawendra Kumar, Dinesh Kumar, Zuber Saiyed, Dipa Kinariwala, Disha Patel, Binita Aring, Neeta Khandelwal, Geeta Vaghela, Sonia Barve, Bhavesh Modi, Kairavi Joshi, Gaurishankar Shrimali, Nidhi Sood, Pranay Shah, R D Dixit, Snehal Bagatharia, Kamlesh J Upadhyay, Ramesh Pandit, Tejas Shah, Ankit Hinsu, Pritesh Sabara, Apurvasinh Puvar, Janvi Raval, Anjali Rajwar, Chaitanya Joshi, Madhvi Joshi
EPI_ISL_444466	B.J. Medical College and Civil hospital	Gujarat Biotechnology Research Centre	Pinal Trivedi, Maharshi Pandya, Amit Kanani, Akanksha Verma, Nitin Savaliya, Raghawendra Kumar, Dinesh Kumar, Zuber Saiyed, Dipa Kinariwala, Disha Patel, Binita Aring, Neeta Khandelwal, Geeta Vaghela, Sonia Barve, Bhavesh Modi, Kairavi Joshi, Gaurishankar Shrimali, Nidhi Sood, Pranay Shah, R D Dixit, Snehal Bagatharia, Kamlesh J Upadhyay, Ramesh Pandit, Tejas Shah, Ankit Hinsu, Pritesh Sabara, Apurvasinh Puvar, Janvi Raval, Monika Gandhi, Sharmistha Majumdar, Chaitanya Joshi, Madhvi Joshi
EPI_ISL_444468	B.J. Medical College and Civil hospital	Gujarat Biotechnology Research Centre	Amit Kanani, Akanksha Verma, Nitin Savaliya, Raghawendra Kumar, Dinesh Kumar, Zuber Saiyed, Dipa Kinariwala, Disha Patel, Binita Aring, Neeta Khandelwal, Geeta Vaghela, Sonia Barve, Bhavesh Modi, Kairavi Joshi, Gaurishankar Shrimali, Nidhi Sood, Pranay Shah, R D Dixit, Snehal Bagatharia, Kamlesh J Upadhyay, Ramesh Pandit, Tejas Shah, Ankit Hinsu, Pritesh Sabara, Apurvasinh Puvar, Janvi Raval, Monika Gandhi, Pinal Trivedi, Maharshi Pandya, Nidhi Patel, Chaitanya Joshi, Madhvi Joshi
EPI_ISL_444469	B.J. Medical College and Civil hospital	Gujarat Biotechnology Research Centre	Akanksha Verma, Nitin Savaliya, Raghawendra Kumar, Dinesh Kumar, Zuber Saiyed, Dipa Kinariwala, Disha Patel, Binita Aring, Neeta Khandelwal, Geeta Vaghela, Sonia Barve, Bhavesh Modi, Kairavi Joshi, Gaurishankar Shrimali, Nidhi Sood, Pranay Shah, R D Dixit, Snehal Bagatharia, Kamlesh J Upadhyay, Ramesh Pandit, Tejas Shah, Ankit Hinsu, Pritesh Sabara, Apurvasinh Puvar, Janvi Raval, Monika Gandhi, Pinal Trivedi, Maharshi Pandya, Amit Kanani, Priti Pandita, Chaitanya Joshi, Madhvi Joshi
EPI_ISL_444471	B.J. Medical College and Civil hospital	Gujarat Biotechnology Research Centre	Raghawendra Kumar, Dinesh Kumar, Zuber Saiyed, Dipa Kinariwala, Disha Patel, Binita Aring, Neeta Khandelwal, Geeta Vaghela, Sonia Barve, Bhavesh Modi,

			Kairavi Joshi, Gaurishankar Shrimali, Nidhi Sood, Pranay Shah, R D Dixit, Snehal Bagatharia, Kamlesh J Upadhyay, Ramesh Pandit, Tejas Shah, Ankit Hinsu, Pritesh Sabara, Apurvasinh Puvar, Janvi Raval, Monika Gandhi, Pinal Trivedi, Maharshi Pandya, Amit Kanani, Akanksha Verma, Nitin Savaliya, Afzal Ansari, Chaitanya Joshi, Madhvi Joshi
EPI_ISL_444474	B.J. Medical College and Civil hospital	Gujarat Biotechnology Research Centre	Dipa Kinariwala, Disha Patel, Binita Aring, Neeta Khandelwal, Geeta Vaghela, Sonia Barve, Bhavesh Modi, Kairavi Joshi, Gaurishankar Shrimali, Nidhi Sood, Pranay Shah, R D Dixit, Snehal Bagatharia, Kamlesh J Upadhyay, Ramesh Pandit, Tejas Shah, Ankit Hinsu, Pritesh Sabara, Apurvasinh Puvar, Janvi Raval, Monika Gandhi, Pinal Trivedi, Maharshi Pandya, Amit Kanani, Akanksha Verma, Nitin Savaliya, Raghawendra Kumar, Dinesh Kumar, Zuber Saiyed, Bhavya Jindal, Chaitanya Joshi, Madhvi Joshi
EPI_ISL_444475	B.J. Medical College and Civil hospital	Gujarat Biotechnology Research Centre	Disha Patel, Binita Aring, Neeta Khandelwal, Geeta Vaghela, Sonia Barve, Bhavesh Modi, Kairavi Joshi, Gaurishankar Shrimali, Nidhi Sood, Pranay Shah, R D Dixit, Snehal Bagatharia, Kamlesh J Upadhyay, Ramesh Pandit, Tejas Shah, Ankit Hinsu, Pritesh Sabara, Apurvasinh Puvar, Janvi Raval, Monika Gandhi, Pinal Trivedi, Maharshi Pandya, Amit Kanani, Akanksha Verma, Nitin Savaliya, Raghawendra Kumar, Dinesh Kumar, Zuber Saiyed, Dipa Kinariwala, Dipeshwari Shewale, Chaitanya Joshi, Madhvi Joshi
EPI_ISL_444476	B.J. Medical College and Civil hospital	Gujarat Biotechnology Research Centre	Binita Aring, Neeta Khandelwal, Geeta Vaghela, Sonia Barve, Bhavesh Modi, Kairavi Joshi, Gaurishankar Shrimali, Nidhi Sood, Pranay Shah, R D Dixit, Snehal Bagatharia, Kamlesh J Upadhyay, Ramesh Pandit, Tejas Shah, Ankit Hinsu, Pritesh Sabara, Apurvasinh Puvar, Janvi Raval, Monika Gandhi, Pinal Trivedi, Maharshi Pandya, Amit Kanani, Akanksha Verma, Nitin Savaliya, Raghawendra Kumar, Dinesh Kumar, Zuber Saiyed, Dipa Kinariwala, Disha Patel, Chaitanya Joshi, Madhvi Joshi, Dipeshwari Shewale
EPI_ISL_444477	B.J. Medical College and Civil hospital	Gujarat Biotechnology Research Centre	Neeta Khandelwal, Geeta Vaghela, Sonia Barve, Bhavesh Modi, Kairavi Joshi, Gaurishankar Shrimali, Nidhi Sood, Pranay Shah, R D Dixit, Snehal Bagatharia, Kamlesh J Upadhyay, Ramesh Pandit, Tejas Shah, Ankit Hinsu, Pritesh Sabara, Apurvasinh Puvar, Janvi Raval, Monika Gandhi, Pinal Trivedi, Maharshi Pandya, Amit Kanani, Akanksha Verma, Nitin Savaliya, Raghawendra Kumar, Dinesh Kumar, Zuber Saiyed, Dipa Kinariwala, Disha Patel, Binita Aring, Sharmistha Majumdar, Chaitanya Joshi, Madhvi Joshi
EPI_ISL_444480	B.J. Medical College and Civil hospital	Gujarat Biotechnology Research Centre	Bhavesh Modi, Kairavi Joshi, Gaurishankar Shrimali, Nidhi Sood, Pranay Shah, R D Dixit, Snehal Bagatharia, Kamlesh J Upadhyay, Ramesh Pandit, Tejas Shah, Ankit Hinsu, Pritesh Sabara, Apurvasinh Puvar, Janvi Raval, Monika Gandhi, Pinal Trivedi, Maharshi Pandya, Amit Kanani, Akanksha Verma, Nitin Savaliya, Raghawendra Kumar, Dinesh Kumar, Zuber Saiyed, Dipa Kinariwala, Disha Patel, Binita Aring, Neeta Khandelwal, Geeta Vaghela, Sonia Barve, Priti Pandita, Chaitanya Joshi, Madhvi Joshi
EPI_ISL_444481	B.J. Medical College and Civil hospital	Gujarat Biotechnology Research Centre	Kairavi Joshi, Gaurishankar Shrimali, Nidhi Sood, Pranay Shah, R D Dixit, Snehal Bagatharia, Kamlesh J Upadhyay, Ramesh Pandit, Tejas Shah, Ankit Hinsu, Pritesh Sabara, Apurvasinh Puvar, Janvi Raval, Monika Gandhi, Pinal Trivedi, Maharshi Pandya, Amit Kanani, Akanksha Verma, Nitin Savaliya, Raghawendra Kumar, Dinesh Kumar, Zuber Saiyed, Dipa Kinariwala, Disha Patel, Binita Aring, Neeta Khandelwal, Geeta Vaghela, Sonia Barve, Bhavesh Modi, Neha Rajpara, Chaitanya Joshi, Madhvi Joshi
EPI_ISL_444482	Gujarat Biotechnology Research Centre	Gujarat Biotechnology Research Centre	Gaurishankar Shrimali, Nidhi Sood, Pranay Shah, R D Dixit, Snehal Bagatharia, Kamlesh J Upadhyay, Ramesh Pandit, Tejas Shah, Ankit Hinsu, Pritesh Sabara, Apurvasinh Puvar, Janvi Raval, Monika Gandhi, Pinal Trivedi, Maharshi Pandya, Amit Kanani, Akanksha Verma, Nitin Savaliya, Raghawendra Kumar, Dinesh Kumar, Zuber Saiyed, Dipa Kinariwala, Disha Patel, Binita Aring, Neeta Khandelwal, Geeta Vaghela, Sonia Barve, Bhavesh Modi, Kairavi Joshi, Afzal Ansari, Chaitanya Joshi, Madhvi Joshi
EPI_ISL_444483	Gujarat Biotechnology Research Centre	Gujarat Biotechnology Research Centre	Nidhi Sood, Pranay Shah, R D Dixit, Snehal Bagatharia, Kamlesh J Upadhyay, Ramesh Pandit, Tejas Shah, Ankit Hinsu, Pritesh Sabara, Apurvasinh Puvar, Janvi Raval, Monika Gandhi, Pinal Trivedi, Maharshi Pandya, Amit Kanani, Akanksha Verma, Nitin Savaliya, Raghawendra Kumar, Dinesh Kumar, Zuber Saiyed, Dipa Kinariwala, Disha Patel, Binita Aring, Neeta Khandelwal, Geeta Vaghela, Sonia Barve, Bhavesh Modi, Kairavi Joshi, Gaurishankar Shrimali, Neelam Nathani, Chaitanya Joshi, Madhvi Joshi
EPI_ISL_444484	Gujarat Biotechnology Research Centre	Gujarat Biotechnology Research Centre	Pranay Shah, R D Dixit, Snehal Bagatharia, Kamlesh J Upadhyay, Ramesh Pandit, Tejas Shah, Ankit Hinsu, Pritesh Sabara, Apurvasinh Puvar, Janvi Raval, Monika Gandhi, Pinal Trivedi, Maharshi Pandya, Amit Kanani, Akanksha Verma, Nitin Savaliya, Raghawendra Kumar, Dinesh Kumar, Zuber Saiyed, Dipa Kinariwala, Disha Patel, Binita Aring, Neeta Khandelwal, Geeta Vaghela, Sonia Barve, Bhavesh Modi, Kairavi Joshi, Gaurishankar Shrimali, Nidhi Sood, Armi Chaudhari, Chaitanya Joshi, Madhvi Joshi
EPI_ISL_444486	Gujarat Biotechnology Research Centre	Gujarat Biotechnology Research Centre	Snehal Bagatharia, Kamlesh J Upadhyay, Ramesh Pandit, Tejas Shah, Ankit Hinsu, Pritesh Sabara, Apurvasinh Puvar, Janvi Raval, Monika Gandhi, Pinal Trivedi, Maharshi Pandya, Amit Kanani, Akanksha Verma, Nitin Savaliya, Raghawendra Kumar, Dinesh Kumar, Zuber Saiyed, Dipa Kinariwala, Disha Patel, Binita Aring, Neeta Khandelwal, Geeta Vaghela, Sonia Barve, Bhavesh Modi, Kairavi Joshi, Gaurishankar Shrimali, Nidhi Sood, Pranay Shah, R D Dixit, Dipeshwari Shewale, Chaitanya Joshi, Madhvi Joshi
EPI_ISL_444493	Departamento de Laboratorios de Salud Publica (DLSP, Division Epidemiologia, Ministerio de Salud Publica)	Facultad de Ciencias (Sección Genética Evolutiva, Sección Virología).	Panzera,Y., Delfraro,A., Ramos,N., Frabasile,S., Calleros,L., Techera,C., Grecco,S., Fuques,E., Goni,N., Coppola,L., Ramos,V., Chiparelli,H., Arbiza,J. and Perez,R.
EPI_ISL_444969	Guangzhou Eighth People's Hospital (Jiahe Sector)	Institute of Human Virology, Zhongshan School of Medicine, Sun Yat-sen University	Junsong Zhang, Fei Yu, Jun Liu, Huimin Fan, Ruosu Ying, Feng Huang, Ting Pan, Bingfeng Liu,Yiwen Zhang, Xu Zhang, Mang Shi, Fengyu Hu, Fang Li, Kai Deng, Hui Zhang
EPI_ISL_444994, EPI_ISL_444995, EPI_ISL_444996, EPI_ISL_444998, EPI_ISL_445000	Naval Health Research Center	Naval Medical Research Center Biological Defense Research Directorate	Logan Voegtly, Regina Cer, Dessiree Pena-Gomez, Adrian Paskey,Kyle Long, Roger Pan, Melinda Balansay-Ames, Chris Myers, Ewell Hollis, Nathaniel Christy, Kimberly Bishop-Lilly
EPI_ISL_445078, EPI_ISL_445079, EPI_ISL_445080, EPI_ISL_445081, EPI_ISL_445082, EPI_ISL_445083, EPI_ISL_445084	Baylor College of Medicine	Baylor College of Medicine: HGSC	Vasanthi Avadhanula, Erin Nicholson, David Henke, Pedro Piedra, Harsha Doddapaneni, Donna Muzny, Qingchang Meng, Hsu Chao, Zeineen Momin, Hua Shen, George Weissenberger, Kavya Kottapalli, Yimiti Meiheerguli, Sejal Salvi, Ginger Metcalf, Vipin Menon, Sara J.J. Cregeen, Matthew C. Ross, Tulin Ayvaz, Richard Sucgang, Kristi L. Hoffman, Matthew Wong, Joseph F. Petrosino
EPI_ISL_445214, EPI_ISL_445215, EPI_ISL_445216	DNA Solution Ltd.	DNA Solution Ltd.	Md. Imran Khan, Kazi Nadim Hasan, Abu Sufian, Mohammed Nafiz Imtiaz Polol, Abdul Khaleque, Mizanur Rahman, MSM Chowdhury, Hasan Ul Haider, Mamudul Hasan Razu, Mala Khan, Mohammad Fazle Alam Rabbi
EPI_ISL_445219	Universidad del Valle, Laboratorio de Microbiologia, VIREM	Universidad del Valle, Universidad Nacional de Colombia-Sede Palmira, International Center for Tropical Agriculture	Beatriz Parra, Diana López-Alvarez, Wilmer J. Cuellar
EPI_ISL_445220	Laborarory for Respiratory Viruses, "Cantacuzino" National Military-Medical Institute for Resararch and Development	Cantacuzino Institute	M.Lazar, L.Ustea, A.Cretu
EPI_ISL_445243	Laboratory for Respiratory Viruses, Cantacuzino National Military-Medical Institute for Research and Development	Cantacuzino Institute	M.Lazar, L.Ustea, A.Cretu
EPI_ISL_447030	B.J. Medical College and Civil hospital	Gujarat Biotechnology Research Centre	Kamlesh J Upadhyay, Ramesh Pandit, Tejas Shah, Ankit Hinsu, Pritesh Sabara, Apurvasinh Puvar, Janvi Raval, Monika Gandhi, Pinal Trivedi, Maharshi Pandya, Amit Kanani, Akanksha Verma, Nitin Savaliya, Raghawendra Kumar, Dinesh Kumar, Zuber Saiyed, Dipa Kinariwala, Disha Patel, Binita Aring, Neeta Khandelwal, Geeta Vaghela, Sonia Barve, Bhavesh Modi, Kairavi Joshi, Gaurishankar Shrimali, Nidhi Sood, Pranay Shah, R D Dixit, Snehal Bagatharia, Anjali Rajwar, Chaitanya Joshi, Madhvi Joshi
EPI_ISL_447031	B.J. Medical College and Civil hospital	Gujarat Biotechnology Research Centre	Ramesh Pandit, Tejas Shah, Ankit Hinsu, Pritesh Sabara, Apurvasinh Puvar, Janvi Raval, Monika Gandhi, Pinal Trivedi, Maharshi Pandya, Amit Kanani, Akanksha Verma, Nitin Savaliya, Raghawendra Kumar, Dinesh Kumar, Zuber Saiyed, Dipa Kinariwala, Disha Patel, Binita Aring, Neeta Khandelwal, Geeta Vaghela, Sonia Barve, Bhavesh Modi, Kairavi Joshi, Gaurishankar Shrimali, Nidhi Sood, Pranay Shah, R D Dixit, Snehal Bagatharia, Kamlesh J Upadhyay, Sharmistha Majumdar, Chaitanya Joshi, Madhvi Joshi
EPI_ISL_447033	B.J. Medical College and Civil hospital	Gujarat Biotechnology Research Centre	Ankit Hinsu, Pritesh Sabara, Apurvasinh Puvar, Janvi Raval, Monika Gandhi, Pinal Trivedi, Maharshi Pandya, Amit Kanani, Akanksha Verma, Nitin Savaliya, Raghawendra Kumar, Dinesh Kumar, Zuber Saiyed, Dipa Kinariwala, Disha Patel, Binita Aring, Neeta Khandelwal, Geeta Vaghela, Sonia Barve, Bhavesh Modi, Kairavi Joshi, Gaurishankar Shrimali, Nidhi Sood, Pranay Shah, R D Dixit, Snehal Bagatharia, Kamlesh J Upadhyay, Ramesh Pandit, Tejas Shah, Nidhi Patel, Chaitanya Joshi, Madhvi Joshi
EPI_ISL_447034	B.J. Medical College and Civil hospital	Gujarat Biotechnology Research Centre	Pritesh Sabara, Apurvasinh Puvar, Janvi Raval, Monika Gandhi, Pinal Trivedi, Maharshi Pandya, Amit Kanani, Akanksha Verma, Nitin Savaliya, Raghawendra

			Kumar, Dinesh Kumar, Zuber Saiyed, Dipa Kinariwala, Disha Patel, Binita Aring, Neeta Khandelwal, Geeta Vaghela, Sonia Barve, Bhavesh Modi, Kairavi Joshi, Gaurishankar Shrimali, Nidhi Sood, Pranay Shah, R D Dixit, Snehal Bagatharia, Kamlesh J Upadhyay, Ramesh Pandit, Tejas Shah, Ankit Hinsu, Priti Pandita, Chaitanya Joshi, Madhvi Joshi
EPI_ISL_447035	B.J. Medical College and Civil hospital	Gujarat Biotechnology Research Centre	Apurvasinh Puvar, Janvi Raval, Monika Gandhi, Pinal Trivedi, Maharshi Pandya, Amit Kanani, Akanksha Verma, Nitin Savaliya, Raghawendra Kumar, Dinesh Kumar, Zuber Saiyed, Dipa Kinariwala, Disha Patel, Binita Aring, Neeta Khandelwal, Geeta Vaghela, Sonia Barve, Bhavesh Modi, Kairavi Joshi, Gaurishankar Shrimali, Nidhi Sood, Pranay Shah, R D Dixit, Snehal Bagatharia, Kamlesh J Upadhyay, Ramesh Pandit, Tejas Shah, Ankit Hinsu, Pritesh Sabara, Neha Rajpara, Chaitanya Joshi, Madhvi Joshi
EPI_ISL_447038	B.J. Medical College and Civil hospital	Gujarat Biotechnology Research Centre	Pinal Trivedi, Maharshi Pandya, Amit Kanani, Akanksha Verma, Nitin Savaliya, Raghawendra Kumar, Dinesh Kumar, Zuber Saiyed, Dipa Kinariwala, Disha Patel, Binita Aring, Neeta Khandelwal, Geeta Vaghela, Sonia Barve, Bhavesh Modi, Kairavi Joshi, Gaurishankar Shrimali, Nidhi Sood, Pranay Shah, R D Dixit, Snehal Bagatharia, Kamlesh J Upadhyay, Ramesh Pandit, Tejas Shah, Ankit Hinsu, Pritesh Sabara, Apurvasinh Puvar, Janvi Raval, Monika Gandhi, Armi Chaudhari, Chaitanya Joshi, Madhvi Joshi
EPI_ISL_447039	B.J. Medical College and Civil hospital	Gujarat Biotechnology Research Centre	Maharshi Pandya, Amit Kanani, Akanksha Verma, Nitin Savaliya, Raghawendra Kumar, Dinesh Kumar, Zuber Saiyed, Dipa Kinariwala, Disha Patel, Binita Aring, Neeta Khandelwal, Geeta Vaghela, Sonia Barve, Bhavesh Modi, Kairavi Joshi, Gaurishankar Shrimali, Nidhi Sood, Pranay Shah, R D Dixit, Snehal Bagatharia, Kamlesh J Upadhyay, Ramesh Pandit, Tejas Shah, Ankit Hinsu, Pritesh Sabara, Apurvasinh Puvar, Janvi Raval, Monika Gandhi, Pinal Trivedi, Bhavya Jindal, Chaitanya Joshi, Madhvi Joshi
EPI_ISL_447042	B.J. Medical College and Civil hospital	Gujarat Biotechnology Research Centre	Nitin Savaliya, Raghawendra Kumar, Dinesh Kumar, Zuber Saiyed, Dipa Kinariwala, Disha Patel, Binita Aring, Neeta Khandelwal, Geeta Vaghela, Sonia Barve, Bhavesh Modi, Kairavi Joshi, Gaurishankar Shrimali, Nidhi Sood, Pranay Shah, R D Dixit, Snehal Bagatharia, Kamlesh J Upadhyay, Ramesh Pandit, Tejas Shah, Ankit Hinsu, Pritesh Sabara, Apurvasinh Puvar, Janvi Raval, Monika Gandhi, Pinal Trivedi, Maharshi Pandya, Amit Kanani, Akanksha Verma, Pooja P Doshi, Chaitanya Joshi, Madhvi Joshi
EPI_ISL_447043	B.J. Medical College and Civil hospital	Gujarat Biotechnology Research Centre	Raghawendra Kumar, Dinesh Kumar, Zuber Saiyed, Dipa Kinariwala, Disha Patel, Binita Aring, Neeta Khandelwal, Geeta Vaghela, Sonia Barve, Bhavesh Modi, Kairavi Joshi, Gaurishankar Shrimali, Nidhi Sood, Pranay Shah, R D Dixit, Snehal Bagatharia, Kamlesh J Upadhyay, Ramesh Pandit, Tejas Shah, Ankit Hinsu, Pritesh Sabara, Apurvasinh Puvar, Janvi Raval, Monika Gandhi, Pinal Trivedi, Maharshi Pandya, Amit Kanani, Akanksha Verma, Nitin Savaliya, Nidhi Patel, Chaitanya Joshi, Madhvi Joshi
EPI_ISL_447044	B.J. Medical College and Civil hospital	Gujarat Biotechnology Research Centre	Dinesh Kumar, Zuber Saiyed, Dipa Kinariwala, Disha Patel, Binita Aring, Neeta Khandelwal, Geeta Vaghela, Sonia Barve, Bhavesh Modi, Kairavi Joshi, Gaurishankar Shrimali, Nidhi Sood, Pranay Shah, R D Dixit, Snehal Bagatharia, Kamlesh J Upadhyay, Ramesh Pandit, Tejas Shah, Ankit Hinsu, Pritesh Sabara, Apurvasinh Puvar, Janvi Raval, Monika Gandhi, Pinal Trivedi, Maharshi Pandya, Amit Kanani, Akanksha Verma, Nitin Savaliya, Raghawendra Kumar, Priti Pandita, Chaitanya Joshi, Madhvi Joshi
EPI_ISL_447045	B.J. Medical College and Civil hospital	Gujarat Biotechnology Research Centre	Zuber Saiyed, Dipa Kinariwala, Disha Patel, Binita Aring, Neeta Khandelwal, Geeta Vaghela, Sonia Barve, Bhavesh Modi, Kairavi Joshi, Gaurishankar Shrimali, Nidhi Sood, Pranay Shah, R D Dixit, Snehal Bagatharia, Kamlesh J Upadhyay, Ramesh Pandit, Tejas Shah, Ankit Hinsu, Pritesh Sabara, Apurvasinh Puvar, Janvi Raval, Monika Gandhi, Pinal Trivedi, Maharshi Pandya, Amit Kanani, Akanksha Verma, Nitin Savaliya, Raghawendra Kumar, Dinesh Kumar, Neha Rajpara, Chaitanya Joshi, Madhvi Joshi
EPI_ISL_447048	GMERS Medical College and Hospital, Gandhinagar	Gujarat Biotechnology Research Centre	Binita Aring, Neeta Khandelwal, Geeta Vaghela, Sonia Barve, Bhavesh Modi, Kairavi Joshi, Gaurishankar Shrimali, Nidhi Sood, Pranay Shah, R D Dixit, Snehal Bagatharia, Kamlesh J Upadhyay, Ramesh Pandit, Tejas Shah, Ankit Hinsu, Pritesh Sabara, Apurvasinh Puvar, Janvi Raval, Monika Gandhi, Pinal Trivedi, Maharshi Pandya, Amit Kanani, Akanksha Verma, Nitin Savaliya, Raghawendra Kumar, Dinesh Kumar, Zuber Saiyed, Dipa Kinariwala, Disha Patel, Armi Chaudhari, Chaitanya Joshi, Madhvi Joshi
EPI_ISL_447053	GMERS Medical College and Hospital, Gandhinagar	Gujarat Biotechnology Research Centre	Kairavi Joshi, Gaurishankar Shrimali, Nidhi Sood, Pranay Shah, R D Dixit, Snehal Bagatharia, Kamlesh J Upadhyay, Ramesh Pandit, Tejas Shah, Ankit Hinsu, Pritesh Sabara, Apurvasinh Puvar, Janvi Raval, Monika Gandhi, Pinal Trivedi, Maharshi Pandya, Amit Kanani, Akanksha Verma, Nitin Savaliya, Raghawendra Kumar, Dinesh Kumar, Zuber Saiyed, Dipa Kinariwala, Disha Patel, Binita Aring, Neeta Khandelwal, Geeta Vaghela, Sonia Barve, Bhavesh Modi, Pooja P Doshi, Chaitanya Joshi, Madhvi Joshi
EPI_ISL_447054	Cantacuzino National Military-Medical Institute for Research and Development	Cantacuzino Institute	M.Lazar, L.Ustea, A.Cretu
EPI_ISL_447251	Central Virology Laboratory	Central Virology Laboratory	Neta Zuckerman, Efrat Bucris, Oran Erster, Danit Sofer, Orna Mor, Ella Mendelson, Michal Mandelboim
EPI_ISL_447252, EPI_ISL_447253, EPI_ISL_447255, EPI_ISL_447257	TSGH-CP molecular lab	TSGH-CP molecular lab	Cherng-Lih Perng, Ming-Jr JIAN, Chih-Kai Chang, Jung-Chung Lin, Kuo-Ming Yeh, Chien-Wen Chen, Sheng-Kang Chiu, Hsing-Yi Chung, Shih-Hung Tsai, Kuo-Sheng Hung, Tien-Yao Chang, Feng-Yee Chang, Hung-Sheng Shang
EPI_ISL_447281, EPI_ISL_447282, EPI_ISL_447283, EPI_ISL_447284, EPI_ISL_447285, EPI_ISL_447286, EPI_ISL_447287, EPI_ISL_447288, EPI_ISL_447289, EPI_ISL_447290, EPI_ISL_447291, EPI_ISL_447292, EPI_ISL_447293, EPI_ISL_447294, EPI_ISL_447295, EPI_ISL_447296, EPI_ISL_447297, EPI_ISL_447299, EPI_ISL_447300, EPI_ISL_447301, EPI_ISL_447302, EPI_ISL_447303, EPI_ISL_447305, EPI_ISL_447306, EPI_ISL_447307, EPI_ISL_447308, EPI_ISL_447309, EPI_ISL_447310			
see above	Microbiology Division, Barzilai University Medical Center	Stern Lab	Stern Lab
EPI_ISL_447534	Gujarat Biotechnology Research Centre	Gujarat Biotechnology Research Centre	Gaurishankar Shrimali, Nidhi Sood, Pranay Shah, R D Dixit, Snehal Bagatharia, Kamlesh J Upadhyay, Ramesh Pandit, Tejas Shah, Ankit Hinsu, Pritesh Sabara, Apurvasinh Puvar, Janvi Raval, Monika Gandhi, Pinal Trivedi, Maharshi Pandya, Amit Kanani, Akanksha Verma, Nitin Savaliya, Raghawendra Kumar, Dinesh Kumar, Zuber Saiyed, Dipa Kinariwala, Disha Patel, Binita Aring, Neeta Khandelwal, Geeta Vaghela, Sonia Barve, Bhavesh Modi, Kairavi Joshi, Nidhi Patel, Chaitanya Joshi, Madhvi Joshi
EPI_ISL_447535	Gujarat Biotechnology Research Centre	Gujarat Biotechnology Research Centre	Nidhi Sood, Pranay Shah, R D Dixit, Snehal Bagatharia, Kamlesh J Upadhyay, Ramesh Pandit, Tejas Shah, Ankit Hinsu, Pritesh Sabara, Apurvasinh Puvar, Janvi Raval, Monika Gandhi, Pinal Trivedi, Maharshi Pandya, Amit Kanani, Akanksha Verma, Nitin Savaliya, Raghawendra Kumar, Dinesh Kumar, Zuber Saiyed, Dipa Kinariwala, Disha Patel, Binita Aring, Neeta Khandelwal, Geeta Vaghela, Sonia Barve, Bhavesh Modi, Kairavi Joshi, Gaurishankar Shrimali, Priti Pandita, Chaitanya Joshi, Madhvi Joshi
EPI_ISL_447536	Gujarat Biotechnology Research Centre	Gujarat Biotechnology Research Centre	Pranay Shah, R D Dixit, Snehal Bagatharia, Kamlesh J Upadhyay, Ramesh Pandit, Tejas Shah, Ankit Hinsu, Pritesh Sabara, Apurvasinh Puvar, Janvi Raval, Monika Gandhi, Pinal Trivedi, Maharshi Pandya, Amit Kanani, Akanksha Verma, Nitin Savaliya, Raghawendra Kumar, Dinesh Kumar, Zuber Saiyed, Dipa Kinariwala, Disha Patel, Binita Aring, Neeta Khandelwal, Geeta Vaghela, Sonia Barve, Bhavesh Modi, Kairavi Joshi, Gaurishankar Shrimali, Nidhi Sood, Neha Rajpara, Chaitanya Joshi, Madhvi Joshi
EPI_ISL_447537	Gujarat Biotechnology Research Centre	Gujarat Biotechnology Research Centre	R D Dixit, Snehal Bagatharia, Kamlesh J Upadhyay, Ramesh Pandit, Tejas Shah, Ankit Hinsu, Pritesh Sabara, Apurvasinh Puvar, Janvi Raval, Monika Gandhi, Pinal Trivedi, Maharshi Pandya, Amit Kanani, Akanksha Verma, Nitin Savaliya, Raghawendra Kumar, Dinesh Kumar, Zuber Saiyed, Dipa Kinariwala, Disha Patel, Binita Aring, Neeta Khandelwal, Geeta Vaghela, Sonia Barve, Bhavesh Modi, Kairavi Joshi, Gaurishankar Shrimali, Nidhi Sood, Pranay Shah, Afzal Ansari, Chaitanya Joshi, Madhvi Joshi
EPI_ISL_447542	Gujarat Biotechnology Research Centre	Gujarat Biotechnology Research Centre	Ankit Hinsu, Pritesh Sabara, Apurvasinh Puvar, Janvi Raval, Monika Gandhi, Pinal Trivedi, Maharshi Pandya, Amit Kanani, Akanksha Verma, Nitin Savaliya, Raghawendra Kumar, Dinesh Kumar, Zuber Saiyed, Dipa Kinariwala, Disha Patel, Binita Aring, Neeta Khandelwal, Geeta Vaghela, Sonia Barve, Bhavesh Modi, Kairavi Joshi, Gaurishankar Shrimali, Nidhi Sood, Pranay Shah, R D Dixit, Snehal Bagatharia, Kamlesh J Upadhyay, Ramesh Pandit, Tejas Shah, Dipeshwari Shewale, Chaitanya Joshi, Madhvi Joshi
EPI_ISL_447543	Gujarat Biotechnology Research Centre	Gujarat Biotechnology Research Centre	Pritesh Sabara, Apurvasinh Puvar, Janvi Raval, Monika Gandhi, Pinal Trivedi, Maharshi Pandya, Amit Kanani, Akanksha Verma, Nitin Savaliya, Raghawendra Kumar, Dinesh Kumar, Zuber Saiyed, Dipa Kinariwala, Disha Patel, Binita Aring, Neeta Khandelwal, Geeta Vaghela, Sonia Barve, Bhavesh Modi, Kairavi Joshi, Gaurishankar Shrimali, Nidhi Sood, Pranay Shah, R D Dixit, Snehal Bagatharia, Kamlesh J Upadhyay, Ramesh Pandit, Tejas Shah, Ankit Hinsu, Sharmistha Majumdar, Chaitanya Joshi, Madhvi Joshi
EPI_ISL_447544	Gujarat Biotechnology Research Centre	Gujarat Biotechnology Research Centre	Apurvasinh Puvar, Janvi Raval, Monika Gandhi, Pinal Trivedi, Maharshi Pandya, Amit Kanani, Akanksha Verma, Nitin Savaliya, Raghawendra Kumar, Dinesh Kumar, Zuber Saiyed, Dipa Kinariwala, Disha Patel, Binita Aring, Neeta Khandelwal, Geeta Vaghela, Sonia Barve, Bhavesh Modi, Kairavi Joshi, Gaurishankar Shrimali, Nidhi Sood, Pranay Shah, R D Dixit, Snehal Bagatharia, Kamlesh J Upadhyay, Ramesh Pandit, Tejas Shah, Ankit Hinsu, Pritesh Sabara, Pooja P Doshi, Chaitanya Joshi, Madhvi Joshi
EPI_ISL_447545	Gujarat Biotechnology Research Centre	Gujarat Biotechnology Research Centre	Janvi Raval, Monika Gandhi, Pinal Trivedi, Maharshi Pandya, Amit Kanani, Akanksha Verma, Nitin Savaliya, Raghawendra Kumar, Dinesh Kumar, Zuber

			Saiyed, Dipa Kinariwala, Disha Patel, Binita Aring, Neeta Khandelwal, Geeta Vaghela, Sonia Barve, Bhavesh Modi, Kairavi Joshi, Gaurishankar Shrimali, Nidhi Sood, Pranay Shah, R D Dixit, Snehal Bagatharia, Kamlesh J Upadhyay, Ramesh Pandit, Tejas Shah, Ankit Hinsu, Pritesh Sabara, Apurvasinh Puvar, Nidhi Patel, Chaitanya Joshi, Madhvi Joshi
EPI_ISL_447546	Gujarat Biotechnology Research Centre	Gujarat Biotechnology Research Centre	Monika Gandhi, Pinal Trivedi, Maharshi Pandya, Amit Kanani, Akanksha Verma, Nitin Savaliya, Raghawendra Kumar, Dinesh Kumar, Zuber Saiyed, Dipa Kinariwala, Disha Patel, Binita Aring, Neeta Khandelwal, Geeta Vaghela, Sonia Barve, Bhavesh Modi, Kairavi Joshi, Gaurishankar Shrimali, Nidhi Sood, Pranay Shah, R D Dixit, Snehal Bagatharia, Kamlesh J Upadhyay, Ramesh Pandit, Tejas Shah, Ankit Hinsu, Pritesh Sabara, Apurvasinh Puvar, Janvi Raval, Priti Pandita, Chaitanya Joshi, Madhvi Joshi
EPI_ISL_447548	GMERS Medical College and Hospital, Gandhinagar	Gujarat Biotechnology Research Centre	Maharshi Pandya, Amit Kanani, Akanksha Verma, Nitin Savaliya, Raghawendra Kumar, Dinesh Kumar, Zuber Saiyed, Dipa Kinariwala, Disha Patel, Binita Aring, Neeta Khandelwal, Geeta Vaghela, Sonia Barve, Bhavesh Modi, Kairavi Joshi, Gaurishankar Shrimali, Nidhi Sood, Pranay Shah, R D Dixit, Snehal Bagatharia, Kamlesh J Upadhyay, Ramesh Pandit, Tejas Shah, Ankit Hinsu, Pritesh Sabara, Apurvasinh Puvar, Janvi Raval, Monika Gandhi, Pinal Trivedi, Afzal Ansari, Chaitanya Joshi, Madhvi Joshi
EPI_ISL_447551	GMERS Medical College and Hospital, Gandhinagar	Gujarat Biotechnology Research Centre	Nitin Savaliya, Raghawendra Kumar, Dinesh Kumar, Zuber Saiyed, Dipa Kinariwala, Disha Patel, Binita Aring, Neeta Khandelwal, Geeta Vaghela, Sonia Barve, Bhavesh Modi, Kairavi Joshi, Gaurishankar Shrimali, Nidhi Sood, Pranay Shah, R D Dixit, Snehal Bagatharia, Kamlesh J Upadhyay, Ramesh Pandit, Tejas Shah, Ankit Hinsu, Pritesh Sabara, Apurvasinh Puvar, Janvi Raval, Monika Gandhi, Pinal Trivedi, Maharshi Pandya, Amit Kanani, Akanksha Verma, Bhavya Jindal, Chaitanya Joshi, Madhvi Joshi
EPI_ISL_447552	GMERS Medical College and Hospital, Gandhinagar	Gujarat Biotechnology Research Centre	Raghawendra Kumar, Dinesh Kumar, Zuber Saiyed, Dipa Kinariwala, Disha Patel, Binita Aring, Neeta Khandelwal, Geeta Vaghela, Sonia Barve, Bhavesh Modi, Kairavi Joshi, Gaurishankar Shrimali, Nidhi Sood, Pranay Shah, R D Dixit, Snehal Bagatharia, Kamlesh J Upadhyay, Ramesh Pandit, Tejas Shah, Ankit Hinsu, Pritesh Sabara, Apurvasinh Puvar, Janvi Raval, Monika Gandhi, Pinal Trivedi, Maharshi Pandya, Amit Kanani, Akanksha Verma, Nitin Savaliya, Anjali Rajwar, Chaitanya Joshi, Madhvi Joshi
EPI_ISL_447553	GMERS Medical College and Hospital, Gandhinagar	Gujarat Biotechnology Research Centre	Dinesh Kumar, Zuber Saiyed, Dipa Kinariwala, Disha Patel, Binita Aring, Neeta Khandelwal, Geeta Vaghela, Sonia Barve, Bhavesh Modi, Kairavi Joshi, Gaurishankar Shrimali, Nidhi Sood, Pranay Shah, R D Dixit, Snehal Bagatharia, Kamlesh J Upadhyay, Ramesh Pandit, Tejas Shah, Ankit Hinsu, Pritesh Sabara, Apurvasinh Puvar, Janvi Raval, Monika Gandhi, Pinal Trivedi, Maharshi Pandya, Amit Kanani, Akanksha Verma, Nitin Savaliya, Raghawendra Kumar, Dipeshwari Shewale, Chaitanya Joshi, Madhvi Joshi
EPI_ISL_447554	GMERS Medical College and Hospital, Gandhinagar	Gujarat Biotechnology Research Centre	Zuber Saiyed, Dipa Kinariwala, Disha Patel, Binita Aring, Neeta Khandelwal, Geeta Vaghela, Sonia Barve, Bhavesh Modi, Kairavi Joshi, Gaurishankar Shrimali, Nidhi Sood, Pranay Shah, R D Dixit, Snehal Bagatharia, Kamlesh J Upadhyay, Ramesh Pandit, Tejas Shah, Ankit Hinsu, Pritesh Sabara, Apurvasinh Puvar, Janvi Raval, Monika Gandhi, Pinal Trivedi, Maharshi Pandya, Amit Kanani, Akanksha Verma, Nitin Savaliya, Raghawendra Kumar, Dinesh Kumar, Sharmistha Majumdar, Chaitanya Joshi, Madhvi Joshi
EPI_ISL_447555	GMERS Medical College and Hospital, Gandhinagar	Gujarat Biotechnology Research Centre	Dipa Kinariwala, Disha Patel, Binita Aring, Neeta Khandelwal, Geeta Vaghela, Sonia Barve, Bhavesh Modi, Kairavi Joshi, Gaurishankar Shrimali, Nidhi Sood, Pranay Shah, R D Dixit, Snehal Bagatharia, Kamlesh J Upadhyay, Ramesh Pandit, Tejas Shah, Ankit Hinsu, Pritesh Sabara, Apurvasinh Puvar, Janvi Raval, Monika Gandhi, Pinal Trivedi, Maharshi Pandya, Amit Kanani, Akanksha Verma, Nitin Savaliya, Raghawendra Kumar, Dinesh Kumar, Zuber Saiyed, Pooja P Doshi, Chaitanya Joshi, Madhvi Joshi
EPI_ISL_447577	CSIR-Centre for Cellular and Molecular Biology	CSIR-Centre for Cellular and Molecular Biology	Namami Gaur, Sakshi Shambhavi, Lamuk Zaveri, Shagufta Khan, Tulasi Nagabandi, Purushotham Vodnala, Payel Mukherjee, Sofia Banu, Priya Singh, Dhiviya Vedagiri, Divya Gupta, Vishal Sah, Santosh Kumar Kuncha, Krishnan Harinivas Harshan, Archana Bharadwaj Siva, Karthik Bharadwaj Tallapaka, Rakesh K Mishra, Divya Tej Sowpati
EPI_ISL_447592, EPI_ISL_447593	TSGH-CP molecular lab	TSGH-CP molecular lab	Cherng-Lih Perng, Ming-Jr JIAN, Chih-Kai Chang, Jung-Chung Lin, Kuo-Ming Yeh, Chien-Wen Chen, Sheng-Kang Chiu, Hsing-Yi Chung, Shih-Hung Tsai, Kuo-Sheng Hung, Tien-Yao Chang, Feng-Yee Chang, Hung-Sheng Shang
EPI_ISL_447614, EPI_ISL_447615, EPI_ISL_447616, EPI_ISL_447617, EPI_ISL_447618, EPI_ISL_447619, EPI_ISL_447620, EPI_ISL_447621, EPI_ISL_447622	Department of Laboratory Medicine, National Taiwan University Hospital	Microbial Genomics Core Lab, National Taiwan University Centers of Genomic and Precision Medicine	Shiou-Hwei Yeh, You-Yu Lin, Ya-Yun Lai, Chiao-Ling Li, Shan-Chwen Chang, Pei-Jer Chen, Sui-Yuan Chang
EPI_ISL_447734, EPI_ISL_447735, EPI_ISL_447736, EPI_ISL_447738, EPI_ISL_447739, EPI_ISL_447740, EPI_ISL_447741, EPI_ISL_447742, EPI_ISL_447743, EPI_ISL_447744, EPI_ISL_447745, EPI_ISL_447746, EPI_ISL_447747, EPI_ISL_447748, EPI_ISL_447750, EPI_ISL_447754			
see above	Grupo de Investigaciones Microbiológicas-UR (GIMUR), Departamento de Biología, Facultad de Ciencias Naturales, Universidad del Rosario, Bogotá, Colombia	Grupo de Investigaciones Microbiológicas-UR (GIMUR), Departamento de Biología, Facultad de Ciencias Naturales, Universidad del Rosario, Bogotá, Colombia Instituto Nacional de Salud, Bogotá, Colombia Icahn School of Medicine at Mount Sinai, New York, USA	Juan David Ramírez, Carolina Florez, Marina Muñoz, Carolina Hernandez, Adriana Castillo, Sergio Castañeda, Nathalia Ballesteros, David Martínez, Laura Vega, Jesús E. Jaimes, Sergio Gomez, Angelica Rico, Lisseth Pardo, Esther C. Barros, Martha L. Ospina, Aníbal A. Teherán, Ana S. Gonzalez-Reiche, Matthew M. Hernandez, Emilia Mia Sordillo, Viviana Simon, Harm van Bakel, Alberto Paniz-Mondolfi
EPI_ISL_447755, EPI_ISL_447756, EPI_ISL_447757, EPI_ISL_447759, EPI_ISL_447760, EPI_ISL_447761, EPI_ISL_447762, EPI_ISL_447763, EPI_ISL_447765, EPI_ISL_447766, EPI_ISL_447767, EPI_ISL_447768, EPI_ISL_447769, EPI_ISL_447771, EPI_ISL_447774, EPI_ISL_447775, EPI_ISL_447777, EPI_ISL_447778, EPI_ISL_447779, EPI_ISL_447780, EPI_ISL_447781, EPI_ISL_447785, EPI_ISL_447786, EPI_ISL_447789, EPI_ISL_447790, EPI_ISL_447791, EPI_ISL_447792, EPI_ISL_447793, EPI_ISL_447794, EPI_ISL_447795, EPI_ISL_447796, EPI_ISL_447797, EPI_ISL_447798, EPI_ISL_447800, EPI_ISL_447802, EPI_ISL_447803, EPI_ISL_447804, EPI_ISL_447805, EPI_ISL_447806, EPI_ISL_447807, EPI_ISL_447808, EPI_ISL_447809, EPI_ISL_447810, EPI_ISL_447812, EPI_ISL_447813, EPI_ISL_447816, EPI_ISL_447817			
see above	Instituto Nacional de Salud, Bogotá, Colombia	Grupo de Investigaciones Microbiológicas-UR (GIMUR), Departamento de Biología, Facultad de Ciencias Naturales, Universidad del Rosario, Bogotá, Colombia Instituto Nacional de Salud, Bogotá, Colombia Icahn School of Medicine at Mount Sinai, New York, USA	Juan David Ramírez, Carolina Florez, Marina Muñoz, Carolina Hernandez, Adriana Castillo, Sergio Castañeda, Nathalia Ballesteros, David Martínez, Laura Vega, Jesús E. Jaimes, Sergio Gomez, Angelica Rico, Lisseth Pardo, Esther C. Barros, Martha L. Ospina, Aníbal A. Teherán, Ana S. Gonzalez-Reiche, Matthew M. Hernandez, Emilia Mia Sordillo, Viviana Simon, Harm van Bakel, Alberto Paniz-Mondolfi
EPI_ISL_447854	CSIR-Centre for Cellular and Molecular Biology	CSIR-Centre for Cellular and Molecular Biology	Payel Mukherjee, Sofia Banu, Priya Singh, Dhiviya Vedagiri, Divya Gupta, Vishal Sah, Santosh Kumar Kuncha, Krishnan Harinivas Harshan, Archana Bharadwaj Siva, Karthik Bharadwaj Tallapaka, Shagufta Khan, Lamuk Zaveri, Namami Gaur, Sakshi Shambhavi, Tulasi Nagabandi, Purushotham Vodnala, Rakesh K Mishra, Divya Tej Sowpati
EPI_ISL_447855	CSIR-Centre for Cellular and Molecular Biology	CSIR-Centre for Cellular and Molecular Biology	Lamuk Zaveri, Shagufta Khan, Namami Gaur, Sakshi Shambhavi, Tulasi Nagabandi, Purushotham Vodnala, Payel Mukherjee, Sofia Banu, Priya Singh, Dhiviya Vedagiri, Divya Gupta, Vishal Sah, Santosh Kumar Kuncha, Krishnan Harinivas Harshan, Archana Bharadwaj Siva, Karthik Bharadwaj Tallapaka, Rakesh K Mishra, Divya Tej Sowpati
EPI_ISL_448222	Pasteur Insitute Ho Chi Minh City	National Key Laboratory of Gene Technology, Institute of Biotechnology, Vietnam Academy of Science and Technology	Le Tung Lam, Nguyen Trung Hieu, Nguyen Hong Trang, Ho Thi Thuong, Nguyen Thi Ngoc Thao, Huynh Thi Kim Loan, Luu Thuy Tien, Tran Huyen Linh, Pham Duy Quang, Luong Chan Quang, Cao Minh Thang, Nguyen Vu Thuong, Hoang Ha, Chu Hoang Ha, Phan Trong Lan, Truong Nam Hai
EPI_ISL_450241, EPI_ISL_450242, EPI_ISL_450243, EPI_ISL_450244, EPI_ISL_450245, EPI_ISL_450248, EPI_ISL_450249, EPI_ISL_450250, EPI_ISL_450251, EPI_ISL_450252, EPI_ISL_450258, EPI_ISL_450259, EPI_ISL_450260, EPI_ISL_450261, EPI_ISL_450262, EPI_ISL_450263, EPI_ISL_450264, EPI_ISL_450265, EPI_ISL_450266, EPI_ISL_450267, EPI_ISL_450271, EPI_ISL_450272, EPI_ISL_450273, EPI_ISL_450274, EPI_ISL_450275, EPI_ISL_450276, EPI_ISL_450277, EPI_ISL_450278, EPI_ISL_450279, EPI_ISL_450280, EPI_ISL_450281, EPI_ISL_450282, EPI_ISL_450283, EPI_ISL_450284, EPI_ISL_450285, EPI_ISL_450286, EPI_ISL_450287, EPI_ISL_450288, EPI_ISL_450289, EPI_ISL_450290, EPI_ISL_450292, EPI_ISL_450293			
see above	WHO National Influenza Centre Russian Federation	WHO National Influenza Centre Russian Federation	Andrey Komissarov, Artem Fadeev, Mariia Sergeeva, Anna Ivanova, Tamila Musaeva, Ksenia Komissarova, Mariia Timofeeva, Veronica Eder, Mariia Pisareva, Daria Danilenko
EPI_ISL_450321	NIV Pune	CSIR-Centre for Cellular and Molecular Biology	Dr V A Potdar, Dr ML Choudhary,Dr Priya Abraham,V. Vipat, S. Jadhav, U. Saha, H. Kengle, A. Awhale, A. Jagtap, A. Gondhalikar, V Malik, N Srivastava, S. Digraskar, P. Malsane, S. Hundekar, K. Patel, Yogesh Balakartik, M. Kakade, S. Jadhav, R. Gunjikar, V. Awtade, S. Bhorekar, P Shinde, S. Salve, B. Minhas S. Bharadwaj, H Kaushal Y. Gurav, S. Tomar,Payel Mukherjee, Sofia Banu, Priya Singh, Dhiviya Vedagiri, Divya Gupta, Vishal Sah, Santosh Kumar Kuncha, Krishnan Harinivas Harshan, Archana Bharadwaj Siva, Karthik Bharadwaj Tallapaka, Shagufta Khan, Lamuk Zaveri, Namami Gaur, Sakshi Shambhavi, Tulasi Nagabandi, Purushotham Vodnala,G. Aditya Kumar, Koushick Sivakumar, Pooja Ramesh Gupta, Rajan Kumar Jha, Shraddha Vijay Lahoti, Deepak Kumar, Devi Prasad Vijayashankara, Disha Nanda, Divya Das, Jotin Gogoi, Manish
EPI_ISL_450322	NIV Pune	CSIR-Centre for Cellular and Molecular Biology	Dr V A Potdar, Dr ML Choudhary,Dr Priya Abraham,V. Vipat, S. Jadhav, U. Saha, H. Kengle, A. Awhale, A. Jagtap, A. Gondhalikar, V Malik, N Srivastava, S. Digraskar, P. Malsane, S. Hundekar, K. Patel, Yogesh Balakartik, M. Kakade, S. Jadhav, R. Gunjikar, V. Awtade, S. Bhorekar, P Shinde, S. Salve, B. Minhas S.

			Bharadwaj, H Kaushal Y. Gurav, S. Tomar,Sofia Banu, Payel Mukherjee, Priya Singh, Dhiviya Vedagiri, Divya Gupta, Vishal Sah, Santosh Kumar Kuncha, Krishnan Harinivas Harshan, Archana Bharadwaj Siva, Karthik Bharadwaj Tallapaka, Shagufta Khan, Lamuk Zaveri, Namami Gaur, Sakshi Shambhavi, Tulasi Nagabandi, Purushotham Vodnala, Disha Nanda, Divya Das, Jotin Gogoi, Manish Bhattacharjee, Ravi Prasad Mukku, Renu Sudhakar, Somesh Gorde, Gangumala Srinivas Reddy, Sujoy Deb, Swati Bayyana, Zeba Rizvi, Rakesh K Mishra
EPI_ISL_450323	NIV Pune	CSIR-Centre for Cellular and Molecular Biology	Dr V A Potdar, Dr ML Choudhary,Dr Priya Abraham,V. Vipat, S. Jadhav, U. Saha, H. Kengle, A. Awhale, A. Jagtap, A. Gondhalikar, V Malik, N Srivastava, S. Digraskar, P. Malsane, S. Hundekar, K. Patel, Yogesh Balakartik, M. Kakade, S. Jadhav, R. Gunjikar, V. Awtade, S. Bhorekar, P Shinde, S. Salve, B. Minhas S. Bharadwaj, H Kaushal Y. Gurav, S. Tomar,Payel Mukherjee, Sofia Banu, Priya Singh, Dhiviya Vedagiri, Divya Gupta, Vishal Sah, Santosh Kumar Kuncha, Krishnan Harinivas Harshan, Archana Bharadwaj Siva, Karthik Bharadwaj Tallapaka, Shagufta Khan, Lamuk Zaveri, Namami Gaur, Sakshi Shambhavi, Tulasi Nagabandi, Purushotham Vodnala,G. Aditya Kumar, Koushick Sivakumar, Pooja Ramesh Gupta, Rajan Kumar Jha, Shraddha Vijay Lahoti, Deepak Kumar, Devi Prasad Vijayashankara, Disha Nanda, Divya Das, Jotin Gogoi, Manish
EPI_ISL_450324	NIV Pune	CSIR-Centre for Cellular and Molecular Biology	Dr V A Potdar, Dr ML Choudhary,Dr Priya Abraham,V. Vipat, S. Jadhav, U. Saha, H. Kengle, A. Awhale, A. Jagtap, A. Gondhalikar, V Malik, N Srivastava, S. Digraskar, P. Malsane, S. Hundekar, K. Patel, Yogesh Balakartik, M. Kakade, S. Jadhav, R. Gunjikar, V. Awtade, S. Bhorekar, P Shinde, S. Salve, B. Minhas S. Bharadwaj, H Kaushal Y. Gurav, S. Tomar,Sofia Banu, Payel Mukherjee, Priya Singh, Dhiviya Vedagiri, Divya Gupta, Vishal Sah, Santosh Kumar Kuncha, Krishnan Harinivas Harshan, Archana Bharadwaj Siva, Karthik Bharadwaj Tallapaka, Shagufta Khan, Lamuk Zaveri, Namami Gaur, Sakshi Shambhavi, Tulasi Nagabandi, Purushotham Vodnala, Disha Nanda, Divya Das, Jotin Gogoi, Manish Bhattacharjee, Ravi Prasad Mukku, Renu Sudhakar, Somesh Gorde, Gangumala Srinivas Reddy, Sujoy Deb, Swati Bayyana, Zeba Rizvi, Rakesh K Mishra
EPI_ISL_450325	NIV Pune	CSIR-Centre for Cellular and Molecular Biology	Dr V A Potdar, Dr ML Choudhary,Dr Priya Abraham,V. Vipat, S. Jadhav, U. Saha, H. Kengle, A. Awhale, A. Jagtap, A. Gondhalikar, V Malik, N Srivastava, S. Digraskar, P. Malsane, S. Hundekar, K. Patel, Yogesh Balakartik, M. Kakade, S. Jadhav, R. Gunjikar, V. Awtade, S. Bhorekar, P Shinde, S. Salve, B. Minhas S. Bharadwaj, H Kaushal Y. Gurav, S. Tomar,Payel Mukherjee, Sofia Banu, Priya Singh, Dhiviya Vedagiri, Divya Gupta, Vishal Sah, Santosh Kumar Kuncha, Krishnan Harinivas Harshan, Archana Bharadwaj Siva, Karthik Bharadwaj Tallapaka, Shagufta Khan, Lamuk Zaveri, Namami Gaur, Sakshi Shambhavi, Tulasi Nagabandi, Purushotham Vodnala,G. Aditya Kumar, Koushick Sivakumar, Pooja Ramesh Gupta, Rajan Kumar Jha, Shraddha Vijay Lahoti, Deepak Kumar, Devi Prasad Vijayashankara, Disha Nanda, Divya Das, Jotin Gogoi, Manish
EPI_ISL_450340	Bangladesh Institute of Tropical & Infectious Diseases, COVID-19 Testing Laboratory	Basic and Applied Research on Jute Project	Rasel Ahmed, Md. Sabbir Hossain, Shah Md Tamim Kabir, Emdadul Mannan Emdad, Md. Nazmul Haq Rony, Eaftekhar Ahmed Rana, Paritous Kumar Biswas, M A Hassan Chowdhury, Md. Shakeel Ahmed, Md. Samiul Haque, Md. Monjurul Alam, Md. Sharifur Rahman, A S M Anwarul Huq, Md. Shahidul Islam, Goutam Buddha Das, AMAM Zonaed Siddiki
EPI_ISL_450341	Bangladesh Institute of Tropical & Infectious Diseases, COVID-19 Testing Laboratory	Basic and Applied Research on Jute Project	Md. Sabbir Hossain, Rasel Ahmed, Shah Md Tamim Kabir, Emdadul Mannan Emdad, Md. Nazmul Haq Rony, Eaftekhar Ahmed Rana, Paritous Kumar Biswas, M A Hassan Chowdhury, Md. Shakeel Ahmed, Md. Samiul Haque, Md. Monjurul Alam, Md. Sharifur Rahman, A S M Anwarul Huq, Md. Shahidul Islam, Goutam Buddha Das, AMAM Zonaed Siddiki
EPI_ISL_450342	Bangladesh Institute of Tropical & Infectious Diseases, COVID-19 Testing Laboratory	Basic and Applied Research on Jute Project	Rasel Ahmed, Md. Sabbir Hossain, Shah Md Tamim Kabir, Emdadul Mannan Emdad, Md. Nazmul Haq Rony, Eaftekhar Ahmed Rana, Paritous Kumar Biswas, M A Hassan Chowdhury, Md. Shakeel Ahmed, Md. Samiul Haque, Md. Monjurul Alam, Md. Sharifur Rahman, A S M Anwarul Huq, Md. Shahidul Islam, Goutam Buddha Das, AMAM Zonaed Siddiki
EPI_ISL_450343	Bangladesh Institute of Tropical & Infectious Diseases, COVID-19 Testing Laboratory	Basic and Applied Research on Jute Project	Md. Sabbir Hossain, Rasel Ahmed, Shah Md Tamim Kabir, Emdadul Mannan Emdad, Md. Nazmul Haq Rony, Eaftekhar Ahmed Rana, Paritous Kumar Biswas, M A Hassan Chowdhury, Md. Shakeel Ahmed, Md. Samiul Haque, Md. Monjurul Alam, Md. Sharifur Rahman, A S M Anwarul Huq, Md. Shahidul Islam, Goutam Buddha Das, AMAM Zonaed Siddiki
EPI_ISL_450508, EPI_ISL_450509, EPI_ISL_450511, EPI_ISL_450512, EPI_ISL_450515	Rafik Hariri University Hospital	Rafik Hariri University Hospital	Rita Feghali
EPI_ISL_450724, EPI_ISL_450725, EPI_ISL_450726, EPI_ISL_450727, EPI_ISL_450728, EPI_ISL_450729, EPI_ISL_450730	Hospital AZ Rivierenland	Institute of Tropical Medicine	Philippe Selhorst, Colin Anthony
EPI_ISL_450746	Laboratory of Molecular Biology, Diagnostyka sp. z o.o.	Laboratory of Recombinant Vaccines	Lukasz Rabalski, Anna Piotrowska-Mietelska, Maciej Kosinski, Boguslaw Szewczyk, Krystyna Bienkowska-Szewczyk
EPI_ISL_450781	Government Medical College-Bhavnagar	Gujarat Biotechnology Research Centre	Kairavi Desai, Saklain Malek, Shirish Patel, Ramesh Pandit, Tejas Shah, Ankit Hinsu, Pritesh Sabara, Apurvasinh Puvar, Janvi Raval, Zarna Patel, Monika Gandhi, Pinal Trivedi, Maharshi Pandya, Amit Kanani, Nidhi Patel, Nitin Savaliya, Raghawendra Kumar, Dinesh Kumar, Zuber Saiyed, Komal Patel, Labdhi Pandya, Snehal Bagatharia, Bhavesh Modi, Gaurishankar Shrimali, R D Dixit, A M Kadri, Akanksha Verma, Chaitanya Joshi, Madhvi Joshi
EPI_ISL_450783	Government Medical College-Bhavnagar	Gujarat Biotechnology Research Centre	Shirish Patel, Kairavi Desai, Saklain Malek, Ankit Hinsu, Pritesh Sabara, Apurvasinh Puvar, Janvi Raval, Zarna Patel, Monika Gandhi, Pinal Trivedi, Maharshi Pandya, Amit Kanani, Nidhi Patel, Nitin Savaliya, Raghawendra Kumar, Dinesh Kumar, Zuber Saiyed, Komal Patel, Labdhi Pandya, Snehal Bagatharia, Ramesh Pandit, Tejas Shah, Bhavesh Modi, Gaurishankar Shrimali, R D Dixit, A M Kadri, Neha Rajpara, Chaitanya Joshi, Madhvi Joshi
EPI_ISL_450784	Government Medical College-Bhavnagar	Gujarat Biotechnology Research Centre	Zarna Patel, Ramesh Pandit, Tejas Shah, Ankit Hinsu, Pritesh Sabara, Apurvasinh Puvar, Janvi Raval, Monika Gandhi, Pinal Trivedi, Maharshi Pandya, Amit Kanani, Nidhi Patel, Nitin Savaliya, Raghawendra Kumar, Dinesh Kumar, Zuber Saiyed, Komal Patel, Labdhi Pandya, Snehal Bagatharia, Kairavi Desai, Saklain Malek, Shirish Patel, Bhavesh Modi, Gaurishankar Shrimali, R D Dixit, A M Kadri, Afzal Ansari, Chaitanya Joshi, Madhvi Joshi
EPI_ISL_450785	Pandit Deendayal Upadhyay Government Medical College, Rajkot	Gujarat Biotechnology Research Centre	Prakash Modi, Sejul Antala, Manish Pattani, Apurvasinh Puvar, Janvi Raval, Zarna Patel, Monika Gandhi, Pinal Trivedi, Maharshi Pandya, Amit Kanani, Nidhi Patel, Nitin Savaliya, Raghawendra Kumar, Dinesh Kumar, Zuber Saiyed, Komal Patel, Labdhi Pandya, Snehal Bagatharia, Ramesh Pandit, Tejas Shah, Ankit Hinsu, Pritesh Sabara, Bhavesh Modi, Gaurishankar Shrimali, R D Dixit, A M Kadri, Neelam Nathani, Chaitanya Joshi, Madhvi Joshi
EPI_ISL_450786	Pandit Deendayal Upadhyay Government Medical College, Rajkot	Gujarat Biotechnology Research Centre	Sejul Antala, Manish Pattani, Prakash Modi, Janvi Raval, Zarna Patel, Monika Gandhi, Pinal Trivedi, Maharshi Pandya, Amit Kanani, Nidhi Patel, Nitin Savaliya, Raghawendra Kumar, Dinesh Kumar, Zuber Saiyed, Komal Patel, Labdhi Pandya, Snehal Bagatharia, Ramesh Pandit, Tejas Shah, Ankit Hinsu, Pritesh Sabara, Apurvasinh Puvar, Bhavesh Modi, Gaurishankar Shrimali, R D Dixit, A M Kadri, Armi Chaudhari, Chaitanya Joshi, Madhvi Joshi
EPI_ISL_450788	Pandit Deendayal Upadhyay Government Medical College, Rajkot	Gujarat Biotechnology Research Centre	Zarna Patel, Tejas Shah, Ankit Hinsu, Pritesh Sabara, Apurvasinh Puvar, Janvi Raval, Monika Gandhi, Pinal Trivedi, Maharshi Pandya, Amit Kanani, Nidhi Patel, Nitin Savaliya, Raghawendra Kumar, Dinesh Kumar, Zuber Saiyed, Komal Patel, Labdhi Pandya, Snehal Bagatharia, Prakash Modi, Sejul Antala, Manish Pattani, Ramesh Pandit, Bhavesh Modi, Gaurishankar Shrimali, R D Dixit, A M Kadri, Camellia Chakraborty, Chaitanya Joshi, Madhvi Joshi
EPI_ISL_450789	Pandit Deendayal Upadhyay Government Medical College, Rajkot	Gujarat Biotechnology Research Centre	Ankit Hinsu, Pritesh Sabara, Apurvasinh Puvar, Janvi Raval, Zarna Patel, Monika Gandhi, Pinal Trivedi, Maharshi Pandya, Amit Kanani, Nidhi Patel, Nitin Savaliya, Raghawendra Kumar, Dinesh Kumar, Zuber Saiyed, Komal Patel, Labdhi Pandya, Snehal Bagatharia, Prakash Modi, Sejul Antala, Manish Pattani, Ramesh Pandit, Tejas Shah, Bhavesh Modi, Gaurishankar Shrimali, R D Dixit, A M Kadri, Siddhant Kumar, Chaitanya Joshi, Madhvi Joshi
EPI_ISL_450790	Pandit Deendayal Upadhyay Government Medical College, Rajkot	Gujarat Biotechnology Research Centre	Zarna Patel, Pritesh Sabara, Apurvasinh Puvar, Janvi Raval, Monika Gandhi, Pinal Trivedi, Maharshi Pandya, Amit Kanani, Nidhi Patel, Nitin Savaliya, Raghawendra Kumar, Dinesh Kumar, Zuber Saiyed, Komal Patel, Labdhi Pandya, Snehal Bagatharia, Prakash Modi, Sejul Antala, Manish Pattani, Ramesh Pandit, Tejas Shah, Ankit Hinsu, Bhavesh Modi, Gaurishankar Shrimali, R D Dixit, A M Kadri, Sharmistha Majumdar, Chaitanya Joshi, Madhvi Joshi
EPI_ISL_450791	Pandit Deendayal Upadhyay Government Medical College, Rajkot	Gujarat Biotechnology Research Centre	Zarna Patel, Apurvasinh Puvar, Janvi Raval, Monika Gandhi, Pinal Trivedi, Maharshi Pandya, Amit Kanani, Nidhi Patel, Nitin Savaliya, Raghawendra Kumar, Dinesh Kumar, Zuber Saiyed, Komal Patel, Labdhi Pandya, Snehal Bagatharia, Prakash Modi, Sejul Antala, Manish Pattani, Ramesh Pandit, Tejas Shah, Ankit Hinsu, Pritesh Sabara, Bhavesh Modi, Gaurishankar Shrimali, R D Dixit, A M Kadri, Pooja P Doshi, Chaitanya Joshi, Madhvi Joshi
EPI_ISL_450839	COVID-19 Laboratory Centre for Advanced Research in Sciences (CARS), University of Dhaka, Dhaka-1000, Bangladesh	DNA Solution Ltd	Sharif Akhteruzzaman, Zeba Islam Seraj, Nazmul Ahsan, Md Imdadul Hoque, MA Malek, Shahryar Nabi, Sabrina Moriom Elius, ABM Khademul Islam, Richard Malo, Imran Khan, Abu Sufian, Sabita Rezwana Rahman, Habibul Bari Shozib, Mamun Ahmed, AHM Nurun Nabi, Mohammad Riazul Islam, Md Mizanur Rahman, Md Ismail Hosen, Latiful Bari, Gazi Nurun Nahar, Haseena Khan, M Anwar Hossain.
EPI_ISL_450840	COVID-19 Laboratory	DNA Solution Ltd. L-5	Sharif Akhteruzzaman, Zeba Islam Seraj, Nazmul Ahsan, Md Imdadul Hoque, MA Malek, Shahryar Nabi, Sabrina Moriom Elius, ABM Khademul Islam, Richard Malo, Imran Khan, Abu Sufian, Sabita Rezwana Rahman, Habibul Bari Shozib, Mamun Ahmed, AHM Nurun Nabi, Mohammad Riazul Islam, Md Mizanur Rahman, Md Ismail Hosen, Latiful Bari, Gazi Nurun Nahar, Haseena Khan, M Anwar Hossain.
EPI_ISL_450841	COVID-19 Laboratory	DNA Solution Ltd	Sharif Akhteruzzaman, Zeba Islam Seraj, Nazmul Ahsan, Md Imdadul Hoque, MA Malek, Shahryar Nabi, Sabrina Moriom Elius, ABM Khademul Islam, Richard

			Malo, Imran Khan, Abu Sufian, Sabita Rezwana Rahman, Habibul Bari Shozib, Mamun Ahmed, AHM Nurun Nabi, Mohammad Riazul Islam, Md Mizanur Rahman, Md Ismail Hosen, Latiful Bari, Gazi Nurun Nahar, Haseena Khan, M Anwar Hossain.
EPI_ISL_450843	COVID-19 Laboratory	DNA Solution Ltd.	Sharif Akhteruzzaman, Zeba Islam Seraj, Nazmul Ahsan, Md Imdadul Hoque, MA Malek, Shahryar Nabi, Sabrina Moriom Elius, ABM Khademul Islam, Richard Malo, Imran Khan, Abu Sufian, Sabita Rezwana Rahman, Habibul Bari Shozib, Mamun Ahmed, AHM Nurun Nabi, Mohammad Riazul Islam, Md Mizanur Rahman, Md Ismail Hosen, Latiful Bari, Gazi Nurun Nahar, Haseena Khan, M Anwar Hossain.
EPI_ISL_451076	West China Hospital of Sichuan University	State Key Laboratory of Biotherapy of Sichuan University	Baowen Du, Minjin Wang, Chao Tanga, Chuan Chena, Yongzhao Zhou, Mingxia Yu, Han-Cheng Wei, Weimin Li, Jing-wen Lin, Jia Geng, Binwu Ying, Lu Chen
EPI_ISL_451149	M.P Shah Government Medocal college Jamnagar	Gujarat Biotechnology Research Centre	Janvi Raval, Zarna Patel, Monika Gandhi, Pinal Trivedi, Maharshi Pandya, Amit Kanani, Nidhi Patel, Nitin Savaliya, Raghawendra Kumar, Dinesh Kumar, Zuber Saiyed, Komal Patel, Labdhi Pandya, Snehal Bagatharia, Ramesh Pandit, Tejas Shah, Ankit Hinsu, Pritesh Sabara, Apurvasinh Puvar, Binita Aring, Bhavesh Modi, Gaurishankar Shrimali, R D Dixit, A M Kadri, Priti Pandita, Chaitanya Joshi, Madhvi Joshi,
EPI_ISL_451150	M.P Shah Government Medocal college Jamnagar	Gujarat Biotechnology Research Centre	Zarna Patel, Monika Gandhi, Pinal Trivedi, Maharshi Pandya, Amit Kanani, Nidhi Patel, Nitin Savaliya, Raghawendra Kumar, Dinesh Kumar, Zuber Saiyed, Komal Patel, Labdhi Pandya, Snehal Bagatharia, Ramesh Pandit, Tejas Shah, Ankit Hinsu, Pritesh Sabara, Apurvasinh Puvar, Binita Aring, Janvi Raval, Bhavesh Modi, Gaurishankar Shrimali, R D Dixit, A M Kadri, Pragya Sharma, Chaitanya Joshi, Madhvi Joshi,
EPI_ISL_451151	M.P Shah Government Medocal college Jamnagar	Gujarat Biotechnology Research Centre	Monika Gandhi, Pinal Trivedi, Maharshi Pandya, Amit Kanani, Nidhi Patel, Nitin Savaliya, Raghawendra Kumar, Dinesh Kumar, Zuber Saiyed, Komal Patel, Labdhi Pandya, Snehal Bagatharia, Ramesh Pandit, Tejas Shah, Ankit Hinsu, Pritesh Sabara, Apurvasinh Puvar, Binita Aring, Janvi Raval, Zarna Patel, Bhavesh Modi, Gaurishankar Shrimali, R D Dixit, A M Kadri, Neha Rajpara, Chaitanya Joshi, Madhvi Joshi,
EPI_ISL_451152	M.P Shah Government Medocal college Jamnagar	Gujarat Biotechnology Research Centre	Pinal Trivedi, Maharshi Pandya, Amit Kanani, Nidhi Patel, Nitin Savaliya, Raghawendra Kumar, Dinesh Kumar, Zuber Saiyed, Komal Patel, Labdhi Pandya, Snehal Bagatharia, Ramesh Pandit, Tejas Shah, Ankit Hinsu, Pritesh Sabara, Apurvasinh Puvar, Binita Aring, Janvi Raval, Zarna Patel, Monika Gandhi, Bhavesh Modi, Gaurishankar Shrimali, R D Dixit, A M Kadri, Afzal Ansari, Chaitanya Joshi, Madhvi Joshi,
EPI_ISL_451157	Government Medical College, Vadodara	Gujarat Biotechnology Research Centre	Nidhi Patel, Nitin Savaliya, Raghawendra Kumar, Dinesh Kumar, Zuber Saiyed, Komal Patel, Labdhi Pandya, Snehal Bagatharia, Ramesh Pandit, Tejas Shah, Ankit Hinsu, Pritesh Sabara, Apurvasinh Puvar, Janvi Raval, Zarna Patel, Monika Gandhi, Pinal Trivedi, Maharshi Pandya, Manish Pattani, Tanuja Javadekar , Amit Kanani, Bhavesh Modi, Gaurishankar Shrimali, R D Dixit, A M Kadri, Camellia Chakraborty, Chaitanya Joshi, Madhvi Joshi
EPI_ISL_451158	Government Medical College, Vadodara	Gujarat Biotechnology Research Centre	Nitin Savaliya, Raghawendra Kumar, Dinesh Kumar, Zuber Saiyed, Komal Patel, Labdhi Pandya, Snehal Bagatharia, Ramesh Pandit, Tejas Shah, Ankit Hinsu, Pritesh Sabara, Apurvasinh Puvar, Janvi Raval, Zarna Patel, Monika Gandhi, Pinal Trivedi, Maharshi Pandya, Manish Pattani, Tanuja Javadekar , Amit Kanani, Nidhi Patel, Bhavesh Modi, Gaurishankar Shrimali, R D Dixit, A M Kadri, Siddhant Kumar, Chaitanya Joshi, Madhvi Joshi
EPI_ISL_451161	Government Medical College, Vadodara	Gujarat Biotechnology Research Centre	Zuber Saiyed, Komal Patel, Labdhi Pandya, Snehal Bagatharia, Ramesh Pandit, Tejas Shah, Ankit Hinsu, Pritesh Sabara, Apurvasinh Puvar, Janvi Raval, Zarna Patel, Monika Gandhi, Pinal Trivedi, Maharshi Pandya, Manish Pattani, Tanuja Javadekar , Amit Kanani, Nidhi Patel, Nitin Savaliya, Raghawendra Kumar, Dinesh Kumar, Bhavesh Modi, Gaurishankar Shrimali, R D Dixit, A M Kadri, Akanksha Verma, Chaitanya Joshi, Madhvi Joshi
EPI_ISL_451162	Government Medical College, Vadodara	Gujarat Biotechnology Research Centre	Komal Patel, Labdhi Pandya, Snehal Bagatharia, Ramesh Pandit, Tejas Shah, Ankit Hinsu, Pritesh Sabara, Apurvasinh Puvar, Janvi Raval, Zarna Patel, Monika Gandhi, Pinal Trivedi, Maharshi Pandya, Manish Pattani, Tanuja Javadekar , Amit Kanani, Nidhi Patel, Nitin Savaliya, Raghawendra Kumar, Dinesh Kumar, Zuber Saiyed, Bhavesh Modi, Gaurishankar Shrimali, R D Dixit, A M Kadri, Priti Pandita, Chaitanya Joshi, Madhvi Joshi
EPI_ISL_451298	Laboratory of Virology, INMI Lazzaro Spallanzani IRCCS	Laboratory of Virology, INMI Lazzaro Spallanzani IRCCS	Cesare E.M. Gruber, Martina Rueca, Barbara Bartolini, Francesco Messina, Antonino Di Caro, Maria R. Capobianchi, Giuseppe Ippolito
EPI_ISL_451299	Laboratory of Virology, INMI Lazzaro Spallanzani IRCCS	Laboratory of Virology, INMI Lazzaro Spallanzani IRCCS	Martina Rueca, Cesare E.M. Gruber, Barbara Bartolini, Francesco Messina, Antonino Di Caro, Maria R. Capobianchi, Giuseppe Ippolito
EPI_ISL_451300	Laboratory of Virology, INMI Lazzaro Spallanzani IRCCS	Laboratory of Virology, INMI Lazzaro Spallanzani IRCCS	Cesare E.M. Gruber, Martina Rueca, Barbara Bartolini, Francesco Messina, Antonino Di Caro, Maria R. Capobianchi, Giuseppe Ippolito
EPI_ISL_451301	Laboratory of Virology, INMI Lazzaro Spallanzani IRCCS	Laboratory of Virology, INMI Lazzaro Spallanzani IRCCS	Martina Rueca, Cesare E.M. Gruber, Barbara Bartolini, Francesco Messina, Antonino Di Caro, Maria R. Capobianchi, Giuseppe Ippolito
EPI_ISL_451302	Laboratory of Virology, INMI Lazzaro Spallanzani IRCCS	Laboratory of Virology, INMI Lazzaro Spallanzani IRCCS	Cesare E.M. Gruber, Martina Rueca, Barbara Bartolini, Francesco Messina, Antonino Di Caro, Maria R. Capobianchi, Giuseppe Ippolito
EPI_ISL_451303	Laboratory of Virology, INMI Lazzaro Spallanzani IRCCS	Laboratory of Virology, INMI Lazzaro Spallanzani IRCCS	Martina Rueca, Cesare E.M. Gruber, Barbara Bartolini, Francesco Messina, Antonino Di Caro, Maria R. Capobianchi, Giuseppe Ippolito
EPI_ISL_451304	Laboratory of Virology, INMI Lazzaro Spallanzani IRCCS	Laboratory of Virology, INMI Lazzaro Spallanzani IRCCS	Cesare E.M. Gruber, Martina Rueca, Barbara Bartolini, Francesco Messina, Antonino Di Caro, Maria R. Capobianchi, Giuseppe Ippolito
EPI_ISL_451306	Molecular Virology Unit, Fondazione IRCCS Policlinico San Matteo , Pavia	Laboratory of Virology, INMI Lazzaro Spallanzani IRCCS	Antonio Piralla, Fausto Baldanti, Martina Rueca, Antonino Di Caro, Maria R. Capobianchi, Cesare E.M. Gruber, Barbara Bartolini
EPI_ISL_451307	Molecular Virology Unit, Fondazione IRCCS Policlinico San Matteo , Pavia	Laboratory of Virology, INMI Lazzaro Spallanzani IRCCS	Fausto Baldanti, Antonio Piralla, Antonino Di Caro, Cesare E.M. Gruber, Martina Rueca, Barbara Bartolini, Maria R. Capobianchi
EPI_ISL_451308	Molecular Virology Unit, Fondazione IRCCS Policlinico San Matteo , Pavia	Laboratory of Virology, INMI Lazzaro Spallanzani IRCCS	Antonio Piralla, Fausto Baldanti, Maria R. Capobianchi, Cesare E.M. Gruber, Martina Rueca, Barbara Bartolini, Antonino Di Caro
EPI_ISL_451309	Molecular Virology Unit, Fondazione IRCCS Policlinico San Matteo , Pavia	Laboratory of Virology, INMI Lazzaro Spallanzani IRCCS	Fausto Baldanti, Antonio Piralla, Cesare E.M. Gruber, Maria R. Capobianchi, Antonino Di Caro, Martina Rueca, Barbara Bartolini
EPI_ISL_451310, EPI_ISL_451311	Hellenic Pasteur Institute, National Influenza Reference laboratory of Southern Greece & Unit of Bioinformatics and Applied Genomics	Hellenic Pasteur Institute, National Influenza Reference laboratory of Southern Greece & Unit of Bioinformatics and Applied Genomics	Vasiliki Pogka, Timokratis Karamitros, Athanasios Kossyvakis, Antonios Kalliaropoulos, Horefti Elina, Evangelidou Maria, Androniki Voulgari-Kokota, Aspasia Kontou, Andreas Mentis
EPI_ISL_451313, EPI_ISL_451314, EPI_ISL_451315, EPI_ISL_451316, EPI_ISL_451318, EPI_ISL_451319, EPI_ISL_451320, EPI_ISL_451321, EPI_ISL_451322, EPI_ISL_451325, EPI_ISL_451326, EPI_ISL_451327, EPI_ISL_451328, EPI_ISL_451329, EPI_ISL_451330, EPI_ISL_451331, EPI_ISL_451334, EPI_ISL_451337, EPI_ISL_451338, EPI_ISL_451344, EPI_ISL_451345, EPI_ISL_451346, EPI_ISL_451348, EPI_ISL_451353, EPI_ISL_451354, EPI_ISL_451356, EPI_ISL_451357, EPI_ISL_451359, EPI_ISL_451360, EPI_ISL_451365, EPI_ISL_451369, EPI_ISL_451370, EPI_ISL_451371, EPI_ISL_451374, EPI_ISL_451376, EPI_ISL_451377, EPI_ISL_451378, EPI_ISL_451379, EPI_ISL_451380, EPI_ISL_451381, EPI_ISL_451382, EPI_ISL_451383, EPI_ISL_451384, EPI_ISL_451385, EPI_ISL_451386, EPI_ISL_451387, EPI_ISL_451388, EPI_ISL_451389, EPI_ISL_451390, EPI_ISL_451391, EPI_ISL_451392, EPI_ISL_451393, EPI_ISL_451394, EPI_ISL_451395, EPI_ISL_451398			
see above	West China Hospital of Sichuan University	State Key Laboratory of Biotherapy of Sichuan University	Baowen Du, Minjin Wang, Chao Tang, Chuan Chen, Yongzhao Zhou, Mingxia Yu, Hancheng Wei, Weimin Li, Jing-wen Lin, Jia Geng, Binwu Ying, Lu Chen
EPI_ISL_451644, EPI_ISL_451645, EPI_ISL_451646, EPI_ISL_451647	Laboratory of Molecular Biology, Diagnostyka sp. z o.o.	Laboratory of Recombinant Vaccines	Lukasz Rabalski, Anna Piotrowska-Mietelska, Maciej Kosinski, Boguslaw Szewczyk, Krystyna Bienkowska-Szewczyk
EPI_ISL_451655, EPI_ISL_451656, EPI_ISL_451657, EPI_ISL_451658, EPI_ISL_451659, EPI_ISL_451660, EPI_ISL_451661, EPI_ISL_451662, EPI_ISL_451663, EPI_ISL_451664	State Sanitary Inspectorate	Laboratory of Recombinant Vaccines	Lukasz Rabalski, Boguslaw Szewczyk, Krystyna Bienkowska-Szewczyk, Jaroslaw Pinkas
EPI_ISL_451666	M.P Shah Government Medocal college Jamnagar	Gujarat Biotechnology Research Centre	Binita Aring, Janvi Raval, Zarna Patel, Monika Gandhi, Pinal Trivedi, Maharshi Pandya, Amit Kanani, Nidhi Patel, Nitin Savaliya, Raghawendra Kumar, Dinesh Kumar, Zuber Saiyed, Komal Patel, Labdhi Pandya, Snehal Bagatharia, Ramesh Pandit, Tejas Shah, Ankit Hinsu, Pritesh Sabara, Apurvasinh Puvar, Bhavesh Modi, Gaurishankar Shrimali, R D Dixit, A M Kadri, Akanksha Verma, Chaitanya Joshi, Madhvi Joshi,
EPI_ISL_451934	Research Unit, University Hospital for Infectious Diseases "Dr. Fran Mihaljevi"	Cicin Sain lab, Helmholtz Centre for Infection Research	Zeeshan Chaudhry, Kathrin Eschke, Željka Maak Šafranko, Ivan-Christian Kurolt
EPI_ISL_451935	CUB Hopital Erasme Laboratoire d'Anatomie Pathologique	CUB Hopital Erasme Laboratoire d'Anatomie Pathologique	Prof. Isabelle Salmon, Dr. Nicky D'Haene
EPI_ISL_451961	Istituto Zooprofilattico Sperimentale Puglia e Basilicata; Dipartimento di Bioscienze, Biotecnologie e Biofarmaceutica dell'Università degli Studi di Bari "A.Moro"; Istituto di Biomembrane. Bioenergetica e Biotecnologie Molecolari del Consiglio Nazionale delle Ricerche di Bari	Beaconlab (Bioinformatics Evolution and Comparative Genomics lab), Dept of Biosciences, University of Milan	Parisi A.,Pesole G., Manzari C., Chiara M.
EPI_ISL_451962	Istituto Zooprofilattico Sperimentale Puglia e Basilicata;	Beaconlab (Bioinformatics, Evolution and Comparative	Parisi A.,Pesole G., Manzari C., Chiara M.

	Dipartimento di Bioscienze, Biotecnologie e Biofarmaceutica dell'Università degli Studi di Bari "A.Moro"; Istituto di Biomembrane. Bioenergetica e Biotecnologie Molecolari del Consiglio Nazionale delle Ricerche di Bari	Genomics lab), Dept of Biosciences, University on Milan	
EPI_ISL_452139	Instituto de Diagnostico y Referencia Epidemiologicos (INDRE)	Instituto de diagnóstico y Referencia Epidemiologicos (INDRE)	Ramirez-Gonzalez Ernesto, Garces-Ayala Fabiola, Araiza-Rodriguez Adnan, Mendieta-Condado Edgar, Rodriguez-Maldonado Abril, Wong-Arambula Claudia, Barrera-Badillo Gisela, Hernandez-Rivas Lucia, Lopez-Martinez Irma
EPI_ISL_452140	CUB Hopital Erasme Laboratoire d'Anatomie Pathologique	CUB Hopital Erasme Laboratoire d'Anatomie Pathologique	Isabelle Salmon, Nicky D'Haene
EPI_ISL_452142	CUB Hopital Erasme Laboratoire d'Anatomie Pathologique	CUB Hopital Erasme Laboratoire d'Anatomie Pathologique	Isabelle Salmon, Nikcy D'Haene
EPI_ISL_452148, EPI_ISL_452149	CUB Hopital Erasme Laboratoire d'Anatomie Pathologique	CUB Hopital Erasme Laboratoire d'Anatomie Pathologique	Isabelle Salmon, Nicky D'Haene
EPI_ISL_452150	CUB Hopital Erasme Laboratoire d'Anatomie Pathologique	CUB Hopital Erasme Laboratoire d'Anatomie Pathologique	Prof. Isabelle Salmon, Dr Nicky D'Haene
EPI_ISL_452151	CUB Hopital Erasme Laboratoire d'Anatomie Pathologique	CUB Hopital Erasme Laboratoire d'Anatomie Pathologique	Prof. Isabelle Salmon, Dr.Nikcy D'Haene
EPI_ISL_452152	CUB Hopital Erasme Laboratoire d'Anatomie Pathologique	CUB Hopital Erasme Laboratoire d'Anatomie Pathologique	Prof. Isabelle Salmon, Dr Nicky D'Haene
EPI_ISL_452192, EPI_ISL_452193, EPI_ISL_452194, EPI_ISL_452195, EPI_ISL_452196, EPI_ISL_452197, EPI_ISL_452198, EPI_ISL_452199, EPI_ISL_452200, EPI_ISL_452201, EPI_ISL_452202, EPI_ISL_452203, EPI_ISL_452204, EPI_ISL_452205, EPI_ISL_452206, EPI_ISL_452207, EPI_ISL_452208, EPI_ISL_452209, EPI_ISL_452210, EPI_ISL_452211, EPI_ISL_452212, EPI_ISL_452213			
see above	NIV Influenza	NIV Influenza	Potdar V
EPI_ISL_452218, EPI_ISL_452219, EPI_ISL_452220, EPI_ISL_452221, EPI_ISL_452222, EPI_ISL_452223	Goethe University Hospital Frankfurt	Institute for Medical Virology, Goethe University Hospital Frankfurt	Tuna Toptan, Sebastian Hoehl, Sandra Westhaus, Denisa Bojkova, Annemarie Berger, Björn Rotter, Klaus Hoffmeier, Jindrich Cinatl, Sandra Ciesek, and Marek Widera
EPI_ISL_452327, EPI_ISL_452328, EPI_ISL_452329, EPI_ISL_452330, EPI_ISL_452331, EPI_ISL_452332, EPI_ISL_452333, EPI_ISL_452334, EPI_ISL_452335, EPI_ISL_452336, EPI_ISL_452337, EPI_ISL_452338, EPI_ISL_452339, EPI_ISL_452340, EPI_ISL_452341, EPI_ISL_452342, EPI_ISL_452343, EPI_ISL_452344, EPI_ISL_452345, EPI_ISL_452346, EPI_ISL_452347, EPI_ISL_452348, EPI_ISL_452349, EPI_ISL_452350, EPI_ISL_452351, EPI_ISL_452352, EPI_ISL_452353, EPI_ISL_452354, EPI_ISL_452355, EPI_ISL_452356, EPI_ISL_452357, EPI_ISL_452358, EPI_ISL_452359, EPI_ISL_452360, EPI_ISL_452361, EPI_ISL_452362, EPI_ISL_452363, EPI_ISL_452364			
see above	Laboratory of Infectious Diseases Center of Beijing Ditan Hospital	Laboratory of Infectious Diseases Center of Beijing Ditan Hospital	Siyuan Yang, Chengjie Jie, Fengting Yu, Yunxia Tang, Liting Yan, Linghang Wang
EPI_ISL_452473, EPI_ISL_452480, EPI_ISL_452481, EPI_ISL_452484, EPI_ISL_452487, EPI_ISL_452488, EPI_ISL_452492, EPI_ISL_452496, EPI_ISL_452498, EPI_ISL_452500, EPI_ISL_452503, EPI_ISL_452509, EPI_ISL_452521, EPI_ISL_452534			
see above	Clínica Universidad de Navarra. Servicio de Enfermedades Infecciosas y Microbiología clínica	SeqCOVID-SPAIN consortium/IBV(CSIC)	Mirian Fernández-Alonso, Jose Luis del Pozo and SeqCOVID-SPAIN consortium
EPI_ISL_454497, EPI_ISL_454498, EPI_ISL_454499, EPI_ISL_454500, EPI_ISL_454501, EPI_ISL_454502, EPI_ISL_454503, EPI_ISL_454504, EPI_ISL_454505, EPI_ISL_454506, EPI_ISL_454507, EPI_ISL_454508, EPI_ISL_454509, EPI_ISL_454510, EPI_ISL_454511, EPI_ISL_454512, EPI_ISL_454513, EPI_ISL_454514, EPI_ISL_454515, EPI_ISL_454516, EPI_ISL_454517, EPI_ISL_454518, EPI_ISL_454519, EPI_ISL_454520			
see above	RSE "National Center for Biotechnology"	RSE "National Center for Biotechnology"	Alexandr Shevtsov, Ilyas Akhmetollayev, Viktoriya Lutsay, Asylulan Amirgazin, Askar Abdaliyev, Akbota Rakhmetova, Zabira Aushakhmetova, Ruslan Kalendar, Yerlan Ramankulov
EPI_ISL_454521, EPI_ISL_454522, EPI_ISL_454524, EPI_ISL_454525, EPI_ISL_454526, EPI_ISL_454527, EPI_ISL_454528, EPI_ISL_454529, EPI_ISL_454530, EPI_ISL_454531, EPI_ISL_454532, EPI_ISL_454533, EPI_ISL_454534, EPI_ISL_454536, EPI_ISL_454537, EPI_ISL_454540, EPI_ISL_454542, EPI_ISL_454543, EPI_ISL_454544, EPI_ISL_454546, EPI_ISL_454547, EPI_ISL_454549, EPI_ISL_454551, EPI_ISL_454552, EPI_ISL_454556, EPI_ISL_454557, EPI_ISL_454558, EPI_ISL_454560, EPI_ISL_454561, EPI_ISL_454562			
see above	NIV Influenza	NIV Influenza	Potdar V
EPI_ISL_454574	nstitute for Public Health	Laboratory for advanced genomics	Filip Roki, Lovro Trgovec-Greif, Neven Sui, Tomislav Rukavina, Igor Jurak, Oliver Vugrek
EPI_ISL_454578, EPI_ISL_454581, EPI_ISL_454583, EPI_ISL_454588, EPI_ISL_454592	University Hospital for Infectious Diseases "Dr. Fran Mihaljevi", Research Unit	University of Zagreb, Centre for research and knowledge transfer in biotechnology	Ivan-Christian Kurolt, Jelena Ivancic Jelecki, Anamarija Slovic
EPI_ISL_454602	Croatian Institute of Public Health	University of Zagreb, Centre for research and knowledge transfer in biotechnology	Irena Tabain, Tatjana Vilibic-Cavlek, Jelena Ivancic Jelecki, Anamarija Slovic
EPI_ISL_454605, EPI_ISL_454606	Institute for Public Health	Laboratory for advanced genomics	Filip Roki, Lovro Trgovec-Greif, Neven Sui, Tomislav Rukavina, Igor Jurak, Oliver Vugrek
EPI_ISL_454732	Russian State Collection of Viruses	Pathogenic Microorganisms Variability Laboratory	Denis Protsenko, Alexey Shchetinin, Maria Nikiforova, Elena Shidlovskaya, Nadezhda Kuznetsova, Vladimir Gushchin, Inna Dolzhikova, Daria Grousova, Andrey Botikov, Denis Logunov, Alexander Gintsburg, Alexey Mazus
EPI_ISL_454749	Japanese Quarantine Stations	Pathogen Genomics Center, National Institute of Infectious Diseases	Tsuyoshi Sekizuka, Kentaro Itokawa, Rina Tanaka, Masanori Hashino, Tsutomu Kageyama, Shinji Saito, Ikuyo Takayama, Hideki Hasegawa, Takuri Takahashi, Hajime Kamiya, Takuya Yamagishi, Motoi Suzuki, Takaji Wakita, Makoto Kuroda
EPI_ISL_454830, EPI_ISL_454832, EPI_ISL_454833	SMS Medical College, Jaipur	CSIR Institute of Genomics and Integrative Biology	Sudhir Bhandari, Rahul Bhoyar, Mohammed Imran, Mohit Divakar, Disha Sharma, Anshul Kumar, Bani Jolly, Rahul Sahlot, Abhinav Jain, Paras Sehgal, Gyan Ranjan, Vinod Scaria, Sridhar Sivasubbu, Sandeep K Mathur
EPI_ISL_454858, EPI_ISL_454862, EPI_ISL_454865, EPI_ISL_454866, EPI_ISL_454867	Translational Health Science and Technology Institute -ESIC medical college and hospital, Faridabad	THSTI Bioassay laboratory	Saurabh Kumar, Jigme Wangchuk, Anil Kumar Pandey, Asim Das, Guruprasad R. Medigeshi
EPI_ISL_455015	Pandit Deendayal Upadhyay Government Medical College, Rajkot	Gujarat Biotechnology Research Centre	Snehal Bagatharia, Prakash Modi, Sejul Antala, Manish Pattani, Ramesh Pandit, Tejas Shah, Ankit Hinsu, Pritesh Sabara, Apurvasinh Puvar, Janvi Raval, Zarna Patel, Monika Gandhi, Pinal Trivedi, Maharshi Pandya, Amit Kanani, Nidhi Patel, Nitin Savaliya, Raghawendra Kumar, Dinesh Kumar, Zuber Saiyed, Komal Patel, Labdhi Pandya, Neha Rajpara, Bhavesh Modi, Gaurishankar Shrimali, R D Dixit, A M Kadri, Umang Mishra, Chaitanya Joshi, Madhvi Joshi
EPI_ISL_455016	Pandit Deendayal Upadhyay Government Medical College, Rajkot	Gujarat Biotechnology Research Centre	Prakash Modi, Sejul Antala, Manish Pattani, Ramesh Pandit, Tejas Shah, Ankit Hinsu, Pritesh Sabara, Apurvasinh Puvar, Janvi Raval, Zarna Patel, Monika Gandhi, Pinal Trivedi, Maharshi Pandya, Amit Kanani, Nidhi Patel, Nitin Savaliya, Raghawendra Kumar, Dinesh Kumar, Zuber Saiyed, Komal Patel, Labdhi Pandya, Snehal Bagatharia, Afzal Ansari, Bhavesh Modi, Gaurishankar Shrimali, R D Dixit, A M Kadri, Umang Mishra, Chaitanya Joshi, Madhvi Joshi
EPI_ISL_455017	Government Medical College, Vadodara	Gujarat Biotechnology Research Centre	Tanuja Javadekar , R N Daveshwar, Ramesh Pandit, Tejas Shah, Ankit Hinsu, Pritesh Sabara, Apurvasinh Puvar, Janvi Raval, Zarna Patel, Monika Gandhi, Pinal Trivedi, Maharshi Pandya, Amit Kanani, Nidhi Patel, Nitin Savaliya, Raghawendra Kumar, Dinesh Kumar, Zuber Saiyed, Komal Patel, Labdhi Pandya, Snehal Bagatharia, Fenil Patel, Bhavesh Modi, Gaurishankar Shrimali, R D Dixit, A M Kadri, Umang Mishra, Chaitanya Joshi, Madhvi Joshi,
EPI_ISL_455018	Government Medical College, Vadodara	Gujarat Biotechnology Research Centre	R N Daveshwar, Ramesh Pandit, Tejas Shah, Ankit Hinsu, Pritesh Sabara, Apurvasinh Puvar, Janvi Raval, Zarna Patel, Monika Gandhi, Pinal Trivedi, Maharshi Pandya, Amit Kanani, Nidhi Patel, Nitin Savaliya, Raghawendra Kumar, Dinesh Kumar, Zuber Saiyed, Komal Patel, Labdhi Pandya, Snehal Bagatharia, Tanuja Javadekar , Neelam Nathani, Bhavesh Modi, Gaurishankar Shrimali, R D Dixit, A M Kadri, Umang Mishra, Chaitanya Joshi, Madhvi Joshi,
EPI_ISL_455019	Government Medical College, Vadodara	Gujarat Biotechnology Research Centre	Ramesh Pandit, Tejas Shah, Ankit Hinsu, Pritesh Sabara, Apurvasinh Puvar, Janvi Raval, Zarna Patel, Monika Gandhi, Pinal Trivedi, Maharshi Pandya, Amit Kanani, Nidhi Patel, Nitin Savaliya, Raghawendra Kumar, Dinesh Kumar, Zuber Saiyed, Komal Patel, Labdhi Pandya, Snehal Bagatharia, Tanuja Javadekar , R N Daveshwar, Armi Chaudhari, Bhavesh Modi, Gaurishankar Shrimali, R D Dixit, A M Kadri, Umang Mishra, Chaitanya Joshi, Madhvi Joshi,
EPI_ISL_455020	Government Medical College, Vadodara	Gujarat Biotechnology Research Centre	Tejas Shah, Ankit Hinsu, Pritesh Sabara, Apurvasinh Puvar, Janvi Raval, Zarna Patel, Monika Gandhi, Pinal Trivedi, Maharshi Pandya, Amit Kanani, Nidhi Patel, Nitin Savaliya, Raghawendra Kumar, Dinesh Kumar, Zuber Saiyed, Komal Patel, Labdhi Pandya, Snehal Bagatharia, Tanuja Javadekar , R N Daveshwar, Ramesh Pandit, Bhavya Jindal, Bhavesh Modi, Gaurishankar Shrimali, R D Dixit, A M Kadri, Umang Mishra, Chaitanya Joshi, Madhvi Joshi,
EPI_ISL_455021	Government Medical College, Vadodara	Gujarat Biotechnology Research Centre	Ankit Hinsu, Pritesh Sabara, Apurvasinh Puvar, Janvi Raval, Zarna Patel, Monika Gandhi, Pinal Trivedi, Maharshi Pandya, Amit Kanani, Nidhi Patel, Nitin Savaliya, Raghawendra Kumar, Dinesh Kumar, Zuber Saiyed, Komal Patel, Labdhi Pandya, Snehal Bagatharia, Tanuja Javadekar , R N Daveshwar, Ramesh Pandit, Tejas Shah, Camellia Chakraborty, Bhavesh Modi, Gaurishankar Shrimali, R D Dixit, A M Kadri, Umang Mishra, Chaitanya Joshi, Madhvi Joshi,
EPI_ISL_455022	Government Medical College, Vadodara	Gujarat Biotechnology Research Centre	Pritesh Sabara, Apurvasinh Puvar, Janvi Raval, Zarna Patel, Monika Gandhi, Pinal Trivedi, Maharshi Pandya, Amit Kanani, Nidhi Patel, Nitin Savaliya,

			Raghawendra Kumar, Dinesh Kumar, Zuber Saiyed, Komal Patel, Labdhi Pandya, Snehal Bagatharia, Tanuja Javadekar , R N Daveshwar, Ramesh Pandit, Tejas Shah, Ankit Hinsu, Siddhant Kumar, Bhavesh Modi, Gaurishankar Shrimali, R D Dixit, A M Kadri, Umang Mishra, Chaitanya Joshi, Madhvi Joshi,
EPI_ISL_455023	Government Medical College, Vadodara	Gujarat Biotechnology Research Centre	Apurvasinh Puvar, Janvi Raval, Zarna Patel, Monika Gandhi, Pinal Trivedi, Maharshi Pandya, Amit Kanani, Nidhi Patel, Nitin Savaliya, Raghawendra Kumar, Dinesh Kumar, Zuber Saiyed, Komal Patel, Labdhi Pandya, Snehal Bagatharia, Tanuja Javadekar , R N Daveshwar, Ramesh Pandit, Tejas Shah, Ankit Hinsu, Pritesh Sabara, Priyanka P Vatsa, Bhavesh Modi, Gaurishankar Shrimali, R D Dixit, A M Kadri, Umang Mishra, Chaitanya Joshi, Madhvi Joshi,
EPI_ISL_455024	Government Medical College, Vadodara	Gujarat Biotechnology Research Centre	Janvi Raval, Zarna Patel, Monika Gandhi, Pinal Trivedi, Maharshi Pandya, Amit Kanani, Nidhi Patel, Nitin Savaliya, Raghawendra Kumar, Dinesh Kumar, Zuber Saiyed, Komal Patel, Labdhi Pandya, Snehal Bagatharia, Tanuja Javadekar , R N Daveshwar, Ramesh Pandit, Tejas Shah, Ankit Hinsu, Pritesh Sabara, Apurvasinh Puvar, Pooja P Doshi, Bhavesh Modi, Gaurishankar Shrimali, R D Dixit, A M Kadri, Umang Mishra, Chaitanya Joshi, Madhvi Joshi,
EPI_ISL_455025	Government Medical College, Vadodara	Gujarat Biotechnology Research Centre	Zarna Patel, Monika Gandhi, Pinal Trivedi, Maharshi Pandya, Amit Kanani, Nidhi Patel, Nitin Savaliya, Raghawendra Kumar, Dinesh Kumar, Zuber Saiyed, Komal Patel, Labdhi Pandya, Snehal Bagatharia, Tanuja Javadekar , R N Daveshwar, Ramesh Pandit, Tejas Shah, Ankit Hinsu, Pritesh Sabara, Apurvasinh Puvar, Janvi Raval, Akanksha Verma, Bhavesh Modi, Gaurishankar Shrimali, R D Dixit, A M Kadri, Umang Mishra, Chaitanya Joshi, Madhvi Joshi,
EPI_ISL_455026	Government Medical College, Vadodara	Gujarat Biotechnology Research Centre	Monika Gandhi, Pinal Trivedi, Maharshi Pandya, Amit Kanani, Nidhi Patel, Nitin Savaliya, Raghawendra Kumar, Dinesh Kumar, Zuber Saiyed, Komal Patel, Labdhi Pandya, Snehal Bagatharia, Tanuja Javadekar , R N Daveshwar, Ramesh Pandit, Tejas Shah, Ankit Hinsu, Pritesh Sabara, Apurvasinh Puvar, Janvi Raval, Zarna Patel, Priti Pandita, Bhavesh Modi, Gaurishankar Shrimali, R D Dixit, A M Kadri, Umang Mishra, Chaitanya Joshi, Madhvi Joshi,
EPI_ISL_455027	Government Medical College, Vadodara	Gujarat Biotechnology Research Centre	Pinal Trivedi, Maharshi Pandya, Amit Kanani, Nidhi Patel, Nitin Savaliya, Raghawendra Kumar, Dinesh Kumar, Zuber Saiyed, Komal Patel, Labdhi Pandya, Snehal Bagatharia, Tanuja Javadekar , R N Daveshwar, Ramesh Pandit, Tejas Shah, Ankit Hinsu, Pritesh Sabara, Apurvasinh Puvar, Janvi Raval, Zarna Patel, Monika Gandhi, Pragya Sharma, Bhavesh Modi, Gaurishankar Shrimali, R D Dixit, A M Kadri, Umang Mishra, Chaitanya Joshi, Madhvi Joshi,
EPI_ISL_455312	Microbiology Unit, Department of Pathology & Laboratory Medicine, IIUM Medical Centre	SEA Microbiome Unit, Faculty of Industrial Sciences & Technology, Universiti Malaysia Pahang	Norhidayah Binti Kamarudin, Ahmad Hafiz Bin Zulkifly, Hajar Fauzan Ahmad, Muhammad Adam Lee Abdullah, Mohd Fazli Farida Asras, Ahmad Mahfuz Gazali, Mohd Nazli Bin Kamarulzaman, IIUM Medical Centre Covid19 Taskforce, UMP Covid19 Team
EPI_ISL_455313	Microbiology Unit, Department of Pathology & Laboratory Medicine, IIUM Medical Centre	SEA Microbiome Unit, Faculty of Industrial Sciences & Technology, Universiti Malaysia Pahang	Hajar Fauzan Ahmad, Norhidayah Kamarudin, Ahmad Hafiz Zulkifly, IIUM Medical Centre Covid19 Taskforce, UMP Covid19 Team
EPI_ISL_455314	Hospital Virgen del Rocio	Instituto de Salud Carlos III	Iglesias-Caballero, M. Molinero Calamita, M. González-Esguevillas, M. Camarero, S. Pozo, F. Casas, I. Jiménez, P. Jiménez, M. Zaballos, A. Monzón, S. Varona, S. Juliá, M. Cuesta, I, J. Lepe
EPI_ISL_455315, EPI_ISL_455316, EPI_ISL_455317, EPI_ISL_455318, EPI_ISL_455319, EPI_ISL_455320, EPI_ISL_455321, EPI_ISL_455322, EPI_ISL_455324	Hospital Virgen de las Nieves	Instituto de Salud Carlos III	Iglesias-Caballero, M. Molinero Calamita, M. González-Esguevillas, M. Camarero, S. Pozo, F. Casas, I. Jiménez, P. Jiménez, M. Zaballos, A. Monzón, S. Varona, S. Juliá, M. Cuesta, I, S. Sanbonmatsu
EPI_ISL_455325	Hospital Universitario de Canarias	Instituto de Salud Carlos III	Iglesias-Caballero, M. Molinero Calamita, M. González-Esguevillas, M. Camarero, S. Pozo, F. Casas, I. Jiménez, P. Jiménez, M. Zaballos, A. Monzón, S. Varona, S. Juliá, M. Cuesta, I, B. Castro
EPI_ISL_455326	Hospital Universitario Insular de Gran Canaria	Instituto de Salud Carlos III	Iglesias-Caballero, M. Molinero Calamita, M. González-Esguevillas, M. Camarero, S. Pozo, F. Casas, I. Jiménez, P. Jiménez, M. Zaballos, A. Monzón, S. Varona, S. Juliá, M. Cuesta, I, A. Hernández
EPI_ISL_455327	Consejeria de Sanidad y Asuntos Sociales	Instituto de Salud Carlos III	Iglesias-Caballero, M. Molinero Calamita, M. González-Esguevillas, M. Camarero, S. Pozo, F. Casas, I. Jiménez, P. Jiménez, M. Zaballos, A. Monzón, S. Varona, S. Juliá, M. Cuesta, I, G. Gutiérrez
EPI_ISL_455328, EPI_ISL_455329, EPI_ISL_455330, EPI_ISL_455331	Complejo Hospitalario Universitario La Coruna	Instituto de Salud Carlos III	Iglesias-Caballero, M. Molinero Calamita, M. González-Esguevillas, M. Camarero, S. Pozo, F. Casas, I. Jiménez, P. Jiménez, M. Zaballos, A. Monzón, S. Varona, S. Juliá, M. Cuesta, I, M.A Canizares
EPI_ISL_455332	Xerencia de Xestión Integrada de Pontevedra e o Salnés	Instituto de Salud Carlos III	Iglesias-Caballero, M. Molinero Calamita, M. González-Esguevillas, M. Camarero, S. Pozo, F. Casas, I. Jiménez, P. Jiménez, M. Zaballos, A. Monzón, S. Varona, S. Juliá, M. Cuesta, I, M. Garcia
EPI_ISL_455333	Complejo Hospitalario Universitario de Santiago	Instituto de Salud Carlos III	Iglesias-Caballero, M. Molinero Calamita, M. González-Esguevillas, M. Camarero, S. Pozo, F. Casas, I. Jiménez, P. Jiménez, M. Zaballos, A. Monzón, S. Varona, S. Juliá, M. Cuesta, I, J. Llovo
EPI_ISL_455334, EPI_ISL_455335	Complejo Hospitalario de Orense	Instituto de Salud Carlos III	Iglesias-Caballero, M. Molinero Calamita, M. González-Esguevillas, M. Camarero, S. Pozo, F. Casas, I. Jiménez, P. Jiménez, M. Zaballos, A. Monzón, S. Varona, S. Juliá, M. Cuesta, I, M. Paz
EPI_ISL_455336, EPI_ISL_455337, EPI_ISL_455338, EPI_ISL_455339, EPI_ISL_455340, EPI_ISL_455341, EPI_ISL_455342, EPI_ISL_455343	Hospital San Pedro	Instituto de Salud Carlos III	Iglesias-Caballero, M. Molinero Calamita, M. González-Esguevillas, M. Camarero, S. Pozo, F. Casas, I. Jiménez, P. Jiménez, M. Zaballos, A. Monzón, S. Varona, S. Juliá, M. Cuesta, I, C. Alonso
EPI_ISL_455344, EPI_ISL_455345, EPI_ISL_455346, EPI_ISL_455347, EPI_ISL_455348, EPI_ISL_455349	Hospital Comarcal de Melilla	Instituto de Salud Carlos III	Iglesias-Caballero, M. Molinero Calamita, M. González-Esguevillas, M. Camarero, S. Pozo, F. Casas, I. Jiménez, P. Jiménez, M. Zaballos, A. Monzón, S. Varona, S. Juliá, M. Cuesta, I, I. Pérez
EPI_ISL_455350, EPI_ISL_455351	Hospital Txagorritxu	Instituto de Salud Carlos III	Iglesias-Caballero, M. Molinero Calamita, M. González-Esguevillas, M. Camarero, S. Pozo, F. Casas, I. Jiménez, P. Jiménez, M. Zaballos, A. Monzón, S. Varona, S. Juliá, M. Cuesta, I, C. Gómez
EPI_ISL_455352, EPI_ISL_455353, EPI_ISL_455354	Hospital de Cruces	Instituto de Salud Carlos III	Iglesias-Caballero, M. Molinero Calamita, M. González-Esguevillas, M. Camarero, S. Pozo, F. Casas, I. Jiménez, P. Jiménez, M. Zaballos, A. Monzón, S. Varona, S. Juliá, M. Cuesta, I, M. Aranzamendi
EPI_ISL_455362	Nigeria Centre for Disease Control (NCDC)	African Centre of Excellence for Genomics of Infectious Diseases (ACEGID), Redeemer's University, Ede, Osun State, Nigeria	Oluniyi P.E., Ajogbasile F.V., Kayode A., Olawoye I., Uwanibe J., Oguzie J., Olumade T., Folarin O.A., Ihekweazu C., Happi C.T.
EPI_ISL_455412, EPI_ISL_455413, EPI_ISL_455419	Nigeria Centre for Disease Control (NCDC)	African Centre of Excellence for Genomics of Infectious Diseases (ACEGID), Redeemer's University, Ede, Osun State, Nigeria	Oluniyi P.E., Ajogbasile F.V., Kayode A., Oguzie J., Olawoye I., Uwanibe J., Olumade T., Folarin O.A., Ihekweazu C., Happi C.T.
EPI_ISL_455422	Nigeria Centre for Disease Control	African Centre of Excellence for Genomics of Infectious Diseases (ACEGID), Redeemer's University, Ede, Osun State, Nigeria	Oluniyi P.E., Ajogbasile F.V., Kayode A., Oguzie J., Olawoye I., Uwanibe J., Olumade T., Folarin O.A., Ihekweazu C., Happi C.T.
EPI_ISL_455423, EPI_ISL_455424	Nigeria Centre for Disease Control (NCDC)	African Centre of Excellence for Genomics of Infectious Diseases (ACEGID), Redeemer's University, Ede, Osun State, Nigeria	Oluniyi P.E., Ajogbasile F.V., Kayode A., Oguzie J., Olawoye I., Uwanibe J., Olumade T., Folarin O.A., Ihekweazu C., Happi C.T.
EPI_ISL_455426	Nigeria Centre for Disease Control	African Centre of Excellence for Genomics of Infectious Diseases (ACEGID), Redeemer's University, Ede, Osun State, Nigeria	Oluniyi P.E., Ajogbasile F.V., Kayode A., Oguzie J., Olawoye I., Uwanibe J., Olumade T., Folarin O.A., Ihekweazu C., Happi C.T.
EPI_ISL_455429, EPI_ISL_455431	Nigeria Centre for Disease Control (NCDC)	African Centre of Excellence for Genomics of Infectious Diseases (ACEGID), Redeemer's University, Ede, Osun State, Nigeria	Oluniyi P.E., Ajogbasile F.V., Kayode A., Oguzie J., Olawoye I., Uwanibe J., Olumade T., Folarin O.A., Ihekweazu C., Happi C.T.
EPI_ISL_455434	Instituto de Diagnostico y Referencia Epidemiologicos (INDRE)	Instituto de Diagnostico y Referencia Epidemiologicos (INDRE)	Taboada Ramírez Blanca. Ramirez-Gonzalez Ernesto, Garces-Ayala Fabiola, Araiza-Rodriguez Adnan, Mendieta-Condado Edgar, Rodriguez-Maldonado Abril, Wong-Arambula Claudia, Barrera-Badillo Gisela, Hernandez-Rivas Lucia, Lopez-Martinez Irma.

EPI_ISL_455435	Instituto de Diagnostico y Referencia Epidemiologicos (INDRE)	Instituto de Diagnostico y Referencia Epidemiologicos (INDRE)	Garces-Ayala Fabiola. Taboada Ramírez Blanca. Ramirez-Gonzalez Ernesto, Araiza-Rodriguez Adnan , Mendieta-Condado Edgar, Rodriguez-Maldonado Abril, Wong-Arambula Claudia, Barrera-Badillo Gisela, Hernandez-Rivas Lucia, Lopez-Martinez Irma
EPI_ISL_455436	Instituto de Diagnostico y Referencia Epidemiologicos (INDRE)	Instituto de Diagnostico y Referencia Epidemiologicos (INDRE)	Garces-Ayala Fabiola. Taboada Ramírez Blanca. Ramirez-Gonzalez Ernesto, Araiza-Rodriguez Adnan, Mendieta-Condado Edgar, Rodriguez-Maldonado Abril, Wong-Arambula Claudia, Barrera-Badillo Gisela, Hernandez-Rivas Lucia, Lopez-Martinez Irma.
EPI_ISL_455437, EPI_ISL_455438	Instituto de Diagnostico y Referencia Epidemiologicos (INDRE)	Instituto de Diagnostico y Referencia Epidemiologicos (INDRE)	Araiza-Rodriguez Adnan, Garces-Ayala Fabiola. Ramirez-Gonzalez Ernesto, Mendieta-Condado Edgar, Rodriguez-Maldonado Abril, Wong-Arambula Claudia, Barrera-Badillo Gisela, Hernandez-Rivas Lucia, Lopez-Martinez Irma, Taboada Ramírez Blanca.
EPI_ISL_455439	Instituto de Diagnostico y Referencia Epidemiologicos (INDRE)	Instituto de Diagnostico y Referencia Epidemiologicos (INDRE)	Mendieta-Condado Edgar, Araiza-Rodriguez Adnan, Garces-Ayala Fabiola, Rodriguez-Maldonado Abril, Wong-Arambula Claudia, Barrera-Badillo Gisela, Hernandez-Rivas Lucia, Lopez-Martinez Irma, Taboada Ramirez Blanca, Ramirez-Gonzalez Ernesto.
EPI_ISL_455455	Instituto de Diagnostico y Referencia Epidemiologicos (INDRE)	Instituto de Diagnostico y Referencia Epidemiologicos (INDRE)	Rodriguez-Maldonado Abril, Mendieta-Condado Edgar, Araiza-Rodriguez Adnan, Garces-Ayala Fabiola. Taboada Ramirez Blanca. Ramirez-Gonzalez Ernesto, Barrera-Badillo Gisela, Hernandez-Rivas Lucia, Lopez-Martinez Irma, Wong-Arambula Claudia.
EPI_ISL_455456	Instituto de Diagnostico y Referencia Epidemiologicos (INDRE)	Instituto de Diagnostico y Referencia Epidemiologicos (INDRE)	Rodriguez-Maldonado Abril, Mendieta-Condado Edgar, Araiza-Rodriguez Adnan, Garces-Ayala Fabiola. Taboada Ramírez Blanca. Ramirez-Gonzalez Ernesto, , Barrera-Badillo Gisela, Hernandez-Rivas Lucia, Lopez-Martinez Irma, Wong-Arambula Claudia.
EPI_ISL_455460, EPI_ISL_455461, EPI_ISL_455462, EPI_ISL_455463, EPI_ISL_455464, EPI_ISL_455465, EPI_ISL_455466, EPI_ISL_455467	Jiangxi Province Center for Disease Control and Prevention	Jiangxi Province Center for Disease Control and Prevention	JianXiong Li,Ying Xiong,Tian Gong,Yong Shi,Jun Zhou,Fang Xiao,ShiWen Liu,XiaoQing Liu,Gang Xu,DaJin Xiao,Xin Ran,YanNi Zhang
EPI_ISL_455468, EPI_ISL_455469, EPI_ISL_455470, EPI_ISL_455471, EPI_ISL_455472, EPI_ISL_455473, EPI_ISL_455474	Laboratory for Respiratory Viruses, Cantacuzino National Military-Medical Institute for Research and Development	Cantacuzino Institute	M.Lazar, L.Ustea, A.Cretu, Tim Durfee
EPI_ISL_455475, EPI_ISL_455477	Laboratory for Respiratory Viruses, Cantacuzino National Military-Medical Institute for Research and Development	Cantacuzino Institute	M.Lazar, L.Ustea, A.Cretu, T.Durfee
EPI_ISL_455566, EPI_ISL_455567	Institute for Public Health	Laboratory for advanced genomics	Filip Roki, Lovro Trgovec-Greif, Neven Sui, Tomislav Rukavina, Igor Jurak, Oliver Vugrek
EPI_ISL_455694, EPI_ISL_455695, EPI_ISL_455696, EPI_ISL_455697, EPI_ISL_455698, EPI_ISL_455699, EPI_ISL_455700, EPI_ISL_455701, EPI_ISL_455702, EPI_ISL_455703, EPI_ISL_455704, EPI_ISL_455705, EPI_ISL_455706, EPI_ISL_455707, EPI_ISL_455708, EPI_ISL_455709, EPI_ISL_455710, EPI_ISL_455711, EPI_ISL_455712			
see above	National Hospital of Tropical Diseases	Oxford University Clinical Research Unit, Hanoi, Vietnam	Nguyen Thi Tam, Van Dinh Trang, Nguyen Thu Trang, Nguyen Thi Ngoc Diep, Le Nguyen Minh Hoa, Pham Ngoc Thach, H. Rogier van Doorn, on behalf of the OUCRU COVID-19 research group
EPI_ISL_455714, EPI_ISL_455716, EPI_ISL_455717, EPI_ISL_455718	National Hospital of Tropical Diseases	Oxford University Clinical Research Unit, Hanoi, Vietnam	Nguyen Thi Tam, Van Dinh Trang, Nguyen Thi Hong Thuong, Vu Thi Ngoc Bich, Nguyen Thu Trang, Nguyen Thi Ngoc Diep, Le Nguyen Minh Hoa, Pham Ngoc Thach, H. Rogier van Doorn, on behalf of the OUCRU COVID-19 research group
EPI_ISL_455790, EPI_ISL_455791	Institute for Medical Research, Infectious Disease Research Centre, National Institutes of Health, Ministry of Health Malaysia	Malaysia Genome Institute	Mohd Noor Mat Isa, Irni Suhayu Sapian, Yusuf Muhammad Noor, Jeyanthi Suppiah, Nurhezreen Md Iqbal, Enizza Kasim, Zarina Mohd Zawawi, Siti Noraini Othman, Mohd Faizal Abu Bakar, Shamsidar Sopie, Azrin Ahmad, Ravindran Thayan, Norazah Ahmad, Tahir Aris, Shahrul Hisham Zainal Ariffin
EPI_ISL_455792, EPI_ISL_455793	Institute for Medical Research, Infectious Disease Research Centre, National Institutes of Health, Ministry of Health Malaysia	Malaysia Genome Institute	Mohd Noor Mat Isa, Irni Suhayu Sapian, Yusuf Muhammad Noor, Jeyanthi Suppiah, Nurhezreen Md Iqbal, Enizza Kasim, Zarina Mohd Zawawi, Siti Noraini Othman, Mohd Faizal Abu Bakar, Shamsidar Sopie, Azrin Ahmad, Ravindran Thayan, Norazah Ahmad, Tahir Aris, Shahrul Hisham Zainal Ariffin
EPI_ISL_456117, EPI_ISL_456119, EPI_ISL_456120, EPI_ISL_456126, EPI_ISL_456127, EPI_ISL_456138, EPI_ISL_456145, EPI_ISL_456146, EPI_ISL_456147, EPI_ISL_456148, EPI_ISL_456149, EPI_ISL_456150, EPI_ISL_456151, EPI_ISL_456152, EPI_ISL_456155			
see above	Instituto Nacional de Salud - Unidad de Secuenciación y Análisis Genómico	Instituto Nacional de Salud, Universidad Cooperativa de Colombia, Instituto Alexander von Humboldt, Imperial College-London, London School of Hygiene & Tropical Medicine	Katherine Laiton-Donato, Diego A. Álvarez-Díaz, Carlos Franco-Muñoz, Jose A. Usme-Ciro, Gloria Puerto, Nicolas D. Franco-Sierra, Mailyn A.Gonzalez, Zulma M. Cucunubá, Christian Julian VillabonaArenas, Liz Villabona-Arenas, Sussy Echeverria, Astrid C. Flórez, Sergio Gomez-Rangel, Luz Dary Rodriguez, Juliana Barbosa, Erika Ospitia, Diana Marcela Walteros-Acero, Martha Lucia Ospina Martinez, Marcela Mercado-Reyes.
EPI_ISL_457702	Oman-NIC	Microbiology laboratory- Sultan Qaboos University Hospital	Fahad Zadjali, Samira Al-Maruqi, Amina Al Jardani, Khulood Al-Mammary, Hanan Al-kindi, Fatma BaAlawi, Hamida AL Barwani, Zeyana AL-Dahmani, Intisar Al-Shukri, Aisha Al-Busaidi, Aisha Al-Amri, Ahlam Al-Amri, Mohammed Al-Tobi, Samiha Al Kharusi, Abdulla Balkhair
EPI_ISL_457703	Oman-NIC	Department of Microbiology and Immunology- SQUH	Fahad Zadjali, Samira Al-Maruqi, Amina Al Jardani, Khulood Al-Mammary, Hanan Al-kindi, Fatma BaAlawi, Hamida AL Barwani, Zeyana AL-Dahmani, Intisar Al-Shukri, Aisha Al-Busaidi, Aisha Al-Amri, Ahlam Al-Amri, Mohammed Al-Tobi, Samiha Al Kharusi, Abdulla Balkhair
EPI_ISL_457705	OMAN-NIC	Department of Microbiology and Immunology- SQUH	Fahad Zadjali, Samira Al-Maruqi, Amina Al Jardani, Khulood Al-Mammary, Hanan Al-kindi, Fatma BaAlawi, Hamida AL Barwani, Zeyana AL-Dahmani, Intisar Al-Shukri, Aisha Al-Busaidi, Aisha Al-Amri, Ahlam Al-Amri, Mohammed Al-Tobi, Samiha Al Kharusi, Abdulla Balkhair
EPI_ISL_457707	Oman-NIC	Department of Microbiology and Immunology- SQUH	Fahad Zadjali, Samira Al-Maruqi, Amina Al Jardani, Khulood Al-Mammary, Hanan Al-kindi, Fatma BaAlawi, Hamida AL Barwani, Zeyana AL-Dahmani, Intisar Al-Shukri, Aisha Al-Busaidi, Aisha Al-Amri, Ahlam Al-Amri, Mohammed Al-Tobi, Samiha Al Kharusi, Abdulla Balkhair
EPI_ISL_457726, EPI_ISL_457730	TSGH-CP molecular lab	TSGH-CP molecular lab	Cherng-Lih Perng, Ming-Jr JIAN, Chih-Kai Chang, Jung-Chung Lin, Kuo-Ming Yeh, Chien-Wen Chen, Sheng-Kang Chiu, Hsing-Yi Chung, Shih-Hung Tsai, Kuo-Sheng Hung, Tien-Yao Chang, Feng-Yee Chang, Hung-Sheng Shang
EPI_ISL_457750	Centogene AG	Centogene AG	Prof. Dr. Peter Bauer, Dr. Krishna Kumar Kandaswamy
EPI_ISL_457824	Bezmialem Vakif University, Dept Microbiology, Medical School, Fatih, Istanbul, Turkey	Bezmialem Vakif University, Medical School & Beykoz Institute of Life Sciences & Biotechnology	Mehmet Z. Doymaz, Merve Kalkan, Nesibe Cetin, Elif Karaaslan, Bilge Sumbul, Filiz Guney
EPI_ISL_457997	Oman-NIC	Oman-NIC	Samira Al-Maruqi, Fahad Zadjali, Amina Al Jardani, Khulood Al-Mammary, Hanan Al-kindi, Fatma BaAlawi, Hamida AL Barwani, Zeyana AL-Dahmani, Intisar Al-Shukri, Aisha Al-Busaidi, Aisha Al-Amri, Ahlam Al-Amri, Mohammed Al-Tobi, Samiha Al Kharusi, Abdulla Balkhair
EPI_ISL_458029	TSGH-CP molecular lab	TSGH-CP molecular lab	Cherng-Lih Perng, Ming-Jr JIAN, Chih-Kai Chang, Jung-Chung Lin, Kuo-Ming Yeh, Chien-Wen Chen, Sheng-Kang Chiu, Hsing-Yi Chung, Shih-Hung Tsai, Kuo-Sheng Hung, Tien-Yao Chang, Feng-Yee Chang, Hung-Sheng Shang
EPI_ISL_458079	Mitra Keluarga Hospital Kenjeran	Institute of Tropical Disease, Universitas Airlangga	Aldise M Nastri, Jezzy R Dewantari, Rima R Prasetya, Krisnoadi Rahardjo, Anastasia W Jefuna, Gatot Soegiarto, Laksmi Wulandari, Retno A Setyoningrum, Resti Yudhawati, Yohko K Shimizu, Mitsuhiro Nishimura, Yasuko Mori, Soetjipto, Kazufumi Shimizu, Maria I Lusida
EPI_ISL_458081	RSUD Bangil Pasuruan	Institute of Tropical Disease, Universitas Airlangga	Jezzy R Dewantari, Rima R Prasetya, Krisnoadi Rahardjo, Aldise M Nastri, Arma Roosalina, Gatot Soegiarto, Laksmi Wulandari, Retno A Setyoningrum, Resti Yudhawati, Yohko K Shimizu, Mitsuhiro Nishimura, Yasuko Mori, Soetjipto, Kazufumi Shimizu, Maria I Lusida
EPI_ISL_458083	Adi Husada Undaan Hospital	Institute of Tropical Disease, Universitas Airlangga	Rima R Prasetya, Krisnoadi Rahardjo, Aldise M Nastri, Jezzy R Dewantari, Irawati Marga, Gatot Soegiarto, Laksmi Wulandari, Retno A Setyoningrum, Resti Yudhawati, Yohko K Shimizu, Mitsuhiro Nishimura, Yasuko Mori, Soetjipto, Kazufumi Shimizu, Maria I Lusida
EPI_ISL_458103	Gujarat Biotechnology Research Centre	Gujarat Biotechnology Research Centre	Ramesh Pandit, Tejas Shah, Ankit Hinsu, Pritesh Sabara, Apurvasinh Puvar, Janvi Raval, Zarna Patel, Monika Gandhi, Pinal Trivedi, Maharshi Pandya, Amit Kanani, Nidhi Patel, Nitin Savaliya, Raghawendra Kumar, Dinesh Kumar, Zuber Saiyed, Komal Patel, Labdhi Pandya, Snehal Bagatharia, Armi Chaudhari, Bhavesh Modi, Gaurishankar Shrimali, R D Dixit, A M Kadri, Umang Mishra, Chaitanya Joshi, Madhvi Joshi, , , , ,
EPI_ISL_458104	Gujarat Biotechnology Research Centre	Gujarat Biotechnology Research Centre	Tejas Shah, Ankit Hinsu, Pritesh Sabara, Apurvasinh Puvar, Janvi Raval, Zarna Patel, Monika Gandhi, Pinal Trivedi, Maharshi Pandya, Amit Kanani, Nidhi Patel, Nitin Savaliya, Raghawendra Kumar, Dinesh Kumar, Zuber Saiyed, Komal Patel, Labdhi Pandya, Snehal Bagatharia, Ramesh Pandit, Bhavya Jindal, Bhavesh Modi, Gaurishankar Shrimali, R D Dixit, A M Kadri, Umang Mishra, Chaitanya Joshi, Madhvi Joshi, , , , ,
EPI_ISL_458105	Gujarat Biotechnology Research Centre	Gujarat Biotechnology Research Centre	Ankit Hinsu, Pritesh Sabara, Apurvasinh Puvar, Janvi Raval, Zarna Patel, Monika Gandhi, Pinal Trivedi, Maharshi Pandya, Amit Kanani, Nidhi Patel, Nitin Savaliya, Raghawendra Kumar, Dinesh Kumar, Zuber Saiyed, Komal Patel, Labdhi Pandya, Snehal Bagatharia, Ramesh Pandit, Tejas Shah, Camellia Chakraborty, Bhavesh Modi, Gaurishankar Shrimali, R D Dixit, A M Kadri, Umang Mishra, Chaitanya Joshi, Madhvi Joshi, , , , ,

EPI_ISL_458116, EPI_ISL_458117, EPI_ISL_458118, EPI_ISL_458120	Oman National Influenza Centre	Department of Microbiology and Immunology-SQUH	Fahad Zadjali, Samira Al-Maruqi, Amina Al Jardani, Khulood Al-Mammary, Hanan Al-kindi, Fatma BaAlawi, Hamida AL Barwani, Zeyana AL-Dahmani, Intisar Al-Shukri, Aisha Al-Busaidi, Aisha Al-Amri, Ahlam Al-Amri, Mohammed Al-Tobi, Samiha Al Kharusi, Abdulla Balkhair
EPI_ISL_458133	National Institute of Biotechnology	Bioinformatics Division, National Institute of Biotechnology	Mohammad Uzzal Hossain, Md. Moniruzzaman, Md. Salim Khan, Md. Nazrul Islam, Md. Hadisur Rahman, Arittra Bhattacharjee, Md. Ruhul Amin, Asif Rashid, Chaman Ara Keya, Keshob Chandra Das, Md. Salimullah
EPI_ISL_458139	Evandro Chagas Institute	Evandro Chagas Institute	Santos, M.C.; Silva, A.M.; Junior, W.D.C.; Barbagelata, L.S.; Ferreira, J.A.; Sousa, E.M.A.; da Silva, P.S.; Resque, H.R; Martins, L.C.; Sousa Junior, E.C.;Viana, G.M.R
EPI_ISL_459866, EPI_ISL_459867, EPI_ISL_459868, EPI_ISL_459869, EPI_ISL_459871, EPI_ISL_459872, EPI_ISL_459873, EPI_ISL_459874, EPI_ISL_459875, EPI_ISL_459877, EPI_ISL_459879, EPI_ISL_459880, EPI_ISL_459881, EPI_ISL_459882, EPI_ISL_459883, EPI_ISL_459885, EPI_ISL_459886, EPI_ISL_459887, EPI_ISL_459888, EPI_ISL_459889, EPI_ISL_459890, EPI_ISL_459891, EPI_ISL_459892			
see above	Kingston Health Sciences Center	Queen's Genomics Lab at Ongwanada (Q-GLO)	Sjaarda CP, Rustom N, Huang D, Perez-Patrigeon S, Hudson ML, Wong H,Guan H, Ayub M, Soares CN, Colautti R, Evans GA, Sheth P
EPI_ISL_459911	Devki Devi Foundation, a unit of Max Healthcare	CSIR-IGIB/Max	Rajesh Pandey#, Samreen Siddiqui, Pooja Sharma, Bansidhar Tarai, Vivekanand A, Bharathram Uppili, Saruchi Wadhwa, Nishu Tyagi, Mitali Mukerji, Poonam Das, Sujeet Jha, Mohammed Faruq, Vinita Jha, Anurag Agrawal
EPI_ISL_459914, EPI_ISL_459915, EPI_ISL_459918, EPI_ISL_459921, EPI_ISL_459922, EPI_ISL_459923, EPI_ISL_459932, EPI_ISL_459933, EPI_ISL_459934, EPI_ISL_459935, EPI_ISL_459936, EPI_ISL_459937, EPI_ISL_459938, EPI_ISL_459939, EPI_ISL_459940, EPI_ISL_459941, EPI_ISL_459942, EPI_ISL_459943			
see above	Devki Devi Foundation, a unit of Max Healthcare	CSIR-IGIB/Max	Rajesh Pandey#, Samreen Siddiqui, Pooja Sharma, Bansidhar Tarai, Vivekanand A, Bharathram Uppili, Saruchi Wadhwa, Nishu Tyagi, Mitali Mukerji, Bansidhar Tarai, Poonam Das, Sujeet Jha, Mohammed Faruq, Vinita Jha, Anurag Agrawal
EPI_ISL_459953	Institute for Medical Research, Infectious Disease Research Centre, National Institutes of Health, Ministry of Health Malaysia	Institute for Medical Research Infectious Disease Research Centre, National Institutes of Health, Ministry of Health Malaysia	Suppiah J, Mohd-Zawawi Z, Kamel KA, Ellan K, Kalyanasundram J, Mohd-Zain R, Thayan R
EPI_ISL_459954, EPI_ISL_459955, EPI_ISL_459956	Institute for Medical Research, Infectious Disease Research Centre, National Institutes of Health, Ministry of Health Malaysia	Institute for Medical Research, Infectious Disease Research Centre, National Institutes of Health, Ministry of Health Malaysia	Suppiah J, Mohd-Zawawi Z, Kamel KA, Ellan K, Kalyanasundram J, Mohd-Zain R, Thayan R
EPI_ISL_459962, EPI_ISL_459963, EPI_ISL_459964	Centogene AG	Centogene AG	Prof. Dr. Peter Bauer, Dr. Krishna Kumar Kandaswamy
EPI_ISL_460079	Molecular Virology Unit, Fondazione IRCCS Policlinico San Matteo , Pavia	Laboratory of Virology, INMI Lazzaro Spallanzani IRCCS	Barbara Bartolini, Cesare E.M. Gruber, Maria R. Capobianchi, Martina Rueca, Antonio Piralla, Fausto Baldanti, Antonino Di Caro
EPI_ISL_460080	Molecular Virology Unit, Fondazione IRCCS Policlinico San Matteo , Pavia	Laboratory of Virology, INMI Lazzaro Spallanzani IRCCS	Antonio Piralla, Barbara Bartolini, Fausto Baldanti, Martina Rueca, Antonino Di Caro, Cesare E.M. Gruber, Maria R. Capobianchi
EPI_ISL_460081	Molecular Virology Unit, Fondazione IRCCS Policlinico San Matteo , Pavia	Laboratory of Virology, INMI Lazzaro Spallanzani IRCCS	Fausto Baldanti, Martina Rueca, Antonio Piralla, Antonino Di Caro, Maria R. Capobianchi, Cesare E.M. Gruber, Barbara Bartolini
EPI_ISL_460082	Molecular Virology Unit, Fondazione IRCCS Policlinico San Matteo , Pavia	Laboratory of Virology, INMI Lazzaro Spallanzani IRCCS	Martina Rueca, Cesare E.M. Gruber, Antonio Piralla, Antonino Di Caro, Barbara Bartolini, Maria R. Capobianchi, Fausto Baldanti
EPI_ISL_460083	Molecular Virology Unit, Fondazione IRCCS Policlinico San Matteo , Pavia	Laboratory of Virology, INMI Lazzaro Spallanzani IRCCS	Martina Rueca, Antonino Di Caro, Cesare E.M. Gruber, Barbara Bartolini, Fausto Baldanti, Antonio Piralla, Maria R. Capobianchi
EPI_ISL_460084	Molecular Virology Unit, Fondazione IRCCS Policlinico San Matteo , Pavia	Laboratory of Virology, INMI Lazzaro Spallanzani IRCCS	Fausto Baldanti, Antonio Piralla, Martina Rueca, Barbara Bartolini, Maria R. Capobianchi, Cesare E.M. Gruber, Antonino Di Caro
EPI_ISL_460085	Molecular Virology Unit, Fondazione IRCCS Policlinico San Matteo , Pavia	Laboratory of Virology, INMI Lazzaro Spallanzani IRCCS	Cesare E.M. Gruber, Maria R. Capobianchi, Barbara Bartolini, Fausto Baldanti, Martina Rueca, Antonio Piralla, Antonino Di Caro
EPI_ISL_460086	Molecular Virology Unit, Fondazione IRCCS Policlinico San Matteo , Pavia	Laboratory of Virology, INMI Lazzaro Spallanzani IRCCS	Maria R. Capobianchi, Fausto Baldanti, Antonio Piralla, Antonino Di Caro, Barbara Bartolini, Cesare E.M. Gruber, Martina Rueca
EPI_ISL_460087	Molecular Virology Unit, Fondazione IRCCS Policlinico San Matteo , Pavia	Laboratory of Virology, INMI Lazzaro Spallanzani IRCCS	Cesare E.M. Gruber, Maria R. Capobianchi, Martina Rueca, Barbara Bartolini, Antonino Di Caro, Antonio Piralla, Fausto Baldanti
EPI_ISL_460088	Molecular Virology Unit, Fondazione IRCCS Policlinico San Matteo , Pavia	Laboratory of Virology, INMI Lazzaro Spallanzani IRCCS	Martina Rueca, Barbara Bartolini, Fausto Baldanti, Maria R. Capobianchi, Cesare E.M. Gruber, Antonino Di Caro, Antonio Piralla
EPI_ISL_460089	Molecular Virology Unit, Fondazione IRCCS Policlinico San Matteo , Pavia	Laboratory of Virology, INMI Lazzaro Spallanzani IRCCS	Antonino Di Caro, Barbara Bartolini, Martina Rueca, Cesare E.M. Gruber, Antonio Piralla, Fausto Baldanti, Maria R. Capobianchi
EPI_ISL_460091	Molecular Virology Unit, Fondazione IRCCS Policlinico San Matteo , Pavia	Laboratory of Virology, INMI Lazzaro Spallanzani IRCCS	Antonino Di Caro, Antonio Piralla, Martina Rueca, Fausto Baldanti, Barbara Bartolini, Maria R. Capobianchi, Cesare E.M. Gruber
EPI_ISL_460092	Molecular Virology Unit, Fondazione IRCCS Policlinico San Matteo , Pavia	Laboratory of Virology, INMI Lazzaro Spallanzani IRCCS	Cesare E.M. Gruber, Martina Rueca, Maria R. Capobianchi, Antonino Di Caro, Antonio Piralla, Barbara Bartolini, Fausto Baldanti
EPI_ISL_460093	Molecular Virology Unit, Fondazione IRCCS Policlinico San Matteo , Pavia	Laboratory of Virology, INMI Lazzaro Spallanzani IRCCS	Maria R. Capobianchi, Antonio Piralla, Antonino Di Caro, Fausto Baldanti, Martina Rueca, Cesare E.M. Gruber, Barbara Bartolini
EPI_ISL_460094	Molecular Virology Unit, Fondazione IRCCS Policlinico San Matteo , Pavia	Laboratory of Virology, INMI Lazzaro Spallanzani IRCCS	Barbara Bartolini, Maria R. Capobianchi, Antonino Di Caro, Antonio Piralla, Cesare E.M. Gruber, Martina Rueca, Fausto Baldanti
EPI_ISL_461478	Government Medical College, Vadodara	Gujarat Biotechnology Research Centre	Fenil Patel, Nidhi Patel, Nitin Savaliya, Raghawendra Kumar, Dinesh Kumar, Zuber Saiyed, Komal Patel, Labdhi Pandya, Snehal Bagatharia, Tanuja Javadekar , R N Daveshwar, Tejas Shah, Ankit Hinsu, Pritesh Sabara, Apurvasinh Puvar, Janvi Raval, Zarna Patel, Monika Gandhi, Pinal Trivedi, Maharshi Pandya, R D Dixit, A M Kadri, Harsh Bakshi, Chaitanya Joshi, Madhvi Joshi,
EPI_ISL_461479	Government Medical College, Vadodara	Gujarat Biotechnology Research Centre	Neelam Nathani, Nitin Savaliya, Raghawendra Kumar, Dinesh Kumar, Zuber Saiyed, Komal Patel, Labdhi Pandya, Snehal Bagatharia, Tanuja Javadekar , R N Daveshwar, Tejas Shah, Ankit Hinsu, Pritesh Sabara, Apurvasinh Puvar, Janvi Raval, Zarna Patel, Monika Gandhi, Pinal Trivedi, Maharshi Pandya, Nidhi Patel, R D Dixit, A M Kadri, Harsh Bakshi, Chaitanya Joshi, Madhvi Joshi,
EPI_ISL_461480	Government Medical College, Vadodara	Gujarat Biotechnology Research Centre	Armi Chaudhari, Raghawendra Kumar, Dinesh Kumar, Zuber Saiyed, Komal Patel, Labdhi Pandya, Snehal Bagatharia, Tanuja Javadekar , R N Daveshwar, Tejas Shah, Ankit Hinsu, Pritesh Sabara, Apurvasinh Puvar, Janvi Raval, Zarna Patel, Monika Gandhi, Pinal Trivedi, Maharshi Pandya, Nidhi Patel, Nitin Savaliya, R D Dixit, A M Kadri, Harsh Bakshi, Chaitanya Joshi, Madhvi Joshi,
EPI_ISL_461481	Pandit Deendayal Upadhyay Government Medical College, Rajkot	Gujarat Biotechnology Research Centre	Bhavya Jindal, Dinesh Kumar, Zuber Saiyed, Komal Patel, Labdhi Pandya, Snehal Bagatharia, Prakash Modi, Sejul Antala, Manish Pattani, Tejas Shah, Ankit Hinsu, Pritesh Sabara, Apurvasinh Puvar, Janvi Raval, Zarna Patel, Monika Gandhi, Pinal Trivedi, Maharshi Pandya, Nidhi Patel, Nitin Savaliya, Raghawendra Kumar, R D Dixit, A M Kadri, Harsh Bakshi, Chaitanya Joshi, Madhvi Joshi
EPI_ISL_461482	Pandit Deendayal Upadhyay Government Medical College, Rajkot	Gujarat Biotechnology Research Centre	Anjali Rajwar, Zuber Saiyed, Komal Patel, Labdhi Pandya, Snehal Bagatharia, Prakash Modi, Sejul Antala, Manish Pattani, Tejas Shah, Ankit Hinsu, Pritesh Sabara, Apurvasinh Puvar, Janvi Raval, Zarna Patel, Monika Gandhi, Pinal Trivedi, Maharshi Pandya, Nidhi Patel, Nitin Savaliya, Raghawendra Kumar, Dinesh Kumar, R D Dixit, A M Kadri, Harsh Bakshi, Chaitanya Joshi, Madhvi Joshi
EPI_ISL_462447, EPI_ISL_462448	Fundació Lluita contra la SIDA (FLSida)/Hospital Universitari Germans Trias i Pujol	IrsiCaixa AIDS Research Lab	Marc Noguera-Julian, Mariona Parera, Maria Pilar Armengol, Marc Corbacho, Maria Ubals, Oriol Mitjà, Lidia Ruiz, Nuria Izquierdo, Jorge Carrillo, Roger Paredes, Julia Blanco, Joaquim Segalés, Bonaventura Clotet
EPI_ISL_462449	Fundació Lluita contra la SIDA (FLSida)/Hospital Universitari Germans Trias i Pujol	IrsiCaixa AIDS Research Lab	Marc Noguera-Julian, Mariona Parera, Maria Pilar Armengol, Marc Corbacho, Maria Ubals, Oriol Mitjà, Lidia Ruiz, Nuria Izquierdo, Jorge Carrillo, Roger Paredes, Julia Blanco, Bonaventura Clotet

EPI_ISL_462478	Fundación Jiménez Díaz	Instituto de Salud Carlos III	Iglesias-Caballero, M. Molinero Calamita, M. González-Esguevillas, M. Camarero, S. Pozo, F. Casas, I. Jiménez, P. Jiménez, M. Zaballos, A. Monzón, S. Varona, S. Juliá, M. Cuesta, I, R. Fernández
EPI_ISL_462479	Hospital Clinic	Instituto de Salud Carlos III	Iglesias-Caballero, M. Molinero Calamita, M. González-Esguevillas, M. Camarero, S. Pozo, F. Casas, I. Jiménez, P. Jiménez, M. Zaballos, A. Monzón, S. Varona, S. Juliá, M. Cuesta, I, M.A Marcos
EPI_ISL_463007	Department of Laboratory Medicine, National Taiwan University Hospital	Microbial Genomics Core Lab, National Taiwan University Centers of Genomic and Precision Medicine	Shiou-Hwei Yeh, You-Yu Lin, Ya-Yun Lai, Chiao-Ling Li, Shan-Chwen Chang, Pei-Jer Chen, Sui-Yuan Chang
EPI_ISL_463008	Institute of Molecular Virology, University Münster	Institute of Molecular Virology, University Münster	Angeles Mecate Zambrano, Linda Brunotte, Stephan Ludwig, Joachim Kühn, Alexander Mellmann
EPI_ISL_463277, EPI_ISL_463278, EPI_ISL_463279, EPI_ISL_463280, EPI_ISL_463281, EPI_ISL_463282, EPI_ISL_463283, EPI_ISL_463284, EPI_ISL_463285, EPI_ISL_463286, EPI_ISL_463287, EPI_ISL_463288, EPI_ISL_463289, EPI_ISL_463290, EPI_ISL_463291, EPI_ISL_463292, EPI_ISL_463293, EPI_ISL_463294, EPI_ISL_463295, EPI_ISL_463296, EPI_ISL_463297, EPI_ISL_463298, EPI_ISL_463299, EPI_ISL_463300			
see above	Ochsner Health	Bioinfoexperts, LLC	Susanna L. Lamers, David J. Nolan, Rebecca Rose, Sissy Cross, David Moraga Amador, Tong Yang, Luke Caruso, Wayra Navia, Lydia Von Borstel, Xiao Hui Zhou, Amy Feehan, Julia-Garcia-Diaz
EPI_ISL_463301	Tuen Mun Hospital	Hong Kong Department of Health	Mak Gannon C.K., Cheng Peter K.C., Lam Edman T.K., Chan Rickjason C.W., Tsang Dominic N.C.
EPI_ISL_463302, EPI_ISL_463303	United Christian Hospital	Hong Kong Department of Health	Mak Gannon C.K., Cheng Peter K.C., Lam Edman T.K., Chan Rickjason C.W., Tsang Dominic N.C.
EPI_ISL_463304	Mrs Wu York Yu GOPC	Hong Kong Department of Health	Mak Gannon C.K., Cheng Peter K.C., Lam Edman T.K., Chan Rickjason C.W., Tsang Dominic N.C.
EPI_ISL_463305	Queen Elizabeth Hospital	Hong Kong Department of Health	Mak Gannon C.K., Cheng Peter K.C., Lam Edman T.K., Chan Rickjason C.W., Tsang Dominic N.C.
EPI_ISL_463306	Prince of Wales Hospital	Hong Kong Department of Health	Mak Gannon C.K., Cheng Peter K.C., Lam Edman T.K., Chan Rickjason C.W., Tsang Dominic N.C.
EPI_ISL_463307	HK Molecular Pathology Disgnostic Centre	Hong Kong Department of Health	Mak Gannon C.K., Cheng Peter K.C., Lam Edman T.K., Chan Rickjason C.W., Tsang Dominic N.C.
EPI_ISL_463309	Prince of Wales Hospital	Hong Kong Department of Health	Mak Gannon C.K., Cheng Peter K.C., Lam Edman T.K., Chan Rickjason C.W., Tsang Dominic N.C.
EPI_ISL_463310	Tseung Kwan O Hospital	Hong Kong Department of Health	Mak Gannon C.K., Cheng Peter K.C., Lam Edman T.K., Chan Rickjason C.W., Tsang Dominic N.C.
EPI_ISL_463311, EPI_ISL_463312	Queen Elizabeth Hospital	Hong Kong Department of Health	Mak Gannon C.K., Cheng Peter K.C., Lam Edman T.K., Chan Rickjason C.W., Tsang Dominic N.C.
EPI_ISL_463313	Central Health Medical Practice	Hong Kong Department of Health	Mak Gannon C.K., Cheng Peter K.C., Lam Edman T.K., Chan Rickjason C.W., Tsang Dominic N.C.
EPI_ISL_463314	Queen Elizabeth Hospital	Hong Kong Department of Health	Mak Gannon C.K., Cheng Peter K.C., Lam Edman T.K., Chan Rickjason C.W., Tsang Dominic N.C.
EPI_ISL_463315	Princess Margaret Hospital	Hong Kong Department of Health	Mak Gannon C.K., Cheng Peter K.C., Lam Edman T.K., Chan Rickjason C.W., Tsang Dominic N.C.
EPI_ISL_463316, EPI_ISL_463317	Yan Chai Hospital	Hong Kong Department of Health	Mak Gannon C.K., Cheng Peter K.C., Lam Edman T.K., Chan Rickjason C.W., Tsang Dominic N.C.
EPI_ISL_463318	Pamela Youde Nethersole Eastern Hospital	Hong Kong Department of Health	Mak Gannon C.K., Cheng Peter K.C., Lam Edman T.K., Chan Rickjason C.W., Tsang Dominic N.C.
EPI_ISL_463319	Prince of Wales Hospital	Hong Kong Department of Health	Mak Gannon C.K., Cheng Peter K.C., Lam Edman T.K., Chan Rickjason C.W., Tsang Dominic N.C.
EPI_ISL_463320	Tseung Kwan O Hospital	Hong Kong Department of Health	Mak Gannon C.K., Cheng Peter K.C., Lam Edman T.K., Chan Rickjason C.W., Tsang Dominic N.C.
EPI_ISL_463321	Queen Mary Hospital	Hong Kong Department of Health	Mak Gannon C.K., Cheng Peter K.C., Lam Edman T.K., Chan Rickjason C.W., Tsang Dominic N.C.
EPI_ISL_463322	North Lantau Hospital	Hong Kong Department of Health	Mak Gannon C.K., Cheng Peter K.C., Lam Edman T.K., Chan Rickjason C.W., Tsang Dominic N.C.
EPI_ISL_463323	Yuen Long JC GOPC	Hong Kong Department of Health	Mak Gannon C.K., Cheng Peter K.C., Lam Edman T.K., Chan Rickjason C.W., Tsang Dominic N.C.
EPI_ISL_463741, EPI_ISL_463742, EPI_ISL_463743, EPI_ISL_463744, EPI_ISL_463745, EPI_ISL_463746, EPI_ISL_463747, EPI_ISL_463748	Department of Molecular Virology, Cyprus Institute of Neurology and Genetics	Department of Molecular Virology, Cyprus Institute of Neurology and Genetics	Jan Richter, George Krashias, Christina Tryfonos, Stavros Bashiardes, Dana Koptides, Christina Christodoulou
EPI_ISL_467300	General Hospital "Abdulah Nakas"	Alea Genetic Center	Rijad Konjhodzic; Lana Salihefendic; Teufik Goletic; Sead Jazic; Dino Pecar; Nihad Fejzic; Damir Marjanovic; Enis Kandic
EPI_ISL_467352, EPI_ISL_467353, EPI_ISL_467367, EPI_ISL_467369	Laboratory of Respiratory Viruses and Measles, Oswaldo Cruz Institute, FIOCRUZ	Laboratory of Respiratory Viruses and Measles, Oswaldo Cruz Institute, FIOCRUZ	Paola Resende, Luciana Appolinario, Fernando Motta, Anna Carolina Paixão, Ana Carolina Mendonça, Aline Mattos, Milene Miranda, Cristiana Garcia, Braulia Caetano, Maria Ogrzewalska, Jonathan Lopes, Marilda Siqueira on behalf of the Fiocruz COVID-19 Genomic Surveillance Network
EPI_ISL_467374	Dinkes Samarinda	Eijkman Institute for Molecular Biology, Ministry of Research and Technology/National Agency for Research and Innovation	Edison Johar, Frilasita A Yudhaputri, Hidayat Trimarsanto, David H Muljono, Safarina G Malik, Khin Saw Myint, Amin Soebandrio
EPI_ISL_467375	RSUP Prof. Dr. R. Kandou Manado	Eijkman Institute for Molecular Biology, Ministry of Research and Technology/National Agency for Research and Innovation	Edison Johar, Frilasita A Yudhaputri, Hidayat Trimarsanto, David H Muljono, Safarina G Malik, Khin Saw Myint, Amin Soebandrio
EPI_ISL_467376	RSUP Fatmawati	Eijkman Institute for Molecular Biology, Ministry of Research and Technology/National Agency for Research and Innovation	Edison Johar, Frilasita A Yudhaputri, Hidayat Trimarsanto, David H Muljono, Safarina G Malik, Khin Saw Myint, Amin Soebandrio
EPI_ISL_467774, EPI_ISL_467775	Molecular diagnostic laboratory of Federal Budget Institution of Science "Central Research Institute of Epidemiology" of The Federal Service on Customers' Rights Protection and Human Well-being Surveillance	Group of Genomics and Postgenomic Technologies of Central Research Institute of Epidemiology	Speranskaya AS, Kaptelova VV, Samoilov AE, Korneenko EV, Sizova TV, Tivanova EV, Shipulina OY, Akimkin VG
EPI_ISL_467809	Cedars-Sinai Medical Center, Department of Pathology & Laboratory Medicine, Molecular Pathology Laboratory	Cedars-Sinai Medical Center, Molecular Pathology Laboratory of Department of Pathology & Laboratory Medicine and Genomic Core	Wenjuan Zhang, John Paul Govindavari, Brian Davis, Stephanie Chen, Jong Taek Kim, Jianbo Song, Jean Lopategui, Jasmine T Plummer, Eric Vail
EPI_ISL_468134, EPI_ISL_468135, EPI_ISL_468136, EPI_ISL_468137, EPI_ISL_468138, EPI_ISL_468139, EPI_ISL_468140, EPI_ISL_468141, EPI_ISL_468142, EPI_ISL_468143, EPI_ISL_468144, EPI_ISL_468145, EPI_ISL_468146, EPI_ISL_468147, EPI_ISL_468148, EPI_ISL_468149, EPI_ISL_468150, EPI_ISL_468151, EPI_ISL_468153, EPI_ISL_468154, EPI_ISL_468155, EPI_ISL_468156			
see above	[Romania, Bucharest] National Institute for Infectious Diseases "Prof. Dr. Matei Bal"	[Romania, Bucharest] National Institute for Infectious Diseases "Prof. Dr. Matei Bal"	Leontina Banica, Marius Cotic, Corina Casangiu, Marius Surleac, Simona Paraschiv
EPI_ISL_468305, EPI_ISL_468307	Centro de Vigilancia a Saude de Diadema	Instituto Adolfo Lutz, Interdiciplinary Procedures Center, Strategic Laboratory	Claudio Tavares Sacchi, Claudia Regina Gonçalves, Erica Valessa Ramos Gomes
EPI_ISL_468308	Hospital Municipal do Tatuape Carmino Caricchio	Instituto Adolfo Lutz, Interdiciplinary Procedures Center, Strategic Laboratory	Claudio Tavares Sacchi, Claudia Regina Gonçalves, Erica Valessa Ramos Gomes
EPI_ISL_468310	Hospital Sao Paulo de Ensino da UNIFESP	Instituto Adolfo Lutz, Interdiciplinary Procedures Center, Strategic Laboratory	Claudio Tavares Sacchi, Claudia Regina Gonçalves, Erica Valessa Ramos Gomes
EPI_ISL_468311, EPI_ISL_468312	Hospital Municipal Dr Ignacio Proenca de Gouvea	Instituto Adolfo Lutz, Interdiciplinary Procedures Center, Strategic Laboratory	Claudio Tavares Sacchi, Claudia Regina Gonçalves, Erica Valessa Ramos Gomes
EPI_ISL_468313	Vigilancia Epidemiologica de São Bernardo do Campo	Instituto Adolfo Lutz, Interdiciplinary Procedures Center, Strategic Laboratory	Claudio Tavares Sacchi, Claudia Regina Gonçalves, Erica Valessa Ramos Gomes
EPI_ISL_468314	CTA Centro de Testagem e Aconselhamento	Instituto Adolfo Lutz, Interdiciplinary Procedures Center,	Claudio Tavares Sacchi, Claudia Regina Gonçalves, Erica Valessa Ramos Gomes

		Strategic Laboratory	
EPI_ISL_468315	Hospital Municipal do Tatuape Carmino Caricchio	Instituto Adolfo Lutz, Interdiciplinary Procedures Center, Strategic Laboratory	Claudio Tavares Sacchi, Claudia Regina Gonçalves, Erica Valessa Ramos Gomes
EPI_ISL_468316	UPA Vila Assis	Instituto Adolfo Lutz, Interdiciplinary Procedures Center, Strategic Laboratory	Claudio Tavares Sacchi, Claudia Regina Gonçalves, Erica Valessa Ramos Gomes
EPI_ISL_468318	Hospital Universitario da USP	Instituto Adolfo Lutz, Interdiciplinary Procedures Center, Strategic Laboratory	Claudio Tavares Sacchi, Claudia Regina Gonçalves, Erica Valessa Ramos Gomes
EPI_ISL_468319	Vigilancia Epidemiologica de São Bernardo do Campo	Instituto Adolfo Lutz, Interdiciplinary Procedures Center, Strategic Laboratory	Claudio Tavares Sacchi, Claudia Regina Gonçalves, Erica Valessa Ramos Gomes
EPI_ISL_468320	Secretaria Municipal de Saude de Hortolandia	Instituto Adolfo Lutz, Interdiciplinary Procedures Center, Strategic Laboratory	Claudio Tavares Sacchi, Claudia Regina Gonçalves, Erica Valessa Ramos Gomes
EPI_ISL_468321	Hospital Universitario da USP	Instituto Adolfo Lutz, Interdiciplinary Procedures Center, Strategic Laboratory	Claudio Tavares Sacchi, Claudia Regina Gonçalves, Erica Valessa Ramos Gomes
EPI_ISL_468591, EPI_ISL_468656	Institute for Public Health	Laboratory for advanced genomics	Filip Roki, Lovro Trgovec-Greif, Neven Sui, Tomislav Rukavina, Igor Jurak, Oliver Vugrek
EPI_ISL_468765, EPI_ISL_468766, EPI_ISL_468768, EPI_ISL_468771, EPI_ISL_468772, EPI_ISL_468774, EPI_ISL_468775, EPI_ISL_468776, EPI_ISL_468780, EPI_ISL_468782, EPI_ISL_468788, EPI_ISL_468791, EPI_ISL_468793, EPI_ISL_468795, EPI_ISL_468798, EPI_ISL_468800, EPI_ISL_468801, EPI_ISL_468804, EPI_ISL_468805, EPI_ISL_468808, EPI_ISL_468809, EPI_ISL_468810, EPI_ISL_468812, EPI_ISL_468814, EPI_ISL_468815, EPI_ISL_468817, EPI_ISL_468820, EPI_ISL_468829, EPI_ISL_468830, EPI_ISL_468832, EPI_ISL_468834, EPI_ISL_468835, EPI_ISL_468841, EPI_ISL_468849, EPI_ISL_468851, EPI_ISL_468855, EPI_ISL_468858, EPI_ISL_468859			
see above	Servicio de Microbiología, Hospital Miguel Servet, Zaragoza	SeqCOVID-SPAIN consortium/IBV(CSIC)	Antonio Rezusta López, Alexander Tristancho Baró, Ana Milagro, Yolanda Gracia Grataloup, Nieves Martínez Cameo and SeqCOVID-SPAIN consortium
EPI_ISL_468914, EPI_ISL_469016, EPI_ISL_469018, EPI_ISL_469019, EPI_ISL_469020, EPI_ISL_469021, EPI_ISL_469022	Istituto Zooprofilattico Sperimentale Puglia e Basilicata; Dipartimento di Bioscienze, Biotecnologie e Biofarmaceutica dell'Università degli Studi di Bari "A.Moro"; Istituto di Biomembrane. Bioenergetica e Biotecnologie Molecolari del Consiglio Nazionale delle Ricerche di Bari	Beaconlab (Bioinformatics, Evolution and Comparative Genomics lab), Dept of Biosciences, University on Milan	Parisi A.,Pesole G., Manzari C., Chiara M.
EPI_ISL_469023	Istituto Zooprofilattico Sperimentale Puglia e Basilicata; Dipartimento di Bioscienze, Biotecnologie e Biofarmaceutica dell'Università degli Studi di Bari "A.Moro"; Istituto di Biomembrane. Bioenergetica e Biotecnologie Molecolari del Consiglio Nazionale delle Ricerche di Bari	Beaconlab (Bioinformatics, Evolution and Comparative Genomics lab), Dept of Biosciences, University on Milan	Parisi A.,Pesole G., Manzari C., Chiara M
EPI_ISL_469275	Egyptian National Cancer Institute (ENCI)	Human Genome Center	Zekri, Abdel Rahman N, Amer,K.E., Ahmed,O.S., Soliman,H.K., Hafez,M.M., Bahnassy,A.A., Abdelhamid,W., Gad,A., Ali,M., Hassan,W., Samir,M., Raouf,A., Hamdy,M.S., Soliman,M.S., Elsissy,M.H., Elkhateeb,S.M., Ezzelarab,M.H., Abouelhoda, Mohamed
EPI_ISL_469282	Service de Virologie Hôpital Saint-Louis	Laboratory of Cell Biology of viral infection, Unit INSERM-U944	Laurent Meertens, Lucie Bonnet-Madin, Constance Delaugerre, Ali Amara
EPI_ISL_469283, EPI_ISL_469284	Service de Virologie Hôpital Saint-Louis	Laboratory Cell Biology of Viral Infection-INSERM unit 944	Laurent Meertens, Lucie Bonnet-Madin, Séverine Mercier-Delarue, Maud SALMONA, Constance Delaugerre, Ali Amara
EPI_ISL_470896	Russian State Collection of Viruses	Pathogenic Microorganisms Variability Laboratory	Alexey Shchetinin, Maria Nikiforova, Elena Shidlovskaya, Nadezhda Kuznetsova, Inna Dolzhikova, Daria Grousova, Andrey Botikov, Denis Logunov, Alexander Gintsburg, Vladimir Gushchin
EPI_ISL_470897, EPI_ISL_470898, EPI_ISL_470899	Pathogenic Microorganisms Variability Laboratory	Pathogenic Microorganisms Variability Laboratory	Alexey Shchetinin, Maria Nikiforova, Elena Shidlovskaya, Nadezhda Kuznetsova, Andrey Botikov, Alexander Gintsburg, Vladimir Gushchin
EPI_ISL_470900, EPI_ISL_470901, EPI_ISL_470902	Influenza etiology and epidemiology laboratory	Pathogenic Microorganisms Variability Laboratory	Alexey Shchetinin, Maria Nikiforova, Elena Shidlovskaya, Nadezhda Kuznetsova, Vladimir Gushchin, Inna Dolzhikova, Daria Grousova, Andrey Botikov, Denis Logunov, Kirill Krasnoslobotsev, Svetlana Trushakova, Elena Burtseva, Ludmila Kolobukhina, Svetlana Smetanina, Alexander Gintsburg
EPI_ISL_470903, EPI_ISL_470904	Influenza etiology and epidemiology laboratory	Pathogenic Microorganisms Variability Laboratory	Alexey Shchetinin, Maria Nikiforova, Elena Shidlovskaya, Nadezhda Kuznetsova, Vladimir Gushchin, Inna Dolzhikova, Daria Grousova, Andrey Botikov, Denis Logunov, Anna Ignatjeva, Evgeniya Mukasheva, Elena Burtseva, Ludmila Kolobukhina, Svetlana Smetanina, Alexander Gintsburg
EPI_ISL_471158, EPI_ISL_471163, EPI_ISL_471164, EPI_ISL_471167, EPI_ISL_471171	MRCG at LSHTM Genomics lab	MRCG at LSHTM Genomics lab	Sesay et al
EPI_ISL_471267	Hospital IESS Babahoyo	Institute of Microbiology, Universidad San Francisco de Quito	Sully Márquez, Belén Prado-Vivar, Juan José Guadalupe, Bernardo Gutiérrez, Francisco Cordova, Ninfa Henriquez, Killen Briones-Zamora, Killen Briones-Claudette, Verónica Barragán, Patricio Rojas-Silva, Gabriel Trueba, Michelle Grunauer, Paúl Cárdenas
EPI_ISL_471456, EPI_ISL_471457, EPI_ISL_471458, EPI_ISL_471459, EPI_ISL_471460	Centre de Virologie des Maladies Tropicales	Functional Genomic Platform/Service Analyses Biologique/UATRS/ Centre National Pour la Recherche Scientifique Et Technique (CNRST)	Hicham EL ANNAZ, Elmostafa EL FAHIME, Marouane MELLOUL, Youssef AKHOUAD, Mly Abdelaziz ELALAOUI, Ahmed REGGAD, Sanaa ALAOUI-Amine, Rachid ABI, Rida TAGAJDID, Zohour KASMY, Safae ELKOCHRI, Nadia TOUIL, Farida HILALI, Abdelkader LAATIRIS, Abdelillah LARAQUI, Tahar BAJJOU , Yassine SEKHSOKH , Idriss-Amine LAHLOU, Mostafa ELOUENNASS, Khalid ENNIBI
EPI_ISL_471472	Hospital Universitari Germans Trias i Pujol(HUGTiP)/Fundació Lluita contra la SIDA (FLSida)/IRTA-CReSA	IrsiCaixa AIDS Research Lab	Marc Noguera-Julian, Pilar Armengol, Jordi Rodón, Julia Vergara, Lidia Ruiz, Nuria Izquierdo, Jorge Carrillo, Roger Paredes, Albert Bensaid, Julia Blanco, Joaquim Segalés, Bonaventura Clotet
EPI_ISL_471528, EPI_ISL_471530	The National Institute of Public Health	State Veterinary Institute Prague and The National Institute of Public Health	Nagy,A;Jirincova,H;Novakova,L;Trnka,D;Vecerova,J
EPI_ISL_471539	Hospital Universitario da USP Sao Paulo	Instituto Adolfo Lutz, Interdiciplinary Procedures Center, Strategic Laboratory	Claudio Tavares Sacchi, Claudia Regina Gonçalves, Erica Valessa Ramos Gomes
EPI_ISL_471540	The National Institute of Public Health	State Veterinary Institute Prague and The National Institute of Public Health	Nagy,A;Jirincova,H;Novakova,L;Trnka,D;Vecerova,J
EPI_ISL_471541	Hospital Geral Santa Marcelina	Instituto Adolfo Lutz, Interdiciplinary Procedures Center, Strategic Laboratory	Claudio Tavares Sacchi, Claudia Regina Gonçalves, Erica Valessa Ramos Gomes
EPI_ISL_471542	Secretaria de Saude de Mogi das Cruzes	Instituto Adolfo Lutz, Interdiciplinary Procedures Center, Strategic Laboratory	Claudio Tavares Sacchi, Claudia Regina Gonçalves, Erica Valessa Ramos Gomes
EPI_ISL_471543	Centro de Saude I Tacito Leite de Carvalho e Silva	Instituto Adolfo Lutz, Interdiciplinary Procedures Center, Strategic Laboratory	Claudio Tavares Sacchi, Claudia Regina Gonçalves, Erica Valessa Ramos Gomes
EPI_ISL_471544	The National Institute of Public Health	State Veterinary Institute Prague and The National Institute of Public Health	Nagy,A;Jirincova,H;Novakova,L;Trnka,D;Vecerova,J
EPI_ISL_471545	Hospital Sao Paulo de Ensino da Unifesp	Instituto Adolfo Lutz, Interdiciplinary Procedures Center, Strategic Laboratory	Claudio Tavares Sacchi, Claudia Regina Gonçalves, Erica Valessa Ramos Gomes
EPI_ISL_471546	AMA DR Jose Soares Hungria	Instituto Adolfo Lutz, Interdiciplinary Procedures Center, Strategic Laboratory	Claudio Tavares Sacchi, Claudia Regina Gonçalves, Erica Valessa Ramos Gomes
EPI_ISL_471547	The National Institute of Public Health	State Veterinary Institute Prague and The National Institute of Public Health	Nagy,A;Jirincova,H;Novakova,L;Trnka,D;Vecerova,J

EPI_ISL_471548	Hospital do Servidor Público Estadual Francisco Morato de Oliveira	Instituto Adolfo Lutz, Interdiciplinary Procedures Center, Strategic Laboratory	Claudio Tavares Sacchi, Claudia Regina Gonçalves, Erica Valessa Ramos Gomes
EPI_ISL_471549	Hospital Municipal Carmen Prudente	Instituto Adolfo Lutz, Interdiciplinary Procedures Center, Strategic Laboratory	Claudio Tavares Sacchi, Claudia Regina Gonçalves, Erica Valessa Ramos Gomes
EPI_ISL_471550	The National Institute of Public Health	State Veterinary Institute Prague and The National Institute of Public Health	Nagy,A;Jirincova,H;Novakova,L;Trnka,D;Vecerova,J
EPI_ISL_471551	Hospital Sao Paulo de Ensino da Unifesp	Instituto Adolfo Lutz, Interdiciplinary Procedures Center, Strategic Laboratory	Claudio Tavares Sacchi, Claudia Regina Gonçalves, Erica Valessa Ramos Gomes
EPI_ISL_471552	Hospital Sancta Maggiore	Instituto Adolfo Lutz, Interdiciplinary Procedures Center, Strategic Laboratory	Claudio Tavares Sacchi, Claudia Regina Gonçalves, Erica Valessa Ramos Gomes
EPI_ISL_471553	The National Institute of Public Health	State Veterinary Institute Prague and The National Institute of Public Health	Nagy,A;Jirincova,H;Novakova,L;Trnka,D;Vecerova,J
EPI_ISL_471554	Hospital Bosque da Saúde	Instituto Adolfo Lutz, Interdiciplinary Procedures Center, Strategic Laboratory	Claudio Tavares Sacchi, Claudia Regina Gonçalves, Erica Valessa Ramos Gomes
EPI_ISL_471555	The National Institute of Public Health	State Veterinary Institute Prague and The National Institute of Public Health	Nagy,A;Jirincova,H;Novakova,L;Trnka,D;Vecerova,J
EPI_ISL_471556	Pronto Socorro Jose Ibrahin	Instituto Adolfo Lutz, Interdiciplinary Procedures Center, Strategic Laboratory	Claudio Tavares Sacchi, Claudia Regina Gonçalves, Erica Valessa Ramos Gomes
EPI_ISL_471562, EPI_ISL_471581, EPI_ISL_471582	Hosp. Municipal Prof. Dr. Alípio Corrêa Netto	Instituto Adolfo Lutz, Interdiciplinary Procedures Center, Strategic Laboratory	Claudio Tavares Sacchi, Claudia Regina Gonçalves, Erica Valessa Ramos Gomes
EPI_ISL_471647	Hospital Municipal de Barueri Dr. Francisco Moran	Instituto Adolfo Lutz, Interdiciplinary Procedures Center, Strategic Laboratory	Claudio Tavares Sacchi, Claudia Regina Gonçalves, Erica Valessa Ramos Gomes
EPI_ISL_471648	UBS e Pronto Socorro Jd. Jacira	Instituto Adolfo Lutz, Interdiciplinary Procedures Center, Strategic Laboratory	Claudio Tavares Sacchi, Claudia Regina Gonçalves, Erica Valessa Ramos Gomes
EPI_ISL_475574, EPI_ISL_475576, EPI_ISL_475577, EPI_ISL_475578, EPI_ISL_475579, EPI_ISL_475580, EPI_ISL_475582, EPI_ISL_475583, EPI_ISL_475585, EPI_ISL_475588, EPI_ISL_475590, EPI_ISL_475593, EPI_ISL_475594, EPI_ISL_475595, EPI_ISL_475596, EPI_ISL_475598, EPI_ISL_475599, EPI_ISL_475600, EPI_ISL_475601, EPI_ISL_475602, EPI_ISL_475603, EPI_ISL_475604, EPI_ISL_475605, EPI_ISL_475606, EPI_ISL_475607, EPI_ISL_475608, EPI_ISL_475609, EPI_ISL_475610, EPI_ISL_475611, EPI_ISL_475612, EPI_ISL_475613, EPI_ISL_475614, EPI_ISL_475615, EPI_ISL_475616, EPI_ISL_475617, EPI_ISL_475618, EPI_ISL_475619, EPI_ISL_475621, EPI_ISL_475622, EPI_ISL_475625, EPI_ISL_475627, EPI_ISL_475628, EPI_ISL_475629, EPI_ISL_475630, EPI_ISL_475631, EPI_ISL_475632, EPI_ISL_475633, EPI_ISL_475634, EPI_ISL_475635, EPI_ISL_475636, EPI_ISL_475637, EPI_ISL_475638, EPI_ISL_475639, EPI_ISL_475640, EPI_ISL_475641, EPI_ISL_475643, EPI_ISL_475644, EPI_ISL_475645, EPI_ISL_475646, EPI_ISL_475647, EPI_ISL_475648, EPI_ISL_475650, EPI_ISL_475651, EPI_ISL_475652, EPI_ISL_475653, EPI_ISL_475654, EPI_ISL_475655, EPI_ISL_475656, EPI_ISL_475657, EPI_ISL_475658, EPI_ISL_475659, EPI_ISL_475660, EPI_ISL_475661, EPI_ISL_475662, EPI_ISL_475663, EPI_ISL_475664, EPI_ISL_475666, EPI_ISL_475667, EPI_ISL_475668, EPI_ISL_475669, EPI_ISL_475670, EPI_ISL_475671, EPI_ISL_475673, EPI_ISL_475674, EPI_ISL_475675, EPI_ISL_475677, EPI_ISL_475678, EPI_ISL_475679, EPI_ISL_475680, EPI_ISL_475681, EPI_ISL_475682, EPI_ISL_475683, EPI_ISL_475684, EPI_ISL_475685, EPI_ISL_475686, EPI_ISL_475687, EPI_ISL_475688, EPI_ISL_475689, EPI_ISL_475690, EPI_ISL_475691, EPI_ISL_475692, EPI_ISL_475694, EPI_ISL_475695, EPI_ISL_475697, EPI_ISL_475698, EPI_ISL_475699, EPI_ISL_475700, EPI_ISL_475701, EPI_ISL_475702, EPI_ISL_475703, EPI_ISL_475705, EPI_ISL_475706, EPI_ISL_475708, EPI_ISL_475709, EPI_ISL_475710, EPI_ISL_475711, EPI_ISL_475712, EPI_ISL_475713, EPI_ISL_475714, EPI_ISL_475715, EPI_ISL_475716			
see above	Cedars-Sinai Medical Center, Department of Pathology & Laboratory Medicine, Molecular Pathology Laboratory	Cedars-Sinai Medical Center, Molecular Pathology Laboratory of Department of Pathology & Laboratory Medicine and Genomic Core	Wenjuan Zhang, John Paul Govindavari, Brian Davis, Stephanie Chen, Jong Taek Kim, Jianbo Song, Jean Lopategui, Jasmine T Plummer, Eric Vail
EPI_ISL_475745, EPI_ISL_475746, EPI_ISL_475747, EPI_ISL_475748, EPI_ISL_475749, EPI_ISL_475751, EPI_ISL_475752, EPI_ISL_475753	Medical Ain Shams Research Institute (MASRI), Ain Shams University	Medical Ain Shams Research Institute (MASRI), Ain Shams University	Hesham Elghazaly , Sara Hassan Agwa, Mahmoud Elmeteini , Ahmad Moustafa , Ashraf Omar, Osama Mansour, Samia Abdo, Hala Hafez, Ghada Ismael , Shaimaa Moustafa , Aya Mohamed, Reham Mamdouh , Hoda Abd Elsatar, Manal Hamdy Elsaid, Fatma Ebied
EPI_ISL_476022	Defence Research & Development Establishment	Defence Research & Development Establishment	Shashi Sharma, Paban Kumar Dash, Jyoti S Kumar, Sushil Kumar Sharma, Ambuj Shrivastava
EPI_ISL_476023	Defence Research & Development Establishment (DRDE)	Defence Research & Development Establishment (DRDE)	Shashi Sharma, Paban Kumar Dash, Sushil Kumar Sharma, Ambuj Shrivastava, Jyoti S. Kumar
EPI_ISL_476067	The National Institute of Public Health	State Veterinary Institute Prague and The National Institute of Public Health	Nagy,A;Jirincova,H;Novakova,L;Trnka,D;Vecerova,J
EPI_ISL_476702, EPI_ISL_476704	Incubadora Venezolana de Ciencia, Venezuela	Incubadora Venezolana de Ciencia, Venezuela / Instituto Nacional de Salud, Bogotá, Colombia / Grupo de Investigaciones Microbiológicas-UR (GIMUR), Departamento de Biología, Facultad de Ciencias Naturales, Universidad del Rosario, Bogotá, Colombia / Icahn School of Medicine at Mount Sinai, New York, USA	Alberto Paniz-Mondolfi, Marina Muñoz, Luis Perez-Garcia, Lourdes Delgado, Carolina Florez, Sergio Gomez, Angelica Rico, Lisseth Pardo, Esther C. Barros, Carolina Hernández, Jesús E. Jaimes, Aníbal A. Teherán, Ana S. Gonzalez-Reiche, Matthew M. Hernandez, Emilia Mia Sordillo, Viviana Simon, Harm van Bakel, Juan David Ramírez
EPI_ISL_476801, EPI_ISL_476802, EPI_ISL_476803, EPI_ISL_476804	Hong Kong Department of Health	School of Public Health, The University of Hong Kong	Dominic N.C. Tsang, Daniel K.W. Chu, Leo L.M. Poon, Malik Peiris
EPI_ISL_476814, EPI_ISL_476818, EPI_ISL_476819	Department of Laboratory Medicine, Tan Tock Seng Hospital	Department of Laboratory Medicine, Tan Tock Seng Hospital	Chen YYC, Zair X, Li C, Tang WY, Maurer-Stroh S, Barkham TMS, Nagarajan N, Sessions OM
EPI_ISL_476832	Medical Biology Department, Kocaeli University	Medical Genetics Department, Kocaeli University	Savli H, Cine N, Sunnetci-Akkoyunlu D, Eren-Keskin S, Ilgazli A, Akhan S, Karadenizli A, Kasap M, Sayan M, Akpinar G, Canturk NZ.
EPI_ISL_476840, EPI_ISL_476844, EPI_ISL_476846, EPI_ISL_476848, EPI_ISL_476849, EPI_ISL_476850, EPI_ISL_476852, EPI_ISL_476853, EPI_ISL_476854, EPI_ISL_476883	Defence Research & Development Establishment (DRDE)	Defence Research & Development Establishment (DRDE)	Shashi Sharma, Paban Kumar Dash, Sushil Kumar Sharma, Ambuj Shrivastava, Jyoti S. Kumar
EPI_ISL_477163	Laboratory of Dr. John Lednicky	University of Florida	John A. Lednicky, Maha A. Elbadry, Kuttichantran Subramaniam, Thomas B. Waltzek, John Glenn Morris, Jr.
EPI_ISL_477164	Department of Virology, Public Health Laboratories Division, National Institute of Health	Department of Virology, Public Health Laboratories Division, National Institute of Health	Nazish Badar, Aamer Ikram, Muhammad Salman, Hamza Ahmed Mirza, Abdul Ahad, Yasir Arshad, Massab Umair
EPI_ISL_477165, EPI_ISL_477167	Department of Virology, Public Health Laboratories Division, National Institute of Health	Department of Virology, Public Health Laboratories Division, National Institute of Health	Nazish Badar,Aamer Ikram, Muhammad Salman, Massab Umair, Hamza Ahmed Mirza, Abdul Ahad, Yasir Arshad
EPI_ISL_477170	Department of Laboratory, Medicine Tan Tock Seng Hospital	Department of Laboratory Medicine Tan Tock Seng Hospital	Chen YYC, Zair X, Li C, Tang WY, Maurer-Stroh S, Barkham TMS, Nagarajan N, Sessions OM
EPI_ISL_477171	Department of Laboratory, Medicine Tan Tock Seng Hospital	Department of Laboratory, Medicine Tan Tock Seng Hospital	Chen YYC, Zair X, Li C, Tang WY, Maurer-Stroh S, Barkham TMS, Nagarajan N, Sessions OM
EPI_ISL_477172, EPI_ISL_477174, EPI_ISL_477175, EPI_ISL_477177, EPI_ISL_477178, EPI_ISL_477182, EPI_ISL_477184, EPI_ISL_477187, EPI_ISL_477188, EPI_ISL_477189, EPI_ISL_477190, EPI_ISL_477191, EPI_ISL_477192			
see above	Department of Laboratory Medicine Tan Tock Seng Hospital	Department of Laboratory Medicine Tan Tock Seng Hospital	Chen YYC, Zair X, Li C, Tang WY, Maurer-Stroh S, Barkham TMS, Nagarajan N, Sessions OM
EPI_ISL_477193, EPI_ISL_477194, EPI_ISL_477202	Istituto Zooprofilattico Sperimentale Puglia e Basilicata;	Beaconlab (Bioinformatics, Evolution and Comparative Genomics lab), Dept of Biosciences, University on Mila	Parisi A.,Pesole G., Manzari C., Chiara M.
EPI_ISL_479482, EPI_ISL_479483, EPI_ISL_479484, EPI_ISL_479485, EPI_ISL_479486, EPI_ISL_479487, EPI_ISL_479488, EPI_ISL_479489, EPI_ISL_479490, EPI_ISL_479491, EPI_ISL_479492			

see above	Department of Laboratory Medicine Tan Tock Seng Hospital	Department of Laboratory Medicine Tan Tock Seng Hospital	Chen YYC, Zair X, Li C, Tang WY, Maurer-Stroh S, Barkham TMS, Nagarajan N, Sessions OM
EPI_ISL_479493, EPI_ISL_479495, EPI_ISL_479498, EPI_ISL_479499, EPI_ISL_479501, EPI_ISL_479502, EPI_ISL_479505, EPI_ISL_479507, EPI_ISL_479509, EPI_ISL_479510, EPI_ISL_479512, EPI_ISL_479513, EPI_ISL_479514, EPI_ISL_479516, EPI_ISL_479517, EPI_ISL_479519, EPI_ISL_479520, EPI_ISL_479521, EPI_ISL_479522, EPI_ISL_479523, EPI_ISL_479526, EPI_ISL_479527, EPI_ISL_479528, EPI_ISL_479529, EPI_ISL_479530, EPI_ISL_479531, EPI_ISL_479532, EPI_ISL_479533, EPI_ISL_479572			
see above	NIV Influenza	NIV Influenza	Potdar V
EPI_ISL_479616, EPI_ISL_479617	Laboratory of Molecular Virology of the International Centre for Genetic Engineering and Biotechnology (ICGEB)	ARGO Open Lab Platform for Genome Sequencing	Licastro, D, Rajasekharan S, Dal Monego S, Segat L, D'Agaro P, Salton F, Confalonieri P, Confalonieri M Marcello A
EPI_ISL_479618, EPI_ISL_479619	Laboratory of Molecular Virology of the International Centre for Genetic Engineering and Biotechnology (ICGEB)	ARGO Open Lab Platform for Genome Sequencing	Licastro, D, Rajasekharan S, Dal Monego S, Segat L, D'Agaro P, Salton F, Confalonieri P, Confalonieri M, Marcello A
EPI_ISL_479620, EPI_ISL_479621, EPI_ISL_479622, EPI_ISL_479623, EPI_ISL_479624	Molecular diagnostic laboratory of Federal Budget Institution of Science "Central Research Institute of Epidemiology" of The Federal Service on Customers' Rights Protection and Human Well-being Surveillance	Group of Genomics and Postgenomic Technologies of Central Research Institute of Epidemiology	Speranskaya AS, Kaptelova VV,Valdokhina AV, Bulanenko VP, Samoilov AE, Korneenko EV, Sizova TV, Tivanova EV, Shipulina OY, Akimkin VG
EPI_ISL_479657	NIV Influenza	NIV Influenza	Potdar V
EPI_ISL_479756, EPI_ISL_479757, EPI_ISL_479758	National Institute of Hygiene and Epidemiology (NIHE)	National Key Laboratory of Gene Technology, Institute of Biotechnology (IBT)	Le Tung Lam, Nguyen Hong Trang, Ho Thi Thuong, Tran Huyen Linh, Ung Thi Hong Trang, Le Thi Thanh, Nguyen Vu Son, Vuong Duc Cuong, Tran Thu Huong, Pham Thi Hien, Nguyen Phuong Anh, Nguyen Le Khanh Hang, Hoang Vu Mai Phuong, Hoang Ha, Taichiro Takemura, Futoshi Hasebe, Chu Hoang Ha, Le Quynh Mai, Dang Duc Anh, Truong Nam Hai
EPI_ISL_479790, EPI_ISL_479791	Laboratory of Molecular Virology of the International Centre for Genetic Engineering and Biotechnology (ICGEB)	ARGO Open Lab Platform for Genome Sequencing	Licastro, D, Rajasekharan S, Dal Monego S, Segat L, D'Agaro P, Salton F, Confalonieri P, Confalonieri M, Marcello A
EPI_ISL_479792, EPI_ISL_479793, EPI_ISL_479794, EPI_ISL_479795	Hokkaido Institute of Public Health	Pathogen Genomics Center, National Institute of Infectious Diseases	Tsuyoshi Sekizuka, Rika Komagome, Kentaro Itokawa, Rina Tanaka, Masanori Hashino, Hajime Kamiya, Motoi Suzuki, Makoto Kuroda
EPI_ISL_479796	Ishikawa Prefectural Institute of Public Health and Environmental Science	Pathogen Genomics Center, National Institute of Infectious Diseases	Tsuyoshi Sekizuka, Sanae Kuramoto, Eri Nariai, Kentaro Itokawa, Rina Tanaka, Masanori Hashino, Hajime Kamiya, Motoi Suzuki, Makoto Kuroda
EPI_ISL_479797, EPI_ISL_479798	Sagamihara City Public Health Research Institute	Pathogen Genomics Center, National Institute of Infectious Diseases	Tsuyoshi Sekizuka, Hiroshi Nakamura, Kentaro Itokawa, Rina Tanaka, Masanori Hashino, Hajime Kamiya, Motoi Suzuki, Makoto Kuroda
EPI_ISL_479799, EPI_ISL_479800	Sapporo City Institute of Public Health	Pathogen Genomics Center, National Institute of Infectious Diseases	Tsuyoshi Sekizuka, Asami Ohnishi, Kentaro Itokawa, Rina Tanaka, Masanori Hashino, Hajime Kamiya, Motoi Suzuki, Makoto Kuroda
EPI_ISL_479801	Hokkaido Institute of Public Health	Pathogen Genomics Center, National Institute of Infectious Diseases	Tsuyoshi Sekizuka, Rika Komagome, Kentaro Itokawa, Rina Tanaka, Masanori Hashino, Hajime Kamiya, Motoi Suzuki, Makoto Kuroda
EPI_ISL_479802, EPI_ISL_479803, EPI_ISL_479804	Sagamihara City Public Health Research Institute	Pathogen Genomics Center, National Institute of Infectious Diseases	Tsuyoshi Sekizuka, Hiroshi Nakamura, Kentaro Itokawa, Rina Tanaka, Masanori Hashino, Hajime Kamiya, Motoi Suzuki, Makoto Kuroda
EPI_ISL_479805, EPI_ISL_479806, EPI_ISL_479807, EPI_ISL_479808	Saitama Prefectural Institute of Public Health	Pathogen Genomics Center, National Institute of Infectious Diseases	Tsuyoshi Sekizuka, Hayato Ehara, Kentaro Itokawa, Rina Tanaka, Masanori Hashino, Hajime Kamiya, Motoi Suzuki, Makoto Kuroda
EPI_ISL_479809, EPI_ISL_479810, EPI_ISL_479811	Chiba Prefectural Institute of Public Health	Pathogen Genomics Center, National Institute of Infectious Diseases	Tsuyoshi Sekizuka, Masakatsu Taira, Kentaro Itokawa, Rina Tanaka, Masanori Hashino, Hajime Kamiya, Motoi Suzuki, Makoto Kuroda
EPI_ISL_479812, EPI_ISL_479813, EPI_ISL_479814, EPI_ISL_479815, EPI_ISL_479816, EPI_ISL_479817, EPI_ISL_479818, EPI_ISL_479819, EPI_ISL_479820	Hokkaido Institute of Public Health	Pathogen Genomics Center, National Institute of Infectious Diseases	Tsuyoshi Sekizuka, Rika Komagome, Kentaro Itokawa, Rina Tanaka, Masanori Hashino, Hajime Kamiya, Motoi Suzuki, Makoto Kuroda
EPI_ISL_479821, EPI_ISL_479822	Department of Infectious Diseases, Kobe Institute of Health	Pathogen Genomics Center, National Institute of Infectious Diseases	Tsuyoshi Sekizuka, Ryohei Nomoto, Kentaro Itokawa, Rina Tanaka, Masanori Hashino, Hajime Kamiya, Motoi Suzuki, Makoto Kuroda
EPI_ISL_479823	Kochi Prefectural Institute of Public Health	Pathogen Genomics Center, National Institute of Infectious Diseases	Tsuyoshi Sekizuka, Akihiko Tokaji, Kentaro Itokawa, Rina Tanaka, Masanori Hashino, Hajime Kamiya, Motoi Suzuki, Makoto Kuroda
EPI_ISL_479824	Kumamoto PrefecturalInstituteof Public-Health and Environmental Science	Pathogen Genomics Center, National Institute of Infectious Diseases	Tsuyoshi Sekizuka, Shunsuke Yahiro, Kentaro Itokawa, Rina Tanaka, Masanori Hashino, Hajime Kamiya, Motoi Suzuki, Makoto Kuroda
EPI_ISL_479825	Tokyo Metropolitan Institute of Public Health	Pathogen Genomics Center, National Institute of Infectious Diseases	Tsuyoshi Sekizuka, Kenji Sadamasu, Takashi Chiba, Mami Nagashima, Kentaro Itokawa, Rina Tanaka, Masanori Hashino, Hajime Kamiya, Motoi Suzuki, Makoto Kuroda
EPI_ISL_479826, EPI_ISL_479827, EPI_ISL_479828, EPI_ISL_479829, EPI_ISL_479830, EPI_ISL_479831, EPI_ISL_479832, EPI_ISL_479833, EPI_ISL_479834, EPI_ISL_479835, EPI_ISL_479836, EPI_ISL_479837, EPI_ISL_479838, EPI_ISL_479839, EPI_ISL_479840, EPI_ISL_479841, EPI_ISL_479842, EPI_ISL_479843, EPI_ISL_479844, EPI_ISL_479845, EPI_ISL_479846, EPI_ISL_479847, EPI_ISL_479848, EPI_ISL_479849			
see above	Sapporo City Institute of Public Health	Pathogen Genomics Center, National Institute of Infectious Diseases	Tsuyoshi Sekizuka, Asami Ohnishi, Kentaro Itokawa, Rina Tanaka, Masanori Hashino, Hajime Kamiya, Motoi Suzuki, Makoto Kuroda
EPI_ISL_479850, EPI_ISL_479851, EPI_ISL_479852, EPI_ISL_479853, EPI_ISL_479854	Gunma Prefectural Institute of Public Health and Environmental Sciences	Pathogen Genomics Center, National Institute of Infectious Diseases	Tsuyoshi Sekizuka, Hiroyuki Tsukagoshi, Kentaro Itokawa, Rina Tanaka, Masanori Hashino, Hajime Kamiya, Motoi Suzuki, Makoto Kuroda
EPI_ISL_479855, EPI_ISL_479856, EPI_ISL_479857, EPI_ISL_479858, EPI_ISL_479859, EPI_ISL_479860, EPI_ISL_479861	Department of Infectious Diseases, Kobe Institute of Health	Pathogen Genomics Center, National Institute of Infectious Diseases	Tsuyoshi Sekizuka, Ryohei Nomoto, Kentaro Itokawa, Rina Tanaka, Masanori Hashino, Hajime Kamiya, Motoi Suzuki, Makoto Kuroda
EPI_ISL_479862, EPI_ISL_479863, EPI_ISL_479864, EPI_ISL_479865, EPI_ISL_479866, EPI_ISL_479867	Wakayama Prefectural Research Center of Environment and Public Health	Pathogen Genomics Center, National Institute of Infectious Diseases	Tsuyoshi Sekizuka, Fumio Terasoma, Yosuke Hamajima, Kentaro Itokawa, Rina Tanaka, Masanori Hashino, Hajime Kamiya, Motoi Suzuki, Makoto Kuroda
EPI_ISL_479868	Department of Infectious Diseases, Kobe Institute of Health	Pathogen Genomics Center, National Institute of Infectious Diseases	Tsuyoshi Sekizuka, Ryohei Nomoto, Kentaro Itokawa, Rina Tanaka, Masanori Hashino, Hajime Kamiya, Motoi Suzuki, Makoto Kuroda
EPI_ISL_479869	Niigata Prefectural Institute of Public Health and Environmental Sciences	Pathogen Genomics Center, National Institute of Infectious Diseases	Tsuyoshi Sekizuka, Reiko Arai, Kentaro Itokawa, Rina Tanaka, Masanori Hashino, Hajime Kamiya, Motoi Suzuki, Makoto Kuroda
EPI_ISL_479870, EPI_ISL_479871	Sagamihara City Public Health Research Institute	Pathogen Genomics Center, National Institute of Infectious Diseases	Tsuyoshi Sekizuka, Hiroshi Nakamura, Kentaro Itokawa, Rina Tanaka, Masanori Hashino, Hajime Kamiya, Motoi Suzuki, Makoto Kuroda
EPI_ISL_479872, EPI_ISL_479873, EPI_ISL_479874, EPI_ISL_479875, EPI_ISL_479876, EPI_ISL_479877, EPI_ISL_479878, EPI_ISL_479879, EPI_ISL_479880, EPI_ISL_479881, EPI_ISL_479882, EPI_ISL_479883, EPI_ISL_479884, EPI_ISL_479885			
see above	Sapporo City Institute of Public Health	Pathogen Genomics Center, National Institute of Infectious Diseases	Tsuyoshi Sekizuka, Asami Ohnishi, Kentaro Itokawa, Rina Tanaka, Masanori Hashino, Hajime Kamiya, Motoi Suzuki, Makoto Kuroda
EPI_ISL_479886, EPI_ISL_479887, EPI_ISL_479888, EPI_ISL_479889,	Tokyo Metropolitan Institute of Public Health	Pathogen Genomics Center, National Institute of Infectious Diseases	Tsuyoshi Sekizuka, Kenji Sadamasu, Takashi Chiba, Mami Nagashima, Kentaro Itokawa, Rina Tanaka, Masanori Hashino, Hajime Kamiya, Motoi Suzuki, Makoto Kuroda

EPI_ISL_479890, EPI_ISL_479891, EPI_ISL_479892, EPI_ISL_479893, EPI_ISL_479894, EPI_ISL_479895			
EPI_ISL_479896, EPI_ISL_479897, EPI_ISL_479898, EPI_ISL_479899, EPI_ISL_479900, EPI_ISL_479901	Gunma Prefectural Institute of Public Health and Environmental Sciences	Pathogen Genomics Center, National Institute of Infectious Diseases	Tsuyoshi Sekizuka, Hiroyuki Tsukagoshi, Kentaro Itokawa, Rina Tanaka, Masanori Hashino, Hajime Kamiya, Motoi Suzuki, Makoto Kuroda
EPI_ISL_479902	Niigata Prefectural Institute of Public Health and Environmental Sciences	Pathogen Genomics Center, National Institute of Infectious Diseases	Tsuyoshi Sekizuka, Reiko Arai, Kentaro Itokawa, Rina Tanaka, Masanori Hashino, Hajime Kamiya, Motoi Suzuki, Makoto Kuroda
EPI_ISL_479903, EPI_ISL_479904, EPI_ISL_479905, EPI_ISL_479906, EPI_ISL_479907, EPI_ISL_479908, EPI_ISL_479909, EPI_ISL_479910, EPI_ISL_479911, EPI_ISL_479912	Himeji City Institute of Environment and Health	Pathogen Genomics Center, National Institute of Infectious Diseases	Tsuyoshi Sekizuka, Kentaro Itokawa, Rina Tanaka, Masanori Hashino, Hajime Kamiya, Motoi Suzuki, Makoto Kuroda
EPI_ISL_479913, EPI_ISL_479914, EPI_ISL_479915, EPI_ISL_479916, EPI_ISL_479917, EPI_ISL_479918, EPI_ISL_479919, EPI_ISL_479920, EPI_ISL_479921, EPI_ISL_479922, EPI_ISL_479923, EPI_ISL_479924			
see above	Niigata City Public Health Research Institute	Pathogen Genomics Center, National Institute of Infectious Diseases	Tsuyoshi Sekizuka, Yurie Takahashi, Kentaro Itokawa, Rina Tanaka, Masanori Hashino, Hajime Kamiya, Motoi Suzuki, Makoto Kuroda
EPI_ISL_479925, EPI_ISL_479926, EPI_ISL_479927	Sakai City Institute of Public Health	Pathogen Genomics Center, National Institute of Infectious Diseases	Tsuyoshi Sekizuka, Tatsuya Miyoshi, Kentaro Itokawa, Rina Tanaka, Masanori Hashino, Hajime Kamiya, Motoi Suzuki, Makoto Kuroda
EPI_ISL_479928, EPI_ISL_479929, EPI_ISL_479930, EPI_ISL_479931, EPI_ISL_479932, EPI_ISL_479933, EPI_ISL_479934, EPI_ISL_479935	Saitama Prefectural Institute of Public Health	Pathogen Genomics Center, National Institute of Infectious Diseases	Tsuyoshi Sekizuka, Hayato Ehara, Kentaro Itokawa, Rina Tanaka, Masanori Hashino, Hajime Kamiya, Motoi Suzuki, Makoto Kuroda
EPI_ISL_479936, EPI_ISL_479937, EPI_ISL_479938, EPI_ISL_479939, EPI_ISL_479940, EPI_ISL_479941, EPI_ISL_479942, EPI_ISL_479943	Ibaraki Prefectural Institute of Public Health	Pathogen Genomics Center, National Institute of Infectious Diseases	Tsuyoshi Sekizuka, Keiko Goto, Kentaro Itokawa, Rina Tanaka, Masanori Hashino, Hajime Kamiya, Motoi Suzuki, Makoto Kuroda
EPI_ISL_479944, EPI_ISL_479945, EPI_ISL_479946, EPI_ISL_479947, EPI_ISL_479948, EPI_ISL_479949, EPI_ISL_479950, EPI_ISL_479951, EPI_ISL_479952, EPI_ISL_479953, EPI_ISL_479954, EPI_ISL_479955, EPI_ISL_479956, EPI_ISL_479957, EPI_ISL_479958			
see above	Osaka Institute of Public Health	Pathogen Genomics Center, National Institute of Infectious Diseases	Tsuyoshi Sekizuka, Satoshi Hiroi, Saeko Morikawa, Kazushi Motomura, Kentaro Itokawa, Rina Tanaka, Masanori Hashino, Hajime Kamiya, Motoi Suzuki, Makoto Kuroda
EPI_ISL_479959, EPI_ISL_479960, EPI_ISL_479961, EPI_ISL_479962, EPI_ISL_479963, EPI_ISL_479964, EPI_ISL_479965	Tokyo Metropolitan Institute of Public Health	Pathogen Genomics Center, National Institute of Infectious Diseases	Tsuyoshi Sekizuka, Kenji Sadamasu, Takashi Chiba, Mami Nagashima, Kentaro Itokawa, Rina Tanaka, Masanori Hashino, Hajime Kamiya, Motoi Suzuki, Makoto Kuroda
EPI_ISL_479966	Osaka Institute of Public Health	Pathogen Genomics Center, National Institute of Infectious Diseases	Tsuyoshi Sekizuka, Satoshi Hiroi, Saeko Morikawa, Kazushi Motomura, Kentaro Itokawa, Rina Tanaka, Masanori Hashino, Hajime Kamiya, Motoi Suzuki, Makoto Kuroda
EPI_ISL_479967, EPI_ISL_479968, EPI_ISL_479969, EPI_ISL_479970, EPI_ISL_479971, EPI_ISL_479972, EPI_ISL_479973, EPI_ISL_479974, EPI_ISL_479975, EPI_ISL_479976, EPI_ISL_479977, EPI_ISL_479978			
see above	Fukui Prefectural Institute of Public Health and Environmental Science	Pathogen Genomics Center, National Institute of Infectious Diseases	Tsuyoshi Sekizuka, Miho Toho, Kentaro Itokawa, Rina Tanaka, Masanori Hashino, Hajime Kamiya, Motoi Suzuki, Makoto Kuroda
EPI_ISL_479979, EPI_ISL_479980, EPI_ISL_479981, EPI_ISL_479982, EPI_ISL_479983, EPI_ISL_479984	Oita Prefectural Institute of Public Health and Environmental Science	Pathogen Genomics Center, National Institute of Infectious Diseases	Tsuyoshi Sekizuka, Mari Sasaki, Kentaro Itokawa, Rina Tanaka, Masanori Hashino, Hajime Kamiya, Motoi Suzuki, Makoto Kuroda
EPI_ISL_479985	Ibaraki Prefectural Institute of Public Health	Pathogen Genomics Center, National Institute of Infectious Diseases	Tsuyoshi Sekizuka, Keiko Goto, Kentaro Itokawa, Rina Tanaka, Masanori Hashino, Hajime Kamiya, Motoi Suzuki, Makoto Kuroda
EPI_ISL_479986, EPI_ISL_479987, EPI_ISL_479988, EPI_ISL_479989	Department of Infectious Diseases, Kobe Institute of Health	Pathogen Genomics Center, National Institute of Infectious Diseases	Tsuyoshi Sekizuka, Ryohei Nomoto, Kentaro Itokawa, Rina Tanaka, Masanori Hashino, Hajime Kamiya, Motoi Suzuki, Makoto Kuroda
EPI_ISL_479990	Kitakyushu City Institute of Health and Environmental Sciences	Pathogen Genomics Center, National Institute of Infectious Diseases	Tsuyoshi Sekizuka, Katsuya Obata, Asuka Kikuchi Kentaro Itokawa, Rina Tanaka, Masanori Hashino, Hajime Kamiya, Motoi Suzuki, Makoto Kuroda
EPI_ISL_479991, EPI_ISL_479992, EPI_ISL_479993, EPI_ISL_479994, EPI_ISL_479995, EPI_ISL_479996	Kumamoto City Public Health Research Institute	Pathogen Genomics Center, National Institute of Infectious Diseases	Tsuyoshi Sekizuka, Kaori Tashiro, Kentaro Itokawa, Rina Tanaka, Masanori Hashino, Hajime Kamiya, Motoi Suzuki, Makoto Kuroda
EPI_ISL_479997, EPI_ISL_479998, EPI_ISL_479999, EPI_ISL_480000, EPI_ISL_480001	Nagano Environmental Conservation Research Institute	Pathogen Genomics Center, National Institute of Infectious Diseases	Tsuyoshi Sekizuka, Naoko Shimodaira, Kentaro Itokawa, Rina Tanaka, Masanori Hashino, Hajime Kamiya, Motoi Suzuki, Makoto Kuroda
EPI_ISL_480002, EPI_ISL_480003	Nagasaki Prefectural Institute for Environmental Research and Public Health	Pathogen Genomics Center, National Institute of Infectious Diseases	Tsuyoshi Sekizuka, Fumiaki Matsumoto, Kentaro Itokawa, Rina Tanaka, Masanori Hashino, Hajime Kamiya, Motoi Suzuki, Makoto Kuroda
EPI_ISL_480004, EPI_ISL_480005, EPI_ISL_480006, EPI_ISL_480007, EPI_ISL_480008, EPI_ISL_480009, EPI_ISL_480010, EPI_ISL_480011, EPI_ISL_480012, EPI_ISL_480013, EPI_ISL_480014			
see above	Chiba Prefectural Institute of Public Health	Pathogen Genomics Center, National Institute of Infectious Diseases	Tsuyoshi Sekizuka, Masakatsu Taira, Kentaro Itokawa, Rina Tanaka, Masanori Hashino, Hajime Kamiya, Motoi Suzuki, Makoto Kuroda
EPI_ISL_480015, EPI_ISL_480016, EPI_ISL_480017, EPI_ISL_480018, EPI_ISL_480019, EPI_ISL_480020	Gunma Prefectural Institute of Public Health and Environmental Sciences	Pathogen Genomics Center, National Institute of Infectious Diseases	Tsuyoshi Sekizuka, Hiroyuki Tsukagoshi, Kentaro Itokawa, Rina Tanaka, Masanori Hashino, Hajime Kamiya, Motoi Suzuki, Makoto Kuroda
EPI_ISL_480021, EPI_ISL_480022, EPI_ISL_480023, EPI_ISL_480024, EPI_ISL_480025, EPI_ISL_480026, EPI_ISL_480027, EPI_ISL_480028, EPI_ISL_480029	Ibaraki Prefectural Institute of Public Health	Pathogen Genomics Center, National Institute of Infectious Diseases	Tsuyoshi Sekizuka, Keiko Goto, Kentaro Itokawa, Rina Tanaka, Masanori Hashino, Hajime Kamiya, Motoi Suzuki, Makoto Kuroda
EPI_ISL_480030, EPI_ISL_480031, EPI_ISL_480032, EPI_ISL_480033, EPI_ISL_480034, EPI_ISL_480035, EPI_ISL_480036, EPI_ISL_480037, EPI_ISL_480038, EPI_ISL_480039, EPI_ISL_480040, EPI_ISL_480041			
see above	Tochigi Prefectural Institute of Public Health and Environmental Science	Pathogen Genomics Center, National Institute of Infectious Diseases	Tsuyoshi Sekizuka, Ako Nakajima, Kentaro Itokawa, Rina Tanaka, Masanori Hashino, Hajime Kamiya, Motoi Suzuki, Makoto Kuroda
EPI_ISL_480042, EPI_ISL_480043, EPI_ISL_480044, EPI_ISL_480045, EPI_ISL_480046, EPI_ISL_480047, EPI_ISL_480048, EPI_ISL_480049, EPI_ISL_480050, EPI_ISL_480051, EPI_ISL_480052, EPI_ISL_480053, EPI_ISL_480054, EPI_ISL_480055, EPI_ISL_480056, EPI_ISL_480057, EPI_ISL_480058, EPI_ISL_480059, EPI_ISL_480060, EPI_ISL_480061, EPI_ISL_480062, EPI_ISL_480063, EPI_ISL_480064			
see above	Nagoya City Public Health Research Institute	Pathogen Genomics Center, National Institute of Infectious Diseases	Tsuyoshi Sekizuka, Takuya Miki, Shinichiro Shibata, Kentaro Itokawa, Rina Tanaka, Masanori Hashino, Hajime Kamiya, Motoi Suzuki, Makoto Kuroda

EPI_ISL_480065, EPI_ISL_480066, EPI_ISL_480067, EPI_ISL_480068, EPI_ISL_480069, EPI_ISL_480070, EPI_ISL_480071, EPI_ISL_480072	Sakai City Institute of Public Health	Pathogen Genomics Center, National Institute of Infectious Diseases	Tsuyoshi Sekizuka, Tatsuya Miyoshi, Kentaro Itokawa, Rina Tanaka, Masanori Hashino, Hajime Kamiya, Motoi Suzuki, Makoto Kuroda
EPI_ISL_480073	Tochigi Prefectural Institute of Public Health and Environmental Science	Pathogen Genomics Center, National Institute of Infectious Diseases	Tsuyoshi Sekizuka, Ako Nakajima, Kentaro Itokawa, Rina Tanaka, Masanori Hashino, Hajime Kamiya, Motoi Suzuki, Makoto Kuroda
EPI_ISL_480074, EPI_ISL_480075, EPI_ISL_480076, EPI_ISL_480077, EPI_ISL_480078, EPI_ISL_480079, EPI_ISL_480080, EPI_ISL_480081, EPI_ISL_480082	Shizuoka City Institute of Environmental Sciences and Public Health	Pathogen Genomics Center, National Institute of Infectious Diseases	Tsuyoshi Sekizuka, Takaharu Maehata,Sou Okamura,Yuji Kanazawa,Kenji Yagi, Kentaro Itokawa, Rina Tanaka, Masanori Hashino, Hajime Kamiya, Motoi Suzuki, Makoto Kuroda
EPI_ISL_480083, EPI_ISL_480084, EPI_ISL_480085, EPI_ISL_480086, EPI_ISL_480087, EPI_ISL_480088, EPI_ISL_480089	Gifu Prefectural Institute of Public Health and Environmental Sciences	Pathogen Genomics Center, National Institute of Infectious Diseases	Tsuyoshi Sekizuka, Yoshihiko Kameyama, Kentaro Itokawa, Rina Tanaka, Masanori Hashino, Hajime Kamiya, Motoi Suzuki, Makoto Kuroda
EPI_ISL_480090, EPI_ISL_480091, EPI_ISL_480092, EPI_ISL_480093, EPI_ISL_480094, EPI_ISL_480095, EPI_ISL_480097, EPI_ISL_480098, EPI_ISL_480099, EPI_ISL_480100, EPI_ISL_480101, EPI_ISL_480102			
see above	Department of Infectious Diseases, Kobe Institute of Health	Pathogen Genomics Center, National Institute of Infectious Diseases	Tsuyoshi Sekizuka, Ryohei Nomoto, Kentaro Itokawa, Rina Tanaka, Masanori Hashino, Hajime Kamiya, Motoi Suzuki, Makoto Kuroda
EPI_ISL_480103, EPI_ISL_480104, EPI_ISL_480105, EPI_ISL_480106, EPI_ISL_480107, EPI_ISL_480108	Koshigaya City Public Health Center	Pathogen Genomics Center, National Institute of Infectious Diseases	Tsuyoshi Sekizuka, Yuka Furui, Aya Tamura, Kyohei Sakata, Takumi Daimon, Yoko Togawa, Yoshiko Hamada, Kentaro Itokawa, Rina Tanaka, Masanori Hashino, Hajime Kamiya, Motoi Suzuki, Makoto Kuroda
EPI_ISL_480109, EPI_ISL_480110, EPI_ISL_480111, EPI_ISL_480112, EPI_ISL_480113, EPI_ISL_480114, EPI_ISL_480115, EPI_ISL_480116, EPI_ISL_480117, EPI_ISL_480118, EPI_ISL_480119			
see above	Oita Prefectural Institute of Public Health and Environmental Science	Pathogen Genomics Center, National Institute of Infectious Diseases	Tsuyoshi Sekizuka, Mari Sasaki, Kentaro Itokawa, Rina Tanaka, Masanori Hashino, Hajime Kamiya, Motoi Suzuki, Makoto Kuroda
EPI_ISL_480120, EPI_ISL_480121, EPI_ISL_480122, EPI_ISL_480123, EPI_ISL_480124, EPI_ISL_480125, EPI_ISL_480126, EPI_ISL_480127, EPI_ISL_480128, EPI_ISL_480129, EPI_ISL_480130, EPI_ISL_480131, EPI_ISL_480132, EPI_ISL_480133, EPI_ISL_480134, EPI_ISL_480135, EPI_ISL_480136, EPI_ISL_480137, EPI_ISL_480138, EPI_ISL_480139, EPI_ISL_480140, EPI_ISL_480141, EPI_ISL_480142, EPI_ISL_480143, EPI_ISL_480144, EPI_ISL_480145, EPI_ISL_480146, EPI_ISL_480147, EPI_ISL_480148, EPI_ISL_480149, EPI_ISL_480150, EPI_ISL_480151, EPI_ISL_480152, EPI_ISL_480153, EPI_ISL_480154, EPI_ISL_480155, EPI_ISL_480156, EPI_ISL_480157, EPI_ISL_480158, EPI_ISL_480159, EPI_ISL_480160, EPI_ISL_480161, EPI_ISL_480162, EPI_ISL_480163, EPI_ISL_480164, EPI_ISL_480165, EPI_ISL_480166, EPI_ISL_480167, EPI_ISL_480168			
see above	Fukui Prefectural Institute of Public Health and Environmental Science	Pathogen Genomics Center, National Institute of Infectious Diseases	Tsuyoshi Sekizuka, Miho Toho, Kentaro Itokawa, Rina Tanaka, Masanori Hashino, Hajime Kamiya, Motoi Suzuki, Makoto Kuroda
EPI_ISL_480169, EPI_ISL_480170, EPI_ISL_480171, EPI_ISL_480172, EPI_ISL_480173, EPI_ISL_480174, EPI_ISL_480175, EPI_ISL_480176, EPI_ISL_480177	Gunma Prefectural Institute of Public Health and Environmental Sciences	Pathogen Genomics Center, National Institute of Infectious Diseases	Tsuyoshi Sekizuka, Hiroyuki Tsukagoshi, Kentaro Itokawa, Rina Tanaka, Masanori Hashino, Hajime Kamiya, Motoi Suzuki, Makoto Kuroda
EPI_ISL_480178, EPI_ISL_480179	Hiroshima City Institute of Public Health	Pathogen Genomics Center, National Institute of Infectious Diseases	Tsuyoshi Sekizuka, Kota Noritsune, Kentaro Itokawa, Rina Tanaka, Masanori Hashino, Hajime Kamiya, Motoi Suzuki, Makoto Kuroda
EPI_ISL_480180, EPI_ISL_480181, EPI_ISL_480182, EPI_ISL_480183, EPI_ISL_480184, EPI_ISL_480185, EPI_ISL_480186, EPI_ISL_480187, EPI_ISL_480188, EPI_ISL_480189	Ibaraki Prefectural Institute of Public Health	Pathogen Genomics Center, National Institute of Infectious Diseases	Tsuyoshi Sekizuka, Keiko Goto, Kentaro Itokawa, Rina Tanaka, Masanori Hashino, Hajime Kamiya, Motoi Suzuki, Makoto Kuroda
EPI_ISL_480190, EPI_ISL_480191, EPI_ISL_480192, EPI_ISL_480193, EPI_ISL_480194, EPI_ISL_480195	Ota Health Center Welfare Section	Pathogen Genomics Center, National Institute of Infectious Diseases	Tsuyoshi Sekizuka, Chika Takahashi, Kentaro Itokawa, Rina Tanaka, Masanori Hashino, Hajime Kamiya, Motoi Suzuki, Makoto Kuroda
EPI_ISL_480196, EPI_ISL_480197, EPI_ISL_480198, EPI_ISL_480199, EPI_ISL_480200, EPI_ISL_480201, EPI_ISL_480202, EPI_ISL_480203	Toyama Institute of Health	Pathogen Genomics Center, National Institute of Infectious Diseases	Tsuyoshi Sekizuka, Masae Itamochi, Kazunori Oishi, Kentaro Itokawa, Rina Tanaka, Masanori Hashino, Hajime Kamiya, Motoi Suzuki, Makoto Kuroda
EPI_ISL_480204	Akita City Public Health Center	Pathogen Genomics Center, National Institute of Infectious Diseases	Tsuyoshi Sekizuka, Koichi Ito, Kentaro Itokawa, Rina Tanaka, Masanori Hashino, Hajime Kamiya, Motoi Suzuki, Makoto Kuroda
EPI_ISL_480205, EPI_ISL_480206, EPI_ISL_480207	Department of Infectious Diseases, Kobe Institute of Health	Pathogen Genomics Center, National Institute of Infectious Diseases	Tsuyoshi Sekizuka, Ryohei Nomoto, Kentaro Itokawa, Rina Tanaka, Masanori Hashino, Hajime Kamiya, Motoi Suzuki, Makoto Kuroda
EPI_ISL_480208	Pathogen Genomics Center, National Institute of Infectious Diseases	Pathogen Genomics Center, National Institute of Infectious Diseases	Tsuyoshi Sekizuka, Ryohei Nomoto, Kentaro Itokawa, Rina Tanaka, Masanori Hashino, Hajime Kamiya, Motoi Suzuki, Makoto Kuroda
EPI_ISL_480209, EPI_ISL_480210, EPI_ISL_480211, EPI_ISL_480212, EPI_ISL_480213, EPI_ISL_480214, EPI_ISL_480215, EPI_ISL_480216, EPI_ISL_480217, EPI_ISL_480218, EPI_ISL_480219, EPI_ISL_480220			
see above	Department of Infectious Diseases, Kobe Institute of Health	Pathogen Genomics Center, National Institute of Infectious Diseases	Tsuyoshi Sekizuka, Ryohei Nomoto, Kentaro Itokawa, Rina Tanaka, Masanori Hashino, Hajime Kamiya, Motoi Suzuki, Makoto Kuroda
EPI_ISL_480221, EPI_ISL_480222, EPI_ISL_480223	Koshigaya City Public Health Center	Pathogen Genomics Center, National Institute of Infectious Diseases	Tsuyoshi Sekizuka, Yuka Furui, Aya Tamura, Kyohei Sakata, Takumi Daimon, Yoko Togawa, Yoshiko Hamada, Kentaro Itokawa, Rina Tanaka, Masanori Hashino, Hajime Kamiya, Motoi Suzuki, Makoto Kuroda
EPI_ISL_480228, EPI_ISL_480229, EPI_ISL_480230, EPI_ISL_480231, EPI_ISL_480232, EPI_ISL_480233, EPI_ISL_480234, EPI_ISL_480235, EPI_ISL_480236, EPI_ISL_480237, EPI_ISL_480238, EPI_ISL_480239, EPI_ISL_480240, EPI_ISL_480241, EPI_ISL_480242, EPI_ISL_480243, EPI_ISL_480244, EPI_ISL_480245, EPI_ISL_480246, EPI_ISL_480247, EPI_ISL_480248, EPI_ISL_480249, EPI_ISL_480250, EPI_ISL_480252, EPI_ISL_480253, EPI_ISL_480254, EPI_ISL_480255, EPI_ISL_480256, EPI_ISL_480257, EPI_ISL_480258, EPI_ISL_480259, EPI_ISL_480260, EPI_ISL_480261, EPI_ISL_480262, EPI_ISL_480263, EPI_ISL_480264, EPI_ISL_480265, EPI_ISL_480266, EPI_ISL_480267, EPI_ISL_480268, EPI_ISL_480269, EPI_ISL_480270, EPI_ISL_480271, EPI_ISL_480272, EPI_ISL_480273, EPI_ISL_480274, EPI_ISL_480277, EPI_ISL_480278, EPI_ISL_480281, EPI_ISL_480282, EPI_ISL_480283, EPI_ISL_480284, EPI_ISL_480285, EPI_ISL_480286, EPI_ISL_480289, EPI_ISL_480291, EPI_ISL_480292			
see above	Genomic Laboratory (GLAB) (Conjoint lab of Health Directorate of Istanbul and Istanbul Technical University)	Genomic Laboratory (GLAB), Istanbul Technical University	Ilker Karacan, Tugba Kizilboga Akgun, Bugra Agaoglu, Gizem Alkurt, Jale Yildiz, Betsi Köse, Elifnaz Çelik, Arzu Irvem, Yasemin Kendir Demirkol, Ozlem Akgun Dogan, Mehtap Aydn, Levent Doganay, Gizem Dinler Doganay
EPI_ISL_480310	National Reference Laboratory "Influenza and acute respiratory diseases"	NRL-HIV	Ivan Ivanov, Ivailo Alexiev, Ivva Philipova
EPI_ISL_480554, EPI_ISL_480556, EPI_ISL_480783, EPI_ISL_480787, EPI_ISL_480789, EPI_ISL_481235, EPI_ISL_481236, EPI_ISL_481237, EPI_ISL_481239, EPI_ISL_481240, EPI_ISL_481243			
see above	Institut Pasteur Dakar	Institut Pasteur de Dakar	Ndongo Dia, Moussa Moise Diagne, Mamadou Diop, Marie Henriette Dior Ndione, Mamadou Malado Jallow, Safietou Sanke, Ousmane Faye, Amadou Alpha Sall.
EPI_ISL_482575, EPI_ISL_482576, EPI_ISL_482577, EPI_ISL_482578, EPI_ISL_482579, EPI_ISL_482580, EPI_ISL_482581, EPI_ISL_482582, EPI_ISL_482583, EPI_ISL_482584, EPI_ISL_482585, EPI_ISL_482586			
see above	Hangzhou Center for Diseases Control and Prevention	Hangzhou Center for Diseases Control and Prevention	Jun Li, Haoqiu Wang, Lingfeng Mao, Hua Yu, Xinfen Yu, Zhou Sun, Xin Qian, Shuchang Chen, Junfang Chen, Xuchu Wang
EPI_ISL_482672, EPI_ISL_482673,	Singapore General Hospital	Department of Microbiology	Nurdyana Abdul Rahman, Kun Lee Lim, Chenhao Li, Kian Sing Chan, Lynette Oon, Kern Rei Chng, Niranjan Nagarajan, Karrie Ko

EPI_ISL_482674, EPI_ISL_482675, EPI_ISL_482676, EPI_ISL_482677, EPI_ISL_482680			
EPI_ISL_482759, EPI_ISL_482760, EPI_ISL_482761, EPI_ISL_482762, EPI_ISL_482763, EPI_ISL_482764, EPI_ISL_482765, EPI_ISL_482766, EPI_ISL_482767, EPI_ISL_482768, EPI_ISL_482769, EPI_ISL_482770, EPI_ISL_482771, EPI_ISL_482772, EPI_ISL_482773, EPI_ISL_482774			
see above	Medical Ain Shams Research Institute (MASRI), Ain Shams University	Medical Ain Shams Research Institute (MASRI), Ain Shams University	Hesham Elghazaly, Sara Hassan Agwa, Ahmad Moustafa, Hala Hafez, Sara Elnakeep, Shaimaa Moustafa, Aya Mohamed, Reham Mamdouh, Ghada Ismael, Ashraf Omar, Osama Mansour, Mahmoud Elmeitini
EPI_ISL_482777	Queen Elizabeth Hospital	Hong Kong Department of Health	Mak Gannon C.K., Cheng Peter K.C., Lam Edman T.K., Chan Rickjason C.W., Tsang Dominic N.C.
EPI_ISL_483035, EPI_ISL_483036, EPI_ISL_483038	Medical Ain Shams Research Institute (MASRI), Ain Shams University	Medical Ain Shams Research Institute (MASRI), Ain Shams University	Hesham Elghazaly, Sara Hassan Agwa, Ahmad Moustafa, Hala Hafez, Sara Elnakeep, Shaimaa Moustafa, Aya Mohamed, Reham Mamdouh, Ghada Ismael, Ashraf Omar, Osama Mansour, Mahmoud Elmeitini
EPI_ISL_483059	Hospital Universitari Germans Trias i Pujol	IrsiCaixa AIDS Research Lab	J. Segalés, M. Puig, J. Rodon, C. Avila-Nieto, J. Carrillo, G. Cantero, M.T. Terrón, S. Cruz, M. Parera ,M. Noguera-Julián, N. Izquierdo-Useros, V. Guallar, E. Vidal, A. Valencia, I. Blanco, J. Blanco, B. Clotet, J. Vergara-Alert
EPI_ISL_483637	National Laboratory of Virology, Szentágothai Research Centre	National Laboratory of Virology, Szentágothai Research Centre	Endre Gábor Tóth, Balázs Somogyi, Ferenc Jakab, Gábor Kemenesi
EPI_ISL_486382	District Surveillence Unit	Department of Neurovirology, National Institute of Mental Health and Neuroscience (NIMHANS)	Chitra Pattabiraman, Vijayalakshmi Reddy, Harsha PK, Risha Rasheed, Shafeeq S Hameed, Manjunatha Venkataswamy, Anita Desai, Ravi Vasanthapuram
EPI_ISL_486383	CV Raman Hospital	Department of Neurovirology, National Institute of Mental Health and Neuroscience (NIMHANS)	Chitra Pattabiraman, Vijayalakshmi Reddy, Harsha PK, Risha Rasheed, Shafeeq S Hameed, Manjunatha Venkataswamy, Anita Desai, Ravi Vasanthapuram
EPI_ISL_486388, EPI_ISL_486389	DH	Department of Neurovirology, National Institute of Mental Health and Neuroscience (NIMHANS)	Chitra Pattabiraman, Vijayalakshmi Reddy, Harsha PK, Risha Rasheed, Shafeeq S Hameed, Manjunatha Venkataswamy, Anita Desai, Ravi Vasanthapuram
EPI_ISL_486392	Victoria Hospital	Department of Neurovirology, National Institute of Mental Health and Neuroscience (NIMHANS)	Chitra Pattabiraman, Vijayalakshmi Reddy, Harsha PK, Risha Rasheed, Shafeeq S Hameed, Manjunatha Venkataswamy, Anita Desai, Ravi Vasanthapuram
EPI_ISL_486394	MIMS	Department of Neurovirology, National Institute of Mental Health and Neuroscience (NIMHANS)	Chitra Pattabiraman, Vijayalakshmi Reddy, Harsha PK, Risha Rasheed, Shafeeq S Hameed, Manjunatha Venkataswamy, Anita Desai, Ravi Vasanthapuram
EPI_ISL_486395	BIMS	Department of Neurovirology, National Institute of Mental Health and Neuroscience (NIMHANS)	Chitra Pattabiraman, Vijayalakshmi Reddy, Harsha PK, Risha Rasheed, Shafeeq S Hameed, Manjunatha Venkataswamy, Anita Desai, Ravi Vasanthapuram
EPI_ISL_486397	KC General Hospital	Department of Neurovirology, National Institute of Mental Health and Neuroscience (NIMHANS)	Chitra Pattabiraman, Vijayalakshmi Reddy, Harsha PK, Risha Rasheed, Shafeeq S Hameed, Manjunatha Venkataswamy, Anita Desai, Ravi Vasanthapuram
EPI_ISL_486398, EPI_ISL_486399	MIMS	Department of Neurovirology, National Institute of Mental Health and Neuroscience (NIMHANS)	Chitra Pattabiraman, Vijayalakshmi Reddy, Harsha PK, Risha Rasheed, Shafeeq S Hameed, Manjunatha Venkataswamy, Anita Desai, Ravi Vasanthapuram
EPI_ISL_486400	Victoria Hospital	Department of Neurovirology, National Institute of Mental Health and Neuroscience (NIMHANS)	Chitra Pattabiraman, Vijayalakshmi Reddy, Harsha PK, Risha Rasheed, Shafeeq S Hameed, Manjunatha Venkataswamy, Anita Desai, Ravi Vasanthapuram
EPI_ISL_486405, EPI_ISL_486408, EPI_ISL_486409	DH	Department of Neurovirology, National Institute of Mental Health and Neuroscience (NIMHANS)	Chitra Pattabiraman, Vijayalakshmi Reddy, Harsha PK, Risha Rasheed, Shafeeq S Hameed, Manjunatha Venkataswamy, Anita Desai, Ravi Vasanthapuram
EPI_ISL_486815, EPI_ISL_486816, EPI_ISL_486817, EPI_ISL_486818, EPI_ISL_486819, EPI_ISL_486820, EPI_ISL_486821, EPI_ISL_486822, EPI_ISL_486823, EPI_ISL_486824, EPI_ISL_486825, EPI_ISL_486826, EPI_ISL_486827, EPI_ISL_486828, EPI_ISL_486829			
see above	Molecular diagnostic laboratory of Federal Budget Institution of Science "Central Research Institute of Epidemiology" of The Federal Service on Customers' Rights Protection and Human Well-being Surveillance	Group of Genomics and Postgenomic Technologies of Central Research Institute of Epidemilology	Speranskaya AS, Kaptelova VV, Valdokhina AV, Bulanenko VP, Samoilov AE, Korneenko EV, Tivanova EV, Shipulina OY, Akimkin VG
EPI_ISL_486856	Emergency County Hospital	Stefan cel Mare, University Metagenomics lab	Lobiuc Andrei et al.
EPI_ISL_486859, EPI_ISL_486860, EPI_ISL_486861, EPI_ISL_486862, EPI_ISL_486863, EPI_ISL_486864, EPI_ISL_486865, EPI_ISL_486866, EPI_ISL_486867, EPI_ISL_486868, EPI_ISL_486870, EPI_ISL_486871, EPI_ISL_486872, EPI_ISL_486873			
see above	Institut Pasteur Dakar	Institut Pasteur de Dakar	Ndongo Dia, Moussa Moise Diagne, Mamadou Diop, Marie Henriette Dior Ndione, Mamadou Malado Jallow, Safietou Sanke, Ousmane Faye, Amadou Alpha Sall.
EPI_ISL_486881	CV Raman Hospital	Department of Neurovirology, National Institute of Mental Health and Neuroscience (NIMHANS)	Chitra Pattabiraman, Vijayalakshmi Reddy, Harsha PK, Risha Rasheed, Shafeeq S Hameed, Manjunatha Venkataswamy, Anita Desai, Ravi Vasanthapuram
EPI_ISL_487107	Nigeria Centre for Disease Control (NCDC)	African Centre of Excellence for Genomics of Infectious Diseases (ACEGID), Redeemer's University, Ede, Osun State, Nigeria	Oluniyi P.E., Ajogbasile F.V., Kayode A., Oguzie J., Olawoye I., Uwanibe J., Olumade T., Folarin O.A., Ihekweazu C., Happi C.T.
EPI_ISL_487377, EPI_ISL_487381	Hellenic Pasteur Institute, National Influenza Reference laboratory of Southern Greece & Unit of Bioinformatics and Applied Genomics	Hellenic Pasteur Institute, National Influenza Reference laboratory of Southern Greece & Unit of Bioinformatics and Applied Genomics	Vasiliki Pogka, Timokratis Karamitros, Athanasios Kossyvakis, Antonios Kalliaropoulos, Horefti Elina, Evangelidou Maria, Androniki Voulgari-Kokota, Aspasia Kontou, Andreas Mentis
EPI_ISL_487435	Queen Astrid Military Hospital	Institute of Tropical Medicine	Philippe Selhorst, Colin Anthony
EPI_ISL_489708, EPI_ISL_489709	The National Institute of Public Health	The National Institute of Public Health and State Veterinary Institute Prague	Nagy,A;Jirincova,H;Novakova,L;Trnka,D;Vecerova,J
EPI_ISL_489995, EPI_ISL_490106	CSIR-CDRI/SGPGI, Lucknow	CSIR-CDRI/SGPGI, Lucknow	Saumya Sarkar, Dharam Veer Singh, Rahul Vishvkarma, Ujjala Ghoshal, Uday Ghoshal, Ravishankar Ramachandran, Tapas Kumar Kundu, Rajender Singh
EPI_ISL_490112	The National Institute of Public Health	The National Institute of Public Health and State Veterinary Institute Prague	Nagy,A;Jirincova,H;Novakova,L;Trnka,D;Vecerova,J
EPI_ISL_491092	The National Institute of Public Health	State Veterinary Institute Prague	Nagy,A;Jirincova,H;Novakova,L;Trnka,D;Vecerova,J
EPI_ISL_491093, EPI_ISL_491094, EPI_ISL_491095	The National Institute of Public Health	The National Institute of Public Health and State Veterinary Institute Prague	Nagy,A; Jirincova,H; Novakova,L; Trnka,D; Vecerova,J
EPI_ISL_491096	CSIR-CDRI/SGPGI, Lucknow	CSIR-CDRI/SGPGI, Lucknow	Saumya Sarkar, Dharam Veer Singh, Rahul Vishvkarma, Ujjala Ghoshal, Uday Ghoshal, Ravishankar Ramachandran, Tapas Kumar Kundu, Rajender Singh
EPI_ISL_491115	Cicin-Sain Lab	Cicin-Sain Lab	M. Zeeshan Chaudhry, Kathrin Eschke, Yeonsu Kim, Luka Cicin-Sain
EPI_ISL_491117, EPI_ISL_491118	The National Institute of Public Health	The National Institute of Public Health and State Veterinary Institute Prague	Nagy,A;Jirincova,H;Novakova,L;Trnka,D;Vecerova,J
EPI_ISL_491436	Laboratorio de Referencia Nacional de Virus Respiratorio. Instituto Nacional de Salud Perú	Laboratorio de Referencia Nacional de Biotecnología y Biología Molecular. Instituto Nacional de Salud Perú	Carlos Padilla Rojas, Karolyn Vega Chozo, Priscila Lope Pari, Omar Caceres Rey, Marco Galarza Perez, Maribel Huaringa Nuñez, Johanna Balbuena Torres, Henri Bailon Calderon, Nancy Rojas Serrano
EPI_ISL_491437	Area de Salud Escazu (Coopesana)	Inciensa, Instituto Costarricense de Investigación y Enseñanza en Nutrición y Salud	Francisco Duarte, Hebleen Brenes, Claudio Soto-Garita, Estela Cordero, Adriana Godinez & Melany Calderon
EPI_ISL_491438	Hospital San Rafael de Alajuela	Inciensa, Instituto Costarricense de Investigación y Enseñanza en Nutrición y Salud	Francisco Duarte, Hebleen Brenes, Claudio Soto-Garita, Estela Cordero, Adriana Godinez & Melany Calderon

EPI_ISL_491439	Hospital Calderon Guardia	Inciensa, Instituto Costarricense de Investigación y Enseñanza en Nutrición y Salud	Francisco Duarte, Hebleen Brenes, Claudio Soto-Garita, Estela Cordero, Adriana Godinez & Melany Calderon
EPI_ISL_491440	Hospital San Rafael de Alajuela	Inciensa, Instituto Costarricense de Investigación y Enseñanza en Nutrición y Salud	Francisco Duarte, Hebleen Brenes, Claudio Soto-Garita, Estela Cordero, Adriana Godinez & Melany Calderon
EPI_ISL_491441, EPI_ISL_491442	Hospital Clinica Biblica	Inciensa, Instituto Costarricense de Investigación y Enseñanza en Nutrición y Salud	Francisco Duarte, Hebleen Brenes, Claudio Soto-Garita, Estela Cordero, Adriana Godinez & Melany Calderon
EPI_ISL_491443	Hospital Fernando Escalante Pradilla	Inciensa, Instituto Costarricense de Investigación y Enseñanza en Nutrición y Salud	Francisco Duarte, Hebleen Brenes, Claudio Soto-Garita, Estela Cordero, Adriana Godinez & Melany Calderon
EPI_ISL_491444	Area de Salud Escazu (Coopesana)	Inciensa, Instituto Costarricense de Investigación y Enseñanza en Nutrición y Salud	Francisco Duarte, Hebleen Brenes, Claudio Soto-Garita, Estela Cordero, Adriana Godinez & Melany Calderon
EPI_ISL_491445	Area de Salud Mata Redonda	Inciensa, Instituto Costarricense de Investigación y Enseñanza en Nutrición y Salud	Francisco Duarte, Hebleen Brenes, Claudio Soto-Garita, Estela Cordero, Adriana Godinez & Melany Calderon
EPI_ISL_491446	Area de Salud Alajuela Central	Inciensa, Instituto Costarricense de Investigación y Enseñanza en Nutrición y Salud	Francisco Duarte, Hebleen Brenes, Claudio Soto-Garita, Estela Cordero, Adriana Godinez & Melany Calderon
EPI_ISL_491447	Hospital Fernando Escalante Pradilla	Inciensa, Instituto Costarricense de Investigación y Enseñanza en Nutrición y Salud	Francisco Duarte, Hebleen Brenes, Claudio Soto-Garita, Estela Cordero, Adriana Godinez & Melany Calderon
EPI_ISL_491448	Hospital San Rafael de Alajuela	Inciensa, Instituto Costarricense de Investigación y Enseñanza en Nutrición y Salud	Francisco Duarte, Hebleen Brenes, Claudio Soto-Garita, Estela Cordero, Adriana Godinez & Melany Calderon
EPI_ISL_491449	Area de Salud Alajuela Sur	Inciensa, Instituto Costarricense de Investigación y Enseñanza en Nutrición y Salud	Francisco Duarte, Hebleen Brenes, Claudio Soto-Garita, Estela Cordero, Adriana Godinez & Melany Calderon
EPI_ISL_491450	Hospital San Juan de Dios	Inciensa, Instituto Costarricense de Investigación y Enseñanza en Nutrición y Salud	Francisco Duarte, Hebleen Brenes, Claudio Soto-Garita, Estela Cordero, Adriana Godinez & Melany Calderon
EPI_ISL_491451	Hospital México	Inciensa, Instituto Costarricense de Investigación y Enseñanza en Nutrición y Salud	Francisco Duarte, Hebleen Brenes, Claudio Soto-Garita, Estela Cordero, Adriana Godinez & Melany Calderon
EPI_ISL_491452	Hospital San Rafael de Alajuela	Inciensa, Instituto Costarricense de Investigación y Enseñanza en Nutrición y Salud	Francisco Duarte, Hebleen Brenes, Claudio Soto-Garita, Estela Cordero, Adriana Godinez & Melany Calderon
EPI_ISL_491453	Hospital México	Inciensa, Instituto Costarricense de Investigación y Enseñanza en Nutrición y Salud	Francisco Duarte, Hebleen Brenes, Claudio Soto-Garita, Estela Cordero, Adriana Godinez & Melany Calderon
EPI_ISL_491454	Hospital San Juan de Dios	Inciensa, Instituto Costarricense de Investigación y Enseñanza en Nutrición y Salud	Francisco Duarte, Hebleen Brenes, Claudio Soto-Garita, Estela Cordero, Adriana Godinez & Melany Calderon
EPI_ISL_491455	Hospital Clinica Biblica	Inciensa, Instituto Costarricense de Investigación y Enseñanza en Nutrición y Salud	Francisco Duarte, Hebleen Brenes, Claudio Soto-Garita, Estela Cordero, Adriana Godinez & Melany Calderon
EPI_ISL_491456	Hospital San Juan de Dios	Inciensa, Instituto Costarricense de Investigación y Enseñanza en Nutrición y Salud	Francisco Duarte, Hebleen Brenes, Claudio Soto-Garita, Estela Cordero, Adriana Godinez & Melany Calderon
EPI_ISL_491457	Area de Salud Los Santos	Inciensa, Instituto Costarricense de Investigación y Enseñanza en Nutrición y Salud	Francisco Duarte, Hebleen Brenes, Claudio Soto-Garita, Estela Cordero, Adriana Godinez & Melany Calderon
EPI_ISL_491463	Laboratorio de Referencia Nacional de Virus Respiratorio. Instituto Nacional de Salud Perú	Laboratorio de Referencia Nacional de Biotecnología y Biología Molecular. Instituto Nacional de Salud Perú	Carlos Padilla Rojas, Karolyn Vega Chozo, Priscila Lope Pari, Omar Caceres Rey, Marco Galarza Perez, Maribel Huaringa Nuñez, Johanna Balbuena Torres, Henri Bailon Calderon, Nancy Rojas Serrano
EPI_ISL_491476	BSL3 Lab, Pendik Veterinary Control Enstitue	Genomic Laboratory (GLAB), Istanbul Technical University	Mustafa HASOKSUZ, Fahriye SARAC, Osman ERGANIS, Serdar UZAR, Hakan ENUL, Cumhur ADIAY, Ahmet SAIT, Orbay SAYI, Kadir YESILBAG, Oguz KARABEY
EPI_ISL_491968, EPI_ISL_491969, EPI_ISL_491970, EPI_ISL_491971, EPI_ISL_491972, EPI_ISL_491973, EPI_ISL_491974	Oman-NIC	Department of Microbiology and Immunology-SQUH	Fahad Zadjali, Samira Al-Maruqi, Amina Al Jardani, Khulood Al-Mammary, Hanan Al-kindi, Fatma BaAlawi, Hamida AL Barwani, Zeyana AL-Dahmani, Intisar Al-Shukri, Aisha Al-Busaidi, Aisha Al-Amri, Ahlam Al-Amri, Mohammed Al-Tobi, Samiha Al Kharusi, Abdulla Balkhair
EPI_ISL_492033	Instituto de Biologia do Exército	Laboratório Metabolismo Macromolecular FirminoTorres de Castro, Instituto de Biofísica Carlos Chagas Filho, Universidade Federal do Rio de Janeiro	Bianca Catarina Azevedo Cabral, Aline Rosa Vianna de Souza, Caleb GM Santos, Marcos Dornelas-Ribeiro, Tatiana LS Nogueira, Nádia Vaez Gonçalves da Cruz, Elizabeth Valentin, Marcio da Costa Cipitelli, Virginia Sara Grancieri do Amaral, Rodrigo Soares de Moura Neto, Clarissa Damaso, Rosane Silva
EPI_ISL_492034, EPI_ISL_492038	Instituto de Biologia do Exército	Laboratório Metabolismo Macromolecular FirminoTorres de Castro, Instituto de Biofísica Carlos Chagas Filho, Universidade Federal do Rio de Janeiro	Bianca Catarina Azevedo Cabral, Aline Rosa Vianna de Souza, Nádia Vaez Gonçalves da Cruz, Caleb GM Santos, Marcos Dornelas-Ribeiro, Tatiana LS Nogueira, Elizabeth Valentin, Marcio da Costa Cipitelli, Virginia Sara Grancieri do Amaral, Rodrigo Soares de Moura Neto, Clarissa Damaso, Rosane Silva
EPI_ISL_492039	Instituto de Biologia do Exército	Laboratório Metabolismo Macromolecular FirminoTorres de Castro, Instituto de Biofísica Carlos Chagas Filho, Universidade Federal do Rio de Janeiro	Bianca Catarina Azevedo Cabral, Aline Rosa Vianna de Souza, Tatiana LS Nogueira, Nádia Vaez Gonçalves da Cruz, Caleb GM Santos, Marcos Dornelas-Ribeiro, Elizabeth Valentin, Marcio da Costa Cipitelli, Virginia Sara Grancieri do Amaral, Rodrigo Soares de Moura Neto, Clarissa Damaso, Rosane Silva
EPI_ISL_492041	Instituto de Biologia do Exército	Laboratório Metabolismo Macromolecular FirminoTorres de Castro, Instituto de Biofísica Carlos Chagas Filho, Universidade Federal do Rio de Janeiro	Bianca Catarina Azevedo Cabral, Aline Rosa Vianna de Souza, Caleb GM Santos, Marcos Dornelas-Ribeiro, Tatiana LS Nogueira, Nádia Vaez Gonçalves da Cruz, Elizabeth Valentin, Marcio da Costa Cipitelli, Virginia Sara Grancieri do Amaral, Rodrigo Soares de Moura Neto, Clarissa Damaso, Rosane Silva
EPI_ISL_492042	Instituto de Biologia do Exército	Laboratório Metabolismo Macromolecular FirminoTorres de Castro, Instituto de Biofísica Carlos Chagas Filho, Universidade Federal do Rio de Janeiro	Bianca Catarina Azevedo Cabral, Aline Rosa Vianna de Souza, Nádia Vaez Gonçalves da Cruz, Caleb GM Santos, Marcos Dornelas-Ribeiro, Tatiana LS Nogueira, Elizabeth Valentin, Marcio da Costa Cipitelli, Virginia Sara Grancieri do Amaral, Rodrigo Soares de Moura Neto, Clarissa Damaso, Rosane Silva
EPI_ISL_492184	INT Fondazione Pascale	INT Fondazione Pascale	Pascale
EPI_ISL_492978, EPI_ISL_492979	Department of Laboratory Medicine Tan Tock Seng Hospital	Department of Laboratory Medicine Tan Tock Seng Hospital	Chen YYC, Zair X, Li C, Tang WY, Maurer-Stroh S, Barkham TMS, Nagarajan N, Sessions OM
EPI_ISL_492982, EPI_ISL_492983, EPI_ISL_492984, EPI_ISL_492985, EPI_ISL_492986, EPI_ISL_492987	IRCCS Sacro Cuore Don Calabria Hospital, Department of Infectious, Tropical Diseases & Microbiology	University of Verona, Department of Biotechnology	Antonio Mori, Michela Deiana, Elena Pomari, Chiara Piubelli; Giulia Lopatriello, Luca Marcolungo, Cristina Beltrami, Chiara Degli Esposti, Emanuela Cosentino, Massimo Delledonne
EPI_ISL_493198, EPI_ISL_493199, EPI_ISL_493200, EPI_ISL_493202, EPI_ISL_493205, EPI_ISL_493206, EPI_ISL_493207	Virology Lab,Department of Pathology, National Cheng Kung University Hospital	Virology Lab,Department of Pathology, National Cheng Kung University Hospital	Huey-Pin Tsai, et al
EPI_ISL_493332	Istituto Zooprofilattico Sperimentale del Mezzogiorno	INMI Lazzaro Spallanzani IRCCS	Cesare E.M. Gruber, Martina Rueca, Barbara Bartolini, Francesco Messina, Antonino Di Caro, Giovanna Fusco, Maurizio Viscardi, Giorgia Borriello, Maria R. Capobianchi
EPI_ISL_493333	Istituto Zooprofilattico Sperimentale del Mezzogiorno	INMI Lazzaro Spallanzani IRCCS	Barbara Bartolini, Martina Rueca, Cesare E.M. Gruber, Francesco Messina, Antonino Di Caro, Giovanna Fusco, Maurizio Viscardi, Giorgia Borriello, Maria R. Capobianchi

EPI_ISL_493334, EPI_ISL_493335, EPI_ISL_493336	Instituto de Diagnostico y Referencia Epidemiologicos (INDRE)	Instituto de Diagnostico y Referencia Epidemiologicos (INDRE)	Gisela Barrera-Badillo , Abril Rodriguez-Maldonado, Claudia Wong-Arambula , Natividad Cruz-Ortiz, Tatiana Nunez-Garcia, Dayanira Arellano-Suarez, Fabiola Garces-Ayala, Edgar Mendieta-Condado, Lucia Hernandez-Rivas, Irma Lopez-Martinez, Ernesto Ramirez-Gonzalez.
EPI_ISL_494757, EPI_ISL_494759, EPI_ISL_494761, EPI_ISL_494771	INT Fondazione Pascale	INT Fondazione Pascale	INT Fondazione Pascale
EPI_ISL_495414, EPI_ISL_495417, EPI_ISL_495423, EPI_ISL_495426, EPI_ISL_495428, EPI_ISL_495432, EPI_ISL_495434, EPI_ISL_495435, EPI_ISL_495437, EPI_ISL_495439, EPI_ISL_495442, EPI_ISL_495443, EPI_ISL_495448, EPI_ISL_495455, EPI_ISL_495457			
see above	Kafkas University, Faculty of Medicine, Department of Medical Microbiology	Kafkas University, Faculty of Medicine, Department of Medical Microbiology	Murat Karamese, Didem Ozgur, E. Ediz Tutuncu
EPI_ISL_495459	Centers for Disease Control and Prevention of Lishui	Department of InspectionCenters for Disease Control and Prevention of Lishui	Wang Xiaoguang,Ji Qiaoying,Ji Jiansong,Ye Bifeng,Ye Ling
EPI_ISL_496534, EPI_ISL_496548, EPI_ISL_496549, EPI_ISL_496551	B.J. Govt. Medical College	National Centre For Cell Science	Dhiraj Paul, Kunal Jani, Radha Chauhan, Janesh Kumar, Vasudevan Seshadri, Girdhari Lal, Rajesh Karyakarte, Suvarna Joshi, Murlidhar Tambe, Sourav Sen, Santosh Karade, Kavita Bala Anand, Shelinder Pal Singh Shergill, Rajiv Mohan Gupta, Manoj Kumar Bhat, Arvind Sahu, Maharashtra COVID-19 Study Group, DBT's PAN-INDIA 1000 SARS-CoV2 RNA genome sequencing consortium, Yogesh S Shouche
EPI_ISL_496555, EPI_ISL_496556, EPI_ISL_496557, EPI_ISL_496587	National Centre For Cell Science	National Centre For Cell Science	Dhiraj Paul, Kunal Jani, Radha Chauhan, Janesh Kumar, Vasudevan Seshadri, Girdhari Lal, Rajesh Karyakarte, Suvarna Joshi, Murlidhar Tambe, Sourav Sen, Santosh Karade, Kavita Bala Anand, Shelinder Pal Singh Shergill, Rajiv Mohan Gupta, Manoj Kumar Bhat, Arvind Sahu, Maharashtra COVID-19 Study Group, DBT's PAN-INDIA 1000 SARS-CoV2 RNA genome sequencing consortium, Yogesh S Shouche
EPI_ISL_497736, EPI_ISL_497738, EPI_ISL_497744, EPI_ISL_497745	Instituto Nacional de Salud, Bogotá, Colombia	Instituto Nacional de Salud, Bogotá, Colombia	Katherine Laiton-Donato, Diego A. Álvarez-Díaz, Carlos Franco-Muñoz, Jonathan Reales, Diego Andrés Prada, Jose A. Usme-Ciro, Nicolas D. Franco-Sierra, Zulma M. Cucunubá, Christian Julian VillabonaArenas, Liz Villabona-Arenas, Sussy Echeverria, Astrid C. Flórez, Carolina Ferro, Diana Marcela Walteros-Acero, Franklin Prieto, Carlos Andrés Durán, Martha Lucia Ospina Martinez, Marcela Mercado-Reyes
EPI_ISL_497758, EPI_ISL_497760	CSIR-CDRI/SGPGI, Lucknow	CSIr-CDRI/SGPGI, Lucknow	Saumya Sarkar, Dharam Veer Singh, Rahul Vishvkarma, Ujjala Ghoshal, Uday Ghoshal, Ravishankar Ramachandran, Tapas Kumar Kundu, Rajender Singh
EPI_ISL_497763, EPI_ISL_497765	CSIR-CDRI/SGPGI, Lucknow	CSIR-CDRI/SGPGI, Lucknow	Saumya Sarkar, Dharam Veer Singh, Rahul Vishvkarma, Ujjala Ghoshal, Uday Ghoshal, Ravishankar Ramachandran, Tapas Kumar Kundu, Rajender Singh
EPI_ISL_497766	CSIR-CDRI/SGPGI, Lucknow	CSIR-CDRi/SGPGI, Lucknow	Saumya Sarkar, Dharam Veer Singh, Rahul Vishvkarma, Ujjala Ghoshal, Uday Ghoshal, Ravishankar Ramachandran, Tapas Kumar Kundu, Rajender Singh
EPI_ISL_498152, EPI_ISL_498153, EPI_ISL_498154, EPI_ISL_498155, EPI_ISL_498156, EPI_ISL_498157, EPI_ISL_498158, EPI_ISL_498159, EPI_ISL_498160, EPI_ISL_498161, EPI_ISL_498162, EPI_ISL_498163, EPI_ISL_498164, EPI_ISL_498165, EPI_ISL_498166, EPI_ISL_498167, EPI_ISL_498168, EPI_ISL_498169, EPI_ISL_498170			
see above	Instituto Nacional de Salud, Bogotá, Colombia	Instituto Nacional de Salud, Bogotá, Colombia	Katherine Laiton-Donato, Diego A. Álvarez-Díaz, Carlos Franco-Muñoz, Jonathan Reales, Diego Andrés Prada, Jose A. Usme-Ciro, Nicolas D. Franco-Sierra, Zulma M. Cucunubá, Christian Julian VillabonaArenas, Liz Villabona-Arenas, Sussy Echeverria, Astrid C. Flórez, Carolina Ferro, Diana Marcela Walteros-Acero, Franklin Prieto, Carlos Andrés Durán, Martha Lucia Ospina Martinez, Marcela Mercado-Reyes
EPI_ISL_498171, EPI_ISL_498172, EPI_ISL_498173, EPI_ISL_498174, EPI_ISL_498175, EPI_ISL_498176, EPI_ISL_498177, EPI_ISL_498178, EPI_ISL_498179, EPI_ISL_498180, EPI_ISL_498181, EPI_ISL_498182, EPI_ISL_498183, EPI_ISL_498184, EPI_ISL_498185, EPI_ISL_498186, EPI_ISL_498187, EPI_ISL_498188, EPI_ISL_498189, EPI_ISL_498190, EPI_ISL_498191, EPI_ISL_498192			
see above	OUCRU	OUCRU	Nguyen Van Vinh Chau, Nguyen Thi Thu Hong, Nguyen Thi Han Ny, Le Nguyen Truc Nhu, Nghiem My Ngoc, Vo Thanh Lam, Nguyen Thanh Dung, Lam Minh Yen, Ngo Ngoc Quang Minh, Le Manh Hung, Nguyen Tri Dung, Dinh Nguyen Huy Man, Lam Anh Nguyet, Tran Chanh Xuan, Tran Tinh Hien, Nguyen Thanh Phong, Tran Nguyen Hoang Tu, Tran Tan Thanh, Nguyen Thanh Truong, Nguyen Tan Binh, Tang Chi Thuong, Guy Thwaites, and Le Van Tan, for OUCRU COVID-19 research group*
EPI_ISL_498229, EPI_ISL_498230, EPI_ISL_498236	Institut Pasteur de Dakar	Institut Pasteur de Dakar	Ndongo Dia, Moussa Moise Diagne, Mamadou Diop, Marie Henriette Dior Ndione, Mamadou Malado Jallow, Safietou Sankhe Mbengue, Ousmane Faye, Amadou Alpha Sall.
EPI_ISL_498551, EPI_ISL_498552, EPI_ISL_498554	Lebanese American University	Lebanese American University	Abi Habib,W., Abdallah,J., El Shesheny,R., Mokhbat,J., Webby,R.J., Goldstein,J. and Kayali,G.
EPI_ISL_499083	Instituto de Virologia "Dr. J. M. Vanella", Facultad de Ciencias Medicas, Universidad Nacional de Cordoba. Laboratorio Central de la Provincia de Cordoba, Argentina. Ministerio de Salud de la provincia de Cordoba, Argentina.	Laboratorio de Virología, Hospital de Niños Ricardo Gutiérrez, CABA, Argentina.	Sandra Gallego, Brenda Konigheim, Sebastian Blanco, Lorena Spinsanti, Javier Aguilar, Adrian Diaz, Gonzalo Castro, Gabriela Barbas, Mercedes Nabaes, Stephanie Goya, Monica Natale, Silvina Lusso, Mariana Viegas.
EPI_ISL_500541	Singapore General Hospital	Department of Microbiology	Nurdyana Abdul Rahman, Kun Lee Lim, Chenhao Li, Kian Sing Chan, Lynette Oon, Kern Rei Chng, Niranjan Nagarajan, Karrie Ko
EPI_ISL_500716	BSL3 Lab Pendik Veterinary Control Institute	Department of Medicinal Genetics, Bursa Uluda University, Faculty of medicine By Sehime Gülsün Temel, Adem Alemdar, Kadir Yeilba	Mustafa HASOKSUZ, Fahriye SARAC, Osman ERGANIS, Serdar UZAR, Hakan ENUL, Cumhur ADIAY, Ahmet SAIT, Orbay SAYI, Kadir YESILBAG, Oguz KARABEY
EPI_ISL_501167, EPI_ISL_501168, EPI_ISL_501169, EPI_ISL_501170, EPI_ISL_501171, EPI_ISL_501172, EPI_ISL_501173, EPI_ISL_501174	Baylor College of Medicine	Baylor College of Medicine: HGSC	Vasanthi Avadhanula, Erin Nicholson, David Henke, Pedro Piedra, Harsha Doddapaneni, Donna Muzny, Qingchang Meng, Hsu Chao, Zeineen Momin, Hua Shen, George Weissenberger, Kavya Kottapalli, Yimiti Meiheerguli, Sejal Salvi, Ginger Metcalf, Vipin Menon, Sara J.J. Cregeen, Matthew C. Ross, Tulin Ayvaz, Richard Sucgang, Kristi L. Hoffman, Matthew Wong, Joseph F. Petrosino
EPI_ISL_501176, EPI_ISL_501177, EPI_ISL_501178, EPI_ISL_501179, EPI_ISL_501180, EPI_ISL_501181, EPI_ISL_501182, EPI_ISL_501183, EPI_ISL_501184, EPI_ISL_501185, EPI_ISL_501186, EPI_ISL_501187, EPI_ISL_501188, EPI_ISL_501189, EPI_ISL_501190, EPI_ISL_501191, EPI_ISL_501192, EPI_ISL_501193, EPI_ISL_501194, EPI_ISL_501195, EPI_ISL_501196, EPI_ISL_501197, EPI_ISL_501198, EPI_ISL_501199, EPI_ISL_501200, EPI_ISL_501201, EPI_ISL_501202, EPI_ISL_501203, EPI_ISL_501204, EPI_ISL_501205, EPI_ISL_501206, EPI_ISL_501207, EPI_ISL_501208, EPI_ISL_501209, EPI_ISL_501210, EPI_ISL_501211, EPI_ISL_501212, EPI_ISL_501213, EPI_ISL_501214, EPI_ISL_501215, EPI_ISL_501216, EPI_ISL_501217, EPI_ISL_501218, EPI_ISL_501219, EPI_ISL_501220, EPI_ISL_501221, EPI_ISL_501222, EPI_ISL_501223, EPI_ISL_501224, EPI_ISL_501225, EPI_ISL_501226, EPI_ISL_501227, EPI_ISL_501228			
see above	Department of Medical Microbiology, University Malaya Medical Centre	Department of Medical Microbiology, Faculty of Medicine, University of Malaya	Yoong Min CHONG, Jennifer Chong, I-Ching SAM, Yoke Fun CHAN, University Malaya Medical Centre COVID Team
EPI_ISL_501230	Hellenic Pasteur Institute, Public Health Laboratories	Hellenic Pasteur Institute, National Influenza Reference laboratory of Southern Greece & Unit of Bioinformatics and Applied Genomics	Vasiliki Pogka, Timokratis Karamitros, Athanasios Kossyvakis, Antonios Kalliaropoulos, Horefti Elina, Evangelidou Maria, Androniki Voulgari-Kokota, Aspasia Kontou, Andreas Mentis
EPI_ISL_501233, EPI_ISL_501236, EPI_ISL_501248, EPI_ISL_501249, EPI_ISL_501250, EPI_ISL_501251	Hellenic Pasteur Institute, National Influenza Reference laboratory of Southern Greece & Unit of Bioinformatics and Applied Genomics	Hellenic Pasteur Institute, National Influenza Reference laboratory of Southern Greece & Unit of Bioinformatics and Applied Genomics	Vasiliki Pogka, Timokratis Karamitros, Athanasios Kossyvakis, Antonios Kalliaropoulos, Horefti Elina, Evangelidou Maria, Androniki Voulgari-Kokota, Aspasia Kontou, Andreas Mentis
EPI_ISL_505003	Biology Dpt	Microbiology and Infections Diseases	Emmanuelle Billon-Denis, Audrey Ferrier-Rembert, Annabelle Garnier, Laurence Cheutin, Clarisse Vigne, Emilie Tessier, Jessica Denis, Olivier Gorgé, Flora Nolent, Isabelle Drouet, Noémie Verguet, Olivier Ferraris, Jean-Nicolas Tournier
EPI_ISL_506996	Department of Medical Microbiology, University Malaya Medical Centre	Department of Medical Microbiology, Faculty of Medicine, University of Malaya	Yoong Min CHONG, Jennifer Chong, I-Ching SAM, Yoke Fun CHAN, University Malaya Medical Centre COVID Team
EPI_ISL_507002, EPI_ISL_507003, EPI_ISL_507004, EPI_ISL_507005, EPI_ISL_507006	Department of Laboratory Medicine, Tan Tock Seng Hospital	Department of Laboratory Medicine, Tan Tock Seng Hospital	Chen YYC, Zair X, Li C, Tang WY, Maurer-Stroh S, Barkham TMS, Nagarajan N, Sessions OM
EPI_ISL_507008	National Veterinary Institute	National Veterinary Institute	Siamak Zohari
EPI_ISL_507206, EPI_ISL_507207, EPI_ISL_507208, EPI_ISL_507209, EPI_ISL_507210, EPI_ISL_507211, EPI_ISL_507212, EPI_ISL_507213, EPI_ISL_507214, EPI_ISL_507215	Department of Experimental Modeling and Pathogenesis of Infectious Diseases	WHO National Influenza Centre Russian Federation	Andrey Komissarov, Artem Fadeev, Mariia Sergeeva, Anna Ivanova, Daria Danilenko
EPI_ISL_507225, EPI_ISL_507226,	WHO National Influenza Centre Russian Federation	WHO National Influenza Centre Russian Federation	Andrey Komissarov, Artem Fadeev, Mariia Sergeeva, Anna Ivanova, Daria Danilenko

EPI_ISL_507261, EPI_ISL_507262, EPI_ISL_507263, EPI_ISL_507264, EPI_ISL_507291, EPI_ISL_507292, EPI_ISL_507293, EPI_ISL_507294			
EPI_ISL_508862, EPI_ISL_508863	Virology Unit, Institut Pasteur de Madagascar	Virology Unit, Institut Pasteur de Madagascar	Christian Ranaivoson, Cara Brook, Norosoa Razanajatovo, Vida Ahyong, Tsiry Randriambolamanantsoa, Michelle Tan, Vololoniaina Raharinosy, Helisoa Razafimanjato, Cristina M. Tato, Joseph L. DeRisi, Soa Fy Andriamandimby, Jean-Michel Heraud
EPI_ISL_509413, EPI_ISL_509416	Acibadem Labcell Cellular Therapy Laboratory	Acibadem Mehmet Ali Aydinlar University School of Medicine, Medical Genetics Department	Ozden Hatirnaz Ng, Sezer Akyoney, Ilayda Sahin, Gunseli Bayram Akcapinar, Ozkan Ozdemir, Derya Dilek Kancagi, Gozde Sir Karakus, Bulut Yurtsever, Cihan Tastan, Ercument Ovali, Ugur Ozbek
EPI_ISL_510689	Hospital Universitari Germans Trias i Pujol(HUGTiP)/Fundació Lluita contra la SIDA (FLSida)/IRTA-CReSA	IrsiCaixa AIDS Research Lab	Pilar Armengol, Marc Noguera-Julian, Jordi Rodón, Julia Vergara, Lidia Ruiz, Nuria Izquierdo, Jorge Carrillo, Roger Paredes, Albert Bensaid, Julia Blanco, Joaquim Segalés, Bonaventura Clotet
EPI_ISL_511891, EPI_ISL_511892, EPI_ISL_511893, EPI_ISL_511894, EPI_ISL_511895, EPI_ISL_511896, EPI_ISL_511897, EPI_ISL_511898	National Hospital of Tropical Diseases	Oxford University Clinical Research Unit, Hanoi, Vietnam	Nguyen Thi Tam, Van Dinh Trang, Nguyen Thi Hong Thuong, Vu Thi Ngoc Bich, Nguyen Thu Trang, Nguyen Thi Ngoc Diep, Le Nguyen Minh Hoa, Pham Ngoc Thach, H. Rogier van Doorn, on behalf of the OUCRU COVID-19 research group
EPI_ISL_512653	Area De Salud Desamparados 1 - Clinica Dr. Marcial Fallas [Grifo Alto/Desampara	Inciensa, Instituto Costarricense de Investigación y Enseñanza en Nutrición y Salud	Francisco Duarte, Hebleen Porras, Claudio Soto-Garita, Estela Cordero, Adriana Godinez & Melany Calderon
EPI_ISL_512844	Department of Medical Research	DMR_Myanmar	Myat Htut Nyunt, Hnin Ohnmar Soe, Kay Thi Aye, Wah Wah Aung,Yi Yi Kyaw, Aung Kyaw Kyaw, Theingi Win Myat, Phyu Win Ei, Aung Zaw Latt, Nan Aye Thida Oo, Lai Lai San, Su Mon Win, Ni Ni Zaw, Htin Lin, Hlaing Myat Thu, Zaw Than Htun
EPI_ISL_512908, EPI_ISL_512909, EPI_ISL_512911, EPI_ISL_512912, EPI_ISL_512913, EPI_ISL_512914, EPI_ISL_512915, EPI_ISL_512916, EPI_ISL_512917	Pathogen Genomics Lab King Abdullah University of Science and Technology(KAUST)	Pathogen Genomics Lab King Abdullah University of Science and Technology(KAUST)	Sharif Hala, Fadwa Alofi, Sara Mfarrej, Amit Kumar Subudhi, Rahul P Salunke, Fathia Ben Rached, Amanda Ooi, Luke Esau, Afrah Alsomali, Asim Khogeer, Jumana Taha, Abdulaziz Alahmadi, Kahled Algithami, Raeece Naeem, Anwar Hashem, Naif Almontashiri, Arnab Pain
EPI_ISL_512918, EPI_ISL_512919, EPI_ISL_512928, EPI_ISL_512929, EPI_ISL_512930, EPI_ISL_512931, EPI_ISL_512932, EPI_ISL_512933, EPI_ISL_512934, EPI_ISL_512935, EPI_ISL_512936, EPI_ISL_512937, EPI_ISL_512938, EPI_ISL_512939, EPI_ISL_512941, EPI_ISL_512942, EPI_ISL_512943, EPI_ISL_512944, EPI_ISL_512945			
see above	Pathogen Genomics Lab King Abdullah University of Science and Technology(KAUST)	Pathogen Genomics Lab King Abdullah University of Science and Technology(KAUST)	Fadwa Alofi, Sharif Hala, Rahul P Salunke, Sara Mfarrej, Amit Kumar Subudhi, Fathia Ben Rached, Amanda, Luke, Afrah Alsomali, Asim Khogeer, Jumana Taha, Abdulaziz Alahmadi, Kahled Algithami, Raeece Naeem, Anwar Hashem, Naif Almontashiri, Arnab Pain
EPI_ISL_512946, EPI_ISL_512947, EPI_ISL_512948, EPI_ISL_512949, EPI_ISL_512950, EPI_ISL_512951, EPI_ISL_512952, EPI_ISL_512953, EPI_ISL_512954, EPI_ISL_512955, EPI_ISL_512956, EPI_ISL_512957, EPI_ISL_512959, EPI_ISL_512963, EPI_ISL_512964, EPI_ISL_512966, EPI_ISL_512967, EPI_ISL_512969, EPI_ISL_512970, EPI_ISL_512971, EPI_ISL_512973, EPI_ISL_512974, EPI_ISL_512975, EPI_ISL_512976, EPI_ISL_512977, EPI_ISL_512979, EPI_ISL_512980, EPI_ISL_512981, EPI_ISL_512982, EPI_ISL_512983, EPI_ISL_512984, EPI_ISL_512986, EPI_ISL_512987, EPI_ISL_512988			
see above	Pathogen Genomics Lab King Abdullah University of Science and Technology(KAUST)	Pathogen Genomics Lab King Abdullah University of Science and Technology(KAUST)	Sara Mfarrej, Raeece Naeem, Rahul P Salunke, Sharif Hala, Fadwa Alofi, Amit Kumar Subudhi, Fathia Ben Rached, Afrah Alsomali, Jumana Taha, Abdulaziz Alahmadi, Asim Khogeer, Nashwa Al-khotani, Anwar Hashem, Naif Almontashiri, Arnab Pain
EPI_ISL_512989, EPI_ISL_512990, EPI_ISL_512991, EPI_ISL_512992, EPI_ISL_512993, EPI_ISL_512994, EPI_ISL_512995, EPI_ISL_512996, EPI_ISL_512997, EPI_ISL_512998, EPI_ISL_512999, EPI_ISL_513000, EPI_ISL_513001			
see above	Pathogen Genomics Lab King Abdullah University of Science and Technology(KAUST)	Pathogen Genomics Lab King Abdullah University of Science and Technology(KAUST)	Amit Kumar Subudhi, Rahul P Salunke, Sara Mfarrej, Sharif Hala, Fadwa Alofi, Fathia Ben Rached, Afrah Alsomali, Asim Khogeer, Nashwa Al-khotani, Raeece Naeem, Anwar Hashem, Naif Almontashiri, Arnab Pain
EPI_ISL_513041, EPI_ISL_513042, EPI_ISL_513043, EPI_ISL_513044, EPI_ISL_513045, EPI_ISL_513046, EPI_ISL_513047, EPI_ISL_513048, EPI_ISL_513049, EPI_ISL_513050, EPI_ISL_513051, EPI_ISL_513052, EPI_ISL_513053, EPI_ISL_513054, EPI_ISL_513055, EPI_ISL_513056, EPI_ISL_513057, EPI_ISL_513058, EPI_ISL_513059, EPI_ISL_513060, EPI_ISL_513061, EPI_ISL_513062			
see above	Pathogen Genomics Lab King Abdullah University of Science and Technology(KAUST)	Pathogen Genomics Lab King Abdullah University of Science and Technology(KAUST)	Rahul P Salunke, Sharif Hala, Raeece Naeem, Sara Mfarrej, Amit Kumar Subudhi, Amanda Ooi, Luke Esau, Fadwa Alofi, Fathia Ben Rached, Afrah Alsomali, Asim Khogeer, Ahmad Bakur Mahmoud, Anwar Hashem, Naif Almontashiri, Arnab Pain
EPI_ISL_513122, EPI_ISL_513123, EPI_ISL_513124, EPI_ISL_513125, EPI_ISL_513126, EPI_ISL_513127, EPI_ISL_513128, EPI_ISL_513129, EPI_ISL_513131, EPI_ISL_513132, EPI_ISL_513133, EPI_ISL_513134, EPI_ISL_513135, EPI_ISL_513137, EPI_ISL_513138, EPI_ISL_513139, EPI_ISL_513141, EPI_ISL_513142, EPI_ISL_513143, EPI_ISL_513144, EPI_ISL_513145, EPI_ISL_513146			
see above	Pathogen Genomics Lab King Abdullah University of Science and Technology(KAUST)	Pathogen Genomics Lab King Abdullah University of Science and Technology(KAUST)	Sharif Hala, Fadwa Alofi, Sara Mfarrej, Amit Kumar Subudhi, Rahul P Salunke, Fathia Ben Rached, Amanda Ooi, Luke Esau, Afrah Alsomali, Asim Khogeer, Jumana Taha, Abdulaziz Alahmadi, Kahled Algithami, Raeece Naeem, Anwar Hashem, Naif Almontashiri, Arnab Pain
EPI_ISL_513147, EPI_ISL_513148, EPI_ISL_513149, EPI_ISL_513150, EPI_ISL_513151, EPI_ISL_513152, EPI_ISL_513153, EPI_ISL_513154, EPI_ISL_513156, EPI_ISL_513157, EPI_ISL_513158, EPI_ISL_513159, EPI_ISL_513160, EPI_ISL_513161, EPI_ISL_513162, EPI_ISL_513163, EPI_ISL_513164, EPI_ISL_513165, EPI_ISL_513166, EPI_ISL_513167			
see above	Pathogen Genomics Lab King Abdullah University of Science and Technology(KAUST)	Pathogen Genomics Lab King Abdullah University of Science and Technology(KAUST)	Fadwa Alofi, Sharif Hala, Rahul P Salunke, Sara Mfarrej, Amit Kumar Subudhi, Fathia Ben Rached, Amanda, Luke, Afrah Alsomali, Asim Khogeer, Jumana Taha, Abdulaziz Alahmadi, Kahled Algithami, Raeece Naeem, Anwar Hashem, Naif Almontashiri, Arnab Pain
EPI_ISL_513168, EPI_ISL_513169, EPI_ISL_513170, EPI_ISL_513171, EPI_ISL_513172, EPI_ISL_513173, EPI_ISL_513174	Pathogen Genomics Lab King Abdullah University of Science and Technology(KAUST)	Pathogen Genomics Lab King Abdullah University of Science and Technology(KAUST)	Sara Mfarrej, Raeece Naeem, Rahul P Salunke, Sharif Hala, Fadwa Alofi, Amit Kumar Subudhi, Fathia Ben Rached, Afrah Alsomali, Jumana Taha, Abdulaziz Alahmadi, Asim Khogeer, Nashwa Al-khotani, Anwar Hashem, Naif Almontashiri, Arnab Pain
EPI_ISL_513175, EPI_ISL_513176, EPI_ISL_513177, EPI_ISL_513178, EPI_ISL_513179, EPI_ISL_513180, EPI_ISL_513181, EPI_ISL_513182, EPI_ISL_513183, EPI_ISL_513184, EPI_ISL_513185, EPI_ISL_513186, EPI_ISL_513187, EPI_ISL_513188, EPI_ISL_513189			
see above	Pathogen Genomics Lab King Abdullah University of Science and Technology(KAUST)	Pathogen Genomics Lab King Abdullah University of Science and Technology(KAUST)	Amit Kumar Subudhi, Rahul P Salunke, Sara Mfarrej, Sharif Hala, Fadwa Alofi, Fathia Ben Rached, Afrah Alsomali, Asim Khogeer, Nashwa Al-khotani, Raeece Naeem, Anwar Hashem, Naif Almontashiri, Arnab Pain
EPI_ISL_513195	Pathogen Genomics Lab King Abdullah University of Science and Technology(KAUST)	Pathogen Genomics Lab King Abdullah University of Science and Technology(KAUST)	Afrah Alsomali, Fathia Ben Rached, Raeece Naeem, Sharif Hala,Rahul P Salunke, Amanda Ooi, Luke Esau, Sara Mfarrej, Amit Kumar Subudhi, Fadwa Alofi, Asim Khogeer, Kahled Algithami, Anwar Hashem, Naif Almontashiri, Arnab Pain
EPI_ISL_513198, EPI_ISL_513205, EPI_ISL_513206	Pathogen Genomics Lab King Abdullah University of Science and Technology(KAUST)	Pathogen Genomics Lab King Abdullah University of Science and Technology(KAUST)	Rahul P Salunke, Sharif Hala, Raeece Naeem, Sara Mfarrej, Amit Kumar Subudhi, Amanda Ooi, Luke Esau, Fadwa Alofi, Fathia Ben Rached, Afrah Alsomali, Asim Khogeer, Ahmad Bakur Mahmoud, Anwar Hashem, Naif Almontashiri, Arnab Pain
EPI_ISL_513208, EPI_ISL_513209, EPI_ISL_513211	Pathogen Genomics Lab King Abdullah University of Science and Technology(KAUST)	Pathogen Genomics Lab King Abdullah University of Science and Technology(KAUST)	Raeece Naeem, Rahul P Salunke, Sharif Hala, Sara Mfarrej, Amit Kumar Subudhi, Fadwa Alofi, Fathia Ben Rached, Afrah Alsomali, Asim Khogeer, Ahmad Bakur Mahmoud, Anwar Hashem, Naif Almontashiri, Arnab Pain
EPI_ISL_513214, EPI_ISL_513215, EPI_ISL_513216, EPI_ISL_513217, EPI_ISL_513218, EPI_ISL_513219, EPI_ISL_513221, EPI_ISL_513222, EPI_ISL_513224, EPI_ISL_513226	Pathogen Genomics Lab King Abdullah University of Science and Technology(KAUST)	Pathogen Genomics Lab King Abdullah University of Science and Technology(KAUST)	Sharif Hala, Fadwa Alofi, Sara Mfarrej, Amit Kumar Subudhi, Rahul P Salunke, Fathia Ben Rached, Amanda Ooi, Luke Esau, Afrah Alsomali, Asim Khogeer, Jumana Taha, Abdulaziz Alahmadi, Kahled Algithami, Raeece Naeem, Anwar Hashem, Naif Almontashiri, Arnab Pain
EPI_ISL_513228, EPI_ISL_513229, EPI_ISL_513230, EPI_ISL_513231, EPI_ISL_513233, EPI_ISL_513234, EPI_ISL_513235, EPI_ISL_513236, EPI_ISL_513238, EPI_ISL_513240, EPI_ISL_513241, EPI_ISL_513242, EPI_ISL_513243, EPI_ISL_513244, EPI_ISL_513245, EPI_ISL_513246, EPI_ISL_513247, EPI_ISL_513248			
see above	Pathogen Genomics Lab King Abdullah University of Science and Technology(KAUST)	Pathogen Genomics Lab King Abdullah University of Science and Technology(KAUST)	Amit Kumar Subudhi, Rahul P Salunke, Sara Mfarrej, Sharif Hala, Fadwa Alofi, Fathia Ben Rached, Afrah Alsomali, Asim Khogeer, Nashwa Al-khotani, Raeece Naeem, Anwar Hashem, Naif Almontashiri, Arnab Pain
EPI_ISL_513249, EPI_ISL_513250, EPI_ISL_513251, EPI_ISL_513252, EPI_ISL_513253, EPI_ISL_513254, EPI_ISL_513257, EPI_ISL_513258, EPI_ISL_513259, EPI_ISL_513260, EPI_ISL_513261, EPI_ISL_513262, EPI_ISL_513263, EPI_ISL_513264			
see above	Pathogen Genomics Lab King Abdullah University of Science and Technology(KAUST)	Pathogen Genomics Lab King Abdullah University of Science and Technology(KAUST)	Afrah Alsomali, Fathia Ben Rached, Raeece Naeem, Sharif Hala,Rahul P Salunke, Amanda Ooi, Luke Esau, Sara Mfarrej, Amit Kumar Subudhi, Fadwa Alofi, Asim Khogeer, Kahled Algithami, Anwar Hashem, Naif Almontashiri, Arnab Pain
EPI_ISL_513314, EPI_ISL_513511,	The National Institute of Public Health	The National Institute of Public Health and State Veterinary	Nagy,A;Jirincova,H;Novakova,L;Trnka,D;Vecerova,J

EPI_ISL_513512		Institute Prague	
EPI_ISL_513513, EPI_ISL_513514, EPI_ISL_513515, EPI_ISL_513516, EPI_ISL_513518, EPI_ISL_513519, EPI_ISL_513520, EPI_ISL_513521, EPI_ISL_513522, EPI_ISL_513524, EPI_ISL_513525, EPI_ISL_513526, EPI_ISL_513527, EPI_ISL_513529, EPI_ISL_513530, EPI_ISL_513531, EPI_ISL_513532, EPI_ISL_513533, EPI_ISL_513534, EPI_ISL_513535, EPI_ISL_513536, EPI_ISL_513537, EPI_ISL_513538, EPI_ISL_513539, EPI_ISL_513540, EPI_ISL_513541, EPI_ISL_513542, EPI_ISL_513543, EPI_ISL_513544, EPI_ISL_513545, EPI_ISL_513546, EPI_ISL_513547, EPI_ISL_513548, EPI_ISL_513549, EPI_ISL_513550, EPI_ISL_513551, EPI_ISL_513553, EPI_ISL_513556, EPI_ISL_513557, EPI_ISL_513558, EPI_ISL_513559, EPI_ISL_513560, EPI_ISL_513561, EPI_ISL_513562, EPI_ISL_513563, EPI_ISL_513564, EPI_ISL_513567, EPI_ISL_513568, EPI_ISL_513571, EPI_ISL_513572, EPI_ISL_513573, EPI_ISL_513574, EPI_ISL_513575, EPI_ISL_513576, EPI_ISL_513577, EPI_ISL_513578, EPI_ISL_513579, EPI_ISL_513580, EPI_ISL_513581, EPI_ISL_513582, EPI_ISL_513583			
see above	Programa de Oncovirologia, Instituto Nacional de Câncer	Programa de Oncovirologia, Instituto Nacional de Câncer	Juliana D. Siqueira, Livia R. Goes, Brunna M. Alves, Claudia Cicala,James Arthos, João P.B. Viola, Andreia C. de Melo, Marcelo A. Soares
EPI_ISL_514126	National Scientific Center for Especially Dangerous Infections (NSCEDI)	Kazakh National Agrarian University (KazNAU) TreeGene LLP Genetic Laboratory	Tabynov Kaissar, Beloussov Vyacheslav, Strochkov Vitaliy, Sandybayev Nurlan, Tabynov Kairat, Turebekov Nurkeldy, Granica Joanna, Solomadin Maksim, Yerubayev Toktassyn, Yespolov Tlektes
EPI_ISL_514131	Rondônia Central Public Health Laboratory (LACEN/RO), vinculated to State Health Secretariat of Rondônia (SESAU/RO)	Molecular Virology Laboratory of Oswaldo Cruz Foundation of Rondônia	Luan Felipo Botelho-Souza, Felipe Souza Nogueira-Lima, Tárcio Peixoto Roca, Alcione de Oliveira dos Santos, Felipe Gomes Naveca, Adriana Cristina Salvador Maia, Cicileia Correia da Silva, Aline Linhares Ferreira de Melo Mendonça, Celina Aparecida Bertoni Lugtenburg, Camila Flávia Gomes Azzi, Juliana Loca Furtado, Suelen Cavalcante, Rita de Cássia Pontello Rampazzo, Caio Henrique Nemeth Santos, Alice Paula Di Sabatino Guimarães, Jansen Fernandes de Medeiros, Fernando Rodrigues Máximo, Juan Miguel Vilallobos-Salcedo and Deusilene Souza Vieira1
EPI_ISL_514132	Rondônia Central Public Health Laboratory (LACEN/RO), vinculated to State Health Secretariat of Rondônia (SESAU/RO)	Molecular Virology Laboratory of Oswaldo Cruz Foundation of Rondônia	Luan Felipo Botelho-Souza, Felipe Souza Nogueira-Lima, Tárcio Peixoto Roca, Alcione de Oliveira dos Santos, Felipe Gomes Naveca, Adriana Cristina Salvador Maia, Cicileia Correia da Silva, Aline Linhares Ferreira de Melo Mendonça, Celina Aparecida Bertoni Lugtenburg, Camila Flávia Gomes Azzi, Juliana Loca Furtado, Suelen Cavalcante, Rita de Cássia Pontello Rampazzo, Caio Henrique Nemeth Santos, Alice Paula Di Sabatino Guimarães, Jansen Fernandes de Medeiros, Fernando Rodrigues Máximo, Juan Miguel Vilallobos-Salcedo and Deusilene Souza Vieira.
EPI_ISL_514133, EPI_ISL_514134, EPI_ISL_514135, EPI_ISL_514136, EPI_ISL_514137	Rondônia Central Public Health Laboratory (LACEN/RO), vinculated to State Health Secretariat of Rondônia (SESAU/RO)	Molecular Virology Laboratory of Oswaldo Cruz Foundation of Rondônia	Luan Felipo Botelho-Souza, Felipe Souza Nogueira-Lima, Tárcio Peixoto Roca, Alcione de Oliveira dos Santos, Felipe Gomes Naveca, Adriana Cristina Salvador Maia, Cicileia Correia da Silva, Aline Linhares Ferreira de Melo Mendonça, Celina Aparecida Bertoni Lugtenburg, Camila Flávia Gomes Azzi, Juliana Loca Furtado, Suelen Cavalcante, Rita de Cássia Pontello Rampazzo, Caio Henrique Nemeth Santos, Alice Paula Di Sabatino Guimarães, Jansen Fernandes de Medeiros, Fernando Rodrigues Máximo, Juan Miguel Vilallobos-Salcedo and Deusilene Souza Vieira
EPI_ISL_515520	Hospital Municipal do Tatuape Carmino Caricchio	Instituto Adolfo Lutz, Interdiciplinary Procedures Center, Strategic Laboratory	Claudio Tavares Sacchi, Claudia Regina Gonçalves, Erica Valessa Ramos Gomes
EPI_ISL_515521	Hospital Municipal Dr Waldemar Tebaldi	Instituto Adolfo Lutz, Interdiciplinary Procedures Center, Strategic Laboratory	Claudio Tavares Sacchi, Claudia Regina Gonçalves, Erica Valessa Ramos Gomes
EPI_ISL_515522	UPA 24HS de Itatiba	Instituto Adolfo Lutz, Interdiciplinary Procedures Center, Strategic Laboratory	Claudio Tavares Sacchi, Claudia Regina Gonçalves, Erica Valessa Ramos Gomes
EPI_ISL_515523, EPI_ISL_515524	PS Municipal Dr Lauro Ribas Braga	Instituto Adolfo Lutz, Interdiciplinary Procedures Center, Strategic Laboratory	Claudio Tavares Sacchi, Claudia Regina Gonçalves, Erica Valessa Ramos Gomes
EPI_ISL_515526	Hospital Municipal do Tatuape Carmino Caricchio	Instituto Adolfo Lutz, Interdiciplinary Procedures Center, Strategic Laboratory	Claudio Tavares Sacchi, Claudia Regina Gonçalves, Erica Valessa Ramos Gomes
EPI_ISL_515527	Hospital Santa Clara	Instituto Adolfo Lutz, Interdiciplinary Procedures Center, Strategic Laboratory	Claudio Tavares Sacchi, Claudia Regina Gonçalves, Erica Valessa Ramos Gomes
EPI_ISL_515528	Hospital Sao Paulo de Ensino da Unifesp	Instituto Adolfo Lutz, Interdiciplinary Procedures Center, Strategic Laboratory	Claudio Tavares Sacchi, Claudia Regina Gonçalves, Erica Valessa Ramos Gomes
EPI_ISL_515529	Pronto Socorro Municipal Julio Tupy	Instituto Adolfo Lutz, Interdiciplinary Procedures Center, Strategic Laboratory	Claudio Tavares Sacchi, Claudia Regina Gonçalves, Erica Valessa Ramos Gomes
EPI_ISL_515541	Hospital Montemagno	Instituto Adolfo Lutz, Interdiciplinary Procedures Center, Strategic Laboratory	Claudio Tavares Sacchi, Claudia Regina Gonçalves, Erica Valessa Ramos Gomes
EPI_ISL_515542	Vigilância Epidemiológica de Leme	Instituto Adolfo Lutz, Interdiciplinary Procedures Center, Strategic Laboratory	Claudio Tavares Sacchi, Claudia Regina Gonçalves, Erica Valessa Ramos Gomes
EPI_ISL_515543	Serviço de Vigilância Sanitária e Epidemiológica	Instituto Adolfo Lutz, Interdiciplinary Procedures Center, Strategic Laboratory	Claudio Tavares Sacchi, Claudia Regina Gonçalves, Erica Valessa Ramos Gomes
EPI_ISL_515544	Ama Dr Jose Soares Hungria	Instituto Adolfo Lutz, Interdiciplinary Procedures Center, Strategic Laboratory	Claudio Tavares Sacchi, Claudia Regina Gonçalves, Erica Valessa Ramos Gomes
EPI_ISL_515545	Hospital Sao Paulo de Ensino da Unifesp	Instituto Adolfo Lutz, Interdiciplinary Procedures Center, Strategic Laboratory	Claudio Tavares Sacchi, Claudia Regina Gonçalves, Erica Valessa Ramos Gomes
EPI_ISL_515546	Hospital Municipal do Tatuape Carmino Caricchio	Instituto Adolfo Lutz, Interdiciplinary Procedures Center, Strategic Laboratory	Claudio Tavares Sacchi, Claudia Regina Gonçalves, Erica Valessa Ramos Gomes
EPI_ISL_515547	Centro Medico da Policia Militar do Estado de Sao Paulo	Instituto Adolfo Lutz, Interdiciplinary Procedures Center, Strategic Laboratory	Claudio Tavares Sacchi, Claudia Regina Gonçalves, Erica Valessa Ramos Gomes
EPI_ISL_515548	Hospital Municipal Dr. Jose Soares Hungria	Instituto Adolfo Lutz, Interdiciplinary Procedures Center, Strategic Laboratory	Claudio Tavares Sacchi, Claudia Regina Gonçalves, Erica Valessa Ramos Gomes
EPI_ISL_515550	UPA Vila Santa Catarina	Instituto Adolfo Lutz, Interdiciplinary Procedures Center, Strategic Laboratory	Claudio Tavares Sacchi, Claudia Regina Gonçalves, Erica Valessa Ramos Gomes
EPI_ISL_515551, EPI_ISL_515552	Hospital Municipal do Tatuape Carmino Caricchio	Instituto Adolfo Lutz, Interdiciplinary Procedures Center, Strategic Laboratory	Claudio Tavares Sacchi, Claudia Regina Gonçalves, Erica Valessa Ramos Gomes
EPI_ISL_515553	Hospital Municipal Dr. Ignacio Proença de Gouvea	Instituto Adolfo Lutz, Interdiciplinary Procedures Center, Strategic Laboratory	Claudio Tavares Sacchi, Claudia Regina Gonçalves, Erica Valessa Ramos Gomes
EPI_ISL_515554	Pronto Socorro Municipal de Perus	Instituto Adolfo Lutz, Interdiciplinary Procedures Center, Strategic Laboratory	Claudio Tavares Sacchi, Claudia Regina Gonçalves, Erica Valessa Ramos Gomes
EPI_ISL_515555	Hospital Geral de Vila Nova Cachoeirinha	Instituto Adolfo Lutz, Interdiciplinary Procedures Center, Strategic Laboratory	Claudio Tavares Sacchi, Claudia Regina Gonçalves, Erica Valessa Ramos Gomes
EPI_ISL_515557	Hospital Municipal Dr. Moysés Deutsch	Instituto Adolfo Lutz, Interdiciplinary Procedures Center, Strategic Laboratory	Claudio Tavares Sacchi, Claudia Regina Gonçalves, Erica Valessa Ramos Gomes
EPI_ISL_515559, EPI_ISL_515560	Hospital Sao Paulo de Ensino da Unifesp	Instituto Adolfo Lutz, Interdiciplinary Procedures Center, Strategic Laboratory	Claudio Tavares Sacchi, Claudia Regina Gonçalves, Erica Valessa Ramos Gomes
EPI_ISL_515561	Hospital Montemagno	Instituto Adolfo Lutz, Interdiciplinary Procedures Center, Strategic Laboratory	Claudio Tavares Sacchi, Claudia Regina Gonçalves, Erica Valessa Ramos Gomes
EPI_ISL_515562	Hospital Municipal Doutor Alexandre Zaio	Instituto Adolfo Lutz, Interdiciplinary Procedures Center, Strategic Laboratory	Claudio Tavares Sacchi, Claudia Regina Gonçalves, Erica Valessa Ramos Gomes
EPI_ISL_515563	Hospital Municipal Dr. Jose Soares Hungria	Instituto Adolfo Lutz, Interdiciplinary Procedures Center, Strategic Laboratory	Claudio Tavares Sacchi, Claudia Regina Gonçalves, Erica Valessa Ramos Gomes

EPI_ISL_515564	Hosp. Municipal Prof. Dr. Alípio Corrêa Netto	Instituto Adolfo Lutz, Interdiciplinary Procedures Center, Strategic Laboratory	Claudio Tavares Sacchi, Claudia Regina Gonçalves, Erica Valessa Ramos Gomes
EPI_ISL_515565	Hospital do Servidor Público Estadual Francisco Morato de Oliveira	Instituto Adolfo Lutz, Interdiciplinary Procedures Center, Strategic Laboratory	Claudio Tavares Sacchi, Claudia Regina Gonçalves, Erica Valessa Ramos Gomes
EPI_ISL_515566	PS Municipal Dr Lauro Ribas Braga	Instituto Adolfo Lutz, Interdiciplinary Procedures Center, Strategic Laboratory	Claudio Tavares Sacchi, Claudia Regina Gonçalves, Erica Valessa Ramos Gomes
EPI_ISL_515938, EPI_ISL_515941, EPI_ISL_515942	CV RAMAN HOSPITAL	Department of Neurovirology, National Institute of Mental Health and Neuroscience (NIMHANS)	Chitra Pattabiraman,Vijayalakshmi Reddy, Harsha PK, Risha Rasheed, Pramada Prasad, Shafeeq S Hameed, Manjunatha Venkataswamy, Anita Desai, Ravi Vasanthapuram
EPI_ISL_515945, EPI_ISL_515947	DH	Department of Neurovirology, National Institute of Mental Health and Neuroscience (NIMHANS)	Chitra Pattabiraman,Vijayalakshmi Reddy, Harsha PK, Risha Rasheed, Pramada Prasad, Shafeeq S Hameed, Manjunatha Venkataswamy, Anita Desai, Ravi Vasanthapuram
EPI_ISL_515952	VICTORIA HOSPITAL	Department of Neurovirology, National Institute of Mental Health and Neuroscience (NIMHANS)	Chitra Pattabiraman,Vijayalakshmi Reddy, Harsha PK, Risha Rasheed, Pramada Prasad, Shafeeq S Hameed, Manjunatha Venkataswamy, Anita Desai, Ravi Vasanthapuram
EPI_ISL_515954, EPI_ISL_515957, EPI_ISL_515958, EPI_ISL_515959, EPI_ISL_515961	DH	Department of Neurovirology, National Institute of Mental Health and Neuroscience (NIMHANS)	Chitra Pattabiraman,Vijayalakshmi Reddy, Harsha PK, Risha Rasheed, Pramada Prasad, Shafeeq S Hameed, Manjunatha Venkataswamy, Anita Desai, Ravi Vasanthapuram
EPI_ISL_515963, EPI_ISL_515964, EPI_ISL_515966, EPI_ISL_515967	VICTORIA HOSPITAL	Department of Neurovirology, National Institute of Mental Health and Neuroscience (NIMHANS)	Chitra Pattabiraman,Vijayalakshmi Reddy, Harsha PK, Risha Rasheed, Pramada Prasad, Shafeeq S Hameed, Manjunatha Venkataswamy, Anita Desai, Ravi Vasanthapuram
EPI_ISL_516622	Instituto de Diagnostico y Referencia Epidemiologicos (INDRE)	Instituto de Diagnostico y Referencia Epidemiologicos (INDRE)	Gisela Barrera-Badillo , Abril Rodriguez-Maldonado, Claudia Wong-Arambula , Natividad Cruz-Ortiz, Tatiana Nunez-Garcia, Dayanira Arellano-Suarez, Fabiola Garces-Ayala, Edgar Mendieta-Condado, Lucia Hernandez-Rivas, Irma Lopez-Martinez, Ernesto Ramirez-Gonzalez.
EPI_ISL_516801, EPI_ISL_516802, EPI_ISL_516803, EPI_ISL_516804, EPI_ISL_516805	Department of Laboratory Medicine, Tan Tock Seng Hospital	Department of Laboratory Medicine, Tan Tock Seng Hospital	Chen YYC, Zair X, Li C, Tang WY, Maurer-Stroh S, Barkham TMS, Nagarajan N, Sessions OM
EPI_ISL_516940, EPI_ISL_516942, EPI_ISL_516943, EPI_ISL_516946, EPI_ISL_516948, EPI_ISL_516949, EPI_ISL_516969, EPI_ISL_516974	King Georges Medical University	CSIR-National Botanical Research Institute	Priti Prasad, Shantanu Prakash, Kishan Sahu, Babita Singh, Suruchi Shukla, Hricha Mishra, Danish Nasar Khan , Om Prakash, MLB Bhatt, SK Barik, Mehar H.Asif,Samir V. Sawant,Amita Jain, Sumit Kr. Bag
EPI_ISL_522547, EPI_ISL_522548	Platforme CYROI	UMR PIMIT Université de La Réunion	David Wilkinson, Camille Lebarbenchon, Patrick Mavingui
EPI_ISL_523811	Universidad Iberoamericana, Instituto de Medicina Tropical & Salud Global	International Centre for Genetic Engineering and Biotechnology (ICGEB) and ARGO Open Lab Platform	Robert Paulino-Ramirez, Eileen Riego, Alejandro Vallejo Degaudenzi, Victor Virgilio Calderon, Leandro Tapia, Danilo Licastro, Simeone Dal Monego, Sreejith Rajasekharan and Alessandro Marcello.
EPI_ISL_523955	Hospital Municipal do Tatuape Carmino Caricchio	Instituto Adolfo Lutz, Interdiciplinary Procedures Center, Strategic Laboratory	Claudio Tavares Sacchi, Claudia Regina Gonçalves, Erica Valessa Ramos Gomes
EPI_ISL_523956	Hospital Regional de Assis	Instituto Adolfo Lutz, Interdiciplinary Procedures Center, Strategic Laboratory	Claudio Tavares Sacchi, Claudia Regina Gonçalves, Erica Valessa Ramos Gomes
EPI_ISL_523957	Hospital Itamaraty	Instituto Adolfo Lutz, Interdiciplinary Procedures Center, Strategic Laboratory	Claudio Tavares Sacchi, Claudia Regina Gonçalves, Erica Valessa Ramos Gomes
EPI_ISL_523958	Pronto Socorro Municipal de Perus	Instituto Adolfo Lutz, Interdiciplinary Procedures Center, Strategic Laboratory	Claudio Tavares Sacchi, Claudia Regina Gonçalves, Erica Valessa Ramos Gomes
EPI_ISL_523963	UBS Vila Silvia	Instituto Adolfo Lutz, Interdiciplinary Procedures Center, Strategic Laboratory	Claudio Tavares Sacchi, Claudia Regina Gonçalves, Erica Valessa Ramos Gomes
EPI_ISL_523965	Hospital do Servidor Público Estadual Francisco Morato de Oliveira	Instituto Adolfo Lutz, Interdiciplinary Procedures Center, Strategic Laboratory	Claudio Tavares Sacchi, Claudia Regina Gonçalves, Erica Valessa Ramos Gomes
EPI_ISL_523967	Hospital Sancta Maggiore	Instituto Adolfo Lutz, Interdiciplinary Procedures Center, Strategic Laboratory	Claudio Tavares Sacchi, Claudia Regina Gonçalves, Erica Valessa Ramos Gomes
EPI_ISL_523969	Hospital Sao Paulo de Ensino da Unifesp	Instituto Adolfo Lutz, Interdiciplinary Procedures Center, Strategic Laboratory	Claudio Tavares Sacchi, Claudia Regina Gonçalves, Erica Valessa Ramos Gomes
EPI_ISL_523970	Conjunto Hospitalar do Mandaqui	Instituto Adolfo Lutz, Interdiciplinary Procedures Center, Strategic Laboratory	Claudio Tavares Sacchi, Claudia Regina Gonçalves, Erica Valessa Ramos Gomes
EPI_ISL_523971	Hospital Geral Santa Marcelina	Instituto Adolfo Lutz, Interdiciplinary Procedures Center, Strategic Laboratory	Claudio Tavares Sacchi, Claudia Regina Gonçalves, Erica Valessa Ramos Gomes
EPI_ISL_523972	Hospital do Servidor Público Estadual Francisco Morato de Oliveira	Instituto Adolfo Lutz, Interdiciplinary Procedures Center, Strategic Laboratory	Claudio Tavares Sacchi, Claudia Regina Gonçalves, Erica Valessa Ramos Gomes
EPI_ISL_523973	PS Municipal Dona Maria Antonieta Ferreira de Barros	Instituto Adolfo Lutz, Interdiciplinary Procedures Center, Strategic Laboratory	Claudio Tavares Sacchi, Claudia Regina Gonçalves, Erica Valessa Ramos Gomes
EPI_ISL_523974	Hospital Municipal do Tatuape Carmino Caricchio	Instituto Adolfo Lutz, Interdiciplinary Procedures Center, Strategic Laboratory	Claudio Tavares Sacchi, Claudia Regina Gonçalves, Erica Valessa Ramos Gomes
EPI_ISL_523975	UPA Tito Lopes	Instituto Adolfo Lutz, Interdiciplinary Procedures Center, Strategic Laboratory	Claudio Tavares Sacchi, Claudia Regina Gonçalves, Erica Valessa Ramos Gomes
EPI_ISL_523976	Hospital Municipal do Tatuape Carmino Caricchio	Instituto Adolfo Lutz, Interdiciplinary Procedures Center, Strategic Laboratory	Claudio Tavares Sacchi, Claudia Regina Gonçalves, Erica Valessa Ramos Gomes
EPI_ISL_523977	Hosp. Municipal Prof. Dr. Alípio Corrêa Netto	Instituto Adolfo Lutz, Interdiciplinary Procedures Center, Strategic Laboratory	Claudio Tavares Sacchi, Claudia Regina Gonçalves, Erica Valessa Ramos Gomes
EPI_ISL_523978	Hospital do Servidor Público Estadual Francisco Morato de Oliveira	Instituto Adolfo Lutz, Interdiciplinary Procedures Center, Strategic Laboratory	Claudio Tavares Sacchi, Claudia Regina Gonçalves, Erica Valessa Ramos Gomes
EPI_ISL_523980	UPA Tito Lopes	Instituto Adolfo Lutz, Interdiciplinary Procedures Center, Strategic Laboratory	Claudio Tavares Sacchi, Claudia Regina Gonçalves, Erica Valessa Ramos Gomes
EPI_ISL_523981	Hospital Sao Paulo de Ensino da Unifesp	Instituto Adolfo Lutz, Interdiciplinary Procedures Center, Strategic Laboratory	Claudio Tavares Sacchi, Claudia Regina Gonçalves, Erica Valessa Ramos Gomes
EPI_ISL_523982	Hospital do Servidor Público Estadual Francisco Morato de Oliveira	Instituto Adolfo Lutz, Interdiciplinary Procedures Center, Strategic Laboratory	Claudio Tavares Sacchi, Claudia Regina Gonçalves, Erica Valessa Ramos Gomes
EPI_ISL_523983	UPA Campo Limpo	Instituto Adolfo Lutz, Interdiciplinary Procedures Center, Strategic Laboratory	Claudio Tavares Sacchi, Claudia Regina Gonçalves, Erica Valessa Ramos Gomes

EPI_ISL_523984	Ama Dr Jose Soares Hungria	Instituto Adolfo Lutz, Interdiciplinary Procedures Center, Strategic Laboratory	Claudio Tavares Sacchi, Claudia Regina Gonçalves, Erica Valessa Ramos Gomes
EPI_ISL_523985	Hospital Municipal Dr. Benedicto Montenegro	Instituto Adolfo Lutz, Interdiciplinary Procedures Center, Strategic Laboratory	Claudio Tavares Sacchi, Claudia Regina Gonçalves, Erica Valessa Ramos Gomes
EPI_ISL_523986	Ama Dr Jose Soares Hungria	Instituto Adolfo Lutz, Interdiciplinary Procedures Center, Strategic Laboratory	Claudio Tavares Sacchi, Claudia Regina Gonçalves, Erica Valessa Ramos Gomes
EPI_ISL_523988	Hospital Sao Paulo de Ensino da Unifesp	Instituto Adolfo Lutz, Interdiciplinary Procedures Center, Strategic Laboratory	Claudio Tavares Sacchi, Claudia Regina Gonçalves, Erica Valessa Ramos Gomes
EPI_ISL_523989	AMA Jardim Joamar	Instituto Adolfo Lutz, Interdiciplinary Procedures Center, Strategic Laboratory	Claudio Tavares Sacchi, Claudia Regina Gonçalves, Erica Valessa Ramos Gomes
EPI_ISL_523990	AMA Jardim Peri	Instituto Adolfo Lutz, Interdiciplinary Procedures Center, Strategic Laboratory	Claudio Tavares Sacchi, Claudia Regina Gonçalves, Erica Valessa Ramos Gomes
EPI_ISL_523991, EPI_ISL_523992	Hospital Municipal Carmen Prudente	Instituto Adolfo Lutz, Interdiciplinary Procedures Center, Strategic Laboratory	Claudio Tavares Sacchi, Claudia Regina Gonçalves, Erica Valessa Ramos Gomes
EPI_ISL_524462	Hospital Metropolitano	Instituto Adolfo Lutz, Interdiciplinary Procedures Center, Strategic Laboratory	Claudio Tavares Sacchi, Claudia Regina Gonçalves, Erica Valessa Ramos Gomes
EPI_ISL_524463	Hospital Regional de Cotia	Instituto Adolfo Lutz, Interdiciplinary Procedures Center, Strategic Laboratory	Claudio Tavares Sacchi, Claudia Regina Gonçalves, Erica Valessa Ramos Gomes
EPI_ISL_524464	Santa Casa de Santa Isabel	Instituto Adolfo Lutz, Interdiciplinary Procedures Center, Strategic Laboratory	Claudio Tavares Sacchi, Claudia Regina Gonçalves, Erica Valessa Ramos Gomes
EPI_ISL_524465	PS Municipal Dr. Caetano Virgílio Neto	Instituto Adolfo Lutz, Interdiciplinary Procedures Center, Strategic Laboratory	Claudio Tavares Sacchi, Claudia Regina Gonçalves, Erica Valessa Ramos Gomes
EPI_ISL_524466	PS Municipal Dr Lauro Ribas Braga	Instituto Adolfo Lutz, Interdiciplinary Procedures Center, Strategic Laboratory	Claudio Tavares Sacchi, Claudia Regina Gonçalves, Erica Valessa Ramos Gomes
EPI_ISL_524467	Hospital Municipal Dr. Moysés Deutsch	Instituto Adolfo Lutz, Interdiciplinary Procedures Center, Strategic Laboratory	Claudio Tavares Sacchi, Claudia Regina Gonçalves, Erica Valessa Ramos Gomes
EPI_ISL_524468	Hospital Municipal Vereador Jose Storopolli	Instituto Adolfo Lutz, Interdiciplinary Procedures Center, Strategic Laboratory	Claudio Tavares Sacchi, Claudia Regina Gonçalves, Erica Valessa Ramos Gomes
EPI_ISL_524469	Santa Casa de Misericordia de Sao Paulo	Instituto Adolfo Lutz, Interdiciplinary Procedures Center, Strategic Laboratory	Claudio Tavares Sacchi, Claudia Regina Gonçalves, Erica Valessa Ramos Gomes
EPI_ISL_524470	Hospital do Servidor Público Estadual Francisco Morato de Oliveira	Instituto Adolfo Lutz, Interdiciplinary Procedures Center, Strategic Laboratory	Claudio Tavares Sacchi, Claudia Regina Gonçalves, Erica Valessa Ramos Gomes
EPI_ISL_524800	Evandro Chagas Institute	Evandro Chagas Institute	Santos, M.C.; Silva, A.M.; Junior, W.D.C.; Barbagelata, L.S.; Ferreira, J.A.; Sousa, E.M.A.; da Silva, P.S.; Resque, H.R; Martins, L.C.; Sousa Junior, E.C.;Viana, G.M.R
EPI_ISL_525467	Universidad Iberoamericana	International Centre for Genetic Engineering and Biotechnology (ICGEB) and ARGO Open Lab Platform	Robert Paulino-Ramirez, Eileen Riego, Alejandro Vallejo Degaudenzi, Victor Virgilio Calderon, Leandro Tapia, Patricia Leon, Danilo Licastro, Simeone Dal Monego, Sreejith Rajasekharan and Alessandro Marcello.
EPI_ISL_525468	Universidad Iberoamericana	International Centre for Genetic Engineering and Biotechnology (ICGEB) and ARGO Open Lab Platform	Robert Paulino-Ramirez, Eileen Riego, Alejandro Vallejo Degaudenzi, Victor Virgilio Calderon, Leandro Tapia,Patricia Leon,Danilo Licastro, Simeone Dal Monego, Sreejith Rajasekharan and Alessandro Marcello.
EPI_ISL_525469	Universidad Iberoamericana	International Centre for Genetic Engineering and Biotechnology (ICGEB) and ARGO Open Lab Platform	Robert Paulino-Ramirez, Eileen Riego, Alejandro Vallejo Degaudenzi, Victor Virgilio Calderon, Leandro Tapia, Patricia Leon, Danilo Licastro, Simeone Dal Monego, Sreejith Rajasekharan and Alessandro Marcello.
EPI_ISL_525474	Centre for Dengue Research	Centre for Dengue Research, USJ, SL	Chandima Jeewandara, Deshni Jayathilaka, Dinuka Ariyaratne, Laksiri Gomes, Diyanath Ranasinghe, Dinuka Guruge, Ruwan Wijayamuni, Gathsaurie Neelika Malavige
EPI_ISL_525476	Centre for Dengue Research	Centre for Dengue Research	Chandima Jeewandara, Deshni Jayathilaka, Dinuka Ariyaratne, Laksiri Gomes, Diyanath Ranasinghe, Dinuka Guruge, Ruwan Wijayamuni, Gathsaurie Neelika Malavige
EPI_ISL_525478, EPI_ISL_525479	Centre for Dengue Research	Centre for Dengue Research	Chandima Jeewandara, Deshni Jayathilaka, Dinuka Ariyaratne, Laksiri Gomes, Diyanath Ranasinghe, Ananda Wijewickrama, Eranga Narangoda, Damayanthi Idampitiya, Gathsaurie Neelika Malavige
EPI_ISL_525553, EPI_ISL_525556, EPI_ISL_525557, EPI_ISL_525570, EPI_ISL_525571	Istituto Zooprofilattico Sperimentale Puglia e Basilicata; Dipartimento di Bioscienze, Biotecnologie e Biofarmaceutica dell'Università degli Studi di Bari "A.Moro"; Istituto di Biomembrane. Bioenergetica e Biotecnologie Molecolari del Consiglio Nazionale delle Ricerche di Bari	Beaconlab (Bioinformatics, Evolution and Comparative Genomics lab), Dept of Biosciences, University on Milan	Parisi A.,Pesole G., Manzari C., Chiara M
EPI_ISL_525578, EPI_ISL_525621, EPI_ISL_525627, EPI_ISL_525631, EPI_ISL_525638, EPI_ISL_525639, EPI_ISL_525640, EPI_ISL_525641, EPI_ISL_525642, EPI_ISL_525646, EPI_ISL_525647, EPI_ISL_525648, EPI_ISL_525649, EPI_ISL_525650, EPI_ISL_525654, EPI_ISL_525658, EPI_ISL_525659, EPI_ISL_525676, EPI_ISL_525677, EPI_ISL_525678, EPI_ISL_525679, EPI_ISL_525680, EPI_ISL_525681, EPI_ISL_525682, EPI_ISL_525683, EPI_ISL_525684, EPI_ISL_525685, EPI_ISL_525686			
see above	Wadsworth Center, New York State Department of Health	Wadsworth Center, New York State Department of Health	Kirsten St. George, Daryl M. Lamson, Sara Griesemer, Jonathan Plitnick, Navjot Singh, Matthew D. Shudt, Erica Lasek-Nesselquist
EPI_ISL_526215, EPI_ISL_526216, EPI_ISL_526217, EPI_ISL_526218, EPI_ISL_526219, EPI_ISL_526220, EPI_ISL_526221, EPI_ISL_526222, EPI_ISL_526226, EPI_ISL_526227, EPI_ISL_526228, EPI_ISL_526229, EPI_ISL_526230, EPI_ISL_526231, EPI_ISL_526232, EPI_ISL_526233, EPI_ISL_526234, EPI_ISL_526235, EPI_ISL_526236, EPI_ISL_526237, EPI_ISL_526238			
see above	Hungarian Defence Forces Military Medical Centre	National Laboratory of Virology, Szentágothai Research Centre	Endre Gábor Tóth, Balázs Somogyi, Bálint Eszenyi, Ferenc Jakab, Gábor Kemenesi
EPI_ISL_526686, EPI_ISL_526687, EPI_ISL_526688	Faith Laboratory, Immunology Institute, Icahn School of Medicine at Mount Sinai	van Bakel Laboratory, Genetics and Genomics Sciences, Icahn School of Medicine at Mount Sinai	Graham J. Britton, Alice Chen-Liaw, Francesca Cossarini, Alexandra Livanos, Matthew P. Spindler, Tamar Plitt, Joseph Eggers, Ilaria Mogno, Ana S. Gonzalez-Reiche, Sophia Sui, Michael Tankelevich, Lauren Tal Grinspan, Rebekah E. Dixon, Divya Jha, Gustavo Martinez-Delgado, Fatima Amanat, Daisy Hoagland, Benjamin R. tenOever, Marla C. Dubinsky, Miriam Merad, Harm Van Bakel, Florian Krammer, Gerold Bongers, Saurabh Mehandru and Jeremiah J. Faith
EPI_ISL_526933, EPI_ISL_526934	Instituto Nacional de Salud, Bogotá, Colombia	Instituto Nacional de Salud, Bogotá, Colombia	Katherine Laiton-Donato, Diego A. Álvarez-Díaz, Carlos Franco-Muñoz, Mauricio Pacheco-Montealegre, Jonathan Reales, Diego Andrés Prada, Jose A. Usme-Ciro, Zulma M. Cucunubá, Christian Julian VillabonaArenas, Liz Villabona-Arenas, Sussy Echeverria, Astrid C. Flórez, Carolina Ferro, Diana Marcela Walteros-Acero, Franklin Prieto, Carlos Andrés Durán, Martha Lucia Ospina Martinez, Marcela Mercado-Reyes
EPI_ISL_526937, EPI_ISL_526938, EPI_ISL_526939, EPI_ISL_526940, EPI_ISL_526941, EPI_ISL_526943, EPI_ISL_526946	Faroese National Reference Laboratory for Fish and Animal Diseases	Faroese National Reference Laboratory for Fish and Animal Diseases	Maria Marjunardóttir Dahl, Petra Elisabeth Petersen, Debes Hammershaimb Christiansen
EPI_ISL_526949, EPI_ISL_526950, EPI_ISL_526951, EPI_ISL_526955, EPI_ISL_526956, EPI_ISL_526959	Instituto Nacional de Salud, Bogotá, Colombia	Instituto Nacional de Salud, Bogotá, Colombia	Katherine Laiton-Donato, Diego A. Álvarez-Díaz, Carlos Franco-Muñoz, Mauricio Pacheco-Montealegre, Jonathan Reales, Diego Andrés Prada, Jose A. Usme-Ciro, Zulma M. Cucunubá, Christian Julian VillabonaArenas, Liz Villabona-Arenas, Sussy Echeverria, Astrid C. Flórez, Carolina Ferro, Diana Marcela Walteros-Acero, Franklin Prieto, Carlos Andrés Durán, Martha Lucia Ospina Martinez, Marcela Mercado-Reyes

EPI_ISL_526963, EPI_ISL_526964, EPI_ISL_526965	Instituto Nacional de Salud, Bogotá, Colombia	Instituto Nacional de Salud, Bogotá, Colombia	Katherine Laiton-Donato, Diego A. Álvarez-Díaz, Carlos Franco-Muñoz, Jonathan Reales, Diego Andrés Prada, Jeadran Malagón-Rojas, Felix Betzler, Wendy K. Jo, Edmilson F. de Oliveira-Filho, Carolina Ferro, Diana Marcela Walteros-Acero, Franklin Prieto, Carlos Andrés Durán, Martha Lucia Ospina Martinez, Marcela Mercado-Reyes
EPI_ISL_527819	Centro de Investigaciones, Universidad de Especialidades Espíritu Santo	Institute of Microbiology, Universidad San Francisco de Quito	Derly Andrade, Juan Carlos Fernandez, Belén Prado-Vivar, Sully Márquez, Juan José Guadalupe, Monica Becerra-Wong, Bernardo Gutiérrez, Gabriel Morey, Ruben Armas, Jose Pedro Barberan, Fernando Espinoza, Edith Lopez, Verónica Barragán, Patricio Rojas-Silva, Gabriel Trueba, Michelle Grunauer, Paúl Cárdenas
EPI_ISL_527856	Hospital Municipal Prof. Waldomiro de Paula	Instituto Adolfo Lutz, Interdiciplinary Procedures Center, Strategic Laboratory	Claudio Tavares Sacchi, Claudia Regina Gonçalves, Erica Valessa Ramos Gomes
EPI_ISL_527857	Hospital Regional Vale do Ribeira	Instituto Adolfo Lutz, Interdiciplinary Procedures Center, Strategic Laboratory	Claudio Tavares Sacchi, Claudia Regina Gonçalves, Erica Valessa Ramos Gomes
EPI_ISL_527858	Pronto Atendimento Sancta Maggiore Jardim Paulista	Instituto Adolfo Lutz, Interdiciplinary Procedures Center, Strategic Laboratory	Claudio Tavares Sacchi, Claudia Regina Gonçalves, Erica Valessa Ramos Gomes
EPI_ISL_527859	Hospital Municipal Vereador Jose Storopolli	Instituto Adolfo Lutz, Interdiciplinary Procedures Center, Strategic Laboratory	Claudio Tavares Sacchi, Claudia Regina Gonçalves, Erica Valessa Ramos Gomes
EPI_ISL_527860	Hospital Municipal de Parelheiros Josanias Castanha Braga	Instituto Adolfo Lutz, Interdiciplinary Procedures Center, Strategic Laboratory	Claudio Tavares Sacchi, Claudia Regina Gonçalves, Erica Valessa Ramos Gomes
EPI_ISL_527861	Hospital e Maternidade Celso Pierro	Instituto Adolfo Lutz, Interdiciplinary Procedures Center, Strategic Laboratory	Av. Dr. Arnaldo, 355 - Brazil, Cerqueira Cesar, São Paulo - SP, 01246-1301
EPI_ISL_527862	Hospital Municipal de Urgência	Instituto Adolfo Lutz, Interdiciplinary Procedures Center, Strategic Laboratory	Claudio Tavares Sacchi, Claudia Regina Gonçalves, Erica Valessa Ramos Gomes
EPI_ISL_527863	Hospital Municipal do Tatuape Carmino Caricchio	Instituto Adolfo Lutz, Interdiciplinary Procedures Center, Strategic Laboratory	Claudio Tavares Sacchi, Claudia Regina Gonçalves, Erica Valessa Ramos Gomes
EPI_ISL_527864	Hospital e Pronto Socorro Comunitário Vila Iolanda	Instituto Adolfo Lutz, Interdiciplinary Procedures Center, Strategic Laboratory	Claudio Tavares Sacchi, Claudia Regina Gonçalves, Erica Valessa Ramos Gomes
EPI_ISL_527865	Hospital e Maternidade São Cristóvão	Instituto Adolfo Lutz, Interdiciplinary Procedures Center, Strategic Laboratory	Claudio Tavares Sacchi, Claudia Regina Gonçalves, Erica Valessa Ramos Gomes
EPI_ISL_527866	PS Municipal Dr Lauro Ribas Braga	Instituto Adolfo Lutz, Interdiciplinary Procedures Center, Strategic Laboratory	Av. Dr. Arnaldo, 355 - Brazil, Cerqueira Cesar, São Paulo - SP, 01246-1301
EPI_ISL_527867	Pronto Socorro Municipal - Balneario São José	Instituto Adolfo Lutz, Interdiciplinary Procedures Center, Strategic Laboratory	Claudio Tavares Sacchi, Claudia Regina Gonçalves, Erica Valessa Ramos Gomes
EPI_ISL_527868	Hospital e Maternidade do Braz	Instituto Adolfo Lutz, Interdiciplinary Procedures Center, Strategic Laboratory	Claudio Tavares Sacchi, Claudia Regina Gonçalves, Erica Valessa Ramos Gomes
EPI_ISL_527869	Hospital Municipal Carmen Prudente	Instituto Adolfo Lutz, Interdiciplinary Procedures Center, Strategic Laboratory	Claudio Tavares Sacchi, Claudia Regina Gonçalves, Erica Valessa Ramos Gomes
EPI_ISL_527870	Hospital Municipal Mário Gatti	Instituto Adolfo Lutz, Interdiciplinary Procedures Center, Strategic Laboratory	Claudio Tavares Sacchi, Claudia Regina Gonçalves, Erica Valessa Ramos Gomes
EPI_ISL_528382, EPI_ISL_528383, EPI_ISL_528384, EPI_ISL_528385	Translational Health Science and Technology Institute -ESIC medical college and hospital, Faridabad	THSTI Bioassay laboratory	Saurabh Kumar, Jigme Wangchuk, Anil Kumar Pandey, Asim Das, Guruprasad R. Medigeshi
EPI_ISL_528538	Alsafar	Alsafar	Andreas Henschel, Gihan Elsir Ahmed Daw Elbait, Samuel Feng, Rifat, Ernesto Damiani, Guan Tay, Habiba Alsafar
EPI_ISL_528539	LVM/UFRJ	LNCC	Gustavo M. Romário M. de Souza; Bruno B. Bezerra; Lucio A. Caldas; Fabio Limonte; Elena Cobos; Sharton V. A. Coelho; Luiz Almeida; Luiza Higga; Isadora A. Correa; Diana Marianni; Luciana B. Arruda; Marcelo Bozza; Orlando Ferreira; Wanderley de Souza; Ana Teresa R. Vasconcelos; Terezinha M. Castineiras; Amilcar Tanuri; Luciana J. Costa.
EPI_ISL_528637, EPI_ISL_528638	LVM/UFRJ	Bioinformatics Laboratory / LNCC	Gustavo D. P. Silva; M. Romário M. de Souza; Bruno B. Bezerra; Lucio A. Caldas; Fabio Limonte; Elena Cobos; Sharton V. A. Coelho; Luiz Almeida; Luiza Higga; Isadora A. Correa; Diana Marianni; Luciana B. Arruda; Marcelo Bozza; Orlando Ferreira; Wanderley de Souza; Ana Teresa R. Vasconcelos; Terezinha M. Castineiras; Amilcar Tanuri; Luciana J. Costa
EPI_ISL_528687, EPI_ISL_528689, EPI_ISL_528692, EPI_ISL_528693, EPI_ISL_528698, EPI_ISL_528699, EPI_ISL_528701, EPI_ISL_528704, EPI_ISL_528706, EPI_ISL_528709, EPI_ISL_528710, EPI_ISL_528711, EPI_ISL_528712, EPI_ISL_528713, EPI_ISL_528714, EPI_ISL_528715, EPI_ISL_528716, EPI_ISL_528717, EPI_ISL_528718, EPI_ISL_528719, EPI_ISL_528720, EPI_ISL_528721			
see above	Alsafar - Khalifa University Abu Dhabi	Alsafar - Khalifa University Abu Dhabi	Andreas Henschel, Gihan Daw Elbait, Samuel Feng, Rifat Hamoudi, Ernesto Damiani, Guan Tay, Habiba Alsafar
EPI_ISL_528738, EPI_ISL_528739, EPI_ISL_528740, EPI_ISL_528741	Malaysia Genome Institute	Malaysia Genome Institute	Mohd Noor Mat Isa, Irni Suhayu Sapian, Yusuf Muhammad Noor, Nurhezreen Md Iqbal, Mohd Faizal Abu Bakar, Enizza Kasim, Shamsidar Sopie, Siti Noraini Othman, Azrin Ahmad, Nor Azfa Johari, Shahrul Hisham Zainal Ariffin
EPI_ISL_528742	Malaysia Genome Institute	Malaysia Genome Institute	Mohd Noor Mat Isa, Irni Suhayu Sapian, Gan Han Ming, Yusuf Muhammad Noor, Tan Ju Lin, Nurhezreen Md Iqbal, Mohd Faizal Abu Bakar, Enizza Kasim, Shamsidar Sopie, Siti Noraini Othman, Azrin Ahmad, Nor Azfa Johari, Shahrul Hisham Zainal Ariffin
EPI_ISL_528749	Santosa Hospital Bandung Central	School of Life Sciences and Technology & School of Pharmacy-Institut Teknologi Bandung; Molecular Genetics Laboratory-Faculty of Medicine-Universitas Padjadjaran; Laboratorium Kesehatan Provinsi Jawa Barat	Husna Nugrahapraja, Azzania Fibriani, Catur Riani, Marselina Irasonia Tan, Yunia Sribudiani, Tarwadi, Ema Rahmawati, Savira Ekawardhani, Hesti Lina Wiraswati, Ryan Bayusantika Ristandi, Rifky Waluyajati Rachman, Cut Nur Cinthia Alamanda, Lia Faridah, Davin H. E. Setiamarga, Rizki Mardian, Hammam Riza, Sony Solistia Wirawan, Agung Eru Wibowo, Irvan Faizal
EPI_ISL_528919	Ospedale Civile S. Liberatore-Atri	Istituto Zooprofilattico Sperimentale dell'Abruzzo e Molise "G.Caporale"	Lorusso A, Marcacci M, Di Domenico M, Curini V, Ancora M, Cammà C, Rinaldi A, Mangone I, Di Pasquale A, Puglia I, Savini G.
EPI_ISL_528920, EPI_ISL_528921	Presidio Ospedaliero "Santo Spirito"-Pescara	Istituto Zooprofilattico Sperimentale dell'Abruzzo e Molise "G.Caporale"	Lorusso A, Marcacci M, Di Domenico M, Curini V, Ancora M, Cammà C, Rinaldi A, Mangone I, Di Pasquale A, Puglia I, Savini G.
EPI_ISL_528922, EPI_ISL_528924	Ospedale "Giuseppe Mazzini"-Teramo	Istituto Zooprofilattico Sperimentale dell'Abruzzo e Molise "G.Caporale"	Lorusso A, Marcacci M, Di Domenico M, Curini V, Ancora M, Cammà C, Rinaldi A, Mangone I, Di Pasquale A, Puglia I, Savini G.
EPI_ISL_528925	Ospedale Regionale San Salvatore-L'Aquila	Istituto Zooprofilattico Sperimentale dell'Abruzzo e Molise "G.Caporale"	Lorusso A, Marcacci M, Di Domenico M, Curini V, Ancora M, Cammà C, Rinaldi A, Mangone I, Di Pasquale A, Puglia I, Savini G.
EPI_ISL_528926, EPI_ISL_528927, EPI_ISL_528928	Ospedale "Giuseppe Mazzini"-Teramo	Istituto Zooprofilattico Sperimentale dell'Abruzzo e Molise "G.Caporale"	Lorusso A, Marcacci M, Di Domenico M, Curini V, Ancora M, Cammà C, Rinaldi A, Mangone I, Di Pasquale A, Puglia I, Savini G.
EPI_ISL_528929	Ospedale Civile S. Liberatore-Atri	Istituto Zooprofilattico Sperimentale dell'Abruzzo e Molise "G.Caporale"	Lorusso A, Marcacci M, Di Domenico M, Curini V, Ancora M, Cammà C, Rinaldi A, Mangone I, Di Pasquale A, Puglia I, Savini G.
EPI_ISL_528934, EPI_ISL_528935, EPI_ISL_528936, EPI_ISL_528937, EPI_ISL_528938, EPI_ISL_528939, EPI_ISL_528940, EPI_ISL_528941, EPI_ISL_528943, EPI_ISL_528944, EPI_ISL_528945, EPI_ISL_528946, EPI_ISL_528947, EPI_ISL_528948, EPI_ISL_528949			
see above	Agenzia di Tutela della Salute di Bergamo	Istituto Zooprofilattico Sperimentale dell'Abruzzo e Molise "G.Caporale"	Lorusso A, Marcacci M, Di Domenico M, Curini V, Ancora M, Cammà C, Rinaldi A, Mangone I, Di Pasquale A, Puglia I, Savini G.

EPI_ISL_528990	Ospedale Civile Maria SS. dello Splendore	Istituto Zooprofilattico Sperimentale dell'Abruzzo e Molise "G.Caporale"	Lorusso A, Marcacci M, Di Domenico M, Curini V, Ancora M, Cammà C, Rinaldi A, Mangone I, Di Pasquale A, Puglia I, Savini G.
EPI_ISL_528991	Ospedale SS Annunziata-Sulmona	Istituto Zooprofilattico Sperimentale dell'Abruzzo e Molise "G.Caporale"	Lorusso A, Marcacci M, Di Domenico M, Curini V, Ancora M, Cammà C, Rinaldi A, Mangone I, Di Pasquale A, Puglia I, Savini G.
EPI_ISL_528993	Ospedale Civile S. Liberatore-Atri	Istituto Zooprofilattico Sperimentale dell'Abruzzo e Molise "G.Caporale"	Lorusso A, Marcacci M, Di Domenico M, Curini V, Ancora M, Cammà C, Rinaldi A, Mangone I, Di Pasquale A, Puglia I, Savini G.
EPI_ISL_528994, EPI_ISL_528995, EPI_ISL_528996, EPI_ISL_528997, EPI_ISL_528998, EPI_ISL_528999, EPI_ISL_529000, EPI_ISL_529001	Servizio di igiene epidemiologia e sanità pubblica (SIESP)-Chieti	Istituto Zooprofilattico Sperimentale dell'Abruzzo e Molise "G.Caporale"	Lorusso A, Marcacci M, Di Domenico M, Curini V, Ancora M, Cammà C, Rinaldi A, Mangone I, Di Pasquale A, Puglia I, Savini G.
EPI_ISL_529009	Ospedale Civile S. Liberatore-Atri	Istituto Zooprofilattico Sperimentale dell'Abruzzo e Molise "G.Caporale"	Lorusso A, Marcacci M, Di Domenico M, Curini V, Ancora M, Cammà C, Rinaldi A, Mangone I, Di Pasquale A, Puglia I, Savini G.
EPI_ISL_529016	Ospedale SS Annunziata-Sulmona	Istituto Zooprofilattico Sperimentale dell'Abruzzo e Molise "G.Caporale"	Lorusso A, Marcacci M, Di Domenico M, Curini V, Ancora M, Cammà C, Rinaldi A, Mangone I, Di Pasquale A, Puglia I, Savini G.
EPI_ISL_529018	Ospedale "Giuseppe Mazzini"-Teramo	Istituto Zooprofilattico Sperimentale dell'Abruzzo e Molise "G.Caporale"	Lorusso A, Marcacci M, Di Domenico M, Curini V, Ancora M, Cammà C, Rinaldi A, Mangone I, Di Pasquale A, Puglia I, Savini G.
EPI_ISL_529019	RSA/RP Villa San Giovanni - Gruppo Edos	Istituto Zooprofilattico Sperimentale dell'Abruzzo e Molise "G.Caporale"	Lorusso A, Marcacci M, Di Domenico M, Curini V, Ancora M, Cammà C, Rinaldi A, Mangone I, Di Pasquale A, Puglia I, Savini G.
EPI_ISL_529020, EPI_ISL_529021	Ospedale Civile S. Liberatore-Atri	Istituto Zooprofilattico Sperimentale dell'Abruzzo e Molise "G.Caporale"	Lorusso A, Marcacci M, Di Domenico M, Curini V, Ancora M, Cammà C, Rinaldi A, Mangone I, Di Pasquale A, Puglia I, Savini G.
EPI_ISL_529022	Ospedale "Ss. Annunziata"	Istituto Zooprofilattico Sperimentale dell'Abruzzo e Molise "G.Caporale"	Lorusso A, Marcacci M, Di Domenico M, Curini V, Ancora M, Cammà C, Rinaldi A, Mangone I, Di Pasquale A, Puglia I, Savini G.
EPI_ISL_529026	Ospedale "Giuseppe Mazzini"-Teramo	Istituto Zooprofilattico Sperimentale dell'Abruzzo e Molise "G.Caporale"	Lorusso A, Marcacci M, Di Domenico M, Curini V, Ancora M, Cammà C, Rinaldi A, Mangone I, Di Pasquale A, Puglia I, Savini G.
EPI_ISL_529035, EPI_ISL_529036	Wadsworth Center, New York State Department of Health	Wadsworth Center, New York State Department of Health	Kirsten St. George, Daryl M. Lamson, Sara Griesemer, Jonathan Plitnick, Navjot Singh, Matthew D. Shudt, Erica Lasek-Nesselquist
EPI_ISL_529138	RSAL Dr. Ramelan Surabaya	Institute of Tropical Disease, Universitas Airlangga	Jezzy R Dewantari, Rima R Prasetya, Krisnoadi Rahardjo, Aldise M Nastri, Radito Soesanto, Gatot Soegiarto, Laksmi Wulandari, Retno A Setyoningrum, Resti Yudhawati, Yohko K Shimizu, Mitsuhiro Nishimura, Yasuko Mori, Soetjipto, Kazufumi Shimizu, Maria I Lusida
EPI_ISL_529961	Husada Utama Hospital	Institute of Tropical Disease, Universitas Airlangga	Krisnoadi Rahardjo, Aldise M Nastri, Jezzy R Dewantari, Rima R Prasetya, Didi Dewanto, Gatot Soegiarto, Laksmi Wulandari, Retno A Setyoningrum, Resti Yudhawati, Yohko K Shimizu, Mitsuhiro Nishimura, Yasuko Mori, Soetjipto, Kazufumi Shimizu, Maria I Lusida
EPI_ISL_529962	Universitas Airlangga Hospital	Institute of Tropical Disease, Universitas Airlangga	Jezzy R Dewantari, Rima R Prasetya, Krisnoadi Rahardjo, Aldise M Nastri, Nasronudin, Gatot Soegiarto, Laksmi Wulandari, Retno A Setyoningrum, Resti Yudhawati, Yohko K Shimizu, Mitsuhiro Nishimura, Yasuko Mori, Soetjipto, Kazufumi Shimizu, Maria I Lusida
EPI_ISL_529964	RSUD Bangil Pasuruan	Institute of Tropical Disease, Universitas Airlangga	Aldise M Nastri, Jezzy R Dewantari, Rima R Prasetya, Krisnoadi Rahardjo, Arma Roosalina, Gatot Soegiarto, Laksmi Wulandari, Retno A Setyoningrum, Resti Yudhawati, Yohko K Shimizu, Mitsuhiro Nishimura, Yasuko Mori, Soetjipto, Kazufumi Shimizu, Maria I Lusida
EPI_ISL_529965	RSUD Dr. Soetomo	Institute of Tropical Disease, Universitas Airlangga	Krisnoadi Rahardjo, Aldise M Nastri, Jezzy R Dewantari, Rima R Prasetya, Joni Wahyuhadi, Gatot Soegiarto, Laksmi Wulandari, Retno A Setyoningrum, Resti Yudhawati, Yohko K Shimizu, Mitsuhiro Nishimura, Yasuko Mori, Soetjipto, Kazufumi Shimizu, Maria I Lusida
EPI_ISL_529966	RSUD Sidoarjo	Institute of Tropical Disease, Universitas Airlangga	Kazufumi Shimizu, Krisnoadi Rahardjo, Aldise M Nastri, Jezzy R Dewantari, Rima R Prasetya, Atok Irawan, Gatot Soegiarto, Laksmi Wulandari, Retno A Setyoningrum, Resti Yudhawati, Yohko K Shimizu, Mitsuhiro Nishimura, Yasuko Mori, Soetjipto, Maria I Lusida
EPI_ISL_530347, EPI_ISL_530348, EPI_ISL_530349, EPI_ISL_530350	The National Institute of Public Health	State Veterinary Institute Prague	Nagy,A;Jirincova,H;Novakova,L;Trnka,D;Vecerova,J
EPI_ISL_534311	UPA III 26 de Agosto	Instituto Adolfo Lutz, Interdiciplinary Procedures Center, Strategic Laboratory	Claudio Tavares Sacchi, Claudia Regina Gonçalves, Erica Valessa Ramos Gomes
EPI_ISL_534312	Distrito Sanitario Sul	Instituto Adolfo Lutz, Interdiciplinary Procedures Center, Strategic Laboratory	Claudio Tavares Sacchi, Claudia Regina Gonçalves, Erica Valessa Ramos Gomes
EPI_ISL_534313	Hospital da Sta Casa de Sto Amaro	Instituto Adolfo Lutz, Interdiciplinary Procedures Center, Strategic Laboratory	Claudio Tavares Sacchi, Claudia Regina Gonçalves, Erica Valessa Ramos Gomes
EPI_ISL_534314	Hospital Universitario da USP de SP	Instituto Adolfo Lutz, Interdiciplinary Procedures Center, Strategic Laboratory	Claudio Tavares Sacchi, Claudia Regina Gonçalves, Erica Valessa Ramos Gomes
EPI_ISL_534315	Serviço de Verificação de Óbitos SVO Guaruulhos	Instituto Adolfo Lutz, Interdiciplinary Procedures Center, Strategic Laboratory	Claudio Tavares Sacchi, Claudia Regina Gonçalves, Erica Valessa Ramos Gomes
EPI_ISL_534316	OS Mun Santana Lauro Ribas Braga	Instituto Adolfo Lutz, Interdiciplinary Procedures Center, Strategic Laboratory	Claudio Tavares Sacchi, Claudia Regina Gonçalves, Erica Valessa Ramos Gomes
EPI_ISL_534317	Hospital Geral de Itapevi	Instituto Adolfo Lutz, Interdiciplinary Procedures Center, Strategic Laboratory	Claudio Tavares Sacchi, Claudia Regina Gonçalves, Erica Valessa Ramos Gomes
EPI_ISL_534318	Hospital Municipal Antonio Giglio	Instituto Adolfo Lutz, Interdiciplinary Procedures Center, Strategic Laboratory	Claudio Tavares Sacchi, Claudia Regina Gonçalves, Erica Valessa Ramos Gomes
EPI_ISL_534319, EPI_ISL_534320	Hospital do Serv Pub ESTAFCO Morato de Oliveira	Instituto Adolfo Lutz, Interdiciplinary Procedures Center, Strategic Laboratory	Claudio Tavares Sacchi, Claudia Regina Gonçalves, Erica Valessa Ramos Gomes
EPI_ISL_534321	PS e Maternidade Nair Fonseca Leitao Arantes	Instituto Adolfo Lutz, Interdiciplinary Procedures Center, Strategic Laboratory	Claudio Tavares Sacchi, Claudia Regina Gonçalves, Erica Valessa Ramos Gomes
EPI_ISL_534322	PS Mun Julio Tupy	Instituto Adolfo Lutz, Interdiciplinary Procedures Center, Strategic Laboratory	Claudio Tavares Sacchi, Claudia Regina Gonçalves, Erica Valessa Ramos Gomes
EPI_ISL_534323	Hospital e Pronto Socorro Comunitario Vila Yolanda	Instituto Adolfo Lutz, Interdiciplinary Procedures Center, Strategic Laboratory	Claudio Tavares Sacchi, Claudia Regina Gonçalves, Erica Valessa Ramos Gomes
EPI_ISL_534324	Hospital Mun Ver Jose Storopolli	Instituto Adolfo Lutz, Interdiciplinary Procedures Center, Strategic Laboratory	Claudio Tavares Sacchi, Claudia Regina Gonçalves, Erica Valessa Ramos Gomes
EPI_ISL_534325	Unidade de Vigilancia em Saude de Guarulhos	Instituto Adolfo Lutz, Interdiciplinary Procedures Center, Strategic Laboratory	Claudio Tavares Sacchi, Claudia Regina Gonçalves, Erica Valessa Ramos Gomes
EPI_ISL_534326	Notre Dame Intermedica Saude AS	Instituto Adolfo Lutz, Interdiciplinary Procedures Center, Strategic Laboratory	Claudio Tavares Sacchi, Claudia Regina Gonçalves, Erica Valessa Ramos Gomes
EPI_ISL_534346, EPI_ISL_534347,	Molecular diagnostic laboratory of Federal Budget Institution	Group of Genomics and Postgenomic Technologies of	Speranskaya AS, Kaptelova VV, Valdokhina AV, Bulanenko VP, Samoilov AE, Korneenko EV, Tivanova EV, Shipulina OY, Akimkin VG

EPI_ISL_534348, EPI_ISL_534349	of Science "Central Research Institute of Epidemiology" of The Federal Service on Customers' Rights Protection and Human Well-being Surveillance	Central Research Institute of Epidemilology	
EPI_ISL_536399	Laboratory of Immunovirology. Universidad de Antioquia	Instituto Nacional de Salud - Unidad de Secuenciación y Genómica	Wbeimar Aguilar-Jimenez, Lizdany Flórez, Francisco J. Díaz, Katherine Laiton-Donato, Carlos Franco-Muñoz, Diego Álvarez-Diaz and Marcela Mercado-Reyes
EPI_ISL_538435, EPI_ISL_538436, EPI_ISL_538437, EPI_ISL_538438, EPI_ISL_538439, EPI_ISL_538440, EPI_ISL_538441, EPI_ISL_538442, EPI_ISL_538443, EPI_ISL_538444, EPI_ISL_538445, EPI_ISL_538446, EPI_ISL_538447, EPI_ISL_538448, EPI_ISL_538449, EPI_ISL_538450, EPI_ISL_538451, EPI_ISL_538452, EPI_ISL_538453, EPI_ISL_538454, EPI_ISL_538455, EPI_ISL_538456, EPI_ISL_538457, EPI_ISL_538458, EPI_ISL_538459, EPI_ISL_538460, EPI_ISL_538461, EPI_ISL_538462, EPI_ISL_538463, EPI_ISL_538464, EPI_ISL_538465, EPI_ISL_538466, EPI_ISL_538467, EPI_ISL_538468, EPI_ISL_538469, EPI_ISL_538470, EPI_ISL_538471, EPI_ISL_538472, EPI_ISL_538473, EPI_ISL_538474, EPI_ISL_538475, EPI_ISL_538476, EPI_ISL_538477, EPI_ISL_538478, EPI_ISL_538479, EPI_ISL_538480, EPI_ISL_538481, EPI_ISL_538482, EPI_ISL_538483, EPI_ISL_538484, EPI_ISL_538485, EPI_ISL_538486, EPI_ISL_538487, EPI_ISL_538488, EPI_ISL_538489, EPI_ISL_538490, EPI_ISL_538491, EPI_ISL_538492, EPI_ISL_538493, EPI_ISL_538494, EPI_ISL_538495, EPI_ISL_538496, EPI_ISL_538497			
see above	Department of Laboratory Medicine, Tan Tock Seng Hospital	Department of Laboratory Medicine, Tan Tock Seng Hospital	Chen YYC, Zair X, Lim JX, Li C, Tang WY, Maurer-Stroh S, Barkham TMS, Nagarajan N, Sessions OM
EPI_ISL_539493, EPI_ISL_539494	The National Institute of Public Health	State Veterinary Institute Prague	Nagy,A;Jirincova,H;Novakova,L;Trnka,D;Vecerova,J
EPI_ISL_539496	Hospital Nostra Senyora de Meritxell	Instituto de Salud Carlos III	Iglesias-Caballero, M. Molinero Calamita, M. González-Esguevillas, M. Camarero, S. Pozo, F. Casas, I. Jiménez, P. Jiménez, M. Zaballos, A. Monzón, S. Varona, S. Juliá, M. Cuesta, I, F. Fernández
EPI_ISL_539497, EPI_ISL_539498	Hospital Virgen de las Nieves	Instituto de Salud Carlos III	Iglesias-Caballero, M. Molinero Calamita, M. González-Esguevillas, M. Camarero, S. Pozo, F. Casas, I. Jiménez, P. Jiménez, M. Zaballos, A. Monzón, S. Varona, S. Juliá, M. Cuesta, I, J. Lepe
EPI_ISL_539500, EPI_ISL_539501, EPI_ISL_539502	Hospital Clínico Universitario Lozano Blesa	Instituto de Salud Carlos III	Iglesias-Caballero, M. Molinero Calamita, M. González-Esguevillas, M. Camarero, S. Pozo, F. Casas, I. Jiménez, P. Jiménez, M. Zaballos, A. Monzón, S. Varona, S. Juliá, M. Cuesta, I, R. Benito
EPI_ISL_539503	Hospital Universitario Miguel Servet	Instituto de Salud Carlos III	Iglesias-Caballero, M. Molinero Calamita, M. González-Esguevillas, M. Camarero, S. Pozo, F. Casas, I. Jiménez, P. Jiménez, M. Zaballos, A. Monzón, S. Varona, S. Juliá, M. Cuesta, I, A. Rezusta
EPI_ISL_539504	Hospital Universitario Miguel Servet	Instituto de Salud Carlos III	Iglesias-Caballero, M. Molinero Calamita, M. González-Esguevillas, M. Camarero, S. Pozo, F. Casas, I. Jiménez, P. Jiménez, M. Zaballos, A. Monzón, S. Varona, S. Juliá, M. Cuesta, I, R. Benito
EPI_ISL_539505, EPI_ISL_539506, EPI_ISL_539507, EPI_ISL_539508	Hospital Universitario Miguel Servet	Instituto de Salud Carlos III	Iglesias-Caballero, M. Molinero Calamita, M. González-Esguevillas, M. Camarero, S. Pozo, F. Casas, I. Jiménez, P. Jiménez, M. Zaballos, A. Monzón, S. Varona, S. Juliá, M. Cuesta, I, A. Rezusta
EPI_ISL_539509, EPI_ISL_539510, EPI_ISL_539511, EPI_ISL_539512	Hospital Clínico Universitario Lozano Blesa	Instituto de Salud Carlos III	Iglesias-Caballero, M. Molinero Calamita, M. González-Esguevillas, M. Camarero, S. Pozo, F. Casas, I. Jiménez, P. Jiménez, M. Zaballos, A. Monzón, S. Varona, S. Juliá, M. Cuesta, I, R. Benito
EPI_ISL_539513, EPI_ISL_539514, EPI_ISL_539515, EPI_ISL_539516, EPI_ISL_539517, EPI_ISL_539518, EPI_ISL_539519	Hospital Universitario Miguel Servet	Instituto de Salud Carlos III	Iglesias-Caballero, M. Molinero Calamita, M. González-Esguevillas, M. Camarero, S. Pozo, F. Casas, I. Jiménez, P. Jiménez, M. Zaballos, A. Monzón, S. Varona, S. Juliá, M. Cuesta, I, A. Rezusta
EPI_ISL_539520, EPI_ISL_539521	Hospital Universitario de Ceuta	Instituto de Salud Carlos III	Iglesias-Caballero, M. Molinero Calamita, M. González-Esguevillas, M. Camarero, S. Pozo, F. Casas, I. Jiménez, P. Jiménez, M. Zaballos, A. Monzón, S. Varona, S. Juliá, M. Cuesta, I, J. López
EPI_ISL_539522	Hospital Universitario de Ceuta	Instituto de Salud Carlos III	Iglesias-Caballero, M. Molinero Calamita, M. González-Esguevillas, M. Camarero, S. Pozo, F. Casas, I. Jiménez, P. Jiménez, M. Zaballos, A. Monzón, S. Varona, S. Juliá, M. Cuesta, I, G. Sánchez
EPI_ISL_539523	Hospital General de Segovia	Instituto de Salud Carlos III	Iglesias-Caballero, M. Molinero Calamita, M. González-Esguevillas, M. Camarero, S. Pozo, F. Casas, I. Jiménez, P. Jiménez, M. Zaballos, A. Monzón, S. Varona, S. Juliá, M. Cuesta, I, S. Hernando
EPI_ISL_539524	Gerencia de Asistencia Sanitaria de Soria	Instituto de Salud Carlos III	Iglesias-Caballero, M. Molinero Calamita, M. González-Esguevillas, M. Camarero, S. Pozo, F. Casas, I. Jiménez, P. Jiménez, M. Zaballos, A. Monzón, S. Varona, S. Juliá, M. Cuesta, I, C. Aldea
EPI_ISL_539525	Hospital Nuestra Señora de Sonsoles	Instituto de Salud Carlos III	Iglesias-Caballero, M. Molinero Calamita, M. González-Esguevillas, M. Camarero, S. Pozo, F. Casas, I. Jiménez, P. Jiménez, M. Zaballos, A. Monzón, S. Varona, S. Juliá, M. Cuesta, I, A. San Pedro
EPI_ISL_539526, EPI_ISL_539527, EPI_ISL_539528, EPI_ISL_539529, EPI_ISL_539530	Consejeria de Sanidad y Asuntos Sociales	Instituto de Salud Carlos III	Iglesias-Caballero, M. Molinero Calamita, M. González-Esguevillas, M. Camarero, S. Pozo, F. Casas, I. Jiménez, P. Jiménez, M. Zaballos, A. Monzón, S. Varona, S. Juliá, M. Cuesta, I, G. Gutiérrez
EPI_ISL_539531	C.H.U Nuestra Señora de Candelaria	Instituto de Salud Carlos III	Iglesias-Caballero, M. Molinero Calamita, M. González-Esguevillas, M. Camarero, S. Pozo, F. Casas, I. Jiménez, P. Jiménez, M. Zaballos, A. Monzón, S. Varona, S. Juliá, M. Cuesta, I, O. Díez
EPI_ISL_539532	Hospital Universitario de Canarias	Instituto de Salud Carlos III	Iglesias-Caballero, M. Molinero Calamita, M. González-Esguevillas, M. Camarero, S. Pozo, F. Casas, I. Jiménez, P. Jiménez, M. Zaballos, A. Monzón, S. Varona, S. Juliá, M. Cuesta, I, B. Castro
EPI_ISL_539534, EPI_ISL_539535, EPI_ISL_539536, EPI_ISL_539537, EPI_ISL_539549, EPI_ISL_539550, EPI_ISL_539551, EPI_ISL_539552, EPI_ISL_539553, EPI_ISL_539554, EPI_ISL_539555, EPI_ISL_539556			
see above	Hospital Clínic	Instituto de Salud Carlos III	Iglesias-Caballero, M. Molinero Calamita, M. González-Esguevillas, M. Camarero, S. Pozo, F. Casas, I. Jiménez, P. Jiménez, M. Zaballos, A. Monzón, S. Varona, S. Juliá, M. Cuesta, I, M.A Marcos
EPI_ISL_539557	Gerencia del área de salud de Badajoz, Llerena y Zafra	Instituto de Salud Carlos III	Iglesias-Caballero, M. Molinero Calamita, M. González-Esguevillas, M. Camarero, S. Pozo, F. Casas, I. Jiménez, P. Jiménez, M. Zaballos, A. Monzón, S. Varona, S. Juliá, M. Cuesta, I, C. Pazos
EPI_ISL_539558	Hospital San Pedro de Alcántara	Instituto de Salud Carlos III	Iglesias-Caballero, M. Molinero Calamita, M. González-Esguevillas, M. Camarero, S. Pozo, F. Casas, I. Jiménez, P. Jiménez, M. Zaballos, A. Monzón, S. Varona, S. Juliá, M. Cuesta, I, E. Cerro
EPI_ISL_539559	Hospital Campo Arañuelo	Instituto de Salud Carlos III	Iglesias-Caballero, M. Molinero Calamita, M. González-Esguevillas, M. Camarero, S. Pozo, F. Casas, I. Jiménez, P. Jiménez, M. Zaballos, A. Monzón, S. Varona, S. Juliá, M. Cuesta, I, J. López
EPI_ISL_539560	Hospital Campo Arañuelo	Instituto de Salud Carlos III	Iglesias-Caballero, M. Molinero Calamita, M. González-Esguevillas, M. Camarero, S. Pozo, F. Casas, I. Jiménez, P. Jiménez, M. Zaballos, A. Monzón, S. Varona, S. Juliá, M. Cuesta, I, G. Rodríguez
EPI_ISL_539561	Hospital San Pedro de Alcántara	Instituto de Salud Carlos III	Iglesias-Caballero, M. Molinero Calamita, M. González-Esguevillas, M. Camarero, S. Pozo, F. Casas, I. Jiménez, P. Jiménez, M. Zaballos, A. Monzón, S. Varona, S. Juliá, M. Cuesta, I, J. López
EPI_ISL_539562	Hospital San Pedro de Alcántara	Instituto de Salud Carlos III	Iglesias-Caballero, M. Molinero Calamita, M. González-Esguevillas, M. Camarero, S. Pozo, F. Casas, I. Jiménez, P. Jiménez, M. Zaballos, A. Monzón, S. Varona, S. Juliá, M. Cuesta, I, M.A Cañizares
EPI_ISL_539569, EPI_ISL_539570, EPI_ISL_539571, EPI_ISL_539572	Complejo Hospitalario de Navarra	Instituto de Salud Carlos III	Iglesias-Caballero, M. Molinero Calamita, M. González-Esguevillas, M. Camarero, S. Pozo, F. Casas, I. Jiménez, P. Jiménez, M. Zaballos, A. Monzón, S. Varona, S. Juliá, M. Cuesta, I, J. López
EPI_ISL_539776	National Institute of Public Health (Czech Republic)	State Veterinary Institute Prague	Nagy, A; Jirincova, H; Novakova, L; Trnka, D; Vecerova, J.
EPI_ISL_539777	The National Institute of Public Health	State Veterinary Institute Prague	Nagy,A;Jirincova,H;Novakova,L;Trnka,D;Vecerova,J
EPI_ISL_539778, EPI_ISL_539779, EPI_ISL_539780, EPI_ISL_539781	National Institute of Public Health (Czech Republic)	State Veterinary Institute Prague	Nagy, A; Jirincova, H; Novakova, L; Trnka, D; Vecerova, J.
EPI_ISL_539820	Queen Mary Hospital	Hong Kong Department of Health	Alan K.L. Tsang, Peter C.W. Yip, Edman T.K. Lam, Rickjason C.W. Chan, Dominic N.C. Tsang
EPI_ISL_539850, EPI_ISL_539851	Pok Oi Hospital	Hong Kong Department of Health	Alan K.L. Tsang, Peter C.W. Yip, Edman T.K. Lam, Rickjason C.W. Chan, Dominic N.C. Tsang

EPI_ISL_540993, EPI_ISL_540994, EPI_ISL_540995, EPI_ISL_540996, EPI_ISL_540997, EPI_ISL_540998, EPI_ISL_540999, EPI_ISL_541000, EPI_ISL_541001	Health and Environmental Research Institute of Gwangju Metropolitan city	Health and Environmental Research Institute of Gwangju Metropolitan city	Min Ji Kim, Ji-eun Lee
EPI_ISL_541138	The National Institute of Public Health	State Veterinary Institute Prague	Nagy,A; Jirincova,H; Novakova,L; Trnka,D; Vecerova,J
EPI_ISL_541335	The National Institute of Public Health	Sídlištní 136/24 165 03, Prague Czech Republic	Nagy,A; Jirincova,H; Novakova,L; Trnka,D; Vecerova,J
EPI_ISL_541352	Laboratory of Respiratory Viruses and Measles, Oswaldo Cruz Institute, FIOCRUZ	Laboratory of Respiratory Viruses and Measles, Oswaldo Cruz Institute, FIOCRUZ	Paola Resende, Roxana Loayza, Cinthia Avila, Luciana Appolinario, Fernando Motta, Anna Carolina Paixao, Ana Carolina Mendonca, Marilda Siqueira on behalf of the Fiocruz COVID-19 Genomic Surveillance Network
EPI_ISL_541372, EPI_ISL_541374, EPI_ISL_541375, EPI_ISL_541376, EPI_ISL_541379, EPI_ISL_541383, EPI_ISL_541385	Laboratório Central de Saúde Pública do Estado de Sergipe (LACEN-SE)	Laboratory of Respiratory Viruses and Measles, Oswaldo Cruz Institute, FIOCRUZ	Paola Resende, Luciana Appolinario, Fernando Motta, Anna Carolina Paixão, Ana Carolina Mendonça, Jonathan Lopes, Clioma Santos, Marilda Siqueira on behalf of the Fiocruz COVID-19 Genomic Surveillance Network
EPI_ISL_541970	Influenza Centre, University of Bergen	Norwegian Institute of Public Health, Department of Virology	Fan Zhou, Rebecca J Cox, Karl A Brokstad, Bjørn Blomberg, Kathrine Stene-Johansen, Kamilla Heddeland Instefjord, Hilde Elshaug, Rasmus Riis Kopperud, Hilde Synnøve Vollan, Karoline Bragstad, Olav Hungnes
EPI_ISL_542099, EPI_ISL_542101, EPI_ISL_542104, EPI_ISL_542106, EPI_ISL_542107, EPI_ISL_542108, EPI_ISL_542110, EPI_ISL_542112, EPI_ISL_542114, EPI_ISL_542116, EPI_ISL_542118, EPI_ISL_542119, EPI_ISL_542120, EPI_ISL_542121, EPI_ISL_542122, EPI_ISL_542124, EPI_ISL_542128, EPI_ISL_542130, EPI_ISL_542131, EPI_ISL_542133, EPI_ISL_542145, EPI_ISL_542146, EPI_ISL_542152, EPI_ISL_542153, EPI_ISL_542154, EPI_ISL_542155, EPI_ISL_542156, EPI_ISL_542158, EPI_ISL_542159, EPI_ISL_542162, EPI_ISL_542165, EPI_ISL_542166, EPI_ISL_542169, EPI_ISL_542170, EPI_ISL_542172, EPI_ISL_542173, EPI_ISL_542174, EPI_ISL_542175, EPI_ISL_542178, EPI_ISL_542180, EPI_ISL_542181, EPI_ISL_542183, EPI_ISL_542192, EPI_ISL_542193, EPI_ISL_542194, EPI_ISL_542195, EPI_ISL_542196, EPI_ISL_542197, EPI_ISL_542198, EPI_ISL_542199, EPI_ISL_542200, EPI_ISL_542201, EPI_ISL_542202, EPI_ISL_542203, EPI_ISL_542204, EPI_ISL_542205, EPI_ISL_542206, EPI_ISL_542207, EPI_ISL_542208, EPI_ISL_542209, EPI_ISL_542210, EPI_ISL_542211, EPI_ISL_542212, EPI_ISL_542213, EPI_ISL_542214, EPI_ISL_542215, EPI_ISL_542216, EPI_ISL_542217, EPI_ISL_542218, EPI_ISL_542219, EPI_ISL_542220, EPI_ISL_542221, EPI_ISL_542222, EPI_ISL_542223, EPI_ISL_542224, EPI_ISL_542225, EPI_ISL_542226, EPI_ISL_542228, EPI_ISL_542229, EPI_ISL_542230, EPI_ISL_542231, EPI_ISL_542232, EPI_ISL_542233, EPI_ISL_542234, EPI_ISL_542236, EPI_ISL_542237, EPI_ISL_542238, EPI_ISL_542239, EPI_ISL_542240, EPI_ISL_542241, EPI_ISL_542242, EPI_ISL_542243, EPI_ISL_542248, EPI_ISL_542249, EPI_ISL_542252, EPI_ISL_542253, EPI_ISL_542254, EPI_ISL_542255, EPI_ISL_542256, EPI_ISL_542257, EPI_ISL_542258, EPI_ISL_542260, EPI_ISL_542261, EPI_ISL_542262, EPI_ISL_542263, EPI_ISL_542264, EPI_ISL_542274, EPI_ISL_542275, EPI_ISL_542276, EPI_ISL_542277			
see above	ASST GOM Niguarda	Dep. Of Oncology and Hemato-Oncology University of Milan	Claudia Alteri, Valeria Cento, Antonio Piralla, Valentino Costabile, Monica Tallarita, Luna Colagrossi, Silvia Renica, Federica Giardina, Federica Novazzi, Stefano Gaiarsa, Elisa Matarazzo, Maria Antonello, Chiara Vismara, Roberto Fumagalli, Oscar Massimiliano Epis, Massimo Puoti, Carlo Federico Perno, Fausto Baldanti
EPI_ISL_542278, EPI_ISL_542279, EPI_ISL_542280, EPI_ISL_542281, EPI_ISL_542282, EPI_ISL_542283, EPI_ISL_542284, EPI_ISL_542285, EPI_ISL_542286, EPI_ISL_542287, EPI_ISL_542288, EPI_ISL_542289, EPI_ISL_542290, EPI_ISL_542291, EPI_ISL_542292, EPI_ISL_542294, EPI_ISL_542297, EPI_ISL_542299, EPI_ISL_542302, EPI_ISL_542303, EPI_ISL_542304, EPI_ISL_542305, EPI_ISL_542306, EPI_ISL_542307, EPI_ISL_542308, EPI_ISL_542309, EPI_ISL_542310, EPI_ISL_542311, EPI_ISL_542312, EPI_ISL_542313, EPI_ISL_542314, EPI_ISL_542315, EPI_ISL_542316, EPI_ISL_542317, EPI_ISL_542318, EPI_ISL_542319, EPI_ISL_542320, EPI_ISL_542321, EPI_ISL_542322, EPI_ISL_542323, EPI_ISL_542324, EPI_ISL_542325, EPI_ISL_542326, EPI_ISL_542327, EPI_ISL_542328, EPI_ISL_542329, EPI_ISL_542330, EPI_ISL_542331, EPI_ISL_542332, EPI_ISL_542333, EPI_ISL_542334, EPI_ISL_542335, EPI_ISL_542336, EPI_ISL_542337, EPI_ISL_542338, EPI_ISL_542339, EPI_ISL_542340, EPI_ISL_542341, EPI_ISL_542342, EPI_ISL_542343, EPI_ISL_542344, EPI_ISL_542345, EPI_ISL_542346, EPI_ISL_542347, EPI_ISL_542348, EPI_ISL_542349, EPI_ISL_542350, EPI_ISL_542351, EPI_ISL_542352, EPI_ISL_542353, EPI_ISL_542354, EPI_ISL_542355, EPI_ISL_542357, EPI_ISL_542358, EPI_ISL_542359, EPI_ISL_542360, EPI_ISL_542361, EPI_ISL_542362, EPI_ISL_542363, EPI_ISL_542364, EPI_ISL_542365, EPI_ISL_542366, EPI_ISL_542367, EPI_ISL_542369, EPI_ISL_542370, EPI_ISL_542371, EPI_ISL_542372, EPI_ISL_542373, EPI_ISL_542374, EPI_ISL_542375, EPI_ISL_542376, EPI_ISL_542377, EPI_ISL_542378, EPI_ISL_542379, EPI_ISL_542381, EPI_ISL_542382, EPI_ISL_542383, EPI_ISL_542384, EPI_ISL_542385, EPI_ISL_542386, EPI_ISL_542387, EPI_ISL_542388, EPI_ISL_542389, EPI_ISL_542390, EPI_ISL_542391, EPI_ISL_542392			
see above	San Matteo Hospital Pavia	Dep. Of Oncology and Hemato-Oncology University of Milan	Claudia Alteri, Valeria Cento, Antonio Piralla, Valentino Costabile, Monica Tallarita, Luna Colagrossi, Silvia Renica, Federica Giardina, Federica Novazzi, Stefano Gaiarsa, Elisa Matarazzo, Maria Antonello, Chiara Vismara, Roberto Fumagalli, Oscar Massimiliano Epis, Massimo Puoti, Carlo Federico Perno, Fausto Baldanti
EPI_ISL_542413, EPI_ISL_542415, EPI_ISL_542419, EPI_ISL_542420, EPI_ISL_542421, EPI_ISL_542422, EPI_ISL_542423, EPI_ISL_542424, EPI_ISL_542425, EPI_ISL_542426, EPI_ISL_542427, EPI_ISL_542428, EPI_ISL_542429, EPI_ISL_542431, EPI_ISL_542432, EPI_ISL_542433, EPI_ISL_542435, EPI_ISL_542440, EPI_ISL_542442, EPI_ISL_542443			
see above	ASST GOM Niguarda	Dep. Of Oncology and Hemato-Oncology University of Milan	Claudia Alteri, Valeria Cento, Antonio Piralla, Valentino Costabile, Monica Tallarita, Luna Colagrossi, Silvia Renica, Federica Giardina, Federica Novazzi, Stefano Gaiarsa, Elisa Matarazzo, Maria Antonello, Chiara Vismara, Roberto Fumagalli, Oscar Massimiliano Epis, Massimo Puoti, Carlo Federico Perno, Fausto Baldanti
EPI_ISL_547570	PS Municipal Dr Augusto Gomes de Mattos	Instituto Adolfo Lutz, Interdiciplinary Procedures Center, Strategic Laboratory	Claudio Tavares Sacchi, Claudia Regina Gonçalves, Erica Valessa Ramos Gomes, Karoline Rodrigues Campos
EPI_ISL_548208, EPI_ISL_548214	Laboratoire de Virologie, HUG	Swiss National Reference Centre for Influenza	LAUBSCHER F.
EPI_ISL_548243, EPI_ISL_548244	Faith Laboratory, Immunology Institute, Icahn School of Medicine at Mount Sinai	van Bakel Laboratory, Genetics and Genomics Sciences, Icahn School of Medicine at Mount Sinai	Graham J. Britton, Alice Chen-Liaw, Francesca Cossarini, Alexandra Livanos, Matthew P. Spindler, Tamar Plitt, Joseph Eggers, Ilaria Mogno, Ana S. Gonzalez-Reiche, Sophia Sui, Michael Tankelevich, Lauren Tal Grinspan, Rebekah E. Dixon, Divya Jha, Gustavo Martinez-Delgado, Fatima Amanat, Daisy Hoagland, Benjamin R. tenOever, Marla C. Dubinsky, Miriam Merad, Harm Van Bakel, Florian Krammer, Gerold Bongers, Saurabh Mehandru and Jeremiah J. Faith
EPI_ISL_548966, EPI_ISL_548967, EPI_ISL_548968, EPI_ISL_548970, EPI_ISL_548971	Expo2020 Emergency Center	Agiomix	Walaa Allam, Cherif Ben Hamada, Cengiz Yakicier, Walid Dridi, Rashid Mohammed, Tamer Degheidy
EPI_ISL_560407	Istituto Zooprofilattico Sperimentale del Mezzogiorno	INMI Lazzaro Spallanzani IRCCS	Barbara Bartolini, Cesare E.M. Gruber, Martina Rueca, Francesco Messina, Antonino Di Caro, Giovanna Fusco, Maurizio Viscardi, Giorgia Borriello, Sergio Brandi, Maria R. Capobianchi
EPI_ISL_560568, EPI_ISL_560569, EPI_ISL_560570, EPI_ISL_560571, EPI_ISL_560572, EPI_ISL_560573, EPI_ISL_560574, EPI_ISL_560575, EPI_ISL_560576, EPI_ISL_560577, EPI_ISL_560578, EPI_ISL_560579, EPI_ISL_560580			
see above	hôpital	National Reference Center for Viruses of Respiratory Infections, Institut Pasteur, Paris	Sylvie Behillil, Fabiana Gambaro, Etienne Simon-Lorière, Vincent Enouf, Maud Vanpeene, Sylvie van der Werf
EPI_ISL_560581, EPI_ISL_560582, EPI_ISL_560583, EPI_ISL_560584, EPI_ISL_560585, EPI_ISL_560586	Hopital	National Reference Center for Viruses of Respiratory Infections, Institut Pasteur, Paris	Sylvie Behillil, Fabiana Gambaro, Etienne Simon-Lorière, Vincent Enouf, Maud Vanpeene, Sylvie van der Werf
EPI_ISL_560591, EPI_ISL_560592, EPI_ISL_560593, EPI_ISL_560594, EPI_ISL_560595, EPI_ISL_560596, EPI_ISL_560597	hopital	National Reference Center for Viruses of Respiratory Infections, Institut Pasteur, Paris	Sylvie Behillil, Fabiana Gambaro, Etienne Simon-Lorière, Vincent Enouf, Maud Vanpeene, Sylvie van der Werf
EPI_ISL_560620, EPI_ISL_560621, EPI_ISL_560622, EPI_ISL_560623, EPI_ISL_560624, EPI_ISL_560625, EPI_ISL_560626, EPI_ISL_560627, EPI_ISL_560628, EPI_ISL_560629, EPI_ISL_560630, EPI_ISL_560631, EPI_ISL_560632, EPI_ISL_560633, EPI_ISL_560634, EPI_ISL_560644			
see above	Hospital	National Reference Center for Viruses of Respiratory Infections, Institut Pasteur, Paris	Sylvie Behillil, Fabiana Gambaro, Etienne Simon-Lorière, Vincent Enouf, Maud Vanpeene, Sylvie van der Werf
EPI_ISL_560991	RSUD Dr. Soetomo	Institute of Tropical Disease, Universitas Airlangga	Rima R Prasetya, Krisnoadi Rahardjo, Aldise M Nastri, Jezzy R Dewantari, Joni Wahyuhadi, Gatot Soegiarto, Laksmi Wulandari, Retno A Setyoningrum, Resti Yudhawati, Yohko K Shimizu, Mitsuhiro Nishimura, Yasuko Mori, Soetjipto, Kazufumi Shimizu, Maria I Lusida
EPI_ISL_568579	Virus Molecular Laboratory of the Microbiology and Virology Department	INMI Lazzaro Spallanzani IRCCS	Cesare E.M. Gruber, Martina Rueca, Barbara Bartolini, Francesco Messina, Silvia Meschi, Francesca Colavita, Concetta Castilletti, Elena Percivalle, Irene Cassaniti, Edoardo Vecchio Nepita, Fausto Baldanti, Maria R. Capobianchi, Antonino Di Caro
EPI_ISL_568686	RSUD Prof. DR. Margono Soekarjo	Eijkman Institute for Molecular Biology, Ministry of Research and Technology/National Agency for Research and Innovation	Frilasita A Yudhaputri, Edison Johar, Hidayat Trimarsanto, Iskandar A Adnan, Willy Agustine, David H Muljono, Safarina G Malik, Herawati Sudoyo, Khin Saw Myint, Amin Soebandrio
EPI_ISL_568687	RSUP Fatmawati	Eijkman Institute for Molecular Biology, Ministry of Research and Technology/National Agency for Research and Innovation	Frilasita A Yudhaputri, Edison Johar, Hidayat Trimarsanto, Iskandar A Adnan, Willy Agustine, David H Muljono, Safarina G Malik, Herawati Sudoyo, Khin Saw Myint, Amin Soebandrio

EPI_ISL_568688	RS Pelni	Eijkman Institute for Molecular Biology, Ministry of Research and Technology/National Agency for Research and Innovation	Frilasita A Yudhaputri, Edison Johar, Hidayat Trimarsanto, Iskandar A Adnan, Willy Agustine, David H Muljono, Safarina G Malik, Herawati Sudoyo, Khin Saw Myint, Amin Soebandrio
EPI_ISL_568693	RS Freeport Tembagapura	Eijkman Institute for Molecular Biology, Ministry of Research and Technology/National Agency for Research and Innovation	Frilasita A Yudhaputri, Edison Johar, Hidayat Trimarsanto, Iskandar A Adnan, Willy Agustine, David H Muljono, Safarina G Malik, Herawati Sudoyo, Khin Saw Myint, Amin Soebandrio
EPI_ISL_568694	RSUP Prof. Dr. R. Kandou Manado	Eijkman Institute for Molecular Biology, Ministry of Research and Technology/National Agency for Research and Innovation	Frilasita A Yudhaputri, Edison Johar, Hidayat Trimarsanto, Iskandar A Adnan, Willy Agustine, David H Muljono, Safarina G Malik, Herawati Sudoyo, Khin Saw Myint, Amin Soebandrio
EPI_ISL_568873, EPI_ISL_568874, EPI_ISL_568875	Malaysia Genome Institute	Malaysia Genome Institute	Mohd Noor Mat Isa, Irni Suhayu Sapian, Yusuf Muhammad Noor, Nurhezreen Md Iqbal, Mohd Faizal Abu Bakar, Enizza Kasim, Shamsidar Sopie, Siti Noraini Othman, Azrin Ahmad, Nor Azfa Johari, Shahrul Hisham Zainal Ariffin
EPI_ISL_569865, EPI_ISL_569866, EPI_ISL_569867, EPI_ISL_569868, EPI_ISL_569869, EPI_ISL_569870, EPI_ISL_569871, EPI_ISL_569872, EPI_ISL_569873, EPI_ISL_569875, EPI_ISL_569876, EPI_ISL_569877, EPI_ISL_569878, EPI_ISL_569879, EPI_ISL_569880, EPI_ISL_569881, EPI_ISL_569882, EPI_ISL_569883, EPI_ISL_569884, EPI_ISL_569885, EPI_ISL_569886			
see above	Amedeo di savoia	Crosetto lab, Karolinska Institutet, SciLifeLab	Michele Simonetti, Maria Grazia Milia, Luuk Harbers, Ning Zhang, Anna Sapino, Valeria Ghisetti, Nicola Crosetto
EPI_ISL_572330	Institute for Virology, University Hospital Duesseldorf, Medical Faculty, Heinrich-Heine-University Duesseldorf	Institute for Virology, University Hospital Duesseldorf, Medical Faculty, Heinrich-Heine-University Duesseldorf	Maximilian Damagnez, Verena Keitel, Björn Jensen, Nadine Lübke, Lisa Müller, Philipp Ostermann, Tina Senff, Ortwin Adams, Philipp Albrecht, Gerald Antoch, Johannes Bode, Edwin Bölke, Saskia Elben, Torsten Feldt, Johannes C. Fischer, , Anselm Kunstein, Caroline Klindt, Alexander Killer, Tom Lüdde, Annemarie Mohring, Jennifer Neubert, Heiner Schaal, Ansgar Schulz, Jörg Timm, Andreas Walker
EPI_ISL_572351, EPI_ISL_572386	Laboratório Central de Saúde Pública do Estado de Pernambuco (LACEN-PE)	WallauLab, Aggeu Magalhaes Institute	Marcelo Henrique Santos Paiva, Duschinka Ribeiro Duarte Guedes, Cássia Docena, Matheus Filgueira Bezerra, Filipe Zimmer Dezordi, Laís Ceschini Machado, Larissa Krokovsky, Elisama Helvecio, Alexandre Freitas da Silva, Luydson Richardson Silva Vasconcelos, Antonio Mauro Rezende, Severino Jefferson Ribeiro da Silva, Kamila Gaudêncio da Silva Sales, Bruna Santos Lima Figueiredo de Sá, Derciliano Lopes da Cruz, Claudio Eduardo Cavalcanti, Armando de Menezes Neto, Caroline Targino Alves da Silva, Renata Pessôa Germano Mendes, Maria Almerice Lopes da Silva, Tiago Gräf, Paola Cristina Resende, Gonzalo Bello, Michelle da Silva Barros, Wheverton Ricardo Correia do Nascimento, Rodrigo Moraes Loyo Arcoverde, Luciane Caroline Albuquerque Bezerra, Sinval Pinto Brandão Filho, Constância Flávia Junqueira Ayres, Gabriel Luz Wallau on behalf of the Fiocruz COVID-19 Genomic Surveillance Network
EPI_ISL_574577	Hospital Municipal Dr. Ignacio Proença de Gouvea	Instituto Adolfo Lutz, Interdiciplinary Procedures Center, Strategic Laboratory	Claudio Tavares Sacchi, Claudia Regina Gonçalves, Erica Valessa Ramos Gomes, Karoline Rodrigues Campos
EPI_ISL_574578	Hospital Municipal Mário Gatti	Instituto Adolfo Lutz, Interdiciplinary Procedures Center, Strategic Laboratory	Claudio Tavares Sacchi, Claudia Regina Gonçalves, Erica Valessa Ramos Gomes, Karoline Rodrigues Campos
EPI_ISL_574579	Hospital Municipal Dr. Ignacio Proença de Gouvea	Instituto Adolfo Lutz, Interdiciplinary Procedures Center, Strategic Laboratory	Claudio Tavares Sacchi, Claudia Regina Gonçalves, Erica Valessa Ramos Gomes, Karoline Rodrigues Campos
EPI_ISL_574580	Hospital Cidade Tiradentes Carmen Prudente	Instituto Adolfo Lutz, Interdiciplinary Procedures Center, Strategic Laboratory	Claudio Tavares Sacchi, Claudia Regina Gonçalves, Erica Valessa Ramos Gomes, Karoline Rodrigues Campos
EPI_ISL_574582	Hospital Municipal Dr. Jose Soares Hungria	Instituto Adolfo Lutz, Interdiciplinary Procedures Center, Strategic Laboratory	Claudio Tavares Sacchi, Claudia Regina Gonçalves, Erica Valessa Ramos Gomes, Karoline Rodrigues Campos
EPI_ISL_574583	Secretaria Municipal de Saude de Jandira	Instituto Adolfo Lutz, Interdiciplinary Procedures Center, Strategic Laboratory	Claudio Tavares Sacchi, Claudia Regina Gonçalves, Erica Valessa Ramos Gomes, Karoline Rodrigues Campos
EPI_ISL_574588	Hospital Estadual Sumare	Instituto Adolfo Lutz, Interdiciplinary Procedures Center, Strategic Laboratory	Claudio Tavares Sacchi, Claudia Regina Gonçalves, Erica Valessa Ramos Gomes, Karoline Rodrigues Campos
EPI_ISL_574589	Hospital Municipal Dr. Jose Soares Hungria	Instituto Adolfo Lutz, Interdiciplinary Procedures Center, Strategic Laboratory	Claudio Tavares Sacchi, Claudia Regina Gonçalves, Erica Valessa Ramos Gomes, Karoline Rodrigues Campos
EPI_ISL_574590	Unidade de Printo Atendimento UPA I Santa Isabel	Instituto Adolfo Lutz, Interdiciplinary Procedures Center, Strategic Laboratory	Claudio Tavares Sacchi, Claudia Regina Gonçalves, Erica Valessa Ramos Gomes, Karoline Rodrigues Campos
EPI_ISL_574591, EPI_ISL_574592	Hospital Domingos Leonardo Ceravolo Presidente Prudente	Instituto Adolfo Lutz, Interdiciplinary Procedures Center, Strategic Laboratory	Claudio Tavares Sacchi, Claudia Regina Gonçalves, Erica Valessa Ramos Gomes, Karoline Rodrigues Campos
EPI_ISL_574593	CS II Dr. Antonio Vicoso Moreira de Rezende Sumare	Instituto Adolfo Lutz, Interdiciplinary Procedures Center, Strategic Laboratory	Claudio Tavares Sacchi, Claudia Regina Gonçalves, Erica Valessa Ramos Gomes, Karoline Rodrigues Campos
EPI_ISL_574595	Hospital Geral de Vila Penteado Dr. Jose Pamgella	Instituto Adolfo Lutz, Interdiciplinary Procedures Center, Strategic Laboratory	Claudio Tavares Sacchi, Claudia Regina Gonçalves, Erica Valessa Ramos Gomes, Karoline Rodrigues Campos
EPI_ISL_574603	RS Freeport Tembagapura	Eijkman Institute for Molecular Biology, Ministry of Research and Technology/National Agency for Research and Innovation	Frilasita A Yudhaputri, Edison Johar, Hidayat Trimarsanto, Iskandar A Adnan, Willy Agustine, David H Muljono, Safarina G Malik, Herawati Sudoyo, Khin Saw Myint, Amin Soebandrio
EPI_ISL_574604	RSUP Prof. Dr. R. Kandou Manado	Eijkman Institute for Molecular Biology, Ministry of Research and Technology/National Agency for Research and Innovation	Frilasita A Yudhaputri, Edison Johar, Hidayat Trimarsanto, Iskandar A Adnan, Willy Agustine, David H Muljono, Safarina G Malik, Herawati Sudoyo, Khin Saw Myint, Amin Soebandrio
EPI_ISL_574605	RSUP dr. Sardjito	Eijkman Institute for Molecular Biology, Ministry of Research and Technology/National Agency for Research and Innovation	Frilasita A Yudhaputri, Edison Johar, Hidayat Trimarsanto, Iskandar A Adnan, Willy Agustine, David H Muljono, Safarina G Malik, Herawati Sudoyo, Khin Saw Myint, Amin Soebandrio
EPI_ISL_574606	RS Siloam Purwakarta	Eijkman Institute for Molecular Biology, Ministry of Research and Technology/National Agency for Research and Innovation	Frilasita A Yudhaputri, Edison Johar, Hidayat Trimarsanto, Iskandar A Adnan, Willy Agustine, David H Muljono, Safarina G Malik, Herawati Sudoyo, Khin Saw Myint, Amin Soebandrio
EPI_ISL_574607	RS Husada	Eijkman Institute for Molecular Biology, Ministry of Research and Technology/National Agency for Research and Innovation	Frilasita A Yudhaputri, Edison Johar, Hidayat Trimarsanto, Iskandar A Adnan, Willy Agustine, David H Muljono, Safarina G Malik, Herawati Sudoyo, Khin Saw Myint, Amin Soebandrio
EPI_ISL_574608	RS Mitra Keluarga Kelapa Gading	Eijkman Institute for Molecular Biology, Ministry of Research and Technology/National Agency for Research and Innovation	Frilasita A Yudhaputri, Edison Johar, Hidayat Trimarsanto, Iskandar A Adnan, Willy Agustine, David H Muljono, Safarina G Malik, Herawati Sudoyo, Khin Saw Myint, Amin Soebandrio
EPI_ISL_574609	RSJPD Harapan Kita	Eijkman Institute for Molecular Biology, Ministry of Research and Technology/National Agency for Research and Innovation	Frilasita A Yudhaputri, Edison Johar, Hidayat Trimarsanto, Iskandar A Adnan, Willy Agustine, David H Muljono, Safarina G Malik, Herawati Sudoyo, Khin Saw Myint, Amin Soebandrio
EPI_ISL_574610	Puskesmas Tambora	Eijkman Institute for Molecular Biology, Ministry of Research and Technology/National Agency for Research and Innovation	Frilasita A Yudhaputri, Edison Johar, Hidayat Trimarsanto, Iskandar A Adnan, Willy Agustine, David H Muljono, Safarina G Malik, Herawati Sudoyo, Khin Saw Myint, Amin Soebandrio
EPI_ISL_574611	RS Premier Bintaro	Eijkman Institute for Molecular Biology, Ministry of Research and Technology/National Agency for Research and	Frilasita A Yudhaputri, Edison Johar, Hidayat Trimarsanto, Iskandar A Adnan, Willy Agustine, David H Muljono, Safarina G Malik, Herawati Sudoyo, Khin Saw Myint, Amin Soebandrio

		Innovation	
EPI_ISL_575330	National Institute for Viral Disease Control and Prevention, China CDC	National Institute for Viral Disease Control and Prevention, China CDC	Rongbao Gao, Kang Xiao, Qinqin Song, ZhiQiang Xia, Dong Xia, Juan Song, Haijun Du, Yuan He, Shuai Pang, Xuancheng Lu, Guizhen Wu, Geogia Fu Gao, Jun Han
EPI_ISL_577629, EPI_ISL_577631	The National Institute of Public Health	State Veterinary Institute Prague	Nagy,A;Jirincova,H;Novakova,L;Trnka,D;Vecerova,J
EPI_ISL_578186	Hospital General Juan Ramón Jiménez	Instituto de Salud Carlos III	Iglesias-Caballero, M. Molinero Calamita, M. González-Esguevillas, M. Camarero, S. Pozo, F. Casas, I. Jiménez, P. Jiménez, M. Zaballos, A. Monzón, S. Varona, S. Juliá, M. Cuesta, I, J. Saavedra
EPI_ISL_578189	Hospital Universitario Miguel Servet	Instituto de Salud Carlos III	Iglesias-Caballero, M. Molinero Calamita, M. González-Esguevillas, M. Camarero, S. Pozo, F. Casas, I. Jiménez, P. Jiménez, M. Zaballos, A. Monzón, S. Varona, S. Juliá, M. Cuesta, I, A. Rezusta
EPI_ISL_578190, EPI_ISL_578191	Hospital Clínico Universitario Lozano Blesa	Instituto de Salud Carlos III	Iglesias-Caballero, M. Molinero Calamita, M. González-Esguevillas, M. Camarero, S. Pozo, F. Casas, I. Jiménez, P. Jiménez, M. Zaballos, A. Monzón, S. Varona, S. Juliá, M. Cuesta, I, R. Benito
EPI_ISL_578192, EPI_ISL_578193	Hospital Universitario Miguel Servet	Instituto de Salud Carlos III	Iglesias-Caballero, M. Molinero Calamita, M. González-Esguevillas, M. Camarero, S. Pozo, F. Casas, I. Jiménez, P. Jiménez, M. Zaballos, A. Monzón, S. Varona, S. Juliá, M. Cuesta, I, A. Rezusta
EPI_ISL_578194	Complejo Hospitalario Universitario La Coruna	Instituto de Salud Carlos III	Iglesias-Caballero, M. Molinero Calamita, M. González-Esguevillas, M. Camarero, S. Pozo, F. Casas, I. Jiménez, P. Jiménez, M. Zaballos, A. Monzón, S. Varona, S. Juliá, M. Cuesta, I, J. López
EPI_ISL_578195	Complejo Hospitalario de Orense	Instituto de Salud Carlos III	Iglesias-Caballero, M. Molinero Calamita, M. González-Esguevillas, M. Camarero, S. Pozo, F. Casas, I. Jiménez, P. Jiménez, M. Zaballos, A. Monzón, S. Varona, S. Juliá, M. Cuesta, I, M. García
EPI_ISL_578196	Hospital San Pedro	Instituto de Salud Carlos III	Iglesias-Caballero, M. Molinero Calamita, M. González-Esguevillas, M. Camarero, S. Pozo, F. Casas, I. Jiménez, P. Jiménez, M. Zaballos, A. Monzón, S. Varona, S. Juliá, M. Cuesta, I, J.M Azcona
EPI_ISL_578197	Hospital San Pedro	Instituto de Salud Carlos III	Iglesias-Caballero, M. Molinero Calamita, M. González-Esguevillas, M. Camarero, S. Pozo, F. Casas, I. Jiménez, P. Jiménez, M. Zaballos, A. Monzón, S. Varona, S. Juliá, M. Cuesta, I, C. Alonso
EPI_ISL_578198, EPI_ISL_578199	Hospital San Pedro	Instituto de Salud Carlos III	Iglesias-Caballero, M. Molinero Calamita, M. González-Esguevillas, M. Camarero, S. Pozo, F. Casas, I. Jiménez, P. Jiménez, M. Zaballos, A. Monzón, S. Varona, S. Juliá, M. Cuesta, I, M. Blasco
EPI_ISL_578200, EPI_ISL_578201	Hospital San Pedro	Instituto de Salud Carlos III	Iglesias-Caballero, M. Molinero Calamita, M. González-Esguevillas, M. Camarero, S. Pozo, F. Casas, I. Jiménez, P. Jiménez, M. Zaballos, A. Monzón, S. Varona, S. Juliá, M. Cuesta, I, C. Alonso
EPI_ISL_582033	Department of Pathology, School of Medicine, Imam Khomeini Hospital, Tehran University of Medical Sciences	Genetics Research Center. University Of Social Welfare And Rehabilitation Sciences	Zohreh Fattahi, Marzieh Mohseni, Khadijeh Jalalvand, Azam Ghaziasadi, Seyedeh elham Mortazavi, Ali Jafarpour, Azar Hadadi, Alireza Abdollahi, Ali Jafarpour, Azam Ghaziasad, Seyedeh elham Mortazavi, Saber Soltani, Reza Najafipour , Kimia Kahrizi, Seyed Mohammad Jazayeri, Hossein Najmabadi
EPI_ISL_582125, EPI_ISL_582126	Sheikh Khalifa Medical City	Molecular Surveillance lab Sheikh Khalifa Medical City	Amirtharaj Francis, Sajeed Abdul, Hala Imambaccus, Sahar Almarzooqi, Hiba Saud, Stefan Weber
EPI_ISL_582127	Antwerp University Hospital	Institute of Tropical Medicine	Philippe Selhorst, Colin Anthony
EPI_ISL_582608, EPI_ISL_582613, EPI_ISL_582620, EPI_ISL_582624, EPI_ISL_582625, EPI_ISL_582632	Sheikh Khalifa Medical City	Molecular/Surveillance lab Sheikh Khalifa Medical City	Amirtharaj Francis, Sajeed Abdul, Hala Imambaccus, Sahar Almarzooqi, Hiba Saud, Stefan Weber
EPI_ISL_583426	Garcia-Sastre Laboratory, Department of Microbiology, Icahn School of Medicine at Mount Sinai	van Bakel Laboratory, Genetics and Genomics Sciences, Icahn School of Medicine at Mount Sinai	Teresa Aydillo, Ana S. Gonzalez-Reiche, Sadaf Aslam, Adriana van de Guchte, Zenab Khan, Ajay Obla, Jayeeta Dutta, Harm van Bakel, Judith Aberg, Adolfo García-Sastre, Gunjan Shah, Tobias Hohl, Genovefa Papanicolaou, Miguel-Angel Perales, Kent Sepkowitz, Ngolela Esther Babady, and Mini Kamboj
EPI_ISL_583427, EPI_ISL_583428	Memorial Sloan Kettering Cancer Center	van Bakel Laboratory, Genetics and Genomics Sciences, Icahn School of Medicine at Mount Sinai	Teresa Aydillo, Ana S. Gonzalez-Reiche, Sadaf Aslam, Adriana van de Guchte, Zenab Khan, Ajay Obla, Jayeeta Dutta, Harm van Bakel, Judith Aberg, Adolfo García-Sastre, Gunjan Shah, Tobias Hohl, Genovefa Papanicolaou, Miguel-Angel Perales, Kent Sepkowitz, Ngolela Esther Babady, and Mini Kamboj
EPI_ISL_583429	Garcia-Sastre Laboratory, Department of Microbiology, Icahn School of Medicine at Mount Sinai	van Bakel Laboratory, Genetics and Genomics Sciences, Icahn School of Medicine at Mount Sinai	Teresa Aydillo, Ana S. Gonzalez-Reiche, Sadaf Aslam, Adriana van de Guchte, Zenab Khan, Ajay Obla, Jayeeta Dutta, Harm van Bakel, Judith Aberg, Adolfo García-Sastre, Gunjan Shah, Tobias Hohl, Genovefa Papanicolaou, Miguel-Angel Perales, Kent Sepkowitz, Ngolela Esther Babady, and Mini Kamboj
EPI_ISL_583430, EPI_ISL_583431, EPI_ISL_583432, EPI_ISL_583433	Memorial Sloan Kettering Cancer Center	van Bakel Laboratory, Genetics and Genomics Sciences, Icahn School of Medicine at Mount Sinai	Teresa Aydillo, Ana S. Gonzalez-Reiche, Sadaf Aslam, Adriana van de Guchte, Zenab Khan, Ajay Obla, Jayeeta Dutta, Harm van Bakel, Judith Aberg, Adolfo García-Sastre, Gunjan Shah, Tobias Hohl, Genovefa Papanicolaou, Miguel-Angel Perales, Kent Sepkowitz, Ngolela Esther Babady, and Mini Kamboj
EPI_ISL_583434, EPI_ISL_583435, EPI_ISL_583436	Garcia-Sastre Laboratory, Department of Microbiology, Icahn School of Medicine at Mount Sinai	van Bakel Laboratory, Genetics and Genomics Sciences, Icahn School of Medicine at Mount Sinai	Teresa Aydillo, Ana S. Gonzalez-Reiche, Sadaf Aslam, Adriana van de Guchte, Zenab Khan, Ajay Obla, Jayeeta Dutta, Harm van Bakel, Judith Aberg, Adolfo García-Sastre, Gunjan Shah, Tobias Hohl, Genovefa Papanicolaou, Miguel-Angel Perales, Kent Sepkowitz, Ngolela Esther Babady, and Mini Kamboj
EPI_ISL_583437, EPI_ISL_583438, EPI_ISL_583439, EPI_ISL_583440	Memorial Sloan Kettering Cancer Center	van Bakel Laboratory, Genetics and Genomics Sciences, Icahn School of Medicine at Mount Sinai	Teresa Aydillo, Ana S. Gonzalez-Reiche, Sadaf Aslam, Adriana van de Guchte, Zenab Khan, Ajay Obla, Jayeeta Dutta, Harm van Bakel, Judith Aberg, Adolfo García-Sastre, Gunjan Shah, Tobias Hohl, Genovefa Papanicolaou, Miguel-Angel Perales, Kent Sepkowitz, Ngolela Esther Babady, and Mini Kamboj
EPI_ISL_583441, EPI_ISL_583442	Garcia-Sastre Laboratory, Department of Microbiology, Icahn School of Medicine at Mount Sinai	van Bakel Laboratory, Genetics and Genomics Sciences, Icahn School of Medicine at Mount Sinai	Teresa Aydillo, Ana S. Gonzalez-Reiche, Sadaf Aslam, Adriana van de Guchte, Zenab Khan, Ajay Obla, Jayeeta Dutta, Harm van Bakel, Judith Aberg, Adolfo García-Sastre, Gunjan Shah, Tobias Hohl, Genovefa Papanicolaou, Miguel-Angel Perales, Kent Sepkowitz, Ngolela Esther Babady, and Mini Kamboj
EPI_ISL_583445, EPI_ISL_583446, EPI_ISL_583447, EPI_ISL_583448, EPI_ISL_583456, EPI_ISL_583457	Memorial Sloan Kettering Cancer Center	van Bakel Laboratory, Genetics and Genomics Sciences, Icahn School of Medicine at Mount Sinai	Teresa Aydillo, Ana S. Gonzalez-Reiche, Sadaf Aslam, Adriana van de Guchte, Zenab Khan, Ajay Obla, Jayeeta Dutta, Harm van Bakel, Judith Aberg, Adolfo García-Sastre, Gunjan Shah, Tobias Hohl, Genovefa Papanicolaou, Miguel-Angel Perales, Kent Sepkowitz, Ngolela Esther Babady, and Mini Kamboj
EPI_ISL_583458, EPI_ISL_583459	Garcia-Sastre Laboratory, Department of Microbiology, Icahn School of Medicine at Mount Sinai	van Bakel Laboratory, Genetics and Genomics Sciences, Icahn School of Medicine at Mount Sinai	Teresa Aydillo, Ana S. Gonzalez-Reiche, Sadaf Aslam, Adriana van de Guchte, Zenab Khan, Ajay Obla, Jayeeta Dutta, Harm van Bakel, Judith Aberg, Adolfo García-Sastre, Gunjan Shah, Tobias Hohl, Genovefa Papanicolaou, Miguel-Angel Perales, Kent Sepkowitz, Ngolela Esther Babady, and Mini Kamboj
EPI_ISL_583460, EPI_ISL_583461, EPI_ISL_583462	Memorial Sloan Kettering Cancer Center	van Bakel Laboratory, Genetics and Genomics Sciences, Icahn School of Medicine at Mount Sinai	Teresa Aydillo, Ana S. Gonzalez-Reiche, Sadaf Aslam, Adriana van de Guchte, Zenab Khan, Ajay Obla, Jayeeta Dutta, Harm van Bakel, Judith Aberg, Adolfo García-Sastre, Gunjan Shah, Tobias Hohl, Genovefa Papanicolaou, Miguel-Angel Perales, Kent Sepkowitz, Ngolela Esther Babady, and Mini Kamboj
EPI_ISL_583463, EPI_ISL_583464	Garcia-Sastre Laboratory, Department of Microbiology, Icahn School of Medicine at Mount Sinai	van Bakel Laboratory, Genetics and Genomics Sciences, Icahn School of Medicine at Mount Sinai	Teresa Aydillo, Ana S. Gonzalez-Reiche, Sadaf Aslam, Adriana van de Guchte, Zenab Khan, Ajay Obla, Jayeeta Dutta, Harm van Bakel, Judith Aberg, Adolfo García-Sastre, Gunjan Shah, Tobias Hohl, Genovefa Papanicolaou, Miguel-Angel Perales, Kent Sepkowitz, Ngolela Esther Babady, and Mini Kamboj
EPI_ISL_583465, EPI_ISL_583466	Memorial Sloan Kettering Cancer Center	van Bakel Laboratory, Genetics and Genomics Sciences, Icahn School of Medicine at Mount Sinai	Teresa Aydillo, Ana S. Gonzalez-Reiche, Sadaf Aslam, Adriana van de Guchte, Zenab Khan, Ajay Obla, Jayeeta Dutta, Harm van Bakel, Judith Aberg, Adolfo García-Sastre, Gunjan Shah, Tobias Hohl, Genovefa Papanicolaou, Miguel-Angel Perales, Kent Sepkowitz, Ngolela Esther Babady, and Mini Kamboj
EPI_ISL_583467	Garcia-Sastre Laboratory, Department of Microbiology, Icahn School of Medicine at Mount Sinai	van Bakel Laboratory, Genetics and Genomics Sciences, Icahn School of Medicine at Mount Sinai	Teresa Aydillo, Ana S. Gonzalez-Reiche, Sadaf Aslam, Adriana van de Guchte, Zenab Khan, Ajay Obla, Jayeeta Dutta, Harm van Bakel, Judith Aberg, Adolfo García-Sastre, Gunjan Shah, Tobias Hohl, Genovefa Papanicolaou, Miguel-Angel Perales, Kent Sepkowitz, Ngolela Esther Babady, and Mini Kamboj
EPI_ISL_583468, EPI_ISL_583469, EPI_ISL_583470, EPI_ISL_583472, EPI_ISL_583475, EPI_ISL_583478, EPI_ISL_583479, EPI_ISL_583480	Memorial Sloan Kettering Cancer Center	van Bakel Laboratory, Genetics and Genomics Sciences, Icahn School of Medicine at Mount Sinai	Teresa Aydillo, Ana S. Gonzalez-Reiche, Sadaf Aslam, Adriana van de Guchte, Zenab Khan, Ajay Obla, Jayeeta Dutta, Harm van Bakel, Judith Aberg, Adolfo García-Sastre, Gunjan Shah, Tobias Hohl, Genovefa Papanicolaou, Miguel-Angel Perales, Kent Sepkowitz, Ngolela Esther Babady, and Mini Kamboj
EPI_ISL_583490	Hospital Estadual Sumare	Instituto Adolfo Lutz, Interdiciplinary Procedures Center, Strategic Laboratory	Claudio Tavares Sacchi, Claudia Regina Gonçalves, Erica Valessa Ramos Gomes, Karoline Rodrigues Campos
EPI_ISL_583491	Centro de Saude Esf IV Zona Rual Domingos de SJ Rio Pardo	Instituto Adolfo Lutz, Interdiciplinary Procedures Center, Strategic Laboratory	Claudio Tavares Sacchi, Claudia Regina Gonçalves, Erica Valessa Ramos Gomes, Karoline Rodrigues Campos
EPI_ISL_583492	Santa Casa Anna Cintra	Instituto Adolfo Lutz, Interdiciplinary Procedures Center, Strategic Laboratory	Claudio Tavares Sacchi, Claudia Regina Gonçalves, Erica Valessa Ramos Gomes, Karoline Rodrigues Campos
EPI_ISL_583493	Vigilância em Saúde de Cajamar	Instituto Adolfo Lutz, Interdiciplinary Procedures Center, Strategic Laboratory	Claudio Tavares Sacchi, Claudia Regina Gonçalves, Erica Valessa Ramos Gomes, Karoline Rodrigues Campos

EPI_ISL_583494	CS II Dr. Antonio Vicoso Moreira de Rezende Sumare	Instituto Adolfo Lutz, Interdiciplinary Procedures Center, Strategic Laboratory	Claudio Tavares Sacchi, Claudia Regina Gonçalves, Erica Valessa Ramos Gomes, Karoline Rodrigues Campos
EPI_ISL_584048	Laboratory of Molecular Virology, Department of Biomedical, Surgical and Dental Sciences University of Milano	Laboratory of Molecular Virology, Department of Biomedical, Surgical and Dental Sciences University of Milano	Delbue,S., Modenese,A., Bianchi,M., Fattori,M., D'Alessandro,S., Pariani,E., Basilico,N., Galli,C. and Ferrante,P.
EPI_ISL_584049	Laboratory of Molecular Virology, Department of Biomedical, Surgical and Dental Sciences University of Milano	Laboratory of Molecular Virology, Department of Biomedical, Surgical and Dental Sciences University of Milano	Delbue,S., Modenese,A., Bianchi,M., Fattori,M., D'Alessandro,S.,Pariani,E., Basilico,N., Galli,C. and Ferrante,P.
EPI_ISL_584052	Laboratory of Molecular Virology, Department of Biomedical, Surgical and Dental Sciences University of Milano	Laboratory of Molecular Virology, Department of Biomedical, Surgical and Dental Sciences University of Milano	Delbue,S., D'Alessandro,S., Modenese,A., Signorini,L., Parapini,S.,Dolci,M., Binda,S., Primache,V., Taramelli,D., Incorvaia,B. and Ferrante,P.
EPI_ISL_584073, EPI_ISL_584079	The National Institute of Public Health	State Veterinary Institute Prague	Nagy,A;Jirincova,H;Novakova,L;Trnka,D;Vecerova,J
EPI_ISL_591471, EPI_ISL_591472, EPI_ISL_591473, EPI_ISL_591474, EPI_ISL_591475, EPI_ISL_591476, EPI_ISL_591477, EPI_ISL_591478, EPI_ISL_591479, EPI_ISL_591480, EPI_ISL_591481, EPI_ISL_591482, EPI_ISL_591483			
see above	Pathogen Genomics Center, National Institute of Infectious Diseases	Pathogen Genomics Center, National Institute of Infectious Diseases	Tsuyoshi Sekizuka, Kentaro Itokawa, Rina Tanaka, Masanori Hashino, Hajime Kamiya, Tomoe Shimada, Makoto Kuroda
EPI_ISL_593478, EPI_ISL_593479	Brigham and Women's Hospital	Jonathan Li Laboratory	Manish C. Choudhary, James Regan, Jonathan Z. Li
EPI_ISL_594185, EPI_ISL_594186, EPI_ISL_594187	Department of Pathology, School of Medicine, Imam Khomeini Hospital, Tehran University of Medical Sciences	Genetics Research Center, University of Social Welfare and Rehabilitation Sciences	Zohreh Fattahi, Marzieh Mohseni, Khadijeh Jalalvand, Azam Ghaziasadi, Seyedeh elham Mortazavi, Ali Jafarpour, Azar Hadadi, Alireza Abdollahi, Ali Jafarpour, Azam Ghaziasad, Seyedeh elham Mortazavi, Saber Soltani, Reza Najafipour, Kimia Kahrizi, Seyed Mohammad Jazayeri, Hossein Najmabadi
EPI_ISL_596453	Booali laboratory, Qom, Iran. Department of Virology, School of Public Health, Tehran University of Medical Sciences, Tehran, Iran.	Genetics Research Center, University of Social Welfare and Rehabilitation Sciences	Zohreh Fattahi, Marzieh Mohseni, Khadijeh Jalalvand, Azam Ghaziasadi, Seyedeh elham Mortazavi, Ali Jafarpour, Mohammad Khazeni, Seyed Amir Momeni, Kimia Kahrizi, Seyed Mohammad Jazayeri, Hossein Najmbadi
EPI_ISL_596454	Infectious Disease and Tropical Medicine Research Center, Resistant Tuberculosis Institute, Zahedan University of Medical Sciences, Zahedan, Iran.	Genetics Research Center, University of Social Welfare and Rehabilitation Sciences	Zohreh Fattahi, Marzieh Mohseni, Khadijeh Jalalvand, Azam Ghaziasadi, Seyedeh elham Mortazavi, Ali Jafarpour, Ebrahim Kord, Seyed Mohammad Hashemi-Shahri, Kimia Kahrizi, Seyed Mohammad Jazayeri, Hossein Najmabadi
EPI_ISL_602412	WHO National Influenza Centre Russian Federation	WHO National Influenza Centre Russian Federation	Andrey Komissarov, Artem Fadeev, Kseniya Komissarova, Anna Ivanova, Dmitry Bazhenov, Daria Danilenko
EPI_ISL_605799, EPI_ISL_605800, EPI_ISL_605801, EPI_ISL_605802, EPI_ISL_605803, EPI_ISL_605804, EPI_ISL_605805, EPI_ISL_605806, EPI_ISL_605807, EPI_ISL_605808, EPI_ISL_605809, EPI_ISL_605810, EPI_ISL_605811, EPI_ISL_605812, EPI_ISL_605813, EPI_ISL_605814, EPI_ISL_605815, EPI_ISL_605816			
see above	Clinical Virology Laboratory, Institute of Liver and Biliary Sciences	ILBS - IGIB	Ekta Gupta, Sheetalnath Rooge, Abhishek Padhi, Reshu Agarwal, Jaswinder Singh Maras, Shridhar Sivasubbu, Vinod Scaria, Shvetank Sharma
EPI_ISL_610237, EPI_ISL_610238	Molecular diagnostic laboratory of Federal Budget Institution of Science "Central Research Institute of Epidemiology" of The Federal Service on Customers' Rights Protection and Human Well-being Surveillance	Group of Genomics and Postgenomic Technologies of Central Research Institute of Epidemilology	Samoilov AE, Kaptelova VV, Bukharina AY, Speranskaya AS, Tivanova EV, Shipulina OY, Akimkin VG
EPI_ISL_610249	Virologisches Institut, Universitätsklinikum Erlangen	Virologisches Institut, Universitätsklinikum Erlangen	Armin Ensser, Klaus Korn, Klaus Überla
EPI_ISL_613419, EPI_ISL_613420, EPI_ISL_613421, EPI_ISL_613422, EPI_ISL_613423, EPI_ISL_613424, EPI_ISL_613425, EPI_ISL_613426, EPI_ISL_613429, EPI_ISL_613431, EPI_ISL_613432, EPI_ISL_613433, EPI_ISL_613434, EPI_ISL_613435, EPI_ISL_613436, EPI_ISL_613437, EPI_ISL_613439, EPI_ISL_613440, EPI_ISL_613445, EPI_ISL_613446			
see above	Institut Pasteur de la Guadeloupe	Institut Pasteur de la Guadeloupe	Marion Barbet, Sylvie Behillil, Méline Bizard, Angela Brisebarre, Camille Capel, Etienne Simon-Lorière, Vincent Enouf, Maud Vanpeene, Sylvie van der Werf, Stéphanie Guyomard, Sébastien Breurec, Antoine Talarmin
EPI_ISL_613447, EPI_ISL_613448, EPI_ISL_613449, EPI_ISL_613450, EPI_ISL_613451, EPI_ISL_613452, EPI_ISL_613453, EPI_ISL_613454, EPI_ISL_613455, EPI_ISL_613456	Institut Pasteur de la Guadeloupe	Institut Pasteur de la Guadeloupe	Marion Barbet, Sylvie Behillil, Méline Bizard, Angela Brisebarre, Camille Capel, Etienne Simon-Lorière, Vincent Enouf, Maud Vanpeene, Sylvie van der Werf, Stéphanie Guyomard, Sébastien Breurec, Radjin Steingrover, Cherina Fleming, Antoine Talarmin
EPI_ISL_613560	Laboratorio Biologia Molecolare Sars Cov2 - UOC Laboratorio Analisi - Servizio Medicina di Laboratorio, Ospedale "San Francesco" - ATS-ASSL Nuoro Via Mannironi 1, 08100 Nuoro	Laboratorio specialistico UOC Ematologia - Ospedale "San Francesco" - ATS-ASSL Nuoro Nuoro	Piras Giovanna, Fancello Tatiana, Asproni Rosanna, Fiamma Maura, Monne Maria Itria, Toja Alessandro, Sanna Filomena, Floris Anna Rita, Sulis Vincenzo, Palmas Angelo Domenico, Casu Gavino, Lo Maglio Iana, Mameli Giuseppe
EPI_ISL_613563, EPI_ISL_613564, EPI_ISL_613707, EPI_ISL_613708	Laboratory of Molecular Biology, Blood Center of Ribeirão Preto	Laboratory of Molecular Biology, Blood Center of Ribeirão Preto, Faculty of Medicine of Ribeirão Preto, University of São Paulo	Svetoslav N Slavov, Marta Giovanetti, Vagner Fonseca, Elaine V Santos, Evandra S Rodrigues, Talita Adelino, Joilson Xavier, Glauco de Carvalho Pereira, Aparecida Y Yamamoto, Diego Villa Clé, Rodrigo T Calado; Dimas T Covas, Luiz CJ Alcantara, Simone Kashima
EPI_ISL_613709, EPI_ISL_613951	Laboratory of Molecular Biology, Blood Center of Ribeirão Preto, Faculty of Medicine of Ribeirão Preto, University of São Paulo	Laboratory of Molecular Biology, Blood Center of Ribeirão Preto, Faculty of Medicine of Ribeirão Preto, University of São Paulo	Svetoslav N Slavov, Marta Giovanetti, Vagner Fonseca, Elaine V Santos, Evandra S Rodrigues, Talita Adelino, Joilson Xavier, Glauco de Carvalho Pereira, Aparecida Y Yamamoto, Diego Villa Clé, Rodrigo T Calado; Dimas T Covas, Luiz CJ Alcantara, Simone Kashima
EPI_ISL_613955	Laboratorio Biologia Molecolare Sars Cov2 - UOC Laboratorio Analisi - Servizio Medicina di Laboratorio, Ospedale "San Francesco" - ATS-ASSL Nuoro	Laboratorio specialistico UOC Ematologia - Ospedale "San Francesco" - ATS-ASSL Nuoro	Piras Giovanna, Fancello Tatiana, Asproni Rosanna, Fiamma Maura, Monne Maria Itria, Toja Alessandro, Sanna Filomena, Floris Anna Rita, Sulis Vincenzo, Palmas Angelo Domenico, Casu Gavino, Lo Maglio Iana, Mameli Giuseppe
EPI_ISL_614281	CHRU Pontchaillou - Laboratoire de Virologie 2, rue Henri Le Guilloux	National Reference Center for Viruses of Respiratory Infections, Institut Pasteur, Paris	Marion Barbet, Sylvie Behillil, Méline Bizard, Angela Brisebarre, Camille Capel, Etienne Simon-Lorière, Vincent Enouf, Maud Vanpeene, Sylvie van der Werf, Gisèle Lagathu
EPI_ISL_614396, EPI_ISL_614397, EPI_ISL_614398, EPI_ISL_614889	Laboratorio Biologia Molecolare Sars Cov2 - UOC Laboratorio Analisi - Servizio Medicina di Laboratorio, Ospedale "San Francesco" - ATS-ASSL Nuoro	Laboratorio specialistico UOC Ematologia - Ospedale "San Francesco" - ATS-ASSL Nuoro	Piras Giovanna, Fancello Tatiana, Asproni Rosanna, Fiamma Maura, Monne Maria Itria, Toja Alessandro, Sanna Filomena, Floris Anna Rita, Sulis Vincenzo, Palmas Angelo Domenico, Casu Gavino, Lo Maglio Iana, Mameli Giuseppe
EPI_ISL_623108, EPI_ISL_623110, EPI_ISL_623112, EPI_ISL_623114, EPI_ISL_623116, EPI_ISL_623118, EPI_ISL_623119, EPI_ISL_623120, EPI_ISL_623124, EPI_ISL_623126, EPI_ISL_623129, EPI_ISL_623130, EPI_ISL_623132, EPI_ISL_623134, EPI_ISL_623136, EPI_ISL_623138, EPI_ISL_623140, EPI_ISL_623142, EPI_ISL_623143, EPI_ISL_623144, EPI_ISL_623146, EPI_ISL_623148, EPI_ISL_623149, EPI_ISL_623152, EPI_ISL_623154, EPI_ISL_623157, EPI_ISL_623158, EPI_ISL_623160, EPI_ISL_623161, EPI_ISL_623162, EPI_ISL_623164, EPI_ISL_623165, EPI_ISL_623166, EPI_ISL_623168			
see above	Laboratorio de Virologia Molecular / UFRJ	Bioinformatics Laboratory / LNCC	Carolina M Voloch, Ronaldo S Francisco Jr, Luiz G P de Almeida, Otavio J. Brustolini, Cynthia C Cardoso, Alexandra L Gerber, Ana Paula de C Guimarães, Diana Mariani, Covid19-UFRJ Workgroup, Luís Cristóvão Pôrto, Renato S Aguiar, Terezinha M P P Castiñeiras, Orlando C. Ferreira, Amilcar Tanuri, Ana Tereza R de Vasconcelos
EPI_ISL_625456	Virology Unit, Institut Pasteur de Madagascar	Virology Unit, Institut Pasteur de Madagascar	Christian Ranaivoson, Cara Brook, Norosoa Razanajatovo, Vida Ahyong, Tsiry Randriambolamanantsoa, Michelle Tan, Vololoniaina Raharinosy, Helisoa Razafimanjato, Cristina M. Tato, Joseph L. DeRisi, Soa Fy Andriamandimby, Jean-Michel Heraud, Philippe Dussart
EPI_ISL_626570, EPI_ISL_626593	The National Institute of Public Health	State Veterinary Institute Prague	Nagy,A;Jirincova,H;Novakova,L;Trnka,D;Vecerova,J
EPI_ISL_631504	Wyckoff Heights Medical Center	New York City Public Health Laboratory	Jade Wang, et al.
EPI_ISL_631510, EPI_ISL_631511	New York Presbyterian/ Weill Cornell Medical Center	New York City Public Health Laboratory	Jade Wang, et al.
EPI_ISL_631513	Montefiore Medical Center	New York City Public Health Laboratory	Jade Wang, et al.
EPI_ISL_631538	Maimonides Medical Center	New York City Public Health Laboratory	Jade Wang, et al.

EPI_ISL_631542	New York Presbyterian Lower Manhattan	New York City Public Health Laboratory	Jade Wang, et al.
EPI_ISL_631546	Maimonides Medical Center	New York City Public Health Laboratory	Jade Wang, et al.
EPI_ISL_631554	Jamaica Hospital Medical Center	New York City Public Health Laboratory	Jade Wang, et al.
EPI_ISL_631556	NYC HH Jacobi Medical Center	New York City Public Health Laboratory	Jade Wang, et al.
EPI_ISL_631558	NYU Langone Health	New York City Public Health Laboratory	Jade Wang, et al.
EPI_ISL_631561	Montefiore Medical Center	New York City Public Health Laboratory	Jade Wang, et al.
EPI_ISL_631562	Brookdale University Hospital Medical Center	New York City Public Health Laboratory	Jade Wang, et al.
EPI_ISL_631568	New York Presbyterian Queens	New York City Public Health Laboratory	Jade Wang, et al.
EPI_ISL_631570	OCME Office Of Chief Medical Examiner	New York City Public Health Laboratory	Jade Wang, et al.
EPI_ISL_631573	Brookdale University Hospital Medical Center	New York City Public Health Laboratory	Jade Wang, et al.
EPI_ISL_631574	Jamaica Hospital Medical Center	New York City Public Health Laboratory	Jade Wang, et al.
EPI_ISL_631577, EPI_ISL_631584, EPI_ISL_631587	NYC HH Lincoln Medical And Mental Health Center	New York City Public Health Laboratory	Jade Wang, et al.
EPI_ISL_631591	OCME Office Of Chief Medical Examiner	New York City Public Health Laboratory	Jade Wang, et al.
EPI_ISL_631593	Jamaica Hospital Medical Center	New York City Public Health Laboratory	Jade Wang, et al.
EPI_ISL_631594	OCME Office Of Chief Medical Examiner	New York City Public Health Laboratory	Jade Wang, et al.
EPI_ISL_631595	Jamaica Hospital Medical Center	New York City Public Health Laboratory	Jade Wang, et al.
EPI_ISL_631596	James J. Peters VA Medical Center	New York City Public Health Laboratory	Jade Wang, et al.
EPI_ISL_631597	Jamaica Hospital Medical Center	New York City Public Health Laboratory	Jade Wang, et al.
EPI_ISL_631598	OCME Office Of Chief Medical Examiner	New York City Public Health Laboratory	Jade Wang, et al.
EPI_ISL_631602	Flushing Hospital Medical Center	New York City Public Health Laboratory	Jade Wang, et al.
EPI_ISL_631603	NYC HH Lincoln Medical And Mental Health Center	New York City Public Health Laboratory	Jade Wang, et al.
EPI_ISL_631605	OCME Office Of Chief Medical Examiner	New York City Public Health Laboratory	Jade Wang, et al.
EPI_ISL_631606	Flushing Hospital Medical Center	New York City Public Health Laboratory	Jade Wang, et al.
EPI_ISL_631607	NYC HH Lincoln Medical And Mental Health Center	New York City Public Health Laboratory	Jade Wang, et al.
EPI_ISL_631610, EPI_ISL_631613, EPI_ISL_631615	Jamaica Hospital Medical Center	New York City Public Health Laboratory	Jade Wang, et al.
EPI_ISL_631616	Flushing Hospital Medical Center	New York City Public Health Laboratory	Jade Wang, et al.
EPI_ISL_631617, EPI_ISL_631618, EPI_ISL_631619	OCME Office Of Chief Medical Examiner	New York City Public Health Laboratory	Jade Wang, et al.
EPI_ISL_631624, EPI_ISL_631625, EPI_ISL_631628, EPI_ISL_631629, EPI_ISL_631633	Richmond University Medical Center	New York City Public Health Laboratory	Jade Wang, et al.
EPI_ISL_631637	Jamaica Hospital Medical Center	New York City Public Health Laboratory	Jade Wang, et al.
EPI_ISL_631686	NYC HH Lincoln Medical And Mental Health Center	New York City Public Health Laboratory	Jade Wang, et al.
EPI_ISL_631687	OCME Office Of Chief Medical Examiner	New York City Public Health Laboratory	Jade Wang, et al.
EPI_ISL_631689, EPI_ISL_631690	Lincoln Medical Center	New York City Public Health Laboratory	Jade Wang, et al.
EPI_ISL_631694, EPI_ISL_631695	OCME Office Of Chief Medical Examiner	New York City Public Health Laboratory	Jade Wang, et al.
EPI_ISL_631697, EPI_ISL_631698	Jamaica Hospital Medical Center	New York City Public Health Laboratory	Jade Wang, et al.
EPI_ISL_631701	Flushing Hospital Medical Center	New York City Public Health Laboratory	Jade Wang, et al.
EPI_ISL_631703, EPI_ISL_631704, EPI_ISL_631705, EPI_ISL_631706	Jamaica Hospital Medical Center	New York City Public Health Laboratory	Jade Wang, et al.
EPI_ISL_631710	Flushing Hospital Medical Center	New York City Public Health Laboratory	Jade Wang, et al.
EPI_ISL_631711, EPI_ISL_631712, EPI_ISL_631716, EPI_ISL_631719, EPI_ISL_631720	Jamaica Hospital Medical Center	New York City Public Health Laboratory	Jade Wang, et al.
EPI_ISL_631724	Flushing Hospital Medical Center	New York City Public Health Laboratory	Jade Wang, et al.
EPI_ISL_631726, EPI_ISL_631728	Jamaica Hospital Medical Center	New York City Public Health Laboratory	Jade Wang, et al.
EPI_ISL_631732	Flushing Hospital Medical Center	New York City Public Health Laboratory	Jade Wang, et al.
EPI_ISL_631733, EPI_ISL_631735, EPI_ISL_631736, EPI_ISL_631740	Jamaica Hospital Medical Center	New York City Public Health Laboratory	Jade Wang, et al.
EPI_ISL_631754	NYC HH Lincoln Medical And Mental Health Center	New York City Public Health Laboratory	Jade Wang, et al.
EPI_ISL_631755, EPI_ISL_631757	Richmond University Medical Center	New York City Public Health Laboratory	Jade Wang, et al.
EPI_ISL_631758, EPI_ISL_631759, EPI_ISL_631760, EPI_ISL_631761, EPI_ISL_631762	OCME Office Of Chief Medical Examiner	New York City Public Health Laboratory	Jade Wang, et al.
EPI_ISL_631767, EPI_ISL_631768	Jamaica Hospital Medical Center	New York City Public Health Laboratory	Jade Wang, et al.
EPI_ISL_631769, EPI_ISL_631770	Flushing Hospital Medical Center	New York City Public Health Laboratory	Jade Wang, et al.
EPI_ISL_631772	Jamaica Hospital Medical Center	New York City Public Health Laboratory	Jade Wang, et al.
EPI_ISL_631773, EPI_ISL_631774	Flushing Hospital Medical Center	New York City Public Health Laboratory	Jade Wang, et al.
EPI_ISL_631775, EPI_ISL_631776, EPI_ISL_631777	Jamaica Hospital Medical Center	New York City Public Health Laboratory	Jade Wang, et al.

EPI_ISL_631778, EPI_ISL_631781, EPI_ISL_631782	St Barnabas Hospital	New York City Public Health Laboratory	Jade Wang, et al.
EPI_ISL_631797	OCME Office Of Chief Medical Examiner	New York City Public Health Laboratory	Jade Wang, et al.
EPI_ISL_631801	Richmond University Medical Center	New York City Public Health Laboratory	Jade Wang, et al.
EPI_ISL_631803, EPI_ISL_631806	Brookdale University Hospital Medical Center	New York City Public Health Laboratory	Jade Wang, et al.
EPI_ISL_631808, EPI_ISL_631811	Wyckoff Heights Medical Center	New York City Public Health Laboratory	Jade Wang, et al.
EPI_ISL_631812, EPI_ISL_631813	Jamaica Hospital Medical Center	New York City Public Health Laboratory	Jade Wang, et al.
EPI_ISL_631815	Richmond University Medical Center	New York City Public Health Laboratory	Jade Wang, et al.
EPI_ISL_631829	Brookdale University Hospital Medical Center	New York City Public Health Laboratory	Jade Wang, et al.
EPI_ISL_631831	VA NY Harbor Healthcare System	New York City Public Health Laboratory	Jade Wang, et al.
EPI_ISL_631835, EPI_ISL_631836	Jamaica Hospital Medical Center	New York City Public Health Laboratory	Jade Wang, et al.
EPI_ISL_631840, EPI_ISL_631841, EPI_ISL_631845	OCME Office Of Chief Medical Examiner	New York City Public Health Laboratory	Jade Wang, et al.
EPI_ISL_631866, EPI_ISL_631867	Jamaica Hospital Medical Center	New York City Public Health Laboratory	Jade Wang, et al.
EPI_ISL_632313	NU-sjukvården	Clinical Microbiology, Sahlgrenska University Hospital,	Johan Ringlander, Josefin Olausson, Hedvig Engström Jakobsson, Magnus Lindh
EPI_ISL_636476, EPI_ISL_636477, EPI_ISL_636485	CNR Virus des Infections Respiratoires - France SUD	CNR Virus des Infections Respiratoires - France SUD	Antonin Bal, Gregory Destras, Gwendolyne Burfin, Hadrien Règue, Alexandre Gaymard, Maude Bouscambert-Duchamp, Florence Morfin-Sherpa, Martine Valette, Bruno Lina, Laurence Josset
EPI_ISL_636834	Laboratório de Imunofarmacologia - Instituto Oswaldo Cruz	Laboratório de Imunofarmacologia - Instituto Oswaldo Cruz	Souza,T.M., Fintelman-Rodrigues,N., De Paula,A.D., Saraiva,F.B., Ferreira,M.A. and Sacramento,C.Q.
EPI_ISL_636964	Pathogen Genomics Lab King Abdullah University of Science and Technology(KAUST)	Pathogen Genomics Lab King Abdullah University of Science and Technology(KAUST)	Rahul P Salunke, Sharif Hala, Raeece Naeem, Sara Mfarrej, Amit Kumar Subudhi, Amanda Ooi, Luke Esau, Fadwa Alofi, Fathia Ben Rached, Afrah Alsomali, Asim Khogeer, Ahmad Bakur Mahmoud, Anwar Hashem, Naif Almontashiri, Arnab Pain
EPI_ISL_636969, EPI_ISL_636970, EPI_ISL_636971	Etlik Veterinary Control Central Research Institute	Etlik Veterinary Control Central Research Institute	Sabri Hacioglu, Ahu Pakdemirli, Dilek Dulger, Erdem Danyer, Ummu Sena Sari, Cevdet Yarali, Ozcan Yildirim
EPI_ISL_637108, EPI_ISL_637109	Laboratorio Biologia Molecolare Sars Cov2 - UOC Laboratorio Analisi - Servizio Medicina di Laboratorio, Ospedale "San Francesco" - ATS-ASSL Nuoro	Laboratorio specialistico UOC Ematologia - Ospedale "San Francesco" - ATS-ASSL Nuoro	Piras Giovanna, Fancello Tatiana, Asproni Rosanna, Fiamma Maura, Monne Maria Itria, Toja Alessandro, Sanna Filomena, Floris Anna Rita, Sulis Vincenzo, Palmas Angelo Domenico, Casu Gavino, Lo Maglio Iana, Mameli Giuseppe
EPI_ISL_648043, EPI_ISL_648044, EPI_ISL_648045, EPI_ISL_648046, EPI_ISL_648047, EPI_ISL_648048, EPI_ISL_648049, EPI_ISL_648050, EPI_ISL_648051, EPI_ISL_648052, EPI_ISL_648053, EPI_ISL_648054, EPI_ISL_648055, EPI_ISL_648056, EPI_ISL_648057, EPI_ISL_648058, EPI_ISL_648059, EPI_ISL_648060, EPI_ISL_648061, EPI_ISL_648062, EPI_ISL_648063, EPI_ISL_648064, EPI_ISL_648065, EPI_ISL_648066, EPI_ISL_648067, EPI_ISL_648068, EPI_ISL_648069, EPI_ISL_648070, EPI_ISL_648071, EPI_ISL_648072, EPI_ISL_648073, EPI_ISL_648074, EPI_ISL_648075, EPI_ISL_648076, EPI_ISL_648077, EPI_ISL_648078, EPI_ISL_648079, EPI_ISL_648080, EPI_ISL_648081, EPI_ISL_648082, EPI_ISL_648083, EPI_ISL_648084, EPI_ISL_648085, EPI_ISL_648086, EPI_ISL_648087, EPI_ISL_648088, EPI_ISL_648089, EPI_ISL_648090, EPI_ISL_648091, EPI_ISL_648092, EPI_ISL_648093, EPI_ISL_648094, EPI_ISL_648095, EPI_ISL_648096, EPI_ISL_648097, EPI_ISL_648098, EPI_ISL_648099, EPI_ISL_648100, EPI_ISL_648101, EPI_ISL_648102, EPI_ISL_648103, EPI_ISL_648104, EPI_ISL_648105, EPI_ISL_648106, EPI_ISL_648107, EPI_ISL_648108, EPI_ISL_648109, EPI_ISL_648110, EPI_ISL_648111, EPI_ISL_648112, EPI_ISL_648113, EPI_ISL_648114, EPI_ISL_648115, EPI_ISL_648116, EPI_ISL_648117, EPI_ISL_648118, EPI_ISL_648119, EPI_ISL_648120, EPI_ISL_648121			
see above	Department of Laboratory Medicine, Tan Tock Seng Hospital	Department of Laboratory Medicine, Tan Tock Seng Hospital	Chen YYC, Zair X, Lim JX, Li C, Tang WY, Maurer-Stroh S, Barkham TMS, Nagarajan N, Sessions OM
EPI_ISL_648265, EPI_ISL_648267, EPI_ISL_648270	Brigham and Women's Hospital	Jonathan Li laboratory	Jonathan Z. Li, Manish C. Choudhary, Upasana D. Adhikari, George Eng, Douglas S Kwon
EPI_ISL_648610, EPI_ISL_648611, EPI_ISL_648612, EPI_ISL_648613, EPI_ISL_648614, EPI_ISL_648615, EPI_ISL_648616, EPI_ISL_648617, EPI_ISL_648618, EPI_ISL_648619, EPI_ISL_648620, EPI_ISL_648621, EPI_ISL_648622, EPI_ISL_648623, EPI_ISL_648624, EPI_ISL_648625, EPI_ISL_648626, EPI_ISL_648627, EPI_ISL_648628, EPI_ISL_648629, EPI_ISL_648630, EPI_ISL_648631, EPI_ISL_648632, EPI_ISL_648633, EPI_ISL_648634, EPI_ISL_648635, EPI_ISL_648636, EPI_ISL_648637, EPI_ISL_648638, EPI_ISL_648639, EPI_ISL_648640, EPI_ISL_648641, EPI_ISL_648642, EPI_ISL_648643, EPI_ISL_648644, EPI_ISL_648645, EPI_ISL_648646, EPI_ISL_648647, EPI_ISL_648648, EPI_ISL_648649, EPI_ISL_648650, EPI_ISL_648651, EPI_ISL_648652, EPI_ISL_648653, EPI_ISL_648654, EPI_ISL_648655, EPI_ISL_648656, EPI_ISL_648657, EPI_ISL_648658, EPI_ISL_648659, EPI_ISL_648660, EPI_ISL_648661, EPI_ISL_648662, EPI_ISL_648663, EPI_ISL_648664, EPI_ISL_648665, EPI_ISL_648666, EPI_ISL_648667, EPI_ISL_648668, EPI_ISL_648669, EPI_ISL_648670, EPI_ISL_648671, EPI_ISL_648672, EPI_ISL_648673, EPI_ISL_648674, EPI_ISL_648675, EPI_ISL_648676, EPI_ISL_648742, EPI_ISL_648743, EPI_ISL_648744, EPI_ISL_648745, EPI_ISL_648746, EPI_ISL_648747, EPI_ISL_648748, EPI_ISL_648749, EPI_ISL_648750, EPI_ISL_648751, EPI_ISL_648752, EPI_ISL_648753, EPI_ISL_648754, EPI_ISL_648755, EPI_ISL_648756, EPI_ISL_648757, EPI_ISL_648758, EPI_ISL_648759, EPI_ISL_648760, EPI_ISL_648761, EPI_ISL_648762, EPI_ISL_648763, EPI_ISL_648764, EPI_ISL_648765, EPI_ISL_648766, EPI_ISL_648767, EPI_ISL_648768, EPI_ISL_648769, EPI_ISL_648770, EPI_ISL_648771, EPI_ISL_648772, EPI_ISL_648773, EPI_ISL_648774, EPI_ISL_648775, EPI_ISL_648776, EPI_ISL_648777, EPI_ISL_648778, EPI_ISL_648779, EPI_ISL_648780, EPI_ISL_648781, EPI_ISL_648782, EPI_ISL_648783, EPI_ISL_648784, EPI_ISL_648785, EPI_ISL_648786, EPI_ISL_648787, EPI_ISL_648788, EPI_ISL_648789, EPI_ISL_648790, EPI_ISL_648791			
see above	Department of Laboratory Medicine, Tan Tock Seng Hospital	Department of Laboratory Medicine, Tan Tock Seng Hospital	Chen YYC, Zair X, Lim JX, Li C, Tang WY, Maurer-Stroh S, Barkham TMS, Nagarajan N, Sessions OM
EPI_ISL_649189	Istituto Zooprofilattico Sperimentale della Puglia e della Basilicata	Istituto Zooprofilattico Sperimentale della Puglia e della Basilicata	Parisi A., Bianco A., Capozzi L., Del Sambro L., Manzulli V, Rondinone V., Pace L., Galante D., Cipolletta D.
EPI_ISL_653813	Istituto Zooprofilattico Sperimentale della Puglia e della Basilicata	Istituto Zooprofilattico Sperimentale della Puglia e della Basilicata	Parisi A., Bianco A., Capozzi L., Del Sambro L., Manzulli V, Rondinone V., Pace L., Cipolletta D., Galante D.
EPI_ISL_653814, EPI_ISL_653815, EPI_ISL_653816, EPI_ISL_653817, EPI_ISL_653818	I.R.C.C.S. "S. De Bellis" - Ente Ospedaliero	Istituto Zooprofilattico Sperimentale della Puglia e della Basilicata	Parisi A., Bianco A., Capozzi L., Del Sambro L., Lippolis A., Notarnicola M., Cipolletta D., Galante D.
EPI_ISL_654016	Laboratory of Microbiology, National Reference Lab, Charles Nicolle Hospital; 2-University of Tunis ElManar, Faculty of Medicine of Tunis, LR99ES09, Tunis, Tunisia	1-Clinical and Experimental Pharmacology Lab, LR16SP02, National Center of Pharmacovigilance, University of Tunis El Manar, Tunis, Tunisia. 2-Neurodegenerative diseases and psychiatric troubles, LR18SP03, Razi Hospital, University of Tunis El Manar, Tunis, Tunisia. 3- Ministry of Health, National Observatory of New and Emerging Diseases, 1006, Tunis, Tunisia	Ilhem Boutiba-Ben Boubaker, Sameh Trabelsi, Nissaf Ben Alaya, Maher Kharrat, Alia Ben Kahla, Jalila Ben Khelil, Salma Abid, Sana Ferjani, Mouna Ben Sassi, Mouna Safer, Imen Mkada, Imen Kacem, Gaies Emna, Soumaya Rammeh, Riadh Daghfous, Riadh Gouider.
EPI_ISL_654867	Pasteur Institute in Ho Chi Minh city	Department of Microbiology and Immunology - Pasteur Institute in Ho Chi Minh city	Hoàng Minh, Cao Minh Thng, Phm Th Thu Hng, V Phm Hng Nhung, ào Huy Mnh, Hunh Th Kim Loan, Nguyn Hoàng Quân, Hunh Phng Tho, Hoàng Nh ào, Nguyn Trung Hiu, Nguyn Hoàng Anh, Nguyn Thu Ngc, Phm Th Nhung, La Hoàng Huy, ng Thanh Giang, Nguyn Th Thanh Thng, Trn Th Hng Kim, Nguyn Thanh Long, Phm Duy Quang, Lng Chn Quang, Hoàng Quc Cng, Phan Trng Lân, Nguyn V Thng
EPI_ISL_654868	Pasteur Institute in Ho Chi Minh city	Department of Microbiology and Immunology - Pasteur Institute in Ho Chi Minh city	ào Huy Mnh, Phm Th Thu Hng, V Phm Hng Nhung, Cao Minh Thng, Hunh Th Kim Loan, Nguyn Hoàng Quân, Hunh Phng Tho, Hoàng Nh ào, Nguyn Trung Hiu, Nguyn Hoàng Anh, Nguyn Thu Ngc, Phm Th Nhung, Nguyn Th Ngc Tho, Hà Bích Ngc, Nguyn Th Thanh Thng, Hoàng Minh, Trn Th Hng Kim, Nguyn Thanh Long, Phm Duy Quang, Lng Chn Quang, Hoàng Quc Cng, Nguyn V Thng, Phan Trng Lân
EPI_ISL_654869	Pasteur Institute in Ho Chi Minh city	Department of Microbiology and Immunology - Pasteur Institute in Ho Chi Minh city	Phm Th Nhung, ào Huy Mnh, Phm Th Thu Hng, V Phm Hng Nhung, Cao Minh Thng, Hunh Th Kim Loan, Nguyn Hoàng Quân, Hunh Phng Tho, Hoàng Nh ào, Nguyn Trung Hiu, Nguyn Hoàng Anh, Nguyn Thu Ngc, Nguyn Th Ngc Tho, ng Thanh Giang, Võ Th Trang ài, Hoàng Minh, Trn Th Hng Kim, Nguyn Thanh Long, Phm Duy Quang, Lng Chn Quang, Hoàng Quc Cng, Phan Trng Lân, Nguyn V Thng
EPI_ISL_654870	Pasteur Institute in Ho Chi Minh city	Department of Microbiology and Immunology - Pasteur Institute in Ho Chi Minh city	Trn Th Hng Kim, Phm Th Thu Hng, ào Huy Mnh, V Phm Hng Nhung, Cao Minh Thng, Hunh Th Kim Loan, Nguyn Hoàng Quân, Hunh Phng Tho, Hoàng Nh ào, Nguyn Trung Hiu, Nguyn Hoàng Anh, Nguyn Thu Ngc, Phm Th Nhung, Nguyn Th Ngc Tho, ng Thanh Giang, Nguyn Th Thanh Thng, Hoàng Kim Loan, Nguyn Thanh Long, Phm Duy Quang, Lng Chn Quang, Hoàng Quc Cng, Nguyn V Thng, Phan Trng Lân
EPI_ISL_654872	Pasteur Institute in Ho Chi Minh city	Department of Microbiology and Immunology - Pasteur Institute in Ho Chi Minh city	Phm Th Thu Hng, ào Huy Mnh, V Phm Hng Nhung, Cao Minh Thng, Hunh Th Kim Loan, Nguyn Hoàng Quân, Hunh Phng Tho, Hoàng Nh ào, Nguyn Trung Hiu, Nguyn Hoàng Anh, Nguyn Thu Ngc, Phm Th Nhung, Nguyn Th Ngc Tho, ng Thanh Giang, Nguyn Th Thanh Thng, Hoàng Minh, Nguyn Hoàng V, Nguyn Thanh Long, Phm Duy Quang, Lng Chn Quang, Hoàng Quc Cng, Phan Trng Lân, Nguyn V Thng

EPI_ISL_654873	Pasteur Institute in Ho Chi Minh city	Department of Microbiology and Immunology - Pasteur Institute in Ho Chi Minh city	Phm Th Thu Hng, ào Huy Mnh, V Phm Hng Nhung, Cao Minh Thng, Hunh Th Kim Loan, Nguyn Hoàng Quân, Hunh Phng Tho, Hoàng Nh ào, Nguyn Trung Hiu, Nguyn Th Thanh Tho, Nguyn Thu Ngc, Phm Th Nhung, Nguyn Th Ngc Tho, ng Thanh Giang, Nguyn Th Thanh Thng, Hoàng Minh, Trn Th Hng Kim, Nguyn Thanh Long, Phm Duy Quang, Lng Chn Quang, Hoàng Quc Cng, Nguyn V Thng, Phan Trng Lân
EPI_ISL_654874	Pasteur Institute in Ho Chi Minh city	Department of Microbiology and Immunology - Pasteur Institute in Ho Chi Minh city	Nguyn Th Ngc Tho, V Phm Hng Nhung, ào Huy Mnh, Phm Th Thu Hng, Cao Minh Thng, Hunh Th Kim Loan, Nguyn Hoàng Quân, Hunh Phng Tho, Hoàng Nh ào, Trn Tôn, Nguyn Hoàng Anh, Nguyn Thu Ngc, Phm Th Nhung, ng Thanh Giang, Nguyn Th Thanh Thng, Hoàng Minh, Trn Th Hng Kim, Nguyn Thanh Long, Phm Duy Quang, Lng Chn Quang, Hoàng Quc Cng, Phan Trng Lân, Nguyn V Thng
EPI_ISL_654875	Pasteur Institute in Ho Chi Minh city	Department of Microbiology and Immunology - Pasteur Institute in Ho Chi Minh city	V Phm Hng Nhung, ào Huy Mnh, Phm Th Thu Hng, Cao Minh Thng, Hunh Th Kim Loan, Nguyn Hoàng Quân, Hunh Phng Tho, Hoàng Nh ào, Nguyn Trung Hiu, Nguyn Hoàng Anh, Nguyn Vit Thinh, Phm Th Nhung, Nguyn Th Ngc Tho, ng Thanh Giang, Nguyn Th Thanh Thng, Hoàng Minh, Trn Th Hng Kim, Nguyn Thanh Long, Phm Duy Quang, Lng Chn Quang, Hoàng Quc Cng, Nguyn V Thng, Phan Trng Lân
EPI_ISL_654876	Pasteur Institute in Ho Chi Minh city	Department of Microbiology and Immunology - Pasteur Institute in Ho Chi Minh city	V Phm Hng Nhung, ào Huy Mnh, Phm Th Thu Hng, Cao Minh Thng, Hunh Th Kim Loan, Nguyn Hoàng Quân, Hunh Phng Tho, Hoàng Nh ào, Nguyn Trung Hiu, Nguyn Hoàng Anh, Nguyn Thu Ngc, Trng Công Hiu, Nguyn Th Ngc Tho, ng Thanh Giang, Nguyn Th Thanh Thng, Hoàng Minh, Trn Th Hng Kim, Nguyn Thanh Long, Phm Duy Quang, Lng Chn Quang, Hoàng Quc Cng, Phan Trng Lân, Nguyn V Thng
EPI_ISL_654877	Pasteur Institute in Ho Chi Minh city	Department of Microbiology and Immunology - Pasteur Institute in Ho Chi Minh city	ng Thanh Giang, Phm Duy Quang, ào Huy Mnh, Phm Th Thu Hng, V Phm Hng Nhung, Cao Minh Thng, Hunh Th Kim Loan, Nguyn Hoàng Quân, Hunh Phng Tho, Hoàng Nh ào, Nguyn Trung Hiu, Nguyn Hoàng Anh, Nguyn Thu Ngc, Phm Th Nhung, Trng Thùy Dung, Nguyn Th Thanh Thng, Hoàng Minh, Trn Th Hng Kim, Nguyn Thanh Long, Lng Chn Quang, Hoàng Quc Cng, Nguyn V Thng, Phan Trng Lân
EPI_ISL_654878	Pasteur Institute in Ho Chi Minh city	Department of Microbiology and Immunology - Pasteur Institute in Ho Chi Minh city	Phm Duy Quang, ào Huy Mnh, Phm Th Thu Hng, V Phm Hng Nhung, Cao Minh Thng, Hunh Th Kim Loan, Nguyn Hoàng Quân, Hunh Phng Tho, Hoàng Nh ào, Nguyn Trung Hiu, Nguyn Hoàng Anh, Nguyn Thu Ngc, Phm Th Nhung, Nguyn Th Ngc Tho, Viên ng Khánh Linh, Nguyn Th Thanh Thng, Hoàng Minh, Trn Th Hng Kim, Nguyn Thanh Long, Lng Chn Quang, Hoàng Quc Cng, Phan Trng Lân, Nguyn V Thng
EPI_ISL_654881	Pasteur Institute in Ho Chi Minh city	Department of Microbiology and Immunology - Pasteur Institute in Ho Chi Minh city	Lng Chn Quang, ào Huy Mnh, Phm Th Thu Hng, V Phm Hng Nhung, Cao Minh Thng, Hunh Th Kim Loan, Nguyn Hoàng Quân, Hunh Phng Tho, Hoàng Nh ào, Nguyn Trung Hiu, Nguyn Hoàng Anh, Nguyn Thu Ngc, Phm Th Nhung, Nguyn Th Ngc Tho, ng Thanh Giang, Nguyn Th Thanh Thng, Hoàng Minh, Nguyn Th Phng Thúy, Nguyn Thanh Long, Phm Duy Quang, Hoàng Quc Cng, Nguyn V Thng, Phan Trng Lân
EPI_ISL_654882	Pasteur Institute in Ho Chi Minh city	Department of Microbiology and Immunology - Pasteur Institute in Ho Chi Minh city	Lng Chn Quang, ào Huy Mnh, Phm Th Thu Hng, V Phm Hng Nhung, Cao Minh Thng, Hunh Th Kim Loan, Nguyn Hoàng Quân, Hunh Phng Tho, Hoàng Nh ào, Nguyn Trung Hiu, Nguyn Hoàng Anh, Nguyn Ngc Anh Tun, Phm Th Nhung, Nguyn Th Ngc Tho, ng Thanh Giang, Nguyn Th Thanh Thng, Hoàng Minh, Trn Th Hng Kim, Nguyn Thanh Long, Phm Duy Quang, Hoàng Quc Cng, Phan Trng Lân, Nguyn V Thng
EPI_ISL_658904	Instituto de Diagnostico y Referencia Epidemiologicos (INDRE)	Instituto de Diagnostico y Referencia Epidemiologicos (INDRE)	Ernesto Ramirez-Gonzalez, Abril Rodriguez-Maldonado, Claudia Wong-Arambula , Natividad Cruz-Ortiz, Tatiana Nunez-Garcia, Dayanira Arellano-Suarez, Fabiola Garces-Ayala, Lucia Hernandez-Rivas, Irma Lopez-Martinez, Gisela Barrera-Badillo, Marisol Galindo-Galindo, Linda Andarade-Sanchez, Octavio Ruiz-Muñiz, Pamela Ramirez-Medina, Roman Canul-Aguilar, Gaudalupe Herrera-Ramirez, Jazmin Galicia-Hernandez.
EPI_ISL_666870	Laboratoire de Virologie, CHU de Caen, Normandie, France.	GRAM2.0, Université de Caen Normandie	Meriadeg LE GOUIL, Januel VOUTEAU, Juliette ECHE, Estelle LEPERCHOIS, Astrid VABRET
EPI_ISL_677673	Wadsworth Center, New York State Department.of Health	Wadsworth Center, New York State Department.of Health	Kirsten St. George, Daryl M. Lamson, Alexis Russel, Jonathan Plitnick, Navjot Singh, John Kelly, Sara Griesemer, Erasmus Schneider, Erica Lasek-Nesselquist
EPI_ISL_677678, EPI_ISL_677679, EPI_ISL_677680, EPI_ISL_677681, EPI_ISL_677682, EPI_ISL_677683, EPI_ISL_677684, EPI_ISL_677685, EPI_ISL_677686, EPI_ISL_677687, EPI_ISL_677688, EPI_ISL_677689			
see above	Vanda Pharmaceuticals Clinical Site	Vanda Pharmaceuticals	Vanda Pharmaceuticals
EPI_ISL_681702, EPI_ISL_681703	Zurita & Zurita Laboratorios	Zurita & Zurita Laboratorios	Gabriela Sevillano, Camilo Zurita-Salinas, Jeannete Zurita
EPI_ISL_684110, EPI_ISL_684111, EPI_ISL_684113, EPI_ISL_684114, EPI_ISL_684115, EPI_ISL_684116, EPI_ISL_684118, EPI_ISL_684119, EPI_ISL_684120, EPI_ISL_684121, EPI_ISL_684122, EPI_ISL_684123, EPI_ISL_684124, EPI_ISL_684125, EPI_ISL_684126, EPI_ISL_684128, EPI_ISL_684129, EPI_ISL_684130, EPI_ISL_684132, EPI_ISL_684133			
see above	Pathogen Genomics Center, National Institute of Infectious Diseases	Pathogen Genomics Center, National Institute of Infectious Diseases	Tsuyoshi Sekizuka, Kentaro Itokawa, Rina Tanaka, Masanori Hashino, Makoto Kuroda
EPI_ISL_684136	Tokyo Metropolitan Institute of Public Health	Pathogen Genomics Center, National Institute of Infectious Diseases	Tsuyoshi Sekizuka, Kentaro Itokawa, Rina Tanaka, Masanori Hashino, Makoto Kuroda
EPI_ISL_684139, EPI_ISL_684142, EPI_ISL_684143, EPI_ISL_684145, EPI_ISL_684146	Pathogen Genomics Center, National Institute of Infectious Diseases	Pathogen Genomics Center, National Institute of Infectious Diseases	Tsuyoshi Sekizuka, Kentaro Itokawa, Rina Tanaka, Masanori Hashino, Makoto Kuroda
EPI_ISL_684149	Saitama Prefectural Institute of Public Health	Pathogen Genomics Center, National Institute of Infectious Diseases	Tsuyoshi Sekizuka, Kentaro Itokawa, Rina Tanaka, Masanori Hashino, Makoto Kuroda
EPI_ISL_684150, EPI_ISL_684151, EPI_ISL_684152, EPI_ISL_684153	Pathogen Genomics Center, National Institute of Infectious Diseases	Pathogen Genomics Center, National Institute of Infectious Diseases	Tsuyoshi Sekizuka, Kentaro Itokawa, Rina Tanaka, Masanori Hashino, Makoto Kuroda
EPI_ISL_684154, EPI_ISL_684155	Tokyo Metropolitan Institute of Public Health	Pathogen Genomics Center, National Institute of Infectious Diseases	Tsuyoshi Sekizuka, Kentaro Itokawa, Rina Tanaka, Masanori Hashino, Makoto Kuroda
EPI_ISL_684156, EPI_ISL_684157, EPI_ISL_684158	Pathogen Genomics Center, National Institute of Infectious Diseases	Pathogen Genomics Center, National Institute of Infectious Diseases	Tsuyoshi Sekizuka, Kentaro Itokawa, Rina Tanaka, Masanori Hashino, Makoto Kuroda
EPI_ISL_684159, EPI_ISL_684160, EPI_ISL_684161	Chiba Prefectural Institute of Public Health	Pathogen Genomics Center, National Institute of Infectious Diseases	Tsuyoshi Sekizuka, Kentaro Itokawa, Rina Tanaka, Masanori Hashino, Makoto Kuroda
EPI_ISL_684162, EPI_ISL_684163, EPI_ISL_684164, EPI_ISL_684166, EPI_ISL_684167, EPI_ISL_684168, EPI_ISL_684170, EPI_ISL_684171, EPI_ISL_684172, EPI_ISL_684173, EPI_ISL_684174, EPI_ISL_684175, EPI_ISL_684176, EPI_ISL_684177, EPI_ISL_684178, EPI_ISL_684179, EPI_ISL_684180, EPI_ISL_684181, EPI_ISL_684182, EPI_ISL_684183, EPI_ISL_684184, EPI_ISL_684185, EPI_ISL_684186, EPI_ISL_684187, EPI_ISL_684188, EPI_ISL_684189, EPI_ISL_684190, EPI_ISL_684191, EPI_ISL_684192, EPI_ISL_684193, EPI_ISL_684194, EPI_ISL_684195, EPI_ISL_684196, EPI_ISL_684197, EPI_ISL_684198, EPI_ISL_684199, EPI_ISL_684200, EPI_ISL_684201, EPI_ISL_684202, EPI_ISL_684203, EPI_ISL_684204, EPI_ISL_684205, EPI_ISL_684206, EPI_ISL_684207, EPI_ISL_684208, EPI_ISL_684209, EPI_ISL_684210, EPI_ISL_684211, EPI_ISL_684212, EPI_ISL_684213, EPI_ISL_684214, EPI_ISL_684215, EPI_ISL_684216, EPI_ISL_684217, EPI_ISL_684218, EPI_ISL_684219			
see above	Pathogen Genomics Center, National Institute of Infectious Diseases	Pathogen Genomics Center, National Institute of Infectious Diseases	Tsuyoshi Sekizuka, Kentaro Itokawa, Rina Tanaka, Masanori Hashino, Makoto Kuroda
EPI_ISL_684220, EPI_ISL_684221, EPI_ISL_684222, EPI_ISL_684223, EPI_ISL_684224, EPI_ISL_684225, EPI_ISL_684226, EPI_ISL_684227, EPI_ISL_684228, EPI_ISL_684229, EPI_ISL_684230, EPI_ISL_684231, EPI_ISL_684232, EPI_ISL_684233, EPI_ISL_684234, EPI_ISL_684235, EPI_ISL_684236, EPI_ISL_684237, EPI_ISL_684238, EPI_ISL_684239, EPI_ISL_684240, EPI_ISL_684241, EPI_ISL_684242, EPI_ISL_684243, EPI_ISL_684244, EPI_ISL_684245, EPI_ISL_684246, EPI_ISL_684247, EPI_ISL_684248, EPI_ISL_684249, EPI_ISL_684250, EPI_ISL_684251, EPI_ISL_684252, EPI_ISL_684253, EPI_ISL_684254, EPI_ISL_684255, EPI_ISL_684256, EPI_ISL_684257, EPI_ISL_684258, EPI_ISL_684259, EPI_ISL_684260, EPI_ISL_684261, EPI_ISL_684262, EPI_ISL_684263, EPI_ISL_684264, EPI_ISL_684265, EPI_ISL_684266, EPI_ISL_684267, EPI_ISL_684268, EPI_ISL_684269, EPI_ISL_684270, EPI_ISL_684271, EPI_ISL_684272, EPI_ISL_684273, EPI_ISL_684274, EPI_ISL_684275, EPI_ISL_684276, EPI_ISL_684277, EPI_ISL_684278, EPI_ISL_684279, EPI_ISL_684280, EPI_ISL_684281, EPI_ISL_684282, EPI_ISL_684283, EPI_ISL_684284, EPI_ISL_684285, EPI_ISL_684286, EPI_ISL_684287, EPI_ISL_684288			
see above	Gunma Prefectural Institute of Public Health and Environmental Sciences	Pathogen Genomics Center, National Institute of Infectious Diseases	Tsuyoshi Sekizuka, Kentaro Itokawa, Rina Tanaka, Masanori Hashino, Makoto Kuroda
EPI_ISL_684289, EPI_ISL_684290, EPI_ISL_684291, EPI_ISL_684292, EPI_ISL_684293, EPI_ISL_684294, EPI_ISL_684295, EPI_ISL_684296, EPI_ISL_684297, EPI_ISL_684298, EPI_ISL_684299, EPI_ISL_684300, EPI_ISL_684301, EPI_ISL_684302, EPI_ISL_684303, EPI_ISL_684304, EPI_ISL_684305, EPI_ISL_684306, EPI_ISL_684307, EPI_ISL_684308, EPI_ISL_684309, EPI_ISL_684310, EPI_ISL_684311, EPI_ISL_684312, EPI_ISL_684315, EPI_ISL_684316, EPI_ISL_684317, EPI_ISL_684319, EPI_ISL_684320			
see above	Pathogen Genomics Center, National Institute of Infectious Diseases	Pathogen Genomics Center, National Institute of Infectious Diseases	Tsuyoshi Sekizuka, Kentaro Itokawa, Rina Tanaka, Masanori Hashino, Makoto Kuroda
EPI_ISL_684325, EPI_ISL_684326, EPI_ISL_684327, EPI_ISL_684328, EPI_ISL_684329, EPI_ISL_684330, EPI_ISL_684331, EPI_ISL_684332, EPI_ISL_684333, EPI_ISL_684334, EPI_ISL_684335, EPI_ISL_684336, EPI_ISL_684337, EPI_ISL_684338			
see above	Gunma Prefectural Institute of Public Health and Environmental Sciences	Pathogen Genomics Center, National Institute of Infectious Diseases	Tsuyoshi Sekizuka, Kentaro Itokawa, Rina Tanaka, Masanori Hashino, Makoto Kuroda
EPI_ISL_684339, EPI_ISL_684340, EPI_ISL_684341, EPI_ISL_684342, EPI_ISL_684343, EPI_ISL_684344, EPI_ISL_684345, EPI_ISL_684346, EPI_ISL_684347, EPI_ISL_684348, EPI_ISL_684349, EPI_ISL_684350, EPI_ISL_684351, EPI_ISL_684352, EPI_ISL_684353, EPI_ISL_684354, EPI_ISL_684355, EPI_ISL_684356, EPI_ISL_684357, EPI_ISL_684358, EPI_ISL_684359, EPI_ISL_684360, EPI_ISL_684361, EPI_ISL_684362, EPI_ISL_684363, EPI_ISL_684365, EPI_ISL_684366, EPI_ISL_684367, EPI_ISL_684368, EPI_ISL_684369, EPI_ISL_684370, EPI_ISL_684371, EPI_ISL_684372, EPI_ISL_684373, EPI_ISL_684374, EPI_ISL_684375,			

EPI_ISL_684376, EPI_ISL_684377, EPI_ISL_684378, EPI_ISL_684379, EPI_ISL_684381, EPI_ISL_684382, EPI_ISL_684383, EPI_ISL_684384, EPI_ISL_684385, EPI_ISL_684386, EPI_ISL_684387, EPI_ISL_684388, EPI_ISL_684389, EPI_ISL_684390, EPI_ISL_684391, EPI_ISL_684392, EPI_ISL_684393, EPI_ISL_684394, EPI_ISL_684395, EPI_ISL_684396, EPI_ISL_684397, EPI_ISL_684398, EPI_ISL_684399, EPI_ISL_684400, EPI_ISL_684401, EPI_ISL_684402, EPI_ISL_684403, EPI_ISL_684404, EPI_ISL_684405, EPI_ISL_684406, EPI_ISL_684407			
see above	Pathogen Genomics Center, National Institute of Infectious Diseases	Pathogen Genomics Center, National Institute of Infectious Diseases	Tsuyoshi Sekizuka, Kentaro Itokawa, Rina Tanaka, Masanori Hashino, Makoto Kuroda
EPI_ISL_684408, EPI_ISL_684409, EPI_ISL_684410, EPI_ISL_684411, EPI_ISL_684412, EPI_ISL_684413, EPI_ISL_684414, EPI_ISL_684415, EPI_ISL_684416, EPI_ISL_684417, EPI_ISL_684418, EPI_ISL_684419, EPI_ISL_684420, EPI_ISL_684421, EPI_ISL_684422, EPI_ISL_684423, EPI_ISL_684424, EPI_ISL_684425, EPI_ISL_684426, EPI_ISL_684427, EPI_ISL_684428, EPI_ISL_684429, EPI_ISL_684430, EPI_ISL_684431, EPI_ISL_684432, EPI_ISL_684433, EPI_ISL_684434, EPI_ISL_684435, EPI_ISL_684436, EPI_ISL_684437, EPI_ISL_684438, EPI_ISL_684439, EPI_ISL_684440, EPI_ISL_684441, EPI_ISL_684442, EPI_ISL_684443, EPI_ISL_684444, EPI_ISL_684445, EPI_ISL_684446, EPI_ISL_684447, EPI_ISL_684448, EPI_ISL_684449, EPI_ISL_684450, EPI_ISL_684451, EPI_ISL_684452, EPI_ISL_684453, EPI_ISL_684454, EPI_ISL_684455, EPI_ISL_684456, EPI_ISL_684457, EPI_ISL_684458, EPI_ISL_684459, EPI_ISL_684460, EPI_ISL_684461, EPI_ISL_684462, EPI_ISL_684463, EPI_ISL_684464, EPI_ISL_684465, EPI_ISL_684466, EPI_ISL_684467, EPI_ISL_684468, EPI_ISL_684469, EPI_ISL_684470, EPI_ISL_684471, EPI_ISL_684472, EPI_ISL_684473, EPI_ISL_684474, EPI_ISL_684475, EPI_ISL_684476, EPI_ISL_684477, EPI_ISL_684478, EPI_ISL_684479, EPI_ISL_684480, EPI_ISL_684481, EPI_ISL_684482, EPI_ISL_684483			
see above	Fukuoka Institute of Health and Environmental Sciences	Pathogen Genomics Center, National Institute of Infectious Diseases	Tsuyoshi Sekizuka, Kentaro Itokawa, Rina Tanaka, Masanori Hashino, Makoto Kuroda
EPI_ISL_684484, EPI_ISL_684485, EPI_ISL_684486, EPI_ISL_684487, EPI_ISL_684488, EPI_ISL_684489, EPI_ISL_684490, EPI_ISL_684491, EPI_ISL_684492, EPI_ISL_684493, EPI_ISL_684494, EPI_ISL_684495, EPI_ISL_684496, EPI_ISL_684497, EPI_ISL_684498, EPI_ISL_684499, EPI_ISL_684500, EPI_ISL_684501, EPI_ISL_684502, EPI_ISL_684503, EPI_ISL_684504, EPI_ISL_684505			
see above	Pathogen Genomics Center, National Institute of Infectious Diseases	Pathogen Genomics Center, National Institute of Infectious Diseases	Tsuyoshi Sekizuka, Kentaro Itokawa, Rina Tanaka, Masanori Hashino, Makoto Kuroda
EPI_ISL_684506, EPI_ISL_684507, EPI_ISL_684508, EPI_ISL_684509, EPI_ISL_684510, EPI_ISL_684511, EPI_ISL_684512, EPI_ISL_684513, EPI_ISL_684514, EPI_ISL_684515, EPI_ISL_684516, EPI_ISL_684517, EPI_ISL_684518, EPI_ISL_684519, EPI_ISL_684520, EPI_ISL_684521, EPI_ISL_684522			
see above	Tokyo Metropolitan Institute of Public Health	Pathogen Genomics Center, National Institute of Infectious Diseases	Tsuyoshi Sekizuka, Kentaro Itokawa, Rina Tanaka, Masanori Hashino, Makoto Kuroda
EPI_ISL_684523, EPI_ISL_684524, EPI_ISL_684525, EPI_ISL_684526, EPI_ISL_684527, EPI_ISL_684529, EPI_ISL_684530, EPI_ISL_684531, EPI_ISL_684532, EPI_ISL_684533, EPI_ISL_684534, EPI_ISL_684535, EPI_ISL_684536, EPI_ISL_684537, EPI_ISL_684538, EPI_ISL_684539, EPI_ISL_684540, EPI_ISL_684541, EPI_ISL_684542, EPI_ISL_684543, EPI_ISL_684544, EPI_ISL_684545, EPI_ISL_684546, EPI_ISL_684547, EPI_ISL_684548, EPI_ISL_684549, EPI_ISL_684550, EPI_ISL_684551, EPI_ISL_684552, EPI_ISL_684553, EPI_ISL_684554, EPI_ISL_684555			
see above	Pathogen Genomics Center, National Institute of Infectious Diseases	Pathogen Genomics Center, National Institute of Infectious Diseases	Tsuyoshi Sekizuka, Kentaro Itokawa, Rina Tanaka, Masanori Hashino, Makoto Kuroda
EPI_ISL_684556, EPI_ISL_684557	Tokyo Metropolitan Institute of Public Health	Pathogen Genomics Center, National Institute of Infectious Diseases	Tsuyoshi Sekizuka, Kentaro Itokawa, Rina Tanaka, Masanori Hashino, Makoto Kuroda
EPI_ISL_684558, EPI_ISL_684559, EPI_ISL_684560, EPI_ISL_684561, EPI_ISL_684562, EPI_ISL_684563, EPI_ISL_684564, EPI_ISL_684565	Pathogen Genomics Center, National Institute of Infectious Diseases	Pathogen Genomics Center, National Institute of Infectious Diseases	Tsuyoshi Sekizuka, Kentaro Itokawa, Rina Tanaka, Masanori Hashino, Makoto Kuroda
EPI_ISL_684566, EPI_ISL_684567, EPI_ISL_684568, EPI_ISL_684569, EPI_ISL_684570, EPI_ISL_684571, EPI_ISL_684572, EPI_ISL_684573, EPI_ISL_684574, EPI_ISL_684575, EPI_ISL_684576			
see above	Gunma Prefectural Institute of Public Health and Environmental Sciences	Pathogen Genomics Center, National Institute of Infectious Diseases	Tsuyoshi Sekizuka, Kentaro Itokawa, Rina Tanaka, Masanori Hashino, Makoto Kuroda
EPI_ISL_684578, EPI_ISL_684579, EPI_ISL_684580, EPI_ISL_684581, EPI_ISL_684582, EPI_ISL_684586, EPI_ISL_684587, EPI_ISL_684588, EPI_ISL_684589, EPI_ISL_684590, EPI_ISL_684591, EPI_ISL_684592, EPI_ISL_684593, EPI_ISL_684596, EPI_ISL_684597, EPI_ISL_684598, EPI_ISL_684599, EPI_ISL_684600, EPI_ISL_684601, EPI_ISL_684602, EPI_ISL_684603, EPI_ISL_684604, EPI_ISL_684605, EPI_ISL_684606, EPI_ISL_684607, EPI_ISL_684608, EPI_ISL_684609, EPI_ISL_684610, EPI_ISL_684611, EPI_ISL_684614			
see above	Pathogen Genomics Center, National Institute of Infectious Diseases	Pathogen Genomics Center, National Institute of Infectious Diseases	Tsuyoshi Sekizuka, Kentaro Itokawa, Rina Tanaka, Masanori Hashino, Makoto Kuroda
EPI_ISL_684615, EPI_ISL_684616, EPI_ISL_684617, EPI_ISL_684619, EPI_ISL_684620, EPI_ISL_684622, EPI_ISL_684623, EPI_ISL_684624, EPI_ISL_684625, EPI_ISL_684626, EPI_ISL_684627, EPI_ISL_684628, EPI_ISL_684629, EPI_ISL_684630, EPI_ISL_684631, EPI_ISL_684632, EPI_ISL_684633			
see above	Tokyo Metropolitan Institute of Public Health	Pathogen Genomics Center, National Institute of Infectious Diseases	Tsuyoshi Sekizuka, Kentaro Itokawa, Rina Tanaka, Masanori Hashino, Makoto Kuroda
EPI_ISL_684634, EPI_ISL_684635, EPI_ISL_684636, EPI_ISL_684637, EPI_ISL_684638, EPI_ISL_684640, EPI_ISL_684641, EPI_ISL_684642, EPI_ISL_684643, EPI_ISL_684644, EPI_ISL_684645, EPI_ISL_684646, EPI_ISL_684647, EPI_ISL_684648, EPI_ISL_684649, EPI_ISL_684650, EPI_ISL_684651, EPI_ISL_684652, EPI_ISL_684653, EPI_ISL_684654, EPI_ISL_684655, EPI_ISL_684656, EPI_ISL_684657, EPI_ISL_684658, EPI_ISL_684659, EPI_ISL_684660, EPI_ISL_684661, EPI_ISL_684662, EPI_ISL_684663, EPI_ISL_684664, EPI_ISL_684665, EPI_ISL_684666, EPI_ISL_684667, EPI_ISL_684668, EPI_ISL_684669, EPI_ISL_684670, EPI_ISL_684671, EPI_ISL_684672, EPI_ISL_684673, EPI_ISL_684674, EPI_ISL_684675, EPI_ISL_684676, EPI_ISL_684677, EPI_ISL_684678, EPI_ISL_684679, EPI_ISL_684680, EPI_ISL_684681, EPI_ISL_684682, EPI_ISL_684683, EPI_ISL_684684, EPI_ISL_684685, EPI_ISL_684686, EPI_ISL_684687, EPI_ISL_684688, EPI_ISL_684689, EPI_ISL_684690, EPI_ISL_684691, EPI_ISL_684692			
see above	Fukuoka Institute of Health and Environmental Sciences	Pathogen Genomics Center, National Institute of Infectious Diseases	Tsuyoshi Sekizuka, Kentaro Itokawa, Rina Tanaka, Masanori Hashino, Makoto Kuroda
EPI_ISL_684693, EPI_ISL_684694, EPI_ISL_684695, EPI_ISL_684696, EPI_ISL_684697, EPI_ISL_684698, EPI_ISL_684699, EPI_ISL_684700, EPI_ISL_684701, EPI_ISL_684702, EPI_ISL_684703, EPI_ISL_684704, EPI_ISL_684705, EPI_ISL_684706, EPI_ISL_684707, EPI_ISL_684708, EPI_ISL_684709, EPI_ISL_684710, EPI_ISL_684711, EPI_ISL_684712, EPI_ISL_684713, EPI_ISL_684714, EPI_ISL_684715, EPI_ISL_684716, EPI_ISL_684717			
see above	Pathogen Genomics Center, National Institute of Infectious Diseases	Pathogen Genomics Center, National Institute of Infectious Diseases	Tsuyoshi Sekizuka, Kentaro Itokawa, Rina Tanaka, Masanori Hashino, Makoto Kuroda
EPI_ISL_684723, EPI_ISL_684724, EPI_ISL_684725, EPI_ISL_684726, EPI_ISL_684727	Fukuoka Institute of Health and Environmental Sciences	Pathogen Genomics Center, National Institute of Infectious Diseases	Tsuyoshi Sekizuka, Kentaro Itokawa, Rina Tanaka, Masanori Hashino, Makoto Kuroda
EPI_ISL_684728, EPI_ISL_684729, EPI_ISL_684730, EPI_ISL_684731, EPI_ISL_684732, EPI_ISL_684734, EPI_ISL_684735	Tokyo Metropolitan Institute of Public Health	Pathogen Genomics Center, National Institute of Infectious Diseases	Tsuyoshi Sekizuka, Kentaro Itokawa, Rina Tanaka, Masanori Hashino, Makoto Kuroda
EPI_ISL_684736, EPI_ISL_684737, EPI_ISL_684738, EPI_ISL_684739, EPI_ISL_684740, EPI_ISL_684741, EPI_ISL_684742, EPI_ISL_684743, EPI_ISL_684744, EPI_ISL_684745, EPI_ISL_684746, EPI_ISL_684747, EPI_ISL_684748, EPI_ISL_684749, EPI_ISL_684750, EPI_ISL_684751, EPI_ISL_684752, EPI_ISL_684753, EPI_ISL_684754, EPI_ISL_684755, EPI_ISL_684756, EPI_ISL_684757, EPI_ISL_684758, EPI_ISL_684759, EPI_ISL_684760, EPI_ISL_684761, EPI_ISL_684762, EPI_ISL_684763, EPI_ISL_684764, EPI_ISL_684765, EPI_ISL_684766, EPI_ISL_684767, EPI_ISL_684768, EPI_ISL_684769, EPI_ISL_684770, EPI_ISL_684771, EPI_ISL_684772, EPI_ISL_684773, EPI_ISL_684774, EPI_ISL_684775, EPI_ISL_684776, EPI_ISL_684777, EPI_ISL_684778, EPI_ISL_684779, EPI_ISL_684780, EPI_ISL_684781, EPI_ISL_684782, EPI_ISL_684783, EPI_ISL_684784, EPI_ISL_684785, EPI_ISL_684786, EPI_ISL_684787, EPI_ISL_684788, EPI_ISL_684789, EPI_ISL_684790, EPI_ISL_684791, EPI_ISL_684792, EPI_ISL_684793, EPI_ISL_684794, EPI_ISL_684795, EPI_ISL_684796, EPI_ISL_684797			
see above	Pathogen Genomics Center, National Institute of Infectious Diseases	Pathogen Genomics Center, National Institute of Infectious Diseases	Tsuyoshi Sekizuka, Kentaro Itokawa, Rina Tanaka, Masanori Hashino, Makoto Kuroda
EPI_ISL_684798, EPI_ISL_684799, EPI_ISL_684800, EPI_ISL_684801, EPI_ISL_684802, EPI_ISL_684803, EPI_ISL_684804, EPI_ISL_684805, EPI_ISL_684806, EPI_ISL_684807, EPI_ISL_684808, EPI_ISL_684809, EPI_ISL_684810, EPI_ISL_684811, EPI_ISL_684812, EPI_ISL_684813, EPI_ISL_684815, EPI_ISL_684816, EPI_ISL_684817, EPI_ISL_684818, EPI_ISL_684819, EPI_ISL_684820, EPI_ISL_684821, EPI_ISL_684822, EPI_ISL_684823, EPI_ISL_684824, EPI_ISL_684825, EPI_ISL_684826, EPI_ISL_684827, EPI_ISL_684828, EPI_ISL_684829, EPI_ISL_684830, EPI_ISL_684831, EPI_ISL_684832, EPI_ISL_684833, EPI_ISL_684834, EPI_ISL_684835, EPI_ISL_684836, EPI_ISL_684837, EPI_ISL_684838, EPI_ISL_684839, EPI_ISL_684840, EPI_ISL_684841, EPI_ISL_684842, EPI_ISL_684843, EPI_ISL_684844, EPI_ISL_684845, EPI_ISL_684846, EPI_ISL_684847, EPI_ISL_684848, EPI_ISL_684849, EPI_ISL_684850, EPI_ISL_684851, EPI_ISL_684852, EPI_ISL_684853, EPI_ISL_684854, EPI_ISL_684855, EPI_ISL_684856			
see above	Yamagata Prefectural Institute of Public Health	Pathogen Genomics Center, National Institute of Infectious Diseases	Tsuyoshi Sekizuka, Kentaro Itokawa, Rina Tanaka, Masanori Hashino, Makoto Kuroda
EPI_ISL_684857, EPI_ISL_684858, EPI_ISL_684859, EPI_ISL_684860, EPI_ISL_684861, EPI_ISL_684862, EPI_ISL_684863, EPI_ISL_684864, EPI_ISL_684865, EPI_ISL_684866, EPI_ISL_684867, EPI_ISL_684868, EPI_ISL_684869, EPI_ISL_684870, EPI_ISL_684871, EPI_ISL_684872, EPI_ISL_684873, EPI_ISL_684874, EPI_ISL_684875, EPI_ISL_684876, EPI_ISL_684877, EPI_ISL_684878, EPI_ISL_684879, EPI_ISL_684880			
see above	Pathogen Genomics Center, National Institute of Infectious Diseases	Pathogen Genomics Center, National Institute of Infectious Diseases	Tsuyoshi Sekizuka, Kentaro Itokawa, Rina Tanaka, Masanori Hashino, Makoto Kuroda
EPI_ISL_684881, EPI_ISL_684882, EPI_ISL_684883, EPI_ISL_684884, EPI_ISL_684885, EPI_ISL_684886, EPI_ISL_684887, EPI_ISL_684888, EPI_ISL_684889, EPI_ISL_684890, EPI_ISL_684891, EPI_ISL_684892, EPI_ISL_684893, EPI_ISL_684894, EPI_ISL_684895, EPI_ISL_684896, EPI_ISL_684897, EPI_ISL_684898, EPI_ISL_684899, EPI_ISL_684900, EPI_ISL_684901, EPI_ISL_684902, EPI_ISL_684903, EPI_ISL_684904, EPI_ISL_684905, EPI_ISL_684906, EPI_ISL_684907, EPI_ISL_684908, EPI_ISL_684909, EPI_ISL_684910, EPI_ISL_684911, EPI_ISL_684912, EPI_ISL_684913, EPI_ISL_684914, EPI_ISL_684915, EPI_ISL_684916,			

EPI_ISL_684917, EPI_ISL_684918, EPI_ISL_684919, EPI_ISL_684920, EPI_ISL_684921, EPI_ISL_684922, EPI_ISL_684923, EPI_ISL_684924, EPI_ISL_684925, EPI_ISL_684926, EPI_ISL_684927, EPI_ISL_684928, EPI_ISL_684929, EPI_ISL_684930, EPI_ISL_684931, EPI_ISL_684932, EPI_ISL_684933, EPI_ISL_684934, EPI_ISL_684935, EPI_ISL_684936, EPI_ISL_684937, EPI_ISL_684938, EPI_ISL_684939, EPI_ISL_684940, EPI_ISL_684941, EPI_ISL_684942, EPI_ISL_684943, EPI_ISL_684944, EPI_ISL_684945, EPI_ISL_684946, EPI_ISL_684947, EPI_ISL_684948, EPI_ISL_684949, EPI_ISL_684950, EPI_ISL_684951, EPI_ISL_684952, EPI_ISL_684953, EPI_ISL_684954, EPI_ISL_684955, EPI_ISL_684956, EPI_ISL_684957, EPI_ISL_684958, EPI_ISL_684959, EPI_ISL_684960, EPI_ISL_684961, EPI_ISL_684962, EPI_ISL_684963, EPI_ISL_684965, EPI_ISL_684966, EPI_ISL_684967, EPI_ISL_684968, EPI_ISL_684969, EPI_ISL_684970, EPI_ISL_684971, EPI_ISL_684972, EPI_ISL_684973, EPI_ISL_684974, EPI_ISL_684975, EPI_ISL_684976, EPI_ISL_684977, EPI_ISL_684978, EPI_ISL_684979, EPI_ISL_684980, EPI_ISL_684981, EPI_ISL_684982, EPI_ISL_684983, EPI_ISL_684984, EPI_ISL_684985, EPI_ISL_684986, EPI_ISL_684987, EPI_ISL_684988, EPI_ISL_684989, EPI_ISL_684990, EPI_ISL_684991, EPI_ISL_684992, EPI_ISL_684993, EPI_ISL_684994, EPI_ISL_684995, EPI_ISL_684996, EPI_ISL_684997, EPI_ISL_684998, EPI_ISL_684999, EPI_ISL_685000, EPI_ISL_685001, EPI_ISL_685002, EPI_ISL_685003, EPI_ISL_685004, EPI_ISL_685005, EPI_ISL_685006, EPI_ISL_685007, EPI_ISL_685008, EPI_ISL_685009, EPI_ISL_685010, EPI_ISL_685011, EPI_ISL_685012, EPI_ISL_685013, EPI_ISL_685014, EPI_ISL_685015, EPI_ISL_685016, EPI_ISL_685017, EPI_ISL_685018, EPI_ISL_685019, EPI_ISL_685020, EPI_ISL_685021, EPI_ISL_685022, EPI_ISL_685023, EPI_ISL_685024, EPI_ISL_685025, EPI_ISL_685026, EPI_ISL_685027, EPI_ISL_685028, EPI_ISL_685029, EPI_ISL_685030, EPI_ISL_685031, EPI_ISL_685032, EPI_ISL_685033, EPI_ISL_685034, EPI_ISL_685035, EPI_ISL_685036, EPI_ISL_685037, EPI_ISL_685038, EPI_ISL_685039, EPI_ISL_685040, EPI_ISL_685041, EPI_ISL_685042, EPI_ISL_685043, EPI_ISL_685044, EPI_ISL_685045, EPI_ISL_685046, EPI_ISL_685047, EPI_ISL_685048, EPI_ISL_685049, EPI_ISL_685050, EPI_ISL_685051, EPI_ISL_685052, EPI_ISL_685053, EPI_ISL_685054, EPI_ISL_685055, EPI_ISL_685056, EPI_ISL_685057, EPI_ISL_685058, EPI_ISL_685059, EPI_ISL_685060, EPI_ISL_685061, EPI_ISL_685062, EPI_ISL_685063, EPI_ISL_685064, EPI_ISL_685065, EPI_ISL_685066, EPI_ISL_685067, EPI_ISL_685068, EPI_ISL_685069, EPI_ISL_685070, EPI_ISL_685071, EPI_ISL_685072, EPI_ISL_685073, EPI_ISL_685074, EPI_ISL_685075, EPI_ISL_685076, EPI_ISL_685077, EPI_ISL_685078, EPI_ISL_685079, EPI_ISL_685080, EPI_ISL_685081, EPI_ISL_685082, EPI_ISL_685083, EPI_ISL_685084, EPI_ISL_685085, EPI_ISL_685086, EPI_ISL_685087, EPI_ISL_685088, EPI_ISL_685089, EPI_ISL_685090, EPI_ISL_685091, EPI_ISL_685092, EPI_ISL_685093, EPI_ISL_685094, EPI_ISL_685095, EPI_ISL_685096, EPI_ISL_685097, EPI_ISL_685098, EPI_ISL_685099, EPI_ISL_685100, EPI_ISL_685101, EPI_ISL_685102, EPI_ISL_685103, EPI_ISL_685104, EPI_ISL_685105, EPI_ISL_685106, EPI_ISL_685107, EPI_ISL_685108, EPI_ISL_685109, EPI_ISL_685110, EPI_ISL_685111, EPI_ISL_685112, EPI_ISL_685113, EPI_ISL_685114, EPI_ISL_685115, EPI_ISL_685116, EPI_ISL_685117, EPI_ISL_685118, EPI_ISL_685119, EPI_ISL_685120, EPI_ISL_685121, EPI_ISL_685122, EPI_ISL_685123, EPI_ISL_685124, EPI_ISL_685126, EPI_ISL_685127, EPI_ISL_685128, EPI_ISL_685129, EPI_ISL_685130, EPI_ISL_685131, EPI_ISL_685132			
see above	Saitama Prefectural Institute of Public Health	Pathogen Genomics Center, National Institute of Infectious Diseases	Tsuyoshi Sekizuka, Kentaro Itokawa, Rina Tanaka, Masanori Hashino, Makoto Kuroda
EPI_ISL_685133, EPI_ISL_685134, EPI_ISL_685135, EPI_ISL_685136, EPI_ISL_685137, EPI_ISL_685138, EPI_ISL_685139, EPI_ISL_685140, EPI_ISL_685141, EPI_ISL_685142, EPI_ISL_685143, EPI_ISL_685144, EPI_ISL_685145, EPI_ISL_685146, EPI_ISL_685147, EPI_ISL_685148, EPI_ISL_685149, EPI_ISL_685150, EPI_ISL_685151, EPI_ISL_685152, EPI_ISL_685153, EPI_ISL_685154, EPI_ISL_685155, EPI_ISL_685156, EPI_ISL_685157, EPI_ISL_685158, EPI_ISL_685159, EPI_ISL_685160, EPI_ISL_685161, EPI_ISL_685162, EPI_ISL_685163, EPI_ISL_685164, EPI_ISL_685165, EPI_ISL_685166, EPI_ISL_685167, EPI_ISL_685168, EPI_ISL_685169, EPI_ISL_685170, EPI_ISL_685171, EPI_ISL_685172, EPI_ISL_685173, EPI_ISL_685174, EPI_ISL_685175, EPI_ISL_685176, EPI_ISL_685177, EPI_ISL_685178, EPI_ISL_685179, EPI_ISL_685180, EPI_ISL_685181, EPI_ISL_685182, EPI_ISL_685183, EPI_ISL_685184, EPI_ISL_685185, EPI_ISL_685186, EPI_ISL_685187, EPI_ISL_685188, EPI_ISL_685189, EPI_ISL_685190, EPI_ISL_685191, EPI_ISL_685192, EPI_ISL_685193, EPI_ISL_685194, EPI_ISL_685195, EPI_ISL_685196, EPI_ISL_685197, EPI_ISL_685198, EPI_ISL_685199, EPI_ISL_685200, EPI_ISL_685201, EPI_ISL_685202, EPI_ISL_685203, EPI_ISL_685204, EPI_ISL_685205, EPI_ISL_685206, EPI_ISL_685207, EPI_ISL_685208, EPI_ISL_685209, EPI_ISL_685210, EPI_ISL_685211, EPI_ISL_685212, EPI_ISL_685213, EPI_ISL_685214, EPI_ISL_685215, EPI_ISL_685216, EPI_ISL_685217, EPI_ISL_685218, EPI_ISL_685219			
see above	Pathogen Genomics Center, National Institute of Infectious Diseases	Pathogen Genomics Center, National Institute of Infectious Diseases	Tsuyoshi Sekizuka, Kentaro Itokawa, Rina Tanaka, Masanori Hashino, Makoto Kuroda
EPI_ISL_685220, EPI_ISL_685221, EPI_ISL_685222, EPI_ISL_685223, EPI_ISL_685224, EPI_ISL_685225, EPI_ISL_685226, EPI_ISL_685227, EPI_ISL_685228, EPI_ISL_685229, EPI_ISL_685230, EPI_ISL_685231, EPI_ISL_685232, EPI_ISL_685233, EPI_ISL_685234, EPI_ISL_685235, EPI_ISL_685236, EPI_ISL_685237			
see above	Tokyo Metropolitan Institute of Public Health	Pathogen Genomics Center, National Institute of Infectious Diseases	Tsuyoshi Sekizuka, Kentaro Itokawa, Rina Tanaka, Masanori Hashino, Makoto Kuroda
EPI_ISL_685238, EPI_ISL_685240, EPI_ISL_685241, EPI_ISL_685242, EPI_ISL_685243, EPI_ISL_685244, EPI_ISL_685245, EPI_ISL_685246, EPI_ISL_685247, EPI_ISL_685248, EPI_ISL_685249, EPI_ISL_685250, EPI_ISL_685251, EPI_ISL_685252, EPI_ISL_685253, EPI_ISL_685254, EPI_ISL_685255, EPI_ISL_685256, EPI_ISL_685257, EPI_ISL_685258, EPI_ISL_685259, EPI_ISL_685260, EPI_ISL_685261, EPI_ISL_685262, EPI_ISL_685263, EPI_ISL_685264, EPI_ISL_685265, EPI_ISL_685266, EPI_ISL_685267, EPI_ISL_685268, EPI_ISL_685269, EPI_ISL_685270, EPI_ISL_685271, EPI_ISL_685272, EPI_ISL_685273, EPI_ISL_685274, EPI_ISL_685275, EPI_ISL_685276, EPI_ISL_685277, EPI_ISL_685278			
see above	Pathogen Genomics Center, National Institute of Infectious Diseases	Pathogen Genomics Center, National Institute of Infectious Diseases	Tsuyoshi Sekizuka, Kentaro Itokawa, Rina Tanaka, Masanori Hashino, Makoto Kuroda
EPI_ISL_685279, EPI_ISL_685280, EPI_ISL_685281	Gunma Prefectural Institute of Public Health and Environmental Sciences	Pathogen Genomics Center, National Institute of Infectious Diseases	Tsuyoshi Sekizuka, Kentaro Itokawa, Rina Tanaka, Masanori Hashino, Makoto Kuroda
EPI_ISL_685283, EPI_ISL_685284, EPI_ISL_685285, EPI_ISL_685286, EPI_ISL_685287, EPI_ISL_685288, EPI_ISL_685289, EPI_ISL_685290, EPI_ISL_685291, EPI_ISL_685292, EPI_ISL_685295			
see above	Pathogen Genomics Center, National Institute of Infectious Diseases	Pathogen Genomics Center, National Institute of Infectious Diseases	Tsuyoshi Sekizuka, Kentaro Itokawa, Rina Tanaka, Masanori Hashino, Makoto Kuroda
EPI_ISL_685297, EPI_ISL_685298, EPI_ISL_685299, EPI_ISL_685300, EPI_ISL_685301, EPI_ISL_685302, EPI_ISL_685303, EPI_ISL_685304, EPI_ISL_685305, EPI_ISL_685306, EPI_ISL_685307, EPI_ISL_685308, EPI_ISL_685309, EPI_ISL_685310			
see above	Niigata Prefectural Institute of Public Health and Environmental Sciences	Pathogen Genomics Center, National Institute of Infectious Diseases	Tsuyoshi Sekizuka, Kentaro Itokawa, Rina Tanaka, Masanori Hashino, Makoto Kuroda
EPI_ISL_685311, EPI_ISL_685312, EPI_ISL_685313, EPI_ISL_685314, EPI_ISL_685315, EPI_ISL_685316, EPI_ISL_685317, EPI_ISL_685318, EPI_ISL_685319, EPI_ISL_685320, EPI_ISL_685321, EPI_ISL_685322, EPI_ISL_685323, EPI_ISL_685324, EPI_ISL_685325, EPI_ISL_685326, EPI_ISL_685327, EPI_ISL_685328, EPI_ISL_685329, EPI_ISL_685330, EPI_ISL_685331, EPI_ISL_685332, EPI_ISL_685333, EPI_ISL_685334, EPI_ISL_685335, EPI_ISL_685336, EPI_ISL_685337, EPI_ISL_685338, EPI_ISL_685339, EPI_ISL_685340, EPI_ISL_685341, EPI_ISL_685342, EPI_ISL_685343, EPI_ISL_685344, EPI_ISL_685345, EPI_ISL_685346, EPI_ISL_685347, EPI_ISL_685348, EPI_ISL_685349, EPI_ISL_685350, EPI_ISL_685351, EPI_ISL_685352, EPI_ISL_685353, EPI_ISL_685354, EPI_ISL_685355, EPI_ISL_685356, EPI_ISL_685357, EPI_ISL_685358, EPI_ISL_685359, EPI_ISL_685360, EPI_ISL_685361, EPI_ISL_685362, EPI_ISL_685363, EPI_ISL_685364, EPI_ISL_685365, EPI_ISL_685366, EPI_ISL_685367, EPI_ISL_685368, EPI_ISL_685369, EPI_ISL_685370, EPI_ISL_685371, EPI_ISL_685372, EPI_ISL_685373, EPI_ISL_685374, EPI_ISL_685375, EPI_ISL_685376, EPI_ISL_685377, EPI_ISL_685378, EPI_ISL_685379, EPI_ISL_685380, EPI_ISL_685381, EPI_ISL_685382, EPI_ISL_685383, EPI_ISL_685384, EPI_ISL_685385, EPI_ISL_685386, EPI_ISL_685387, EPI_ISL_685388, EPI_ISL_685389, EPI_ISL_685390, EPI_ISL_685391, EPI_ISL_685392, EPI_ISL_685393, EPI_ISL_685394, EPI_ISL_685395, EPI_ISL_685396, EPI_ISL_685397, EPI_ISL_685400, EPI_ISL_685401, EPI_ISL_685402, EPI_ISL_685403, EPI_ISL_685404, EPI_ISL_685405, EPI_ISL_685406			
see above	Pathogen Genomics Center, National Institute of Infectious Diseases	Pathogen Genomics Center, National Institute of Infectious Diseases	Tsuyoshi Sekizuka, Kentaro Itokawa, Rina Tanaka, Masanori Hashino, Makoto Kuroda
EPI_ISL_685407, EPI_ISL_685408, EPI_ISL_685409, EPI_ISL_685410, EPI_ISL_685411, EPI_ISL_685412, EPI_ISL_685413, EPI_ISL_685414, EPI_ISL_685415, EPI_ISL_685416, EPI_ISL_685417, EPI_ISL_685418, EPI_ISL_685419, EPI_ISL_685420, EPI_ISL_685421, EPI_ISL_685422, EPI_ISL_685423, EPI_ISL_685424, EPI_ISL_685425, EPI_ISL_685426, EPI_ISL_685427, EPI_ISL_685428, EPI_ISL_685429, EPI_ISL_685430, EPI_ISL_685431, EPI_ISL_685432, EPI_ISL_685433, EPI_ISL_685434, EPI_ISL_685435, EPI_ISL_685436, EPI_ISL_685437, EPI_ISL_685438, EPI_ISL_685439, EPI_ISL_685440, EPI_ISL_685441, EPI_ISL_685442, EPI_ISL_685443, EPI_ISL_685444, EPI_ISL_685445, EPI_ISL_685446, EPI_ISL_685447, EPI_ISL_685448, EPI_ISL_685449, EPI_ISL_685450, EPI_ISL_685451, EPI_ISL_685452, EPI_ISL_685453, EPI_ISL_685454, EPI_ISL_685455, EPI_ISL_685456, EPI_ISL_685457, EPI_ISL_685458, EPI_ISL_685459, EPI_ISL_685460, EPI_ISL_685461, EPI_ISL_685462, EPI_ISL_685463, EPI_ISL_685464, EPI_ISL_685465, EPI_ISL_685466, EPI_ISL_685467, EPI_ISL_685468, EPI_ISL_685469, EPI_ISL_685470, EPI_ISL_685471, EPI_ISL_685472, EPI_ISL_685473, EPI_ISL_685474, EPI_ISL_685475, EPI_ISL_685476, EPI_ISL_685477, EPI_ISL_685478, EPI_ISL_685479, EPI_ISL_685480, EPI_ISL_685481, EPI_ISL_685482, EPI_ISL_685483, EPI_ISL_685484, EPI_ISL_685485, EPI_ISL_685486, EPI_ISL_685487, EPI_ISL_685488, EPI_ISL_685489, EPI_ISL_685490, EPI_ISL_685491, EPI_ISL_685492, EPI_ISL_685493, EPI_ISL_685494, EPI_ISL_685495, EPI_ISL_685496, EPI_ISL_685497			
see above	Tokyo Metropolitan Institute of Public Health	Pathogen Genomics Center, National Institute of Infectious Diseases	Tsuyoshi Sekizuka, Kentaro Itokawa, Rina Tanaka, Masanori Hashino, Makoto Kuroda
EPI_ISL_685498, EPI_ISL_685499, EPI_ISL_685500, EPI_ISL_685503, EPI_ISL_685504	Yamagata Prefectural Institute of Public Health	Pathogen Genomics Center, National Institute of Infectious Diseases	Tsuyoshi Sekizuka, Kentaro Itokawa, Rina Tanaka, Masanori Hashino, Makoto Kuroda
EPI_ISL_685505, EPI_ISL_685506, EPI_ISL_685507, EPI_ISL_685508, EPI_ISL_685509, EPI_ISL_685510, EPI_ISL_685511, EPI_ISL_685512, EPI_ISL_685513, EPI_ISL_685514, EPI_ISL_685515, EPI_ISL_685516, EPI_ISL_685517, EPI_ISL_685518, EPI_ISL_685519, EPI_ISL_685520, EPI_ISL_685521, EPI_ISL_685522, EPI_ISL_685523, EPI_ISL_685524, EPI_ISL_685525, EPI_ISL_685526, EPI_ISL_685527, EPI_ISL_685528, EPI_ISL_685529, EPI_ISL_685530, EPI_ISL_685531, EPI_ISL_685532, EPI_ISL_685533, EPI_ISL_685534, EPI_ISL_685535, EPI_ISL_685536, EPI_ISL_685537, EPI_ISL_685538, EPI_ISL_685539, EPI_ISL_685540, EPI_ISL_685541, EPI_ISL_685542, EPI_ISL_685543, EPI_ISL_685544, EPI_ISL_685545, EPI_ISL_685546, EPI_ISL_685547, EPI_ISL_685548, EPI_ISL_685549, EPI_ISL_685550, EPI_ISL_685551, EPI_ISL_685552, EPI_ISL_685553, EPI_ISL_685554, EPI_ISL_685555, EPI_ISL_685556, EPI_ISL_685557, EPI_ISL_685558, EPI_ISL_685559, EPI_ISL_685560, EPI_ISL_685561, EPI_ISL_685562, EPI_ISL_685563, EPI_ISL_685564, EPI_ISL_685565, EPI_ISL_685566, EPI_ISL_685567, EPI_ISL_685568, EPI_ISL_685569, EPI_ISL_685570, EPI_ISL_685571, EPI_ISL_685572, EPI_ISL_685573, EPI_ISL_685574, EPI_ISL_685575, EPI_ISL_685576, EPI_ISL_685577, EPI_ISL_685578, EPI_ISL_685579, EPI_ISL_685580, EPI_ISL_685581, EPI_ISL_685582, EPI_ISL_685584, EPI_ISL_685585, EPI_ISL_685586, EPI_ISL_685587, EPI_ISL_685588, EPI_ISL_685589, EPI_ISL_685590, EPI_ISL_685591, EPI_ISL_685592, EPI_ISL_685593, EPI_ISL_685594, EPI_ISL_685595, EPI_ISL_685596, EPI_ISL_685597, EPI_ISL_685598, EPI_ISL_685599, EPI_ISL_685600, EPI_ISL_685601, EPI_ISL_685602, EPI_ISL_685603, EPI_ISL_685604, EPI_ISL_685605			
see above	Pathogen Genomics Center, National Institute of Infectious Diseases	Pathogen Genomics Center, National Institute of Infectious Diseases	Tsuyoshi Sekizuka, Kentaro Itokawa, Rina Tanaka, Masanori Hashino, Makoto Kuroda
EPI_ISL_685606, EPI_ISL_685607, EPI_ISL_685608, EPI_ISL_685609, EPI_ISL_685610, EPI_ISL_685611, EPI_ISL_685612, EPI_ISL_685613, EPI_ISL_685614, EPI_ISL_685615, EPI_ISL_685616			
see above	Tokyo Metropolitan Institute of Public Health	Pathogen Genomics Center, National Institute of Infectious Diseases	Tsuyoshi Sekizuka, Kentaro Itokawa, Rina Tanaka, Masanori Hashino, Makoto Kuroda
EPI_ISL_685617, EPI_ISL_685618, EPI_ISL_685619, EPI_ISL_685620, EPI_ISL_685621, EPI_ISL_685622, EPI_ISL_685623, EPI_ISL_685624, EPI_ISL_685625, EPI_ISL_685626, EPI_ISL_685627, EPI_ISL_685628, EPI_ISL_685629, EPI_ISL_685630, EPI_ISL_685631, EPI_ISL_685632, EPI_ISL_685633, EPI_ISL_685634, EPI_ISL_685635, EPI_ISL_685636, EPI_ISL_685637, EPI_ISL_685638, EPI_ISL_685639, EPI_ISL_685640, EPI_ISL_685641, EPI_ISL_685642, EPI_ISL_685643, EPI_ISL_685644, EPI_ISL_685645, EPI_ISL_685646, EPI_ISL_685647, EPI_ISL_685648, EPI_ISL_685649, EPI_ISL_685650, EPI_ISL_685651, EPI_ISL_685652, EPI_ISL_685653, EPI_ISL_685654, EPI_ISL_685655, EPI_ISL_685656, EPI_ISL_685657, EPI_ISL_685658, EPI_ISL_685659, EPI_ISL_685660, EPI_ISL_685661, EPI_ISL_685662, EPI_ISL_685663, EPI_ISL_685664, EPI_ISL_685665			
see above	Pathogen Genomics Center, National Institute of Infectious Diseases	Pathogen Genomics Center, National Institute of Infectious Diseases	Tsuyoshi Sekizuka, Kentaro Itokawa, Rina Tanaka, Masanori Hashino, Makoto Kuroda
EPI_ISL_685666, EPI_ISL_685667, EPI_ISL_685668, EPI_ISL_685669, EPI_ISL_685670, EPI_ISL_685671, EPI_ISL_685672, EPI_ISL_685673, EPI_ISL_685674, EPI_ISL_685675, EPI_ISL_685676, EPI_ISL_685677, EPI_ISL_685678, EPI_ISL_685679, EPI_ISL_685680, EPI_ISL_685681, EPI_ISL_685682, EPI_ISL_685683, EPI_ISL_685684, EPI_ISL_685685, EPI_ISL_685686, EPI_ISL_685687, EPI_ISL_685688, EPI_ISL_685689, EPI_ISL_685690, EPI_ISL_685691, EPI_ISL_685692, EPI_ISL_685693, EPI_ISL_685694			
see above	Tokyo Metropolitan Institute of Public Health	Pathogen Genomics Center, National Institute of Infectious	Tsuyoshi Sekizuka, Kentaro Itokawa, Rina Tanaka, Masanori Hashino, Makoto Kuroda

		Diseases	
EPI_ISL_685695, EPI_ISL_685696, EPI_ISL_685697, EPI_ISL_685698, EPI_ISL_685699, EPI_ISL_685700, EPI_ISL_685701, EPI_ISL_685702, EPI_ISL_685703, EPI_ISL_685704, EPI_ISL_685705, EPI_ISL_685706, EPI_ISL_685707, EPI_ISL_685708, EPI_ISL_685709, EPI_ISL_685710, EPI_ISL_685711, EPI_ISL_685712, EPI_ISL_685713, EPI_ISL_685714, EPI_ISL_685715, EPI_ISL_685716, EPI_ISL_685717, EPI_ISL_685718, EPI_ISL_685719, EPI_ISL_685720, EPI_ISL_685721, EPI_ISL_685722, EPI_ISL_685723, EPI_ISL_685724, EPI_ISL_685725, EPI_ISL_685726, EPI_ISL_685727, EPI_ISL_685728, EPI_ISL_685729, EPI_ISL_685730, EPI_ISL_685733, EPI_ISL_685734, EPI_ISL_685735, EPI_ISL_685736, EPI_ISL_685737, EPI_ISL_685738, EPI_ISL_685739, EPI_ISL_685740, EPI_ISL_685741, EPI_ISL_685742, EPI_ISL_685743, EPI_ISL_685744, EPI_ISL_685745, EPI_ISL_685746, EPI_ISL_685747, EPI_ISL_685748, EPI_ISL_685749, EPI_ISL_685750, EPI_ISL_685751, EPI_ISL_685752, EPI_ISL_685753, EPI_ISL_685754, EPI_ISL_685755, EPI_ISL_685756, EPI_ISL_685757, EPI_ISL_685758, EPI_ISL_685759, EPI_ISL_685760, EPI_ISL_685761, EPI_ISL_685762, EPI_ISL_685763, EPI_ISL_685764, EPI_ISL_685765, EPI_ISL_685766, EPI_ISL_685767, EPI_ISL_685768, EPI_ISL_685769, EPI_ISL_685770, EPI_ISL_685771, EPI_ISL_685772, EPI_ISL_685773, EPI_ISL_685774, EPI_ISL_685775, EPI_ISL_685776, EPI_ISL_685777, EPI_ISL_685778, EPI_ISL_685779, EPI_ISL_685780, EPI_ISL_685781, EPI_ISL_685782, EPI_ISL_685783, EPI_ISL_685784, EPI_ISL_685785, EPI_ISL_685786, EPI_ISL_685787, EPI_ISL_685788, EPI_ISL_685789, EPI_ISL_685790, EPI_ISL_685791, EPI_ISL_685792, EPI_ISL_685793, EPI_ISL_685794, EPI_ISL_685795, EPI_ISL_685796, EPI_ISL_685797, EPI_ISL_685798, EPI_ISL_685799, EPI_ISL_685800, EPI_ISL_685801, EPI_ISL_685802, EPI_ISL_685803, EPI_ISL_685804, EPI_ISL_685805, EPI_ISL_685806, EPI_ISL_685807, EPI_ISL_685808, EPI_ISL_685809, EPI_ISL_685810, EPI_ISL_685811, EPI_ISL_685812, EPI_ISL_685813, EPI_ISL_685814, EPI_ISL_685815, EPI_ISL_685816, EPI_ISL_685817, EPI_ISL_685818, EPI_ISL_685819, EPI_ISL_685820, EPI_ISL_685821, EPI_ISL_685822, EPI_ISL_685823, EPI_ISL_685824, EPI_ISL_685825, EPI_ISL_685826, EPI_ISL_685827, EPI_ISL_685828, EPI_ISL_685829, EPI_ISL_685830, EPI_ISL_685831, EPI_ISL_685832, EPI_ISL_685833, EPI_ISL_685834, EPI_ISL_685835, EPI_ISL_685836, EPI_ISL_685837, EPI_ISL_685838, EPI_ISL_685839, EPI_ISL_685840, EPI_ISL_685841, EPI_ISL_685842, EPI_ISL_685843, EPI_ISL_685844, EPI_ISL_685845, EPI_ISL_685846, EPI_ISL_685847, EPI_ISL_685848, EPI_ISL_685849, EPI_ISL_685850, EPI_ISL_685851, EPI_ISL_685852, EPI_ISL_685853, EPI_ISL_685854, EPI_ISL_685855, EPI_ISL_685856, EPI_ISL_685857, EPI_ISL_685858, EPI_ISL_685859, EPI_ISL_685860, EPI_ISL_685861, EPI_ISL_685862, EPI_ISL_685863, EPI_ISL_685864, EPI_ISL_685865, EPI_ISL_685866, EPI_ISL_685867, EPI_ISL_685868, EPI_ISL_685869, EPI_ISL_685870, EPI_ISL_685871, EPI_ISL_685872, EPI_ISL_685873, EPI_ISL_685874, EPI_ISL_685875, EPI_ISL_685876, EPI_ISL_685877, EPI_ISL_685878, EPI_ISL_685879, EPI_ISL_685880, EPI_ISL_685881, EPI_ISL_685882, EPI_ISL_685883, EPI_ISL_685884, EPI_ISL_685885, EPI_ISL_685886, EPI_ISL_685887, EPI_ISL_685888, EPI_ISL_685889, EPI_ISL_685890, EPI_ISL_685891, EPI_ISL_685892, EPI_ISL_685893, EPI_ISL_685894, EPI_ISL_685895, EPI_ISL_685896, EPI_ISL_685897, EPI_ISL_685898, EPI_ISL_685899, EPI_ISL_685900, EPI_ISL_685901, EPI_ISL_685902, EPI_ISL_685903, EPI_ISL_685904, EPI_ISL_685905, EPI_ISL_685906, EPI_ISL_685907, EPI_ISL_685908, EPI_ISL_685909, EPI_ISL_685911, EPI_ISL_685912, EPI_ISL_685913, EPI_ISL_685914, EPI_ISL_685915, EPI_ISL_685916			
see above	Pathogen Genomics Center, National Institute of Infectious Diseases	Pathogen Genomics Center, National Institute of Infectious Diseases	Tsuyoshi Sekizuka, Kentaro Itokawa, Rina Tanaka, Masanori Hashino, Makoto Kuroda
EPI_ISL_685918, EPI_ISL_685919, EPI_ISL_685920, EPI_ISL_685921, EPI_ISL_685922, EPI_ISL_685924	Fukuoka Institute of Health and Environmental Sciences	Pathogen Genomics Center, National Institute of Infectious Diseases	Tsuyoshi Sekizuka, Kentaro Itokawa, Rina Tanaka, Masanori Hashino, Makoto Kuroda
EPI_ISL_685925, EPI_ISL_685926, EPI_ISL_685927, EPI_ISL_685928, EPI_ISL_685929, EPI_ISL_685930, EPI_ISL_685931, EPI_ISL_685932, EPI_ISL_685933, EPI_ISL_685934, EPI_ISL_685935, EPI_ISL_685936, EPI_ISL_685937, EPI_ISL_685947, EPI_ISL_685949, EPI_ISL_685950, EPI_ISL_685953, EPI_ISL_685954, EPI_ISL_685955, EPI_ISL_685957, EPI_ISL_685958, EPI_ISL_685959, EPI_ISL_685960, EPI_ISL_685961, EPI_ISL_685962			
see above	Pathogen Genomics Center, National Institute of Infectious Diseases	Pathogen Genomics Center, National Institute of Infectious Diseases	Tsuyoshi Sekizuka, Kentaro Itokawa, Rina Tanaka, Masanori Hashino, Makoto Kuroda
EPI_ISL_685987	Tokyo Metropolitan Institute of Public Health	Pathogen Genomics Center, National Institute of Infectious Diseases	Tsuyoshi Sekizuka, Kentaro Itokawa, Rina Tanaka, Masanori Hashino, Makoto Kuroda
EPI_ISL_685988, EPI_ISL_685989, EPI_ISL_685990, EPI_ISL_685991, EPI_ISL_685992, EPI_ISL_685993, EPI_ISL_685994, EPI_ISL_685995, EPI_ISL_685996, EPI_ISL_685997, EPI_ISL_685998, EPI_ISL_685999, EPI_ISL_686000, EPI_ISL_686001, EPI_ISL_686002, EPI_ISL_686003, EPI_ISL_686004, EPI_ISL_686005, EPI_ISL_686006, EPI_ISL_686007, EPI_ISL_686008, EPI_ISL_686009, EPI_ISL_686010, EPI_ISL_686011, EPI_ISL_686012, EPI_ISL_686013, EPI_ISL_686014, EPI_ISL_686015, EPI_ISL_686016, EPI_ISL_686017, EPI_ISL_686018, EPI_ISL_686019, EPI_ISL_686020, EPI_ISL_686021, EPI_ISL_686022, EPI_ISL_686023, EPI_ISL_686024, EPI_ISL_686025, EPI_ISL_686026, EPI_ISL_686027, EPI_ISL_686028, EPI_ISL_686029, EPI_ISL_686030, EPI_ISL_686031, EPI_ISL_686032, EPI_ISL_686033, EPI_ISL_686034, EPI_ISL_686035, EPI_ISL_686036, EPI_ISL_686037, EPI_ISL_686038, EPI_ISL_686039, EPI_ISL_686040, EPI_ISL_686041, EPI_ISL_686042, EPI_ISL_686043, EPI_ISL_686044, EPI_ISL_686045, EPI_ISL_686046, EPI_ISL_686047, EPI_ISL_686048, EPI_ISL_686049, EPI_ISL_686050, EPI_ISL_686051, EPI_ISL_686052, EPI_ISL_686053, EPI_ISL_686054, EPI_ISL_686055, EPI_ISL_686056, EPI_ISL_686057, EPI_ISL_686058, EPI_ISL_686059, EPI_ISL_686060, EPI_ISL_686061, EPI_ISL_686062, EPI_ISL_686063, EPI_ISL_686064, EPI_ISL_686065, EPI_ISL_686066, EPI_ISL_686067, EPI_ISL_686068, EPI_ISL_686069, EPI_ISL_686070, EPI_ISL_686071, EPI_ISL_686072, EPI_ISL_686073, EPI_ISL_686074, EPI_ISL_686075			
see above	Pathogen Genomics Center, National Institute of Infectious Diseases	Pathogen Genomics Center, National Institute of Infectious Diseases	Tsuyoshi Sekizuka, Kentaro Itokawa, Rina Tanaka, Masanori Hashino, Makoto Kuroda
EPI_ISL_686083, EPI_ISL_686084, EPI_ISL_686085, EPI_ISL_686086, EPI_ISL_686087, EPI_ISL_686088, EPI_ISL_686089, EPI_ISL_686090, EPI_ISL_686091, EPI_ISL_686092, EPI_ISL_686093, EPI_ISL_686094, EPI_ISL_686095, EPI_ISL_686096, EPI_ISL_686097, EPI_ISL_686098, EPI_ISL_686099, EPI_ISL_686100, EPI_ISL_686101, EPI_ISL_686102, EPI_ISL_686103, EPI_ISL_686104, EPI_ISL_686105, EPI_ISL_686106, EPI_ISL_686107			
see above	Saitama Prefectural Institute of Public Health	Pathogen Genomics Center, National Institute of Infectious Diseases	Tsuyoshi Sekizuka, Kentaro Itokawa, Rina Tanaka, Masanori Hashino, Makoto Kuroda
EPI_ISL_686845, EPI_ISL_686846, EPI_ISL_686847, EPI_ISL_686848, EPI_ISL_686849, EPI_ISL_686850, EPI_ISL_686851, EPI_ISL_686852, EPI_ISL_686853, EPI_ISL_686854, EPI_ISL_686855, EPI_ISL_686856, EPI_ISL_686857, EPI_ISL_686858, EPI_ISL_686859, EPI_ISL_686860, EPI_ISL_686861, EPI_ISL_686862, EPI_ISL_686863, EPI_ISL_686864, EPI_ISL_686865, EPI_ISL_686866, EPI_ISL_686867, EPI_ISL_686868, EPI_ISL_686869, EPI_ISL_686870, EPI_ISL_686871, EPI_ISL_686872, EPI_ISL_686873			
see above	Pathogen Genomics Center, National Institute of Infectious Diseases	Pathogen Genomics Center, National Institute of Infectious Diseases	Tsuyoshi Sekizuka, Kentaro Itokawa, Rina Tanaka, Masanori Hashino, Makoto Kuroda
EPI_ISL_687043, EPI_ISL_687044, EPI_ISL_687045, EPI_ISL_687046, EPI_ISL_687047, EPI_ISL_687048, EPI_ISL_687049, EPI_ISL_687050, EPI_ISL_687051, EPI_ISL_687052, EPI_ISL_687053, EPI_ISL_687054, EPI_ISL_687055, EPI_ISL_687056, EPI_ISL_687057, EPI_ISL_687058, EPI_ISL_687059, EPI_ISL_687060, EPI_ISL_687061, EPI_ISL_687062, EPI_ISL_687063, EPI_ISL_687064, EPI_ISL_687065, EPI_ISL_687066, EPI_ISL_687067, EPI_ISL_687068, EPI_ISL_687069, EPI_ISL_687070			
see above	Kanagawa Prefectural Institute of Public Health	Pathogen Genomics Center, National Institute of Infectious Diseases	Tsuyoshi Sekizuka, Kentaro Itokawa, Rina Tanaka, Masanori Hashino, Makoto Kuroda
EPI_ISL_687618, EPI_ISL_687921, EPI_ISL_687922, EPI_ISL_687923, EPI_ISL_687924, EPI_ISL_687925, EPI_ISL_687926, EPI_ISL_687927, EPI_ISL_687928, EPI_ISL_687929, EPI_ISL_687930, EPI_ISL_687931, EPI_ISL_688084, EPI_ISL_688085, EPI_ISL_688624, EPI_ISL_688757, EPI_ISL_688758, EPI_ISL_688759, EPI_ISL_688760, EPI_ISL_688761, EPI_ISL_688762, EPI_ISL_688763, EPI_ISL_688764, EPI_ISL_688765, EPI_ISL_688766, EPI_ISL_688767, EPI_ISL_688768, EPI_ISL_688769, EPI_ISL_688770, EPI_ISL_688771, EPI_ISL_688772, EPI_ISL_688773, EPI_ISL_688774, EPI_ISL_688775, EPI_ISL_688776, EPI_ISL_688777, EPI_ISL_688778, EPI_ISL_688779, EPI_ISL_688780, EPI_ISL_688781, EPI_ISL_688782, EPI_ISL_688783, EPI_ISL_688784, EPI_ISL_688785, EPI_ISL_688786, EPI_ISL_688787, EPI_ISL_688788, EPI_ISL_688789, EPI_ISL_688790, EPI_ISL_688791, EPI_ISL_688792, EPI_ISL_688793, EPI_ISL_688794, EPI_ISL_688795, EPI_ISL_688796, EPI_ISL_688797, EPI_ISL_688798, EPI_ISL_688799, EPI_ISL_688800, EPI_ISL_688801, EPI_ISL_688802, EPI_ISL_688803, EPI_ISL_688804, EPI_ISL_688805, EPI_ISL_688806, EPI_ISL_688807, EPI_ISL_688808, EPI_ISL_688809, EPI_ISL_688810, EPI_ISL_688811, EPI_ISL_688812, EPI_ISL_688813			
see above	Pathogen Genomics Center, National Institute of Infectious Diseases	Pathogen Genomics Center, National Institute of Infectious Diseases	Tsuyoshi Sekizuka, Kentaro Itokawa, Rina Tanaka, Masanori Hashino, Makoto Kuroda
EPI_ISL_689047, EPI_ISL_689048, EPI_ISL_689049, EPI_ISL_689050, EPI_ISL_689051, EPI_ISL_689052, EPI_ISL_689053, EPI_ISL_689054, EPI_ISL_689055, EPI_ISL_689056, EPI_ISL_689057, EPI_ISL_689058, EPI_ISL_689059, EPI_ISL_689060, EPI_ISL_689061, EPI_ISL_689062, EPI_ISL_689063, EPI_ISL_689064, EPI_ISL_689065, EPI_ISL_689066, EPI_ISL_689067, EPI_ISL_689068, EPI_ISL_689069, EPI_ISL_689070, EPI_ISL_689071, EPI_ISL_689072, EPI_ISL_689073, EPI_ISL_689074, EPI_ISL_689075, EPI_ISL_689076			
see above	Gunma Prefectural Institute of Public Health and Environmental Sciences	Pathogen Genomics Center, National Institute of Infectious Diseases	Tsuyoshi Sekizuka, Kentaro Itokawa, Rina Tanaka, Masanori Hashino, Makoto Kuroda
EPI_ISL_689643, EPI_ISL_689680	Pathogen Genomics Center, National Institute of Infectious Diseases	Pathogen Genomics Center, National Institute of Infectious Diseases	Tsuyoshi Sekizuka, Kentaro Itokawa, Rina Tanaka, Masanori Hashino, Makoto Kuroda
EPI_ISL_689686, EPI_ISL_689687, EPI_ISL_689688, EPI_ISL_689689, EPI_ISL_689690, EPI_ISL_689691	Gunma Prefectural Institute of Public Health and Environmental Sciences	Pathogen Genomics Center, National Institute of Infectious Diseases	Tsuyoshi Sekizuka, Kentaro Itokawa, Rina Tanaka, Masanori Hashino, Makoto Kuroda
EPI_ISL_690614, EPI_ISL_690615, EPI_ISL_690617	Pathogen Genomics Center, National Institute of Infectious Diseases	Pathogen Genomics Center, National Institute of Infectious Diseases	Tsuyoshi Sekizuka, Kentaro Itokawa, Rina Tanaka, Masanori Hashino, Makoto Kuroda
EPI_ISL_690619	Saitama Prefectural Institute of Public Health	Pathogen Genomics Center, National Institute of Infectious Diseases	Tsuyoshi Sekizuka, Kentaro Itokawa, Rina Tanaka, Masanori Hashino, Makoto Kuroda
EPI_ISL_690620, EPI_ISL_690621, EPI_ISL_690622, EPI_ISL_690623, EPI_ISL_690625	Pathogen Genomics Center, National Institute of Infectious Diseases	Pathogen Genomics Center, National Institute of Infectious Diseases	Tsuyoshi Sekizuka, Kentaro Itokawa, Rina Tanaka, Masanori Hashino, Makoto Kuroda
EPI_ISL_690631	Saitama Prefectural Institute of Public Health	Pathogen Genomics Center, National Institute of Infectious Diseases	Tsuyoshi Sekizuka, Kentaro Itokawa, Rina Tanaka, Masanori Hashino, Makoto Kuroda
EPI_ISL_690636, EPI_ISL_690637	Pathogen Genomics Center, National Institute of Infectious Diseases	Pathogen Genomics Center, National Institute of Infectious Diseases	Tsuyoshi Sekizuka, Kentaro Itokawa, Rina Tanaka, Masanori Hashino, Makoto Kuroda
EPI_ISL_690641, EPI_ISL_690642, EPI_ISL_690643, EPI_ISL_690644, EPI_ISL_690645, EPI_ISL_690646, EPI_ISL_690647, EPI_ISL_690648, EPI_ISL_690649, EPI_ISL_690650, EPI_ISL_690651, EPI_ISL_690652, EPI_ISL_690653, EPI_ISL_690654, EPI_ISL_690655, EPI_ISL_690656, EPI_ISL_690657, EPI_ISL_690658, EPI_ISL_690659, EPI_ISL_690660, EPI_ISL_690661, EPI_ISL_690662, EPI_ISL_690663, EPI_ISL_690664, EPI_ISL_690665, EPI_ISL_690666, EPI_ISL_690667, EPI_ISL_690668, EPI_ISL_690669, EPI_ISL_690670, EPI_ISL_690671, EPI_ISL_690672, EPI_ISL_690673, EPI_ISL_690674, EPI_ISL_690675, EPI_ISL_690676, EPI_ISL_690677, EPI_ISL_690678, EPI_ISL_690679, EPI_ISL_690680			
see above	Saitama Prefectural Institute of Public Health	Pathogen Genomics Center, National Institute of Infectious	Tsuyoshi Sekizuka, Kentaro Itokawa, Rina Tanaka, Masanori Hashino, Makoto Kuroda

		Diseases	
EPI_ISL_690684, EPI_ISL_690685, EPI_ISL_690686, EPI_ISL_690708, EPI_ISL_690709, EPI_ISL_690710, EPI_ISL_690711, EPI_ISL_690712, EPI_ISL_690713, EPI_ISL_690714, EPI_ISL_690715, EPI_ISL_690716, EPI_ISL_690717, EPI_ISL_690718, EPI_ISL_690719, EPI_ISL_690720, EPI_ISL_690721, EPI_ISL_690722, EPI_ISL_690723, EPI_ISL_690724, EPI_ISL_690725, EPI_ISL_690726, EPI_ISL_690727, EPI_ISL_690728, EPI_ISL_690729, EPI_ISL_690730, EPI_ISL_690731, EPI_ISL_690732, EPI_ISL_690733, EPI_ISL_690734, EPI_ISL_690735, EPI_ISL_690736, EPI_ISL_690737, EPI_ISL_690738, EPI_ISL_690739, EPI_ISL_690740, EPI_ISL_690741, EPI_ISL_690742, EPI_ISL_690743, EPI_ISL_690745, EPI_ISL_690746, EPI_ISL_690747, EPI_ISL_690748, EPI_ISL_690749, EPI_ISL_690750, EPI_ISL_690751, EPI_ISL_690752, EPI_ISL_690753, EPI_ISL_690754, EPI_ISL_690755, EPI_ISL_690756, EPI_ISL_690757, EPI_ISL_690758, EPI_ISL_690759, EPI_ISL_690760, EPI_ISL_690761, EPI_ISL_690762, EPI_ISL_690763, EPI_ISL_690764, EPI_ISL_690765, EPI_ISL_690766, EPI_ISL_690767, EPI_ISL_690768, EPI_ISL_690769, EPI_ISL_690770, EPI_ISL_690771, EPI_ISL_690772, EPI_ISL_690773, EPI_ISL_690774, EPI_ISL_690775, EPI_ISL_690776, EPI_ISL_690777, EPI_ISL_690779, EPI_ISL_690780, EPI_ISL_690781, EPI_ISL_690782, EPI_ISL_690783, EPI_ISL_690784, EPI_ISL_690785, EPI_ISL_690786, EPI_ISL_690787, EPI_ISL_690788, EPI_ISL_690789, EPI_ISL_690790, EPI_ISL_690791			
see above	Pathogen Genomics Center, National Institute of Infectious Diseases	Pathogen Genomics Center, National Institute of Infectious Diseases	Tsuyoshi Sekizuka, Kentaro Itokawa, Rina Tanaka, Masanori Hashino, Makoto Kuroda
EPI_ISL_690792, EPI_ISL_690793	Tokyo Metropolitan Institute of Public Health	Pathogen Genomics Center, National Institute of Infectious Diseases	Tsuyoshi Sekizuka, Kentaro Itokawa, Rina Tanaka, Masanori Hashino, Makoto Kuroda
EPI_ISL_690818, EPI_ISL_690819, EPI_ISL_690820, EPI_ISL_690821, EPI_ISL_690822, EPI_ISL_690823, EPI_ISL_690824, EPI_ISL_690825, EPI_ISL_690826, EPI_ISL_690827, EPI_ISL_690828, EPI_ISL_690829, EPI_ISL_690830, EPI_ISL_690831, EPI_ISL_690832, EPI_ISL_690833, EPI_ISL_690834, EPI_ISL_690835, EPI_ISL_690836			
see above	Kanagawa Prefectural Institute of Public Health	Pathogen Genomics Center, National Institute of Infectious Diseases	Tsuyoshi Sekizuka, Kentaro Itokawa, Rina Tanaka, Masanori Hashino, Makoto Kuroda
EPI_ISL_690837, EPI_ISL_690838, EPI_ISL_690839, EPI_ISL_690840, EPI_ISL_690841, EPI_ISL_690842, EPI_ISL_690843	Sakai City Institute of Public Health	Pathogen Genomics Center, National Institute of Infectious Diseases	Tsuyoshi Sekizuka, Kentaro Itokawa, Rina Tanaka, Masanori Hashino, Makoto Kuroda
EPI_ISL_690845, EPI_ISL_690846, EPI_ISL_690847, EPI_ISL_690848, EPI_ISL_690849, EPI_ISL_690850, EPI_ISL_690851, EPI_ISL_690852, EPI_ISL_690853, EPI_ISL_690854, EPI_ISL_690855, EPI_ISL_690856, EPI_ISL_690857, EPI_ISL_690858, EPI_ISL_690859, EPI_ISL_690860, EPI_ISL_690861, EPI_ISL_690862, EPI_ISL_690863, EPI_ISL_690864, EPI_ISL_690865, EPI_ISL_690866, EPI_ISL_690867, EPI_ISL_690868, EPI_ISL_690869, EPI_ISL_690870, EPI_ISL_690871, EPI_ISL_690872, EPI_ISL_690873, EPI_ISL_690874, EPI_ISL_690875, EPI_ISL_690876, EPI_ISL_690877, EPI_ISL_690878, EPI_ISL_690879, EPI_ISL_690880, EPI_ISL_690881, EPI_ISL_690882, EPI_ISL_690883, EPI_ISL_690884, EPI_ISL_690885, EPI_ISL_690886, EPI_ISL_690887, EPI_ISL_690888, EPI_ISL_690889, EPI_ISL_690890, EPI_ISL_690891, EPI_ISL_690892, EPI_ISL_690893, EPI_ISL_690894, EPI_ISL_690895, EPI_ISL_690896, EPI_ISL_690897, EPI_ISL_690898, EPI_ISL_690899, EPI_ISL_690900, EPI_ISL_690901, EPI_ISL_690909, EPI_ISL_690910, EPI_ISL_690911, EPI_ISL_690912, EPI_ISL_690913, EPI_ISL_690914, EPI_ISL_690915, EPI_ISL_690916, EPI_ISL_690917, EPI_ISL_690918, EPI_ISL_690919, EPI_ISL_690920, EPI_ISL_690921, EPI_ISL_690922, EPI_ISL_690923, EPI_ISL_690924, EPI_ISL_690925, EPI_ISL_690926, EPI_ISL_690927, EPI_ISL_690928, EPI_ISL_690929, EPI_ISL_690930, EPI_ISL_690931, EPI_ISL_690932, EPI_ISL_690933, EPI_ISL_690934, EPI_ISL_690935, EPI_ISL_690936, EPI_ISL_690937, EPI_ISL_690938, EPI_ISL_690939, EPI_ISL_690940, EPI_ISL_690941, EPI_ISL_690942, EPI_ISL_690943, EPI_ISL_690944, EPI_ISL_690945, EPI_ISL_690946, EPI_ISL_690947, EPI_ISL_690948, EPI_ISL_690949, EPI_ISL_690950, EPI_ISL_690951, EPI_ISL_690952, EPI_ISL_690953, EPI_ISL_690954, EPI_ISL_690955, EPI_ISL_690956, EPI_ISL_690957, EPI_ISL_690958, EPI_ISL_690959, EPI_ISL_690960, EPI_ISL_690961, EPI_ISL_690962, EPI_ISL_690963, EPI_ISL_690964, EPI_ISL_690965, EPI_ISL_690966, EPI_ISL_690967, EPI_ISL_690968, EPI_ISL_690969, EPI_ISL_690970, EPI_ISL_690971, EPI_ISL_690972, EPI_ISL_690973, EPI_ISL_690974, EPI_ISL_690975, EPI_ISL_690976, EPI_ISL_690977, EPI_ISL_690978, EPI_ISL_690979, EPI_ISL_690980			
see above	Pathogen Genomics Center, National Institute of Infectious Diseases	Pathogen Genomics Center, National Institute of Infectious Diseases	Tsuyoshi Sekizuka, Kentaro Itokawa, Rina Tanaka, Masanori Hashino, Makoto Kuroda
EPI_ISL_690982, EPI_ISL_690983, EPI_ISL_690984, EPI_ISL_690985, EPI_ISL_690986	Fukuoka Institute of Health and Environmental Sciences	Pathogen Genomics Center, National Institute of Infectious Diseases	Tsuyoshi Sekizuka, Kentaro Itokawa, Rina Tanaka, Masanori Hashino, Makoto Kuroda
EPI_ISL_690987, EPI_ISL_690988, EPI_ISL_690989, EPI_ISL_690990, EPI_ISL_690991, EPI_ISL_690992, EPI_ISL_690993, EPI_ISL_690994, EPI_ISL_690995, EPI_ISL_690996, EPI_ISL_690997, EPI_ISL_690998, EPI_ISL_690999, EPI_ISL_691000, EPI_ISL_691001, EPI_ISL_691002, EPI_ISL_691003, EPI_ISL_691004, EPI_ISL_691005, EPI_ISL_691006, EPI_ISL_691007, EPI_ISL_691008, EPI_ISL_691009, EPI_ISL_691010, EPI_ISL_691011, EPI_ISL_691012, EPI_ISL_691013, EPI_ISL_691014, EPI_ISL_691015, EPI_ISL_691016, EPI_ISL_691017, EPI_ISL_691018, EPI_ISL_691019, EPI_ISL_691020, EPI_ISL_691021, EPI_ISL_691022, EPI_ISL_691023, EPI_ISL_691024, EPI_ISL_691025, EPI_ISL_691026, EPI_ISL_691027, EPI_ISL_691028, EPI_ISL_691029, EPI_ISL_691030, EPI_ISL_691031, EPI_ISL_691032, EPI_ISL_691033, EPI_ISL_691034, EPI_ISL_691035, EPI_ISL_691036, EPI_ISL_691037, EPI_ISL_691038, EPI_ISL_691039, EPI_ISL_691040, EPI_ISL_691041, EPI_ISL_691042, EPI_ISL_691043, EPI_ISL_691044, EPI_ISL_691045, EPI_ISL_691046, EPI_ISL_691047, EPI_ISL_691048, EPI_ISL_691049, EPI_ISL_691050, EPI_ISL_691051, EPI_ISL_691052, EPI_ISL_691053, EPI_ISL_691054, EPI_ISL_691055, EPI_ISL_691056, EPI_ISL_691057, EPI_ISL_691058, EPI_ISL_691059, EPI_ISL_691060, EPI_ISL_691061, EPI_ISL_691062, EPI_ISL_691063, EPI_ISL_691064, EPI_ISL_691065, EPI_ISL_691066, EPI_ISL_691067, EPI_ISL_691068, EPI_ISL_691069, EPI_ISL_691070, EPI_ISL_691071, EPI_ISL_691072, EPI_ISL_691073, EPI_ISL_691074, EPI_ISL_691075, EPI_ISL_691076, EPI_ISL_691077, EPI_ISL_691078, EPI_ISL_691079, EPI_ISL_691080, EPI_ISL_691081, EPI_ISL_691082, EPI_ISL_691083, EPI_ISL_691084, EPI_ISL_691085, EPI_ISL_691086, EPI_ISL_691087, EPI_ISL_691088, EPI_ISL_691089, EPI_ISL_691090, EPI_ISL_691091, EPI_ISL_691092, EPI_ISL_691093, EPI_ISL_691094, EPI_ISL_691095, EPI_ISL_691096, EPI_ISL_691097, EPI_ISL_691098, EPI_ISL_691099, EPI_ISL_691100, EPI_ISL_691101, EPI_ISL_691102, EPI_ISL_691103, EPI_ISL_691104, EPI_ISL_691105, EPI_ISL_691106, EPI_ISL_691107, EPI_ISL_691109, EPI_ISL_691110, EPI_ISL_691111, EPI_ISL_691112, EPI_ISL_691113, EPI_ISL_691114, EPI_ISL_691115, EPI_ISL_691116, EPI_ISL_691117, EPI_ISL_691118, EPI_ISL_691119, EPI_ISL_691120, EPI_ISL_691121, EPI_ISL_691122, EPI_ISL_691123, EPI_ISL_691124, EPI_ISL_691125, EPI_ISL_691126, EPI_ISL_691127, EPI_ISL_691128, EPI_ISL_691129, EPI_ISL_691130, EPI_ISL_691131, EPI_ISL_691132, EPI_ISL_691133, EPI_ISL_691134, EPI_ISL_691135, EPI_ISL_691136, EPI_ISL_691137, EPI_ISL_691138, EPI_ISL_691139, EPI_ISL_691140, EPI_ISL_691141, EPI_ISL_691142, EPI_ISL_691143, EPI_ISL_691144, EPI_ISL_691145, EPI_ISL_691146, EPI_ISL_691147, EPI_ISL_691148, EPI_ISL_691149, EPI_ISL_691150, EPI_ISL_691151, EPI_ISL_691152, EPI_ISL_691153, EPI_ISL_691154, EPI_ISL_691155, EPI_ISL_691156, EPI_ISL_691157, EPI_ISL_691158, EPI_ISL_691159, EPI_ISL_691160, EPI_ISL_691161, EPI_ISL_691162, EPI_ISL_691163, EPI_ISL_691164, EPI_ISL_691165, EPI_ISL_691166, EPI_ISL_691167, EPI_ISL_691168, EPI_ISL_691169, EPI_ISL_691170, EPI_ISL_691171, EPI_ISL_691172, EPI_ISL_691173, EPI_ISL_691174, EPI_ISL_691175, EPI_ISL_691176, EPI_ISL_691177, EPI_ISL_691178, EPI_ISL_691179, EPI_ISL_691180, EPI_ISL_691181, EPI_ISL_691182, EPI_ISL_691183, EPI_ISL_691184, EPI_ISL_691185, EPI_ISL_691186, EPI_ISL_691187, EPI_ISL_691188, EPI_ISL_691189, EPI_ISL_691190, EPI_ISL_691191, EPI_ISL_691192, EPI_ISL_691193, EPI_ISL_691194, EPI_ISL_691195, EPI_ISL_691196, EPI_ISL_691197, EPI_ISL_691198, EPI_ISL_691199, EPI_ISL_691200, EPI_ISL_691201, EPI_ISL_691202, EPI_ISL_691203, EPI_ISL_691204, EPI_ISL_691205, EPI_ISL_691206, EPI_ISL_691207, EPI_ISL_691208, EPI_ISL_691209, EPI_ISL_691210, EPI_ISL_691211, EPI_ISL_691212, EPI_ISL_691213, EPI_ISL_691214, EPI_ISL_691215, EPI_ISL_691216, EPI_ISL_691217, EPI_ISL_691218, EPI_ISL_691219, EPI_ISL_691220, EPI_ISL_691221, EPI_ISL_691222, EPI_ISL_691223, EPI_ISL_691224, EPI_ISL_691225, EPI_ISL_691226, EPI_ISL_691227, EPI_ISL_691228, EPI_ISL_691229, EPI_ISL_691230, EPI_ISL_691231, EPI_ISL_691232, EPI_ISL_691233, EPI_ISL_691234, EPI_ISL_691235, EPI_ISL_691236, EPI_ISL_691237, EPI_ISL_691238, EPI_ISL_691240, EPI_ISL_691241, EPI_ISL_691242, EPI_ISL_691243, EPI_ISL_691244, EPI_ISL_691245, EPI_ISL_691246, EPI_ISL_691247, EPI_ISL_691249, EPI_ISL_691250, EPI_ISL_691251, EPI_ISL_691257, EPI_ISL_691258, EPI_ISL_691259, EPI_ISL_691273, EPI_ISL_691274, EPI_ISL_691289, EPI_ISL_691290, EPI_ISL_691291, EPI_ISL_691292, EPI_ISL_691293, EPI_ISL_691294, EPI_ISL_691295, EPI_ISL_691296, EPI_ISL_691297, EPI_ISL_691298, EPI_ISL_691299, EPI_ISL_691300, EPI_ISL_691301, EPI_ISL_691302, EPI_ISL_691303, EPI_ISL_691304, EPI_ISL_691305, EPI_ISL_691306, EPI_ISL_691307, EPI_ISL_691308, EPI_ISL_691309, EPI_ISL_691310, EPI_ISL_691311, EPI_ISL_691312, EPI_ISL_691313, EPI_ISL_691314, EPI_ISL_691315, EPI_ISL_691316, EPI_ISL_691317, EPI_ISL_691318, EPI_ISL_691319, EPI_ISL_691320, EPI_ISL_691321, EPI_ISL_691322, EPI_ISL_691323, EPI_ISL_691324, EPI_ISL_691325, EPI_ISL_691326, EPI_ISL_691327, EPI_ISL_691328, EPI_ISL_691329, EPI_ISL_691330, EPI_ISL_691331, EPI_ISL_691332, EPI_ISL_691333, EPI_ISL_691334, EPI_ISL_691335, EPI_ISL_691336, EPI_ISL_691337, EPI_ISL_691338, EPI_ISL_691339, EPI_ISL_691340, EPI_ISL_691341, EPI_ISL_691342, EPI_ISL_691343, EPI_ISL_691344, EPI_ISL_691345, EPI_ISL_691346, EPI_ISL_691347, EPI_ISL_691348, EPI_ISL_691349, EPI_ISL_691350, EPI_ISL_691351, EPI_ISL_691352, EPI_ISL_691353, EPI_ISL_691354, EPI_ISL_691355, EPI_ISL_691356, EPI_ISL_691357, EPI_ISL_691358, EPI_ISL_691359, EPI_ISL_691360, EPI_ISL_691361, EPI_ISL_691362, EPI_ISL_691363, EPI_ISL_691379, EPI_ISL_691380, EPI_ISL_691381, EPI_ISL_691382, EPI_ISL_691383, EPI_ISL_691384, EPI_ISL_691385, EPI_ISL_691395, EPI_ISL_691396, EPI_ISL_691397, EPI_ISL_691398, EPI_ISL_691399, EPI_ISL_691400, EPI_ISL_691401, EPI_ISL_691402, EPI_ISL_691403, EPI_ISL_691404, EPI_ISL_691405, EPI_ISL_691406, EPI_ISL_691407, EPI_ISL_691408, EPI_ISL_691409, EPI_ISL_691410, EPI_ISL_691411, EPI_ISL_691412, EPI_ISL_691413, EPI_ISL_691414, EPI_ISL_691415, EPI_ISL_691416, EPI_ISL_691417, EPI_ISL_691418, EPI_ISL_691419, EPI_ISL_691420, EPI_ISL_691421, EPI_ISL_691422, EPI_ISL_691423, EPI_ISL_691424, EPI_ISL_691425, EPI_ISL_691426, EPI_ISL_691427, EPI_ISL_691428, EPI_ISL_691429, EPI_ISL_691430, EPI_ISL_691431, EPI_ISL_691432, EPI_ISL_691433, EPI_ISL_691434, EPI_ISL_691435, EPI_ISL_691436, EPI_ISL_691437, EPI_ISL_691438, EPI_ISL_691439, EPI_ISL_691440, EPI_ISL_691441, EPI_ISL_691442, EPI_ISL_691443, EPI_ISL_691444, EPI_ISL_691445, EPI_ISL_691446, EPI_ISL_691447, EPI_ISL_691448, EPI_ISL_691449, EPI_ISL_691450, EPI_ISL_691451, EPI_ISL_691452, EPI_ISL_691453, EPI_ISL_691454, EPI_ISL_691455, EPI_ISL_691456, EPI_ISL_691457, EPI_ISL_691458, EPI_ISL_691459, EPI_ISL_691460, EPI_ISL_691461, EPI_ISL_691462, EPI_ISL_691463, EPI_ISL_691464, EPI_ISL_691465, EPI_ISL_691466, EPI_ISL_691467, EPI_ISL_691468, EPI_ISL_691469, EPI_ISL_691470, EPI_ISL_691471, EPI_ISL_691472, EPI_ISL_691473, EPI_ISL_691474, EPI_ISL_691475, EPI_ISL_691476, EPI_ISL_691477, EPI_ISL_691478, EPI_ISL_691479, EPI_ISL_691480, EPI_ISL_691481, EPI_ISL_691482, EPI_ISL_691483, EPI_ISL_691484, EPI_ISL_691485, EPI_ISL_691486, EPI_ISL_691487, EPI_ISL_691488, EPI_ISL_691489, EPI_ISL_691490, EPI_ISL_691491, EPI_ISL_691492, EPI_ISL_691493, EPI_ISL_691494, EPI_ISL_691495, EPI_ISL_691496, EPI_ISL_691497, EPI_ISL_691498, EPI_ISL_691499, EPI_ISL_691500, EPI_ISL_691501, EPI_ISL_691507, EPI_ISL_691508			
see above	Pathogen Genomics Center, National Institute of Infectious Diseases	Pathogen Genomics Center, National Institute of Infectious Diseases	Tsuyoshi Sekizuka, Kentaro Itokawa, Rina Tanaka, Masanori Hashino, Makoto Kuroda
EPI_ISL_691509, EPI_ISL_691510, EPI_ISL_691511, EPI_ISL_691512, EPI_ISL_691513, EPI_ISL_691514, EPI_ISL_691515, EPI_ISL_691516, EPI_ISL_691517, EPI_ISL_691518, EPI_ISL_691519, EPI_ISL_691520, EPI_ISL_691521, EPI_ISL_691522, EPI_ISL_691523, EPI_ISL_691524, EPI_ISL_691525, EPI_ISL_691526, EPI_ISL_691527, EPI_ISL_691528, EPI_ISL_691529, EPI_ISL_691530, EPI_ISL_691531, EPI_ISL_691532, EPI_ISL_691533, EPI_ISL_691534, EPI_ISL_691535, EPI_ISL_691536, EPI_ISL_691537, EPI_ISL_691538, EPI_ISL_691539, EPI_ISL_691540, EPI_ISL_691541, EPI_ISL_691542			
see above	Sakai City Institute of Public Health	Pathogen Genomics Center, National Institute of Infectious Diseases	Tsuyoshi Sekizuka, Kentaro Itokawa, Rina Tanaka, Masanori Hashino, Makoto Kuroda
EPI_ISL_691681	Consejeria de Sanidad y Asuntos Sociales	Instituto de Salud Carlos III	Iglesias-Caballero, M. Camarero, S. Molinero Calamita, M. González-Esguevillas, M. Pozo, F. Casas, I. Jiménez, P. Jiménez, M. Zaballos, A. Monzón, S. Varona, S. Juliá, M. Cuesta, I. Gutiérrez, G.
EPI_ISL_691682	Hospital General Universitario de Ciudad Real	Instituto de Salud Carlos III	Iglesias-Caballero, M. Camarero, S. Molinero Calamita, M. González-Esguevillas, M. Pozo, F. Casas, I. Jiménez, P. Jiménez, M. Zaballos, A. Monzón, S. Varona, S. Juliá, M. Cuesta, I. Jarilla, M.
EPI_ISL_691683	Hospital General Universitario de Ciudad Real	Instituto de Salud Carlos III	Iglesias-Caballero, M. Camarero, S. Molinero Calamita, M. González-Esguevillas, M. Pozo, F. Casas, I. Jiménez, P. Jiménez, M. Zaballos, A. Monzón, S. Varona, S. Juliá, M. Cuesta, I. Madrigal, M.
EPI_ISL_691684	Complejo Hospitalario Universitario de Albacete	Instituto de Salud Carlos III	Iglesias-Caballero, M. Camarero, S. Molinero Calamita, M. González-Esguevillas, M. Pozo, F. Casas, I. Jiménez, P. Jiménez, M. Zaballos, A. Monzón, S. Varona, S. Juliá, M. Cuesta, I. Martínez, E.
EPI_ISL_691757, EPI_ISL_691758, EPI_ISL_691767, EPI_ISL_691861, EPI_ISL_691872, EPI_ISL_691873, EPI_ISL_691874, EPI_ISL_691875, EPI_ISL_691876, EPI_ISL_691877, EPI_ISL_691878, EPI_ISL_691879, EPI_ISL_691880, EPI_ISL_691881, EPI_ISL_691882, EPI_ISL_691883, EPI_ISL_691884, EPI_ISL_691885, EPI_ISL_691886, EPI_ISL_691887, EPI_ISL_691888, EPI_ISL_691889, EPI_ISL_691890, EPI_ISL_691891, EPI_ISL_691892, EPI_ISL_691893, EPI_ISL_691894, EPI_ISL_691895, EPI_ISL_691896, EPI_ISL_691897, EPI_ISL_691898, EPI_ISL_691899, EPI_ISL_691900, EPI_ISL_691901, EPI_ISL_691902, EPI_ISL_691903, EPI_ISL_691904, EPI_ISL_691905, EPI_ISL_691906, EPI_ISL_691907, EPI_ISL_691908, EPI_ISL_691909, EPI_ISL_691910, EPI_ISL_691911, EPI_ISL_691912, EPI_ISL_691913, EPI_ISL_691914, EPI_ISL_691915, EPI_ISL_691916, EPI_ISL_691917, EPI_ISL_691918, EPI_ISL_691919, EPI_ISL_691920, EPI_ISL_691921, EPI_ISL_691922, EPI_ISL_691923, EPI_ISL_691924, EPI_ISL_691925, EPI_ISL_691926, EPI_ISL_691927, EPI_ISL_691928, EPI_ISL_691929, EPI_ISL_691930, EPI_ISL_691931, EPI_ISL_691932, EPI_ISL_691933, EPI_ISL_691934, EPI_ISL_691935, EPI_ISL_691936, EPI_ISL_691937, EPI_ISL_691938, EPI_ISL_691939, EPI_ISL_691940, EPI_ISL_691941, EPI_ISL_691942, EPI_ISL_691943, EPI_ISL_691944, EPI_ISL_691945, EPI_ISL_691946, EPI_ISL_691947, EPI_ISL_691948, EPI_ISL_691949, EPI_ISL_691950, EPI_ISL_691951, EPI_ISL_691952, EPI_ISL_691953, EPI_ISL_691954, EPI_ISL_691955, EPI_ISL_691956, EPI_ISL_691957, EPI_ISL_691958, EPI_ISL_691959, EPI_ISL_691960, EPI_ISL_691961, EPI_ISL_691962, EPI_ISL_691963, EPI_ISL_691964, EPI_ISL_691965, EPI_ISL_691966, EPI_ISL_691967, EPI_ISL_691968, EPI_ISL_691969, EPI_ISL_691970, EPI_ISL_691971, EPI_ISL_691972, EPI_ISL_691973, EPI_ISL_691974, EPI_ISL_691975, EPI_ISL_691976, EPI_ISL_691977, EPI_ISL_691978, EPI_ISL_691979, EPI_ISL_691980, EPI_ISL_691981, EPI_ISL_691982, EPI_ISL_691983, EPI_ISL_691984, EPI_ISL_691985, EPI_ISL_691986, EPI_ISL_691987, EPI_ISL_691988, EPI_ISL_691989, EPI_ISL_691990, EPI_ISL_691991, EPI_ISL_691992, EPI_ISL_691993,			

EPI_ISL_691994, EPI_ISL_691995, EPI_ISL_691996, EPI_ISL_691997, EPI_ISL_691998, EPI_ISL_691999, EPI_ISL_692000, EPI_ISL_692001, EPI_ISL_692002, EPI_ISL_692003, EPI_ISL_692004, EPI_ISL_692005, EPI_ISL_692006, EPI_ISL_692007, EPI_ISL_692008, EPI_ISL_692009, EPI_ISL_692010, EPI_ISL_692011, EPI_ISL_692012, EPI_ISL_692013, EPI_ISL_692014, EPI_ISL_692015, EPI_ISL_692016, EPI_ISL_692017, EPI_ISL_692018, EPI_ISL_692019, EPI_ISL_692020, EPI_ISL_692021, EPI_ISL_692022, EPI_ISL_692023, EPI_ISL_692024, EPI_ISL_692025, EPI_ISL_692026, EPI_ISL_692027, EPI_ISL_692028, EPI_ISL_692029, EPI_ISL_692030, EPI_ISL_692031, EPI_ISL_692032, EPI_ISL_692033, EPI_ISL_692034, EPI_ISL_692035, EPI_ISL_692036, EPI_ISL_692037, EPI_ISL_692038, EPI_ISL_692039, EPI_ISL_692040, EPI_ISL_692041, EPI_ISL_692042, EPI_ISL_692043, EPI_ISL_692044, EPI_ISL_692045, EPI_ISL_692046, EPI_ISL_692059, EPI_ISL_692060, EPI_ISL_692197, EPI_ISL_692527, EPI_ISL_692597			
see above	Pathogen Genomics Center, National Institute of Infectious Diseases	Pathogen Genomics Center, National Institute of Infectious Diseases	Tsuyoshi Sekizuka, Kentaro Itokawa, Rina Tanaka, Masanori Hashino, Makoto Kuroda
EPI_ISL_693195	Hospital e Pronto Socorro Portinari	Instituto Adolfo Lutz, Interdiciplinary Procedures Center, Strategic Laboratory	Claudio Tavares Sacchi, Claudia Regina Gonçalves, Erica Valessa Ramos Gomes, Karoline Rodrigues Campos
EPI_ISL_693196	Hospital Santa Clara	Instituto Adolfo Lutz, Interdiciplinary Procedures Center, Strategic Laboratory	Claudio Tavares Sacchi, Claudia Regina Gonçalves, Erica Valessa Ramos Gomes, Karoline Rodrigues Campos
EPI_ISL_693198	Santa Casa de Misericordia de Sao Paulo - Hospital Central	Instituto Adolfo Lutz, Interdiciplinary Procedures Center, Strategic Laboratory	Claudio Tavares Sacchi, Claudia Regina Gonçalves, Erica Valessa Ramos Gomes, Karoline Rodrigues Campos
EPI_ISL_693200	Hospital e Maternidade Mairipora	Instituto Adolfo Lutz, Interdiciplinary Procedures Center, Strategic Laboratory	Claudio Tavares Sacchi, Claudia Regina Gonçalves, Erica Valessa Ramos Gomes, Karoline Rodrigues Campos
EPI_ISL_693201	Hospital Sao Paulo de Ensino da Unifesp	Instituto Adolfo Lutz, Interdiciplinary Procedures Center, Strategic Laboratory	Claudio Tavares Sacchi, Claudia Regina Gonçalves, Erica Valessa Ramos Gomes, Karoline Rodrigues Campos
EPI_ISL_693202	Pronto Socorro Municipal Prof. Joao Catarin Mezomo	Instituto Adolfo Lutz, Interdiciplinary Procedures Center, Strategic Laboratory	Claudio Tavares Sacchi, Claudia Regina Gonçalves, Erica Valessa Ramos Gomes, Karoline Rodrigues Campos
EPI_ISL_693203	Hospital Municipal Doutor Arthur Ribeiro de Saboya	Instituto Adolfo Lutz, Interdiciplinary Procedures Center, Strategic Laboratory	Claudio Tavares Sacchi, Claudia Regina Gonçalves, Erica Valessa Ramos Gomes, Karoline Rodrigues Campos
EPI_ISL_699656, EPI_ISL_699657	1-Laboratory of Microbiology, National Reference Lab, Charles Nicolle Hospital; 2-University of Tunis ElManar, Faculty of Medicine of Tunis, LR99ES09, Tunis, Tunisia	1-Clinical and Experimental Pharmacology Lab, LR16SP02, National Center of Pharmacovigilance, University of Tunis El Manar, Tunis, Tunisia. 2-Neurodegenerative diseases and psychiatric troubles, LR18SP03, Razi Hospital, University of Tunis El Manar, Tunis, Tunisia. 3- Ministry of Health, National Observatory of New and Emerging Diseases, 1006, Tunis, Tunisia	Ilhem Boutiba-Ben Boubaker, Sameh Trabelsi, Nissaf Ben Alaya, Maher Kharrat, Alia Ben Kahla, Jalila Ben Khelil, Salma Abid, Sana Ferjani, Asma Ferjani, Mouna Ben Sassi, Mouna Safer, Guedi Berrabeh,Salwa Mrabet, Hanen ElJebari, Gaies Emna, Riadh Daghfous, Riadh Gouider.
EPI_ISL_699658, EPI_ISL_699659, EPI_ISL_699660, EPI_ISL_699661, EPI_ISL_699662, EPI_ISL_699663, EPI_ISL_699664, EPI_ISL_699665, EPI_ISL_699666, EPI_ISL_699667, EPI_ISL_699668, EPI_ISL_699669, EPI_ISL_699670, EPI_ISL_699671, EPI_ISL_699672, EPI_ISL_699674, EPI_ISL_699675, EPI_ISL_699676, EPI_ISL_699677, EPI_ISL_699678, EPI_ISL_699680, EPI_ISL_699681, EPI_ISL_699682, EPI_ISL_699683, EPI_ISL_699684, EPI_ISL_699685, EPI_ISL_699686, EPI_ISL_699687, EPI_ISL_699688, EPI_ISL_699691, EPI_ISL_699692, EPI_ISL_699693, EPI_ISL_699694, EPI_ISL_699695, EPI_ISL_699696, EPI_ISL_699697, EPI_ISL_699698, EPI_ISL_699699, EPI_ISL_699700, EPI_ISL_699701, EPI_ISL_699702, EPI_ISL_699703, EPI_ISL_699704, EPI_ISL_699705, EPI_ISL_699706, EPI_ISL_699709, EPI_ISL_699711, EPI_ISL_699712, EPI_ISL_699713, EPI_ISL_699714, EPI_ISL_699715, EPI_ISL_699723			
see above	Hematopathology Laboratory, ACTREC, TMC	Hematopathology Laboratory, ACTREC, TMC	Hematopathology Laboratory, ACTREC
EPI_ISL_700371	CHU Nantes	CNR Virus des Infections Respiratoires - France SUD	Antonin Bal, Louise Castain, Gregory Destras, Gwendolyne Burfin, Hadrien Règue, Quentin Semanas, Martine Valette, Bruno Lina, Virginie Ferré, Celine Bressollette, Laurence Josset
EPI_ISL_707791	1-Laboratory of Microbiology, National Reference Lab, Charles Nicolle Hospital; 2-University of Tunis ElManar, Faculty of Medicine of Tunis, LR99ES09, Tunis, Tunisia	1-Clinical and Experimental Pharmacology Lab, LR16SP02, National Center of Pharmacovigilance, University of Tunis El Manar, Tunis, Tunisia. 2-Neurodegenerative diseases and psychiatric troubles, LR18SP03, Razi Hospital, University of Tunis El Manar, Tunis, Tunisia. 3- Ministry of Health, National Observatory of New and Emerging Diseases, 1006, Tunis, Tunisia	Ilhem Boutiba-Ben Boubaker, Sameh Trabelsi, Nissaf Ben Alaya, Maher Kharrat, Alia Ben Kahla, Jalila Ben Khelil, Salma Abid, Sana Ferjani, Mouna Ben Sassi, Mouna Safer, Awatef El MOussi, Habiba Ben Romdhane, Souissi Amira, Ines Mdini, Hanen El Jebari, Asma Ferjani, Gaies Emna, Riadh Daghfous, Riadh Gouider.
EPI_ISL_718132, EPI_ISL_718133, EPI_ISL_718136, EPI_ISL_718137, EPI_ISL_718138, EPI_ISL_718139, EPI_ISL_718140, EPI_ISL_718141, EPI_ISL_718142, EPI_ISL_718144, EPI_ISL_718145, EPI_ISL_718148, EPI_ISL_718149, EPI_ISL_718150, EPI_ISL_718151, EPI_ISL_718152, EPI_ISL_718153, EPI_ISL_718154, EPI_ISL_718155, EPI_ISL_718157, EPI_ISL_718158, EPI_ISL_718159, EPI_ISL_718160, EPI_ISL_718161, EPI_ISL_718162, EPI_ISL_718163, EPI_ISL_718164, EPI_ISL_718165, EPI_ISL_718166, EPI_ISL_718221			
see above	Ministry of Health Hospitals	Institute of Health and Community Medicine	David Perera, Ooi Mong How, Chua Hock Hin, Tonnii Sia Loong Loong, Wong Jyn Shan, Wong Kiing Aik, Chan Chia Jui
EPI_ISL_718273	Fujita Health University Okazaki Medical Center	Department of Virology and Parasitology, Fujita Health University School of Medicine	Takayuki Murata, Aki Sakurai, Satoshi Komoto, Tomihiko Ide,Takuma Ishihara, Masahiro suzuki, Yohei Doi
EPI_ISL_722899	Dipartimento di Scienze Biomediche e Oncologia Umana - Azienda Ospedaliero Universitaria Consorziale Policlinico	Istituto Zooprofilattico Sperimentale della Puglia e della Basilicata	Parisi A., Bianco A., Capozzi L., Del Sambro L., Chironna M., Loconsole D.
EPI_ISL_727998, EPI_ISL_727999	Fujita Health University Okazaki Medical Center	Department of Virology and Parasitology, Fujita Health University School of Medicine	Takayuki Murata, Yohei Doi, Takuma Ishihara, Masahiro suzuki, Aki Sakurai, Satoshi Komoto, Tomihiko Ide
EPI_ISL_728000	Fujita Health University Okazaki Medical Center	Department of Virology and Parasitology, Fujita Health University School of Medicin	Takayuki Murata, Yohei Doi, Takuma Ishihara, Masahiro suzuki, Aki Sakurai, Satoshi Komoto, Tomihiko Ide
EPI_ISL_728153, EPI_ISL_728154, EPI_ISL_728155	Fujita Health University Okazaki Medical Center	Department of Virology and Parasitology, Fujita Health University School of Medicine	Takayuki Murata, Yohei Doi, Takuma Ishihara, Masahiro suzuki, Aki Sakurai, Satoshi Komoto, Tomihiko Ide
EPI_ISL_728156, EPI_ISL_728159	Fujita Health University Okazaki Medical Center	Department of Virology and Parasitology, Fujita Health University School of Medicine	Takayuki Murata, Aki Sakurai, Satoshi Komoto, Tomihiko Ide,Takuma Ishihara, Masahiro suzuki, Yohei Doi
EPI_ISL_728206, EPI_ISL_728207, EPI_ISL_728208	Fujita Health University Okazaki Medical Center	Fujita health University, Department of Microbiology	Takayuki Murata, Aki Sakurai, Satoshi Komoto, Tomihiko Ide,Takuma Ishihara, Masahiro suzuki, Yohei Doi
EPI_ISL_729797, EPI_ISL_729798	Laboratório Central de Saúde Pública do Estado do Rio Grande do Sul (LACEN-RS)	Laboratory of Respiratory Viruses and Measles, Oswaldo Cruz Institute, FIOCRUZ	Paola Resende, Luciana Appolinario, Fernando Motta, Anna Carolina Paixão, Ana Carolina Mendonça, Tatiana Schaffer Gregianini, Marilda Tereza Mar da Rosa, Marilda Siqueira on behalf of the Fiocruz COVID-19 Genomic Surveillance Network
EPI_ISL_732556, EPI_ISL_732557, EPI_ISL_732558	Bundeswehr Institute of Microbiology	Bundeswehr Institute of Microbiology	Elham Khatamzas, Markus Antwerpen, Mathias Walter, Alexandra Rehn, Sabine Zange, Enrico Georgi, Michael von Bergwelt-Baildon, Roman Wölfel
EPI_ISL_735260, EPI_ISL_735261, EPI_ISL_735262, EPI_ISL_735263, EPI_ISL_735264, EPI_ISL_735265, EPI_ISL_735266, EPI_ISL_735267, EPI_ISL_735268, EPI_ISL_735269, EPI_ISL_735270, EPI_ISL_735271, EPI_ISL_735272, EPI_ISL_735273, EPI_ISL_735274, EPI_ISL_735275, EPI_ISL_735276, EPI_ISL_735277, EPI_ISL_735278, EPI_ISL_735279, EPI_ISL_735280, EPI_ISL_735281, EPI_ISL_735282, EPI_ISL_735283, EPI_ISL_735284, EPI_ISL_735285, EPI_ISL_735286, EPI_ISL_735288, EPI_ISL_735289, EPI_ISL_735290, EPI_ISL_735292, EPI_ISL_735293, EPI_ISL_735294, EPI_ISL_735295, EPI_ISL_735296, EPI_ISL_735297, EPI_ISL_735298, EPI_ISL_735299, EPI_ISL_735300, EPI_ISL_735301, EPI_ISL_735302, EPI_ISL_735303, EPI_ISL_735304, EPI_ISL_735305			
see above	Genomic Laboratory (GLAB) (Conjoint lab of Health Directorate of Istanbul and Istanbul Technical University)	Genomic Laboratory (GLAB), Istanbul Technical University	Ilker Karacan, Tugba Kizilboga Akgun, Nihat Bugra Agaoglu, Payam Zolfagharian, Mehtap Aydin, Gizem Alkurt, Jale Yildiz, Betsi Kose, Nisan Denizce Can, Ayse Serra Ozel, Nilsun Altunal, Arzu Irvem, Yasemin Kendir Demirkol, Ozlem Akgun Dogan, Levent Doganay, Gizem Dinler Doganay
EPI_ISL_735399	Hospital Municiplal Dr Ignacio de gouvea	Instituto Adolfo Lutz, Interdiciplinary Procedures Center, Strategic Laboratory	Claudio Tavares Sacchi, Claudia Regina Gonçalves, Erica Valessa Ramos Gomes, Karoline Rodrigues Campos
EPI_ISL_738147, EPI_ISL_738194, EPI_ISL_738243	Microbiology and Virology Unit, Florence Careggi University Hospital	Microbiology and Virology Unit, Florence Careggi University Hospital	Vincenzo Di Pilato, Marco Coppi, Alberto Antonelli, Simona Pollini, Gian Maria Rossolini
EPI_ISL_751297, EPI_ISL_751301	Devki Devi Foundation, a unit of Max Healthcare	CSIR-IGIB/Max	Rajesh Pandey#, Samreen Siddiqui, Janani Srinivasa Vasudevan, Akshay Kanakan, Ranjeet Maurya, Uzma Shamim, Bansidhar Tarai, Akansha Tyagi, Mitali Mukerji, Poonam Das, Sujeet Jha, Mohammed Faruq, Anurag Agrawal
EPI_ISL_751320, EPI_ISL_751322, EPI_ISL_751333, EPI_ISL_751334, EPI_ISL_751336, EPI_ISL_751347, EPI_ISL_751348, EPI_ISL_751349, EPI_ISL_751350, EPI_ISL_751351, EPI_ISL_751352, EPI_ISL_751353, EPI_ISL_751355, EPI_ISL_751356, EPI_ISL_751358, EPI_ISL_751359, EPI_ISL_751360, EPI_ISL_751361,			

EPI_ISL_751362, EPI_ISL_751363, EPI_ISL_751364, EPI_ISL_751365, EPI_ISL_751366, EPI_ISL_751367, EPI_ISL_751368, EPI_ISL_751369, EPI_ISL_751370, EPI_ISL_751371, EPI_ISL_751372, EPI_ISL_751373, EPI_ISL_751374, EPI_ISL_751375, EPI_ISL_751376, EPI_ISL_751377, EPI_ISL_751378, EPI_ISL_751380, EPI_ISL_751381, EPI_ISL_751382, EPI_ISL_751383, EPI_ISL_751384, EPI_ISL_751386, EPI_ISL_751387, EPI_ISL_751388, EPI_ISL_751389, EPI_ISL_751390, EPI_ISL_751391, EPI_ISL_751392, EPI_ISL_751393, EPI_ISL_751394, EPI_ISL_751395, EPI_ISL_751397, EPI_ISL_751398, EPI_ISL_751399, EPI_ISL_751400, EPI_ISL_751401, EPI_ISL_751402, EPI_ISL_751403, EPI_ISL_751404, EPI_ISL_751405, EPI_ISL_751407, EPI_ISL_751408, EPI_ISL_751409, EPI_ISL_751410, EPI_ISL_751411, EPI_ISL_751413, EPI_ISL_751414, EPI_ISL_751415, EPI_ISL_751416, EPI_ISL_751417, EPI_ISL_751418, EPI_ISL_751419, EPI_ISL_751420, EPI_ISL_751421, EPI_ISL_751422, EPI_ISL_751423, EPI_ISL_751427, EPI_ISL_751429, EPI_ISL_751430, EPI_ISL_751431, EPI_ISL_751435, EPI_ISL_751439, EPI_ISL_751440, EPI_ISL_751442, EPI_ISL_751444, EPI_ISL_751445, EPI_ISL_751446

see above	IRCCS Sacro Cuore Don Calabria Hospital, Department of Infectious, Tropical Diseases & Microbiology	University of Verona, Department of Biotechnology	Antonio Mori, Michela Deiana, Elena Pomari, Chiara Piubelli; Giulia Lopatriello, Luca Marcolungo, Cristina Beltrami, Chiara Degli Esposti, Emanuela Cosentino, Massimo Delledonne
EPI_ISL_759955	Primasatya Husada Citra Hospital	Institute of Tropical Disease, Universitas Airlangga	Aldise M Nastri, Jezzy R Dewantari, Rima R Prasetya, Krisnoadi Rahardjo, Pudji Djanuartono, Gatot Soegiarto, Laksmi Wulandari, Resti Yudhawati, Soetjipto, Yasuko Mori, Maria I Lusida, Kazufumi Shimizu
EPI_ISL_759956	Husada Utama Hospital	Institute of Tropical Disease, Universitas Airlangga	Jezzy R Dewantari, Rima R Prasetya, Krisnoadi Rahardjo, Aldise M Nastri, Didi Dewanto, Gatot Soegiarto, Laksmi Wulandari, Resti Yudhawati, Soetjipto, Yasuko Mori, Maria I Lusida, Kazufumi Shimizu
EPI_ISL_759957	Siti Khodijah Hospital	Institute of Tropical Disease, Universitas Airlangga	Rima R Prasetya, Krisnoadi Rahardjo, Aldise M Nastri, Jezzy R Dewantari, Muhammad Hamdan, Gatot Soegiarto, Laksmi Wulandari, Resti Yudhawati, Soetjipto, Yasuko Mori, Maria I Lusida, Kazufumi Shimizu
EPI_ISL_759958	Universitas Airlangga Hospital	Institute of Tropical Disease, Universitas Airlangga	Maria I Lusida, Krisnoadi Rahardjo, Aldise M Nastri, Jezzy R Dewantari, Rima R Prasetya, Nasronudin, Gatot Soegiarto, Laksmi Wulandari, Resti Yudhawati, Soetjipto, Yasuko Mori, Kazufumi Shimizu
EPI_ISL_759959	Premier Hospital Surabaya	Institute of Tropical Disease, Universitas Airlangga	Krisnoadi Rahardjo, Aldise M Nastri, Jezzy R Dewantari, Rima R Prasetya, Hartono Tanto, Gatot Soegiarto, Laksmi Wulandari, Resti Yudhawati, Soetjipto, Yasuko Mori, Maria I Lusida, Kazufumi Shimizu
EPI_ISL_759961	Universitas Airlangga Hospital	Institute of Tropical Disease, Universitas Airlangga	Jezzy R Dewantari, Rima R Prasetya, Krisnoadi Rahardjo, Aldise M Nastri, Nasronudin, Gatot Soegiarto, Laksmi Wulandari, Resti Yudhawati, Soetjipto, Yasuko Mori, Maria I Lusida, Kazufumi Shimizu
EPI_ISL_759962	RSUD Dr. Soetomo	Institute of Tropical Disease, Universitas Airlangga	Krisnoadi Rahardjo, Aldise M Nastri, Jezzy R Dewantari, Rima R Prasetya, Joni Wahyuhadi, Gatot Soegiarto, Laksmi Wulandari, Resti Yudhawati, Yasuko Mori, Soetjipto, Kazufumi Shimizu ,Maria I Lusida
EPI_ISL_759964	Mitra Keluarga Hospital Waru	Institute of Tropical Disease, Universitas Airlangga	Jezzy R Dewantari, Rima R Prasetya, Krisnoadi Rahardjo, Aldise M Nastri, Christina Dian Anggraeni, Gatot Soegiarto, Laksmi Wulandari, Resti Yudhawati, Yasuko Mori, Soetjipto, Kazufumi Shimizu ,Maria I Lusida
EPI_ISL_759965	RSUD Dr. Soetomo	Institute of Tropical Disease, Universitas Airlangga	Rima R Prasetya, Krisnoadi Rahardjo, Aldise M Nastri, Jezzy R Dewantari, Joni Wahyuhadi, Gatot Soegiarto, Laksmi Wulandari, Resti Yudhawati, Yasuko Mori, Soetjipto, Kazufumi Shimizu ,Maria I Lusida
EPI_ISL_759966	RSUD Sidoarjo	Institute of Tropical Disease, Universitas Airlangga	Krisnoadi Rahardjo, Aldise M Nastri, Jezzy R Dewantari, Rima R Prasetya, Atok Irawan, Gatot Soegiarto, Laksmi Wulandari, Resti Yudhawati, Yasuko Mori, Soetjipto, Kazufumi Shimizu ,Maria I Lusida
EPI_ISL_759967	RSUD Dr. Iskak Tulungagung	Institute of Tropical Disease, Universitas Airlangga	Rima R Prasetya, Krisnoadi Rahardjo, Aldise M Nastri, Jezzy R Dewantari, Supriyanto Dharmoredjo, Gatot Soegiarto, Laksmi Wulandari, Resti Yudhawati, Soetjipto, Yasuko Mori, Maria I Lusida, Kazufumi Shimizu
EPI_ISL_766032	RSUP Dr. Sardjito	Eijkman Institute for Molecular Biology, Ministry of Research and Technology/National Agency for Research and Innovation	Frilasita A Yudhaputri, Edison Johar, Hidayat Trimarsanto, David H Muljono, Safarina G Malik, Khin Saw Myint, Amin Soebandrio
EPI_ISL_766034	RS Mitra Keluarga Bintaro	Eijkman Institute for Molecular Biology, Ministry of Research and Technology/National Agency for Research and Innovation	Frilasita A Yudhaputri, Edison Johar, Hidayat Trimarsanto, David H Muljono, Safarina G Malik, Khin Saw Myint, Amin Soebandrio
EPI_ISL_766035	RS Bhayangkara Brimob Kelapa Dua	Eijkman Institute for Molecular Biology, Ministry of Research and Technology/National Agency for Research and Innovation	Frilasita A Yudhaputri, Edison Johar, Hidayat Trimarsanto, David H Muljono, Safarina G Malik, Khin Saw Myint, Amin Soebandrio
EPI_ISL_766036	RS Mitra Keluarga Bintaro	Eijkman Institute for Molecular Biology, Ministry of Research and Technology/National Agency for Research and Innovation	Frilasita A Yudhaputri, Edison Johar, Hidayat Trimarsanto, David H Muljono, Safarina G Malik, Khin Saw Myint, Amin Soebandrio
EPI_ISL_766037	Puskesmas Palmerah	Eijkman Institute for Molecular Biology, Ministry of Research and Technology/National Agency for Research and Innovation	Frilasita A Yudhaputri, Edison Johar, Hidayat Trimarsanto, David H Muljono, Safarina G Malik, Khin Saw Myint, Amin Soebandrio
EPI_ISL_766038	RSJPD Harapan Kita	Eijkman Institute for Molecular Biology, Ministry of Research and Technology/National Agency for Research and Innovation	Frilasita A Yudhaputri, Edison Johar, Hidayat Trimarsanto, David H Muljono, Safarina G Malik, Khin Saw Myint, Amin Soebandrio
EPI_ISL_766039	RS Bhayangkara Tk. III	Eijkman Institute for Molecular Biology, Ministry of Research and Technology/National Agency for Research and Innovation	Frilasita A Yudhaputri, Edison Johar, Hidayat Trimarsanto, David H Muljono, Safarina G Malik, Khin Saw Myint, Amin Soebandrio
EPI_ISL_766040	RS Tingkat II Moh. Ridwan Meuraksa	Eijkman Institute for Molecular Biology, Ministry of Research and Technology/National Agency for Research and Innovation	Frilasita A Yudhaputri, Edison Johar, Hidayat Trimarsanto, David H Muljono, Safarina G Malik, Khin Saw Myint, Amin Soebandrio
EPI_ISL_766041	RS Polri	Eijkman Institute for Molecular Biology, Ministry of Research and Technology/National Agency for Research and Innovation	Frilasita A Yudhaputri, Edison Johar, Hidayat Trimarsanto, David H Muljono, Safarina G Malik, Khin Saw Myint, Amin Soebandrio
EPI_ISL_766042	RS Sari Asih Ciledug	Eijkman Institute for Molecular Biology, Ministry of Research and Technology/National Agency for Research and Innovation	Frilasita A Yudhaputri, Edison Johar, Hidayat Trimarsanto, David H Muljono, Safarina G Malik, Khin Saw Myint, Amin Soebandrio
EPI_ISL_766043	RS Pelni	Eijkman Institute for Molecular Biology, Ministry of Research and Technology/National Agency for Research and Innovation	Frilasita A Yudhaputri, Edison Johar, Hidayat Trimarsanto, David H Muljono, Safarina G Malik, Khin Saw Myint, Amin Soebandrio
EPI_ISL_766044	RSUP Fatmawati	Eijkman Institute for Molecular Biology, Ministry of Research and Technology/National Agency for Research and Innovation	Frilasita A Yudhaputri, Edison Johar, Hidayat Trimarsanto, David H Muljono, Safarina G Malik, Khin Saw Myint, Amin Soebandrio
EPI_ISL_766045	RS Pelni	Eijkman Institute for Molecular Biology, Ministry of Research and Technology/National Agency for Research and Innovation	Frilasita A Yudhaputri, Edison Johar, Hidayat Trimarsanto, David H Muljono, Safarina G Malik, Khin Saw Myint, Amin Soebandrio
EPI_ISL_766048	RS Bhayangkara Tk. III	Eijkman Institute for Molecular Biology, Ministry of Research and Technology/National Agency for Research and Innovation	Frilasita A Yudhaputri, Edison Johar, Hidayat Trimarsanto, David H Muljono, Safarina G Malik, Khin Saw Myint, Amin Soebandrio
EPI_ISL_767043	University of Bari Biomedical Sciences and Human Oncology	University of Bari Biomedical Sciences and Human Oncology	Maria Chironna, Anna Sallustio, Daniela Loconsole, Marisa Accogli
EPI_ISL_767405	Ochsner Health	BioInfo Experts, LLC	Rebecca Rose, Amy Feehan, David J. Nolan, Sissy Cross, David Moraga Amador, Tong Yang, Luke Caruso, Wayra Navia, Lydia Von Borstel, Xiao Hui Zhou,

			Julia-Garcia-Diaz, Susanna L. Lamers
EPI_ISL_775259	INT Fondazione Pascale	INT Fondazione Pascale	INT Fondazione Pascale
EPI_ISL_776750, EPI_ISL_776751, EPI_ISL_776752, EPI_ISL_776753, EPI_ISL_776754, EPI_ISL_776755, EPI_ISL_776756	Instituto Adolfo Lutz - Central	Instituto Adolfo Lutz, Interdiciplinary Procedures Center, Strategic Laboratory	Claudio Tavares Sacchi, Claudia Regina Gonçalves, Erica Valessa Ramos Gomes, Karoline Rodrigues Campos
EPI_ISL_776757, EPI_ISL_776758	Instituto Adolfo Lutz - Regional de Marilia	Instituto Adolfo Lutz, Interdiciplinary Procedures Center, Strategic Laboratory	Claudio Tavares Sacchi, Claudia Regina Gonçalves, Erica Valessa Ramos Gomes, Karoline Rodrigues Campos
EPI_ISL_776759, EPI_ISL_776760, EPI_ISL_776761, EPI_ISL_776762, EPI_ISL_776763	Instituto Adolfo Lutz - Central	Instituto Adolfo Lutz, Interdiciplinary Procedures Center, Strategic Laboratory	Claudio Tavares Sacchi, Claudia Regina Gonçalves, Erica Valessa Ramos Gomes, Karoline Rodrigues Campos
EPI_ISL_779704, EPI_ISL_779709, EPI_ISL_779712, EPI_ISL_779713, EPI_ISL_779714, EPI_ISL_780378, EPI_ISL_780381, EPI_ISL_780413	Laboratory of Infectious Diseases, Department of Biomedical and Clinical Sciences L. Sacco, University of Milan	Laboratory of Infectious Diseases, Department of Biomedical and Clinical Sciences L. Sacco, University of Milan	Alessia Lai, Annalisa Bergna, Carla Della Ventura, Claudia Balotta, Massimo Galli, Gianguglielmo Zehender on behalf of SARS-CoV-2 ITALIAN RESEARCH ENTERPRISE-(SCIRE) Collaborative Group
EPI_ISL_788944, EPI_ISL_788945, EPI_ISL_788947, EPI_ISL_788962, EPI_ISL_788963, EPI_ISL_788964, EPI_ISL_788966, EPI_ISL_788967	Ospedale "Di Venere"	Beaconlab (Bioinformatics, Evolution and Comparative Genomics lab), Dept of Biosciences, University on Milan	Iacobellis M, d'Avenia M, Piluscio R, Parisi A, Chiara M, Manzari C, Pesole G
EPI_ISL_792101, EPI_ISL_792102	Instituto Adolfo Lutz - Central	Instituto Adolfo Lutz, Interdiciplinary Procedures Center, Strategic Laboratory	Claudio Tavares Sacchi, Claudia Regina Gonçalves, Erica Valessa Ramos Gomes, Karoline Rodrigues Campos
EPI_ISL_792103	Instituto Adolfo Lutz - Regional de Santo Andre	Instituto Adolfo Lutz, Interdiciplinary Procedures Center, Strategic Laboratory	Claudio Tavares Sacchi, Claudia Regina Gonçalves, Erica Valessa Ramos Gomes, Karoline Rodrigues Campos
EPI_ISL_792104, EPI_ISL_792105, EPI_ISL_792106, EPI_ISL_792107, EPI_ISL_792108, EPI_ISL_792109, EPI_ISL_792110, EPI_ISL_792111, EPI_ISL_792112, EPI_ISL_792113, EPI_ISL_792114			
see above	Instituto Adolfo Lutz - Central	Instituto Adolfo Lutz, Interdiciplinary Procedures Center, Strategic Laboratory	Claudio Tavares Sacchi, Claudia Regina Gonçalves, Erica Valessa Ramos Gomes, Karoline Rodrigues Campos
EPI_ISL_794745	Istituto Zooprofilattico Sperimentale della Puglia e della Basilicata	Istituto Zooprofilattico Sperimentale della Puglia e della Basilicata	Parisi A., Bianco A., Capozzi L., Del Sambro L., Manzulli V, Rondinone V., Pace L., Cipolletta D., Galante D.
EPI_ISL_807150	Makmal Kesihatan Awam Kebangsaan, Kementerian Kesihatan Malaysia.	Integrative Pharmacogenomics Institute (iPROMISE)	Mohd Zaki Salleh, Teh Lay Kek, Ariza Adnan, Sazzli Shahlan Kassim, Siti Hamimah Sheikh Abdul Kadir, Fadzilah Mohd Nor, Mohd Nur Fakhruzzaman Noorizhab, Siti Farah Alwani Mohd Nawi, Wang Seok Mui, Hani Mat Hussin, Nor Zahrin Hasran, Lim Wai Feng
EPI_ISL_807151, EPI_ISL_807153	Makmal Kesihatan Awam Kebangsaan, Kementerian Kesihatan Malaysia	Integrative Pharmacogenomics Institute (iPROMISE)	Mohd Zaki Salleh, Teh Lay Kek, Ariza Adnan, Sazzli Shahlan Kassim, Siti Hamimah Sheikh Abdul Kadir, Fadzilah Mohd Nor, Mohd Nur Fakhruzzaman Noorizhab, Siti Farah Alwani Mohd Nawi, Wang Seok Mui, Hani Mat Hussin, Nor Zahrin Hasran, Lim Wai Feng
EPI_ISL_811148	Laboratorio de Ecologia de Doencas Transmissiveis na Amazonia, Instituto Leonidas e Maria Deane - Fiocruz Amazonia	Laboratorio de Ecologia de Doencas Transmissiveis na Amazonia, Instituto Leonidas e Maria Deane - Fiocruz Amazonia	Valdinete Nascimento, Victor Souza, André Corado, Fernanda Nascimento, George Silva, Ágatha Costa, Debora Duarte, Luciana Gonçalves, Matilde Mejía, Karina Pessoa, Maria Júlia Brandão, Michele Jesus, Felipe Naveca on behalf of the Fiocruz COVID-19 Genomic Surveillance Network
EPI_ISL_832178	Hospital	National Reference Center for Viruses of Respiratory Infections, Institut Pasteur, Paris	Marion Barbet, Sylvie Behillil, Méline Bizard, Angela Brisebarre, Camille Capel, Etienne Simon-Lorière, Vincent Enouf, Maud Vanpeene, Sylvie van der Werf, Laurent Roudiere
EPI_ISL_832198	Laboratory Analyses Med	National Reference Center for Viruses of Respiratory Infections, Institut Pasteur, Paris	Marion Barbet, Sylvie Behillil, Méline Bizard, Angela Brisebarre, Camille Capel, Etienne Simon-Lorière, Vincent Enouf, Maud Vanpeene, Sylvie van der Werf, Elise Liscoet
EPI_ISL_832202	Hospital	National Reference Center for Viruses of Respiratory Infections, Institut Pasteur, Paris	Marion Barbet, Sylvie Behillil, Méline Bizard, Angela Brisebarre, Camille Capel, Etienne Simon-Lorière, Vincent Enouf, Maud Vanpeene, Sylvie van der Werf, Patricia Stoessel-Thouvenin
EPI_ISL_833152, EPI_ISL_833153, EPI_ISL_833154, EPI_ISL_833155	Instituto Adolfo Lutz - Central	Instituto Adolfo Lutz, Interdiciplinary Procedures Center, Strategic Laboratory	Claudio Tavares Sacchi, Claudia Regina Gonçalves, Erica Valessa Ramos Gomes, Karoline Rodrigues Campos
EPI_ISL_833200	Hôpital Bichat Claude Bernard, Laboratoire de Virologie	IAME UMR1137 Inserm, Université de Paris, Hôpital Bichat	Antoine Bridier, Amélie Recoing, Quentin Le Hingrat, Lena Daniel, Siham Hamri, Gilles Collin, Alexandre Storto, Mélanie Bertine, Charlotte Charpentier, Nadhira Houhou-Fidouh, Diane Descamps, Benoit Visseaux
EPI_ISL_837565, EPI_ISL_837566, EPI_ISL_837568, EPI_ISL_837570, EPI_ISL_837574, EPI_ISL_837575, EPI_ISL_837577	Centro Nacional de Enfermedades Tropicales (CENETROP)	Laboratory of Respiratory Viruses and Measles, Oswaldo Cruz Institute, FIOCRUZ	Paola Resende, Roxana Loayza, Cinthia Avila, Luciana Appolinario, Fernando Motta, Anna Carolina Paixao, Ana Carolina Mendonca, Marilda Siqueira on behalf of the Fiocruz COVID-19 Genomic Surveillance Network
EPI_ISL_837604, EPI_ISL_837605, EPI_ISL_837606, EPI_ISL_837608, EPI_ISL_837609, EPI_ISL_837610, EPI_ISL_837611, EPI_ISL_837612, EPI_ISL_837613, EPI_ISL_837614, EPI_ISL_837615, EPI_ISL_837616, EPI_ISL_837617, EPI_ISL_837618, EPI_ISL_837619, EPI_ISL_837620, EPI_ISL_837621, EPI_ISL_837622, EPI_ISL_837623, EPI_ISL_837624, EPI_ISL_837625, EPI_ISL_837626, EPI_ISL_837627, EPI_ISL_837628, EPI_ISL_837629, EPI_ISL_837630, EPI_ISL_837631, EPI_ISL_837632, EPI_ISL_837633, EPI_ISL_837634, EPI_ISL_837635, EPI_ISL_837636, EPI_ISL_837637, EPI_ISL_837638, EPI_ISL_837639, EPI_ISL_837640, EPI_ISL_837641, EPI_ISL_837642, EPI_ISL_837643, EPI_ISL_837644, EPI_ISL_837645, EPI_ISL_837646, EPI_ISL_837647, EPI_ISL_837648, EPI_ISL_837649, EPI_ISL_837650, EPI_ISL_837651, EPI_ISL_837652, EPI_ISL_837653, EPI_ISL_837654, EPI_ISL_837655, EPI_ISL_837656, EPI_ISL_837657, EPI_ISL_837658, EPI_ISL_837659, EPI_ISL_837660, EPI_ISL_837661, EPI_ISL_837662, EPI_ISL_837663, EPI_ISL_837664, EPI_ISL_837665, EPI_ISL_837666, EPI_ISL_837667, EPI_ISL_837669, EPI_ISL_837670, EPI_ISL_837671, EPI_ISL_837672, EPI_ISL_837673, EPI_ISL_837674, EPI_ISL_837675, EPI_ISL_837676, EPI_ISL_837677, EPI_ISL_837678, EPI_ISL_837679, EPI_ISL_837680, EPI_ISL_837681, EPI_ISL_837682, EPI_ISL_837683, EPI_ISL_837684, EPI_ISL_837685, EPI_ISL_837686, EPI_ISL_837687, EPI_ISL_837688, EPI_ISL_837689, EPI_ISL_837690, EPI_ISL_837691, EPI_ISL_837692, EPI_ISL_837693, EPI_ISL_837694, EPI_ISL_837695, EPI_ISL_837696, EPI_ISL_837697			
see above	Instituto Nacional de Enfermedades Respiratorias (INER)	Instituto Nacional de Enfermedades Respiratorias (INER)	Celia Boukadida, Margarita Matías-Florentino, Alma Rincón-Rubio, Hector Esteban Paz-Juárez, Olivia Briceño, Edgar Sevilla-Reyes, Fidencio Mejía-Nepomuceno, Mario Mújica-Sánchez, Eduardo Becerril-Vargas, José Arturo Martínez-Orozco, Alejandra Hernández-Terán, Jorge Salas-Hernández, Santiago Ávila-Ríos, Joel Armando Vázquez-Pérez
EPI_ISL_848624	Evandro Chagas Institute	Evandro Chagas Institute	Santos, M.C.; Silva, A.M.; Junior, W.D.C.; Barbagelata, L.S.; Ferreira, J.A.; Sousa, E.M.A.; da Silva, P.S.; Pinheiro, K.C.; L.C.; Sousa Junior, E.C.
EPI_ISL_851048	Gandhi Medical College and Hospital	CSIR-Centre for Cellular and Molecular Biology	Nagamani Kammili, Winnie Thomas, Madhavi Latha Manolla, Shailaja VV, Sudhamadhuri Devara, Vanisree Rajoli, Archana GJ, Sushma Rajyalakshmi Gudiseva, Sunitha Pakalapati, Manisha Rani, Amrithesh Kumar, Raja Rao Mesepogu, Vinay Shekar Reddy, Thrilok Chander Bingi, Sofia Banu, Divya Tej Sowpati
EPI_ISL_851049	Gandhi Medical College and Hospital	CSIR-Centre for Cellular and Molecular Biology	Nagamani Kammili, Madhavi Latha Manolla, Winnie Thomas, Shailaja VV, Sudhamadhuri Devara, Vanisree Rajoli, Archana GJ,Sushma Rajyalakshmi Gudiseva, Sunitha Pakalapati, Manisha Rani, Amrithesh Kumar, Raja Rao Mesepogu, Vinay Shekar Reddy, Thrilok Chander Bingi, Sofia Banu, Divya Tej Sowpati
EPI_ISL_853820, EPI_ISL_853821	Department of Pathology, Landeskrankenhaus Graz II, Medical University Graz	Bergthaler laboratory, CeMM Research Center for Molecular Medicine of the Austrian Academy of Sciences	Lukas Endler, Alexandra Popa, Benedikt Agerer, Jakob-Wendelin Genger, Alexander Lercher, Anna Schedl, Thomas Penz, Michael Schuster, Jan Laine, Martin Senekowitsch, Christoph Bock, Andreas Bergthaler
EPI_ISL_856695	University of Zambia, School of Veterinary Medicine, Disease Control	University of Zambia, School of Veterinary Medicine, Disease Control	Simulundu,E., Kapata,N., Mupeta,F., Kapata,P.C., Saasa,N., Changula,K., Muleya,W., Chitanga,S., Chambaro,H., Mubemba,B., Masahiro,K., Chanda,D., Mulenga,L., Fwoloshi,S., Shibemba,A.L., Kapaya,F., Zulu,P., Musonda,K., Monze,M., Sinyange,N., Liwewe,M.M., Kapin'a,M., Chipimo,P.J., Ngosa,W., Morales,A.N., Kayeyi,N., Malama,K., Tembo,J., Bates,M., Sawa,H., Takada,A., Nalubamba,K.S., Mukonka,V., Chilufya,C. and Zumla,A.
EPI_ISL_856971	Laboratory of Histology-Embryology, Molecular Carcinogenesis Group Medical School National and Kapodistrian University of Athens	Prof. Gorgoulis Lab	Gorgoulis V.G.
EPI_ISL_861419	Instituto de Diagnostico y Referencia Epidemiologicos	Instituto de Diagnostico y Referencia Epidemiologicos	Fabiola Garces-Ayala, Gisela Barrera-Badillo , Abril Rodriguez-Maldonado, Claudia Wong-Arambula , Natividad Cruz-Ortiz, Tatiana Nunez-Garcia, Dayanira

	(INDRE)	(INDRE)	Arellano-Suarez, Adnan-Araiza Rodríguez, Edgar Mendieta-Condado, Lucia Hernandez-Rivas, Irma Lopez-Martinez, Ernesto Ramirez-Gonzalez.
EPI_ISL_861420	Instituto de Diagnostico y Referencia Epidemiologicos (INDRE)	Instituto de Diagnostico y Referencia Epidemiologicos (INDRE)	Abril Rodriguez-Maldonado, Gisela Barrera-Badillo ,Claudia Wong-Arambula , Natividad Cruz-Ortiz, Tatiana Nunez-Garcia, Dayanira Arellano-Suarez, Fabiola Garces-Ayala, Adnan-Araiza Rodríguez, Edgar Mendieta-Condado, Lucia Hernandez-Rivas, Irma Lopez-Martinez, Ernesto Ramirez-Gonzalez.
EPI_ISL_861625, EPI_ISL_861626, EPI_ISL_861627	Instituto Adolfo Lutz - Central	Instituto Adolfo Lutz, Interdiciplinary Procedures Center, Strategic Laboratory	Claudio Tavares Sacchi, Claudia Regina Gonçalves, Erica Valessa Ramos Gomes, Karoline Rodrigues Campos
EPI_ISL_861628	Laboratorio Municipal de Guarulhos	Instituto Adolfo Lutz, Interdiciplinary Procedures Center, Strategic Laboratory	Claudio Tavares Sacchi, Claudia Regina Gonçalves, Erica Valessa Ramos Gomes, Karoline Rodrigues Campos
EPI_ISL_861629, EPI_ISL_861630, EPI_ISL_861631, EPI_ISL_861632, EPI_ISL_861633, EPI_ISL_861634	Instituto Adolfo Lutz - Central	Instituto Adolfo Lutz, Interdiciplinary Procedures Center, Strategic Laboratory	Claudio Tavares Sacchi, Claudia Regina Gonçalves, Erica Valessa Ramos Gomes, Karoline Rodrigues Campos
EPI_ISL_861635	Hospital e Maternidade Madre Theodora	Instituto Adolfo Lutz, Interdiciplinary Procedures Center, Strategic Laboratory	Claudio Tavares Sacchi, Claudia Regina Gonçalves, Erica Valessa Ramos Gomes, Karoline Rodrigues Campos
EPI_ISL_861636	Hospital Geral de Sao Mateus São Paulo	Instituto Adolfo Lutz, Interdiciplinary Procedures Center, Strategic Laboratory	Claudio Tavares Sacchi, Claudia Regina Gonçalves, Erica Valessa Ramos Gomes, Karoline Rodrigues Campos
EPI_ISL_861637	Hospital Nipo Brasileiro	Instituto Adolfo Lutz, Interdiciplinary Procedures Center, Strategic Laboratory	Claudio Tavares Sacchi, Claudia Regina Gonçalves, Erica Valessa Ramos Gomes, Karoline Rodrigues Campos
EPI_ISL_861638	Hospital Municipal Dr. Moyses Deutsch	Instituto Adolfo Lutz, Interdiciplinary Procedures Center, Strategic Laboratory	Claudio Tavares Sacchi, Claudia Regina Gonçalves, Erica Valessa Ramos Gomes, Karoline Rodrigues Campos
EPI_ISL_861639	Hospital Sao Paulo de Ensino da Unifesp	Instituto Adolfo Lutz, Interdiciplinary Procedures Center, Strategic Laboratory	Claudio Tavares Sacchi, Claudia Regina Gonçalves, Erica Valessa Ramos Gomes, Karoline Rodrigues Campos
EPI_ISL_861640, EPI_ISL_861641	Hospital Municipal Dr. Moyses Deutsch	Instituto Adolfo Lutz, Interdiciplinary Procedures Center, Strategic Laboratory	Claudio Tavares Sacchi, Claudia Regina Gonçalves, Erica Valessa Ramos Gomes, Karoline Rodrigues Campos
EPI_ISL_861642, EPI_ISL_861643	Instituto Adolfo Lutz - Central	Instituto Adolfo Lutz, Interdiciplinary Procedures Center, Strategic Laboratory	Claudio Tavares Sacchi, Claudia Regina Gonçalves, Erica Valessa Ramos Gomes, Karoline Rodrigues Campos
EPI_ISL_861644	Hospital Santa Virginia	Instituto Adolfo Lutz, Interdiciplinary Procedures Center, Strategic Laboratory	Claudio Tavares Sacchi, Claudia Regina Gonçalves, Erica Valessa Ramos Gomes, Karoline Rodrigues Campos
EPI_ISL_861645	Hospital e Pronto Socorro Comunitario Vila Iolanda	Instituto Adolfo Lutz, Interdiciplinary Procedures Center, Strategic Laboratory	Claudio Tavares Sacchi, Claudia Regina Gonçalves, Erica Valessa Ramos Gomes, Karoline Rodrigues Campos
EPI_ISL_861650	Hospital Santa Marcelina Sao Paulo	Instituto Adolfo Lutz, Interdiciplinary Procedures Center, Strategic Laboratory	Claudio Tavares Sacchi, Claudia Regina Gonçalves, Erica Valessa Ramos Gomes, Karoline Rodrigues Campos
EPI_ISL_861651	AMA Jardim Brasil	Instituto Adolfo Lutz, Interdiciplinary Procedures Center, Strategic Laboratory	Claudio Tavares Sacchi, Claudia Regina Gonçalves, Erica Valessa Ramos Gomes, Karoline Rodrigues Campos
EPI_ISL_861652	AMA Wamberto Dias da Costa	Instituto Adolfo Lutz, Interdiciplinary Procedures Center, Strategic Laboratory	Claudio Tavares Sacchi, Claudia Regina Gonçalves, Erica Valessa Ramos Gomes, Karoline Rodrigues Campos
EPI_ISL_861653	Hospital Santa Virgínia	Instituto Adolfo Lutz, Interdiciplinary Procedures Center, Strategic Laboratory	Claudio Tavares Sacchi, Claudia Regina Gonçalves, Erica Valessa Ramos Gomes, Karoline Rodrigues Campos
EPI_ISL_861654, EPI_ISL_861655	Hospital Santa Marcelina Sao Paulo	Instituto Adolfo Lutz, Interdiciplinary Procedures Center, Strategic Laboratory	Claudio Tavares Sacchi, Claudia Regina Gonçalves, Erica Valessa Ramos Gomes, Karoline Rodrigues Campos
EPI_ISL_861656	UPA de Jandira	Instituto Adolfo Lutz, Interdiciplinary Procedures Center, Strategic Laboratory	Claudio Tavares Sacchi, Claudia Regina Gonçalves, Erica Valessa Ramos Gomes, Karoline Rodrigues Campos
EPI_ISL_861659, EPI_ISL_861661	PS e Maternidade Nair Fonseca Leitao Arantes	Instituto Adolfo Lutz, Interdiciplinary Procedures Center, Strategic Laboratory	Claudio Tavares Sacchi, Claudia Regina Gonçalves, Erica Valessa Ramos Gomes, Karoline Rodrigues Campos
EPI_ISL_861663	Instituto Adolfo Lutz - Central	Instituto Adolfo Lutz, Interdiciplinary Procedures Center, Strategic Laboratory	Claudio Tavares Sacchi, Claudia Regina Gonçalves, Erica Valessa Ramos Gomes, Karoline Rodrigues Campos
EPI_ISL_862077	National Influenza Center, Virology Department	National Influenza Center	J Yavarian,K Sadeghi, NZ Shafiei Jandaghi, V Salimi, A Nejati, N Ghavvami,F Ajaminejad and T Mokhtari Azad
EPI_ISL_862078	National Influenza Center, Virology Department	National Influenza Center	A Nejati, J Yavarian,K Sadeghi, NZ Shafiei Jandaghi, V Salimi, N Ghavvami,F Ajaminejad and T Mokhtari Azad
EPI_ISL_862567	Gerencia de Asistencia Sanitaria de Soria	Instituto de Salud Carlos III	Iglesias-Caballero, M. Molinero Calamita, M. González-Esguevillas, M. Camarero, S. Pozo, F. Casas, I. Jiménez, P. Jiménez, M. Zaballos, A. Monzón, S. Varona, S. Juliá, M. Cuesta, I, C. Aldea
EPI_ISL_862627, EPI_ISL_862628, EPI_ISL_862629, EPI_ISL_862630, EPI_ISL_862631, EPI_ISL_862632, EPI_ISL_862633, EPI_ISL_862634, EPI_ISL_862635, EPI_ISL_862636	Hospital Clínic	Instituto de Salud Carlos III	Iglesias-Caballero, M.Camarero, S. Molinero Calamita, M. González-Esguevillas, M. Pozo, F. Casas, I. Jiménez, P. Jiménez, M. Zaballos, A. Monzón, S. Varona, S. Juliá, M. Cuesta, I. Marcos, M.A.
EPI_ISL_862637, EPI_ISL_862638, EPI_ISL_862639	Hospital Nuestra Señora de Sonsoles	Instituto de Salud Carlos III	Iglesias-Caballero, M. Molinero Calamita, M. González-Esguevillas, M. Camarero, S. Pozo, F. Casas, I. Jiménez, P. Jiménez, M. Zaballos, A. Monzón, S. Varona, S. Juliá, M. Cuesta, I, A. San Pedro
EPI_ISL_862640	Gerencia de Asistencia Sanitaria de Soria	Instituto de Salud Carlos III	Iglesias-Caballero, M. Molinero Calamita, M. González-Esguevillas, M. Camarero, S. Pozo, F. Casas, I. Jiménez, P. Jiménez, M. Zaballos, A. Monzón, S. Varona, S. Juliá, M. Cuesta, I, C. Aldea
EPI_ISL_862641, EPI_ISL_862642, EPI_ISL_862643, EPI_ISL_862644	Consejería de Sanidad y Asuntos Sociales	Instituto de Salud Carlos III	Iglesias-Caballero, M. Camarero, S. Molinero Calamita, M. González-Esguevillas, M. Pozo, F. Casas, I. Jiménez, P. Jiménez, M. Zaballos, A. Monzón, S. Varona, S. Juliá, M. Cuesta, I. Gutiérrez, G.
EPI_ISL_862645, EPI_ISL_862646, EPI_ISL_862647, EPI_ISL_862648, EPI_ISL_862649, EPI_ISL_862650, EPI_ISL_862651	Complejo Hospitalario de Navarra	Instituto de Salud Carlos III	Iglesias-Caballero, M.Camarero, S. Molinero Calamita, M. González-Esguevillas, M. Pozo, F. Casas, I. Jiménez, P. Jiménez, M. Zaballos, A. Monzón, S. Varona, S. Juliá, M. Cuesta, I. Ezpeleta, C.
EPI_ISL_862652, EPI_ISL_862653	Clínica Universidad de Navarra	Instituto de Salud Carlos III	Iglesias-Caballero, M.Camarero, S. Molinero Calamita, M. González-Esguevillas, M. Pozo, F. Casas, I. Jiménez, P. Jiménez, M. Zaballos, A. Monzón, S. Varona, S. Juliá, M. Cuesta, I. Fernández, M.
EPI_ISL_862654	Hospital San Pedro de Alcántara	Instituto de Salud Carlos III	Iglesias-Caballero, M.Camarero, S. Molinero Calamita, M. González-Esguevillas, M. Pozo, F. Casas, I. Jiménez, P. Jiménez, M. Zaballos, A. Monzón, S. Varona, S. Juliá, M. Cuesta, I. Rodríguez, G.
EPI_ISL_862655, EPI_ISL_862656, EPI_ISL_862657, EPI_ISL_862658	Hospital Universitario de Ceuta	Instituto de Salud Carlos III	Iglesias-Caballero, M. Camarero, S. Molinero Calamita, M. González-Esguevillas, M. Pozo, F. Casas, I. Jiménez, P. Jiménez, M. Zaballos, A. Monzón, S. Varona, S. Juliá, M. Cuesta, I. Hijano, S.
EPI_ISL_862837, EPI_ISL_862838	Fujita Health University Hospital	Fujita health University, School of Medicine, Department of Microbiology	Masahiro suzuki, Yohei Doi

EPI_ISL_862839	Fujita Health University Bantane Hospital	Fujita health University, School of Medicine, Department of Microbiology	Masahiro suzuki, Yohei Doi
EPI_ISL_862840, EPI_ISL_862841, EPI_ISL_862842	Fujita Health University Hospital	Fujita health University, School of Medicine, Department of Microbiology	Masahiro suzuki, Yohei Doi
EPI_ISL_862843, EPI_ISL_862844, EPI_ISL_862845, EPI_ISL_862846, EPI_ISL_862847	Nara Medical University Hospital	Fujita health University, School of Medicine, Department of Microbiology	Masahiro suzuki, Yohei Doi
EPI_ISL_862848	Japanese Red Cross Narita Hospital	Fujita health University, School of Medicine, Department of Microbiology	Masahiro suzuki, Yohei Doi
EPI_ISL_862849, EPI_ISL_862850, EPI_ISL_862851	Ehime Prefectural Central Hospital	Fujita health University, School of Medicine, Department of Microbiology	Masahiro suzuki, Yohei Doi
EPI_ISL_862852, EPI_ISL_862853, EPI_ISL_862854	Tosei General Hospital	Fujita health University, School of Medicine, Department of Microbiology	Masahiro suzuki, Yohei Doi
EPI_ISL_862855	National Hospital Organization Kanazawa Medical Center	Fujita health University, School of Medicine, Department of Microbiology	Masahiro suzuki, Yohei Doi
EPI_ISL_862856	Japanese Red Cross Narita Hospital	Fujita health University, School of Medicine, Department of Microbiology	Masahiro suzuki, Yohei Doi
EPI_ISL_862857	Fujita Health University Bantane Hospital	Fujita health University, School of Medicine, Department of Microbiology	Masahiro suzuki, Yohei Doi
EPI_ISL_862858	Isehara Kyodo Hospital	Fujita health University, School of Medicine, Department of Microbiology	Masahiro suzuki, Yohei Doi
EPI_ISL_862859, EPI_ISL_862860	Nara Medical University Hospital	Fujita health University, School of Medicine, Department of Microbiology	Masahiro suzuki, Yohei Doi
EPI_ISL_862861, EPI_ISL_862862	Sagamihara Kyodo Hospital	Fujita health University, School of Medicine, Department of Microbiology	Masahiro suzuki, Yohei Doi
EPI_ISL_862863, EPI_ISL_862864, EPI_ISL_862865	University Hospital Kyoto Prefectural University of Medicine	Fujita health University, School of Medicine, Department of Microbiology	Masahiro suzuki, Yohei Doi
EPI_ISL_862866	Nara Medical University Hospital	Fujita health University, School of Medicine, Department of Microbiology	Masahiro suzuki, Yohei Doi
EPI_ISL_862867	Yokohama City University Hospital	Fujita health University, School of Medicine, Department of Microbiology	Masahiro suzuki, Yohei Doi
EPI_ISL_862868	Fujita Health University Bantane Hospital	Fujita health University, School of Medicine, Department of Microbiology	Masahiro suzuki, Yohei Doi
EPI_ISL_862869	Saiseikai Niigata Hospital	Fujita health University, School of Medicine, Department of Microbiology	Masahiro suzuki, Yohei Doi
EPI_ISL_862871	Nara Medical University Hospital	Fujita health University, School of Medicine, Department of Microbiology	Masahiro suzuki, Yohei Doi
EPI_ISL_862872, EPI_ISL_862873	Japanese Red Cross Narita Hospital	Fujita health University, School of Medicine, Department of Microbiology	Masahiro suzuki, Yohei Doi
EPI_ISL_862874	Japanese Red Cross Nagoya Daiichi Hospital	Fujita health University, School of Medicine, Department of Microbiology	Masahiro suzuki, Yohei Doi
EPI_ISL_862875	Komatsu Municipal Hospital	Fujita health University, School of Medicine, Department of Microbiology	Masahiro suzuki, Yohei Doi
EPI_ISL_862876	NTT East Sapporo Hospital	Fujita health University, School of Medicine, Department of Microbiology	Masahiro suzuki, Yohei Doi
EPI_ISL_862877, EPI_ISL_862878	Nara Medical University Hospital	Fujita health University, School of Medicine, Department of Microbiology	Masahiro suzuki, Yohei Doi
EPI_ISL_862879	Ehime Prefectural Central Hospital	Fujita health University, School of Medicine, Department of Microbiology	Masahiro suzuki, Yohei Doi
EPI_ISL_862880	Nara Medical University Hospital	Fujita health University, School of Medicine, Department of Microbiology	Masahiro suzuki, Yohei Doi
EPI_ISL_862881	Kitakyushu Municipal Medical Center	Fujita health University, School of Medicine, Department of Microbiology	Masahiro suzuki, Yohei Doi
EPI_ISL_862882, EPI_ISL_862883, EPI_ISL_862884	University Hospital Kyoto Prefectural University of Medicine	Fujita health University, School of Medicine, Department of Microbiology	Masahiro suzuki, Yohei Doi
EPI_ISL_862885	Japanese Red Cross Narita Hospital	Fujita health University, School of Medicine, Department of Microbiology	Masahiro suzuki, Yohei Doi
EPI_ISL_871973	Hospital Universitario Severo Ochoa	Instituto de Salud Carlos III	Iglesias-Caballero, M. Camarero, S. Molinero Calamita, M. González-Esguevillas, M. Pozo, F. Casas, I. Jiménez, P. Jiménez, M. Zaballos, A. Monzón, S. Varona, S. Juliá, M. Cuesta, I. García, M.L.
EPI_ISL_871974, EPI_ISL_871975	Hospital Infanta Cristina	Instituto de Salud Carlos III	Iglesias-Caballero, M. Camarero, S. Molinero Calamita, M. González-Esguevillas, M. Pozo, F. Casas, I. Jiménez, P. Jiménez, M. Zaballos, A. Monzón, S. Varona, S. Juliá, M. Cuesta, I. García, P.
EPI_ISL_871976, EPI_ISL_871977	Hospital Universitario Lozano Bielsa	Instituto de Salud Carlos III	Iglesias-Caballero, M. Camarero, S. Molinero Calamita, M. González-Esguevillas, M. Pozo, F. Casas, I. Jiménez, P. Jiménez, M. Zaballos, A. Monzón, S. Varona, S. Juliá, M. Cuesta, I. Benito, R.
EPI_ISL_871978	Hospital Universitario Miguel Servet	Instituto de Salud Carlos III	Iglesias-Caballero, M. Camarero, S. Molinero Calamita, M. González-Esguevillas, M. Pozo, F. Casas, I. Jiménez, P. Jiménez, M. Zaballos, A. Monzón, S. Varona, S. Juliá, M. Cuesta, I. Rezusta, A.
EPI_ISL_871979	Hospital Infanta Cristina	Instituto de Salud Carlos III	Iglesias-Caballero, M. Camarero, S. Molinero Calamita, M. González-Esguevillas, M. Pozo, F. Casas, I. Jiménez, P. Jiménez, M. Zaballos, A. Monzón, S. Varona, S. Juliá, M. Cuesta, I. García, P.
EPI_ISL_871980, EPI_ISL_871981, EPI_ISL_871982, EPI_ISL_871983	Hospital Universitario Miguel Servet	Instituto de Salud Carlos III	Iglesias-Caballero, M. Camarero, S. Molinero Calamita, M. González-Esguevillas, M. Pozo, F. Casas, I. Jiménez, P. Jiménez, M. Zaballos, A. Monzón, S. Varona, S. Juliá, M. Cuesta, I. Rezusta, A.
EPI_ISL_872027, EPI_ISL_872028, EPI_ISL_872029, EPI_ISL_872030,	Ospedale "Di Venere"	Beaconlab (Bioinformatics, Evolution and Comparative Genomics lab), Dept of Biosciences, University on Milan	Iacobellis M, d'Avenia M, Piluscio R, Parisi A, Chiara M, Manzari C, Pesole G

EPI_ISL_872031, EPI_ISL_872032, EPI_ISL_872033, EPI_ISL_872034, EPI_ISL_872035, EPI_ISL_872036			
EPI_ISL_872088	Instituto de Diagnostico y Referencia Epidemiologicos (INDRE)	Instituto de Diagnostico y Referencia Epidemiologicos (INDRE)	Fabiola Garces-Ayala, Gisela Barrera-Badillo , Abril Rodriguez-Maldonado, Claudia Wong-Arambula , Natividad Cruz-Ortiz, Tatiana Nunez-Garcia, Dayanira Arellano-Suarez, Adnan-Araiza Rodríguez, Edgar Mendieta-Condado, Lucia Hernandez-Rivas, Irma Lopez-Martinez, Ernesto Ramirez-Gonzalez.
EPI_ISL_872089	Instituto de Diagnostico y Referencia Epidemiologicos (INDRE)	Instituto de Diagnostico y Referencia Epidemiologicos (INDRE)	Abril Rodriguez-Maldonado, Gisela Barrera-Badillo ,Claudia Wong-Arambula , Natividad Cruz-Ortiz, Tatiana Nunez-Garcia, Dayanira Arellano-Suarez, Fabiola Garces-Ayala, Adnan-Araiza Rodríguez, Edgar Mendieta-Condado, Lucia Hernandez-Rivas, Irma Lopez-Martinez, Ernesto Ramirez-Gonzalez.
EPI_ISL_872091	Instituto de Diagnostico y Referencia Epidemiologicos (INDRE)	Instituto de Diagnostico y Referencia Epidemiologicos (INDRE)	Adnan-Araiza Rodríguez,Claudia Wong-Arambula, Gisela Barrera-Badillo , Abril Rodriguez-Maldonado, Natividad Cruz-Ortiz, Tatiana Nunez-Garcia, Dayanira Arellano-Suarez, Fabiola Garces-Ayala, Edgar Mendieta-Condado, Lucia Hernandez-Rivas, Irma Lopez-Martinez, Ernesto Ramirez-Gonzalez.
EPI_ISL_872092	Instituto de Diagnostico y Referencia Epidemiologicos (INDRE)	Instituto de Diagnostico y Referencia Epidemiologicos (INDRE)	Ernesto Ramirez-Gonzalez, Adnan-Araiza Rodríguez,Claudia Wong-Arambula, Gisela Barrera-Badillo , Abril Rodriguez-Maldonado, Natividad Cruz-Ortiz, Tatiana Nunez-Garcia, Dayanira Arellano-Suarez, Fabiola Garces-Ayala, Edgar Mendieta-Condado, Lucia Hernandez-Rivas, Irma Lopez-Martinez.
EPI_ISL_872093	Instituto de Diagnostico y Referencia Epidemiologicos (INDRE)	Instituto de Diagnostico y Referencia Epidemiologicos (INDRE)	Edgar Mendieta-Condado, Ernesto Ramirez-Gonzalez, Adnan-Araiza Rodríguez,Claudia Wong-Arambula, Gisela Barrera-Badillo , Abril Rodriguez-Maldonado, Natividad Cruz-Ortiz, Tatiana Nunez-Garcia, Dayanira Arellano-Suarez, Fabiola Garces-Ayala, Lucia Hernandez-Rivas, Irma Lopez-Martinez.
EPI_ISL_872097	Instituto de Diagnostico y Referencia Epidemiologicos (INDRE)	Instituto de Diagnostico y Referencia Epidemiologicos (INDRE)	Abril Rodriguez-Maldonado, Gisela Barrera-Badillo ,Claudia Wong-Arambula , Natividad Cruz-Ortiz, Tatiana Nunez-Garcia, Dayanira Arellano-Suarez, Fabiola Garces-Ayala, Adnan-Araiza Rodríguez, Edgar Mendieta-Condado, Lucia Hernandez-Rivas, Irma Lopez-Martinez, Ernesto Ramirez-Gonzalez.
EPI_ISL_872099	Instituto de Diagnostico y Referencia Epidemiologicos (INDRE)	Instituto de Diagnostico y Referencia Epidemiologicos (INDRE)	Fabiola Garces-Ayala, Gisela Barrera-Badillo , Abril Rodriguez-Maldonado, Claudia Wong-Arambula , Natividad Cruz-Ortiz, Tatiana Nunez-Garcia, Dayanira Arellano-Suarez, Adnan-Araiza Rodríguez, Edgar Mendieta-Condado, Lucia Hernandez-Rivas, Irma Lopez-Martinez, Ernesto Ramirez-Gonzalez.
EPI_ISL_876811, EPI_ISL_876812, EPI_ISL_876813	Istituto Zooprofilattico Sperimentale della Puglia e della Basilicata	Istituto Zooprofilattico Sperimentale della Puglia e della Basilicata	Parisi A., Bianco A., Capozzi L., Del Sambro L., Manzulli V, Rondinone V., Pace L., Cipolletta D., Galante D.
EPI_ISL_877036	1.AO Universitaria 'S. Giovanni di Dio e Ruggi D'Aragona, Scuola Medica Salernitana' Hospital / 2.UOC di Virologia e Microbiologia, Università della Campania 'L. Vanvitelli' / 3.AO Universitaria 'Federico II' Napoli Hospital / 4.AORN 'San Giuseppe Moscati' Avellino Hospital / 5.AO 'San Pio - presidio G. Rummo' Benevento Hospital / 6.AO 'Sant'Anna e San Sebastiano' Caserta Hospital / 7.PO 'Maria Santissima Addolorata' Eboli Hospital / 8.Biogem Istituto di Ricerche Genetiche	1. Genome Research Center for Health (CRGS) / 2. Laboratory of Molecular Medicine and Genomics(LMMGe) / 3. Center for Research in Pure and Applied Mathematics (CRMPA)	Giorgio Giurato (Corresponding Author), Francesca Rizzo (Corresponding Author), Alessandro Weisz (Corresponding Author), Gianluigi Franci, Giovanni Nassa, Pasquale Pagliano, Roberta Tarallo, Elena Alexandrova, Ylenia D'Agostino, Carlo Ferravante, Jessica Lamberti, Viola Melone, Domenico Memoli, Valeria Mirici Cappa, Domenico Palumbo, Giovanni Pecoraro, Assunta Sellitto, Oriana Strianese, Ilaria Terenzi, Giuseppe Fenza, Aniello Gentile, Antonello Saccomanno, Sonia Amabile, Teresa Rocco, Annamaria Salvati, Emilia Vaccaro, Massimiliano Galdiero, Michele Cennamo, Giuseppe Portella, Maria Grazia Foti, Mariarosaria Ingino, Maria Landi, Maurizio Fumi, Vincenzo Rocco, Rita Greco, Vittoria Letizia, Arnolfo Petruzziello, Maddalena Schioppa, Gregorio Goffredi, Francesca Marciano, Michele Caraglia, Alessia Cossu, Marianna Scrima
EPI_ISL_888678	Laboratory for Respiratory Viruses, Cantacuzino National Military-Medical Institute for Research and Development	Cantacuzino Institute Virology	Luiza Ustea, Nicoleta Paraschiv, Mihaela Lazar
EPI_ISL_888982	RS Pondok Indah Hospital - Pondok Indah	Eijkman Institute for Molecular Biology, Ministry of Research and Technology/National Agency for Research and Innovation	Edison Johar, Frilasita A Yudhaputri, Hidayat Trimarsanto, Iskandar Adnan, Lydia V. Panggalo, Sukma Oktavianthi, Willy Agustine, Safarina G Malik, Khin Saw Myint, Amin Soebandrio
EPI_ISL_889373, EPI_ISL_889374, EPI_ISL_889375, EPI_ISL_889377, EPI_ISL_889378, EPI_ISL_889379, EPI_ISL_889380, EPI_ISL_889384, EPI_ISL_889385, EPI_ISL_889386, EPI_ISL_889387, EPI_ISL_889388, EPI_ISL_889389, EPI_ISL_889390, EPI_ISL_889391, EPI_ISL_889392, EPI_ISL_889393			
see above	Olomouc University Hospital	Institute of Applied Biotechnologies a.s.	Petr Klempt, Ondej Brzo, Martin Kašný, Kateina Kvapilová, Marián Hajdúch, Petr Kvapil
EPI_ISL_895747, EPI_ISL_895748, EPI_ISL_895768, EPI_ISL_895769, EPI_ISL_895789, EPI_ISL_895803, EPI_ISL_895805, EPI_ISL_895818, EPI_ISL_895829, EPI_ISL_895833, EPI_ISL_895836, EPI_ISL_895838, EPI_ISL_895839, EPI_ISL_895840, EPI_ISL_895843, EPI_ISL_895844, EPI_ISL_895845, EPI_ISL_895846			
see above	Molecular biology division, Institute of Clinical Biochemistry and Diagnostics, Charles University, Faculty of Medicine in Hradec Králové and University Hospital Hradec Králové	Molecular biology division, Institute of Clinical Biochemistry and Diagnostics, Charles University, Faculty of Medicine in Hradec Králové and University Hospital Hradec Králové	Helena Kovaíková, Petr Brož, Ivana Baranová, Kateina Hrochová, Tereza Baková, Jitka Novotná, Kateina Pehlíková, Vladimír Palika. Cooperation project with BioVendor-R&D and bioinformatics company BIOXSYS s r.o.
EPI_ISL_900726	Bozeman Health Deaconess Hospital	Wiedenheft lab, Montana State University	Artem Nemudryi, Anna Nemudraia, Tanner Wiegand, Joseph Nichols, Deann T. Snyder, Jodi F. Hedges, Calvin Cicha, Helen Lee, Karl K. Vanderwood, Diane Bimczok, Mark A. Jutila and Blake Wiedenheft
EPI_ISL_906065	Day Hospital de Ermelino Matarazzo	Instituto Adolfo Lutz, Interdiciplinary Procedures Center, Strategic Laboratory	Claudio Tavares Sacchi, Claudia Regina Gonçalves, Erica Valessa Ramos Gomes, Karoline Rodrigues Campos
EPI_ISL_906066	Hospital Nipo Brasileiro	Instituto Adolfo Lutz, Interdiciplinary Procedures Center, Strategic Laboratory	Claudio Tavares Sacchi, Claudia Regina Gonçalves, Erica Valessa Ramos Gomes, Karoline Rodrigues Campos
EPI_ISL_906067	PS e Maternidade Nair Fonseca Leitao Arantes	Instituto Adolfo Lutz, Interdiciplinary Procedures Center, Strategic Laboratory	Claudio Tavares Sacchi, Claudia Regina Gonçalves, Erica Valessa Ramos Gomes, Karoline Rodrigues Campos
EPI_ISL_906769, EPI_ISL_906775	Dr. Leonard A. Miller Centre for Health Services	National Microbiology Laboratory (NML)	Anna Majer, Shari Tyson, Grace Seo, Philip Mabon, Elsie Grudeski, Rhiannon Huzarewich, Russell Mandes, Anneliese Landgraff, Jennifer Tanner, Natalie Knox, Morag Graham, Gary Van Domselaar, Robert Needle, Yang Yu, Laura Gilbert, George Zahariadis, Nathalie Bastien, Yan Li, Timothy Booth, Darian Hole, Madison Chapel, Kerri Smith, CanCOGeN's metadata curation team, Public Health Agency of Canada CanCOGeN team
EPI_ISL_911513	Laboratoire de Virologie, CHU de Caen, Normandie, France.	GRAM2.0, Université de Caen Normandie Laboratoire de Virologie, CHU de Caen	Meriadeg LE GOUIL, Januel VOUTEAU, Juliette ECHE, Estelle LEPERCHOIS, Astrid VABRET
EPI_ISL_940753	University of Bari Biomedical Sciences and Human Oncology	University of Bari Biomedical Sciences and Human Oncology	Chironna M., Sallustio A., Loconsole D., Accogli M.
EPI_ISL_941104, EPI_ISL_941105, EPI_ISL_941106, EPI_ISL_941107, EPI_ISL_941108, EPI_ISL_941109, EPI_ISL_941110, EPI_ISL_941111, EPI_ISL_941112, EPI_ISL_941113, EPI_ISL_941114, EPI_ISL_941115, EPI_ISL_941116, EPI_ISL_941117, EPI_ISL_941118, EPI_ISL_941119, EPI_ISL_941120, EPI_ISL_941121, EPI_ISL_941122, EPI_ISL_941123, EPI_ISL_941125, EPI_ISL_941126, EPI_ISL_941127, EPI_ISL_941129, EPI_ISL_941130, EPI_ISL_941131, EPI_ISL_941132, EPI_ISL_941133, EPI_ISL_941134, EPI_ISL_941135, EPI_ISL_941137, EPI_ISL_941138, EPI_ISL_941139, EPI_ISL_941140, EPI_ISL_941141, EPI_ISL_941142, EPI_ISL_941143, EPI_ISL_941144, EPI_ISL_941145, EPI_ISL_941146, EPI_ISL_941147, EPI_ISL_941148, EPI_ISL_941149, EPI_ISL_941150, EPI_ISL_941151, EPI_ISL_941152, EPI_ISL_941153, EPI_ISL_941154, EPI_ISL_941155, EPI_ISL_941156, EPI_ISL_941157, EPI_ISL_941158, EPI_ISL_941159, EPI_ISL_941160, EPI_ISL_941161, EPI_ISL_941162			
see above	Centro de Investigaciones en Microbiología y Biotecnología-UR (CIMBIUR), Facultad de Ciencias Naturales, Universidad del Rosario, Bogotá, Colombia	Centro de Investigaciones en Microbiología y Biotecnología-UR (CIMBIUR), Facultad de Ciencias Naturales, Universidad del Rosario, Bogotá, Colombia Icahn School of Medicine at Mount Sinai, New York, USA	Nathalia Ballesteros, Marina Muñoz, Luz Helena Patiño, Carolina Hernández, Felipe González-Casabianca, Iván Carroll, Mauricio Santos-Vega, Jaime Cascante, Andrés Angel, Alejandro Feged-Rivadeneira, Mónica Palma-Cuero, Carolina Flórez, Sergio Gomez, Adriana van de Guchte, Zenab Khan, Jayeeta Dutta, Hala Alejel Alshammary, Ana S. Gonzalez-Reiche, Matthew M. Hernandez, Emilia Mia Sordillo, Viviana Simon, Harm van Bakel, Alberto Paniz-Mondolfi, Juan David Ramírez
EPI_ISL_941952, EPI_ISL_941964, EPI_ISL_941965, EPI_ISL_941966, EPI_ISL_941967, EPI_ISL_941968, EPI_ISL_941969, EPI_ISL_941970, EPI_ISL_941971, EPI_ISL_941972, EPI_ISL_941973, EPI_ISL_941974, EPI_ISL_941975, EPI_ISL_941976, EPI_ISL_941977, EPI_ISL_941978, EPI_ISL_941979, EPI_ISL_941980, EPI_ISL_941981, EPI_ISL_941982, EPI_ISL_941983			
see above	Centro de Investigaciones en Microbiología y Biotecnología-UR (CIMBIUR), Facultad de Ciencias Naturales, Universidad del Rosario, Bogotá, Colombia	Centro de Investigaciones en Microbiología y Biotecnología-UR (CIMBIUR), Facultad de Ciencias Naturales, Universidad del Rosario, Bogotá, Colombia Instituto Nacional de Salud, Bogotá, Colombia Icahn School of Medicine at Mount Sinai, New York, USA	Luz Helena Patiño, Marina Muñoz, Nathalia Ballesteros, Carolina Hernández, Carolina Flórez, Sergio Gomez, Adriana van de Guchte, Zenab Khan, Jayeeta Dutta, Hala Alejel Alshammary, Ana S. Gonzalez-Reiche, Matthew M. Hernandez, Emilia Mia Sordillo, Viviana Simon, Harm van Bakel, Alberto Paniz-Mondolfi, Juan David Ramírez
EPI_ISL_941984, EPI_ISL_941985	Instituto Nacional de Salud, Bogotá, Colombia	Centro de Investigaciones en Microbiología y Biotecnología-UR (CIMBIUR), Facultad de Ciencias Naturales, Universidad del Rosario, Bogotá, Colombia Instituto Nacional de Salud, Bogotá, Colombia Icahn School of Medicine at Mount Sinai, New York, USA	Luz Helena Patiño, Marina Muñoz, Nathalia Ballesteros, Carolina Hernández, Carolina Flórez, Sergio Gomez, Adriana van de Guchte, Zenab Khan, Jayeeta Dutta, Hala Alejel Alshammary, Ana S. Gonzalez-Reiche, Matthew M. Hernandez, Emilia Mia Sordillo, Viviana Simon, Harm van Bakel, Alberto Paniz-Mondolfi, Juan David Ramírez

EPI_ISL_941999, EPI_ISL_942000, EPI_ISL_942001, EPI_ISL_942002, EPI_ISL_944635, EPI_ISL_944638	Centro de Investigaciones en Microbiología y Biotecnología-UR (CIMBIUR), Facultad de Ciencias Naturales, Universidad del Rosario, Bogotá, Colombia	Centro de Investigaciones en Microbiología y Biotecnología-UR (CIMBIUR), Facultad de Ciencias Naturales, Universidad del Rosario, Bogotá, Colombia Instituto Nacional de Salud, Bogotá, Colombia Icahn School of Medicine at Mount Sinai, New York, USA	Luz Helena Patiño, Marina Muñoz, Nathalia Ballesteros, Carolina Hernández, Carolina Flórez, Sergio Gomez, Adriana van de Guchte, Zenab Khan, Jayeeta Dutta, Hala Alejel Alshammary, Ana S. Gonzalez-Reiche, Matthew M. Hernandez, Emilia Mia Sordillo, Viviana Simon, Harm van Bakel, Alberto Paniz-Mondolfi, Juan David Ramírez
EPI_ISL_949244, EPI_ISL_949245, EPI_ISL_949246, EPI_ISL_949247	Cliniques universitaires Saint-Luc	UCLouvain/IREC/MBLG	Jean Ruelle, Lysa Pinsmaye, Benoit Kabamba Mukadi
EPI_ISL_949248	Cliniques Saint-Pierre Ottignies	UCLouvain/IREC/MBLG	Jean Ruelle, Lysa Pinsmaye, Benoit Kabamba Mukadi
EPI_ISL_949249, EPI_ISL_949250, EPI_ISL_949252	Cliniques universitaires Saint-Luc	UCLouvain/IREC/MBLG	Jean Ruelle, Lysa Pinsmaye, Benoit Kabamba Mukadi
EPI_ISL_955234	HE CMN LA RAZA	BIOBANCO / COCTI	Borja-Aburto VH, Grajales-Muñiz C, Santacruz Tinoco CE, Rojas-Mendoza T, Ochoa Carrera LA, Sánchez A, Grande R, Isa P, Taboada B, López S, Arias C, Muñoz-Medina JE
EPI_ISL_955235	UMF 23 UNIDAD MORELOS	BIOBANCO / COCTI	Borja-Aburto VH, Grajales-Muñiz C, Santacruz Tinoco CE, Rojas-Mendoza T, Ochoa Carrera LA, Sánchez A, Grande R, Isa P, Taboada B, López S, Arias C, Muñoz-Medina JE
EPI_ISL_955236	HGR 1 VICENTE GUERRERO	BIOBANCO / COCTI	Borja-Aburto VH, Grajales-Muñiz C, Santacruz Tinoco CE, Rojas-Mendoza T, Ochoa Carrera LA, Sánchez A, Grande R, Isa P, Taboada B, López S, Arias C, Muñoz-Medina JE
EPI_ISL_955237	UMF 75 NETZAHUALCOYOTL	BIOBANCO / COCTI	Borja-Aburto VH, Grajales-Muñiz C, Santacruz Tinoco CE, Rojas-Mendoza T, Ochoa Carrera LA, Sánchez A, Grande R, Isa P, Taboada B, López S, Arias C, Muñoz-Medina JE
EPI_ISL_955238	HI CMN LA RAZA	BIOBANCO / COCTI	Borja-Aburto VH, Grajales-Muñiz C, Santacruz Tinoco CE, Rojas-Mendoza T, Ochoa Carrera LA, Sánchez A, Grande R, Isa P, Taboada B, López S, Arias C, Muñoz-Medina JE
EPI_ISL_955239	HE CMN LA RAZA	BIOBANCO / COCTI	Borja-Aburto VH, Grajales-Muñiz C, Santacruz Tinoco CE, Rojas-Mendoza T, Ochoa Carrera LA, Sánchez A, Grande R, Isa P, Taboada B, López S, Arias C, Muñoz-Medina JE
EPI_ISL_955240, EPI_ISL_955241	HGZ 30 MEXICALI	BIOBANCO / COCTI	Borja-Aburto VH, Grajales-Muñiz C, Santacruz Tinoco CE, Rojas-Mendoza T, Ochoa Carrera LA, Sánchez A, Grande R, Isa P, Taboada B, López S, Arias C, Muñoz-Medina JE
EPI_ISL_955242, EPI_ISL_955243	HGSMF 26 CABO SAN LUCAS	BIOBANCO / COCTI	Borja-Aburto VH, Grajales-Muñiz C, Santacruz Tinoco CE, Rojas-Mendoza T, Ochoa Carrera LA, Sánchez A, Grande R, Isa P, Taboada B, López S, Arias C, Muñoz-Medina JE
EPI_ISL_955244	UMF 26 ACAPULCO	BIOBANCO / COCTI	Borja-Aburto VH, Grajales-Muñiz C, Santacruz Tinoco CE, Rojas-Mendoza T, Ochoa Carrera LA, Sánchez A, Grande R, Isa P, Taboada B, López S, Arias C, Muñoz-Medina JE
EPI_ISL_955245	HGR 1 VICENTE GUERRERO	BIOBANCO / COCTI	Borja-Aburto VH, Grajales-Muñiz C, Santacruz Tinoco CE, Rojas-Mendoza T, Ochoa Carrera LA, Sánchez A, Grande R, Isa P, Taboada B, López S, Arias C, Muñoz-Medina JE
EPI_ISL_955246	HGOMF 60 TLANEPANTLA	BIOBANCO / COCTI	Borja-Aburto VH, Grajales-Muñiz C, Santacruz Tinoco CE, Rojas-Mendoza T, Ochoa Carrera LA, Sánchez A, Grande R, Isa P, Taboada B, López S, Arias C, Muñoz-Medina JE
EPI_ISL_955247	HGR 200 TECAMAC	BIOBANCO / COCTI	Borja-Aburto VH, Grajales-Muñiz C, Santacruz Tinoco CE, Rojas-Mendoza T, Ochoa Carrera LA, Sánchez A, Grande R, Isa P, Taboada B, López S, Arias C, Muñoz-Medina JE
EPI_ISL_955248	BCO SANGRE CM LA RAZA	BIOBANCO / COCTI	Borja-Aburto VH, Grajales-Muñiz C, Santacruz Tinoco CE, Rojas-Mendoza T, Ochoa Carrera LA, Sánchez A, Grande R, Isa P, Taboada B, López S, Arias C, Muñoz-Medina JE
EPI_ISL_955249	HGZ 2A PASO-TRONCOSO	BIOBANCO / COCTI	Borja-Aburto VH, Grajales-Muñiz C, Santacruz Tinoco CE, Rojas-Mendoza T, Ochoa Carrera LA, Sánchez A, Grande R, Isa P, Taboada B, López S, Arias C, Muñoz-Medina JE
EPI_ISL_955250, EPI_ISL_955251, EPI_ISL_955252, EPI_ISL_955253	HGSMF 26 CABO SAN LUCAS	BIOBANCO / COCTI	Borja-Aburto VH, Grajales-Muñiz C, Santacruz Tinoco CE, Rojas-Mendoza T, Ochoa Carrera LA, Sánchez A, Grande R, Isa P, Taboada B, López S, Arias C, Muñoz-Medina JE
EPI_ISL_955254	UMF 28 DEL VALLE	BIOBANCO / COCTI	Borja-Aburto VH, Grajales-Muñiz C, Santacruz Tinoco CE, Rojas-Mendoza T, Ochoa Carrera LA, Sánchez A, Grande R, Isa P, Taboada B, López S, Arias C, Muñoz-Medina JE
EPI_ISL_955255	HGSMF 26 CABO SAN LUCAS	BIOBANCO / COCTI	Borja-Aburto VH, Grajales-Muñiz C, Santacruz Tinoco CE, Rojas-Mendoza T, Ochoa Carrera LA, Sánchez A, Grande R, Isa P, Taboada B, López S, Arias C, Muñoz-Medina JE
EPI_ISL_955256	HGZ 1 CARLOS MC GREGOR	BIOBANCO / COCTI	Borja-Aburto VH, Grajales-Muñiz C, Santacruz Tinoco CE, Rojas-Mendoza T, Ochoa Carrera LA, Sánchez A, Grande R, Isa P, Taboada B, López S, Arias C, Muñoz-Medina JE
EPI_ISL_955257	HGZ 32 VILLA COAPA	BIOBANCO / COCTI	Borja-Aburto VH, Grajales-Muñiz C, Santacruz Tinoco CE, Rojas-Mendoza T, Ochoa Carrera LA, Sánchez A, Grande R, Isa P, Taboada B, López S, Arias C, Muñoz-Medina JE
EPI_ISL_956270	Universitas Airlangga Hospital	Institute of Tropical Disease, Universitas Airlangga	Aldise M Nastri, Jezzy R Dewantari, Rima R Prasetya, Krisnoadi Rahardjo, Nasronudin, Gatot Soegiarto, Laksmi Wulandari, Resti Yudhawati, Soetjipto, Yasuko Mori, Maria I Lusida, Kazufumi Shimizu
EPI_ISL_956307	Primasatya Husada Citra Hospital	Institute of Tropical Disease, Universitas Airlangga	Krisnoadi Rahardjo, Aldise M Nastri, Jezzy R Dewantari, Rima R Prasetya, Pudji Djanuartono, Gatot Soegiarto, Laksmi Wulandari, Resti Yudhawati, Yasuko Mori, Soetjipto, Kazufumi Shimizu, Maria I Lusida
EPI_ISL_956308	RSUD Dr. M. Soewandhie	Institute of Tropical Disease, Universitas Airlangga	Jezzy R Dewantari, Rima R Prasetya, Krisnoadi Rahardjo, Aldise M Nastri, Febria Rachmanita, Gatot Soegiarto, Laksmi Wulandari, Resti Yudhawati, Yasuko Mori, Soetjipto, Kazufumi Shimizu, Maria I Lusida
EPI_ISL_956384	Isolation - Virology Unit, Institut Pasteur du Cambodge; Sequencing - US National Institute of Allergy and Infectious Diseases Cambodia, US Naval Medical Research Unit -2, National Public Health Laboratory, National Institute for Public Health, Cambodia	Virology Unit, Institut Pasteur du Cambodge	Vireak Heang, Jennifer Bohl, Sophana Chea, Sreyngim Lay, Ly Sovann, Kraing Sidonn, Yi Sengdoeurn, Chin Savuth, Chau Darapheak, Veasna Duong, Jose A Garcia-Rivera, Jessica Manning, Erik A Karlsson
EPI_ISL_956385, EPI_ISL_956386, EPI_ISL_956387, EPI_ISL_956388, EPI_ISL_956389, EPI_ISL_956390, EPI_ISL_956391, EPI_ISL_956392, EPI_ISL_956393, EPI_ISL_956394, EPI_ISL_956395, EPI_ISL_956396, EPI_ISL_956397, EPI_ISL_956398, EPI_ISL_956399, EPI_ISL_956400, EPI_ISL_956401			
see above	Isolation - Virology Unit, Institut Pasteur du Cambodge; Sequencing - US National Institute of Allergy and Infectious Diseases Cambodia, US Naval Medical Research Unit -2, Cambodia National Institute for Public Health	Virology Unit, Institut Pasteur du Cambodge	Vireak Heang, Jennifer Bohl, Sophana Chea, Sreyngim Lay, Ly Sovann, Kraing Sidonn, Yi Sengdoeurn, Chin Savuth, Chau Darapheak, Veasna Duong, Jose A Garcia-Rivera, Jessica Manning, Erik A Karlsson
EPI_ISL_959275	National Influenza Center, Virology Department	National Influenza Center	V Salimi,NZ Shafiei Jandaghi, J Yavarian, A Nejati, K Sadeghi, N Ghavvami,F Ajaminejad and T Mokhtari Azad
EPI_ISL_959276	National Influenza Center, Virology Department	National Influenza Center	NZ Shafiei Jandaghi, V Salimi, A Nejati, K Sadeghi, J Yavarian, N Ghavvami,F Ajaminejad and T Mokhtari Azad
EPI_ISL_959280	National Influenza Center, Virology Department	National Influenza Center	J Yavarian, NZ Shafiei Jandaghi, V Salimi, A Nejati, K Sadeghi, F Ajaminejad,N Ghavvami and T Mokhtari Azad
EPI_ISL_959283	National Influenza Center, Virology Department	National Influenza Center	NZ Shafiei Jandaghi, V Salimi, A Nejati, K Sadeghi, J Yavarian, F Ajaminejad,N Ghavvami and T Mokhtari Azad
EPI_ISL_962528, EPI_ISL_962529, EPI_ISL_962530, EPI_ISL_962531, EPI_ISL_962532, EPI_ISL_962533, EPI_ISL_962534, EPI_ISL_962535, EPI_ISL_962536, EPI_ISL_962537, EPI_ISL_962538, EPI_ISL_962539, EPI_ISL_962540, EPI_ISL_962541, EPI_ISL_962542, EPI_ISL_962543, EPI_ISL_962544, EPI_ISL_962545, EPI_ISL_962546, EPI_ISL_962547, EPI_ISL_962548, EPI_ISL_962549, EPI_ISL_962550, EPI_ISL_962551, EPI_ISL_962552, EPI_ISL_962553, EPI_ISL_962554, EPI_ISL_962555, EPI_ISL_962556, EPI_ISL_962557, EPI_ISL_962558, EPI_ISL_962559, EPI_ISL_962560, EPI_ISL_962561, EPI_ISL_962562, EPI_ISL_962563, EPI_ISL_962564, EPI_ISL_962565, EPI_ISL_962566, EPI_ISL_962567, EPI_ISL_962568, EPI_ISL_962569, EPI_ISL_962570, EPI_ISL_962571, EPI_ISL_962572, EPI_ISL_962573, EPI_ISL_962574, EPI_ISL_962575, EPI_ISL_962576, EPI_ISL_962577, EPI_ISL_962578, EPI_ISL_962579, EPI_ISL_962580, EPI_ISL_962581,			

EPI_ISL_962582, EPI_ISL_962583, EPI_ISL_962584, EPI_ISL_962868, EPI_ISL_962869, EPI_ISL_962870, EPI_ISL_962871, EPI_ISL_962873, EPI_ISL_962874, EPI_ISL_962875

see above	Infectious Disease Control and Prevention Institute	Infectious Disease Control and Prevention Institute	Xiaolin Jiang, Mingxiao Yao, Yuwei Zhang, Yujie He, Zengqiang Kou, Ti Liu, Yan Li, Jianxing Wang, Bo Pang, Julong Wu
EPI_ISL_964878, EPI_ISL_964879	Institute of Virology Ulm University Medical Center Prof. Dr. Thomas Stamminger	Münch Lab / Ulm University Medical Center Kirchhoff Lab / Ulm University Medical Center Sparrer Lab / Ulm University Medical Center Blum Lab / LMU Munich	Thomas Stamminger, Helmut Blum, Stefan Krebs, Alexander Graf, Rüdiger Gross, Janis Müller, Carina Conzelmann, Jan Münch, Frank Kirchhoff, Konstantin Sparrer
EPI_ISL_977357, EPI_ISL_977411	University of Zambia, School of Veterinary Medicine	UNZAVET and PATH	Mulenga Mwenda-Chimfwembe, Ngonda Saasa, Daniel Bridges
EPI_ISL_982182	Hôpital Henri Mondor	Department of Virology, Henri Mondor University Hospital, Assistance Publique Hôpitaux de Paris, Université Paris-Est Créteil, INSERM U955	Christophe Rodriguez, Slim Fourati, Vanessa Demontant, Guillaume Gricourt, Melissa N'Debi, Alexandre Soulier, Elisabeth Trawinski, Jean-Michel Pawlotsky
EPI_ISL_984277	National Institute of Health Research and Development	National Institute of Health Research and Development	Kindi Adam, Holy Arif Wibowo, Ririn Ramadhany, Yuni Rukminiati, Agustiningsih, Hana Apsari Pawestri, Subangkit, Kartika Dewi Puspa, Arie Ardiansyah Nugraha, Hartanti Dian Ikawati, Krisna Nur Andriana Pangesti, Ni Ketut Susilarini, Nur Ika Hariastuti, Uly Alfi Nikmah, Mursinah, Asri Febriyani, Reni Herman, Nike Susanti, Herna, Tati Febriyanti, Natalie Laurencia Kipuw, Fauzul Muna, Irene Lorinda Indalao, Aulia Rizki, Nelly Puspandari, Vivi Setiawaty
EPI_ISL_985034	Omics Sciences Lab	Omics Sciences Lab	Derly Andrade Molina, Rubén Armas González, Gabriel Morey León, Darlyn Amaya, Katheryn Sacheri Viteri, Edith Lopez Montanero, Pedro Barberán, Fernando Espinoza Fuentes, Juan Carlos Fernández Cadena
EPI_ISL_985394	Department of Internal Medicine, College of Medicine, Chosun University	Department of Internal Medicine, College of Medicine, Chosun University	Dong-Min Kim

We gratefully acknowledge the following Authors from the Originating laboratories responsible for obtaining the specimens, as well as the Submitting laboratories where the genome data were generated and shared via GISAID, on which this research is based.

All Submitters of data may be contacted directly via www.gisaid.org

Authors are sorted alphabetically.

Accession ID	Originating Laboratory	Submitting Laboratory	Authors
EPI_ISL_1007672, EPI_ISL_1007673, EPI_ISL_1007674, EPI_ISL_1007675, EPI_ISL_1007676	Department of Internal Medicine, College of Medicine, Chosun University	Department of Internal Medicine, College of Medicine, Chosun University	Dong-Min Kim
EPI_ISL_1014678	National Influenza Center, Virology Department	National Influenza Center	J Yavarian,K Sadeghi, NZ Shafiei Jandaghi, V Salimi, A Nejati, N Ghavvami,F Ajaminejad and T Mokhtari Azad
EPI_ISL_1014679	National Influenza Center, Virology Department	National Influenza Center	A Nejati, J Yavarian,K Sadeghi, NZ Shafiei Jandaghi, V Salimi, N Ghavvami,F Ajaminejad and T Mokhtari Azad
EPI_ISL_1014680	National Influenza Center, Virology Department	National Influenza Center	K Sadeghi, A Nejati, J Yavarian, NZ Shafiei Jandaghi, V Salimi, F Ajaminejad,N Ghavvami and T Mokhtari Azad
EPI_ISL_1014681	National Influenza Center, Virology Department	National Influenza Center	V Salimi,NZ Shafiei Jandaghi, J Yavarian, A Nejati, K Sadeghi, N Ghavvami,F Ajaminejad and T Mokhtari Azad
EPI_ISL_1014687	National Influenza Center, Virology Department	National Influenza Center	J Yavarian,K Sadeghi, NZ Shafiei Jandaghi, V Salimi, A Nejati, N Ghavvami,F Ajaminejad and T Mokhtari Azad
EPI_ISL_1015362	Pathogenic Microorganisms Variability Laboratory	Pathogenic Microorganisms Variability Laboratory	Alexey Shchetinin, Olesya Venchakova, Maria Nikiforova, Andrei Siniavin, Nadezhda Kuznetsova, Elena Shidlovskaya, Elizaveta Divisenko, Kirill Krasnoslobotsev, Evgeniya Mukasheva, Anna Ignatieva, Svetlana Trushakova, Andrey Pochtovyy, Valeria Bacalin, Evgeny Usachev, Olga Burgasova, Ludmila Kolobukhina, Svetlana Smetanina, Elena Burtseva, Denis Logunov, Vladimir Gushchin, Alexander Gintsburg
EPI_ISL_1018118, EPI_ISL_1018119, EPI_ISL_1018120, EPI_ISL_1018121, EPI_ISL_1018122, EPI_ISL_1018125, EPI_ISL_1018126, EPI_ISL_1018127, EPI_ISL_1018128, EPI_ISL_1018129, EPI_ISL_1018130, EPI_ISL_1018131, EPI_ISL_1018132, EPI_ISL_1018133, EPI_ISL_1018134, EPI_ISL_1018135, EPI_ISL_1018136, EPI_ISL_1018137, EPI_ISL_1018138, EPI_ISL_1018139, EPI_ISL_1018140, EPI_ISL_1018141, EPI_ISL_1018142, EPI_ISL_1018143, EPI_ISL_1018144, EPI_ISL_1018145, EPI_ISL_1018146, EPI_ISL_1018147, EPI_ISL_1018148, EPI_ISL_1018149, EPI_ISL_1018150, EPI_ISL_1018151, EPI_ISL_1018152, EPI_ISL_1018153, EPI_ISL_1018154, EPI_ISL_1018155, EPI_ISL_1018156, EPI_ISL_1018157, EPI_ISL_1018158, EPI_ISL_1018159, EPI_ISL_1018160, EPI_ISL_1018161, EPI_ISL_1018162, EPI_ISL_1018163, EPI_ISL_1018164, EPI_ISL_1018165, EPI_ISL_1018166, EPI_ISL_1018167, EPI_ISL_1018168, EPI_ISL_1018169, EPI_ISL_1018170, EPI_ISL_1018171, EPI_ISL_1018172, EPI_ISL_1018173, EPI_ISL_1018174, EPI_ISL_1018175, EPI_ISL_1018176, EPI_ISL_1018177, EPI_ISL_1018178, EPI_ISL_1018179, EPI_ISL_1018180, EPI_ISL_1018181, EPI_ISL_1018182, EPI_ISL_1018183, EPI_ISL_1018184, EPI_ISL_1018185, EPI_ISL_1018186, EPI_ISL_1018187, EPI_ISL_1018188, EPI_ISL_1018189, EPI_ISL_1018190, EPI_ISL_1018191, EPI_ISL_1018192, EPI_ISL_1018197, EPI_ISL_1018198, EPI_ISL_1018199, EPI_ISL_1018200, EPI_ISL_1018201, EPI_ISL_1018203, EPI_ISL_1018204, EPI_ISL_1018206, EPI_ISL_1018207, EPI_ISL_1018208, EPI_ISL_1018209, EPI_ISL_1018210, EPI_ISL_1018211, EPI_ISL_1018212, EPI_ISL_1018213, EPI_ISL_1018214, EPI_ISL_1018215, EPI_ISL_1018216, EPI_ISL_1018217, EPI_ISL_1018230, EPI_ISL_1018231, EPI_ISL_1018232, EPI_ISL_1018233, EPI_ISL_1018236, EPI_ISL_1018237, EPI_ISL_1018238, EPI_ISL_1018239, EPI_ISL_1018240, EPI_ISL_1018243, EPI_ISL_1018244, EPI_ISL_1018245, EPI_ISL_1018246, EPI_ISL_1018247, EPI_ISL_1018248, EPI_ISL_1018249, EPI_ISL_1018250, EPI_ISL_1018251, EPI_ISL_1018252, EPI_ISL_1018253, EPI_ISL_1018254, EPI_ISL_1018255, EPI_ISL_1018256, EPI_ISL_1018257, EPI_ISL_1018259, EPI_ISL_1018260, EPI_ISL_1018262, EPI_ISL_1018263, EPI_ISL_1018264, EPI_ISL_1018265, EPI_ISL_1018266, EPI_ISL_1018268, EPI_ISL_1018269, EPI_ISL_1018270, EPI_ISL_1018271, EPI_ISL_1018273, EPI_ISL_1018274, EPI_ISL_1018275, EPI_ISL_1018276, EPI_ISL_1018279, EPI_ISL_1018280, EPI_ISL_1018281, EPI_ISL_1018282, EPI_ISL_1018284, EPI_ISL_1018286			
see above	Department of Health Technology and Informatics, The Hong Kong Polytechnic University	Department of Health Technology and Informatics, The Hong Kong Polytechnic University	Gilman Kit-Hang Siu, Lam-Kwong Lee, Kenneth Siu-Sing Leung, Jake Siu-Lun Leung, Timothy Ting-Leung Ng, Chloe Toi-Mei Chan, Kingsley King-Gee Tam, Hiu-Yin Lao, Denise Sze-Hang Wong, Alan Ka-Lun Wu, Miranda Chong-Yee Yau, Yvette Wai-Man Lai, Kitty Sau-Chun Fung, Sandy Ka-Yee Chau, Barry Kin-Chung Wong, Wing-Kin To, Kristine Luk, Alex Yat-Man Ho, Tak-Lun Que, Kam-Tong Yip, Wing Cheong Yam, David Ho-Keung Shum, Shea Ping Yip
EPI_ISL_1023841	Centro de Desenvolvimento Tecnológico em Saúde - CDTS	Centro de Desenvolvimento Tecnológico em Saúde - CDTS	Souza,T.M., Fintelman-Rodrigues,N., De Paula,A.D., Saraiva,F.B., Ferreira,M.A. and Sacramento,C.Q.
EPI_ISL_1039696	Instituto Adolfo Lutz - Regional de Presidente Prudente	Instituto Adolfo Lutz, Interdiciplinary Procedures Center, Strategic Laboratory	Claudio Tavares Sacchi, Claudia Regina Gonçalves, Erica Valessa Ramos Gomes, Karoline Rodrigues Campos
EPI_ISL_1039702	Instituto Adolfo Lutz - Regional de Aracatuba	Instituto Adolfo Lutz, Interdiciplinary Procedures Center, Strategic Laboratory	Claudio Tavares Sacchi, Claudia Regina Gonçalves, Erica Valessa Ramos Gomes, Karoline Rodrigues Campos
EPI_ISL_1040841, EPI_ISL_1040842, EPI_ISL_1040843, EPI_ISL_1040844, EPI_ISL_1040845, EPI_ISL_1040846, EPI_ISL_1040847, EPI_ISL_1040848, EPI_ISL_1040849, EPI_ISL_1040850, EPI_ISL_1040851			
see above	LACEN do Mato Grosso do Sul	Instituto Adolfo Lutz, Interdiciplinary Procedures Center, Strategic Laboratory	Claudio Tavares Sacchi, Claudia Regina Gonçalves, Erica Valessa Ramos Gomes, Karoline Rodrigues Campos
EPI_ISL_1040923	Grupo de Investigación en Enfermedades Tropicales del Ejército (GINETEJ), Laboratorio de Referencia e Investigación, Dirección de Sanidad Ejército, Bogotá, Colombia	Centro de Investigaciones en Microbiología y Biotecnología-UR (CIMBIUR), Facultad de Ciencias Naturales, Universidad del Rosario, Bogotá, Colombia	Juan David Ramírez, Marina Muñoz, Nathalia Ballesteros, Luz H. Patiño, Sergio Castañeda, Carlos A. Rincón, Claudia P. Mendez, Magda C. Oliveros, Julie J. Perez, Elizabeth K. Marquez, Frank de los Santos Ortiz, Camilo Correa-Cardenas, Maria C. Duque, Alberto Paniz-Mondolfi
EPI_ISL_1054940, EPI_ISL_1054944, EPI_ISL_1054947, EPI_ISL_1054960, EPI_ISL_1054964, EPI_ISL_1054968, EPI_ISL_1054978, EPI_ISL_1054980, EPI_ISL_1054983, EPI_ISL_1054988, EPI_ISL_1054995			
see above	Instituto de Diagnostico y Referencia Epidemiologicos INDRE_RNLSP	Instituto de Diagnostico y Referencia Epidemiologicos (INDRE)	Claudia Wong-Arambula, Abril Rodriguez-Maldonado, Fabiola Garces-Ayala, Adnan Araiza-Rodriguez, David Fragoso-Fonseca, Sergio Rangel-Guerrero, Mayra Jimenez-Morales, Nancy Munoz-Hernandez, Natividad Cruz-Ortiz, Tatiana Nunez-Garcia, Gisela Barrera-Badillo, Lucia Hernandez-Rivas, Irma Lopez-Martinez, Ernesto Ramirez-Gonzalez.
EPI_ISL_1060735, EPI_ISL_1060736, EPI_ISL_1060743, EPI_ISL_1060752, EPI_ISL_1060757, EPI_ISL_1060758, EPI_ISL_1060760, EPI_ISL_1060762	Instituto de Diagnostico y Referencia Epidemiologicos (INDRE)_RNLSP	Instituto de Diagnostico y Referencia Epidemiologicos (INDRE)	Claudia Wong-Arambula, Abril Rodriguez-Maldonado, Fabiola Garces-Ayala, Adnan Araiza-Rodriguez, David Fragoso-Fonseca, Sergio Rangel-Guerrero, Mayra Jimenez-Morales, Nancy Munoz-Hernandez, Natividad Cruz-Ortiz, Tatiana Nunez-Garcia, Gisela Barrera-Badillo, Lucia Hernandez-Rivas, Irma Lopez-Martinez, Ernesto Ramirez-Gonzalez.
EPI_ISL_1081834, EPI_ISL_1081835	Virus Research and Diagnostic Laboratory, Medical College Level, Kasturba Hospital for Infectious Diseases	National Institute of Virology-Microbial Containment Complex, Indian Council of Medical Research	Pragya D. Yadav, Jayanti Shastri
EPI_ISL_1091786	LDSP	Universidad Nacional de Colombia - Laboratorio Genómico One Health	Andres F. Cardona-Rios, Daniel O. Maldonado-Perez, Laura Silvana Perez, Karl A Ciuoderis, Maria Angélica Maya, Idabely Betancur Ortiz, Sandra Ines Cano, Diego A. Álvarez-Díaz, Carlos Franco-Muñoz, Marcela Mercado-Reyes, Jorge E. Osorio, Juan P. Hernandez-Ortiz
EPI_ISL_1098839	Medical Ain Shams Research Institute (MASRI), Ain Shams University	Medical Ain Shams Research Institute (MASRI), Ain Shams University	Hesham Elghazaly, Sara Hassan Agwa, Hala Hafez , Fatma Ebied, Manal Hamdy Elsaid, Mohamed Elhadidi, Aya Mohamed , Shimaa Moustafa, Reham Kassab, Iman Foda , Samia Abdou Girgis, Hoda Ezz Elarab, Hagar Elshora, Osama Mansour.
EPI_ISL_1103556	Kurnool Medical College (KMC)	CSIR Institute of Genomics and Integrative Biology	Pallavali Roja Rani, Mohamed Imran, Juturu Vijaya Lakshmi, Bani Jolly, Abhinav Jain, Avileli Surekha, Vigneshwar Senthivel, Pulala Chandrasekhar, Mohit Kumar Divakar, Damam Srinivasulu, Rahul C Bhoyar, PR Vanaja, Vinod Scaria, Sridhar Sivasubbu
EPI_ISL_1111068, EPI_ISL_1111070, EPI_ISL_1111071, EPI_ISL_1111072, EPI_ISL_1111073	Laboratorio de Referencia Nacional de Enteropatógenos. Instituto Nacional de Salud del Perú	Laboratorio de Referencia Nacional de Enteropatógenos. Instituto Nacional de Salud del Perú	Ronnie Gavilan Chavez, Junior Caro Castro, Willi Quino Sifuentes, Veronica Hurtado Vela, Iris Silva Molina, Fiorella Orellana Peralta
EPI_ISL_1111074, EPI_ISL_1111082, EPI_ISL_1111083, EPI_ISL_1111084, EPI_ISL_1111085, EPI_ISL_1111099, EPI_ISL_1111121, EPI_ISL_1111165, EPI_ISL_1111166, EPI_ISL_1111167, EPI_ISL_1111168, EPI_ISL_1111173, EPI_ISL_1111191, EPI_ISL_1111241, EPI_ISL_1111242, EPI_ISL_1111243, EPI_ISL_1111244, EPI_ISL_1111245, EPI_ISL_1111246, EPI_ISL_1111247, EPI_ISL_1111248, EPI_ISL_1111249, EPI_ISL_1111250, EPI_ISL_1111251, EPI_ISL_1111252, EPI_ISL_1111253, EPI_ISL_1111254, EPI_ISL_1111255, EPI_ISL_1111256, EPI_ISL_1111257, EPI_ISL_1111258, EPI_ISL_1111259, EPI_ISL_1111260, EPI_ISL_1111261, EPI_ISL_1111262, EPI_ISL_1111263, EPI_ISL_1111264, EPI_ISL_1111265, EPI_ISL_1111266, EPI_ISL_1111311, EPI_ISL_1111357, EPI_ISL_1111359, EPI_ISL_1111360, EPI_ISL_1111361, EPI_ISL_1111362, EPI_ISL_1111363, EPI_ISL_1111364, EPI_ISL_1111365, EPI_ISL_1111383, EPI_ISL_1111384			
see above	Laboratorio de Referencia Nacional de Virus Respiratorio. Instituto Nacional de Salud Perú	Laboratorio de Referencia Nacional de Enteropatógenos. Instituto Nacional de Salud del Perú	Ronnie Gavilan Chavez, Junior Caro Castro, Willi Quino Sifuentes, Veronica Hurtado Vela, Iris Silva Molina, Fiorella Orellana Peralta
EPI_ISL_1111385, EPI_ISL_1111386, EPI_ISL_1111387, EPI_ISL_1111388, EPI_ISL_1111389, EPI_ISL_1111390, EPI_ISL_1111391, EPI_ISL_1111392, EPI_ISL_1111393, EPI_ISL_1111394, EPI_ISL_1111395, EPI_ISL_1111396, EPI_ISL_1111397, EPI_ISL_1111398, EPI_ISL_1111399, EPI_ISL_1111400, EPI_ISL_1111401, EPI_ISL_1111402, EPI_ISL_1111403, EPI_ISL_1111404, EPI_ISL_1111405, EPI_ISL_1111406, EPI_ISL_1111407, EPI_ISL_1111408, EPI_ISL_1111409, EPI_ISL_1111410, EPI_ISL_1111411, EPI_ISL_1111412, EPI_ISL_1111413, EPI_ISL_1111414, EPI_ISL_1111415, EPI_ISL_1111416, EPI_ISL_1111417, EPI_ISL_1111418, EPI_ISL_1111419, EPI_ISL_1111420, EPI_ISL_1111421, EPI_ISL_1111422, EPI_ISL_1111423, EPI_ISL_1111424, EPI_ISL_1111425, EPI_ISL_1111426, EPI_ISL_1111427, EPI_ISL_1111428, EPI_ISL_1111429, EPI_ISL_1111430, EPI_ISL_1111431, EPI_ISL_1111432, EPI_ISL_1111433, EPI_ISL_1111434, EPI_ISL_1111435, EPI_ISL_1111436, EPI_ISL_1111437, EPI_ISL_1111438, EPI_ISL_1111439, EPI_ISL_1111440, EPI_ISL_1111441, EPI_ISL_1111442, EPI_ISL_1111443, EPI_ISL_1111444, EPI_ISL_1111445			

see above	Laboratorio de Referencia Nacional de Enteropatógenos. Instituto Nacional de Salud del Perú	Laboratorio de Referencia Nacional de Enteropatógenos. Instituto Nacional de Salud del Perú	Ronnie Gavilan Chavez, Junior Caro Castro, Willi Quino Sifuentes, Veronica Hurtado Vela, Iris Silva Molina, Fiorella Orellana Peralta
EPI_ISL_1112951, EPI_ISL_1112952, EPI_ISL_1112953, EPI_ISL_1112954, EPI_ISL_1112955, EPI_ISL_1112957, EPI_ISL_1112958, EPI_ISL_1112959, EPI_ISL_1112960, EPI_ISL_1112961, EPI_ISL_1112962, EPI_ISL_1112963, EPI_ISL_1112964, EPI_ISL_1112965, EPI_ISL_1112966, EPI_ISL_1112967, EPI_ISL_1112968, EPI_ISL_1112969, EPI_ISL_1112970, EPI_ISL_1112971, EPI_ISL_1112972, EPI_ISL_1112973, EPI_ISL_1112974, EPI_ISL_1112975, EPI_ISL_1112976, EPI_ISL_1112977, EPI_ISL_1112978, EPI_ISL_1112979, EPI_ISL_1112980, EPI_ISL_1112981, EPI_ISL_1112982, EPI_ISL_1112983, EPI_ISL_1112984, EPI_ISL_1112985, EPI_ISL_1112986, EPI_ISL_1112987, EPI_ISL_1112988, EPI_ISL_1112989, EPI_ISL_1112990, EPI_ISL_1112991, EPI_ISL_1112993, EPI_ISL_1112994, EPI_ISL_1112995, EPI_ISL_1112996, EPI_ISL_1112997, EPI_ISL_1112998, EPI_ISL_1112999, EPI_ISL_1113000, EPI_ISL_1113001, EPI_ISL_1113002, EPI_ISL_1113003, EPI_ISL_1113004, EPI_ISL_1113005, EPI_ISL_1113006, EPI_ISL_1113007, EPI_ISL_1113008, EPI_ISL_1113009, EPI_ISL_1113010, EPI_ISL_1113011, EPI_ISL_1113012, EPI_ISL_1113013, EPI_ISL_1113014, EPI_ISL_1113015, EPI_ISL_1113016, EPI_ISL_1113017, EPI_ISL_1113018, EPI_ISL_1113019, EPI_ISL_1113020, EPI_ISL_1113021, EPI_ISL_1113022, EPI_ISL_1113023, EPI_ISL_1113024, EPI_ISL_1113025, EPI_ISL_1113026, EPI_ISL_1113027, EPI_ISL_1113028, EPI_ISL_1113029, EPI_ISL_1113030, EPI_ISL_1113031, EPI_ISL_1113032, EPI_ISL_1113033, EPI_ISL_1113034, EPI_ISL_1113035, EPI_ISL_1113036, EPI_ISL_1113037, EPI_ISL_1113038, EPI_ISL_1113039, EPI_ISL_1113040, EPI_ISL_1113041			
see above	Baylor College of Medicine/ GCID	Baylor College of Medicine/ GCID	Vasanthi Avadhanula, Erin Nicholson, David Henke, Pedro Piedra, Harsha Doddapaneni, Donna Muzny, Qingchang Meng, Hsu Chao, Zeineen Momin, Hua Shen, George Weissenberger, Kavya Kottapalli, Yimiti Meiheerguli, Sejal Salvi, Ginger Metcalf, Vipin Menon, Sara J.J. Cregeen, Matthew C. Ross, Tulin Ayvaz, Richard Sucgang, Kristi L. Hoffman, Matthew Wong, Joseph F. Petrosino
EPI_ISL_1118929	Institute of Clinical Hygiene, Medical Microbiology and Infectiology, General Hospital Nürnberg, Paracelsus Medical University, Nuremberg, Germany	Department of Molecular & Medical Virology, Ruhr University Bochum, 44801 Bochum, Germany | Institute of Virology, Charité - Universitätsmedizin Berlin, corporate member of Freie Universität Berlin, Humboldt-Universität zu Berlin and Berlin Institute of Health (BIH), Berlin, Germany	Daniel Todt, Toni Luise Meister, Baxolele Mhlekude, Christine Goffinet, Jörg Steinmann, Stephanie Pfaender, Yannick Brüggemann, Eike Steinmann
EPI_ISL_1121326	IAL Regional de Bauru	Instituto Adolfo Lutz, Interdiciplinary Procedures Center, Strategic Laboratory	Claudio Tavares Sacchi, Claudia Regina Gonçalves, Erica Valessa Ramos Gomes, Karoline Rodrigues Campos, Caio Vinicius Dias Lopes
EPI_ISL_1121327, EPI_ISL_1121328, EPI_ISL_1121329, EPI_ISL_1121330	LACEN do Mato Grosso do Sul	Instituto Adolfo Lutz, Interdiciplinary Procedures Center, Strategic Laboratory	Claudio Tavares Sacchi, Claudia Regina Gonçalves, Erica Valessa Ramos Gomes, Karoline Rodrigues Campos, Caio Vinicius Dias Lopes
EPI_ISL_1129216, EPI_ISL_1129217, EPI_ISL_1129218, EPI_ISL_1129219, EPI_ISL_1129220, EPI_ISL_1129221, EPI_ISL_1129222, EPI_ISL_1129223, EPI_ISL_1129224, EPI_ISL_1129225, EPI_ISL_1129226, EPI_ISL_1129227			
see above	Virus Research and Diagnostic Laboratory, Medical College Level, Kasturba Hospital for Infectious Diseases	National Institute of Virology-Microbial Containment Complex, Indian Council of Medical Research	Pragya D. Yadav, Jayanti Shastri
EPI_ISL_1138997, EPI_ISL_1138999	Rajendra Institute of Medical Sciences, Ranchi, Jharkhand	Indian Council of Medical Research-National Institute of Virology, Microbial Containment Complex	Pragya D. Yadav, Manoj Kumar
EPI_ISL_1139052, EPI_ISL_1139053, EPI_ISL_1139054, EPI_ISL_1139055	LACEN do Mato Grosso do Sul	Instituto Adolfo Lutz, Interdiciplinary Procedures Center, Strategic Laboratory	Claudio Tavares Sacchi, Claudia Regina Gonçalves, Erica Valessa Ramos Gomes, Karoline Rodrigues Campos, Caio Vinicius Dias Lopes
EPI_ISL_1139122, EPI_ISL_1139123, EPI_ISL_1139124, EPI_ISL_1139125, EPI_ISL_1139126, EPI_ISL_1139127, EPI_ISL_1139128, EPI_ISL_1139129, EPI_ISL_1139130, EPI_ISL_1139131, EPI_ISL_1139132, EPI_ISL_1139133, EPI_ISL_1139134, EPI_ISL_1139135, EPI_ISL_1139136, EPI_ISL_1139137, EPI_ISL_1139138, EPI_ISL_1139139, EPI_ISL_1139140, EPI_ISL_1139141, EPI_ISL_1139142, EPI_ISL_1139143, EPI_ISL_1139144, EPI_ISL_1139145			
see above	ICMR-RMRC, Dibrugarh	Indian Council of Medical Research-National Institute of Virology, Microbial Containment Complex	Pragya D. Yadav, Biswajyoti Borkakoty
EPI_ISL_1163701, EPI_ISL_1163702, EPI_ISL_1163703, EPI_ISL_1163704, EPI_ISL_1163705, EPI_ISL_1163706, EPI_ISL_1163707, EPI_ISL_1163708	LABCOVID_HCPA	LABRESIS_HCPA	Martins AF, Wink PL, Volpato F, Rosset C, de Paris F, Monteiro F, Zavascki AP, Barth AL
EPI_ISL_1164934, EPI_ISL_1164935	B.J. Medical College and Civil hospital	Indian Council of Medical Research-National Institute of Virology, Microbial Containment Complex	Pragya D. Yadav, Disha Patel
EPI_ISL_1165067	Siti Khodijah Hospital	Institute of Tropical Disease, Universitas Airlangga	Aldise M Nastri, Jezzy R Dewantari, Rima R Prasetya, Krisnoadi Rahardjo, Muhammad Hamdan, Gatot Soegiarto, Laksmi Wulandari, Resti Yudhawati, Yasuko Mori, Soetjipto, Kazufumi Shimizu, Maria I Lusida
EPI_ISL_1165068	Anwar Medika General Hospital	Institute of Tropical Disease, Universitas Airlangga	Jezzy R Dewantari, Rima R Prasetya, Krisnoadi Rahardjo, Aldise M Nastri, Nungky Taniasari ,Gatot Soegiarto, Laksmi Wulandari, Resti Yudhawati, Yasuko Mori, Soetjipto, Kazufumi Shimizu, Maria I Lusida
EPI_ISL_1165069	Siti Khodijah Hospital	Institute of Tropical Disease, Universitas Airlangga	Rima R Prasetya, Krisnoadi Rahardjo, Aldise M Nastri, Jezzy R Dewantari, Muhammad Hamdan, Gatot Soegiarto, Laksmi Wulandari, Resti Yudhawati, Yasuko Mori, Soetjipto, Kazufumi Shimizu, Maria I Lusida
EPI_ISL_1165075	Paru Hospital	Institute of Tropical Disease, Universitas Airlangga	Jezzy R Dewantari, Rima R Prasetya, Krisnoadi Rahardjo, Aldise M Nastri, Dyah Retno, Gatot Soegiarto, Laksmi Wulandari, Resti Yudhawati, Yasuko Mori, Soetjipto, Kazufumi Shimizu, Maria I Lusida
EPI_ISL_1165093, EPI_ISL_1165094, EPI_ISL_1165095, EPI_ISL_1165096, EPI_ISL_1165097, EPI_ISL_1165098, EPI_ISL_1165099, EPI_ISL_1165100, EPI_ISL_1165101, EPI_ISL_1165102, EPI_ISL_1165103, EPI_ISL_1165104, EPI_ISL_1165105, EPI_ISL_1165106, EPI_ISL_1165107, EPI_ISL_1165108, EPI_ISL_1165109, EPI_ISL_1165110, EPI_ISL_1165111, EPI_ISL_1165112, EPI_ISL_1165113, EPI_ISL_1165114, EPI_ISL_1165115, EPI_ISL_1165116, EPI_ISL_1165117, EPI_ISL_1165118, EPI_ISL_1165119, EPI_ISL_1165120, EPI_ISL_1165121, EPI_ISL_1165122, EPI_ISL_1165123, EPI_ISL_1165124, EPI_ISL_1165125, EPI_ISL_1165126, EPI_ISL_1165127, EPI_ISL_1165128, EPI_ISL_1165129, EPI_ISL_1165130, EPI_ISL_1165132, EPI_ISL_1165133, EPI_ISL_1165134, EPI_ISL_1165136, EPI_ISL_1165137			
see above	Government Medical College, Patiala, Punjab	Indian Council of Medical Research-National Institute of Virology, Microbial Containment Complex	Pragya D. Yadav, Rupinder Bakshi
EPI_ISL_1168693, EPI_ISL_1168694, EPI_ISL_1168695	Puerto Rico Department of Health	Centers for Disease Control and Prevention, Dengue Branch	Gilberto A. Santiago, Glenda Gonzalez, Betzabel Flores, Keyla Charriez, Gabriela Paz-Bailey, Jorge L. Munoz-Jordan
EPI_ISL_1171620	Instituto Adolfo Lutz Central	Instituto Adolfo Lutz, Interdiciplinary Procedures Center, Strategic Laboratory	Claudio Tavares Sacchi, Claudia Regina Gonçalves, Erica Valessa Ramos Gomes, Karoline Rodrigues Campos, Caio Vinicius Dias Lopes
EPI_ISL_1173511	Mubarak Al-Kabeer Hospital	Virology Unit, Department of Microbiology, Faculty of Medicine, Kuwait	Nada Madi, Mohammed Sadeq, Anfal Al-Adwani
EPI_ISL_1180186, EPI_ISL_1180187	Dept. of Microbiology, All India Institute of Medical Sciences, Jodhpur	Indian Council of Medical Research-National Institute of Virology, Microbial Containment Complex	Pragya D. Yadav, Vijaylakshmi Nag
EPI_ISL_1180195, EPI_ISL_1180196, EPI_ISL_1180197, EPI_ISL_1180198, EPI_ISL_1180199, EPI_ISL_1180200, EPI_ISL_1180201, EPI_ISL_1180202, EPI_ISL_1180203, EPI_ISL_1180204, EPI_ISL_1180205, EPI_ISL_1180206, EPI_ISL_1180207			
see above	VRDL, GMC, Haldwani , Nainital	Indian Council of Medical Research-National Institute of Virology, Microbial Containment Complex	Pragya D. Yadav, Vinita Rawat
EPI_ISL_1181363, EPI_ISL_1181385, EPI_ISL_1181386, EPI_ISL_1181387, EPI_ISL_1181388, EPI_ISL_1181459	Laboratory of Respiratory Viruses and Measles, Oswaldo Cruz Institute, FIOCRUZ	Laboratory of Respiratory Viruses and Measles, Oswaldo Cruz Institute, FIOCRUZ	Paola Resende, Luciana Appolinario, Fernando Motta, Anna Carolina Paixao, Ana Carolina Mendonca, Alice Sampaio Rocha, Renata Serrano Lopes, Marilda Siqueira on behalf of the Fiocruz COVID-19 Genomic Surveillance Network
EPI_ISL_1181474	Laboratorio Central de Saude Publica do Estado do Parana (LACEN-PR)	Laboratory of Respiratory Viruses and Measles, Oswaldo Cruz Institute, FIOCRUZ	Paola Resende, Luciana Appolinario, Fernando Motta, Anna Carolina Paixao, Ana Carolina Mendonca, Alice Sampaio Rocha, Renata Serrano Lopes, Maria do Carmo Debur, Irina Nastassja Riediger, Marilda Siqueira on behalf of the Fiocruz COVID-19 Genomic Surveillance Network
EPI_ISL_1181476	Laboratory of Respiratory Viruses and Measles, Oswaldo Cruz Institute, FIOCRUZ	Laboratory of Respiratory Viruses and Measles, Oswaldo Cruz Institute, FIOCRUZ	Paola Resende, Luciana Appolinario, Fernando Motta, Anna Carolina Paixao, Ana Carolina Mendonca, Alice Sampaio Rocha, Renata Serrano Lopes, Marilda Siqueira on behalf of the Fiocruz COVID-19 Genomic Surveillance Network
EPI_ISL_1181479, EPI_ISL_1181487	Laboratorio de Imunologia de Transplantes de Goias LTDA (HLAGYN)	Laboratory of Respiratory Viruses and Measles, Oswaldo Cruz Institute, FIOCRUZ	Paola Resende, Luciana Appolinario, Fernando Motta, Anna Carolina Paixao, Ana Carolina Mendonca, Alice Sampaio Rocha, Renata Serrano Lopes , Alessandro Leonardo Alvares Magalhaes, Fernando Antonio Vinhal dos Santos, Erika Lopes Rocha Batista, Marilda Siqueira on behalf of the Fiocruz COVID-19 Genomic Surveillance Network
EPI_ISL_1181491, EPI_ISL_1181518	Laboratory of Respiratory Viruses and Measles, Oswaldo	Laboratory of Respiratory Viruses and Measles, Oswaldo	Paola Resende, Luciana Appolinario, Fernando Motta, Anna Carolina Paixao, Ana Carolina Mendonca, Alice Sampaio Rocha, Renata Serrano Lopes, Marilda

	Cruz Institute, FIOCRUZ	Cruz Institute, FIOCRUZ	Siqueira on behalf of the Fiocruz COVID-19 Genomic Surveillance Network
EPI_ISL_1181530	Laboratorio Central de Saude Publica do Estado do Parana (LACEN-PR)	Laboratory of Respiratory Viruses and Measles, Oswaldo Cruz Institute, FIOCRUZ	Paola Resende, Luciana Appolinario, Fernando Motta, Anna Carolina Paixao, Ana Carolina Mendonca, Alice Sampaio Rocha, Renata Serrano Lopes, Maria do Carmo Debur, Irina Nastassja Riediger, Marilda Siqueira on behalf of the Fiocruz COVID-19 Genomic Surveillance Network
EPI_ISL_1181532	Laboratorio de Imunologia de Transplantes de Goias LTDA (HLAGYN)	Laboratory of Respiratory Viruses and Measles, Oswaldo Cruz Institute, FIOCRUZ	Paola Resende, Luciana Appolinario, Fernando Motta, Anna Carolina Paixao, Ana Carolina Mendonca, Alice Sampaio Rocha, Renata Serrano Lopes , Alessandro Leonardo Alvares Magalhaes, Fernando Antonio Vinhal dos Santos, Erika Lopes Rocha Batista, Marilda Siqueira on behalf of the Fiocruz COVID-19 Genomic Surveillance Network
EPI_ISL_1181536, EPI_ISL_1181576	Laboratorio Central de Saude Publica do Estado do Parana (LACEN-PR)	Laboratory of Respiratory Viruses and Measles, Oswaldo Cruz Institute, FIOCRUZ	Paola Resende, Luciana Appolinario, Fernando Motta, Anna Carolina Paixao, Ana Carolina Mendonca, Alice Sampaio Rocha, Renata Serrano Lopes, Maria do Carmo Debur, Irina Nastassja Riediger, Marilda Siqueira on behalf of the Fiocruz COVID-19 Genomic Surveillance Network
EPI_ISL_1191738, EPI_ISL_1191739	Laboratorio Biologia Molecolare Sars Cov2 - UOC Laboratorio Analisi - Servizio Medicina di Laboratorio, Ospedale "San Francesco" - ATS-ASSL Nuoro	Laboratorio specialistico UOC Ematologia - Ospedale "San Francesco" - ATS-ASSL Nuoro	Piras Giovanna, Asproni Rosanna,,Fiamma Maura, Monne Maria Itria, Palmas Angelo Domenico, Lo Maglio Iana, Mameli Giuseppe
EPI_ISL_1191781	Virology Center Adolfo Lutz Institute Sao Paulo Brazil	Retrovirus Laboratory Adolfo Lutz Institute	Gabriela Bastos Cabral, Giselle I S Lopez-Lopes, Cintia Ahagon, Audrey Cilli, Paula Morena Guimaraes, Igor Mohamed Hussein, Luis Brigido
EPI_ISL_1192077, EPI_ISL_1192078	Sri Venkateswara Institute of Medical Sciences SVIMS, Tirupati	Indian Council of Medical Research-National Institute of Virology, Microbial Containment Complex	Pragya D. Yadav, Usha Kalawat
EPI_ISL_1192079	Viral Research and Diagnostic Laboratory, Dr. R.P. Govt. Medical College,Kangra	Indian Council of Medical Research-National Institute of Virology, Microbial Containment Complex	Pragya D. Yadav, S.C. Jaryal
EPI_ISL_1192080, EPI_ISL_1192081, EPI_ISL_1192082, EPI_ISL_1192083, EPI_ISL_1192084	Sri Venkateswara Institute of Medical Sciences SVIMS, Tirupati	Indian Council of Medical Research-National Institute of Virology, Microbial Containment Complex	Pragya D. Yadav, Usha Kalawat
EPI_ISL_1192092	Indira Gandhi Medical College & Hospital, Shimla	Indian Council of Medical Research-National Institute of Virology, Microbial Containment Complex	Pragya D. Yadav, Santwana Verma
EPI_ISL_1192093, EPI_ISL_1192094	Sri Venkateswara Institute of Medical Sciences SVIMS, Tirupati	Indian Council of Medical Research-National Institute of Virology, Microbial Containment Complex	Pragya D. Yadav, Usha Kalawat
EPI_ISL_1192098, EPI_ISL_1192099	Indira Gandhi Medical College & Hospital, Shimla	Indian Council of Medical Research-National Institute of Virology, Microbial Containment Complex	Pragya D. Yadav, Santwana Verma
EPI_ISL_1192101, EPI_ISL_1192102, EPI_ISL_1192103, EPI_ISL_1192104, EPI_ISL_1192105, EPI_ISL_1192106, EPI_ISL_1192107, EPI_ISL_1192108, EPI_ISL_1192109, EPI_ISL_1192110, EPI_ISL_1192111, EPI_ISL_1192112, EPI_ISL_1192113, EPI_ISL_1192121, EPI_ISL_1192122, EPI_ISL_1192123, EPI_ISL_1192124			
see above	Sri Venkateswara Institute of Medical Sciences SVIMS, Tirupati	Indian Council of Medical Research-National Institute of Virology, Microbial Containment Complex	Pragya D. Yadav, Usha Kalawat
EPI_ISL_1192134, EPI_ISL_1192135, EPI_ISL_1192136, EPI_ISL_1192137, EPI_ISL_1192138, EPI_ISL_1192139, EPI_ISL_1192140, EPI_ISL_1192141, EPI_ISL_1192142, EPI_ISL_1192143, EPI_ISL_1192144, EPI_ISL_1192145, EPI_ISL_1192146, EPI_ISL_1192147, EPI_ISL_1192148			
see above	Indira Gandhi Medical College & Hospital, Shimla	Indian Council of Medical Research-National Institute of Virology, Microbial Containment Complex	Pragya D. Yadav, Santwana Verma
EPI_ISL_1192151, EPI_ISL_1192152, EPI_ISL_1192153, EPI_ISL_1192154, EPI_ISL_1192155, EPI_ISL_1192156, EPI_ISL_1192157, EPI_ISL_1192158	Viral Research and Diagnostic Laboratory, Dr. R.P. Govt. Medical College,Kangra	Indian Council of Medical Research-National Institute of Virology, Microbial Containment Complex	Pragya D. Yadav, S.C. Jaryal
EPI_ISL_1196283, EPI_ISL_1196284	LACEN do Mato Grosso do Sul	Instituto Adolfo Lutz, Interdiciplinary Procedures Center, Strategic Laboratory	Claudio Tavares Sacchi, Claudia Regina Gonçalves, Erica Valessa Ramos Gomes, Karoline Rodrigues Campos, Caio Vinicius Dias Lopes
EPI_ISL_1231314, EPI_ISL_1231315, EPI_ISL_1231316, EPI_ISL_1231317, EPI_ISL_1231318, EPI_ISL_1231319, EPI_ISL_1231320, EPI_ISL_1231321, EPI_ISL_1231322, EPI_ISL_1231323, EPI_ISL_1231324			
see above	Depratment of Microbiology, Viral Research and Diagnostic Laboratory	Indian Council of Medical Research-National Institute of Virology, Microbial Containment Complex	Pragya D. Yadav, Tapan Majumdar
EPI_ISL_1238787, EPI_ISL_1238788, EPI_ISL_1238789, EPI_ISL_1238790, EPI_ISL_1238791	Ecological and Evolutionary Genomics. UGA-CINVESTAV	Unidad Universitaria de Secuenciación Masiva y Bioinformática (UUSMB). IBT-UNAM	Angelica Cibrian
EPI_ISL_1241861	Institute of Life Sciences	Institute of Life Sciences	Sanchari Chatterjee, Ankita Datey, Soumya Sengupta, Sandhya Suranjika, Gargee Bhattacharya, Arup Ghosh, Atimukta Jha, Safal Rajesh Walia, Supriya Suman Keshry, Eshna Laha, Sunil Raghav, Amol Suryawanshi, Rupesh Dash, Shantibhushan Senapati, Tushar K. Beuria, Rajeeb Swain, Gulam Hussain Syed, Punit Prasad, Soma Chattopadhyay ,Odisha COVID-19 Study Group, ILS COVID-19 Team and Ajay Parida.
EPI_ISL_1242007	Institute of Life Sciences	Institute of Life Sciences	Sanchari Chatterjee, Ankita Datey, Soumya Sengupta, Sandhya Suranjika, Gargee Bhattacharya, Arup Ghosh, Atimukta Jha, Safal Rajesh Walia, Supriya Suman Keshry, Eshna Laha, Sunil Raghav, Amol Suryawanshi, Rupesh Dash, Shantibhushan Senapati, Tushar K. Beuria, Rajeeb Swain, Gulam Hussain Syed, Punit Prasad, Soma Chattopadhyay ,Odisha COVID-19 Study Group, ILS COVID-19 Team and Ajay Parida
EPI_ISL_1251264	Government Medical College, Patiala, Punjab	Indian Council of Medical Research-National Institute of Virology, Microbial Containment Complex	Pragya D. Yadav, Rupinder Bakshi
EPI_ISL_1251269	Viral Research and Diagnostic Laboratory, Dr. R.P. Govt. Medical College,Kangra	Indian Council of Medical Research-National Institute of Virology, Microbial Containment Complex	Pragya D. Yadav, S.C. Jaryal
EPI_ISL_1251270, EPI_ISL_1251271	Depratment of Microbiology, Viral Research and Diagnostic Laboratory	Indian Council of Medical Research-National Institute of Virology, Microbial Containment Complex	Pragya D. Yadav, Tapan Majumdar
EPI_ISL_1255113, EPI_ISL_1255114, EPI_ISL_1255115, EPI_ISL_1255117, EPI_ISL_1255228, EPI_ISL_1255242, EPI_ISL_1255244, EPI_ISL_1255245, EPI_ISL_1255246, EPI_ISL_1255247, EPI_ISL_1255248, EPI_ISL_1255249			
see above	West African Centre for Cell Biology of Infectious Pathogens (WACCBIP), University of Ghana, Accra, Ghana	West African Centre for Cell Biology of Infectious Pathogens (WACCBIP), University of Ghana, Volta Road, Legon-Accra, Ghana	Collins M. Morang'a, Joyce M. Ngoi, Evelyn B. Quansah, Samirah Saiid, Dominic S.Y. Amuzu, Vincent Appiah, Philip M. Soglo, Vanessa Magnussen, Aisha Mohammed, Kesego Tapela, Nelson Kibinge, Abdoulaye B Diallo, Frederick Kumi-Ansah, Theophilus Odoom, Oliver D Boakye5, Emmanuella Amoako4, Abdul-Karim Abass, , Samuel Kaba Akoriyea, Frederick Tei-Maya, Lucas N. Amenga-Etego, Dam Kenneth Mibut, Yaw Bediako, Benjamin Demah Nuertey, Gordon A Awandare, Peter K Quashie, Gordon A Awandare, Yaw Bediako
EPI_ISL_1255389, EPI_ISL_1255390	Institute of Life Sciences	Institute of Life Sciences	Sanchari Chatterjee, Ankita Datey, Soumya Sengupta, Sandhya Suranjika, Gargee Bhattacharya, Arup Ghosh, Atimukta Jha, Safal Rajesh Walia, Supriya Suman Keshry, Eshna Laha, Sunil Raghav, Amol Suryawanshi, Rupesh Dash, Shantibhushan Senapati, Tushar K. Beuria, Rajeeb Swain, Gulam Hussain Syed, Punit Prasad, Soma Chattopadhyay ,Odisha COVID-19 Study Group, ILS COVID-19 Team and Ajay Parida
EPI_ISL_1260838, EPI_ISL_1260839	VRDL, GMC, Haldwani , Nainital	Indian Council of Medical Research-National Institute of Virology, Microbial Containment Complex	Pragya D. Yadav, Vinita Rawat
EPI_ISL_1279947	National Institute of Infectious Diseases-Prof. Dr. Matei Bals Molecular Diagnostics Laboratory	National Institute of Infectious Diseases-Prof. Dr. Matei Bals Molecular Diagnostics Laboratory	Leontina Banica, Marius Surleac, Corina Casangiu, Petre Milu, Andreea Tudor, Simona Paraschiv, Dan Otelea
EPI_ISL_1301549	Instituto de Diagnostico y Referencia Epidemiologicos InDRE_RNLSP	Instituto de Biotecnología de la UNAM	Authors from IBT, IMSS, InDRE and INER (in alphabetical order): Carlos F. Arias, Santiago Ávila-Ríos, Gisela Barrera-Badillo, Eduardo Becerril-Vargas, Celia Boukadida, Natividad Cruz-Ortiz, Larissa Fernandes-Matano, Ricardo Grande, Lucia Hernandez-Rivas, Alejandra Hernández-Terán, Pavel Isa, Irma Lopez-Martinez, José Arturo Martínez-Orozco, Margarita Matías-Florentino, Fidencio Mejía-Nepomuceno, Edgar Mendieta-Condado, Mario Mújica-Sánchez,

			José Esteban Muñoz-Medina, Tatiana Nunez-Garcia, Luis Alberto Ochoa-Carrera, Hector Esteban Paz-Juárez, Francisco Pulido, José Ernesto Ramírez-González, Alma Rincón-Rubio, Teresita Rojas-Mendoza, Jorge Salas-Hernández, Alejandro Sanchez-Flores, Clara Esperanza Santacruz-Tinoco, Andrea Santos Coy-Arechavaleta, Blanca Taboada, Gloria Vazquez, Joel Armando Vázquez-Pérez, Jerome Jean Verleyen
EPI_ISL_1301551, EPI_ISL_1301554	Instituto Nacional de Enfermedades Respiratorias (INER)	Instituto de Biotecnología de la UNAM	Authors from IBT, IMSS, InDRE and INER (in alphabetical order): Carlos F. Arias, Santiago Ávila-Ríos, Gisela Barrera-Badillo, Eduardo Becerril-Vargas, Celia Boukadida, Natividad Cruz-Ortiz, Larissa Fernandes-Matano, Ricardo Grande, Lucia Hernandez-Rivas, Alejandra Hernández-Terán, Pavel Isa, Irma Lopez-Martinez, José Arturo Martínez-Orozco, Margarita Matías-Florentino, Fidencio Mejía-Nepomuceno, Edgar Mendieta-Condado, Mario Mújica-Sánchez, José Esteban Muñoz-Medina, Tatiana Nunez-Garcia, Luis Alberto Ochoa-Carrera, Hector Esteban Paz-Juárez, Francisco Pulido, José Ernesto Ramírez-González, Alma Rincón-Rubio, Teresita Rojas-Mendoza, Jorge Salas-Hernández, Alejandro Sanchez-Flores, Clara Esperanza Santacruz-Tinoco, Andrea Santos Coy-Arechavaleta, Blanca Taboada, Gloria Vazquez, Joel Armando Vázquez-Pérez, Jerome Jean Verleyen
EPI_ISL_1301555, EPI_ISL_1301556, EPI_ISL_1301557, EPI_ISL_1301558, EPI_ISL_1301559, EPI_ISL_1301560, EPI_ISL_1301561, EPI_ISL_1301562, EPI_ISL_1301563, EPI_ISL_1301565, EPI_ISL_1301566, EPI_ISL_1301567, EPI_ISL_1301568, EPI_ISL_1301569, EPI_ISL_1301570, EPI_ISL_1301571, EPI_ISL_1301572, EPI_ISL_1301573, EPI_ISL_1301574, EPI_ISL_1301585, EPI_ISL_1301586, EPI_ISL_1301587, EPI_ISL_1301588, EPI_ISL_1301589, EPI_ISL_1301590, EPI_ISL_1301592			
see above	Instituto de Diagnostico y Referencia Epidemiologicos InDRE_RNLSP	Instituto de Biotecnología de la UNAM	Authors from IBT, IMSS, InDRE and INER (in alphabetical order): Carlos F. Arias, Santiago Ávila-Ríos, Gisela Barrera-Badillo, Eduardo Becerril-Vargas, Celia Boukadida, Natividad Cruz-Ortiz, Larissa Fernandes-Matano, Ricardo Grande, Lucia Hernandez-Rivas, Alejandra Hernández-Terán, Pavel Isa, Irma Lopez-Martinez, José Arturo Martínez-Orozco, Margarita Matías-Florentino, Fidencio Mejía-Nepomuceno, Edgar Mendieta-Condado, Mario Mújica-Sánchez, José Esteban Muñoz-Medina, Tatiana Nunez-Garcia, Luis Alberto Ochoa-Carrera, Hector Esteban Paz-Juárez, Francisco Pulido, José Ernesto Ramírez-González, Alma Rincón-Rubio, Teresita Rojas-Mendoza, Jorge Salas-Hernández, Alejandro Sanchez-Flores, Clara Esperanza Santacruz-Tinoco, Andrea Santos Coy-Arechavaleta, Blanca Taboada, Gloria Vazquez, Joel Armando Vázquez-Pérez, Jerome Jean Verleyen
EPI_ISL_1301597	Laboratorio Central de Epidemiología IMSS	Instituto de Biotecnología de la UNAM	Authors from IBT, IMSS, InDRE and INER (in alphabetical order): Carlos F. Arias, Santiago Ávila-Ríos, Gisela Barrera-Badillo, Eduardo Becerril-Vargas, Celia Boukadida, Natividad Cruz-Ortiz, Larissa Fernandes-Matano, Ricardo Grande, Lucia Hernandez-Rivas, Alejandra Hernández-Terán, Pavel Isa, Irma Lopez-Martinez, José Arturo Martínez-Orozco, Margarita Matías-Florentino, Fidencio Mejía-Nepomuceno, Edgar Mendieta-Condado, Mario Mújica-Sánchez, José Esteban Muñoz-Medina, Tatiana Nunez-Garcia, Luis Alberto Ochoa-Carrera, Hector Esteban Paz-Juárez, Francisco Pulido, José Ernesto Ramírez-González, Alma Rincón-Rubio, Teresita Rojas-Mendoza, Jorge Salas-Hernández, Alejandro Sanchez-Flores, Clara Esperanza Santacruz-Tinoco, Andrea Santos Coy-Arechavaleta, Blanca Taboada, Gloria Vazquez, Joel Armando Vázquez-Pérez, Jerome Jean Verleyen
EPI_ISL_1301602, EPI_ISL_1301603, EPI_ISL_1301604, EPI_ISL_1301618, EPI_ISL_1301619	Instituto Nacional de Enfermedades Respiratorias (INER)	Instituto de Biotecnología de la UNAM	Authors from IBT, IMSS, InDRE and INER (in alphabetical order): Carlos F. Arias, Santiago Ávila-Ríos, Gisela Barrera-Badillo, Eduardo Becerril-Vargas, Celia Boukadida, Natividad Cruz-Ortiz, Larissa Fernandes-Matano, Ricardo Grande, Lucia Hernandez-Rivas, Alejandra Hernández-Terán, Pavel Isa, Irma Lopez-Martinez, José Arturo Martínez-Orozco, Margarita Matías-Florentino, Fidencio Mejía-Nepomuceno, Edgar Mendieta-Condado, Mario Mújica-Sánchez, José Esteban Muñoz-Medina, Tatiana Nunez-Garcia, Luis Alberto Ochoa-Carrera, Hector Esteban Paz-Juárez, Francisco Pulido, José Ernesto Ramírez-González, Alma Rincón-Rubio, Teresita Rojas-Mendoza, Jorge Salas-Hernández, Alejandro Sanchez-Flores, Clara Esperanza Santacruz-Tinoco, Andrea Santos Coy-Arechavaleta, Blanca Taboada, Gloria Vazquez, Joel Armando Vázquez-Pérez, Jerome Jean Verleyen
EPI_ISL_1301672, EPI_ISL_1301673, EPI_ISL_1301674, EPI_ISL_1301675, EPI_ISL_1301676, EPI_ISL_1301677, EPI_ISL_1301678	Laboratorio Central de Epidemiología IMSS	Instituto de Biotecnología de la UNAM	Authors from IBT, IMSS, InDRE and INER (in alphabetical order): Carlos F. Arias, Santiago Ávila-Ríos, Gisela Barrera-Badillo, Eduardo Becerril-Vargas, Celia Boukadida, Natividad Cruz-Ortiz, Larissa Fernandes-Matano, Ricardo Grande, Lucia Hernandez-Rivas, Alejandra Hernández-Terán, Pavel Isa, Irma Lopez-Martinez, José Arturo Martínez-Orozco, Margarita Matías-Florentino, Fidencio Mejía-Nepomuceno, Edgar Mendieta-Condado, Mario Mújica-Sánchez, José Esteban Muñoz-Medina, Tatiana Nunez-Garcia, Luis Alberto Ochoa-Carrera, Hector Esteban Paz-Juárez, Francisco Pulido, José Ernesto Ramírez-González, Alma Rincón-Rubio, Teresita Rojas-Mendoza, Jorge Salas-Hernández, Alejandro Sanchez-Flores, Clara Esperanza Santacruz-Tinoco, Andrea Santos Coy-Arechavaleta, Blanca Taboada, Gloria Vazquez, Joel Armando Vázquez-Pérez, Jerome Jean Verleyen
EPI_ISL_1301688, EPI_ISL_1301689, EPI_ISL_1301691, EPI_ISL_1301692, EPI_ISL_1301693, EPI_ISL_1301695, EPI_ISL_1301696, EPI_ISL_1301697, EPI_ISL_1301698, EPI_ISL_1301699, EPI_ISL_1301700, EPI_ISL_1301701, EPI_ISL_1301702, EPI_ISL_1301703, EPI_ISL_1301704, EPI_ISL_1301705, EPI_ISL_1301706, EPI_ISL_1301707, EPI_ISL_1301708, EPI_ISL_1301711, EPI_ISL_1301712, EPI_ISL_1301713			
see above	Instituto de Diagnostico y Referencia Epidemiologicos InDRE_RNLSP	Instituto de Biotecnología de la UNAM	Authors from IBT, IMSS, InDRE and INER (in alphabetical order): Carlos F. Arias, Santiago Ávila-Ríos, Gisela Barrera-Badillo, Eduardo Becerril-Vargas, Celia Boukadida, Natividad Cruz-Ortiz, Larissa Fernandes-Matano, Ricardo Grande, Lucia Hernandez-Rivas, Alejandra Hernández-Terán, Pavel Isa, Irma Lopez-Martinez, José Arturo Martínez-Orozco, Margarita Matías-Florentino, Fidencio Mejía-Nepomuceno, Edgar Mendieta-Condado, Mario Mújica-Sánchez, José Esteban Muñoz-Medina, Tatiana Nunez-Garcia, Luis Alberto Ochoa-Carrera, Hector Esteban Paz-Juárez, Francisco Pulido, José Ernesto Ramírez-González, Alma Rincón-Rubio, Teresita Rojas-Mendoza, Jorge Salas-Hernández, Alejandro Sanchez-Flores, Clara Esperanza Santacruz-Tinoco, Andrea Santos Coy-Arechavaleta, Blanca Taboada, Gloria Vazquez, Joel Armando Vázquez-Pérez, Jerome Jean Verleyen
EPI_ISL_1301725, EPI_ISL_1301726, EPI_ISL_1301727, EPI_ISL_1301728, EPI_ISL_1301729	Instituto Nacional de Enfermedades Respiratorias (INER)	Instituto de Biotecnología de la UNAM	Authors from IBT, IMSS, InDRE and INER (in alphabetical order): Carlos F. Arias, Santiago Ávila-Ríos, Gisela Barrera-Badillo, Eduardo Becerril-Vargas, Celia Boukadida, Natividad Cruz-Ortiz, Larissa Fernandes-Matano, Ricardo Grande, Lucia Hernandez-Rivas, Alejandra Hernández-Terán, Pavel Isa, Irma Lopez-Martinez, José Arturo Martínez-Orozco, Margarita Matías-Florentino, Fidencio Mejía-Nepomuceno, Edgar Mendieta-Condado, Mario Mújica-Sánchez, José Esteban Muñoz-Medina, Tatiana Nunez-Garcia, Luis Alberto Ochoa-Carrera, Hector Esteban Paz-Juárez, Francisco Pulido, José Ernesto Ramírez-González, Alma Rincón-Rubio, Teresita Rojas-Mendoza, Jorge Salas-Hernández, Alejandro Sanchez-Flores, Clara Esperanza Santacruz-Tinoco, Andrea Santos Coy-Arechavaleta, Blanca Taboada, Gloria Vazquez, Joel Armando Vázquez-Pérez, Jerome Jean Verleyen
EPI_ISL_1301730	Instituto de Diagnostico y Referencia Epidemiologicos InDRE_RNLSP	Instituto de Biotecnología de la UNAM	Authors from IBT, IMSS, InDRE and INER (in alphabetical order): Carlos F. Arias, Santiago Ávila-Ríos, Gisela Barrera-Badillo, Eduardo Becerril-Vargas, Celia Boukadida, Natividad Cruz-Ortiz, Larissa Fernandes-Matano, Ricardo Grande, Lucia Hernandez-Rivas, Alejandra Hernández-Terán, Pavel Isa, Irma Lopez-Martinez, José Arturo Martínez-Orozco, Margarita Matías-Florentino, Fidencio Mejía-Nepomuceno, Edgar Mendieta-Condado, Mario Mújica-Sánchez, José Esteban Muñoz-Medina, Tatiana Nunez-Garcia, Luis Alberto Ochoa-Carrera, Hector Esteban Paz-Juárez, Francisco Pulido, José Ernesto Ramírez-González, Alma Rincón-Rubio, Teresita Rojas-Mendoza, Jorge Salas-Hernández, Alejandro Sanchez-Flores, Clara Esperanza Santacruz-Tinoco, Andrea Santos Coy-Arechavaleta, Blanca Taboada, Gloria Vazquez, Joel Armando Vázquez-Pérez, Jerome Jean Verleyen
EPI_ISL_1301732, EPI_ISL_1301733	Instituto Nacional de Enfermedades Respiratorias (INER)	Instituto de Biotecnología de la UNAM	Authors from IBT, IMSS, InDRE and INER (in alphabetical order): Carlos F. Arias, Santiago Ávila-Ríos, Gisela Barrera-Badillo, Eduardo Becerril-Vargas, Celia Boukadida, Natividad Cruz-Ortiz, Larissa Fernandes-Matano, Ricardo Grande, Lucia Hernandez-Rivas, Alejandra Hernández-Terán, Pavel Isa, Irma Lopez-Martinez, José Arturo Martínez-Orozco, Margarita Matías-Florentino, Fidencio Mejía-Nepomuceno, Edgar Mendieta-Condado, Mario Mújica-Sánchez, José Esteban Muñoz-Medina, Tatiana Nunez-Garcia, Luis Alberto Ochoa-Carrera, Hector Esteban Paz-Juárez, Francisco Pulido, José Ernesto Ramírez-González, Alma Rincón-Rubio, Teresita Rojas-Mendoza, Jorge Salas-Hernández, Alejandro Sanchez-Flores, Clara Esperanza Santacruz-Tinoco, Andrea Santos Coy-Arechavaleta, Blanca Taboada, Gloria Vazquez, Joel Armando Vázquez-Pérez, Jerome Jean Verleyen
EPI_ISL_1335896	Columbia Asia Hospital Medan	Faculty of Medicine, Universitas Sumatera Utara; Institute of Tropical Disease, Universitas Airlangga	Aldise M Nastri, Jezzy R Dewantari, Rima R Prasetya, Krisnoadi Rahardjo, Yasuko Mori, Soetjipto, Kazufumi Shimizu, Maria I Lusida, Meliani, R Andika D Cahyadi, Mirzan Hasibuan, Irbah R Nainggolan, Ramadhan Bestari, Franciscus Ginting, Muhammad Ichwan, R Lia Kusumawati, Inke N D Lubis
EPI_ISL_1340760, EPI_ISL_1340763	Departamento de Virologia, Laboratorio Central de Salud Pública, Avenida Venezuela y Teniente Escurra, Asunción, Paraguay	Laboratory of Respiratory Viruses and Measles, Oswaldo Cruz Institute, FIOCRUZ	Paola Resende, Cynthia Vazquez, Luciana Appolinario, Fernando Motta, Anna Carolina Paixao, Ana Carolina Mendonca, Alice Sampaio Rocha, Renata Serrano Lopes, Marilda Siqueira on behalf of the Fiocruz COVID-19 Genomic Surveillance Network
EPI_ISL_1357605	Dr. Ann Chahroudi's Lab	Dr. Thomas Vanderford's Lab	Thomas Vanderford, Maud Mavigner, Nils Schoof, Ann Chahroudi
EPI_ISL_1372187	Cliniques universitaires Saint-Luc	UCLouvain/IREC/MBLG	Jean Ruelle, Lysa Pinsmaye, Eleonore Ngyuvula Mantu, Benoit Kabamba Mukadi
EPI_ISL_1398364	Emam Ali Hospital	Razi Vaccine and Serum Research Institute	Amir Kaffashi, Jiabin Huang, S Reza Banihashemi, Mohammad Hossein Fallah Mehrabadi, Mohsen Lotfi, Morteza Taghizadeh, Akbar Khorasani, Mohsen Bashashati, Sayed Hamidreza Mozhgani
EPI_ISL_1398925	Emam Ali Hospital	Razi Vaccine and Serum Research Institute	Amir Kaffashi, Jiabin Huang, S Reza Banihashemi, Mohammad Hossein Fallah Mehrabadi, Mohsen Lotfi, Morteza Taghizadeh, Akbar Khorasani, Mohsen Bashashati, Sayed Hamidreza Mozhgani
EPI_ISL_1404131, EPI_ISL_1404132, EPI_ISL_1404133, EPI_ISL_1404134, EPI_ISL_1404135, EPI_ISL_1404136, EPI_ISL_1404137, EPI_ISL_1404138, EPI_ISL_1404139, EPI_ISL_1404140, EPI_ISL_1404141, EPI_ISL_1404142			
see above	HCRC	HCRC	Laurens Lambrechts, Jolien Van Cleemput, Nick Vereecke, Sebastiaan Theuns, Janneke Cox, Piet Maes, Linos Vandekerckhove
EPI_ISL_1406696	Laboratory of Respiratory Viruses Teaching and Clinical Center of the Medical University of Lodz	Biobank Lab, Department of Molecular Biophysics, Faculty of Biology and Environmental Protection, University of Lodz	Strapagiel Dominik, Sobalska-Kwapis Marta, Dród Izabela, Tymoniuk Bogusaw, Traczyk-Borszyska Magdalena, Dastych Jaroslaw, Dziadek Jarosaw, Paradowska Edyta, Borowiec Maciej.
EPI_ISL_1416165, EPI_ISL_1416192,	Institut National d'hygiène	"Unité Mixte Internationale TransVIHMI (UMI 233 IRD -	Mounerou SALOU, Christelle BUTEL, Wembo A. HALATOKO,Issaka Maman, Abla A. KONOU, Amivi EHLAN, Adodo SADJI, Kokou TEGUENI,Sidonie

EPI_ISL_1416222		U1175 INSERM - Université de Montpellier) IRD (Institut de recherche pour le développement)"	A.M.KAGNISSODE, Akoélé SILIADIN, Alassane OURO-MEDELI, Messanh DOUFFAN,Déléma MABA,Sika DOSSIM, Améyo DORKENOO, Mireille PRINCE-DAVID,Anoumou DAGNRA,Laetitia SERRANO,Ahidjo AYOUBA,Eric DELAPORTE, Martine PEETERS
EPI_ISL_1424060, EPI_ISL_1424063	Instituto Nacional de Salud- Dirección de Investigación en Salud Pública	Instituto Nacional de Salud- Dirección de Investigación en Salud Pública	Katherine Laiton-Donato, Carlos Franco-Muñoz, Diego A. Álvarez-Díaz, Mauricio Pacheco, Jhonnatan Reales-González, Diego Andrés Prada, Sheryll Corchuelo, Maria T. Herrera-Sepúlveda, Julian Naizaque, Gerardo Santamaría, Magdalena Wiesner, Martha Lucia Ospina Martínez, Marcela Mercado-Reyes.
EPI_ISL_1433132, EPI_ISL_1434443, EPI_ISL_1435763, EPI_ISL_1436817	Institut National d'Hygiène	Unité Mixte Internationale TransVIHMI (UMI 233 IRD - U1175 INSERM - Université de Montpellier) IRD (Institut de recherche pour le développement)	Mounerou SALOU, Christelle BUTEL, Wembo A. HALATOKO,Issaka Maman, Abla A. KONOU, Amivi EHLAN, Adodo SADJI, Kokou TEGUENI,Sidonie A.M.KAGNISSODE, Akoélé SILIADIN, Alassane OURO-MEDELI, Messanh DOUFFAN,Déléma MABA,Sika DOSSIM, Améyo DORKENOO, Mireille PRINCE-DAVID,Anoumou DAGNRA,Laetitia SERRANO,Ahidjo AYOUBA,Eric DELAPORTE, Martine PEETERS
EPI_ISL_1447347	Diagnostic and Research Center of Infectious Diseases, Medical Faculty, Andalas University	Diagnostic and Research Center of Infectious Diseases, Medical Faculty, Andalas University	Andani Eka Putra, Syafrizayanti, Ikhwan R. Sudji, Linosefa, Dede Rahman Agustian, Ayu Novita Trisnawati, Siskalil Fahma, Nia Ayuni Putri, Sekar Asri Tresnaningtyas, SM Rezvi, Fauzul Azhim, Dessy Arisanty, Gestina Aliska, Desmawati, Nita Afriani ,Syandrez Prima Putra, Juane Plantika Menra, Yolani Syaputri, Mutia Lailani
EPI_ISL_1468432, EPI_ISL_1468434	LACEN do Mato Grosso do Sul	Instituto Adolfo Lutz, Interdiciplinary Procedures Center, Strategic Laboratory	Claudio Tavares Sacchi, Claudia Regina Gonçalves, Erica Valessa Ramos Gomes, Karoline Rodrigues Campos, Caio Vinicius Dias Lopes
EPI_ISL_1469587	Hospital Sapiranga	Epiclin	Fernando Hayashi Sant'Anna, Ana Paula Muterle, Janira Prichula, Juliana Comerlato, Carolina Comerlato, Eliana Márcia Da Ros Wendland
EPI_ISL_1469713, EPI_ISL_1469724	Fundação Hospitalar de Sapucaia do Sul	Epiclin	Fernando Hayashi Sant'Anna, Ana Paula Muterle, Janira Prichula, Juliana Comerlato, Carolina Comerlato, Eliana Márcia Da Ros Wendland
EPI_ISL_1469840	Hospital Sapiranga	Epiclin	Fernando Hayashi Sant'Anna, Ana Paula Muterle, Janira Prichula, Juliana Comerlato, Carolina Comerlato, Eliana Márcia Da Ros Wendland
EPI_ISL_1489924, EPI_ISL_1489925, EPI_ISL_1489926	Laboratory of Respiratory Viruses Teaching and Clinical Center of the Medical University of Lodz	Biobank Lab, Department of Molecular Biophysics, Faculty of Biology and Environmental Protection, University of Lodz	Strapagiel Dominik, Sobalska-Kwapis Marta, Dród Izabela, Tymoniuk Bogusaw, Traczyk-Borszyska Magdalena, Dastych Jaroslaw, Dziadek Jarosaw, Paradowska Edyta, Borowiec Maciej.
EPI_ISL_1494717	Centro de Investigación en Ciencias de la Salud y Biomedicina, U.A.S.L.P	Centro de Investigación en Ciencias de la Salud y Biomedicina, U.A.S.L.P	MD PhD Sofía Bernal Silva
EPI_ISL_1494725, EPI_ISL_1494726, EPI_ISL_1494727	Centro de Investigación en Ciencias de la Salud y Biomedicina, U.A.S.L.P.	Centro de Investigación en Ciencias de la Salud y Biomedicina, U.A.S.L.P.	MD PhD Sofía Bernal Silva
EPI_ISL_1495043, EPI_ISL_1495045, EPI_ISL_1495047, EPI_ISL_1495049, EPI_ISL_1495051, EPI_ISL_1495053, EPI_ISL_1495054	Laboratory of Respiratory Viruses Teaching and Clinical Center of the Medical University of Lodz	Biobank Lab, Department of Molecular Biophysics, Faculty of Biology and Environmental Protection, University of Lodz	Strapagiel Dominik, Sobalska-Kwapis Marta, Dród Izabela, Tymoniuk Bogusaw, Traczyk-Borszyska Magdalena, Dastych Jaroslaw, Dziadek Jarosaw, Paradowska Edyta, Borowiec Maciej.
EPI_ISL_1500866	Centro de Investigación en Ciencias de la Salud y Biomedicina, U.A.S.L.P.	Centro de Investigación en Ciencias de la Salud y Biomedicina, U.A.S.L.P.	MD PhD Sofía Bernal Silva
EPI_ISL_1502848, EPI_ISL_1502850, EPI_ISL_1502851, EPI_ISL_1502852, EPI_ISL_1502853, EPI_ISL_1502854, EPI_ISL_1502855, EPI_ISL_1502856, EPI_ISL_1502857, EPI_ISL_1502858, EPI_ISL_1502859, EPI_ISL_1502860			
see above	Gorgas Memorial Laboratory of Health Studies	Gorgas Memorial Laboratory of Health Studies	Gonzalez Claudia, Leyda Abrego, Moreno Ambar, Oris Chavarria, Jessica Gondola, Marlenne Castillo, Ortiz Alma, Castillo Jorge, Moreno Brechla, Franco Danilo, Lopez-Verges Sandra, Martinez Alexander
EPI_ISL_1502865	Hospital Regional Dr. Luis "Chicho" Fábrega	Gorgas Memorial Laboratory of Health Studies	Gonzalez Claudia, Herrera Melissa, Herrera Genarino, Leyda Abrego, Moreno Ambar, Oris Chavarria, Jessica Gondola, Marlenne Castillo, Ortiz Alma, Castillo Jorge, Moreno Brechla, Franco Danilo, Lopez-Verges Sandra, Martinez Alexander
EPI_ISL_1502866, EPI_ISL_1502871, EPI_ISL_1502874, EPI_ISL_1502876, EPI_ISL_1502877, EPI_ISL_1502879, EPI_ISL_1502881, EPI_ISL_1502884, EPI_ISL_1502885, EPI_ISL_1502886	Gorgas Memorial Laboratory of Health Studies	Gorgas Memorial Laboratory of Health Studies	Gonzalez Claudia, Leyda Abrego, Moreno Ambar, Oris Chavarria, Jessica Gondola, Marlenne Castillo, Ortiz Alma, Castillo Jorge, Moreno Brechla, Franco Danilo, Lopez-Verges Sandra, Martinez Alexander
EPI_ISL_1510667, EPI_ISL_1510668	University of Rome Tor Vergata: Departm Experim Medicine Chair of Virology	University of Rome Tor Vergata: Departm Experim Medicine Chair of Virology	Maria Concetta Bellocchi
EPI_ISL_1517392	Inciensa, Instituto Costarricense de Investigación y Enseñanza en Nutrición y Salud	Inciensa, Instituto Costarricense de Investigación y Enseñanza en Nutrición y Salud	Cristian Peréz-Corrales, Valeria Peralta-Barquero & Patricia Salas
EPI_ISL_1517401, EPI_ISL_1517402, EPI_ISL_1517403	Inciensa, Instituto Costarricense de Investigación y Enseñanza en Nutrición y Salud	Inciensa, Instituto Costarricense de Investigación y Enseñanza en Nutrición y Salud	Cristian Peréz-Corrales & Valeria Peralta-Barquero
EPI_ISL_1517404	Inciensa, Instituto Costarricense de Investigación y Enseñanza en Nutrición y Salud	Inciensa, Instituto Costarricense de Investigación y Enseñanza en Nutrición y Salud	Cristian Peréz-Corrales, Valeria Peralta-Barquero & Mauricio Bolaños
EPI_ISL_1517405, EPI_ISL_1517406, EPI_ISL_1517407	Inciensa, Instituto Costarricense de Investigación y Enseñanza en Nutrición y Salud	Inciensa, Instituto Costarricense de Investigación y Enseñanza en Nutrición y Salud	Cristian Peréz-Corrales, Valeria Peralta-Barquero & Gallegos-Carrillo B
EPI_ISL_1527373, EPI_ISL_1527374, EPI_ISL_1527375, EPI_ISL_1527376, EPI_ISL_1527377, EPI_ISL_1527378, EPI_ISL_1527379, EPI_ISL_1527380, EPI_ISL_1527381	Molecular diagnostic laboratory of Federal Budget Institution of Science "Central Research Institute of Epidemiology" of The Federal Service on Customers' Rights Protection and Human Well-being Surveillance	Group of Genomics and Postgenomic Technologies of Central Research Institute of Epidemilology	Samoilov AE, Kaptelova VV, Korneenko EV, Saenko SS, Shipulina OY, Speranskaya AS, Tivanova EV, Kondrasheva LY, Akimkin VG
EPI_ISL_1532282, EPI_ISL_1532283, EPI_ISL_1532284, EPI_ISL_1532285	Oman-National Influenza Center	Biotechnology & OMICs Laboratory	Ahmed Al Harrasi, Aisha Al-Amri, Intisar Al-Shukri, Amal Al-Maani, Sajjad Asaf, Bilal Hussain, Samiya Al-Zadjali, Ahmed N Al-Rawahi, Saqib Bilal, Abdul Latif Khan, Samira Al-Mahruqi, Ahmed Al-Rawahi, Hanan Al-Kindi, Amina Al-Jardani.
EPI_ISL_1532286, EPI_ISL_1532287, EPI_ISL_1532288, EPI_ISL_1532289	Oman-National Influenza Center	Biotechnology & OMICs Laboratory	Intisar Al-Shukri, Ahmed Al Harrasi, Aisha Al-Amri, Amal Al-Maani, Sajjad Asaf, Fahad Al-Balushi, Laila Al-Balushi, Ahmed N Al-Rawahi, Saqib Bilal, Abdul Latif Khan, Samira Al-Mahruqi, Ahmed Al-Rawahi, Hanan Al-Kindi, Amina Al-Jardani.
EPI_ISL_1532290	Oman-National Influenza Center	Biotechnology & OMICs Laboratory	Aisha Al-Amri, Ahmed Al Harrasi, Intisar Al-Shukri, Amal Al-Maani, Sajjad Asaf, Bilal Hussain, Samiya Al-Zadjali, Ahmed N Al-Rawahi, Saqib Bilal, Abdul Latif Khan, Samira Al-Mahruqi, Ahmed Al-Rawahi, Hanan Al-Kindi, Amina Al-Jardani.
EPI_ISL_1532302	Oman-National Influenza Center	Biotechnology & OMICs Laboratory	Intisar Al-Shukri, Ahmed Al Harrasi, Aisha Al-Amri, Amal Al-Maani, Sajjad Asaf, Fahad Al-Balushi, Laila Al-Balushi, Ahmed N Al-Rawahi, Saqib Bilal, Abdul Latif Khan, Samira Al-Mahruqi, Ahmed Al-Rawahi, Hanan Al-Kindi, Amina Al-Jardani.
EPI_ISL_1532303	Oman-National Influenza Center	Biotechnology & OMICs Laboratory	Aisha Al-Amri, Ahmed Al Harrasi, Intisar Al-Shukri, Amal Al-Maani, Sajjad Asaf, Bilal Hussain, Samiya Al-Zadjali, Ahmed N Al-Rawahi, Saqib Bilal, Abdul Latif Khan, Samira Al-Mahruqi, Ahmed Al-Rawahi, Hanan Al-Kindi, Amina Al-Jardani.
EPI_ISL_1532305	Oman-National Influenza Center	Biotechnology & OMICs Laboratory	Abdul Latif Khan, Aisha Al-Amri, Intisar Al-Shukri, Amal Al-Maani, Sajjad Asaf, Bilal Hussain, Samiya Al-Zadjali, Ahmed N Al-Rawahi, Saqib Bilal, Samira Al-Mahruqi, Ahmed Al-Rawahi, Hanan Al-Kindi, Amina Al-Jardani, Ahmed Al Harrasi.
EPI_ISL_1532794	Microbiology Department. Complexo Hospitalario Universitario de Vigo	Microbiology Department. Complexo Hospitalario Universitario de Vigo	Alfaya N, Alonso I, Alvarez M, Cabrera JJ, Carballo R, Cores O, Cortizo S, del-Campo V, Martinez L, Mediero G, Perez S, Potel C, Regueiro B, Rey S, Vasallo FJ
EPI_ISL_1533066, EPI_ISL_1533077, EPI_ISL_1533094	Diagnostic and Research Center of Infectious Diseases, Medical Faculty, Andalas University	Diagnostic and Research Center of Infectious Diseases, Medical Faculty, Andalas University	Andani Eka Putra, Syafrizayanti, Ikhwan R. Sudji, Linosefa, Dede Rahman Agustian, Ayu Novita Trisnawati, Siskalil Fahma, Nia Ayuni Putri, Sekar Asri Tresnaningtyas, SM Rezvi, Fauzul Azhim, Dessy Arisanty, Gestina Aliska, Desmawati, Nita Afriani ,Syandrez Prima Putra, Juane Plantika Menra, Yolani Syaputri, Mutia Lailani
EPI_ISL_1543934	Laboratory of Respiratory Viruses Teaching and Clinical Center of the Medical University of Lodz	Laboratory of Respiratory Viruses Teaching and Clinical Center of the Medical University of Lodz	Borowiec Maciej, Strapagiel Dominik, Sobalska-Kwapis Marta, Dród Izabela, Tymoniuk Bogusaw, Traczyk-Borszyska Magdalena, Dastych Jaroslaw, Dziadek Jarosaw, Paradowska Edyta
EPI_ISL_1543937, EPI_ISL_1543985,	Laboratory of Respiratory Viruses Teaching and Clinical	Laboratory of Respiratory Viruses Teaching and Clinical	Borowiec Maciej, Strapagiel Dominik, Sobalska-Kwapis Marta, Dród Izabela, Tymoniuk Bogusaw, Traczyk-Borszyska Magdalena, Dastych Jaroslaw, Dziadek

EPI_ISL_1543987	Center of the Medical University of Lodz	Center of the Medical University of Lodz	Jarosaw, Paradowska Edyta,
EPI_ISL_1550388	Instituto Oswaldo Cruz	Laboratorio de Virologia, Faculdade de Medicina, Universidade Federal de Mato Grosso, campus Cuiabá	María de Fátima Ferreira, Renata Dezengrini Slhessarenko, Janeth Aracely Ramirez Pavon, Luciano Nakazato, Valeria Dutra, Rosane Christiane Hahn, Paola Cristina Resende
EPI_ISL_1626483, EPI_ISL_1626485, EPI_ISL_1626486, EPI_ISL_1626487, EPI_ISL_1626491, EPI_ISL_1626492, EPI_ISL_1626494, EPI_ISL_1626496, EPI_ISL_1626497, EPI_ISL_1626498, EPI_ISL_1626503, EPI_ISL_1626504, EPI_ISL_1626505, EPI_ISL_1626506, EPI_ISL_1626507, EPI_ISL_1626508, EPI_ISL_1626509, EPI_ISL_1626510, EPI_ISL_1626511, EPI_ISL_1626513, EPI_ISL_1626518			
see above	NSTU COVID-19 Diagnostic Center	NSU Genome Research Institute (NGRI)	Maqsud Hossain, Tahrima Saiha Huq, Aura Rahman, Md. Aminul Islam, Syeda Naushin Tabassum, Kazi Nadim Hasan, Abdul Khaleque, Abdus Sadique, Mohammad Salim Hossain, Newaz Mohammed Bahadur, Firoz Ahmed, Hasan Mahmud Reza
EPI_ISL_1672647	ID and BG Hospital / CSIR - Indian Institute of Chemical Biology (CSIR-IICB)	CSIR Institute of Genomics and Integrative Biology (CSIR-IGIB)	Rajesh Pandey, Janani SV, Ranjeet Maurya, Akshay Kanakan, Neha Jha, Priyanka Mehta, Partha Chattopadhyay, Priti Devi, Shweta Sahni, Azka Khan, Sachin Sharma, Yogiraj Ray, Shekhar Ranjan Paul, Sandip Paul, Debaleena Bhowmik, Dipyaman Ganguly
EPI_ISL_1701311	Laboratório de diagnóstico molecular da COVID-19 - Bahia, Santo Antônio de Jesus, Brazil	Laboratório Baculovírus	Josilene R Pinheiro, Jaime H Amorim, Aline Belmok, Fernando L Melo, Bergmann M Ribeiro
EPI_ISL_1708505, EPI_ISL_1708506	Collection of WHO National Influenza Center, N.F. Gamaleya National Research Center for Epidemiology and Microbiology of the Ministry of Health of the Russian Federation	Laboratory of genome analysis, N.F. Gamaleya National Research Center for Epidemiology and Microbiology of the Ministry of Health of the Russian Federation	Olga Voronina, Ekaterina Aksenova, Natalia Ryzhova, Marina Kunda, Kirill Krasnoslobodtsev, Elena Burtseva, Lyudmila Kolobukhina, Svetlana Smetanina, Denis Logunov
EPI_ISL_1708507, EPI_ISL_1708510	State collection of viruses, N.F. Gamaleya National Research Center for Epidemiology and Microbiology of the Ministry of Health of the Russian Federation	Laboratory of genome analysis, N.F. Gamaleya National Research Center for Epidemiology and Microbiology of the Ministry of Health of the Russian Federation	Olga Voronina, Ekaterina Aksenova, Natalia Ryzhova, Marina Kunda, Inna Dolzhikova, Andrey Botikov, Daria Grousova, Anna Kovyrshina, Denis Logunov
EPI_ISL_1716880, EPI_ISL_1752589, EPI_ISL_1752590, EPI_ISL_1752591	Showa University PCR Center	Showa University PCR Center	Fumihiro Ishikawa, Hideto Oyamada, Yuko Udaka, Keiko Ishino, Yuji Kiuchi
EPI_ISL_1761308, EPI_ISL_1761309	Tokyo Medical and Dental University	Tokyo Medical and Dental University	Kousuke Tanimoto, Yukie Tanaka, Yoko Nukui, Akinori Kimura, Hiroaki Takeuchi
EPI_ISL_1795043, EPI_ISL_1795044, EPI_ISL_1795045, EPI_ISL_1795046, EPI_ISL_1795047	Oman-NIC	Department of Microbiology and Immunology-SQUH	Fahad Zadjali, Samira Al-Maruqi, Amina Al Jardani, Khulood Al-Mammary, Hanan Al-kindi, Fatma BaAlawi, Hamida AL Barwani, Zeyana AL-Dahmani, Intisar Al-Shukri, Aisha Al-Busaidi, Aisha Al-Amri, Ahlam Al-Amri, Mohammed Al-Tobi, Samiha Al Kharusi, Abdulla Balkhair
EPI_ISL_1795048	Oman-NIC	Department of Microbiology and Immunology-SQUH	Samira Al-Maruqi,Fahad Zadjali, Amina Al Jardani, Khulood Al-Mammary, Hanan Al-kindi, Fatma BaAlawi, Hamida AL Barwani, Zeyana AL-Dahmani, Intisar Al-Shukri, Aisha Al-Busaidi, Aisha Al-Amri, Ahlam Al-Amri, Mohammed Al-Tobi, Samiha Al Kharusi, Abdulla Balkhair
EPI_ISL_1795049, EPI_ISL_1795050, EPI_ISL_1795051, EPI_ISL_1795052	Oman-NIC	Oman-National Influenza Center-Department of Microbiology and Immunology-SQUH	Samira Al-Maruqi,Fahad Zadjali, Amina Al Jardani, Khulood Al-Mammary, Hanan Al-kindi, Fatma BaAlawi, Hamida AL Barwani, Zeyana AL-Dahmani, Intisar Al-Shukri, Aisha Al-Busaidi, Aisha Al-Amri, Ahlam Al-Amri, Mohammed Al-Tobi, Samiha Al Kharusi, Abdulla Balkhair
EPI_ISL_1799498	Laboratório de Microbiologia Molecular - Universidade FEEVALE	Molecular Microbiology Laboratory	Alana Witt Hansen, Fágner Henrique Heldt, Fernando Rosado Spilki, Flávio Silveira, Juliana Schons Gularte, Juliane Deise Fleck, Mariana Soares da Silva, Meriane Demoliner, Matheus Nunes Weber, Paula Rodrigues de Almeida, Micheli Filippi.
EPI_ISL_1799499, EPI_ISL_1799501, EPI_ISL_1799502, EPI_ISL_1799505, EPI_ISL_1799507	Laboratório de Microbiologia Molecular - Universidade FEEVALE	Molecular Microbiology Laboratory	Alana Witt Hansen, Fágner Henrique Heldt, Fernando Rosado Spilki, Flávio Silveira, Juliana Schons Gulart, Juliane Deise Fleck, Mariana Soares da Silva, Meriane Demoliner, Matheus Nunes Weber, Paula Rodrigues de Almeida, Michele Filippi.
EPI_ISL_1819072, EPI_ISL_1819073, EPI_ISL_1819074, EPI_ISL_1819075, EPI_ISL_1819076, EPI_ISL_1819077	Oman-NIC	Oman-National Influenza Center-Department of Microbiology and Immunology-SQUH	Samira Al-Maruqi,Fahad Zadjali, Amina Al Jardani, Khulood Al-Mammary, Hanan Al-kindi, Fatma BaAlawi, Hamida AL Barwani, Zeyana AL-Dahmani, Intisar Al-Shukri, Aisha Al-Busaidi, Aisha Al-Amri, Ahlam Al-Amri, Mohammed Al-Tobi, Samiha Al Kharusi, Abdulla Balkhair
EPI_ISL_1859361	Federal State Budgetary Educational Institution of Higher Education «Privolzhsky Research Medical University» of the Ministry of Health of the Russian Federation, Academic Consortium	FEDERAL RESEARCH AND CLINICAL CENTER OF PHYSICAL-CHEMICAL MEDICINE OF FEDERAL MEDICAL BIOLOGICAL AGENCY, Academic Consortium	Vladislav Babenko, Ramiz Bakhtyev, Vladimir Baklaushev, Larisa Balykova, Pavel Bashkirov, Anna Blagonravova, Daria Boldyreva, Dmitry Fedorov, Ilshat Gafurov, Raushaniya Gaifullina, Yulia Galeeva, Elena Galova, Vadim Govorun, Elena Ilina, Konstantin Ivanov, Daria Kharlampieva, Polina Khromova, Ksenia Klimina, Konstantin Kolontarev, Nadezhda Kolyshkina, Andrey Koritsky, Vyacheslav Kuropatkin, Vasily Lazarev, Alexander Manolov, Valentin Manuvera, Daria Matyushkina, Ekaterina Moskaleva, Varvara Musarova, Oleg Ogarkov, Elizaveta Orlova, Alexander Pavlenko, Alla Petrova, Natalia Pozhenko, Dmitry Pushkar, Alexander Rumyantsev, Sergey Rumyantsev, Vladimir Rumyantsev, Lyubov Rychkova, Alexander Samoilov, Irina Shirokova, Vyacheslav Sinkov, Svetlana Solovieva, Polina Tikhonova, Galina Trifonova, Alexander Troitsky, Alexander Tulichev, Yuri Udalov, Anna Varizhuk, Alexander Vasiliev, Vladimir Veselovsky, Alexey Volnukhin, Gaukhar Yusubalieva
EPI_ISL_1859397	FEDERAL STATE BUDGETARY INSTITUTION OF HEALTHCARE HOSPITAL OF THE RUSSIAN ACADEMY OF SCIENCES (TROITSK), Academic Consortium	FEDERAL RESEARCH AND CLINICAL CENTER OF PHYSICAL-CHEMICAL MEDICINE OF FEDERAL MEDICAL BIOLOGICAL AGENCY, Academic Consortium	Vladislav Babenko, Ramiz Bakhtyev, Vladimir Baklaushev, Larisa Balykova, Pavel Bashkirov, Anna Blagonravova, Daria Boldyreva, Dmitry Fedorov, Ilshat Gafurov, Raushaniya Gaifullina, Yulia Galeeva, Elena Galova, Vadim Govorun, Elena Ilina, Konstantin Ivanov, Daria Kharlampieva, Polina Khromova, Ksenia Klimina, Konstantin Kolontarev, Nadezhda Kolyshkina, Andrey Koritsky, Vyacheslav Kuropatkin, Vasily Lazarev, Alexander Manolov, Valentin Manuvera, Daria Matyushkina, Ekaterina Moskaleva, Varvara Musarova, Oleg Ogarkov, Elizaveta Orlova, Alexander Pavlenko, Alla Petrova, Natalia Pozhenko, Dmitry Pushkar, Alexander Rumyantsev, Sergey Rumyantsev, Vladimir Rumyantsev, Lyubov Rychkova, Alexander Samoilov, Irina Shirokova, Vyacheslav Sinkov, Svetlana Solovieva, Polina Tikhonova, Galina Trifonova, Alexander Troitsky, Alexander Tulichev, Yuri Udalov, Anna Varizhuk, Alexander Vasiliev, Vladimir Veselovsky, Alexey Volnukhin, Gaukhar Yusubalieva
EPI_ISL_1859412	Federal State Budgetary Educational Institution of Higher Education «Privolzhsky Research Medical University» of the Ministry of Health of the Russian Federation, Academic Consortium	FEDERAL RESEARCH AND CLINICAL CENTER OF PHYSICAL-CHEMICAL MEDICINE OF FEDERAL MEDICAL BIOLOGICAL AGENCY, Academic Consortium	Vladislav Babenko, Ramiz Bakhtyev, Vladimir Baklaushev, Larisa Balykova, Pavel Bashkirov, Anna Blagonravova, Daria Boldyreva, Dmitry Fedorov, Ilshat Gafurov, Raushaniya Gaifullina, Yulia Galeeva, Elena Galova, Vadim Govorun, Elena Ilina, Konstantin Ivanov, Daria Kharlampieva, Polina Khromova, Ksenia Klimina, Konstantin Kolontarev, Nadezhda Kolyshkina, Andrey Koritsky, Vyacheslav Kuropatkin, Vasily Lazarev, Alexander Manolov, Valentin Manuvera, Daria Matyushkina, Ekaterina Moskaleva, Varvara Musarova, Oleg Ogarkov, Elizaveta Orlova, Alexander Pavlenko, Alla Petrova, Natalia Pozhenko, Dmitry Pushkar, Alexander Rumyantsev, Sergey Rumyantsev, Vladimir Rumyantsev, Lyubov Rychkova, Alexander Samoilov, Irina Shirokova, Vyacheslav Sinkov, Svetlana Solovieva, Polina Tikhonova, Galina Trifonova, Alexander Troitsky, Alexander Tulichev, Yuri Udalov, Anna Varizhuk, Alexander Vasiliev, Vladimir Veselovsky, Alexey Volnukhin, Gaukhar Yusubalieva
EPI_ISL_1859425	Federal State Public Scientific Institution «Scientific entre for Family Health and Human Reproduction Problems», Academic Consortium	FEDERAL RESEARCH AND CLINICAL CENTER OF PHYSICAL-CHEMICAL MEDICINE OF FEDERAL MEDICAL BIOLOGICAL AGENCY, Academic Consortium	Vladislav Babenko, Ramiz Bakhtyev, Vladimir Baklaushev, Larisa Balykova, Pavel Bashkirov, Anna Blagonravova, Daria Boldyreva, Dmitry Fedorov, Ilshat Gafurov, Raushaniya Gaifullina, Yulia Galeeva, Elena Galova, Vadim Govorun, Elena Ilina, Konstantin Ivanov, Daria Kharlampieva, Polina Khromova, Ksenia Klimina, Konstantin Kolontarev, Nadezhda Kolyshkina, Andrey Koritsky, Vyacheslav Kuropatkin, Vasily Lazarev, Alexander Manolov, Valentin Manuvera, Daria Matyushkina, Ekaterina Moskaleva, Varvara Musarova, Oleg Ogarkov, Elizaveta Orlova, Alexander Pavlenko, Alla Petrova, Natalia Pozhenko, Dmitry Pushkar, Alexander Rumyantsev, Sergey Rumyantsev, Vladimir Rumyantsev, Lyubov Rychkova, Alexander Samoilov, Irina Shirokova, Vyacheslav Sinkov, Svetlana Solovieva, Polina Tikhonova, Galina Trifonova, Alexander Troitsky, Alexander Tulichev, Yuri Udalov, Anna Varizhuk, Alexander Vasiliev, Vladimir Veselovsky, Alexey Volnukhin, Gaukhar Yusubalieva
EPI_ISL_1859429	FEDERAL STATE BUDGETARY INSTITUTION OF HEALTHCARE HOSPITAL OF THE RUSSIAN ACADEMY OF SCIENCES (TROITSK), Academic Consortium	FEDERAL RESEARCH AND CLINICAL CENTER OF PHYSICAL-CHEMICAL MEDICINE OF FEDERAL MEDICAL BIOLOGICAL AGENCY, Academic Consortium	Vladislav Babenko, Ramiz Bakhtyev, Vladimir Baklaushev, Larisa Balykova, Pavel Bashkirov, Anna Blagonravova, Daria Boldyreva, Dmitry Fedorov, Ilshat Gafurov, Raushaniya Gaifullina, Yulia Galeeva, Elena Galova, Vadim Govorun, Elena Ilina, Konstantin Ivanov, Daria Kharlampieva, Polina Khromova, Ksenia Klimina, Konstantin Kolontarev, Nadezhda Kolyshkina, Andrey Koritsky, Vyacheslav Kuropatkin, Vasily Lazarev, Alexander Manolov, Valentin Manuvera, Daria Matyushkina, Ekaterina Moskaleva, Varvara Musarova, Oleg Ogarkov, Elizaveta Orlova, Alexander Pavlenko, Alla Petrova, Natalia Pozhenko, Dmitry Pushkar, Alexander Rumyantsev, Sergey Rumyantsev, Vladimir Rumyantsev, Lyubov Rychkova, Alexander Samoilov, Irina Shirokova, Vyacheslav Sinkov, Svetlana Solovieva, Polina Tikhonova, Galina Trifonova, Alexander Troitsky, Alexander Tulichev, Yuri Udalov, Anna Varizhuk, Alexander Vasiliev, Vladimir Veselovsky, Alexey Volnukhin, Gaukhar Yusubalieva
EPI_ISL_1859435	Federal State Public Scientific Institution «Scientific entre for Family Health and Human Reproduction Problems», Academic Consortium	FEDERAL RESEARCH AND CLINICAL CENTER OF PHYSICAL-CHEMICAL MEDICINE OF FEDERAL MEDICAL BIOLOGICAL AGENCY, Academic Consortium	Vladislav Babenko, Ramiz Bakhtyev, Vladimir Baklaushev, Larisa Balykova, Pavel Bashkirov, Anna Blagonravova, Daria Boldyreva, Dmitry Fedorov, Ilshat Gafurov, Raushaniya Gaifullina, Yulia Galeeva, Elena Galova, Vadim Govorun, Elena Ilina, Konstantin Ivanov, Daria Kharlampieva, Polina Khromova, Ksenia Klimina, Konstantin Kolontarev, Nadezhda Kolyshkina, Andrey Koritsky, Vyacheslav Kuropatkin, Vasily Lazarev, Alexander Manolov, Valentin Manuvera, Daria Matyushkina, Ekaterina Moskaleva, Varvara Musarova, Oleg Ogarkov, Elizaveta Orlova, Alexander Pavlenko, Alla Petrova, Natalia Pozhenko, Dmitry Pushkar, Alexander Rumyantsev, Sergey Rumyantsev, Vladimir Rumyantsev, Lyubov Rychkova, Alexander Samoilov, Irina Shirokova, Vyacheslav Sinkov, Svetlana Solovieva, Polina Tikhonova, Galina Trifonova, Alexander Troitsky, Alexander Tulichev, Yuri Udalov, Anna Varizhuk, Alexander Vasiliev, Vladimir Veselovsky, Alexey Volnukhin, Gaukhar Yusubalieva

EPI_ISL_1859447	City Clinical Hospital named after S.I. Spasokukotsky of Moscow Healthcare Department, Academic Consortium	FEDERAL RESEARCH AND CLINICAL CENTER OF PHYSICAL-CHEMICAL MEDICINE OF FEDERAL MEDICAL BIOLOGICAL AGENCY, Academic Consortium	Vladislav Babenko, Ramiz Bakhtyev, Vladimir Baklaushev, Larisa Balykova, Pavel Bashkirov, Anna Blagonravova, Daria Boldyreva, Dmitry Fedorov, Ilshat Gafurov, Raushaniya Gaifullina, Yulia Galeeva, Elena Galova, Vadim Govorun, Elena Ilina, Konstantin Ivanov, Daria Kharlampieva, Polina Khromova, Ksenia Klimina, Konstantin Kolontarev, Nadezhda Kolyshkina, Andrey Koritsky, Vyacheslav Kuropatkin, Vasily Lazarev, Alexander Manolov, Valentin Manuvera, Daria Matyushkina, Ekaterina Moskaleva, Varvara Musarova, Oleg Ogarkov, Elizaveta Orlova, Alexander Pavlenko, Alla Petrova, Natalia Pozhenko, Dmitry Pushkar, Alexander Rumyantsev, Sergey Rumyantsev, Vladimir Rumyantsev, Lyubov Rychkova, Alexander Samoilov, Irina Shirokova, Vyacheslav Sinkov, Svetlana Solovieva, Polina Tikhonova, Galina Trifonova, Alexander Troitsky, Alexander Tulichev, Yuri Udalov, Anna Varizhuk, Alexander Vasiliev, Vladimir Veselovsky, Alexey Volnukhin, Gaukhar Yusubalieva
EPI_ISL_1859449	Federal State Budgetary Educational Institution of Higher Education «Privolzhsky Research Medical University» of the Ministry of Health of the Russian Federation, Academic Consortium	FEDERAL RESEARCH AND CLINICAL CENTER OF PHYSICAL-CHEMICAL MEDICINE OF FEDERAL MEDICAL BIOLOGICAL AGENCY, Academic Consortium	Vladislav Babenko, Ramiz Bakhtyev, Vladimir Baklaushev, Larisa Balykova, Pavel Bashkirov, Anna Blagonravova, Daria Boldyreva, Dmitry Fedorov, Ilshat Gafurov, Raushaniya Gaifullina, Yulia Galeeva, Elena Galova, Vadim Govorun, Elena Ilina, Konstantin Ivanov, Daria Kharlampieva, Polina Khromova, Ksenia Klimina, Konstantin Kolontarev, Nadezhda Kolyshkina, Andrey Koritsky, Vyacheslav Kuropatkin, Vasily Lazarev, Alexander Manolov, Valentin Manuvera, Daria Matyushkina, Ekaterina Moskaleva, Varvara Musarova, Oleg Ogarkov, Elizaveta Orlova, Alexander Pavlenko, Alla Petrova, Natalia Pozhenko, Dmitry Pushkar, Alexander Rumyantsev, Sergey Rumyantsev, Vladimir Rumyantsev, Lyubov Rychkova, Alexander Samoilov, Irina Shirokova, Vyacheslav Sinkov, Svetlana Solovieva, Polina Tikhonova, Galina Trifonova, Alexander Troitsky, Alexander Tulichev, Yuri Udalov, Anna Varizhuk, Alexander Vasiliev, Vladimir Veselovsky, Alexey Volnukhin, Gaukhar Yusubalieva
EPI_ISL_1859456, EPI_ISL_1859488, EPI_ISL_1859492, EPI_ISL_1859500	Federal State Public Scientific Institution «Scientific entre for Family Health and Human Reproduction Problems», Academic Consortium	FEDERAL RESEARCH AND CLINICAL CENTER OF PHYSICAL-CHEMICAL MEDICINE OF FEDERAL MEDICAL BIOLOGICAL AGENCY, Academic Consortium	Vladislav Babenko, Ramiz Bakhtyev, Vladimir Baklaushev, Larisa Balykova, Pavel Bashkirov, Anna Blagonravova, Daria Boldyreva, Dmitry Fedorov, Ilshat Gafurov, Raushaniya Gaifullina, Yulia Galeeva, Elena Galova, Vadim Govorun, Elena Ilina, Konstantin Ivanov, Daria Kharlampieva, Polina Khromova, Ksenia Klimina, Konstantin Kolontarev, Nadezhda Kolyshkina, Andrey Koritsky, Vyacheslav Kuropatkin, Vasily Lazarev, Alexander Manolov, Valentin Manuvera, Daria Matyushkina, Ekaterina Moskaleva, Varvara Musarova, Oleg Ogarkov, Elizaveta Orlova, Alexander Pavlenko, Alla Petrova, Natalia Pozhenko, Dmitry Pushkar, Alexander Rumyantsev, Sergey Rumyantsev, Vladimir Rumyantsev, Lyubov Rychkova, Alexander Samoilov, Irina Shirokova, Vyacheslav Sinkov, Svetlana Solovieva, Polina Tikhonova, Galina Trifonova, Alexander Troitsky, Alexander Tulichev, Yuri Udalov, Anna Varizhuk, Alexander Vasiliev, Vladimir Veselovsky, Alexey Volnukhin, Gaukhar Yusubalieva
EPI_ISL_1859505	Federal State Budgetary Educational Institution of Higher Education «Privolzhsky Research Medical University» of the Ministry of Health of the Russian Federation, Academic Consortium	FEDERAL RESEARCH AND CLINICAL CENTER OF PHYSICAL-CHEMICAL MEDICINE OF FEDERAL MEDICAL BIOLOGICAL AGENCY, Academic Consortium	Vladislav Babenko, Ramiz Bakhtyev, Vladimir Baklaushev, Larisa Balykova, Pavel Bashkirov, Anna Blagonravova, Daria Boldyreva, Dmitry Fedorov, Ilshat Gafurov, Raushaniya Gaifullina, Yulia Galeeva, Elena Galova, Vadim Govorun, Elena Ilina, Konstantin Ivanov, Daria Kharlampieva, Polina Khromova, Ksenia Klimina, Konstantin Kolontarev, Nadezhda Kolyshkina, Andrey Koritsky, Vyacheslav Kuropatkin, Vasily Lazarev, Alexander Manolov, Valentin Manuvera, Daria Matyushkina, Ekaterina Moskaleva, Varvara Musarova, Oleg Ogarkov, Elizaveta Orlova, Alexander Pavlenko, Alla Petrova, Natalia Pozhenko, Dmitry Pushkar, Alexander Rumyantsev, Sergey Rumyantsev, Vladimir Rumyantsev, Lyubov Rychkova, Alexander Samoilov, Irina Shirokova, Vyacheslav Sinkov, Svetlana Solovieva, Polina Tikhonova, Galina Trifonova, Alexander Troitsky, Alexander Tulichev, Yuri Udalov, Anna Varizhuk, Alexander Vasiliev, Vladimir Veselovsky, Alexey Volnukhin, Gaukhar Yusubalieva
EPI_ISL_1859507	Federal Research and Clinical Center of Specialized Medical Care and Medical Technologies, Federal Biomedical Agency of the Russian Federation, Academic Consortium	FEDERAL RESEARCH AND CLINICAL CENTER OF PHYSICAL-CHEMICAL MEDICINE OF FEDERAL MEDICAL BIOLOGICAL AGENCY, Academic Consortium	Vladislav Babenko, Ramiz Bakhtyev, Vladimir Baklaushev, Larisa Balykova, Pavel Bashkirov, Anna Blagonravova, Daria Boldyreva, Dmitry Fedorov, Ilshat Gafurov, Raushaniya Gaifullina, Yulia Galeeva, Elena Galova, Vadim Govorun, Elena Ilina, Konstantin Ivanov, Daria Kharlampieva, Polina Khromova, Ksenia Klimina, Konstantin Kolontarev, Nadezhda Kolyshkina, Andrey Koritsky, Vyacheslav Kuropatkin, Vasily Lazarev, Alexander Manolov, Valentin Manuvera, Daria Matyushkina, Ekaterina Moskaleva, Varvara Musarova, Oleg Ogarkov, Elizaveta Orlova, Alexander Pavlenko, Alla Petrova, Natalia Pozhenko, Dmitry Pushkar, Alexander Rumyantsev, Sergey Rumyantsev, Vladimir Rumyantsev, Lyubov Rychkova, Alexander Samoilov, Irina Shirokova, Vyacheslav Sinkov, Svetlana Solovieva, Polina Tikhonova, Galina Trifonova, Alexander Troitsky, Alexander Tulichev, Yuri Udalov, Anna Varizhuk, Alexander Vasiliev, Vladimir Veselovsky, Alexey Volnukhin, Gaukhar Yusubalieva
EPI_ISL_1859537	City Clinical Hospital named after S.I. Spasokukotsky of Moscow Healthcare Department, Academic Consortium	FEDERAL RESEARCH AND CLINICAL CENTER OF PHYSICAL-CHEMICAL MEDICINE OF FEDERAL MEDICAL BIOLOGICAL AGENCY, Academic Consortium	Vladislav Babenko, Ramiz Bakhtyev, Vladimir Baklaushev, Larisa Balykova, Pavel Bashkirov, Anna Blagonravova, Daria Boldyreva, Dmitry Fedorov, Ilshat Gafurov, Raushaniya Gaifullina, Yulia Galeeva, Elena Galova, Vadim Govorun, Elena Ilina, Konstantin Ivanov, Daria Kharlampieva, Polina Khromova, Ksenia Klimina, Konstantin Kolontarev, Nadezhda Kolyshkina, Andrey Koritsky, Vyacheslav Kuropatkin, Vasily Lazarev, Alexander Manolov, Valentin Manuvera, Daria Matyushkina, Ekaterina Moskaleva, Varvara Musarova, Oleg Ogarkov, Elizaveta Orlova, Alexander Pavlenko, Alla Petrova, Natalia Pozhenko, Dmitry Pushkar, Alexander Rumyantsev, Sergey Rumyantsev, Vladimir Rumyantsev, Lyubov Rychkova, Alexander Samoilov, Irina Shirokova, Vyacheslav Sinkov, Svetlana Solovieva, Polina Tikhonova, Galina Trifonova, Alexander Troitsky, Alexander Tulichev, Yuri Udalov, Anna Varizhuk, Alexander Vasiliev, Vladimir Veselovsky, Alexey Volnukhin, Gaukhar Yusubalieva
EPI_ISL_1859553	Federal Research and Clinical Center of Specialized Medical Care and Medical Technologies, Federal Biomedical Agency of the Russian Federation, Academic Consortium	FEDERAL RESEARCH AND CLINICAL CENTER OF PHYSICAL-CHEMICAL MEDICINE OF FEDERAL MEDICAL BIOLOGICAL AGENCY, Academic Consortium	Vladislav Babenko, Ramiz Bakhtyev, Vladimir Baklaushev, Larisa Balykova, Pavel Bashkirov, Anna Blagonravova, Daria Boldyreva, Dmitry Fedorov, Ilshat Gafurov, Raushaniya Gaifullina, Yulia Galeeva, Elena Galova, Vadim Govorun, Elena Ilina, Konstantin Ivanov, Daria Kharlampieva, Polina Khromova, Ksenia Klimina, Konstantin Kolontarev, Nadezhda Kolyshkina, Andrey Koritsky, Vyacheslav Kuropatkin, Vasily Lazarev, Alexander Manolov, Valentin Manuvera, Daria Matyushkina, Ekaterina Moskaleva, Varvara Musarova, Oleg Ogarkov, Elizaveta Orlova, Alexander Pavlenko, Alla Petrova, Natalia Pozhenko, Dmitry Pushkar, Alexander Rumyantsev, Sergey Rumyantsev, Vladimir Rumyantsev, Lyubov Rychkova, Alexander Samoilov, Irina Shirokova, Vyacheslav Sinkov, Svetlana Solovieva, Polina Tikhonova, Galina Trifonova, Alexander Troitsky, Alexander Tulichev, Yuri Udalov, Anna Varizhuk, Alexander Vasiliev, Vladimir Veselovsky, Alexey Volnukhin, Gaukhar Yusubalieva
EPI_ISL_1859564, EPI_ISL_1859566	Federal State Public Scientific Institution «Scientific entre for Family Health and Human Reproduction Problems», Academic Consortium	FEDERAL RESEARCH AND CLINICAL CENTER OF PHYSICAL-CHEMICAL MEDICINE OF FEDERAL MEDICAL BIOLOGICAL AGENCY, Academic Consortium	Vladislav Babenko, Ramiz Bakhtyev, Vladimir Baklaushev, Larisa Balykova, Pavel Bashkirov, Anna Blagonravova, Daria Boldyreva, Dmitry Fedorov, Ilshat Gafurov, Raushaniya Gaifullina, Yulia Galeeva, Elena Galova, Vadim Govorun, Elena Ilina, Konstantin Ivanov, Daria Kharlampieva, Polina Khromova, Ksenia Klimina, Konstantin Kolontarev, Nadezhda Kolyshkina, Andrey Koritsky, Vyacheslav Kuropatkin, Vasily Lazarev, Alexander Manolov, Valentin Manuvera, Daria Matyushkina, Ekaterina Moskaleva, Varvara Musarova, Oleg Ogarkov, Elizaveta Orlova, Alexander Pavlenko, Alla Petrova, Natalia Pozhenko, Dmitry Pushkar, Alexander Rumyantsev, Sergey Rumyantsev, Vladimir Rumyantsev, Lyubov Rychkova, Alexander Samoilov, Irina Shirokova, Vyacheslav Sinkov, Svetlana Solovieva, Polina Tikhonova, Galina Trifonova, Alexander Troitsky, Alexander Tulichev, Yuri Udalov, Anna Varizhuk, Alexander Vasiliev, Vladimir Veselovsky, Alexey Volnukhin, Gaukhar Yusubalieva
EPI_ISL_1859581	City Clinical Hospital named after S.I. Spasokukotsky of Moscow Healthcare Department, Academic Consortium	FEDERAL RESEARCH AND CLINICAL CENTER OF PHYSICAL-CHEMICAL MEDICINE OF FEDERAL MEDICAL BIOLOGICAL AGENCY, Academic Consortium	Vladislav Babenko, Ramiz Bakhtyev, Vladimir Baklaushev, Larisa Balykova, Pavel Bashkirov, Anna Blagonravova, Daria Boldyreva, Dmitry Fedorov, Ilshat Gafurov, Raushaniya Gaifullina, Yulia Galeeva, Elena Galova, Vadim Govorun, Elena Ilina, Konstantin Ivanov, Daria Kharlampieva, Polina Khromova, Ksenia Klimina, Konstantin Kolontarev, Nadezhda Kolyshkina, Andrey Koritsky, Vyacheslav Kuropatkin, Vasily Lazarev, Alexander Manolov, Valentin Manuvera, Daria Matyushkina, Ekaterina Moskaleva, Varvara Musarova, Oleg Ogarkov, Elizaveta Orlova, Alexander Pavlenko, Alla Petrova, Natalia Pozhenko, Dmitry Pushkar, Alexander Rumyantsev, Sergey Rumyantsev, Vladimir Rumyantsev, Lyubov Rychkova, Alexander Samoilov, Irina Shirokova, Vyacheslav Sinkov, Svetlana Solovieva, Polina Tikhonova, Galina Trifonova, Alexander Troitsky, Alexander Tulichev, Yuri Udalov, Anna Varizhuk, Alexander Vasiliev, Vladimir Veselovsky, Alexey Volnukhin, Gaukhar Yusubalieva
EPI_ISL_1859586, EPI_ISL_1859592	FEDERAL STATE BUDGETARY INSTITUTION OF HEALTHCARE HOSPITAL OF THE RUSSIAN ACADEMY OF SCIENCES (TROITSK), Academic Consortium	FEDERAL RESEARCH AND CLINICAL CENTER OF PHYSICAL-CHEMICAL MEDICINE OF FEDERAL MEDICAL BIOLOGICAL AGENCY, Academic Consortium	Vladislav Babenko, Ramiz Bakhtyev, Vladimir Baklaushev, Larisa Balykova, Pavel Bashkirov, Anna Blagonravova, Daria Boldyreva, Dmitry Fedorov, Ilshat Gafurov, Raushaniya Gaifullina, Yulia Galeeva, Elena Galova, Vadim Govorun, Elena Ilina, Konstantin Ivanov, Daria Kharlampieva, Polina Khromova, Ksenia Klimina, Konstantin Kolontarev, Nadezhda Kolyshkina, Andrey Koritsky, Vyacheslav Kuropatkin, Vasily Lazarev, Alexander Manolov, Valentin Manuvera, Daria Matyushkina, Ekaterina Moskaleva, Varvara Musarova, Oleg Ogarkov, Elizaveta Orlova, Alexander Pavlenko, Alla Petrova, Natalia Pozhenko, Dmitry Pushkar, Alexander Rumyantsev, Sergey Rumyantsev, Vladimir Rumyantsev, Lyubov Rychkova, Alexander Samoilov, Irina Shirokova, Vyacheslav Sinkov, Svetlana Solovieva, Polina Tikhonova, Galina Trifonova, Alexander Troitsky, Alexander Tulichev, Yuri Udalov, Anna Varizhuk, Alexander Vasiliev, Vladimir Veselovsky, Alexey Volnukhin, Gaukhar Yusubalieva
EPI_ISL_1859609	Federal State Budgetary Educational Institution of Higher Education «Privolzhsky Research Medical University» of the Ministry of Health of the Russian Federation, Academic Consortium	FEDERAL RESEARCH AND CLINICAL CENTER OF PHYSICAL-CHEMICAL MEDICINE OF FEDERAL MEDICAL BIOLOGICAL AGENCY, Academic Consortium	Vladislav Babenko, Ramiz Bakhtyev, Vladimir Baklaushev, Larisa Balykova, Pavel Bashkirov, Anna Blagonravova, Daria Boldyreva, Dmitry Fedorov, Ilshat Gafurov, Raushaniya Gaifullina, Yulia Galeeva, Elena Galova, Vadim Govorun, Elena Ilina, Konstantin Ivanov, Daria Kharlampieva, Polina Khromova, Ksenia Klimina, Konstantin Kolontarev, Nadezhda Kolyshkina, Andrey Koritsky, Vyacheslav Kuropatkin, Vasily Lazarev, Alexander Manolov, Valentin Manuvera, Daria Matyushkina, Ekaterina Moskaleva, Varvara Musarova, Oleg Ogarkov, Elizaveta Orlova, Alexander Pavlenko, Alla Petrova, Natalia Pozhenko, Dmitry Pushkar, Alexander Rumyantsev, Sergey Rumyantsev, Vladimir Rumyantsev, Lyubov Rychkova, Alexander Samoilov, Irina Shirokova, Vyacheslav Sinkov, Svetlana Solovieva, Polina Tikhonova, Galina Trifonova, Alexander Troitsky, Alexander Tulichev, Yuri Udalov, Anna Varizhuk, Alexander Vasiliev, Vladimir Veselovsky, Alexey Volnukhin, Gaukhar Yusubalieva
EPI_ISL_1859615, EPI_ISL_1859617	City Clinical Hospital named after S.I. Spasokukotsky of Moscow Healthcare Department, Academic Consortium	FEDERAL RESEARCH AND CLINICAL CENTER OF PHYSICAL-CHEMICAL MEDICINE OF FEDERAL MEDICAL BIOLOGICAL AGENCY, Academic Consortium	Vladislav Babenko, Ramiz Bakhtyev, Vladimir Baklaushev, Larisa Balykova, Pavel Bashkirov, Anna Blagonravova, Daria Boldyreva, Dmitry Fedorov, Ilshat Gafurov, Raushaniya Gaifullina, Yulia Galeeva, Elena Galova, Vadim Govorun, Elena Ilina, Konstantin Ivanov, Daria Kharlampieva, Polina Khromova, Ksenia Klimina, Konstantin Kolontarev, Nadezhda Kolyshkina, Andrey Koritsky, Vyacheslav Kuropatkin, Vasily Lazarev, Alexander Manolov, Valentin Manuvera, Daria Matyushkina, Ekaterina Moskaleva, Varvara Musarova, Oleg Ogarkov, Elizaveta Orlova, Alexander Pavlenko, Alla Petrova, Natalia Pozhenko, Dmitry Pushkar, Alexander Rumyantsev, Sergey Rumyantsev, Vladimir Rumyantsev, Lyubov Rychkova, Alexander Samoilov, Irina Shirokova, Vyacheslav Sinkov,

			Svetlana Solovieva, Polina Tikhonova, Galina Trifonova, Alexander Troitsky, Alexander Tulichev, Yuri Udalov, Anna Varizhuk, Alexander Vasiliev, Vladimir Veselovsky, Alexey Volnukhin, Gaukhar Yusubalieva
EPI_ISL_1859619	Federal State Public Scientific Institution «Scientific entre for Family Health and Human Reproduction Problems», Academic Consortium	FEDERAL RESEARCH AND CLINICAL CENTER OF PHYSICAL-CHEMICAL MEDICINE OF FEDERAL MEDICAL BIOLOGICAL AGENCY, Academic Consortium	Vladislav Babenko, Ramiz Bakhtyev, Vladimir Baklaushev, Larisa Balykova, Pavel Bashkirov, Anna Blagonravova, Daria Boldyreva, Dmitry Fedorov, Ilshat Gafurov, Raushaniya Gaifullina, Yulia Galeeva, Elena Galova, Vadim Govorun, Elena Ilina, Konstantin Ivanov, Daria Kharlampieva, Polina Khromova, Ksenia Klimina, Konstantin Kolontarev, Nadezhda Kolyshkina, Andrey Koritsky, Vyacheslav Kuropatkin, Vasily Lazarev, Alexander Manolov, Valentin Manuvera, Daria Matyushkina, Ekaterina Moskaleva, Varvara Musarova, Oleg Ogarkov, Elizaveta Orlova, Alexander Pavlenko, Alla Petrova, Natalia Pozhenko, Dmitry Pushkar, Alexander Rumyantsev, Sergey Rumyantsev, Vladimir Rumyantsev, Lyubov Rychkova, Alexander Samoilov, Irina Shirokova, Vyacheslav Sinkov, Svetlana Solovieva, Polina Tikhonova, Galina Trifonova, Alexander Troitsky, Alexander Tulichev, Yuri Udalov, Anna Varizhuk, Alexander Vasiliev, Vladimir Veselovsky, Alexey Volnukhin, Gaukhar Yusubalieva
EPI_ISL_1859622	City Clinical Hospital named after S.I. Spasokukotsky of Moscow Healthcare Department, Academic Consortium	FEDERAL RESEARCH AND CLINICAL CENTER OF PHYSICAL-CHEMICAL MEDICINE OF FEDERAL MEDICAL BIOLOGICAL AGENCY, Academic Consortium	Vladislav Babenko, Ramiz Bakhtyev, Vladimir Baklaushev, Larisa Balykova, Pavel Bashkirov, Anna Blagonravova, Daria Boldyreva, Dmitry Fedorov, Ilshat Gafurov, Raushaniya Gaifullina, Yulia Galeeva, Elena Galova, Vadim Govorun, Elena Ilina, Konstantin Ivanov, Daria Kharlampieva, Polina Khromova, Ksenia Klimina, Konstantin Kolontarev, Nadezhda Kolyshkina, Andrey Koritsky, Vyacheslav Kuropatkin, Vasily Lazarev, Alexander Manolov, Valentin Manuvera, Daria Matyushkina, Ekaterina Moskaleva, Varvara Musarova, Oleg Ogarkov, Elizaveta Orlova, Alexander Pavlenko, Alla Petrova, Natalia Pozhenko, Dmitry Pushkar, Alexander Rumyantsev, Sergey Rumyantsev, Vladimir Rumyantsev, Lyubov Rychkova, Alexander Samoilov, Irina Shirokova, Vyacheslav Sinkov, Svetlana Solovieva, Polina Tikhonova, Galina Trifonova, Alexander Troitsky, Alexander Tulichev, Yuri Udalov, Anna Varizhuk, Alexander Vasiliev, Vladimir Veselovsky, Alexey Volnukhin, Gaukhar Yusubalieva
EPI_ISL_1859624, EPI_ISL_1859632	Federal State Public Scientific Institution «Scientific entre for Family Health and Human Reproduction Problems», Academic Consortium	FEDERAL RESEARCH AND CLINICAL CENTER OF PHYSICAL-CHEMICAL MEDICINE OF FEDERAL MEDICAL BIOLOGICAL AGENCY, Academic Consortium	Vladislav Babenko, Ramiz Bakhtyev, Vladimir Baklaushev, Larisa Balykova, Pavel Bashkirov, Anna Blagonravova, Daria Boldyreva, Dmitry Fedorov, Ilshat Gafurov, Raushaniya Gaifullina, Yulia Galeeva, Elena Galova, Vadim Govorun, Elena Ilina, Konstantin Ivanov, Daria Kharlampieva, Polina Khromova, Ksenia Klimina, Konstantin Kolontarev, Nadezhda Kolyshkina, Andrey Koritsky, Vyacheslav Kuropatkin, Vasily Lazarev, Alexander Manolov, Valentin Manuvera, Daria Matyushkina, Ekaterina Moskaleva, Varvara Musarova, Oleg Ogarkov, Elizaveta Orlova, Alexander Pavlenko, Alla Petrova, Natalia Pozhenko, Dmitry Pushkar, Alexander Rumyantsev, Sergey Rumyantsev, Vladimir Rumyantsev, Lyubov Rychkova, Alexander Samoilov, Irina Shirokova, Vyacheslav Sinkov, Svetlana Solovieva, Polina Tikhonova, Galina Trifonova, Alexander Troitsky, Alexander Tulichev, Yuri Udalov, Anna Varizhuk, Alexander Vasiliev, Vladimir Veselovsky, Alexey Volnukhin, Gaukhar Yusubalieva
EPI_ISL_1859635	Federal Research and Clinical Center of Specialized Medical Care and Medical Technologies, Federal Biomedical Agency of the Russian Federation, Academic Consortium	FEDERAL RESEARCH AND CLINICAL CENTER OF PHYSICAL-CHEMICAL MEDICINE OF FEDERAL MEDICAL BIOLOGICAL AGENCY, Academic Consortium	Vladislav Babenko, Ramiz Bakhtyev, Vladimir Baklaushev, Larisa Balykova, Pavel Bashkirov, Anna Blagonravova, Daria Boldyreva, Dmitry Fedorov, Ilshat Gafurov, Raushaniya Gaifullina, Yulia Galeeva, Elena Galova, Vadim Govorun, Elena Ilina, Konstantin Ivanov, Daria Kharlampieva, Polina Khromova, Ksenia Klimina, Konstantin Kolontarev, Nadezhda Kolyshkina, Andrey Koritsky, Vyacheslav Kuropatkin, Vasily Lazarev, Alexander Manolov, Valentin Manuvera, Daria Matyushkina, Ekaterina Moskaleva, Varvara Musarova, Oleg Ogarkov, Elizaveta Orlova, Alexander Pavlenko, Alla Petrova, Natalia Pozhenko, Dmitry Pushkar, Alexander Rumyantsev, Sergey Rumyantsev, Vladimir Rumyantsev, Lyubov Rychkova, Alexander Samoilov, Irina Shirokova, Vyacheslav Sinkov, Svetlana Solovieva, Polina Tikhonova, Galina Trifonova, Alexander Troitsky, Alexander Tulichev, Yuri Udalov, Anna Varizhuk, Alexander Vasiliev, Vladimir Veselovsky, Alexey Volnukhin, Gaukhar Yusubalieva
EPI_ISL_1859645	FEDERAL STATE BUDGETARY INSTITUTION OF HEALTHCARE HOSPITAL OF THE RUSSIAN ACADEMY OF SCIENCES (TROITSK), Academic Consortium	FEDERAL RESEARCH AND CLINICAL CENTER OF PHYSICAL-CHEMICAL MEDICINE OF FEDERAL MEDICAL BIOLOGICAL AGENCY, Academic Consortium	Vladislav Babenko, Ramiz Bakhtyev, Vladimir Baklaushev, Larisa Balykova, Pavel Bashkirov, Anna Blagonravova, Daria Boldyreva, Dmitry Fedorov, Ilshat Gafurov, Raushaniya Gaifullina, Yulia Galeeva, Elena Galova, Vadim Govorun, Elena Ilina, Konstantin Ivanov, Daria Kharlampieva, Polina Khromova, Ksenia Klimina, Konstantin Kolontarev, Nadezhda Kolyshkina, Andrey Koritsky, Vyacheslav Kuropatkin, Vasily Lazarev, Alexander Manolov, Valentin Manuvera, Daria Matyushkina, Ekaterina Moskaleva, Varvara Musarova, Oleg Ogarkov, Elizaveta Orlova, Alexander Pavlenko, Alla Petrova, Natalia Pozhenko, Dmitry Pushkar, Alexander Rumyantsev, Sergey Rumyantsev, Vladimir Rumyantsev, Lyubov Rychkova, Alexander Samoilov, Irina Shirokova, Vyacheslav Sinkov, Svetlana Solovieva, Polina Tikhonova, Galina Trifonova, Alexander Troitsky, Alexander Tulichev, Yuri Udalov, Anna Varizhuk, Alexander Vasiliev, Vladimir Veselovsky, Alexey Volnukhin, Gaukhar Yusubalieva
EPI_ISL_1859663	Federal State Public Scientific Institution «Scientific entre for Family Health and Human Reproduction Problems», Academic Consortium	FEDERAL RESEARCH AND CLINICAL CENTER OF PHYSICAL-CHEMICAL MEDICINE OF FEDERAL MEDICAL BIOLOGICAL AGENCY, Academic Consortium	Vladislav Babenko, Ramiz Bakhtyev, Vladimir Baklaushev, Larisa Balykova, Pavel Bashkirov, Anna Blagonravova, Daria Boldyreva, Dmitry Fedorov, Ilshat Gafurov, Raushaniya Gaifullina, Yulia Galeeva, Elena Galova, Vadim Govorun, Elena Ilina, Konstantin Ivanov, Daria Kharlampieva, Polina Khromova, Ksenia Klimina, Konstantin Kolontarev, Nadezhda Kolyshkina, Andrey Koritsky, Vyacheslav Kuropatkin, Vasily Lazarev, Alexander Manolov, Valentin Manuvera, Daria Matyushkina, Ekaterina Moskaleva, Varvara Musarova, Oleg Ogarkov, Elizaveta Orlova, Alexander Pavlenko, Alla Petrova, Natalia Pozhenko, Dmitry Pushkar, Alexander Rumyantsev, Sergey Rumyantsev, Vladimir Rumyantsev, Lyubov Rychkova, Alexander Samoilov, Irina Shirokova, Vyacheslav Sinkov, Svetlana Solovieva, Polina Tikhonova, Galina Trifonova, Alexander Troitsky, Alexander Tulichev, Yuri Udalov, Anna Varizhuk, Alexander Vasiliev, Vladimir Veselovsky, Alexey Volnukhin, Gaukhar Yusubalieva
EPI_ISL_1859669	Federal State Budgetary Educational Institution of Higher Education «Privolzhsky Research Medical University» of the Ministry of Health of the Russian Federation, Academic Consortium	FEDERAL RESEARCH AND CLINICAL CENTER OF PHYSICAL-CHEMICAL MEDICINE OF FEDERAL MEDICAL BIOLOGICAL AGENCY, Academic Consortium	Vladislav Babenko, Ramiz Bakhtyev, Vladimir Baklaushev, Larisa Balykova, Pavel Bashkirov, Anna Blagonravova, Daria Boldyreva, Dmitry Fedorov, Ilshat Gafurov, Raushaniya Gaifullina, Yulia Galeeva, Elena Galova, Vadim Govorun, Elena Ilina, Konstantin Ivanov, Daria Kharlampieva, Polina Khromova, Ksenia Klimina, Konstantin Kolontarev, Nadezhda Kolyshkina, Andrey Koritsky, Vyacheslav Kuropatkin, Vasily Lazarev, Alexander Manolov, Valentin Manuvera, Daria Matyushkina, Ekaterina Moskaleva, Varvara Musarova, Oleg Ogarkov, Elizaveta Orlova, Alexander Pavlenko, Alla Petrova, Natalia Pozhenko, Dmitry Pushkar, Alexander Rumyantsev, Sergey Rumyantsev, Vladimir Rumyantsev, Lyubov Rychkova, Alexander Samoilov, Irina Shirokova, Vyacheslav Sinkov, Svetlana Solovieva, Polina Tikhonova, Galina Trifonova, Alexander Troitsky, Alexander Tulichev, Yuri Udalov, Anna Varizhuk, Alexander Vasiliev, Vladimir Veselovsky, Alexey Volnukhin, Gaukhar Yusubalieva
EPI_ISL_1859688, EPI_ISL_1859720, EPI_ISL_1859742	Federal State Public Scientific Institution «Scientific entre for Family Health and Human Reproduction Problems», Academic Consortium	FEDERAL RESEARCH AND CLINICAL CENTER OF PHYSICAL-CHEMICAL MEDICINE OF FEDERAL MEDICAL BIOLOGICAL AGENCY, Academic Consortium	Vladislav Babenko, Ramiz Bakhtyev, Vladimir Baklaushev, Larisa Balykova, Pavel Bashkirov, Anna Blagonravova, Daria Boldyreva, Dmitry Fedorov, Ilshat Gafurov, Raushaniya Gaifullina, Yulia Galeeva, Elena Galova, Vadim Govorun, Elena Ilina, Konstantin Ivanov, Daria Kharlampieva, Polina Khromova, Ksenia Klimina, Konstantin Kolontarev, Nadezhda Kolyshkina, Andrey Koritsky, Vyacheslav Kuropatkin, Vasily Lazarev, Alexander Manolov, Valentin Manuvera, Daria Matyushkina, Ekaterina Moskaleva, Varvara Musarova, Oleg Ogarkov, Elizaveta Orlova, Alexander Pavlenko, Alla Petrova, Natalia Pozhenko, Dmitry Pushkar, Alexander Rumyantsev, Sergey Rumyantsev, Vladimir Rumyantsev, Lyubov Rychkova, Alexander Samoilov, Irina Shirokova, Vyacheslav Sinkov, Svetlana Solovieva, Polina Tikhonova, Galina Trifonova, Alexander Troitsky, Alexander Tulichev, Yuri Udalov, Anna Varizhuk, Alexander Vasiliev, Vladimir Veselovsky, Alexey Volnukhin, Gaukhar Yusubalieva
EPI_ISL_1859762	Federal State Budgetary Educational Institution of Higher Education «Privolzhsky Research Medical University» of the Ministry of Health of the Russian Federation, Academic Consortium	FEDERAL RESEARCH AND CLINICAL CENTER OF PHYSICAL-CHEMICAL MEDICINE OF FEDERAL MEDICAL BIOLOGICAL AGENCY, Academic Consortium	Vladislav Babenko, Ramiz Bakhtyev, Vladimir Baklaushev, Larisa Balykova, Pavel Bashkirov, Anna Blagonravova, Daria Boldyreva, Dmitry Fedorov, Ilshat Gafurov, Raushaniya Gaifullina, Yulia Galeeva, Elena Galova, Vadim Govorun, Elena Ilina, Konstantin Ivanov, Daria Kharlampieva, Polina Khromova, Ksenia Klimina, Konstantin Kolontarev, Nadezhda Kolyshkina, Andrey Koritsky, Vyacheslav Kuropatkin, Vasily Lazarev, Alexander Manolov, Valentin Manuvera, Daria Matyushkina, Ekaterina Moskaleva, Varvara Musarova, Oleg Ogarkov, Elizaveta Orlova, Alexander Pavlenko, Alla Petrova, Natalia Pozhenko, Dmitry Pushkar, Alexander Rumyantsev, Sergey Rumyantsev, Vladimir Rumyantsev, Lyubov Rychkova, Alexander Samoilov, Irina Shirokova, Vyacheslav Sinkov, Svetlana Solovieva, Polina Tikhonova, Galina Trifonova, Alexander Troitsky, Alexander Tulichev, Yuri Udalov, Anna Varizhuk, Alexander Vasiliev, Vladimir Veselovsky, Alexey Volnukhin, Gaukhar Yusubalieva
EPI_ISL_1910397, EPI_ISL_1910398	Centre Hospitalier Universitaire Clermont-Ferrand	CHU Clermont-Ferrand, service de virologie	Bisseux Maxime, Mirand Audrey, Combes Patricia, Henquell Cécile
EPI_ISL_1972894	National Public Health Laboratory Malaysia	National Public Health Laboratory Malaysia	Noorliza MN, JL Tan, Chong CK, Norhayati R, Yukie C, Norazimah T Rehan SAB Selvanesan S, Hannah PYP, NurulAina MCA, W.NurAfiza WMA Zirwatul AA, Hani MH, Nurulsyahida I, Aziyati O, Ushananthiny R, Kamal HKZ, Mohd AY, YF Ngeow
EPI_ISL_2000568, EPI_ISL_2000569	Tropical Medicine Institute Pedro Kouri	Respiratory Infections Laboratory	Yahisel Tejero Suarez, Lissette Perez Santos, Yanet Pintos Saavedra, Daylin Mederos, Odalys Valdes, Mirtha Elisa Aguado, Vivian Kouri Cardella
EPI_ISL_2001481, EPI_ISL_2001482	National Public Health Laboratory Malaysia	National Public Health Laboratory Malaysia	Noorliza MN, JL Tan, Chong CK, Norhayati R, Yukie C, Norazimah T Rehan SAB Selvanesan S, Hannah PYP, NurulAina MCA, W.NurAfiza WMA Zirwatul AA, Hani MH, Nurulsyahida I, Aziyati O, Ushananthiny R, Kamal HKZ, Mohd AY, YF Ngeow
EPI_ISL_2013325, EPI_ISL_2015101, EPI_ISL_2015102, EPI_ISL_2015105, EPI_ISL_2015106, EPI_ISL_2015107, EPI_ISL_2015108, EPI_ISL_2015109, EPI_ISL_2015110, EPI_ISL_2015111, EPI_ISL_2015115, EPI_ISL_2015124, EPI_ISL_2015126, EPI_ISL_2015141			
see above	IU-Cerrahpasa, Cerrahpasa School of Medicine, COVID-19 Lab	IU-Cerrahpasa, Cerrahpasa School of Medicine, COVID-19 Lab	Mert Kuskucu, Yesim Tuyji Tok, Kenan Midilli
EPI_ISL_2040230	Cliniques universitaires Saint-Luc	UCLouvain/IREC/MBLG	Jean Ruelle, Eleonore Ngyuvula, Benoit Kabamba Mukadi
EPI_ISL_2081876, EPI_ISL_2081878, EPI_ISL_2081886, EPI_ISL_2081887, EPI_ISL_2081902, EPI_ISL_2081905, EPI_ISL_2081909, EPI_ISL_2081910, EPI_ISL_2081912, EPI_ISL_2081915, EPI_ISL_2081929, EPI_ISL_2081930			
see above	National Center of Infectious and Parasitic Diseases	National Center of Infectious and Parasitic Diseases	Alexiev, Ivanov, Korsun, Stoitsova, Philipova, Dimitrova, Grigorova L., Hristova, Donchev, Stoykov, Trifonova, Dobrinov, Grigorova I., Kantardjiev
EPI_ISL_2103371, EPI_ISL_2103373	HOSPITAL SAN JUAN DE DIOS	Inciensa, Instituto Costarricense de Investigación y	Francisco Duarte, Hebleen Brenes, Claudio Soto-Garita, Estela Cordero, Adriana Godinez & Melany Calderon

		Enseñanza en Nutrición y Salud	
EPI_ISL_2148602	Clínica Meta	Instituto de Virología-Universidad El Bosque	L. Johana Madroñero, Eliana P. Calvo, Maria Fernanda Montañez-Suarez, Adriana Franco, Andres F. Cardona-Rios, Maria Stella López, Jhann Andrés Arturo, Lilia Bernal-Cepeda, Sonia P. Bohórquez, María Angélica Calderón-Peláez, Sigrid J. Camacho-Ortega, Carolina Coronel-Ruiz, Felix G. Delgado, Lady Lopez, Myriam Lucia Velandia-Romero, Laura Silvana Pérez, Karl A Ciuoderis, Jorge E. Osorio, Juan P. Hernandez-Ortiz, Jaime E. Castellanos
EPI_ISL_2171040	Dhulikhel Hospital, Kathmandu University Hospital	Molecular and Genomics Research Lab, Dhulikhel Hospital, Kathmandu University Hospital	Rajeev Shrestha, Nishan Katuwal, Navin Adhikari, Manu Vanaerschot, Dipesh Tamrakar, Meghnath Dhimal, Pradip Gyanwali, Saroj Bhattarai, Surendra Kumar Madhup
EPI_ISL_2196362	Lboratorio Central de Saude Publica do Estado do Parana (LACEN/PR)	Laboratory of Respiratory Viruses and Measles, Oswaldo Cruz Institute, FIOCRUZ	Paola Resende, Luciana Appolinario, Fernando Motta, Anna Carolina Paixao, Ana Carolina Mendonca, Alice Sampaio Rocha, Taina Venas, Elisa Cavalcante Pereira, Renata Serrano Lopes, Irina Riediger, Marilda Siqueira on behalf of the Fiocruz COVID-19 Genomic Surveillance Network
EPI_ISL_2226216	Department of Internal Medicine, College of Medicine, Chosun University	Department of Internal Medicine, College of Medicine, Chosun University	Dong-Min Kim
EPI_ISL_2231475, EPI_ISL_2231478, EPI_ISL_2231485	San Diego County Public Health Laboratory	Andersen lab at Scripps Research	SEARCH Alliance San Diego with Ashleigh Murphy, Jovan Shephard, Brett Austin
EPI_ISL_2276072, EPI_ISL_2276073, EPI_ISL_2276074, EPI_ISL_2276076	Regional Authority of Public Health Trenín	Veterinary institute in Zvolen Slovakia	Dirbáková Z., Buková L., Reizigová L., Beová K., Sujová S., Mokryšová S., Tinák M., Mojžiš M.
EPI_ISL_2278896	Institut National d'Hygiène (INH)	Unité Mixte Internationale TransVIHMI (UMI 233 IRD - U1175 INSERM - Université de Montpellier)IRD (Institut de recherche pour le développement)	Mounerou SALOU, Christelle BUTEL, Wembo A. HALATOKO,Issaka Maman, Abla A. KONOU, Amivi EHLAN, Adodo SADJI, Kokou TEGUENI,Sidonie A.M.KAGNISSODE, Akoélé SILIADIN, Alassane OURO-MEDELI, Messanh DOUFFAN,Déléma MABA,Sika DOSSIM, Améyo DORKENOO, Mireille PRINCE-DAVID,Anoumou DAGNRA,Laetitia SERRANO,Ahidjo AYOUBA,Eric DELAPORTE, Martine PEETERS
EPI_ISL_2285105, EPI_ISL_2285126, EPI_ISL_2285155	Microbiology Department. Complexo Hospitalario Universitario de Vigo	Microbiology Department. Complexo Hospitalario Universitario de Vigo	Alfaya N, Alonso I, Alvarez M, Cabrera JJ, Carballo R, Cores O, Cortizo S, del-Campo V, Martinez L, Mediero G, Perez S, Potel C, Regueiro B, Rey S, Vasallo FJ
EPI_ISL_2322456, EPI_ISL_2322457, EPI_ISL_2322458, EPI_ISL_2322459, EPI_ISL_2322460, EPI_ISL_2322461, EPI_ISL_2322462, EPI_ISL_2322463, EPI_ISL_2322464	Genome Analysis Center, Yamanashi Central Hospital	Genome Analysis Center, Yamanashi Central Hospital	Yosuke Hirotsu
EPI_ISL_2339878, EPI_ISL_2339890, EPI_ISL_2339907	Centro de Estudio de Enfermedades Autoinmunes (CREA), Universidad del Rosario, Bogota, Colombia	Centro de Investigaciones en Microbiología y Biotecnología-UR (CIMBIUR), Facultad de Ciencias Naturales, Universidad del Rosario, Bogotá, Colombia	Juan-Manuel Anaya, Gustavo Salguero, Juan Esteban Gallo, Carolina Ramírez-Santana, Sergio Castañeda, Nathalia Ballesteros, Marina Muñoz, Luz H. Patiño, Juan David Ramírez
EPI_ISL_2350809, EPI_ISL_2350810, EPI_ISL_2350811, EPI_ISL_2350812, EPI_ISL_2350813, EPI_ISL_2350814, EPI_ISL_2350815, EPI_ISL_2350816	Molecular Lab, Evercare hospital Dhaka	International Institute for Zoonosis Control, Hokkaido university	Junya Yamagishi, Mizanur Rahman
EPI_ISL_2351932	Centre for Human Virology and Genomics, Microbiology Department, Nigerian Institute of Medical Research	Central Research Laboratory, Nigerian Institute of Medical Research	Chika Kingsley Onwuamah, Ayorinde Babatunde James, Joseph Ojonugwa Shaibu, Rahaman A. Ahmed, Azuka Patrick Okwuraiwe, Olufemi Samuel Amoo, Sola Ajibaye, Bamidele Iwalokun, Rosemary Ajuma Audu, Babatunde Lawal Salako
EPI_ISL_2364491, EPI_ISL_2364492, EPI_ISL_2364493, EPI_ISL_2364494, EPI_ISL_2364495, EPI_ISL_2364496, EPI_ISL_2364497, EPI_ISL_2364498, EPI_ISL_2364499	Genome Analysis Center, Yamanashi Central Hospital	Genome Analysis Center, Yamanashi Central Hospital	Yosuke Hirotsu
EPI_ISL_2365883	Microbiology Department. Complexo Hospitalario Universitario de Vigo	Microbiology Department. Complexo Hospitalario Universitario de Vigo	Alfaya N, Alonso I, Alvarez M, Cabrera JJ, Carballo R, Cores O, Cortizo S, del-Campo V, Martinez L, Mediero G, Perez S, Potel C, Regueiro B, Rey S, Vasallo FJ
EPI_ISL_2382422, EPI_ISL_2382423	Centre for Human Virology and Genomics, Microbiology Department, Nigerian Institute of Medical Research	Central Research Laboratory, Nigerian Institute of Medical Research	Chika Kingsley Onwuamah, Ayorinde Babatunde James, Joseph Ojonugwa Shaibu, Rahaman A. Ahmed, Azuka Patrick Okwuraiwe, Sola Ajibaye, Sharon Abimbola, Olufemi Samuel Amoo, Muinah Adenike Fowora, Phasha-Muchemenye Mmatshepho, Joy Ayoola, Nyam Itse Yusuf, Grace Oni, Yusuf Jimoh, Josiah Ayoola Isong, Uyi Emokpae, Ngozi Mirabel Otuonye, Rosemary Ajuma Audu, Bamidele Iwalokun, Babatunde Lawal Salako
EPI_ISL_2397307	NHLS-IALCH	KRISP, KZN Research Innovation and Sequencing Platform	Karim F, Moosa MYS, Gosnell BI, Cele S, Giandhari J, Pillay S, Tegally H, Wilkinson E, San JE, Msomi N, Mlisana K, Khan K, Bernstein M, Manickchund N, Singh L, Ramphal U, COMMIT-KZN Team, Hanekom W, Lessells RJ, Sigal A, de Oliveira T
EPI_ISL_2398288, EPI_ISL_2398290	Centre for Human Virology and Genomics, Microbiology Department, Nigerian Institute of Medical Research	Central Research Laboratory, Nigerian Institute of Medical Research	Chika Kingsley Onwuamah, Ayorinde Babatunde James, Joseph Ojonugwa Shaibu, Rahaman A. Ahmed, Azuka Patrick Okwuraiwe, Sola Ajibaye, Sharon Abimbola, Olufemi Samuel Amoo, Muinah Adenike Fowora, Phasha-Muchemenye Mmatshepho, Joy Ayoola, Nyam Itse Yusuf, Grace Oni, Yusuf Jimoh, Josiah Ayoola Isong, Uyi Emokpae, Ngozi Mirabel Otuonye, Rosemary Ajuma Audu, Bamidele Iwalokun, Babatunde Lawal Salako
EPI_ISL_2448653	Molecular diagnostic laboratory of Federal Budget Institution of Science "Central Research Institute of Epidemiology" of The Federal Service on Customers' Rights Protection and Human Well-being Surveillance	Group of Genomics and Postgenomic Technologies of Central Research Institute of Epidemiology	Samoilov A.E., Kaptelova V.V., Korneenko E.V., Valdohina A.V., Bulanenko V.P., Saenko S.S., Sinitsyn S.O., Golubeva A.G., Speranskaya A.S., Tivanova E.V., Shipulina O.Y., Akimkin V.G.
EPI_ISL_2458065	Adolfo Lutz Institute Santos Regional Center	Retrovirus Laboratory Adolfo Lutz Institute	Gabriela Bastos Cabral, Giselle I S Lopez-Lopes, Cintia Ahagon, Audrey Cilli, Paula Morena Guimaraes, Igor Mohamed Hussein, Luis Brigido
EPI_ISL_2476401	Department of Molecular and Translational Medicine, Section of Microbiology, University of Brescia, ASST Spedali Civili, Brescia	Department of Molecular and Translational Medicine, Section of Microbiology, University of Brescia, ASST Spedali Civili, Brescia	Simona Fiorentini, Serena Messali, Alberto Zani, Francesca Caccuri, Arnaldo Caruso
EPI_ISL_2484147	Mater Misericordiae University Hospital Dublin	University College Dublin, CFS, School of Public Health	Maureen Lynch, Guerrino Macori, Séamus Fanning, Edel O' Regan, Eoin Hunt, Dermot O'Callaghan, Brian McCullagh, Cormac Jennings, Anne Fortune
EPI_ISL_2484215, EPI_ISL_2484216, EPI_ISL_2484217	University of Brescia - Dept. of Molecular and Translational Medicine	Istituto Zooprofilattico Sperimentale della Lombardia e dell'Emilia Romagna (IZSLER), Risk Analysis and Genomic Epidemiology Unit	Caruso Arnaldo, Fiorentini Simona, Caccuri Francesca, Messali Serena, Zani Alberto, Marina Morganti, Erika Scaltriti, Stefano Pongolini
EPI_ISL_2501079, EPI_ISL_2501080, EPI_ISL_2501082, EPI_ISL_2501083, EPI_ISL_2501085, EPI_ISL_2501086, EPI_ISL_2502018, EPI_ISL_2502020, EPI_ISL_2502021, EPI_ISL_2502022, EPI_ISL_2502025			
see above	Centre for Human Virology and Genomics, Microbiology Department, Nigerian Institute of Medical Research	Central Research Laboratory, Nigerian Institute of Medical Research	Chika Kingsley Onwuamah, Ayorinde Babatunde James, Joseph Ojonugwa Shaibu, Rahaman A. Ahmed, Azuka Patrick Okwuraiwe, Sola Ajibaye, Sharon Abimbola, Olufemi Samuel Amoo, Muinah Adenike Fowora, Phasha-Muchemenye Mmatshepho, Joy Ayoola, Nyam Itse Yusuf, Grace Oni, Yusuf Jimoh, Josiah Ayoola Isong, Uyi Emokpae, Ngozi Mirabel Otuonye, Rosemary Ajuma Audu, Bamidele Iwalokun, Babatunde Lawal Salako
EPI_ISL_2502630, EPI_ISL_2502635, EPI_ISL_2502636, EPI_ISL_2502638, EPI_ISL_2502649, EPI_ISL_2502656, EPI_ISL_2502661	All India Institute of Medical Sciences Delhi Hospital	Virology Laboratory, AIIMS Delhi	Aashish Choudhary, Sumedha Bagga, Lata Rani, Dibyabhaba Pradhan, Pooja Pandey, Jyoti Jethani, Shivram Dhakad, Nazneen Arif, Deepankar Srigyan, Megha Brijwal, Lalit Dar, Manish Soneja, Rakesh Lodha, Puneet Kaur, Ritu Gupta, Subrata Sinha, Chitra Sarkar, Randeep Guleria
EPI_ISL_2603237, EPI_ISL_2603251, EPI_ISL_2603255	Microbiology Department. Complexo Hospitalario Universitario de Vigo	Microbiology Department. Complexo Hospitalario Universitario de Vigo	Alfaya N, Alonso I, Alvarez M, Cabrera JJ, Carballo R, Cores O, Cortizo S, del-Campo V, Martinez L, Mediero G, Perez S, Potel C, Regueiro B, Rey S, Vasallo FJ
EPI_ISL_2603816	Oman-National Influenza Center	Biotechnology & OMICs Laboratory	Intisar Al-Shukri, Ahmed Al Harrasi, Aisha Al-Amri, Amal Al-Maani, Sajjad Asaf, Fahad Al-Balushi, Laila Al-Balushi, Ahmed N Al-Rawahi, Saqib Bilal, Abdul Latif Khan, Samira Al-Mahruqi, Ahmed Al-Rawahi, Hanan Al-Kindi, Amina Al-Jardani.
EPI_ISL_2614543	IdenGene Medicina Diagnostica SA	Instituto Adolfo Lutz, Interdiciplinary Procedures Center,	Claudio Tavares Sacchi, Claudia Regina Gonçalves, Erica Valessa Ramos Gomes, Karoline Rodrigues Campos, Caio Vinicius Dias Lopes, Leonardo Jose

		Strategic Laboratory	Tadeu de Araujo
EPI_ISL_2625925	Microbiology Unit ASST Grande Ospedale Metropolitano Niguarda, Milan, Italy	Microbiology Unit ASST Grande Ospedale Metropolitano Niguarda, Milan, Italy	Merli M. et al.
EPI_ISL_2671836, EPI_ISL_2671846, EPI_ISL_2671847, EPI_ISL_2671848	Juntendo University Hospital	Juntendo University Hospital	Koji Tuchiya, Yoshie Hosaka
EPI_ISL_2690146, EPI_ISL_2690147, EPI_ISL_2690148, EPI_ISL_2690149, EPI_ISL_2690180, EPI_ISL_2690181, EPI_ISL_2690182	Ochsner Health	Bioinfoexperts, LLC	Judy Crabtree, Amy Feehan, Rebecca Rose, David J. Nolan, Tessa LaFleur, Julia-Garcia-Diaz, Lucio Miele, Susanna L. Lamers
EPI_ISL_2692890	Department of Infectious Disease, Ardabil University of Medical Sciences, Ardabil, Iran	Genetics Research Center, University of Social Welfare and Rehabilitation Sciences	Zohreh Fattahi, Marzieh Mohseni, Khadijeh Jalalvand, Azam Ghaziasadi, Seyedeh elham Mortazavi, Ali Jafarpour, Shahram Habibzadeh, Farhad Jeddi, Kimia Kahrizi, Seyed Mohammad Jazayeri, Hossein Najmabadi.
EPI_ISL_2692893	Pars Hospital Laboratory, Rasht, Iran.	Genetics Research Center, University of Social Welfare and Rehabilitation Sciences	Zohreh Fattahi, Marzieh Mohseni, Khadijeh Jalalvand, Issa Jahanzad, Kimia Kahrizi, Hossein Najmabadi.
EPI_ISL_2692895	Department of pathology, school of medicine, Imam Hospital complex, Tehran university of medical sciences, Tehran, Iran	Genetics Research Center, University of Social Welfare and Rehabilitation Sciences	Zohreh Fattahi, Marzieh Mohseni, Khadijeh Jalalvand, Azam Ghaziasadi, Seyedeh elham Mortazavi, Ali Jafarpour, Azar Hadadi, Alireza Abdollahi, Seyedeh elham Mortazavi, Saber Soltani, Reza Najafipour, Kimia Kahrizi, Seyed Mohammad Jazayeri, Hossein Najmabadi.
EPI_ISL_2692901	Department of pathology, school of medicine, Imam Hospital complex, Tehran university of medical sciences, Tehran,Iran	Genetics Research Center, University of Social Welfare and Rehabilitation Sciences	Zohreh Fattahi, Marzieh Mohseni, Khadijeh Jalalvand, Azam Ghaziasadi, Seyedeh elham Mortazavi, Ali Jafarpour, Azar Hadadi, Alireza Abdollahi, Saber Soltani, Reza Najafipour, Kimia Kahrizi, Seyed Mohammad Jazayeri, Hossein Najmabadi.
EPI_ISL_2692904	Pars Hospital Laboratory, Rasht, Iran.	Genetics Research Center, University of Social Welfare and Rehabilitation Sciences	Zohreh Fattahi, Marzieh Mohseni, Khadijeh Jalalvand, Issa Jahanzad, Kimia Kahrizi, Hossein Najmabadi.
EPI_ISL_2692906, EPI_ISL_2692907, EPI_ISL_2692908	Department of pathology, school of medicine, Imam Hospital complex, Tehran university of medical sciences, Tehran, Iran	Genetics Research Center, University of Social Welfare and Rehabilitation Sciences	Zohreh Fattahi, Marzieh Mohseni, Khadijeh Jalalvand, Azam Ghaziasadi, Seyedeh elham Mortazavi, Ali Jafarpour, Azar Hadadi, Alireza Abdollahi, Saber Soltani, Reza Najafipour, Kimia Kahrizi, Seyed Mohammad Jazayeri, Hossein Najmabadi.
EPI_ISL_2692910	Pars Hospital Laboratory, Rasht, Iran.	Genetics Research Center, University of Social Welfare and Rehabilitation Sciences	Zohreh Fattahi, Marzieh Mohseni, Khadijeh Jalalvand, Issa Jahanzad ,Kimia Kahrizi, Hossein Najmabadi.
EPI_ISL_2692953	Pars Hospital Laboratory, Rasht, Iran.	Genetics Research Center, University of Social Welfare and Rehabilitation Sciences	Zohreh Fattahi, Marzieh Mohseni, Khadijeh Jalalvand, Issa Jahanzad, Kimia Kahrizi, Hossein Najmabadi.
EPI_ISL_2692982, EPI_ISL_2692984, EPI_ISL_2692985, EPI_ISL_2692988	Department of pathology, school of medicine, Imam Hospital complex, Tehran university of medical sciences, Tehran, Iran	Genetics Research Center, University of Social Welfare and Rehabilitation Sciences	Zohreh Fattahi, Marzieh Mohseni, Khadijeh Jalalvand, Azam Ghaziasadi, Seyedeh elham Mortazavi, Ali Jafarpour, Azar Hadadi, Alireza Abdollahi, Saber Soltani, Reza Najafipour, Kimia Kahrizi, Seyed Mohammad Jazayeri, Hossein Najmabadi.
EPI_ISL_2692996	Oman-National Influenza Center	Biotechnology & OMICs Laboratory	Ahmed Al Harrasi, Aisha Al-Amri, Intisar Al-Shukri, Amal Al-Maani, Sajjad Asaf, Bilal Hussain, Samiya Al-Zadjali, Ahmed N Al-Rawahi, Saqib Bilal, Abdul Latif Khan, Samira Al-Mahruqi, Ahmed Al-Rawahi, Hanan Al-Kindi,
EPI_ISL_2692997	Oman-National Influenza Center	Biotechnology & OMICs Laboratory	Aisha Al-Amri, Ahmed Al Harrasi, Intisar Al-Shukri, Amal Al-Maani, Sajjad Asaf, Bilal Hussain, Samiya Al-Zadjali, Ahmed N Al-Rawahi, Saqib Bilal, Abdul Latif Khan, Samira Al-Mahruqi, Ahmed Al-Rawahi, Hanan Al-Kindi,Amina Al-Jardani.
EPI_ISL_2694588, EPI_ISL_2694589, EPI_ISL_2694590	Virology Centre, School of public health, Tehran University of Medical Sciences, Tehran, Iran	Genetics Research Center, University of Social Welfare and Rehabilitation Sciences	Zohreh Fattahi, Marzieh Mohseni, Khadijeh Jalalvand, Jila Yavarian, Kimia Kahrizi, Talat Mokhtari-Azad, Hossein Najmabadi.
EPI_ISL_2694591	Department of pathology, school of medicine, Imam Hospital complex, Tehran university of medical sciences, Tehran, Iran	Genetics Research Center, University of Social Welfare and Rehabilitation Sciences	Zohreh Fattahi, Marzieh Mohseni, Khadijeh Jalalvand, Jila Yavarian, Kimia Kahrizi, Talat Mokhtari-Azad, Hossein Najmabadi.
EPI_ISL_2694592	Virology Centre, School of public health, Tehran University of Medical Sciences, Tehran, Iran	Genetics Research Center, University of Social Welfare and Rehabilitation Sciences	Zohreh Fattahi, Marzieh Mohseni, Khadijeh Jalalvand, Jila Yavarian, Kimia Kahrizi, Talat Mokhtari-Azad, Hossein Najmabadi.
EPI_ISL_2696940, EPI_ISL_2696941	All India Institute of Medical Sciences Delhi Hospital	Virology Laboratory, AIIMS Delhi	Aashish Choudhary, Sumedha Bagga, Lata Rani, Dibyabhaba Pradhan, Pooja Pandey, Jyoti Jethani, Shivram Dhakad, Nazneen Arif, Deepankar Srigyan, Megha Brijwal, Lalit Dar, Manish Soneja, Rakesh Lodha, Puneet Kaur, Ritu Gupta, Subrata Sinha, Chitra Sarkar, Randeep Guleria
EPI_ISL_2710839, EPI_ISL_2710840, EPI_ISL_2710841, EPI_ISL_2710842, EPI_ISL_2710843, EPI_ISL_2710844, EPI_ISL_2710845, EPI_ISL_2710846, EPI_ISL_2710847, EPI_ISL_2710848, EPI_ISL_2710849, EPI_ISL_2710850, EPI_ISL_2710851, EPI_ISL_2710852, EPI_ISL_2710853, EPI_ISL_2710854, EPI_ISL_2710855, EPI_ISL_2710856, EPI_ISL_2710857, EPI_ISL_2710858, EPI_ISL_2710859, EPI_ISL_2710860, EPI_ISL_2710861, EPI_ISL_2710862, EPI_ISL_2710863, EPI_ISL_2710864, EPI_ISL_2710865, EPI_ISL_2710866, EPI_ISL_2710867, EPI_ISL_2710868, EPI_ISL_2710869, EPI_ISL_2710870, EPI_ISL_2710871, EPI_ISL_2710883, EPI_ISL_2713076, EPI_ISL_2713119			
see above	Department of Health Technology and Informatics, The Hong Kong Polytechnic University	Department of Health Technology and Informatics, The Hong Kong Polytechnic University	Gilman Kit-Hang Siu, Lam-Kwong Lee, Kenneth Siu-Sing Leung, Jake Siu-Lun Leung, Wing-Hei Lo, Timothy Ting-Leung Ng, Chloe Toi-Mei Chan, Hoi-Ching Jim, Wison Tak-Fai Wong, Kingsley King-Gee Tam, Hiu-Yin Lao, Denise Sze-Hang Wong, Alan Ka-Lun Wu, Miranda Ching-Yee Yau, Yvette Wai-Man Lai, Kitty Sau-Chun Fung, Sandy Ka-Yee Chau, Barry Kin-Chung Wong, Wing-Kin To, Kristine Luk, Alex Yat-Man Ho, Tak-Lun Que, Kam-Tong Yip, Wing Cheong Yam, David Ho-Keung Shum, Shea Ping Yip
EPI_ISL_2727892, EPI_ISL_2727893, EPI_ISL_2727894	All India Institute of Medical Sciences Delhi Hospital	Virology Laboratory, AIIMS Delhi	Aashish Choudhary, Sumedha Bagga, Lata Rani, Dibyabhaba Pradhan, Pooja Pandey, Jyoti Jethani, Shivram Dhakad, Nazneen Arif, Deepankar Srigyan, Megha Brijwal, Lalit Dar, Manish Soneja, Rakesh Lodha, Puneet Kaur, Ritu Gupta, Subrata Sinha, Chitra Sarkar, Randeep Guleria
EPI_ISL_2731461, EPI_ISL_2731466, EPI_ISL_2731467	Lboratorio Central de Saude Publica do Estado do Parana (LACEN/PR)	Laboratory of Respiratory Viruses and Measles, Oswaldo Cruz Institute, FIOCRUZ	Paola Resende, Luciana Appolinario, Fernando Motta, Ana Carolina Paixao, Ana Carolina Mendonca, Alice Sampaio Rocha, Taina Venas, Elisa Cavalcante Pereira, Renata Serrano Lopes, Irina Riediger, Marilda Siqueira on behalf of the Fiocruz COVID-19 Genomic Surveillance Network
EPI_ISL_2779398, EPI_ISL_2779399	MOH-K	USAMRD-A, Basic Science Laboratory	Gathii Kimita, Josphat Nyataya, Esther Omuseni, Faith Sigei, Alan Lemtudo, Eric Muthanje, Brian Andika, Rehema Liyai, Rachel Githii, Clement Masakwe, Stephen Ochola, George Awinda, Carol Kifude, Beth Mutai, John Waitumbi
EPI_ISL_2779418, EPI_ISL_2779421, EPI_ISL_2779424, EPI_ISL_2779425, EPI_ISL_2779426, EPI_ISL_2779427, EPI_ISL_2779428, EPI_ISL_2779429, EPI_ISL_2779430	Port Health Clinic, Busia border point	USAMRD-A, Basic Science Laboratory	Gathii Kimita, Josphat Nyataya, Esther Omuseni, Faith Sigei, Alan Lemtudo, Eric Muthanje, Brian Andika, Rehema Liyai, Rachel Githii, Clement Masakwe, Stephen Ochola, George Awinda, Carol Kifude, Beth Mutai, John Waitumbi
EPI_ISL_2779431, EPI_ISL_2779432, EPI_ISL_2779433	DFMH	USAMRD-A, Basic Science Laboratory	Gathii Kimita, Josphat Nyataya, Esther Omuseni, Faith Sigei, Alan Lemtudo, Eric Muthanje, Brian Andika, Rehema Liyai, Rachel Githii, Clement Masakwe, Stephen Ochola, George Awinda, Carol Kifude, Beth Mutai, John Waitumbi
EPI_ISL_2779435, EPI_ISL_2779436, EPI_ISL_2779438, EPI_ISL_2779439, EPI_ISL_2779441, EPI_ISL_2779442, EPI_ISL_2779443, EPI_ISL_2779444, EPI_ISL_2779445, EPI_ISL_2779446, EPI_ISL_2779448			
see above	MOH-K	USAMRD-A, Basic Science Laboratory	Gathii Kimita, Josphat Nyataya, Esther Omuseni, Faith Sigei, Alan Lemtudo, Eric Muthanje, Brian Andika, Rehema Liyai, Rachel Githii, Clement Masakwe, Stephen Ochola, George Awinda, Carol Kifude, Beth Mutai, John Waitumbi
EPI_ISL_2779451, EPI_ISL_2779452	MOH-HQ	USAMRD-A, Basic Science Laboratory	Gathii Kimita, Josphat Nyataya, Esther Omuseni, Faith Sigei, Alan Lemtudo, Eric Muthanje, Brian Andika, Rehema Liyai, Rachel Githii, Clement Masakwe, Stephen Ochola, George Awinda, Carol Kifude, Beth Mutai, John Waitumbi
EPI_ISL_2779454	MOH-K	USAMRD-A, Basic Science Laboratory	Gathii Kimita, Josphat Nyataya, Esther Omuseni, Faith Sigei, Alan Lemtudo, Eric Muthanje, Brian Andika, Rehema Liyai, Rachel Githii, Clement Masakwe, Stephen Ochola, George Awinda, Carol Kifude, Beth Mutai, John Waitumbi
EPI_ISL_2779455	KCRH KEMRI/DEID Surveillance Site	USAMRD-A, Basic Science Laboratory	Gathii Kimita, Josphat Nyataya, Esther Omuseni, Faith Sigei, Alan Lemtudo, Eric Muthanje, Brian Andika, Rehema Liyai, Rachel Githii, Clement Masakwe, Stephen Ochola, George Awinda, Carol Kifude, Beth Mutai, John Waitumbi
EPI_ISL_2779467, EPI_ISL_2779469, EPI_ISL_2779470, EPI_ISL_2779471, EPI_ISL_2779472, EPI_ISL_2779473	MOH-K	USAMRD-A, Basic Science Laboratory	Gathii Kimita, Josphat Nyataya, Esther Omuseni, Faith Sigei, Alan Lemtudo, Eric Muthanje, Brian Andika, Rehema Liyai, Rachel Githii, Clement Masakwe, Stephen Ochola, George Awinda, Carol Kifude, Beth Mutai, John Waitumbi
EPI_ISL_2779474	DFMH	USAMRD-A, Basic Science Laboratory	Gathii Kimita, Josphat Nyataya, Esther Omuseni, Faith Sigei, Alan Lemtudo, Eric Muthanje, Brian Andika, Rehema Liyai, Rachel Githii, Clement Masakwe,

			Stephen Ochola, George Awinda, Carol Kifude, Beth Mutai, John Waitumbi
EPI_ISL_2779475	MOH-K	USAMRD-A, Basic Science Laboratory	Gathii Kimita, Josphat Nyataya, Esther Omuseni, Faith Sigei, Alan Lemtudo, Eric Muthanje, Brian Andika, Rehema Liyai, Rachel Githii, Clement Masakwe, Stephen Ochola, George Awinda, Carol Kifude, Beth Mutai, John Waitumbi
EPI_ISL_2779482	MOH-HQ	USAMRD-A, Basic Science Laboratory	Gathii Kimita, Josphat Nyataya, Esther Omuseni, Faith Sigei, Alan Lemtudo, Eric Muthanje, Brian Andika, Rehema Liyai, Rachel Githii, Clement Masakwe, Stephen Ochola, George Awinda, Carol Kifude, Beth Mutai, John Waitumbi
EPI_ISL_2779484, EPI_ISL_2779485	DFMH	USAMRD-A, Basic Science Laboratory	Gathii Kimita, Josphat Nyataya, Esther Omuseni, Faith Sigei, Alan Lemtudo, Eric Muthanje, Brian Andika, Rehema Liyai, Rachel Githii, Clement Masakwe, Stephen Ochola, George Awinda, Carol Kifude, Beth Mutai, John Waitumbi
EPI_ISL_2779489, EPI_ISL_2779490	Lanet hospital	USAMRD-A, Basic Science Laboratory	Gathii Kimita, Josphat Nyataya, Esther Omuseni, Faith Sigei, Alan Lemtudo, Eric Muthanje, Brian Andika, Rehema Liyai, Rachel Githii, Clement Masakwe, Stephen Ochola, George Awinda, Carol Kifude, Beth Mutai, John Waitumbi
EPI_ISL_2779495, EPI_ISL_2779496, EPI_ISL_2779497, EPI_ISL_2779498, EPI_ISL_2779499, EPI_ISL_2779500, EPI_ISL_2779501, EPI_ISL_2779502, EPI_ISL_2779503, EPI_ISL_2779504, EPI_ISL_2779505, EPI_ISL_2779506, EPI_ISL_2779507, EPI_ISL_2779508			
see above	Port Health Clinic, Malaba border point	USAMRD-A, Basic Science Laboratory	Gathii Kimita, Josphat Nyataya, Esther Omuseni, Faith Sigei, Alan Lemtudo, Eric Muthanje, Brian Andika, Rehema Liyai, Rachel Githii, Clement Masakwe, Stephen Ochola, George Awinda, Carol Kifude, Beth Mutai, John Waitumbi
EPI_ISL_2779522, EPI_ISL_2779523	MOH-HQS	USAMRD-A, Basic Science Laboratory	Gathii Kimita, Josphat Nyataya, Esther Omuseni, Faith Sigei, Alan Lemtudo, Eric Muthanje, Brian Andika, Rehema Liyai, Rachel Githii, Clement Masakwe, Stephen Ochola, George Awinda, Carol Kifude, Beth Mutai, John Waitumbi
EPI_ISL_2779524	DFMH	USAMRD-A, Basic Science Laboratory	Gathii Kimita, Josphat Nyataya, Esther Omuseni, Faith Sigei, Alan Lemtudo, Eric Muthanje, Brian Andika, Rehema Liyai, Rachel Githii, Clement Masakwe, Stephen Ochola, George Awinda, Carol Kifude, Beth Mutai, John Waitumbi
EPI_ISL_2779525	LAB-BMC	USAMRD-A, Basic Science Laboratory	Gathii Kimita, Josphat Nyataya, Esther Omuseni, Faith Sigei, Alan Lemtudo, Eric Muthanje, Brian Andika, Rehema Liyai, Rachel Githii, Clement Masakwe, Stephen Ochola, George Awinda, Carol Kifude, Beth Mutai, John Waitumbi
EPI_ISL_2779538, EPI_ISL_2779540, EPI_ISL_2779541	MOH-HQ	USAMRD-A, Basic Science Laboratory	Gathii Kimita, Josphat Nyataya, Esther Omuseni, Faith Sigei, Alan Lemtudo, Eric Muthanje, Brian Andika, Rehema Liyai, Rachel Githii, Clement Masakwe, Stephen Ochola, George Awinda, Carol Kifude, Beth Mutai, John Waitumbi
EPI_ISL_2779542, EPI_ISL_2779543	DFMH	USAMRD-A, Basic Science Laboratory	Gathii Kimita, Josphat Nyataya, Esther Omuseni, Faith Sigei, Alan Lemtudo, Eric Muthanje, Brian Andika, Rehema Liyai, Rachel Githii, Clement Masakwe, Stephen Ochola, George Awinda, Carol Kifude, Beth Mutai, John Waitumbi
EPI_ISL_2779549	MOH-K	USAMRD-A, Basic Science Laboratory	Gathii Kimita, Josphat Nyataya, Esther Omuseni, Faith Sigei, Alan Lemtudo, Eric Muthanje, Brian Andika, Rehema Liyai, Rachel Githii, Clement Masakwe, Stephen Ochola, George Awinda, Carol Kifude, Beth Mutai, John Waitumbi
EPI_ISL_2790004	All India Institute of Medical Sciences Delhi Hospital	Virology Laboratory, AIIMS Delhi	Aashish Choudhary, Sumedha Bagga, Lata Rani, Dibyabhaba Pradhan, Pooja Pandey, Jyoti Jethani, Shivram Dhakad, Nazneen Arif, Deepankar Srigyan, Megha Brijwal, Lalit Dar, Manish Soneja, Rakesh Lodha, Puneet Kaur, Ritu Gupta, Subrata Sinha, Chitra Sarkar, Randeep Guleria
EPI_ISL_2790881, EPI_ISL_2790882	Labo Analyses Med	National Reference Center for Viruses of Respiratory Infections, Institut Pasteur, Paris	Marion Barbet, Sylvie Behillil, Méline Bizard, Angela Brisebarre, Camille Capel, Vincent Enouf, Louise Lefrançois, Frédéric Lemoine, Christophe Malabat, Corinne Maufrais, Emmanuelle Permal, Etienne Simon-Lorière, Maud Vanpeene, Sylvie Van der Werf ,StéPhane Romand
EPI_ISL_2790889, EPI_ISL_2790890, EPI_ISL_2790891, EPI_ISL_2790892, EPI_ISL_2790893, EPI_ISL_2790894, EPI_ISL_2790895, EPI_ISL_2790896, EPI_ISL_2790897, EPI_ISL_2790898, EPI_ISL_2790899, EPI_ISL_2790900			
see above	Labo Analyses Med	National Reference Center for Viruses of Respiratory Infections, Institut Pasteur, Paris	Marion Barbet, Sylvie Behillil, Méline Bizard, Angela Brisebarre, Camille Capel, Vincent Enouf, Louise Lefrançois, Frédéric Lemoine, Christophe Malabat, Corinne Maufrais, Emmanuelle Permal, Etienne Simon-Lorière, Maud Vanpeene, Sylvie Van der Werf ,Brieuc Gestin
EPI_ISL_2835364, EPI_ISL_2835367, EPI_ISL_2835368, EPI_ISL_2835369, EPI_ISL_2835370, EPI_ISL_2835374, EPI_ISL_2835380, EPI_ISL_2835381, EPI_ISL_2835382, EPI_ISL_2835383, EPI_ISL_2835384, EPI_ISL_2835385, EPI_ISL_2835386, EPI_ISL_2835387, EPI_ISL_2835388, EPI_ISL_2835389, EPI_ISL_2835390, EPI_ISL_2835391, EPI_ISL_2835392, EPI_ISL_2835393, EPI_ISL_2835394, EPI_ISL_2835395, EPI_ISL_2835396, EPI_ISL_2835397, EPI_ISL_2835398, EPI_ISL_2835399, EPI_ISL_2835400, EPI_ISL_2835401, EPI_ISL_2835402, EPI_ISL_2835403, EPI_ISL_2835404, EPI_ISL_2835405, EPI_ISL_2835407, EPI_ISL_2835408, EPI_ISL_2835409, EPI_ISL_2835410, EPI_ISL_2835411, EPI_ISL_2835412, EPI_ISL_2835413, EPI_ISL_2835414, EPI_ISL_2835415, EPI_ISL_2835416, EPI_ISL_2835417, EPI_ISL_2835420, EPI_ISL_2835421, EPI_ISL_2835422, EPI_ISL_2835423, EPI_ISL_2835424, EPI_ISL_2835425, EPI_ISL_2835426, EPI_ISL_2835427, EPI_ISL_2835429, EPI_ISL_2835430, EPI_ISL_2835431, EPI_ISL_2835432, EPI_ISL_2835433, EPI_ISL_2835434, EPI_ISL_2835435, EPI_ISL_2835436, EPI_ISL_2835437, EPI_ISL_2835439, EPI_ISL_2835440, EPI_ISL_2835441, EPI_ISL_2835442, EPI_ISL_2835443, EPI_ISL_2835444, EPI_ISL_2835445, EPI_ISL_2835446, EPI_ISL_2835447, EPI_ISL_2835448, EPI_ISL_2835449, EPI_ISL_2835450, EPI_ISL_2835451, EPI_ISL_2835452, EPI_ISL_2835453, EPI_ISL_2835454, EPI_ISL_2835455, EPI_ISL_2835456, EPI_ISL_2835457, EPI_ISL_2835458, EPI_ISL_2835459, EPI_ISL_2835460, EPI_ISL_2835461, EPI_ISL_2835462, EPI_ISL_2835463, EPI_ISL_2835464, EPI_ISL_2835465, EPI_ISL_2835466, EPI_ISL_2835467, EPI_ISL_2835468, EPI_ISL_2835469, EPI_ISL_2835470, EPI_ISL_2835471, EPI_ISL_2835472, EPI_ISL_2835473, EPI_ISL_2835474, EPI_ISL_2835475, EPI_ISL_2835476, EPI_ISL_2835477, EPI_ISL_2835478, EPI_ISL_2835479, EPI_ISL_2835480, EPI_ISL_2835481, EPI_ISL_2835482, EPI_ISL_2835483, EPI_ISL_2835484, EPI_ISL_2835485, EPI_ISL_2835486, EPI_ISL_2835487, EPI_ISL_2835488, EPI_ISL_2835489			
see above	Instituto de Diagnostico y Referencia Epidemiologicos (InDRE)	Andersen lab at Scripps Research	SEARCH Alliance with Gisela Barrera Badillo, Jose Ernesto Ramirez Gonzalez, Maria Natividad Cruz Ortiz, Nervain Benjamin Contreras Gonzalez, Tatiana Ernestina Nunez Garcia, Dayanira Sarith Arellano Suarez, Irma Lopez Martinez, Rita Flores Leon, Beatriz Olivares Flores
EPI_ISL_2860774, EPI_ISL_2860775, EPI_ISL_2860776	Department of Health Technology and Informatics, The Hong Kong Polytechnic University	Department of Health Technology and Informatics, The Hong Kong Polytechnic University	Gilman Kit-Hang Siu, Lam-Kwong Lee, Kenneth Siu-Sing Leung, Jake Siu-Lun Leung, Wing-Hei Lo, Timothy Ting-Leung Ng, Chloe Toi-Mei Chan, Hoi-Ching Jim, Ivan Tak-Fai Wong, Kingsley King-Gee Tam, Hiu-Yin Lao, Denise Sze-Hang Wong, Alan Ka-Lun Wu, Miranda Chong-Yee Yau, Yvette Wai-Man Lai, Kitty Sau-Chun Fung, Sandy Ka-Yee Chau, Barry Kin-Chung Wong, Wing-Kin To, Kristine Luk, Alex Yat-Man Ho, Tak-Lun Que, Kam-Tong Yip, Wing Cheong Yam, David Ho-Keung Shum, Shea Ping Yip
EPI_ISL_455476	Laboratory for Respiratory Viruses, Cantacuzino National Military-Medical Institute for Research and Development	Cantacuzino Institute	M.Lazar, L.Ustea, A.Cretu, T.Durfee
EPI_ISL_458086	B.J. Medical College and Civil hospital	Gujarat Biotechnology Research Centre	Dhaval Vaghela, Ramesh Patel, Pranay Shah, Kamlesh J Upadhyay, Ramesh Pandit, Tejas Shah, Ankit Hinsu, Pritesh Sabara, Apurvasinh Puvar, Janvi Raval, Zarna Patel, Monika Gandhi, Pinal Trivedi, Maharshi Pandya, Amit Kanani, Nidhi Patel, Nitin Savaliya, Raghawendra Kumar, Dinesh Kumar, Zuber Saiyed, Komal Patel, Labdhi Pandya, Snehal Bagatharia, Neha Rajpara, Bhavesh Modi, Gaurishankar Shrimali, R D Dixit, A M Kadri, Umang Mishra, Chaitanya Joshi, Madhvi Joshi
EPI_ISL_458087	B.J. Medical College and Civil hospital	Gujarat Biotechnology Research Centre	Ramesh Patel, Pranay Shah, Kamlesh J Upadhyay, Ramesh Pandit, Tejas Shah, Ankit Hinsu, Pritesh Sabara, Apurvasinh Puvar, Janvi Raval, Zarna Patel, Monika Gandhi, Pinal Trivedi, Maharshi Pandya, Amit Kanani, Nidhi Patel, Nitin Savaliya, Raghawendra Kumar, Dinesh Kumar, Zuber Saiyed, Komal Patel, Labdhi Pandya, Snehal Bagatharia, Dhaval Vaghela, Afzal Ansari, Bhavesh Modi, Gaurishankar Shrimali, R D Dixit, A M Kadri, Umang Mishra, Chaitanya Joshi, Madhvi Joshi
EPI_ISL_458088	B.J. Medical College and Civil hospital	Gujarat Biotechnology Research Centre	Pranay Shah, Kamlesh J Upadhyay, Ramesh Pandit, Tejas Shah, Ankit Hinsu, Pritesh Sabara, Apurvasinh Puvar, Janvi Raval, Zarna Patel, Monika Gandhi, Pinal Trivedi, Maharshi Pandya, Amit Kanani, Nidhi Patel, Nitin Savaliya, Raghawendra Kumar, Dinesh Kumar, Zuber Saiyed, Komal Patel, Labdhi Pandya, Snehal Bagatharia, Dhaval Vaghela, Ramesh Patel, Fenil Patel, Bhavesh Modi, Gaurishankar Shrimali, R D Dixit, A M Kadri, Umang Mishra, Chaitanya Joshi, Madhvi Joshi
EPI_ISL_458089	B.J. Medical College and Civil hospital	Gujarat Biotechnology Research Centre	Pranay Shah, Kamlesh J Upadhyay, Ramesh Pandit, Tejas Shah, Ankit Hinsu, Pritesh Sabara, Apurvasinh Puvar, Janvi Raval, Zarna Patel, Monika Gandhi, Pinal Trivedi, Maharshi Pandya, Amit Kanani, Nidhi Patel, Nitin Savaliya, Raghawendra Kumar, Dinesh Kumar, Zuber Saiyed, Komal Patel, Labdhi Pandya, Snehal Bagatharia, Dhaval Vaghela, Ramesh Patel, Neelam Nathani, Bhavesh Modi, Gaurishankar Shrimali, R D Dixit, A M Kadri, Umang Mishra, Chaitanya Joshi, Madhvi Joshi
EPI_ISL_458090	B.J. Medical College and Civil hospital	Gujarat Biotechnology Research Centre	Kamlesh J Upadhyay, Ramesh Pandit, Tejas Shah, Ankit Hinsu, Pritesh Sabara, Apurvasinh Puvar, Janvi Raval, Zarna Patel, Monika Gandhi, Pinal Trivedi, Maharshi Pandya, Amit Kanani, Nidhi Patel, Nitin Savaliya, Raghawendra Kumar, Dinesh Kumar, Zuber Saiyed, Komal Patel, Labdhi Pandya, Snehal Bagatharia, Dhaval Vaghela, Ramesh Patel, Pranay Shah, Armi Chaudhari, Bhavesh Modi, Gaurishankar Shrimali, R D Dixit, A M Kadri, Umang Mishra, Chaitanya Joshi, Madhvi Joshi
EPI_ISL_458091	B.J. Medical College and Civil hospital	Gujarat Biotechnology Research Centre	Maharshi Pandya, Amit Kanani, Nidhi Patel, Nitin Savaliya, Raghawendra Kumar, Dinesh Kumar, Zuber Saiyed, Komal Patel, Labdhi Pandya, Snehal Bagatharia, Dhaval Vaghela, Ramesh Patel, Pranay Shah, Kamlesh J Upadhyay, Ramesh Pandit, Tejas Shah, Ankit Hinsu, Pritesh Sabara, Apurvasinh Puvar, Janvi Raval, Zarna Patel, Monika Gandhi, Pinal Trivedi, Bhavya Jindal, Bhavesh Modi, Gaurishankar Shrimali, R D Dixit, A M Kadri, Umang Mishra, Chaitanya Joshi, Madhvi Joshi
EPI_ISL_458092	B.J. Medical College and Civil hospital	Gujarat Biotechnology Research Centre	Amit Kanani, Nidhi Patel, Nitin Savaliya, Raghawendra Kumar, Dinesh Kumar, Zuber Saiyed, Komal Patel, Labdhi Pandya, Snehal Bagatharia, Dhaval Vaghela, Ramesh Patel, Pranay Shah, Kamlesh J Upadhyay, Ramesh Pandit, Tejas Shah, Ankit Hinsu, Pritesh Sabara, Apurvasinh Puvar, Janvi Raval, Zarna Patel, Monika Gandhi, Pinal Trivedi, Maharshi Pandya, Camellia Chakraborty, Bhavesh Modi, Gaurishankar Shrimali, R D Dixit, A M Kadri, Umang Mishra, Chaitanya Joshi, Madhvi Joshi

EPI_ISL_458093	B.J. Medical College and Civil hospital	Gujarat Biotechnology Research Centre	Nidhi Patel, Nitin Savaliya, Raghawendra Kumar, Dinesh Kumar, Zuber Saiyed, Komal Patel, Labdhi Pandya, Snehal Bagatharia, Dhaval Vaghela, Ramesh Patel, Pranay Shah, Kamlesh J Upadhyay, Ramesh Pandit, Tejas Shah, Ankit Hinsu, Pritesh Sabara, Apurvasinh Puvar, Janvi Raval, Zarna Patel, Monika Gandhi, Pinal Trivedi, Maharshi Pandya, Amit Kanani, Siddhant Kumar, Bhavesh Modi, Gaurishankar Shrimali, R D Dixit, A M Kadri, Umang Mishra, Chaitanya Joshi, Madhvi Joshi
EPI_ISL_458094	B.J. Medical College and Civil hospital	Gujarat Biotechnology Research Centre	Nitin Savaliya, Raghawendra Kumar, Dinesh Kumar, Zuber Saiyed, Komal Patel, Labdhi Pandya, Snehal Bagatharia, Dhaval Vaghela, Ramesh Patel, Pranay Shah, Kamlesh J Upadhyay, Ramesh Pandit, Tejas Shah, Ankit Hinsu, Pritesh Sabara, Apurvasinh Puvar, Janvi Raval, Zarna Patel, Monika Gandhi, Pinal Trivedi, Maharshi Pandya, Amit Kanani, Nidhi Patel, Priyanka P Vatsa, Bhavesh Modi, Gaurishankar Shrimali, R D Dixit, A M Kadri, Umang Mishra, Chaitanya Joshi, Madhvi Joshi
EPI_ISL_458095	B.J. Medical College and Civil hospital	Gujarat Biotechnology Research Centre	Raghawendra Kumar, Dinesh Kumar, Zuber Saiyed, Komal Patel, Labdhi Pandya, Snehal Bagatharia, Dhaval Vaghela, Ramesh Patel, Pranay Shah, Kamlesh J Upadhyay, Ramesh Pandit, Tejas Shah, Ankit Hinsu, Pritesh Sabara, Apurvasinh Puvar, Janvi Raval, Zarna Patel, Monika Gandhi, Pinal Trivedi, Maharshi Pandya, Amit Kanani, Nidhi Patel, Nitin Savaliya, Pooja P Doshi, Bhavesh Modi, Gaurishankar Shrimali, R D Dixit, A M Kadri, Umang Mishra, Chaitanya Joshi, Madhvi Joshi
EPI_ISL_458096	B.J. Medical College and Civil hospital	Gujarat Biotechnology Research Centre	Dinesh Kumar, Zuber Saiyed, Komal Patel, Labdhi Pandya, Snehal Bagatharia, Dhaval Vaghela, Ramesh Patel, Pranay Shah, Kamlesh J Upadhyay, Ramesh Pandit, Tejas Shah, Ankit Hinsu, Pritesh Sabara, Apurvasinh Puvar, Janvi Raval, Zarna Patel, Monika Gandhi, Pinal Trivedi, Maharshi Pandya, Amit Kanani, Nidhi Patel, Nitin Savaliya, Raghawendra Kumar, Akanksha Verma, Bhavesh Modi, Gaurishankar Shrimali, R D Dixit, A M Kadri, Umang Mishra, Chaitanya Joshi, Madhvi Joshi
EPI_ISL_458097	B.J. Medical College and Civil hospital	Gujarat Biotechnology Research Centre	Zuber Saiyed, Komal Patel, Labdhi Pandya, Snehal Bagatharia, Dhaval Vaghela, Ramesh Patel, Pranay Shah, Kamlesh J Upadhyay, Ramesh Pandit, Tejas Shah, Ankit Hinsu, Pritesh Sabara, Apurvasinh Puvar, Janvi Raval, Zarna Patel, Monika Gandhi, Pinal Trivedi, Maharshi Pandya, Amit Kanani, Nidhi Patel, Nitin Savaliya, Raghawendra Kumar, Dinesh Kumar, Priti Pandita, Bhavesh Modi, Gaurishankar Shrimali, R D Dixit, A M Kadri, Umang Mishra, Chaitanya Joshi, Madhvi Joshi
EPI_ISL_458098	B.J. Medical College and Civil hospital	Gujarat Biotechnology Research Centre	Komal Patel, Labdhi Pandya, Snehal Bagatharia, Dhaval Vaghela, Ramesh Patel, Pranay Shah, Kamlesh J Upadhyay, Ramesh Pandit, Tejas Shah, Ankit Hinsu, Pritesh Sabara, Apurvasinh Puvar, Janvi Raval, Zarna Patel, Monika Gandhi, Pinal Trivedi, Maharshi Pandya, Amit Kanani, Nidhi Patel, Nitin Savaliya, Raghawendra Kumar, Dinesh Kumar, Zuber Saiyed, Pragya Sharma, Bhavesh Modi, Gaurishankar Shrimali, R D Dixit, A M Kadri, Umang Mishra, Chaitanya Joshi, Madhvi Joshi
EPI_ISL_458099	B.J. Medical College and Civil hospital	Gujarat Biotechnology Research Centre	Labdhi Pandya, Snehal Bagatharia, Dhaval Vaghela, Ramesh Patel, Pranay Shah, Kamlesh J Upadhyay, Ramesh Pandit, Tejas Shah, Ankit Hinsu, Pritesh Sabara, Apurvasinh Puvar, Janvi Raval, Zarna Patel, Monika Gandhi, Pinal Trivedi, Maharshi Pandya, Amit Kanani, Nidhi Patel, Nitin Savaliya, Raghawendra Kumar, Dinesh Kumar, Zuber Saiyed, Komal Patel, Neha Rajpara, Bhavesh Modi, Gaurishankar Shrimali, R D Dixit, A M Kadri, Umang Mishra, Chaitanya Joshi, Madhvi Joshi
EPI_ISL_458100	B.J. Medical College and Civil hospital	Gujarat Biotechnology Research Centre	Snehal Bagatharia, Dhaval Vaghela, Ramesh Patel, Pranay Shah, Kamlesh J Upadhyay, Ramesh Pandit, Tejas Shah, Ankit Hinsu, Pritesh Sabara, Apurvasinh Puvar, Janvi Raval, Zarna Patel, Monika Gandhi, Pinal Trivedi, Maharshi Pandya, Amit Kanani, Nidhi Patel, Nitin Savaliya, Raghawendra Kumar, Dinesh Kumar, Zuber Saiyed, Komal Patel, Labdhi Pandya, Afzal Ansari, Bhavesh Modi, Gaurishankar Shrimali, R D Dixit, A M Kadri, Umang Mishra, Chaitanya Joshi, Madhvi Joshi
EPI_ISL_458101	B.J. Medical College and Civil hospital	Gujarat Biotechnology Research Centre	Dhaval Vaghela, Ramesh Patel, Pranay Shah, Kamlesh J Upadhyay, Ramesh Pandit, Tejas Shah, Ankit Hinsu, Pritesh Sabara, Apurvasinh Puvar, Janvi Raval, Zarna Patel, Monika Gandhi, Pinal Trivedi, Maharshi Pandya, Amit Kanani, Nidhi Patel, Nitin Savaliya, Raghawendra Kumar, Dinesh Kumar, Zuber Saiyed, Komal Patel, Labdhi Pandya, Snehal Bagatharia, Fenil Patel, Bhavesh Modi, Gaurishankar Shrimali, R D Dixit, A M Kadri, Umang Mishra, Chaitanya Joshi, Madhvi Joshi
EPI_ISL_458102	B.J. Medical College and Civil hospital	Gujarat Biotechnology Research Centre	Ramesh Patel, Pranay Shah, Kamlesh J Upadhyay, Ramesh Pandit, Tejas Shah, Ankit Hinsu, Pritesh Sabara, Apurvasinh Puvar, Janvi Raval, Zarna Patel, Monika Gandhi, Pinal Trivedi, Maharshi Pandya, Amit Kanani, Nidhi Patel, Nitin Savaliya, Raghawendra Kumar, Dinesh Kumar, Zuber Saiyed, Komal Patel, Labdhi Pandya, Snehal Bagatharia, Dhaval Vaghela, Neelam Nathani, Bhavesh Modi, Gaurishankar Shrimali, R D Dixit, A M Kadri, Umang Mishra, Chaitanya Joshi, Madhvi Joshi
EPI_ISL_458106	Gujarat Biotechnology Research Centre	Gujarat Biotechnology Research Centre	Pritesh Sabara, Apurvasinh Puvar, Janvi Raval, Zarna Patel, Monika Gandhi, Pinal Trivedi, Maharshi Pandya, Amit Kanani, Nidhi Patel, Nitin Savaliya, Raghawendra Kumar, Dinesh Kumar, Zuber Saiyed, Komal Patel, Labdhi Pandya, Snehal Bagatharia, Ramesh Pandit, Tejas Shah, Ankit Hinsu, Siddhant Kumar, Bhavesh Modi, Gaurishankar Shrimali, R D Dixit, A M Kadri, Umang Mishra, Chaitanya Joshi, Madhvi Joshi, , , , ,
EPI_ISL_458107	Gujarat Biotechnology Research Centre	Gujarat Biotechnology Research Centre	Apurvasinh Puvar, Janvi Raval, Zarna Patel, Monika Gandhi, Pinal Trivedi, Maharshi Pandya, Amit Kanani, Nidhi Patel, Nitin Savaliya, Raghawendra Kumar, Dinesh Kumar, Zuber Saiyed, Komal Patel, Labdhi Pandya, Snehal Bagatharia, Ramesh Pandit, Tejas Shah, Ankit Hinsu, Pritesh Sabara, Priyanka P Vatsa, Bhavesh Modi, Gaurishankar Shrimali, R D Dixit, A M Kadri, Umang Mishra, Chaitanya Joshi, Madhvi Joshi, , , , ,
EPI_ISL_458111	Gujarat Biotechnology Research Centre	Gujarat Biotechnology Research Centre	Pinal Trivedi, Maharshi Pandya, Amit Kanani, Nidhi Patel, Nitin Savaliya, Raghawendra Kumar, Dinesh Kumar, Zuber Saiyed, Komal Patel, Labdhi Pandya, Snehal Bagatharia, Ramesh Pandit, Tejas Shah, Ankit Hinsu, Pritesh Sabara, Apurvasinh Puvar, Janvi Raval, Zarna Patel, Monika Gandhi, Pragya Sharma, Bhavesh Modi, Gaurishankar Shrimali, R D Dixit, A M Kadri, Umang Mishra, Chaitanya Joshi, Madhvi Joshi, , , , ,
EPI_ISL_458112	Gujarat Biotechnology Research Centre	Gujarat Biotechnology Research Centre	Maharshi Pandya, Amit Kanani, Nidhi Patel, Nitin Savaliya, Raghawendra Kumar, Dinesh Kumar, Zuber Saiyed, Komal Patel, Labdhi Pandya, Snehal Bagatharia, Ramesh Pandit, Tejas Shah, Ankit Hinsu, Pritesh Sabara, Apurvasinh Puvar, Janvi Raval, Zarna Patel, Monika Gandhi, Pinal Trivedi, Neha Rajpara, Bhavesh Modi, Gaurishankar Shrimali, R D Dixit, A M Kadri, Umang Mishra, Chaitanya Joshi, Madhvi Joshi, , , , ,
EPI_ISL_458113	Gujarat Biotechnology Research Centre	Gujarat Biotechnology Research Centre	Amit Kanani, Nidhi Patel, Nitin Savaliya, Raghawendra Kumar, Dinesh Kumar, Zuber Saiyed, Komal Patel, Labdhi Pandya, Snehal Bagatharia, Ramesh Pandit, Tejas Shah, Ankit Hinsu, Pritesh Sabara, Apurvasinh Puvar, Janvi Raval, Zarna Patel, Monika Gandhi, Pinal Trivedi, Maharshi Pandya, Afzal Ansari, Bhavesh Modi, Gaurishankar Shrimali, R D Dixit, A M Kadri, Umang Mishra, Chaitanya Joshi, Madhvi Joshi, , , , ,
EPI_ISL_459919, EPI_ISL_459920	Devki Devi Foundation, a unit of Max Healthcare	CSIR-IGIB/Max	Rajesh Pandey#, Samreen Siddiqui, Pooja Sharma, Bansidhar Tarai, Vivekanand A, Bharathram Uppili, Saruchi Wadhwa, Nishu Tyagi, Mitali Mukerji, Bansidhar Tarai, Poonam Das, Sujeet Jha, Mohammed Faruq, Vinita Jha, Anurag Agrawal
EPI_ISL_461483	B.J. Medical College and Civil hospital	Gujarat Biotechnology Research Centre	Dipeshwari Shewale, Komal Patel, Labdhi Pandya, Snehal Bagatharia, Pranay Shah, Kamlesh J Upadhyay, Tejas Shah, Ankit Hinsu, Pritesh Sabara, Apurvasinh Puvar, Janvi Raval, Zarna Patel, Monika Gandhi, Pinal Trivedi, Maharshi Pandya, Nidhi Patel, Nitin Savaliya, Raghawendra Kumar, Dinesh Kumar, Zuber Saiyed, R D Dixit, A M Kadri, Harsh Bakshi, Chaitanya Joshi, Madhvi Joshi,
EPI_ISL_461484	B.J. Medical College and Civil hospital	Gujarat Biotechnology Research Centre	Priyanka P Vatsa, Labdhi Pandya, Snehal Bagatharia, Pranay Shah, Kamlesh J Upadhyay, Tejas Shah, Ankit Hinsu, Pritesh Sabara, Apurvasinh Puvar, Janvi Raval, Zarna Patel, Monika Gandhi, Pinal Trivedi, Maharshi Pandya, Nidhi Patel, Nitin Savaliya, Raghawendra Kumar, Dinesh Kumar, Zuber Saiyed, Komal Patel, R D Dixit, A M Kadri, Harsh Bakshi, Chaitanya Joshi, Madhvi Joshi,
EPI_ISL_461485	B.J. Medical College and Civil hospital	Gujarat Biotechnology Research Centre	Pooja P Doshi, Snehal Bagatharia, Pranay Shah, Kamlesh J Upadhyay, Tejas Shah, Ankit Hinsu, Pritesh Sabara, Apurvasinh Puvar, Janvi Raval, Zarna Patel, Monika Gandhi, Pinal Trivedi, Maharshi Pandya, Nidhi Patel, Nitin Savaliya, Raghawendra Kumar, Dinesh Kumar, Zuber Saiyed, Komal Patel, Labdhi Pandya, R D Dixit, A M Kadri, Harsh Bakshi, Chaitanya Joshi, Madhvi Joshi,
EPI_ISL_461486	B.J. Medical College and Civil hospital	Gujarat Biotechnology Research Centre	Akanksha Verma, Pranay Shah, Kamlesh J Upadhyay, Tejas Shah, Ankit Hinsu, Pritesh Sabara, Apurvasinh Puvar, Janvi Raval, Zarna Patel, Monika Gandhi, Pinal Trivedi, Maharshi Pandya, Nidhi Patel, Nitin Savaliya, Raghawendra Kumar, Dinesh Kumar, Zuber Saiyed, Komal Patel, Labdhi Pandya, Snehal Bagatharia, R D Dixit, A M Kadri, Harsh Bakshi, Chaitanya Joshi, Madhvi Joshi,
EPI_ISL_461487	B.J. Medical College and Civil hospital	Gujarat Biotechnology Research Centre	Priti Pandita, Kamlesh J Upadhyay, Tejas Shah, Ankit Hinsu, Pritesh Sabara, Apurvasinh Puvar, Janvi Raval, Zarna Patel, Monika Gandhi, Pinal Trivedi, Maharshi Pandya, Nidhi Patel, Nitin Savaliya, Raghawendra Kumar, Dinesh Kumar, Zuber Saiyed, Komal Patel, Labdhi Pandya, Snehal Bagatharia, Pranay Shah, R D Dixit, A M Kadri, Harsh Bakshi, Chaitanya Joshi, Madhvi Joshi,
EPI_ISL_461488	B.J. Medical College and Civil hospital	Gujarat Biotechnology Research Centre	Pragya Sharma, Tejas Shah, Ankit Hinsu, Pritesh Sabara, Apurvasinh Puvar, Janvi Raval, Zarna Patel, Monika Gandhi, Pinal Trivedi, Maharshi Pandya, Nidhi Patel, Nitin Savaliya, Raghawendra Kumar, Dinesh Kumar, Zuber Saiyed, Komal Patel, Labdhi Pandya, Snehal Bagatharia, Pranay Shah, Kamlesh J Upadhyay, R D Dixit, A M Kadri, Harsh Bakshi, Chaitanya Joshi, Madhvi Joshi,
EPI_ISL_461489	B.J. Medical College and Civil hospital	Gujarat Biotechnology Research Centre	Neha Rajpara, Ankit Hinsu, Pritesh Sabara, Apurvasinh Puvar, Janvi Raval, Zarna Patel, Monika Gandhi, Pinal Trivedi, Maharshi Pandya, Nidhi Patel, Nitin

			Savaliya, Raghawendra Kumar, Dinesh Kumar, Zuber Saiyed, Komal Patel, Labdhi Pandya, Snehal Bagatharia, Pranay Shah, Kamlesh J Upadhyay, Tejas Shah, R D Dixit, A M Kadri, Harsh Bakshi, Chaitanya Joshi, Madhvi Joshi,
EPI_ISL_461490	B.J. Medical College and Civil hospital	Gujarat Biotechnology Research Centre	Afzal Ansari, Pritesh Sabara, Apurvasinh Puvar, Janvi Raval, Zarna Patel, Monika Gandhi, Pinal Trivedi, Maharshi Pandya, Nidhi Patel, Nitin Savaliya, Raghawendra Kumar, Dinesh Kumar, Zuber Saiyed, Komal Patel, Labdhi Pandya, Snehal Bagatharia, Pranay Shah, Kamlesh J Upadhyay, Tejas Shah, Ankit Hinsu, R D Dixit, A M Kadri, Harsh Bakshi, Chaitanya Joshi, Madhvi Joshi,
EPI_ISL_461491	B.J. Medical College and Civil hospital	Gujarat Biotechnology Research Centre	Fenil Patel, Apurvasinh Puvar, Janvi Raval, Zarna Patel, Monika Gandhi, Pinal Trivedi, Maharshi Pandya, Nidhi Patel, Nitin Savaliya, Raghawendra Kumar, Dinesh Kumar, Zuber Saiyed, Komal Patel, Labdhi Pandya, Snehal Bagatharia, Pranay Shah, Kamlesh J Upadhyay, Tejas Shah, Ankit Hinsu, Pritesh Sabara, R D Dixit, A M Kadri, Harsh Bakshi, Chaitanya Joshi, Madhvi Joshi,
EPI_ISL_461492	B.J. Medical College and Civil hospital	Gujarat Biotechnology Research Centre	Neelam Nathani, Janvi Raval, Zarna Patel, Monika Gandhi, Pinal Trivedi, Maharshi Pandya, Nidhi Patel, Nitin Savaliya, Raghawendra Kumar, Dinesh Kumar, Zuber Saiyed, Komal Patel, Labdhi Pandya, Snehal Bagatharia, Pranay Shah, Kamlesh J Upadhyay, Tejas Shah, Ankit Hinsu, Pritesh Sabara, Apurvasinh Puvar, R D Dixit, A M Kadri, Harsh Bakshi, Chaitanya Joshi, Madhvi Joshi,
EPI_ISL_461493	B.J. Medical College and Civil hospital	Gujarat Biotechnology Research Centre	Armi Chaudhari, Zarna Patel, Monika Gandhi, Pinal Trivedi, Maharshi Pandya, Nidhi Patel, Nitin Savaliya, Raghawendra Kumar, Dinesh Kumar, Zuber Saiyed, Komal Patel, Labdhi Pandya, Snehal Bagatharia, Pranay Shah, Kamlesh J Upadhyay, Tejas Shah, Ankit Hinsu, Pritesh Sabara, Apurvasinh Puvar, Janvi Raval, R D Dixit, A M Kadri, Harsh Bakshi, Chaitanya Joshi, Madhvi Joshi,
EPI_ISL_461494	B.J. Medical College and Civil hospital	Gujarat Biotechnology Research Centre	Bhavya Jindal, Monika Gandhi, Pinal Trivedi, Maharshi Pandya, Nidhi Patel, Nitin Savaliya, Raghawendra Kumar, Dinesh Kumar, Zuber Saiyed, Komal Patel, Labdhi Pandya, Snehal Bagatharia, Pranay Shah, Kamlesh J Upadhyay, Tejas Shah, Ankit Hinsu, Pritesh Sabara, Apurvasinh Puvar, Janvi Raval, Zarna Patel, R D Dixit, A M Kadri, Harsh Bakshi, Chaitanya Joshi, Madhvi Joshi,
EPI_ISL_461495	B.J. Medical College and Civil hospital	Gujarat Biotechnology Research Centre	Anjali Rajwar, Pinal Trivedi, Maharshi Pandya, Nidhi Patel, Nitin Savaliya, Raghawendra Kumar, Dinesh Kumar, Zuber Saiyed, Komal Patel, Labdhi Pandya, Snehal Bagatharia, Pranay Shah, Kamlesh J Upadhyay, Tejas Shah, Ankit Hinsu, Pritesh Sabara, Apurvasinh Puvar, Janvi Raval, Zarna Patel, Monika Gandhi, R D Dixit, A M Kadri, Harsh Bakshi, Chaitanya Joshi, Madhvi Joshi,
EPI_ISL_461496	B.J. Medical College and Civil hospital	Gujarat Biotechnology Research Centre	Dipeshwari Shewale, Maharshi Pandya, Nidhi Patel, Nitin Savaliya, Raghawendra Kumar, Dinesh Kumar, Zuber Saiyed, Komal Patel, Labdhi Pandya, Snehal Bagatharia, Pranay Shah, Kamlesh J Upadhyay, Tejas Shah, Ankit Hinsu, Pritesh Sabara, Apurvasinh Puvar, Janvi Raval, Zarna Patel, Monika Gandhi, Pinal Trivedi, R D Dixit, A M Kadri, Harsh Bakshi, Chaitanya Joshi, Madhvi Joshi,
EPI_ISL_461497	B.J. Medical College and Civil hospital	Gujarat Biotechnology Research Centre	Priyanka P Vatsa, Nidhi Patel, Nitin Savaliya, Raghawendra Kumar, Dinesh Kumar, Zuber Saiyed, Komal Patel, Labdhi Pandya, Snehal Bagatharia, Pranay Shah, Kamlesh J Upadhyay, Tejas Shah, Ankit Hinsu, Pritesh Sabara, Apurvasinh Puvar, Janvi Raval, Zarna Patel, Monika Gandhi, Pinal Trivedi, Maharshi Pandya, R D Dixit, A M Kadri, Harsh Bakshi, Chaitanya Joshi, Madhvi Joshi,
EPI_ISL_461498	B.J. Medical College and Civil hospital	Gujarat Biotechnology Research Centre	Pooja P Doshi, Nitin Savaliya, Raghawendra Kumar, Dinesh Kumar, Zuber Saiyed, Komal Patel, Labdhi Pandya, Snehal Bagatharia, Pranay Shah, Kamlesh J Upadhyay, Tejas Shah, Ankit Hinsu, Pritesh Sabara, Apurvasinh Puvar, Janvi Raval, Zarna Patel, Monika Gandhi, Pinal Trivedi, Maharshi Pandya, Nidhi Patel, R D Dixit, A M Kadri, Harsh Bakshi, Chaitanya Joshi, Madhvi Joshi,
EPI_ISL_461499	B.J. Medical College and Civil hospital	Gujarat Biotechnology Research Centre	Akanksha Verma, Raghawendra Kumar, Dinesh Kumar, Zuber Saiyed, Komal Patel, Labdhi Pandya, Snehal Bagatharia, Pranay Shah, Kamlesh J Upadhyay, Tejas Shah, Ankit Hinsu, Pritesh Sabara, Apurvasinh Puvar, Janvi Raval, Zarna Patel, Monika Gandhi, Pinal Trivedi, Maharshi Pandya, Nidhi Patel, Nitin Savaliya, R D Dixit, A M Kadri, Harsh Bakshi, Chaitanya Joshi, Madhvi Joshi,
EPI_ISL_461500	B.J. Medical College and Civil hospital	Gujarat Biotechnology Research Centre	Priti Pandita, Dinesh Kumar, Zuber Saiyed, Komal Patel, Labdhi Pandya, Snehal Bagatharia, Pranay Shah, Kamlesh J Upadhyay, Tejas Shah, Ankit Hinsu, Pritesh Sabara, Apurvasinh Puvar, Janvi Raval, Zarna Patel, Monika Gandhi, Pinal Trivedi, Maharshi Pandya, Nidhi Patel, Nitin Savaliya, Raghawendra Kumar, R D Dixit, A M Kadri, Harsh Bakshi, Chaitanya Joshi, Madhvi Joshi,
EPI_ISL_461501	B.J. Medical College and Civil hospital	Gujarat Biotechnology Research Centre	Pragya Sharma, Zuber Saiyed, Komal Patel, Labdhi Pandya, Snehal Bagatharia, Pranay Shah, Kamlesh J Upadhyay, Tejas Shah, Ankit Hinsu, Pritesh Sabara, Apurvasinh Puvar, Janvi Raval, Zarna Patel, Monika Gandhi, Pinal Trivedi, Maharshi Pandya, Nidhi Patel, Nitin Savaliya, Raghawendra Kumar, Dinesh Kumar, R D Dixit, A M Kadri, Harsh Bakshi, Chaitanya Joshi, Madhvi Joshi,
EPI_ISL_461502	B.J. Medical College and Civil hospital	Gujarat Biotechnology Research Centre	Neha Rajpara, Komal Patel, Labdhi Pandya, Snehal Bagatharia, Pranay Shah, Kamlesh J Upadhyay, Tejas Shah, Ankit Hinsu, Pritesh Sabara, Apurvasinh Puvar, Janvi Raval, Zarna Patel, Monika Gandhi, Pinal Trivedi, Maharshi Pandya, Nidhi Patel, Nitin Savaliya, Raghawendra Kumar, Dinesh Kumar, Zuber Saiyed, R D Dixit, A M Kadri, Harsh Bakshi, Chaitanya Joshi, Madhvi Joshi,
EPI_ISL_461503	B.J. Medical College and Civil hospital	Gujarat Biotechnology Research Centre	Afzal Ansari, Labdhi Pandya, Snehal Bagatharia, Pranay Shah, Kamlesh J Upadhyay, Tejas Shah, Ankit Hinsu, Pritesh Sabara, Apurvasinh Puvar, Janvi Raval, Zarna Patel, Monika Gandhi, Pinal Trivedi, Maharshi Pandya, Nidhi Patel, Nitin Savaliya, Raghawendra Kumar, Dinesh Kumar, Zuber Saiyed, Komal Patel, R D Dixit, A M Kadri, Harsh Bakshi, Chaitanya Joshi, Madhvi Joshi,
EPI_ISL_461504	B.J. Medical College and Civil hospital	Gujarat Biotechnology Research Centre	Snehal Bagatharia, Pranay Shah, Kamlesh J Upadhyay, Tejas Shah, Ankit Hinsu, Pritesh Sabara, Apurvasinh Puvar, Janvi Raval, Zarna Patel, Monika Gandhi, Pinal Trivedi, Maharshi Pandya, Nidhi Patel, Nitin Savaliya, Raghawendra Kumar, Dinesh Kumar, Zuber Saiyed, Komal Patel, Labdhi Pandya, Fenil Patel, R D Dixit, A M Kadri, Harsh Bakshi, Chaitanya Joshi, Madhvi Joshi,
EPI_ISL_461505	B.J. Medical College and Civil hospital	Gujarat Biotechnology Research Centre	Pranay Shah, Kamlesh J Upadhyay, Tejas Shah, Ankit Hinsu, Pritesh Sabara, Apurvasinh Puvar, Janvi Raval, Zarna Patel, Monika Gandhi, Pinal Trivedi, Maharshi Pandya, Nidhi Patel, Nitin Savaliya, Raghawendra Kumar, Dinesh Kumar, Zuber Saiyed, Komal Patel, Labdhi Pandya, Snehal Bagatharia, Neelam Nathani, R D Dixit, A M Kadri, Harsh Bakshi, Chaitanya Joshi, Madhvi Joshi,
EPI_ISL_461506	B.J. Medical College and Civil hospital	Gujarat Biotechnology Research Centre	Kamlesh J Upadhyay, Tejas Shah, Ankit Hinsu, Pritesh Sabara, Apurvasinh Puvar, Janvi Raval, Zarna Patel, Monika Gandhi, Pinal Trivedi, Maharshi Pandya, Nidhi Patel, Nitin Savaliya, Raghawendra Kumar, Dinesh Kumar, Zuber Saiyed, Komal Patel, Labdhi Pandya, Snehal Bagatharia, Pranay Shah, Armi Chaudhari, R D Dixit, A M Kadri, Harsh Bakshi, Chaitanya Joshi, Madhvi Joshi,
EPI_ISL_462753	University Clinical Hospital of Mostar	University of Sarajevo Veterinary Faculty	Goletic, T., Softic, A., Goletic, S., Ostojic, M., Hukic, M., Eterovic, T., Seho-Alic, A.
EPI_ISL_467029	GMERS Medical College and Hospital, Gandhinagar	Gujarat Biotechnology Research Centre	Seema Bhatt, Gaurishankar Shrimali, Bhavesh Modi, Bharti Rajani, Tejas Shah, Ankit Hinsu, Pritesh Sabara, Apurvasinh Puvar, Janvi Raval, Zarna Patel, Monika Gandhi, Pinal Trivedi, Maharshi Pandya, Nidhi Patel, Nitin Savaliya, Raghawendra Kumar, Dinesh Kumar, Zuber Saiyed, Komal Patel, Labdhi Pandya, Snehal Bagatharia, Bhavya Jindal, R D Dixit, A M Kadri, Harsh Bakshi, Chaitanya Joshi, Madhvi Joshi
EPI_ISL_467030	GMERS Medical College and Hospital, Gandhinagar	Gujarat Biotechnology Research Centre	Gaurishankar Shrimali, Bhavesh Modi, Bharti Rajani, Tejas Shah, Ankit Hinsu, Pritesh Sabara, Apurvasinh Puvar, Janvi Raval, Zarna Patel, Monika Gandhi, Pinal Trivedi, Maharshi Pandya, Nidhi Patel, Nitin Savaliya, Raghawendra Kumar, Dinesh Kumar, Zuber Saiyed, Komal Patel, Labdhi Pandya, Snehal Bagatharia, Seema Bhatt, Priyanka P Vatsa, R D Dixit, A M Kadri, Harsh Bakshi, Chaitanya Joshi, Madhvi Joshi
EPI_ISL_467031	GMERS Medical College and Hospital, Gandhinagar	Gujarat Biotechnology Research Centre	Bhavesh Modi, Bharti Rajani, Tejas Shah, Ankit Hinsu, Pritesh Sabara, Apurvasinh Puvar, Janvi Raval, Zarna Patel, Monika Gandhi, Pinal Trivedi, Maharshi Pandya, Nidhi Patel, Nitin Savaliya, Raghawendra Kumar, Dinesh Kumar, Zuber Saiyed, Komal Patel, Labdhi Pandya, Snehal Bagatharia, Seema Bhatt, Gaurishankar Shrimali, Pooja P Doshi, R D Dixit, A M Kadri, Harsh Bakshi, Chaitanya Joshi, Madhvi Joshi
EPI_ISL_467032	GMERS Medical College and Hospital, Gandhinagar	Gujarat Biotechnology Research Centre	Bharti Rajani, Tejas Shah, Ankit Hinsu, Pritesh Sabara, Apurvasinh Puvar, Janvi Raval, Zarna Patel, Monika Gandhi, Pinal Trivedi, Maharshi Pandya, Nidhi Patel, Nitin Savaliya, Raghawendra Kumar, Dinesh Kumar, Zuber Saiyed, Komal Patel, Labdhi Pandya, Snehal Bagatharia, Seema Bhatt, Gaurishankar Shrimali, Bhavesh Modi, Akanksha Verma, R D Dixit, A M Kadri, Harsh Bakshi, Chaitanya Joshi, Madhvi Joshi
EPI_ISL_467033	GMERS Medical College and Hospital, Gandhinagar	Gujarat Biotechnology Research Centre	Tejas Shah, Ankit Hinsu, Pritesh Sabara, Apurvasinh Puvar, Janvi Raval, Zarna Patel, Monika Gandhi, Pinal Trivedi, Maharshi Pandya, Nidhi Patel, Nitin Savaliya, Raghawendra Kumar, Dinesh Kumar, Zuber Saiyed, Komal Patel, Labdhi Pandya, Snehal Bagatharia, Seema Bhatt, Gaurishankar Shrimali, Bhavesh Modi, Bharti Rajani, Priti Pandita, R D Dixit, A M Kadri, Harsh Bakshi, Chaitanya Joshi, Madhvi Joshi
EPI_ISL_467034	GMERS Medical College and Hospital, Gandhinagar	Gujarat Biotechnology Research Centre	Ankit Hinsu, Pritesh Sabara, Apurvasinh Puvar, Janvi Raval, Zarna Patel, Monika Gandhi, Pinal Trivedi, Maharshi Pandya, Nidhi Patel, Nitin Savaliya, Raghawendra Kumar, Dinesh Kumar, Zuber Saiyed, Komal Patel, Labdhi Pandya, Snehal Bagatharia, Seema Bhatt, Gaurishankar Shrimali, Bhavesh Modi, Bharti Rajani, Tejas Shah, Pragya Sharma, R D Dixit, A M Kadri, Harsh Bakshi, Chaitanya Joshi, Madhvi Joshi
EPI_ISL_467035	GMERS Medical College and Hospital, Gandhinagar	Gujarat Biotechnology Research Centre	Pritesh Sabara, Apurvasinh Puvar, Janvi Raval, Zarna Patel, Monika Gandhi, Pinal Trivedi, Maharshi Pandya, Nidhi Patel, Nitin Savaliya, Raghawendra Kumar, Dinesh Kumar, Zuber Saiyed, Komal Patel, Labdhi Pandya, Snehal Bagatharia, Seema Bhatt, Gaurishankar Shrimali, Bhavesh Modi, Bharti Rajani, Tejas Shah, Ankit Hinsu, Neha Rajpara, R D Dixit, A M Kadri, Harsh Bakshi, Chaitanya Joshi, Madhvi Joshi

EPI_ISL_467036	GMERS Medical College and Hospital, Gandhinagar	Gujarat Biotechnology Research Centre	Apurvasinh Puvar, Janvi Raval, Zarna Patel, Monika Gandhi, Pinal Trivedi, Maharshi Pandya, Nidhi Patel, Nitin Savaliya, Raghawendra Kumar, Dinesh Kumar, Zuber Saiyed, Komal Patel, Labdhi Pandya, Snehal Bagatharia, Seema Bhatt, Gaurishankar Shrimali, Bhavesh Modi, Bharti Rajani, Tejas Shah, Ankit Hinsu, Pritesh Sabara, Afzal Ansari, R D Dixit, A M Kadri, Harsh Bakshi, Chaitanya Joshi, Madhvi Joshi
EPI_ISL_467037	GMERS Medical College and Hospital, Gandhinagar	Gujarat Biotechnology Research Centre	Janvi Raval, Zarna Patel, Monika Gandhi, Pinal Trivedi, Maharshi Pandya, Nidhi Patel, Nitin Savaliya, Raghawendra Kumar, Dinesh Kumar, Zuber Saiyed, Komal Patel, Labdhi Pandya, Snehal Bagatharia, Seema Bhatt, Gaurishankar Shrimali, Bhavesh Modi, Bharti Rajani, Tejas Shah, Ankit Hinsu, Pritesh Sabara, Apurvasinh Puvar, Fenil Patel, R D Dixit, A M Kadri, Harsh Bakshi, Chaitanya Joshi, Madhvi Joshi
EPI_ISL_467038	GMERS Medical College and Hospital, Gandhinagar	Gujarat Biotechnology Research Centre	Zarna Patel, Monika Gandhi, Pinal Trivedi, Maharshi Pandya, Nidhi Patel, Nitin Savaliya, Raghawendra Kumar, Dinesh Kumar, Zuber Saiyed, Komal Patel, Labdhi Pandya, Snehal Bagatharia, Seema Bhatt, Gaurishankar Shrimali, Bhavesh Modi, Bharti Rajani, Tejas Shah, Ankit Hinsu, Pritesh Sabara, Apurvasinh Puvar, Janvi Raval, Neelam Nathani, R D Dixit, A M Kadri, Harsh Bakshi, Chaitanya Joshi, Madhvi Joshi
EPI_ISL_467039	Government Medical College, Vadodara	Gujarat Biotechnology Research Centre	Meenakshi Shah, Neena Doshi, Varsha Godbole, Tejas Shah, Ankit Hinsu, Pritesh Sabara, Apurvasinh Puvar, Janvi Raval, Zarna Patel, Monika Gandhi, Pinal Trivedi, Maharshi Pandya, Nidhi Patel, Nitin Savaliya, Raghawendra Kumar, Dinesh Kumar, Zuber Saiyed, Komal Patel, Labdhi Pandya, Snehal Bagatharia, Armi Chaudhari, R D Dixit, A M Kadri, Harsh Bakshi, Chaitanya Joshi, Madhvi Joshi,
EPI_ISL_467040	Government Medical College, Vadodara	Gujarat Biotechnology Research Centre	Neena Doshi, Varsha Godbole, Tejas Shah, Ankit Hinsu, Pritesh Sabara, Apurvasinh Puvar, Janvi Raval, Zarna Patel, Monika Gandhi, Pinal Trivedi, Maharshi Pandya, Nidhi Patel, Nitin Savaliya, Raghawendra Kumar, Dinesh Kumar, Zuber Saiyed, Komal Patel, Labdhi Pandya, Snehal Bagatharia, Meenakshi Shah, Bhavya Jindal, R D Dixit, A M Kadri, Harsh Bakshi, Chaitanya Joshi, Madhvi Joshi,
EPI_ISL_467041	B.J. Medical College and Civil hospital	Gujarat Biotechnology Research Centre	Monika Gandhi, Pinal Trivedi, Maharshi Pandya, Nidhi Patel, Nitin Savaliya, Raghawendra Kumar, Dinesh Kumar, Zuber Saiyed, Komal Patel, Labdhi Pandya, Snehal Bagatharia, Pranay Shah, Kamlesh J Upadhyay, Nirav Mungalpara, Tejas Shah, Ankit Hinsu, Pritesh Sabara, Apurvasinh Puvar, Janvi Raval, Zarna Patel, Priyanka P Vatsa, R D Dixit, A M Kadri, Harsh Bakshi, Chaitanya Joshi, Madhvi Joshi,
EPI_ISL_467042	B.J. Medical College and Civil hospital	Gujarat Biotechnology Research Centre	Pinal Trivedi, Maharshi Pandya, Nidhi Patel, Nitin Savaliya, Raghawendra Kumar, Dinesh Kumar, Zuber Saiyed, Komal Patel, Labdhi Pandya, Snehal Bagatharia, Pranay Shah, Kamlesh J Upadhyay, Nirav Mungalpara, Tejas Shah, Ankit Hinsu, Pritesh Sabara, Apurvasinh Puvar, Janvi Raval, Zarna Patel, Monika Gandhi, Pooja P Doshi, R D Dixit, A M Kadri, Harsh Bakshi, Chaitanya Joshi, Madhvi Joshi,
EPI_ISL_467043	B.J. Medical College and Civil hospital	Gujarat Biotechnology Research Centre	Maharshi Pandya, Nidhi Patel, Nitin Savaliya, Raghawendra Kumar, Dinesh Kumar, Zuber Saiyed, Komal Patel, Labdhi Pandya, Snehal Bagatharia, Pranay Shah, Kamlesh J Upadhyay, Nirav Mungalpara, Tejas Shah, Ankit Hinsu, Pritesh Sabara, Apurvasinh Puvar, Janvi Raval, Zarna Patel, Monika Gandhi, Pinal Trivedi, Akanksha Verma, R D Dixit, A M Kadri, Harsh Bakshi, Chaitanya Joshi, Madhvi Joshi,
EPI_ISL_467044	B.J. Medical College and Civil hospital	Gujarat Biotechnology Research Centre	Nidhi Patel, Nitin Savaliya, Raghawendra Kumar, Dinesh Kumar, Zuber Saiyed, Komal Patel, Labdhi Pandya, Snehal Bagatharia, Pranay Shah, Kamlesh J Upadhyay, Nirav Mungalpara, Tejas Shah, Ankit Hinsu, Pritesh Sabara, Apurvasinh Puvar, Janvi Raval, Zarna Patel, Monika Gandhi, Pinal Trivedi, Maharshi Pandya, Priti Pandita, R D Dixit, A M Kadri, Harsh Bakshi, Chaitanya Joshi, Madhvi Joshi,
EPI_ISL_467045	B.J. Medical College and Civil hospital	Gujarat Biotechnology Research Centre	Nitin Savaliya, Raghawendra Kumar, Dinesh Kumar, Zuber Saiyed, Komal Patel, Labdhi Pandya, Snehal Bagatharia, Pranay Shah, Kamlesh J Upadhyay, Nirav Mungalpara, Tejas Shah, Ankit Hinsu, Pritesh Sabara, Apurvasinh Puvar, Janvi Raval, Zarna Patel, Monika Gandhi, Pinal Trivedi, Maharshi Pandya, Nidhi Patel, Pragya Sharma, R D Dixit, A M Kadri, Harsh Bakshi, Chaitanya Joshi, Madhvi Joshi,
EPI_ISL_467046	B.J. Medical College and Civil hospital	Gujarat Biotechnology Research Centre	Raghawendra Kumar, Dinesh Kumar, Zuber Saiyed, Komal Patel, Labdhi Pandya, Snehal Bagatharia, Pranay Shah, Kamlesh J Upadhyay, Nirav Mungalpara, Tejas Shah, Ankit Hinsu, Pritesh Sabara, Apurvasinh Puvar, Janvi Raval, Zarna Patel, Monika Gandhi, Pinal Trivedi, Maharshi Pandya, Nidhi Patel, Nitin Savaliya, Neha Rajpara, R D Dixit, A M Kadri, Harsh Bakshi, Chaitanya Joshi, Madhvi Joshi,
EPI_ISL_467047	B.J. Medical College and Civil hospital	Gujarat Biotechnology Research Centre	Dinesh Kumar, Zuber Saiyed, Komal Patel, Labdhi Pandya, Snehal Bagatharia, Pranay Shah, Kamlesh J Upadhyay, Nirav Mungalpara, Tejas Shah, Ankit Hinsu, Pritesh Sabara, Apurvasinh Puvar, Janvi Raval, Zarna Patel, Monika Gandhi, Pinal Trivedi, Maharshi Pandya, Nidhi Patel, Nitin Savaliya, Raghawendra Kumar, Afzal Ansari, R D Dixit, A M Kadri, Harsh Bakshi, Chaitanya Joshi, Madhvi Joshi,
EPI_ISL_467048	B.J. Medical College and Civil hospital	Gujarat Biotechnology Research Centre	Zuber Saiyed, Komal Patel, Labdhi Pandya, Snehal Bagatharia, Pranay Shah, Kamlesh J Upadhyay, Nirav Mungalpara, Tejas Shah, Ankit Hinsu, Pritesh Sabara, Apurvasinh Puvar, Janvi Raval, Zarna Patel, Monika Gandhi, Pinal Trivedi, Maharshi Pandya, Nidhi Patel, Nitin Savaliya, Raghawendra Kumar, Dinesh Kumar, Fenil Patel, R D Dixit, A M Kadri, Harsh Bakshi, Chaitanya Joshi, Madhvi Joshi,
EPI_ISL_467049	B.J. Medical College and Civil hospital	Gujarat Biotechnology Research Centre	Komal Patel, Labdhi Pandya, Snehal Bagatharia, Pranay Shah, Kamlesh J Upadhyay, Nirav Mungalpara, Tejas Shah, Ankit Hinsu, Pritesh Sabara, Apurvasinh Puvar, Janvi Raval, Zarna Patel, Monika Gandhi, Pinal Trivedi, Maharshi Pandya, Nidhi Patel, Nitin Savaliya, Raghawendra Kumar, Dinesh Kumar, Zuber Saiyed, Neelam Nathani, R D Dixit, A M Kadri, Harsh Bakshi, Chaitanya Joshi, Madhvi Joshi,
EPI_ISL_467050	B.J. Medical College and Civil hospital	Gujarat Biotechnology Research Centre	Labdhi Pandya, Snehal Bagatharia, Pranay Shah, Kamlesh J Upadhyay, Nirav Mungalpara, Tejas Shah, Ankit Hinsu, Pritesh Sabara, Apurvasinh Puvar, Janvi Raval, Zarna Patel, Monika Gandhi, Pinal Trivedi, Maharshi Pandya, Nidhi Patel, Nitin Savaliya, Raghawendra Kumar, Dinesh Kumar, Zuber Saiyed, Komal Patel, Armi Chaudhari, R D Dixit, A M Kadri, Harsh Bakshi, Chaitanya Joshi, Madhvi Joshi,
EPI_ISL_467051	B.J. Medical College and Civil hospital	Gujarat Biotechnology Research Centre	Snehal Bagatharia, Pranay Shah, Kamlesh J Upadhyay, Nirav Mungalpara, Tejas Shah, Ankit Hinsu, Pritesh Sabara, Apurvasinh Puvar, Janvi Raval, Zarna Patel, Monika Gandhi, Pinal Trivedi, Maharshi Pandya, Nidhi Patel, Nitin Savaliya, Raghawendra Kumar, Dinesh Kumar, Zuber Saiyed, Komal Patel, Labdhi Pandya, Bhavya Jindal, R D Dixit, A M Kadri, Harsh Bakshi, Chaitanya Joshi, Madhvi Joshi,
EPI_ISL_467052	B.J. Medical College and Civil hospital	Gujarat Biotechnology Research Centre	Pranay Shah, Kamlesh J Upadhyay, Nirav Mungalpara, Tejas Shah, Ankit Hinsu, Pritesh Sabara, Apurvasinh Puvar, Janvi Raval, Zarna Patel, Monika Gandhi, Pinal Trivedi, Maharshi Pandya, Nidhi Patel, Nitin Savaliya, Raghawendra Kumar, Dinesh Kumar, Zuber Saiyed, Komal Patel, Labdhi Pandya, Snehal Bagatharia, Priyanka P Vatsa, R D Dixit, A M Kadri, Harsh Bakshi, Chaitanya Joshi, Madhvi Joshi,
EPI_ISL_467053	B.J. Medical College and Civil hospital	Gujarat Biotechnology Research Centre	Kamlesh J Upadhyay, Nirav Mungalpara, Tejas Shah, Ankit Hinsu, Pritesh Sabara, Apurvasinh Puvar, Janvi Raval, Zarna Patel, Monika Gandhi, Pinal Trivedi, Maharshi Pandya, Nidhi Patel, Nitin Savaliya, Raghawendra Kumar, Dinesh Kumar, Zuber Saiyed, Komal Patel, Labdhi Pandya, Snehal Bagatharia, Pranay Shah, Pooja P Doshi, R D Dixit, A M Kadri, Harsh Bakshi, Chaitanya Joshi, Madhvi Joshi,
EPI_ISL_467054	B.J. Medical College and Civil hospital	Gujarat Biotechnology Research Centre	Nirav Mungalpara, Tejas Shah, Ankit Hinsu, Pritesh Sabara, Apurvasinh Puvar, Janvi Raval, Zarna Patel, Monika Gandhi, Pinal Trivedi, Maharshi Pandya, Nidhi Patel, Nitin Savaliya, Raghawendra Kumar, Dinesh Kumar, Zuber Saiyed, Komal Patel, Labdhi Pandya, Snehal Bagatharia, Pranay Shah, Kamlesh J Upadhyay, Akanksha Verma, R D Dixit, A M Kadri, Harsh Bakshi, Chaitanya Joshi, Madhvi Joshi,
EPI_ISL_468158	[Romania, Bucharest] National Institute for Infectious Diseases "Prof. Dr. Matei Bal"	[Romania, Bucharest] National Institute for Infectious Diseases "Prof. Dr. Matei Bal"	Leontina Banica, Marius Cotic, Corina Casangiu, Marius Surleac, Simona Paraschiv
EPI_ISL_469024	B.J. Medical College and Civil hospital	Gujarat Biotechnology Research Centre	Tejas Shah, Ankit Hinsu, Pritesh Sabara, Apurvasinh Puvar, Janvi Raval, Zarna Patel, Monika Gandhi, Pinal Trivedi, Maharshi Pandya, Nidhi Patel, Nitin Savaliya, Raghawendra Kumar, Dinesh Kumar, Zuber Saiyed, Komal Patel, Labdhi Pandya, Snehal Bagatharia, Pranay Shah, Kamlesh J Upadhyay, Nirav Mungalpara, Priti Pandita, R D Dixit, A M Kadri, Harsh Bakshi, Chaitanya Joshi, Madhvi Joshi
EPI_ISL_469025	B.J. Medical College and Civil hospital	Gujarat Biotechnology Research Centre	Ankit Hinsu, Pritesh Sabara, Apurvasinh Puvar, Janvi Raval, Zarna Patel, Monika Gandhi, Pinal Trivedi, Maharshi Pandya, Nidhi Patel, Nitin Savaliya, Raghawendra Kumar, Dinesh Kumar, Zuber Saiyed, Komal Patel, Labdhi Pandya, Snehal Bagatharia, Pranay Shah, Kamlesh J Upadhyay, Nirav Mungalpara, Tejas Shah, Pragya Sharma, R D Dixit, A M Kadri, Harsh Bakshi, Chaitanya Joshi, Madhvi Joshi
EPI_ISL_469026	B.J. Medical College and Civil hospital	Gujarat Biotechnology Research Centre	Pritesh Sabara, Apurvasinh Puvar, Janvi Raval, Zarna Patel, Monika Gandhi, Pinal Trivedi, Maharshi Pandya, Nidhi Patel, Nitin Savaliya, Raghawendra Kumar, Dinesh Kumar, Zuber Saiyed, Komal Patel, Labdhi Pandya, Snehal Bagatharia, Pranay Shah, Kamlesh J Upadhyay, Nirav Mungalpara, Tejas Shah, Ankit Hinsu, Neha Rajpara, R D Dixit, A M Kadri, Harsh Bakshi, Chaitanya Joshi, Madhvi Joshi
EPI_ISL_469027	B.J. Medical College and Civil hospital	Gujarat Biotechnology Research Centre	Apurvasinh Puvar, Janvi Raval, Zarna Patel, Monika Gandhi, Pinal Trivedi, Maharshi Pandya, Nidhi Patel, Nitin Savaliya, Raghawendra Kumar, Dinesh Kumar, Zuber Saiyed, Komal Patel, Labdhi Pandya, Snehal Bagatharia, Pranay Shah, Kamlesh J Upadhyay, Nirav Mungalpara, Tejas Shah, Ankit Hinsu, Pritesh Sabara, Afzal Ansari, R D Dixit, A M Kadri, Harsh Bakshi, Chaitanya Joshi, Madhvi Joshi
EPI_ISL_469028	B.J. Medical College and Civil hospital	Gujarat Biotechnology Research Centre	Janvi Raval, Zarna Patel, Monika Gandhi, Pinal Trivedi, Maharshi Pandya, Nidhi Patel, Nitin Savaliya, Raghawendra Kumar, Dinesh Kumar, Zuber Saiyed, Komal Patel, Labdhi Pandya, Snehal Bagatharia, Pranay Shah, Kamlesh J Upadhyay, Nirav Mungalpara, Tejas Shah, Ankit Hinsu, Pritesh Sabara, Apurvasinh Puvar, Fenil Patel, R D Dixit, A M Kadri, Harsh Bakshi, Chaitanya Joshi, Madhvi Joshi
EPI_ISL_469029	Government Medical College, Vadodara	Gujarat Biotechnology Research Centre	Zarna Patel, Monika Gandhi, Pinal Trivedi, Maharshi Pandya, Nidhi Patel, Nitin Savaliya, Raghawendra Kumar, Dinesh Kumar, Zuber Saiyed, Komal Patel,

			Labdhi Pandya, Snehal Bagatharia, Meenakshi Shah, Neena Doshi, Varsha Godbole, Tejas Shah, Ankit Hinsu, Pritesh Sabara, Apurvasinh Puvar, Janvi Raval, Neelam Nathani, R D Dixit, A M Kadri, Harsh Bakshi, Chaitanya Joshi, Madhvi Joshi
EPI_ISL_469030	Government Medical College, Vadodara	Gujarat Biotechnology Research Centre	Monika Gandhi, Pinal Trivedi, Maharshi Pandya, Nidhi Patel, Nitin Savaliya, Raghawendra Kumar, Dinesh Kumar, Zuber Saiyed, Komal Patel, Labdhi Pandya, Snehal Bagatharia, Meenakshi Shah, Neena Doshi, Varsha Godbole, Tejas Shah, Ankit Hinsu, Pritesh Sabara, Apurvasinh Puvar, Janvi Raval, Zarna Patel, Armi Chaudhari, R D Dixit, A M Kadri, Harsh Bakshi, Chaitanya Joshi, Madhvi Joshi
EPI_ISL_469031	Government Medical College, Vadodara	Gujarat Biotechnology Research Centre	Pinal Trivedi, Maharshi Pandya, Nidhi Patel, Nitin Savaliya, Raghawendra Kumar, Dinesh Kumar, Zuber Saiyed, Komal Patel, Labdhi Pandya, Snehal Bagatharia, Meenakshi Shah, Neena Doshi, Varsha Godbole, Tejas Shah, Ankit Hinsu, Pritesh Sabara, Apurvasinh Puvar, Janvi Raval, Zarna Patel, Monika Gandhi, Bhavya Jindal, R D Dixit, A M Kadri, Harsh Bakshi, Chaitanya Joshi, Madhvi Joshi
EPI_ISL_469032	Government Medical College, Vadodara	Gujarat Biotechnology Research Centre	Maharshi Pandya, Nidhi Patel, Nitin Savaliya, Raghawendra Kumar, Dinesh Kumar, Zuber Saiyed, Komal Patel, Labdhi Pandya, Snehal Bagatharia, Meenakshi Shah, Neena Doshi, Varsha Godbole, Tejas Shah, Ankit Hinsu, Pritesh Sabara, Apurvasinh Puvar, Janvi Raval, Zarna Patel, Monika Gandhi, Pinal Trivedi, Pragya Sharma, R D Dixit, A M Kadri, Harsh Bakshi, Chaitanya Joshi, Madhvi Joshi
EPI_ISL_469033	Government Medical College, Vadodara	Gujarat Biotechnology Research Centre	Nidhi Patel, Nitin Savaliya, Raghawendra Kumar, Dinesh Kumar, Zuber Saiyed, Komal Patel, Labdhi Pandya, Snehal Bagatharia, Meenakshi Shah, Neena Doshi, Varsha Godbole, Tejas Shah, Ankit Hinsu, Pritesh Sabara, Apurvasinh Puvar, Janvi Raval, Zarna Patel, Monika Gandhi, Pinal Trivedi, Maharshi Pandya, Priyanka P Vatsa, R D Dixit, A M Kadri, Harsh Bakshi, Chaitanya Joshi, Madhvi Joshi
EPI_ISL_469034	Government Medical College, Vadodara	Gujarat Biotechnology Research Centre	Nitin Savaliya, Raghawendra Kumar, Dinesh Kumar, Zuber Saiyed, Komal Patel, Labdhi Pandya, Snehal Bagatharia, Meenakshi Shah, Neena Doshi, Varsha Godbole, Tejas Shah, Ankit Hinsu, Pritesh Sabara, Apurvasinh Puvar, Janvi Raval, Zarna Patel, Monika Gandhi, Pinal Trivedi, Maharshi Pandya, Nidhi Patel, Pooja P Doshi, R D Dixit, A M Kadri, Harsh Bakshi, Chaitanya Joshi, Madhvi Joshi
EPI_ISL_469035	Government Medical College, Vadodara	Gujarat Biotechnology Research Centre	Raghawendra Kumar, Dinesh Kumar, Zuber Saiyed, Komal Patel, Labdhi Pandya, Snehal Bagatharia, Meenakshi Shah, Neena Doshi, Varsha Godbole, Tejas Shah, Ankit Hinsu, Pritesh Sabara, Apurvasinh Puvar, Janvi Raval, Zarna Patel, Monika Gandhi, Pinal Trivedi, Maharshi Pandya, Nidhi Patel, Nitin Savaliya, Akanksha Verma, R D Dixit, A M Kadri, Harsh Bakshi, Chaitanya Joshi, Madhvi Joshi
EPI_ISL_469036	Government Medical College, Vadodara	Gujarat Biotechnology Research Centre	Dinesh Kumar, Zuber Saiyed, Komal Patel, Labdhi Pandya, Snehal Bagatharia, Meenakshi Shah, Neena Doshi, Varsha Godbole, Tejas Shah, Ankit Hinsu, Pritesh Sabara, Apurvasinh Puvar, Janvi Raval, Zarna Patel, Monika Gandhi, Pinal Trivedi, Maharshi Pandya, Nidhi Patel, Nitin Savaliya, Raghawendra Kumar, Priti Pandita, R D Dixit, A M Kadri, Harsh Bakshi, Chaitanya Joshi, Madhvi Joshi
EPI_ISL_469037	GMERS Medical College & Hospital	Gujarat Biotechnology Research Centre	Zuber Saiyed, Komal Patel, Labdhi Pandya, Snehal Bagatharia, Meenakshi Shah, Neena Doshi, Varsha Godbole, Tejas Shah, Ankit Hinsu, Pritesh Sabara, Apurvasinh Puvar, Janvi Raval, Zarna Patel, Monika Gandhi, Pinal Trivedi, Maharshi Pandya, Nidhi Patel, Nitin Savaliya, Raghawendra Kumar, Dinesh Kumar, Pragya Sharma, R D Dixit, A M Kadri, Harsh Bakshi, Chaitanya Joshi, Madhvi Joshi
EPI_ISL_469038	GMERS Medical College & Hospital	Gujarat Biotechnology Research Centre	Komal Patel, Labdhi Pandya, Snehal Bagatharia, Meenakshi Shah, Neena Doshi, Varsha Godbole, Tejas Shah, Ankit Hinsu, Pritesh Sabara, Apurvasinh Puvar, Janvi Raval, Zarna Patel, Monika Gandhi, Pinal Trivedi, Maharshi Pandya, Nidhi Patel, Nitin Savaliya, Raghawendra Kumar, Dinesh Kumar, Zuber Saiyed, Neha Rajpara, R D Dixit, A M Kadri, Harsh Bakshi, Chaitanya Joshi, Madhvi Joshi
EPI_ISL_469039	GMERS Medical College & Hospital	Gujarat Biotechnology Research Centre	Labdhi Pandya, Snehal Bagatharia, Meenakshi Shah, Neena Doshi, Varsha Godbole, Tejas Shah, Ankit Hinsu, Pritesh Sabara, Apurvasinh Puvar, Janvi Raval, Zarna Patel, Monika Gandhi, Pinal Trivedi, Maharshi Pandya, Nidhi Patel, Nitin Savaliya, Raghawendra Kumar, Dinesh Kumar, Zuber Saiyed, Komal Patel, Afzal Ansari, R D Dixit, A M Kadri, Harsh Bakshi, Chaitanya Joshi, Madhvi Joshi
EPI_ISL_469040	GMERS Medical College & Hospital	Gujarat Biotechnology Research Centre	Snehal Bagatharia, Meenakshi Shah, Neena Doshi, Varsha Godbole, Tejas Shah, Ankit Hinsu, Pritesh Sabara, Apurvasinh Puvar, Janvi Raval, Zarna Patel, Monika Gandhi, Pinal Trivedi, Maharshi Pandya, Nidhi Patel, Nitin Savaliya, Raghawendra Kumar, Dinesh Kumar, Zuber Saiyed, Komal Patel, Labdhi Pandya, Fenil Patel, R D Dixit, A M Kadri, Harsh Bakshi, Chaitanya Joshi, Madhvi Joshi
EPI_ISL_469041	GMERS Medical College & Hospital	Gujarat Biotechnology Research Centre	Meenakshi Shah, Neena Doshi, Varsha Godbole, Tejas Shah, Ankit Hinsu, Pritesh Sabara, Apurvasinh Puvar, Janvi Raval, Zarna Patel, Monika Gandhi, Pinal Trivedi, Maharshi Pandya, Nidhi Patel, Nitin Savaliya, Raghawendra Kumar, Dinesh Kumar, Zuber Saiyed, Komal Patel, Labdhi Pandya, Snehal Bagatharia, Neelam Nathani, R D Dixit, A M Kadri, Harsh Bakshi, Chaitanya Joshi, Madhvi Joshi
EPI_ISL_469042	GMERS Medical College & Hospital	Gujarat Biotechnology Research Centre	Neena Doshi, Varsha Godbole, Tejas Shah, Ankit Hinsu, Pritesh Sabara, Apurvasinh Puvar, Janvi Raval, Zarna Patel, Monika Gandhi, Pinal Trivedi, Maharshi Pandya, Nidhi Patel, Nitin Savaliya, Raghawendra Kumar, Dinesh Kumar, Zuber Saiyed, Komal Patel, Labdhi Pandya, Snehal Bagatharia, Meenakshi Shah, Armi Chaudhari, R D Dixit, A M Kadri, Harsh Bakshi, Chaitanya Joshi, Madhvi Joshi
EPI_ISL_469043	Dr. N. D. Desai Medical College & Hospital	Gujarat Biotechnology Research Centre	J G Buch, Jigar Gusani, Supreet Prabhu, Tejas Shah, Ankit Hinsu, Pritesh Sabara, Apurvasinh Puvar, Janvi Raval, Zarna Patel, Monika Gandhi, Pinal Trivedi, Maharshi Pandya, Nidhi Patel, Nitin Savaliya, Raghawendra Kumar, Dinesh Kumar, Zuber Saiyed, Komal Patel, Labdhi Pandya, Snehal Bagatharia, Bhavya Jindal, R D Dixit, A M Kadri, Harsh Bakshi, Chaitanya Joshi, Madhvi Joshi
EPI_ISL_469044	Dr. N. D. Desai Medical College & Hospital	Gujarat Biotechnology Research Centre	Jigar Gusani, Supreet Prabhu, Tejas Shah, Ankit Hinsu, Pritesh Sabara, Apurvasinh Puvar, Janvi Raval, Zarna Patel, Monika Gandhi, Pinal Trivedi, Maharshi Pandya, Nidhi Patel, Nitin Savaliya, Raghawendra Kumar, Dinesh Kumar, Zuber Saiyed, Komal Patel, Labdhi Pandya, Snehal Bagatharia, J G Buch, Neha Rajpara, R D Dixit, A M Kadri, Harsh Bakshi, Chaitanya Joshi, Madhvi Joshi
EPI_ISL_469045	Dr. N. D. Desai Medical College & Hospital	Gujarat Biotechnology Research Centre	Supreet Prabhu, Tejas Shah, Ankit Hinsu, Pritesh Sabara, Apurvasinh Puvar, Janvi Raval, Zarna Patel, Monika Gandhi, Pinal Trivedi, Maharshi Pandya, Nidhi Patel, Nitin Savaliya, Raghawendra Kumar, Dinesh Kumar, Zuber Saiyed, Komal Patel, Labdhi Pandya, Snehal Bagatharia, J G Buch, Jigar Gusani, Priyanka P Vatsa, R D Dixit, A M Kadri, Harsh Bakshi, Chaitanya Joshi, Madhvi Joshi
EPI_ISL_469046	Dr. N. D. Desai Medical College & Hospital	Gujarat Biotechnology Research Centre	Tejas Shah, Ankit Hinsu, Pritesh Sabara, Apurvasinh Puvar, Janvi Raval, Zarna Patel, Monika Gandhi, Pinal Trivedi, Maharshi Pandya, Nidhi Patel, Nitin Savaliya, Raghawendra Kumar, Dinesh Kumar, Zuber Saiyed, Komal Patel, Labdhi Pandya, Snehal Bagatharia, J G Buch, Jigar Gusani, Supreet Prabhu, Pooja P Doshi, R D Dixit, A M Kadri, Harsh Bakshi, Chaitanya Joshi, Madhvi Joshi
EPI_ISL_469047	Dr. N. D. Desai Medical College & Hospital	Gujarat Biotechnology Research Centre	Ankit Hinsu, Pritesh Sabara, Apurvasinh Puvar, Janvi Raval, Zarna Patel, Monika Gandhi, Pinal Trivedi, Maharshi Pandya, Nidhi Patel, Nitin Savaliya, Raghawendra Kumar, Dinesh Kumar, Zuber Saiyed, Komal Patel, Labdhi Pandya, Snehal Bagatharia, J G Buch, Jigar Gusani, Supreet Prabhu, Tejas Shah, Akanksha Verma, R D Dixit, A M Kadri, Harsh Bakshi, Chaitanya Joshi, Madhvi Joshi
EPI_ISL_469048	Banas Medical College and Research Institute	Gujarat Biotechnology Research Centre	Radhika Khara, Sunil R Joshi, Viren s Doshi, Zarna Patel, Monika Gandhi, Pinal Trivedi, Maharshi Pandya, Nidhi Patel, Nitin Savaliya, Raghawendra Kumar, Dinesh Kumar, Zuber Saiyed, Komal Patel, Labdhi Pandya, Snehal Bagatharia, Tejas Shah, Ankit Hinsu, Pritesh Sabara, Apurvasinh Puvar, Janvi Raval, Priti Pandita, R D Dixit, A M Kadri, Harsh Bakshi, Chaitanya Joshi, Madhvi Joshi
EPI_ISL_471269, EPI_ISL_471270, EPI_ISL_471271	Hospital Oncológico Solca Núcleo de Quito	Institute of Microbiology, Universidad San Francisco de Quito	Sully Márquez, Belén Prado-Vivar, Juan José Guadalupe, Bernardo Gutiérrez, Marcos Di Stefano, Grace Salazar, Verónica Barragán, Patricio Rojas-Silva, Gabriel Trueba, Michelle Grunauer, Paúl Cárdenas
EPI_ISL_471416, EPI_ISL_471417, EPI_ISL_471419, EPI_ISL_471420, EPI_ISL_471422, EPI_ISL_471423, EPI_ISL_471424	Laboratory for Respiratory Viruses, National Influenza Centre, Cantacuzino National Military-Medical Institute for Research and Development	Cantacuzino Institute	Luiza Ustea, Nicoleta Paraschiv, Tim Durfee, Mihaela Lazar
EPI_ISL_475026	Banas Medical College and Research Institute	Gujarat Biotechnology Research Centre	Sunil R Joshi, Viren s Doshi, Pritesh Sabara, Apurvasinh Puvar, Janvi Raval, Zarna Patel, Monika Gandhi, Pinal Trivedi, Maharshi Pandya, Nidhi Patel, Nitin Savaliya, Raghawendra Kumar, Dinesh Kumar, Zuber Saiyed, Komal Patel, Labdhi Pandya, Snehal Bagatharia, Radhika Khara, Neha Rajpara, R D Dixit, A M Kadri, Harsh Bakshi, Chaitanya Joshi, Madhvi Joshi
EPI_ISL_475027	Banas Medical College and Research Institute	Gujarat Biotechnology Research Centre	Viren s Doshi, Pritesh Sabara, Apurvasinh Puvar, Janvi Raval, Zarna Patel, Monika Gandhi, Pinal Trivedi, Maharshi Pandya, Nidhi Patel, Nitin Savaliya, Raghawendra Kumar, Dinesh Kumar, Zuber Saiyed, Komal Patel, Labdhi Pandya, Snehal Bagatharia, Radhika Khara, Sunil R Joshi, Afzal Ansari, R D Dixit, A M Kadri, Harsh Bakshi, Chaitanya Joshi, Madhvi Joshi
EPI_ISL_475028	Banas Medical College and Research Institute	Gujarat Biotechnology Research Centre	Pritesh Sabara, Apurvasinh Puvar, Janvi Raval, Zarna Patel, Monika Gandhi, Pinal Trivedi, Maharshi Pandya, Nidhi Patel, Nitin Savaliya, Raghawendra Kumar, Dinesh Kumar, Zuber Saiyed, Komal Patel, Labdhi Pandya, Snehal Bagatharia, Radhika Khara, Sunil R Joshi, Viren s Doshi, Fenil Patel, R D Dixit, A M Kadri, Harsh Bakshi, Chaitanya Joshi, Madhvi Joshi
EPI_ISL_475029	Banas Medical College and Research Institute	Gujarat Biotechnology Research Centre	Apurvasinh Puvar, Janvi Raval, Zarna Patel, Monika Gandhi, Pinal Trivedi, Maharshi Pandya, Nidhi Patel, Nitin Savaliya, Raghawendra Kumar, Dinesh

			Kumar, Zuber Saiyed, Komal Patel, Labdhi Pandya, Snehal Bagatharia, Radhika Khara, Sunil R Joshi, Viren s Doshi, Pritesh Sabara, Neelam Nathani, R D Dixit, A M Kadri, Harsh Bakshi, Chaitanya Joshi, Madhvi Joshi
EPI_ISL_475030	Department of MicroBiology, Government Medical College, Surat	Gujarat Biotechnology Research Centre	Janvi Raval, Zarna Patel, Monika Gandhi, Pinal Trivedi, Maharshi Pandya, Nidhi Patel, Nitin Savaliya, Raghawendra Kumar, Dinesh Kumar, Zuber Saiyed, Komal Patel, Labdhi Pandya, Snehal Bagatharia, Naresh Chauhan, Summaiya Mullan, Amit gamit, Pritesh Sabara, Apurvasinh Puvar, Armi Chaudhari, R D Dixit, A M Kadri, Harsh Bakshi, Chaitanya Joshi, Madhvi Joshi
EPI_ISL_475031	Department of MicroBiology, Government Medical College, Surat	Gujarat Biotechnology Research Centre	Zarna Patel, Monika Gandhi, Pinal Trivedi, Maharshi Pandya, Nidhi Patel, Nitin Savaliya, Raghawendra Kumar, Dinesh Kumar, Zuber Saiyed, Komal Patel, Labdhi Pandya, Snehal Bagatharia, Naresh Chauhan, Summaiya Mullan, Amit gamit, Pritesh Sabara, Apurvasinh Puvar, Janvi Raval, Bhavya Jindal, R D Dixit, A M Kadri, Harsh Bakshi, Chaitanya Joshi, Madhvi Joshi
EPI_ISL_475032	Department of MicroBiology, Government Medical College, Surat	Gujarat Biotechnology Research Centre	Monika Gandhi, Pinal Trivedi, Maharshi Pandya, Nidhi Patel, Nitin Savaliya, Raghawendra Kumar, Dinesh Kumar, Zuber Saiyed, Komal Patel, Labdhi Pandya, Snehal Bagatharia, Naresh Chauhan, Summaiya Mullan, Amit gamit, Pritesh Sabara, Apurvasinh Puvar, Janvi Raval, Zarna Patel, Priyanka P Vatsa, R D Dixit, A M Kadri, Harsh Bakshi, Chaitanya Joshi, Madhvi Joshi
EPI_ISL_475033	Department of MicroBiology, Government Medical College, Surat	Gujarat Biotechnology Research Centre	Pinal Trivedi, Maharshi Pandya, Nidhi Patel, Nitin Savaliya, Raghawendra Kumar, Dinesh Kumar, Zuber Saiyed, Komal Patel, Labdhi Pandya, Snehal Bagatharia, Naresh Chauhan, Summaiya Mullan, Amit gamit, Pritesh Sabara, Apurvasinh Puvar, Janvi Raval, Zarna Patel, Monika Gandhi, Pooja P Doshi, R D Dixit, A M Kadri, Harsh Bakshi, Chaitanya Joshi, Madhvi Joshi
EPI_ISL_475034	Department of MicroBiology, Government Medical College, Surat	Gujarat Biotechnology Research Centre	Maharshi Pandya, Nidhi Patel, Nitin Savaliya, Raghawendra Kumar, Dinesh Kumar, Zuber Saiyed, Komal Patel, Labdhi Pandya, Snehal Bagatharia, Naresh Chauhan, Summaiya Mullan, Amit gamit, Pritesh Sabara, Apurvasinh Puvar, Janvi Raval, Zarna Patel, Monika Gandhi, Pinal Trivedi, Akanksha Verma, R D Dixit, A M Kadri, Harsh Bakshi, Chaitanya Joshi, Madhvi Joshi
EPI_ISL_475035	Department of MicroBiology, Government Medical College, Surat	Gujarat Biotechnology Research Centre	Nidhi Patel, Nitin Savaliya, Raghawendra Kumar, Dinesh Kumar, Zuber Saiyed, Komal Patel, Labdhi Pandya, Snehal Bagatharia, Naresh Chauhan, Summaiya Mullan, Amit gamit, Pritesh Sabara, Apurvasinh Puvar, Janvi Raval, Zarna Patel, Monika Gandhi, Pinal Trivedi, Maharshi Pandya, Priti Pandita, R D Dixit, A M Kadri, Harsh Bakshi, Chaitanya Joshi, Madhvi Joshi
EPI_ISL_475036	Department of MicroBiology, Government Medical College, Surat	Gujarat Biotechnology Research Centre	Nitin Savaliya, Raghawendra Kumar, Dinesh Kumar, Zuber Saiyed, Komal Patel, Labdhi Pandya, Snehal Bagatharia, Naresh Chauhan, Summaiya Mullan, Amit gamit, Pritesh Sabara, Apurvasinh Puvar, Janvi Raval, Zarna Patel, Monika Gandhi, Pinal Trivedi, Maharshi Pandya, Nidhi Patel, Pragya Sharma, R D Dixit, A M Kadri, Harsh Bakshi, Chaitanya Joshi, Madhvi Joshi
EPI_ISL_475037	Department of MicroBiology, Government Medical College, Surat	Gujarat Biotechnology Research Centre	Raghawendra Kumar, Dinesh Kumar, Zuber Saiyed, Komal Patel, Labdhi Pandya, Snehal Bagatharia, Naresh Chauhan, Summaiya Mullan, Amit gamit, Pritesh Sabara, Apurvasinh Puvar, Janvi Raval, Zarna Patel, Monika Gandhi, Pinal Trivedi, Maharshi Pandya, Nidhi Patel, Nitin Savaliya, Neha Rajpara, R D Dixit, A M Kadri, Harsh Bakshi, Chaitanya Joshi, Madhvi Joshi
EPI_ISL_475038	Department of MicroBiology, Government Medical College, Surat	Gujarat Biotechnology Research Centre	Dinesh Kumar, Zuber Saiyed, Komal Patel, Labdhi Pandya, Snehal Bagatharia, Naresh Chauhan, Summaiya Mullan, Amit gamit, Pritesh Sabara, Apurvasinh Puvar, Janvi Raval, Zarna Patel, Monika Gandhi, Pinal Trivedi, Maharshi Pandya, Nidhi Patel, Nitin Savaliya, Raghawendra Kumar, Afzal Ansari, R D Dixit, A M Kadri, Harsh Bakshi, Chaitanya Joshi, Madhvi Joshi
EPI_ISL_475039	Department of MicroBiology, Government Medical College, Surat	Gujarat Biotechnology Research Centre	Zuber Saiyed, Komal Patel, Labdhi Pandya, Snehal Bagatharia, Naresh Chauhan, Summaiya Mullan, Amit gamit, Pritesh Sabara, Apurvasinh Puvar, Janvi Raval, Zarna Patel, Monika Gandhi, Pinal Trivedi, Maharshi Pandya, Nidhi Patel, Nitin Savaliya, Raghawendra Kumar, Dinesh Kumar, Fenil Patel, R D Dixit, A M Kadri, Harsh Bakshi, Chaitanya Joshi, Madhvi Joshi
EPI_ISL_475040	Department of MicroBiology, Government Medical College, Surat	Gujarat Biotechnology Research Centre	Komal Patel, Labdhi Pandya, Snehal Bagatharia, Naresh Chauhan, Summaiya Mullan, Amit gamit, Pritesh Sabara, Apurvasinh Puvar, Janvi Raval, Zarna Patel, Monika Gandhi, Pinal Trivedi, Maharshi Pandya, Nidhi Patel, Nitin Savaliya, Raghawendra Kumar, Dinesh Kumar, Zuber Saiyed, Neelam Nathani, R D Dixit, A M Kadri, Harsh Bakshi, Chaitanya Joshi, Madhvi Joshi
EPI_ISL_475043	Department of MicroBiology, Government Medical College, Surat	Gujarat Biotechnology Research Centre	Naresh Chauhan, Summaiya Mullan, Amit gamit, Pritesh Sabara, Apurvasinh Puvar, Janvi Raval, Zarna Patel, Monika Gandhi, Pinal Trivedi, Maharshi Pandya, Nidhi Patel, Nitin Savaliya, Raghawendra Kumar, Dinesh Kumar, Zuber Saiyed, Komal Patel, Labdhi Pandya, Snehal Bagatharia, Priyanka P Vatsa, R D Dixit, A M Kadri, Harsh Bakshi, Chaitanya Joshi, Madhvi Joshi
EPI_ISL_475044	Department of MicroBiology, Government Medical College, Surat	Gujarat Biotechnology Research Centre	Summaiya Mullan, Amit gamit, Pritesh Sabara, Apurvasinh Puvar, Janvi Raval, Zarna Patel, Monika Gandhi, Pinal Trivedi, Maharshi Pandya, Nidhi Patel, Nitin Savaliya, Raghawendra Kumar, Dinesh Kumar, Zuber Saiyed, Komal Patel, Labdhi Pandya, Snehal Bagatharia, Naresh Chauhan, Pooja P Doshi, R D Dixit, A M Kadri, Harsh Bakshi, Chaitanya Joshi, Madhvi Joshi
EPI_ISL_475045	Department of MicroBiology, Government Medical College, Surat	Gujarat Biotechnology Research Centre	Amit gamit, Pritesh Sabara, Apurvasinh Puvar, Janvi Raval, Zarna Patel, Monika Gandhi, Pinal Trivedi, Maharshi Pandya, Nidhi Patel, Nitin Savaliya, Raghawendra Kumar, Dinesh Kumar, Zuber Saiyed, Komal Patel, Labdhi Pandya, Snehal Bagatharia, Naresh Chauhan, Summaiya Mullan, Akanksha Verma, R D Dixit, A M Kadri, Harsh Bakshi, Chaitanya Joshi, Madhvi Joshi
EPI_ISL_475046	Department of MicroBiology, Government Medical College, Surat	Gujarat Biotechnology Research Centre	Pritesh Sabara, Apurvasinh Puvar, Janvi Raval, Zarna Patel, Monika Gandhi, Pinal Trivedi, Maharshi Pandya, Nidhi Patel, Nitin Savaliya, Raghawendra Kumar, Dinesh Kumar, Zuber Saiyed, Komal Patel, Labdhi Pandya, Snehal Bagatharia, Naresh Chauhan, Summaiya Mullan, Amit gamit, Priti Pandita, R D Dixit, A M Kadri, Harsh Bakshi, Chaitanya Joshi, Madhvi Joshi
EPI_ISL_475047	GMERS Medical College & Hospital	Gujarat Biotechnology Research Centre	Apurvasinh Puvar, Janvi Raval, Zarna Patel, Monika Gandhi, Pinal Trivedi, Maharshi Pandya, Nidhi Patel, Nitin Savaliya, Raghawendra Kumar, Dinesh Kumar, Zuber Saiyed, Komal Patel, Labdhi Pandya, Snehal Bagatharia, Meenakshi Shah, Neena Doshi, Varsha Godbole, Pritesh Sabara, Pragya Sharma, R D Dixit, A M Kadri, Harsh Bakshi, Chaitanya Joshi, Madhvi Joshi
EPI_ISL_475048	GMERS Medical College & Hospital	Gujarat Biotechnology Research Centre	Janvi Raval, Zarna Patel, Monika Gandhi, Pinal Trivedi, Maharshi Pandya, Nidhi Patel, Nitin Savaliya, Raghawendra Kumar, Dinesh Kumar, Zuber Saiyed, Komal Patel, Labdhi Pandya, Snehal Bagatharia, Meenakshi Shah, Neena Doshi, Varsha Godbole, Pritesh Sabara, Apurvasinh Puvar, Neha Rajpara, R D Dixit, A M Kadri, Harsh Bakshi, Chaitanya Joshi, Madhvi Joshi
EPI_ISL_475050	GMERS Medical College & Hospital	Gujarat Biotechnology Research Centre	Monika Gandhi, Pinal Trivedi, Maharshi Pandya, Nidhi Patel, Nitin Savaliya, Raghawendra Kumar, Dinesh Kumar, Zuber Saiyed, Komal Patel, Labdhi Pandya, Snehal Bagatharia, Meenakshi Shah, Neena Doshi, Varsha Godbole, Pritesh Sabara, Apurvasinh Puvar, Janvi Raval, Zarna Patel, Fenil Patel, R D Dixit, A M Kadri, Harsh Bakshi, Chaitanya Joshi, Madhvi Joshi
EPI_ISL_475051	GMERS Medical College & Hospital	Gujarat Biotechnology Research Centre	Pinal Trivedi, Maharshi Pandya, Nidhi Patel, Nitin Savaliya, Raghawendra Kumar, Dinesh Kumar, Zuber Saiyed, Komal Patel, Labdhi Pandya, Snehal Bagatharia, Meenakshi Shah, Neena Doshi, Varsha Godbole, Pritesh Sabara, Apurvasinh Puvar, Janvi Raval, Zarna Patel, Monika Gandhi, Neelam Nathani, R D Dixit, A M Kadri, Harsh Bakshi, Chaitanya Joshi, Madhvi Joshi
EPI_ISL_475052	GMERS Medical College & Hospital	Gujarat Biotechnology Research Centre	Maharshi Pandya, Nidhi Patel, Nitin Savaliya, Raghawendra Kumar, Dinesh Kumar, Zuber Saiyed, Komal Patel, Labdhi Pandya, Snehal Bagatharia, Meenakshi Shah, Neena Doshi, Varsha Godbole, Pritesh Sabara, Apurvasinh Puvar, Janvi Raval, Zarna Patel, Monika Gandhi, Pinal Trivedi, Armi Chaudhari, R D Dixit, A M Kadri, Harsh Bakshi, Chaitanya Joshi, Madhvi Joshi
EPI_ISL_475053	GMERS Medical College & Hospital	Gujarat Biotechnology Research Centre	Nidhi Patel, Nitin Savaliya, Raghawendra Kumar, Dinesh Kumar, Zuber Saiyed, Komal Patel, Labdhi Pandya, Snehal Bagatharia, Meenakshi Shah, Neena Doshi, Varsha Godbole, Pritesh Sabara, Apurvasinh Puvar, Janvi Raval, Zarna Patel, Monika Gandhi, Pinal Trivedi, Maharshi Pandya, Bhavya Jindal, R D Dixit, A M Kadri, Harsh Bakshi, Chaitanya Joshi, Madhvi Joshi
EPI_ISL_475056	Dr. N. D. Desai Medical College & Hospital	Gujarat Biotechnology Research Centre	Dinesh Kumar, Zuber Saiyed, Komal Patel, Labdhi Pandya, Snehal Bagatharia, J G Buch, Jigar Gusani, Supreet Prabhu, Pritesh Sabara, Apurvasinh Puvar, Janvi Raval, Zarna Patel, Monika Gandhi, Pinal Trivedi, Maharshi Pandya, Nidhi Patel, Nitin Savaliya, Raghawendra Kumar, Akanksha Verma, R D Dixit, A M Kadri, Harsh Bakshi, Chaitanya Joshi, Madhvi Joshi
EPI_ISL_475057	Dr. N. D. Desai Medical College & Hospital	Gujarat Biotechnology Research Centre	Zuber Saiyed, Komal Patel, Labdhi Pandya, Supreet Prabhu, Snehal Bagatharia, Jigar Gusani, J G Buch, Pritesh Sabara, Apurvasinh Puvar, Janvi Raval, Zarna Patel, Monika Gandhi, Pinal Trivedi, Maharshi Pandya, Nidhi Patel, Nitin Savaliya, Raghawendra Kumar, Dinesh Kumar, Priti Pandita, R D Dixit, A M Kadri, Harsh Bakshi, Chaitanya Joshi, Madhvi Joshi
EPI_ISL_475059	GAIMS & G K General Hospital	Gujarat Biotechnology Research Centre	Hitesh Assudani, Babulal Babhoria, Labdhi Pandya, Snehal Bagatharia, Pritesh Sabara, Apurvasinh Puvar, Janvi Raval, Zarna Patel, Monika Gandhi, Pinal Trivedi, Maharshi Pandya, Nidhi Patel, Nitin Savaliya, Raghawendra Kumar, Dinesh Kumar, Zuber Saiyed, Komal Patel, Neha Rajpara, R D Dixit, A M Kadri, Harsh Bakshi, Chaitanya Joshi, Madhvi Joshi
EPI_ISL_476148, EPI_ISL_476149,	Institut Pasteur Dakar	Institut Pasteur de Dakar	Ndongo Dia, Moussa Moise Diagne, Mamadou Diop, Ousmane Faye, Amadou Alpha Sall

EPI_ISL_476492			
EPI_ISL_476493	Institut Pasteur Dakar	Institut Pasteur de Dakar	Ndongo Dia, Moussa Moise Diagne, Mamadou Diop, Ousmane Faye, Amadou alpha Sall
EPI_ISL_476494	Institut Pasteur Dakar	Institut Pasteur de Dakar	Ndongo Dia, Moussa Moise Diagne, Mamadou Diop, Ousmane Faye, Amadou Alpha Sall
EPI_ISL_476495	Institut Pasteur Dakar	Institut Pasteur de Dakar	Ndongo Dia, Moussa Moise Diagne, Mamadou Diop, Ousmane Faye, Amadou alpha Sall
EPI_ISL_476514	Institut Pasteur Dakar	Institut Pasteur de Dakar	Ndongo Dia, Moussa Moise Diagne, Mamadou Diop, Ousmane Faye, Amadou Alpha Sall
EPI_ISL_476516	Institut Pasteur Dakar	Institut Pasteur de Dakar	Ndongo Dia, Moussa Moise Diagne, mamadou Diop, Ousmane Faye, Amadou Alpha Sall
EPI_ISL_476558, EPI_ISL_476562	Institut Pasteur Dakar	Institut Pasteur de Dakar	Ndongo Dia, Moussa Moise Diagne, Mamadou Diop, Ousmane Faye, Amadou Alpha Sall
EPI_ISL_476564	Institut Pasteur Dakar	Institut Pasteur de Dakar	Ndongo Dia, Moussa Moise Diagne, Mamadou diop, Ousmane Faye, Amadou alpha Sall
EPI_ISL_476569	Institut Pasteur Dakar	Institut Pasteur de Dakar	Ndongo Dia, Moussa Moise, Mamadou Diop, Ousmane Faye, Amadou Alpha Sall
EPI_ISL_476572, EPI_ISL_476574	Institut Pasteur Dakar	Institut Pasteur de Dakar	Ndongo Dia, Moussa Moise Diagne, Mamadou Diop, Ousmane Faye, Amadou Alpha Sall
EPI_ISL_476855	GMERS Medical College & Hospital, Gotri, Vadodara	Gujarat Biotechnology Research Centre	Apurvasinh Puvar, Janvi Raval, Zarna Patel, Monika Gandhi, Pinal Trivedi, Maharshi Pandya, Nidhi Patel, Nitin Savaliya, Raghawendra Kumar, Dinesh Kumar, Zuber Saiyed, Komal Patel, Labdhi Pandya, Afzal Ansari, Nikha Trivedi, Meenakshi Shah, Neena Doshi, Varsha Godbole, R D Dixit, A M Kadri, Harsh Bakshi, Chaitanya Joshi, Madhvi Joshi
EPI_ISL_476856	GMERS Medical College & Hospital, Gotri, Vadodara	Gujarat Biotechnology Research Centre	Janvi Raval, Zarna Patel, Monika Gandhi, Pinal Trivedi, Maharshi Pandya, Nidhi Patel, Nitin Savaliya, Raghawendra Kumar, Dinesh Kumar, Zuber Saiyed, Komal Patel, Labdhi Pandya, Afzal Ansari, Nikha Trivedi, Meenakshi Shah, Neena Doshi, Varsha Godbole, Apurvasinh Puvar, R D Dixit, A M Kadri, Harsh Bakshi, Chaitanya Joshi, Madhvi Joshi
EPI_ISL_476857	GMERS Medical College & Hospital, Gotri, Vadodara	Gujarat Biotechnology Research Centre	Zarna Patel, Monika Gandhi, Pinal Trivedi, Maharshi Pandya, Nidhi Patel, Nitin Savaliya, Raghawendra Kumar, Dinesh Kumar, Zuber Saiyed, Komal Patel, Labdhi Pandya, Afzal Ansari, Nikha Trivedi, Meenakshi Shah, Neena Doshi, Varsha Godbole, Apurvasinh Puvar, Janvi Raval, R D Dixit, A M Kadri, Harsh Bakshi, Chaitanya Joshi, Madhvi Joshi
EPI_ISL_476858	GMERS Medical College & Hospital, Gotri, Vadodara	Gujarat Biotechnology Research Centre	Monika Gandhi, Pinal Trivedi, Maharshi Pandya, Nidhi Patel, Nitin Savaliya, Raghawendra Kumar, Dinesh Kumar, Zuber Saiyed, Komal Patel, Labdhi Pandya, Afzal Ansari, Nikha Trivedi, Meenakshi Shah, Neena Doshi, Varsha Godbole, Apurvasinh Puvar, Janvi Raval, Zarna Patel, R D Dixit, A M Kadri, Harsh Bakshi, Chaitanya Joshi, Madhvi Joshi
EPI_ISL_476859	GMERS Medical College & Hospital, Gotri, Vadodara	Gujarat Biotechnology Research Centre	Pinal Trivedi, Maharshi Pandya, Nidhi Patel, Nitin Savaliya, Raghawendra Kumar, Dinesh Kumar, Zuber Saiyed, Komal Patel, Labdhi Pandya, Afzal Ansari, Nikha Trivedi, Meenakshi Shah, Neena Doshi, Varsha Godbole, Apurvasinh Puvar, Janvi Raval, Zarna Patel, Monika Gandhi, R D Dixit, A M Kadri, Harsh Bakshi, Chaitanya Joshi, Madhvi Joshi
EPI_ISL_476860	GMERS Medical College & Hospital, Gotri, Vadodara	Gujarat Biotechnology Research Centre	Maharshi Pandya, Nidhi Patel, Nitin Savaliya, Raghawendra Kumar, Dinesh Kumar, Zuber Saiyed, Komal Patel, Labdhi Pandya, Afzal Ansari, Nikha Trivedi, Meenakshi Shah, Neena Doshi, Varsha Godbole, Apurvasinh Puvar, Janvi Raval, Zarna Patel, Monika Gandhi, Pinal Trivedi, R D Dixit, A M Kadri, Harsh Bakshi, Chaitanya Joshi, Madhvi Joshi
EPI_ISL_476861	GMERS Medical College & Hospital, Gotri, Vadodara	Gujarat Biotechnology Research Centre	Nidhi Patel, Nitin Savaliya, Raghawendra Kumar, Dinesh Kumar, Zuber Saiyed, Komal Patel, Labdhi Pandya, Afzal Ansari, Nikha Trivedi, Meenakshi Shah, Neena Doshi, Varsha Godbole, Apurvasinh Puvar, Janvi Raval, Zarna Patel, Monika Gandhi, Pinal Trivedi, Maharshi Pandya, R D Dixit, A M Kadri, Harsh Bakshi, Chaitanya Joshi, Madhvi Joshi
EPI_ISL_476862	GMERS Medical College & Hospital, Gotri, Vadodara	Gujarat Biotechnology Research Centre	Nitin Savaliya, Raghawendra Kumar, Dinesh Kumar, Zuber Saiyed, Komal Patel, Labdhi Pandya, Afzal Ansari, Nikha Trivedi, Meenakshi Shah, Neena Doshi, Varsha Godbole, Apurvasinh Puvar, Janvi Raval, Zarna Patel, Monika Gandhi, Pinal Trivedi, Maharshi Pandya, Nidhi Patel, R D Dixit, A M Kadri, Harsh Bakshi, Chaitanya Joshi, Madhvi Joshi
EPI_ISL_476863	GMERS Medical College and Hospital, Gandhinagar	Gujarat Biotechnology Research Centre	Raghawendra Kumar, Dinesh Kumar, Zuber Saiyed, Komal Patel, Labdhi Pandya, Afzal Ansari, Nikha Trivedi, Seema Bhatt, Gaurishankar Shrimali, Bhavesh Modi, Bharti Rajani, Apurvasinh Puvar, Janvi Raval, Zarna Patel, Monika Gandhi, Pinal Trivedi, Maharshi Pandya, Nidhi Patel, Nitin Savaliya, R D Dixit, A M Kadri, Harsh Bakshi, Chaitanya Joshi, Madhvi Joshi
EPI_ISL_476864	GMERS Medical College and Hospital, Gandhinagar	Gujarat Biotechnology Research Centre	Dinesh Kumar, Zuber Saiyed, Komal Patel, Labdhi Pandya, Afzal Ansari, Nikha Trivedi, Seema Bhatt, Gaurishankar Shrimali, Bhavesh Modi, Bharti Rajani, Apurvasinh Puvar, Janvi Raval, Zarna Patel, Monika Gandhi, Pinal Trivedi, Maharshi Pandya, Nidhi Patel, Nitin Savaliya, Raghawendra Kumar, R D Dixit, A M Kadri, Harsh Bakshi, Chaitanya Joshi, Madhvi Joshi
EPI_ISL_476865	GMERS Medical College and Hospital, Gandhinagar	Gujarat Biotechnology Research Centre	Zuber Saiyed, Komal Patel, Labdhi Pandya, Afzal Ansari, Nikha Trivedi, Seema Bhatt, Gaurishankar Shrimali, Bhavesh Modi, Bharti Rajani, Apurvasinh Puvar, Janvi Raval, Zarna Patel, Monika Gandhi, Pinal Trivedi, Maharshi Pandya, Nidhi Patel, Nitin Savaliya, Raghawendra Kumar, Dinesh Kumar, R D Dixit, A M Kadri, Harsh Bakshi, Chaitanya Joshi, Madhvi Joshi
EPI_ISL_476866	GMERS Medical College and Hospital, Gandhinagar	Gujarat Biotechnology Research Centre	Komal Patel, Labdhi Pandya, Afzal Ansari, Nikha Trivedi, Seema Bhatt, Gaurishankar Shrimali, Bhavesh Modi, Bharti Rajani, Apurvasinh Puvar, Janvi Raval, Zarna Patel, Monika Gandhi, Pinal Trivedi, Maharshi Pandya, Nidhi Patel, Nitin Savaliya, Raghawendra Kumar, Dinesh Kumar, Zuber Saiyed, R D Dixit, A M Kadri, Harsh Bakshi, Chaitanya Joshi, Madhvi Joshi
EPI_ISL_476867	Banas Medical College and Research Institute	Gujarat Biotechnology Research Centre	Labdhi Pandya, Afzal Ansari, Nikha Trivedi, Radhika Khara, Sunil R Joshi, Viren s Doshi, Apurvasinh Puvar, Janvi Raval, Zarna Patel, Monika Gandhi, Pinal Trivedi, Maharshi Pandya, Nidhi Patel, Nitin Savaliya, Raghawendra Kumar, Dinesh Kumar, Zuber Saiyed, Komal Patel, R D Dixit, A M Kadri, Harsh Bakshi, Chaitanya Joshi, Madhvi Joshi
EPI_ISL_476868	Banas Medical College and Research Institute	Gujarat Biotechnology Research Centre	Afzal Ansari, Nikha Trivedi, Radhika Khara, Sunil R Joshi, Viren s Doshi, Apurvasinh Puvar, Janvi Raval, Zarna Patel, Monika Gandhi, Pinal Trivedi, Maharshi Pandya, Nidhi Patel, Nitin Savaliya, Raghawendra Kumar, Dinesh Kumar, Zuber Saiyed, Komal Patel, Labdhi Pandya, R D Dixit, A M Kadri, Harsh Bakshi, Chaitanya Joshi, Madhvi Joshi
EPI_ISL_476869	Department of MicroBiology, Government Medical College, Surat	Gujarat Biotechnology Research Centre	Nikha Trivedi, Naresh Chauhan, Summaiya Mullan, Amit gamit, Apurvasinh Puvar, Janvi Raval, Zarna Patel, Monika Gandhi, Pinal Trivedi, Maharshi Pandya, Nidhi Patel, Nitin Savaliya, Raghawendra Kumar, Dinesh Kumar, Zuber Saiyed, Komal Patel, Labdhi Pandya, Afzal Ansari, R D Dixit, A M Kadri, Harsh Bakshi, Chaitanya Joshi, Madhvi Joshi
EPI_ISL_476870	Department of MicroBiology, Government Medical College, Surat	Gujarat Biotechnology Research Centre	Naresh Chauhan, Summaiya Mullan, Amit gamit, Apurvasinh Puvar, Janvi Raval, Zarna Patel, Monika Gandhi, Pinal Trivedi, Maharshi Pandya, Nidhi Patel, Nitin Savaliya, Raghawendra Kumar, Dinesh Kumar, Zuber Saiyed, Komal Patel, Labdhi Pandya, Afzal Ansari, Nikha Trivedi, R D Dixit, A M Kadri, Harsh Bakshi, Chaitanya Joshi, Madhvi Joshi
EPI_ISL_476871	Department of MicroBiology, Government Medical College, Surat	Gujarat Biotechnology Research Centre	Summaiya Mullan, Amit gamit, Apurvasinh Puvar, Janvi Raval, Zarna Patel, Monika Gandhi, Pinal Trivedi, Maharshi Pandya, Nidhi Patel, Nitin Savaliya, Raghawendra Kumar, Dinesh Kumar, Zuber Saiyed, Komal Patel, Labdhi Pandya, Afzal Ansari, Nikha Trivedi, Naresh Chauhan, R D Dixit, A M Kadri, Harsh Bakshi, Chaitanya Joshi, Madhvi Joshi
EPI_ISL_476872	Department of MicroBiology, Government Medical College, Surat	Gujarat Biotechnology Research Centre	Amit gamit, Apurvasinh Puvar, Janvi Raval, Zarna Patel, Monika Gandhi, Pinal Trivedi, Maharshi Pandya, Nidhi Patel, Nitin Savaliya, Raghawendra Kumar, Dinesh Kumar, Zuber Saiyed, Komal Patel, Labdhi Pandya, Afzal Ansari, Nikha Trivedi, Naresh Chauhan, Summaiya Mullan, R D Dixit, A M Kadri, Harsh Bakshi, Chaitanya Joshi, Madhvi Joshi
EPI_ISL_476873	Department of MicroBiology, Government Medical College, Surat	Gujarat Biotechnology Research Centre	Apurvasinh Puvar, Janvi Raval, Zarna Patel, Monika Gandhi, Pinal Trivedi, Maharshi Pandya, Nidhi Patel, Nitin Savaliya, Raghawendra Kumar, Dinesh Kumar, Zuber Saiyed, Komal Patel, Labdhi Pandya, Afzal Ansari, Nikha Trivedi, Naresh Chauhan, Summaiya Mullan, Amit gamit, R D Dixit, A M Kadri, Harsh Bakshi, Chaitanya Joshi, Madhvi Joshi
EPI_ISL_476874	Department of MicroBiology, Government Medical College, Surat	Gujarat Biotechnology Research Centre	Janvi Raval, Zarna Patel, Monika Gandhi, Pinal Trivedi, Maharshi Pandya, Nidhi Patel, Nitin Savaliya, Raghawendra Kumar, Dinesh Kumar, Zuber Saiyed, Komal Patel, Labdhi Pandya, Afzal Ansari, Nikha Trivedi, Naresh Chauhan, Summaiya Mullan, Amit gamit, Apurvasinh Puvar, R D Dixit, A M Kadri, Harsh Bakshi, Chaitanya Joshi, Madhvi Joshi
EPI_ISL_476875	Department of MicroBiology, Government Medical College, Surat	Gujarat Biotechnology Research Centre	Zarna Patel, Monika Gandhi, Pinal Trivedi, Maharshi Pandya, Nidhi Patel, Nitin Savaliya, Raghawendra Kumar, Dinesh Kumar, Zuber Saiyed, Komal Patel, Labdhi Pandya, Afzal Ansari, Nikha Trivedi, Naresh Chauhan, Summaiya Mullan, Amit gamit, Apurvasinh Puvar, Janvi Raval, R D Dixit, A M Kadri, Harsh Bakshi, Chaitanya Joshi, Madhvi Joshi

EPI_ISL_476876	Department of MicroBiology, Government Medical College, Surat	Gujarat Biotechnology Research Centre	Pinal Trivedi, Maharshi Pandya, Nidhi Patel, Nitin Savaliya, Raghawendra Kumar, Dinesh Kumar, Zuber Saiyed, Komal Patel, Labdhi Pandya, Afzal Ansari, Nikha Trivedi, Naresh Chauhan, Summaiya Mullan, Amit gamit, Apurvasinh Puvar, Janvi Raval, Zarna Patel, Monika Gandhi, R D Dixit, A M Kadri, Harsh Bakshi, Chaitanya Joshi, Madhvi Joshi
EPI_ISL_476877	Department of MicroBiology, Government Medical College, Surat	Gujarat Biotechnology Research Centre	Maharshi Pandya, Nidhi Patel, Nitin Savaliya, Raghawendra Kumar, Dinesh Kumar, Zuber Saiyed, Komal Patel, Labdhi Pandya, Afzal Ansari, Nikha Trivedi, Naresh Chauhan, Summaiya Mullan, Amit gamit, Apurvasinh Puvar, Janvi Raval, Zarna Patel, Monika Gandhi, Pinal Trivedi, R D Dixit, A M Kadri, Harsh Bakshi, Chaitanya Joshi, Madhvi Joshi
EPI_ISL_476878	Department of MicroBiology, Government Medical College, Surat	Gujarat Biotechnology Research Centre	Nidhi Patel, Nitin Savaliya, Raghawendra Kumar, Dinesh Kumar, Zuber Saiyed, Komal Patel, Labdhi Pandya, Afzal Ansari, Nikha Trivedi, Naresh Chauhan, Summaiya Mullan, Amit gamit, Apurvasinh Puvar, Janvi Raval, Zarna Patel, Monika Gandhi, Pinal Trivedi, Maharshi Pandya, R D Dixit, A M Kadri, Harsh Bakshi, Chaitanya Joshi, Madhvi Joshi
EPI_ISL_476879	Department of MicroBiology, Government Medical College, Surat	Gujarat Biotechnology Research Centre	Nitin Savaliya, Raghawendra Kumar, Dinesh Kumar, Zuber Saiyed, Komal Patel, Labdhi Pandya, Afzal Ansari, Nikha Trivedi, Naresh Chauhan, Summaiya Mullan, Amit gamit, Apurvasinh Puvar, Janvi Raval, Zarna Patel, Monika Gandhi, Pinal Trivedi, Maharshi Pandya, Nidhi Patel, R D Dixit, A M Kadri, Harsh Bakshi, Chaitanya Joshi, Madhvi Joshi
EPI_ISL_476880	Department of MicroBiology, Government Medical College, Surat	Gujarat Biotechnology Research Centre	Raghawendra Kumar, Dinesh Kumar, Zuber Saiyed, Komal Patel, Labdhi Pandya, Afzal Ansari, Nikha Trivedi, Naresh Chauhan, Summaiya Mullan, Amit gamit, Apurvasinh Puvar, Janvi Raval, Zarna Patel, Monika Gandhi, Pinal Trivedi, Maharshi Pandya, Nidhi Patel, Nitin Savaliya, R D Dixit, A M Kadri, Harsh Bakshi, Chaitanya Joshi, Madhvi Joshi
EPI_ISL_476881	Department of MicroBiology, Government Medical College, Surat	Gujarat Biotechnology Research Centre	Dinesh Kumar, Zuber Saiyed, Komal Patel, Labdhi Pandya, Afzal Ansari, Nikha Trivedi, Naresh Chauhan, Summaiya Mullan, Amit gamit, Apurvasinh Puvar, Janvi Raval, Zarna Patel, Monika Gandhi, Pinal Trivedi, Maharshi Pandya, Nidhi Patel, Nitin Savaliya, Raghawendra Kumar, R D Dixit, A M Kadri, Harsh Bakshi, Chaitanya Joshi, Madhvi Joshi
EPI_ISL_476882	Department of MicroBiology, Government Medical College, Surat	Gujarat Biotechnology Research Centre	Zuber Saiyed, Komal Patel, Labdhi Pandya, Afzal Ansari, Nikha Trivedi, Naresh Chauhan, Summaiya Mullan, Amit gamit, Apurvasinh Puvar, Janvi Raval, Zarna Patel, Monika Gandhi, Pinal Trivedi, Maharshi Pandya, Nidhi Patel, Nitin Savaliya, Raghawendra Kumar, Dinesh Kumar, R D Dixit, A M Kadri, Harsh Bakshi, Chaitanya Joshi, Madhvi Joshi
EPI_ISL_476884, EPI_ISL_476885, EPI_ISL_476886, EPI_ISL_476887, EPI_ISL_476888, EPI_ISL_476889, EPI_ISL_476890, EPI_ISL_476891, EPI_ISL_476892, EPI_ISL_476893, EPI_ISL_476894, EPI_ISL_476895, EPI_ISL_476896			
see above	Defence Research & Development Establishment (DRDE)	Defence Research & Development Establishment (DRDE)	Shashi Sharma, Paban Kumar Dash, Sushil Kumar Sharma, Ambuj Shrivastava, Jyoti S. Kumar
EPI_ISL_477015	Institute of Microbiology, Universidad San Francisco de Quito	Institute of Microbiology, Universidad San Francisco de Quito	Sully Márquez, Belén Prado-Vivar, Juan José Guadalupe, Monica Becerra-Wong, Carla Torres, Bernardo Gutiérrez, Jorge Luis Velez, Verónica Barragán, Patricio Rojas-Silva, Gabriel Trueba, Michelle Grunauer, Paúl Cárdenas
EPI_ISL_477016	Institute of Microbiology, Universidad San Francisco de Quito	Institute of Microbiology, Universidad San Francisco de Quito	Juan José Guadalupe, Sully Márquez, Belén Prado-Vivar, Monica Becerra-Wong, Carla Torres, Bernardo Gutiérrez, Jorge Luis Velez, Verónica Barragán, Patricio Rojas-Silva, Gabriel Trueba, Michelle Grunauer, Paúl Cárdenas
EPI_ISL_477143, EPI_ISL_477150, EPI_ISL_477155, EPI_ISL_477156	Institut Pasteur Dakar	Institut Pasteur de Dakar	Ndongo Dia, Moussa Moise Diagne, Mamadou Diop, Mamadou Malado Jallow, Marie Henriette Dior Ndione, Safietou Sankhe, Ousmane Faye, Amadou Alpha Sall.
EPI_ISL_477180	Department of Laboratory Medicine Tan Tock Seng Hospital	Department of Laboratory Medicine Tan Tock Seng Hospital	Chen YYC, Zair X, Li C, Tang WY, Maurer-Stroh S, Barkham TMS, Nagarajan N, Sessions OM
EPI_ISL_477183	Department of MicroBiology, Government Medical College, Surat	Gujarat Biotechnology Research Centre	Monika Gandhi, Pinal Trivedi, Maharshi Pandya, Nidhi Patel, Nitin Savaliya, Raghawendra Kumar, Dinesh Kumar, Zuber Saiyed, Komal Patel, Labdhi Pandya, Afzal Ansari, Nikha Trivedi, Naresh Chauhan, Summaiya Mullan, Amit gamit, Apurvasinh Puvar, Janvi Raval, Zarna Patel, R D Dixit, A M Kadri, Harsh Bakshi, Chaitanya Joshi, Madhvi Joshi
EPI_ISL_479534, EPI_ISL_479535, EPI_ISL_479536, EPI_ISL_479537, EPI_ISL_479538, EPI_ISL_479539, EPI_ISL_479540, EPI_ISL_479541, EPI_ISL_479543, EPI_ISL_479544, EPI_ISL_479546, EPI_ISL_479547, EPI_ISL_479548, EPI_ISL_479550, EPI_ISL_479551, EPI_ISL_479552, EPI_ISL_479553			
see above	NIV Influenza	NIV Influenza	Potdar V
EPI_ISL_482682, EPI_ISL_482683, EPI_ISL_482684, EPI_ISL_482685, EPI_ISL_482686, EPI_ISL_482687, EPI_ISL_482688, EPI_ISL_482689, EPI_ISL_482690, EPI_ISL_482691, EPI_ISL_482692, EPI_ISL_482693, EPI_ISL_482694, EPI_ISL_482695, EPI_ISL_482696, EPI_ISL_482697, EPI_ISL_482698, EPI_ISL_482699			
see above	Singapore General Hospital	Department of Microbiology	Nurdyana Abdul Rahman, Kun Lee Lim, Chenhao Li, Kian Sing Chan, Lynette Oon, Kern Rei Chng, Niranjan Nagarajan, Karrie Ko
EPI_ISL_482702, EPI_ISL_482704, EPI_ISL_482705, EPI_ISL_482708, EPI_ISL_482709	Molecular Diagnostics Services (MDS)	KRISP, KZN Research Innovation and Sequencing Platform	Giandhari J, Pillay S, Lessells R, Chimukangara B, Mdlalose K, York D, Khan S, Tegally H, Wilkinson E, de Oliveira T
EPI_ISL_482710, EPI_ISL_482711, EPI_ISL_482712	NHLS-IALCH	KRISP, KZN Research Innovation and Sequencing Platform	Giandhari J, Pillay S, Lessells R, Chimukangara B, Mdlalose K, York D, Khan S, Tegally H, Wilkinson E, de Oliveira T
EPI_ISL_482716, EPI_ISL_482717, EPI_ISL_482718, EPI_ISL_482719, EPI_ISL_482720, EPI_ISL_482721, EPI_ISL_482722, EPI_ISL_482723	Molecular Diagnostics Services (MDS)	KRISP, KZN Research Innovation and Sequencing Platform	Giandhari J, Pillay S, Lessells R, Chimukangara B, Mdlalose K, York D, Khan S, Tegally H, Wilkinson E, de Oliveira T
EPI_ISL_482726	NHLS-IALCH	KRISP, KZN Research Innovation and Sequencing Platform	Giandhari J, Pillay S, Lessells R, Chimukangara B, Mdlalose K, York D, Khan S, Tegally H, Wilkinson E, de Oliveira T
EPI_ISL_482778, EPI_ISL_482782, EPI_ISL_482783	Tuen Mun Hospital	Hong Kong Department of Health	Mak Gannon C.K., Cheng Peter K.C., Lam Edman T.K., Chan Rickjason C.W., Tsang Dominic N.C.
EPI_ISL_482852, EPI_ISL_482853, EPI_ISL_482862, EPI_ISL_482867	Molecular Diagnostics Services (MDS)	KRISP, KZN Research Innovation and Sequencing Platform	Giandhari J, Pillay S, Lessells R, Chimukangara B, Mdlalose K, York D, Khan S, Tegally H, Wilkinson E, de Oliveira T
EPI_ISL_482874, EPI_ISL_482875, EPI_ISL_482876, EPI_ISL_482877, EPI_ISL_482878	Institut Pasteur Dakar	Institut Pasteur de Dakar	Ndongo Dia, Moussa Moise Diagne, Mamadou Diop, Marie Henriette Dior Ndione, Mamadou malado Jallow, Safietou Sankhe, Ousmane Faye, Amadou Alpha Sall.
EPI_ISL_483566	Clinical Microbiology Laboratory- Basurto University Hospital	Biocruces-Bizkaia	Mikel J. Urrutikoetxea-Gutierrez, Ana Belén Belén de la Hoz, Matxalen Vidal-García, Mº Carmen Nieto Toboso, Estibaliz Ugalde-Zarraga, José Luis Díaz de Tuesta del Arco
EPI_ISL_483570	Clinical Microbiology Laboratory- Basurto University Hospita	Biocruces-Bizkaia	Mikel J. Urrutikoetxea-Gutierrez, Ana Belén Belén de la Hoz, Matxalen Vidal-García, Mº Carmen Nieto Toboso, Estibaliz Ugalde-Zarraga, José Luis Díaz de Tuesta del Arco
EPI_ISL_483571, EPI_ISL_483572, EPI_ISL_483573	Clinical Microbiology Laboratory- Basurto University Hospital	Biocruces-Bizkaia	Mikel J. Urrutikoetxea-Gutierrez, Ana Belén Belén de la Hoz, Matxalen Vidal-García, Mº Carmen Nieto Toboso, Estibaliz Ugalde-Zarraga, José Luis Díaz de Tuesta del Arco
EPI_ISL_483820	GMERS Medical College and Hospital, Gandhinagar	Gujarat Biotechnology Research Centre	Komal Patel, Labdhi Pandya, Afzal Ansari, Nikha Trivedi, Seema Bhatt, Gaurishankar Shrimali, Bhavesh Modi, Bharti Rajani, Apurvasinh Puvar, Janvi Raval, Zarna Patel, Monika Gandhi, Pinal Trivedi, Maharshi Pandya, Nidhi Patel, Nitin Savaliya, Raghawendra Kumar, Dinesh Kumar, Zuber Saiyed, R D Dixit, A M Kadri, Harsh Bakshi, Chaitanya Joshi, Madhvi Joshi
EPI_ISL_483821	Government Medical College, Vadodara	Gujarat Biotechnology Research Centre	Labdhi Pandya, Afzal Ansari, Nikha Trivedi, Meenakshi Shah, Neena Doshi, Varsha Godbole, Apurvasinh Puvar, Janvi Raval, Zarna Patel, Monika Gandhi, Pinal Trivedi, Maharshi Pandya, Nidhi Patel, Nitin Savaliya, Raghawendra Kumar, Dinesh Kumar, Zuber Saiyed, Komal Patel, R D Dixit, A M Kadri, Harsh Bakshi, Chaitanya Joshi, Madhvi Joshi
EPI_ISL_483822	Government Medical College, Vadodara	Gujarat Biotechnology Research Centre	Afzal Ansari, Nikha Trivedi, Meenakshi Shah, Neena Doshi, Varsha Godbole, Apurvasinh Puvar, Janvi Raval, Zarna Patel, Monika Gandhi, Pinal Trivedi, Maharshi Pandya, Nidhi Patel, Nitin Savaliya, Raghawendra Kumar, Dinesh Kumar, Zuber Saiyed, Komal Patel, Labdhi Pandya, R D Dixit, A M Kadri, Harsh Bakshi, Chaitanya Joshi, Madhvi Joshi
EPI_ISL_483823	GMERS Medical College Himmatnagar	Gujarat Biotechnology Research Centre	Nikha Trivedi, Himanshu Khatri, Mayur Gandhi, Apurvasinh Puvar, Janvi Raval, Zarna Patel, Monika Gandhi, Pinal Trivedi, Maharshi Pandya, Nidhi Patel, Nitin Savaliya, Raghawendra Kumar, Dinesh Kumar, Zuber Saiyed, Komal Patel, Labdhi Pandya, Afzal Ansari, R D Dixit, A M Kadri, Harsh Bakshi, Chaitanya Joshi, Madhvi Joshi

EPI_ISL_483824	GMERS Medical College Himmatnagar	Gujarat Biotechnology Research Centre	Himanshu Khatri, Mayur Gandhi, Apurvasinh Puvar, Janvi Raval, Zarna Patel, Monika Gandhi, Pinal Trivedi, Maharshi Pandya, Nidhi Patel, Nitin Savaliya, Raghawendra Kumar, Dinesh Kumar, Zuber Saiyed, Komal Patel, Labdhi Pandya, Afzal Ansari, Nikha Trivedi, R D Dixit, A M Kadri, Harsh Bakshi, Chaitanya Joshi, Madhvi Joshi
EPI_ISL_483825	GMERS Medical College Himmatnagar	Gujarat Biotechnology Research Centre	Mayur Gandhi, Apurvasinh Puvar, Janvi Raval, Zarna Patel, Monika Gandhi, Pinal Trivedi, Maharshi Pandya, Nidhi Patel, Nitin Savaliya, Raghawendra Kumar, Dinesh Kumar, Zuber Saiyed, Komal Patel, Labdhi Pandya, Afzal Ansari, Nikha Trivedi, Himanshu Khatri, R D Dixit, A M Kadri, Harsh Bakshi, Chaitanya Joshi, Madhvi Joshi
EPI_ISL_483826	GMERS Medical College Himmatnagar	Gujarat Biotechnology Research Centre	Apurvasinh Puvar, Janvi Raval, Zarna Patel, Monika Gandhi, Pinal Trivedi, Maharshi Pandya, Nidhi Patel, Nitin Savaliya, Raghawendra Kumar, Dinesh Kumar, Zuber Saiyed, Komal Patel, Labdhi Pandya, Afzal Ansari, Nikha Trivedi, Himanshu Khatri, Mayur Gandhi, R D Dixit, A M Kadri, Harsh Bakshi, Chaitanya Joshi, Madhvi Joshi
EPI_ISL_483827	GMERS Medical College Himmatnagar	Gujarat Biotechnology Research Centre	Janvi Raval, Zarna Patel, Monika Gandhi, Pinal Trivedi, Maharshi Pandya, Nidhi Patel, Nitin Savaliya, Raghawendra Kumar, Dinesh Kumar, Zuber Saiyed, Komal Patel, Labdhi Pandya, Afzal Ansari, Nikha Trivedi, Himanshu Khatri, Mayur Gandhi, Apurvasinh Puvar, R D Dixit, A M Kadri, Harsh Bakshi, Chaitanya Joshi, Madhvi Joshi
EPI_ISL_483828	GMERS Medical College Himmatnagar	Gujarat Biotechnology Research Centre	Zarna Patel, Monika Gandhi, Pinal Trivedi, Maharshi Pandya, Nidhi Patel, Nitin Savaliya, Raghawendra Kumar, Dinesh Kumar, Zuber Saiyed, Komal Patel, Labdhi Pandya, Afzal Ansari, Nikha Trivedi, Himanshu Khatri, Mayur Gandhi, Apurvasinh Puvar, Janvi Raval, R D Dixit, A M Kadri, Harsh Bakshi, Chaitanya Joshi, Madhvi Joshi
EPI_ISL_483829	Pandit Deendayal Upadhyay Government Medical College, Rajkot	Gujarat Biotechnology Research Centre	Monika Gandhi, Pinal Trivedi, Maharshi Pandya, Nidhi Patel, Nitin Savaliya, Raghawendra Kumar, Dinesh Kumar, Zuber Saiyed, Komal Patel, Labdhi Pandya, Afzal Ansari, Nikha Trivedi, Gauravi Dhruv, Arti Trivedi, Apurvasinh Puvar, Janvi Raval, Zarna Patel, R D Dixit, A M Kadri, Harsh Bakshi, Chaitanya Joshi, Madhvi Joshi
EPI_ISL_483830	Pandit Deendayal Upadhyay Government Medical College, Rajkot	Gujarat Biotechnology Research Centre	Pinal Trivedi, Maharshi Pandya, Nidhi Patel, Nitin Savaliya, Raghawendra Kumar, Dinesh Kumar, Zuber Saiyed, Komal Patel, Labdhi Pandya, Afzal Ansari, Nikha Trivedi, Gauravi Dhruv, Arti Trivedi, Apurvasinh Puvar, Janvi Raval, Zarna Patel, Monika Gandhi, R D Dixit, A M Kadri, Harsh Bakshi, Chaitanya Joshi, Madhvi Joshi
EPI_ISL_483831	Pandit Deendayal Upadhyay Government Medical College, Rajkot	Gujarat Biotechnology Research Centre	Maharshi Pandya, Nidhi Patel, Nitin Savaliya, Raghawendra Kumar, Dinesh Kumar, Zuber Saiyed, Komal Patel, Labdhi Pandya, Afzal Ansari, Nikha Trivedi, Gauravi Dhruv, Arti Trivedi, Apurvasinh Puvar, Janvi Raval, Zarna Patel, Monika Gandhi, Pinal Trivedi, R D Dixit, A M Kadri, Harsh Bakshi, Chaitanya Joshi, Madhvi Joshi
EPI_ISL_483832	Pandit Deendayal Upadhyay Government Medical College, Rajkot	Gujarat Biotechnology Research Centre	Nidhi Patel, Nitin Savaliya, Raghawendra Kumar, Dinesh Kumar, Zuber Saiyed, Komal Patel, Labdhi Pandya, Afzal Ansari, Nikha Trivedi, Gauravi Dhruv, Arti Trivedi, Apurvasinh Puvar, Janvi Raval, Zarna Patel, Monika Gandhi, Pinal Trivedi, Maharshi Pandya, R D Dixit, A M Kadri, Harsh Bakshi, Chaitanya Joshi, Madhvi Joshi
EPI_ISL_483833	Pandit Deendayal Upadhyay Government Medical College, Rajkot	Gujarat Biotechnology Research Centre	Nitin Savaliya, Raghawendra Kumar, Dinesh Kumar, Zuber Saiyed, Komal Patel, Labdhi Pandya, Afzal Ansari, Nikha Trivedi, Gauravi Dhruv, Arti Trivedi, Apurvasinh Puvar, Janvi Raval, Zarna Patel, Monika Gandhi, Pinal Trivedi, Maharshi Pandya, Nidhi Patel, R D Dixit, A M Kadri, Harsh Bakshi, Chaitanya Joshi, Madhvi Joshi
EPI_ISL_483834	Pandit Deendayal Upadhyay Government Medical College, Rajkot	Gujarat Biotechnology Research Centre	Raghawendra Kumar, Dinesh Kumar, Zuber Saiyed, Komal Patel, Labdhi Pandya, Afzal Ansari, Nikha Trivedi, Gauravi Dhruv, Arti Trivedi, Apurvasinh Puvar, Janvi Raval, Zarna Patel, Monika Gandhi, Pinal Trivedi, Maharshi Pandya, Nidhi Patel, Nitin Savaliya, R D Dixit, A M Kadri, Harsh Bakshi, Chaitanya Joshi, Madhvi Joshi
EPI_ISL_483835	Pandit Deendayal Upadhyay Government Medical College, Rajkot	Gujarat Biotechnology Research Centre	Dinesh Kumar, Zuber Saiyed, Komal Patel, Labdhi Pandya, Afzal Ansari, Nikha Trivedi, Gauravi Dhruv, Arti Trivedi, Apurvasinh Puvar, Janvi Raval, Zarna Patel, Monika Gandhi, Pinal Trivedi, Maharshi Pandya, Nidhi Patel, Nitin Savaliya, Raghawendra Kumar, R D Dixit, A M Kadri, Harsh Bakshi, Chaitanya Joshi, Madhvi Joshi
EPI_ISL_483836	Pandit Deendayal Upadhyay Government Medical College, Rajkot	Gujarat Biotechnology Research Centre	Zuber Saiyed, Komal Patel, Labdhi Pandya, Afzal Ansari, Nikha Trivedi, Gauravi Dhruv, Arti Trivedi, Apurvasinh Puvar, Janvi Raval, Zarna Patel, Monika Gandhi, Pinal Trivedi, Maharshi Pandya, Nidhi Patel, Nitin Savaliya, Raghawendra Kumar, Dinesh Kumar, R D Dixit, A M Kadri, Harsh Bakshi, Chaitanya Joshi, Madhvi Joshi
EPI_ISL_483837	Pandit Deendayal Upadhyay Government Medical College, Rajkot	Gujarat Biotechnology Research Centre	Komal Patel, Labdhi Pandya, Afzal Ansari, Nikha Trivedi, Gauravi Dhruv, Arti Trivedi, Apurvasinh Puvar, Janvi Raval, Zarna Patel, Monika Gandhi, Pinal Trivedi, Maharshi Pandya, Nidhi Patel, Nitin Savaliya, Raghawendra Kumar, Dinesh Kumar, Zuber Saiyed, R D Dixit, A M Kadri, Harsh Bakshi, Chaitanya Joshi, Madhvi Joshi
EPI_ISL_483838	Pandit Deendayal Upadhyay Government Medical College, Rajkot	Gujarat Biotechnology Research Centre	Labdhi Pandya, Afzal Ansari, Nikha Trivedi, Gauravi Dhruv, Arti Trivedi, Apurvasinh Puvar, Janvi Raval, Zarna Patel, Monika Gandhi, Pinal Trivedi, Maharshi Pandya, Nidhi Patel, Nitin Savaliya, Raghawendra Kumar, Dinesh Kumar, Zuber Saiyed, Komal Patel, R D Dixit, A M Kadri, Harsh Bakshi, Chaitanya Joshi, Madhvi Joshi
EPI_ISL_483839	Pandit Deendayal Upadhyay Government Medical College, Rajkot	Gujarat Biotechnology Research Centre	Afzal Ansari, Nikha Trivedi, Gauravi Dhruv, Arti Trivedi, Apurvasinh Puvar, Janvi Raval, Zarna Patel, Monika Gandhi, Pinal Trivedi, Maharshi Pandya, Nidhi Patel, Nitin Savaliya, Raghawendra Kumar, Dinesh Kumar, Zuber Saiyed, Komal Patel, Labdhi Pandya, R D Dixit, A M Kadri, Harsh Bakshi, Chaitanya Joshi, Madhvi Joshi
EPI_ISL_483840	Pandit Deendayal Upadhyay Government Medical College, Rajkot	Gujarat Biotechnology Research Centre	Nikha Trivedi, Gauravi Dhruv, Arti Trivedi, Apurvasinh Puvar, Janvi Raval, Zarna Patel, Monika Gandhi, Pinal Trivedi, Maharshi Pandya, Nidhi Patel, Nitin Savaliya, Raghawendra Kumar, Dinesh Kumar, Zuber Saiyed, Komal Patel, Labdhi Pandya, Afzal Ansari, R D Dixit, A M Kadri, Harsh Bakshi, Chaitanya Joshi, Madhvi Joshi
EPI_ISL_483841	Pandit Deendayal Upadhyay Government Medical College, Rajkot	Gujarat Biotechnology Research Centre	Gauravi Dhruv, Arti Trivedi, Apurvasinh Puvar, Janvi Raval, Zarna Patel, Monika Gandhi, Pinal Trivedi, Maharshi Pandya, Nidhi Patel, Nitin Savaliya, Raghawendra Kumar, Dinesh Kumar, Zuber Saiyed, Komal Patel, Labdhi Pandya, Afzal Ansari, Nikha Trivedi, R D Dixit, A M Kadri, Harsh Bakshi, Chaitanya Joshi, Madhvi Joshi
EPI_ISL_483842	Pandit Deendayal Upadhyay Government Medical College, Rajkot	Gujarat Biotechnology Research Centre	Arti Trivedi, Apurvasinh Puvar, Janvi Raval, Zarna Patel, Monika Gandhi, Pinal Trivedi, Maharshi Pandya, Nidhi Patel, Nitin Savaliya, Raghawendra Kumar, Dinesh Kumar, Zuber Saiyed, Komal Patel, Labdhi Pandya, Afzal Ansari, Nikha Trivedi, Gauravi Dhruv, R D Dixit, A M Kadri, Harsh Bakshi, Chaitanya Joshi, Madhvi Joshi
EPI_ISL_483843	Pandit Deendayal Upadhyay Government Medical College, Rajkot	Gujarat Biotechnology Research Centre	Apurvasinh Puvar, Janvi Raval, Zarna Patel, Monika Gandhi, Pinal Trivedi, Maharshi Pandya, Nidhi Patel, Nitin Savaliya, Raghawendra Kumar, Dinesh Kumar, Zuber Saiyed, Komal Patel, Labdhi Pandya, Afzal Ansari, Nikha Trivedi, Gauravi Dhruv, Arti Trivedi, R D Dixit, A M Kadri, Harsh Bakshi, Chaitanya Joshi, Madhvi Joshi
EPI_ISL_483844	Pandit Deendayal Upadhyay Government Medical College, Rajkot	Gujarat Biotechnology Research Centre	Janvi Raval, Zarna Patel, Monika Gandhi, Pinal Trivedi, Maharshi Pandya, Nidhi Patel, Nitin Savaliya, Raghawendra Kumar, Dinesh Kumar, Zuber Saiyed, Komal Patel, Labdhi Pandya, Afzal Ansari, Nikha Trivedi, Gauravi Dhruv, Arti Trivedi, Apurvasinh Puvar, R D Dixit, A M Kadri, Harsh Bakshi, Chaitanya Joshi, Madhvi Joshi
EPI_ISL_483845	Pandit Deendayal Upadhyay Government Medical College, Rajkot	Gujarat Biotechnology Research Centre	Zarna Patel, Monika Gandhi, Pinal Trivedi, Maharshi Pandya, Nidhi Patel, Nitin Savaliya, Raghawendra Kumar, Dinesh Kumar, Zuber Saiyed, Komal Patel, Labdhi Pandya, Afzal Ansari, Nikha Trivedi, Gauravi Dhruv, Arti Trivedi, Apurvasinh Puvar, Janvi Raval, R D Dixit, A M Kadri, Harsh Bakshi, Chaitanya Joshi, Madhvi Joshi
EPI_ISL_483846	Pandit Deendayal Upadhyay Government Medical College, Rajkot	Gujarat Biotechnology Research Centre	Monika Gandhi, Pinal Trivedi, Maharshi Pandya, Nidhi Patel, Nitin Savaliya, Raghawendra Kumar, Dinesh Kumar, Zuber Saiyed, Komal Patel, Labdhi Pandya, Afzal Ansari, Nikha Trivedi, Gauravi Dhruv, Arti Trivedi, Apurvasinh Puvar, Janvi Raval, Zarna Patel, R D Dixit, A M Kadri, Harsh Bakshi, Chaitanya Joshi, Madhvi Joshi
EPI_ISL_483847	Pandit Deendayal Upadhyay Government Medical College, Rajkot	Gujarat Biotechnology Research Centre	Pinal Trivedi, Maharshi Pandya, Nidhi Patel, Nitin Savaliya, Raghawendra Kumar, Dinesh Kumar, Zuber Saiyed, Komal Patel, Labdhi Pandya, Afzal Ansari, Nikha Trivedi, Gauravi Dhruv, Arti Trivedi, Apurvasinh Puvar, Janvi Raval, Zarna Patel, Monika Gandhi, R D Dixit, A M Kadri, Harsh Bakshi, Chaitanya Joshi, Madhvi Joshi
EPI_ISL_483848	Pandit Deendayal Upadhyay Government Medical College, Rajkot	Gujarat Biotechnology Research Centre	Maharshi Pandya, Nidhi Patel, Nitin Savaliya, Raghawendra Kumar, Dinesh Kumar, Zuber Saiyed, Komal Patel, Labdhi Pandya, Afzal Ansari, Nikha Trivedi, Gauravi Dhruv, Arti Trivedi, Apurvasinh Puvar, Janvi Raval, Zarna Patel, Monika Gandhi, Pinal Trivedi, R D Dixit, A M Kadri, Harsh Bakshi, Chaitanya Joshi, Madhvi Joshi

EPI_ISL_483849	Pandit Deendayal Upadhyay Government Medical College, Rajkot	Gujarat Biotechnology Research Centre	Nidhi Patel, Nitin Savaliya, Raghawendra Kumar, Dinesh Kumar, Zuber Saiyed, Komal Patel, Labdhi Pandya, Afzal Ansari, Nikha Trivedi, Gauravi Dhruv, Arti Trivedi, Apurvasinh Puvar, Janvi Raval, Zarna Patel, Monika Gandhi, Pinal Trivedi, Maharshi Pandya, R D Dixit, A M Kadri, Harsh Bakshi, Chaitanya Joshi, Madhvi Joshi
EPI_ISL_483850	Department of Microbiology, Government Medical College, Surat	Gujarat Biotechnology Research Centre	Nitin Savaliya, Raghawendra Kumar, Dinesh Kumar, Zuber Saiyed, Komal Patel, Labdhi Pandya, Afzal Ansari, Nikha Trivedi, Naresh Chauhan, Summaiya Mullan, Amit gamit, Apurvasinh Puvar, Janvi Raval, Zarna Patel, Monika Gandhi, Pinal Trivedi, Maharshi Pandya, Nidhi Patel, R D Dixit, A M Kadri, Harsh Bakshi, Chaitanya Joshi, Madhvi Joshi
EPI_ISL_483851	Department of Microbiology, Government Medical College, Surat	Gujarat Biotechnology Research Centre	Raghawendra Kumar, Dinesh Kumar, Zuber Saiyed, Komal Patel, Labdhi Pandya, Afzal Ansari, Nikha Trivedi, Naresh Chauhan, Summaiya Mullan, Amit gamit, Apurvasinh Puvar, Janvi Raval, Zarna Patel, Monika Gandhi, Pinal Trivedi, Maharshi Pandya, Nidhi Patel, Nitin Savaliya, R D Dixit, A M Kadri, Harsh Bakshi, Chaitanya Joshi, Madhvi Joshi
EPI_ISL_483852	Department of Microbiology, Government Medical College, Surat	Gujarat Biotechnology Research Centre	Dinesh Kumar, Zuber Saiyed, Komal Patel, Labdhi Pandya, Afzal Ansari, Nikha Trivedi, Naresh Chauhan, Summaiya Mullan, Amit gamit, Apurvasinh Puvar, Janvi Raval, Zarna Patel, Monika Gandhi, Pinal Trivedi, Maharshi Pandya, Nidhi Patel, Nitin Savaliya, Raghawendra Kumar, R D Dixit, A M Kadri, Harsh Bakshi, Chaitanya Joshi, Madhvi Joshi
EPI_ISL_483853	Department of Microbiology, Government Medical College, Surat	Gujarat Biotechnology Research Centre	Zuber Saiyed, Komal Patel, Labdhi Pandya, Afzal Ansari, Nikha Trivedi, Naresh Chauhan, Summaiya Mullan, Amit gamit, Apurvasinh Puvar, Janvi Raval, Zarna Patel, Monika Gandhi, Pinal Trivedi, Maharshi Pandya, Nidhi Patel, Nitin Savaliya, Raghawendra Kumar, Dinesh Kumar, R D Dixit, A M Kadri, Harsh Bakshi, Chaitanya Joshi, Madhvi Joshi
EPI_ISL_483854	Department of Microbiology, Government Medical College, Surat	Gujarat Biotechnology Research Centre	Komal Patel, Labdhi Pandya, Afzal Ansari, Nikha Trivedi, Naresh Chauhan, Summaiya Mullan, Amit gamit, Apurvasinh Puvar, Janvi Raval, Zarna Patel, Monika Gandhi, Pinal Trivedi, Maharshi Pandya, Nidhi Patel, Nitin Savaliya, Raghawendra Kumar, Dinesh Kumar, Zuber Saiyed, R D Dixit, A M Kadri, Harsh Bakshi, Chaitanya Joshi, Madhvi Joshi
EPI_ISL_483855	Department of Microbiology, Government Medical College, Surat	Gujarat Biotechnology Research Centre	Labdhi Pandya, Afzal Ansari, Nikha Trivedi, Naresh Chauhan, Summaiya Mullan, Amit gamit, Apurvasinh Puvar, Janvi Raval, Zarna Patel, Monika Gandhi, Pinal Trivedi, Maharshi Pandya, Nidhi Patel, Nitin Savaliya, Raghawendra Kumar, Dinesh Kumar, Zuber Saiyed, Komal Patel, R D Dixit, A M Kadri, Harsh Bakshi, Chaitanya Joshi, Madhvi Joshi
EPI_ISL_483856	Department of Microbiology, Government Medical College, Surat	Gujarat Biotechnology Research Centre	Afzal Ansari, Nikha Trivedi, Naresh Chauhan, Summaiya Mullan, Amit gamit, Apurvasinh Puvar, Janvi Raval, Zarna Patel, Monika Gandhi, Pinal Trivedi, Maharshi Pandya, Nidhi Patel, Nitin Savaliya, Raghawendra Kumar, Dinesh Kumar, Zuber Saiyed, Komal Patel, Labdhi Pandya, R D Dixit, A M Kadri, Harsh Bakshi, Chaitanya Joshi, Madhvi Joshi
EPI_ISL_483857	Department of Microbiology, Government Medical College, Surat	Gujarat Biotechnology Research Centre	Nikha Trivedi, Naresh Chauhan, Summaiya Mullan, Amit gamit, Apurvasinh Puvar, Janvi Raval, Zarna Patel, Monika Gandhi, Pinal Trivedi, Maharshi Pandya, Nidhi Patel, Nitin Savaliya, Raghawendra Kumar, Dinesh Kumar, Zuber Saiyed, Komal Patel, Labdhi Pandya, Afzal Ansari, R D Dixit, A M Kadri, Harsh Bakshi, Chaitanya Joshi, Madhvi Joshi
EPI_ISL_483858	Department of Microbiology, Government Medical College, Surat	Gujarat Biotechnology Research Centre	Naresh Chauhan, Summaiya Mullan, Amit gamit, Apurvasinh Puvar, Janvi Raval, Zarna Patel, Monika Gandhi, Pinal Trivedi, Maharshi Pandya, Nidhi Patel, Nitin Savaliya, Raghawendra Kumar, Dinesh Kumar, Zuber Saiyed, Komal Patel, Labdhi Pandya, Afzal Ansari, Nikha Trivedi, R D Dixit, A M Kadri, Harsh Bakshi, Chaitanya Joshi, Madhvi Joshi
EPI_ISL_483859	Department of Microbiology, Government Medical College, Surat	Gujarat Biotechnology Research Centre	Summaiya Mullan, Amit gamit, Apurvasinh Puvar, Janvi Raval, Zarna Patel, Monika Gandhi, Pinal Trivedi, Maharshi Pandya, Nidhi Patel, Nitin Savaliya, Raghawendra Kumar, Dinesh Kumar, Zuber Saiyed, Komal Patel, Labdhi Pandya, Afzal Ansari, Nikha Trivedi, Naresh Chauhan, R D Dixit, A M Kadri, Harsh Bakshi, Chaitanya Joshi, Madhvi Joshi
EPI_ISL_483860	Department of Microbiology, Government Medical College, Surat	Gujarat Biotechnology Research Centre	Amit gamit, Apurvasinh Puvar, Janvi Raval, Zarna Patel, Monika Gandhi, Pinal Trivedi, Maharshi Pandya, Nidhi Patel, Nitin Savaliya, Raghawendra Kumar, Dinesh Kumar, Zuber Saiyed, Komal Patel, Labdhi Pandya, Afzal Ansari, Nikha Trivedi, Naresh Chauhan, Summaiya Mullan, R D Dixit, A M Kadri, Harsh Bakshi, Chaitanya Joshi, Madhvi Joshi
EPI_ISL_483861	Department of Microbiology, Government Medical College, Surat	Gujarat Biotechnology Research Centre	Apurvasinh Puvar, Janvi Raval, Zarna Patel, Monika Gandhi, Pinal Trivedi, Maharshi Pandya, Nidhi Patel, Nitin Savaliya, Raghawendra Kumar, Dinesh Kumar, Zuber Saiyed, Komal Patel, Labdhi Pandya, Afzal Ansari, Nikha Trivedi, Naresh Chauhan, Summaiya Mullan, Amit gamit, R D Dixit, A M Kadri, Harsh Bakshi, Chaitanya Joshi, Madhvi Joshi
EPI_ISL_483862	Department of Microbiology, Government Medical College, Surat	Gujarat Biotechnology Research Centre	Janvi Raval, Zarna Patel, Monika Gandhi, Pinal Trivedi, Maharshi Pandya, Nidhi Patel, Nitin Savaliya, Raghawendra Kumar, Dinesh Kumar, Zuber Saiyed, Komal Patel, Labdhi Pandya, Afzal Ansari, Nikha Trivedi, Naresh Chauhan, Summaiya Mullan, Amit gamit, Apurvasinh Puvar, R D Dixit, A M Kadri, Harsh Bakshi, Chaitanya Joshi, Madhvi Joshi
EPI_ISL_483863	Department of Microbiology, Government Medical College, Surat	Gujarat Biotechnology Research Centre	Zarna Patel, Monika Gandhi, Pinal Trivedi, Maharshi Pandya, Nidhi Patel, Nitin Savaliya, Raghawendra Kumar, Dinesh Kumar, Zuber Saiyed, Komal Patel, Labdhi Pandya, Afzal Ansari, Nikha Trivedi, Naresh Chauhan, Summaiya Mullan, Amit gamit, Apurvasinh Puvar, Janvi Raval, R D Dixit, A M Kadri, Harsh Bakshi, Chaitanya Joshi, Madhvi Joshi
EPI_ISL_483864	Department of Microbiology, Government Medical College, Surat	Gujarat Biotechnology Research Centre	Monika Gandhi, Pinal Trivedi, Maharshi Pandya, Nidhi Patel, Nitin Savaliya, Raghawendra Kumar, Dinesh Kumar, Zuber Saiyed, Komal Patel, Labdhi Pandya, Afzal Ansari, Nikha Trivedi, Naresh Chauhan, Summaiya Mullan, Amit gamit, Apurvasinh Puvar, Janvi Raval, Zarna Patel, R D Dixit, A M Kadri, Harsh Bakshi, Chaitanya Joshi, Madhvi Joshi
EPI_ISL_483865	Department of Microbiology, Government Medical College, Surat	Gujarat Biotechnology Research Centre	Pinal Trivedi, Maharshi Pandya, Nidhi Patel, Nitin Savaliya, Raghawendra Kumar, Dinesh Kumar, Zuber Saiyed, Komal Patel, Labdhi Pandya, Afzal Ansari, Nikha Trivedi, Naresh Chauhan, Summaiya Mullan, Amit gamit, Apurvasinh Puvar, Janvi Raval, Zarna Patel, Monika Gandhi, R D Dixit, A M Kadri, Harsh Bakshi, Chaitanya Joshi, Madhvi Joshi
EPI_ISL_483866	Department of Microbiology, Government Medical College, Surat	Gujarat Biotechnology Research Centre	Maharshi Pandya, Nidhi Patel, Nitin Savaliya, Raghawendra Kumar, Dinesh Kumar, Zuber Saiyed, Komal Patel, Labdhi Pandya, Afzal Ansari, Nikha Trivedi, Naresh Chauhan, Summaiya Mullan, Amit gamit, Apurvasinh Puvar, Janvi Raval, Zarna Patel, Monika Gandhi, Pinal Trivedi, R D Dixit, A M Kadri, Harsh Bakshi, Chaitanya Joshi, Madhvi Joshi
EPI_ISL_483867	Department of Microbiology, Government Medical College, Surat	Gujarat Biotechnology Research Centre	Nidhi Patel, Nitin Savaliya, Raghawendra Kumar, Dinesh Kumar, Zuber Saiyed, Komal Patel, Labdhi Pandya, Afzal Ansari, Nikha Trivedi, Naresh Chauhan, Summaiya Mullan, Amit gamit, Apurvasinh Puvar, Janvi Raval, Zarna Patel, Monika Gandhi, Pinal Trivedi, Maharshi Pandya, R D Dixit, A M Kadri, Harsh Bakshi, Chaitanya Joshi, Madhvi Joshi
EPI_ISL_483868	Department of Microbiology, Government Medical College, Surat	Gujarat Biotechnology Research Centre	Nitin Savaliya, Raghawendra Kumar, Dinesh Kumar, Zuber Saiyed, Komal Patel, Labdhi Pandya, Afzal Ansari, Nikha Trivedi, Naresh Chauhan, Summaiya Mullan, Amit gamit, Apurvasinh Puvar, Janvi Raval, Zarna Patel, Monika Gandhi, Pinal Trivedi, Maharshi Pandya, Nidhi Patel, R D Dixit, A M Kadri, Harsh Bakshi, Chaitanya Joshi, Madhvi Joshi
EPI_ISL_483869	Department of Microbiology, Government Medical College, Surat	Gujarat Biotechnology Research Centre	Raghawendra Kumar, Dinesh Kumar, Zuber Saiyed, Komal Patel, Labdhi Pandya, Afzal Ansari, Nikha Trivedi, Naresh Chauhan, Summaiya Mullan, Amit gamit, Apurvasinh Puvar, Janvi Raval, Zarna Patel, Monika Gandhi, Pinal Trivedi, Maharshi Pandya, Nidhi Patel, Nitin Savaliya, R D Dixit, A M Kadri, Harsh Bakshi, Chaitanya Joshi, Madhvi Joshi
EPI_ISL_483870	Department of Microbiology, Government Medical College, Surat	Gujarat Biotechnology Research Centre	Dinesh Kumar, Zuber Saiyed, Komal Patel, Labdhi Pandya, Afzal Ansari, Nikha Trivedi, Naresh Chauhan, Summaiya Mullan, Amit gamit, Apurvasinh Puvar, Janvi Raval, Zarna Patel, Monika Gandhi, Pinal Trivedi, Maharshi Pandya, Nidhi Patel, Nitin Savaliya, Raghawendra Kumar, R D Dixit, A M Kadri, Harsh Bakshi, Chaitanya Joshi, Madhvi Joshi
EPI_ISL_483871	Department of Microbiology, Government Medical College, Surat	Gujarat Biotechnology Research Centre	Zuber Saiyed, Komal Patel, Labdhi Pandya, Afzal Ansari, Nikha Trivedi, Naresh Chauhan, Summaiya Mullan, Amit gamit, Apurvasinh Puvar, Janvi Raval, Zarna Patel, Monika Gandhi, Pinal Trivedi, Maharshi Pandya, Nidhi Patel, Nitin Savaliya, Raghawendra Kumar, Dinesh Kumar, R D Dixit, A M Kadri, Harsh Bakshi, Chaitanya Joshi, Madhvi Joshi
EPI_ISL_483872	Department of Microbiology, Government Medical College, Surat	Gujarat Biotechnology Research Centre	Komal Patel, Labdhi Pandya, Afzal Ansari, Nikha Trivedi, Naresh Chauhan, Summaiya Mullan, Amit gamit, Apurvasinh Puvar, Janvi Raval, Zarna Patel, Monika Gandhi, Pinal Trivedi, Maharshi Pandya, Nidhi Patel, Nitin Savaliya, Raghawendra Kumar, Dinesh Kumar, Zuber Saiyed, R D Dixit, A M Kadri, Harsh Bakshi, Chaitanya Joshi, Madhvi Joshi
EPI_ISL_483873	Department of Microbiology, Government Medical College, Surat	Gujarat Biotechnology Research Centre	Labdhi Pandya, Afzal Ansari, Nikha Trivedi, Naresh Chauhan, Summaiya Mullan, Amit gamit, Apurvasinh Puvar, Janvi Raval, Zarna Patel, Monika Gandhi, Pinal Trivedi, Maharshi Pandya, Nidhi Patel, Nitin Savaliya, Raghawendra Kumar, Dinesh Kumar, Zuber Saiyed, Komal Patel, R D Dixit, A M Kadri, Harsh Bakshi, Chaitanya Joshi, Madhvi Joshi

EPI_ISL_483874	Department of Microbiology, Government Medical College, Surat	Gujarat Biotechnology Research Centre	Afzal Ansari, Nikha Trivedi, Naresh Chauhan, Summaiya Mullan, Amit gamit, Apurvasinh Puvar, Janvi Raval, Zarna Patel, Monika Gandhi, Pinal Trivedi, Maharshi Pandya, Nidhi Patel, Nitin Savaliya, Raghawendra Kumar, Dinesh Kumar, Zuber Saiyed, Komal Patel, Labdhi Pandya, R D Dixit, A M Kadri, Harsh Bakshi, Chaitanya Joshi, Madhvi Joshi
EPI_ISL_483875	Department of Microbiology, Government Medical College, Surat	Gujarat Biotechnology Research Centre	Nikha Trivedi, Naresh Chauhan, Summaiya Mullan, Amit gamit, Apurvasinh Puvar, Janvi Raval, Zarna Patel, Monika Gandhi, Pinal Trivedi, Maharshi Pandya, Nidhi Patel, Nitin Savaliya, Raghawendra Kumar, Dinesh Kumar, Zuber Saiyed, Komal Patel, Labdhi Pandya, Afzal Ansari, R D Dixit, A M Kadri, Harsh Bakshi, Chaitanya Joshi, Madhvi Joshi
EPI_ISL_483876	Department of Microbiology, Government Medical College, Surat	Gujarat Biotechnology Research Centre	Naresh Chauhan, Summaiya Mullan, Amit gamit, Apurvasinh Puvar, Janvi Raval, Zarna Patel, Monika Gandhi, Pinal Trivedi, Maharshi Pandya, Nidhi Patel, Nitin Savaliya, Raghawendra Kumar, Dinesh Kumar, Zuber Saiyed, Komal Patel, Labdhi Pandya, Afzal Ansari, Nikha Trivedi, R D Dixit, A M Kadri, Harsh Bakshi, Chaitanya Joshi, Madhvi Joshi
EPI_ISL_483877	Department of Microbiology, Government Medical College, Surat	Gujarat Biotechnology Research Centre	Summaiya Mullan, Amit gamit, Apurvasinh Puvar, Janvi Raval, Zarna Patel, Monika Gandhi, Pinal Trivedi, Maharshi Pandya, Nidhi Patel, Nitin Savaliya, Raghawendra Kumar, Dinesh Kumar, Zuber Saiyed, Komal Patel, Labdhi Pandya, Afzal Ansari, Nikha Trivedi, Naresh Chauhan, R D Dixit, A M Kadri, Harsh Bakshi, Chaitanya Joshi, Madhvi Joshi
EPI_ISL_483878	Department of Microbiology, Government Medical College, Surat	Gujarat Biotechnology Research Centre	Amit gamit, Apurvasinh Puvar, Janvi Raval, Zarna Patel, Monika Gandhi, Pinal Trivedi, Maharshi Pandya, Nidhi Patel, Nitin Savaliya, Raghawendra Kumar, Dinesh Kumar, Zuber Saiyed, Komal Patel, Labdhi Pandya, Afzal Ansari, Nikha Trivedi, Naresh Chauhan, Summaiya Mullan, R D Dixit, A M Kadri, Harsh Bakshi, Chaitanya Joshi, Madhvi Joshi
EPI_ISL_483879	Department of Microbiology, Government Medical College, Surat	Gujarat Biotechnology Research Centre	Apurvasinh Puvar, Janvi Raval, Zarna Patel, Monika Gandhi, Pinal Trivedi, Maharshi Pandya, Nidhi Patel, Nitin Savaliya, Raghawendra Kumar, Dinesh Kumar, Zuber Saiyed, Komal Patel, Labdhi Pandya, Afzal Ansari, Nikha Trivedi, Naresh Chauhan, Summaiya Mullan, Amit gamit, R D Dixit, A M Kadri, Harsh Bakshi, Chaitanya Joshi, Madhvi Joshi
EPI_ISL_485635, EPI_ISL_485708, EPI_ISL_485710	Institut Pasteur Dakar	Institut Pasteur de Dakar	Ndongo Dia, Moussa Moise Diagne, Mamadou diop, Marie Henriette Dior Ndione, Mamadou Malado Jallow, Safietou Sanke, Ousmane Faye, Amadou Alpha Sall.
EPI_ISL_485712	Institut Pasteur	Institut Pasteur de Dakar	Ndongo Dia, Moussa Moise Diagne, Mamadou diop, Marie Henriette Dior Ndione, Mamadou Malado Jallow, Safietou Sanke, Ousmane Faye, Amadou Alpha Sall.
EPI_ISL_485713, EPI_ISL_485715, EPI_ISL_485716, EPI_ISL_485717	Institut Pasteur Dakar	Institut Pasteur de Dakar	Ndongo Dia, Moussa Moise Diagne, Mamadou diop, Marie Henriette Dior Ndione, Mamadou Malado Jallow, Safietou Sanke, Ousmane Faye, Amadou Alpha Sall.
EPI_ISL_486834	Suceava County Energency Hospital "Sf. Ioan cel Nou"	SMU Metagenomics lab	Lobiuc Andrei, Antoniadis Panagiotis
EPI_ISL_486842, EPI_ISL_486843, EPI_ISL_486844	Institute of Microbiology, Universidad San Francisco de Quito	Institute of Microbiology, Universidad San Francisco de Quito	Belén Prado-Vivar, Sully Márquez, Juan José Guadalupe, Monica Becerra-Wong, Carla Torres, Bernardo Gutiérrez, Fausto Maldonado, Geovanny Carzola, Verónica Barragán, Patricio Rojas-Silva, Gabriel Trueba, Michelle Grunauer, Paúl Cárdenas
EPI_ISL_486845, EPI_ISL_486846, EPI_ISL_486847, EPI_ISL_486848, EPI_ISL_486849, EPI_ISL_486850, EPI_ISL_486851	Institute of Microbiology, Universidad San Francisco de Quito	Institute of Microbiology, Universidad San Francisco de Quito	Belén Prado-Vivar, Sully Márquez, Juan José Guadalupe, Monica Becerra-Wong, Carla Torres, Bernardo Gutiérrez, Jonathan Araujo, Verónica Barragán, Patricio Rojas-Silva, Gabriel Trueba, Michelle Grunauer, Paúl Cárdenas
EPI_ISL_486852	CDRI/SGPGI	CSIR-CDRI/SGPGI	Saumya Sarkar, Dharam Veer Singh, Rahul Vishvkarma, Ujjala Ghoshal, Uday Ghoshal, Ravishankar Ramachandran, Tapas Kumar Kundu, Rajender Singh
EPI_ISL_486853	CSIR-CDRI/SGPGI	CSIR-CDRI/SGPGI	Saumya Sarkar, Dharam Veer Singh, Rahul Vishvkarma, Ujjala Ghoshal, Uday Ghoshal, Ravishankar Ramachandran, Tapas Kumar Kundu, Rajender Singh
EPI_ISL_486854	Emergency County Hospital Suceava	Stefan cel Mare, University Metagenomics lab	Lobiuc Andrei et al.
EPI_ISL_486876	Clinical Microbiology Laboratory- Basurto University Hospital	Biocruces-Bizkaia	Mikel J. Urrutikoetxea-Gutierrez, Ana Belén Belén de la Hoz, Matxalen Vidal-García, Mº Carmen Nieto Toboso, Estibaliz Ugalde-Zarraga, José Luis Díaz de Tuesta del Arco
EPI_ISL_487277, EPI_ISL_487280, EPI_ISL_487281, EPI_ISL_487288, EPI_ISL_487295, EPI_ISL_487297, EPI_ISL_487304, EPI_ISL_487308, EPI_ISL_487311, EPI_ISL_487312, EPI_ISL_487313, EPI_ISL_487314, EPI_ISL_487316, EPI_ISL_487318, EPI_ISL_487319, EPI_ISL_487320, EPI_ISL_487321, EPI_ISL_487322, EPI_ISL_487324, EPI_ISL_487325, EPI_ISL_487328			
see above	NHLS-IALCH	KRISP, KZN Research Innovation and Sequencing Platform	Giandhari J, Pillay S, Lessells R, Chimukangara B, Mdlalose K, York D, Khan S, Tegally H, Wilkinson E, de Oliveira T
EPI_ISL_487329, EPI_ISL_487330, EPI_ISL_487332, EPI_ISL_487334, EPI_ISL_487336, EPI_ISL_487337, EPI_ISL_487338, EPI_ISL_487339, EPI_ISL_487340	Molecular Diagnostics Services (MDS)	KRISP, KZN Research Innovation and Sequencing Platform	Giandhari J, Pillay S, Lessells R, Chimukangara B, Mdlalose K, York D, Khan S, Tegally H, Wilkinson E, de Oliveira T
EPI_ISL_487432, EPI_ISL_487433, EPI_ISL_487434, EPI_ISL_487436	Queen Astrid Military Hospital	Institute of Tropical Medicine	Philippe Selhorst, Colin Anthony
EPI_ISL_489833, EPI_ISL_489834, EPI_ISL_489835	Clinical Microbiology Laboratory- Basurto University Hospital	Biocruces-Bizkaia	Mikel J. Urrutikoetxea-Gutierrez, Ana Belén Belén de la Hoz, Matxalen Vidal-García, Mº Carmen Nieto Toboso, Estibaliz Ugalde-Zarraga, José Luis Díaz de Tuesta del Arco
EPI_ISL_490101, EPI_ISL_490103	Institute for Medical Research, Infectious Disease Research Centre, National Institutes of Health, Ministry of Health Malaysia	Institute for Medical Research, Infectious Disease Research Centre, National Institutes of Health, Ministry of Health Malaysia	Suppiah J, Mohd-Zawawi Z, Kamel K, Kalyanasundram J, Thayan R
EPI_ISL_490202, EPI_ISL_490203, EPI_ISL_490204, EPI_ISL_490977	Clinical Microbiology Laboratory- Basurto University Hospital	Biocruces-Bizkaia	Mikel J. Urrutikoetxea-Gutierrez, Ana Belén Belén de la Hoz, Matxalen Vidal-García, Mº Carmen Nieto Toboso, Estibaliz Ugalde-Zarraga, José Luis Díaz de Tuesta del Arco
EPI_ISL_491976, EPI_ISL_491977, EPI_ISL_491978, EPI_ISL_491979, EPI_ISL_491980, EPI_ISL_491981, EPI_ISL_491982, EPI_ISL_491983, EPI_ISL_491984, EPI_ISL_491985, EPI_ISL_491986, EPI_ISL_491988, EPI_ISL_491989, EPI_ISL_491990, EPI_ISL_491991, EPI_ISL_491992, EPI_ISL_491994, EPI_ISL_491995, EPI_ISL_491996, EPI_ISL_491997, EPI_ISL_491998, EPI_ISL_491999, EPI_ISL_492000, EPI_ISL_492001, EPI_ISL_492002, EPI_ISL_492003, EPI_ISL_492005, EPI_ISL_492006, EPI_ISL_492007, EPI_ISL_492008, EPI_ISL_492009, EPI_ISL_492010, EPI_ISL_492011, EPI_ISL_492012, EPI_ISL_492014, EPI_ISL_492016, EPI_ISL_492017, EPI_ISL_492019, EPI_ISL_492020, EPI_ISL_492021, EPI_ISL_492022, EPI_ISL_492023, EPI_ISL_492024, EPI_ISL_492025, EPI_ISL_492026			
see above	Oman-NIC	Department of Microbiology and Immunology-SQUH	Fahad Zadjali, Samira Al-Maruqi, Amina Al Jardani, Khulood Al-Mammary, Hanan Al-kindi, Fatma BaAlawi, Hamida AL Barwani, Zeyana AL-Dahmani, Intisar Al-Shukri, Aisha Al-Busaidi, Aisha Al-Amri, Ahlam Al-Amri, Mohammed Al-Tobi, Samiha Al Kharusi, Abdulla Balkhair
EPI_ISL_492035	Instituto de Biologia do Exército	Laboratório Metabolismo Macromolecular FirminoTorres de Castro, Instituto de Biofísica Carlos Chagas Filho, Universidade Federal do Rio de Janeiro	Bianca Catarina Azevedo Cabral, Aline Rosa Vianna de Souza, Tatiana LS Nogueira, Nádia Vaez Gonçalves da Cruz, Caleb GM Santos, Marcos Dornelas-Ribeiro, Elizabeth Valentin, Marcio da Costa Cipitelli, Virginia Sara Grancieri do Amaral, Rodrigo Soares de Moura Neto, Clarissa Damaso, Rosane Silva
EPI_ISL_492036	Instituto de Biologia do Exército	Laboratório Metabolismo Macromolecular FirminoTorres de Castro, Instituto de Biofísica Carlos Chagas Filho, Universidade Federal do Rio de Janeiro	Bianca Catarina Azevedo Cabral, Aline Rosa Vianna de Souza , Marcos Dornelas-Ribeiro, Tatiana LS Nogueira, Nádia Vaez Gonçalves da Cruz, Caleb GM Santos, Elizabeth Valentin, Marcio da Costa Cipitelli, Virginia Sara Grancieri do Amaral, Rodrigo Soares de Moura Neto, Clarissa Damaso, Rosane Silva
EPI_ISL_492043	Instituto de Biologia do Exército	Laboratório Metabolismo Macromolecular FirminoTorres de Castro, Instituto de Biofísica Carlos Chagas Filho, Universidade Federal do Rio de Janeiro	Bianca Catarina Azevedo Cabral, Aline Rosa Vianna de Souza, Tatiana LS Nogueira, Nádia Vaez Gonçalves da Cruz, Caleb GM Santos, Marcos Dornelas-Ribeiro, Elizabeth Valentin, Marcio da Costa Cipitelli, Virginia Sara Grancieri do Amaral, Rodrigo Soares de Moura Neto, Clarissa Damaso, Rosane Silva
EPI_ISL_492044	Instituto de Biologia do Exército	Laboratório Metabolismo Macromolecular FirminoTorres de Castro, Instituto de Biofísica Carlos Chagas Filho, Universidade Federal do Rio de Janeiro	Bianca Catarina Azevedo Cabral, Aline Rosa Vianna de Souza , Marcos Dornelas-Ribeiro, Tatiana LS Nogueira, Nádia Vaez Gonçalves da Cruz, Caleb GM Santos, Elizabeth Valentin, Marcio da Costa Cipitelli, Virginia Sara Grancieri do Amaral, Rodrigo Soares de Moura Neto, Clarissa Damaso, Rosane Silva
EPI_ISL_492045	Instituto de Biologia do Exército	Laboratório Metabolismo Macromolecular FirminoTorres de Castro, Instituto de Biofísica Carlos Chagas Filho, Universidade Federal do Rio de Janeiro	Bianca Catarina Azevedo Cabral, Aline Rosa Vianna de Souza, Caleb GM Santos, Marcos Dornelas-Ribeiro, Tatiana LS Nogueira, Nádia Vaez Gonçalves da Cruz, Elizabeth Valentin, Marcio da Costa Cipitelli, Virginia Sara Grancieri do Amaral, Rodrigo Soares de Moura Neto, Clarissa Damaso, Rosane Silva

EPI_ISL_492048	Instituto de Biologia do Exército	Laboratório Metabolismo Macromolecular FirminoTorres de Castro, Instituto de Biofísica Carlos Chagas Filho, Universidade Federal do Rio de Janeiro	Bianca Catarina Azevedo Cabral, Aline Rosa Vianna de Souza , Marcos Dornelas-Ribeiro, Tatiana LS Nogueira, Nádia Vaez Gonçalves da Cruz, Caleb GM Santos, Elizabeth Valentin, Marcio da Costa Cipitelli, Virginia Sara Grancieri do Amaral, Rodrigo Soares de Moura Neto, Clarissa Damaso, Rosane Silva
EPI_ISL_493346, EPI_ISL_493348	Instituto de Diagnostico y Referencia Epidemiologicos (INDRE)	Instituto de Diagnostico y Referencia Epidemiologicos (INDRE)	Ernesto Ramirez-Gonzalez, Abril Rodriguez-Maldonado, Claudia Wong-Arambula , Natividad Cruz-Ortiz, Tatiana Nunez-Garcia, Dayanira Arellano-Suarez, Adnan Araiza-Rodriguez, Fabiola Garces-Ayala, Lucia Hernandez-Rivas, Irma Lopez-Martinez, Gisela Barrera-Badillo.
EPI_ISL_495014	B.J. Medical College and Civil hospital	Gujarat Biotechnology Research Centre	Janvi Raval, Zarna Patel, Monika Gandhi, Pinal Trivedi, Maharshi Pandya, Nidhi Patel, Nitin Savaliya, Raghawendra Kumar, Dinesh Kumar, Zuber Saiyed, Komal Patel, Labdhi Pandya, Afzal Ansari, Nikha Trivedi, Pranay Shah, Kamlesh J Upadhyay, Sanjay Kapadia, Apurvasinh Puvar, R D Dixit, A M Kadri, Harsh Bakshi, Chaitanya Joshi, Madhvi Joshi
EPI_ISL_495015	B.J. Medical College and Civil hospital	Gujarat Biotechnology Research Centre	Zarna Patel, Monika Gandhi, Pinal Trivedi, Maharshi Pandya, Nidhi Patel, Nitin Savaliya, Raghawendra Kumar, Dinesh Kumar, Zuber Saiyed, Komal Patel, Labdhi Pandya, Afzal Ansari, Nikha Trivedi, Pranay Shah, Kamlesh J Upadhyay, Sanjay Kapadia, Apurvasinh Puvar, Janvi Raval, R D Dixit, A M Kadri, Harsh Bakshi, Chaitanya Joshi, Madhvi Joshi
EPI_ISL_495016	B.J. Medical College and Civil hospital	Gujarat Biotechnology Research Centre	Monika Gandhi, Pinal Trivedi, Maharshi Pandya, Nidhi Patel, Nitin Savaliya, Raghawendra Kumar, Dinesh Kumar, Zuber Saiyed, Komal Patel, Labdhi Pandya, Afzal Ansari, Nikha Trivedi, Pranay Shah, Kamlesh J Upadhyay, Sanjay Kapadia, Apurvasinh Puvar, Janvi Raval, Zarna Patel, R D Dixit, A M Kadri, Harsh Bakshi, Chaitanya Joshi, Madhvi Joshi
EPI_ISL_495017	B.J. Medical College and Civil hospital	Gujarat Biotechnology Research Centre	Pinal Trivedi, Maharshi Pandya, Nidhi Patel, Nitin Savaliya, Raghawendra Kumar, Dinesh Kumar, Zuber Saiyed, Komal Patel, Labdhi Pandya, Afzal Ansari, Nikha Trivedi, Pranay Shah, Kamlesh J Upadhyay, Sanjay Kapadia, Apurvasinh Puvar, Janvi Raval, Zarna Patel, Monika Gandhi, R D Dixit, A M Kadri, Harsh Bakshi, Chaitanya Joshi, Madhvi Joshi
EPI_ISL_495018	B.J. Medical College and Civil hospital	Gujarat Biotechnology Research Centre	Maharshi Pandya, Nidhi Patel, Nitin Savaliya, Raghawendra Kumar, Dinesh Kumar, Zuber Saiyed, Komal Patel, Labdhi Pandya, Afzal Ansari, Nikha Trivedi, Pranay Shah, Kamlesh J Upadhyay, Sanjay Kapadia, Apurvasinh Puvar, Janvi Raval, Zarna Patel, Monika Gandhi, Pinal Trivedi, R D Dixit, A M Kadri, Harsh Bakshi, Chaitanya Joshi, Madhvi Joshi
EPI_ISL_495019	B.J. Medical College and Civil hospital	Gujarat Biotechnology Research Centre	Nidhi Patel, Nitin Savaliya, Raghawendra Kumar, Dinesh Kumar, Zuber Saiyed, Komal Patel, Labdhi Pandya, Afzal Ansari, Nikha Trivedi, Pranay Shah, Kamlesh J Upadhyay, Sanjay Kapadia, Apurvasinh Puvar, Janvi Raval, Zarna Patel, Monika Gandhi, Pinal Trivedi, Maharshi Pandya, R D Dixit, A M Kadri, Harsh Bakshi, Chaitanya Joshi, Madhvi Joshi
EPI_ISL_495020	Government Medical College, Bhavnagar	Gujarat Biotechnology Research Centre	Kairavi Desai, Saklin Malek, Shirish Patel, Nitin Savaliya, Raghawendra Kumar, Dinesh Kumar, Zuber Saiyed, Komal Patel, Labdhi Pandya, Afzal Ansari, Nikha Trivedi, Apurvasinh Puvar, Janvi Raval, Zarna Patel, Monika Gandhi, Pinal Trivedi, Maharshi Pandya, Nidhi Patel, R D Dixit, A M Kadri, Harsh Bakshi, Chaitanya Joshi, Madhvi Joshi
EPI_ISL_495021	Government Medical College, Bhavnagar	Gujarat Biotechnology Research Centre	Saklin Malek, Shirish Patel, Kairavi Desai, Raghawendra Kumar, Dinesh Kumar, Zuber Saiyed, Komal Patel, Labdhi Pandya, Afzal Ansari, Nikha Trivedi, Apurvasinh Puvar, Janvi Raval, Zarna Patel, Monika Gandhi, Pinal Trivedi, Maharshi Pandya, Nidhi Patel, Nitin Savaliya, R D Dixit, A M Kadri, Harsh Bakshi, Chaitanya Joshi, Madhvi Joshi
EPI_ISL_495022	Government Medical College, Bhavnagar	Gujarat Biotechnology Research Centre	Shirish Patel, Kairavi Desai, Saklin Malek, Dinesh Kumar, Zuber Saiyed, Komal Patel, Labdhi Pandya, Afzal Ansari, Nikha Trivedi, Apurvasinh Puvar, Janvi Raval, Zarna Patel, Monika Gandhi, Pinal Trivedi, Maharshi Pandya, Nidhi Patel, Nitin Savaliya, Raghawendra Kumar, R D Dixit, A M Kadri, Harsh Bakshi, Chaitanya Joshi, Madhvi Joshi
EPI_ISL_495023	Government Medical College, Bhavnagar	Gujarat Biotechnology Research Centre	Nitin Savaliya, Raghawendra Kumar, Dinesh Kumar, Zuber Saiyed, Komal Patel, Labdhi Pandya, Afzal Ansari, Nikha Trivedi, Kairavi Desai, Saklin Malek, Shirish Patel, Apurvasinh Puvar, Janvi Raval, Zarna Patel, Monika Gandhi, Pinal Trivedi, Maharshi Pandya, Nidhi Patel, R D Dixit, A M Kadri, Harsh Bakshi, Chaitanya Joshi, Madhvi Joshi
EPI_ISL_495024	Government Medical College, Bhavnagar	Gujarat Biotechnology Research Centre	Raghawendra Kumar, Dinesh Kumar, Zuber Saiyed, Komal Patel, Labdhi Pandya, Afzal Ansari, Nikha Trivedi, Kairavi Desai, Saklin Malek, Shirish Patel, Apurvasinh Puvar, Janvi Raval, Zarna Patel, Monika Gandhi, Pinal Trivedi, Maharshi Pandya, Nidhi Patel, Nitin Savaliya, R D Dixit, A M Kadri, Harsh Bakshi, Chaitanya Joshi, Madhvi Joshi
EPI_ISL_495025	Government Medical College, Bhavnagar	Gujarat Biotechnology Research Centre	Dinesh Kumar, Zuber Saiyed, Komal Patel, Labdhi Pandya, Afzal Ansari, Nikha Trivedi, Kairavi Desai, Saklin Malek, Shirish Patel, Apurvasinh Puvar, Janvi Raval, Zarna Patel, Monika Gandhi, Pinal Trivedi, Maharshi Pandya, Nidhi Patel, Nitin Savaliya, Raghawendra Kumar, R D Dixit, A M Kadri, Harsh Bakshi, Chaitanya Joshi, Madhvi Joshi
EPI_ISL_495026	GMERS Medical College & Hospital, Gotri, Vadodara	Gujarat Biotechnology Research Centre	Komal Patel, Labdhi Pandya, Afzal Ansari, Nikha Trivedi, Meenakshi Shah, Neena Doshi, Varsha Godbole, Apurvasinh Puvar, Janvi Raval, Zarna Patel, Monika Gandhi, Pinal Trivedi, Maharshi Pandya, Nidhi Patel, Nitin Savaliya, Raghawendra Kumar, Dinesh Kumar, Zuber Saiyed, R D Dixit, A M Kadri, Harsh Bakshi, Chaitanya Joshi, Madhvi Joshi
EPI_ISL_495027	GMERS Medical College & Hospital, Gotri, Vadodara	Gujarat Biotechnology Research Centre	Labdhi Pandya, Afzal Ansari, Nikha Trivedi, Meenakshi Shah, Neena Doshi, Varsha Godbole, Apurvasinh Puvar, Janvi Raval, Zarna Patel, Monika Gandhi, Pinal Trivedi, Maharshi Pandya, Nidhi Patel, Nitin Savaliya, Raghawendra Kumar, Dinesh Kumar, Zuber Saiyed, Komal Patel, R D Dixit, A M Kadri, Harsh Bakshi, Chaitanya Joshi, Madhvi Joshi
EPI_ISL_495028	GMERS Medical College & Hospital, Gotri, Vadodara	Gujarat Biotechnology Research Centre	Afzal Ansari, Nikha Trivedi, Meenakshi Shah, Neena Doshi, Varsha Godbole, Apurvasinh Puvar, Janvi Raval, Zarna Patel, Monika Gandhi, Pinal Trivedi, Maharshi Pandya, Nidhi Patel, Nitin Savaliya, Raghawendra Kumar, Dinesh Kumar, Zuber Saiyed, Komal Patel, Labdhi Pandya, R D Dixit, A M Kadri, Harsh Bakshi, Chaitanya Joshi, Madhvi Joshi
EPI_ISL_495029	GMERS Medical College & Hospital, Gotri, Vadodara	Gujarat Biotechnology Research Centre	Nikha Trivedi, Meenakshi Shah, Neena Doshi, Varsha Godbole, Apurvasinh Puvar, Janvi Raval, Zarna Patel, Monika Gandhi, Pinal Trivedi, Maharshi Pandya, Nidhi Patel, Nitin Savaliya, Raghawendra Kumar, Dinesh Kumar, Zuber Saiyed, Komal Patel, Labdhi Pandya, Afzal Ansari, R D Dixit, A M Kadri, Harsh Bakshi, Chaitanya Joshi, Madhvi Joshi
EPI_ISL_495030	GMERS Medical College & Hospital, Gotri, Vadodara	Gujarat Biotechnology Research Centre	Meenakshi Shah, Neena Doshi, Varsha Godbole, Apurvasinh Puvar, Janvi Raval, Zarna Patel, Monika Gandhi, Pinal Trivedi, Maharshi Pandya, Nidhi Patel, Nitin Savaliya, Raghawendra Kumar, Dinesh Kumar, Zuber Saiyed, Komal Patel, Labdhi Pandya, Afzal Ansari, Nikha Trivedi, R D Dixit, A M Kadri, Harsh Bakshi, Chaitanya Joshi, Madhvi Joshi
EPI_ISL_495031	GMERS Medical College & Hospital, Gotri, Vadodara	Gujarat Biotechnology Research Centre	Neena Doshi, Varsha Godbole, Apurvasinh Puvar, Janvi Raval, Zarna Patel, Monika Gandhi, Pinal Trivedi, Maharshi Pandya, Nidhi Patel, Nitin Savaliya, Raghawendra Kumar, Dinesh Kumar, Zuber Saiyed, Komal Patel, Labdhi Pandya, Afzal Ansari, Nikha Trivedi, Meenakshi Shah, R D Dixit, A M Kadri, Harsh Bakshi, Chaitanya Joshi, Madhvi Joshi
EPI_ISL_495032	GMERS Medical College & Hospital, Gotri, Vadodara	Gujarat Biotechnology Research Centre	Varsha Godbole, Apurvasinh Puvar, Janvi Raval, Zarna Patel, Monika Gandhi, Pinal Trivedi, Maharshi Pandya, Nidhi Patel, Nitin Savaliya, Raghawendra Kumar, Dinesh Kumar, Zuber Saiyed, Komal Patel, Labdhi Pandya, Afzal Ansari, Nikha Trivedi, Meenakshi Shah, Neena Doshi, R D Dixit, A M Kadri, Harsh Bakshi, Chaitanya Joshi, Madhvi Joshi
EPI_ISL_495033	GMERS Medical College & Hospital, Gotri, Vadodara	Gujarat Biotechnology Research Centre	Apurvasinh Puvar, Janvi Raval, Zarna Patel, Monika Gandhi, Pinal Trivedi, Maharshi Pandya, Nidhi Patel, Nitin Savaliya, Raghawendra Kumar, Dinesh Kumar, Zuber Saiyed, Komal Patel, Labdhi Pandya, Afzal Ansari, Nikha Trivedi, Meenakshi Shah, Neena Doshi, Varsha Godbole, R D Dixit, A M Kadri, Harsh Bakshi, Chaitanya Joshi, Madhvi Joshi
EPI_ISL_495034	GMERS Medical College & Hospital, Gotri, Vadodara	Gujarat Biotechnology Research Centre	Janvi Raval, Zarna Patel, Monika Gandhi, Pinal Trivedi, Maharshi Pandya, Nidhi Patel, Nitin Savaliya, Raghawendra Kumar, Dinesh Kumar, Zuber Saiyed, Komal Patel, Labdhi Pandya, Afzal Ansari, Nikha Trivedi, Meenakshi Shah, Neena Doshi, Varsha Godbole, Apurvasinh Puvar, R D Dixit, A M Kadri, Harsh Bakshi, Chaitanya Joshi, Madhvi Joshi
EPI_ISL_495035	GMERS Medical College & Hospital, Gotri, Vadodara	Gujarat Biotechnology Research Centre	Zarna Patel, Monika Gandhi, Pinal Trivedi, Maharshi Pandya, Nidhi Patel, Nitin Savaliya, Raghawendra Kumar, Dinesh Kumar, Zuber Saiyed, Komal Patel, Labdhi Pandya, Afzal Ansari, Nikha Trivedi, Meenakshi Shah, Neena Doshi, Varsha Godbole, Apurvasinh Puvar, Janvi Raval, R D Dixit, A M Kadri, Harsh Bakshi, Chaitanya Joshi, Madhvi Joshi
EPI_ISL_495038	GMERS Medical College & Hospital, Gotri, Vadodara	Gujarat Biotechnology Research Centre	Maharshi Pandya, Nidhi Patel, Nitin Savaliya, Raghawendra Kumar, Dinesh Kumar, Zuber Saiyed, Komal Patel, Labdhi Pandya, Afzal Ansari, Nikha Trivedi, Meenakshi Shah, Neena Doshi, Varsha Godbole, Apurvasinh Puvar, Janvi Raval, Zarna Patel, Monika Gandhi, Pinal Trivedi, R D Dixit, A M Kadri, Harsh Bakshi, Chaitanya Joshi, Madhvi Joshi
EPI_ISL_495039	GMERS Medical College & Hospital, Gotri, Vadodara	Gujarat Biotechnology Research Centre	Nidhi Patel, Nitin Savaliya, Raghawendra Kumar, Dinesh Kumar, Zuber Saiyed, Komal Patel, Labdhi Pandya, Afzal Ansari, Nikha Trivedi, Meenakshi Shah,

			Neena Doshi, Varsha Godbole, Apurvasinh Puvar, Janvi Raval, Zarna Patel, Monika Gandhi, Pinal Trivedi, Maharshi Pandya, R D Dixit, A M Kadri, Harsh Bakshi, Chaitanya Joshi, Madhvi Joshi
EPI_ISL_495042	GMERS Medical College & Hospital, Gotri, Vadodara	Gujarat Biotechnology Research Centre	Dinesh Kumar, Zuber Saiyed, Komal Patel, Labdhi Pandya, Afzal Ansari, Nikha Trivedi, Meenakshi Shah, Neena Doshi, Varsha Godbole, Apurvasinh Puvar, Janvi Raval, Zarna Patel, Monika Gandhi, Pinal Trivedi, Maharshi Pandya, Nidhi Patel, Nitin Savaliya, Raghawendra Kumar, R D Dixit, A M Kadri, Harsh Bakshi, Chaitanya Joshi, Madhvi Joshi
EPI_ISL_495043	GMERS Medical College & Hospital, Gotri, Vadodara	Gujarat Biotechnology Research Centre	Zuber Saiyed, Komal Patel, Labdhi Pandya, Afzal Ansari, Nikha Trivedi, Meenakshi Shah, Neena Doshi, Varsha Godbole, Apurvasinh Puvar, Janvi Raval, Zarna Patel, Monika Gandhi, Pinal Trivedi, Maharshi Pandya, Nidhi Patel, Nitin Savaliya, Raghawendra Kumar, Dinesh Kumar, R D Dixit, A M Kadri, Harsh Bakshi, Chaitanya Joshi, Madhvi Joshi
EPI_ISL_495044	GMERS Medical College & Hospital, Gotri, Vadodara	Gujarat Biotechnology Research Centre	Komal Patel, Labdhi Pandya, Afzal Ansari, Nikha Trivedi, Meenakshi Shah, Neena Doshi, Varsha Godbole, Apurvasinh Puvar, Janvi Raval, Zarna Patel, Monika Gandhi, Pinal Trivedi, Maharshi Pandya, Nidhi Patel, Nitin Savaliya, Raghawendra Kumar, Dinesh Kumar, Zuber Saiyed, R D Dixit, A M Kadri, Harsh Bakshi, Chaitanya Joshi, Madhvi Joshi
EPI_ISL_495045	GMERS Medical College & Hospital, Gotri, Vadodara	Gujarat Biotechnology Research Centre	Labdhi Pandya, Afzal Ansari, Nikha Trivedi, Meenakshi Shah, Neena Doshi, Varsha Godbole, Apurvasinh Puvar, Janvi Raval, Zarna Patel, Monika Gandhi, Pinal Trivedi, Maharshi Pandya, Nidhi Patel, Nitin Savaliya, Raghawendra Kumar, Dinesh Kumar, Zuber Saiyed, Komal Patel, R D Dixit, A M Kadri, Harsh Bakshi, Chaitanya Joshi, Madhvi Joshi
EPI_ISL_495046	GMERS Medical College & Hospital, Gotri, Vadodara	Gujarat Biotechnology Research Centre	Afzal Ansari, Nikha Trivedi, Meenakshi Shah, Neena Doshi, Varsha Godbole, Apurvasinh Puvar, Janvi Raval, Zarna Patel, Monika Gandhi, Pinal Trivedi, Maharshi Pandya, Nidhi Patel, Nitin Savaliya, Raghawendra Kumar, Dinesh Kumar, Zuber Saiyed, Komal Patel, Labdhi Pandya, R D Dixit, A M Kadri, Harsh Bakshi, Chaitanya Joshi, Madhvi Joshi
EPI_ISL_495047	GMERS Medical College & Hospital, Gotri, Vadodara	Gujarat Biotechnology Research Centre	Nikha Trivedi, Meenakshi Shah, Neena Doshi, Varsha Godbole, Apurvasinh Puvar, Janvi Raval, Zarna Patel, Monika Gandhi, Pinal Trivedi, Maharshi Pandya, Nidhi Patel, Nitin Savaliya, Raghawendra Kumar, Dinesh Kumar, Zuber Saiyed, Komal Patel, Labdhi Pandya, Afzal Ansari, R D Dixit, A M Kadri, Harsh Bakshi, Chaitanya Joshi, Madhvi Joshi
EPI_ISL_495048	GMERS Medical College & Hospital, Gotri, Vadodara	Gujarat Biotechnology Research Centre	Meenakshi Shah, Neena Doshi, Varsha Godbole, Apurvasinh Puvar, Janvi Raval, Zarna Patel, Monika Gandhi, Pinal Trivedi, Maharshi Pandya, Nidhi Patel, Nitin Savaliya, Raghawendra Kumar, Dinesh Kumar, Zuber Saiyed, Komal Patel, Labdhi Pandya, Afzal Ansari, Nikha Trivedi, R D Dixit, A M Kadri, Harsh Bakshi, Chaitanya Joshi, Madhvi Joshi
EPI_ISL_495049	GMERS Medical College & Hospital, Gotri, Vadodara	Gujarat Biotechnology Research Centre	Neena Doshi, Varsha Godbole, Apurvasinh Puvar, Janvi Raval, Zarna Patel, Monika Gandhi, Pinal Trivedi, Maharshi Pandya, Nidhi Patel, Nitin Savaliya, Raghawendra Kumar, Dinesh Kumar, Zuber Saiyed, Komal Patel, Labdhi Pandya, Afzal Ansari, Nikha Trivedi, Meenakshi Shah, R D Dixit, A M Kadri, Harsh Bakshi, Chaitanya Joshi, Madhvi Joshi
EPI_ISL_495050	GMERS Medical College & Hospital, Gotri, Vadodara	Gujarat Biotechnology Research Centre	Varsha Godbole, Apurvasinh Puvar, Janvi Raval, Zarna Patel, Monika Gandhi, Pinal Trivedi, Maharshi Pandya, Nidhi Patel, Nitin Savaliya, Raghawendra Kumar, Dinesh Kumar, Zuber Saiyed, Komal Patel, Labdhi Pandya, Afzal Ansari, Nikha Trivedi, Meenakshi Shah, Neena Doshi, R D Dixit, A M Kadri, Harsh Bakshi, Chaitanya Joshi, Madhvi Joshi
EPI_ISL_495051	GMERS Medical College & Hospital, Gotri, Vadodara	Gujarat Biotechnology Research Centre	Apurvasinh Puvar, Janvi Raval, Zarna Patel, Monika Gandhi, Pinal Trivedi, Maharshi Pandya, Nidhi Patel, Nitin Savaliya, Raghawendra Kumar, Dinesh Kumar, Zuber Saiyed, Komal Patel, Labdhi Pandya, Afzal Ansari, Nikha Trivedi, Meenakshi Shah, Neena Doshi, Varsha Godbole, R D Dixit, A M Kadri, Harsh Bakshi, Chaitanya Joshi, Madhvi Joshi
EPI_ISL_495052	GMERS Medical College & Hospital, Gotri, Vadodara	Gujarat Biotechnology Research Centre	Janvi Raval, Zarna Patel, Monika Gandhi, Pinal Trivedi, Maharshi Pandya, Nidhi Patel, Nitin Savaliya, Raghawendra Kumar, Dinesh Kumar, Zuber Saiyed, Komal Patel, Labdhi Pandya, Afzal Ansari, Nikha Trivedi, Meenakshi Shah, Neena Doshi, Varsha Godbole, Apurvasinh Puvar, R D Dixit, A M Kadri, Harsh Bakshi, Chaitanya Joshi, Madhvi Joshi
EPI_ISL_495053	GMERS Medical College & Hospital, Gotri, Vadodara	Gujarat Biotechnology Research Centre	Zarna Patel, Monika Gandhi, Pinal Trivedi, Maharshi Pandya, Nidhi Patel, Nitin Savaliya, Raghawendra Kumar, Dinesh Kumar, Zuber Saiyed, Komal Patel, Labdhi Pandya, Afzal Ansari, Nikha Trivedi, Meenakshi Shah, Neena Doshi, Varsha Godbole, Apurvasinh Puvar, Janvi Raval, R D Dixit, A M Kadri, Harsh Bakshi, Chaitanya Joshi, Madhvi Joshi
EPI_ISL_495054	GMERS Medical College & Hospital, Gotri, Vadodara	Gujarat Biotechnology Research Centre	Monika Gandhi, Pinal Trivedi, Maharshi Pandya, Nidhi Patel, Nitin Savaliya, Raghawendra Kumar, Dinesh Kumar, Zuber Saiyed, Komal Patel, Labdhi Pandya, Afzal Ansari, Nikha Trivedi, Meenakshi Shah, Neena Doshi, Varsha Godbole, Apurvasinh Puvar, Janvi Raval, Zarna Patel, R D Dixit, A M Kadri, Harsh Bakshi, Chaitanya Joshi, Madhvi Joshi
EPI_ISL_495055	GMERS Medical College & Hospital, Gotri, Vadodara	Gujarat Biotechnology Research Centre	Pinal Trivedi, Maharshi Pandya, Nidhi Patel, Nitin Savaliya, Raghawendra Kumar, Dinesh Kumar, Zuber Saiyed, Komal Patel, Labdhi Pandya, Afzal Ansari, Nikha Trivedi, Meenakshi Shah, Neena Doshi, Varsha Godbole, Apurvasinh Puvar, Janvi Raval, Zarna Patel, Monika Gandhi, R D Dixit, A M Kadri, Harsh Bakshi, Chaitanya Joshi, Madhvi Joshi
EPI_ISL_495056	GMERS Medical College & Hospital, Gotri, Vadodara	Gujarat Biotechnology Research Centre	Maharshi Pandya, Nidhi Patel, Nitin Savaliya, Raghawendra Kumar, Dinesh Kumar, Zuber Saiyed, Komal Patel, Labdhi Pandya, Afzal Ansari, Nikha Trivedi, Meenakshi Shah, Neena Doshi, Varsha Godbole, Apurvasinh Puvar, Janvi Raval, Zarna Patel, Monika Gandhi, Pinal Trivedi, R D Dixit, A M Kadri, Harsh Bakshi, Chaitanya Joshi, Madhvi Joshi
EPI_ISL_495057	GMERS Medical College & Hospital, Gotri, Vadodara	Gujarat Biotechnology Research Centre	Nidhi Patel, Nitin Savaliya, Raghawendra Kumar, Dinesh Kumar, Zuber Saiyed, Komal Patel, Labdhi Pandya, Afzal Ansari, Nikha Trivedi, Meenakshi Shah, Neena Doshi, Varsha Godbole, Apurvasinh Puvar, Janvi Raval, Zarna Patel, Monika Gandhi, Pinal Trivedi, Maharshi Pandya, R D Dixit, A M Kadri, Harsh Bakshi, Chaitanya Joshi, Madhvi Joshi
EPI_ISL_495058	GMERS Medical College & Hospital, Gotri, Vadodara	Gujarat Biotechnology Research Centre	Nitin Savaliya, Raghawendra Kumar, Dinesh Kumar, Zuber Saiyed, Komal Patel, Labdhi Pandya, Afzal Ansari, Nikha Trivedi, Meenakshi Shah, Neena Doshi, Varsha Godbole, Apurvasinh Puvar, Janvi Raval, Zarna Patel, Monika Gandhi, Pinal Trivedi, Maharshi Pandya, Nidhi Patel, R D Dixit, A M Kadri, Harsh Bakshi, Chaitanya Joshi, Madhvi Joshi
EPI_ISL_495059	GMERS Medical College & Hospital, Gotri, Vadodara	Gujarat Biotechnology Research Centre	Raghawendra Kumar, Dinesh Kumar, Zuber Saiyed, Komal Patel, Labdhi Pandya, Afzal Ansari, Nikha Trivedi, Meenakshi Shah, Neena Doshi, Varsha Godbole, Apurvasinh Puvar, Janvi Raval, Zarna Patel, Monika Gandhi, Pinal Trivedi, Maharshi Pandya, Nidhi Patel, Nitin Savaliya, R D Dixit, A M Kadri, Harsh Bakshi, Chaitanya Joshi, Madhvi Joshi
EPI_ISL_495060	GMERS Medical College & Hospital, Gotri, Vadodara	Gujarat Biotechnology Research Centre	Dinesh Kumar, Zuber Saiyed, Komal Patel, Labdhi Pandya, Afzal Ansari, Nikha Trivedi, Meenakshi Shah, Neena Doshi, Varsha Godbole, Apurvasinh Puvar, Janvi Raval, Zarna Patel, Monika Gandhi, Pinal Trivedi, Maharshi Pandya, Nidhi Patel, Nitin Savaliya, Raghawendra Kumar, R D Dixit, A M Kadri, Harsh Bakshi, Chaitanya Joshi, Madhvi Joshi
EPI_ISL_495061	GMERS Medical College & Hospital, Gotri, Vadodara	Gujarat Biotechnology Research Centre	Zuber Saiyed, Komal Patel, Labdhi Pandya, Afzal Ansari, Nikha Trivedi, Meenakshi Shah, Neena Doshi, Varsha Godbole, Apurvasinh Puvar, Janvi Raval, Zarna Patel, Monika Gandhi, Pinal Trivedi, Maharshi Pandya, Nidhi Patel, Nitin Savaliya, Raghawendra Kumar, Dinesh Kumar, R D Dixit, A M Kadri, Harsh Bakshi, Chaitanya Joshi, Madhvi Joshi
EPI_ISL_495062	GMERS Medical College & Hospital, Gotri, Vadodara	Gujarat Biotechnology Research Centre	Komal Patel, Labdhi Pandya, Afzal Ansari, Nikha Trivedi, Meenakshi Shah, Neena Doshi, Varsha Godbole, Apurvasinh Puvar, Janvi Raval, Zarna Patel, Monika Gandhi, Pinal Trivedi, Maharshi Pandya, Nidhi Patel, Nitin Savaliya, Raghawendra Kumar, Dinesh Kumar, Zuber Saiyed, R D Dixit, A M Kadri, Harsh Bakshi, Chaitanya Joshi, Madhvi Joshi
EPI_ISL_495063	GMERS Medical College & Hospital, Himmatnagar	Gujarat Biotechnology Research Centre	Labdhi Pandya, Afzal Ansari, Nikha Trivedi, Himanshu Khatri, Mayur Gandhi, Apurvasinh Puvar, Janvi Raval, Zarna Patel, Monika Gandhi, Pinal Trivedi, Maharshi Pandya, Nidhi Patel, Nitin Savaliya, Raghawendra Kumar, Dinesh Kumar, Zuber Saiyed, Komal Patel, R D Dixit, A M Kadri, Harsh Bakshi, Chaitanya Joshi, Madhvi Joshi,
EPI_ISL_495064	GMERS Medical College & Hospital, Himmatnagar	Gujarat Biotechnology Research Centre	Afzal Ansari, Nikha Trivedi, Himanshu Khatri, Mayur Gandhi, Apurvasinh Puvar, Janvi Raval, Zarna Patel, Monika Gandhi, Pinal Trivedi, Maharshi Pandya, Nidhi Patel, Nitin Savaliya, Raghawendra Kumar, Dinesh Kumar, Zuber Saiyed, Komal Patel, Labdhi Pandya, R D Dixit, A M Kadri, Harsh Bakshi, Chaitanya Joshi, Madhvi Joshi,
EPI_ISL_495065	GAIMS & G K General Hospital	Gujarat Biotechnology Research Centre	Nikha Trivedi, Babulal Babhoria, Hitesh Assudani, Apurvasinh Puvar, Janvi Raval, Zarna Patel, Monika Gandhi, Pinal Trivedi, Maharshi Pandya, Nidhi Patel, Nitin Savaliya, Raghawendra Kumar, Dinesh Kumar, Zuber Saiyed, Komal Patel, Labdhi Pandya, Afzal Ansari, R D Dixit, A M Kadri, Harsh Bakshi, Chaitanya Joshi, Madhvi Joshi,
EPI_ISL_495066	GAIMS & G K General Hospital	Gujarat Biotechnology Research Centre	Babulal Babhoria, Hitesh Assudani, Apurvasinh Puvar, Janvi Raval, Zarna Patel, Monika Gandhi, Pinal Trivedi, Maharshi Pandya, Nidhi Patel, Nitin Savaliya,

			Raghawendra Kumar, Dinesh Kumar, Zuber Saiyed, Komal Patel, Labdhi Pandya, Afzal Ansari, Nikha Trivedi, R D Dixit, A M Kadri, Harsh Bakshi, Chaitanya Joshi, Madhvi Joshi,
EPI_ISL_495067	Dr. N. D. Desai Medical College & Hospital	Gujarat Biotechnology Research Centre	J G Buch, Jigar Gusani, Supreet Prabhu, Apurvasinh Puvar, Janvi Raval, Zarna Patel, Monika Gandhi, Pinal Trivedi, Maharshi Pandya, Nidhi Patel, Nitin Savaliya, Raghawendra Kumar, Dinesh Kumar, Zuber Saiyed, Komal Patel, Labdhi Pandya, Afzal Ansari, Nikha Trivedi, R D Dixit, A M Kadri, Harsh Bakshi, Chaitanya Joshi, Madhvi Joshi
EPI_ISL_495068	Dr. N. D. Desai Medical College & Hospital	Gujarat Biotechnology Research Centre	Jigar Gusani, Supreet Prabhu, Apurvasinh Puvar, Janvi Raval, Zarna Patel, Monika Gandhi, Pinal Trivedi, Maharshi Pandya, Nidhi Patel, Nitin Savaliya, Raghawendra Kumar, Dinesh Kumar, Zuber Saiyed, Komal Patel, Labdhi Pandya, Afzal Ansari, Nikha Trivedi, J G Buch, R D Dixit, A M Kadri, Harsh Bakshi, Chaitanya Joshi, Madhvi Joshi
EPI_ISL_495069	Dr. N. D. Desai Medical College & Hospital	Gujarat Biotechnology Research Centre	Supreet Prabhu, Apurvasinh Puvar, Janvi Raval, Zarna Patel, Monika Gandhi, Pinal Trivedi, Maharshi Pandya, Nidhi Patel, Nitin Savaliya, Raghawendra Kumar, Dinesh Kumar, Zuber Saiyed, Komal Patel, Labdhi Pandya, Afzal Ansari, Nikha Trivedi, J G Buch, Jigar Gusani, R D Dixit, A M Kadri, Harsh Bakshi, Chaitanya Joshi, Madhvi Joshi
EPI_ISL_495070	Department of MicroBiology, Government Medical College, Surat	Gujarat Biotechnology Research Centre	Apurvasinh Puvar, Janvi Raval, Zarna Patel, Monika Gandhi, Pinal Trivedi, Maharshi Pandya, Nidhi Patel, Nitin Savaliya, Raghawendra Kumar, Dinesh Kumar, Zuber Saiyed, Komal Patel, Labdhi Pandya, Afzal Ansari, Nikha Trivedi, Naresh Chauhan, Summaiya Mullan, Amit gamit, R D Dixit, A M Kadri, Harsh Bakshi, Chaitanya Joshi, Madhvi Joshi
EPI_ISL_495071	Department of MicroBiology, Government Medical College, Surat	Gujarat Biotechnology Research Centre	Janvi Raval, Zarna Patel, Monika Gandhi, Pinal Trivedi, Maharshi Pandya, Nidhi Patel, Nitin Savaliya, Raghawendra Kumar, Dinesh Kumar, Zuber Saiyed, Komal Patel, Labdhi Pandya, Afzal Ansari, Nikha Trivedi, Naresh Chauhan, Summaiya Mullan, Amit gamit, Apurvasinh Puvar, R D Dixit, A M Kadri, Harsh Bakshi, Chaitanya Joshi, Madhvi Joshi
EPI_ISL_495072	Department of MicroBiology, Government Medical College, Surat	Gujarat Biotechnology Research Centre	Zarna Patel, Monika Gandhi, Pinal Trivedi, Maharshi Pandya, Nidhi Patel, Nitin Savaliya, Raghawendra Kumar, Dinesh Kumar, Zuber Saiyed, Komal Patel, Labdhi Pandya, Afzal Ansari, Nikha Trivedi, Naresh Chauhan, Summaiya Mullan, Amit gamit, Apurvasinh Puvar, Janvi Raval, R D Dixit, A M Kadri, Harsh Bakshi, Chaitanya Joshi, Madhvi Joshi
EPI_ISL_495073	Department of MicroBiology, Government Medical College, Surat	Gujarat Biotechnology Research Centre	Monika Gandhi, Pinal Trivedi, Maharshi Pandya, Nidhi Patel, Nitin Savaliya, Raghawendra Kumar, Dinesh Kumar, Zuber Saiyed, Komal Patel, Labdhi Pandya, Afzal Ansari, Nikha Trivedi, Naresh Chauhan, Summaiya Mullan, Amit gamit, Apurvasinh Puvar, Janvi Raval, Zarna Patel, R D Dixit, A M Kadri, Harsh Bakshi, Chaitanya Joshi, Madhvi Joshi
EPI_ISL_495074	Department of MicroBiology, Government Medical College, Surat	Gujarat Biotechnology Research Centre	Pinal Trivedi, Maharshi Pandya, Nidhi Patel, Nitin Savaliya, Raghawendra Kumar, Dinesh Kumar, Zuber Saiyed, Komal Patel, Labdhi Pandya, Afzal Ansari, Nikha Trivedi, Naresh Chauhan, Summaiya Mullan, Amit gamit, Apurvasinh Puvar, Janvi Raval, Zarna Patel, Monika Gandhi, R D Dixit, A M Kadri, Harsh Bakshi, Chaitanya Joshi, Madhvi Joshi
EPI_ISL_495075	Department of MicroBiology, Government Medical College, Surat	Gujarat Biotechnology Research Centre	Nidhi Patel, Nitin Savaliya, Raghawendra Kumar, Dinesh Kumar, Zuber Saiyed, Komal Patel, Labdhi Pandya, Afzal Ansari, Nikha Trivedi, Naresh Chauhan, Summaiya Mullan, Amit gamit, Apurvasinh Puvar, Janvi Raval, Zarna Patel, Monika Gandhi, Pinal Trivedi, Maharshi Pandya, R D Dixit, A M Kadri, Harsh Bakshi, Chaitanya Joshi, Madhvi Joshi
EPI_ISL_495076	Department of MicroBiology, Government Medical College, Surat	Gujarat Biotechnology Research Centre	Nitin Savaliya, Raghawendra Kumar, Dinesh Kumar, Zuber Saiyed, Komal Patel, Labdhi Pandya, Afzal Ansari, Nikha Trivedi, Naresh Chauhan, Summaiya Mullan, Amit gamit, Apurvasinh Puvar, Janvi Raval, Zarna Patel, Monika Gandhi, Pinal Trivedi, Maharshi Pandya, Nidhi Patel, R D Dixit, A M Kadri, Harsh Bakshi, Chaitanya Joshi, Madhvi Joshi
EPI_ISL_495077	Department of MicroBiology, Government Medical College, Surat	Gujarat Biotechnology Research Centre	Raghawendra Kumar, Dinesh Kumar, Zuber Saiyed, Komal Patel, Labdhi Pandya, Afzal Ansari, Nikha Trivedi, Naresh Chauhan, Summaiya Mullan, Amit gamit, Apurvasinh Puvar, Janvi Raval, Zarna Patel, Monika Gandhi, Pinal Trivedi, Maharshi Pandya, Nidhi Patel, Nitin Savaliya, R D Dixit, A M Kadri, Harsh Bakshi, Chaitanya Joshi, Madhvi Joshi
EPI_ISL_495078	Department of MicroBiology, Government Medical College, Surat	Gujarat Biotechnology Research Centre	Dinesh Kumar, Zuber Saiyed, Komal Patel, Labdhi Pandya, Afzal Ansari, Nikha Trivedi, Naresh Chauhan, Summaiya Mullan, Amit gamit, Apurvasinh Puvar, Janvi Raval, Zarna Patel, Monika Gandhi, Pinal Trivedi, Maharshi Pandya, Nidhi Patel, Nitin Savaliya, Raghawendra Kumar, R D Dixit, A M Kadri, Harsh Bakshi, Chaitanya Joshi, Madhvi Joshi
EPI_ISL_495079	Department of MicroBiology, Government Medical College, Surat	Gujarat Biotechnology Research Centre	Afzal Ansari, Nikha Trivedi, Naresh Chauhan, Summaiya Mullan, Amit gamit, Apurvasinh Puvar, Janvi Raval, Zarna Patel, Monika Gandhi, Pinal Trivedi, Maharshi Pandya, Nidhi Patel, Nitin Savaliya, Raghawendra Kumar, Dinesh Kumar, Zuber Saiyed, Komal Patel, Labdhi Pandya, R D Dixit, A M Kadri, Harsh Bakshi, Chaitanya Joshi, Madhvi Joshi
EPI_ISL_495080	Department of MicroBiology, Government Medical College, Surat	Gujarat Biotechnology Research Centre	Nikha Trivedi, Naresh Chauhan, Summaiya Mullan, Amit gamit, Apurvasinh Puvar, Janvi Raval, Zarna Patel, Monika Gandhi, Pinal Trivedi, Maharshi Pandya, Nidhi Patel, Nitin Savaliya, Raghawendra Kumar, Dinesh Kumar, Zuber Saiyed, Komal Patel, Labdhi Pandya, Afzal Ansari, R D Dixit, A M Kadri, Harsh Bakshi, Chaitanya Joshi, Madhvi Joshi
EPI_ISL_495411, EPI_ISL_495412, EPI_ISL_495416, EPI_ISL_495418, EPI_ISL_495420, EPI_ISL_495421, EPI_ISL_495422, EPI_ISL_495425, EPI_ISL_495429, EPI_ISL_495431, EPI_ISL_495433, EPI_ISL_495436, EPI_ISL_495438, EPI_ISL_495444, EPI_ISL_495445, EPI_ISL_495447, EPI_ISL_495449, EPI_ISL_495450, EPI_ISL_495451, EPI_ISL_495452, EPI_ISL_495453, EPI_ISL_495454, EPI_ISL_495456			
see above	Kafkas University, Faculty of Medicine, Department of Medical Microbiology	Kafkas University, Faculty of Medicine, Department of Medical Microbiology	Murat Karamese, Didem Ozgur, E. Ediz Tutuncu
EPI_ISL_495520, EPI_ISL_495527, EPI_ISL_495528, EPI_ISL_495529, EPI_ISL_495530, EPI_ISL_495534	NHLS-IALCH	KRISP, KZN Research Innovation and Sequencing Platform	Giandhari J, Pillay S, Lessells R, Chimukangara B, Mdlalose K, York D, Khan S, Tegally H, Wilkinson E, de Oliveira T
EPI_ISL_495535, EPI_ISL_495536, EPI_ISL_495537, EPI_ISL_495538, EPI_ISL_495539, EPI_ISL_495540, EPI_ISL_495541, EPI_ISL_495542	Medical Disagnostics Services (MDS)	KRISP, KZN Research Innovation and Sequencing Platform	Giandhari J, Pillay S, Lessells R, Chimukangara B, Mdlalose K, York D, Khan S, Tegally H, Wilkinson E, de Oliveira T
EPI_ISL_495543, EPI_ISL_495544, EPI_ISL_495546, EPI_ISL_495549, EPI_ISL_495550, EPI_ISL_495557, EPI_ISL_495558, EPI_ISL_495559, EPI_ISL_495561, EPI_ISL_495562	NHLS-IALCH	KRISP, KZN Research Innovation and Sequencing Platform	Giandhari J, Pillay S, Lessells R, Chimukangara B, Mdlalose K, York D, Khan S, Tegally H, Wilkinson E, de Oliveira T
EPI_ISL_496520, EPI_ISL_496529, EPI_ISL_496533, EPI_ISL_496537, EPI_ISL_496540, EPI_ISL_496541, EPI_ISL_496542, EPI_ISL_496543, EPI_ISL_496544, EPI_ISL_496545	Armed Forces Medical College	National Centre For Cell Science	Dhiraj Paul, Kunal Jani, Radha Chauhan, Janesh Kumar, Vasudevan Seshadri, Girdhari Lal, Rajesh Karyakarte, Suvarna Joshi, Murlidhar Tambe, Sourav Sen, Santosh Karade, Kavita Bala Anand, Shelinder Pal Singh Shergill, Rajiv Mohan Gupta, Manoj Kumar Bhat, Arvind Sahu, Maharashtra COVID-19 Study Group, DBT's PAN-INDIA 1000 SARS-CoV2 RNA genome sequencing consortium, Yogesh S Shouche
EPI_ISL_496559, EPI_ISL_496560, EPI_ISL_496571, EPI_ISL_496581	National Centre For Cell Science	National Centre For Cell Science	Dhiraj Paul, Kunal Jani, Radha Chauhan, Janesh Kumar, Vasudevan Seshadri, Girdhari Lal, Rajesh Karyakarte, Suvarna Joshi, Murlidhar Tambe, Sourav Sen, Santosh Karade, Kavita Bala Anand, Shelinder Pal Singh Shergill, Rajiv Mohan Gupta, Manoj Kumar Bhat, Arvind Sahu, Maharashtra COVID-19 Study Group, DBT's PAN-INDIA 1000 SARS-CoV2 RNA genome sequencing consortium, Yogesh S Shouche
EPI_ISL_497950	Shaoxing CDC	Zhejiang Provincial Center for Disease Control and Prevention	Yin Chen, Yanjun Zhang, Haiyan Mao, Junhang Pan, Xiuyu Lou, Yi Sun, Hao Yan, Zhen Li, Wen Shi
EPI_ISL_498055, EPI_ISL_498060, EPI_ISL_498061, EPI_ISL_498062, EPI_ISL_498064, EPI_ISL_498065, EPI_ISL_498066, EPI_ISL_498067, EPI_ISL_498069, EPI_ISL_498070, EPI_ISL_498071, EPI_ISL_498072, EPI_ISL_498073, EPI_ISL_498074, EPI_ISL_498075, EPI_ISL_498077, EPI_ISL_498078, EPI_ISL_498079, EPI_ISL_498081, EPI_ISL_498082, EPI_ISL_498084, EPI_ISL_498085, EPI_ISL_498086, EPI_ISL_498087, EPI_ISL_498088, EPI_ISL_498089, EPI_ISL_498090, EPI_ISL_498091, EPI_ISL_498092, EPI_ISL_498093, EPI_ISL_498094, EPI_ISL_498095, EPI_ISL_498096, EPI_ISL_498097, EPI_ISL_498098, EPI_ISL_498099, EPI_ISL_498100, EPI_ISL_498101, EPI_ISL_498102, EPI_ISL_498103, EPI_ISL_498104, EPI_ISL_498105, EPI_ISL_498106, EPI_ISL_498107, EPI_ISL_498108, EPI_ISL_498109, EPI_ISL_498110, EPI_ISL_498111, EPI_ISL_498112, EPI_ISL_498113, EPI_ISL_498114, EPI_ISL_498115, EPI_ISL_498116, EPI_ISL_498117, EPI_ISL_498118, EPI_ISL_498119, EPI_ISL_498120, EPI_ISL_498121, EPI_ISL_498122, EPI_ISL_498123, EPI_ISL_498124, EPI_ISL_498125, EPI_ISL_498126			
see above	NHLS-IALCH	KRISP, KZN Research Innovation and Sequencing Platform	Giandhari J, Pillay S, Lessells R, Chimukangara B, Mdlalose K, York D, Khan S, Tegally H, Wilkinson E, de Oliveira T

EPI_ISL_498238, EPI_ISL_498242, EPI_ISL_498243, EPI_ISL_498249, EPI_ISL_498250	Institut Pasteur de Dakar	Institut Pasteur de Dakar	Ndongo Dia, Moussa Moise Diagne, Mamadou Diop, Marie Henriette Dior Ndione, Mamadou Malado Jallow, Safietou Sankhe Mbengue, Ousmane Faye, Amadou Alpha Sall.
EPI_ISL_498558, EPI_ISL_498559, EPI_ISL_498560, EPI_ISL_498561, EPI_ISL_498562, EPI_ISL_498563	Laboratory of Molecular Virology International Center for Genetic Engineering and Biotechnology (ICGEB)	ARGO Open Lab Platform for Genome Sequencing	Licastro D, Rajasekharan S, Dal Monego S, Segat L, D'Agaro P, Marcello A
EPI_ISL_500539, EPI_ISL_500540, EPI_ISL_500542, EPI_ISL_500543, EPI_ISL_500544, EPI_ISL_500545, EPI_ISL_500546, EPI_ISL_500547, EPI_ISL_500548, EPI_ISL_500550, EPI_ISL_500551, EPI_ISL_500552, EPI_ISL_500553, EPI_ISL_500554, EPI_ISL_500555, EPI_ISL_500556, EPI_ISL_500557, EPI_ISL_500558, EPI_ISL_500559, EPI_ISL_500560, EPI_ISL_500561, EPI_ISL_500562, EPI_ISL_500563, EPI_ISL_500564, EPI_ISL_500565, EPI_ISL_500566, EPI_ISL_500567, EPI_ISL_500568, EPI_ISL_500569, EPI_ISL_500570, EPI_ISL_500571, EPI_ISL_500572			
see above	Singapore General Hospital	Department of Microbiology	Nurdyana Abdul Rahman, Kun Lee Lim, Chenhao Li, Kian Sing Chan, Lynette Oon, Kern Rei Chng, Niranjan Nagarajan, Karrie Ko
EPI_ISL_500946	GMERS Medical College & Hospital, Gotri, Vadodara	Gujarat Biotechnology Research Centre	Zuber Saiyed, Komal Patel, Labdhi Pandya, Afzal Ansari, Nikha Trivedi, Meenakshi Shah, Neena Doshi, Varsha Godbole, Apurvasinh Puvar, Janvi Raval, Zarna Patel, Monika Gandhi, Pinal Trivedi, Maharshi Pandya, Nidhi Patel, Nitin Savaliya, Raghawendra Kumar, Dinesh Kumar, R D Dixit, A M Kadri, Harsh Bakshi, Chaitanya Joshi, Madhvi Joshi
EPI_ISL_500947	Department of MicroBiology, Government Medical College, Surat	Gujarat Biotechnology Research Centre	Maharshi Pandya, Nidhi Patel, Nitin Savaliya, Raghawendra Kumar, Dinesh Kumar, Zuber Saiyed, Komal Patel, Labdhi Pandya, Afzal Ansari, Nikha Trivedi, Naresh Chauhan, Summaiya Mullan, Amit gamit, Apurvasinh Puvar, Janvi Raval, Zarna Patel, Monika Gandhi, Pinal Trivedi, R D Dixit, A M Kadri, Harsh Bakshi, Chaitanya Joshi, Madhvi Joshi
EPI_ISL_500948	Department of MicroBiology, Government Medical College, Surat	Gujarat Biotechnology Research Centre	Zuber Saiyed, Komal Patel, Labdhi Pandya, Afzal Ansari, Nikha Trivedi, Naresh Chauhan, Summaiya Mullan, Amit gamit, Apurvasinh Puvar, Janvi Raval, Zarna Patel, Monika Gandhi, Pinal Trivedi, Maharshi Pandya, Nidhi Patel, Nitin Savaliya, Raghawendra Kumar, Dinesh Kumar, R D Dixit, A M Kadri, Harsh Bakshi, Chaitanya Joshi, Madhvi Joshi
EPI_ISL_500949	Department of MicroBiology, Government Medical College, Surat	Gujarat Biotechnology Research Centre	Komal Patel, Labdhi Pandya, Afzal Ansari, Nikha Trivedi, Naresh Chauhan, Summaiya Mullan, Amit gamit, Apurvasinh Puvar, Janvi Raval, Zarna Patel, Monika Gandhi, Pinal Trivedi, Maharshi Pandya, Nidhi Patel, Nitin Savaliya, Raghawendra Kumar, Dinesh Kumar, Zuber Saiyed, R D Dixit, A M Kadri, Harsh Bakshi, Chaitanya Joshi, Madhvi Joshi
EPI_ISL_500950	Department of MicroBiology, Government Medical College, Surat	Gujarat Biotechnology Research Centre	Labdhi Pandya, Afzal Ansari, Nikha Trivedi, Naresh Chauhan, Summaiya Mullan, Amit gamit, Apurvasinh Puvar, Janvi Raval, Zarna Patel, Monika Gandhi, Pinal Trivedi, Maharshi Pandya, Nidhi Patel, Nitin Savaliya, Raghawendra Kumar, Dinesh Kumar, Zuber Saiyed, Komal Patel, R D Dixit, A M Kadri, Harsh Bakshi, Chaitanya Joshi, Madhvi Joshi
EPI_ISL_507274, EPI_ISL_507279	WHO National Influenza Centre Russian Federation	WHO National Influenza Centre Russian Federation	Andrey Komissarov, Artem Fadeev, Mariia Sergeeva, Anna Ivanova, Daria Danilenko
EPI_ISL_509223, EPI_ISL_509224, EPI_ISL_509225, EPI_ISL_509226, EPI_ISL_509228, EPI_ISL_509229, EPI_ISL_509230, EPI_ISL_509231, EPI_ISL_509232, EPI_ISL_509233, EPI_ISL_509234, EPI_ISL_509235, EPI_ISL_509236, EPI_ISL_509237, EPI_ISL_509238, EPI_ISL_509239, EPI_ISL_509240, EPI_ISL_509241, EPI_ISL_509242, EPI_ISL_509243, EPI_ISL_509244, EPI_ISL_509245, EPI_ISL_509246, EPI_ISL_509247, EPI_ISL_509248, EPI_ISL_509249, EPI_ISL_509250, EPI_ISL_509251, EPI_ISL_509252, EPI_ISL_509253, EPI_ISL_509254, EPI_ISL_509255, EPI_ISL_509256, EPI_ISL_509257, EPI_ISL_509258, EPI_ISL_509260, EPI_ISL_509261, EPI_ISL_509262, EPI_ISL_509263, EPI_ISL_509264, EPI_ISL_509265, EPI_ISL_509266, EPI_ISL_509267, EPI_ISL_509269, EPI_ISL_509270, EPI_ISL_509271, EPI_ISL_509274, EPI_ISL_509275, EPI_ISL_509276, EPI_ISL_509279, EPI_ISL_509281, EPI_ISL_509283, EPI_ISL_509284, EPI_ISL_509285, EPI_ISL_509286, EPI_ISL_509287, EPI_ISL_509288, EPI_ISL_509289, EPI_ISL_509290, EPI_ISL_509292, EPI_ISL_509293, EPI_ISL_509294, EPI_ISL_509295, EPI_ISL_509296, EPI_ISL_509297, EPI_ISL_509299, EPI_ISL_509300, EPI_ISL_509301, EPI_ISL_509302, EPI_ISL_509303, EPI_ISL_509304, EPI_ISL_509305, EPI_ISL_509306, EPI_ISL_509307, EPI_ISL_509308, EPI_ISL_509309, EPI_ISL_509310, EPI_ISL_509311, EPI_ISL_509312, EPI_ISL_509313, EPI_ISL_509314, EPI_ISL_509315, EPI_ISL_509316, EPI_ISL_509317, EPI_ISL_509318, EPI_ISL_509319, EPI_ISL_509320, EPI_ISL_509321, EPI_ISL_509322, EPI_ISL_509323, EPI_ISL_509324, EPI_ISL_509325, EPI_ISL_509326, EPI_ISL_509327, EPI_ISL_509328, EPI_ISL_509329, EPI_ISL_509330, EPI_ISL_509331, EPI_ISL_509332, EPI_ISL_509333, EPI_ISL_509334, EPI_ISL_509335, EPI_ISL_509336, EPI_ISL_509337, EPI_ISL_509338, EPI_ISL_509339, EPI_ISL_509340, EPI_ISL_509341, EPI_ISL_509342, EPI_ISL_509343, EPI_ISL_509344, EPI_ISL_509345, EPI_ISL_509346, EPI_ISL_509347, EPI_ISL_509348, EPI_ISL_509349, EPI_ISL_509350, EPI_ISL_509351, EPI_ISL_509352, EPI_ISL_509353, EPI_ISL_509354, EPI_ISL_509355, EPI_ISL_509356, EPI_ISL_509357, EPI_ISL_509358, EPI_ISL_509360, EPI_ISL_509361, EPI_ISL_509362, EPI_ISL_509363, EPI_ISL_509364, EPI_ISL_509365, EPI_ISL_509366, EPI_ISL_509367, EPI_ISL_509368, EPI_ISL_509369			
see above	NHLS-IALCH	KRISP, KZN Research Innovation and Sequencing Platform	Giandhari J, Pillay S, Lessells R, Mdlalose K, York D, Tegally H, Wilkinson E, de Oliveira T
EPI_ISL_509373, EPI_ISL_509374, EPI_ISL_509375, EPI_ISL_509377, EPI_ISL_509378, EPI_ISL_509379, EPI_ISL_509380, EPI_ISL_509381, EPI_ISL_509382, EPI_ISL_509383, EPI_ISL_509384, EPI_ISL_509385, EPI_ISL_509386, EPI_ISL_509387, EPI_ISL_509388, EPI_ISL_509389, EPI_ISL_509390, EPI_ISL_509391, EPI_ISL_509392, EPI_ISL_509393, EPI_ISL_509394			
see above	Singapore General Hospital	Department of Microbiology	Nurdyana Abdul Rahman, Kun Lee Lim, Chenhao Li, Kian Sing Chan, Lynette Oon, Kern Rei Chng, Niranjan Nagarajan, Karrie Ko
EPI_ISL_510081, EPI_ISL_510082	Communicable Disease Branch	Hong Kong Department of Health	Mak Gannon C.K., Lam Edman T.K., Chan Rickjason C.W., Tsang Dominic N.C.
EPI_ISL_510083	Princess Margaret Hospital	Hong Kong Department of Health	Mak Gannon C.K., Lam Edman T.K., Chan Rickjason C.W., Tsang Dominic N.C.
EPI_ISL_510084	Prince of Wales Hospital	Hong Kong Department of Health	Mak Gannon C.K., Lam Edman T.K., Chan Rickjason C.W., Tsang Dominic N.C.
EPI_ISL_510085	Yan Chai Hospital	Hong Kong Department of Health	Mak Gannon C.K., Lam Edman T.K., Chan Rickjason C.W., Tsang Dominic N.C.
EPI_ISL_510086	Kwong Wah Hospital	Hong Kong Department of Health	Mak Gannon C.K., Lam Edman T.K., Chan Rickjason C.W., Tsang Dominic N.C.
EPI_ISL_510087	Private medical practitioner	Hong Kong Department of Health	Mak Gannon C.K., Lam Edman T.K., Chan Rickjason C.W., Tsang Dominic N.C.
EPI_ISL_510088	United Christian Hospital	Hong Kong Department of Health	Mak Gannon C.K., Lam Edman T.K., Chan Rickjason C.W., Tsang Dominic N.C.
EPI_ISL_510089	Queen Elizabeth Hospital	Hong Kong Department of Health	Mak Gannon C.K., Lam Edman T.K., Chan Rickjason C.W., Tsang Dominic N.C.
EPI_ISL_510090	Hong Kong Baptist Hospital	Hong Kong Department of Health	Mak Gannon C.K., Lam Edman T.K., Chan Rickjason C.W., Tsang Dominic N.C.
EPI_ISL_510091	Kwong Wah Hospital	Hong Kong Department of Health	Mak Gannon C.K., Lam Edman T.K., Chan Rickjason C.W., Tsang Dominic N.C.
EPI_ISL_510092, EPI_ISL_510093, EPI_ISL_510094, EPI_ISL_510095, EPI_ISL_510096	Queen Elizabeth Hospital	Hong Kong Department of Health	Mak Gannon C.K., Lam Edman T.K., Chan Rickjason C.W., Tsang Dominic N.C.
EPI_ISL_510097, EPI_ISL_510098	United Christian Hospital	Hong Kong Department of Health	Mak Gannon C.K., Lam Edman T.K., Chan Rickjason C.W., Tsang Dominic N.C.
EPI_ISL_510099	Queen Mary Hospital	Hong Kong Department of Health	Mak Gannon C.K., Lam Edman T.K., Chan Rickjason C.W., Tsang Dominic N.C.
EPI_ISL_510100	Tuen Mun Hospital	Hong Kong Department of Health	Mak Gannon C.K., Lam Edman T.K., Chan Rickjason C.W., Tsang Dominic N.C.
EPI_ISL_510101	Pamela Youde Nethersole Eastern Hospital	Hong Kong Department of Health	Mak Gannon C.K., Lam Edman T.K., Chan Rickjason C.W., Tsang Dominic N.C.
EPI_ISL_511879	Laboratorium Kesehatan Provinsi Jawa Barat	Molecular Genetics Laboratory-Faculty of Medicine-Universitas Padjadjaran; School of Life Sciences and Technology & School of Pharmacy-Institut Teknologi Bandung; Laboratorium Kesehatan Provinsi Jawa Barat	Marselina Irasonia Tan, Yunia Sribudiani, Catur Riani, Azzania Fibriani, Husna Nugrahapraja, Tarwadi, Ema Rahmawati, Hesti Lina Wiraswati, Lia Faridah, Savira Ekawardhani, Ryan Bayusantika Ristandi, Rifky Waluyajati Rachman, Cut Nur Cinthia Alamanda, Hammam Riza, Soni Solistia Wirawan, Agung Eru Wibowo
EPI_ISL_512058	B.J. Medical College and Civil hospital, Ahmedabad	Gujarat Biotechnology Research Centre	Monika Gandhi, Pinal Trivedi, Maharshi Pandya, Nidhi Patel, Nitin Savaliya, Raghawendra Kumar, Dinesh Kumar, Zuber Saiyed, Komal Patel, Labdhi Pandya, Afzal Ansari, Nikha Trivedi, Pranay Shah, Kamlesh J Upadhyay, Sanjay Kapadia, Apurvasinh Puvar, Janvi Raval, Zarna Patel, R D Dixit, A M Kadri, Harsh Bakshi, Chaitanya Joshi, Madhvi Joshi
EPI_ISL_512059	B.J. Medical College and Civil hospital, Ahmedabad	Gujarat Biotechnology Research Centre	Pinal Trivedi, Maharshi Pandya, Nidhi Patel, Nitin Savaliya, Raghawendra Kumar, Dinesh Kumar, Zuber Saiyed, Komal Patel, Labdhi Pandya, Afzal Ansari, Nikha Trivedi, Pranay Shah, Kamlesh J Upadhyay, Sanjay Kapadia, Apurvasinh Puvar, Janvi Raval, Zarna Patel, Monika Gandhi, R D Dixit, A M Kadri, Harsh Bakshi, Chaitanya Joshi, Madhvi Joshi
EPI_ISL_512060	B.J. Medical College and Civil hospital, Ahmedabad	Gujarat Biotechnology Research Centre	Maharshi Pandya, Nidhi Patel, Nitin Savaliya, Raghawendra Kumar, Dinesh Kumar, Zuber Saiyed, Komal Patel, Labdhi Pandya, Afzal Ansari, Nikha Trivedi, Pranay Shah, Kamlesh J Upadhyay, Sanjay Kapadia, Apurvasinh Puvar, Janvi Raval, Zarna Patel, Monika Gandhi, Pinal Trivedi, R D Dixit, A M Kadri, Harsh Bakshi, Chaitanya Joshi, Madhvi Joshi
EPI_ISL_512063	B.J. Medical College and Civil hospital, Ahmedabad	Gujarat Biotechnology Research Centre	Raghawendra Kumar, Dinesh Kumar, Zuber Saiyed, Komal Patel, Labdhi Pandya, Afzal Ansari, Nikha Trivedi, Pranay Shah, Kamlesh J Upadhyay, Sanjay Kapadia, Apurvasinh Puvar, Janvi Raval, Zarna Patel, Monika Gandhi, Pinal Trivedi, Maharshi Pandya, Nidhi Patel, Nitin Savaliya, R D Dixit, A M Kadri, Harsh Bakshi, Chaitanya Joshi, Madhvi Joshi
EPI_ISL_512064	B.J. Medical College and Civil hospital, Ahmedabad	Gujarat Biotechnology Research Centre	Dinesh Kumar, Zuber Saiyed, Komal Patel, Labdhi Pandya, Afzal Ansari, Nikha Trivedi, Pranay Shah, Kamlesh J Upadhyay, Sanjay Kapadia, Apurvasinh

			Puvar, Janvi Raval, Zarna Patel, Monika Gandhi, Pinal Trivedi, Maharshi Pandya, Nidhi Patel, Nitin Savaliya, Raghawendra Kumar, R D Dixit, A M Kadri, Harsh Bakshi, Chaitanya Joshi, Madhvi Joshi
EPI_ISL_512065	B.J. Medical College and Civil hospital, Ahmedabad	Gujarat Biotechnology Research Centre	Zuber Saiyed, Komal Patel, Labdhi Pandya, Afzal Ansari, Nikha Trivedi, Pranay Shah, Kamlesh J Upadhyay, Sanjay Kapadia, Apurvasinh Puvar, Janvi Raval, Zarna Patel, Monika Gandhi, Pinal Trivedi, Maharshi Pandya, Nidhi Patel, Nitin Savaliya, Raghawendra Kumar, Dinesh Kumar, R D Dixit, A M Kadri, Harsh Bakshi, Chaitanya Joshi, Madhvi Joshi
EPI_ISL_512066	Sardar Vallabhbhai Patel Institute of Medical Sciences & Research	Gujarat Biotechnology Research Centre	Komal Patel, Labdhi Pandya, Afzal Ansari, Nikha Trivedi, Pranay Shah, Kamlesh J Upadhyay, Sanjay Kapadia, Apurvasinh Puvar, Janvi Raval, Zarna Patel, Monika Gandhi, Pinal Trivedi, Maharshi Pandya, Nidhi Patel, Nitin Savaliya, Raghawendra Kumar, Dinesh Kumar, Zuber Saiyed, R D Dixit, A M Kadri, Harsh Bakshi, Chaitanya Joshi, Madhvi Joshi
EPI_ISL_512067	Sardar Vallabhbhai Patel Institute of Medical Sciences & Research	Gujarat Biotechnology Research Centre	Labdhi Pandya, Afzal Ansari, Nikha Trivedi, Pranay Shah, Kamlesh J Upadhyay, Sanjay Kapadia, Apurvasinh Puvar, Janvi Raval, Zarna Patel, Monika Gandhi, Pinal Trivedi, Maharshi Pandya, Nidhi Patel, Nitin Savaliya, Raghawendra Kumar, Dinesh Kumar, Zuber Saiyed, Komal Patel, R D Dixit, A M Kadri, Harsh Bakshi, Chaitanya Joshi, Madhvi Joshi
EPI_ISL_512068	Sardar Vallabhbhai Patel Institute of Medical Sciences & Research	Gujarat Biotechnology Research Centre	Afzal Ansari, Nikha Trivedi, Pranay Shah, Kamlesh J Upadhyay, Sanjay Kapadia, Apurvasinh Puvar, Janvi Raval, Zarna Patel, Monika Gandhi, Pinal Trivedi, Maharshi Pandya, Nidhi Patel, Nitin Savaliya, Raghawendra Kumar, Dinesh Kumar, Zuber Saiyed, Komal Patel, Labdhi Pandya, R D Dixit, A M Kadri, Harsh Bakshi, Chaitanya Joshi, Madhvi Joshi
EPI_ISL_512069	Sardar Vallabhbhai Patel Institute of Medical Sciences & Research	Gujarat Biotechnology Research Centre	Nikha Trivedi, Pranay Shah, Kamlesh J Upadhyay, Sanjay Kapadia, Apurvasinh Puvar, Janvi Raval, Zarna Patel, Monika Gandhi, Pinal Trivedi, Maharshi Pandya, Nidhi Patel, Nitin Savaliya, Raghawendra Kumar, Dinesh Kumar, Zuber Saiyed, Komal Patel, Labdhi Pandya, Afzal Ansari, R D Dixit, A M Kadri, Harsh Bakshi, Chaitanya Joshi, Madhvi Joshi
EPI_ISL_512070	Department of MicroBiology, Government Medical College, Surat	Gujarat Biotechnology Research Centre	Naresh Chauhan, Summaiya Mullan, Amit gamit, Apurvasinh Puvar, Janvi Raval, Zarna Patel, Monika Gandhi, Pinal Trivedi, Maharshi Pandya, Nidhi Patel, Nitin Savaliya, Raghawendra Kumar, Dinesh Kumar, Zuber Saiyed, Komal Patel, Labdhi Pandya, Afzal Ansari, Nikha Trivedi, R D Dixit, A M Kadri, Harsh Bakshi, Chaitanya Joshi, Madhvi Joshi
EPI_ISL_512071	Department of MicroBiology, Government Medical College, Surat	Gujarat Biotechnology Research Centre	Summaiya Mullan, Amit gamit, Apurvasinh Puvar, Janvi Raval, Zarna Patel, Monika Gandhi, Pinal Trivedi, Maharshi Pandya, Nidhi Patel, Nitin Savaliya, Raghawendra Kumar, Dinesh Kumar, Zuber Saiyed, Komal Patel, Labdhi Pandya, Afzal Ansari, Nikha Trivedi, Naresh Chauhan, R D Dixit, A M Kadri, Harsh Bakshi, Chaitanya Joshi, Madhvi Joshi
EPI_ISL_512072	Saikrishna Hospital, Mehsana	Gujarat Biotechnology Research Centre	Harshadbhai Parmar, Apurvasinh Puvar, Janvi Raval, Zarna Patel, Monika Gandhi, Pinal Trivedi, Maharshi Pandya, Nidhi Patel, Nitin Savaliya, Raghawendra Kumar, Dinesh Kumar, Zuber Saiyed, Komal Patel, Labdhi Pandya, Afzal Ansari, Nikha Trivedi, R D Dixit, A M Kadri, Harsh Bakshi, Chaitanya Joshi, Madhvi Joshi
EPI_ISL_512073	Saikrishna Hospital, Mehsana	Gujarat Biotechnology Research Centre	Apurvasinh Puvar, Janvi Raval, Zarna Patel, Monika Gandhi, Pinal Trivedi, Maharshi Pandya, Nidhi Patel, Nitin Savaliya, Raghawendra Kumar, Dinesh Kumar, Zuber Saiyed, Komal Patel, Labdhi Pandya, Afzal Ansari, Nikha Trivedi, Harshadbhai Parmar, R D Dixit, A M Kadri, Harsh Bakshi, Chaitanya Joshi, Madhvi Joshi
EPI_ISL_512074	Saikrishna Hospital, Mehsana	Gujarat Biotechnology Research Centre	Janvi Raval, Zarna Patel, Monika Gandhi, Pinal Trivedi, Maharshi Pandya, Nidhi Patel, Nitin Savaliya, Raghawendra Kumar, Dinesh Kumar, Zuber Saiyed, Komal Patel, Labdhi Pandya, Afzal Ansari, Nikha Trivedi, Harshadbhai Parmar, Apurvasinh Puvar, R D Dixit, A M Kadri, Harsh Bakshi, Chaitanya Joshi, Madhvi Joshi
EPI_ISL_512075	Dr. RSS Hospital, Modasa	Gujarat Biotechnology Research Centre	Monika Gandhi, Pinal Trivedi, Maharshi Pandya, Nidhi Patel, Nitin Savaliya, Raghawendra Kumar, Dinesh Kumar, Zuber Saiyed, Komal Patel, Labdhi Pandya, Afzal Ansari, Nikha Trivedi, Harsh Chaudhari, Apurvasinh Puvar, Janvi Raval, Zarna Patel, R D Dixit, A M Kadri, Harsh Bakshi, Chaitanya Joshi, Madhvi Joshi
EPI_ISL_512076	Dr. RSS Hospital, Modasa	Gujarat Biotechnology Research Centre	Pinal Trivedi, Maharshi Pandya, Nidhi Patel, Nitin Savaliya, Raghawendra Kumar, Dinesh Kumar, Zuber Saiyed, Komal Patel, Labdhi Pandya, Afzal Ansari, Nikha Trivedi, Harsh Chaudhari, Apurvasinh Puvar, Janvi Raval, Zarna Patel, Monika Gandhi, R D Dixit, A M Kadri, Harsh Bakshi, Chaitanya Joshi, Madhvi Joshi
EPI_ISL_512077	Dr. RSS Hospital, Modasa	Gujarat Biotechnology Research Centre	Maharshi Pandya, Nidhi Patel, Nitin Savaliya, Raghawendra Kumar, Dinesh Kumar, Zuber Saiyed, Komal Patel, Labdhi Pandya, Afzal Ansari, Nikha Trivedi, Harsh Chaudhari, Apurvasinh Puvar, Janvi Raval, Zarna Patel, Monika Gandhi, Pinal Trivedi, R D Dixit, A M Kadri, Harsh Bakshi, Chaitanya Joshi, Madhvi Joshi
EPI_ISL_512670	Centro Nacional De Rehabilitacion Humberto Araya Rojas (Cenare)	Inciensa, Instituto Costarricense de Investigación y Enseñanza en Nutrición y Salud	Francisco Duarte, Hebleen Porras, Claudio Soto-Garita, Estela Cordero, Adriana Godinez & Melany Calderon
EPI_ISL_512846	O.I.J. MORGUE JUDICIAL	Inciensa, Instituto Costarricense de Investigación y Enseñanza en Nutrición y Salud	Francisco Duarte, Hebleen Porras, Claudio Soto-Garita, Estela Cordero, Adriana Godinez & Melany Calderon
EPI_ISL_512874, EPI_ISL_512875, EPI_ISL_512876, EPI_ISL_512877, EPI_ISL_512878, EPI_ISL_512879, EPI_ISL_512880, EPI_ISL_512881, EPI_ISL_512882, EPI_ISL_512883, EPI_ISL_512884, EPI_ISL_512885, EPI_ISL_512886, EPI_ISL_512887, EPI_ISL_512888, EPI_ISL_512889, EPI_ISL_512890, EPI_ISL_512891, EPI_ISL_512892, EPI_ISL_512893, EPI_ISL_512894, EPI_ISL_512895, EPI_ISL_512896, EPI_ISL_512897, EPI_ISL_512898, EPI_ISL_512899, EPI_ISL_512900, EPI_ISL_512901, EPI_ISL_512902, EPI_ISL_512903			
see above	Pathogen Genomics Lab King Abdullah University of Science and Technology(KAUST)	Pathogen Genomics Lab King Abdullah University of Science and Technology(KAUST)	Raeece Naeem, Rahul P Salunke, Sharif Hala, Sara Mfarrej, Amit Kumar Subudhi, Fadwa Alofi, Fathia Ben Rached, Afrah Alsomali, Asim Khogeer, Ahmad Bakur Mahmoud, Anwar Hashem, Naif Almontashiri, Arnab Pain
EPI_ISL_512904, EPI_ISL_512905, EPI_ISL_512906, EPI_ISL_512907	Pathogen Genomics Lab King Abdullah University of Science and Technology(KAUST)	Pathogen Genomics Lab King Abdullah University of Science and Technology(KAUST)	Fathia Ben Rached, Raeece Naeem, Sharif Hala, Fadwa Alofi, Rahul P Salunke, Sara Mfarrej, Amit Kumar Subudhi, Afrah Alsomali, Asim Khogeer, Ahmad Bakur Mahmoud, Anwar Hashem, Naif Almontashiri, Arnab Pain
EPI_ISL_512920, EPI_ISL_512921, EPI_ISL_512922, EPI_ISL_512923, EPI_ISL_512924, EPI_ISL_512926, EPI_ISL_512927	Pathogen Genomics Lab King Abdullah University of Science and Technology(KAUST)	Pathogen Genomics Lab King Abdullah University of Science and Technology(KAUST)	Fadwa Alofi, Sharif Hala, Rahul P Salunke, Sara Mfarrej, Amit Kumar Subudhi, Fathia Ben Rached, Amanda, Luke, Afrah Alsomali, Asim Khogeer, Jumana Taha, Abdulaziz Alahmadi, Kahled Algithami, Raeece Naeem, Anwar Hashem, Naif Almontashiri, Arnab Pain
EPI_ISL_513002, EPI_ISL_513003, EPI_ISL_513004, EPI_ISL_513005, EPI_ISL_513006, EPI_ISL_513008, EPI_ISL_513009, EPI_ISL_513010, EPI_ISL_513012, EPI_ISL_513013, EPI_ISL_513014, EPI_ISL_513015, EPI_ISL_513016, EPI_ISL_513017, EPI_ISL_513018, EPI_ISL_513019, EPI_ISL_513020, EPI_ISL_513021, EPI_ISL_513022, EPI_ISL_513023, EPI_ISL_513024, EPI_ISL_513025, EPI_ISL_513026			
see above	Pathogen Genomics Lab King Abdullah University of Science and Technology(KAUST)	Pathogen Genomics Lab King Abdullah University of Science and Technology(KAUST)	Afrah Alsomali, Fathia Ben Rached, Raeece Naeem, Sharif Hala,Rahul P Salunke, Amanda Ooi, Luke Esau, Sara Mfarrej, Amit Kumar Subudhi, Fadwa Alofi, Asim Khogeer, Kahled Algithami, Anwar Hashem, Naif Almontashiri, Arnab Pain
EPI_ISL_513027, EPI_ISL_513028, EPI_ISL_513029, EPI_ISL_513032, EPI_ISL_513033, EPI_ISL_513034, EPI_ISL_513035, EPI_ISL_513036, EPI_ISL_513037, EPI_ISL_513038, EPI_ISL_513039, EPI_ISL_513040, EPI_ISL_513063			
see above	Pathogen Genomics Lab King Abdullah University of Science and Technology(KAUST)	Pathogen Genomics Lab King Abdullah University of Science and Technology(KAUST)	Rahul P Salunke, Sharif Hala, Raeece Naeem, Sara Mfarrej, Amit Kumar Subudhi, Amanda Ooi, Luke Esau, Fadwa Alofi, Fathia Ben Rached, Afrah Alsomali, Asim Khogeer, Ahmad Bakur Mahmoud, Anwar Hashem, Naif Almontashiri, Arnab Pain
EPI_ISL_513064, EPI_ISL_513065, EPI_ISL_513066, EPI_ISL_513067, EPI_ISL_513068, EPI_ISL_513069, EPI_ISL_513070, EPI_ISL_513071, EPI_ISL_513072, EPI_ISL_513073, EPI_ISL_513074, EPI_ISL_513075, EPI_ISL_513076			
see above	Pathogen Genomics Lab King Abdullah University of Science and Technology(KAUST)	Pathogen Genomics Lab King Abdullah University of Science and Technology(KAUST)	Raeece Naeem, Rahul P Salunke, Sharif Hala, Sara Mfarrej, Amit Kumar Subudhi, Fadwa Alofi, Fathia Ben Rached, Afrah Alsomali, Asim Khogeer, Ahmad Bakur Mahmoud, Anwar Hashem, Naif Almontashiri, Arnab Pain
EPI_ISL_513078, EPI_ISL_513079, EPI_ISL_513081, EPI_ISL_513082, EPI_ISL_513083, EPI_ISL_513086, EPI_ISL_513087, EPI_ISL_513089, EPI_ISL_513090, EPI_ISL_513092, EPI_ISL_513093, EPI_ISL_513094, EPI_ISL_513095, EPI_ISL_513096, EPI_ISL_513097, EPI_ISL_513098, EPI_ISL_513099, EPI_ISL_513100, EPI_ISL_513101, EPI_ISL_513102, EPI_ISL_513103, EPI_ISL_513104, EPI_ISL_513105, EPI_ISL_513106, EPI_ISL_513107, EPI_ISL_513108, EPI_ISL_513109, EPI_ISL_513111, EPI_ISL_513112, EPI_ISL_513113, EPI_ISL_513114, EPI_ISL_513115, EPI_ISL_513116, EPI_ISL_513117, EPI_ISL_513118, EPI_ISL_513119, EPI_ISL_513120			
see above	Pathogen Genomics Lab King Abdullah University of Science and Technology(KAUST)	Pathogen Genomics Lab King Abdullah University of Science and Technology(KAUST)	Fathia Ben Rached, Raeece Naeem, Sharif Hala, Fadwa Alofi, Rahul P Salunke, Sara Mfarrej, Amit Kumar Subudhi, Afrah Alsomali, Asim Khogeer, Ahmad Bakur Mahmoud, Anwar Hashem, Naif Almontashiri, Arnab Pain
EPI_ISL_513190, EPI_ISL_513191, EPI_ISL_513192, EPI_ISL_513193, EPI_ISL_513194, EPI_ISL_513196, EPI_ISL_513197	Pathogen Genomics Lab King Abdullah University of Science and Technology(KAUST)	Pathogen Genomics Lab King Abdullah University of Science and Technology(KAUST)	Afrah Alsomali, Fathia Ben Rached, Raeece Naeem, Sharif Hala,Rahul P Salunke, Amanda Ooi, Luke Esau, Sara Mfarrej, Amit Kumar Subudhi, Fadwa Alofi, Asim Khogeer, Kahled Algithami, Anwar Hashem, Naif Almontashiri, Arnab Pain

EPI_ISL_513199, EPI_ISL_513200, EPI_ISL_513201, EPI_ISL_513202, EPI_ISL_513203, EPI_ISL_513204, EPI_ISL_513207	Pathogen Genomics Lab King Abdullah University of Science and Technology(KAUST)	Pathogen Genomics Lab King Abdullah University of Science and Technology(KAUST)	Rahul P Salunke, Sharif Hala, Raeece Naeem, Sara Mfarrej, Amit Kumar Subudhi, Amanda Ooi, Luke Esau, Fadwa Alofi, Fathia Ben Rached, Afrah Alsomali, Asim Khogeer, Ahmad Bakur Mahmoud, Anwar Hashem, Naif Almontashiri, Arnab Pain
EPI_ISL_513210, EPI_ISL_513212, EPI_ISL_513213	Pathogen Genomics Lab King Abdullah University of Science and Technology(KAUST)	Pathogen Genomics Lab King Abdullah University of Science and Technology(KAUST)	Raeece Naeem, Rahul P Salunke, Sharif Hala, Sara Mfarrej, Amit Kumar Subudhi, Fadwa Alofi, Fathia Ben Rached, Afrah Alsomali, Asim Khogeer, Ahmad Bakur Mahmoud, Anwar Hashem, Naif Almontashiri, Arnab Pain
EPI_ISL_514093	National Scientific Center for Especially Dangerous Infections (NSCEDI)	Kazakh National Agrarian University (KazNAU) TreeGene LLP Genetic Laboratory	Beloussov Vyacheslav, Strochkov Vitaliy, Tabynov Kaissar, Sandybayev Nurlan, Tabynov Kairat, Turebekov Nurkeldy, Granica Joanna, Solomadin Maksim, Yerubayev Toktassyn, Yespolov Tlektes
EPI_ISL_514127	National Scientific Center for Especially Dangerous Infections (NSCEDI)	Kazakh National Agrarian University (KazNAU) TreeGene LLP Genetic Laboratory	Tabynov Kairat, Beloussov Vyacheslav, Strochkov Vitaliy, Sandybayev Nurlan, Tabynov Kaissar, Turebekov Nurkeldy, Granica Joanna, Solomadin Maksim, Yerubayev Toktassyn, Yespolov Tlektes
EPI_ISL_514138	Rondônia Central Public Health Laboratory (LACEN/RO), vinculated to State Health Secretariat of Rondônia (SESAU/RO)	Molecular Virology Laboratory of Oswaldo Cruz Foundation of Rondônia	Luan Felipo Botelho-Souza, Felipe Souza Nogueira-Lima, Tárcio Peixoto Roca, Alcione de Oliveira dos Santos, Felipe Gomes Naveca, Adriana Cristina Salvador Maia, Cicileia Correia da Silva, Aline Linhares Ferreira de Melo Mendonça, Celina Aparecida Bertoni Lugtenburg, Camila Flávia Gomes Azzi, Juliana Loca Furtado, Suelen Cavalcante, Rita de Cássia Pontello Rampazzo, Caio Henrique Nemeth Santos, Alice Paula Di Sabatino Guimarães, Jansen Fernandes de Medeiros, Fernando Rodrigues Máximo, Juan Miguel Vilallobos-Salcedo and Deusilene Souza Vieira
EPI_ISL_514434	NSTU COVID-19 Diagnostic Center,	NSU Genome Research Institute (NGRI), North South University	Dr. Muhammad Maqsud Hossain, Aura Rahman, Prof. Firoz Ahmed, Tahrima Huq, Abdus Sadique, Jahidul Alam, Md Aminul Islam, Prof. Md. Didar-Ul-Alam, Prof. Kazi Nadim Hasan, Prof. Abdul Khaleque, Prof, Hasan Mahmud Reza
EPI_ISL_514435	Dr. RSS Hospital, Modasa	Gujarat Biotechnology Research Centre	Harsh Chaudhari, Zarna Patel, Monika Gandhi, Pinal Trivedi, Maharshi Pandya, Nidhi Patel, Nitin Savaliya, Raghawendra Kumar, Dinesh Kumar, Zuber Saiyed, Komal Patel, Labdhi Pandya, Afzal Ansari, Nikha Trivedi, Apurvasinh Puvar, Janvi Raval, R D Dixit, A M Kadri, Harsh Bakshi, Chaitanya Joshi, Madhvi Joshi
EPI_ISL_514436	Dr. RSS Hospital, Modasa	Gujarat Biotechnology Research Centre	Zarna Patel, Monika Gandhi, Pinal Trivedi, Maharshi Pandya, Nidhi Patel, Nitin Savaliya, Raghawendra Kumar, Dinesh Kumar, Zuber Saiyed, Komal Patel, Labdhi Pandya, Afzal Ansari, Nikha Trivedi, Harsh Chaudhari, Apurvasinh Puvar, Janvi Raval, R D Dixit, A M Kadri, Harsh Bakshi, Chaitanya Joshi, Madhvi Joshi
EPI_ISL_514437	Dr. RSS Hospital, Modasa	Gujarat Biotechnology Research Centre	Nidhi Patel, Nitin Savaliya, Raghawendra Kumar, Dinesh Kumar, Zuber Saiyed, Komal Patel, Labdhi Pandya, Afzal Ansari, Nikha Trivedi, Harsh Chaudhari, Apurvasinh Puvar, Janvi Raval, Zarna Patel, Monika Gandhi, Pinal Trivedi, Maharshi Pandya, R D Dixit, A M Kadri, Harsh Bakshi, Chaitanya Joshi, Madhvi Joshi
EPI_ISL_514440	NSTU COVID-19 Diagnostic Center	NSU Genome Research Institute (NGRI), North South University	Dr. Muhammad Maqsud Hossain, Aura Rahman, Prof. Firoz Ahmed, Tahrima Huq, Abdus Sadique, Tamanna Afroze, Jahidul Alam, Md Aminul Islam, Prof. Md. Didar-Ul-Alam, Prof. Kazi Nadim Hasan, Prof. Abdul Khaleque, Prof, Hasan Mahmud Reza
EPI_ISL_514441	NSTU COVID-19 Diagnostic Center	NSU Genome Research Institute (NGRI), North South University	Dr. Muhammad Maqsud Hossain, Aura Rahman, Prof. Firoz Ahmed, Tahrima Huq, Abdus Sadique, Jahidul Alam, Md Aminul Islam, Prof. Md. Didar-Ul-Alam, Prof. Kazi Nadim Hasan, Prof. Abdul Khaleque, Prof, Hasan Mahmud Reza
EPI_ISL_514442, EPI_ISL_514580	NSTU COVID-19 Diagnostic Center	NSU Genome Research Institute (NGRI), North South University	Dr. Muhammad Maqsud Hossain, Aura Rahman, Prof. Firoz Ahmed, Tahrima Huq, Abdus Sadique, Jahidul Alam, Tamanna Afroze, Md Aminul Islam, Prof. Md. Didar-Ul-Alam, Prof. Kazi Nadim Hasan, Prof. Abdul Khaleque, Prof, Hasan Mahmud Reza
EPI_ISL_514581	B.J. Medical College and Civil hospital, Ahmedabad	Gujarat Biotechnology Research Centre	Nitin Savaliya, Raghawendra Kumar, Dinesh Kumar, Zuber Saiyed, Komal Patel, Labdhi Pandya, Afzal Ansari, Nikha Trivedi, Pranay Shah, Kamlesh J Upadhyay, Sanjay Kapadia, Apurvasinh Puvar, Janvi Raval, Zarna Patel, Monika Gandhi, Pinal Trivedi, Maharshi Pandya, Nidhi Patel, R D Dixit, A M Kadri, Harsh Bakshi, Chaitanya Joshi, Madhvi Joshi
EPI_ISL_514582	B.J. Medical College and Civil hospital, Ahmedabad	Gujarat Biotechnology Research Centre	Raghawendra Kumar, Dinesh Kumar, Zuber Saiyed, Komal Patel, Labdhi Pandya, Afzal Ansari, Nikha Trivedi, Pranay Shah, Kamlesh J Upadhyay, Sanjay Kapadia, Apurvasinh Puvar, Janvi Raval, Zarna Patel, Monika Gandhi, Pinal Trivedi, Maharshi Pandya, Nidhi Patel, Nitin Savaliya, R D Dixit, A M Kadri, Harsh Bakshi, Chaitanya Joshi, Madhvi Joshi
EPI_ISL_514583	B.J. Medical College and Civil hospital, Ahmedabad	Gujarat Biotechnology Research Centre	Dinesh Kumar, Zuber Saiyed, Komal Patel, Labdhi Pandya, Afzal Ansari, Nikha Trivedi, Pranay Shah, Kamlesh J Upadhyay, Sanjay Kapadia, Apurvasinh Puvar, Janvi Raval, Zarna Patel, Monika Gandhi, Pinal Trivedi, Maharshi Pandya, Nidhi Patel, Nitin Savaliya, Raghawendra Kumar, R D Dixit, A M Kadri, Harsh Bakshi, Chaitanya Joshi, Madhvi Joshi
EPI_ISL_514584	B.J. Medical College and Civil hospital, Ahmedabad	Gujarat Biotechnology Research Centre	Zuber Saiyed, Komal Patel, Labdhi Pandya, Afzal Ansari, Nikha Trivedi, Pranay Shah, Kamlesh J Upadhyay, Sanjay Kapadia, Apurvasinh Puvar, Janvi Raval, Zarna Patel, Monika Gandhi, Pinal Trivedi, Maharshi Pandya, Nidhi Patel, Nitin Savaliya, Raghawendra Kumar, Dinesh Kumar, R D Dixit, A M Kadri, Harsh Bakshi, Chaitanya Joshi, Madhvi Joshi
EPI_ISL_514585	B.J. Medical College and Civil hospital, Ahmedabad	Gujarat Biotechnology Research Centre	Komal Patel, Labdhi Pandya, Afzal Ansari, Nikha Trivedi, Pranay Shah, Kamlesh J Upadhyay, Sanjay Kapadia, Apurvasinh Puvar, Janvi Raval, Zarna Patel, Monika Gandhi, Pinal Trivedi, Maharshi Pandya, Nidhi Patel, Nitin Savaliya, Raghawendra Kumar, Dinesh Kumar, Zuber Saiyed, R D Dixit, A M Kadri, Harsh Bakshi, Chaitanya Joshi, Madhvi Joshi
EPI_ISL_514586	B.J. Medical College and Civil hospital, Ahmedabad	Gujarat Biotechnology Research Centre	Labdhi Pandya, Afzal Ansari, Nikha Trivedi, Pranay Shah, Kamlesh J Upadhyay, Sanjay Kapadia, Apurvasinh Puvar, Janvi Raval, Zarna Patel, Monika Gandhi, Pinal Trivedi, Maharshi Pandya, Nidhi Patel, Nitin Savaliya, Raghawendra Kumar, Dinesh Kumar, Zuber Saiyed, Komal Patel, R D Dixit, A M Kadri, Harsh Bakshi, Chaitanya Joshi, Madhvi Joshi
EPI_ISL_514587	B.J. Medical College and Civil hospital, Ahmedabad	Gujarat Biotechnology Research Centre	Afzal Ansari, Nikha Trivedi, Pranay Shah, Kamlesh J Upadhyay, Sanjay Kapadia, Apurvasinh Puvar, Janvi Raval, Zarna Patel, Monika Gandhi, Pinal Trivedi, Maharshi Pandya, Nidhi Patel, Nitin Savaliya, Raghawendra Kumar, Dinesh Kumar, Zuber Saiyed, Komal Patel, Labdhi Pandya, R D Dixit, A M Kadri, Harsh Bakshi, Chaitanya Joshi, Madhvi Joshi
EPI_ISL_514588	B.J. Medical College and Civil hospital, Ahmedabad	Gujarat Biotechnology Research Centre	Nikha Trivedi, Pranay Shah, Kamlesh J Upadhyay, Sanjay Kapadia, Apurvasinh Puvar, Janvi Raval, Zarna Patel, Monika Gandhi, Pinal Trivedi, Maharshi Pandya, Nidhi Patel, Nitin Savaliya, Raghawendra Kumar, Dinesh Kumar, Zuber Saiyed, Komal Patel, Labdhi Pandya, Afzal Ansari, R D Dixit, A M Kadri, Harsh Bakshi, Chaitanya Joshi, Madhvi Joshi
EPI_ISL_514589	B.J. Medical College and Civil hospital, Ahmedabad	Gujarat Biotechnology Research Centre	Pranay Shah, Kamlesh J Upadhyay, Sanjay Kapadia, Apurvasinh Puvar, Janvi Raval, Zarna Patel, Monika Gandhi, Pinal Trivedi, Maharshi Pandya, Nidhi Patel, Nitin Savaliya, Raghawendra Kumar, Dinesh Kumar, Zuber Saiyed, Komal Patel, Labdhi Pandya, Afzal Ansari, Nikha Trivedi, R D Dixit, A M Kadri, Harsh Bakshi, Chaitanya Joshi, Madhvi Joshi
EPI_ISL_514590	B.J. Medical College and Civil hospital, Ahmedabad	Gujarat Biotechnology Research Centre	Kamlesh J Upadhyay, Sanjay Kapadia, Apurvasinh Puvar, Janvi Raval, Zarna Patel, Monika Gandhi, Pinal Trivedi, Maharshi Pandya, Nidhi Patel, Nitin Savaliya, Raghawendra Kumar, Dinesh Kumar, Zuber Saiyed, Komal Patel, Labdhi Pandya, Afzal Ansari, Nikha Trivedi, Pranay Shah, R D Dixit, A M Kadri, Harsh Bakshi, Chaitanya Joshi, Madhvi Joshi
EPI_ISL_514591	B.J. Medical College and Civil hospital, Ahmedabad	Gujarat Biotechnology Research Centre	Sanjay Kapadia,Apurvasinh Puvar,Janvi Raval,Zarna Patel, Monika Gandhi, Pinal Trivedi, Maharshi Pandya, Nidhi Patel, Nitin Savaliya, Raghawendra Kumar, Dinesh Kumar, Zuber Saiyed, Komal Patel, Labdhi Pandya, Afzal Ansari, Nikha Trivedi, Pranay Shah, Kamlesh J Upadhyay, R D Dixit, A M Kadri, Harsh Bakshi, Chaitanya Joshi, Madhvi Joshi
EPI_ISL_514592	B.J. Medical College and Civil hospital, Ahmedabad	Gujarat Biotechnology Research Centre	Apurvasinh Puvar, Janvi Raval, Zarna Patel, Monika Gandhi, Pinal Trivedi, Maharshi Pandya, Nidhi Patel, Nitin Savaliya, Raghawendra Kumar, Dinesh Kumar, Zuber Saiyed, Komal Patel, Labdhi Pandya, Afzal Ansari, Nikha Trivedi, Pranay Shah, Kamlesh J Upadhyay, Sanjay Kapadia, R D Dixit, A M Kadri, Harsh Bakshi, Chaitanya Joshi, Madhvi Joshi
EPI_ISL_514593	B.J. Medical College and Civil hospital, Ahmedabad	Gujarat Biotechnology Research Centre	Janvi Raval, Zarna Patel, Monika Gandhi, Pinal Trivedi, Maharshi Pandya, Nidhi Patel, Nitin Savaliya, Raghawendra Kumar, Dinesh Kumar, Zuber Saiyed, Komal Patel, Labdhi Pandya, Afzal Ansari, Nikha Trivedi, Pranay Shah, Kamlesh J Upadhyay, Sanjay Kapadia, Apurvasinh Puvar, R D Dixit, A M Kadri, Harsh Bakshi, Chaitanya Joshi, Madhvi Joshi
EPI_ISL_514594	B.J. Medical College and Civil hospital, Ahmedabad	Gujarat Biotechnology Research Centre	Zarna Patel, Monika Gandhi, Pinal Trivedi, Maharshi Pandya, Nidhi Patel, Nitin Savaliya, Raghawendra Kumar, Dinesh Kumar, Zuber Saiyed, Komal Patel, Labdhi Pandya, Afzal Ansari, Nikha Trivedi, Pranay Shah, Kamlesh J Upadhyay, Sanjay Kapadia, Apurvasinh Puvar, Janvi Raval, R D Dixit, A M Kadri, Harsh Bakshi, Chaitanya Joshi, Madhvi Joshi
EPI_ISL_514595	B.J. Medical College and Civil hospital, Ahmedabad	Gujarat Biotechnology Research Centre	Monika Gandhi, Pinal Trivedi, Maharshi Pandya, Nidhi Patel, Nitin Savaliya, Raghawendra Kumar, Dinesh Kumar, Zuber Saiyed, Komal Patel, Labdhi Pandya,

			Afzal Ansari, Nikha Trivedi, Pranay Shah, Kamlesh J Upadhyay, Sanjay Kapadia, Apurvasinh Puvar, Janvi Raval, Zarna Patel, R D Dixit, A M Kadri, Harsh Bakshi, Chaitanya Joshi, Madhvi Joshi
EPI_ISL_514596	B.J. Medical College and Civil hospital, Ahmedabad	Gujarat Biotechnology Research Centre	Pinal Trivedi, Maharshi Pandya, Nidhi Patel, Nitin Savaliya, Raghawendra Kumar, Dinesh Kumar, Zuber Saiyed, Komal Patel, Labdhi Pandya, Afzal Ansari, Nikha Trivedi, Pranay Shah, Kamlesh J Upadhyay, Sanjay Kapadia, Apurvasinh Puvar, Janvi Raval, Zarna Patel, Monika Gandhi, R D Dixit, A M Kadri, Harsh Bakshi, Chaitanya Joshi, Madhvi Joshi
EPI_ISL_514597	B.J. Medical College and Civil hospital, Ahmedabad	Gujarat Biotechnology Research Centre	Maharshi Pandya, Nidhi Patel, Nitin Savaliya, Raghawendra Kumar, Dinesh Kumar, Zuber Saiyed, Komal Patel, Labdhi Pandya, Afzal Ansari, Nikha Trivedi, Pranay Shah, Kamlesh J Upadhyay, Sanjay Kapadia, Apurvasinh Puvar, Janvi Raval, Zarna Patel, Monika Gandhi, Pinal Trivedi, R D Dixit, A M Kadri, Harsh Bakshi, Chaitanya Joshi, Madhvi Joshi
EPI_ISL_514598	B.J. Medical College and Civil hospital, Ahmedabad	Gujarat Biotechnology Research Centre	Nidhi Patel, Nitin Savaliya, Raghawendra Kumar, Dinesh Kumar, Zuber Saiyed, Komal Patel, Labdhi Pandya, Afzal Ansari, Nikha Trivedi, Pranay Shah, Kamlesh J Upadhyay, Sanjay Kapadia, Apurvasinh Puvar, Janvi Raval, Zarna Patel, Monika Gandhi, Pinal Trivedi, Maharshi Pandya, R D Dixit, A M Kadri, Harsh Bakshi, Chaitanya Joshi, Madhvi Joshi
EPI_ISL_514599	B.J. Medical College and Civil hospital, Ahmedabad	Gujarat Biotechnology Research Centre	Nitin Savaliya, Raghawendra Kumar, Dinesh Kumar, Zuber Saiyed, Komal Patel, Labdhi Pandya, Afzal Ansari, Nikha Trivedi, Pranay Shah, Kamlesh J Upadhyay, Sanjay Kapadia, Apurvasinh Puvar, Janvi Raval, Zarna Patel, Monika Gandhi, Pinal Trivedi, Maharshi Pandya, Nidhi Patel, R D Dixit, A M Kadri, Harsh Bakshi, Chaitanya Joshi, Madhvi Joshi
EPI_ISL_514600	B.J. Medical College and Civil hospital, Ahmedabad	Gujarat Biotechnology Research Centre	Raghawendra Kumar, Dinesh Kumar, Zuber Saiyed, Komal Patel, Labdhi Pandya, Afzal Ansari, Nikha Trivedi, Pranay Shah, Kamlesh J Upadhyay, Sanjay Kapadia, Apurvasinh Puvar, Janvi Raval, Zarna Patel, Monika Gandhi, Pinal Trivedi, Maharshi Pandya, Nidhi Patel, Nitin Savaliya, R D Dixit, A M Kadri, Harsh Bakshi, Chaitanya Joshi, Madhvi Joshi
EPI_ISL_514601	B.J. Medical College and Civil hospital, Ahmedabad	Gujarat Biotechnology Research Centre	Dinesh Kumar, Zuber Saiyed, Komal Patel, Labdhi Pandya, Afzal Ansari, Nikha Trivedi, Pranay Shah, Kamlesh J Upadhyay, Sanjay Kapadia, Apurvasinh Puvar, Janvi Raval, Zarna Patel, Monika Gandhi, Pinal Trivedi, Maharshi Pandya, Nidhi Patel, Nitin Savaliya, Raghawendra Kumar, R D Dixit, A M Kadri, Harsh Bakshi, Chaitanya Joshi, Madhvi Joshi
EPI_ISL_514602	B.J. Medical College and Civil hospital, Ahmedabad	Gujarat Biotechnology Research Centre	Zuber Saiyed, Komal Patel, Labdhi Pandya, Afzal Ansari, Nikha Trivedi, Pranay Shah, Kamlesh J Upadhyay, Sanjay Kapadia, Apurvasinh Puvar, Janvi Raval, Zarna Patel, Monika Gandhi, Pinal Trivedi, Maharshi Pandya, Nidhi Patel, Nitin Savaliya, Raghawendra Kumar, Dinesh Kumar, R D Dixit, A M Kadri, Harsh Bakshi, Chaitanya Joshi, Madhvi Joshi
EPI_ISL_514603	B.J. Medical College and Civil hospital, Ahmedabad	Gujarat Biotechnology Research Centre	Komal Patel, Labdhi Pandya, Afzal Ansari, Nikha Trivedi, Pranay Shah, Kamlesh J Upadhyay, Sanjay Kapadia, Apurvasinh Puvar, Janvi Raval, Zarna Patel, Monika Gandhi, Pinal Trivedi, Maharshi Pandya, Nidhi Patel, Nitin Savaliya, Raghawendra Kumar, Dinesh Kumar, Zuber Saiyed, R D Dixit, A M Kadri, Harsh Bakshi, Chaitanya Joshi, Madhvi Joshi
EPI_ISL_514604	B.J. Medical College and Civil hospital, Ahmedabad	Gujarat Biotechnology Research Centre	Labdhi Pandya, Afzal Ansari, Nikha Trivedi, Pranay Shah, Kamlesh J Upadhyay, Sanjay Kapadia, Apurvasinh Puvar, Janvi Raval, Zarna Patel, Monika Gandhi, Pinal Trivedi, Maharshi Pandya, Nidhi Patel, Nitin Savaliya, Raghawendra Kumar, Dinesh Kumar, Zuber Saiyed, Komal Patel, R D Dixit, A M Kadri, Harsh Bakshi, Chaitanya Joshi, Madhvi Joshi
EPI_ISL_514605	B.J. Medical College and Civil hospital, Ahmedabad	Gujarat Biotechnology Research Centre	Afzal Ansari, Nikha Trivedi, Pranay Shah, Kamlesh J Upadhyay, Sanjay Kapadia, Apurvasinh Puvar, Janvi Raval, Zarna Patel, Monika Gandhi, Pinal Trivedi, Maharshi Pandya, Nidhi Patel, Nitin Savaliya, Raghawendra Kumar, Dinesh Kumar, Zuber Saiyed, Komal Patel, Labdhi Pandya, R D Dixit, A M Kadri, Harsh Bakshi, Chaitanya Joshi, Madhvi Joshi
EPI_ISL_514606	B.J. Medical College and Civil hospital, Ahmedabad	Gujarat Biotechnology Research Centre	Nikha Trivedi, Pranay Shah, Kamlesh J Upadhyay, Sanjay Kapadia, Apurvasinh Puvar, Janvi Raval, Zarna Patel, Monika Gandhi, Pinal Trivedi, Maharshi Pandya, Nidhi Patel, Nitin Savaliya, Raghawendra Kumar, Dinesh Kumar, Zuber Saiyed, Komal Patel, Labdhi Pandya, Afzal Ansari, R D Dixit, A M Kadri, Harsh Bakshi, Chaitanya Joshi, Madhvi Joshi
EPI_ISL_514607	B.J. Medical College and Civil hospital, Ahmedabad	Gujarat Biotechnology Research Centre	Pranay Shah, Kamlesh J Upadhyay, Sanjay Kapadia, Apurvasinh Puvar, Janvi Raval, Zarna Patel, Monika Gandhi, Pinal Trivedi, Maharshi Pandya, Nidhi Patel, Nitin Savaliya, Raghawendra Kumar, Dinesh Kumar, Zuber Saiyed, Komal Patel, Labdhi Pandya, Afzal Ansari, Nikha Trivedi, R D Dixit, A M Kadri, Harsh Bakshi, Chaitanya Joshi, Madhvi Joshi
EPI_ISL_514608	B.J. Medical College and Civil hospital, Ahmedabad	Gujarat Biotechnology Research Centre	Kamlesh J Upadhyay, Sanjay Kapadia, Apurvasinh Puvar, Janvi Raval, Zarna Patel, Monika Gandhi, Pinal Trivedi, Maharshi Pandya, Nidhi Patel, Nitin Savaliya, Raghawendra Kumar, Dinesh Kumar, Zuber Saiyed, Komal Patel, Labdhi Pandya, Afzal Ansari, Nikha Trivedi, Pranay Shah, R D Dixit, A M Kadri, Harsh Bakshi, Chaitanya Joshi, Madhvi Joshi
EPI_ISL_514609	B.J. Medical College and Civil hospital, Ahmedabad	Gujarat Biotechnology Research Centre	Sanjay Kapadia, Apurvasinh Puvar, Janvi Raval, Zarna Patel, Monika Gandhi, Pinal Trivedi, Maharshi Pandya, Nidhi Patel, Nitin Savaliya, Raghawendra Kumar, Dinesh Kumar, Zuber Saiyed, Komal Patel, Labdhi Pandya, Afzal Ansari, Nikha Trivedi, Pranay Shah, Kamlesh J Upadhyay, R D Dixit, A M Kadri, Harsh Bakshi, Chaitanya Joshi, Madhvi Joshi
EPI_ISL_514610	GMERS Medical College & Hospital, Gotri, Vadodara	Gujarat Biotechnology Research Centre	Meenakshi Shah, Neena Doshi, Varsha Godbole, Apurvasinh Puvar, Janvi Raval, Zarna Patel, Monika Gandhi, Pinal Trivedi, Maharshi Pandya, Nidhi Patel, Nitin Savaliya, Raghawendra Kumar, Dinesh Kumar, Zuber Saiyed, Komal Patel, Labdhi Pandya, Afzal Ansari, Nikha Trivedi, Pranay Shah, Kamlesh J Upadhyay, Sanjay Kapadia, R D Dixit, A M Kadri, Harsh Bakshi, Chaitanya Joshi, Madhvi Joshi,
EPI_ISL_514611	GMERS Medical College and Hospital, Gandhinagar	Gujarat Biotechnology Research Centre	Seema Bhatt, Gaurishankar Shrimali, Bhavesh Modi, Bharti Rajani, Apurvasinh Puvar, Janvi Raval, Zarna Patel, Monika Gandhi, Pinal Trivedi, Maharshi Pandya, Nidhi Patel, Nitin Savaliya, Raghawendra Kumar, Dinesh Kumar, Zuber Saiyed, Komal Patel, Labdhi Pandya, Afzal Ansari, Nikha Trivedi, Pranay Shah, Kamlesh J Upadhyay, Sanjay Kapadia, R D Dixit, A M Kadri, Harsh Bakshi, Chaitanya Joshi, Madhvi Joshi
EPI_ISL_514612	GMERS Medical College and Hospital, Gandhinagar	Gujarat Biotechnology Research Centre	Gaurishankar Shrimali, Bhavesh Modi, Bharti Rajani, Apurvasinh Puvar, Janvi Raval, Zarna Patel, Monika Gandhi, Pinal Trivedi, Maharshi Pandya, Nidhi Patel, Nitin Savaliya, Raghawendra Kumar, Dinesh Kumar, Zuber Saiyed, Komal Patel, Labdhi Pandya, Afzal Ansari, Nikha Trivedi, Pranay Shah, Kamlesh J Upadhyay, Sanjay Kapadia, Seema Bhatt, R D Dixit, A M Kadri, Harsh Bakshi, Chaitanya Joshi, Madhvi Joshi
EPI_ISL_514613, EPI_ISL_514615	NSTU COVID-19 Diagnostic Center	NSU Genome Research Institute (NGRI), North South University	Dr. Muhammad Maqsud Hossain, Aura Rahman, Prof. Firoz Ahmed, Tahrima Huq, Abdus Sadique, Jahidul Alam, Tamanna Afroze, Md Aminul Islam, Prof. Md. Didar-Ul-Alam, Prof. Kazi Nadim Hasan, Prof. Abdul Khaleque, Prof, Hasan Mahmud Reza
EPI_ISL_515466	Discovery DNA	Discovery DNA	Dustin Hittel, Marina Kerr, Leo Dimnik, Desmond Koo, Alice Li, Aneal Khan
EPI_ISL_515525	National Influenza Center - Instituto Adolfo Lutz	Instituto Adolfo Lutz, Interdiciplinary Procedures Center, Strategic Laboratory	Claudio Tavares Sacchi, Claudia Regina Gonçalves, Erica Valessa Ramos Gomes
EPI_ISL_515568, EPI_ISL_515569, EPI_ISL_515570, EPI_ISL_515573, EPI_ISL_515574, EPI_ISL_515575, EPI_ISL_515579, EPI_ISL_515580, EPI_ISL_515582, EPI_ISL_515584, EPI_ISL_515585, EPI_ISL_515586, EPI_ISL_515589, EPI_ISL_515592, EPI_ISL_515593, EPI_ISL_515594, EPI_ISL_515596, EPI_ISL_515597, EPI_ISL_515600, EPI_ISL_515601, EPI_ISL_515602, EPI_ISL_515603, EPI_ISL_515604, EPI_ISL_515606, EPI_ISL_515607, EPI_ISL_515610, EPI_ISL_515612, EPI_ISL_515613, EPI_ISL_515615, EPI_ISL_515616, EPI_ISL_515619, EPI_ISL_515622, EPI_ISL_515623, EPI_ISL_515627, EPI_ISL_515628, EPI_ISL_515631, EPI_ISL_515633, EPI_ISL_515634, EPI_ISL_515635, EPI_ISL_515639, EPI_ISL_515640, EPI_ISL_515641, EPI_ISL_515642, EPI_ISL_515643, EPI_ISL_515644, EPI_ISL_515645, EPI_ISL_515647, EPI_ISL_515649, EPI_ISL_515650, EPI_ISL_515651, EPI_ISL_515652, EPI_ISL_515653, EPI_ISL_515654, EPI_ISL_515655, EPI_ISL_515656, EPI_ISL_515657, EPI_ISL_515658, EPI_ISL_515659, EPI_ISL_515660, EPI_ISL_515661, EPI_ISL_515662, EPI_ISL_515665, EPI_ISL_515666, EPI_ISL_515668, EPI_ISL_515669, EPI_ISL_515671, EPI_ISL_515672, EPI_ISL_515674, EPI_ISL_515675, EPI_ISL_515676, EPI_ISL_515678, EPI_ISL_515679, EPI_ISL_515680, EPI_ISL_515681, EPI_ISL_515682, EPI_ISL_515683, EPI_ISL_515684, EPI_ISL_515685, EPI_ISL_515686, EPI_ISL_515687, EPI_ISL_515688, EPI_ISL_515689, EPI_ISL_515690, EPI_ISL_515691, EPI_ISL_515692, EPI_ISL_515693, EPI_ISL_515694, EPI_ISL_515696, EPI_ISL_515697, EPI_ISL_515698, EPI_ISL_515699, EPI_ISL_515700, EPI_ISL_515701, EPI_ISL_515702, EPI_ISL_515703, EPI_ISL_515704, EPI_ISL_515705, EPI_ISL_515706, EPI_ISL_515707, EPI_ISL_515708, EPI_ISL_515709, EPI_ISL_515710, EPI_ISL_515711, EPI_ISL_515712, EPI_ISL_515714, EPI_ISL_515715, EPI_ISL_515716, EPI_ISL_515718, EPI_ISL_515719, EPI_ISL_515720, EPI_ISL_515721, EPI_ISL_515722, EPI_ISL_515723, EPI_ISL_515724, EPI_ISL_515725, EPI_ISL_515728, EPI_ISL_515729, EPI_ISL_515730, EPI_ISL_515731, EPI_ISL_515732, EPI_ISL_515733, EPI_ISL_515734, EPI_ISL_515736, EPI_ISL_515737, EPI_ISL_515738, EPI_ISL_515739, EPI_ISL_515740, EPI_ISL_515741, EPI_ISL_515743, EPI_ISL_515744, EPI_ISL_515745, EPI_ISL_515747, EPI_ISL_515748, EPI_ISL_515749, EPI_ISL_515751, EPI_ISL_515752, EPI_ISL_515753, EPI_ISL_515754, EPI_ISL_515756, EPI_ISL_515757, EPI_ISL_515758, EPI_ISL_515759, EPI_ISL_515760, EPI_ISL_515762, EPI_ISL_515763, EPI_ISL_515764, EPI_ISL_515767, EPI_ISL_515769, EPI_ISL_515771, EPI_ISL_515773, EPI_ISL_515775, EPI_ISL_515776, EPI_ISL_515777, EPI_ISL_515778, EPI_ISL_515780, EPI_ISL_515781, EPI_ISL_515782, EPI_ISL_515783, EPI_ISL_515784, EPI_ISL_515787, EPI_ISL_515789, EPI_ISL_515790, EPI_ISL_515791, EPI_ISL_515792, EPI_ISL_515794, EPI_ISL_515796, EPI_ISL_515797, EPI_ISL_515799			
see above	NHLS-IALCH	KRISP, KZN Research Innovation and Sequencing Platform	Giandhari J, Pillay S, Lessells R, Mdlalose K, York D, Khan S, Tegally H, Wilkinson E, de Oliveira T
EPI_ISL_515801, EPI_ISL_515802, EPI_ISL_515803, EPI_ISL_515804, EPI_ISL_515805, EPI_ISL_515806, EPI_ISL_515807, EPI_ISL_515808, EPI_ISL_515809, EPI_ISL_515810, EPI_ISL_515811, EPI_ISL_515812, EPI_ISL_515813, EPI_ISL_515814, EPI_ISL_515815, EPI_ISL_515816, EPI_ISL_515817, EPI_ISL_515818, EPI_ISL_515819, EPI_ISL_515820, EPI_ISL_515821, EPI_ISL_515822, EPI_ISL_515823, EPI_ISL_515824, EPI_ISL_515825, EPI_ISL_515826, EPI_ISL_515827, EPI_ISL_515828, EPI_ISL_515829, EPI_ISL_515830, EPI_ISL_515831, EPI_ISL_515832, EPI_ISL_515833, EPI_ISL_515835, EPI_ISL_515836, EPI_ISL_515837, EPI_ISL_515838, EPI_ISL_515839, EPI_ISL_515840, EPI_ISL_515841, EPI_ISL_515842, EPI_ISL_515843, EPI_ISL_515844, EPI_ISL_515845, EPI_ISL_515846, EPI_ISL_515847, EPI_ISL_515848, EPI_ISL_515849, EPI_ISL_515850, EPI_ISL_515851, EPI_ISL_515852, EPI_ISL_515853, EPI_ISL_515854, EPI_ISL_515855, EPI_ISL_515856, EPI_ISL_515857, EPI_ISL_515858, EPI_ISL_515859, EPI_ISL_515860, EPI_ISL_515861, EPI_ISL_515862, EPI_ISL_515863, EPI_ISL_515864, EPI_ISL_515865, EPI_ISL_515866, EPI_ISL_515867, EPI_ISL_515868, EPI_ISL_515869, EPI_ISL_515870, EPI_ISL_515871, EPI_ISL_515872, EPI_ISL_515873, EPI_ISL_515874, EPI_ISL_515875, EPI_ISL_515876, EPI_ISL_515877, EPI_ISL_515878, EPI_ISL_515880, EPI_ISL_515881, EPI_ISL_515882, EPI_ISL_515883, EPI_ISL_515884, EPI_ISL_515885, EPI_ISL_515886, EPI_ISL_515887, EPI_ISL_515888, EPI_ISL_515889, EPI_ISL_515890, EPI_ISL_515891, EPI_ISL_515892, EPI_ISL_515893			

see above	Medical Disagnostics Services (MDS)	KRISP, KZN Research Innovation and Sequencing Platform	Giandhari J, Pillay S, Lessells R, ChimukangaraB, Mdlalose K, York D, Khan S, Tegally H, Wilkinson E, de Oliveira T
EPI_ISL_515968, EPI_ISL_515969, EPI_ISL_515970, EPI_ISL_515971, EPI_ISL_515972, EPI_ISL_515973	MIMS	Department of Neurovirology, National Institute of Mental Health and Neuroscience (NIMHANS)	Chitra Pattabiraman,Vijayalakshmi Reddy, Harsha PK, Risha Rasheed, Pramada Prasad, Shafeeq S Hameed, Manjunatha Venkataswamy, Anita Desai, Ravi Vasanthapuram
EPI_ISL_516609	Instituto de Diagnostico y Referencia Epidemiologicos (INDRE)	Instituto de Diagnostico y Referencia Epidemiologicos (INDRE)	Ernesto Ramirez-Gonzalez, Abril Rodriguez-Maldonado, Claudia Wong-Arambula , Natividad Cruz-Ortiz, Tatiana Nunez-Garcia, Dayanira Arellano-Suarez, Adnan Araiza-Rodriguez, Fabiola Garces-Ayala, Lucia Hernandez-Rivas, Irma Lopez-Martinez, Gisela Barrera-Badillo.
EPI_ISL_516611	Instituto de Diagnostico y Referencia Epidemiologicos (INDRE)	Instituto de Diagnostico y Referencia Epidemiologicos (INDRE)	Gisela Barrera-Badillo , Abril Rodriguez-Maldonado, Claudia Wong-Arambula , Natividad Cruz-Ortiz, Tatiana Nunez-Garcia, Dayanira Arellano-Suarez, Fabiola Garces-Ayala, Edgar Mendieta-Condado, Lucia Hernandez-Rivas, Irma Lopez-Martinez, Ernesto Ramirez-Gonzalez.
EPI_ISL_516613	Instituto de Diagnostico y Referencia Epidemiologicos (INDRE)	Instituto de Diagnostico y Referencia Epidemiologicos (INDRE)	Ernesto Ramirez-Gonzalez, Abril Rodriguez-Maldonado, Claudia Wong-Arambula , Natividad Cruz-Ortiz, Tatiana Nunez-Garcia, Dayanira Arellano-Suarez, Adnan Araiza-Rodriguez, Edgar Mendieta-Condado, Lucia Hernandez-Rivas, Irma Lopez-Martinez, Gisela Barrera-Badillo.
EPI_ISL_516618	Instituto de Diagnostico y Referencia Epidemiologicos (INDRE)	Instituto de Diagnostico y Referencia Epidemiologicos (INDRE)	Gisela Barrera-Badillo , Abril Rodriguez-Maldonado, Claudia Wong-Arambula , Natividad Cruz-Ortiz, Tatiana Nunez-Garcia, Dayanira Arellano-Suarez, Fabiola Garces-Ayala, Edgar Mendieta-Condado, Lucia Hernandez-Rivas, Irma Lopez-Martinez, Ernesto Ramirez-Gonzalez.
EPI_ISL_516620	Instituto de Diagnostico y Referencia Epidemiologicos (INDRE)	Instituto de Diagnostico y Referencia Epidemiologicos (INDRE)	Gisela Barrera-Badillo , Abril Rodriguez-Maldonado, Claudia Wong-Arambula , Natividad Cruz-Ortiz, Tatiana Nunez-Garcia, Dayanira Arellano-Suarez, Adnan Araiza-Rodriguez, Edgar Mendieta-Condado, Lucia Hernandez-Rivas, Irma Lopez-Martinez, Ernesto Ramirez-Gonzalez.
EPI_ISL_516624, EPI_ISL_516625	Instituto de Diagnostico y Referencia Epidemiologicos (INDRE)	Instituto de Diagnostico y Referencia Epidemiologicos (INDRE)	Gisela Barrera-Badillo , Abril Rodriguez-Maldonado, Claudia Wong-Arambula , Natividad Cruz-Ortiz, Tatiana Nunez-Garcia, Dayanira Arellano-Suarez, Fabiola Garces-Ayala, Edgar Mendieta-Condado, Lucia Hernandez-Rivas, Irma Lopez-Martinez, Ernesto Ramirez-Gonzalez.
EPI_ISL_516748, EPI_ISL_516749, EPI_ISL_516750, EPI_ISL_516752, EPI_ISL_516753, EPI_ISL_516755, EPI_ISL_516756, EPI_ISL_516757, EPI_ISL_516758, EPI_ISL_516762, EPI_ISL_516763, EPI_ISL_516764, EPI_ISL_516765, EPI_ISL_516766, EPI_ISL_516767, EPI_ISL_516768, EPI_ISL_516770, EPI_ISL_516771, EPI_ISL_516772, EPI_ISL_516773, EPI_ISL_516774, EPI_ISL_516775, EPI_ISL_516776, EPI_ISL_516777, EPI_ISL_516778, EPI_ISL_516779			
see above	van Bakel Laboratory, Genetics and Genomics Sciences, Icahn School of Medicine at Mount Sinai	van Bakel Laboratory, Genetics and Genomics Sciences, Icahn School of Medicine at Mount Sinai	Andrew G. Letizia, Irene Ramos, Ajay Obla, Carl Goforth, Dawn Weir, Yongchao Ge, Marcas M. Bamman, Jayeeta Dutta, Ethan Ellis, Luis Estrella, Mary-Catherine George, Ana S. Gonzalez-Reiche, Darnell Graham, Adriana van de Guchte, Ramiro Gutierrez, Franca Jones, Aspasia Kalomoiri, Rhonda Lizewski, Stephen Lizewski, Jan Marayag, Nada Marjanovic, Eugene V. Millar, Venugopalan Nair, German Nudelman, Edgar Nunez, Brian Pike, James Regeimbal, Stas Rirak , Ernesto Santa Ana, Rachel S. Gelernter Sealfon, Robert Sebra, Mark Simons, Alessandra Soares-Schanoski, Michael Termini, Sindhu Vangeti, Carlos Williams, Harm van Bakel, Stuart C. Sealfon
EPI_ISL_516800	Rumah Sakit Akademik Universitas Gadjah Mada	Genetics Working Group (Pokja Genetik) Faculty of Medicine, Public Health and Nursing Universitas Gadjah Mada (FK-KMK UGM); Disease Investigation Center Wates Ministry of Agriculture Indonesia; Department of Microbiology FK-KMK UGM; Laboratorium Diagnostik Yayasan Tahija World Mosquito Program (WMP) Yogyakarta Center for Tropical Medicine FK-KMK UGM; Integrated Research center FK-KMK UGM; Department of Computer Science and Electronics FMIPA UGM	Gunadi, Hendra Wibawa, . Marcellus, Mohamad S. Hakim, Edwin W. Daniwijaya, Ludhang P. Rizki, Endah Supriyati, Eggi Arguni, Titik Nuryastuti, Tri Wibawa, Dwi AA Nugrahaningsih, Afiahayati, Siswanto, Alvin S. Kalim, Desyifa Mursalin
EPI_ISL_516806	Rumah Sakit PKU Gamping	Genetics Working Group (Pokja Genetik) Faculty of Medicine, Public Health and Nursing Universitas Gadjah Mada (FK-KMK UGM); Disease Investigation Center Wates Ministry of Agriculture Indonesia; Department of Microbiology FK-KMK UGM; Laboratorium Diagnostik Yayasan Tahija World Mosquito Program (WMP) Yogyakarta Center for Tropical Medicine FK-KMK UGM; Integrated Research center FK-KMK UGM; Department of Computer Science and Electronics FMIPA UGM	Gunadi, Hendra Wibawa, . Marcellus, Mohamad S. Hakim, Edwin W. Daniwijaya, Ludhang P. Rizki, Endah Supriyati, Eggi Arguni, Titik Nuryastuti, Tri Wibawa, Dwi AA Nugrahaningsih, . Afiahayati , . Siswanto, Ardorisye Saptaty Fornia, Kemala Athollah
EPI_ISL_516829	RSUD Nyi Ageng Serang	Genetics Working Group (Pokja Genetik) Faculty of Medicine, Public Health and Nursing Universitas Gadjah Mada (FK-KMK UGM), Disease Investigation Center Wates Ministry of Agriculture Indonesia, Department of Microbiology FK-KMK UGM, Laboratorium Diagnostik Yayasan Tahija World Mosquito Program (WMP) Yogyakarta Center for Tropical Medicine FK-KMK UGM, Integrated Research Center FK-KMK UGM, Department of Computer Science and Electronics FMIPA UGM	Gunadi, Hendra Wibawa, . Marcellus, Mohamad S. Hakim, Edwin W. Daniwijaya, Ludhang P. Rizki, Endah Supriyati, Eggi Arguni, Titik Nuryastuti, Tri Wibawa, Dwi AA Nugrahaningsih, Afiahayati , . Siswanto, Beby Dewi Sartika, Dyah Ayu Puspitarani
EPI_ISL_516934, EPI_ISL_516935, EPI_ISL_516938	Nicolae Testemitanu State University of Medicine and Pharmacy	International Centre for Genetic Engineering and Biotechnology (ICGEB) and ARGO Open Lab Platform for Genome Sequencing	Ulinici M, Licastro D, Dal Monego S, Rajasekharan S, Marcello A
EPI_ISL_516976, EPI_ISL_516977, EPI_ISL_516980, EPI_ISL_516981, EPI_ISL_516982, EPI_ISL_516983, EPI_ISL_516984, EPI_ISL_516986	King Georges Medical University	CSIR-National Botanical Research Institute	Priti Prasad, Shantanu Prakash, Kishan Sahu, Babita Singh, Suruchi Shukla, Hricha Mishra, Danish Nasar Khan , Om Prakash, MLB Bhatt, SK Barik, Mehar H.Asif,Samir V. Sawant,Amita Jain, Sumit Kr. Bag
EPI_ISL_517996, EPI_ISL_517997, EPI_ISL_517998, EPI_ISL_517999, EPI_ISL_518001, EPI_ISL_518002, EPI_ISL_518003, EPI_ISL_518004, EPI_ISL_518005, EPI_ISL_518006, EPI_ISL_518007, EPI_ISL_518008, EPI_ISL_518009, EPI_ISL_518010, EPI_ISL_518011, EPI_ISL_518013, EPI_ISL_518014, EPI_ISL_518015, EPI_ISL_518016			
see above	Singapore General Hospital	Department of Microbiology	Nurdyana Abdul Rahman, Kun Lee Lim, Chenhao Li, Kian Sing Chan, Lynette Oon, Kern Rei Chng, Niranjan Nagarajan, Karrie Ko
EPI_ISL_518034, EPI_ISL_518035, EPI_ISL_518037, EPI_ISL_518038, EPI_ISL_518039, EPI_ISL_518040, EPI_ISL_518041, EPI_ISL_518042, EPI_ISL_518043, EPI_ISL_518044, EPI_ISL_518045, EPI_ISL_518046, EPI_ISL_518047, EPI_ISL_518048, EPI_ISL_518049, EPI_ISL_518050, EPI_ISL_518051, EPI_ISL_518052			
see above	NHLS-IALCH	KRISP, KZN Research Innovation and Sequencing Platform	Giandhari J, Pillay S, Lessells R, Mdlalose K, York D, Khan S, Tegally H, Wilkinson E, de Oliveira T
EPI_ISL_518819	Qadr Hospital, Tangerang, Banten	Biosafety Level-3 Laboratory, Indonesian Institute of Sciences (LIPI)	Anik Budhi Dharmayanthi, Syam Budi Iryanto, Andri Wardiana, Anggia Prasetyoputri, Isa Nuryana, Ade Andriani, Ario Betha Juanssilfero, Asep M Ridwanuloh, Ahmad Fathoni, Rifki Sadikin, Ratih Asmana Ningrum, Wien Kusharyoto, Puspita Lisdiyanti
EPI_ISL_522350	KU Leuven, Rega Institute, Clinical and Epidemiological Virology	KU Leuven, Rega Institute, Clinical and Epidemiological Virology	Tony Wawina-Bokalanga, Joan Marti-Carerras, Bert Vanmechelen, Piet Maes
EPI_ISL_522874, EPI_ISL_522877	Instituto Nacional de Medicina Genomica	Instituto Nacional de Medicina Genomica	Hidalgo-Miranda A, Mendoza-Vargas A, Reyes-Grajeda JP, Cisneros-Villanueva M, Cedro-Tanda A, Hurtado-Cordova E, Peñaloza-Figueroa F, Herrera-Montalvo LA
EPI_ISL_522878	Instituto Nacional de Medicina Genomica	Instituto Nacional de Medicina Genomica	Hidalgo-Miranda A, Mendoza-Vargas A, Reyes-Grajeda JP, Cisneros-Villanueva M, Hurtado-Cordova E, Cedro-Tanda A, Peñaloza-Figueroa F, Herrera-Montalvo LA
EPI_ISL_522880, EPI_ISL_522940, EPI_ISL_522941, EPI_ISL_522942, EPI_ISL_522978, EPI_ISL_522980, EPI_ISL_522981, EPI_ISL_522982, EPI_ISL_522984, EPI_ISL_522986, EPI_ISL_523500			
see above	Instituto Nacional de Medicina Genómica	Instituto Nacional de Medicina Genómica	Hidalgo-Miranda A, Mendoza-Vargas A, Reyes-Grajeda JP, Cisneros-Villanueva M, Cedro-Tanda A, Hurtado-Cordova E, Peñaloza-Figueroa F, Herrera-Montalvo LA
EPI_ISL_523824	Communicable Disease Branch	Hong Kong Department of Health	Mak Gannon C.K., Lam Edman T.K., Chan Rickjason C.W., Tsang Dominic N.C.

EPI_ISL_523825	United Christian Hospital	Hong Kong Department of Health	Mak Gannon C.K., Lam Edman T.K., Chan Rickjason C.W., Tsang Dominic N.C.
EPI_ISL_523826, EPI_ISL_523827	Queen Mary Hospital	Hong Kong Department of Health	Mak Gannon C.K., Lam Edman T.K., Chan Rickjason C.W., Tsang Dominic N.C.
EPI_ISL_523828, EPI_ISL_523829	Tuen Mun Hospital	Hong Kong Department of Health	Mak Gannon C.K., Lam Edman T.K., Chan Rickjason C.W., Tsang Dominic N.C.
EPI_ISL_523830, EPI_ISL_523831	Our Lady of Maryknoll Hospital	Hong Kong Department of Health	Mak Gannon C.K., Lam Edman T.K., Chan Rickjason C.W., Tsang Dominic N.C.
EPI_ISL_523832, EPI_ISL_523834	Tuen Mun Hospital	Hong Kong Department of Health	Mak Gannon C.K., Lam Edman T.K., Chan Rickjason C.W., Tsang Dominic N.C.
EPI_ISL_523835, EPI_ISL_523836	Queen Elizabeth Hospital	Hong Kong Department of Health	Mak Gannon C.K., Lam Edman T.K., Chan Rickjason C.W., Tsang Dominic N.C.
EPI_ISL_523837	Yan Chai Hospital	Hong Kong Department of Health	Mak Gannon C.K., Lam Edman T.K., Chan Rickjason C.W., Tsang Dominic N.C.
EPI_ISL_523838	Princess Margaret Hospital	Hong Kong Department of Health	Mak Gannon C.K., Lam Edman T.K., Chan Rickjason C.W., Tsang Dominic N.C.
EPI_ISL_523839	Tseung Kwan O Hospital	Hong Kong Department of Health	Mak Gannon C.K., Lam Edman T.K., Chan Rickjason C.W., Tsang Dominic N.C.
EPI_ISL_523840	Our Lady of Maryknoll Hospital	Hong Kong Department of Health	Mak Gannon C.K., Lam Edman T.K., Chan Rickjason C.W., Tsang Dominic N.C.
EPI_ISL_523841	Queen Elizabeth Hospital	Hong Kong Department of Health	Mak Gannon C.K., Lam Edman T.K., Chan Rickjason C.W., Tsang Dominic N.C.
EPI_ISL_523842	Kwong Wah Hospital	Hong Kong Department of Health	Mak Gannon C.K., Lam Edman T.K., Chan Rickjason C.W., Tsang Dominic N.C.
EPI_ISL_523843, EPI_ISL_523844, EPI_ISL_523845, EPI_ISL_523846, EPI_ISL_523847	Queen Elizabeth Hospital	Hong Kong Department of Health	Mak Gannon C.K., Lam Edman T.K., Chan Rickjason C.W., Tsang Dominic N.C.
EPI_ISL_523848, EPI_ISL_523849	United Christian Hospital	Hong Kong Department of Health	Mak Gannon C.K., Lam Edman T.K., Chan Rickjason C.W., Tsang Dominic N.C.
EPI_ISL_523850, EPI_ISL_523851, EPI_ISL_523852	Tseung Kwan O Hospital	Hong Kong Department of Health	Mak Gannon C.K., Lam Edman T.K., Chan Rickjason C.W., Tsang Dominic N.C.
EPI_ISL_523853	Hong Kong Baptist Hospital	Hong Kong Department of Health	Mak Gannon C.K., Lam Edman T.K., Chan Rickjason C.W., Tsang Dominic N.C.
EPI_ISL_523854	Alice Ho Miu Ling Nethersole Hospital	Hong Kong Department of Health	Mak Gannon C.K., Lam Edman T.K., Chan Rickjason C.W., Tsang Dominic N.C.
EPI_ISL_523855	Kwong Wah Hospital	Hong Kong Department of Health	Mak Gannon C.K., Lam Edman T.K., Chan Rickjason C.W., Tsang Dominic N.C.
EPI_ISL_523959	Pronto Socorro Municipal de Perus	Instituto Adolfo Lutz, Interdiciplinary Procedures Center, Strategic Laboratory	Claudio Tavares Sacchi, Claudia Regina Gonçalves, Erica Valessa Ramos Gomes
EPI_ISL_524445, EPI_ISL_524447, EPI_ISL_524448, EPI_ISL_524449	Singapore General Hospital	Department of Microbiology	Nurdyana Abdul Rahman, Kun Lee Lim, Chenhao Li, Kian Sing Chan, Lynette Oon, Kern Rei Chng, Niranjan Nagarajan, Karrie Ko
EPI_ISL_524713	B.J. Medical College and Civil hospital, Ahmedabad	Gujarat Biotechnology Research Centre	Apurvasinh Puvar, Janvi Raval, Zarna Patel, Monika Gandhi, Pinal Trivedi, Maharshi Pandya, Nidhi Patel, Nitin Savaliya, Raghawendra Kumar, Dinesh Kumar, Zuber Saiyed, Komal Patel, Labdhi Pandya, Afzal Ansari, Nikha Trivedi, Pranay Shah, Kamlesh J Upadhyay, Sanjay Kapadia, R D Dixit, A M Kadri, Harsh Bakshi, Chaitanya Joshi, Madhvi Joshi
EPI_ISL_524714	B.J. Medical College and Civil hospital, Ahmedabad	Gujarat Biotechnology Research Centre	Janvi Raval, Zarna Patel, Monika Gandhi, Pinal Trivedi, Maharshi Pandya, Nidhi Patel, Nitin Savaliya, Raghawendra Kumar, Dinesh Kumar, Zuber Saiyed, Komal Patel, Labdhi Pandya, Afzal Ansari, Nikha Trivedi, Pranay Shah, Kamlesh J Upadhyay, Sanjay Kapadia, Apurvasinh Puvar, R D Dixit, A M Kadri, Harsh Bakshi, Chaitanya Joshi, Madhvi Joshi
EPI_ISL_524715	B.J. Medical College and Civil hospital, Ahmedabad	Gujarat Biotechnology Research Centre	Zarna Patel, Monika Gandhi, Pinal Trivedi, Maharshi Pandya, Nidhi Patel, Nitin Savaliya, Raghawendra Kumar, Dinesh Kumar, Zuber Saiyed, Komal Patel, Labdhi Pandya, Afzal Ansari, Nikha Trivedi, Pranay Shah, Kamlesh J Upadhyay, Sanjay Kapadia, Apurvasinh Puvar, Janvi Raval, R D Dixit, A M Kadri, Harsh Bakshi, Chaitanya Joshi, Madhvi Joshi
EPI_ISL_524716	B.J. Medical College and Civil hospital, Ahmedabad	Gujarat Biotechnology Research Centre	Monika Gandhi, Pinal Trivedi, Maharshi Pandya, Nidhi Patel, Nitin Savaliya, Raghawendra Kumar, Dinesh Kumar, Zuber Saiyed, Komal Patel, Labdhi Pandya, Afzal Ansari, Nikha Trivedi, Pranay Shah, Kamlesh J Upadhyay, Sanjay Kapadia, Apurvasinh Puvar, Janvi Raval, Zarna Patel, R D Dixit, A M Kadri, Harsh Bakshi, Chaitanya Joshi, Madhvi Joshi
EPI_ISL_524717	B.J. Medical College and Civil hospital, Ahmedabad	Gujarat Biotechnology Research Centre	Pinal Trivedi, Maharshi Pandya, Nidhi Patel, Nitin Savaliya, Raghawendra Kumar, Dinesh Kumar, Zuber Saiyed, Komal Patel, Labdhi Pandya, Afzal Ansari, Nikha Trivedi, Pranay Shah, Kamlesh J Upadhyay, Sanjay Kapadia, Apurvasinh Puvar, Janvi Raval, Zarna Patel, Monika Gandhi, R D Dixit, A M Kadri, Harsh Bakshi, Chaitanya Joshi, Madhvi Joshi
EPI_ISL_524718	B.J. Medical College and Civil hospital, Ahmedabad	Gujarat Biotechnology Research Centre	Maharshi Pandya, Nidhi Patel, Nitin Savaliya, Raghawendra Kumar, Dinesh Kumar, Zuber Saiyed, Komal Patel, Labdhi Pandya, Afzal Ansari, Nikha Trivedi, Pranay Shah, Kamlesh J Upadhyay, Sanjay Kapadia, Apurvasinh Puvar, Janvi Raval, Zarna Patel, Monika Gandhi, Pinal Trivedi, R D Dixit, A M Kadri, Harsh Bakshi, Chaitanya Joshi, Madhvi Joshi
EPI_ISL_524719	B.J. Medical College and Civil hospital, Ahmedabad	Gujarat Biotechnology Research Centre	Nidhi Patel, Nitin Savaliya, Raghawendra Kumar, Dinesh Kumar, Zuber Saiyed, Komal Patel, Labdhi Pandya, Afzal Ansari, Nikha Trivedi, Pranay Shah, Kamlesh J Upadhyay, Sanjay Kapadia, Apurvasinh Puvar, Janvi Raval, Zarna Patel, Monika Gandhi, Pinal Trivedi, Maharshi Pandya, R D Dixit, A M Kadri, Harsh Bakshi, Chaitanya Joshi, Madhvi Joshi
EPI_ISL_524720	B.J. Medical College and Civil hospital, Ahmedabad	Gujarat Biotechnology Research Centre	Nitin Savaliya, Raghawendra Kumar, Dinesh Kumar, Zuber Saiyed, Komal Patel, Labdhi Pandya, Afzal Ansari, Nikha Trivedi, Pranay Shah, Kamlesh J Upadhyay, Sanjay Kapadia, Apurvasinh Puvar, Janvi Raval, Zarna Patel, Monika Gandhi, Pinal Trivedi, Maharshi Pandya, Nidhi Patel, R D Dixit, A M Kadri, Harsh Bakshi, Chaitanya Joshi, Madhvi Joshi
EPI_ISL_524721	B.J. Medical College and Civil hospital, Ahmedabad	Gujarat Biotechnology Research Centre	Raghawendra Kumar, Dinesh Kumar, Zuber Saiyed, Komal Patel, Labdhi Pandya, Afzal Ansari, Nikha Trivedi, Pranay Shah, Kamlesh J Upadhyay, Sanjay Kapadia, Apurvasinh Puvar, Janvi Raval, Zarna Patel, Monika Gandhi, Pinal Trivedi, Maharshi Pandya, Nidhi Patel, Nitin Savaliya, R D Dixit, A M Kadri, Harsh Bakshi, Chaitanya Joshi, Madhvi Joshi
EPI_ISL_524722	B.J. Medical College and Civil hospital, Ahmedabad	Gujarat Biotechnology Research Centre	Dinesh Kumar, Zuber Saiyed, Komal Patel, Labdhi Pandya, Afzal Ansari, Nikha Trivedi, Pranay Shah, Kamlesh J Upadhyay, Sanjay Kapadia, Apurvasinh Puvar, Janvi Raval, Zarna Patel, Monika Gandhi, Pinal Trivedi, Maharshi Pandya, Nidhi Patel, Nitin Savaliya, Raghawendra Kumar, R D Dixit, A M Kadri, Harsh Bakshi, Chaitanya Joshi, Madhvi Joshi
EPI_ISL_524723	B.J. Medical College and Civil hospital, Ahmedabad	Gujarat Biotechnology Research Centre	Zuber Saiyed, Komal Patel, Labdhi Pandya, Afzal Ansari, Nikha Trivedi, Pranay Shah, Kamlesh J Upadhyay, Sanjay Kapadia, Apurvasinh Puvar, Janvi Raval, Zarna Patel, Monika Gandhi, Pinal Trivedi, Maharshi Pandya, Nidhi Patel, Nitin Savaliya, Raghawendra Kumar, Dinesh Kumar, R D Dixit, A M Kadri, Harsh Bakshi, Chaitanya Joshi, Madhvi Joshi
EPI_ISL_524724	B.J. Medical College and Civil hospital, Ahmedabad	Gujarat Biotechnology Research Centre	Komal Patel, Labdhi Pandya, Afzal Ansari, Nikha Trivedi, Pranay Shah, Kamlesh J Upadhyay, Sanjay Kapadia, Apurvasinh Puvar, Janvi Raval, Zarna Patel, Monika Gandhi, Pinal Trivedi, Maharshi Pandya, Nidhi Patel, Nitin Savaliya, Raghawendra Kumar, Dinesh Kumar, Zuber Saiyed, R D Dixit, A M Kadri, Harsh Bakshi, Chaitanya Joshi, Madhvi Joshi
EPI_ISL_524725	B.J. Medical College and Civil hospital, Ahmedabad	Gujarat Biotechnology Research Centre	Labdhi Pandya, Afzal Ansari, Nikha Trivedi, Pranay Shah, Kamlesh J Upadhyay, Sanjay Kapadia, Apurvasinh Puvar, Janvi Raval, Zarna Patel, Monika Gandhi, Pinal Trivedi, Maharshi Pandya, Nidhi Patel, Nitin Savaliya, Raghawendra Kumar, Dinesh Kumar, Zuber Saiyed, Komal Patel, R D Dixit, A M Kadri, Harsh Bakshi, Chaitanya Joshi, Madhvi Joshi
EPI_ISL_524726	B.J. Medical College and Civil hospital, Ahmedabad	Gujarat Biotechnology Research Centre	Afzal Ansari, Nikha Trivedi, Pranay Shah, Kamlesh J Upadhyay, Sanjay Kapadia, Apurvasinh Puvar, Janvi Raval, Zarna Patel, Monika Gandhi, Pinal Trivedi, Maharshi Pandya, Nidhi Patel, Nitin Savaliya, Raghawendra Kumar, Dinesh Kumar, Zuber Saiyed, Komal Patel, Labdhi Pandya, R D Dixit, A M Kadri, Harsh Bakshi, Chaitanya Joshi, Madhvi Joshi
EPI_ISL_524727	B.J. Medical College and Civil hospital, Ahmedabad	Gujarat Biotechnology Research Centre	Nikha Trivedi, Pranay Shah, Kamlesh J Upadhyay, Sanjay Kapadia, Apurvasinh Puvar, Janvi Raval, Zarna Patel, Monika Gandhi, Pinal Trivedi, Maharshi Pandya, Nidhi Patel, Nitin Savaliya, Raghawendra Kumar, Dinesh Kumar, Zuber Saiyed, Komal Patel, Labdhi Pandya, Afzal Ansari, R D Dixit, A M Kadri, Harsh Bakshi, Chaitanya Joshi, Madhvi Joshi

EPI_ISL_524728	B.J. Medical College and Civil hospital, Ahmedabad	Gujarat Biotechnology Research Centre	Pranay Shah, Kamlesh J Upadhyay, Sanjay Kapadia, Apurvasinh Puvar, Janvi Raval, Zarna Patel, Monika Gandhi, Pinal Trivedi, Maharshi Pandya, Nidhi Patel, Nitin Savaliya, Raghawendra Kumar, Dinesh Kumar, Zuber Saiyed, Komal Patel, Labdhi Pandya, Afzal Ansari, Nikha Trivedi, R D Dixit, A M Kadri, Harsh Bakshi, Chaitanya Joshi, Madhvi Joshi
EPI_ISL_524729	B.J. Medical College and Civil hospital, Ahmedabad	Gujarat Biotechnology Research Centre	Kamlesh J Upadhyay, Sanjay Kapadia, Apurvasinh Puvar, Janvi Raval, Zarna Patel, Monika Gandhi, Pinal Trivedi, Maharshi Pandya, Nidhi Patel, Nitin Savaliya, Raghawendra Kumar, Dinesh Kumar, Zuber Saiyed, Komal Patel, Labdhi Pandya, Afzal Ansari, Nikha Trivedi, Pranay Shah, R D Dixit, A M Kadri, Harsh Bakshi, Chaitanya Joshi, Madhvi Joshi
EPI_ISL_524730	GMERS Medical College & Hospital, Gotri, Vadodara	Gujarat Biotechnology Research Centre	Meenakshi Shah, Neena Doshi, Varsha Godbole, Apurvasinh Puvar, Janvi Raval, Zarna Patel, Monika Gandhi, Pinal Trivedi, Maharshi Pandya, Nidhi Patel, Nitin Savaliya, Raghawendra Kumar, Dinesh Kumar, Zuber Saiyed, Komal Patel, Labdhi Pandya, Afzal Ansari, Nikha Trivedi, R D Dixit, A M Kadri, Harsh Bakshi, Chaitanya Joshi, Madhvi Joshi
EPI_ISL_524731	GMERS Medical College & Hospital, Gotri, Vadodara	Gujarat Biotechnology Research Centre	Neena Doshi, Varsha Godbole, Apurvasinh Puvar, Janvi Raval, Zarna Patel, Monika Gandhi, Pinal Trivedi, Maharshi Pandya, Nidhi Patel, Nitin Savaliya, Raghawendra Kumar, Dinesh Kumar, Zuber Saiyed, Komal Patel, Labdhi Pandya, Afzal Ansari, Nikha Trivedi, Meenakshi Shah, R D Dixit, A M Kadri, Harsh Bakshi, Chaitanya Joshi, Madhvi Joshi
EPI_ISL_524732	Dr. RSS Hospital, Modasa	Gujarat Biotechnology Research Centre	Harsh Chaudhari, Apurvasinh Puvar, Janvi Raval, Zarna Patel, Monika Gandhi, Pinal Trivedi, Maharshi Pandya, Nidhi Patel, Nitin Savaliya, Raghawendra Kumar, Dinesh Kumar, Zuber Saiyed, Komal Patel, Labdhi Pandya, Afzal Ansari, Nikha Trivedi, R D Dixit, A M Kadri, Harsh Bakshi, Chaitanya Joshi, Madhvi Joshi, Neena Doshi, Varsha Godbole
EPI_ISL_524733	GMERS Medical College and Hospital, Dharpur, Patan	Gujarat Biotechnology Research Centre	A N Parmar, Apurvasinh Puvar, Janvi Raval, Zarna Patel, Monika Gandhi, Pinal Trivedi, Maharshi Pandya, Nidhi Patel, Nitin Savaliya, Raghawendra Kumar, Dinesh Kumar, Zuber Saiyed, Komal Patel, Labdhi Pandya, Afzal Ansari, Nikha Trivedi, R D Dixit, A M Kadri, Harsh Bakshi, Chaitanya Joshi, Madhvi Joshi
EPI_ISL_524734	GMERS Medical College and Hospital, Dharpur, Patan	Gujarat Biotechnology Research Centre	Apurvasinh Puvar, Janvi Raval, Zarna Patel, Monika Gandhi, Pinal Trivedi, Maharshi Pandya, Nidhi Patel, Nitin Savaliya, Raghawendra Kumar, Dinesh Kumar, Zuber Saiyed, Komal Patel, Labdhi Pandya, Afzal Ansari, Nikha Trivedi, A N Parmar, R D Dixit, A M Kadri, Harsh Bakshi, Chaitanya Joshi, Madhvi Joshi
EPI_ISL_524735	Government General Hospital, Jam Khambhaliya, Devbhoomi Dwarka	Gujarat Biotechnology Research Centre	Harish Matani, Janvi Raval, Zarna Patel, Monika Gandhi, Pinal Trivedi, Maharshi Pandya, Nidhi Patel, Nitin Savaliya, Raghawendra Kumar, Dinesh Kumar, Zuber Saiyed, Komal Patel, Labdhi Pandya, Afzal Ansari, Nikha Trivedi, Apurvasinh Puvar, R D Dixit, A M Kadri, Harsh Bakshi, Chaitanya Joshi, Madhvi Joshi
EPI_ISL_524736	Government General Hospital, Jam Khambhaliya, Devbhoomi Dwarka	Gujarat Biotechnology Research Centre	Janvi Raval, Zarna Patel, Monika Gandhi, Pinal Trivedi, Maharshi Pandya, Nidhi Patel, Nitin Savaliya, Raghawendra Kumar, Dinesh Kumar, Zuber Saiyed, Komal Patel, Labdhi Pandya, Afzal Ansari, Nikha Trivedi, Harish Matani, Apurvasinh Puvar, R D Dixit, A M Kadri, Harsh Bakshi, Chaitanya Joshi, Madhvi Joshi
EPI_ISL_524737	GMERS Medical College and Hospital, Gandhinagar	Gujarat Biotechnology Research Centre	Zarna Patel, Monika Gandhi, Pinal Trivedi, Maharshi Pandya, Nidhi Patel, Nitin Savaliya, Raghawendra Kumar, Dinesh Kumar, Zuber Saiyed, Komal Patel, Labdhi Pandya, Afzal Ansari, Nikha Trivedi, Seema Bhatt, Gaurishankar Shrimali, Bhavesh Modi, Bharti Rajani, Apurvasinh Puvar, Janvi Raval, R D Dixit, A M Kadri, Harsh Bakshi, Chaitanya Joshi, Madhvi Joshi
EPI_ISL_524738	GMERS Medical College and Hospital, Gandhinagar	Gujarat Biotechnology Research Centre	Monika Gandhi, Pinal Trivedi, Maharshi Pandya, Nidhi Patel, Nitin Savaliya, Raghawendra Kumar, Dinesh Kumar, Zuber Saiyed, Komal Patel, Labdhi Pandya, Afzal Ansari, Nikha Trivedi, Seema Bhatt, Gaurishankar Shrimali, Bhavesh Modi, Bharti Rajani, Apurvasinh Puvar, Janvi Raval, Zarna Patel, R D Dixit, A M Kadri, Harsh Bakshi, Chaitanya Joshi, Madhvi Joshi
EPI_ISL_524739	GMERS Medical College and Hospital, Gandhinagar	Gujarat Biotechnology Research Centre	Pinal Trivedi, Maharshi Pandya, Nidhi Patel, Nitin Savaliya, Raghawendra Kumar, Dinesh Kumar, Zuber Saiyed, Komal Patel, Labdhi Pandya, Afzal Ansari, Nikha Trivedi, Seema Bhatt, Gaurishankar Shrimali, Bhavesh Modi, Bharti Rajani, Apurvasinh Puvar, Janvi Raval, Zarna Patel, Monika Gandhi, R D Dixit, A M Kadri, Harsh Bakshi, Chaitanya Joshi, Madhvi Joshi
EPI_ISL_524740	GMERS Medical College and Hospital, Gandhinagar	Gujarat Biotechnology Research Centre	Maharshi Pandya, Nidhi Patel, Nitin Savaliya, Raghawendra Kumar, Dinesh Kumar, Zuber Saiyed, Komal Patel, Labdhi Pandya, Afzal Ansari, Nikha Trivedi, Seema Bhatt, Gaurishankar Shrimali, Bhavesh Modi, Bharti Rajani, Apurvasinh Puvar, Janvi Raval, Zarna Patel, Monika Gandhi, Pinal Trivedi, R D Dixit, A M Kadri, Harsh Bakshi, Chaitanya Joshi, Madhvi Joshi
EPI_ISL_524741	GMERS Medical College and Hospital, Gandhinagar	Gujarat Biotechnology Research Centre	Nidhi Patel, Nitin Savaliya, Raghawendra Kumar, Dinesh Kumar, Zuber Saiyed, Komal Patel, Labdhi Pandya, Afzal Ansari, Nikha Trivedi, Seema Bhatt, Gaurishankar Shrimali, Bhavesh Modi, Bharti Rajani, Apurvasinh Puvar, Janvi Raval, Zarna Patel, Monika Gandhi, Pinal Trivedi, Maharshi Pandya, R D Dixit, A M Kadri, Harsh Bakshi, Chaitanya Joshi, Madhvi Joshi
EPI_ISL_524742	GMERS Medical College and Hospital, Gandhinagar	Gujarat Biotechnology Research Centre	Dinesh Kumar, Zuber Saiyed, Komal Patel, Labdhi Pandya, Afzal Ansari, Nikha Trivedi, Seema Bhatt, Gaurishankar Shrimali, Bhavesh Modi, Bharti Rajani, Apurvasinh Puvar, Janvi Raval, Zarna Patel, Monika Gandhi, Pinal Trivedi, Maharshi Pandya, Nidhi Patel, Nitin Savaliya, Raghawendra Kumar, R D Dixit, A M Kadri, Harsh Bakshi, Chaitanya Joshi, Madhvi Joshi
EPI_ISL_524743	GMERS Medical College and Hospital, Gandhinagar	Gujarat Biotechnology Research Centre	Zuber Saiyed, Komal Patel, Labdhi Pandya, Afzal Ansari, Nikha Trivedi, Seema Bhatt, Gaurishankar Shrimali, Bhavesh Modi, Bharti Rajani, Apurvasinh Puvar, Janvi Raval, Zarna Patel, Monika Gandhi, Pinal Trivedi, Maharshi Pandya, Nidhi Patel, Nitin Savaliya, Raghawendra Kumar, Dinesh Kumar, R D Dixit, A M Kadri, Harsh Bakshi, Chaitanya Joshi, Madhvi Joshi
EPI_ISL_524744	GMERS Medical College and Hospital, Gandhinagar	Gujarat Biotechnology Research Centre	Komal Patel, Labdhi Pandya, Afzal Ansari, Nikha Trivedi, Seema Bhatt, Gaurishankar Shrimali, Bhavesh Modi, Bharti Rajani, Apurvasinh Puvar, Janvi Raval, Zarna Patel, Monika Gandhi, Pinal Trivedi, Maharshi Pandya, Nidhi Patel, Nitin Savaliya, Raghawendra Kumar, Dinesh Kumar, Zuber Saiyed, R D Dixit, A M Kadri, Harsh Bakshi, Chaitanya Joshi, Madhvi Joshi
EPI_ISL_524745	GMERS Medical College and Hospital, Gandhinagar	Gujarat Biotechnology Research Centre	Labdhi Pandya, Afzal Ansari, Nikha Trivedi, Seema Bhatt, Gaurishankar Shrimali, Bhavesh Modi, Bharti Rajani, Apurvasinh Puvar, Janvi Raval, Zarna Patel, Monika Gandhi, Pinal Trivedi, Maharshi Pandya, Nidhi Patel, Nitin Savaliya, Raghawendra Kumar, Dinesh Kumar, Zuber Saiyed, Komal Patel, R D Dixit, A M Kadri, Harsh Bakshi, Chaitanya Joshi, Madhvi Joshi
EPI_ISL_524746	Sir M P Shah Government Medical College, Jamnagar	Gujarat Biotechnology Research Centre	Binita Aring, Afzal Ansari, Nikha Trivedi, Apurvasinh Puvar, Janvi Raval, Zarna Patel, Monika Gandhi, Pinal Trivedi, Maharshi Pandya, Nidhi Patel, Nitin Savaliya, Raghawendra Kumar, Dinesh Kumar, Zuber Saiyed, Komal Patel, Labdhi Pandya, R D Dixit, A M Kadri, Harsh Bakshi, Chaitanya Joshi, Madhvi Joshi
EPI_ISL_524747	Sir M P Shah Government Medical College, Jamnagar	Gujarat Biotechnology Research Centre	Afzal Ansari, Nikha Trivedi, Binita Aring, Apurvasinh Puvar, Janvi Raval, Zarna Patel, Monika Gandhi, Pinal Trivedi, Maharshi Pandya, Nidhi Patel, Nitin Savaliya, Raghawendra Kumar, Dinesh Kumar, Zuber Saiyed, Komal Patel, Labdhi Pandya, R D Dixit, A M Kadri, Harsh Bakshi, Chaitanya Joshi, Madhvi Joshi
EPI_ISL_524748	Sir M P Shah Government Medical College, Jamnagar	Gujarat Biotechnology Research Centre	Nikha Trivedi, Apurvasinh Puvar, Janvi Raval, Binita Aring, Zarna Patel, Monika Gandhi, Pinal Trivedi, Maharshi Pandya, Nidhi Patel, Nitin Savaliya, Raghawendra Kumar, Dinesh Kumar, Zuber Saiyed, Komal Patel, Labdhi Pandya, Afzal Ansari, R D Dixit, A M Kadri, Harsh Bakshi, Chaitanya Joshi, Madhvi Joshi
EPI_ISL_524749	Sir M P Shah Government Medical College, Jamnagar	Gujarat Biotechnology Research Centre	Apurvasinh Puvar, Janvi Raval, Zarna Patel, Monika Gandhi, Pinal Trivedi, Maharshi Pandya, Nidhi Patel, Nitin Savaliya, Raghawendra Kumar, Dinesh Kumar, Zuber Saiyed, Komal Patel, Labdhi Pandya, Afzal Ansari, Nikha Trivedi, Binita Aring, R D Dixit, A M Kadri, Harsh Bakshi, Chaitanya Joshi, Madhvi Joshi
EPI_ISL_524750	Sir M P Shah Government Medical College, Jamnagar	Gujarat Biotechnology Research Centre	Janvi Raval, Zarna Patel, Monika Gandhi, Pinal Trivedi, Maharshi Pandya, Nidhi Patel, Nitin Savaliya, Raghawendra Kumar, Dinesh Kumar, Zuber Saiyed, Komal Patel, Labdhi Pandya, Afzal Ansari, Nikha Trivedi, Binita Aring, Apurvasinh Puvar, R D Dixit, A M Kadri, Harsh Bakshi, Chaitanya Joshi, Madhvi Joshi
EPI_ISL_524751	Sir M P Shah Government Medical College, Jamnagar	Gujarat Biotechnology Research Centre	Zarna Patel, Monika Gandhi, Pinal Trivedi, Maharshi Pandya, Nidhi Patel, Nitin Savaliya, Raghawendra Kumar, Dinesh Kumar, Zuber Saiyed, Komal Patel, Labdhi Pandya, Afzal Ansari, Nikha Trivedi, Binita Aring, Apurvasinh Puvar, Janvi Raval, R D Dixit, A M Kadri, Harsh Bakshi, Chaitanya Joshi, Madhvi Joshi
EPI_ISL_524752	GMERS Medical College and Hospital, Dharpur, Patan	Gujarat Biotechnology Research Centre	Monika Gandhi, Pinal Trivedi, Maharshi Pandya, Nidhi Patel, Nitin Savaliya, Raghawendra Kumar, Dinesh Kumar, Zuber Saiyed, Komal Patel, Labdhi Pandya, Afzal Ansari, Nikha Trivedi, A N Parmar, Apurvasinh Puvar, Janvi Raval, Zarna Patel, R D Dixit, A M Kadri, Harsh Bakshi, Chaitanya Joshi, Madhvi Joshi
EPI_ISL_524753	GMERS Medical College and Hospital, Dharpur, Patan	Gujarat Biotechnology Research Centre	Pinal Trivedi, Maharshi Pandya, Nidhi Patel, Nitin Savaliya, Raghawendra Kumar, Dinesh Kumar, Zuber Saiyed, Komal Patel, Labdhi Pandya, Afzal Ansari, Nikha Trivedi, A N Parmar, Apurvasinh Puvar, Janvi Raval, Zarna Patel, Monika Gandhi, R D Dixit, A M Kadri, Harsh Bakshi, Chaitanya Joshi, Madhvi Joshi
EPI_ISL_524754	GMERS Medical College and Hospital, Dharpur, Patan	Gujarat Biotechnology Research Centre	Maharshi Pandya, Nidhi Patel, Nitin Savaliya, Raghawendra Kumar, Dinesh Kumar, Zuber Saiyed, Komal Patel, Labdhi Pandya, Afzal Ansari, Nikha Trivedi, A N Parmar, Apurvasinh Puvar, Janvi Raval, Zarna Patel, Monika Gandhi, Pinal Trivedi, R D Dixit, A M Kadri, Harsh Bakshi, Chaitanya Joshi, Madhvi Joshi
EPI_ISL_524755	GMERS Medical College and Hospital, Dharpur, Patan	Gujarat Biotechnology Research Centre	Nidhi Patel, Nitin Savaliya, Raghawendra Kumar, Dinesh Kumar, Zuber Saiyed, Komal Patel, Labdhi Pandya, Afzal Ansari, Nikha Trivedi, A N Parmar,

			Apurvasinh Puvar, Janvi Raval, Zarna Patel, Monika Gandhi, Pinal Trivedi, Maharshi Pandya, R D Dixit, A M Kadri, Harsh Bakshi, Chaitanya Joshi, Madhvi Joshi
EPI_ISL_524756	GMERS Medical College and Hospital, Dharpur, Patan	Gujarat Biotechnology Research Centre	Nitin Savaliya, Raghawendra Kumar, Dinesh Kumar, Zuber Saiyed, Komal Patel, Labdhi Pandya, Afzal Ansari, Nikha Trivedi, A N Parmar, Apurvasinh Puvar, Janvi Raval, Zarna Patel, Monika Gandhi, Pinal Trivedi, Maharshi Pandya, Nidhi Patel, R D Dixit, A M Kadri, Harsh Bakshi, Chaitanya Joshi, Madhvi Joshi
EPI_ISL_524757	GMERS Medical College and Hospital, Dharpur, Patan	Gujarat Biotechnology Research Centre	Raghawendra Kumar, Dinesh Kumar, Zuber Saiyed, Komal Patel, Labdhi Pandya, Afzal Ansari, Nikha Trivedi, A N Parmar, Apurvasinh Puvar, Janvi Raval, Zarna Patel, Monika Gandhi, Pinal Trivedi, Maharshi Pandya, Nidhi Patel, Nitin Savaliya, R D Dixit, A M Kadri, Harsh Bakshi, Chaitanya Joshi, Madhvi Joshi
EPI_ISL_524758	GMERS Medical College and Hospital, Dharpur, Patan	Gujarat Biotechnology Research Centre	Dinesh Kumar, Zuber Saiyed, Komal Patel, Labdhi Pandya, Afzal Ansari, Nikha Trivedi, A N Parmar, Apurvasinh Puvar, Janvi Raval, Zarna Patel, Monika Gandhi, Pinal Trivedi, Maharshi Pandya, Nidhi Patel, Nitin Savaliya, Raghawendra Kumar, R D Dixit, A M Kadri, Harsh Bakshi, Chaitanya Joshi, Madhvi Joshi
EPI_ISL_524759	GMERS Medical College and Hospital, Dharpur, Patan	Gujarat Biotechnology Research Centre	Zuber Saiyed, Komal Patel, Labdhi Pandya, Afzal Ansari, Nikha Trivedi, A N Parmar, Apurvasinh Puvar, Janvi Raval, Zarna Patel, Monika Gandhi, Pinal Trivedi, Maharshi Pandya, Nidhi Patel, Nitin Savaliya, Raghawendra Kumar, Dinesh Kumar, R D Dixit, A M Kadri, Harsh Bakshi, Chaitanya Joshi, Madhvi Joshi
EPI_ISL_524760	GMERS Medical College and Hospital, Dharpur, Patan	Gujarat Biotechnology Research Centre	Komal Patel, Labdhi Pandya, Afzal Ansari, Nikha Trivedi, A N Parmar, Apurvasinh Puvar, Janvi Raval, Zarna Patel, Monika Gandhi, Pinal Trivedi, Maharshi Pandya, Nidhi Patel, Nitin Savaliya, Raghawendra Kumar, Dinesh Kumar, Zuber Saiyed, R D Dixit, A M Kadri, Harsh Bakshi, Chaitanya Joshi, Madhvi Joshi
EPI_ISL_524761	GMERS Medical College and Hospital, Dharpur, Patan	Gujarat Biotechnology Research Centre	Labdhi Pandya, Afzal Ansari, Nikha Trivedi, A N Parmar, Apurvasinh Puvar, Janvi Raval, Zarna Patel, Monika Gandhi, Pinal Trivedi, Maharshi Pandya, Nidhi Patel, Nitin Savaliya, Raghawendra Kumar, Dinesh Kumar, Zuber Saiyed, Komal Patel, R D Dixit, A M Kadri, Harsh Bakshi, Chaitanya Joshi, Madhvi Joshi
EPI_ISL_524762	GMERS Medical College and Hospital, Dharpur, Patan	Gujarat Biotechnology Research Centre	Afzal Ansari, Nikha Trivedi, A N Parmar, Apurvasinh Puvar, Janvi Raval, Zarna Patel, Monika Gandhi, Pinal Trivedi, Maharshi Pandya, Nidhi Patel, Nitin Savaliya, Raghawendra Kumar, Dinesh Kumar, Zuber Saiyed, Komal Patel, Labdhi Pandya, R D Dixit, A M Kadri, Harsh Bakshi, Chaitanya Joshi, Madhvi Joshi
EPI_ISL_524763	GMERS Medical College and Hospital, Dharpur, Patan	Gujarat Biotechnology Research Centre	Nikha Trivedi, A N Parmar, Apurvasinh Puvar, Janvi Raval, Zarna Patel, Monika Gandhi, Pinal Trivedi, Maharshi Pandya, Nidhi Patel, Nitin Savaliya, Raghawendra Kumar, Dinesh Kumar, Zuber Saiyed, Komal Patel, Labdhi Pandya, Afzal Ansari, R D Dixit, A M Kadri, Harsh Bakshi, Chaitanya Joshi, Madhvi Joshi
EPI_ISL_524764	GMERS Medical College and Hospital, Dharpur, Patan	Gujarat Biotechnology Research Centre	A N Parmar, Apurvasinh Puvar, Janvi Raval, Zarna Patel, Monika Gandhi, Pinal Trivedi, Maharshi Pandya, Nidhi Patel, Nitin Savaliya, Raghawendra Kumar, Dinesh Kumar, Zuber Saiyed, Komal Patel, Labdhi Pandya, Afzal Ansari, Nikha Trivedi, R D Dixit, A M Kadri, Harsh Bakshi, Chaitanya Joshi, Madhvi Joshi
EPI_ISL_524765	GMERS Medical College and Hospital, Dharpur, Patan	Gujarat Biotechnology Research Centre	Apurvasinh Puvar, Janvi Raval, Zarna Patel, Monika Gandhi, Pinal Trivedi, Maharshi Pandya, Nidhi Patel, Nitin Savaliya, Raghawendra Kumar, Dinesh Kumar, Zuber Saiyed, Komal Patel, Labdhi Pandya, Afzal Ansari, Nikha Trivedi, A N Parmar, R D Dixit, A M Kadri, Harsh Bakshi, Chaitanya Joshi, Madhvi Joshi
EPI_ISL_524766	GMERS Medical College and Hospital, Dharpur, Patan	Gujarat Biotechnology Research Centre	Janvi Raval, Zarna Patel, Monika Gandhi, Pinal Trivedi, Maharshi Pandya, Nidhi Patel, Nitin Savaliya, Raghawendra Kumar, Dinesh Kumar, Zuber Saiyed, Komal Patel, Labdhi Pandya, Afzal Ansari, Nikha Trivedi, Apurvasinh Puvar, A N Parmar, R D Dixit, A M Kadri, Harsh Bakshi, Chaitanya Joshi, Madhvi Joshi
EPI_ISL_525419	GMERS Medical College and Hospital, Gandhinagar	Gujarat Biotechnology Research Centre	Nitin Savaliya, Raghawendra Kumar, Dinesh Kumar, Zuber Saiyed, Komal Patel, Labdhi Pandya, Afzal Ansari, Nikha Trivedi, Seema Bhatt, Gaurishankar Shrimali, Bhavesh Modi, Bharti Rajani, Apurvasinh Puvar, Janvi Raval, Zarna Patel, Monika Gandhi, Pinal Trivedi, Maharshi Pandya, Nidhi Patel, R D Dixit, A M Kadri, Harsh Bakshi, Chaitanya Joshi, Madhvi Joshi
EPI_ISL_525420	GMERS Medical College and Hospital, Gandhinagar	Gujarat Biotechnology Research Centre	Raghawendra Kumar, Dinesh Kumar, Zuber Saiyed, Komal Patel, Labdhi Pandya, Afzal Ansari, Nikha Trivedi, Seema Bhatt, Gaurishankar Shrimali, Bhavesh Modi, Bharti Rajani, Apurvasinh Puvar, Janvi Raval, Zarna Patel, Monika Gandhi, Pinal Trivedi, Maharshi Pandya, Nidhi Patel, Nitin Savaliya, R D Dixit, A M Kadri, Harsh Bakshi, Chaitanya Joshi, Madhvi Joshi
EPI_ISL_525421	B.J. Medical College and Civil hospital, Ahmedabad	Gujarat Biotechnology Research Centre	Zarna Patel, Monika Gandhi, Pinal Trivedi, Maharshi Pandya, Nidhi Patel, Nitin Savaliya, Raghawendra Kumar, Dinesh Kumar, Zuber Saiyed, Komal Patel, Labdhi Pandya, Afzal Ansari, Nikha Trivedi, Pranay Shah, Kamlesh J Upadhyay, Sanjay Kapadia, Apurvasinh Puvar, Janvi Raval, R D Dixit, A M Kadri, Harsh Bakshi, Chaitanya Joshi, Madhvi Joshi
EPI_ISL_525422	B.J. Medical College and Civil hospital, Ahmedabad	Gujarat Biotechnology Research Centre	Monika Gandhi, Pinal Trivedi, Maharshi Pandya, Nidhi Patel, Nitin Savaliya, Raghawendra Kumar, Dinesh Kumar, Zuber Saiyed, Komal Patel, Labdhi Pandya, Afzal Ansari, Nikha Trivedi, Pranay Shah, Kamlesh J Upadhyay, Sanjay Kapadia, Apurvasinh Puvar, Janvi Raval, Zarna Patel, R D Dixit, A M Kadri, Harsh Bakshi, Chaitanya Joshi, Madhvi Joshi
EPI_ISL_525430	Institute of Microbiology, Universidad San Francisco de Quito	Institute of Microbiology, Universidad San Francisco de Quito	Juan José Guadalupe, Monica Becerra-Wong, Belén Prado-Vivar, Sully Márquez, Bernardo Gutiérrez, Verónica Barragán, Patricio Rojas-Silva, Gabriel Trueba, Michelle Grunauer, Paúl Cárdenas
EPI_ISL_525481	Centre for Dengue Research	Centre for Dengue Research	Chandima Jeewandara, Deshni Jayathilaka, Dinuka Ariyaratne, Laksiri Gomes, Diyanath Ranasinghe, Ananda Wijewickrama, Eranga Narangoda, Damayanthi Idampitiya, Gathsaurie Neelika Malavige
EPI_ISL_525486, EPI_ISL_525489	Centre for Dengue Research	Centre for Dengue Research	Chandima Jeewandara, Deshni Jayathilaka, Dinuka Ariyaratne, Laksiri Gomes, Diyanath Ranasinghe, Ananda Wijewickrama, Malika Karunaratne, Eranga Narangoda, Damayanthi Idampitiya, Gathsaurie Neelika Malavige
EPI_ISL_525492	RSUP dr. SOERADJI TIRTONEGORO	Genetics Working Group (Pokja Genetik) Faculty of Medicine, Public Health and Nursing Universitas Gadjah Mada (FK-KMK UGM), Disease Investigation Center Wates Ministry of Agriculture Indonesia, Department of Microbiology FK-KMK UGM, Laboratorium Diagnostik Yayasan Tahija World Mosquito Program (WMP) Yogyakarta Center for Tropical Medicine FK-KMK UGM, Integrated Research Center FK-KMK UGM, Department of Computer Science and Electronics FMIPA UGM	Gunadi, Hendra Wibawa, . Marcellus, Mohamad S. Hakim, Edwin W. Daniwijaya, Ludhang P. Rizki, Endah Supriyati, Eggi Arguni, Titik Nuryastuti, Tri Wibawa, Dwi AA Nugrahaningsih, Afiahayati , . Siswanto, Kurniyanto, Indah Juliana, Alvin S. Kalim, Dwiki Afandy
EPI_ISL_525495, EPI_ISL_525496	Laboratory of Molecular Virology of the International Centre for Genetic Engineering and Biotechnology (ICGEB)	ARGO Open Lab Platform for Genome Sequencing	Licastro D, Rajasekharan S, Dal Monego S, Segat L, D'Agaro P, Marcello A
EPI_ISL_525572	Istituto Zooprofilattico Sperimentale Puglia e Basilicata; Dipartimento di Bioscienze, Biotecnologie e Biofarmaceutica dell'Università degli Studi di Bari "A.Moro"; Istituto di Biomembrane. Bioenergetica e Biotecnologie Molecolari del Consiglio Nazionale delle Ricerche di Bari	Beaconlab (Bioinformatics, Evolution and Comparative Genomics lab), Dept of Biosciences, University on Milan	Parisi A.,Pesole G., Manzari C., Chiara M
EPI_ISL_526223, EPI_ISL_526224, EPI_ISL_526225	Hungarian Defence Forces Military Medical Centre	National Laboratory of Virology, Szentágothai Research Centre	Endre Gábor Tóth, Balázs Somogyi, Bálint Eszenyi, Ferenc Jakab, Gábor Kemenesi
EPI_ISL_526958, EPI_ISL_526967, EPI_ISL_526971	Instituto Nacional de Salud, Bogotá, Colombia	Instituto Nacional de Salud, Bogotá, Colombia	Katherine Laiton-Donato, Diego A. Álvarez-Díaz, Carlos Franco-Muñoz, Mauricio Pacheco-Montealegre, Jonathan Reales, Diego Andrés Prada, Jose A. Usme-Ciro, Zulma M. Cucunubá, Christian Julian VillabonaArenas, Liz Villabona-Arenas, Sussy Echeverria, Astrid C. Flórez, Carolina Ferro, Diana Marcela Walteros-Acero, Franklin Prieto, Carlos Andrés Durán, Martha Lucia Ospina Martinez, Marcela Mercado-Reyes
EPI_ISL_527742	Centro Nacional De Rehabilitacion Humberto Araya Rojas (Cenare)	Inciensa, Instituto Costarricense de Investigación y Enseñanza en Nutrición y Salud	Francisco Duarte, Hebleen Porras, Claudio Soto-Garita, Estela Cordero, Adriana Godinez & Melany Calderon
EPI_ISL_527753	Hospital San Vicente De Paul	Inciensa, Instituto Costarricense de Investigación y Enseñanza en Nutrición y Salud	Francisco Duarte, Hebleen Porras, Claudio Soto-Garita, Estela Cordero, Adriana Godinez & Melany Calderon
EPI_ISL_527818	Centro de Investigaciones, Universidad de Especialidades Espíritu Santo	Institute of Microbiology, Universidad San Francisco de Quito	Derly Andrade, Juan Carlos Fernandez, Belén Prado-Vivar, Sully Márquez, Juan José Guadalupe, Monica Becerra-Wong, Bernardo Gutiérrez, Gabriel Morey, Ruben Armas, Jose Pedro Barberan, Fernando Espinoza, Edith Lopez, Verónica Barragán, Patricio Rojas-Silva, Gabriel Trueba, Michelle Grunauer, Paúl Cárdenas

EPI_ISL_528691, EPI_ISL_528694, EPI_ISL_528695, EPI_ISL_528696, EPI_ISL_528700, EPI_ISL_528702, EPI_ISL_528703, EPI_ISL_528705, EPI_ISL_528707, EPI_ISL_528708	Alsafar - Khalifa University Abu Dhabi	Alsafar - Khalifa University Abu Dhabi	Andreas Henschel, Gihan Daw Elbait, Samuel Feng, Rifat Hamoudi, Ernesto Damiani, Guan Tay, Habiba Alsafar
EPI_ISL_528743, EPI_ISL_528744	Malaysia Genome Institute	Malaysia Genome Institute	Mohd Noor Mat Isa, Irni Suhayu Sapian, Yusuf Muhammad Noor, Nurhezreen Md Iqbal, Mohd Faizal Abu Bakar, Enizza Kasim, Shamsidar Sopie, Siti Noraini Othman, Azrin Ahmad, Nor Azfa Johari, Shahrul Hisham Zainal Ariffin
EPI_ISL_528745	Laboratorium Kesehatan Provinsi Jawa Barat	School of Life Sciences and Technology & School of Pharmacy-Institut Teknologi Bandung; Molecular Genetics Laboratory-Faculty of Medicine-Universitas Padjadjaran; Laboratorium Kesehatan Provinsi Jawa Barat	Marselina Irasonia Tan, Yunia Sribudiani, Catur Riani, Azzania Fibriani, Husna Nugrahapraja, Tarwadi, Ema Rahmawati, Savira Ekawardhani, Hesti Lina Wiraswati, Ryan Bayusantika Ristandi, Rifky Waluyajati Rachman, Cut Nur Cinthia Alamanda, Lia Faridah, Tri Hanggono Achmad, Mas Rizky A.A. Syamsunarno, Fensi Amalina, Hammam Riza, Sony Solistia Wirawan, Agung Eru Wibowo, Irvan Faizal
EPI_ISL_528746	Immanuel Hospital	Molecular Genetics Laboratory-Faculty of Medicine-Universitas Padjadjaran; School of Life Sciences and Technology & School of Pharmacy-Institut Teknologi Bandung; Laboratorium Kesehatan Provinsi Jawa Barat	Yunia Sribudiani,Tri Hanggono Achmad, Mas Rizky A.A. Syamsunarno, Fensi Amalina, Catur Riani, Azzania Fibriani, Husna Nugrahapraja, Marselina Irasonia Tan, Tarwadi, Ema Rahmawati, Savira Ekawardhani, Hesti Lina Wiraswati, Ryan Bayusantika Ristandi, Rifky Waluyajati Rachman, Cut Nur Cinthia Alamanda, Lia Faridah, Hammam Riza, Sony Solistia Wirawan, Agung Eru Wibowo, Irvan Faizal
EPI_ISL_528747	Santo Borromeus Hospital	School of Pharmacy & School of Life Sciences and Technology - Institut Teknologi Bandung; Molecular Genetics Laboratory-Faculty of Medicine-Universitas Padjadjaran; Laboratorium Kesehatan Provinsi Jawa Barat	Catur Riani, Marselina Irasonia Tan, Yunia Sribudiani, Azzania Fibriani, Husna Nugrahapraja, Tarwadi, Ema Rahmawati, Savira Ekawardhani, Hesti Lina Wiraswati, Ryan Bayusantika Ristandi, Rifky Waluyajati Rachman, Cut Nur Cinthia Alamanda, Lia Faridah, Miftahul Faridl, Karimatu Khoirunnisa, Hammam Riza, Sony Solistia Wirawan, Agung Eru Wibowo, Irvan Faizal
EPI_ISL_528748	Dinkes Provinsi Jawa Barat	School of Life Sciences and Technology & School of Pharmacy-Institut Teknologi Bandung; Molecular Genetics Laboratory-Faculty of Medicine-Universitas Padjadjaran; Laboratorium Kesehatan Provinsi Jawa Barat	Azzania Fibriani, Catur Riani, Marselina Irasonia Tan, Yunia Sribudiani, Husna Nugrahapraja, Tarwadi, Ema Rahmawati, Savira Ekawardhani, Hesti Lina Wiraswati, Ryan Bayusantika Ristandi, Rifky Waluyajati Rachman, Cut Nur Cinthia Alamanda, Lia Faridah, Gusti Ayu Prani Pradani, Adelina Khristiani Rahayu, Hammam Riza, Sony Solistia Wirawan, Agung Eru Wibowo, Irvan Faizal
EPI_ISL_528750	Santo Borromeus Hospital	School of Life Sciences and Technology & School of Pharmacy-Institut Teknologi Bandung; Molecular Genetics Laboratory-Faculty of Medicine-Universitas Padjadjaran; Laboratorium Kesehatan Provinsi Jawa Barat	Marselina Irasonia Tan, Yunia Sribudiani, Catur Riani, Azzania Fibriani, Husna Nugrahapraja, Tarwadi, Ema Rahmawati, Savira Ekawardhani, Hesti Lina Wiraswati, Ryan Bayusantika Ristandi, Rifky Waluyajati Rachman, Cut Nur Cinthia Alamanda, Lia Faridah, Miftahul Faridl, Karimatu Khoirunnisa, Hammam Riza, Sony Solistia Wirawan, Agung Eru Wibowo, Irvan Faizal
EPI_ISL_528751	Santo Borromeus Hospital	Molecular Genetics Laboratory-Faculty of Medicine-Universitas Padjadjaran; School of Life Sciences and Technology & School of Pharmacy-Institut Teknologi Bandung; Laboratorium Kesehatan Provinsi Jawa Barat	Yunia Sribudiani, Tri Hanggono Achmad, Mas Rizky A.A. Syamsunarno, Fensi Amalina, Catur Riani, Azzania Fibriani, Husna Nugrahapraja, Marselina Irasonia Tan, Tarwadi, Ema Rahmawati, Savira Ekawardhani, Hesti Lina Wiraswati, Ryan Bayusantika Ristandi, Rifky Waluyajati Rachman, Cut Nur Cinthia Alamanda, Lia Faridah, Hammam Riza, Sony Solistia Wirawan, Agung Eru Wibowo, Irvan Faizal
EPI_ISL_528752	Dr. H. A. Rotinsulu Lung Hospital	School of Pharmacy & School of Life Sciences and Technology - Institut Teknologi Bandung; Molecular Genetics Laboratory-Faculty of Medicine-Universitas Padjadjaran; Laboratorium Kesehatan Provinsi Jawa Barat	Catur Riani, Marselina Irasonia Tan, Yunia Sribudiani, Azzania Fibriani, Husna Nugrahapraja, Tarwadi, Ema Rahmawati, Savira Ekawardhani, Hesti Lina Wiraswati, Ryan Bayusantika Ristandi, Rifky Waluyajati Rachman, Cut Nur Cinthia Alamanda, Lia Faridah, Gusti Ayu Prani Pradani, Adelina Khristiani Rahayu, Hammam Riza, Sony Solistia Wirawan, Agung Eru Wibowo, Irvan Faizal
EPI_ISL_528753	Dinkes Kota Bogor	School of Life Sciences and Technology & School of Pharmacy-Institut Teknologi Bandung; Molecular Genetics Laboratory-Faculty of Medicine-Universitas Padjadjaran; Laboratorium Kesehatan Provinsi Jawa Barat	Azzania Fibriani, Catur Riani, Marselina Irasonia Tan, Yunia Sribudiani, Husna Nugrahapraja, Tarwadi, Ema Rahmawati, Savira Ekawardhani, Hesti Lina Wiraswati, Ryan Bayusantika Ristandi, Rifky Waluyajati Rachman, Cut Nur Cinthia Alamanda, Lia Faridah, Davin H. E. Setiamarga, Rizki Mardian , Hammam Riza, Sony Solistia Wirawan, Agung Eru Wibowo, Irvan Faizal
EPI_ISL_528759	Santo Borromeus Hospital	School of Life Sciences and Technology & School of Pharmacy-Institut Teknologi Bandung; Molecular Genetics Laboratory-Faculty of Medicine-Universitas Padjadjaran; Laboratorium Kesehatan Provinsi Jawa Barat	Husna Nugrahapraja, Azzania Fibriani, Catur Riani, Marselina Irasonia Tan, Yunia Sribudiani, Tarwadi, Ema Rahmawati, Savira Ekawardhani, Hesti Lina Wiraswati, Ryan Bayusantika Ristandi, Rifky Waluyajati Rachman, Cut Nur Cinthia Alamanda, Lia Faridah, Tri Hanggono Achmad, Mas Rizky A.A. Syamsunarno, Fensi Amalina, Hammam Riza, Sony Solistia Wirawan, Agung Eru Wibowo, Irvan Faizal
EPI_ISL_528809	Department of Medicine, Gandhi hospital, Hyderabad	CSIR-Centre for Cellular and Molecular Biology	Rajarao Mesipogu , Thrilok Chander Bingi ,Vinayasekhar Aedula,Tulasi Nagabandi, Namami Gaur, Sakshi Shambhavi, Lamuk Zaveri, Shagufta Khan, Nikhil Hajirnis, M Soujanya Reddy, Pratheusa Maccha, Purushotham Vodnala, Payel Mukherjee, Sofia Banu, Priya Singh, Onkar Kulkarni, Dhiviya Vedagiri, Divya Gupta, Vishal Sah, Santosh Kumar Kuncha, Krishnan Harinivas Harshan, Archana Bharadwaj Siva, Karthik Bharadwaj Tallapaka,G. Aditya Kumar, Koushick Sivakumar, Pooja Ramesh Gupta, Rajan Kumar Jha, Shraddha Vijay Lahoti, Rakesh K Mishra, Divya Tej Sowpati
EPI_ISL_528810, EPI_ISL_528811, EPI_ISL_528812	Department of Medicine, Gandhi hospital, Hyderabad	CSIR-Centre for Cellular and Molecular Biology	Thrilok Chander Bingi,Rajarao Mesipogu ,Vinayasekhar Aedula,Lamuk Zaveri, Shagufta Khan, Namami Gaur, Sakshi Shambhavi, Nikhil Hajirnis, M Soujanya Reddy, Pratheusa Maccha, Tulasi Nagabandi, Purushotham Vodnala, Payel Mukherjee, Sofia Banu, Priya Singh, Onkar Kulkarni, Dhiviya Vedagiri, Divya Gupta, Vishal Sah, Santosh Kumar Kuncha, Krishnan Harinivas Harshan, Archana Bharadwaj Siva, Karthik Bharadwaj Tallapaka, Renu Sudhakar, Somesh Gorde, Gangumala Srinivas Reddy, Sujoy Deb, Swati Bayyana, Rakesh K Mishra, Divya Tej Sowpati
EPI_ISL_528813	Department of Medicine, Gandhi hospital, Hyderabad	CSIR-Centre for Cellular and Molecular Biology	Vinayasekhar Aedula,Thrilok Chander Bingi, Rajarao Mesipogu, Shagufta Khan, Lamuk Zaveri, Namami Gaur, Sakshi Shambhavi, Nikhil Hajirnis, M Soujanya Reddy, Pratheusa Maccha,Tulasi Nagabandi, Purushotham Vodnala, Payel Mukherjee, Sofia Banu, Priya Singh, Onkar Kulkarni, Dhiviya Vedagiri, Divya Gupta, Vishal Sah, Santosh Kumar Kuncha, Krishnan Harinivas Harshan, Archana Bharadwaj Siva, Karthik Bharadwaj Tallapaka,Umesh Kumar, Unis Ahmad Bhat, Ajay Sarawagi, Priyanka Pant, Rajkanwar Nathawat, Rakesh K Mishra, Divya Tej Sowpati
EPI_ISL_528814	Department of Medicine, Gandhi hospital, Hyderabad	CSIR-Centre for Cellular and Molecular Biology	Rajarao Mesipogu , Thrilok Chander Bingi ,Vinayasekhar Aedula,Tulasi Nagabandi, Namami Gaur, Sakshi Shambhavi, Lamuk Zaveri, Shagufta Khan, Nikhil Hajirnis, M Soujanya Reddy, Pratheusa Maccha, Purushotham Vodnala, Payel Mukherjee, Sofia Banu, Priya Singh, Onkar Kulkarni, Dhiviya Vedagiri, Divya Gupta, Vishal Sah, Santosh Kumar Kuncha, Krishnan Harinivas Harshan, Archana Bharadwaj Siva, Karthik Bharadwaj Tallapaka,G. Aditya Kumar, Koushick Sivakumar, Pooja Ramesh Gupta, Rajan Kumar Jha, Shraddha Vijay Lahoti, Rakesh K Mishra, Divya Tej Sowpati
EPI_ISL_528815	Department of Medicine, Gandhi hospital, Hyderabad	CSIR-Centre for Cellular and Molecular Biology	Vinayasekhar Aedula,Thrilok Chander Bingi, Rajarao Mesipogu, Shagufta Khan, Lamuk Zaveri, Namami Gaur, Sakshi Shambhavi, Nikhil Hajirnis, M Soujanya Reddy, Pratheusa Maccha,Tulasi Nagabandi, Purushotham Vodnala, Payel Mukherjee, Sofia Banu, Priya Singh, Onkar Kulkarni, Dhiviya Vedagiri, Divya Gupta, Vishal Sah, Santosh Kumar Kuncha, Krishnan Harinivas Harshan, Archana Bharadwaj Siva, Karthik Bharadwaj Tallapaka,Umesh Kumar, Unis Ahmad Bhat, Ajay Sarawagi, Priyanka Pant, Rajkanwar Nathawat, Rakesh K Mishra, Divya Tej Sowpati
EPI_ISL_528816	Department of Medicine, Gandhi hospital, Hyderabad	CSIR-Centre for Cellular and Molecular Biology	Rajarao Mesipogu , Thrilok Chander Bingi ,Vinayasekhar Aedula,Tulasi Nagabandi, Namami Gaur, Sakshi Shambhavi, Lamuk Zaveri, Shagufta Khan, Nikhil Hajirnis, M Soujanya Reddy, Pratheusa Maccha, Purushotham Vodnala, Payel Mukherjee, Sofia Banu, Priya Singh, Onkar Kulkarni, Dhiviya Vedagiri, Divya Gupta, Vishal Sah, Santosh Kumar Kuncha, Krishnan Harinivas Harshan, Archana Bharadwaj Siva, Karthik Bharadwaj Tallapaka,G. Aditya Kumar, Koushick Sivakumar, Pooja Ramesh Gupta, Rajan Kumar Jha, Shraddha Vijay Lahoti, Rakesh K Mishra, Divya Tej Sowpati
EPI_ISL_528817	Department of Medicine, Gandhi hospital, Hyderabad	CSIR-Centre for Cellular and Molecular Biology	Thrilok Chander Bingi,Rajarao Mesipogu ,Vinayasekhar Aedula,Lamuk Zaveri, Shagufta Khan, Namami Gaur, Sakshi Shambhavi, Nikhil Hajirnis, M Soujanya Reddy, Pratheusa Maccha, Tulasi Nagabandi, Purushotham Vodnala, Payel Mukherjee, Sofia Banu, Priya Singh, Onkar Kulkarni, Dhiviya Vedagiri, Divya Gupta, Vishal Sah, Santosh Kumar Kuncha, Krishnan Harinivas Harshan, Archana Bharadwaj Siva, Karthik Bharadwaj Tallapaka, Renu Sudhakar, Somesh Gorde, Gangumala Srinivas Reddy, Sujoy Deb, Swati Bayyana, Rakesh K Mishra, Divya Tej Sowpati
EPI_ISL_528818	Department of Medicine, Gandhi hospital, Hyderabad	CSIR-Centre for Cellular and Molecular Biology	Vinayasekhar Aedula,Thrilok Chander Bingi, Rajarao Mesipogu, Shagufta Khan, Lamuk Zaveri, Namami Gaur, Sakshi Shambhavi, Nikhil Hajirnis, M Soujanya Reddy, Pratheusa Maccha,Tulasi Nagabandi, Purushotham Vodnala, Payel Mukherjee, Sofia Banu, Priya Singh, Onkar Kulkarni, Dhiviya Vedagiri, Divya Gupta, Vishal Sah, Santosh Kumar Kuncha, Krishnan Harinivas Harshan, Archana Bharadwaj Siva, Karthik Bharadwaj Tallapaka,Umesh Kumar, Unis Ahmad Bhat, Ajay Sarawagi, Priyanka Pant, Rajkanwar Nathawat, Rakesh K Mishra, Divya Tej Sowpati
EPI_ISL_528819, EPI_ISL_528820	Department of Medicine, Gandhi hospital, Hyderabad	CSIR-Centre for Cellular and Molecular Biology	Rajarao Mesipogu , Thrilok Chander Bingi ,Vinayasekhar Aedula,Tulasi Nagabandi, Namami Gaur, Sakshi Shambhavi, Lamuk Zaveri, Shagufta Khan, Nikhil Hajirnis, M Soujanya Reddy, Pratheusa Maccha, Purushotham Vodnala, Payel Mukherjee, Sofia Banu, Priya Singh, Onkar Kulkarni, Dhiviya Vedagiri, Divya Gupta, Vishal Sah, Santosh Kumar Kuncha, Krishnan Harinivas Harshan, Archana Bharadwaj Siva, Karthik Bharadwaj Tallapaka,G. Aditya Kumar, Koushick

			Sivakumar, Pooja Ramesh Gupta, Rajan Kumar Jha, Shraddha Vijay Lahoti, Rakesh K Mishra, Divya Tej Sowpati
EPI_ISL_528821	Department of Medicine, Gandhi hospital, Hyderabad	CSIR-Centre for Cellular and Molecular Biology	Thrilok Chander Bingi,Rajarao Mesipogu ,Vinayasekhar Aedula,Lamuk Zaveri, Shagufta Khan, Namami Gaur, Sakshi Shambhavi, Nikhil Hajirnis, M Soujanya Reddy, Pratheusa Maccha, Tulasi Nagabandi, Purushotham Vodnala, Payel Mukherjee, Sofia Banu, Priya Singh, Onkar Kulkarni, Dhiviya Vedagiri, Divya Gupta, Vishal Sah, Santosh Kumar Kuncha, Krishnan Harinivas Harshan, Archana Bharadwaj Siva, Karthik Bharadwaj Tallapaka, Renu Sudhakar, Somesh Gorde, Gangumala Srinivas Reddy, Sujoy Deb, Swati Bayyana, Rakesh K Mishra, Divya Tej Sowpati
EPI_ISL_528822	Department of Medicine, Gandhi hospital, Hyderabad	CSIR-Centre for Cellular and Molecular Biology	Vinayasekhar Aedula,Thrilok Chander Bingi, Rajarao Mesipogu, Shagufta Khan, Lamuk Zaveri, Namami Gaur, Sakshi Shambhavi, Nikhil Hajirnis, M Soujanya Reddy, Pratheusa Maccha,Tulasi Nagabandi, Purushotham Vodnala, Payel Mukherjee, Sofia Banu, Priya Singh, Onkar Kulkarni, Dhiviya Vedagiri, Divya Gupta, Vishal Sah, Santosh Kumar Kuncha, Krishnan Harinivas Harshan, Archana Bharadwaj Siva, Karthik Bharadwaj Tallapaka,Umesh Kumar, Unis Ahmad Bhat, Ajay Sarawagi, Priyanka Pant, Rajkanwar Nathawat, Rakesh K Mishra, Divya Tej Sowpati
EPI_ISL_529003, EPI_ISL_529004, EPI_ISL_529005	Servizio di igiene epidemiologia e sanità pubblica (SIESP)-Chieti	Istituto Zooprofilattico Sperimentale dell'Abruzzo e Molise "G.Caporale"	Lorusso A, Marcacci M, Di Domenico M, Curini V, Ancora M, Cammà C, Rinaldi A, Mangone I, Di Pasquale A, Puglia I, Savini G.
EPI_ISL_529006	Servizio di igiene e sanità pubblica (SIESP)-Teramo	Istituto Zooprofilattico Sperimentale dell'Abruzzo e Molise "G.Caporale"	Lorusso A, Marcacci M, Di Domenico M, Curini V, Ancora M, Cammà C, Rinaldi A, Mangone I, Di Pasquale A, Puglia I, Savini G.
EPI_ISL_529007	Ospedale Civile S. Liberatore-Atri	Istituto Zooprofilattico Sperimentale dell'Abruzzo e Molise "G.Caporale"	Lorusso A, Marcacci M, Di Domenico M, Curini V, Ancora M, Cammà C, Rinaldi A, Mangone I, Di Pasquale A, Puglia I, Savini G.
EPI_ISL_529010, EPI_ISL_529011, EPI_ISL_529012	Servizio Igiene Epidemiologia e Sanità Pubblica (SIESP)-L'Aquila	Istituto Zooprofilattico Sperimentale dell'Abruzzo e Molise "G.Caporale"	Lorusso A, Marcacci M, Di Domenico M, Curini V, Ancora M, Cammà C, Rinaldi A, Mangone I, Di Pasquale A, Puglia I, Savini G.
EPI_ISL_529013	Presidio Ospedaliero "S.Filippo e Nicola"-Avezzano	Istituto Zooprofilattico Sperimentale dell'Abruzzo e Molise "G.Caporale"	Lorusso A, Marcacci M, Di Domenico M, Curini V, Ancora M, Cammà C, Rinaldi A, Mangone I, Di Pasquale A, Puglia I, Savini G.
EPI_ISL_529014, EPI_ISL_529015	Ospedale "Ss. Annunziata"	Istituto Zooprofilattico Sperimentale dell'Abruzzo e Molise "G.Caporale"	Lorusso A, Marcacci M, Di Domenico M, Curini V, Ancora M, Cammà C, Rinaldi A, Mangone I, Di Pasquale A, Puglia I, Savini G.
EPI_ISL_529031	Central Molecular Microbiology Laboratory, Clinical and Chemical Pathology Department, Faculty of Medicine, CAIRO UNIVERSITY	Next Generation Sequencing Reference Laboratory, Faculty of Medicine, Cairo University and The Center for Genome and Microbiome Research, Faculty of Pharmacy, CAIRO UNIVERSITY	May Sherif Soliman, May Abdelfattah, Ramy Karam Aziz
EPI_ISL_529032	Central Molecular Microbiology Laboratory and Next Generation Sequencing Reference Laboratory, Clinical and Chemical Pathology Department, Faculty of Medicine, CAIRO UNIVERSITY	Next Generation Sequencing Reference Laboratory, Faculty of Medicine, CAIRO UNIVERSITY and The Center for Genome and Microbiome Research, Faculty of Pharmacy, CAIRO UNIVERSITY	May Sherif Soliman, May Abdelfattah, Ramy Karam Aziz
EPI_ISL_529720, EPI_ISL_529721, EPI_ISL_529723, EPI_ISL_529724, EPI_ISL_529726, EPI_ISL_529728, EPI_ISL_529729, EPI_ISL_529730, EPI_ISL_529731, EPI_ISL_529732, EPI_ISL_529733, EPI_ISL_529734, EPI_ISL_529735, EPI_ISL_529736, EPI_ISL_529737, EPI_ISL_529738, EPI_ISL_529739, EPI_ISL_529740, EPI_ISL_529741, EPI_ISL_529742, EPI_ISL_529743, EPI_ISL_529744, EPI_ISL_529745, EPI_ISL_529747, EPI_ISL_529748, EPI_ISL_529749, EPI_ISL_529750, EPI_ISL_529751, EPI_ISL_529752, EPI_ISL_529753, EPI_ISL_529755, EPI_ISL_529756, EPI_ISL_529757, EPI_ISL_529758, EPI_ISL_529759, EPI_ISL_529760, EPI_ISL_529761, EPI_ISL_529762, EPI_ISL_529763, EPI_ISL_529764, EPI_ISL_529765, EPI_ISL_529766, EPI_ISL_529767, EPI_ISL_529769, EPI_ISL_529770, EPI_ISL_529771, EPI_ISL_529772, EPI_ISL_529773, EPI_ISL_529774, EPI_ISL_529775, EPI_ISL_529776, EPI_ISL_529777, EPI_ISL_529778, EPI_ISL_529779, EPI_ISL_529780, EPI_ISL_529782, EPI_ISL_529783, EPI_ISL_529784, EPI_ISL_529785, EPI_ISL_529786, EPI_ISL_529787, EPI_ISL_529788, EPI_ISL_529789, EPI_ISL_529790, EPI_ISL_529791, EPI_ISL_529792, EPI_ISL_529793, EPI_ISL_529794, EPI_ISL_529796, EPI_ISL_529797, EPI_ISL_529798, EPI_ISL_529799, EPI_ISL_529800, EPI_ISL_529801, EPI_ISL_529802			
see above	NHLS-IALCH	KRISP, KZN Research Innovation and Sequencing Platform	Giandhari J, Pillay S, Lessells R, Mdlalose K, York D, Khan S, Tegally H, Wilkinson E, de Oliveira T
EPI_ISL_529963	Universitas Airlangga Hospital	Institute of Tropical Disease, Universitas Airlangga	Rima R Prasetya, Krisnoadi Rahardjo, Aldise M Nastri, Jezzy R Dewantari, Nasronudin, Gatot Soegiarto, Laksmi Wulandari, Retno A Setyoningrum, Resti Yudhawati, Yohko K Shimizu, Mitsuhiro Nishimura, Yasuko Mori, Soetjipto, Kazufumi Shimizu, Maria I Lusida
EPI_ISL_529967	RSUD Sidoarjo	Institute of Tropical Disease, Universitas Airlangga	Aldise M Nastri, Jezzy R Dewantari, Rima R Prasetya, Krisnoadi Rahardjo, Atok Irawan, Gatot Soegiarto, Laksmi Wulandari, Retno A Setyoningrum, Resti Yudhawati, Yohko K Shimizu, Mitsuhiro Nishimura, Yasuko Mori, Soetjipto, Kazufumi Shimizu, Maria I Lusida
EPI_ISL_534336	Department of Laboratory Medicine, National Taiwan University Hospital	Microbial Genomics Core Lab, National Taiwan University Centers of Genomic and Precision Medicine	Shiou-Hwei Yeh, You-Yu Lin, Ya-Yun Lai, Chiao-Ling Li, Shan-Chwen Chang, Pei-Jer Chen, Sui-Yuan Chang
EPI_ISL_534337, EPI_ISL_534338, EPI_ISL_534339, EPI_ISL_534340, EPI_ISL_534341, EPI_ISL_534342, EPI_ISL_534343, EPI_ISL_534344, EPI_ISL_534345	Molecular diagnostic laboratory of Federal Budget Institution of Science "Central Research Institute of Epidemiology" of The Federal Service on Customers' Rights Protection and Human Well-being Surveillance	Group of Genomics and Postgenomic Technologies of Central Research Institute of Epidemilology	Speranskaya AS, Kaptelova VV, Samoilov AE, Valdokhina AV, Bulanenko VP, Bukharina A.Y., Tivanova EV, Shipulina OY, Akimkin VG
EPI_ISL_535390, EPI_ISL_535392, EPI_ISL_535393, EPI_ISL_535394, EPI_ISL_535395, EPI_ISL_535396, EPI_ISL_535397, EPI_ISL_535398, EPI_ISL_535399, EPI_ISL_535400, EPI_ISL_535403, EPI_ISL_535404, EPI_ISL_535405, EPI_ISL_535406, EPI_ISL_535408, EPI_ISL_535410, EPI_ISL_535412, EPI_ISL_535413, EPI_ISL_535414, EPI_ISL_535415, EPI_ISL_535416, EPI_ISL_535417, EPI_ISL_535418, EPI_ISL_535420, EPI_ISL_535421, EPI_ISL_535422, EPI_ISL_535424, EPI_ISL_535425, EPI_ISL_535426, EPI_ISL_535427, EPI_ISL_535428, EPI_ISL_535430, EPI_ISL_535431, EPI_ISL_535432, EPI_ISL_535433, EPI_ISL_535434, EPI_ISL_535435, EPI_ISL_535436, EPI_ISL_535438, EPI_ISL_535439, EPI_ISL_535440, EPI_ISL_535441, EPI_ISL_535442, EPI_ISL_535443, EPI_ISL_535444, EPI_ISL_535445, EPI_ISL_535446, EPI_ISL_535447, EPI_ISL_535448, EPI_ISL_535449, EPI_ISL_535450, EPI_ISL_535451, EPI_ISL_535452, EPI_ISL_535454, EPI_ISL_535455, EPI_ISL_535456, EPI_ISL_535457, EPI_ISL_535458, EPI_ISL_535459, EPI_ISL_535460, EPI_ISL_535461, EPI_ISL_535462, EPI_ISL_535463, EPI_ISL_535464, EPI_ISL_535465, EPI_ISL_535466, EPI_ISL_535467, EPI_ISL_535468, EPI_ISL_535469, EPI_ISL_535470, EPI_ISL_535471, EPI_ISL_535472, EPI_ISL_535473, EPI_ISL_535475, EPI_ISL_535476, EPI_ISL_535477, EPI_ISL_535478, EPI_ISL_535479, EPI_ISL_535480, EPI_ISL_535481, EPI_ISL_535483, EPI_ISL_535484, EPI_ISL_535485, EPI_ISL_535487, EPI_ISL_535488, EPI_ISL_535496, EPI_ISL_535497, EPI_ISL_535498, EPI_ISL_535499, EPI_ISL_535500, EPI_ISL_535501, EPI_ISL_535502, EPI_ISL_535503, EPI_ISL_535504, EPI_ISL_535505, EPI_ISL_535506, EPI_ISL_535507, EPI_ISL_535508, EPI_ISL_535509, EPI_ISL_535510, EPI_ISL_535511, EPI_ISL_535512, EPI_ISL_535513, EPI_ISL_535514, EPI_ISL_535515, EPI_ISL_535516, EPI_ISL_535518, EPI_ISL_535519, EPI_ISL_535520, EPI_ISL_535522, EPI_ISL_535523, EPI_ISL_535526, EPI_ISL_535527, EPI_ISL_535529, EPI_ISL_535530, EPI_ISL_535531, EPI_ISL_535532, EPI_ISL_535533, EPI_ISL_535534, EPI_ISL_535536, EPI_ISL_535537, EPI_ISL_535539, EPI_ISL_535540, EPI_ISL_535541, EPI_ISL_535542, EPI_ISL_535543, EPI_ISL_535544, EPI_ISL_535545, EPI_ISL_535546, EPI_ISL_535547, EPI_ISL_535551, EPI_ISL_535552, EPI_ISL_535553, EPI_ISL_535554, EPI_ISL_535555, EPI_ISL_535556, EPI_ISL_535557, EPI_ISL_535558, EPI_ISL_535559, EPI_ISL_535560, EPI_ISL_535561, EPI_ISL_535562, EPI_ISL_535563, EPI_ISL_535564, EPI_ISL_535565, EPI_ISL_535566, EPI_ISL_535567, EPI_ISL_535568, EPI_ISL_535569, EPI_ISL_535570, EPI_ISL_535571, EPI_ISL_535572			
see above	NHLS-IALCH	KRISP, KZN Research Innovation and Sequencing Platform	Giandhari J, Pillay S, Lessells R, Mdlalose K, York D, Khan S, Tegally H, Wilkinson E, de Oliveira T
EPI_ISL_535575	Hospital Universitari Germans Trias i Pujol(HUGTiP)/Fundació Lluita contra la SIDA (FLSida)	IrsiCaixa AIDS Research Lab	Marc Noguera-Julian, Mariona Parera, Maria Pilar Armengol, Marta Massanella, Ester Ballana, Lidia Ruiz, Nuria Izquierdo, Jorge Carrillo, Roger Paredes, Julia Blanco, Joaquim Segalés, Bonaventura Clotet
EPI_ISL_536411, EPI_ISL_536793	Medtimes Molecular Laboratory	Medtimes Molecular Laboratory	Eric Chan, Winsome Wong, Jacqueline Tam, Isaac Chow
EPI_ISL_538500, EPI_ISL_538501	RS Prima Husada Cipta Medan North Sumatra	National Institute of Health Research and Development	Pawestri, HA; Subangkit; Puspa, KD; Nugraha, AA; Ikawati, HD; Pangesti, KNA; Soekarso, T; Paisal; Setiawaty,V.
EPI_ISL_538502	RSUD Sultan Imanudin Pangkalan Bun Center Kalimantan	National Institute of Health Research and Development	Pawestri, HA; Subangkit; Puspa, KD; Nugraha, AA; Ikawati, HD; Pangesti, KNA; Soekarso, T; Paisal; Setiawaty,V.
EPI_ISL_538552	Hospital Universitari Germans Trias i Pujol(HUGTiP)/Fundació Lluita contra la SIDA (FLSida)	IrsiCaixa AIDS Research Lab	Marc Noguera-Julian, Mariona Parera, Maria Pilar Armengol, Marta Massanella, Ester Ballana, Lidia Ruiz, Nuria Izquierdo, Jorge Carrillo, Roger Paredes, Julia Blanco, Joaquim Segalés, Bonaventura Clotet
EPI_ISL_539495	Centers for Disease Control and Prevention, Dengue Branch	Centers for Disease Control and Prevention, Dengue Branch	Gilberto A. Santiago, Glenda Gonzalez, Betzabel Flores, Keyla Charriez, Jorge L. Munoz-Jordan, Gabriela Paz-Bailey, Janice Perez, Vanessa Rivera-Amill, Diego Sainz de la Peña, Jorge Bertran
EPI_ISL_539538, EPI_ISL_539539, EPI_ISL_539540, EPI_ISL_539541, EPI_ISL_539542, EPI_ISL_539543, EPI_ISL_539544, EPI_ISL_539547, EPI_ISL_539548	Hospital Clínic	Instituto de Salud Carlos III	Iglesias-Caballero, M. Molinero Calamita, M. González-Esguevillas, M. Camarero, S. Pozo, F. Casas, I. Jiménez, P. Jiménez, M. Zaballos, A. Monzón, S. Varona, S. Juliá, M. Cuesta, I, M.A Marcos
EPI_ISL_539567	Hospital Comarcal de Melilla	Instituto de Salud Carlos III	Iglesias-Caballero, M. Molinero Calamita, M. González-Esguevillas, M. Camarero, S. Pozo, F. Casas, I. Jiménez, P. Jiménez, M. Zaballos, A. Monzón, S. Varona, S. Juliá, M. Cuesta, I, J. López
EPI_ISL_539568	Hospital Comarcal de Melilla	Instituto de Salud Carlos III	Iglesias-Caballero, M. Molinero Calamita, M. González-Esguevillas, M. Camarero, S. Pozo, F. Casas, I. Jiménez, P. Jiménez, M. Zaballos, A. Monzón, S. Varona, S. Juliá, M. Cuesta, I, C. Ezpeleta

EPI_ISL_539782	National Institute of Public Health (Czech Republic)	State Veterinary Institute Prague	Nagy, A; Jirincova, H; Novakova, L; Trnka, D; Vecerova, J.
EPI_ISL_539803	Queen Mary Hospital	Hong Kong Department of Health	Alan K.L. Tsang, Peter C.W. Yip, Edman T.K. Lam, Rickjason C.W. Chan, Dominic N.C. Tsang
EPI_ISL_539804	Yan Chai Hospital	Hong Kong Department of Health	Alan K.L. Tsang, Peter C.W. Yip, Edman T.K. Lam, Rickjason C.W. Chan, Dominic N.C. Tsang
EPI_ISL_539805	Kwong Wah Hospital	Hong Kong Department of Health	Alan K.L. Tsang, Peter C.W. Yip, Edman T.K. Lam, Rickjason C.W. Chan, Dominic N.C. Tsang
EPI_ISL_539806, EPI_ISL_539807, EPI_ISL_539808	Princess Margaret Hospital	Hong Kong Department of Health	Alan K.L. Tsang, Peter C.W. Yip, Edman T.K. Lam, Rickjason C.W. Chan, Dominic N.C. Tsang
EPI_ISL_539809, EPI_ISL_539811, EPI_ISL_539812	Asiaworld Expo Command Post	Hong Kong Department of Health	Alan K.L. Tsang, Peter C.W. Yip, Edman T.K. Lam, Rickjason C.W. Chan, Dominic N.C. Tsang
EPI_ISL_539813	Prince of Wales Hospital	Hong Kong Department of Health	Alan K.L. Tsang, Peter C.W. Yip, Edman T.K. Lam, Rickjason C.W. Chan, Dominic N.C. Tsang
EPI_ISL_539814	Tuen Mun Hospital	Hong Kong Department of Health	Alan K.L. Tsang, Peter C.W. Yip, Edman T.K. Lam, Rickjason C.W. Chan, Dominic N.C. Tsang
EPI_ISL_539815	Queen Elizabeth Hospital	Hong Kong Department of Health	Alan K.L. Tsang, Peter C.W. Yip, Edman T.K. Lam, Rickjason C.W. Chan, Dominic N.C. Tsang
EPI_ISL_539816, EPI_ISL_539817	Queen Mary Hospital	Hong Kong Department of Health	Alan K.L. Tsang, Peter C.W. Yip, Edman T.K. Lam, Rickjason C.W. Chan, Dominic N.C. Tsang
EPI_ISL_539818, EPI_ISL_539819	Tuen Mun Hospital	Hong Kong Department of Health	Alan K.L. Tsang, Peter C.W. Yip, Edman T.K. Lam, Rickjason C.W. Chan, Dominic N.C. Tsang
EPI_ISL_539821	Asiaworld Expo Command Post	Hong Kong Department of Health	Alan K.L. Tsang, Peter C.W. Yip, Edman T.K. Lam, Rickjason C.W. Chan, Dominic N.C. Tsang
EPI_ISL_539822, EPI_ISL_539823	Communicable Disease Branch	Hong Kong Department of Health	Alan K.L. Tsang, Peter C.W. Yip, Edman T.K. Lam, Rickjason C.W. Chan, Dominic N.C. Tsang
EPI_ISL_541002, EPI_ISL_541003, EPI_ISL_541004, EPI_ISL_541005, EPI_ISL_541006, EPI_ISL_541007	Health and Environmental Research Institute of Gwangju Metropolitan city	Health and Environmental Research Institute of Gwangju Metropolitan city	Min Ji Kim, Ji-eun Lee
EPI_ISL_541082	The National Institute of Public Health	State Veterinary Institute Prague	Nagy,A;Jirincova,H;Novakova,L;Trnka,D;Vecerova,J
EPI_ISL_541332, EPI_ISL_541333, EPI_ISL_541334, EPI_ISL_541336, EPI_ISL_541337	The National Institute of Public Health	State Veterinary Institute Prague	Nagy,A; Jirincova,H; Novakova,L; Trnka,D; Vecerova,J
EPI_ISL_545580, EPI_ISL_545954, EPI_ISL_545956	The National Institute of Public Health	State Veterinary Institute Prague	Nagy,A;Jirincova,H;Novakova,L;Trnka,D;Vecerova,J
EPI_ISL_545957, EPI_ISL_546436, EPI_ISL_546935, EPI_ISL_546936	The National Institute of Public Health	State Veterinary Institute Prague	Nagy,A; Jirincova,H; Novakova,L; Trnka,D; Vecerova,J
EPI_ISL_547571	Hospital Municipal Antônio Giglio	Instituto Adolfo Lutz, Interdiciplinary Procedures Center, Strategic Laboratory	Claudio Tavares Sacchi, Claudia Regina Gonçalves, Erica Valessa Ramos Gomes, Karoline Rodrigues Campos
EPI_ISL_547573	Vigilância em Saúde de Cajamar	Instituto Adolfo Lutz, Interdiciplinary Procedures Center, Strategic Laboratory	Claudio Tavares Sacchi, Claudia Regina Gonçalves, Erica Valessa Ramos Gomes, Karoline Rodrigues Campos
EPI_ISL_547574	Hospital Universitario da USP	Instituto Adolfo Lutz, Interdiciplinary Procedures Center, Strategic Laboratory	Claudio Tavares Sacchi, Claudia Regina Gonçalves, Erica Valessa Ramos Gomes, Karoline Rodrigues Campos
EPI_ISL_547575	SVO Jundiaí	Instituto Adolfo Lutz, Interdiciplinary Procedures Center, Strategic Laboratory	Claudio Tavares Sacchi, Claudia Regina Gonçalves, Erica Valessa Ramos Gomes, Karoline Rodrigues Campos
EPI_ISL_547576	Secretaria Municipal de Saúde	Instituto Adolfo Lutz, Interdiciplinary Procedures Center, Strategic Laboratory	Claudio Tavares Sacchi, Claudia Regina Gonçalves, Erica Valessa Ramos Gomes, Karoline Rodrigues Campos
EPI_ISL_547577	Hospital e Maternidade Nossa Senhora das Graças	Instituto Adolfo Lutz, Interdiciplinary Procedures Center, Strategic Laboratory	Claudio Tavares Sacchi, Claudia Regina Gonçalves, Erica Valessa Ramos Gomes, Karoline Rodrigues Campos
EPI_ISL_547578	Hospital Doutor Domingos Leonardo Cerávolo	Instituto Adolfo Lutz, Interdiciplinary Procedures Center, Strategic Laboratory	Claudio Tavares Sacchi, Claudia Regina Gonçalves, Erica Valessa Ramos Gomes, Karoline Rodrigues Campos
EPI_ISL_547579	Santa Casa de Misericórdia de Araçatuba	Instituto Adolfo Lutz, Interdiciplinary Procedures Center, Strategic Laboratory	Claudio Tavares Sacchi, Claudia Regina Gonçalves, Erica Valessa Ramos Gomes, Karoline Rodrigues Campos
EPI_ISL_547580	Santa Casa da Misericórdia de Presidente Prudente	Instituto Adolfo Lutz, Interdiciplinary Procedures Center, Strategic Laboratory	Claudio Tavares Sacchi, Claudia Regina Gonçalves, Erica Valessa Ramos Gomes, Karoline Rodrigues Campos
EPI_ISL_547966, EPI_ISL_547967, EPI_ISL_547968	The National Institute of Public Health	State Veterinary Institute Prague	Nagy,A;Jirincova,H;Novakova,L;Trnka,D;Vecerova,J
EPI_ISL_560408, EPI_ISL_560409, EPI_ISL_560410, EPI_ISL_560411, EPI_ISL_560412	The National Institute of Public Health	State Veterinary Institute Prague	Nagy, A; Jirincova, H; Novakova, L; Trnka, D; Vecerova, J
EPI_ISL_560587, EPI_ISL_560588, EPI_ISL_560589, EPI_ISL_560590	Hopital	National Reference Center for Viruses of Respiratory Infections, Institut Pasteur, Paris	Sylvie Behillil, Fabiana Gambaro, Etienne Simon-Lorière, Vincent Enouf, Maud Vanpeene, Sylvie van der Werf
EPI_ISL_560598, EPI_ISL_560599, EPI_ISL_560600, EPI_ISL_560601, EPI_ISL_560602, EPI_ISL_560603, EPI_ISL_560604, EPI_ISL_560605, EPI_ISL_560606, EPI_ISL_560607, EPI_ISL_560608, EPI_ISL_560609, EPI_ISL_560610, EPI_ISL_560611, EPI_ISL_560612, EPI_ISL_560613, EPI_ISL_560614, EPI_ISL_560615, EPI_ISL_560616, EPI_ISL_560617, EPI_ISL_560618, EPI_ISL_560636			
see above	Hospital	National Reference Center for Viruses of Respiratory Infections, Institut Pasteur, Paris	Sylvie Behillil, Fabiana Gambaro, Etienne Simon-Lorière, Vincent Enouf, Maud Vanpeene, Sylvie van der Werf
EPI_ISL_560637, EPI_ISL_560638, EPI_ISL_560639, EPI_ISL_560640, EPI_ISL_560641, EPI_ISL_560642	Labo Analyses Med	National Reference Center for Viruses of Respiratory Infections, Institut Pasteur, Paris	Sylvie Behillil, Fabiana Gambaro, Etienne Simon-Lorière, Vincent Enouf, Maud Vanpeene, Sylvie van der Werf
EPI_ISL_560643, EPI_ISL_560645, EPI_ISL_560646	Hospital	National Reference Center for Viruses of Respiratory Infections, Institut Pasteur, Paris	Sylvie Behillil, Fabiana Gambaro, Etienne Simon-Lorière, Vincent Enouf, Maud Vanpeene, Sylvie van der Werf
EPI_ISL_566077, EPI_ISL_566078, EPI_ISL_566079, EPI_ISL_566080, EPI_ISL_566081, EPI_ISL_566082, EPI_ISL_566083	Pathogenic Microorganisms Variability Laboratory	WHO National Influenza Centre Russian Federation	Andrey Komissarov, Artem Fadeev, Anna Ivanova, Kseniya Komissarova, Daria Danilenko, Dmitry Lioznov, Nadezhda Kuznetsova, Elena Shidlovskaya, Elizaveta Divisenko, Ekaterina Milashenko, Kirill Krasnoslobotsev, Evgeniya Mukasheva, Anna Ignatieva, Svetlana Trushakova, Alexey Shchetinin, Maria Nikiforova, Andrey Pochtovyy, Valeria Bacalin, Evgeny Usachev, Olga Burgasova, Ludmila Kolobukhina, Svetlana Smetanina, Elena Burtseva, Artem Tkachuk, Vladimir Gushchin, Alexander Gintsburg
EPI_ISL_568689	RS Pondok Indah Puri Indah	Eijkman Institute for Molecular Biology, Ministry of Research and Technology/National Agency for Research and Innovation	Frilasita A Yudhaputri, Edison Johar, Hidayat Trimarsanto, Iskandar A Adnan, Willy Agustine, David H Muljono, Safarina G Malik, Herawati Sudoyo, Khin Saw Myint, Amin Soebandrio
EPI_ISL_568691	RSUP Fatmawati	Eijkman Institute for Molecular Biology, Ministry of Research and Technology/National Agency for Research and Innovation	Frilasita A Yudhaputri, Edison Johar, Hidayat Trimarsanto, Iskandar A Adnan, Willy Agustine, David H Muljono, Safarina G Malik, Herawati Sudoyo, Khin Saw Myint, Amin Soebandrio

EPI_ISL_568692	RS Kramat 128	Eijkman Institute for Molecular Biology, Ministry of Research and Technology/National Agency for Research and Innovation	Frilasita A Yudhaputri, Edison Johar, Hidayat Trimarsanto, Iskandar A Adnan, Willy Agustine, David H Muljono, Safarina G Malik, Herawati Sudoyo, Khin Saw Myint, Amin Soebandrio
EPI_ISL_572331, EPI_ISL_572397	Institute for Virology, University Hospital Duesseldorf, Medical Faculty, Heinrich-Heine-University Duesseldorf	Institute for Virology, University Hospital Duesseldorf, Medical Faculty, Heinrich-Heine-University Duesseldorf	Maximilian Damagnez, Verena Keitel, Björn Jensen, Nadine Lübke, Lisa Müller, Philipp Ostermann, Tina Senff, Ortwin Adams, Philipp Albrecht, Gerald Antoch, Johannes Bode, Edwin Bölke, Saskia Elben, Torsten Feldt, Johannes C. Fischer, , Anselm Kunstein, Caroline Klindt, Alexander Killer, Tom Lüdde, Annemarie Mohring, Jennifer Neubert, Heiner Schaal, Ansgar Schulz, Jörg Timm, Andreas Walker
EPI_ISL_572398	Laboratory of Biology and Identification of Arboviruses	Pathogenic Microorganisms Variability Laboratory	Alexey Shchetinin, Maria Nikiforova, Andrei Siniavin, Victor Larichev, Alina Kozlova, Muhammad Saifullin, Alexey Prilipov, Vladimir Gushchin, Alexander Gintsburg
EPI_ISL_573152, EPI_ISL_574259	Institute for Virology, University Hospital Duesseldorf, Medical Faculty, Heinrich-Heine-University Duesseldorf	Institute for Virology, University Hospital Duesseldorf, Medical Faculty, Heinrich-Heine-University Duesseldorf	Maximilian Damagnez, Verena Keitel, Björn Jensen, Nadine Lübke, Lisa Müller, Philipp Ostermann, Tina Senff, Ortwin Adams, Philipp Albrecht, Gerald Antoch, Johannes Bode, Edwin Bölke, Saskia Elben, Torsten Feldt, Johannes C. Fischer, , Anselm Kunstein, Caroline Klindt, Alexander Killer, Tom Lüdde, Annemarie Mohring, Jennifer Neubert, Heiner Schaal, Ansgar Schulz, Jörg Timm, Andreas Walker
EPI_ISL_574594	Hospital Escola da Universidade de Taubate	Instituto Adolfo Lutz, Interdiciplinary Procedures Center, Strategic Laboratory	Claudio Tavares Sacchi, Claudia Regina Gonçalves, Erica Valessa Ramos Gomes, Karoline Rodrigues Campos
EPI_ISL_574596	CS II Dr. Antonio Vicoso Moreira de Rezende Sumare	Instituto Adolfo Lutz, Interdiciplinary Procedures Center, Strategic Laboratory	Claudio Tavares Sacchi, Claudia Regina Gonçalves, Erica Valessa Ramos Gomes, Karoline Rodrigues Campos
EPI_ISL_574597	Secretaria Municipal de Saude de Jarinu	Instituto Adolfo Lutz, Interdiciplinary Procedures Center, Strategic Laboratory	Claudio Tavares Sacchi, Claudia Regina Gonçalves, Erica Valessa Ramos Gomes, Karoline Rodrigues Campos
EPI_ISL_574598	Servico de Verificacao de Obito SVO	Instituto Adolfo Lutz, Interdiciplinary Procedures Center, Strategic Laboratory	Claudio Tavares Sacchi, Claudia Regina Gonçalves, Erica Valessa Ramos Gomes, Karoline Rodrigues Campos
EPI_ISL_574612	RS Hermina Mekarsari	Eijkman Institute for Molecular Biology, Ministry of Research and Technology/National Agency for Research and Innovation	Frilasita A Yudhaputri, Edison Johar, Hidayat Trimarsanto, Iskandar A Adnan, Willy Agustine, David H Muljono, Safarina G Malik, Herawati Sudoyo, Khin Saw Myint, Amin Soebandrio
EPI_ISL_574614	RS Kramat 128	Eijkman Institute for Molecular Biology, Ministry of Research and Technology/National Agency for Research and Innovation	Frilasita A Yudhaputri, Edison Johar, Hidayat Trimarsanto, Iskandar A Adnan, Willy Agustine, David H Muljono, Safarina G Malik, Herawati Sudoyo, Khin Saw Myint, Amin Soebandrio
EPI_ISL_574615	RS Harapan Bunda	Eijkman Institute for Molecular Biology, Ministry of Research and Technology/National Agency for Research and Innovation	Frilasita A Yudhaputri, Edison Johar, Hidayat Trimarsanto, Iskandar A Adnan, Willy Agustine, David H Muljono, Safarina G Malik, Herawati Sudoyo, Khin Saw Myint, Amin Soebandrio
EPI_ISL_574616, EPI_ISL_574617	RS Kramat 128	Eijkman Institute for Molecular Biology, Ministry of Research and Technology/National Agency for Research and Innovation	Frilasita A Yudhaputri, Edison Johar, Hidayat Trimarsanto, Iskandar A Adnan, Willy Agustine, David H Muljono, Safarina G Malik, Herawati Sudoyo, Khin Saw Myint, Amin Soebandrio
EPI_ISL_574618	RS Kramat 128	Eijkman Institute for Molecular Biology, Ministry of Research and Technology/National Agency for Research and Innovation	Hidayat Trimarsanto, Frilasita A Yudhaputri, Edison Johar, Iskandar A Adnan, Willy Agustine, David H Muljono, Safarina G Malik, Herawati Sudoyo, Khin Saw Myint, Amin Soebandrio
EPI_ISL_574619, EPI_ISL_574620, EPI_ISL_574621, EPI_ISL_574622, EPI_ISL_574623	BTKLPP Kelas I Manado	Eijkman Institute for Molecular Biology, Ministry of Research and Technology/National Agency for Research and Innovation	Hidayat Trimarsanto, Frilasita A Yudhaputri, Edison Johar, Iskandar A Adnan, Willy Agustine, David H Muljono, Safarina G Malik, Herawati Sudoyo, Khin Saw Myint, Amin Soebandrio
EPI_ISL_575331	RSUD Wates	Genetics Working Group (Pokja Genetik) Faculty of Medicine, Public Health and Nursing Universitas Gadjah Mada (FK-KMK UGM); Disease Investigation Center Wates Ministry of Agriculture Indonesia; Department of Microbiology FK-KMK UGM; Laboratorium Diagnostik Yayasan Tahija World Mosquito Program (WMP) Yogyakarta Center for Tropical Medicine FK-KMK UGM; Integrated Research Center FK-KMK UGM; Department of Computer Science and Electronics FMIPA UGM	Gunadi, Hendra Wibawa, . Marcellus, Mohamad S. Hakim, Edwin W. Daniwijaya, Ludhang P. Rizki, Endah Supriyati, Eggi Arguni, Titik Nuryastuti, Tri Wibawa, Dwi AA Nugrahaningsih, Afiahayati, Siswanto, Kristy Iskandar, Nungki Anggorowati, Bastianto Kusumajaya, Zumrati Ahmad, Alvin S. Kalim, Susan Simanjaya
EPI_ISL_576113	RSUP Dr. Sardjito	Genetics Working Group (Pokja Genetik) Faculty of Medicine, Public Health and Nursing Universitas Gadjah Mada (FK-KMK UGM); Disease Investigation Center Wates Ministry of Agriculture Indonesia; Department of Microbiology FK-KMK UGM; Laboratorium Diagnostik Yayasan Tahija World Mosquito Program (WMP) Yogyakarta Center for Tropical Medicine FK-KMK UGM; Integrated Research Center FK-KMK UGM; Department of Computer Science and Electronics FMIPA UGM	Gunadi, Hendra Wibawa, . Marcellus, Mohamad S. Hakim, Edwin W. Daniwijaya, Ludhang P. Rizki, Endah Supriyati, Eggi Arguni, Titik Nuryastuti, Tri Wibawa, Dwi AA Nugrahaningsih, Afiahayati, Siswanto, Kristy Iskandar, Nungki Anggorowati, Ika Trisnawati, Riat El Khair, William Widitjiarso, Fadil Fahri
EPI_ISL_576114	RSUP Dr. Sardjito	Genetics Working Group (Pokja Genetik) Faculty of Medicine, Public Health and Nursing Universitas Gadjah Mada (FK-KMK UGM); Disease Investigation Center Wates Ministry of Agriculture Indonesia; Department of Microbiology FK-KMK UGM; Laboratorium Diagnostik Yayasan Tahija World Mosquito Program (WMP) Yogyakarta Center for Tropical Medicine FK-KMK UGM; Integrated Research Center FK-KMK UGM; Department of Computer Science and Electronics FMIPA UGM	Gunadi, Hendra Wibawa, . Marcellus, Mohamad S. Hakim, Edwin W. Daniwijaya, Ludhang P. Rizki, Endah Supriyati, Eggi Arguni, Titik Nuryastuti, Tri Wibawa, Dwi AA Nugrahaningsih, Afiahayati, Siswanto, Kristy Iskandar, Nungki Anggorowati, Ika Trisnawati, Riat El Khair, Audric Kenny Tedja, Aditya Rifqi Fauzi
EPI_ISL_576115	RSUP Dr Sardjito	Genetics Working Group (Pokja Genetik) Faculty of Medicine, Public Health and Nursing Universitas Gadjah Mada (FK-KMK UGM); Disease Investigation Center Wates Ministry of Agriculture Indonesia; Department of Microbiology FK-KMK UGM; Laboratorium Diagnostik Yayasan Tahija World Mosquito Program (WMP) Yogyakarta Center for Tropical Medicine FK-KMK UGM; Integrated Research Center FK-KMK UGM; Department of Computer Science and Electronics FMIPA UGM	Gunadi, Hendra Wibawa, . Marcellus, Mohamad S. Hakim, Edwin W. Daniwijaya, Ludhang P. Rizki, Endah Supriyati, Eggi Arguni, Titik Nuryastuti, Tri Wibawa, Dwi AA Nugrahaningsih, Afiahayati, Siswanto, Kristy Iskandar, Nungki Anggorowati, Ika Trisnawati, Riat El Khair, Yunika Puspadewi, Osman Sianipar, Umi Solekhah Intansari, Elizabeth Henny Herningtiyas, Ira Puspitawati, Nur Imma Fatimah Harahap, Untung Wirawan, Maria Patricia Inggriani
EPI_ISL_576116	RSUP Dr. Sardjito	Genetics Working Group (Pokja Genetik) Faculty of Medicine, Public Health and Nursing Universitas Gadjah Mada (FK-KMK UGM); Disease Investigation Center Wates Ministry of Agriculture Indonesia; Department of Microbiology FK-KMK UGM; Laboratorium Diagnostik Yayasan Tahija	Gunadi, Hendra Wibawa, . Marcellus, Mohamad S. Hakim, Edwin W. Daniwijaya, Ludhang P. Rizki, Endah Supriyati, Eggi Arguni, Titik Nuryastuti, Tri Wibawa, Dwi AA Nugrahaningsih, Afiahayati, Siswanto, Kristy Iskandar, Nungki Anggorowati, Ika Trisnawati, Sumardi, Eko Budiono, Bambang Sigit Riyanto, Heni Retnowulan, Munawar Gani, Satria Maulana, Nur Rahmi Ananda, Riat El Khair, Yunika Puspadewi, Osman Sianipar, Umi Solekhah Intansari, Elizabeth Henny Herningtiyas, Ira Puspitawati, Nur Imma Fatimah Harahap, Ririn Enggy Yuliyanti, Alvin Santoso Kalim

		World Mosquito Program (WMP) Yogyakarta Center for Tropical Medicine FK-KMK UGM; Integrated Research Center FK-KMK UGM; Department of Computer Science and Electronics FMIPA UGM	
EPI_ISL_576128	RSUP Dr. Sardjito	Genetics Working Group (Pokja Genetik) Faculty of Medicine, Public Health and Nursing Universitas Gadjah Mada (FK-KMK UGM); Disease Investigation Center Wates Ministry of Agriculture Indonesia; Department of Microbiology FK-KMK UGM; Laboratorium Diagnostik Yayasan Tahija World Mosquito Program (WMP) Yogyakarta Center for Tropical Medicine FK-KMK UGM; Integrated Research Center FK-KMK UGM; Department of Computer Science and Electronics FMIPA UGM	Gunadi, Hendra Wibawa, Marcellus, Mohamad S. Hakim, Edwin W. Daniwijaya, Ludhang P. Rizki, Endah Supriyati, Eggi Arguni, Titik Nuryastuti, Tri Wibawa, Dwi AA Nugrahaningsih, Afiahayati, Siswanto, Kristy Iskandar, Nungki Anggorowati, Ika Trisnawati, Sumardi, Eko Budiono, Bambang Sigit Riyanto, Heni Retnowulan, Munawar Gani, Satria Maulana, Nur Rahmi Ananda, Riat El Khair, Yunika Puspadewi, Osman Sianipar, Umi Solekhah Intansari, Elizabeth Henny Herningtiyas, Ira Puspitawati, Nur Imma Fatimah Harahap, Dyah Ayu Puspitarani, Kemala Athollah
EPI_ISL_576130	RSUP Dr. Sardjito	Genetics Working Group (Pokja Genetik) Faculty of Medicine, Public Health and Nursing Universitas Gadjah Mada (FK-KMK UGM); Disease Investigation Center Wates Ministry of Agriculture Indonesia; Department of Microbiology FK-KMK UGM; Laboratorium Diagnostik Yayasan Tahija World Mosquito Program (WMP) Yogyakarta Center for Tropical Medicine FK-KMK UGM; Integrated Research Center FK-KMK UGM; Department of Computer Science and Electronics FMIPA UGM	Gunadi, Hendra Wibawa, Marcellus, Mohamad S. Hakim, Edwin W. Daniwijaya, Ludhang P. Rizki, Endah Supriyati, Eggi Arguni, Titik Nuryastuti, Tri Wibawa, Dwi AA Nugrahaningsih, Afiahayati, Siswanto, Kristy Iskandar, Nungki Anggorowati, Rina Triasih, Amalia Setyati, Dwikisworo Setyowireni, Ida Safitri Laksanawati, Cahya Dewi Satria, Titis Widowati, Elisabeth Siti Herini, Riat El Khair, Yunika Puspadewi, Osman Sianipar, Umi Solekhah Intansari, Elizabeth Henny Herningtiyas, Ira Puspitawati, Nur Imma Fatimah Harahap, Alvin S. Kalim, Susan Simanjaya, Desyifa Mursalin
EPI_ISL_576145	RSA Universitas Gadjah Mada	Genetics Working Group (Pokja Genetik) Faculty of Medicine, Public Health and Nursing Universitas Gadjah Mada (FK-KMK UGM); Disease Investigation Center Wates Ministry of Agriculture Indonesia; Department of Microbiology FK-KMK UGM; Laboratorium Diagnostik Yayasan Tahija World Mosquito Program (WMP) Yogyakarta Center for Tropical Medicine FK-KMK UGM; Integrated Research Center FK-KMK UGM; Department of Computer Science and Electronics FMIPA UGM	Gunadi, Hendra Wibawa, Marcellus, Mohamad S. Hakim, Edwin W. Daniwijaya, Ludhang P. Rizki, Endah Supriyati, Eggi Arguni, Titik Nuryastuti, Tri Wibawa, Dwi AA Nugrahaningsih, Afiahayati, Siswanto, Kristy Iskandar, Nungki Anggorowati, William Widitjiarso, Fadil Fahri
EPI_ISL_576146, EPI_ISL_576147, EPI_ISL_576148	Department of Respiratory & Other Viral Infections of L.V. Gromashevsky Institute of Epidemiology & Infectious Diseases NAMS of Ukraine	Department of Respiratory & Other Viral Infections of L.V. Gromashevsky Institute of Epidemiology & Infectious Diseases NAMS of Ukraine, JSC "Farmak"	Alla Mironenko, Ihor Kravchuk, Liudmyla Bolotova, Larysa Radchenko, Nataliia Teteriuk
EPI_ISL_576149	Department of Respiratory & Other Viral Infections of L.V. Gromashevsky Institute of Epidemiology & Infectious Diseases NAMS of Ukraine	Department of Respiratory & Other Viral Infections of L.V. Gromashevsky Institute of Epidemiology & Infectious Diseases NAMS of Ukraine, JSC "Farmak"	Alla Mironenko, Andriy Goy, Ihor Kravchuk, Liudmyla Bolotova, Larysa Radchenko, Nataliia Teteriuk
EPI_ISL_576258, EPI_ISL_576259, EPI_ISL_576260	Instituto de Diagnostico y Referencia Epidemiologicos (INDRE)	Instituto de Diagnostico y Referencia Epidemiologicos (INDRE)	Gisela Barrera-Badillo , Abril Rodriguez-Maldonado, Claudia Wong-Arambula , Natividad Cruz-Ortiz, Tatiana Nunez-Garcia, Dayanira Arellano-Suarez, Fabiola Garces-Ayala, Edgar Mendieta-Condado, Lucia Hernandez-Rivas, Irma Lopez-Martinez, Ernesto Ramirez-Gonzalez.
EPI_ISL_576261, EPI_ISL_576262, EPI_ISL_576263	Instituto de Diagnostico y Referencia Epidemiologicos (INDRE)	Instituto de Diagnostico y Referencia Epidemiologicos (INDRE)	Gisela Barrera-Badillo , Abril Rodriguez-Maldonado, Claudia Wong-Arambula , Natividad Cruz-Ortiz, Tatiana Nunez-Garcia, Dayanira Arellano-Suarez, Adnan Araiza-Rodriguez, Edgar Mendieta-Condado, Lucia Hernandez-Rivas, Irma Lopez-Martinez, Ernesto Ramirez-Gonzalez.
EPI_ISL_576264, EPI_ISL_576265	Instituto de Diagnostico y Referencia Epidemiologicos (INDRE)	Instituto de Diagnostico y Referencia Epidemiologicos (INDRE)	Ernesto Ramirez-Gonzalez, Abril Rodriguez-Maldonado, Claudia Wong-Arambula , Natividad Cruz-Ortiz, Tatiana Nunez-Garcia, Dayanira Arellano-Suarez, Adnan Araiza-Rodriguez, Edgar Mendieta-Condado, Lucia Hernandez-Rivas, Irma Lopez-Martinez, Gisela Barrera-Badillo.
EPI_ISL_576266, EPI_ISL_576267, EPI_ISL_576268, EPI_ISL_576269, EPI_ISL_576270, EPI_ISL_576271	Instituto de Diagnostico y Referencia Epidemiologicos (INDRE)	Instituto de Diagnostico y Referencia Epidemiologicos (INDRE)	Ernesto Ramirez-Gonzalez, Abril Rodriguez-Maldonado, Claudia Wong-Arambula , Natividad Cruz-Ortiz, Tatiana Nunez-Garcia, Dayanira Arellano-Suarez, Adnan Araiza-Rodriguez, Fabiola Garces-Ayala, Lucia Hernandez-Rivas, Irma Lopez-Martinez, Gisela Barrera-Badillo.
EPI_ISL_576272	Instituto de Diagnostico y Referencia Epidemiologicos (INDRE)	Instituto de Diagnostico y Referencia Epidemiologicos (INDRE)	Gisela Barrera-Badillo , Abril Rodriguez-Maldonado, Claudia Wong-Arambula , Natividad Cruz-Ortiz, Tatiana Nunez-Garcia, Dayanira Arellano-Suarez, Fabiola Garces-Ayala, Edgar Mendieta-Condado, Lucia Hernandez-Rivas, Irma Lopez-Martinez, Ernesto Ramirez-Gonzalez.
EPI_ISL_576273	Instituto de Diagnostico y Referencia Epidemiologicos (INDRE)	Instituto de Diagnostico y Referencia Epidemiologicos (INDRE)	Ernesto Ramirez-Gonzalez, Abril Rodriguez-Maldonado, Claudia Wong-Arambula , Natividad Cruz-Ortiz, Tatiana Nunez-Garcia, Dayanira Arellano-Suarez, Adnan Araiza-Rodriguez, Fabiola Garces-Ayala, Lucia Hernandez-Rivas, Irma Lopez-Martinez, Gisela Barrera-Badillo.
EPI_ISL_576274, EPI_ISL_576275, EPI_ISL_576276	Instituto de Diagnostico y Referencia Epidemiologicos (INDRE)	Instituto de Diagnostico y Referencia Epidemiologicos (INDRE)	Gisela Barrera-Badillo , Abril Rodriguez-Maldonado, Claudia Wong-Arambula , Natividad Cruz-Ortiz, Tatiana Nunez-Garcia, Dayanira Arellano-Suarez, Adnan Araiza-Rodriguez, Edgar Mendieta-Condado, Lucia Hernandez-Rivas, Irma Lopez-Martinez, Ernesto Ramirez-Gonzalez.
EPI_ISL_576277, EPI_ISL_576278	Instituto de Diagnostico y Referencia Epidemiologicos (INDRE)	Instituto de Diagnostico y Referencia Epidemiologicos (INDRE)	Ernesto Ramirez-Gonzalez, Abril Rodriguez-Maldonado, Claudia Wong-Arambula , Natividad Cruz-Ortiz, Tatiana Nunez-Garcia, Dayanira Arellano-Suarez, Adnan Araiza-Rodriguez, Edgar Mendieta-Condado, Lucia Hernandez-Rivas, Irma Lopez-Martinez, Gisela Barrera-Badillo.
EPI_ISL_576279	Instituto de Diagnostico y Referencia Epidemiologicos (INDRE)	Instituto de Diagnostico y Referencia Epidemiologicos (INDRE)	Ernesto Ramirez-Gonzalez, Abril Rodriguez-Maldonado, Claudia Wong-Arambula , Natividad Cruz-Ortiz, Tatiana Nunez-Garcia, Dayanira Arellano-Suarez, Adnan Araiza-Rodriguez, Fabiola Garces-Ayala, Lucia Hernandez-Rivas, Irma Lopez-Martinez, Gisela Barrera-Badillo.
EPI_ISL_577624, EPI_ISL_577625, EPI_ISL_577626, EPI_ISL_577627, EPI_ISL_577628, EPI_ISL_577632, EPI_ISL_577633, EPI_ISL_577634, EPI_ISL_577635, EPI_ISL_577636, EPI_ISL_577637, EPI_ISL_577638			
see above	The National Institute of Public Health	State Veterinary Institute Prague	Nagy,A;Jirincova,H;Novakova,L;Trnka,D;Vecerova,J
EPI_ISL_578187, EPI_ISL_578188	Hospital Virgen de las Nieves	Instituto de Salud Carlos III	Iglesias-Caballero, M. Molinero Calamita, M. González-Esguevillas, M. Camarero, S. Pozo, F. Casas, I. Jiménez, P. Jiménez, M. Zaballos, A. Monzón, S. Varona, S. Juliá, M. Cuesta, I, J.M Navarro
EPI_ISL_582030	Biology Department, College of Science, Al-Muthanna University	International Centre for Genetic Engineering and Biotechnology (ICGEB) and ARGO Open Lab Platform	Nihad Al-Rashedi, Danilo Licastro, Sreejith Rajasekharan, Simeone Dal Monego, Alessandro Marcello
EPI_ISL_582124	Malaysia Genome Institute	Malaysia Genome Institute	Mohd Noor Mat Isa, Irni Suhayu Sapian, Yusuf Muhammad Noor, Nurhezreen Md Iqbal, Mohd Faizal Abu Bakar, Enizza Kasim, Shamsidar Sopie, Siti Noraini Othman, Azrin Ahmad, Nor Azfa Johari, Shahrul Hisham Zainal Ariffin
EPI_ISL_582509	Department of Respiratory and other Viral Infections of L.V.Gromashevsky Institute of Epidemiology & Infectious Diseases NAMS of Ukraine	Department of Respiratory and other Viral Infections of L.V.Gromashevsky Institute of Epidemiology & Infectious Diseases NAMS of Ukraine, JSC "Farmak"	Alla Mironenko, Andriy Goy, Ihor Kravchuk, Ludmyla Bolotova, Larysa Radchenko, Nataliia Teteriuk
EPI_ISL_582510	Department of Respiratory and other Viral Infections of L.V.Gromashevsky Institute of Epidemiology & Infectious Diseases NAMS of Ukraine	Department of Respiratory and other Viral Infections of L.V.Gromashevsky Institute of Epidemiology & Infectious Diseases NAMS of Ukrain, JSC "Farmak"	Alla Mironenko, Andriy Goy, Ihor Kravchuk, Ludmyla Bolotova, Larysa Radchenko, Nataliia Teteriuk
EPI_ISL_582511, EPI_ISL_582512, EPI_ISL_582513	Department of Respiratory and other Viral Infections of L.V.Gromashevsky Institute of Epidemiology & Infectious Diseases NAMS of Ukrain	Department of Respiratory and other Viral Infections of L.V.Gromashevsky Institute of Epidemiology & Infectious Diseases NAMS of Ukrain, JSC "Farmak"	Alla Mironenko, Andriy Goy, Ihor Kravchuk, Ludmyla Bolotova, Larysa Radchenko, Nataliia Teteriuk
EPI_ISL_582642, EPI_ISL_582643, EPI_ISL_582644, EPI_ISL_582645, EPI_ISL_582646, EPI_ISL_582647, EPI_ISL_582648, EPI_ISL_582649, EPI_ISL_582650, EPI_ISL_582651, EPI_ISL_582652, EPI_ISL_582653, EPI_ISL_582657			
see above	Sheikh Khalifa Medical City	Molecular/Surveillance lab Sheikh Khalifa Medical City	Amirtharaj Francis, Sajeed Abdul, Hala Imambaccus, Sahar Almarzooqi, Hiba Saud, Stefan Weber

EPI_ISL_583443, EPI_ISL_583449, EPI_ISL_583450, EPI_ISL_583451	Memorial Sloan Kettering Cancer Center	van Bakel Laboratory, Genetics and Genomics Sciences, Icahn School of Medicine at Mount Sinai	Teresa Aydillo, Ana S. Gonzalez-Reiche, Sadaf Aslam, Adriana van de Guchte, Zenab Khan, Ajay Obla, Jayeeta Dutta, Harm van Bakel, Judith Aberg, Adolfo García-Sastre, Gunjan Shah, Tobias Hohl, Genovefa Papanicolaou, Miguel-Angel Perales, Kent Sepkowitz, Ngolela Esther Babady, and Mini Kamboj
EPI_ISL_583452, EPI_ISL_583453, EPI_ISL_583454, EPI_ISL_583473	Garcia-Sastre Laboratory, Department of Microbiology, Icahn School of Medicine at Mount Sinai	van Bakel Laboratory, Genetics and Genomics Sciences, Icahn School of Medicine at Mount Sinai	Teresa Aydillo, Ana S. Gonzalez-Reiche, Sadaf Aslam, Adriana van de Guchte, Zenab Khan, Ajay Obla, Jayeeta Dutta, Harm van Bakel, Judith Aberg, Adolfo García-Sastre, Gunjan Shah, Tobias Hohl, Genovefa Papanicolaou, Miguel-Angel Perales, Kent Sepkowitz, Ngolela Esther Babady, and Mini Kamboj
EPI_ISL_583476	Memorial Sloan Kettering Cancer Center	van Bakel Laboratory, Genetics and Genomics Sciences, Icahn School of Medicine at Mount Sinai	Teresa Aydillo, Ana S. Gonzalez-Reiche, Sadaf Aslam, Adriana van de Guchte, Zenab Khan, Ajay Obla, Jayeeta Dutta, Harm van Bakel, Judith Aberg, Adolfo García-Sastre, Gunjan Shah, Tobias Hohl, Genovefa Papanicolaou, Miguel-Angel Perales, Kent Sepkowitz, Ngolela Esther Babady, and Mini Kamboj
EPI_ISL_583477	Garcia-Sastre Laboratory, Department of Microbiology, Icahn School of Medicine at Mount Sinai	van Bakel Laboratory, Genetics and Genomics Sciences, Icahn School of Medicine at Mount Sinai	Teresa Aydillo, Ana S. Gonzalez-Reiche, Sadaf Aslam, Adriana van de Guchte, Zenab Khan, Ajay Obla, Jayeeta Dutta, Harm van Bakel, Judith Aberg, Adolfo García-Sastre, Gunjan Shah, Tobias Hohl, Genovefa Papanicolaou, Miguel-Angel Perales, Kent Sepkowitz, Ngolela Esther Babady, and Mini Kamboj
EPI_ISL_583495	Serviço de Verificação de Óbitos SVO Guaruulhos	Instituto Adolfo Lutz, Interdiciplinary Procedures Center, Strategic Laboratory	Claudio Tavares Sacchi, Claudia Regina Gonçalves, Erica Valessa Ramos Gomes, Karoline Rodrigues Campos
EPI_ISL_583496	UPA Jandira	Instituto Adolfo Lutz, Interdiciplinary Procedures Center, Strategic Laboratory	Claudio Tavares Sacchi, Claudia Regina Gonçalves, Erica Valessa Ramos Gomes, Karoline Rodrigues Campos
EPI_ISL_583497	Complexo Hospitalar Ouro Verde de Campinas	Instituto Adolfo Lutz, Interdiciplinary Procedures Center, Strategic Laboratory	Claudio Tavares Sacchi, Claudia Regina Gonçalves, Erica Valessa Ramos Gomes, Karoline Rodrigues Campos
EPI_ISL_583498	Hospital Municipal Dr. Waldemar Tebaldi	Instituto Adolfo Lutz, Interdiciplinary Procedures Center, Strategic Laboratory	Claudio Tavares Sacchi, Claudia Regina Gonçalves, Erica Valessa Ramos Gomes, Karoline Rodrigues Campos
EPI_ISL_583499	Distrito Sanitario Sul Campinas	Instituto Adolfo Lutz, Interdiciplinary Procedures Center, Strategic Laboratory	Claudio Tavares Sacchi, Claudia Regina Gonçalves, Erica Valessa Ramos Gomes, Karoline Rodrigues Campos
EPI_ISL_583500	Centro de Saude I Tacito Leite de Carvalho e Silva	Instituto Adolfo Lutz, Interdiciplinary Procedures Center, Strategic Laboratory	Claudio Tavares Sacchi, Claudia Regina Gonçalves, Erica Valessa Ramos Gomes, Karoline Rodrigues Campos
EPI_ISL_583501	Hospital Estadual de CampanhaCOVID 19 Barradas	Instituto Adolfo Lutz, Interdiciplinary Procedures Center, Strategic Laboratory	Claudio Tavares Sacchi, Claudia Regina Gonçalves, Erica Valessa Ramos Gomes, Karoline Rodrigues Campos
EPI_ISL_583502	Serv de Vig Sanitaria Epidemio e CTRL de Zoonoses Guaruja	Instituto Adolfo Lutz, Interdiciplinary Procedures Center, Strategic Laboratory	Claudio Tavares Sacchi, Claudia Regina Gonçalves, Erica Valessa Ramos Gomes, Karoline Rodrigues Campos
EPI_ISL_583503	CTA Centro de Testagem e Aconselhamento	Instituto Adolfo Lutz, Interdiciplinary Procedures Center, Strategic Laboratory	Claudio Tavares Sacchi, Claudia Regina Gonçalves, Erica Valessa Ramos Gomes, Karoline Rodrigues Campos
EPI_ISL_583504, EPI_ISL_583505	Casa de Saude Stella Maris	Instituto Adolfo Lutz, Interdiciplinary Procedures Center, Strategic Laboratory	Claudio Tavares Sacchi, Claudia Regina Gonçalves, Erica Valessa Ramos Gomes, Karoline Rodrigues Campos
EPI_ISL_583894, EPI_ISL_583895, EPI_ISL_583896, EPI_ISL_583897, EPI_ISL_583898, EPI_ISL_583899, EPI_ISL_583900	Singapore General Hospital	Department of Microbiology	Nurdyana Abdul Rahman, Kun Lee Lim, Chenhao Li, Sui Sin Goh, Kenneth Xin Long Chan, Kian Sing Chan, Lynette Oon, Kern Rei Chng, Niranjan Nagarajan, Karrie Ko
EPI_ISL_584074, EPI_ISL_584075, EPI_ISL_584076, EPI_ISL_584077, EPI_ISL_584078, EPI_ISL_584080	The National Institute of Public Health	State Veterinary Institute Prague	Nagy,A;Jirincova,H;Novakova,L;Trnka,D;Vecerova,J
EPI_ISL_586513	B.J. Medical College and Civil hospital, Ahmedabad	Gujarat Biotechnology Research Centre	Pinal Trivedi, Maharshi Pandya, Nidhi Patel, Nitin Savaliya, Raghawendra Kumar, Dinesh Kumar, Zuber Saiyed, Komal Patel, Labdhi Pandya, Afzal Ansari, Nikha Trivedi, Pranay Shah, Kamlesh J Upadhyay, Sanjay Kapadia, Apurvasinh Puvar, Janvi Raval, Zarna Patel, Monika Gandhi, R D Dixit, A M Kadri, Harsh Bakshi, Chaitanya Joshi, Madhvi Joshi
EPI_ISL_586514	B.J. Medical College and Civil hospital, Ahmedabad	Gujarat Biotechnology Research Centre	Maharshi Pandya, Nidhi Patel, Nitin Savaliya, Raghawendra Kumar, Dinesh Kumar, Zuber Saiyed, Komal Patel, Labdhi Pandya, Afzal Ansari, Nikha Trivedi, Pranay Shah, Kamlesh J Upadhyay, Sanjay Kapadia, Apurvasinh Puvar, Janvi Raval, Zarna Patel, Monika Gandhi, Pinal Trivedi, R D Dixit, A M Kadri, Harsh Bakshi, Chaitanya Joshi, Madhvi Joshi
EPI_ISL_586515	B.J. Medical College and Civil hospital, Ahmedabad	Gujarat Biotechnology Research Centre	Nidhi Patel, Nitin Savaliya, Raghawendra Kumar, Dinesh Kumar, Zuber Saiyed, Komal Patel, Labdhi Pandya, Afzal Ansari, Nikha Trivedi, Pranay Shah, Kamlesh J Upadhyay, Sanjay Kapadia, Apurvasinh Puvar, Janvi Raval, Zarna Patel, Monika Gandhi, Pinal Trivedi, Maharshi Pandya, R D Dixit, A M Kadri, Harsh Bakshi, Chaitanya Joshi, Madhvi Joshi
EPI_ISL_586516	B.J. Medical College and Civil hospital, Ahmedabad	Gujarat Biotechnology Research Centre	Nitin Savaliya, Raghawendra Kumar, Dinesh Kumar, Zuber Saiyed, Komal Patel, Labdhi Pandya, Afzal Ansari, Nikha Trivedi, Pranay Shah, Kamlesh J Upadhyay, Sanjay Kapadia, Apurvasinh Puvar, Janvi Raval, Zarna Patel, Monika Gandhi, Pinal Trivedi, Maharshi Pandya, Nidhi Patel, R D Dixit, A M Kadri, Harsh Bakshi, Chaitanya Joshi, Madhvi Joshi
EPI_ISL_586517	B.J. Medical College and Civil hospital, Ahmedabad	Gujarat Biotechnology Research Centre	Raghawendra Kumar, Dinesh Kumar, Zuber Saiyed, Komal Patel, Labdhi Pandya, Afzal Ansari, Nikha Trivedi, Pranay Shah, Kamlesh J Upadhyay, Sanjay Kapadia, Apurvasinh Puvar, Janvi Raval, Zarna Patel, Monika Gandhi, Pinal Trivedi, Maharshi Pandya, Nidhi Patel, Nitin Savaliya, R D Dixit, A M Kadri, Harsh Bakshi, Chaitanya Joshi, Madhvi Joshi
EPI_ISL_586518	B.J. Medical College and Civil hospital, Ahmedabad	Gujarat Biotechnology Research Centre	Dinesh Kumar, Zuber Saiyed, Komal Patel, Labdhi Pandya, Afzal Ansari, Nikha Trivedi, Pranay Shah, Kamlesh J Upadhyay, Sanjay Kapadia, Apurvasinh Puvar, Janvi Raval, Zarna Patel, Monika Gandhi, Pinal Trivedi, Maharshi Pandya, Nidhi Patel, Nitin Savaliya, Raghawendra Kumar, R D Dixit, A M Kadri, Harsh Bakshi, Chaitanya Joshi, Madhvi Joshi
EPI_ISL_586519	B.J. Medical College and Civil hospital, Ahmedabad	Gujarat Biotechnology Research Centre	Zuber Saiyed, Komal Patel, Labdhi Pandya, Afzal Ansari, Nikha Trivedi, Pranay Shah, Kamlesh J Upadhyay, Sanjay Kapadia, Apurvasinh Puvar, Janvi Raval, Zarna Patel, Monika Gandhi, Pinal Trivedi, Maharshi Pandya, Nidhi Patel, Nitin Savaliya, Raghawendra Kumar, Dinesh Kumar, R D Dixit, A M Kadri, Harsh Bakshi, Chaitanya Joshi, Madhvi Joshi
EPI_ISL_586520	B.J. Medical College and Civil hospital, Ahmedabad	Gujarat Biotechnology Research Centre	Komal Patel, Labdhi Pandya, Afzal Ansari, Nikha Trivedi, Pranay Shah, Kamlesh J Upadhyay, Sanjay Kapadia, Apurvasinh Puvar, Janvi Raval, Zarna Patel, Monika Gandhi, Pinal Trivedi, Maharshi Pandya, Nidhi Patel, Nitin Savaliya, Raghawendra Kumar, Dinesh Kumar, Zuber Saiyed, R D Dixit, A M Kadri, Harsh Bakshi, Chaitanya Joshi, Madhvi Joshi
EPI_ISL_586521	B.J. Medical College and Civil hospital, Ahmedabad	Gujarat Biotechnology Research Centre	Labdhi Pandya, Afzal Ansari, Nikha Trivedi, Pranay Shah, Kamlesh J Upadhyay, Sanjay Kapadia, Apurvasinh Puvar, Janvi Raval, Zarna Patel, Monika Gandhi, Pinal Trivedi, Maharshi Pandya, Nidhi Patel, Nitin Savaliya, Raghawendra Kumar, Dinesh Kumar, Zuber Saiyed, Komal Patel, R D Dixit, A M Kadri, Harsh Bakshi, Chaitanya Joshi, Madhvi Joshi
EPI_ISL_586522	B.J. Medical College and Civil hospital, Ahmedabad	Gujarat Biotechnology Research Centre	Afzal Ansari, Nikha Trivedi, Pranay Shah, Kamlesh J Upadhyay, Sanjay Kapadia, Apurvasinh Puvar, Janvi Raval, Zarna Patel, Monika Gandhi, Pinal Trivedi, Maharshi Pandya, Nidhi Patel, Nitin Savaliya, Raghawendra Kumar, Dinesh Kumar, Zuber Saiyed, Komal Patel, Labdhi Pandya, R D Dixit, A M Kadri, Harsh Bakshi, Chaitanya Joshi, Madhvi Joshi
EPI_ISL_586523	B.J. Medical College and Civil hospital, Ahmedabad	Gujarat Biotechnology Research Centre	Nikha Trivedi, Pranay Shah, Kamlesh J Upadhyay, Sanjay Kapadia, Apurvasinh Puvar, Janvi Raval, Zarna Patel, Monika Gandhi, Pinal Trivedi, Maharshi Pandya, Nidhi Patel, Nitin Savaliya, Raghawendra Kumar, Dinesh Kumar, Zuber Saiyed, Komal Patel, Labdhi Pandya, Afzal Ansari, R D Dixit, A M Kadri, Harsh Bakshi, Chaitanya Joshi, Madhvi Joshi
EPI_ISL_586524	B.J. Medical College and Civil hospital, Ahmedabad	Gujarat Biotechnology Research Centre	Pranay Shah, Kamlesh J Upadhyay, Sanjay Kapadia, Apurvasinh Puvar, Janvi Raval, Zarna Patel, Monika Gandhi, Pinal Trivedi, Maharshi Pandya, Nidhi Patel, Nitin Savaliya, Raghawendra Kumar, Dinesh Kumar, Zuber Saiyed, Komal Patel, Labdhi Pandya, Afzal Ansari, Nikha Trivedi, R D Dixit, A M Kadri, Harsh Bakshi, Chaitanya Joshi, Madhvi Joshi
EPI_ISL_586525	B.J. Medical College and Civil hospital, Ahmedabad	Gujarat Biotechnology Research Centre	Kamlesh J Upadhyay, Sanjay Kapadia, Apurvasinh Puvar, Janvi Raval, Zarna Patel, Monika Gandhi, Pinal Trivedi, Maharshi Pandya, Nidhi Patel, Nitin Savaliya, Raghawendra Kumar, Dinesh Kumar, Zuber Saiyed, Komal Patel, Labdhi Pandya, Afzal Ansari, Nikha Trivedi, Pranay Shah, R D Dixit, A M Kadri,

			Harsh Bakshi, Chaitanya Joshi, Madhvi Joshi
EPI_ISL_586526	B.J. Medical College and Civil hospital, Ahmedabad	Gujarat Biotechnology Research Centre	Sanjay Kapadia, Apurvasinh Puvar, Janvi Raval, Zarna Patel, Monika Gandhi, Pinal Trivedi, Maharshi Pandya, Nidhi Patel, Nitin Savaliya, Raghawendra Kumar, Dinesh Kumar, Zuber Saiyed, Komal Patel, Labdhi Pandya, Afzal Ansari, Nikha Trivedi, Pranay Shah, Kamlesh J Upadhyay, R D Dixit, A M Kadri, Harsh Bakshi, Chaitanya Joshi, Madhvi Joshi
EPI_ISL_586527	B.J. Medical College and Civil hospital, Ahmedabad	Gujarat Biotechnology Research Centre	Apurvasinh Puvar, Janvi Raval, Zarna Patel, Monika Gandhi, Pinal Trivedi, Maharshi Pandya, Nidhi Patel, Nitin Savaliya, Raghawendra Kumar, Dinesh Kumar, Zuber Saiyed, Komal Patel, Labdhi Pandya, Afzal Ansari, Nikha Trivedi, Pranay Shah, Kamlesh J Upadhyay, Sanjay Kapadia, R D Dixit, A M Kadri, Harsh Bakshi, Chaitanya Joshi, Madhvi Joshi
EPI_ISL_586528	B.J. Medical College and Civil hospital, Ahmedabad	Gujarat Biotechnology Research Centre	Zarna Patel, Monika Gandhi, Pinal Trivedi, Maharshi Pandya, Nidhi Patel, Nitin Savaliya, Raghawendra Kumar, Dinesh Kumar, Zuber Saiyed, Komal Patel, Labdhi Pandya, Afzal Ansari, Nikha Trivedi, Pranay Shah, Kamlesh J Upadhyay, Sanjay Kapadia, Apurvasinh Puvar, Janvi Raval, R D Dixit, A M Kadri, Harsh Bakshi, Chaitanya Joshi, Madhvi Joshi
EPI_ISL_586529	B.J. Medical College and Civil hospital, Ahmedabad	Gujarat Biotechnology Research Centre	Monika Gandhi, Pinal Trivedi, Maharshi Pandya, Nidhi Patel, Nitin Savaliya, Raghawendra Kumar, Dinesh Kumar, Zuber Saiyed, Komal Patel, Labdhi Pandya, Afzal Ansari, Nikha Trivedi, Pranay Shah, Kamlesh J Upadhyay, Sanjay Kapadia, Apurvasinh Puvar, Janvi Raval, Zarna Patel, R D Dixit, A M Kadri, Harsh Bakshi, Chaitanya Joshi, Madhvi Joshi
EPI_ISL_586530	B.J. Medical College and Civil hospital, Ahmedabad	Gujarat Biotechnology Research Centre	Pinal Trivedi, Maharshi Pandya, Nidhi Patel, Nitin Savaliya, Raghawendra Kumar, Dinesh Kumar, Zuber Saiyed, Komal Patel, Labdhi Pandya, Afzal Ansari, Nikha Trivedi, Pranay Shah, Kamlesh J Upadhyay, Sanjay Kapadia, Apurvasinh Puvar, Janvi Raval, Zarna Patel, Monika Gandhi, R D Dixit, A M Kadri, Harsh Bakshi, Chaitanya Joshi, Madhvi Joshi
EPI_ISL_586531	General Hospital,Chhotaudepur	Gujarat Biotechnology Research Centre	Yogesh Parmar, Arti Thakur, Dharak Pandya, Maharshi Pandya, Nidhi Patel, Nitin Savaliya, Raghawendra Kumar, Dinesh Kumar, Zuber Saiyed, Komal Patel, Labdhi Pandya, Afzal Ansari, Nikha Trivedi, Apurvasinh Puvar, Janvi Raval, Zarna Patel, Monika Gandhi, Pinal Trivedi, R D Dixit, A M Kadri, Harsh Bakshi, Chaitanya Joshi, Madhvi Joshi
EPI_ISL_586532	General Hospital,Chhotaudepur	Gujarat Biotechnology Research Centre	Arti Thakur, Dharak Pandya, Maharshi Pandya, Nidhi Patel, Nitin Savaliya, Raghawendra Kumar, Dinesh Kumar, Zuber Saiyed, Komal Patel, Labdhi Pandya, Afzal Ansari, Nikha Trivedi, Apurvasinh Puvar, Janvi Raval, Zarna Patel, Monika Gandhi, Pinal Trivedi, Yogesh Parmar, R D Dixit, A M Kadri, Harsh Bakshi, Chaitanya Joshi, Madhvi Joshi
EPI_ISL_586533	General Hospital,Chhotaudepur	Gujarat Biotechnology Research Centre	Dharak Pandya, Maharshi Pandya, Nidhi Patel, Nitin Savaliya, Raghawendra Kumar, Dinesh Kumar, Zuber Saiyed, Komal Patel, Labdhi Pandya, Afzal Ansari, Nikha Trivedi, Apurvasinh Puvar, Janvi Raval, Zarna Patel, Monika Gandhi, Pinal Trivedi, Yogesh Parmar, Arti Thakur, R D Dixit, A M Kadri, Harsh Bakshi, Chaitanya Joshi, Madhvi Joshi
EPI_ISL_586534	General Hospital,Chhotaudepur	Gujarat Biotechnology Research Centre	Maharshi Pandya, Nidhi Patel, Nitin Savaliya, Raghawendra Kumar, Dinesh Kumar, Zuber Saiyed, Komal Patel, Labdhi Pandya, Afzal Ansari, Nikha Trivedi, Apurvasinh Puvar, Janvi Raval, Zarna Patel, Monika Gandhi, Pinal Trivedi, Yogesh Parmar, Arti Thakur, Dharak Pandya, R D Dixit, A M Kadri, Harsh Bakshi, Chaitanya Joshi, Madhvi Joshi
EPI_ISL_586535	General Hospital,Chhotaudepur	Gujarat Biotechnology Research Centre	Nidhi Patel, Nitin Savaliya, Raghawendra Kumar, Dinesh Kumar, Zuber Saiyed, Komal Patel, Labdhi Pandya, Afzal Ansari, Nikha Trivedi, Apurvasinh Puvar, Janvi Raval, Zarna Patel, Monika Gandhi, Pinal Trivedi, Yogesh Parmar, Arti Thakur, Dharak Pandya, Maharshi Pandya, R D Dixit, A M Kadri, Harsh Bakshi, Chaitanya Joshi, Madhvi Joshi
EPI_ISL_586536	Zydus Medical Hospital,Dahod	Gujarat Biotechnology Research Centre	Kalpesh Labana, Mohit Desai, Nitin Savaliya, Raghawendra Kumar, Dinesh Kumar, Zuber Saiyed, Komal Patel, Labdhi Pandya, Afzal Ansari, Nikha Trivedi, Apurvasinh Puvar, Janvi Raval, Zarna Patel, Monika Gandhi, Pinal Trivedi, Maharshi Pandya, Nidhi Patel, R D Dixit, A M Kadri, Harsh Bakshi, Chaitanya Joshi, Madhvi Joshi
EPI_ISL_586537	Zydus Medical Hospital,Dahod	Gujarat Biotechnology Research Centre	Mohit Desai, Nitin Savaliya, Raghawendra Kumar, Dinesh Kumar, Zuber Saiyed, Komal Patel, Labdhi Pandya, Afzal Ansari, Nikha Trivedi, Apurvasinh Puvar, Janvi Raval, Zarna Patel, Monika Gandhi, Pinal Trivedi, Maharshi Pandya, Nidhi Patel, Kalpesh Labana, R D Dixit, A M Kadri, Harsh Bakshi, Chaitanya Joshi, Madhvi Joshi
EPI_ISL_586538	Zydus Medical Hospital,Dahod	Gujarat Biotechnology Research Centre	Nitin Savaliya, Raghawendra Kumar, Dinesh Kumar, Zuber Saiyed, Komal Patel, Labdhi Pandya, Afzal Ansari, Nikha Trivedi, Apurvasinh Puvar, Janvi Raval, Zarna Patel, Monika Gandhi, Pinal Trivedi, Maharshi Pandya, Nidhi Patel, Kalpesh Labana, Mohit Desai, R D Dixit, A M Kadri, Harsh Bakshi, Chaitanya Joshi, Madhvi Joshi
EPI_ISL_586539	Zydus Medical Hospital,Dahod	Gujarat Biotechnology Research Centre	Raghawendra Kumar, Dinesh Kumar, Zuber Saiyed, Komal Patel, Labdhi Pandya, Afzal Ansari, Nikha Trivedi, Apurvasinh Puvar, Janvi Raval, Zarna Patel, Monika Gandhi, Pinal Trivedi, Maharshi Pandya, Nidhi Patel, Kalpesh Labana, Mohit Desai, Nitin Savaliya, R D Dixit, A M Kadri, Harsh Bakshi, Chaitanya Joshi, Madhvi Joshi
EPI_ISL_586540	Zydus Medical Hospital,Dahod	Gujarat Biotechnology Research Centre	Dinesh Kumar, Zuber Saiyed, Komal Patel, Labdhi Pandya, Afzal Ansari, Nikha Trivedi, Apurvasinh Puvar, Janvi Raval, Zarna Patel, Monika Gandhi, Pinal Trivedi, Maharshi Pandya, Nidhi Patel, Kalpesh Labana, Mohit Desai, Nitin Savaliya, Raghawendra Kumar, R D Dixit, A M Kadri, Harsh Bakshi, Chaitanya Joshi, Madhvi Joshi
EPI_ISL_586541	Zydus Medical Hospital,Dahod	Gujarat Biotechnology Research Centre	Zuber Saiyed, Komal Patel, Labdhi Pandya, Afzal Ansari, Nikha Trivedi, Apurvasinh Puvar, Janvi Raval, Zarna Patel, Monika Gandhi, Pinal Trivedi, Maharshi Pandya, Nidhi Patel, Kalpesh Labana, Mohit Desai, Nitin Savaliya, Raghawendra Kumar, Dinesh Kumar, R D Dixit, A M Kadri, Harsh Bakshi, Chaitanya Joshi, Madhvi Joshi
EPI_ISL_586542	Government General Hospital, Jam Khambhaliya, Devbhoomi Dwarka	Gujarat Biotechnology Research Centre	Komal Patel, Labdhi Pandya, Afzal Ansari, Nikha Trivedi, Harish Matani, Apurvasinh Puvar, Janvi Raval, Zarna Patel, Monika Gandhi, Pinal Trivedi, Maharshi Pandya, Nidhi Patel, Nitin Savaliya, Raghawendra Kumar, Dinesh Kumar, Zuber Saiyed, R D Dixit, A M Kadri, Harsh Bakshi, Chaitanya Joshi, Madhvi Joshi
EPI_ISL_586543	Government General Hospital, Jam Khambhaliya, Devbhoomi Dwarka	Gujarat Biotechnology Research Centre	Labdhi Pandya, Afzal Ansari, Nikha Trivedi, Harish Matani, Apurvasinh Puvar, Janvi Raval, Zarna Patel, Monika Gandhi, Pinal Trivedi, Maharshi Pandya, Nidhi Patel, Nitin Savaliya, Raghawendra Kumar, Dinesh Kumar, Zuber Saiyed, Komal Patel, R D Dixit, A M Kadri, Harsh Bakshi, Chaitanya Joshi, Madhvi Joshi
EPI_ISL_586544	Government General Hospital, Jam Khambhaliya, Devbhoomi Dwarka	Gujarat Biotechnology Research Centre	Afzal Ansari, Nikha Trivedi, Harish Matani, Apurvasinh Puvar, Janvi Raval, Zarna Patel, Monika Gandhi, Pinal Trivedi, Maharshi Pandya, Nidhi Patel, Nitin Savaliya, Raghawendra Kumar, Dinesh Kumar, Zuber Saiyed, Komal Patel, Labdhi Pandya, R D Dixit, A M Kadri, Harsh Bakshi, Chaitanya Joshi, Madhvi Joshi
EPI_ISL_586545	Government General Hospital, Jam Khambhaliya, Devbhoomi Dwarka	Gujarat Biotechnology Research Centre	Nikha Trivedi, Harish Matani, Apurvasinh Puvar, Janvi Raval, Zarna Patel, Monika Gandhi, Pinal Trivedi, Maharshi Pandya, Nidhi Patel, Nitin Savaliya, Raghawendra Kumar, Dinesh Kumar, Zuber Saiyed, Komal Patel, Labdhi Pandya, Afzal Ansari, R D Dixit, A M Kadri, Harsh Bakshi, Chaitanya Joshi, Madhvi Joshi
EPI_ISL_586546	General Hospital,Godhra	Gujarat Biotechnology Research Centre	Neela Katara, Pinal Gandhi, Sangeeta Kumar, Apurvasinh Puvar, Janvi Raval, Zarna Patel, Monika Gandhi, Pinal Trivedi, Maharshi Pandya, Nidhi Patel, Nitin Savaliya, Raghawendra Kumar, Dinesh Kumar, Zuber Saiyed, Komal Patel, Labdhi Pandya, Afzal Ansari, Nikha Trivedi, R D Dixit, A M Kadri, Harsh Bakshi, Chaitanya Joshi, Madhvi Joshi
EPI_ISL_586547	General Hospital,Godhra	Gujarat Biotechnology Research Centre	Pinal Gandhi, Sangeeta Kumar, Apurvasinh Puvar, Janvi Raval, Zarna Patel, Monika Gandhi, Pinal Trivedi, Maharshi Pandya, Nidhi Patel, Nitin Savaliya, Raghawendra Kumar, Dinesh Kumar, Zuber Saiyed, Komal Patel, Labdhi Pandya, Afzal Ansari, Nikha Trivedi, Neela Katara, R D Dixit, A M Kadri, Harsh Bakshi, Chaitanya Joshi, Madhvi Joshi
EPI_ISL_586548	General Hospital,Godhra	Gujarat Biotechnology Research Centre	Sangeeta Kumar, Apurvasinh Puvar, Janvi Raval, Zarna Patel, Monika Gandhi, Pinal Trivedi, Maharshi Pandya, Nidhi Patel, Nitin Savaliya, Raghawendra Kumar, Dinesh Kumar, Zuber Saiyed, Komal Patel, Labdhi Pandya, Afzal Ansari, Nikha Trivedi, Neela Katara, Pinal Gandhi, R D Dixit, A M Kadri, Harsh Bakshi, Chaitanya Joshi, Madhvi Joshi
EPI_ISL_586549	CCC,Veraval	Gujarat Biotechnology Research Centre	Jignesh Parmar, Jitendra Bamrotia, Apurvasinh Puvar, Janvi Raval, Zarna Patel, Monika Gandhi, Pinal Trivedi, Maharshi Pandya, Nidhi Patel, Nitin Savaliya, Raghawendra Kumar, Dinesh Kumar, Zuber Saiyed, Komal Patel, Labdhi Pandya, Afzal Ansari, Nikha Trivedi, R D Dixit, A M Kadri, Harsh Bakshi, Chaitanya Joshi, Madhvi Joshi
EPI_ISL_586550	CCC,Veraval	Gujarat Biotechnology Research Centre	Jitendra Bamrotia, Apurvasinh Puvar, Janvi Raval, Zarna Patel, Monika Gandhi, Pinal Trivedi, Maharshi Pandya, Nidhi Patel, Nitin Savaliya, Raghawendra Kumar, Dinesh Kumar, Zuber Saiyed, Komal Patel, Labdhi Pandya, Afzal Ansari, Nikha Trivedi, Jignesh Parmar, R D Dixit, A M Kadri, Harsh Bakshi, Chaitanya Joshi, Madhvi Joshi

EPI_ISL_586551	Government Hospital,Veraval	Gujarat Biotechnology Research Centre	Monika Gandhi, Pinal Trivedi, Maharshi Pandya, Nidhi Patel, Nitin Savaliya, Raghawendra Kumar, Dinesh Kumar, Zuber Saiyed, Komal Patel, Labdhi Pandya, Afzal Ansari, Nikha Trivedi, Jignesh Parmar, Jitendra Bamrotia, Apurvasinh Puvar, Janvi Raval, Zarna Patel, R D Dixit, A M Kadri, Harsh Bakshi, Chaitanya Joshi, Madhvi Joshi
EPI_ISL_586552	Government Hospital,Veraval	Gujarat Biotechnology Research Centre	Pinal Trivedi, Maharshi Pandya, Nidhi Patel, Nitin Savaliya, Raghawendra Kumar, Dinesh Kumar, Zuber Saiyed, Komal Patel, Labdhi Pandya, Afzal Ansari, Nikha Trivedi, Jignesh Parmar, Jitendra Bamrotia, Apurvasinh Puvar, Janvi Raval, Zarna Patel, Monika Gandhi, R D Dixit, A M Kadri, Harsh Bakshi, Chaitanya Joshi, Madhvi Joshi
EPI_ISL_586553	Government Hospital,Veraval	Gujarat Biotechnology Research Centre	Maharshi Pandya, Nidhi Patel, Nitin Savaliya, Raghawendra Kumar, Dinesh Kumar, Zuber Saiyed, Komal Patel, Labdhi Pandya, Afzal Ansari, Nikha Trivedi, Jignesh Parmar, Jitendra Bamrotia, Apurvasinh Puvar, Janvi Raval, Zarna Patel, Monika Gandhi, Pinal Trivedi, R D Dixit, A M Kadri, Harsh Bakshi, Chaitanya Joshi, Madhvi Joshi
EPI_ISL_586554	Government Hospital,Veraval	Gujarat Biotechnology Research Centre	Nidhi Patel, Nitin Savaliya, Raghawendra Kumar, Dinesh Kumar, Zuber Saiyed, Komal Patel, Labdhi Pandya, Afzal Ansari, Nikha Trivedi, Jignesh Parmar, Jitendra Bamrotia, Apurvasinh Puvar, Janvi Raval, Zarna Patel, Monika Gandhi, Pinal Trivedi, Maharshi Pandya, R D Dixit, A M Kadri, Harsh Bakshi, Chaitanya Joshi, Madhvi Joshi
EPI_ISL_586555	CCC,Veraval	Gujarat Biotechnology Research Centre	Nitin Savaliya, Raghawendra Kumar, Dinesh Kumar, Zuber Saiyed, Komal Patel, Labdhi Pandya, Afzal Ansari, Nikha Trivedi, Jignesh Parmar, Jitendra Bamrotia, Apurvasinh Puvar, Janvi Raval, Zarna Patel, Monika Gandhi, Pinal Trivedi, Maharshi Pandya, Nidhi Patel, R D Dixit, A M Kadri, Harsh Bakshi, Chaitanya Joshi, Madhvi Joshi
EPI_ISL_586556	CCC,Veraval	Gujarat Biotechnology Research Centre	Raghawendra Kumar, Dinesh Kumar, Zuber Saiyed, Komal Patel, Labdhi Pandya, Afzal Ansari, Nikha Trivedi, Jignesh Parmar, Jitendra Bamrotia, Apurvasinh Puvar, Janvi Raval, Zarna Patel, Monika Gandhi, Pinal Trivedi, Maharshi Pandya, Nidhi Patel, Nitin Savaliya, R D Dixit, A M Kadri, Harsh Bakshi, Chaitanya Joshi, Madhvi Joshi
EPI_ISL_586557	CCC,Veraval	Gujarat Biotechnology Research Centre	Dinesh Kumar, Zuber Saiyed, Komal Patel, Labdhi Pandya, Afzal Ansari, Nikha Trivedi, Jignesh Parmar, Jitendra Bamrotia, Apurvasinh Puvar, Janvi Raval, Zarna Patel, Monika Gandhi, Pinal Trivedi, Maharshi Pandya, Nidhi Patel, Nitin Savaliya, Raghawendra Kumar, R D Dixit, A M Kadri, Harsh Bakshi, Chaitanya Joshi, Madhvi Joshi
EPI_ISL_586558	CCC,Veraval	Gujarat Biotechnology Research Centre	Zuber Saiyed, Komal Patel, Labdhi Pandya, Afzal Ansari, Nikha Trivedi, Jignesh Parmar, Jitendra Bamrotia, Apurvasinh Puvar, Janvi Raval, Zarna Patel, Monika Gandhi, Pinal Trivedi, Maharshi Pandya, Nidhi Patel, Nitin Savaliya, Raghawendra Kumar, Dinesh Kumar, R D Dixit, A M Kadri, Harsh Bakshi, Chaitanya Joshi, Madhvi Joshi
EPI_ISL_586559	CCC,Veraval	Gujarat Biotechnology Research Centre	Komal Patel, Labdhi Pandya, Afzal Ansari, Nikha Trivedi, Jignesh Parmar, Jitendra Bamrotia, Apurvasinh Puvar, Janvi Raval, Zarna Patel, Monika Gandhi, Pinal Trivedi, Maharshi Pandya, Nidhi Patel, Nitin Savaliya, Raghawendra Kumar, Dinesh Kumar, Zuber Saiyed, R D Dixit, A M Kadri, Harsh Bakshi, Chaitanya Joshi, Madhvi Joshi
EPI_ISL_586560	CCC,Veraval	Gujarat Biotechnology Research Centre	Labdhi Pandya, Afzal Ansari, Nikha Trivedi, Jignesh Parmar, Jitendra Bamrotia, Apurvasinh Puvar, Janvi Raval, Zarna Patel, Monika Gandhi, Pinal Trivedi, Maharshi Pandya, Nidhi Patel, Nitin Savaliya, Raghawendra Kumar, Dinesh Kumar, Zuber Saiyed, Komal Patel, R D Dixit, A M Kadri, Harsh Bakshi, Chaitanya Joshi, Madhvi Joshi
EPI_ISL_586561	CCC,Veraval	Gujarat Biotechnology Research Centre	Afzal Ansari, Nikha Trivedi, Jignesh Parmar, Jitendra Bamrotia, Apurvasinh Puvar, Janvi Raval, Zarna Patel, Monika Gandhi, Pinal Trivedi, Maharshi Pandya, Nidhi Patel, Nitin Savaliya, Raghawendra Kumar, Dinesh Kumar, Zuber Saiyed, Komal Patel, Labdhi Pandya, R D Dixit, A M Kadri, Harsh Bakshi, Chaitanya Joshi, Madhvi Joshi
EPI_ISL_586562	GMERS GMERS Medical College and Hospital, Junagadh	Gujarat Biotechnology Research Centre	Nikha Trivedi, Bhavesh Bagda , Ravi Dedaniya, Apurvasinh Puvar, Janvi Raval, Zarna Patel, Monika Gandhi, Pinal Trivedi, Maharshi Pandya, Nidhi Patel, Nitin Savaliya, Raghawendra Kumar, Dinesh Kumar, Zuber Saiyed, Komal Patel, Labdhi Pandya, Afzal Ansari, R D Dixit, A M Kadri, Harsh Bakshi, Chaitanya Joshi, Madhvi Joshi
EPI_ISL_586563	GMERS GMERS Medical College and Hospital, Junagadh	Gujarat Biotechnology Research Centre	Bhavesh Bagda , Ravi Dedaniya, Apurvasinh Puvar, Janvi Raval, Zarna Patel, Monika Gandhi, Pinal Trivedi, Maharshi Pandya, Nidhi Patel, Nitin Savaliya, Raghawendra Kumar, Dinesh Kumar, Zuber Saiyed, Komal Patel, Labdhi Pandya, Afzal Ansari, Nikha Trivedi, R D Dixit, A M Kadri, Harsh Bakshi, Chaitanya Joshi, Madhvi Joshi
EPI_ISL_586564	GMERS GMERS Medical College and Hospital, Junagadh	Gujarat Biotechnology Research Centre	Ravi Dedaniya, Apurvasinh Puvar, Janvi Raval, Zarna Patel, Monika Gandhi, Pinal Trivedi, Maharshi Pandya, Nidhi Patel, Nitin Savaliya, Raghawendra Kumar, Dinesh Kumar, Zuber Saiyed, Komal Patel, Labdhi Pandya, Afzal Ansari, Nikha Trivedi, Bhavesh Bagda , R D Dixit, A M Kadri, Harsh Bakshi, Chaitanya Joshi, Madhvi Joshi
EPI_ISL_586565	GMERS GMERS Medical College and Hospital, Junagadh	Gujarat Biotechnology Research Centre	Apurvasinh Puvar, Janvi Raval, Zarna Patel, Monika Gandhi, Pinal Trivedi, Maharshi Pandya, Nidhi Patel, Nitin Savaliya, Raghawendra Kumar, Dinesh Kumar, Zuber Saiyed, Komal Patel, Labdhi Pandya, Afzal Ansari, Nikha Trivedi, Bhavesh Bagda , Ravi Dedaniya, R D Dixit, A M Kadri, Harsh Bakshi, Chaitanya Joshi, Madhvi Joshi
EPI_ISL_586566	GMERS GMERS Medical College and Hospital, Junagadh	Gujarat Biotechnology Research Centre	Janvi Raval, Zarna Patel, Monika Gandhi, Pinal Trivedi, Maharshi Pandya, Nidhi Patel, Nitin Savaliya, Raghawendra Kumar, Dinesh Kumar, Zuber Saiyed, Komal Patel, Labdhi Pandya, Afzal Ansari, Nikha Trivedi, Bhavesh Bagda , Ravi Dedaniya, Apurvasinh Puvar, R D Dixit, A M Kadri, Harsh Bakshi, Chaitanya Joshi, Madhvi Joshi
EPI_ISL_586567, EPI_ISL_586568	Saikrishna Hospital,Mehsana	Gujarat Biotechnology Research Centre	Zarna Patel, Monika Gandhi, Pinal Trivedi, Maharshi Pandya, Nidhi Patel, Nitin Savaliya, Raghawendra Kumar, Dinesh Kumar, Zuber Saiyed, Komal Patel, Labdhi Pandya, Afzal Ansari, Nikha Trivedi, Harshadbhai Parmar, Apurvasinh Puvar, Janvi Raval, R D Dixit, A M Kadri, Harsh Bakshi, Chaitanya Joshi, Madhvi Joshi
EPI_ISL_590693	INMI Lazzaro Spallanzani IRCCS	INMI Lazzaro Spallanzani IRCCS	Martina Rueca, Barbara Bartolini, Cesare E.M. Gruber, Francesco Messina, Emanuela Giombini, Beatrice Valli, Eleonora Lalle, Simone Lanini, Francesco Vairo, Maria R. Capobianchi, Antonino Di Caro
EPI_ISL_590694	INMI Lazzaro Spallanzani IRCCS	INMI Lazzaro Spallanzani IRCCS	Barbara Bartolini, Martina Rueca, Francesco Messina, Cesare E.M. Gruber, Emanuela Giombini, Beatrice Valli, Eleonora Lalle, Simone Lanini, Francesco Vairo, Maria R. Capobianchi, Antonino Di Caro
EPI_ISL_590695	INMI Lazzaro Spallanzani IRCCS	INMI Lazzaro Spallanzani IRCCS	Cesare E.M. Gruber, Francesco Messina, Barbara Bartolini, Martina Rueca, Emanuela Giombini, Beatrice Valli, Eleonora Lalle, Simone Lanini, Francesco Vairo, Antonino Di Caro, Maria R. Capobianchi
EPI_ISL_590696	INMI Lazzaro Spallanzani IRCCS	INMI Lazzaro Spallanzani IRCCS	Cesare E.M. Gruber, Barbara Bartolini, Francesco Messina, Martina Rueca, Emanuela Giombini, Beatrice Valli, Eleonora Lalle, Simone Lanini, Francesco Vairo, Antonino Di Caro, Maria R. Capobianchi
EPI_ISL_590697	INMI Lazzaro Spallanzani IRCCS	INMI Lazzaro Spallanzani IRCCS	Martina Rueca, Cesare E.M. Gruber, Barbara Bartolini, Francesco Messina, Emanuela Giombini, Beatrice Valli, Eleonora Lalle, Simone Lanini, Francesco Vairo, Antonino Di Caro, Maria R. Capobianchi
EPI_ISL_590698	INMI Lazzaro Spallanzani IRCCS	INMI Lazzaro Spallanzani IRCCS	Barbara Bartolini, Francesco Messina, Cesare E.M. Gruber, Martina Rueca, Emanuela Giombini, Beatrice Valli, Eleonora Lalle, Simone Lanini, Francesco Vairo, Maria R. Capobianchi, Antonino Di Caro
EPI_ISL_593480, EPI_ISL_593553, EPI_ISL_593554, EPI_ISL_593555, EPI_ISL_593556	Brigham and Women's Hospital	Jonathan Li Laboratory	Manish C. Choudhary, James Regan, Jonathan Z. Li
EPI_ISL_593596	Swedish Medical Center, Seattle, WA, USA	Institute for Systems Biology	Jason D. Goldman, Kai Wang, Katharina Röltgen, Sandra C. A. Nielsen, Jared C. Roach, Samia N. Naccache, Fan Yang, Oliver F. Wirz, Kathryn E. Yost, Ji-Yeun Lee, Kelly Chun, Terri Wrin,, Christos J. Petropoulos, Inyoul Lee, Shannon Fallen, Paula M. Manner, Julie A. Wallick, Heather A. Algren, RN, Kim M. Murray, Yapeng Su, Jennifer Hadlock, Joshua Jeharajah, William R. Berrington, George P. Pappas, Sonam T. Nyatsatsang, Alexander L. Greninger, Ansuman T. Satpathy, John S. Pauk, Scott D. Boyd, James R. Heath
EPI_ISL_593912	Hospital, Marange Silvange	National Reference Center for Viruses of Respiratory Infections, Institut Pasteur, Paris	Sylvie Behillil, Fabiana Gambaro, Etienne Simon-Lorière, Vincent Enouf, Maud Vanpeene, Sylvie van der Werf
EPI_ISL_593917	Hospital, Montigny les Metz	National Reference Center for Viruses of Respiratory Infections, Institut Pasteur, Paris	Sylvie Behillil, Fabiana Gambaro, Etienne Simon-Lorière, Vincent Enouf, Maud Vanpeene, Sylvie van der Werf

EPI_ISL_593932	Labo Analyses Med, Puteaux	National Reference Center for Viruses of Respiratory Infections, Institut Pasteur, Paris	Sylvie Behillil, Fabiana Gambaro, Etienne Simon-Lorière, Vincent Enouf, Maud Vanpeene, Sylvie van der Werf
EPI_ISL_594118	Centro de Investigaciones, Universidad de Especialidades Espíritu Santo	Institute of Microbiology, Universidad San Francisco de Quito	Derly Andrade, Juan Carlos Fernandez, Belén Prado-Vivar, Sully Márquez, Juan José Guadalupe, Monica Becerra-Wong, Bernardo Gutiérrez, Gabriel Morey, Ruben Armas, Jose Pedro Barberan, Fernando Espinoza, Edith Lopez, Verónica Barragán, Patricio Rojas-Silva, Gabriel Trueba, Michelle Grunauer, Paúl Cárdenas
EPI_ISL_594188	Department of Pathology, School of Medicine, Imam Khomeini Hospital, Tehran University of Medical Sciences	Genetics Research Center, University of Social Welfare and Rehabilitation Sciences	Zohreh Fattahi, Marzieh Mohseni, Khadijeh Jalalvand, Azam Ghaziasadi, Seyedeh elham Mortazavi, Ali Jafarpour, Azar Hadadi, Alireza Abdollahi, Ali Jafarpour, Azam Ghaziasad, Seyedeh elham Mortazavi, Saber Soltani, Reza Najafipour, Kimia Kahrizi, Seyed Mohammad Jazayeri, Hossein Najmabadi
EPI_ISL_596228	WHO National Influenza Centre Russian Federation	WHO National Influenza Centre Russian Federation	Andrey Komissarov, Artem Fadeev, Anna Ivanova, Kseniya Komissarova, Dmitry Bazhenov, Daria Danilenko, Ksenia Safina, Elena Nabieva, Georgii Bazykin, Dmitry Lioznov
EPI_ISL_596264, EPI_ISL_596265	WHO National Influenza Centre Russian Federation	WHO National Influenza Centre Russian Federation	Andrey Komissarov, Artem Fadeev, Anna Ivanova, Mariia Sergeeva, Kseniya Komissarova, Dmitry Bazhenov, Daria Danilenko
EPI_ISL_596266, EPI_ISL_596267	WHO National Influenza Centre Russian Federation	WHO National Influenza Centre Russian Federation	Andrey Komissarov, Artem Fadeev, Anna Ivanova, Kseniya Komissarova, Dmitry Bazhenov, Daria Danilenko
EPI_ISL_596449	Institute for Medical Research, Infectious Disease Research Centre, National Institutes of Health, Ministry of Health Malaysia	Institute for Medical Research, Infectious Disease Research Centre, National Institutes of Health, Ministry of Health Malaysia	Suppiah J, Kamel K, Mohd-Zawawi Z, Thayan R
EPI_ISL_596451, EPI_ISL_596455	Department of Pathology, School of Medicine, Imam Khomeini Hospital, Tehran University of Medical Sciences	Genetics Research Center, University of Social Welfare and Rehabilitation Sciences	Zohreh Fattahi, Marzieh Mohseni, Khadijeh Jalalvand, Azam Ghaziasadi, Seyedeh elham Mortazavi, Ali Jafarpour, Azar Hadadi, Alireza Abdollahi, Ali Jafarpour, Azam Ghaziasad, Seyedeh elham Mortazavi, Saber Soltani, Reza Najafipour, Kimia Kahrizi, Seyed Mohammad Jazayeri, Hossein Najmabadi
EPI_ISL_602582, EPI_ISL_602583	Private medical practitioner	Hong Kong Department of Health	Mak Gannon C.K., Lam Edman T.K., Chan Rickjason C.W., Tsang Dominic N.C.
EPI_ISL_602584	Queen Mary Hospital	Hong Kong Department of Health	Mak Gannon C.K., Lam Edman T.K., Chan Rickjason C.W., Tsang Dominic N.C.
EPI_ISL_602585	Private medical practitioner	Hong Kong Department of Health	Mak Gannon C.K., Lam Edman T.K., Chan Rickjason C.W., Tsang Dominic N.C.
EPI_ISL_602586	Queen Elizabeth Hospital	Hong Kong Department of Health	Mak Gannon C.K., Lam Edman T.K., Chan Rickjason C.W., Tsang Dominic N.C.
EPI_ISL_602587	Private medical practitioner	Hong Kong Department of Health	Mak Gannon C.K., Lam Edman T.K., Chan Rickjason C.W., Tsang Dominic N.C.
EPI_ISL_602588	United Christian Hospital	Hong Kong Department of Health	Mak Gannon C.K., Lam Edman T.K., Chan Rickjason C.W., Tsang Dominic N.C.
EPI_ISL_602589	Queen Elizabeth Hospital	Hong Kong Department of Health	Mak Gannon C.K., Lam Edman T.K., Chan Rickjason C.W., Tsang Dominic N.C.
EPI_ISL_602590	Caritas Medical Centre	Hong Kong Department of Health	Mak Gannon C.K., Lam Edman T.K., Chan Rickjason C.W., Tsang Dominic N.C.
EPI_ISL_602591	Queen Elizabeth Hospital	Hong Kong Department of Health	Mak Gannon C.K., Lam Edman T.K., Chan Rickjason C.W., Tsang Dominic N.C.
EPI_ISL_602592	United Christian Hospital	Hong Kong Department of Health	Mak Gannon C.K., Lam Edman T.K., Chan Rickjason C.W., Tsang Dominic N.C.
EPI_ISL_602593	Queen Mary Hospital	Hong Kong Department of Health	Mak Gannon C.K., Lam Edman T.K., Chan Rickjason C.W., Tsang Dominic N.C.
EPI_ISL_602594	Prince of Wales Hospital	Hong Kong Department of Health	Mak Gannon C.K., Lam Edman T.K., Chan Rickjason C.W., Tsang Dominic N.C.
EPI_ISL_602595, EPI_ISL_602596	Pamela Youde Nethersole Eastern Hospital	Hong Kong Department of Health	Mak Gannon C.K., Lam Edman T.K., Chan Rickjason C.W., Tsang Dominic N.C.
EPI_ISL_602597	Private medical practitioner	Hong Kong Department of Health	Mak Gannon C.K., Lam Edman T.K., Chan Rickjason C.W., Tsang Dominic N.C.
EPI_ISL_602598	Our Lady of Maryknoll Hospital	Hong Kong Department of Health	Mak Gannon C.K., Lam Edman T.K., Chan Rickjason C.W., Tsang Dominic N.C.
EPI_ISL_602599	Tseung Kwan O Hospital	Hong Kong Department of Health	Mak Gannon C.K., Lam Edman T.K., Chan Rickjason C.W., Tsang Dominic N.C.
EPI_ISL_602600	United Christian Hospital	Hong Kong Department of Health	Mak Gannon C.K., Lam Edman T.K., Chan Rickjason C.W., Tsang Dominic N.C.
EPI_ISL_602601	Tseung Kwan O Hospital	Hong Kong Department of Health	Mak Gannon C.K., Lam Edman T.K., Chan Rickjason C.W., Tsang Dominic N.C.
EPI_ISL_602602	Ruttonjee Hospital	Hong Kong Department of Health	Mak Gannon C.K., Lam Edman T.K., Chan Rickjason C.W., Tsang Dominic N.C.
EPI_ISL_602603, EPI_ISL_602604	Queen Mary Hospital	Hong Kong Department of Health	Mak Gannon C.K., Lam Edman T.K., Chan Rickjason C.W., Tsang Dominic N.C.
EPI_ISL_602605	Pamela Youde Nethersole Eastern Hospital	Hong Kong Department of Health	Mak Gannon C.K., Lam Edman T.K., Chan Rickjason C.W., Tsang Dominic N.C.
EPI_ISL_602606	Queen Elizabeth Hospital	Hong Kong Department of Health	Mak Gannon C.K., Lam Edman T.K., Chan Rickjason C.W., Tsang Dominic N.C.
EPI_ISL_602607	Tuen Mun Hospital	Hong Kong Department of Health	Mak Gannon C.K., Lam Edman T.K., Chan Rickjason C.W., Tsang Dominic N.C.
EPI_ISL_602608	United Christian Hospital	Hong Kong Department of Health	Mak Gannon C.K., Lam Edman T.K., Chan Rickjason C.W., Tsang Dominic N.C.
EPI_ISL_602609	Pamela Youde Nethersole Eastern Hospital	Hong Kong Department of Health	Mak Gannon C.K., Lam Edman T.K., Chan Rickjason C.W., Tsang Dominic N.C.
EPI_ISL_602610	Queen Mary Hospital	Hong Kong Department of Health	Mak Gannon C.K., Lam Edman T.K., Chan Rickjason C.W., Tsang Dominic N.C.
EPI_ISL_602611	Queen Elizabeth Hospital	Hong Kong Department of Health	Mak Gannon C.K., Lam Edman T.K., Chan Rickjason C.W., Tsang Dominic N.C.
EPI_ISL_602612	Tseung Kwan O Hospital	Hong Kong Department of Health	Mak Gannon C.K., Lam Edman T.K., Chan Rickjason C.W., Tsang Dominic N.C.
EPI_ISL_602613, EPI_ISL_602614, EPI_ISL_602615	Pamela Youde Nethersole Eastern Hospital	Hong Kong Department of Health	Mak Gannon C.K., Lam Edman T.K., Chan Rickjason C.W., Tsang Dominic N.C.
EPI_ISL_602616	Kwong Wah Hospital	Hong Kong Department of Health	Mak Gannon C.K., Lam Edman T.K., Chan Rickjason C.W., Tsang Dominic N.C.
EPI_ISL_602617, EPI_ISL_602618	Queen Mary Hospital	Hong Kong Department of Health	Mak Gannon C.K., Lam Edman T.K., Chan Rickjason C.W., Tsang Dominic N.C.
EPI_ISL_602619	United Christian Hospital	Hong Kong Department of Health	Mak Gannon C.K., Lam Edman T.K., Chan Rickjason C.W., Tsang Dominic N.C.
EPI_ISL_602620	Queen Elizabeth Hospital	Hong Kong Department of Health	Mak Gannon C.K., Lam Edman T.K., Chan Rickjason C.W., Tsang Dominic N.C.
EPI_ISL_602621	GLENEAGLES HONG KONG (CLINICAL LAB)	Hong Kong Department of Health	Mak Gannon C.K., Lam Edman T.K., Chan Rickjason C.W., Tsang Dominic N.C.
EPI_ISL_602622	AHRI-Sigal	KRISP, KZN Research Innovation and Sequencing Platform	Gazy I, Sigla, Karim F, Cele S, Giandhari J, Pillay S, Tegally H, Wilkinson E, de Oliveira T
EPI_ISL_602624, EPI_ISL_602625, EPI_ISL_602626, EPI_ISL_602627, EPI_ISL_602628, EPI_ISL_602629, EPI_ISL_602630	AHRI-Sigal	KRISP, KZN Research Innovation and Sequencing Platform	Gazy I, Sigl A, Karim F, Cele S, Giandhari J, Pillay S, Tegally H, Wilkinson E, de Oliveira T
EPI_ISL_602632, EPI_ISL_602633, EPI_ISL_602634, EPI_ISL_602635, EPI_ISL_602636, EPI_ISL_602637, EPI_ISL_602638, EPI_ISL_602640, EPI_ISL_602641, EPI_ISL_602644, EPI_ISL_602645, EPI_ISL_602650, EPI_ISL_602654, EPI_ISL_602659, EPI_ISL_602660, EPI_ISL_602661, EPI_ISL_602666, EPI_ISL_602667, EPI_ISL_602668, EPI_ISL_602669, EPI_ISL_602670, EPI_ISL_602671, EPI_ISL_602672, EPI_ISL_602673, EPI_ISL_602674, EPI_ISL_602675, EPI_ISL_602676, EPI_ISL_602677, EPI_ISL_602678, EPI_ISL_602679, EPI_ISL_602680, EPI_ISL_602681			
see above	NHLS-IALCH	KRISP, KZN Research Innovation and Sequencing Platform	Giandhari J, Pillay S, Lessells R, Mdlalose K, York D, Khan S, Tegally H, Wilkinson E, de Oliveira T
EPI_ISL_603021	Pronto Socorro Dr. Conrado Cesarino Nuvolini	Instituto Adolfo Lutz, Interdiciplinary Procedures Center, Strategic Laboratory	Claudio Tavares Sacchi, Claudia Regina Gonçalves, Erica Valessa Ramos Gomes, Karoline Rodrigues Campos
EPI_ISL_603022	Departamento de Vigilância à Saúde	Instituto Adolfo Lutz, Interdiciplinary Procedures Center, Strategic Laboratory	Claudio Tavares Sacchi, Claudia Regina Gonçalves, Erica Valessa Ramos Gomes, Karoline Rodrigues Campos
EPI_ISL_603023	Vigilância em Saúde Visa Sul	Instituto Adolfo Lutz, Interdiciplinary Procedures Center,	Claudio Tavares Sacchi, Claudia Regina Gonçalves, Erica Valessa Ramos Gomes, Karoline Rodrigues Campos

		Strategic Laboratory	
EPI_ISL_603024	Santa Casa de Misericordia de Araçatuba	Instituto Adolfo Lutz, Interdiciplinary Procedures Center, Strategic Laboratory	Claudio Tavares Sacchi, Claudia Regina Gonçalves, Erica Valessa Ramos Gomes, Karoline Rodrigues Campos
EPI_ISL_603025	UPA Central de Caraguatatuba	Instituto Adolfo Lutz, Interdiciplinary Procedures Center, Strategic Laboratory	Claudio Tavares Sacchi, Claudia Regina Gonçalves, Erica Valessa Ramos Gomes, Karoline Rodrigues Campos
EPI_ISL_603026	Santa Casa da Misericórdia de Presidente Prudente	Instituto Adolfo Lutz, Interdiciplinary Procedures Center, Strategic Laboratory	Claudio Tavares Sacchi, Claudia Regina Gonçalves, Erica Valessa Ramos Gomes, Karoline Rodrigues Campos
EPI_ISL_603027	Santa Casa de Misericordia de Araçatuba	Instituto Adolfo Lutz, Interdiciplinary Procedures Center, Strategic Laboratory	Claudio Tavares Sacchi, Claudia Regina Gonçalves, Erica Valessa Ramos Gomes, Karoline Rodrigues Campos
EPI_ISL_603028	Hospital Municipal Santa Ana	Instituto Adolfo Lutz, Interdiciplinary Procedures Center, Strategic Laboratory	Claudio Tavares Sacchi, Claudia Regina Gonçalves, Erica Valessa Ramos Gomes, Karoline Rodrigues Campos
EPI_ISL_603029	Hospital Municipal Mário Gatti	Instituto Adolfo Lutz, Interdiciplinary Procedures Center, Strategic Laboratory	Claudio Tavares Sacchi, Claudia Regina Gonçalves, Erica Valessa Ramos Gomes, Karoline Rodrigues Campos
EPI_ISL_603030	Hospital Domingos Leonardo Ceravolo Presidente Prudente	Instituto Adolfo Lutz, Interdiciplinary Procedures Center, Strategic Laboratory	Claudio Tavares Sacchi, Claudia Regina Gonçalves, Erica Valessa Ramos Gomes, Karoline Rodrigues Campos
EPI_ISL_603031	Santa Casa de Presidente Epitácio	Instituto Adolfo Lutz, Interdiciplinary Procedures Center, Strategic Laboratory	Claudio Tavares Sacchi, Claudia Regina Gonçalves, Erica Valessa Ramos Gomes, Karoline Rodrigues Campos
EPI_ISL_603032	Santa Casa da Misericórdia de Presidente Prudente	Instituto Adolfo Lutz, Interdiciplinary Procedures Center, Strategic Laboratory	Claudio Tavares Sacchi, Claudia Regina Gonçalves, Erica Valessa Ramos Gomes, Karoline Rodrigues Campos
EPI_ISL_603033	Vigilancia Epidemiologica de São Bernardo do Campo	Instituto Adolfo Lutz, Interdiciplinary Procedures Center, Strategic Laboratory	Claudio Tavares Sacchi, Claudia Regina Gonçalves, Erica Valessa Ramos Gomes, Karoline Rodrigues Campos
EPI_ISL_603034	Departamento de Vigilância à Saúde	Instituto Adolfo Lutz, Interdiciplinary Procedures Center, Strategic Laboratory	Claudio Tavares Sacchi, Claudia Regina Gonçalves, Erica Valessa Ramos Gomes, Karoline Rodrigues Campos
EPI_ISL_603035	Secretaria Municipal de Saúde	Instituto Adolfo Lutz, Interdiciplinary Procedures Center, Strategic Laboratory	Claudio Tavares Sacchi, Claudia Regina Gonçalves, Erica Valessa Ramos Gomes, Karoline Rodrigues Campos
EPI_ISL_603036	Hospital Santa Ana	Instituto Adolfo Lutz, Interdiciplinary Procedures Center, Strategic Laboratory	Claudio Tavares Sacchi, Claudia Regina Gonçalves, Erica Valessa Ramos Gomes, Karoline Rodrigues Campos
EPI_ISL_603037	Hospital Geral de Pedreira	Instituto Adolfo Lutz, Interdiciplinary Procedures Center, Strategic Laboratory	Claudio Tavares Sacchi, Claudia Regina Gonçalves, Erica Valessa Ramos Gomes, Karoline Rodrigues Campos
EPI_ISL_603038	Santa Casa de Misericordia de Araçatuba	Instituto Adolfo Lutz, Interdiciplinary Procedures Center, Strategic Laboratory	Claudio Tavares Sacchi, Claudia Regina Gonçalves, Erica Valessa Ramos Gomes, Karoline Rodrigues Campos
EPI_ISL_603039	Hospital Municipal Mário Gatti	Instituto Adolfo Lutz, Interdiciplinary Procedures Center, Strategic Laboratory	Claudio Tavares Sacchi, Claudia Regina Gonçalves, Erica Valessa Ramos Gomes, Karoline Rodrigues Campos
EPI_ISL_603137	INMI Lazzaro Spallanzani IRCCS	INMI Lazzaro Spallanzani IRCCS	Cesare E.M. Gruber, Martina Rueca, Barbara Bartolini, Francesco Messina, Emanuela Giombini, Simone Lanini, Antonino Di Caro, Maria R. Capobianchi
EPI_ISL_603138	INMI Lazzaro Spallanzani IRCCS	INMI Lazzaro Spallanzani IRCCS	Martina Rueca, Francesco Messina, Barbara Bartolini, Cesare E.M. Gruber, Emanuela Giombini, Simone Lanini, Antonino Di Caro, Maria R. Capobianchi
EPI_ISL_603139	INMI Lazzaro Spallanzani IRCCS	INMI Lazzaro Spallanzani IRCCS	Martina Rueca, Cesare E.M. Gruber, Barbara Bartolini, Francesco Messina, Emanuela Giombini, Simone Lanini, Antonino Di Caro, Maria R. Capobianchi
EPI_ISL_603140	INMI Lazzaro Spallanzani IRCCS	INMI Lazzaro Spallanzani IRCCS	Martina Rueca, Cesare E.M. Gruber, Francesco Messina, Barbara Bartolini, Emanuela Giombini, Simone Lanini, Antonino Di Caro, Maria R. Capobianchi
EPI_ISL_603141	INMI Lazzaro Spallanzani IRCCS	INMI Lazzaro Spallanzani IRCCS	Francesco Messina, Cesare E.M. Gruber, Martina Rueca, Barbara Bartolini, Emanuela Giombini, Simone Lanini, Maria R. Capobianchi, Antonino Di Caro
EPI_ISL_603142	INMI Lazzaro Spallanzani IRCCS	INMI Lazzaro Spallanzani IRCCS	Barbara Bartolini, Cesare E.M. Gruber, Francesco Messina, Martina Rueca, Simone Lanini, Emanuela Giombini, Maria R. Capobianchi, Antonino Di Caro
EPI_ISL_603143	INMI Lazzaro Spallanzani IRCCS	INMI Lazzaro Spallanzani IRCCS	Martina Rueca, Francesco Messina, Cesare E.M. Gruber, Barbara Bartolini, Emanuela Giombini, Simone Lanini, Antonino Di Caro, Maria R. Capobianchi
EPI_ISL_603144	INMI Lazzaro Spallanzani IRCCS	INMI Lazzaro Spallanzani IRCCS	Cesare E.M. Gruber, Martina Rueca, Francesco Messina, Barbara Bartolini, Emanuela Giombini, Simone Lanini, Maria R. Capobianchi, Antonino Di Caro
EPI_ISL_603145	INMI Lazzaro Spallanzani IRCCS	INMI Lazzaro Spallanzani IRCCS	Francesco Messina, Cesare E.M. Gruber, Martina Rueca, Barbara Bartolini, Emanuela Giombini, Simone Lanini, Antonino Di Caro, Maria R. Capobianchi
EPI_ISL_603146	INMI Lazzaro Spallanzani IRCCS	INMI Lazzaro Spallanzani IRCCS	Cesare E.M. Gruber, Martina Rueca, Barbara Bartolini, Emanuela Giombini, Francesco Messina, Simone Lanini, Maria R. Capobianchi, Antonino Di Caro
EPI_ISL_603147	INMI Lazzaro Spallanzani IRCCS	INMI Lazzaro Spallanzani IRCCS	Cesare E.M. Gruber, Barbara Bartolini, Francesco Messina, Martina Rueca, Simone Lanini, Emanuela Giombini, Antonino Di Caro, Maria R. Capobianchi
EPI_ISL_603148	INMI Lazzaro Spallanzani IRCCS	INMI Lazzaro Spallanzani IRCCS	Cesare E.M. Gruber, Francesco Messina, Martina Rueca, Simone Lanini, Emanuela Giombini, Barbara Bartolini, Maria R. Capobianchi, Antonino Di Caro
EPI_ISL_603149	INMI Lazzaro Spallanzani IRCCS	INMI Lazzaro Spallanzani IRCCS	Francesco Messina, Barbara Bartolini, Cesare E.M. Gruber, Martina Rueca, Simone Lanini, Emanuela Giombini, Patrizia Massarelli, Antonino Di Caro, Maria R. Capobianchi
EPI_ISL_603150	INMI Lazzaro Spallanzani IRCCS	INMI Lazzaro Spallanzani IRCCS	Barbara Bartolini, Francesco Messina, Martina Rueca, Cesare E.M. Gruber, Simone Lanini, Emanuela Giombini, Patrizia Massarelli, Maria R. Capobianchi, Antonino Di Caro
EPI_ISL_603151	INMI Lazzaro Spallanzani IRCCS	INMI Lazzaro Spallanzani IRCCS	Francesco Messina, Barbara Bartolini, Cesare E.M. Gruber, Martina Rueca, Emanuela Giombini, Simone Lanini, Patrizia Massarelli, Maria R. Capobianchi, Antonino Di Caro
EPI_ISL_603152	INMI Lazzaro Spallanzani IRCCS	INMI Lazzaro Spallanzani IRCCS	Barbara Bartolini, Cesare E.M. Gruber, Francesco Messina, Martina Rueca, Emanuela Giombini, Simone Lanini, Patrizia Massarelli, Antonino Di Caro, Maria R. Capobianchi
EPI_ISL_603153	INMI Lazzaro Spallanzani IRCCS	INMI Lazzaro Spallanzani IRCCS	Francesco Messina, Cesare E.M. Gruber, Barbara Bartolini, Simone Lanini, Emanuela Giombini, Martina Rueca, Marcello Meledandri, Maria Letizia Schiavone, Annunziata Tamburro, Antonino Di Caro, Maria R. Capobianchi
EPI_ISL_603154	INMI Lazzaro Spallanzani IRCCS	INMI Lazzaro Spallanzani IRCCS	Martina Rueca, Francesco Messina, Cesare E.M. Gruber, Barbara Bartolini, Emanuela Giombini, Simone Lanini, Antonino Di Caro, Maria R. Capobianchi
EPI_ISL_603155	INMI Lazzaro Spallanzani IRCCS	INMI Lazzaro Spallanzani IRCCS	Martina Rueca, Francesco Messina, Barbara Bartolini, Cesare E.M. Gruber, Simone Lanini, Emanuela Giombini, Maria R. Capobianchi, Antonino Di Caro
EPI_ISL_603156	INMI Lazzaro Spallanzani IRCCS	INMI Lazzaro Spallanzani IRCCS	Barbara Bartolini, Francesco Messina, Cesare E.M. Gruber, Martina Rueca, Emanuela Giombini, Simone Lanini, Maria R. Capobianchi, Antonino Di Caro
EPI_ISL_603157	INMI Lazzaro Spallanzani IRCCS	INMI Lazzaro Spallanzani IRCCS	Francesco Messina, Martina Rueca, Barbara Bartolini, Cesare E.M. Gruber, Simone Lanini, Emanuela Giombini, Fulvia Pimpinelli, Antonino Di Caro, Maria R. Capobianchi
EPI_ISL_603158	INMI Lazzaro Spallanzani IRCCS	INMI Lazzaro Spallanzani IRCCS	Barbara Bartolini, Cesare E.M. Gruber, Francesco Messina, Martina Rueca, Emanuela Giombini, Simone Lanini, Fulvia Pimpinelli, Maria R. Capobianchi, Antonino Di Caro
EPI_ISL_603159	INMI Lazzaro Spallanzani IRCCS	INMI Lazzaro Spallanzani IRCCS	Barbara Bartolini, Francesco Messina, Cesare E.M. Gruber, Martina Rueca, Simone Lanini, Emanuela Giombini, Fulvia Pimpinelli, Maria R. Capobianchi, Antonino Di Caro
EPI_ISL_603160	INMI Lazzaro Spallanzani IRCCS	INMI Lazzaro Spallanzani IRCCS	Barbara Bartolini, Simone Lanini, Emanuela Giombini, Francesco Messina, Cesare E.M. Gruber, Martina Rueca, Fulvia Pimpinelli, Maria R. Capobianchi, Antonino Di Caro
EPI_ISL_603161	INMI Lazzaro Spallanzani IRCCS	INMI Lazzaro Spallanzani IRCCS	Cesare E.M. Gruber, Barbara Bartolini, Francesco Messina, Martina Rueca, Emanuela Giombini, Simone Lanini, Fulvia Pimpinelli, Antonino Di Caro, Maria R.

			Capobianchi
EPI_ISL_603162	INMI Lazzaro Spallanzani IRCCS	INMI Lazzaro Spallanzani IRCCS	Cesare E.M. Gruber, Francesco Messina, Martina Rueca, Barbara Bartolini, Emanuela Giombini, Simone Lanini, Fulvia Pimpinelli, Antonino Di Caro, Maria R. Capobianchi
EPI_ISL_603163	INMI Lazzaro Spallanzani IRCCS	INMI Lazzaro Spallanzani IRCCS	Cesare E.M. Gruber, Martina Rueca, Emanuela Giombini, Simone Lanini, Francesco Messina, Barbara Bartolini, Fulvia Pimpinelli, Maria R. Capobianchi, Antonino Di Caro
EPI_ISL_603164	INMI Lazzaro Spallanzani IRCCS	INMI Lazzaro Spallanzani IRCCS	Cesare E.M. Gruber, Francesco Messina, Martina Rueca, Barbara Bartolini, Emanuela Giombini, Simone Lanini, Fulvia Pimpinelli, Antonino Di Caro, Maria R. Capobianchi
EPI_ISL_603165	INMI Lazzaro Spallanzani IRCCS	INMI Lazzaro Spallanzani IRCCS	Martina Rueca, Francesco Messina, Barbara Bartolini, Cesare E.M. Gruber, Emanuela Giombini, Simone Lanini, Fulvia Pimpinelli, Maria R. Capobianchi, Antonino Di Caro
EPI_ISL_603166	INMI Lazzaro Spallanzani IRCCS	INMI Lazzaro Spallanzani IRCCS	Martina Rueca, Cesare E.M. Gruber, Barbara Bartolini, Francesco Messina, Simone Lanini, Emanuela Giombini, Fulvia Pimpinelli, Maria R. Capobianchi, Antonino Di Caro
EPI_ISL_603167	INMI Lazzaro Spallanzani IRCCS	INMI Lazzaro Spallanzani IRCCS	Martina Rueca, Cesare E.M. Gruber, Francesco Messina, Barbara Bartolini, Simone Lanini, Emanuela Giombini, Fulvia Pimpinelli, Maria R. Capobianchi, Antonino Di Caro
EPI_ISL_603168	INMI Lazzaro Spallanzani IRCCS	INMI Lazzaro Spallanzani IRCCS	Martina Rueca, Barbara Bartolini, Cesare E.M. Gruber, Simone Lanini, Emanuela Giombini, Francesco Messina, Fulvia Pimpinelli, Antonino Di Caro, Maria R. Capobianchi
EPI_ISL_603169	INMI Lazzaro Spallanzani IRCCS	INMI Lazzaro Spallanzani IRCCS	Francesco Messina, Barbara Bartolini, Cesare E.M. Gruber, Martina Rueca, Simone Lanini, Emanuela Giombini, Fulvia Pimpinelli, Maria R. Capobianchi, Antonino Di Caro
EPI_ISL_603170	INMI Lazzaro Spallanzani IRCCS	INMI Lazzaro Spallanzani IRCCS	Martina Rueca, Barbara Bartolini, Cesare E.M. Gruber, Francesco Messina, Simone Lanini, Emanuela Giombini, Maria R. Capobianchi, Antonino Di Caro
EPI_ISL_603171	INMI Lazzaro Spallanzani IRCCS	INMI Lazzaro Spallanzani IRCCS	Barbara Bartolini, Cesare E.M. Gruber, Francesco Messina, Martina Rueca, Emanuela Giombini, Simone Lanini, Mariarosa Gaudio, Antonino Di Caro, Maria R. Capobianchi
EPI_ISL_603172	INMI Lazzaro Spallanzani IRCCS	INMI Lazzaro Spallanzani IRCCS	Cesare E.M. Gruber, Martina Rueca, Barbara Bartolini, Simone Lanini, Francesco Messina, Emanuela Giombini, Mariarosa Gaudio, Antonino Di Caro, Maria R. Capobianchi
EPI_ISL_603173	INMI Lazzaro Spallanzani IRCCS	INMI Lazzaro Spallanzani IRCCS	Barbara Bartolini, Cesare E.M. Gruber, Martina Rueca, Francesco Messina, Simone Lanini, Emanuela Giombini, Giuseppina Cappiello, Maria R. Capobianchi, Antonino Di Caro
EPI_ISL_603174	INMI Lazzaro Spallanzani IRCCS	INMI Lazzaro Spallanzani IRCCS	Francesco Messina, Barbara Bartolini, Martina Rueca, Emanuela Giombini, Simone Lanini, Cesare E.M. Gruber, Mariarosa Gaudio, Maria R. Capobianchi, Antonino Di Caro
EPI_ISL_603175	INMI Lazzaro Spallanzani IRCCS	INMI Lazzaro Spallanzani IRCCS	Barbara Bartolini, Cesare E.M. Gruber, Simone Lanini, Emanuela Giombini, Francesco Messina, Martina Rueca, Mariarosa Gaudio, Antonino Di Caro, Maria R. Capobianchi
EPI_ISL_603176	INMI Lazzaro Spallanzani IRCCS	INMI Lazzaro Spallanzani IRCCS	Cesare E.M. Gruber, Barbara Bartolini, Francesco Messina, Martina Rueca, Simone Lanini, Emanuela Giombini, Antonino Di Caro, Maria R. Capobianchi
EPI_ISL_603223	National Institute of Laboratory Medicine and Referral Center	Genomic Research Lab, BCSIR	Md. Murshed Hasan Sarkar, Abu Sayeed Mohammad Mahmud, Mohammad Samir Uzzaman, Eshrar Osman, Md. Ahashan Habib, Shahina Akter, Tanjina Akhter Banu, Barna Goswami, Iffat Jahan, Md. Saddam Hossain, Tasnim Nafisa, Md. Maruf Ahmed Molla, Mahmuda Yeasmin, Asish Kumar Ghosh, A. K. M. Shamsuzzaman, Monira Parveen, Md. Masum Hossain Arif, Md. Salim Khan
EPI_ISL_610162	RSUD Dr. Tjitrowardojo	Genetics Working Group (Pokja Genetik) Faculty of Medicine, Public Health and Nursing Universitas Gadjah Mada (FK-KMK UGM); Disease Investigation Center Wates Ministry of Agriculture Indonesia; Department of Microbiology FK-KMK UGM; Laboratorium Diagnostik Yayasan Tahija World Mosquito Program (WMP) Yogyakarta Center for Tropical Medicine FK-KMK UGM; Integrated Research Center FK-KMK UGM; Department of Computer Science and Electronics FMIPA UGM; Balai Besar Teknik Kesehatan Lingkungan dan Pengendalian Penyakit (BBTKLPP) Yogyakarta	Gunadi, Hendra Wibawa, Marcellus, Mohamad S. Hakim, Edwin W. Daniwijaya, Ludhang P. Rizki, Endah Supriyati, Eggi Arguni, Titik Nuryastuti, Tri Wibawa, Dwi AA Nugrahaningsih, Afiahayati, Siswanto, Kristy Iskandar, Nungki Anggorowati, Irene, Indaryati, Havid Setyawan, Wuryanto, Susan Simanjaya, Alvin Santoso Kalim
EPI_ISL_610227, EPI_ISL_610228, EPI_ISL_610229, EPI_ISL_610230, EPI_ISL_610231, EPI_ISL_610232, EPI_ISL_610233, EPI_ISL_610234, EPI_ISL_610235, EPI_ISL_610236, EPI_ISL_610239, EPI_ISL_610240, EPI_ISL_610241, EPI_ISL_610242, EPI_ISL_610243, EPI_ISL_610244			
see above	Molecular diagnostic laboratory of Federal Budget Institution of Science "Central Research Institute of Epidemiology" of The Federal Service on Customers' Rights Protection and Human Well-being Surveillance	Group of Genomics and Postgenomic Technologies of Central Research Institute of Epidemilology	Samoilov AE, Kaptelova VV, Valdokhina AV, Bulanenko VP, Speranskaya AS, Tivanova EV, Shipulina OY, Akimkin VG
EPI_ISL_613457	QUARANTINE CAMP	Hong Kong Department of Health	Alan K.L. Tsang, Peter C.W. Yip, Edman T.K. Lam, Rickjason C.W. Chan, Dominic N.C. Tsang
EPI_ISL_613545, EPI_ISL_613546, EPI_ISL_613547, EPI_ISL_613548, EPI_ISL_613550	CHRU Pontchaillou - Laboratoire de Virologie 2, rue Henri Le Guilloux	National Reference Center for Viruses of Respiratory Infections, Institut Pasteur, Paris	Marion Barbet, Sylvie Behillil, Méline Bizard, Angela Brisebarre, Camille Capel, Etienne Simon-Lorière, Vincent Enouf, Maud Vanpeene, Sylvie van der Werf, Gisèle Lagathu
EPI_ISL_614265, EPI_ISL_614266, EPI_ISL_614267, EPI_ISL_614268, EPI_ISL_614269, EPI_ISL_614270, EPI_ISL_614271, EPI_ISL_614272, EPI_ISL_614273, EPI_ISL_614274, EPI_ISL_614275, EPI_ISL_614276, EPI_ISL_614277, EPI_ISL_614278, EPI_ISL_614279, EPI_ISL_614280			
see above	Eurofins	National Reference Center for Viruses of Respiratory Infections, Institut Pasteur, Paris	Marion Barbet, Sylvie Behillil, Méline Bizard, Angela Brisebarre, Camille Capel, Etienne Simon-Lorière, Vincent Enouf, Maud Vanpeene, Sylvie van der Werf
EPI_ISL_614307, EPI_ISL_614308, EPI_ISL_614310, EPI_ISL_614311, EPI_ISL_614312	Faroese National Reference Laboratory for Fish and Animal Diseases	Faroese National Reference Laboratory for Fish and Animal Diseases	Maria Marjunardóttir Dahl, Petra Elisabeth Petersen, Debes Hammershaimb Christiansen
EPI_ISL_623145, EPI_ISL_623147	Laboratorio de Virologia Molecular / UFRJ	Bioinformatics Laboratory / LNCC	Carolina M Voloch, Ronaldo S Francisco Jr, Luiz G P de Almeida, Otavio J. Brustolini, Cynthia C Cardoso, Alexandra L Gerber, Ana Paula de C Guimarães, Diana Mariani, Covid19-UFRJ Workgroup, Luís Cristóvão Pôrto, Renato S Aguiar, Terezinha M P P Castiñeiras, Orlando C. Ferreira, Amilcar Tanuri, Ana Tereza R de Vasconcelos
EPI_ISL_632312	NU-sjukvården	Clinical microbiology, Sahlgrenska University Hospital	Johan Ringlander, Josefin Olausson, Hedvig Engström Jakobsson, Magnus Lindh
EPI_ISL_632936	Puskesmas Mlati 1 Sleman	Genetics Working Group (Pokja Genetik) Faculty of Medicine, Public Health and Nursing Universitas Gadjah Mada (FK-KMK UGM); Disease Investigation Center Wates Ministry of Agriculture Indonesia; Department of Microbiology FK-KMK UGM; Laboratorium Diagnostik Yayasan Tahija World Mosquito Program (WMP) Yogyakarta Center for Tropical Medicine FK-KMK UGM; Integrated Research Center FK-KMK UGM; Department of Computer Science and Electronics FMIPA UGM; RSUP Dr. Sardjito	Gunadi, Hendra Wibawa, Marcellus, Mohamad S. Hakim, Edwin W. Daniwijaya, Ludhang P. Rizki, Endah Supriyati, Eggi Arguni, Titik Nuryastuti, Tri Wibawa, Dwi AA Nugrahaningsih, Afiahayati, Siswanto, Kristy Iskandar, Nungki Anggorowati, Ika Trisnawati, Sumardi, Eko Budiono, Bambang Sigit Riyanto, Heni Retnowulan, Munawar Gani, Satria Maulana, Nur Rahmi Ananda, Riat El Khair, Yunika Puspadewi, Osman Sianipar, Umi Solekhah Intansari, Elizabeth Henny Herningtiyas, Ira Puspitawati, Nur Imma Fatimah Harahap, Ernawati, Untung Wirawan , William Widitjiarso
EPI_ISL_636737, EPI_ISL_636835, EPI_ISL_636837	Laboratório de Imunofarmacologia - Instituto Oswaldo Cruz	Laboratório de Imunofarmacologia - Instituto Oswaldo Cruz	Souza,T.M., Fintelman-Rodrigues,N., De Paula,A.D., Saraiva,F.B., Ferreira,M.A. and Sacramento,C.Q.

EPI_ISL_636965	Pathogen Genomics Lab King Abdullah University of Science and Technology(KAUST)	Pathogen Genomics Lab King Abdullah University of Science and Technology(KAUST)	Raeece Naeem, Rahul P Salunke, Sharif Hala, Sara Mfarrej, Amit Kumar Subudhi, Fadwa Alofi, Fathia Ben Rached, Afrah Alsomali, Asim Khogeer, Ahmad Bakur Mahmoud, Anwar Hashem, Naif Almontashiri, Arnab Pain
EPI_ISL_636966, EPI_ISL_636967	Pathogen Genomics Lab King Abdullah University of Science and Technology(KAUST)	Pathogen Genomics Lab King Abdullah University of Science and Technology(KAUST)	Fathia Ben Rached, Raeece Naeem, Sharif Hala, Fadwa Alofi, Rahul P Salunke, Sara Mfarrej, Amit Kumar Subudhi, Afrah Alsomali, Asim Khogeer, Ahmad Bakur Mahmoud, Anwar Hashem, Naif Almontashiri, Arnab Pain
EPI_ISL_636972	Pathogen Genomics Lab King Abdullah University of Science and Technology (KAUST)	Pathogen Genomics Lab King Abdullah University of Science and Technology (KAUST)	Afrah Alsomali, Fathia Ben Rached, Raeece Naeem, Sharif Hala,Rahul P Salunke, Amanda Ooi, Luke Esau, Sara Mfarrej, Amit Kumar Subudhi, Fadwa Alofi, Asim Khogeer, Kahled Algithami, Anwar Hashem, Naif Almontashiri, Arnab Pain
EPI_ISL_636973	Public Health Lab	Public Health Lab	Alwasti, H
EPI_ISL_636977	HP Pemba	KRISP, KZN Research Innovation and Sequencing Platform	Ismael N, Giandhari J, Pillay S, Tegally H, Wilkinson E, de Oliveira T, Nadia Sitoe, Paulo Arnaldo, Nedio Mabunda
EPI_ISL_636980	CS Xai Xai	KRISP, KZN Research Innovation and Sequencing Platform	Ismael N, Giandhari J, Pillay S, Tegally H, Wilkinson E, de Oliveira T, Nadia Sitoe, Paulo Arnaldo, Nedio Mabunda
EPI_ISL_637022	Tin Shui Wai Hospital	Hong Kong Department of Health	Mak Gannon C.K., Lam Edman T.K., Chan Rickjason C.W., Tsang Dominic N.C.
EPI_ISL_637023	Queen Elizabeth Hospital	Hong Kong Department of Health	Mak Gannon C.K., Lam Edman T.K., Chan Rickjason C.W., Tsang Dominic N.C.
EPI_ISL_637024	Pamela Youde Nethersole Eastern Hospital	Hong Kong Department of Health	Mak Gannon C.K., Lam Edman T.K., Chan Rickjason C.W., Tsang Dominic N.C.
EPI_ISL_637025	Queen Elizabeth Hospital	Hong Kong Department of Health	Mak Gannon C.K., Lam Edman T.K., Chan Rickjason C.W., Tsang Dominic N.C.
EPI_ISL_637026	Kwong Wah Hospital	Hong Kong Department of Health	Mak Gannon C.K., Lam Edman T.K., Chan Rickjason C.W., Tsang Dominic N.C.
EPI_ISL_637027	Hong Kong Baptist Hospital	Hong Kong Department of Health	Mak Gannon C.K., Lam Edman T.K., Chan Rickjason C.W., Tsang Dominic N.C.
EPI_ISL_637028	Queen Mary Hospital	Hong Kong Department of Health	Mak Gannon C.K., Lam Edman T.K., Chan Rickjason C.W., Tsang Dominic N.C.
EPI_ISL_637029	Caritas Medical Centre	Hong Kong Department of Health	Mak Gannon C.K., Lam Edman T.K., Chan Rickjason C.W., Tsang Dominic N.C.
EPI_ISL_637030, EPI_ISL_637031	Prince of Wales Hospital	Hong Kong Department of Health	Mak Gannon C.K., Lam Edman T.K., Chan Rickjason C.W., Tsang Dominic N.C.
EPI_ISL_637032	Private medical practitioner	Hong Kong Department of Health	Mak Gannon C.K., Lam Edman T.K., Chan Rickjason C.W., Tsang Dominic N.C.
EPI_ISL_637033	Chun Yeung Estate Quarantine Center	Hong Kong Department of Health	Mak Gannon C.K., Lam Edman T.K., Chan Rickjason C.W., Tsang Dominic N.C.
EPI_ISL_637034	Queen Elizabeth Hospital	Hong Kong Department of Health	Mak Gannon C.K., Lam Edman T.K., Chan Rickjason C.W., Tsang Dominic N.C.
EPI_ISL_637035	Princess Margaret Hospital	Hong Kong Department of Health	Mak Gannon C.K., Lam Edman T.K., Chan Rickjason C.W., Tsang Dominic N.C.
EPI_ISL_637036	Tseung Kwan O Hospital	Hong Kong Department of Health	Mak Gannon C.K., Lam Edman T.K., Chan Rickjason C.W., Tsang Dominic N.C.
EPI_ISL_637037, EPI_ISL_637038, EPI_ISL_637039	Queen Elizabeth Hospital	Hong Kong Department of Health	Mak Gannon C.K., Lam Edman T.K., Chan Rickjason C.W., Tsang Dominic N.C.
EPI_ISL_637040	Prince of Wales Hospital	Hong Kong Department of Health	Mak Gannon C.K., Lam Edman T.K., Chan Rickjason C.W., Tsang Dominic N.C.
EPI_ISL_637041	Private medical practitioner	Hong Kong Department of Health	Mak Gannon C.K., Lam Edman T.K., Chan Rickjason C.W., Tsang Dominic N.C.
EPI_ISL_637042	Prince of Wales Hospital	Hong Kong Department of Health	Mak Gannon C.K., Lam Edman T.K., Chan Rickjason C.W., Tsang Dominic N.C.
EPI_ISL_637043	Temporary Specimen Collection Centre at the AsiaWorld-Expo	Hong Kong Department of Health	Mak Gannon C.K., Lam Edman T.K., Chan Rickjason C.W., Tsang Dominic N.C.
EPI_ISL_648217	SILAB	Laboratorio Mixto de Biotecnología Acuática (LMBA)	Joaquín Ezpeleta, Ignacio García Labari, Victoria Posner, Vanina Villanova, Pablo Casal, Sofía Lavista Llanos, Federico Remes Lenicov, Ana Paletta, Flavio Spetale, Agustina Cerri, Silvana Spinelli, Elisa Bolatti, Diego Chouhy, María Re, Gastón Viarengo, Ana Cavatorta, Julian Acosta, Javier Murillo, Laura Angelone, Leandro Ciappina, Pilar Bulacio, Adriana Giri, Silvia Arranz, Elizabeth Tapia
EPI_ISL_648678, EPI_ISL_648679, EPI_ISL_648680, EPI_ISL_648681, EPI_ISL_648682, EPI_ISL_648683, EPI_ISL_648684, EPI_ISL_648685, EPI_ISL_648686, EPI_ISL_648687, EPI_ISL_648688, EPI_ISL_648689, EPI_ISL_648690, EPI_ISL_648691, EPI_ISL_648692, EPI_ISL_648693, EPI_ISL_648694, EPI_ISL_648695, EPI_ISL_648696, EPI_ISL_648697, EPI_ISL_648698, EPI_ISL_648699, EPI_ISL_648700, EPI_ISL_648701, EPI_ISL_648702, EPI_ISL_648703, EPI_ISL_648704, EPI_ISL_648705, EPI_ISL_648706, EPI_ISL_648707, EPI_ISL_648708, EPI_ISL_648709, EPI_ISL_648710, EPI_ISL_648711, EPI_ISL_648712, EPI_ISL_648713, EPI_ISL_648714, EPI_ISL_648715, EPI_ISL_648716, EPI_ISL_648717, EPI_ISL_648718, EPI_ISL_648719, EPI_ISL_648720, EPI_ISL_648721, EPI_ISL_648722, EPI_ISL_648723, EPI_ISL_648724, EPI_ISL_648725, EPI_ISL_648726, EPI_ISL_648727, EPI_ISL_648728, EPI_ISL_648729, EPI_ISL_648730, EPI_ISL_648731, EPI_ISL_648732, EPI_ISL_648733, EPI_ISL_648734, EPI_ISL_648735, EPI_ISL_648736, EPI_ISL_648737, EPI_ISL_648738, EPI_ISL_648739, EPI_ISL_648740, EPI_ISL_648741, EPI_ISL_648792, EPI_ISL_648793, EPI_ISL_648794, EPI_ISL_648795, EPI_ISL_648796, EPI_ISL_648797, EPI_ISL_648798, EPI_ISL_648799, EPI_ISL_648800, EPI_ISL_648801, EPI_ISL_648802, EPI_ISL_648803, EPI_ISL_648804, EPI_ISL_648805, EPI_ISL_648806, EPI_ISL_648807, EPI_ISL_648808, EPI_ISL_648809, EPI_ISL_648810, EPI_ISL_648811, EPI_ISL_648812, EPI_ISL_648813, EPI_ISL_648814, EPI_ISL_648815, EPI_ISL_648816, EPI_ISL_648817, EPI_ISL_648818, EPI_ISL_648819, EPI_ISL_648820			
see above	Department of Laboratory Medicine, Tan Tock Seng Hospital	Department of Laboratory Medicine, Tan Tock Seng Hospital	Chen YYC, Zair X, Lim JX, Li C, Tang WY, Maurer-Stroh S, Barkham TMS, Nagarajan N, Sessions OM
EPI_ISL_649154	Queen Astrid Military Hospital	Institute of Tropical Medicine	Philippe Selhorst, Colin Anthony
EPI_ISL_649190	Istituto Zooprofilattico Sperimentale della Puglia e della Basilicata	Istituto Zooprofilattico Sperimentale della Puglia e della Basilicata	Parisi A., Bianco A., Capozzi L., Del Sambro L., Manzulli V, Rondinone V., Pace L., Galante D., Cipolletta D.
EPI_ISL_649191	Istituto Zooprofilattico Sperimentale della Puglia e della Basilicata	Istituto Zooprofilattico Sperimentale della Puglia e della Basilicata	Parisi A., Bianco A., Capozzi L., Del Sambro L., Manzulli V, Rondinone V., Pace L., Cipolletta D., Galante D.
EPI_ISL_649938	I.R.C.C.S. "S. De Bellis" - Ente Ospedaliero	Istituto Zooprofilattico Sperimentale della Puglia e della Basilicata	Parisi A., Bianco A., Capozzi L., Del Sambro L., Lippolis A., Notarnicola M., Cipolletta D., Galante D.
EPI_ISL_649939	I.R.C.C.S. "S. De Bellis" - Ente Ospedaliero	Istituto Zooprofilattico Sperimentale della Puglia e della Basilicata	Parisi A., Bianco A., Capozzi L., Del Sambro L., Lippolis A., Notarnicola M., Manzulli V, Rondinone V., Pace L.
EPI_ISL_649940	Istituto Zooprofilattico Sperimentale della Puglia e della Basilicata	Istituto Zooprofilattico Sperimentale della Puglia e della Basilicata	Parisi A., Bianco A., Capozzi L., Del Sambro L., Manzulli V, Rondinone V., Pace L., Cipolletta D., Galante D.
EPI_ISL_653746, EPI_ISL_653747, EPI_ISL_653755, EPI_ISL_653756, EPI_ISL_653757	Instituto Nacional de Salud, Bogotá, Colombia	Instituto Nacional de Salud, Bogotá, Colombia	Katherine Laiton-Donato, Diego A. Álvarez-Díaz, Carlos Franco-Muñoz, Mauricio Pacheco-Montealegre, Jonathan Reales, Diego Andrés Prada, Jose A. Usme-Ciro, Zulma M. Cucunubá, Christian Julian VillabonaArenas, Liz Villabona-Arenas, Sussy Echeverria, Astrid C. Flórez, Carolina Ferro, Diana Marcela Walteros-Acero, Franklin Prieto, Carlos Andrés Durán, Martha Lucia Ospina Martinez, Marcela Mercado-Reyes
EPI_ISL_653763, EPI_ISL_653764	I.R.C.C.S. "S. De Bellis" - Ente Ospedaliero	Istituto Zooprofilattico Sperimentale della Puglia e della Basilicata	Parisi A., Bianco A., Capozzi L., Del Sambro L., Lippolis A., Notarnicola M., Manzulli V, Rondinone V., Pace L.
EPI_ISL_653823	I.R.C.C.S. "S. De Bellis" - Ente Ospedaliero	Istituto Zooprofilattico Sperimentale della Puglia e della Basilicata	Parisi A., Bianco A., Capozzi L., Del Sambro L., Lippolis A., Notarnicola M., Cipolletta D., Galante D.
EPI_ISL_653922, EPI_ISL_653923, EPI_ISL_653924, EPI_ISL_653925, EPI_ISL_653926, EPI_ISL_653927, EPI_ISL_653928, EPI_ISL_653929, EPI_ISL_653930, EPI_ISL_653931, EPI_ISL_653932			
see above	Molecular diagnostic laboratory of Federal Budget Institution of Science "Central Research Institute of Epidemiology" of The Federal Service on Customers' Rights Protection and Human Well-being Surveillance	Group of Genomics and Postgenomic Technologies of Central Research Institute of Epidemilology	Samoilov AE, Kaptelova VV, Dudorova A.V., Speranskaya AS, Tivanova EV, Shipulina OY, Akimkin VG
EPI_ISL_654818	Department of Respiratory and other Viral Infections of L.V.Gromashevsky Institute of Epidemiology & Infectious Diseases NAMS of Ukrain	Department of Respiratory and other Viral Infections of L.V.Gromashevsky Institute of Epidemiology & Infectious Diseases NAMS of Ukrain, JSC "Farmak"	Alla Mironenko, Andriy Goy, Ihor Kravchuk, Ludmyla Bolotova, Larysa Radchenko, Nataliia Teteriuk
EPI_ISL_654819, EPI_ISL_654820	Department of Respiratory and other Viral Infections of L.V.Gromashevsky Institute of Epidemiology & Infectious	Department of Respiratory and other Viral Infections of L.V.Gromashevsky Institute of Epidemiology & Infectious	Alla Mironenko, Andriy Goy, Ihor Kravchuk, Ludmyla Bolotova, Larysa Radchenko, Nataliia Teteriuk

	Diseases NAMS of Ukrain	Diseases NAMS of Ukrain, JSC "Farmak"	
EPI_ISL_658896	Instituto de Diagnostico y Referencia Epidemiologicos (INDRE)	Instituto de Diagnostico y Referencia Epidemiologicos (INDRE)	Ernesto Ramirez-Gonzalez, Abril Rodriguez-Maldonado, Claudia Wong-Arambula , Natividad Cruz-Ortiz, Tatiana Nunez-Garcia, Dayanira Arellano-Suarez, Fabiola Garces-Ayala, Lucia Hernandez-Rivas, Irma Lopez-Martinez, Gisela Barrera-Badillo.
EPI_ISL_660069	Zurita & Zurita Laboratorios	Zurita & Zurita Laboratorios	Gabriela Sevillano Camilo Zurita-Salinas Karen Loaiza David Ortega-Paredes Jeannete Zurita
EPI_ISL_660109, EPI_ISL_660110	Division of Infectious Diseases, Department of Internal Medicine, Yonsei University College of Medicine	Department of biotechnology, Yonsei University	Minwoo Kim, Youn-Jung Lee, Jae sun Yoon, Jin Young Ahn, Jung Ho Kim, Jun Yong Choi*, and Jong-Won Oh*
EPI_ISL_660165, EPI_ISL_660166, EPI_ISL_660168, EPI_ISL_660169, EPI_ISL_660171, EPI_ISL_660172, EPI_ISL_660173, EPI_ISL_660174, EPI_ISL_660175, EPI_ISL_660176, EPI_ISL_660181			
see above	NHLS-IALCH	KRISP, KZN Research Innovation and Sequencing Platform	Gazy I, Sigal A, Karim F, Cele S, Giandhari J, Pillay S, Tegally H, Wilkinson E, de Oliveira T
EPI_ISL_660435, EPI_ISL_660436, EPI_ISL_660437, EPI_ISL_660438	Molecular diagnostic laboratory of Federal Budget Institution of Science "Central Research Institute of Epidemiology" of The Federal Service on Customers' Rights Protection and Human Well-being Surveillance	Group of Genomics and Postgenomic Technologies of Central Research Institute of Epidemilology	Samoilov AE, Kaptelova VV, Valdokhina AV, Bulanenko VP, Speranskaya AS, Tivanova EV, Shipulina OY, Akimkin VG
EPI_ISL_671419, EPI_ISL_671420, EPI_ISL_671421, EPI_ISL_671423, EPI_ISL_671424, EPI_ISL_671425, EPI_ISL_671427, EPI_ISL_671429, EPI_ISL_671437	University of Debrecen, Department of Medical Microbiology	National Laboratory of Virology, Szentágothai Research Centre	Endre Gábor Tóth, Balázs Somogyi, Brigitta Zana, Eszter Csoma, Ferenc Jakab, Gábor Kemenesi
EPI_ISL_677728, EPI_ISL_677729, EPI_ISL_677730, EPI_ISL_677731, EPI_ISL_677732, EPI_ISL_677733, EPI_ISL_677734, EPI_ISL_677735, EPI_ISL_677736, EPI_ISL_677737, EPI_ISL_677738			
see above	University of Szeged, Institute of Clinical Microbiology	National Laboratory of Virology, Szentágothai Research Centre	Endre Gábor Tóth, Balázs Somogyi, Brigitta, Gabriella Terhes, Ferenc Jakab, Gábor Kemenesi
EPI_ISL_682242	HOSPITAL SAN VICENTE DE PAUL	Inciensa, Instituto Costarricense de Investigación y Enseñanza en Nutrición y Salud	Francisco Duarte, Hebleen Porras, Claudio Soto-Garita, Estela Cordero, Adriana Godinez, Melany Calderon & Mariel López
EPI_ISL_682250, EPI_ISL_682252	AREA DE SALUD ESCAZU (COOPESANA)	Inciensa, Instituto Costarricense de Investigación y Enseñanza en Nutrición y Salud	Francisco Duarte, Hebleen Porras, Claudio Soto-Garita, Estela Cordero, Adriana Godinez & Melany Calderon
EPI_ISL_682255	HOSPITAL SAN VICENTE DE PAUL	Inciensa, Instituto Costarricense de Investigación y Enseñanza en Nutrición y Salud	Francisco Duarte, Hebleen Porras, Claudio Soto-Garita, Estela Cordero, Adriana Godinez & Melany Calderon
EPI_ISL_682257	HOSPITAL SAN JUAN DE DIOS	Inciensa, Instituto Costarricense de Investigación y Enseñanza en Nutrición y Salud	Francisco Duarte, Hebleen Porras, Claudio Soto-Garita, Estela Cordero, Adriana Godinez & Melany Calderon
EPI_ISL_682261	TAMIZAJE COMUNITARIO- PASO CANOAS	Inciensa, Instituto Costarricense de Investigación y Enseñanza en Nutrición y Salud	Francisco Duarte, Hebleen Porras, Claudio Soto-Garita, Estela Cordero, Adriana Godinez & Melany Calderon
EPI_ISL_682262, EPI_ISL_682264, EPI_ISL_682266, EPI_ISL_682273	HOSPITAL SAN JUAN DE DIOS	Inciensa, Instituto Costarricense de Investigación y Enseñanza en Nutrición y Salud	Francisco Duarte, Hebleen Porras, Claudio Soto-Garita, Estela Cordero, Adriana Godinez & Melany Calderon
EPI_ISL_685731, EPI_ISL_685732, EPI_ISL_685910, EPI_ISL_685917, EPI_ISL_685938, EPI_ISL_685939, EPI_ISL_685940, EPI_ISL_685941, EPI_ISL_685942, EPI_ISL_685943, EPI_ISL_685944, EPI_ISL_685945, EPI_ISL_685946, EPI_ISL_685948, EPI_ISL_685951, EPI_ISL_685952			
see above	Pathogen Genomics Center, National Institute of Infectious Diseases	Pathogen Genomics Center, National Institute of Infectious Diseases	Tsuyoshi Sekizuka, Kentaro Itokawa, Rina Tanaka, Masanori Hashino, Makoto Kuroda
EPI_ISL_685963, EPI_ISL_685964, EPI_ISL_685965, EPI_ISL_685966, EPI_ISL_685967, EPI_ISL_685968, EPI_ISL_685969, EPI_ISL_685970, EPI_ISL_685971, EPI_ISL_685972, EPI_ISL_685973, EPI_ISL_685974, EPI_ISL_685975, EPI_ISL_685976, EPI_ISL_685977, EPI_ISL_685978, EPI_ISL_685979, EPI_ISL_685980, EPI_ISL_685981, EPI_ISL_685982, EPI_ISL_685983, EPI_ISL_685984, EPI_ISL_685985, EPI_ISL_685986			
see above	Tokyo Metropolitan Institute of Public Health	Pathogen Genomics Center, National Institute of Infectious Diseases	Tsuyoshi Sekizuka, Kentaro Itokawa, Rina Tanaka, Masanori Hashino, Makoto Kuroda
EPI_ISL_686076, EPI_ISL_686077, EPI_ISL_686078, EPI_ISL_686079, EPI_ISL_686080, EPI_ISL_686081, EPI_ISL_686082, EPI_ISL_686109, EPI_ISL_686110, EPI_ISL_686111, EPI_ISL_686112, EPI_ISL_686113, EPI_ISL_686114, EPI_ISL_686115, EPI_ISL_686116, EPI_ISL_686117, EPI_ISL_686118, EPI_ISL_686119, EPI_ISL_686120, EPI_ISL_686121, EPI_ISL_686122, EPI_ISL_686123, EPI_ISL_686124, EPI_ISL_686125, EPI_ISL_686126, EPI_ISL_686127, EPI_ISL_686128, EPI_ISL_686129, EPI_ISL_686130, EPI_ISL_686131, EPI_ISL_686132, EPI_ISL_686133, EPI_ISL_686134, EPI_ISL_686135, EPI_ISL_686136, EPI_ISL_686137, EPI_ISL_686138, EPI_ISL_686139, EPI_ISL_686140, EPI_ISL_686141, EPI_ISL_686142, EPI_ISL_686143, EPI_ISL_686144, EPI_ISL_686145, EPI_ISL_686146, EPI_ISL_686147, EPI_ISL_686148, EPI_ISL_686149, EPI_ISL_686150, EPI_ISL_686151, EPI_ISL_686152, EPI_ISL_686153, EPI_ISL_686154, EPI_ISL_686155, EPI_ISL_686156, EPI_ISL_686157, EPI_ISL_686158, EPI_ISL_686159, EPI_ISL_686160, EPI_ISL_686161, EPI_ISL_686162, EPI_ISL_686163, EPI_ISL_686164, EPI_ISL_686165, EPI_ISL_686166, EPI_ISL_686167, EPI_ISL_686168, EPI_ISL_686169, EPI_ISL_686170, EPI_ISL_686171, EPI_ISL_686172, EPI_ISL_686173, EPI_ISL_686174, EPI_ISL_686175, EPI_ISL_686176, EPI_ISL_686177, EPI_ISL_686178, EPI_ISL_686179, EPI_ISL_686180, EPI_ISL_686181, EPI_ISL_686182, EPI_ISL_686183, EPI_ISL_686184, EPI_ISL_686185, EPI_ISL_686186, EPI_ISL_686187, EPI_ISL_686188, EPI_ISL_686189, EPI_ISL_686191, EPI_ISL_686192, EPI_ISL_686193, EPI_ISL_686194, EPI_ISL_686195, EPI_ISL_686196, EPI_ISL_686197, EPI_ISL_686198, EPI_ISL_686199, EPI_ISL_686200, EPI_ISL_686201, EPI_ISL_686202, EPI_ISL_686203, EPI_ISL_686204, EPI_ISL_686205, EPI_ISL_686206, EPI_ISL_686207, EPI_ISL_686208, EPI_ISL_686209, EPI_ISL_686210, EPI_ISL_686211, EPI_ISL_686212, EPI_ISL_686213, EPI_ISL_686215, EPI_ISL_686216, EPI_ISL_686223, EPI_ISL_686224, EPI_ISL_686225, EPI_ISL_686226, EPI_ISL_686227, EPI_ISL_686228, EPI_ISL_686283, EPI_ISL_686284, EPI_ISL_686285, EPI_ISL_686286, EPI_ISL_686287, EPI_ISL_686288, EPI_ISL_686289, EPI_ISL_686290, EPI_ISL_686291, EPI_ISL_686292, EPI_ISL_686293, EPI_ISL_686294, EPI_ISL_686295, EPI_ISL_686296, EPI_ISL_686297, EPI_ISL_686298, EPI_ISL_686299, EPI_ISL_686301, EPI_ISL_686302, EPI_ISL_686303, EPI_ISL_686304, EPI_ISL_686305, EPI_ISL_686306, EPI_ISL_686307, EPI_ISL_686308, EPI_ISL_686309, EPI_ISL_686310, EPI_ISL_686311, EPI_ISL_686312, EPI_ISL_686313, EPI_ISL_686314, EPI_ISL_686315, EPI_ISL_686316, EPI_ISL_686317, EPI_ISL_686318, EPI_ISL_686319, EPI_ISL_686320, EPI_ISL_686321, EPI_ISL_686322, EPI_ISL_686323, EPI_ISL_686324, EPI_ISL_686325, EPI_ISL_686326, EPI_ISL_686327, EPI_ISL_686328, EPI_ISL_686329, EPI_ISL_686330, EPI_ISL_686331, EPI_ISL_686332, EPI_ISL_686333, EPI_ISL_686334, EPI_ISL_686335, EPI_ISL_686367, EPI_ISL_686368, EPI_ISL_686369, EPI_ISL_686370, EPI_ISL_686371, EPI_ISL_686372, EPI_ISL_686373, EPI_ISL_686374, EPI_ISL_686375, EPI_ISL_686376, EPI_ISL_686377, EPI_ISL_686378, EPI_ISL_686379, EPI_ISL_686380, EPI_ISL_686381, EPI_ISL_686382, EPI_ISL_686383, EPI_ISL_686384, EPI_ISL_686385, EPI_ISL_686386, EPI_ISL_686387, EPI_ISL_686388, EPI_ISL_686389, EPI_ISL_686390, EPI_ISL_686391, EPI_ISL_686392, EPI_ISL_686393, EPI_ISL_686394, EPI_ISL_686395, EPI_ISL_686396, EPI_ISL_686397, EPI_ISL_686398, EPI_ISL_686399, EPI_ISL_686400, EPI_ISL_686401, EPI_ISL_686402, EPI_ISL_686403, EPI_ISL_686404, EPI_ISL_686405, EPI_ISL_686406, EPI_ISL_686407, EPI_ISL_686408, EPI_ISL_686410, EPI_ISL_686411, EPI_ISL_686412, EPI_ISL_686413, EPI_ISL_686414, EPI_ISL_686415, EPI_ISL_686418, EPI_ISL_686419, EPI_ISL_686420, EPI_ISL_686421, EPI_ISL_686422, EPI_ISL_686423, EPI_ISL_686424, EPI_ISL_686425, EPI_ISL_686426, EPI_ISL_686427, EPI_ISL_686429, EPI_ISL_686430, EPI_ISL_686431, EPI_ISL_686432, EPI_ISL_686433, EPI_ISL_686434, EPI_ISL_686435, EPI_ISL_686436, EPI_ISL_686437, EPI_ISL_686438, EPI_ISL_686439, EPI_ISL_686440, EPI_ISL_686441, EPI_ISL_686442, EPI_ISL_686443, EPI_ISL_686444, EPI_ISL_686445, EPI_ISL_686446, EPI_ISL_686447, EPI_ISL_686469, EPI_ISL_686470, EPI_ISL_686471, EPI_ISL_686472, EPI_ISL_686473, EPI_ISL_686474, EPI_ISL_686475, EPI_ISL_686476, EPI_ISL_686477, EPI_ISL_686478, EPI_ISL_686479, EPI_ISL_686480, EPI_ISL_686481, EPI_ISL_686482, EPI_ISL_686483, EPI_ISL_686484, EPI_ISL_686485, EPI_ISL_686486, EPI_ISL_686487, EPI_ISL_686488, EPI_ISL_686489, EPI_ISL_686490, EPI_ISL_686491, EPI_ISL_686492, EPI_ISL_686493			
see above	Pathogen Genomics Center, National Institute of Infectious Diseases	Pathogen Genomics Center, National Institute of Infectious Diseases	Tsuyoshi Sekizuka, Kentaro Itokawa, Rina Tanaka, Masanori Hashino, Makoto Kuroda
EPI_ISL_686521	Sakai City Institute of Public Health	Pathogen Genomics Center, National Institute of Infectious Diseases	Tsuyoshi Sekizuka, Kentaro Itokawa, Rina Tanaka, Masanori Hashino, Makoto Kuroda
EPI_ISL_686612, EPI_ISL_686613, EPI_ISL_686614, EPI_ISL_686615, EPI_ISL_686616, EPI_ISL_686617, EPI_ISL_686618, EPI_ISL_686619, EPI_ISL_686620, EPI_ISL_686621, EPI_ISL_686622, EPI_ISL_686623, EPI_ISL_686624, EPI_ISL_686625, EPI_ISL_686626, EPI_ISL_686627, EPI_ISL_686628, EPI_ISL_686629, EPI_ISL_686630, EPI_ISL_686631, EPI_ISL_686632, EPI_ISL_686633, EPI_ISL_686634, EPI_ISL_686635, EPI_ISL_686636, EPI_ISL_686637, EPI_ISL_686638, EPI_ISL_686639, EPI_ISL_686640, EPI_ISL_686641, EPI_ISL_686642, EPI_ISL_686643, EPI_ISL_686644, EPI_ISL_686645, EPI_ISL_686646, EPI_ISL_686647, EPI_ISL_686648, EPI_ISL_686649, EPI_ISL_686650, EPI_ISL_686651, EPI_ISL_686652, EPI_ISL_686653			
see above	Pathogen Genomics Center, National Institute of Infectious Diseases	Pathogen Genomics Center, National Institute of Infectious Diseases	Tsuyoshi Sekizuka, Kentaro Itokawa, Rina Tanaka, Masanori Hashino, Makoto Kuroda
EPI_ISL_686659, EPI_ISL_686660, EPI_ISL_686661, EPI_ISL_686662, EPI_ISL_686663, EPI_ISL_686664, EPI_ISL_686665, EPI_ISL_686666, EPI_ISL_686667, EPI_ISL_686668, EPI_ISL_686669, EPI_ISL_686670, EPI_ISL_686671, EPI_ISL_686672, EPI_ISL_686673, EPI_ISL_686674, EPI_ISL_686675, EPI_ISL_686676, EPI_ISL_686677, EPI_ISL_686678, EPI_ISL_686679, EPI_ISL_686680, EPI_ISL_686681, EPI_ISL_686682, EPI_ISL_686683, EPI_ISL_686684, EPI_ISL_686685, EPI_ISL_686686, EPI_ISL_686687, EPI_ISL_686688, EPI_ISL_686689, EPI_ISL_686690, EPI_ISL_686691, EPI_ISL_686692, EPI_ISL_686693, EPI_ISL_686694, EPI_ISL_686695, EPI_ISL_686696, EPI_ISL_686697, EPI_ISL_686698, EPI_ISL_686699, EPI_ISL_686700, EPI_ISL_686701, EPI_ISL_686702, EPI_ISL_686703, EPI_ISL_686704, EPI_ISL_686705, EPI_ISL_686706, EPI_ISL_686707, EPI_ISL_686708, EPI_ISL_686709, EPI_ISL_686710, EPI_ISL_686711, EPI_ISL_686712, EPI_ISL_686713, EPI_ISL_686714, EPI_ISL_686715, EPI_ISL_686716, EPI_ISL_686717, EPI_ISL_686718, EPI_ISL_686719, EPI_ISL_686720, EPI_ISL_686721, EPI_ISL_686722, EPI_ISL_686723, EPI_ISL_686724, EPI_ISL_686725, EPI_ISL_686726, EPI_ISL_686727, EPI_ISL_686728, EPI_ISL_686729, EPI_ISL_686730, EPI_ISL_686731, EPI_ISL_686732, EPI_ISL_686733, EPI_ISL_686734, EPI_ISL_686735, EPI_ISL_686736, EPI_ISL_686737, EPI_ISL_686738, EPI_ISL_686739, EPI_ISL_686740, EPI_ISL_686741, EPI_ISL_686742, EPI_ISL_686743, EPI_ISL_686744, EPI_ISL_686745, EPI_ISL_686746, EPI_ISL_686747, EPI_ISL_686748, EPI_ISL_686749, EPI_ISL_686750, EPI_ISL_686751, EPI_ISL_686752, EPI_ISL_686753, EPI_ISL_686754, EPI_ISL_686755, EPI_ISL_686756, EPI_ISL_686757, EPI_ISL_686758, EPI_ISL_686759, EPI_ISL_686760, EPI_ISL_686761, EPI_ISL_686762, EPI_ISL_686763, EPI_ISL_686764, EPI_ISL_686765, EPI_ISL_686766, EPI_ISL_686767, EPI_ISL_686768, EPI_ISL_686769, EPI_ISL_686770, EPI_ISL_686771, EPI_ISL_686772, EPI_ISL_686773, EPI_ISL_686774, EPI_ISL_686775, EPI_ISL_686776, EPI_ISL_686777, EPI_ISL_686778, EPI_ISL_686779, EPI_ISL_686780, EPI_ISL_686781, EPI_ISL_686782, EPI_ISL_686783, EPI_ISL_686784, EPI_ISL_686785, EPI_ISL_686786, EPI_ISL_686787, EPI_ISL_686788, EPI_ISL_686789, EPI_ISL_686790, EPI_ISL_686791, EPI_ISL_686792, EPI_ISL_686793, EPI_ISL_686794, EPI_ISL_686795, EPI_ISL_686796, EPI_ISL_686797, EPI_ISL_686798, EPI_ISL_686799, EPI_ISL_686800, EPI_ISL_686801, EPI_ISL_686802, EPI_ISL_686803, EPI_ISL_686804, EPI_ISL_686805, EPI_ISL_686806, EPI_ISL_686807, EPI_ISL_686808, EPI_ISL_686809, EPI_ISL_686810, EPI_ISL_686811, EPI_ISL_686812, EPI_ISL_686813, EPI_ISL_686814, EPI_ISL_686815, EPI_ISL_686816, EPI_ISL_686817, EPI_ISL_686818, EPI_ISL_686819, EPI_ISL_686820, EPI_ISL_686821, EPI_ISL_686822, EPI_ISL_686823, EPI_ISL_686824, EPI_ISL_686825, EPI_ISL_686826, EPI_ISL_686827, EPI_ISL_686828, EPI_ISL_686829, EPI_ISL_686830			

see above	Chiba Prefectural Institute of Public Health	Pathogen Genomics Center, National Institute of Infectious Diseases	Tsuyoshi Sekizuka, Kentaro Itokawa, Rina Tanaka, Masanori Hashino, Makoto Kuroda
EPI_ISL_686874, EPI_ISL_686875, EPI_ISL_686876, EPI_ISL_686877, EPI_ISL_686878, EPI_ISL_686879, EPI_ISL_686880, EPI_ISL_686881, EPI_ISL_686882, EPI_ISL_686883, EPI_ISL_686884, EPI_ISL_686885, EPI_ISL_686886, EPI_ISL_686887, EPI_ISL_686888, EPI_ISL_686889, EPI_ISL_686890, EPI_ISL_686891, EPI_ISL_686892, EPI_ISL_686893, EPI_ISL_686894, EPI_ISL_686895, EPI_ISL_686896, EPI_ISL_686897, EPI_ISL_686898, EPI_ISL_686899, EPI_ISL_686900, EPI_ISL_686901, EPI_ISL_686902, EPI_ISL_686903, EPI_ISL_686904, EPI_ISL_686905, EPI_ISL_686906, EPI_ISL_686907, EPI_ISL_686908, EPI_ISL_686909, EPI_ISL_686910, EPI_ISL_686911, EPI_ISL_686912, EPI_ISL_686913, EPI_ISL_686914, EPI_ISL_686915, EPI_ISL_686916, EPI_ISL_686917, EPI_ISL_686918, EPI_ISL_686919, EPI_ISL_686920, EPI_ISL_686921, EPI_ISL_686922, EPI_ISL_686923, EPI_ISL_686924, EPI_ISL_686925, EPI_ISL_686926, EPI_ISL_686927, EPI_ISL_686928, EPI_ISL_686929, EPI_ISL_686930, EPI_ISL_686931, EPI_ISL_686932, EPI_ISL_686933, EPI_ISL_686934, EPI_ISL_686935, EPI_ISL_686936, EPI_ISL_686937, EPI_ISL_686938, EPI_ISL_686939, EPI_ISL_686940, EPI_ISL_686941, EPI_ISL_686942, EPI_ISL_686943, EPI_ISL_686944, EPI_ISL_686945, EPI_ISL_686946, EPI_ISL_686947, EPI_ISL_686948, EPI_ISL_686949, EPI_ISL_686950, EPI_ISL_686951, EPI_ISL_686952, EPI_ISL_686953, EPI_ISL_686954, EPI_ISL_686955, EPI_ISL_686956, EPI_ISL_686957, EPI_ISL_686958, EPI_ISL_686959, EPI_ISL_686960, EPI_ISL_686961, EPI_ISL_686962, EPI_ISL_686963, EPI_ISL_686964, EPI_ISL_686965, EPI_ISL_686966, EPI_ISL_686967, EPI_ISL_686968, EPI_ISL_686969, EPI_ISL_686970, EPI_ISL_686971, EPI_ISL_686972, EPI_ISL_686973, EPI_ISL_686974, EPI_ISL_686975, EPI_ISL_686976, EPI_ISL_686977, EPI_ISL_686978, EPI_ISL_686979, EPI_ISL_686980, EPI_ISL_686981, EPI_ISL_686982, EPI_ISL_686983, EPI_ISL_686984, EPI_ISL_686985, EPI_ISL_686986, EPI_ISL_686987, EPI_ISL_686988, EPI_ISL_686989, EPI_ISL_686990, EPI_ISL_686991, EPI_ISL_686992, EPI_ISL_686993, EPI_ISL_686994, EPI_ISL_686995, EPI_ISL_686996, EPI_ISL_686997, EPI_ISL_686998, EPI_ISL_686999, EPI_ISL_687000, EPI_ISL_687001, EPI_ISL_687002, EPI_ISL_687003, EPI_ISL_687004, EPI_ISL_687005, EPI_ISL_687006, EPI_ISL_687007, EPI_ISL_687008, EPI_ISL_687009, EPI_ISL_687010, EPI_ISL_687011, EPI_ISL_687012, EPI_ISL_687013, EPI_ISL_687014, EPI_ISL_687015, EPI_ISL_687016, EPI_ISL_687017, EPI_ISL_687018, EPI_ISL_687019, EPI_ISL_687020, EPI_ISL_687021, EPI_ISL_687022, EPI_ISL_687023, EPI_ISL_687024, EPI_ISL_687025, EPI_ISL_687026, EPI_ISL_687027, EPI_ISL_687028, EPI_ISL_687029, EPI_ISL_687030, EPI_ISL_687031, EPI_ISL_687032, EPI_ISL_687033, EPI_ISL_687034, EPI_ISL_687035, EPI_ISL_687036, EPI_ISL_687037, EPI_ISL_687038, EPI_ISL_687039, EPI_ISL_687040, EPI_ISL_687041, EPI_ISL_687042			
see above	Pathogen Genomics Center, National Institute of Infectious Diseases	Pathogen Genomics Center, National Institute of Infectious Diseases	Tsuyoshi Sekizuka, Kentaro Itokawa, Rina Tanaka, Masanori Hashino, Makoto Kuroda
EPI_ISL_687071, EPI_ISL_687072, EPI_ISL_687073, EPI_ISL_687074, EPI_ISL_687075, EPI_ISL_687076, EPI_ISL_687077, EPI_ISL_687078	Kanagawa Prefectural Institute of Public Health	Pathogen Genomics Center, National Institute of Infectious Diseases	Tsuyoshi Sekizuka, Kentaro Itokawa, Rina Tanaka, Masanori Hashino, Makoto Kuroda
EPI_ISL_687079, EPI_ISL_687080, EPI_ISL_687081, EPI_ISL_687082, EPI_ISL_687083, EPI_ISL_687084, EPI_ISL_687085, EPI_ISL_687086, EPI_ISL_687087, EPI_ISL_687088, EPI_ISL_687089, EPI_ISL_687090, EPI_ISL_687091, EPI_ISL_687092, EPI_ISL_687093, EPI_ISL_687094, EPI_ISL_687095, EPI_ISL_687096, EPI_ISL_687097, EPI_ISL_687098, EPI_ISL_687100, EPI_ISL_687101, EPI_ISL_687102, EPI_ISL_687103, EPI_ISL_687104, EPI_ISL_687105, EPI_ISL_687106, EPI_ISL_687107, EPI_ISL_687108, EPI_ISL_687109, EPI_ISL_687110, EPI_ISL_687111, EPI_ISL_687112, EPI_ISL_687113, EPI_ISL_687114, EPI_ISL_687115, EPI_ISL_687116, EPI_ISL_687117, EPI_ISL_687118, EPI_ISL_687119, EPI_ISL_687120, EPI_ISL_687121, EPI_ISL_687122, EPI_ISL_687123, EPI_ISL_687124, EPI_ISL_687125, EPI_ISL_687126, EPI_ISL_687127, EPI_ISL_687128, EPI_ISL_687129, EPI_ISL_687130, EPI_ISL_687131, EPI_ISL_687132, EPI_ISL_687133, EPI_ISL_687134, EPI_ISL_687135, EPI_ISL_687136, EPI_ISL_687137, EPI_ISL_687139, EPI_ISL_687140, EPI_ISL_687141, EPI_ISL_687142, EPI_ISL_687143, EPI_ISL_687145, EPI_ISL_687146, EPI_ISL_687147, EPI_ISL_687152, EPI_ISL_687153, EPI_ISL_687154, EPI_ISL_687155, EPI_ISL_687156, EPI_ISL_687157, EPI_ISL_687163, EPI_ISL_687164, EPI_ISL_687165, EPI_ISL_687166, EPI_ISL_687172, EPI_ISL_687173, EPI_ISL_687174, EPI_ISL_687177, EPI_ISL_687179, EPI_ISL_687181, EPI_ISL_687182, EPI_ISL_687184, EPI_ISL_687185, EPI_ISL_687188, EPI_ISL_687189, EPI_ISL_687190, EPI_ISL_687191, EPI_ISL_687192, EPI_ISL_687193, EPI_ISL_687194, EPI_ISL_687195, EPI_ISL_687196, EPI_ISL_687197, EPI_ISL_687198, EPI_ISL_687199, EPI_ISL_687200, EPI_ISL_687201, EPI_ISL_687202, EPI_ISL_687203, EPI_ISL_687204, EPI_ISL_687205, EPI_ISL_687206, EPI_ISL_687207, EPI_ISL_687208, EPI_ISL_687209, EPI_ISL_687210, EPI_ISL_687211, EPI_ISL_687212, EPI_ISL_687213, EPI_ISL_687214, EPI_ISL_687215, EPI_ISL_687216, EPI_ISL_687217, EPI_ISL_687218, EPI_ISL_687219, EPI_ISL_687220, EPI_ISL_687221, EPI_ISL_687222, EPI_ISL_687223, EPI_ISL_687224, EPI_ISL_687225, EPI_ISL_687226, EPI_ISL_687227, EPI_ISL_687228, EPI_ISL_687229, EPI_ISL_687230, EPI_ISL_687231, EPI_ISL_687232, EPI_ISL_687233, EPI_ISL_687234, EPI_ISL_687235, EPI_ISL_687236, EPI_ISL_687237, EPI_ISL_687238, EPI_ISL_687239, EPI_ISL_687240, EPI_ISL_687241, EPI_ISL_687242, EPI_ISL_687243, EPI_ISL_687244, EPI_ISL_687245, EPI_ISL_687255, EPI_ISL_687256, EPI_ISL_687257, EPI_ISL_687258, EPI_ISL_687259, EPI_ISL_687260, EPI_ISL_687261, EPI_ISL_687262, EPI_ISL_687263, EPI_ISL_687264, EPI_ISL_687265, EPI_ISL_687266, EPI_ISL_687267, EPI_ISL_687268, EPI_ISL_687269, EPI_ISL_687270, EPI_ISL_687271, EPI_ISL_687272, EPI_ISL_687273, EPI_ISL_687274, EPI_ISL_687275, EPI_ISL_687276, EPI_ISL_687277, EPI_ISL_687278, EPI_ISL_687279, EPI_ISL_687280, EPI_ISL_687281, EPI_ISL_687282, EPI_ISL_687283, EPI_ISL_687284, EPI_ISL_687285, EPI_ISL_687286, EPI_ISL_687287, EPI_ISL_687288, EPI_ISL_687289, EPI_ISL_687290, EPI_ISL_687291, EPI_ISL_687292, EPI_ISL_687293, EPI_ISL_687294, EPI_ISL_687295, EPI_ISL_687296, EPI_ISL_687297, EPI_ISL_687298, EPI_ISL_687299, EPI_ISL_687300, EPI_ISL_687301, EPI_ISL_687302, EPI_ISL_687303, EPI_ISL_687304, EPI_ISL_687305, EPI_ISL_687306, EPI_ISL_687307, EPI_ISL_687308, EPI_ISL_687309, EPI_ISL_687310, EPI_ISL_687311, EPI_ISL_687312, EPI_ISL_687313, EPI_ISL_687314, EPI_ISL_687315, EPI_ISL_687316, EPI_ISL_687317, EPI_ISL_687318, EPI_ISL_687319, EPI_ISL_687320, EPI_ISL_687321, EPI_ISL_687322, EPI_ISL_687323, EPI_ISL_687324, EPI_ISL_687325, EPI_ISL_687326, EPI_ISL_687327, EPI_ISL_687328, EPI_ISL_687329, EPI_ISL_687330, EPI_ISL_687331, EPI_ISL_687332, EPI_ISL_687333, EPI_ISL_687334, EPI_ISL_687335, EPI_ISL_687336, EPI_ISL_687337, EPI_ISL_687338, EPI_ISL_687339, EPI_ISL_687340, EPI_ISL_687341, EPI_ISL_687342, EPI_ISL_687343, EPI_ISL_687344, EPI_ISL_687345, EPI_ISL_687346, EPI_ISL_687347, EPI_ISL_687348, EPI_ISL_687349, EPI_ISL_687350, EPI_ISL_687351, EPI_ISL_687352, EPI_ISL_687353, EPI_ISL_687354, EPI_ISL_687355, EPI_ISL_687356, EPI_ISL_687357, EPI_ISL_687358, EPI_ISL_687359, EPI_ISL_687361, EPI_ISL_687362, EPI_ISL_687363, EPI_ISL_687364, EPI_ISL_687365, EPI_ISL_687366, EPI_ISL_687367, EPI_ISL_687368, EPI_ISL_687369, EPI_ISL_687370, EPI_ISL_687371, EPI_ISL_687372, EPI_ISL_687373, EPI_ISL_687374, EPI_ISL_687375, EPI_ISL_687376, EPI_ISL_687377, EPI_ISL_687378, EPI_ISL_687379, EPI_ISL_687380, EPI_ISL_687381, EPI_ISL_687382, EPI_ISL_687383, EPI_ISL_687384, EPI_ISL_687385, EPI_ISL_687386, EPI_ISL_687387, EPI_ISL_687388, EPI_ISL_687389, EPI_ISL_687390, EPI_ISL_687391, EPI_ISL_687392, EPI_ISL_687393, EPI_ISL_687394, EPI_ISL_687395, EPI_ISL_687396, EPI_ISL_687397, EPI_ISL_687398, EPI_ISL_687399, EPI_ISL_687400, EPI_ISL_687401, EPI_ISL_687402, EPI_ISL_687403, EPI_ISL_687404, EPI_ISL_687405, EPI_ISL_687406, EPI_ISL_687407, EPI_ISL_687408, EPI_ISL_687409, EPI_ISL_687410, EPI_ISL_687411, EPI_ISL_687412, EPI_ISL_687413, EPI_ISL_687414, EPI_ISL_687415, EPI_ISL_687416, EPI_ISL_687417, EPI_ISL_687418, EPI_ISL_687419, EPI_ISL_687420, EPI_ISL_687421, EPI_ISL_687422, EPI_ISL_687423, EPI_ISL_687424, EPI_ISL_687425, EPI_ISL_687426, EPI_ISL_687427, EPI_ISL_687428, EPI_ISL_687429, EPI_ISL_687430, EPI_ISL_687431, EPI_ISL_687432, EPI_ISL_687433, EPI_ISL_687434, EPI_ISL_687435, EPI_ISL_687436, EPI_ISL_687437, EPI_ISL_687438, EPI_ISL_687439, EPI_ISL_687440, EPI_ISL_687441, EPI_ISL_687442, EPI_ISL_687443, EPI_ISL_687444, EPI_ISL_687445, EPI_ISL_687446, EPI_ISL_687447, EPI_ISL_687448, EPI_ISL_687449, EPI_ISL_687450, EPI_ISL_687451, EPI_ISL_687452, EPI_ISL_687453, EPI_ISL_687454, EPI_ISL_687455, EPI_ISL_687456, EPI_ISL_687459, EPI_ISL_687460, EPI_ISL_687461, EPI_ISL_687462, EPI_ISL_687463, EPI_ISL_687464, EPI_ISL_687465, EPI_ISL_687466, EPI_ISL_687467, EPI_ISL_687468, EPI_ISL_687469, EPI_ISL_687470, EPI_ISL_687471, EPI_ISL_687472, EPI_ISL_687473, EPI_ISL_687474, EPI_ISL_687475, EPI_ISL_687476, EPI_ISL_687477, EPI_ISL_687478, EPI_ISL_687479, EPI_ISL_687480, EPI_ISL_687481, EPI_ISL_687482, EPI_ISL_687483, EPI_ISL_687484, EPI_ISL_687485, EPI_ISL_687486, EPI_ISL_687487, EPI_ISL_687488, EPI_ISL_687489, EPI_ISL_687490, EPI_ISL_687491, EPI_ISL_687492, EPI_ISL_687493, EPI_ISL_687494, EPI_ISL_687495, EPI_ISL_687496, EPI_ISL_687497, EPI_ISL_687498, EPI_ISL_687499, EPI_ISL_687500, EPI_ISL_687501, EPI_ISL_687502, EPI_ISL_687503, EPI_ISL_687504, EPI_ISL_687505, EPI_ISL_687506, EPI_ISL_687507, EPI_ISL_687508, EPI_ISL_687509, EPI_ISL_687510, EPI_ISL_687511, EPI_ISL_687512, EPI_ISL_687513, EPI_ISL_687514, EPI_ISL_687515, EPI_ISL_687516, EPI_ISL_687517, EPI_ISL_687518, EPI_ISL_687519, EPI_ISL_687520, EPI_ISL_687521, EPI_ISL_687522, EPI_ISL_687561, EPI_ISL_687562, EPI_ISL_687563, EPI_ISL_687564, EPI_ISL_687565, EPI_ISL_687566, EPI_ISL_687567, EPI_ISL_687568, EPI_ISL_687569, EPI_ISL_687570, EPI_ISL_687572, EPI_ISL_687573, EPI_ISL_687574, EPI_ISL_687575, EPI_ISL_687576, EPI_ISL_687577, EPI_ISL_687578, EPI_ISL_687579, EPI_ISL_687580, EPI_ISL_687581, EPI_ISL_687582, EPI_ISL_687583, EPI_ISL_687584, EPI_ISL_687585, EPI_ISL_687586, EPI_ISL_687587, EPI_ISL_687588, EPI_ISL_687589, EPI_ISL_687590, EPI_ISL_687591, EPI_ISL_687592, EPI_ISL_687593, EPI_ISL_687594, EPI_ISL_687595, EPI_ISL_687596, EPI_ISL_687597, EPI_ISL_687598, EPI_ISL_687599, EPI_ISL_687600, EPI_ISL_687609, EPI_ISL_687610, EPI_ISL_687611, EPI_ISL_687614, EPI_ISL_687616, EPI_ISL_687617			
see above	Pathogen Genomics Center, National Institute of Infectious Diseases	Pathogen Genomics Center, National Institute of Infectious Diseases	Tsuyoshi Sekizuka, Kentaro Itokawa, Rina Tanaka, Masanori Hashino, Makoto Kuroda
EPI_ISL_687619, EPI_ISL_687620	Saitama Prefectural Institute of Public Health	Pathogen Genomics Center, National Institute of Infectious Diseases	Tsuyoshi Sekizuka, Kentaro Itokawa, Rina Tanaka, Masanori Hashino, Makoto Kuroda
EPI_ISL_687624, EPI_ISL_687625, EPI_ISL_687626, EPI_ISL_687627, EPI_ISL_687628, EPI_ISL_687629, EPI_ISL_687630, EPI_ISL_687631, EPI_ISL_687632, EPI_ISL_687633, EPI_ISL_687634, EPI_ISL_687635, EPI_ISL_687636, EPI_ISL_687637, EPI_ISL_687638, EPI_ISL_687639, EPI_ISL_687640, EPI_ISL_687641, EPI_ISL_687642, EPI_ISL_687643, EPI_ISL_687644, EPI_ISL_687645, EPI_ISL_687646, EPI_ISL_687647, EPI_ISL_687648, EPI_ISL_687649, EPI_ISL_687650, EPI_ISL_687651, EPI_ISL_687652, EPI_ISL_687653, EPI_ISL_687654, EPI_ISL_687655, EPI_ISL_687659, EPI_ISL_687660, EPI_ISL_687661, EPI_ISL_687662, EPI_ISL_687663, EPI_ISL_687664, EPI_ISL_687665, EPI_ISL_687666, EPI_ISL_687667, EPI_ISL_687668, EPI_ISL_687673, EPI_ISL_687674, EPI_ISL_687675			
see above	Pathogen Genomics Center, National Institute of Infectious Diseases	Pathogen Genomics Center, National Institute of Infectious Diseases	Tsuyoshi Sekizuka, Kentaro Itokawa, Rina Tanaka, Masanori Hashino, Makoto Kuroda
EPI_ISL_687699, EPI_ISL_687700, EPI_ISL_687701, EPI_ISL_687702, EPI_ISL_687703, EPI_ISL_687704, EPI_ISL_687705, EPI_ISL_687706, EPI_ISL_687707, EPI_ISL_687708, EPI_ISL_687709, EPI_ISL_687710, EPI_ISL_687712, EPI_ISL_687714			
see above	Niigata Prefectural Institute of Public Health and Environmental Sciences	Pathogen Genomics Center, National Institute of Infectious Diseases	Tsuyoshi Sekizuka, Kentaro Itokawa, Rina Tanaka, Masanori Hashino, Makoto Kuroda
EPI_ISL_687718, EPI_ISL_687719, EPI_ISL_687720, EPI_ISL_687721, EPI_ISL_687722, EPI_ISL_687723, EPI_ISL_687724, EPI_ISL_687725, EPI_ISL_687726, EPI_ISL_687727, EPI_ISL_687728, EPI_ISL_687729, EPI_ISL_687730, EPI_ISL_687731, EPI_ISL_687732, EPI_ISL_687733, EPI_ISL_687734, EPI_ISL_687735, EPI_ISL_687736, EPI_ISL_687737, EPI_ISL_687738, EPI_ISL_687739, EPI_ISL_687740, EPI_ISL_687741, EPI_ISL_687742, EPI_ISL_687746, EPI_ISL_687747, EPI_ISL_687748, EPI_ISL_687749, EPI_ISL_687750, EPI_ISL_687751, EPI_ISL_687752, EPI_ISL_687753, EPI_ISL_687754, EPI_ISL_687755, EPI_ISL_687756, EPI_ISL_687757, EPI_ISL_687758, EPI_ISL_687759, EPI_ISL_687760, EPI_ISL_687761, EPI_ISL_687762, EPI_ISL_687763, EPI_ISL_687764, EPI_ISL_687765, EPI_ISL_687766, EPI_ISL_687767, EPI_ISL_687768, EPI_ISL_687769, EPI_ISL_687770, EPI_ISL_687771, EPI_ISL_687772, EPI_ISL_687773, EPI_ISL_687774, EPI_ISL_687775, EPI_ISL_687776, EPI_ISL_687777, EPI_ISL_687778, EPI_ISL_687779, EPI_ISL_687780, EPI_ISL_687781, EPI_ISL_687782, EPI_ISL_687783, EPI_ISL_687784, EPI_ISL_687785, EPI_ISL_687786, EPI_ISL_687787, EPI_ISL_687788, EPI_ISL_687789, EPI_ISL_687790, EPI_ISL_687791, EPI_ISL_687792, EPI_ISL_687793, EPI_ISL_687794, EPI_ISL_687795, EPI_ISL_687796, EPI_ISL_687797, EPI_ISL_687798, EPI_ISL_687799, EPI_ISL_687800, EPI_ISL_687801, EPI_ISL_687802, EPI_ISL_687803, EPI_ISL_687804, EPI_ISL_687805, EPI_ISL_687806, EPI_ISL_687807, EPI_ISL_687808, EPI_ISL_687809, EPI_ISL_687810, EPI_ISL_687811, EPI_ISL_687812, EPI_ISL_687813, EPI_ISL_687814, EPI_ISL_687815, EPI_ISL_687816, EPI_ISL_687817, EPI_ISL_687818, EPI_ISL_687819, EPI_ISL_687820, EPI_ISL_687821, EPI_ISL_687822, EPI_ISL_687823, EPI_ISL_687824, EPI_ISL_687825, EPI_ISL_687826, EPI_ISL_687827, EPI_ISL_687828, EPI_ISL_687829, EPI_ISL_687830, EPI_ISL_687831, EPI_ISL_687832, EPI_ISL_687833, EPI_ISL_687834, EPI_ISL_687835, EPI_ISL_687836, EPI_ISL_687837, EPI_ISL_687838, EPI_ISL_687839, EPI_ISL_687840, EPI_ISL_687841, EPI_ISL_687842, EPI_ISL_687843, EPI_ISL_687844, EPI_ISL_687845, EPI_ISL_687846, EPI_ISL_687847, EPI_ISL_687848, EPI_ISL_687849, EPI_ISL_687850, EPI_ISL_687851, EPI_ISL_687852, EPI_ISL_687853, EPI_ISL_687854, EPI_ISL_687855, EPI_ISL_687856, EPI_ISL_687857, EPI_ISL_687858, EPI_ISL_687859, EPI_ISL_687860, EPI_ISL_687861, EPI_ISL_687862, EPI_ISL_687863, EPI_ISL_687864, EPI_ISL_687865, EPI_ISL_687866, EPI_ISL_687867, EPI_ISL_687868, EPI_ISL_687869, EPI_ISL_687870, EPI_ISL_687871, EPI_ISL_687872, EPI_ISL_687873, EPI_ISL_687874, EPI_ISL_687875, EPI_ISL_687876, EPI_ISL_687877, EPI_ISL_687878, EPI_ISL_687879, EPI_ISL_687880, EPI_ISL_687881, EPI_ISL_687882, EPI_ISL_687883, EPI_ISL_687884, EPI_ISL_687885, EPI_ISL_687886, EPI_ISL_687887, EPI_ISL_687888, EPI_ISL_687889, EPI_ISL_687890, EPI_ISL_687891, EPI_ISL_687892, EPI_ISL_687893, EPI_ISL_687894, EPI_ISL_687895, EPI_ISL_687896, EPI_ISL_687897, EPI_ISL_687898, EPI_ISL_687899, EPI_ISL_687900, EPI_ISL_687901, EPI_ISL_687902, EPI_ISL_687903, EPI_ISL_687904, EPI_ISL_687905, EPI_ISL_687906, EPI_ISL_687907, EPI_ISL_687908, EPI_ISL_687909, EPI_ISL_687910, EPI_ISL_687911, EPI_ISL_687912, EPI_ISL_687913, EPI_ISL_687914, EPI_ISL_687915, EPI_ISL_687916, EPI_ISL_687917, EPI_ISL_687918, EPI_ISL_687919, EPI_ISL_687932, EPI_ISL_687933, EPI_ISL_687934, EPI_ISL_687935, EPI_ISL_687936, EPI_ISL_687937, EPI_ISL_687938, EPI_ISL_687939, EPI_ISL_687940, EPI_ISL_687941, EPI_ISL_687942, EPI_ISL_687943, EPI_ISL_687973, EPI_ISL_687974, EPI_ISL_687975, EPI_ISL_687976, EPI_ISL_687977, EPI_ISL_687978, EPI_ISL_687979, EPI_ISL_687980, EPI_ISL_687981, EPI_ISL_687982, EPI_ISL_687983, EPI_ISL_687984, EPI_ISL_687985, EPI_ISL_687986, EPI_ISL_687987, EPI_ISL_687988, EPI_ISL_687989, EPI_ISL_687990, EPI_ISL_687991, EPI_ISL_687992, EPI_ISL_687993, EPI_ISL_687994, EPI_ISL_687995, EPI_ISL_687996, EPI_ISL_687997, EPI_ISL_687998, EPI_ISL_687999, EPI_ISL_688000, EPI_ISL_688001, EPI_ISL_688002, EPI_ISL_688003, EPI_ISL_688004, EPI_ISL_688005, EPI_ISL_688006, EPI_ISL_688056, EPI_ISL_688057, EPI_ISL_688058, EPI_ISL_688059, EPI_ISL_688060, EPI_ISL_688061, EPI_ISL_688062, EPI_ISL_688063, EPI_ISL_688064, EPI_ISL_688065, EPI_ISL_688066, EPI_ISL_688067, EPI_ISL_688068, EPI_ISL_688069, EPI_ISL_688070, EPI_ISL_688071, EPI_ISL_688072, EPI_ISL_688073, EPI_ISL_688074, EPI_ISL_688075, EPI_ISL_688076, EPI_ISL_688077, EPI_ISL_688078, EPI_ISL_688079, EPI_ISL_688080, EPI_ISL_688081, EPI_ISL_688082, EPI_ISL_688083, EPI_ISL_688086, EPI_ISL_688087, EPI_ISL_688088, EPI_ISL_688089, EPI_ISL_688090, EPI_ISL_688091, EPI_ISL_688092, EPI_ISL_688093, EPI_ISL_688094, EPI_ISL_688095, EPI_ISL_688096, EPI_ISL_688097, EPI_ISL_688098, EPI_ISL_688099, EPI_ISL_688100, EPI_ISL_688109, EPI_ISL_688110, EPI_ISL_688111, EPI_ISL_688112, EPI_ISL_688113, EPI_ISL_688114, EPI_ISL_688115, EPI_ISL_688116, EPI_ISL_688117, EPI_ISL_688118, EPI_ISL_688119, EPI_ISL_688120, EPI_ISL_688121, EPI_ISL_688122, EPI_ISL_688123, EPI_ISL_688124, EPI_ISL_688125, EPI_ISL_688126, EPI_ISL_688127, EPI_ISL_688128, EPI_ISL_688129, EPI_ISL_688130, EPI_ISL_688131, EPI_ISL_688132, EPI_ISL_688133, EPI_ISL_688134, EPI_ISL_688135, EPI_ISL_688136, EPI_ISL_688137, EPI_ISL_688138, EPI_ISL_688139, EPI_ISL_688140, EPI_ISL_688141, EPI_ISL_688142, EPI_ISL_688143, EPI_ISL_688144, EPI_ISL_688145, EPI_ISL_688146, EPI_ISL_688147, EPI_ISL_688148, EPI_ISL_688149, EPI_ISL_688150, EPI_ISL_688151, EPI_ISL_688152, EPI_ISL_688153, EPI_ISL_688154, EPI_ISL_688155, EPI_ISL_688156, EPI_ISL_688157, EPI_ISL_688158, EPI_ISL_688159, EPI_ISL_688160, EPI_ISL_688161, EPI_ISL_688162, EPI_ISL_688163, EPI_ISL_688164, EPI_ISL_688165, EPI_ISL_688166, EPI_ISL_688167, EPI_ISL_688168, EPI_ISL_688169, EPI_ISL_688170, EPI_ISL_688171, EPI_ISL_688172, EPI_ISL_688173, EPI_ISL_688174, EPI_ISL_688175, EPI_ISL_688176, EPI_ISL_688177, EPI_ISL_688178, EPI_ISL_688179, EPI_ISL_688180, EPI_ISL_688181, EPI_ISL_688182, EPI_ISL_688183, EPI_ISL_688184, EPI_ISL_688185, EPI_ISL_688186, EPI_ISL_688187, EPI_ISL_688188, EPI_ISL_688189, EPI_ISL_688190, EPI_ISL_688191, EPI_ISL_688192, EPI_ISL_688193, EPI_ISL_688194, EPI_ISL_688195, EPI_ISL_688196, EPI_ISL_688197, EPI_ISL_688198, EPI_ISL_688199, EPI_ISL_688200, EPI_ISL_688201, EPI_ISL_688202, EPI_ISL_688203, EPI_ISL_688204, EPI_ISL_688205, EPI_ISL_688206, EPI_ISL_688207, EPI_ISL_688208, EPI_ISL_688209, EPI_ISL_688210, EPI_ISL_688211, EPI_ISL_688212, EPI_ISL_688213, EPI_ISL_688214, EPI_ISL_688215, EPI_ISL_688216, EPI_ISL_688217, EPI_ISL_688218, EPI_ISL_688219, EPI_ISL_688220, EPI_ISL_688221, EPI_ISL_688222, EPI_ISL_688223, EPI_ISL_688224, EPI_ISL_688225, EPI_ISL_688226, EPI_ISL_688227, EPI_ISL_688228, EPI_ISL_688229, EPI_ISL_688230, EPI_ISL_688231, EPI_ISL_688232, EPI_ISL_688233, EPI_ISL_688234, EPI_ISL_688235, EPI_ISL_688236, EPI_ISL_688237, EPI_ISL_688238, EPI_ISL_688239, EPI_ISL_688240, EPI_ISL_688241, EPI_ISL_688242, EPI_ISL_688243, EPI_ISL_688244, EPI_ISL_688245, EPI_ISL_688246, EPI_ISL_688247, EPI_ISL_688248, EPI_ISL_688249, EPI_ISL_688250, EPI_ISL_688269, EPI_ISL_688270, EPI_ISL_688271, EPI_ISL_688272, EPI_ISL_688273, EPI_ISL_688274, EPI_ISL_688275, EPI_ISL_688276, EPI_ISL_688277, EPI_ISL_688278, EPI_ISL_688279, EPI_ISL_688280, EPI_ISL_688281, EPI_ISL_688282, EPI_ISL_688283			

EPI_ISL_688284, EPI_ISL_688285, EPI_ISL_688286, EPI_ISL_688287, EPI_ISL_688288, EPI_ISL_688289, EPI_ISL_688290, EPI_ISL_688291, EPI_ISL_688292, EPI_ISL_688293, EPI_ISL_688294, EPI_ISL_688295, EPI_ISL_688296, EPI_ISL_688297, EPI_ISL_688298, EPI_ISL_688299, EPI_ISL_688300, EPI_ISL_688301, EPI_ISL_688302, EPI_ISL_688303, EPI_ISL_688304, EPI_ISL_688305, EPI_ISL_688306, EPI_ISL_688307, EPI_ISL_688308, EPI_ISL_688309, EPI_ISL_688310, EPI_ISL_688311, EPI_ISL_688312, EPI_ISL_688313, EPI_ISL_688314, EPI_ISL_688315, EPI_ISL_688316, EPI_ISL_688317, EPI_ISL_688318, EPI_ISL_688319, EPI_ISL_688320, EPI_ISL_688321, EPI_ISL_688322, EPI_ISL_688323, EPI_ISL_688324, EPI_ISL_688325, EPI_ISL_688326, EPI_ISL_688327, EPI_ISL_688328, EPI_ISL_688329, EPI_ISL_688330, EPI_ISL_688331, EPI_ISL_688332, EPI_ISL_688333, EPI_ISL_688334, EPI_ISL_688335, EPI_ISL_688336, EPI_ISL_688337, EPI_ISL_688338, EPI_ISL_688339, EPI_ISL_688340, EPI_ISL_688341, EPI_ISL_688342, EPI_ISL_688343, EPI_ISL_688344, EPI_ISL_688345, EPI_ISL_688346, EPI_ISL_688347, EPI_ISL_688348, EPI_ISL_688349, EPI_ISL_688350, EPI_ISL_688351, EPI_ISL_688352, EPI_ISL_688353, EPI_ISL_688354, EPI_ISL_688355, EPI_ISL_688356, EPI_ISL_688357, EPI_ISL_688358, EPI_ISL_688359, EPI_ISL_688360, EPI_ISL_688361, EPI_ISL_688362, EPI_ISL_688363, EPI_ISL_688364, EPI_ISL_688365, EPI_ISL_688366, EPI_ISL_688367, EPI_ISL_688368, EPI_ISL_688369, EPI_ISL_688370, EPI_ISL_688371, EPI_ISL_688372, EPI_ISL_688373, EPI_ISL_688374, EPI_ISL_688375, EPI_ISL_688376, EPI_ISL_688377, EPI_ISL_688378, EPI_ISL_688379, EPI_ISL_688380, EPI_ISL_688381, EPI_ISL_688382, EPI_ISL_688383, EPI_ISL_688384, EPI_ISL_688385, EPI_ISL_688386, EPI_ISL_688387, EPI_ISL_688388, EPI_ISL_688389, EPI_ISL_688390, EPI_ISL_688391, EPI_ISL_688392, EPI_ISL_688393, EPI_ISL_688394, EPI_ISL_688395, EPI_ISL_688396, EPI_ISL_688397, EPI_ISL_688398, EPI_ISL_688399, EPI_ISL_688400, EPI_ISL_688401			
see above	Pathogen Genomics Center, National Institute of Infectious Diseases	Pathogen Genomics Center, National Institute of Infectious Diseases	Tsuyoshi Sekizuka, Kentaro Itokawa, Rina Tanaka, Masanori Hashino, Makoto Kuroda
EPI_ISL_688402, EPI_ISL_688403, EPI_ISL_688404, EPI_ISL_688405, EPI_ISL_688406, EPI_ISL_688407, EPI_ISL_688408, EPI_ISL_688409, EPI_ISL_688410, EPI_ISL_688411, EPI_ISL_688412, EPI_ISL_688413, EPI_ISL_688414, EPI_ISL_688415, EPI_ISL_688416, EPI_ISL_688417, EPI_ISL_688418, EPI_ISL_688419, EPI_ISL_688420, EPI_ISL_688421, EPI_ISL_688422, EPI_ISL_688423, EPI_ISL_688424, EPI_ISL_688425, EPI_ISL_688426, EPI_ISL_688427, EPI_ISL_688428, EPI_ISL_688429, EPI_ISL_688430, EPI_ISL_688431, EPI_ISL_688432, EPI_ISL_688433, EPI_ISL_688434, EPI_ISL_688435, EPI_ISL_688436, EPI_ISL_688437, EPI_ISL_688438, EPI_ISL_688439, EPI_ISL_688440, EPI_ISL_688441, EPI_ISL_688442, EPI_ISL_688443, EPI_ISL_688444, EPI_ISL_688445, EPI_ISL_688446, EPI_ISL_688447, EPI_ISL_688448, EPI_ISL_688449, EPI_ISL_688450, EPI_ISL_688451, EPI_ISL_688452, EPI_ISL_688453, EPI_ISL_688454, EPI_ISL_688455, EPI_ISL_688456, EPI_ISL_688457, EPI_ISL_688458, EPI_ISL_688459, EPI_ISL_688460, EPI_ISL_688461, EPI_ISL_688462, EPI_ISL_688463, EPI_ISL_688464, EPI_ISL_688465, EPI_ISL_688466, EPI_ISL_688467, EPI_ISL_688468, EPI_ISL_688469, EPI_ISL_688470, EPI_ISL_688471, EPI_ISL_688472, EPI_ISL_688473, EPI_ISL_688474, EPI_ISL_688475, EPI_ISL_688476, EPI_ISL_688477, EPI_ISL_688478, EPI_ISL_688479, EPI_ISL_688480, EPI_ISL_688481, EPI_ISL_688482, EPI_ISL_688483, EPI_ISL_688484, EPI_ISL_688485, EPI_ISL_688486, EPI_ISL_688487, EPI_ISL_688488, EPI_ISL_688489, EPI_ISL_688490, EPI_ISL_688491, EPI_ISL_688492, EPI_ISL_688493, EPI_ISL_688494, EPI_ISL_688495, EPI_ISL_688496, EPI_ISL_688497, EPI_ISL_688498, EPI_ISL_688499, EPI_ISL_688500, EPI_ISL_688501, EPI_ISL_688502, EPI_ISL_688503, EPI_ISL_688504, EPI_ISL_688505, EPI_ISL_688506, EPI_ISL_688507, EPI_ISL_688508, EPI_ISL_688509, EPI_ISL_688510, EPI_ISL_688511, EPI_ISL_688512, EPI_ISL_688513, EPI_ISL_688514, EPI_ISL_688515, EPI_ISL_688516, EPI_ISL_688517, EPI_ISL_688518, EPI_ISL_688519, EPI_ISL_688520, EPI_ISL_688521, EPI_ISL_688522, EPI_ISL_688523, EPI_ISL_688524, EPI_ISL_688525, EPI_ISL_688526, EPI_ISL_688527, EPI_ISL_688528, EPI_ISL_688529, EPI_ISL_688530, EPI_ISL_688531, EPI_ISL_688532, EPI_ISL_688533, EPI_ISL_688534, EPI_ISL_688535, EPI_ISL_688536, EPI_ISL_688537, EPI_ISL_688538, EPI_ISL_688539, EPI_ISL_688540, EPI_ISL_688541, EPI_ISL_688542, EPI_ISL_688543, EPI_ISL_688544, EPI_ISL_688545, EPI_ISL_688546, EPI_ISL_688547, EPI_ISL_688548, EPI_ISL_688549, EPI_ISL_688550, EPI_ISL_688551, EPI_ISL_688552, EPI_ISL_688553, EPI_ISL_688554, EPI_ISL_688555, EPI_ISL_688556, EPI_ISL_688557, EPI_ISL_688558, EPI_ISL_688559, EPI_ISL_688560, EPI_ISL_688561, EPI_ISL_688562, EPI_ISL_688563, EPI_ISL_688564, EPI_ISL_688565, EPI_ISL_688566, EPI_ISL_688567, EPI_ISL_688568, EPI_ISL_688569, EPI_ISL_688570, EPI_ISL_688571, EPI_ISL_688572, EPI_ISL_688573, EPI_ISL_688574, EPI_ISL_688575, EPI_ISL_688576, EPI_ISL_688577, EPI_ISL_688578, EPI_ISL_688579, EPI_ISL_688580, EPI_ISL_688581, EPI_ISL_688582			
see above	Saitama Prefectural Institute of Public Health	Pathogen Genomics Center, National Institute of Infectious Diseases	Tsuyoshi Sekizuka, Kentaro Itokawa, Rina Tanaka, Masanori Hashino, Makoto Kuroda
EPI_ISL_688583, EPI_ISL_688584, EPI_ISL_688585, EPI_ISL_688586, EPI_ISL_688587, EPI_ISL_688588, EPI_ISL_688589, EPI_ISL_688590, EPI_ISL_688591, EPI_ISL_688592, EPI_ISL_688593, EPI_ISL_688594, EPI_ISL_688595, EPI_ISL_688596, EPI_ISL_688597, EPI_ISL_688598, EPI_ISL_688599, EPI_ISL_688600, EPI_ISL_688601, EPI_ISL_688602, EPI_ISL_688603, EPI_ISL_688604			
see above	Pathogen Genomics Center, National Institute of Infectious Diseases	Pathogen Genomics Center, National Institute of Infectious Diseases	Tsuyoshi Sekizuka, Kentaro Itokawa, Rina Tanaka, Masanori Hashino, Makoto Kuroda
see above	Tokyo Metropolitan Institute of Public Health	Pathogen Genomics Center, National Institute of Infectious Diseases	Tsuyoshi Sekizuka, Kentaro Itokawa, Rina Tanaka, Masanori Hashino, Makoto Kuroda
EPI_ISL_688605, EPI_ISL_688606, EPI_ISL_688607, EPI_ISL_688608, EPI_ISL_688609, EPI_ISL_688610, EPI_ISL_688611	Pathogen Genomics Center, National Institute of Infectious Diseases	Pathogen Genomics Center, National Institute of Infectious Diseases	Tsuyoshi Sekizuka, Kentaro Itokawa, Rina Tanaka, Masanori Hashino, Makoto Kuroda
EPI_ISL_688612	Fukuoka Institute of Health and Environmental Sciences	Pathogen Genomics Center, National Institute of Infectious Diseases	Tsuyoshi Sekizuka, Kentaro Itokawa, Rina Tanaka, Masanori Hashino, Makoto Kuroda
EPI_ISL_688613, EPI_ISL_688616, EPI_ISL_688617, EPI_ISL_688618	Pathogen Genomics Center, National Institute of Infectious Diseases	Pathogen Genomics Center, National Institute of Infectious Diseases	Tsuyoshi Sekizuka, Kentaro Itokawa, Rina Tanaka, Masanori Hashino, Makoto Kuroda
EPI_ISL_688619, EPI_ISL_688620, EPI_ISL_688621, EPI_ISL_688622, EPI_ISL_688623	Fukuoka Institute of Health and Environmental Sciences	Pathogen Genomics Center, National Institute of Infectious Diseases	Tsuyoshi Sekizuka, Kentaro Itokawa, Rina Tanaka, Masanori Hashino, Makoto Kuroda
EPI_ISL_688625, EPI_ISL_688632, EPI_ISL_688633	Pathogen Genomics Center, National Institute of Infectious Diseases	Pathogen Genomics Center, National Institute of Infectious Diseases	Tsuyoshi Sekizuka, Kentaro Itokawa, Rina Tanaka, Masanori Hashino, Makoto Kuroda
EPI_ISL_688634	Fukuoka Institute of Health and Environmental Sciences	Pathogen Genomics Center, National Institute of Infectious Diseases	Tsuyoshi Sekizuka, Kentaro Itokawa, Rina Tanaka, Masanori Hashino, Makoto Kuroda
EPI_ISL_688635, EPI_ISL_688636, EPI_ISL_688637, EPI_ISL_688638, EPI_ISL_688639, EPI_ISL_688640, EPI_ISL_688641, EPI_ISL_688642, EPI_ISL_688643, EPI_ISL_688644, EPI_ISL_688645, EPI_ISL_688646, EPI_ISL_688647, EPI_ISL_688648, EPI_ISL_688649, EPI_ISL_688650, EPI_ISL_688651, EPI_ISL_688652, EPI_ISL_688653, EPI_ISL_688654, EPI_ISL_688655, EPI_ISL_688656, EPI_ISL_688657, EPI_ISL_688658, EPI_ISL_688659, EPI_ISL_688660, EPI_ISL_688661, EPI_ISL_688662, EPI_ISL_688663, EPI_ISL_688664, EPI_ISL_688665, EPI_ISL_688666, EPI_ISL_688667, EPI_ISL_688668, EPI_ISL_688669, EPI_ISL_688670, EPI_ISL_688671, EPI_ISL_688672, EPI_ISL_688673, EPI_ISL_688674, EPI_ISL_688675, EPI_ISL_688676, EPI_ISL_688677, EPI_ISL_688678, EPI_ISL_688679, EPI_ISL_688680, EPI_ISL_688681, EPI_ISL_688682, EPI_ISL_688683, EPI_ISL_688684, EPI_ISL_688685, EPI_ISL_688686, EPI_ISL_688687, EPI_ISL_688688, EPI_ISL_688689, EPI_ISL_688690, EPI_ISL_688691, EPI_ISL_688692, EPI_ISL_688693, EPI_ISL_688694, EPI_ISL_688695, EPI_ISL_688696, EPI_ISL_688697, EPI_ISL_688698, EPI_ISL_688699			
see above	Pathogen Genomics Center, National Institute of Infectious Diseases	Pathogen Genomics Center, National Institute of Infectious Diseases	Tsuyoshi Sekizuka, Kentaro Itokawa, Rina Tanaka, Masanori Hashino, Makoto Kuroda
EPI_ISL_688700, EPI_ISL_688701, EPI_ISL_688702, EPI_ISL_688703, EPI_ISL_688704, EPI_ISL_688705, EPI_ISL_688706, EPI_ISL_688707, EPI_ISL_688708, EPI_ISL_688709	Sakai City Institute of Public Health	Pathogen Genomics Center, National Institute of Infectious Diseases	Tsuyoshi Sekizuka, Kentaro Itokawa, Rina Tanaka, Masanori Hashino, Makoto Kuroda
EPI_ISL_688710, EPI_ISL_688711, EPI_ISL_688712, EPI_ISL_688713, EPI_ISL_688714, EPI_ISL_688715, EPI_ISL_688716, EPI_ISL_688717, EPI_ISL_688718, EPI_ISL_688719, EPI_ISL_688720, EPI_ISL_688721, EPI_ISL_688722, EPI_ISL_688723, EPI_ISL_688724, EPI_ISL_688725, EPI_ISL_688726, EPI_ISL_688727, EPI_ISL_688728, EPI_ISL_688729, EPI_ISL_688730, EPI_ISL_688731, EPI_ISL_688732, EPI_ISL_688733, EPI_ISL_688734, EPI_ISL_688735, EPI_ISL_688736, EPI_ISL_688737, EPI_ISL_688738, EPI_ISL_688739, EPI_ISL_688740, EPI_ISL_688741, EPI_ISL_688742, EPI_ISL_688743, EPI_ISL_688744, EPI_ISL_688745, EPI_ISL_688747, EPI_ISL_688748, EPI_ISL_688749, EPI_ISL_688750, EPI_ISL_688751, EPI_ISL_688752, EPI_ISL_688753, EPI_ISL_688754, EPI_ISL_688755, EPI_ISL_688756, EPI_ISL_688814, EPI_ISL_688815, EPI_ISL_688816, EPI_ISL_688817, EPI_ISL_688818, EPI_ISL_688819, EPI_ISL_688820, EPI_ISL_688821, EPI_ISL_688822, EPI_ISL_688823, EPI_ISL_688824, EPI_ISL_688825, EPI_ISL_688826, EPI_ISL_688827, EPI_ISL_688828, EPI_ISL_688829, EPI_ISL_688830, EPI_ISL_688831, EPI_ISL_688832, EPI_ISL_688833, EPI_ISL_688834, EPI_ISL_688835, EPI_ISL_688836, EPI_ISL_688837, EPI_ISL_688838, EPI_ISL_688839, EPI_ISL_688840, EPI_ISL_688841, EPI_ISL_688842, EPI_ISL_688843, EPI_ISL_688844, EPI_ISL_688845, EPI_ISL_688846, EPI_ISL_688847, EPI_ISL_688848, EPI_ISL_688849, EPI_ISL_688850, EPI_ISL_688851, EPI_ISL_688852, EPI_ISL_688853, EPI_ISL_688854, EPI_ISL_688855, EPI_ISL_688856, EPI_ISL_688857, EPI_ISL_688858, EPI_ISL_688859, EPI_ISL_688860, EPI_ISL_688861, EPI_ISL_688862, EPI_ISL_688863, EPI_ISL_688864, EPI_ISL_688865, EPI_ISL_688866, EPI_ISL_688867, EPI_ISL_688868, EPI_ISL_688869, EPI_ISL_688870, EPI_ISL_688871, EPI_ISL_688872, EPI_ISL_688873, EPI_ISL_688874, EPI_ISL_688875, EPI_ISL_688876, EPI_ISL_688877, EPI_ISL_688878, EPI_ISL_688879, EPI_ISL_688880, EPI_ISL_688881, EPI_ISL_688882, EPI_ISL_688883, EPI_ISL_688884, EPI_ISL_688885, EPI_ISL_688886, EPI_ISL_688887, EPI_ISL_688888, EPI_ISL_688889, EPI_ISL_688890, EPI_ISL_688891, EPI_ISL_688892, EPI_ISL_688893, EPI_ISL_688894, EPI_ISL_688895, EPI_ISL_688896			
see above	Pathogen Genomics Center, National Institute of Infectious Diseases	Pathogen Genomics Center, National Institute of Infectious Diseases	Tsuyoshi Sekizuka, Kentaro Itokawa, Rina Tanaka, Masanori Hashino, Makoto Kuroda
EPI_ISL_688897, EPI_ISL_688898, EPI_ISL_688899, EPI_ISL_688900, EPI_ISL_688901, EPI_ISL_688902, EPI_ISL_688903, EPI_ISL_688904, EPI_ISL_688905, EPI_ISL_688906, EPI_ISL_688907, EPI_ISL_688908, EPI_ISL_688909, EPI_ISL_688910, EPI_ISL_688911, EPI_ISL_688912, EPI_ISL_688913, EPI_ISL_688914, EPI_ISL_688915, EPI_ISL_688916, EPI_ISL_688917, EPI_ISL_688918, EPI_ISL_688919, EPI_ISL_688920, EPI_ISL_688921, EPI_ISL_688922, EPI_ISL_688923, EPI_ISL_688924, EPI_ISL_688925, EPI_ISL_688926, EPI_ISL_688927, EPI_ISL_688928, EPI_ISL_688929, EPI_ISL_688930, EPI_ISL_688931, EPI_ISL_688932, EPI_ISL_688933, EPI_ISL_688934, EPI_ISL_688935, EPI_ISL_688936, EPI_ISL_688937, EPI_ISL_688938, EPI_ISL_688939, EPI_ISL_688940, EPI_ISL_688941, EPI_ISL_688942, EPI_ISL_688943, EPI_ISL_688944, EPI_ISL_688945, EPI_ISL_688946, EPI_ISL_688947, EPI_ISL_688948, EPI_ISL_688949, EPI_ISL_688950, EPI_ISL_688951, EPI_ISL_688952, EPI_ISL_688953, EPI_ISL_688954, EPI_ISL_688955, EPI_ISL_688956, EPI_ISL_688957, EPI_ISL_688958, EPI_ISL_688959, EPI_ISL_688960, EPI_ISL_688961, EPI_ISL_688962, EPI_ISL_688963, EPI_ISL_688964, EPI_ISL_688965, EPI_ISL_688966, EPI_ISL_688967, EPI_ISL_688968			
see above	Fukuoka Institute of Health and Environmental Sciences	Pathogen Genomics Center, National Institute of Infectious Diseases	Tsuyoshi Sekizuka, Kentaro Itokawa, Rina Tanaka, Masanori Hashino, Makoto Kuroda
EPI_ISL_688969, EPI_ISL_688970, EPI_ISL_688971, EPI_ISL_688972, EPI_ISL_688973, EPI_ISL_688974, EPI_ISL_688975, EPI_ISL_688976, EPI_ISL_688977, EPI_ISL_688978, EPI_ISL_688979, EPI_ISL_688980, EPI_ISL_688981, EPI_ISL_688982, EPI_ISL_688983, EPI_ISL_688984, EPI_ISL_688985, EPI_ISL_688986, EPI_ISL_688987, EPI_ISL_688988, EPI_ISL_688989, EPI_ISL_688990, EPI_ISL_688991, EPI_ISL_688992, EPI_ISL_688993, EPI_ISL_688994, EPI_ISL_688995, EPI_ISL_688996, EPI_ISL_688997, EPI_ISL_688998, EPI_ISL_688999, EPI_ISL_689000, EPI_ISL_689001, EPI_ISL_689002, EPI_ISL_689003, EPI_ISL_689004, EPI_ISL_689005, EPI_ISL_689006, EPI_ISL_689007, EPI_ISL_689008, EPI_ISL_689009, EPI_ISL_689010, EPI_ISL_689011, EPI_ISL_689012, EPI_ISL_689013, EPI_ISL_689014, EPI_ISL_689015, EPI_ISL_689016, EPI_ISL_689017, EPI_ISL_689018, EPI_ISL_689019, EPI_ISL_689020, EPI_ISL_689021, EPI_ISL_689022, EPI_ISL_689023, EPI_ISL_689024, EPI_ISL_689025, EPI_ISL_689026, EPI_ISL_689027, EPI_ISL_689028, EPI_ISL_689029, EPI_ISL_689030, EPI_ISL_689031, EPI_ISL_689032, EPI_ISL_689033, EPI_ISL_689034, EPI_ISL_689035, EPI_ISL_689036, EPI_ISL_689037, EPI_ISL_689038, EPI_ISL_689039, EPI_ISL_689040, EPI_ISL_689041, EPI_ISL_689042, EPI_ISL_689043, EPI_ISL_689044, EPI_ISL_689045, EPI_ISL_689046			
see above	Pathogen Genomics Center, National Institute of Infectious Diseases	Pathogen Genomics Center, National Institute of Infectious Diseases	Tsuyoshi Sekizuka, Kentaro Itokawa, Rina Tanaka, Masanori Hashino, Makoto Kuroda

EPI_ISL_689077, EPI_ISL_689078, EPI_ISL_689079, EPI_ISL_689080, EPI_ISL_689081, EPI_ISL_689082, EPI_ISL_689083, EPI_ISL_689084, EPI_ISL_689085, EPI_ISL_689086, EPI_ISL_689087, EPI_ISL_689088

see above	Tokyo Metropolitan Institute of Public Health	Pathogen Genomics Center, National Institute of Infectious Diseases	Tsuyoshi Sekizuka, Kentaro Itokawa, Rina Tanaka, Masanori Hashino, Makoto Kuroda

EPI_ISL_689089, EPI_ISL_689090, EPI_ISL_689091, EPI_ISL_689092, EPI_ISL_689093, EPI_ISL_689094, EPI_ISL_689095, EPI_ISL_689096, EPI_ISL_689097, EPI_ISL_689098, EPI_ISL_689099, EPI_ISL_689100, EPI_ISL_689101, EPI_ISL_689102, EPI_ISL_689103, EPI_ISL_689104, EPI_ISL_689105, EPI_ISL_689106, EPI_ISL_689107, EPI_ISL_689108, EPI_ISL_689109, EPI_ISL_689110, EPI_ISL_689111, EPI_ISL_689112, EPI_ISL_689113, EPI_ISL_689114, EPI_ISL_689115, EPI_ISL_689116, EPI_ISL_689117, EPI_ISL_689118, EPI_ISL_689119, EPI_ISL_689120, EPI_ISL_689121, EPI_ISL_689122, EPI_ISL_689123, EPI_ISL_689124, EPI_ISL_689125, EPI_ISL_689126, EPI_ISL_689127, EPI_ISL_689128, EPI_ISL_689129, EPI_ISL_689130, EPI_ISL_689131, EPI_ISL_689132, EPI_ISL_689133, EPI_ISL_689134, EPI_ISL_689135, EPI_ISL_689136, EPI_ISL_689137, EPI_ISL_689138, EPI_ISL_689139, EPI_ISL_689140, EPI_ISL_689141, EPI_ISL_689142, EPI_ISL_689143, EPI_ISL_689144, EPI_ISL_689145, EPI_ISL_689146, EPI_ISL_689147, EPI_ISL_689148, EPI_ISL_689149, EPI_ISL_689150, EPI_ISL_689151, EPI_ISL_689152, EPI_ISL_689153, EPI_ISL_689154, EPI_ISL_689155, EPI_ISL_689156, EPI_ISL_689157, EPI_ISL_689158, EPI_ISL_689159, EPI_ISL_689160, EPI_ISL_689161, EPI_ISL_689162, EPI_ISL_689163, EPI_ISL_689164, EPI_ISL_689165, EPI_ISL_689166, EPI_ISL_689167, EPI_ISL_689168, EPI_ISL_689169, EPI_ISL_689170, EPI_ISL_689171, EPI_ISL_689172, EPI_ISL_689173, EPI_ISL_689174, EPI_ISL_689175, EPI_ISL_689176, EPI_ISL_689177, EPI_ISL_689178, EPI_ISL_689179, EPI_ISL_689180, EPI_ISL_689181, EPI_ISL_689182, EPI_ISL_689183, EPI_ISL_689184, EPI_ISL_689185, EPI_ISL_689186, EPI_ISL_689187, EPI_ISL_689188, EPI_ISL_689189, EPI_ISL_689190, EPI_ISL_689191, EPI_ISL_689192, EPI_ISL_689193, EPI_ISL_689194, EPI_ISL_689195, EPI_ISL_689196, EPI_ISL_689197, EPI_ISL_689198, EPI_ISL_689199, EPI_ISL_689200, EPI_ISL_689201, EPI_ISL_689202, EPI_ISL_689203, EPI_ISL_689204, EPI_ISL_689205, EPI_ISL_689206, EPI_ISL_689207, EPI_ISL_689208, EPI_ISL_689209, EPI_ISL_689210, EPI_ISL_689211, EPI_ISL_689212, EPI_ISL_689213, EPI_ISL_689214, EPI_ISL_689215, EPI_ISL_689216, EPI_ISL_689217, EPI_ISL_689218, EPI_ISL_689219, EPI_ISL_689220, EPI_ISL_689221, EPI_ISL_689222, EPI_ISL_689223, EPI_ISL_689224, EPI_ISL_689225, EPI_ISL_689226, EPI_ISL_689227, EPI_ISL_689228, EPI_ISL_689229, EPI_ISL_689230, EPI_ISL_689231, EPI_ISL_689232, EPI_ISL_689233, EPI_ISL_689234, EPI_ISL_689235, EPI_ISL_689236, EPI_ISL_689237, EPI_ISL_689238, EPI_ISL_689239, EPI_ISL_689240, EPI_ISL_689241, EPI_ISL_689242, EPI_ISL_689243, EPI_ISL_689244, EPI_ISL_689245, EPI_ISL_689246, EPI_ISL_689247, EPI_ISL_689248, EPI_ISL_689249, EPI_ISL_689250, EPI_ISL_689251, EPI_ISL_689252, EPI_ISL_689253, EPI_ISL_689254, EPI_ISL_689255, EPI_ISL_689256, EPI_ISL_689257, EPI_ISL_689258, EPI_ISL_689259, EPI_ISL_689260, EPI_ISL_689261, EPI_ISL_689262, EPI_ISL_689263, EPI_ISL_689264, EPI_ISL_689265, EPI_ISL_689266, EPI_ISL_689267, EPI_ISL_689268, EPI_ISL_689269, EPI_ISL_689270, EPI_ISL_689271, EPI_ISL_689272, EPI_ISL_689273, EPI_ISL_689274, EPI_ISL_689275, EPI_ISL_689276, EPI_ISL_689277, EPI_ISL_689278, EPI_ISL_689279, EPI_ISL_689280, EPI_ISL_689281, EPI_ISL_689282, EPI_ISL_689283, EPI_ISL_689284, EPI_ISL_689285, EPI_ISL_689286, EPI_ISL_689287, EPI_ISL_689288, EPI_ISL_689289, EPI_ISL_689290, EPI_ISL_689291, EPI_ISL_689292, EPI_ISL_689293, EPI_ISL_689294, EPI_ISL_689295, EPI_ISL_689296, EPI_ISL_689297, EPI_ISL_689298, EPI_ISL_689299, EPI_ISL_689300, EPI_ISL_689301, EPI_ISL_689302, EPI_ISL_689303, EPI_ISL_689304, EPI_ISL_689305, EPI_ISL_689306, EPI_ISL_689307, EPI_ISL_689308, EPI_ISL_689310, EPI_ISL_689311, EPI_ISL_689312, EPI_ISL_689313, EPI_ISL_689314, EPI_ISL_689315, EPI_ISL_689316, EPI_ISL_689317, EPI_ISL_689318, EPI_ISL_689319, EPI_ISL_689320, EPI_ISL_689321, EPI_ISL_689322, EPI_ISL_689323, EPI_ISL_689324, EPI_ISL_689325, EPI_ISL_689326, EPI_ISL_689327, EPI_ISL_689328, EPI_ISL_689329, EPI_ISL_689330, EPI_ISL_689331, EPI_ISL_689332, EPI_ISL_689333, EPI_ISL_689334, EPI_ISL_689335, EPI_ISL_689336, EPI_ISL_689337, EPI_ISL_689338, EPI_ISL_689339, EPI_ISL_689340, EPI_ISL_689341, EPI_ISL_689342, EPI_ISL_689343, EPI_ISL_689344, EPI_ISL_689345, EPI_ISL_689346, EPI_ISL_689347, EPI_ISL_689348, EPI_ISL_689349, EPI_ISL_689350, EPI_ISL_689351, EPI_ISL_689352, EPI_ISL_689353, EPI_ISL_689354, EPI_ISL_689355, EPI_ISL_689356, EPI_ISL_689357, EPI_ISL_689358, EPI_ISL_689359, EPI_ISL_689360, EPI_ISL_689361, EPI_ISL_689362, EPI_ISL_689363, EPI_ISL_689364, EPI_ISL_689365, EPI_ISL_689366, EPI_ISL_689367, EPI_ISL_689368, EPI_ISL_689369, EPI_ISL_689370, EPI_ISL_689371, EPI_ISL_689372, EPI_ISL_689373, EPI_ISL_689374, EPI_ISL_689375, EPI_ISL_689376, EPI_ISL_689377, EPI_ISL_689378, EPI_ISL_689379, EPI_ISL_689380, EPI_ISL_689381, EPI_ISL_689382, EPI_ISL_689383, EPI_ISL_689384, EPI_ISL_689385, EPI_ISL_689386, EPI_ISL_689387, EPI_ISL_689388, EPI_ISL_689389, EPI_ISL_689390, EPI_ISL_689391, EPI_ISL_689392, EPI_ISL_689393, EPI_ISL_689394, EPI_ISL_689395, EPI_ISL_689396, EPI_ISL_689397, EPI_ISL_689398, EPI_ISL_689399, EPI_ISL_689400, EPI_ISL_689401, EPI_ISL_689402, EPI_ISL_689403, EPI_ISL_689404, EPI_ISL_689405, EPI_ISL_689406, EPI_ISL_689407, EPI_ISL_689408, EPI_ISL_689409, EPI_ISL_689410, EPI_ISL_689411, EPI_ISL_689412, EPI_ISL_689413, EPI_ISL_689414, EPI_ISL_689415, EPI_ISL_689416, EPI_ISL_689417, EPI_ISL_689418, EPI_ISL_689419, EPI_ISL_689420, EPI_ISL_689421, EPI_ISL_689422, EPI_ISL_689423, EPI_ISL_689424, EPI_ISL_689425, EPI_ISL_689426, EPI_ISL_689427, EPI_ISL_689428, EPI_ISL_689429, EPI_ISL_689430, EPI_ISL_689431, EPI_ISL_689432, EPI_ISL_689433, EPI_ISL_689434, EPI_ISL_689435, EPI_ISL_689436, EPI_ISL_689437, EPI_ISL_689438, EPI_ISL_689439, EPI_ISL_689440, EPI_ISL_689441, EPI_ISL_689442, EPI_ISL_689443, EPI_ISL_689444, EPI_ISL_689445, EPI_ISL_689446, EPI_ISL_689447, EPI_ISL_689448, EPI_ISL_689449, EPI_ISL_689450, EPI_ISL_689451, EPI_ISL_689452, EPI_ISL_689453, EPI_ISL_689454, EPI_ISL_689455, EPI_ISL_689456, EPI_ISL_689457, EPI_ISL_689458, EPI_ISL_689459

see above	Pathogen Genomics Center, National Institute of Infectious Diseases	Pathogen Genomics Center, National Institute of Infectious Diseases	Tsuyoshi Sekizuka, Kentaro Itokawa, Rina Tanaka, Masanori Hashino, Makoto Kuroda

EPI_ISL_689460, EPI_ISL_689461, EPI_ISL_689462, EPI_ISL_689463, EPI_ISL_689464, EPI_ISL_689465, EPI_ISL_689466, EPI_ISL_689467, EPI_ISL_689468, EPI_ISL_689469, EPI_ISL_689470, EPI_ISL_689471, EPI_ISL_689472, EPI_ISL_689473, EPI_ISL_689474, EPI_ISL_689475, EPI_ISL_689476, EPI_ISL_689477, EPI_ISL_689478, EPI_ISL_689479, EPI_ISL_689480, EPI_ISL_689481, EPI_ISL_689482, EPI_ISL_689483, EPI_ISL_689484, EPI_ISL_689485, EPI_ISL_689486, EPI_ISL_689487, EPI_ISL_689488, EPI_ISL_689489, EPI_ISL_689490, EPI_ISL_689491, EPI_ISL_689492, EPI_ISL_689493, EPI_ISL_689494, EPI_ISL_689495, EPI_ISL_689496

see above	Fukuoka Institute of Health and Environmental Sciences	Pathogen Genomics Center, National Institute of Infectious Diseases	Tsuyoshi Sekizuka, Kentaro Itokawa, Rina Tanaka, Masanori Hashino, Makoto Kuroda

EPI_ISL_689497, EPI_ISL_689498, EPI_ISL_689499, EPI_ISL_689500, EPI_ISL_689501, EPI_ISL_689502, EPI_ISL_689503, EPI_ISL_689504, EPI_ISL_689505, EPI_ISL_689506, EPI_ISL_689507, EPI_ISL_689508, EPI_ISL_689509, EPI_ISL_689510, EPI_ISL_689511, EPI_ISL_689512, EPI_ISL_689513, EPI_ISL_689514, EPI_ISL_689515, EPI_ISL_689516, EPI_ISL_689517, EPI_ISL_689518, EPI_ISL_689519, EPI_ISL_689520, EPI_ISL_689521, EPI_ISL_689522, EPI_ISL_689523, EPI_ISL_689524, EPI_ISL_689525, EPI_ISL_689526, EPI_ISL_689527, EPI_ISL_689528, EPI_ISL_689529, EPI_ISL_689530, EPI_ISL_689531, EPI_ISL_689532, EPI_ISL_689533, EPI_ISL_689534, EPI_ISL_689535, EPI_ISL_689536, EPI_ISL_689537, EPI_ISL_689538, EPI_ISL_689539, EPI_ISL_689540, EPI_ISL_689541, EPI_ISL_689542, EPI_ISL_689543, EPI_ISL_689544, EPI_ISL_689545, EPI_ISL_689546, EPI_ISL_689547, EPI_ISL_689548, EPI_ISL_689551, EPI_ISL_689552, EPI_ISL_689553, EPI_ISL_689554, EPI_ISL_689555, EPI_ISL_689556, EPI_ISL_689557, EPI_ISL_689558, EPI_ISL_689559, EPI_ISL_689560, EPI_ISL_689561, EPI_ISL_689562, EPI_ISL_689563, EPI_ISL_689564, EPI_ISL_689565, EPI_ISL_689566, EPI_ISL_689567, EPI_ISL_689568, EPI_ISL_689569, EPI_ISL_689570, EPI_ISL_689571, EPI_ISL_689572, EPI_ISL_689573, EPI_ISL_689574, EPI_ISL_689575, EPI_ISL_689576, EPI_ISL_689577, EPI_ISL_689578, EPI_ISL_689579, EPI_ISL_689580, EPI_ISL_689581, EPI_ISL_689582, EPI_ISL_689583, EPI_ISL_689584, EPI_ISL_689585, EPI_ISL_689586, EPI_ISL_689587, EPI_ISL_689588, EPI_ISL_689589, EPI_ISL_689590, EPI_ISL_689591, EPI_ISL_689601, EPI_ISL_689602, EPI_ISL_689603, EPI_ISL_689604, EPI_ISL_689605, EPI_ISL_689606, EPI_ISL_689607, EPI_ISL_689608, EPI_ISL_689609, EPI_ISL_689610, EPI_ISL_689611, EPI_ISL_689624, EPI_ISL_689625, EPI_ISL_689626, EPI_ISL_689627, EPI_ISL_689628, EPI_ISL_689629, EPI_ISL_689630, EPI_ISL_689631, EPI_ISL_689632, EPI_ISL_689633, EPI_ISL_689634, EPI_ISL_689635, EPI_ISL_689636, EPI_ISL_689637, EPI_ISL_689638, EPI_ISL_689639, EPI_ISL_689641, EPI_ISL_689642, EPI_ISL_689644, EPI_ISL_689645, EPI_ISL_689647, EPI_ISL_689648, EPI_ISL_689649, EPI_ISL_689650, EPI_ISL_689651, EPI_ISL_689652, EPI_ISL_689653, EPI_ISL_689654, EPI_ISL_689655, EPI_ISL_689656, EPI_ISL_689657, EPI_ISL_689658, EPI_ISL_689659, EPI_ISL_689660, EPI_ISL_689661, EPI_ISL_689662, EPI_ISL_689663, EPI_ISL_689664, EPI_ISL_689665, EPI_ISL_689666, EPI_ISL_689667, EPI_ISL_689668, EPI_ISL_689669, EPI_ISL_689670, EPI_ISL_689671, EPI_ISL_689672, EPI_ISL_689673, EPI_ISL_689674, EPI_ISL_689675, EPI_ISL_689676, EPI_ISL_689677, EPI_ISL_689678, EPI_ISL_689679, EPI_ISL_689681, EPI_ISL_689682, EPI_ISL_689683, EPI_ISL_689684, EPI_ISL_689685

see above	Pathogen Genomics Center, National Institute of Infectious Diseases	Pathogen Genomics Center, National Institute of Infectious Diseases	Tsuyoshi Sekizuka, Kentaro Itokawa, Rina Tanaka, Masanori Hashino, Makoto Kuroda
EPI_ISL_689692, EPI_ISL_689693, EPI_ISL_689694, EPI_ISL_689695, EPI_ISL_689696, EPI_ISL_689697	Fukuoka Institute of Health and Environmental Sciences	Pathogen Genomics Center, National Institute of Infectious Diseases	Tsuyoshi Sekizuka, Kentaro Itokawa, Rina Tanaka, Masanori Hashino, Makoto Kuroda

EPI_ISL_689698, EPI_ISL_689699, EPI_ISL_689700, EPI_ISL_689701, EPI_ISL_689702, EPI_ISL_689703, EPI_ISL_689704, EPI_ISL_689705, EPI_ISL_689706, EPI_ISL_689707, EPI_ISL_689708, EPI_ISL_689709, EPI_ISL_689710, EPI_ISL_689711, EPI_ISL_689712, EPI_ISL_689713, EPI_ISL_689714, EPI_ISL_689715, EPI_ISL_689716, EPI_ISL_689717, EPI_ISL_689718, EPI_ISL_689719, EPI_ISL_689720, EPI_ISL_689721, EPI_ISL_689722, EPI_ISL_689723, EPI_ISL_689724, EPI_ISL_689725, EPI_ISL_689726, EPI_ISL_689727, EPI_ISL_689728, EPI_ISL_689729, EPI_ISL_689730, EPI_ISL_689731, EPI_ISL_689732, EPI_ISL_689733, EPI_ISL_689734, EPI_ISL_689735, EPI_ISL_689736, EPI_ISL_689737, EPI_ISL_689738, EPI_ISL_689739, EPI_ISL_689740, EPI_ISL_689741, EPI_ISL_689742, EPI_ISL_689743, EPI_ISL_689744, EPI_ISL_689745, EPI_ISL_689746, EPI_ISL_689747, EPI_ISL_689748, EPI_ISL_689749, EPI_ISL_689750, EPI_ISL_689751, EPI_ISL_689752, EPI_ISL_689753, EPI_ISL_689754, EPI_ISL_689755, EPI_ISL_689756, EPI_ISL_689757, EPI_ISL_689758

see above	Pathogen Genomics Center, National Institute of Infectious Diseases	Pathogen Genomics Center, National Institute of Infectious Diseases	Tsuyoshi Sekizuka, Kentaro Itokawa, Rina Tanaka, Masanori Hashino, Makoto Kuroda

EPI_ISL_689759, EPI_ISL_689760, EPI_ISL_689761, EPI_ISL_689762, EPI_ISL_689763, EPI_ISL_689764, EPI_ISL_689765, EPI_ISL_689766, EPI_ISL_689767, EPI_ISL_689768, EPI_ISL_689769, EPI_ISL_689770, EPI_ISL_689771, EPI_ISL_689772, EPI_ISL_689773, EPI_ISL_689774, EPI_ISL_689775, EPI_ISL_689776, EPI_ISL_689777, EPI_ISL_689778, EPI_ISL_689779, EPI_ISL_689780, EPI_ISL_689781, EPI_ISL_689782, EPI_ISL_689783, EPI_ISL_689784, EPI_ISL_689785, EPI_ISL_689786, EPI_ISL_689787, EPI_ISL_689788, EPI_ISL_689789, EPI_ISL_689790, EPI_ISL_689791, EPI_ISL_689792, EPI_ISL_689793, EPI_ISL_689794, EPI_ISL_689795, EPI_ISL_689796, EPI_ISL_689797, EPI_ISL_689798, EPI_ISL_689799, EPI_ISL_689800, EPI_ISL_689801, EPI_ISL_689802, EPI_ISL_689803, EPI_ISL_689804, EPI_ISL_689805, EPI_ISL_689806, EPI_ISL_689807, EPI_ISL_689808, EPI_ISL_689809, EPI_ISL_689810, EPI_ISL_689811, EPI_ISL_689812, EPI_ISL_689813, EPI_ISL_689814, EPI_ISL_689815, EPI_ISL_689816, EPI_ISL_689817, EPI_ISL_689818, EPI_ISL_689819, EPI_ISL_689820, EPI_ISL_689821, EPI_ISL_689822, EPI_ISL_689823, EPI_ISL_689824, EPI_ISL_689825

see above	Fukuoka Institute of Health and Environmental Sciences	Pathogen Genomics Center, National Institute of Infectious Diseases	Tsuyoshi Sekizuka, Kentaro Itokawa, Rina Tanaka, Masanori Hashino, Makoto Kuroda

EPI_ISL_689826, EPI_ISL_689827, EPI_ISL_689828, EPI_ISL_689829, EPI_ISL_689830, EPI_ISL_689831, EPI_ISL_689832, EPI_ISL_689833, EPI_ISL_689834, EPI_ISL_689835, EPI_ISL_689836, EPI_ISL_689837, EPI_ISL_689838, EPI_ISL_689839, EPI_ISL_689840, EPI_ISL_689841, EPI_ISL_689842, EPI_ISL_689843, EPI_ISL_689844, EPI_ISL_689845, EPI_ISL_689846, EPI_ISL_689847, EPI_ISL_689848, EPI_ISL_689849, EPI_ISL_689850, EPI_ISL_689851, EPI_ISL_689852, EPI_ISL_689853, EPI_ISL_689854, EPI_ISL_689855, EPI_ISL_689856, EPI_ISL_689857, EPI_ISL_689858, EPI_ISL_689859, EPI_ISL_689860, EPI_ISL_689861, EPI_ISL_689862, EPI_ISL_689863, EPI_ISL_689864, EPI_ISL_689865, EPI_ISL_689866, EPI_ISL_689867, EPI_ISL_689868, EPI_ISL_689869, EPI_ISL_689870, EPI_ISL_689871, EPI_ISL_689872, EPI_ISL_689873, EPI_ISL_689874, EPI_ISL_689875, EPI_ISL_689876, EPI_ISL_689877, EPI_ISL_689878, EPI_ISL_689879, EPI_ISL_689880, EPI_ISL_689881, EPI_ISL_689882, EPI_ISL_689883, EPI_ISL_689884, EPI_ISL_689885, EPI_ISL_689886, EPI_ISL_689887, EPI_ISL_689888, EPI_ISL_689889, EPI_ISL_689890, EPI_ISL_689891, EPI_ISL_689892, EPI_ISL_689893, EPI_ISL_689894, EPI_ISL_689895, EPI_ISL_689896, EPI_ISL_689897, EPI_ISL_689898, EPI_ISL_689899, EPI_ISL_689900, EPI_ISL_689901, EPI_ISL_689902, EPI_ISL_689903, EPI_ISL_689904, EPI_ISL_689905, EPI_ISL_689906, EPI_ISL_689907, EPI_ISL_689908, EPI_ISL_689909, EPI_ISL_689910, EPI_ISL_689911, EPI_ISL_689912, EPI_ISL_689913, EPI_ISL_689914, EPI_ISL_689915, EPI_ISL_689916, EPI_ISL_689917, EPI_ISL_689918, EPI_ISL_689919, EPI_ISL_689920, EPI_ISL_689921, EPI_ISL_689922, EPI_ISL_689923, EPI_ISL_689924, EPI_ISL_689925, EPI_ISL_689926, EPI_ISL_689927, EPI_ISL_689928, EPI_ISL_689929, EPI_ISL_689930, EPI_ISL_689931, EPI_ISL_689932, EPI_ISL_689933, EPI_ISL_689934, EPI_ISL_689935, EPI_ISL_689936, EPI_ISL_689937, EPI_ISL_689938, EPI_ISL_689939, EPI_ISL_689940, EPI_ISL_689941, EPI_ISL_689942, EPI_ISL_689943, EPI_ISL_689944, EPI_ISL_689945, EPI_ISL_689946, EPI_ISL_689947, EPI_ISL_689948, EPI_ISL_689949, EPI_ISL_689950, EPI_ISL_689951, EPI_ISL_689952, EPI_ISL_689953, EPI_ISL_689954, EPI_ISL_689955, EPI_ISL_689956, EPI_ISL_689957, EPI_ISL_689958, EPI_ISL_689959, EPI_ISL_689960, EPI_ISL_689961, EPI_ISL_689962, EPI_ISL_689963, EPI_ISL_689964, EPI_ISL_689965, EPI_ISL_689966, EPI_ISL_689967, EPI_ISL_689968, EPI_ISL_689969, EPI_ISL_689970, EPI_ISL_689971, EPI_ISL_689972, EPI_ISL_689973, EPI_ISL_689974, EPI_ISL_689975, EPI_ISL_689976, EPI_ISL_689977, EPI_ISL_689978, EPI_ISL_689979, EPI_ISL_689980, EPI_ISL_689981, EPI_ISL_689982, EPI_ISL_689983, EPI_ISL_689984, EPI_ISL_689985, EPI_ISL_689986, EPI_ISL_689987, EPI_ISL_689988, EPI_ISL_689989, EPI_ISL_689990, EPI_ISL_689991, EPI_ISL_689992, EPI_ISL_689993, EPI_ISL_689994, EPI_ISL_689995, EPI_ISL_689996, EPI_ISL_689997, EPI_ISL_689998, EPI_ISL_689999, EPI_ISL_690000, EPI_ISL_690001, EPI_ISL_690002, EPI_ISL_690003, EPI_ISL_690004, EPI_ISL_690005, EPI_ISL_690006, EPI_ISL_690007, EPI_ISL_690008, EPI_ISL_690009, EPI_ISL_690010

see above	Pathogen Genomics Center, National Institute of Infectious Diseases	Pathogen Genomics Center, National Institute of Infectious Diseases	Tsuyoshi Sekizuka, Kentaro Itokawa, Rina Tanaka, Masanori Hashino, Makoto Kuroda

EPI_ISL_690011, EPI_ISL_690012, EPI_ISL_690013, EPI_ISL_690014, EPI_ISL_690015, EPI_ISL_690016, EPI_ISL_690017, EPI_ISL_690018, EPI_ISL_690019, EPI_ISL_690020, EPI_ISL_690021, EPI_ISL_690022, EPI_ISL_690023, EPI_ISL_690024, EPI_ISL_690025, EPI_ISL_690026, EPI_ISL_690027, EPI_ISL_690028

see above	Sakai City Institute of Public Health	Pathogen Genomics Center, National Institute of Infectious Diseases	Tsuyoshi Sekizuka, Kentaro Itokawa, Rina Tanaka, Masanori Hashino, Makoto Kuroda

EPI_ISL_690029, EPI_ISL_690030, EPI_ISL_690031, EPI_ISL_690032, EPI_ISL_690033, EPI_ISL_690034, EPI_ISL_690035, EPI_ISL_690036, EPI_ISL_690037, EPI_ISL_690038, EPI_ISL_690039, EPI_ISL_690040, EPI_ISL_690041, EPI_ISL_690042, EPI_ISL_690043, EPI_ISL_690044, EPI_ISL_690045, EPI_ISL_690046, EPI_ISL_690047, EPI_ISL_690048, EPI_ISL_690049, EPI_ISL_690050, EPI_ISL_690051, EPI_ISL_690052, EPI_ISL_690053, EPI_ISL_690054, EPI_ISL_690055, EPI_ISL_690056, EPI_ISL_690057, EPI_ISL_690058, EPI_ISL_690059, EPI_ISL_690060, EPI_ISL_690061, EPI_ISL_690062, EPI_ISL_690063, EPI_ISL_690064, EPI_ISL_690065, EPI_ISL_690066, EPI_ISL_690067, EPI_ISL_690068, EPI_ISL_690069, EPI_ISL_690070, EPI_ISL_690071, EPI_ISL_690072, EPI_ISL_690073, EPI_ISL_690074, EPI_ISL_690075, EPI_ISL_690076, EPI_ISL_690077, EPI_ISL_690078, EPI_ISL_690079, EPI_ISL_690080, EPI_ISL_690081, EPI_ISL_690082, EPI_ISL_690083, EPI_ISL_690084, EPI_ISL_690085, EPI_ISL_690086, EPI_ISL_690087, EPI_ISL_690088, EPI_ISL_690089, EPI_ISL_690090, EPI_ISL_690091, EPI_ISL_690092, EPI_ISL_690093, EPI_ISL_690094, EPI_ISL_690095, EPI_ISL_690096, EPI_ISL_690097, EPI_ISL_690098, EPI_ISL_690099, EPI_ISL_690100, EPI_ISL_690101, EPI_ISL_690102, EPI_ISL_690103, EPI_ISL_690104, EPI_ISL_690105, EPI_ISL_690106, EPI_ISL_690107, EPI_ISL_690108, EPI_ISL_690109, EPI_ISL_690110, EPI_ISL_690111, EPI_ISL_690112, EPI_ISL_690113, EPI_ISL_690114, EPI_ISL_690115, EPI_ISL_690116, EPI_ISL_690117, EPI_ISL_690118, EPI_ISL_690119, EPI_ISL_690120, EPI_ISL_690121, EPI_ISL_690122, EPI_ISL_690123, EPI_ISL_690124, EPI_ISL_690125, EPI_ISL_690126, EPI_ISL_690127, EPI_ISL_690128, EPI_ISL_690129, EPI_ISL_690130, EPI_ISL_690131, EPI_ISL_690132, EPI_ISL_690133, EPI_ISL_690134, EPI_ISL_690135, EPI_ISL_690136,

EPI_ISL_690137, EPI_ISL_690138, EPI_ISL_690139, EPI_ISL_690140, EPI_ISL_690141, EPI_ISL_690142, EPI_ISL_690143, EPI_ISL_690144, EPI_ISL_690145, EPI_ISL_690146, EPI_ISL_690147, EPI_ISL_690148, EPI_ISL_690149, EPI_ISL_690150, EPI_ISL_690151, EPI_ISL_690152, EPI_ISL_690153, EPI_ISL_690154, EPI_ISL_690155, EPI_ISL_690156, EPI_ISL_690157, EPI_ISL_690158, EPI_ISL_690159, EPI_ISL_690160, EPI_ISL_690161, EPI_ISL_690162, EPI_ISL_690163, EPI_ISL_690164, EPI_ISL_690165, EPI_ISL_690166, EPI_ISL_690167, EPI_ISL_690168, EPI_ISL_690169, EPI_ISL_690170, EPI_ISL_690171, EPI_ISL_690172, EPI_ISL_690173, EPI_ISL_690174, EPI_ISL_690175, EPI_ISL_690176, EPI_ISL_690177, EPI_ISL_690178, EPI_ISL_690179, EPI_ISL_690180, EPI_ISL_690181, EPI_ISL_690182, EPI_ISL_690183, EPI_ISL_690184, EPI_ISL_690185, EPI_ISL_690186, EPI_ISL_690187, EPI_ISL_690188, EPI_ISL_690189, EPI_ISL_690190, EPI_ISL_690191, EPI_ISL_690192, EPI_ISL_690193, EPI_ISL_690194, EPI_ISL_690195, EPI_ISL_690196, EPI_ISL_690197, EPI_ISL_690198, EPI_ISL_690199, EPI_ISL_690200, EPI_ISL_690201, EPI_ISL_690202, EPI_ISL_690203, EPI_ISL_690204, EPI_ISL_690205, EPI_ISL_690206, EPI_ISL_690207, EPI_ISL_690208, EPI_ISL_690209			
see above	Pathogen Genomics Center, National Institute of Infectious Diseases	Pathogen Genomics Center, National Institute of Infectious Diseases	Tsuyoshi Sekizuka, Kentaro Itokawa, Rina Tanaka, Masanori Hashino, Makoto Kuroda
EPI_ISL_690210, EPI_ISL_690211, EPI_ISL_690212, EPI_ISL_690213, EPI_ISL_690214, EPI_ISL_690215, EPI_ISL_690216, EPI_ISL_690217, EPI_ISL_690218, EPI_ISL_690219, EPI_ISL_690220, EPI_ISL_690221, EPI_ISL_690222, EPI_ISL_690223, EPI_ISL_690224, EPI_ISL_690225, EPI_ISL_690226, EPI_ISL_690227, EPI_ISL_690228, EPI_ISL_690229, EPI_ISL_690230, EPI_ISL_690231, EPI_ISL_690232, EPI_ISL_690233, EPI_ISL_690234, EPI_ISL_690235, EPI_ISL_690236, EPI_ISL_690237, EPI_ISL_690238, EPI_ISL_690239, EPI_ISL_690240, EPI_ISL_690241, EPI_ISL_690242, EPI_ISL_690243, EPI_ISL_690244, EPI_ISL_690245, EPI_ISL_690246, EPI_ISL_690247, EPI_ISL_690248, EPI_ISL_690249, EPI_ISL_690250, EPI_ISL_690251, EPI_ISL_690252, EPI_ISL_690253			
see above	Gunma Prefectural Institute of Public Health and Environmental Sciences	Pathogen Genomics Center, National Institute of Infectious Diseases	Tsuyoshi Sekizuka, Kentaro Itokawa, Rina Tanaka, Masanori Hashino, Makoto Kuroda
EPI_ISL_690254, EPI_ISL_690255, EPI_ISL_690256, EPI_ISL_690257, EPI_ISL_690258, EPI_ISL_690259, EPI_ISL_690260, EPI_ISL_690261, EPI_ISL_690262, EPI_ISL_690263, EPI_ISL_690264, EPI_ISL_690265, EPI_ISL_690266, EPI_ISL_690267, EPI_ISL_690268, EPI_ISL_690269, EPI_ISL_690270, EPI_ISL_690271, EPI_ISL_690272, EPI_ISL_690273, EPI_ISL_690274, EPI_ISL_690275, EPI_ISL_690276, EPI_ISL_690277, EPI_ISL_690278, EPI_ISL_690279, EPI_ISL_690280, EPI_ISL_690281, EPI_ISL_690282, EPI_ISL_690283, EPI_ISL_690284, EPI_ISL_690285, EPI_ISL_690286, EPI_ISL_690287, EPI_ISL_690288, EPI_ISL_690289, EPI_ISL_690290, EPI_ISL_690291, EPI_ISL_690292, EPI_ISL_690293, EPI_ISL_690294, EPI_ISL_690295, EPI_ISL_690296, EPI_ISL_690297, EPI_ISL_690298, EPI_ISL_690299, EPI_ISL_690300, EPI_ISL_690301, EPI_ISL_690302, EPI_ISL_690303, EPI_ISL_690304, EPI_ISL_690305, EPI_ISL_690306, EPI_ISL_690307, EPI_ISL_690308, EPI_ISL_690309, EPI_ISL_690310, EPI_ISL_690311, EPI_ISL_690312, EPI_ISL_690313, EPI_ISL_690314, EPI_ISL_690315, EPI_ISL_690316, EPI_ISL_690317, EPI_ISL_690318, EPI_ISL_690319, EPI_ISL_690320, EPI_ISL_690321, EPI_ISL_690322, EPI_ISL_690323, EPI_ISL_690324, EPI_ISL_690325, EPI_ISL_690326, EPI_ISL_690327, EPI_ISL_690328, EPI_ISL_690329, EPI_ISL_690330, EPI_ISL_690331, EPI_ISL_690332, EPI_ISL_690333, EPI_ISL_690334, EPI_ISL_690335, EPI_ISL_690336, EPI_ISL_690337, EPI_ISL_690338, EPI_ISL_690339, EPI_ISL_690340, EPI_ISL_690341, EPI_ISL_690342, EPI_ISL_690343, EPI_ISL_690344, EPI_ISL_690345, EPI_ISL_690346, EPI_ISL_690347, EPI_ISL_690348, EPI_ISL_690349, EPI_ISL_690350, EPI_ISL_690351, EPI_ISL_690352, EPI_ISL_690353, EPI_ISL_690354, EPI_ISL_690355, EPI_ISL_690356, EPI_ISL_690357, EPI_ISL_690358, EPI_ISL_690359, EPI_ISL_690360, EPI_ISL_690361, EPI_ISL_690362, EPI_ISL_690363, EPI_ISL_690364, EPI_ISL_690365, EPI_ISL_690366, EPI_ISL_690367, EPI_ISL_690368, EPI_ISL_690369, EPI_ISL_690370, EPI_ISL_690371, EPI_ISL_690372, EPI_ISL_690373, EPI_ISL_690374, EPI_ISL_690375, EPI_ISL_690376, EPI_ISL_690377, EPI_ISL_690378, EPI_ISL_690379, EPI_ISL_690380, EPI_ISL_690381, EPI_ISL_690382, EPI_ISL_690383, EPI_ISL_690384, EPI_ISL_690385, EPI_ISL_690386, EPI_ISL_690387, EPI_ISL_690388, EPI_ISL_690389, EPI_ISL_690390, EPI_ISL_690391, EPI_ISL_690392, EPI_ISL_690393, EPI_ISL_690394, EPI_ISL_690395, EPI_ISL_690396, EPI_ISL_690397, EPI_ISL_690398, EPI_ISL_690399, EPI_ISL_690400, EPI_ISL_690401, EPI_ISL_690402, EPI_ISL_690403, EPI_ISL_690404, EPI_ISL_690405, EPI_ISL_690406, EPI_ISL_690407, EPI_ISL_690408, EPI_ISL_690409, EPI_ISL_690410, EPI_ISL_690411, EPI_ISL_690412, EPI_ISL_690413, EPI_ISL_690414, EPI_ISL_690415, EPI_ISL_690416, EPI_ISL_690417, EPI_ISL_690418, EPI_ISL_690419, EPI_ISL_690420, EPI_ISL_690421, EPI_ISL_690422, EPI_ISL_690423, EPI_ISL_690424, EPI_ISL_690425, EPI_ISL_690426, EPI_ISL_690427, EPI_ISL_690428, EPI_ISL_690429, EPI_ISL_690430, EPI_ISL_690431			
see above	Pathogen Genomics Center, National Institute of Infectious Diseases	Pathogen Genomics Center, National Institute of Infectious Diseases	Tsuyoshi Sekizuka, Kentaro Itokawa, Rina Tanaka, Masanori Hashino, Makoto Kuroda
EPI_ISL_690432, EPI_ISL_690433, EPI_ISL_690434, EPI_ISL_690435, EPI_ISL_690436, EPI_ISL_690437, EPI_ISL_690438, EPI_ISL_690439, EPI_ISL_690440, EPI_ISL_690441, EPI_ISL_690442, EPI_ISL_690443, EPI_ISL_690444, EPI_ISL_690445, EPI_ISL_690446, EPI_ISL_690447, EPI_ISL_690448, EPI_ISL_690449, EPI_ISL_690450, EPI_ISL_690451, EPI_ISL_690452, EPI_ISL_690453, EPI_ISL_690454, EPI_ISL_690455, EPI_ISL_690456, EPI_ISL_690457, EPI_ISL_690458, EPI_ISL_690459, EPI_ISL_690460, EPI_ISL_690461, EPI_ISL_690462, EPI_ISL_690463, EPI_ISL_690464, EPI_ISL_690465, EPI_ISL_690466, EPI_ISL_690467, EPI_ISL_690468, EPI_ISL_690469, EPI_ISL_690470, EPI_ISL_690471, EPI_ISL_690472, EPI_ISL_690473, EPI_ISL_690474, EPI_ISL_690475, EPI_ISL_690476, EPI_ISL_690477, EPI_ISL_690478, EPI_ISL_690479, EPI_ISL_690480, EPI_ISL_690481, EPI_ISL_690482, EPI_ISL_690483, EPI_ISL_690484, EPI_ISL_690485, EPI_ISL_690486, EPI_ISL_690487, EPI_ISL_690488, EPI_ISL_690489, EPI_ISL_690490, EPI_ISL_690491, EPI_ISL_690492, EPI_ISL_690493, EPI_ISL_690494, EPI_ISL_690495, EPI_ISL_690496, EPI_ISL_690497, EPI_ISL_690498, EPI_ISL_690499, EPI_ISL_690500, EPI_ISL_690501, EPI_ISL_690502, EPI_ISL_690503, EPI_ISL_690504, EPI_ISL_690505, EPI_ISL_690506, EPI_ISL_690507, EPI_ISL_690508, EPI_ISL_690509, EPI_ISL_690510, EPI_ISL_690511, EPI_ISL_690512, EPI_ISL_690513, EPI_ISL_690514, EPI_ISL_690515, EPI_ISL_690516, EPI_ISL_690517, EPI_ISL_690518, EPI_ISL_690519, EPI_ISL_690520, EPI_ISL_690521, EPI_ISL_690522, EPI_ISL_690523, EPI_ISL_690524, EPI_ISL_690525, EPI_ISL_690526			
see above	Fukuoka Institute of Health and Environmental Sciences	Pathogen Genomics Center, National Institute of Infectious Diseases	Tsuyoshi Sekizuka, Kentaro Itokawa, Rina Tanaka, Masanori Hashino, Makoto Kuroda
EPI_ISL_690527, EPI_ISL_690528, EPI_ISL_690529, EPI_ISL_690530, EPI_ISL_690531, EPI_ISL_690532, EPI_ISL_690533, EPI_ISL_690534, EPI_ISL_690535, EPI_ISL_690536, EPI_ISL_690537, EPI_ISL_690538, EPI_ISL_690539, EPI_ISL_690540, EPI_ISL_690541, EPI_ISL_690542, EPI_ISL_690543, EPI_ISL_690544, EPI_ISL_690545, EPI_ISL_690546, EPI_ISL_690547, EPI_ISL_690548, EPI_ISL_690549, EPI_ISL_690550, EPI_ISL_690551, EPI_ISL_690552, EPI_ISL_690553, EPI_ISL_690554, EPI_ISL_690555, EPI_ISL_690556, EPI_ISL_690557, EPI_ISL_690558, EPI_ISL_690559, EPI_ISL_690560, EPI_ISL_690561, EPI_ISL_690562, EPI_ISL_690563, EPI_ISL_690564, EPI_ISL_690565, EPI_ISL_690566, EPI_ISL_690567, EPI_ISL_690568, EPI_ISL_690569, EPI_ISL_690570, EPI_ISL_690571, EPI_ISL_690572, EPI_ISL_690573, EPI_ISL_690574, EPI_ISL_690575, EPI_ISL_690576, EPI_ISL_690577, EPI_ISL_690578, EPI_ISL_690579, EPI_ISL_690580, EPI_ISL_690581, EPI_ISL_690582, EPI_ISL_690583, EPI_ISL_690584, EPI_ISL_690585, EPI_ISL_690586, EPI_ISL_690587, EPI_ISL_690588, EPI_ISL_690589, EPI_ISL_690590, EPI_ISL_690591, EPI_ISL_690592, EPI_ISL_690593, EPI_ISL_690594, EPI_ISL_690595, EPI_ISL_690596, EPI_ISL_690597, EPI_ISL_690598, EPI_ISL_690599, EPI_ISL_690600, EPI_ISL_690601, EPI_ISL_690602, EPI_ISL_690603, EPI_ISL_690604, EPI_ISL_690605, EPI_ISL_690606, EPI_ISL_690607, EPI_ISL_690608, EPI_ISL_690609, EPI_ISL_690610, EPI_ISL_690611, EPI_ISL_690616, EPI_ISL_690624, EPI_ISL_690632, EPI_ISL_690633, EPI_ISL_690634, EPI_ISL_690635			
see above	Pathogen Genomics Center, National Institute of Infectious Diseases	Pathogen Genomics Center, National Institute of Infectious Diseases	Tsuyoshi Sekizuka, Kentaro Itokawa, Rina Tanaka, Masanori Hashino, Makoto Kuroda
EPI_ISL_690638, EPI_ISL_690639, EPI_ISL_690640	Tokyo Metropolitan Institute of Public Health	Pathogen Genomics Center, National Institute of Infectious Diseases	Tsuyoshi Sekizuka, Kentaro Itokawa, Rina Tanaka, Masanori Hashino, Makoto Kuroda
EPI_ISL_690681, EPI_ISL_690682, EPI_ISL_690683	Saitama Prefectural Institute of Public Health	Pathogen Genomics Center, National Institute of Infectious Diseases	Tsuyoshi Sekizuka, Kentaro Itokawa, Rina Tanaka, Masanori Hashino, Makoto Kuroda
EPI_ISL_690687, EPI_ISL_690688, EPI_ISL_690689, EPI_ISL_690690, EPI_ISL_690691, EPI_ISL_690692, EPI_ISL_690693, EPI_ISL_690694, EPI_ISL_690695, EPI_ISL_690696, EPI_ISL_690697, EPI_ISL_690698, EPI_ISL_690699, EPI_ISL_690700, EPI_ISL_690701, EPI_ISL_690702, EPI_ISL_690703, EPI_ISL_690704, EPI_ISL_690705, EPI_ISL_690706, EPI_ISL_690707, EPI_ISL_690744, EPI_ISL_690778			
see above	Pathogen Genomics Center, National Institute of Infectious Diseases	Pathogen Genomics Center, National Institute of Infectious Diseases	Tsuyoshi Sekizuka, Kentaro Itokawa, Rina Tanaka, Masanori Hashino, Makoto Kuroda
EPI_ISL_690794, EPI_ISL_690795, EPI_ISL_690796, EPI_ISL_690797, EPI_ISL_690798, EPI_ISL_690799, EPI_ISL_690800, EPI_ISL_690801, EPI_ISL_690802, EPI_ISL_690803, EPI_ISL_690804, EPI_ISL_690805, EPI_ISL_690806, EPI_ISL_690807, EPI_ISL_690808, EPI_ISL_690809, EPI_ISL_690810, EPI_ISL_690811, EPI_ISL_690812, EPI_ISL_690813, EPI_ISL_690814, EPI_ISL_690815, EPI_ISL_690816, EPI_ISL_690817			
see above	Tokyo Metropolitan Institute of Public Health	Pathogen Genomics Center, National Institute of Infectious Diseases	Tsuyoshi Sekizuka, Kentaro Itokawa, Rina Tanaka, Masanori Hashino, Makoto Kuroda
EPI_ISL_690844	Sakai City Institute of Public Health	Pathogen Genomics Center, National Institute of Infectious Diseases	Tsuyoshi Sekizuka, Kentaro Itokawa, Rina Tanaka, Masanori Hashino, Makoto Kuroda
EPI_ISL_690902, EPI_ISL_690903, EPI_ISL_690904, EPI_ISL_690905, EPI_ISL_690906, EPI_ISL_690907, EPI_ISL_690908	Tokyo Metropolitan Institute of Public Health	Pathogen Genomics Center, National Institute of Infectious Diseases	Tsuyoshi Sekizuka, Kentaro Itokawa, Rina Tanaka, Masanori Hashino, Makoto Kuroda
EPI_ISL_690981, EPI_ISL_691108, EPI_ISL_691239, EPI_ISL_691248, EPI_ISL_691252, EPI_ISL_691253, EPI_ISL_691254, EPI_ISL_691255, EPI_ISL_691256, EPI_ISL_691260, EPI_ISL_691261, EPI_ISL_691262, EPI_ISL_691263, EPI_ISL_691264, EPI_ISL_691265, EPI_ISL_691266, EPI_ISL_691267, EPI_ISL_691268, EPI_ISL_691269, EPI_ISL_691270, EPI_ISL_691271, EPI_ISL_691272, EPI_ISL_691275, EPI_ISL_691276, EPI_ISL_691277, EPI_ISL_691278, EPI_ISL_691279, EPI_ISL_691280, EPI_ISL_691281, EPI_ISL_691282, EPI_ISL_691283			
see above	Pathogen Genomics Center, National Institute of Infectious Diseases	Pathogen Genomics Center, National Institute of Infectious Diseases	Tsuyoshi Sekizuka, Kentaro Itokawa, Rina Tanaka, Masanori Hashino, Makoto Kuroda
EPI_ISL_691284, EPI_ISL_691285, EPI_ISL_691286, EPI_ISL_691287, EPI_ISL_691288	Chiba Prefectural Institute of Public Health	Pathogen Genomics Center, National Institute of Infectious Diseases	Tsuyoshi Sekizuka, Kentaro Itokawa, Rina Tanaka, Masanori Hashino, Makoto Kuroda
EPI_ISL_691364, EPI_ISL_691365, EPI_ISL_691366, EPI_ISL_691367, EPI_ISL_691368, EPI_ISL_691369, EPI_ISL_691370, EPI_ISL_691371, EPI_ISL_691372, EPI_ISL_691373, EPI_ISL_691374, EPI_ISL_691375, EPI_ISL_691376, EPI_ISL_691377, EPI_ISL_691378			
see above	Saitama Prefectural Institute of Public Health	Pathogen Genomics Center, National Institute of Infectious Diseases	Tsuyoshi Sekizuka, Kentaro Itokawa, Rina Tanaka, Masanori Hashino, Makoto Kuroda
EPI_ISL_691386, EPI_ISL_691387, EPI_ISL_691388, EPI_ISL_691389, EPI_ISL_691390, EPI_ISL_691391, EPI_ISL_691392, EPI_ISL_691393, EPI_ISL_691394, EPI_ISL_691502, EPI_ISL_691503, EPI_ISL_691504, EPI_ISL_691505, EPI_ISL_691506			
see above	Pathogen Genomics Center, National Institute of Infectious Diseases	Pathogen Genomics Center, National Institute of Infectious Diseases	Tsuyoshi Sekizuka, Kentaro Itokawa, Rina Tanaka, Masanori Hashino, Makoto Kuroda
EPI_ISL_691543, EPI_ISL_691544, EPI_ISL_691545	Sakai City Institute of Public Health	Pathogen Genomics Center, National Institute of Infectious Diseases	Tsuyoshi Sekizuka, Kentaro Itokawa, Rina Tanaka, Masanori Hashino, Makoto Kuroda
EPI_ISL_691546, EPI_ISL_691547,	Pathogen Genomics Center, National Institute of Infectious	Pathogen Genomics Center, National Institute of Infectious	Tsuyoshi Sekizuka, Kentaro Itokawa, Rina Tanaka, Masanori Hashino, Makoto Kuroda

EPI_ISL_691548, EPI_ISL_691549	Diseases	Diseases	
EPI_ISL_691550, EPI_ISL_691551	Fukuoka Institute of Health and Environmental Sciences	Pathogen Genomics Center, National Institute of Infectious Diseases	Tsuyoshi Sekizuka, Kentaro Itokawa, Rina Tanaka, Masanori Hashino, Makoto Kuroda
EPI_ISL_691552, EPI_ISL_691553, EPI_ISL_691554, EPI_ISL_691555, EPI_ISL_691556, EPI_ISL_691557, EPI_ISL_691558, EPI_ISL_691559, EPI_ISL_691560, EPI_ISL_691561, EPI_ISL_691562, EPI_ISL_691563, EPI_ISL_691564, EPI_ISL_691565, EPI_ISL_691566, EPI_ISL_691567, EPI_ISL_691568, EPI_ISL_691569, EPI_ISL_691570, EPI_ISL_691571, EPI_ISL_691572, EPI_ISL_691573, EPI_ISL_691574, EPI_ISL_691575, EPI_ISL_691576, EPI_ISL_691577, EPI_ISL_691578, EPI_ISL_691579, EPI_ISL_691580, EPI_ISL_691581, EPI_ISL_691582, EPI_ISL_691583, EPI_ISL_691584, EPI_ISL_691585, EPI_ISL_691586, EPI_ISL_691587, EPI_ISL_691588, EPI_ISL_691589, EPI_ISL_691590, EPI_ISL_691591, EPI_ISL_691592, EPI_ISL_691593, EPI_ISL_691594, EPI_ISL_691595, EPI_ISL_691596, EPI_ISL_691597, EPI_ISL_691598, EPI_ISL_691599, EPI_ISL_691600, EPI_ISL_691601, EPI_ISL_691602, EPI_ISL_691603, EPI_ISL_691604, EPI_ISL_691605, EPI_ISL_691606, EPI_ISL_691607, EPI_ISL_691608, EPI_ISL_691609, EPI_ISL_691610, EPI_ISL_691611			
see above	Pathogen Genomics Center, National Institute of Infectious Diseases	Pathogen Genomics Center, National Institute of Infectious Diseases	Tsuyoshi Sekizuka, Kentaro Itokawa, Rina Tanaka, Masanori Hashino, Makoto Kuroda
EPI_ISL_691700, EPI_ISL_691701, EPI_ISL_691702, EPI_ISL_691703, EPI_ISL_691704, EPI_ISL_691705, EPI_ISL_691706, EPI_ISL_691707, EPI_ISL_691708, EPI_ISL_691709, EPI_ISL_691710, EPI_ISL_691711, EPI_ISL_691712, EPI_ISL_691713, EPI_ISL_691714, EPI_ISL_691715, EPI_ISL_691716, EPI_ISL_691717, EPI_ISL_691718, EPI_ISL_691719, EPI_ISL_691720, EPI_ISL_691721, EPI_ISL_691722, EPI_ISL_691723, EPI_ISL_691724, EPI_ISL_691725, EPI_ISL_691726, EPI_ISL_691727, EPI_ISL_691728			
see above	Hospital Clínico San Carlos	Instituto de Salud Carlos III	Iglesias-Caballero, M. Camarero, S. Molinero Calamita, M. González-Esguevillas, M. Pozo, F. Casas, I. Jiménez, P. Jiménez, M. Zaballos, A. Monzón, S. Varona, S. Juliá, M. Cuesta, I. Rodríguez, I.
EPI_ISL_691744, EPI_ISL_691748, EPI_ISL_691752	Pathogen Genomics Center, National Institute of Infectious Diseases	Pathogen Genomics Center, National Institute of Infectious Diseases	Tsuyoshi Sekizuka, Kentaro Itokawa, Rina Tanaka, Masanori Hashino, Makoto Kuroda
EPI_ISL_691753	Sakai City Institute of Public Health	Pathogen Genomics Center, National Institute of Infectious Diseases	Tsuyoshi Sekizuka, Kentaro Itokawa, Rina Tanaka, Masanori Hashino, Makoto Kuroda
EPI_ISL_691754, EPI_ISL_691759, EPI_ISL_691760, EPI_ISL_691761, EPI_ISL_691762, EPI_ISL_691763	Pathogen Genomics Center, National Institute of Infectious Diseases	Pathogen Genomics Center, National Institute of Infectious Diseases	Tsuyoshi Sekizuka, Kentaro Itokawa, Rina Tanaka, Masanori Hashino, Makoto Kuroda
EPI_ISL_691764, EPI_ISL_691765	Fukuoka Institute of Health and Environmental Sciences	Pathogen Genomics Center, National Institute of Infectious Diseases	Tsuyoshi Sekizuka, Kentaro Itokawa, Rina Tanaka, Masanori Hashino, Makoto Kuroda
EPI_ISL_691766	Saitama Prefectural Institute of Public Health	Pathogen Genomics Center, National Institute of Infectious Diseases	Tsuyoshi Sekizuka, Kentaro Itokawa, Rina Tanaka, Masanori Hashino, Makoto Kuroda
EPI_ISL_691768, EPI_ISL_691769, EPI_ISL_691770, EPI_ISL_691771, EPI_ISL_691772, EPI_ISL_691773, EPI_ISL_691774, EPI_ISL_691775, EPI_ISL_691776, EPI_ISL_691777, EPI_ISL_691778, EPI_ISL_691779, EPI_ISL_691780, EPI_ISL_691781, EPI_ISL_691782, EPI_ISL_691783, EPI_ISL_691784, EPI_ISL_691785, EPI_ISL_691786, EPI_ISL_691787, EPI_ISL_691788, EPI_ISL_691789, EPI_ISL_691790, EPI_ISL_691791, EPI_ISL_691792, EPI_ISL_691793, EPI_ISL_691794, EPI_ISL_691795, EPI_ISL_691796, EPI_ISL_691797, EPI_ISL_691798, EPI_ISL_691799, EPI_ISL_691800, EPI_ISL_691801, EPI_ISL_691802, EPI_ISL_691803, EPI_ISL_691804, EPI_ISL_691805, EPI_ISL_691806, EPI_ISL_691807, EPI_ISL_691808, EPI_ISL_691809, EPI_ISL_691810, EPI_ISL_691811, EPI_ISL_691812, EPI_ISL_691813, EPI_ISL_691814, EPI_ISL_691815, EPI_ISL_691816, EPI_ISL_691817, EPI_ISL_691818, EPI_ISL_691819, EPI_ISL_691820, EPI_ISL_691821, EPI_ISL_691822, EPI_ISL_691823, EPI_ISL_691824, EPI_ISL_691825, EPI_ISL_691826, EPI_ISL_691827, EPI_ISL_691828, EPI_ISL_691829, EPI_ISL_691830, EPI_ISL_691831, EPI_ISL_691832, EPI_ISL_691833, EPI_ISL_691834, EPI_ISL_691835, EPI_ISL_691836, EPI_ISL_691837, EPI_ISL_691838, EPI_ISL_691839, EPI_ISL_691840, EPI_ISL_691841, EPI_ISL_691842, EPI_ISL_691843, EPI_ISL_691844, EPI_ISL_691845, EPI_ISL_691846, EPI_ISL_691847, EPI_ISL_691848, EPI_ISL_691849, EPI_ISL_691850, EPI_ISL_691851, EPI_ISL_691852, EPI_ISL_691853, EPI_ISL_691854, EPI_ISL_691855, EPI_ISL_691856, EPI_ISL_691857, EPI_ISL_691858, EPI_ISL_691859, EPI_ISL_691860, EPI_ISL_691862, EPI_ISL_691863, EPI_ISL_691864, EPI_ISL_691865, EPI_ISL_691866, EPI_ISL_691867, EPI_ISL_691868, EPI_ISL_691869, EPI_ISL_691870, EPI_ISL_691871			
see above	Pathogen Genomics Center, National Institute of Infectious Diseases	Pathogen Genomics Center, National Institute of Infectious Diseases	Tsuyoshi Sekizuka, Kentaro Itokawa, Rina Tanaka, Masanori Hashino, Makoto Kuroda
EPI_ISL_692047	Gunma Prefectural Institute of Public Health and Environmental Sciences	Pathogen Genomics Center, National Institute of Infectious Diseases	Tsuyoshi Sekizuka, Kentaro Itokawa, Rina Tanaka, Masanori Hashino, Makoto Kuroda
EPI_ISL_692048, EPI_ISL_692049, EPI_ISL_692050	Fukuoka Institute of Health and Environmental Sciences	Pathogen Genomics Center, National Institute of Infectious Diseases	Tsuyoshi Sekizuka, Kentaro Itokawa, Rina Tanaka, Masanori Hashino, Makoto Kuroda
EPI_ISL_692051, EPI_ISL_692052	Niigata Prefectural Institute of Public Health and Environmental Sciences	Pathogen Genomics Center, National Institute of Infectious Diseases	Tsuyoshi Sekizuka, Kentaro Itokawa, Rina Tanaka, Masanori Hashino, Makoto Kuroda
EPI_ISL_692053, EPI_ISL_692054, EPI_ISL_692055, EPI_ISL_692056, EPI_ISL_692057, EPI_ISL_692058	Sakai City Institute of Public Health	Pathogen Genomics Center, National Institute of Infectious Diseases	Tsuyoshi Sekizuka, Kentaro Itokawa, Rina Tanaka, Masanori Hashino, Makoto Kuroda
EPI_ISL_692061, EPI_ISL_692062, EPI_ISL_692063, EPI_ISL_692064, EPI_ISL_692065, EPI_ISL_692066, EPI_ISL_692067, EPI_ISL_692068, EPI_ISL_692069, EPI_ISL_692070, EPI_ISL_692071, EPI_ISL_692072, EPI_ISL_692073, EPI_ISL_692074, EPI_ISL_692075, EPI_ISL_692076, EPI_ISL_692077, EPI_ISL_692078, EPI_ISL_692079, EPI_ISL_692080, EPI_ISL_692081, EPI_ISL_692082, EPI_ISL_692083, EPI_ISL_692084, EPI_ISL_692085, EPI_ISL_692086, EPI_ISL_692087, EPI_ISL_692088, EPI_ISL_692089, EPI_ISL_692090, EPI_ISL_692091, EPI_ISL_692092, EPI_ISL_692093			
see above	Pathogen Genomics Center, National Institute of Infectious Diseases	Pathogen Genomics Center, National Institute of Infectious Diseases	Tsuyoshi Sekizuka, Kentaro Itokawa, Rina Tanaka, Masanori Hashino, Makoto Kuroda
EPI_ISL_692094, EPI_ISL_692095, EPI_ISL_692096, EPI_ISL_692097	Yamagata Prefectural Institute of Public Health	Pathogen Genomics Center, National Institute of Infectious Diseases	Tsuyoshi Sekizuka, Kentaro Itokawa, Rina Tanaka, Masanori Hashino, Makoto Kuroda
EPI_ISL_692098, EPI_ISL_692099, EPI_ISL_692100, EPI_ISL_692101, EPI_ISL_692102, EPI_ISL_692103, EPI_ISL_692104, EPI_ISL_692105, EPI_ISL_692106, EPI_ISL_692107, EPI_ISL_692108, EPI_ISL_692109, EPI_ISL_692110, EPI_ISL_692111, EPI_ISL_692112, EPI_ISL_692113, EPI_ISL_692114, EPI_ISL_692115, EPI_ISL_692116, EPI_ISL_692117, EPI_ISL_692118			
see above	Pathogen Genomics Center, National Institute of Infectious Diseases	Pathogen Genomics Center, National Institute of Infectious Diseases	Tsuyoshi Sekizuka, Kentaro Itokawa, Rina Tanaka, Masanori Hashino, Makoto Kuroda
EPI_ISL_692119, EPI_ISL_692120, EPI_ISL_692121, EPI_ISL_692122, EPI_ISL_692123, EPI_ISL_692124, EPI_ISL_692125, EPI_ISL_692126, EPI_ISL_692127, EPI_ISL_692128	Tokyo Metropolitan Institute of Public Health	Pathogen Genomics Center, National Institute of Infectious Diseases	Tsuyoshi Sekizuka, Kentaro Itokawa, Rina Tanaka, Masanori Hashino, Makoto Kuroda
EPI_ISL_692129, EPI_ISL_692130, EPI_ISL_692131, EPI_ISL_692132, EPI_ISL_692133, EPI_ISL_692134, EPI_ISL_692135, EPI_ISL_692136, EPI_ISL_692137, EPI_ISL_692138, EPI_ISL_692139, EPI_ISL_692140, EPI_ISL_692141, EPI_ISL_692142, EPI_ISL_692143, EPI_ISL_692144, EPI_ISL_692145, EPI_ISL_692146, EPI_ISL_692147, EPI_ISL_692148, EPI_ISL_692149, EPI_ISL_692150, EPI_ISL_692151, EPI_ISL_692152, EPI_ISL_692153, EPI_ISL_692154, EPI_ISL_692155, EPI_ISL_692156, EPI_ISL_692157, EPI_ISL_692158, EPI_ISL_692159, EPI_ISL_692160, EPI_ISL_692161			
see above	Pathogen Genomics Center, National Institute of Infectious Diseases	Pathogen Genomics Center, National Institute of Infectious Diseases	Tsuyoshi Sekizuka, Kentaro Itokawa, Rina Tanaka, Masanori Hashino, Makoto Kuroda
EPI_ISL_692162, EPI_ISL_692163	Tokyo Metropolitan Institute of Public Health	Pathogen Genomics Center, National Institute of Infectious Diseases	Tsuyoshi Sekizuka, Kentaro Itokawa, Rina Tanaka, Masanori Hashino, Makoto Kuroda
EPI_ISL_692164, EPI_ISL_692165, EPI_ISL_692166, EPI_ISL_692167, EPI_ISL_692168, EPI_ISL_692169, EPI_ISL_692170, EPI_ISL_692171, EPI_ISL_692172, EPI_ISL_692173, EPI_ISL_692174, EPI_ISL_692175, EPI_ISL_692176, EPI_ISL_692177, EPI_ISL_692178, EPI_ISL_692179, EPI_ISL_692180, EPI_ISL_692181, EPI_ISL_692182, EPI_ISL_692183, EPI_ISL_692184, EPI_ISL_692185, EPI_ISL_692186, EPI_ISL_692187, EPI_ISL_692188, EPI_ISL_692189, EPI_ISL_692190, EPI_ISL_692191, EPI_ISL_692192, EPI_ISL_692193, EPI_ISL_692194, EPI_ISL_692195, EPI_ISL_692196, EPI_ISL_692198, EPI_ISL_692199, EPI_ISL_692200, EPI_ISL_692201, EPI_ISL_692202, EPI_ISL_692203, EPI_ISL_692204, EPI_ISL_692205, EPI_ISL_692206, EPI_ISL_692207, EPI_ISL_692208, EPI_ISL_692209, EPI_ISL_692210, EPI_ISL_692211, EPI_ISL_692212, EPI_ISL_692213, EPI_ISL_692214, EPI_ISL_692215, EPI_ISL_692216, EPI_ISL_692217, EPI_ISL_692218			
see above	Pathogen Genomics Center, National Institute of Infectious Diseases	Pathogen Genomics Center, National Institute of Infectious Diseases	Tsuyoshi Sekizuka, Kentaro Itokawa, Rina Tanaka, Masanori Hashino, Makoto Kuroda
EPI_ISL_692219, EPI_ISL_692220, EPI_ISL_692221, EPI_ISL_692222, EPI_ISL_692223, EPI_ISL_692224, EPI_ISL_692225, EPI_ISL_692226, EPI_ISL_692227, EPI_ISL_692228, EPI_ISL_692229			
see above	Fukuoka Institute of Health and Environmental Sciences	Pathogen Genomics Center, National Institute of Infectious Diseases	Tsuyoshi Sekizuka, Kentaro Itokawa, Rina Tanaka, Masanori Hashino, Makoto Kuroda
EPI_ISL_692230, EPI_ISL_692231, EPI_ISL_692232, EPI_ISL_692233, EPI_ISL_692234, EPI_ISL_692235, EPI_ISL_692236, EPI_ISL_692237, EPI_ISL_692238, EPI_ISL_692239, EPI_ISL_692240, EPI_ISL_692241, EPI_ISL_692242, EPI_ISL_692243, EPI_ISL_692244, EPI_ISL_692245, EPI_ISL_692246, EPI_ISL_692247, EPI_ISL_692248, EPI_ISL_692249, EPI_ISL_692250, EPI_ISL_692251, EPI_ISL_692252, EPI_ISL_692253, EPI_ISL_692254, EPI_ISL_692255, EPI_ISL_692256, EPI_ISL_692257, EPI_ISL_692258, EPI_ISL_692259, EPI_ISL_692260, EPI_ISL_692261, EPI_ISL_692262, EPI_ISL_692263, EPI_ISL_692264, EPI_ISL_692265, EPI_ISL_692266, EPI_ISL_692267, EPI_ISL_692268, EPI_ISL_692269, EPI_ISL_692270, EPI_ISL_692271, EPI_ISL_692272, EPI_ISL_692273, EPI_ISL_692274, EPI_ISL_692275, EPI_ISL_692276, EPI_ISL_692277, EPI_ISL_692278, EPI_ISL_692279, EPI_ISL_692280, EPI_ISL_692281, EPI_ISL_692282, EPI_ISL_692283			

see above	Pathogen Genomics Center, National Institute of Infectious Diseases	Pathogen Genomics Center, National Institute of Infectious Diseases	Tsuyoshi Sekizuka, Kentaro Itokawa, Rina Tanaka, Masanori Hashino, Makoto Kuroda
EPI_ISL_692284, EPI_ISL_692285, EPI_ISL_692286, EPI_ISL_692287, EPI_ISL_692288, EPI_ISL_692289, EPI_ISL_692290, EPI_ISL_692291, EPI_ISL_692292, EPI_ISL_692293, EPI_ISL_692294, EPI_ISL_692295, EPI_ISL_692296, EPI_ISL_692297, EPI_ISL_692298, EPI_ISL_692299, EPI_ISL_692300, EPI_ISL_692301, EPI_ISL_692302, EPI_ISL_692303, EPI_ISL_692304, EPI_ISL_692305, EPI_ISL_692306, EPI_ISL_692307, EPI_ISL_692308, EPI_ISL_692309, EPI_ISL_692310, EPI_ISL_692311, EPI_ISL_692312, EPI_ISL_692313, EPI_ISL_692314, EPI_ISL_692315			
see above	Sakai City Institute of Public Health	Pathogen Genomics Center, National Institute of Infectious Diseases	Tsuyoshi Sekizuka, Kentaro Itokawa, Rina Tanaka, Masanori Hashino, Makoto Kuroda
EPI_ISL_692316, EPI_ISL_692317, EPI_ISL_692318, EPI_ISL_692319, EPI_ISL_692320, EPI_ISL_692321, EPI_ISL_692322, EPI_ISL_692323, EPI_ISL_692324, EPI_ISL_692325, EPI_ISL_692326, EPI_ISL_692327			
see above	Gunma Prefectural Institute of Public Health and Environmental Sciences	Pathogen Genomics Center, National Institute of Infectious Diseases	Tsuyoshi Sekizuka, Kentaro Itokawa, Rina Tanaka, Masanori Hashino, Makoto Kuroda
EPI_ISL_692328, EPI_ISL_692329, EPI_ISL_692330, EPI_ISL_692331, EPI_ISL_692332, EPI_ISL_692333, EPI_ISL_692334, EPI_ISL_692335, EPI_ISL_692336, EPI_ISL_692337, EPI_ISL_692338, EPI_ISL_692339, EPI_ISL_692340, EPI_ISL_692341, EPI_ISL_692342, EPI_ISL_692343, EPI_ISL_692344, EPI_ISL_692345, EPI_ISL_692346, EPI_ISL_692347, EPI_ISL_692348, EPI_ISL_692349, EPI_ISL_692350, EPI_ISL_692351, EPI_ISL_692352, EPI_ISL_692353, EPI_ISL_692354, EPI_ISL_692355, EPI_ISL_692356, EPI_ISL_692357, EPI_ISL_692358, EPI_ISL_692359, EPI_ISL_692360, EPI_ISL_692361, EPI_ISL_692362, EPI_ISL_692363, EPI_ISL_692364, EPI_ISL_692365, EPI_ISL_692366, EPI_ISL_692367, EPI_ISL_692368, EPI_ISL_692369, EPI_ISL_692370, EPI_ISL_692371, EPI_ISL_692372, EPI_ISL_692373, EPI_ISL_692374, EPI_ISL_692375, EPI_ISL_692376, EPI_ISL_692377, EPI_ISL_692378, EPI_ISL_692379, EPI_ISL_692380, EPI_ISL_692381, EPI_ISL_692382, EPI_ISL_692383, EPI_ISL_692384, EPI_ISL_692385, EPI_ISL_692386, EPI_ISL_692387, EPI_ISL_692388, EPI_ISL_692389, EPI_ISL_692390, EPI_ISL_692391, EPI_ISL_692392, EPI_ISL_692393, EPI_ISL_692394, EPI_ISL_692395, EPI_ISL_692396, EPI_ISL_692397, EPI_ISL_692398, EPI_ISL_692399, EPI_ISL_692400, EPI_ISL_692401, EPI_ISL_692402, EPI_ISL_692403, EPI_ISL_692404, EPI_ISL_692405, EPI_ISL_692406, EPI_ISL_692407, EPI_ISL_692408, EPI_ISL_692409, EPI_ISL_692410, EPI_ISL_692411, EPI_ISL_692412, EPI_ISL_692413, EPI_ISL_692414, EPI_ISL_692415, EPI_ISL_692416, EPI_ISL_692417, EPI_ISL_692418, EPI_ISL_692419, EPI_ISL_692420, EPI_ISL_692421, EPI_ISL_692422, EPI_ISL_692423, EPI_ISL_692424, EPI_ISL_692425, EPI_ISL_692426, EPI_ISL_692427, EPI_ISL_692428, EPI_ISL_692429, EPI_ISL_692430, EPI_ISL_692431, EPI_ISL_692432, EPI_ISL_692433, EPI_ISL_692434, EPI_ISL_692435, EPI_ISL_692436, EPI_ISL_692437, EPI_ISL_692438, EPI_ISL_692439, EPI_ISL_692440, EPI_ISL_692441			
see above	Pathogen Genomics Center, National Institute of Infectious Diseases	Pathogen Genomics Center, National Institute of Infectious Diseases	Tsuyoshi Sekizuka, Kentaro Itokawa, Rina Tanaka, Masanori Hashino, Makoto Kuroda
EPI_ISL_692442, EPI_ISL_692443, EPI_ISL_692444, EPI_ISL_692445, EPI_ISL_692446, EPI_ISL_692447, EPI_ISL_692448, EPI_ISL_692449, EPI_ISL_692450, EPI_ISL_692451, EPI_ISL_692452, EPI_ISL_692453, EPI_ISL_692454, EPI_ISL_692455, EPI_ISL_692456, EPI_ISL_692457, EPI_ISL_692458, EPI_ISL_692459, EPI_ISL_692460, EPI_ISL_692461, EPI_ISL_692462, EPI_ISL_692463, EPI_ISL_692464, EPI_ISL_692465, EPI_ISL_692466, EPI_ISL_692467, EPI_ISL_692468, EPI_ISL_692469, EPI_ISL_692470, EPI_ISL_692471, EPI_ISL_692472, EPI_ISL_692473, EPI_ISL_692474, EPI_ISL_692475, EPI_ISL_692476, EPI_ISL_692477, EPI_ISL_692478, EPI_ISL_692479, EPI_ISL_692480, EPI_ISL_692481, EPI_ISL_692482, EPI_ISL_692483, EPI_ISL_692484, EPI_ISL_692485, EPI_ISL_692486, EPI_ISL_692487, EPI_ISL_692488, EPI_ISL_692489, EPI_ISL_692490, EPI_ISL_692491, EPI_ISL_692492, EPI_ISL_692493, EPI_ISL_692494, EPI_ISL_692495, EPI_ISL_692496, EPI_ISL_692497			
see above	Fukuoka Institute of Health and Environmental Sciences	Pathogen Genomics Center, National Institute of Infectious Diseases	Tsuyoshi Sekizuka, Kentaro Itokawa, Rina Tanaka, Masanori Hashino, Makoto Kuroda
EPI_ISL_692498, EPI_ISL_692499, EPI_ISL_692500, EPI_ISL_692501, EPI_ISL_692502, EPI_ISL_692503, EPI_ISL_692504, EPI_ISL_692505, EPI_ISL_692506, EPI_ISL_692507, EPI_ISL_692508, EPI_ISL_692509, EPI_ISL_692510, EPI_ISL_692511, EPI_ISL_692512, EPI_ISL_692513, EPI_ISL_692514, EPI_ISL_692515, EPI_ISL_692516, EPI_ISL_692517, EPI_ISL_692518, EPI_ISL_692519, EPI_ISL_692520, EPI_ISL_692521, EPI_ISL_692522, EPI_ISL_692523, EPI_ISL_692524, EPI_ISL_692525, EPI_ISL_692526, EPI_ISL_692528, EPI_ISL_692529, EPI_ISL_692530, EPI_ISL_692531, EPI_ISL_692532, EPI_ISL_692533, EPI_ISL_692534, EPI_ISL_692535, EPI_ISL_692536, EPI_ISL_692537, EPI_ISL_692538, EPI_ISL_692539, EPI_ISL_692540, EPI_ISL_692541, EPI_ISL_692542, EPI_ISL_692543, EPI_ISL_692544, EPI_ISL_692545, EPI_ISL_692546, EPI_ISL_692547, EPI_ISL_692548, EPI_ISL_692549, EPI_ISL_692550, EPI_ISL_692551, EPI_ISL_692552, EPI_ISL_692553, EPI_ISL_692554, EPI_ISL_692555, EPI_ISL_692556, EPI_ISL_692557, EPI_ISL_692558, EPI_ISL_692559, EPI_ISL_692560, EPI_ISL_692561, EPI_ISL_692562, EPI_ISL_692563, EPI_ISL_692564, EPI_ISL_692565, EPI_ISL_692566, EPI_ISL_692567, EPI_ISL_692568, EPI_ISL_692569, EPI_ISL_692570, EPI_ISL_692571, EPI_ISL_692572, EPI_ISL_692573, EPI_ISL_692574, EPI_ISL_692575, EPI_ISL_692576, EPI_ISL_692577, EPI_ISL_692578, EPI_ISL_692579, EPI_ISL_692580, EPI_ISL_692581, EPI_ISL_692582, EPI_ISL_692583, EPI_ISL_692584, EPI_ISL_692585, EPI_ISL_692586, EPI_ISL_692587, EPI_ISL_692588, EPI_ISL_692589, EPI_ISL_692590, EPI_ISL_692591, EPI_ISL_692592, EPI_ISL_692593, EPI_ISL_692594, EPI_ISL_692595, EPI_ISL_692596, EPI_ISL_692598, EPI_ISL_692599, EPI_ISL_692600, EPI_ISL_692601, EPI_ISL_692602, EPI_ISL_692603, EPI_ISL_692604, EPI_ISL_692605, EPI_ISL_692606, EPI_ISL_692607, EPI_ISL_692608, EPI_ISL_692609, EPI_ISL_692610, EPI_ISL_692611, EPI_ISL_692612, EPI_ISL_692613, EPI_ISL_692614, EPI_ISL_692615, EPI_ISL_692616, EPI_ISL_692617, EPI_ISL_692618, EPI_ISL_692619, EPI_ISL_692620, EPI_ISL_692621, EPI_ISL_692622, EPI_ISL_692623, EPI_ISL_692624, EPI_ISL_692625, EPI_ISL_692626, EPI_ISL_692627, EPI_ISL_692628, EPI_ISL_692629, EPI_ISL_692630, EPI_ISL_692631, EPI_ISL_692632, EPI_ISL_692633, EPI_ISL_692634, EPI_ISL_692635, EPI_ISL_692636, EPI_ISL_692637, EPI_ISL_692638, EPI_ISL_692639, EPI_ISL_692640, EPI_ISL_692641, EPI_ISL_692642, EPI_ISL_692643, EPI_ISL_692644, EPI_ISL_692645, EPI_ISL_692646, EPI_ISL_692647, EPI_ISL_692648, EPI_ISL_692649, EPI_ISL_692650, EPI_ISL_692651, EPI_ISL_692652			
see above	Pathogen Genomics Center, National Institute of Infectious Diseases	Pathogen Genomics Center, National Institute of Infectious Diseases	Tsuyoshi Sekizuka, Kentaro Itokawa, Rina Tanaka, Masanori Hashino, Makoto Kuroda
EPI_ISL_692653, EPI_ISL_692654, EPI_ISL_692655, EPI_ISL_692656, EPI_ISL_692657, EPI_ISL_692658, EPI_ISL_692659, EPI_ISL_692660, EPI_ISL_692661, EPI_ISL_692662, EPI_ISL_692663, EPI_ISL_692664, EPI_ISL_692665, EPI_ISL_692666, EPI_ISL_692667, EPI_ISL_692668, EPI_ISL_692669, EPI_ISL_692670, EPI_ISL_692671, EPI_ISL_692672, EPI_ISL_692673, EPI_ISL_692674, EPI_ISL_692675, EPI_ISL_692676, EPI_ISL_692677, EPI_ISL_692678, EPI_ISL_692679			
see above	Sakai City Institute of Public Health	Pathogen Genomics Center, National Institute of Infectious Diseases	Tsuyoshi Sekizuka, Kentaro Itokawa, Rina Tanaka, Masanori Hashino, Makoto Kuroda
EPI_ISL_692680, EPI_ISL_692681, EPI_ISL_692682, EPI_ISL_692683, EPI_ISL_692684, EPI_ISL_692685, EPI_ISL_692686, EPI_ISL_692687, EPI_ISL_692688, EPI_ISL_692689, EPI_ISL_692690, EPI_ISL_692691, EPI_ISL_692692, EPI_ISL_692693, EPI_ISL_692694, EPI_ISL_692695, EPI_ISL_692696, EPI_ISL_692697, EPI_ISL_692698, EPI_ISL_692699, EPI_ISL_692700, EPI_ISL_692701, EPI_ISL_692702, EPI_ISL_692703, EPI_ISL_692704, EPI_ISL_692705, EPI_ISL_692706, EPI_ISL_692707, EPI_ISL_692708, EPI_ISL_692709, EPI_ISL_692710, EPI_ISL_692711, EPI_ISL_692712, EPI_ISL_692713, EPI_ISL_692714, EPI_ISL_692715, EPI_ISL_692716, EPI_ISL_692717, EPI_ISL_692718, EPI_ISL_692719, EPI_ISL_692720, EPI_ISL_692721, EPI_ISL_692722, EPI_ISL_692723, EPI_ISL_692724, EPI_ISL_692725, EPI_ISL_692726, EPI_ISL_692727, EPI_ISL_692728, EPI_ISL_692729, EPI_ISL_692730, EPI_ISL_692731, EPI_ISL_692732			
see above	Pathogen Genomics Center, National Institute of Infectious Diseases	Pathogen Genomics Center, National Institute of Infectious Diseases	Tsuyoshi Sekizuka, Kentaro Itokawa, Rina Tanaka, Masanori Hashino, Makoto Kuroda
EPI_ISL_693199	Hospital do Servidor Publico Estadual Francisco Morato de Oliveira	Instituto Adolfo Lutz, Interdiciplinary Procedures Center, Strategic Laboratory	Claudio Tavares Sacchi, Claudia Regina Gonçalves, Erica Valessa Ramos Gomes, Karoline Rodrigues Campos
EPI_ISL_693204	Pronto Socorro Dr. Conrado Cesarino Nuvolini	Instituto Adolfo Lutz, Interdiciplinary Procedures Center, Strategic Laboratory	Claudio Tavares Sacchi, Claudia Regina Gonçalves, Erica Valessa Ramos Gomes, Karoline Rodrigues Campos
EPI_ISL_693205	Hospital de Campanha Covid-19 Assis	Instituto Adolfo Lutz, Interdiciplinary Procedures Center, Strategic Laboratory	Claudio Tavares Sacchi, Claudia Regina Gonçalves, Erica Valessa Ramos Gomes, Karoline Rodrigues Campos
EPI_ISL_693206	Hospital Municipal Mario Gatti	Instituto Adolfo Lutz, Interdiciplinary Procedures Center, Strategic Laboratory	Claudio Tavares Sacchi, Claudia Regina Gonçalves, Erica Valessa Ramos Gomes, Karoline Rodrigues Campos
EPI_ISL_693207	Cs II Doutor Antonio Vicoso Moreira de Rezende	Instituto Adolfo Lutz, Interdiciplinary Procedures Center, Strategic Laboratory	Claudio Tavares Sacchi, Claudia Regina Gonçalves, Erica Valessa Ramos Gomes, Karoline Rodrigues Campos
EPI_ISL_693208, EPI_ISL_693209	Hospital Municipal Antonio Giglio	Instituto Adolfo Lutz, Interdiciplinary Procedures Center, Strategic Laboratory	Claudio Tavares Sacchi, Claudia Regina Gonçalves, Erica Valessa Ramos Gomes, Karoline Rodrigues Campos
EPI_ISL_693210	Pronto-Socorro Dr. Osmar Mesquita	Instituto Adolfo Lutz, Interdiciplinary Procedures Center, Strategic Laboratory	Claudio Tavares Sacchi, Claudia Regina Gonçalves, Erica Valessa Ramos Gomes, Karoline Rodrigues Campos
EPI_ISL_693211	Santa Casa de Misericordia e Maternidade	Instituto Adolfo Lutz, Interdiciplinary Procedures Center, Strategic Laboratory	Claudio Tavares Sacchi, Claudia Regina Gonçalves, Erica Valessa Ramos Gomes, Karoline Rodrigues Campos
EPI_ISL_693212	Santa Casa de Misericordia de Braganca Paulista	Instituto Adolfo Lutz, Interdiciplinary Procedures Center, Strategic Laboratory	Claudio Tavares Sacchi, Claudia Regina Gonçalves, Erica Valessa Ramos Gomes, Karoline Rodrigues Campos
EPI_ISL_693213	Hospital E Maternidade Municipal Governador Mario Covas	Instituto Adolfo Lutz, Interdiciplinary Procedures Center, Strategic Laboratory	Claudio Tavares Sacchi, Claudia Regina Gonçalves, Erica Valessa Ramos Gomes, Karoline Rodrigues Campos
EPI_ISL_693214	Unidade de Pronto Atendimento Central de Caraguatatuba	Instituto Adolfo Lutz, Interdiciplinary Procedures Center, Strategic Laboratory	Claudio Tavares Sacchi, Claudia Regina Gonçalves, Erica Valessa Ramos Gomes, Karoline Rodrigues Campos
EPI_ISL_693215	Secretaria Municipal de Saúde de Iracemapolis	Instituto Adolfo Lutz, Interdiciplinary Procedures Center, Strategic Laboratory	Claudio Tavares Sacchi, Claudia Regina Gonçalves, Erica Valessa Ramos Gomes, Karoline Rodrigues Campos
EPI_ISL_693216, EPI_ISL_693217	Unidade de Vigilância Epiudemiológica de Araras	Instituto Adolfo Lutz, Interdiciplinary Procedures Center, Strategic Laboratory	Claudio Tavares Sacchi, Claudia Regina Gonçalves, Erica Valessa Ramos Gomes, Karoline Rodrigues Campos
EPI_ISL_693218	Hospital Domingos Leonardo Ceravolo Presidente Prudente	Instituto Adolfo Lutz, Interdiciplinary Procedures Center, Strategic Laboratory	Claudio Tavares Sacchi, Claudia Regina Gonçalves, Erica Valessa Ramos Gomes, Karoline Rodrigues Campos
EPI_ISL_693219	Santa Casa da Misericórdia de Presidente Prudente	Instituto Adolfo Lutz, Interdiciplinary Procedures Center,	Claudio Tavares Sacchi, Claudia Regina Gonçalves, Erica Valessa Ramos Gomes, Karoline Rodrigues Campos

		Strategic Laboratory	
EPI_ISL_693220	Laboratório Municipal de Piracicaba	Instituto Adolfo Lutz, Interdiciplinary Procedures Center, Strategic Laboratory	Claudio Tavares Sacchi, Claudia Regina Gonçalves, Erica Valessa Ramos Gomes, Karoline Rodrigues Campos
EPI_ISL_693221, EPI_ISL_693222	Secretaria Municipal de Saúde de Birigui	Instituto Adolfo Lutz, Interdiciplinary Procedures Center, Strategic Laboratory	Claudio Tavares Sacchi, Claudia Regina Gonçalves, Erica Valessa Ramos Gomes, Karoline Rodrigues Campos
EPI_ISL_693223, EPI_ISL_693224	Laboratório Municipal de Piracicaba	Instituto Adolfo Lutz, Interdiciplinary Procedures Center, Strategic Laboratory	Claudio Tavares Sacchi, Claudia Regina Gonçalves, Erica Valessa Ramos Gomes, Karoline Rodrigues Campos
EPI_ISL_693225	Ubs Vila Rosa - Olímpia Gomes De Almeida	Instituto Adolfo Lutz, Interdiciplinary Procedures Center, Strategic Laboratory	Claudio Tavares Sacchi, Claudia Regina Gonçalves, Erica Valessa Ramos Gomes, Karoline Rodrigues Campos
EPI_ISL_693226	Unidade de Pronto Atendimento Sao José	Instituto Adolfo Lutz, Interdiciplinary Procedures Center, Strategic Laboratory	Claudio Tavares Sacchi, Claudia Regina Gonçalves, Erica Valessa Ramos Gomes, Karoline Rodrigues Campos
EPI_ISL_693227	UBS Vila Marchi	Instituto Adolfo Lutz, Interdiciplinary Procedures Center, Strategic Laboratory	Claudio Tavares Sacchi, Claudia Regina Gonçalves, Erica Valessa Ramos Gomes, Karoline Rodrigues Campos
EPI_ISL_693228	Secretaria Municipal de Sorocaba	Instituto Adolfo Lutz, Interdiciplinary Procedures Center, Strategic Laboratory	Claudio Tavares Sacchi, Claudia Regina Gonçalves, Erica Valessa Ramos Gomes, Karoline Rodrigues Campos
EPI_ISL_693229	Hospital 8 de Maio	Instituto Adolfo Lutz, Interdiciplinary Procedures Center, Strategic Laboratory	Claudio Tavares Sacchi, Claudia Regina Gonçalves, Erica Valessa Ramos Gomes, Karoline Rodrigues Campos
EPI_ISL_693230	Hospital e Pronto Socorro Portinari	Instituto Adolfo Lutz, Interdiciplinary Procedures Center, Strategic Laboratory	Claudio Tavares Sacchi, Claudia Regina Gonçalves, Erica Valessa Ramos Gomes, Karoline Rodrigues Campos
EPI_ISL_693231	Pronto Socorro Municipal de Santa Branca	Instituto Adolfo Lutz, Interdiciplinary Procedures Center, Strategic Laboratory	Claudio Tavares Sacchi, Claudia Regina Gonçalves, Erica Valessa Ramos Gomes, Karoline Rodrigues Campos
EPI_ISL_693232	Hospital e Pronto Socorro Portinari	Instituto Adolfo Lutz, Interdiciplinary Procedures Center, Strategic Laboratory	Claudio Tavares Sacchi, Claudia Regina Gonçalves, Erica Valessa Ramos Gomes, Karoline Rodrigues Campos
EPI_ISL_693233	Hospital Santa Cruz	Instituto Adolfo Lutz, Interdiciplinary Procedures Center, Strategic Laboratory	Claudio Tavares Sacchi, Claudia Regina Gonçalves, Erica Valessa Ramos Gomes, Karoline Rodrigues Campos
EPI_ISL_693234	Upa Vereador Jose Da Rocha Goncalves	Instituto Adolfo Lutz, Interdiciplinary Procedures Center, Strategic Laboratory	Claudio Tavares Sacchi, Claudia Regina Gonçalves, Erica Valessa Ramos Gomes, Karoline Rodrigues Campos
EPI_ISL_693235	Casmi Centro Atendimento Saude da Mulher e Infancia	Instituto Adolfo Lutz, Interdiciplinary Procedures Center, Strategic Laboratory	Claudio Tavares Sacchi, Claudia Regina Gonçalves, Erica Valessa Ramos Gomes, Karoline Rodrigues Campos
EPI_ISL_693236	Hospital Santa Marcelina Sao Paulo	Instituto Adolfo Lutz, Interdiciplinary Procedures Center, Strategic Laboratory	Claudio Tavares Sacchi, Claudia Regina Gonçalves, Erica Valessa Ramos Gomes, Karoline Rodrigues Campos
EPI_ISL_693237	UPA Santa Isabel	Instituto Adolfo Lutz, Interdiciplinary Procedures Center, Strategic Laboratory	Claudio Tavares Sacchi, Claudia Regina Gonçalves, Erica Valessa Ramos Gomes, Karoline Rodrigues Campos
EPI_ISL_693238, EPI_ISL_693239	Secao Centro de Diagnostico Secedi	Instituto Adolfo Lutz, Interdiciplinary Procedures Center, Strategic Laboratory	Claudio Tavares Sacchi, Claudia Regina Gonçalves, Erica Valessa Ramos Gomes, Karoline Rodrigues Campos
EPI_ISL_693240	Centro de Vigilância a Saude de Diadema	Instituto Adolfo Lutz, Interdiciplinary Procedures Center, Strategic Laboratory	Claudio Tavares Sacchi, Claudia Regina Gonçalves, Erica Valessa Ramos Gomes, Karoline Rodrigues Campos
EPI_ISL_693241	Hospital e Maternidade Sao Lucas	Instituto Adolfo Lutz, Interdiciplinary Procedures Center, Strategic Laboratory	Claudio Tavares Sacchi, Claudia Regina Gonçalves, Erica Valessa Ramos Gomes, Karoline Rodrigues Campos
EPI_ISL_693242	Centro de Vigilância a Saude de Diadema	Instituto Adolfo Lutz, Interdiciplinary Procedures Center, Strategic Laboratory	Claudio Tavares Sacchi, Claudia Regina Gonçalves, Erica Valessa Ramos Gomes, Karoline Rodrigues Campos
EPI_ISL_693243	Laboratório Municipal de Piracicaba	Instituto Adolfo Lutz, Interdiciplinary Procedures Center, Strategic Laboratory	Claudio Tavares Sacchi, Claudia Regina Gonçalves, Erica Valessa Ramos Gomes, Karoline Rodrigues Campos
EPI_ISL_693244	Centro Médico da Polícia Militar do Estado de Sao Paulo	Instituto Adolfo Lutz, Interdiciplinary Procedures Center, Strategic Laboratory	Claudio Tavares Sacchi, Claudia Regina Gonçalves, Erica Valessa Ramos Gomes, Karoline Rodrigues Campos
EPI_ISL_693245	UPA Santa Isabel	Instituto Adolfo Lutz, Interdiciplinary Procedures Center, Strategic Laboratory	Claudio Tavares Sacchi, Claudia Regina Gonçalves, Erica Valessa Ramos Gomes, Karoline Rodrigues Campos
EPI_ISL_693246	Laboratorio Municipal de Rio Grande da Serra	Instituto Adolfo Lutz, Interdiciplinary Procedures Center, Strategic Laboratory	Claudio Tavares Sacchi, Claudia Regina Gonçalves, Erica Valessa Ramos Gomes, Karoline Rodrigues Campos
EPI_ISL_693247	Secao Centro de Diagnostico Secedi	Instituto Adolfo Lutz, Interdiciplinary Procedures Center, Strategic Laboratory	Claudio Tavares Sacchi, Claudia Regina Gonçalves, Erica Valessa Ramos Gomes, Karoline Rodrigues Campos
EPI_ISL_693248	Centro Municipal de Epidemiologia e Imunizações	Instituto Adolfo Lutz, Interdiciplinary Procedures Center, Strategic Laboratory	Claudio Tavares Sacchi, Claudia Regina Gonçalves, Erica Valessa Ramos Gomes, Karoline Rodrigues Campos
EPI_ISL_699716, EPI_ISL_699717, EPI_ISL_699718, EPI_ISL_699719, EPI_ISL_699720, EPI_ISL_699721, EPI_ISL_699724, EPI_ISL_699725, EPI_ISL_699726, EPI_ISL_699727, EPI_ISL_699728, EPI_ISL_699729, EPI_ISL_699730, EPI_ISL_699731, EPI_ISL_699732, EPI_ISL_699733, EPI_ISL_699735, EPI_ISL_699736, EPI_ISL_699737, EPI_ISL_699738, EPI_ISL_699739, EPI_ISL_699740, EPI_ISL_699741, EPI_ISL_699742, EPI_ISL_699743, EPI_ISL_699744, EPI_ISL_699745, EPI_ISL_699746, EPI_ISL_699747, EPI_ISL_699748, EPI_ISL_699749, EPI_ISL_699750, EPI_ISL_699751, EPI_ISL_699754, EPI_ISL_699759, EPI_ISL_699760, EPI_ISL_699761, EPI_ISL_699763, EPI_ISL_699764, EPI_ISL_699765, EPI_ISL_699766, EPI_ISL_699769, EPI_ISL_699770, EPI_ISL_699771, EPI_ISL_699772, EPI_ISL_699773, EPI_ISL_699774, EPI_ISL_699775, EPI_ISL_699776, EPI_ISL_699777, EPI_ISL_699778, EPI_ISL_699779, EPI_ISL_699780, EPI_ISL_699782, EPI_ISL_699783, EPI_ISL_699785, EPI_ISL_699786, EPI_ISL_699787, EPI_ISL_699788, EPI_ISL_699790, EPI_ISL_699791, EPI_ISL_699792, EPI_ISL_699793, EPI_ISL_699794, EPI_ISL_699795, EPI_ISL_699796, EPI_ISL_699797, EPI_ISL_699798, EPI_ISL_699799, EPI_ISL_699802, EPI_ISL_699803, EPI_ISL_699804, EPI_ISL_699805, EPI_ISL_699806, EPI_ISL_699807, EPI_ISL_699808, EPI_ISL_699809, EPI_ISL_699811, EPI_ISL_699813, EPI_ISL_699815, EPI_ISL_699816, EPI_ISL_699818, EPI_ISL_699820, EPI_ISL_699821, EPI_ISL_699822, EPI_ISL_699823, EPI_ISL_699826, EPI_ISL_699828, EPI_ISL_699829, EPI_ISL_699830, EPI_ISL_699832, EPI_ISL_699833, EPI_ISL_699834, EPI_ISL_699835, EPI_ISL_699838, EPI_ISL_699839, EPI_ISL_699840, EPI_ISL_699841, EPI_ISL_699843, EPI_ISL_699844, EPI_ISL_699845, EPI_ISL_699846, EPI_ISL_699847, EPI_ISL_699851, EPI_ISL_699852, EPI_ISL_699853, EPI_ISL_699854, EPI_ISL_699855, EPI_ISL_699856, EPI_ISL_699857, EPI_ISL_699858, EPI_ISL_699859, EPI_ISL_699860, EPI_ISL_699861, EPI_ISL_699862, EPI_ISL_699863, EPI_ISL_699864, EPI_ISL_699865, EPI_ISL_699866, EPI_ISL_699867, EPI_ISL_699868, EPI_ISL_699869, EPI_ISL_699870, EPI_ISL_699871, EPI_ISL_699872, EPI_ISL_699873, EPI_ISL_699874, EPI_ISL_699878, EPI_ISL_699879, EPI_ISL_699880, EPI_ISL_699882, EPI_ISL_699883, EPI_ISL_699884, EPI_ISL_699886, EPI_ISL_699887, EPI_ISL_699888, EPI_ISL_699889, EPI_ISL_699890, EPI_ISL_699891, EPI_ISL_699893, EPI_ISL_699894, EPI_ISL_699897, EPI_ISL_699898, EPI_ISL_699899, EPI_ISL_699902, EPI_ISL_699904, EPI_ISL_699906, EPI_ISL_699907, EPI_ISL_699909, EPI_ISL_699910, EPI_ISL_699911, EPI_ISL_699912, EPI_ISL_699914, EPI_ISL_699915, EPI_ISL_699916, EPI_ISL_699917, EPI_ISL_699918, EPI_ISL_699919, EPI_ISL_699920, EPI_ISL_699921, EPI_ISL_699922, EPI_ISL_699923, EPI_ISL_699924, EPI_ISL_699925, EPI_ISL_699926, EPI_ISL_699927, EPI_ISL_699928, EPI_ISL_699930, EPI_ISL_699931, EPI_ISL_699932, EPI_ISL_699933, EPI_ISL_699934, EPI_ISL_699935, EPI_ISL_699936, EPI_ISL_699937, EPI_ISL_699938, EPI_ISL_699939, EPI_ISL_699940, EPI_ISL_699941, EPI_ISL_699942, EPI_ISL_699943, EPI_ISL_699944, EPI_ISL_699945, EPI_ISL_699946, EPI_ISL_699947, EPI_ISL_699948, EPI_ISL_699949, EPI_ISL_699950, EPI_ISL_699951, EPI_ISL_699952, EPI_ISL_699953, EPI_ISL_699954, EPI_ISL_699955, EPI_ISL_699956, EPI_ISL_699957, EPI_ISL_699958, EPI_ISL_699961, EPI_ISL_699962, EPI_ISL_699963, EPI_ISL_699964, EPI_ISL_699966, EPI_ISL_699967, EPI_ISL_699968, EPI_ISL_699970, EPI_ISL_699971, EPI_ISL_699972, EPI_ISL_699973, EPI_ISL_699974, EPI_ISL_699975, EPI_ISL_699979, EPI_ISL_699980, EPI_ISL_699981, EPI_ISL_699983, EPI_ISL_699984, EPI_ISL_699985, EPI_ISL_699986, EPI_ISL_699987, EPI_ISL_699988, EPI_ISL_699989, EPI_ISL_699990, EPI_ISL_699991, EPI_ISL_699993, EPI_ISL_699994, EPI_ISL_699996, EPI_ISL_699997, EPI_ISL_699998, EPI_ISL_699999, EPI_ISL_700000, EPI_ISL_700002, EPI_ISL_700003, EPI_ISL_700004, EPI_ISL_700005, EPI_ISL_700006, EPI_ISL_700009, EPI_ISL_700010, EPI_ISL_700011, EPI_ISL_700012, EPI_ISL_700013, EPI_ISL_700016, EPI_ISL_700017, EPI_ISL_700018, EPI_ISL_700020, EPI_ISL_700021, EPI_ISL_700024, EPI_ISL_700025, EPI_ISL_700027, EPI_ISL_700028, EPI_ISL_700030, EPI_ISL_700031, EPI_ISL_700033, EPI_ISL_700034, EPI_ISL_700036, EPI_ISL_700037, EPI_ISL_700038, EPI_ISL_700039, EPI_ISL_700040, EPI_ISL_700041, EPI_ISL_700043, EPI_ISL_700044, EPI_ISL_700046, EPI_ISL_700047, EPI_ISL_700048, EPI_ISL_700049, EPI_ISL_700050, EPI_ISL_700051, EPI_ISL_700052, EPI_ISL_700053, EPI_ISL_700054, EPI_ISL_700055, EPI_ISL_700056, EPI_ISL_700057, EPI_ISL_700058, EPI_ISL_700059, EPI_ISL_700061, EPI_ISL_700064, EPI_ISL_700065, EPI_ISL_700066, EPI_ISL_700067, EPI_ISL_700068, EPI_ISL_700069, EPI_ISL_700070, EPI_ISL_700071, EPI_ISL_700072, EPI_ISL_700073, EPI_ISL_700074, EPI_ISL_700076, EPI_ISL_700077, EPI_ISL_700078, EPI_ISL_700079, EPI_ISL_700080, EPI_ISL_700081, EPI_ISL_700082, EPI_ISL_700083, EPI_ISL_700084, EPI_ISL_700085, EPI_ISL_700086, EPI_ISL_700088, EPI_ISL_700089, EPI_ISL_700091, EPI_ISL_700092, EPI_ISL_700094, EPI_ISL_700096, EPI_ISL_700097, EPI_ISL_700098, EPI_ISL_700099, EPI_ISL_700100, EPI_ISL_700101, EPI_ISL_700102, EPI_ISL_700103, EPI_ISL_700104, EPI_ISL_700107, EPI_ISL_700108, EPI_ISL_700109, EPI_ISL_700110, EPI_ISL_700111, EPI_ISL_700112, EPI_ISL_700113, EPI_ISL_700114, EPI_ISL_700115, EPI_ISL_700116, EPI_ISL_700118, EPI_ISL_700120, EPI_ISL_700123, EPI_ISL_700124, EPI_ISL_700125, EPI_ISL_700127, EPI_ISL_700128, EPI_ISL_700129, EPI_ISL_700131, EPI_ISL_700132, EPI_ISL_700136, EPI_ISL_700138, EPI_ISL_700140, EPI_ISL_700143, EPI_ISL_700144, EPI_ISL_700145, EPI_ISL_700146, EPI_ISL_700147, EPI_ISL_700149, EPI_ISL_700150, EPI_ISL_700151, EPI_ISL_700154,			

EPI_ISL_700155, EPI_ISL_700156, EPI_ISL_700157, EPI_ISL_700158, EPI_ISL_700160, EPI_ISL_700161, EPI_ISL_700162, EPI_ISL_700164, EPI_ISL_700166, EPI_ISL_700167, EPI_ISL_700168, EPI_ISL_700169, EPI_ISL_700172, EPI_ISL_700176, EPI_ISL_700177, EPI_ISL_700179, EPI_ISL_700180, EPI_ISL_700181, EPI_ISL_700183, EPI_ISL_700184, EPI_ISL_700185, EPI_ISL_700187, EPI_ISL_700188, EPI_ISL_700189, EPI_ISL_700190, EPI_ISL_700191, EPI_ISL_700192, EPI_ISL_700193, EPI_ISL_700194, EPI_ISL_700195, EPI_ISL_700196, EPI_ISL_700197, EPI_ISL_700198, EPI_ISL_700199, EPI_ISL_700200, EPI_ISL_700201, EPI_ISL_700202, EPI_ISL_700203, EPI_ISL_700204, EPI_ISL_700205, EPI_ISL_700206, EPI_ISL_700208, EPI_ISL_700209, EPI_ISL_700210, EPI_ISL_700211, EPI_ISL_700212, EPI_ISL_700214, EPI_ISL_700215, EPI_ISL_700216, EPI_ISL_700217			
see above	Hematopathology Laboratory, ACTREC, TMC	Hematopathology Laboratory, ACTREC, TMC	Hematopathology Laboratory, ACTREC
EPI_ISL_707928	Fujita Health University Hospital	Fujita Health University School of Medicine, Department of Microbiology	Masahiro Suzuki, Aki Sakurai, Yohei Doi
EPI_ISL_707936, EPI_ISL_707937, EPI_ISL_707938, EPI_ISL_707939, EPI_ISL_708184, EPI_ISL_708185, EPI_ISL_708186, EPI_ISL_708188, EPI_ISL_708189, EPI_ISL_708190, EPI_ISL_708191, EPI_ISL_708192, EPI_ISL_708193, EPI_ISL_708194, EPI_ISL_708195			
see above	Pamukkale University Hospital	Pamukkale University Department of Medical Genetics	Onur TOKGUN et al.
EPI_ISL_708529	Secretária Municipal de Saude de Fernandópolis	Instituto Adolfo Lutz, Interdiciplinary Procedures Center, Strategic Laboratory	Claudio Tavares Sacchi, Claudia Regina Gonçalves, Erica Valessa Ramos Gomes, Carlos Henrique Camargo, Karoline Rodrigues Campos, Fernanda Modesto Tolentino Binhardi, Maricelia Navarro Pinheiro Flores, Marcia Maria Costa Nunes Soares, Janaina Other Martins Montanha
EPI_ISL_708839, EPI_ISL_708840	National Institute of Blood Diseases (NIBD), Molecular Biology Lab	Genomics Lab NIBD	Samina Naz Mukry, Sayed Ali Raza, Shariq Ahmed, Aneeta Shahni, Gul Sufaida, Arshi Naz , Tahir Sultan Shamsi
EPI_ISL_718134, EPI_ISL_718135, EPI_ISL_718143, EPI_ISL_718146, EPI_ISL_718156	Ministry of Health Hospitals	Institute of Health and Community Medicine	David Perera, Ooi Mong How, Chua Hock Hin, Tonnii Sia Loong Loong, Wong Jyn Shan, Wong Kiing Aik, Chan Chia Jui
EPI_ISL_718267, EPI_ISL_718268, EPI_ISL_718271, EPI_ISL_718272, EPI_ISL_718274, EPI_ISL_718275, EPI_ISL_718277, EPI_ISL_718279	Institute for Medical Research, Infectious Disease Research Centre, National Institutes of Health, Ministry of Health Malaysia	Institute for Medical Research, Infectious Disease Research Centre, National Institutes of Health, Ministry of Health Malaysia	Suppiah J, Kamel K, Mohd-Zawawi Z, Thayan R
EPI_ISL_722851	I.R.C.C.S. "S. De Bellis" - Ente Ospedaliero	Istituto Zooprofilattico Sperimentale della Puglia e della Basilicata	Parisi A., Bianco A., Capozzi L., Del Sambro L., Lippolis A., Notarnicola M., Manzulli V, Rondinone V., Pace L.
EPI_ISL_722852	I.R.C.C.S. "S. De Bellis" - Ente Ospedaliero	Istituto Zooprofilattico Sperimentale della Puglia e della Basilicata	Parisi A., Bianco A., Capozzi L., Del Sambro L., Lippolis A., Notarnicola M., Cipolletta D., Galante D.
EPI_ISL_722853	I.R.C.C.S. "S. De Bellis" - Ente Ospedaliero	Istituto Zooprofilattico Sperimentale della Puglia e della Basilicata	Parisi A., Bianco A., Capozzi L., Del Sambro L., Lippolis A., Notarnicola M., Manzulli V, Rondinone V., Pace L.
EPI_ISL_722854	I.R.C.C.S. "S. De Bellis" - Ente Ospedaliero	Istituto Zooprofilattico Sperimentale della Puglia e della Basilicata	Parisi A., Bianco A., Capozzi L., Del Sambro L., Lippolis A., Notarnicola M., Cipolletta D., Galante D.
EPI_ISL_722855, EPI_ISL_722856, EPI_ISL_722857, EPI_ISL_722858	Dipartimento di Scienze Biomediche e Oncologia Umana - Azienda Ospedaliero Universitaria Consorziale Policlinico	Istituto Zooprofilattico Sperimentale della Puglia e della Basilicata	Parisi A., Bianco A., Capozzi L., Del Sambro L., Chironna M., Loconsole D.
EPI_ISL_722859, EPI_ISL_722860	I.R.C.C.S. "S. De Bellis" - Ente Ospedaliero	Istituto Zooprofilattico Sperimentale della Puglia e della Basilicata	Parisi A., Bianco A., Capozzi L., Del Sambro L., Lippolis A., Notarnicola M., Manzulli V, Rondinone V., Pace L.
EPI_ISL_722861	I.R.C.C.S. "S. De Bellis" - Ente Ospedaliero	Istituto Zooprofilattico Sperimentale della Puglia e della Basilicata	Parisi A., Bianco A., Capozzi L., Del Sambro L., Lippolis A., Notarnicola M., Cipolletta D., Galante D.
EPI_ISL_722862	I.R.C.C.S. "S. De Bellis" - Ente Ospedaliero	Istituto Zooprofilattico Sperimentale della Puglia e della Basilicata	Parisi A., Bianco A., Capozzi L., Del Sambro L., Lippolis A., Notarnicola M., Manzulli V, Rondinone V., Pace L.
EPI_ISL_722863	I.R.C.C.S. "S. De Bellis" - Ente Ospedaliero	Istituto Zooprofilattico Sperimentale della Puglia e della Basilicata	Parisi A., Bianco A., Capozzi L., Del Sambro L., Lippolis A., Notarnicola M., Cipolletta D., Galante D.
EPI_ISL_722864, EPI_ISL_722865	I.R.C.C.S. "S. De Bellis" - Ente Ospedaliero	Istituto Zooprofilattico Sperimentale della Puglia e della Basilicata	Parisi A., Bianco A., Capozzi L., Del Sambro L., Lippolis A., Notarnicola M., Manzulli V, Rondinone V., Pace L.
EPI_ISL_722866	I.R.C.C.S. "S. De Bellis" - Ente Ospedaliero	Istituto Zooprofilattico Sperimentale della Puglia e della Basilicata	Parisi A., Bianco A., Capozzi L., Del Sambro L., Lippolis A., Notarnicola M., Cipolletta D., Galante D.
EPI_ISL_722867, EPI_ISL_722868	I.R.C.C.S. "S. De Bellis" - Ente Ospedaliero	Istituto Zooprofilattico Sperimentale della Puglia e della Basilicata	Parisi A., Bianco A., Capozzi L., Del Sambro L., Lippolis A., Notarnicola M., Manzulli V, Rondinone V., Pace L.
EPI_ISL_722869	I.R.C.C.S. "S. De Bellis" - Ente Ospedaliero	Istituto Zooprofilattico Sperimentale della Puglia e della Basilicata	Parisi A., Bianco A., Capozzi L., Del Sambro L., Lippolis A., Notarnicola M., Cipolletta D., Galante D.
EPI_ISL_722870	I.R.C.C.S. "S. De Bellis" - Ente Ospedaliero	Istituto Zooprofilattico Sperimentale della Puglia e della Basilicata	Parisi A., Bianco A., Capozzi L., Del Sambro L., Lippolis A., Notarnicola M., Manzulli V, Rondinone V., Pace L.
EPI_ISL_722871	I.R.C.C.S. "S. De Bellis" - Ente Ospedaliero	Istituto Zooprofilattico Sperimentale della Puglia e della Basilicata	Parisi A., Bianco A., Capozzi L., Del Sambro L., Lippolis A., Notarnicola M., Cipolletta D., Galante D.
EPI_ISL_722872	Dipartimento di Scienze Biomediche e Oncologia Umana - Azienda Ospedaliero Universitaria Consorziale Policlinico	Istituto Zooprofilattico Sperimentale della Puglia e della Basilicata	Parisi A., Bianco A., Capozzi L., Del Sambro L., Chironna M., Loconsole D.
EPI_ISL_722892	Istituto Zooprofilattico Sperimentale della Puglia e della Basilicata	Istituto Zooprofilattico Sperimentale della Puglia e della Basilicata	Parisi A., Bianco A., Capozzi L., Del Sambro L., Manzulli V, Rondinone V., Pace L., Cipolletta D., Galante D.
EPI_ISL_722895	I.R.C.C.S. "S. De Bellis" - Ente Ospedaliero	Istituto Zooprofilattico Sperimentale della Puglia e della Basilicata	Parisi A., Bianco A., Capozzi L., Del Sambro L., Lippolis A., Notarnicola M., Cipolletta D., Galante D.
EPI_ISL_722897	Dipartimento di Scienze Biomediche e Oncologia Umana - Azienda Ospedaliero Universitaria Consorziale Policlinico	Istituto Zooprofilattico Sperimentale della Puglia e della Basilicata	Parisi A., Bianco A., Capozzi L., Del Sambro L., Chironna M., Loconsole D.
EPI_ISL_722898, EPI_ISL_722900	I.R.C.C.S. "S. De Bellis" - Ente Ospedaliero	Istituto Zooprofilattico Sperimentale della Puglia e della Basilicata	Parisi A., Bianco A., Capozzi L., Del Sambro L., Lippolis A., Notarnicola M., Cipolletta D., Galante D.
EPI_ISL_729800, EPI_ISL_729848, EPI_ISL_729849	Laboratório Central de Saúde Pública do Estado do Rio Grande do Sul (LACEN-RS)	Laboratory of Respiratory Viruses and Measles, Oswaldo Cruz Institute, FIOCRUZ	Paola Resende, Luciana Appolinario, Fernando Motta, Anna Carolina Paixão, Ana Carolina Mendonça, Tatiana Schaffer Gregianini, Marilda Tereza Mar da Rosa, Marilda Siqueira on behalf of the Fiocruz COVID-19 Genomic Surveillance Network
EPI_ISL_730578	Home Quarantine Taskforce	Hong Kong Department of Health	Mak Gannon C.K., Lam Edman T.K., Chan Rickjason C.W., Tsang Dominic N.C.
EPI_ISL_730601	Temporary Specimen Collection Centre at the AsiaWorld-Expo	Hong Kong Department of Health	Mak Gannon C.K., Lam Edman T.K., Chan Rickjason C.W., Tsang Dominic N.C.
EPI_ISL_732531, EPI_ISL_732532, EPI_ISL_732533, EPI_ISL_732534, EPI_ISL_732535, EPI_ISL_732536, EPI_ISL_732537, EPI_ISL_732538, EPI_ISL_732539, EPI_ISL_732540, EPI_ISL_732559, EPI_ISL_732560			
see above	Bundeswehr Institute of Microbiology	Bundeswehr Institute of Microbiology	Elham Khatamzas, Markus Antwerpen, Mathias Walter, Alexandra Rehn, Sabine Zange, Enrico Georgi, Michael von Bergwelt-Baildon, Roman Wölfel
EPI_ISL_735306, EPI_ISL_735307, EPI_ISL_735308, EPI_ISL_735309, EPI_ISL_735310, EPI_ISL_735311, EPI_ISL_735312, EPI_ISL_735313, EPI_ISL_735314, EPI_ISL_735315, EPI_ISL_735316, EPI_ISL_735317, EPI_ISL_735318, EPI_ISL_735319, EPI_ISL_735320, EPI_ISL_735321, EPI_ISL_735322, EPI_ISL_735323, EPI_ISL_735324, EPI_ISL_735325, EPI_ISL_735326, EPI_ISL_735327, EPI_ISL_735328, EPI_ISL_735329, EPI_ISL_735330, EPI_ISL_735331, EPI_ISL_735332, EPI_ISL_735333, EPI_ISL_735334, EPI_ISL_735335, EPI_ISL_735336, EPI_ISL_735337, EPI_ISL_735338, EPI_ISL_735339, EPI_ISL_735340, EPI_ISL_735342, EPI_ISL_735343, EPI_ISL_735344, EPI_ISL_735345, EPI_ISL_735346, EPI_ISL_735347, EPI_ISL_735348, EPI_ISL_735349, EPI_ISL_735350, EPI_ISL_735351, EPI_ISL_735352, EPI_ISL_735353, EPI_ISL_735354, EPI_ISL_735355, EPI_ISL_735356, EPI_ISL_735357, EPI_ISL_735358, EPI_ISL_735359, EPI_ISL_735360, EPI_ISL_735361, EPI_ISL_735362, EPI_ISL_735363, EPI_ISL_735364, EPI_ISL_735365			

see above	Genomic Laboratory (GLAB) (Conjoint lab of Health Directorate of Istanbul and Istanbul Technical University)	Genomic Laboratory (GLAB), Istanbul Technical University	Ilker Karacan, Tugba Kizilboga Akgun, Nihat Bugra Agaoglu, Payam Zolfagharian, Mehtap Aydin, Gizem Alkurt, Jale Yildiz, Betsi Kose, Nisan Denizce Can, Ayse Serra Ozel, Nilsun Altunal, Arzu Irvem, Yasemin Kendir Demirkol, Ozlem Akgun Dogan, Levent Doganay, Gizem Dinler Doganay
EPI_ISL_735396	Hospital de Camplanha COVID 19 SER	Instituto Adolfo Lutz, Interdiciplinary Procedures Center, Strategic Laboratory	Claudio Tavares Sacchi, Claudia Regina Gonçalves, Erica Valessa Ramos Gomes, Karoline Rodrigues Campos
EPI_ISL_735397	Unidade Respiratória Nova Hortolandia	Instituto Adolfo Lutz, Interdiciplinary Procedures Center, Strategic Laboratory	Claudio Tavares Sacchi, Claudia Regina Gonçalves, Erica Valessa Ramos Gomes, Karoline Rodrigues Campos
EPI_ISL_735398	Laboratorio Fleury	Instituto Adolfo Lutz, Interdiciplinary Procedures Center, Strategic Laboratory	Claudio Tavares Sacchi, Claudia Regina Gonçalves, Erica Valessa Ramos Gomes, Karoline Rodrigues Campos
EPI_ISL_735400	Instituto Adolfo Lutz - Regional de Santos	Instituto Adolfo Lutz, Interdiciplinary Procedures Center, Strategic Laboratory	Claudio Tavares Sacchi, Claudia Regina Gonçalves, Erica Valessa Ramos Gomes, Karoline Rodrigues Campos
EPI_ISL_735401, EPI_ISL_735402, EPI_ISL_735403, EPI_ISL_735404	Instituto Adolfo Lutz - Regional de Rio Claro	Instituto Adolfo Lutz, Interdiciplinary Procedures Center, Strategic Laboratory	Claudio Tavares Sacchi, Claudia Regina Gonçalves, Erica Valessa Ramos Gomes, Karoline Rodrigues Campos
EPI_ISL_735405	Secretaria Minucipal de Saude de Birigui	Instituto Adolfo Lutz, Interdiciplinary Procedures Center, Strategic Laboratory	Claudio Tavares Sacchi, Claudia Regina Gonçalves, Erica Valessa Ramos Gomes, Karoline Rodrigues Campos
EPI_ISL_735406	Unidade de Pronto Atendimento UPA I Sta Isabel	Instituto Adolfo Lutz, Interdiciplinary Procedures Center, Strategic Laboratory	Claudio Tavares Sacchi, Claudia Regina Gonçalves, Erica Valessa Ramos Gomes, Karoline Rodrigues Campos
EPI_ISL_735407	Santa Casa de Marilia	Instituto Adolfo Lutz, Interdiciplinary Procedures Center, Strategic Laboratory	Claudio Tavares Sacchi, Claudia Regina Gonçalves, Erica Valessa Ramos Gomes, Karoline Rodrigues Campos
EPI_ISL_735408	COVID 19 Centro de Combate ao Coronavirus CCC Jandira	Instituto Adolfo Lutz, Interdiciplinary Procedures Center, Strategic Laboratory	Claudio Tavares Sacchi, Claudia Regina Gonçalves, Erica Valessa Ramos Gomes, Karoline Rodrigues Campos
EPI_ISL_735409	Unidade de Pronto Atendimento Carlos Lourenco	Instituto Adolfo Lutz, Interdiciplinary Procedures Center, Strategic Laboratory	Claudio Tavares Sacchi, Claudia Regina Gonçalves, Erica Valessa Ramos Gomes, Karoline Rodrigues Campos
EPI_ISL_735410	Instituto Adolfo Lutz - Regional de Rio Claro	Instituto Adolfo Lutz, Interdiciplinary Procedures Center, Strategic Laboratory	Claudio Tavares Sacchi, Claudia Regina Gonçalves, Erica Valessa Ramos Gomes, Karoline Rodrigues Campos
EPI_ISL_735411	Centro de Vigilancia a Saude de Diadema	Instituto Adolfo Lutz, Interdiciplinary Procedures Center, Strategic Laboratory	Claudio Tavares Sacchi, Claudia Regina Gonçalves, Erica Valessa Ramos Gomes, Karoline Rodrigues Campos
EPI_ISL_735412	Hospital e Pronto Socorro Portinari	Instituto Adolfo Lutz, Interdiciplinary Procedures Center, Strategic Laboratory	Claudio Tavares Sacchi, Claudia Regina Gonçalves, Erica Valessa Ramos Gomes, Karoline Rodrigues Campos
EPI_ISL_735413	Militello Centro de Diagnosticos e Biopesquisa Clinica	Instituto Adolfo Lutz, Interdiciplinary Procedures Center, Strategic Laboratory	Claudio Tavares Sacchi, Claudia Regina Gonçalves, Erica Valessa Ramos Gomes, Karoline Rodrigues Campos
EPI_ISL_735414, EPI_ISL_735415	Unidade de Pronto Atendimento de Agenor de Campos	Instituto Adolfo Lutz, Interdiciplinary Procedures Center, Strategic Laboratory	Claudio Tavares Sacchi, Claudia Regina Gonçalves, Erica Valessa Ramos Gomes, Karoline Rodrigues Campos
EPI_ISL_735416	Centro de Saude II Dr Jose Paione Mococa	Instituto Adolfo Lutz, Interdiciplinary Procedures Center, Strategic Laboratory	Claudio Tavares Sacchi, Claudia Regina Gonçalves, Erica Valessa Ramos Gomes, Karoline Rodrigues Campos
EPI_ISL_735417	Unidade de Pronto Atendimento de Agenor de Campos	Instituto Adolfo Lutz, Interdiciplinary Procedures Center, Strategic Laboratory	Claudio Tavares Sacchi, Claudia Regina Gonçalves, Erica Valessa Ramos Gomes, Karoline Rodrigues Campos
EPI_ISL_735418	Hospital Regional do Vale do Paraiba	Instituto Adolfo Lutz, Interdiciplinary Procedures Center, Strategic Laboratory	Claudio Tavares Sacchi, Claudia Regina Gonçalves, Erica Valessa Ramos Gomes, Karoline Rodrigues Campos
EPI_ISL_735419	UBS Alvarenga	Instituto Adolfo Lutz, Interdiciplinary Procedures Center, Strategic Laboratory	Claudio Tavares Sacchi, Claudia Regina Gonçalves, Erica Valessa Ramos Gomes, Karoline Rodrigues Campos
EPI_ISL_735420	UBS Riacho Grande	Instituto Adolfo Lutz, Interdiciplinary Procedures Center, Strategic Laboratory	Claudio Tavares Sacchi, Claudia Regina Gonçalves, Erica Valessa Ramos Gomes, Karoline Rodrigues Campos
EPI_ISL_735421	UBS Sta Terezinha	Instituto Adolfo Lutz, Interdiciplinary Procedures Center, Strategic Laboratory	Claudio Tavares Sacchi, Claudia Regina Gonçalves, Erica Valessa Ramos Gomes, Karoline Rodrigues Campos
EPI_ISL_735422	UBS Dematchi	Instituto Adolfo Lutz, Interdiciplinary Procedures Center, Strategic Laboratory	Claudio Tavares Sacchi, Claudia Regina Gonçalves, Erica Valessa Ramos Gomes, Karoline Rodrigues Campos
EPI_ISL_735423, EPI_ISL_735424	Centro de Vigilancia a Saude de Diadema	Instituto Adolfo Lutz, Interdiciplinary Procedures Center, Strategic Laboratory	Claudio Tavares Sacchi, Claudia Regina Gonçalves, Erica Valessa Ramos Gomes, Karoline Rodrigues Campos
EPI_ISL_735425	Hospital e Maternidade Sao Lucas	Instituto Adolfo Lutz, Interdiciplinary Procedures Center, Strategic Laboratory	Claudio Tavares Sacchi, Claudia Regina Gonçalves, Erica Valessa Ramos Gomes, Karoline Rodrigues Campos
EPI_ISL_735426	Centro de Vigilancia a Saude de Diadema	Instituto Adolfo Lutz, Interdiciplinary Procedures Center, Strategic Laboratory	Claudio Tavares Sacchi, Claudia Regina Gonçalves, Erica Valessa Ramos Gomes, Karoline Rodrigues Campos
EPI_ISL_735427	Instituto Adolfo Lutz - Regional de Santos	Instituto Adolfo Lutz, Interdiciplinary Procedures Center, Strategic Laboratory	Claudio Tavares Sacchi, Claudia Regina Gonçalves, Erica Valessa Ramos Gomes, Karoline Rodrigues Campos
EPI_ISL_735428, EPI_ISL_735429	Hospital Nipo Brasileiro	Instituto Adolfo Lutz, Interdiciplinary Procedures Center, Strategic Laboratory	Claudio Tavares Sacchi, Claudia Regina Gonçalves, Erica Valessa Ramos Gomes, Karoline Rodrigues Campos
EPI_ISL_735430	Instituto Adolfo Lutz - Regional de Santos	Instituto Adolfo Lutz, Interdiciplinary Procedures Center, Strategic Laboratory	Claudio Tavares Sacchi, Claudia Regina Gonçalves, Erica Valessa Ramos Gomes, Karoline Rodrigues Campos
EPI_ISL_735431, EPI_ISL_735432	Hospital Nipo Brasileiro	Instituto Adolfo Lutz, Interdiciplinary Procedures Center, Strategic Laboratory	Claudio Tavares Sacchi, Claudia Regina Gonçalves, Erica Valessa Ramos Gomes, Karoline Rodrigues Campos
EPI_ISL_735433	Posto de Atendimento Saude Cidade Pasc Cajati	Instituto Adolfo Lutz, Interdiciplinary Procedures Center, Strategic Laboratory	Claudio Tavares Sacchi, Claudia Regina Gonçalves, Erica Valessa Ramos Gomes, Karoline Rodrigues Campos
EPI_ISL_735436	Nucleic Acid Testing - Rwanda National Reference Laboratory	GIGA Medical Genomics	Yvan Butera,Keith Durkin, Maria Artesi, Bouchra Boujemla, Robert Rutayisire, Patrick Tuyisenge, Esperence Umumararungu, Sébastien Bontems, Marie-Pierre Hayette, Swaibu Gatare, Jacob Souopgui, Sabin Nsanzimana, Vincent Bours, Léon Mutesa
EPI_ISL_735437	Nucleic Acid Testing - Rwanda National Reference Laboratory	GIGA Medical Genomics	Yvan Butera, Keith Durkin, Maria Artesi, Bouchra Boujemla, Robert Rutayisire, Patrick Tuyisenge, Esperence Umumararungu, Sébastien Bontems, Marie-Pierre Hayette, Swaibu Gatare, Jacob Souopgui, Sabin Nsanzimana, Vincent Bours, Léon Mutesa
EPI_ISL_735438	Nuclei Acid Testing - Rwanda National Reference Laboratory	GIGA Medical Genomics	Yvan Butera, Keith Durkin, Maria Artesi, Bouchra Boujemla, Robert Rutayisire, Patrick Tuyisenge, Esperence Umumararungu, Sébastien Bontems, Marie-Pierre Hayette, Swaibu Gatare, Jacob Souopgui, Sabin Nsanzimana, Vincent Bours, Léon Mutesa
EPI_ISL_737201	National Reference Laboratory, Nigeria Centre for Disease Control.	National Reference Laboratory, Nigeria Centre for Disease Control, Gaduwa,Abuja, Nigeria	Dr Ndodo Nnaemeka, Olusola Akanbi, Chimaobi Chukwu, Dr Adesuyi Omoare, oluwaseyi Ajegbe, Grace Esebanmen, Nwando Mba, Shirlee Wohl, Anthony Ahumibe, Celestina Obiekea, Catherine Okoi, Akinpelu Afolabi, Kingsley Njoku, Dr Sikiru Badaru, Dr Chinwe Ochu, Dr Chikwe Ihekweazu

EPI_ISL_738122, EPI_ISL_738123, EPI_ISL_738124, EPI_ISL_738125, EPI_ISL_738126, EPI_ISL_738127, EPI_ISL_738128, EPI_ISL_738130	IZSM-U.O.C. Virologia	Istituto Zooprofilattico Sperimentale del Mezzogiorno	Maurizio Viscardi, Lorena Cardillo, Giovanna Fusco
EPI_ISL_745191	Mubarak Al-Kabeer Hospital	Virology Unit, Microbiology Department, Faculty of Medicine, Kuwait University	Nada Madi
EPI_ISL_745223, EPI_ISL_745259	Molecular diagnostic laboratory of Federal Budget Institution of Science "Central Research Institute of Epidemiology" of The Federal Service on Customers' Rights Protection and Human Well-being Surveillance	Group of Genomics and Postgenomic Technologies of Central Research Institute of Epidemilology	Samoilov AE, Kaptelova VV, Korneenko EV, Dudorova AV, Saenko SS, Speranskaya AS, Tivanova EV, Shipulina OY, Akimkin VG
EPI_ISL_747234	National Institute of Health Research and Development	National Institute of Health Research and Development	Subangkit; Indrasari,ND; Wulandari,D; Monika,M; Pawestri,HA; Puspa,KD; Nugraha,AA; Ikawati,HD; Pangesti,KNA; Soekarso,T; Paisal; Setiawaty,Vivi
EPI_ISL_747242	Pathogen Lab (BSL3), Biomedical Innovation Department, Applied and Experimental Biology Division, Scientific Research Center and High Education from Ensenada (CICESE)	Pathogen Laboratory (BSL3), Biomedical Innovation Department, Experimental and Applied Biology Division, Scientific Research Center and High Education from Ensenada (CICESE)	Cervantes-Luevano K, Martinez M, Saavedra A, Galindo C and Licea-Navarro A
EPI_ISL_751245, EPI_ISL_751246, EPI_ISL_751277, EPI_ISL_751278, EPI_ISL_751279, EPI_ISL_751280, EPI_ISL_751281, EPI_ISL_751282, EPI_ISL_751283, EPI_ISL_751284, EPI_ISL_751285, EPI_ISL_751286, EPI_ISL_751287, EPI_ISL_751288, EPI_ISL_751289, EPI_ISL_751290, EPI_ISL_751291, EPI_ISL_751292, EPI_ISL_751293, EPI_ISL_751294, EPI_ISL_751295, EPI_ISL_751296, EPI_ISL_751298, EPI_ISL_751299, EPI_ISL_751300, EPI_ISL_751302, EPI_ISL_751303, EPI_ISL_751304, EPI_ISL_751305, EPI_ISL_751306, EPI_ISL_751307, EPI_ISL_751308, EPI_ISL_751309, EPI_ISL_751310, EPI_ISL_751311, EPI_ISL_751312, EPI_ISL_751313, EPI_ISL_751315, EPI_ISL_751317			
see above	Devki Devi Foundation, a unit of Max Healthcare	CSIR-IGIB/Max	Rajesh Pandey#, Samreen Siddiqui, Janani Srinivasa Vasudevan, Akshay Kanakan, Ranjeet Maurya, Uzma Shamim, Bansidhar Tarai, Akansha Tyagi, Mitali Mukerji, Poonam Das, Sujeet Jha, Mohammed Faruq, Anurag Agrawal
EPI_ISL_751337	IRCCS Sacro Cuore Don Calabria Hospital, Department of Infectious, Tropical Diseases & Microbiology	University of Verona, Department of Biotechnology	Antonio Mori, Michela Deiana, Elena Pomari, Chiara Piubelli; Giulia Lopatriello, Luca Marcolungo, Cristina Beltrami, Chiara Degli Esposti, Emanuela Cosentino, Massimo Delledonne
EPI_ISL_753699	RSUD Ulin Banjarmasin Kalimantan Selatan	National Institute of Health Research and Development	Subangkit;Pawestri,HA;Puspa,KD;Nugraha,AA;Ikawati,HD;Pangesti,KNA;Soekarso,T;Puspandari,N;Pasaribu,M;Setiawaty,V
EPI_ISL_754239	Santosa Hospital Bandung Central	"School of Life Sciences and Technology & School of Pharmacy-Institut Teknologi Bandung; Molecular Genetics Laboratory-Faculty of Medicine-Universitas Padjadjaran; Laboratorium Kesehatan Provinsi Jawa Barat"	Marselina Irasonia Tan, Yunia Sribudiani, Catur Riani, Azzania Fibriani, Husna Nugrahapraja, Tarwadi, Ema Rahmawati, Savira Ekawardhani, Hesti Lina Wiraswati, Ryan Bayusantika Ristandi, Rifky Waluyajati Rachman, Cut Nur Cinthia Alamanda, Lia Faridah, Miftahul Faridl, Karimatu Khoirunnisa, Hammam Riza, Soni Solistia Wirawan, Agung Eru Wibowo, Irvan Faizal
EPI_ISL_754241	Dinkes Tasikmalaya	"School of Life Sciences and Technology & School of Pharmacy-Institut Teknologi Bandung; Molecular Genetics Laboratory-Faculty of Medicine-Universitas Padjadjaran; Laboratorium Kesehatan Provinsi Jawa Barat"	Husna Nugrahapraja, Marselina Irasonia Tan, Yunia Sribudiani, Catur Riani, Azzania Fibriani, Tarwadi, Ema Rahmawati, Savira Ekawardhani, Hesti Lina Wiraswati, Ryan Bayusantika Ristandi, Rifky Waluyajati Rachman, Cut Nur Cinthia Alamanda, Lia Faridah, Miftahul Faridl, Karimatu Khoirunnisa, Hammam Riza, Soni Solistia Wirawan, Agung Eru Wibowo, Irvan Faizal
EPI_ISL_756293	Center for Biotechnology and Cell Therapy, São Rafael Hospital, Salvador, Brazil	Center for Biotechnology and Cell Therapy, São Rafael Hospital, Salvador, Brazil	Carolina Kymie Vasques Nonaka, Marília Miranda Franco, Tiago Gräf, Ana Verena Almeida Mendes, Renato Santana de Aguiar, Marta Giovanetti, Bruno Solano de Freitas Souza
EPI_ISL_756401	National Institute of Health Research and Development	National Institute of Health Research and Development	Adam,K; Wibowo,HA; Ramadhany,R; Rukminiati,Y; Agustiningsih; Pawestri,HA; Subangkit; Puspa,KD; Nugraha,AA; Ikawati,HD; Pangesti,KNA; Soekarso,T; Susilarini,NK; Hariastuti,NI; Nikmah,UA; Mursinah; Febriyani,A; Herman,R; Susanti,N; Herna; Febriyanti,T; Nurhadi,M; Kurniawati,J; Kipuw,NL; Muna,F; Indalao,IL; Rizki,A; Puspandari,N; Setiawaty,Vivi
EPI_ISL_759960	Surabaya Public Health Office	Institute of Tropical Disease, Universitas Airlangga	Aldise M Nastri, Jezzy R Dewantari, Rima R Prasetya, Krisnoadi Rahardjo, Febria Rachmanita, Gatot Soegiarto, Laksmi Wulandari, Resti Yudhawati, Soetjipto, Yasuko Mori, Maria I Lusida, Kazufumi Shimizu
EPI_ISL_759963	RSUD Sidoarjo	Institute of Tropical Disease, Universitas Airlangga	Aldise M Nastri, Jezzy R Dewantari, Rima R Prasetya, Krisnoadi Rahardjo, Atok Irawan, Gatot Soegiarto, Laksmi Wulandari, Resti Yudhawati, Yasuko Mori, Soetjipto, Kazufumi Shimizu ,Maria I Lusida
EPI_ISL_760031	Hong Kong Department of Health	School of Public Health, The University of Hong Kong	Daniel Chu, Haogao Gu, Pavithra Krishnan, Daisy Ng, Gigi Liu, Carrie Wan, Malik Peiris, Leo Poon
EPI_ISL_770009	Area De Salud Escazu (Coopesana)	Inciensa, Instituto Costarricense de Investigación y Enseñanza en Nutrición y Salud	Francisco Duarte, Hebleen Porras, Claudio Soto-Garita, Estela Cordero, Adriana Godínez & Melany Calderón
EPI_ISL_776764, EPI_ISL_776765	Instituto Adolfo Lutz - Regional de Santo Andre	Instituto Adolfo Lutz, Interdiciplinary Procedures Center, Strategic Laboratory	Claudio Tavares Sacchi, Claudia Regina Gonçalves, Erica Valessa Ramos Gomes, Karoline Rodrigues Campos
EPI_ISL_779383, EPI_ISL_779391, EPI_ISL_779395	Siloam Hospital Lippo Village	Mochtar Riady Institute for Nanotechnology-Universitas Pelita Harapan	Aksar C Lages, David Rustandi, Ivet M Suriapranata, Rinaldy Kusuma, Febi Andriani, Tri Shinta Kurniasih, Riska N Taufik, Young O Larasati, Rury M Wahyuni, Irawan Yusuf
EPI_ISL_780418	CERBA LAB	CERBA LAB	Roquebert B; Costa JM; Hedbaut E; Trombert S; Lecorche E; Verdurme L; Malek Ramdane, Olivi M; Haïm-Boukobza S
EPI_ISL_788933, EPI_ISL_788936	Centre de Recherche et de Formation en Infectiologie Guinée	TransVIHMI, IRD/INSERM/Monpellier University	Alpha Kabinet KEITA, Abdoul Karim SOUMAH, Abdoulaye TOURE, Moriba POVOGUI, Joel KOIVOGUI, Jean-louis MONEMOU, Mamadou Saliou SOW, Penda Malhado DIALLO, Mamadou Bhoye KEITA, Alimou CAMARA, Kaba KOUROUMA, Mandiou DIAKITE, Mamadou Saliou BAH, Sakoba KEITA, Bouna Yatassaye, Christelle BUTEL, Laetitia SERRANO, Ahidjo AYOUBA, Eric DELAPORTE, Martine PEETERS
EPI_ISL_788937	Centre de Recherche et de Formation en Infectiologie Guinée	TransVIHMI, IRD/INSERM/Monpellier University	Alpha Kabinet KEITA, Abdoul Karim SOUMAH, Abdoulaye TOURE, Moriba POVOGUI, Joel KOIVOGUI, Jean-louis MONEMOU, Mamadou Saliou SOW, Penda Malhado DIALLO, Mamadou Bhoye KEITA, Alimou CAMARA, Kaba KOUROUMA, Mandiou DIAKITE, Mamadou Saliou BAH, Sakoba KEITA, Bouna
EPI_ISL_788938	Centre de Recherche et de Formation en Infectiologie Guinée	TransVIHMI, IRD/INSERM/Monpellier University	Alpha Kabinet KEITA, Abdoul Karim SOUMAH, Abdoulaye TOURE, Moriba POVOGUI, Joel KOIVOGUI, Jean-louis MONEMOU, Mamadou Saliou SOW, Penda Malhado DIALLO, Mamadou Bhoye KEITA, Alimou CAMARA, Kaba KOUROUMA, Mandiou DIAKITE, Mamadou Saliou BAH, Sakoba KEITA, Bouna Yatassaye, Christelle BUTEL, Laetitia SERRANO, Ahidjo AYOUBA, Eric DELAPORTE, Martine PEETERS
EPI_ISL_788953, EPI_ISL_788954, EPI_ISL_788955, EPI_ISL_788956, EPI_ISL_788957, EPI_ISL_788958, EPI_ISL_788959, EPI_ISL_788960, EPI_ISL_788961, EPI_ISL_788970, EPI_ISL_788971, EPI_ISL_788972, EPI_ISL_788973, EPI_ISL_788974, EPI_ISL_788975, EPI_ISL_788976, EPI_ISL_788977, EPI_ISL_788978			
see above	Ospedale "Di Venere"	Beaconlab (Bioinformatics, Evolution and Comparative Genomics lab), Dept of Biosciences, University on Milan	Iacobellis M, d'Avenia M, Piluscio R, Parisi A, Chiara M, Manzari C, Pesole G
EPI_ISL_792115, EPI_ISL_792116	Instituto Adolfo Lutz - Regional de Taubate	Instituto Adolfo Lutz, Interdiciplinary Procedures Center, Strategic Laboratory	Claudio Tavares Sacchi, Claudia Regina Gonçalves, Erica Valessa Ramos Gomes, Karoline Rodrigues Campos
EPI_ISL_804949, EPI_ISL_804950, EPI_ISL_804951, EPI_ISL_804952, EPI_ISL_804953, EPI_ISL_804954, EPI_ISL_804955, EPI_ISL_804956, EPI_ISL_804957, EPI_ISL_804958, EPI_ISL_804959, EPI_ISL_804960, EPI_ISL_804961, EPI_ISL_804962, EPI_ISL_804965, EPI_ISL_804966, EPI_ISL_804967, EPI_ISL_804970, EPI_ISL_804977			
see above	Hospital Comarcal de Melilla	Instituto de Salud Carlos III	Iglesias-Caballero, M. Molinero Calamita, M. González-Esguevillas, M. Camarero, S. Pozo, F. Casas, I. Jiménez, P. Jiménez, M. Zaballos, A. Monzón, S. Varona, S. Juliá, M. Cuesta, I, J. López
EPI_ISL_807157	Mubarak Al-Kabeer Hospital	Virology Unit, Department of Microbiology, Faculty of Medicine	Dr. Nada Madi, Dr. Mohammed Sadeq, Mrs. Anfal Al-Adwani
EPI_ISL_811161, EPI_ISL_811162, EPI_ISL_811163, EPI_ISL_811164, EPI_ISL_811165, EPI_ISL_811166, EPI_ISL_811167, EPI_ISL_811168, EPI_ISL_811169, EPI_ISL_811170, EPI_ISL_811171, EPI_ISL_811172, EPI_ISL_811173, EPI_ISL_811174, EPI_ISL_811175, EPI_ISL_811176, EPI_ISL_811177, EPI_ISL_811178, EPI_ISL_811179, EPI_ISL_811180, EPI_ISL_811181, EPI_ISL_811182, EPI_ISL_811183, EPI_ISL_811184, EPI_ISL_811185, EPI_ISL_811186, EPI_ISL_811187, EPI_ISL_811188, EPI_ISL_811189, EPI_ISL_811190, EPI_ISL_811191, EPI_ISL_811192, EPI_ISL_811193			
see above	Dharwad	CSIR Institute of Genomics and Integrative Biology	Dr. Shivarudrappa B Bhairappanavar, Rahul Bhoyar, Mohammed Imran, Mohit Divakar, Disha Sharma, Dr. Vijay A Yenagi, Dr. Suresh B Arakera, Dr. Amit Ugargol, Dr. Rgavendra B Nayak, Bani Jolly, Abhinav Jain, Paras Sehgal, Gyan Ranjan, Vinod Scaria, Sridhar Sivasubbu
EPI_ISL_826520	University of Bari Biomedical Sciences and Human Oncology	University of Bari Biomedical Sciences and Human Oncology	Chironna M, Sallustio A., Loconsole D., Accogli M.

EPI_ISL_831474, EPI_ISL_831645, EPI_ISL_831646, EPI_ISL_831660, EPI_ISL_831678, EPI_ISL_831681, EPI_ISL_831683, EPI_ISL_831685, EPI_ISL_831688, EPI_ISL_831689, EPI_ISL_831892, EPI_ISL_831898, EPI_ISL_831913, EPI_ISL_831938			
see above	Laboratório de Microbiologia Molecular - Universidade FEEVALE	Universidade Federal de Ciências da Saúde de Porto Alegre	Vinícius Bonetti Franceschi, Amanda de Menezes Mayer, Gabriel Dickin Caldana, Carla Andretta Moreira Neves, Patrícia Aline Gröhs Ferrareze, Gabriela Bettella Cybis, Ricardo Ariel Zimerman, Lívia Kmetzsch, Fernando Rosado Spilki, Claudia Elizabeth Thompson
EPI_ISL_833156	Instituto Adolfo Lutz - Regional de Sorocaba	Instituto Adolfo Lutz, Interdiciplinary Procedures Center, Strategic Laboratory	Claudio Tavares Sacchi, Claudia Regina Gonçalves, Erica Valessa Ramos Gomes, Karoline Rodrigues Campos
EPI_ISL_833191, EPI_ISL_833192, EPI_ISL_833193, EPI_ISL_833194, EPI_ISL_833195, EPI_ISL_833197, EPI_ISL_833198, EPI_ISL_833199	Hôpital Bichat Claude Bernard, Laboratoire de Virologie	IAME UMR1137 Inserm, Université de Paris, Hôpital Bichat	Antoine Bridier, Amélie Recoing, Quentin Le Hingrat, Lena Daniel, Siham Hamri, Gilles Collin, Alexandre Storto, Mélanie Bertine, Charlotte Charpentier, Nadhira Houhou-Fidouh, Diane Descamps, Benoit Visseaux
EPI_ISL_833391	Klinik Apotek Dein, Jakarta, Indonesia	Biosafety Level-3 Laboratory, Indonesian Institute of Sciences (LIPI)	Andri Wardiana, Anggia Prasetyoputri, Isa Nuryana, Ade Andriani, Anik Budhi Dharmayanthi, Syam Budi Iryanto, Ahmad Fathoni, Herman Irawan, Dadang Supriatna, Ratih Asmana Ningrum
EPI_ISL_833392	RSU Sayang Bunda, Makasar,Indonesia	Biosafety Level-3 Laboratory, Indonesian Institute of Sciences (LIPI)	Anggia Prasetyoputri, Isa Nuryana, Ade Andriani, Anik Budhi Dharmayanthi, Syam Budi Iryanto, Andri Wardiana, Ahmad Fathoni, Dini Nurdiani, Wahyuni, Ratih Asmana Ningrum
EPI_ISL_833493	RS PMI Bogor, Indonesia	Biosafety Level-3 Laboratory, Indonesian Institute of Sciences (LIPI)	Isa Nuryana, Ade Andriani, Anik Budhi Dharmayanthi, Syam Budi Iryanto, Andri Wardiana, Anggia Prasetyoputri, Ahmad Fathoni, Indriawati, Nurulliawaty, Ratih Asmana Ningrum
EPI_ISL_833494	RS PMI Bogor, Indonesia	Biosafety Level-3 Laboratory, Indonesian Institute of Sciences (LIPI)	Ade Andriani, Anik Budhi Dharmayanthi, Syam Budi Iryanto, Andri Wardiana, Anggia Prasetyoputri, Isa Nuryana, Ahmad Fathoni, Maritsa Nurfatwa, Anky Zannati, Ratih Asmana Ningrum
EPI_ISL_833495	Klinik Apotek Dein, Jakarta, Indonesia	Biosafety Level-3 Laboratory, Indonesian Institute of Sciences (LIPI)	Ade Andriani, Anik Budhi Dharmayanthi, Syam Budi Iryanto, Andri Wardiana, Anggia Prasetyoputri, Isa Nuryana, Ahmad Fathoni, Hariyatun, Akhirta Atikana, Ratih Asmana Ningrum
EPI_ISL_833497	Klinik Apotek Dein, Jakarta, Indonesia	Biosafety Level-3 Laboratory, Indonesian Institute of Sciences (LIPI)	Syam Budi Iryanto, Andri Wardiana, Anggia Prasetyoputri, Anik Budhi Dharmayanthi, Isa Nuryana, Ade Andriani, Ahmad Fathoni, Saiful anwar, Pamella Apriliana, Ratih Asmana Ningrum
EPI_ISL_833498	Kartika Pulomas Hospital, Jakarta, Indonesia	Biosafety Level-3 Laboratory, Indonesian Institute of Sciences (LIPI)	Anggia Prasetyoputri, Isa Nuryana, Ade Andriani, Anik Budhi Dharmayanthi, Syam Budi Iryanto, Andri Wardiana, Ahmad Fathoni, Winda Tasia, Pinka Alisa Diena Shafa, Ratih Asmana Ningrum
EPI_ISL_833499	RS Qadr, Tangerang, Indonesia	Biosafety Level-3 Laboratory, Indonesian Institute of Sciences (LIPI)	Isa Nuryana, Ade Andriani, Ahmad Fathoni, Anik Budhi Dharmayanthi, Syam Budi Iryanto, Andri Wardiana, Anggia Prasetyoputri, Ratna Dwi Ramadani, Sugiyono Saputra, Ratih Asmana Ningrum
EPI_ISL_833500	Klinik Apotek Dein, Jakarta, Indonesia	Biosafety Level-3 Laboratory, Indonesian Institute of Sciences (LIPI)	Anik Budhi Dharmayanthi, Syam Budi Iryanto, Andri Wardiana, Anggia Prasetyoputri, Isa Nuryana, Ade Andriani, Ahmad Fathoni, Ratih Asmana Ningrum
EPI_ISL_833503	RS MMC, Jakarta, Indonesia	Biosafety Level-3 Laboratory, Indonesian Institute of Sciences (LIPI)	Anggia Prasetyoputri, Isa Nuryana, Anik Budhi Dharmayanthi, Syam Budi Iryanto, Andri Wardiana, Ade Andriani, Ahmad Fathoni, Idris, Ruby Setiawan, Ratih Asmana Ningrum
EPI_ISL_833504	RS MMC, Jakarta, Indonesia	Biosafety Level-3 Laboratory, Indonesian Institute of Sciences (LIPI)	Isa Nuryana, Anik Budhi Dharmayanthi, Syam Budi Iryanto, Andri Wardiana, Anggia Prasetyoputri, Ade Andriani, Ahmad Fathoni, Ahmad Randy, Listiana Oktavia, Ratih Asmana Ningrum
EPI_ISL_833506	RS PMI, Bogor, Indonesia	Biosafety Level-3 Laboratory, Indonesian Institute of Sciences (LIPI)	Isa Nuryana, Ade Andriani, Ahmad Fathoni, Anik Budhi Dharmayanthi, Syam Budi Iryanto, Andri Wardiana, Anggia Prasetyoputri, Satrio Haryo Benowo, Gilang Akbariani, Ratih Asmana Ningrum
EPI_ISL_833571	RS Qadr, Tangerang, Indonesia	Biosafety Level-3 Laboratory, Indonesian Institute of Sciences (LIPI)	Ade Andriani, Ahmad Fathoni, Anik Budhi Dharmayanthi, Syam Budi Iryanto, Andri Wardiana, Anggia Prasetyoputri, Isa Nuryana, Erwin Fajar Hasrianda, Ryan Haryo Setiawan, Ratih Asmana Ningrum
EPI_ISL_837564, EPI_ISL_837569	Centro Nacional de Enfermedades Tropicales (CENETROP)	Laboratory of Respiratory Viruses and Measles, Oswaldo Cruz Institute, FIOCRUZ	Paola Resende, Roxana Loayza, Cinthia Avila, Luciana Appolinario, Fernando Motta, Anna Carolina Paixao, Ana Carolina Mendonca, Marilda Siqueira on behalf of the Fiocruz COVID-19 Genomic Surveillance Network
EPI_ISL_837602, EPI_ISL_837603, EPI_ISL_837607, EPI_ISL_837699, EPI_ISL_837700, EPI_ISL_837701, EPI_ISL_837702, EPI_ISL_837703, EPI_ISL_837704, EPI_ISL_837705, EPI_ISL_837706, EPI_ISL_837707, EPI_ISL_837708, EPI_ISL_837709, EPI_ISL_837710, EPI_ISL_837711, EPI_ISL_837712, EPI_ISL_837713, EPI_ISL_837715, EPI_ISL_837716, EPI_ISL_837717, EPI_ISL_837718, EPI_ISL_837719, EPI_ISL_837720, EPI_ISL_837721, EPI_ISL_837722, EPI_ISL_837723, EPI_ISL_837724, EPI_ISL_837725, EPI_ISL_837726, EPI_ISL_837727, EPI_ISL_837728, EPI_ISL_837729, EPI_ISL_837730, EPI_ISL_837731, EPI_ISL_837732, EPI_ISL_837733, EPI_ISL_837734, EPI_ISL_837735, EPI_ISL_837736, EPI_ISL_837737, EPI_ISL_837738, EPI_ISL_837739, EPI_ISL_837740, EPI_ISL_837741, EPI_ISL_837742, EPI_ISL_837743, EPI_ISL_837745, EPI_ISL_837746, EPI_ISL_837747, EPI_ISL_837748, EPI_ISL_837749, EPI_ISL_837750, EPI_ISL_837751, EPI_ISL_837753, EPI_ISL_837754, EPI_ISL_837755, EPI_ISL_837756, EPI_ISL_837757, EPI_ISL_837758, EPI_ISL_837761, EPI_ISL_837762, EPI_ISL_837763, EPI_ISL_837764, EPI_ISL_837765, EPI_ISL_837766, EPI_ISL_837767, EPI_ISL_837768, EPI_ISL_837769, EPI_ISL_837770, EPI_ISL_837771, EPI_ISL_837772, EPI_ISL_837773, EPI_ISL_837774, EPI_ISL_837775, EPI_ISL_837776, EPI_ISL_837777, EPI_ISL_837778, EPI_ISL_837780, EPI_ISL_837781, EPI_ISL_837782, EPI_ISL_837783, EPI_ISL_837784, EPI_ISL_837785, EPI_ISL_837786, EPI_ISL_837787, EPI_ISL_837789, EPI_ISL_837790, EPI_ISL_837791, EPI_ISL_837793, EPI_ISL_837794, EPI_ISL_837795, EPI_ISL_837796, EPI_ISL_837797, EPI_ISL_837798, EPI_ISL_837799, EPI_ISL_837800, EPI_ISL_837802, EPI_ISL_837803, EPI_ISL_837804, EPI_ISL_837805, EPI_ISL_837806, EPI_ISL_837807, EPI_ISL_837808, EPI_ISL_837809, EPI_ISL_843191, EPI_ISL_843192			
see above	Instituto Nacional de Enfermedades Respiratorias (INER)	Instituto Nacional de Enfermedades Respiratorias (INER)	Celia Boukadida, Margarita Matías-Florentino, Alma Rincón-Rubio, Hector Esteban Paz-Juárez, Olivia Briceño, Edgar Sevilla-Reyes, Fidencio Mejía-Nepomuceno, Mario Mújica-Sánchez, Eduardo Becerril-Vargas, José Arturo Martínez-Orozco, Alejandra Hernández-Terán, Jorge Salas-Hernández, Santiago Ávila-Ríos, Joel Armando Vázquez-Pérez
EPI_ISL_848574	Evandro Chagas Institute	Evandro Chagas Institute	Santos, M.C.; Silva, A.M.; Junior, W.D.C.; Barbagelata, L.S.; Ferreira, J.A.; Sousa, E.M.A.; da Silva, P.S.; Pinheiro, K.C.; L.C.; Sousa Junior, E.C.
EPI_ISL_849650, EPI_ISL_849652	Servizio di igiene epidemiologia e sanità pubblica (SIESP)-Chieti	Istituto Zooprofilattico Sperimentale dell'Abruzzo e Molise "G.Caporale"	Lorusso A, Marcacci M, Di Domenico M, Curini V, Ancora M, Cammà C, Rinaldi A, Mangone I, Di Pasquale A, Puglia I, Savini G.
EPI_ISL_849653	Servizio di igiene e sanità pubblica (SIESP)-Teramo	Istituto Zooprofilattico Sperimentale dell'Abruzzo e Molise "G.Caporale"	Lorusso A, Marcacci M, Di Domenico M, Curini V, Ancora M, Cammà C, Rinaldi A, Mangone I, Di Pasquale A, Puglia I, Savini G.
EPI_ISL_849654, EPI_ISL_849656, EPI_ISL_849657, EPI_ISL_849659, EPI_ISL_849661	Servizio Igiene Epidemiologia e Sanità Pubblica (SIESP)-L'Aquila	Istituto Zooprofilattico Sperimentale dell'Abruzzo e Molise "G.Caporale"	Lorusso A, Marcacci M, Di Domenico M, Curini V, Ancora M, Cammà C, Rinaldi A, Mangone I, Di Pasquale A, Puglia I, Savini G.
EPI_ISL_849662, EPI_ISL_849663	Servizio di igiene epidemiologia e sanità pubblica (SIESP)-Chieti	Istituto Zooprofilattico Sperimentale dell'Abruzzo e Molise "G.Caporale"	Lorusso A, Marcacci M, Di Domenico M, Curini V, Ancora M, Cammà C, Rinaldi A, Mangone I, Di Pasquale A, Puglia I, Savini G.
EPI_ISL_850507	Gandhi Medical College and Hospital	VRDL-Gandhi Medical College	Nagamani Kammili, Rajeshwar Rao, Madhavi Latha Manolla, Winnie Thomas, Shailaja VV, Sudhamadhuri Devara, Vanisree Rajoli, Archana GJ,Sushma Rajyalakshmi Gudiseva, Sunitha Pakalapati, Manisha Rani, Amrithesh Kumar, Raja Rao Mesepogu, Vinay Shekar Reddy, Thrilok Chander Bingi, Sofia Banu, Divya Tej Sowpati
EPI_ISL_850509	Gandhi Medical College and Hospital	VRDL-Gandhi Medical College	Nagamani Kammili, Rajeshwar Rao, Winnie Thomas, Madhavi Latha Manolla, Shailaja VV, Sudhamadhuri Devara, Vanisree Rajoli, Archana GJ, Sushma Rajyalakshmi Gudiseva, Sunitha Pakalapati, Manisha Rani, Amrithesh Kumar, Raja Rao Mesepogu, Vinay Shekar Reddy, Thrilok Chander Bingi, Sofia Banu, Divya Tej Sowpati
EPI_ISL_850510	Gandhi Medical College and Hospital	MDRU-DHR,Gandhi Medical College	Nagamani Kammili, Winnie Thomas, Madhavi Latha Manolla, Shailaja VV, Sudhamadhuri Devara, Vanisree Rajoli, Archana GJ, Sushma Rajyalakshmi Gudiseva, Sunitha Pakalapati, Manisha Rani, Amrithesh Kumar, Raja Rao Mesepogu, Vinay Shekar Reddy, Thrilok Chander Bingi, Sofia Banu, Divya Tej Sowpati
EPI_ISL_850514	Gandhi Medical College and Hospital	MDRU-DHR,Gandhi Medical College	Nagamani Kammili, Madhavi Latha Manolla, Winnie Thomas, Shailaja VV, Sudhamadhuri Devara, Vanisree Rajoli, Archana GJ,Sushma Rajyalakshmi Gudiseva, Sunitha Pakalapati, Manisha Rani, Amrithesh Kumar, Raja Rao Mesepogu, Vinay Shekar Reddy, Thrilok Chander Bingi, Sofia Banu, Divya Tej Sowpati
EPI_ISL_851050	Gandhi Medical College and Hospital	CSIR-Centre for Cellular and Molecular Biology	Nagamani Kammili, Winnie Thomas, Madhavi Latha Manolla, Shailaja VV, Sudhamadhuri Devara, Vanisree Rajoli, Archana GJ, Sushma Rajyalakshmi Gudiseva, Sunitha Pakalapati, Manisha Rani, Amrithesh Kumar, Raja Rao Mesepogu, Vinay Shekar Reddy, Thrilok Chander Bingi, Sofia Banu, Divya Tej Sowpati
EPI_ISL_851051	Gandhi Medical College and Hospital	CSIR-Centre for Cellular and Molecular Biology	Nagamani Kammili, Madhavi Latha Manolla, Winnie Thomas, Shailaja VV, Sudhamadhuri Devara, Vanisree Rajoli, Archana GJ,Sushma Rajyalakshmi Gudiseva, Sunitha Pakalapati, Manisha Rani, Amrithesh Kumar, Raja Rao Mesepogu, Vinay Shekar Reddy, Thrilok Chander Bingi, Sofia Banu, Divya Tej

			Sowpati
EPI_ISL_852580, EPI_ISL_852581, EPI_ISL_852582, EPI_ISL_852586, EPI_ISL_852588, EPI_ISL_852591, EPI_ISL_852592	Max von Pettenkofer Institute, Virology, National Reference Center for Retroviruses, LMU München	Laboratory for Functional Genome Analysis, Dept. Genomics, Gene Center of the LMU Munich	Max Muenchhoff, Stefan Krebs, Alexander Graf, Oliver Keppler, Helmut Blum
EPI_ISL_853819	Department of Pathology, Landeskrankenhaus Graz II, Medical University Graz	Bergthaler laboratory, CeMM Research Center for Molecular Medicine of the Austrian Academy of Sciences	Lukas Endler, Alexandra Popa, Benedikt Agerer, Jakob-Wendelin Genger, Alexander Lercher, Anna Schedl, Thomas Penz, Michael Schuster, Jan Laine, Martin Senekowitsch, Christoph Bock, Andreas Bergthaler
EPI_ISL_861646, EPI_ISL_861647	Hospital Santa Marcelina Sao Paulo	Instituto Adolfo Lutz, Interdiciplinary Procedures Center, Strategic Laboratory	Claudio Tavares Sacchi, Claudia Regina Gonçalves, Erica Valessa Ramos Gomes, Karoline Rodrigues Campos
EPI_ISL_861648	Hospital e Pronto Socorro Portinari	Instituto Adolfo Lutz, Interdiciplinary Procedures Center, Strategic Laboratory	Claudio Tavares Sacchi, Claudia Regina Gonçalves, Erica Valessa Ramos Gomes, Karoline Rodrigues Campos
EPI_ISL_861649	Hospital Renascença Campinas	Instituto Adolfo Lutz, Interdiciplinary Procedures Center, Strategic Laboratory	Claudio Tavares Sacchi, Claudia Regina Gonçalves, Erica Valessa Ramos Gomes, Karoline Rodrigues Campos
EPI_ISL_861657	Hospital e Maternidade Sino Brasileiro	Instituto Adolfo Lutz, Interdiciplinary Procedures Center, Strategic Laboratory	Claudio Tavares Sacchi, Claudia Regina Gonçalves, Erica Valessa Ramos Gomes, Karoline Rodrigues Campos
EPI_ISL_861658	Hospital Municipal Antônio Giglio	Instituto Adolfo Lutz, Interdiciplinary Procedures Center, Strategic Laboratory	Claudio Tavares Sacchi, Claudia Regina Gonçalves, Erica Valessa Ramos Gomes, Karoline Rodrigues Campos
EPI_ISL_861660	PS e Maternidade Nair Fonseca Leitao Arantes	Instituto Adolfo Lutz, Interdiciplinary Procedures Center, Strategic Laboratory	Claudio Tavares Sacchi, Claudia Regina Gonçalves, Erica Valessa Ramos Gomes, Karoline Rodrigues Campos
EPI_ISL_861662	CS I Tacito Leite de Carvalho e Silva	Instituto Adolfo Lutz, Interdiciplinary Procedures Center, Strategic Laboratory	Claudio Tavares Sacchi, Claudia Regina Gonçalves, Erica Valessa Ramos Gomes, Karoline Rodrigues Campos
EPI_ISL_861664	Instituto Adolfo Lutz - Regional de Campinas	Instituto Adolfo Lutz, Interdiciplinary Procedures Center, Strategic Laboratory	Claudio Tavares Sacchi, Claudia Regina Gonçalves, Erica Valessa Ramos Gomes, Karoline Rodrigues Campos
EPI_ISL_861665	Instituto Adolfo Lutz - Regional de Taubate	Instituto Adolfo Lutz, Interdiciplinary Procedures Center, Strategic Laboratory	Claudio Tavares Sacchi, Claudia Regina Gonçalves, Erica Valessa Ramos Gomes, Karoline Rodrigues Campos
EPI_ISL_861666	PSF Dr. Antonio Pires de Almeida	Instituto Adolfo Lutz, Interdiciplinary Procedures Center, Strategic Laboratory	Claudio Tavares Sacchi, Claudia Regina Gonçalves, Erica Valessa Ramos Gomes, Karoline Rodrigues Campos
EPI_ISL_861667	Instituto Adolfo Lutz - Regional de Rio Claro	Instituto Adolfo Lutz, Interdiciplinary Procedures Center, Strategic Laboratory	Claudio Tavares Sacchi, Claudia Regina Gonçalves, Erica Valessa Ramos Gomes, Karoline Rodrigues Campos
EPI_ISL_862594	Hospital Comarcal de Melilla	Instituto de Salud Carlos III	Iglesias-Caballero, M. Camarero, S. Molinero Calamita, M. González-Esguevillas, M. Pozo, F. Casas, I. Jiménez, P. Jiménez, M. Zaballos, A. Monzón, S. Varona, S. Juliá, M. Cuesta, I. López, J.
EPI_ISL_871790	Multidisciplinary Research Unit, DHR-ICMR, Institute of Medical Sciences, Banaras Hindu University	Multidisciplinary Research Unit, DHR-ICMR, Institute of Medical Sciences, Banaras Hindu University	Royana Singh, Priyoneel Basu, Ashish, Nitish Kumar Singh, Abhay Kumar Yadav, Manpreet Kaur, Arup Acharjee, Deepa Devadas, Chetan Sahni, Gulshan Kumar, Tribhuwan Mohan Mohapatra, Richa Arya, Prashant Singh, Jay Prakash Maurya, Surendra Pratap Mishra
EPI_ISL_871984	Hospital Universitario Severo Ochoa	Instituto de Salud Carlos III	Iglesias-Caballero, M. Camarero, S. Molinero Calamita, M. González-Esguevillas, M. Pozo, F. Casas, I. Jiménez, P. Jiménez, M. Zaballos, A. Monzón, S. Varona, S. Juliá, M. Cuesta, I. García, M.L.
EPI_ISL_871985, EPI_ISL_871986, EPI_ISL_871987, EPI_ISL_871988, EPI_ISL_871989, EPI_ISL_871990	Hospital Clínico San Carlos	Instituto de Salud Carlos III	Iglesias-Caballero, M. Camarero, S. Molinero Calamita, M. González-Esguevillas, M. Pozo, F. Casas, I. Jiménez, P. Jiménez, M. Zaballos, A. Monzón, S. Varona, S. Juliá, M. Cuesta, I. Rodríguez, I.
EPI_ISL_871991	Hospital Universitario Severo Ochoa	Instituto de Salud Carlos III	Iglesias-Caballero, M. Camarero, S. Molinero Calamita, M. González-Esguevillas, M. Pozo, F. Casas, I. Jiménez, P. Jiménez, M. Zaballos, A. Monzón, S. Varona, S. Juliá, M. Cuesta, I. García, M.L.
EPI_ISL_871992, EPI_ISL_871993	Hospital Clínico San Carlos	Instituto de Salud Carlos III	Iglesias-Caballero, M. Camarero, S. Molinero Calamita, M. González-Esguevillas, M. Pozo, F. Casas, I. Jiménez, P. Jiménez, M. Zaballos, A. Monzón, S. Varona, S. Juliá, M. Cuesta, I. Rodríguez, I.
EPI_ISL_872009, EPI_ISL_872010, EPI_ISL_872011, EPI_ISL_872012, EPI_ISL_872014, EPI_ISL_872016, EPI_ISL_872017, EPI_ISL_872018, EPI_ISL_872019, EPI_ISL_872020, EPI_ISL_872021, EPI_ISL_872022, EPI_ISL_872023, EPI_ISL_872024, EPI_ISL_872025, EPI_ISL_872026			
see above	Ospedale "Di Venere"	Beaconlab (Bioinformatics, Evolution and Comparative Genomics lab), Dept of Biosciences, University on Milan	Iacobellis M, d'Avenia M, Piluscio R, Parisi A, Chiara M, Manzari C, Pesole G
EPI_ISL_872628, EPI_ISL_872629, EPI_ISL_872630, EPI_ISL_872631, EPI_ISL_872632, EPI_ISL_872633, EPI_ISL_872634, EPI_ISL_872635, EPI_ISL_872636, EPI_ISL_872637, EPI_ISL_872638, EPI_ISL_872639, EPI_ISL_872640			
see above	Pathogenic Microorganisms Variability Laboratory	Pathogenic Microorganisms Variability Laboratory	Alexey Shchetinin, Olesya Venchakova, Maria Nikiforova, Andrei Siniavin, Nadezhda Kuznetsova, Elena Shidlovskaya, Elizaveta Divisenko, Kirill Krasnoslobotsev, Evgeniya Mukasheva, Anna Ignatieva, Svetlana Trushakova, Andrey Pochtovyy, Valeria Bacalin, Evgeny Usachev, Olga Burgasova, Ludmila Kolobukhina, Svetlana Smetanina, Elena Burtseva, Denis Logunov, Vladimir Gushchin, Alexander Gintsburg
EPI_ISL_872641, EPI_ISL_872642, EPI_ISL_875515	Influenza etiology and epidemiology laboratory	Pathogenic Microorganisms Variability Laboratory	Alexey Shchetinin, Olesya Venchakova, Maria Nikiforova, Andrei Siniavin, Nadezhda Kuznetsova, Elena Shidlovskaya, Elizaveta Divisenko, Kirill Krasnoslobotsev, Evgeniya Mukasheva, Anna Ignatieva, Svetlana Trushakova, Andrey Pochtovyy, Valeria Bacalin, Evgeny Usachev, Olga Burgasova, Ludmila Kolobukhina, Svetlana Smetanina, Elena Burtseva, Denis Logunov, Vladimir Gushchin, Alexander Gintsburg
EPI_ISL_882657	LACEN do Estado do Piaui, Dr. Costa Alvarenga	Instituto Adolfo Lutz, Interdiciplinary Procedures Center, Strategic Laboratory	Claudio Tavares Sacchi, Claudia Regina Gonçalves, Erica Valessa Ramos Gomes, Karoline Rodrigues Campos
EPI_ISL_889357, EPI_ISL_889358, EPI_ISL_889359, EPI_ISL_889360, EPI_ISL_889362, EPI_ISL_889363, EPI_ISL_889364, EPI_ISL_889365	Motol University Hospital	Institute of Applied Biotechnologies a.s.	Petr Klempt, Ondej Brzo, Martin Kašný, Kateina Kvapilová, Pavel Devínek, Petr Kvapil
EPI_ISL_895834	Molecular biology division, Institute of Clinical Biochemistry and Diagnostics, Charles University, Faculty of Medicine in Hradec Králové and University Hospital Hradec Králové	Molecular biology division, Institute of Clinical Biochemistry and Diagnostics, Charles University, Faculty of Medicine in Hradec Králové and University Hospital Hradec Králové	Helena Kovaíková, Petr Brož, Ivana Baranová, Kateina Hrochová, Tereza Baková, Jitka Novotná, Kateina Pehlíková, Vladimír Palika. Cooperation project with BioVendor-R&D and bioinformatics company BIOXSYS s r.o.
EPI_ISL_900691, EPI_ISL_900692, EPI_ISL_900693, EPI_ISL_900694, EPI_ISL_900695, EPI_ISL_900696, EPI_ISL_900697, EPI_ISL_900698, EPI_ISL_900699, EPI_ISL_900700, EPI_ISL_900701, EPI_ISL_900702, EPI_ISL_900704, EPI_ISL_900705, EPI_ISL_900706, EPI_ISL_900707, EPI_ISL_900708, EPI_ISL_900709, EPI_ISL_900710, EPI_ISL_900711, EPI_ISL_900712, EPI_ISL_900713, EPI_ISL_900714, EPI_ISL_900715, EPI_ISL_900716, EPI_ISL_900717, EPI_ISL_900718, EPI_ISL_900719, EPI_ISL_900720, EPI_ISL_900721, EPI_ISL_900722, EPI_ISL_900723, EPI_ISL_900724, EPI_ISL_900725, EPI_ISL_900727, EPI_ISL_900728, EPI_ISL_900729, EPI_ISL_900730, EPI_ISL_900731, EPI_ISL_900732, EPI_ISL_900733, EPI_ISL_900734, EPI_ISL_900735, EPI_ISL_900736, EPI_ISL_900737, EPI_ISL_900738, EPI_ISL_900740, EPI_ISL_900741			
see above	Bozeman Health Deaconess Hospital	Wiedenheft lab, Montana State University	Artem Nemudryi, Anna Nemudraia, Tanner Wiegand, Joseph Nichols, Deann T. Snyder, Jodi F. Hedges, Calvin Cicha, Helen Lee, Karl K. Vanderwood, Diane Bimczok, Mark A. Jutila and Blake Wiedenheft
EPI_ISL_902917	Tanjungpura University Hospital	Tanjungpura University Hospital	Andriani; Mahyarudin; Novianry V; Liana DF; Shofiyah SS Astuti P; Kahtan MI; Rialita A; Putri EA; Windarti W; Sastriawan W
EPI_ISL_903339, EPI_ISL_903340, EPI_ISL_903341, EPI_ISL_903342, EPI_ISL_903343, EPI_ISL_903344, EPI_ISL_903345, EPI_ISL_903346	Bozeman Health Deaconess Hospital	Wiedenheft lab, Montana State University	Artem Nemudryi, Anna Nemudraia, Tanner Wiegand, Joseph Nichols, Deann T. Snyder, Jodi F. Hedges, Calvin Cicha, Helen Lee, Karl K. Vanderwood, Diane Bimczok, Mark A. Jutila and Blake Wiedenheft
EPI_ISL_906060	Tilia Laboratories s.r.o.	Tilia Laboratories s.r.o.	Sona Pekova, MD, PhD.

EPI_ISL_913914	Instituto de Diagnostico y Referencia Epidemiologicos INDRE_RNLSP	Instituto de Diagnostico y Referencia Epidemiologicos (INDRE)	Claudia Wong-Arambula, Abril Rodriguez-Maldonado, Fabiola Garces-Ayala, Adnan Araiza-Rodriguez, David Fragoso-Fonseca, Sergio Rangel-Guerrero, Mayra Jimenez-Morales, Nancy Munoz-Hernandez, Natividad Cruz-Ortiz, Tatiana Nunez-Garcia, Gisela Barrera-Badillo, Lucia Hernandez-Rivas, Irma Lopez-Martinez, Ernesto Ramirez-Gonzalez.
EPI_ISL_930855, EPI_ISL_930857, EPI_ISL_930858	Central Laboratory of Public Health of Rio Grande do Sul (Lacen-RS)	State Center for Health Surveillance of the Health Department of the State of Rio Grande do Sul (CEVS/SES-RS)	Barcellos R, Campos A, Dornelles C, Godinho F, Gonzalez A, Gregianini T, Molina C, Salvato R, Schaurich A,
EPI_ISL_940608	Laboratório Sao Lucas	Instituto Adolfo Lutz, Interdiciplinary Procedures Center, Strategic Laboratory	Claudio Tavares Sacchi, Claudia Regina Gonçalves, Erica Valessa Ramos Gomes, Karoline Rodrigues Campos
EPI_ISL_940756, EPI_ISL_940895, EPI_ISL_940896, EPI_ISL_940897, EPI_ISL_940898, EPI_ISL_940899, EPI_ISL_940900, EPI_ISL_940902, EPI_ISL_940903, EPI_ISL_940904, EPI_ISL_940906, EPI_ISL_940907, EPI_ISL_940908, EPI_ISL_940909, EPI_ISL_940910, EPI_ISL_940911, EPI_ISL_940912, EPI_ISL_940913, EPI_ISL_940914, EPI_ISL_940916, EPI_ISL_940918, EPI_ISL_940919, EPI_ISL_940921, EPI_ISL_940922, EPI_ISL_940923, EPI_ISL_940924, EPI_ISL_940925, EPI_ISL_940927, EPI_ISL_940928, EPI_ISL_940929, EPI_ISL_940930, EPI_ISL_940931, EPI_ISL_940932, EPI_ISL_940933, EPI_ISL_940934, EPI_ISL_940935, EPI_ISL_940950, EPI_ISL_940951, EPI_ISL_940952, EPI_ISL_940953, EPI_ISL_940955, EPI_ISL_940956, EPI_ISL_940957, EPI_ISL_940958, EPI_ISL_940959			
see above	Centers for Disease Control and Prevention, Dengue Branch	Centers for Disease Control and Prevention, Dengue Branch	Gilberto A. Santiago, Glenda Gonzalez, Betzabel Flores, Keyla Charriez, Gabriela Paz-Bailey, Jorge L. Munoz-Jordan
EPI_ISL_941986, EPI_ISL_941987, EPI_ISL_941988, EPI_ISL_941989, EPI_ISL_941990, EPI_ISL_941991, EPI_ISL_941992, EPI_ISL_941993, EPI_ISL_941994	Instituto Nacional de Salud, Bogotá, Colombia	Centro de Investigaciones en Microbiología y Biotecnología-UR (CIMBIUR), Facultad de Ciencias Naturales, Universidad del Rosario, Bogotá, Colombia Instituto Nacional de Salud, Bogotá, Colombia Icahn School of Medicine at Mount Sinai, New York, USA	Luz Helena Patiño, Marina Muñoz, Nathalia Ballesteros, Carolina Hernández, Carolina Flórez, Sergio Gomez, Adriana van de Guchte, Zenab Khan, Jayeeta Dutta, Hala Alejel Alshammary, Ana S. Gonzalez-Reiche, Matthew M. Hernandez, Emilia Mia Sordillo, Viviana Simon, Harm van Bakel, Alberto Paniz-Mondolfi, Juan David Ramírez
EPI_ISL_941995, EPI_ISL_941996, EPI_ISL_941997, EPI_ISL_941998	Centro de Investigaciones en Microbiología y Biotecnología-UR (CIMBIUR), Facultad de Ciencias Naturales, Universidad del Rosario, Bogotá, Colombia	Centro de Investigaciones en Microbiología y Biotecnología-UR (CIMBIUR), Facultad de Ciencias Naturales, Universidad del Rosario, Bogotá, Colombia Instituto Nacional de Salud, Bogotá, Colombia Icahn School of Medicine at Mount Sinai, New York, USA	Luz Helena Patiño, Marina Muñoz, Nathalia Ballesteros, Carolina Hernández, Carolina Flórez, Sergio Gomez, Adriana van de Guchte, Zenab Khan, Jayeeta Dutta, Hala Alejel Alshammary, Ana S. Gonzalez-Reiche, Matthew M. Hernandez, Emilia Mia Sordillo, Viviana Simon, Harm van Bakel, Alberto Paniz-Mondolfi, Juan David Ramírez
EPI_ISL_942003	Instituto Nacional de Salud, Bogotá, Colombia	Centro de Investigaciones en Microbiología y Biotecnología-UR (CIMBIUR), Facultad de Ciencias Naturales, Universidad del Rosario, Bogotá, Colombia Instituto Nacional de Salud, Bogotá, Colombia Icahn School of Medicine at Mount Sinai, New York, USA	Luz Helena Patiño, Marina Muñoz, Nathalia Ballesteros, Carolina Hernández, Carolina Flórez, Sergio Gomez, Adriana van de Guchte, Zenab Khan, Jayeeta Dutta, Hala Alejel Alshammary, Ana S. Gonzalez-Reiche, Matthew M. Hernandez, Emilia Mia Sordillo, Viviana Simon, Harm van Bakel, Alberto Paniz-Mondolfi, Juan David Ramírez
EPI_ISL_942004	Centro de Investigaciones en Microbiología y Biotecnología-UR (CIMBIUR), Facultad de Ciencias Naturales, Universidad del Rosario, Bogotá, Colombia	Centro de Investigaciones en Microbiología y Biotecnología-UR (CIMBIUR), Facultad de Ciencias Naturales, Universidad del Rosario, Bogotá, Colombia Instituto Nacional de Salud, Bogotá, Colombia Icahn School of Medicine at Mount Sinai, New York, USA	Luz Helena Patiño, Marina Muñoz, Nathalia Ballesteros, Carolina Hernández, Carolina Flórez, Sergio Gomez, Adriana van de Guchte, Zenab Khan, Jayeeta Dutta, Hala Alejel Alshammary, Ana S. Gonzalez-Reiche, Matthew M. Hernandez, Emilia Mia Sordillo, Viviana Simon, Harm van Bakel, Alberto Paniz-Mondolfi, Juan David Ramírez
EPI_ISL_942375, EPI_ISL_942897, EPI_ISL_942898	Central Laboratory of Public Health of Rio Grande do Sul (Lacen-RS)	State Center for Health Surveillance of the Health Department of the State of Rio Grande do Sul (CEVS/SES-RS)	Barcellos R, Campos A, Crescente L, Da Silva A, Dornelles C, Fonseca V, Garay L, Godinho F, Gonzalez A, Gregianini T, Molina C, Salvato R, Schaurich A
EPI_ISL_942929	Instituto de Diagnostico y Referencia Epidemiologicos INDRE_RNLSP	Instituto de Diagnostico y Referencia Epidemiologicos (INDRE)	Claudia Wong-Arambula, Abril Rodriguez-Maldonado, Fabiola Garces-Ayala, Natividad Cruz-Ortiz, Tatiana Nunez-Garcia, Gisela Barrera-Badillo, Lucia Hernandez-Rivas, Irma Lopez-Martinez, Ernesto Ramirez-Gonzalez.
EPI_ISL_942930, EPI_ISL_942931	Central Laboratory of Public Health of Rio Grande do Sul (Lacen-RS)	State Center for Health Surveillance of the Health Department of the State of Rio Grande do Sul (CEVS/SES-RS)	Barcellos R, Campos A, Crescente L, Da Silva A, Dornelles C, Fonseca V, Garay L, Godinho F, Gonzalez A, Gregianini T, Molina C, Salvato R, Schaurich A
EPI_ISL_943574, EPI_ISL_943575, EPI_ISL_943576, EPI_ISL_943577, EPI_ISL_943581, EPI_ISL_943582, EPI_ISL_943584, EPI_ISL_943586, EPI_ISL_943587, EPI_ISL_943588, EPI_ISL_943590			
see above	Central Laboratory of Public Health of Rio Grande do Sul (Lacen-RS)	State Center for Health Surveillance of the Health Department of the State of Rio Grande do Sul (CEVS/SES-RS)	Aline Campos, Amanda da Silva, Anelise Schaurich, Claudia Dornelles, Cynthia Molina, Fernanda Godinho, Lara Crescente, Leticia Garay, Regina Barcellos, Richard Salvato, Tatiana Gregianini, Vagner Fonseca
EPI_ISL_943988	LACEN do Estado de Goias	Instituto Adolfo Lutz, Interdiciplinary Procedures Center, Strategic Laboratory	Claudio Tavares Sacchi, Claudia Regina Gonçalves, Erica Valessa Ramos Gomes, Karoline Rodrigues Campos
EPI_ISL_954810	Hospital General Universitario de Ciudad Real	Instituto de Salud Carlos III	Iglesias-Caballero, M. Camarero, S. Sandonís,V. Vázquez, S. Pozo, F. Casas, I. Jiménez, P. Zaballos, A. Monzón, S. Varona, S. Cuesta, I. Illescas, S.
EPI_ISL_955142	University of Sarajevo, Veterinary Faculty, Laboratory for Molecular Diagnostic and Research Laboratory	University of Sarajevo, Veterinary Faculty, Laboratory for Molecular Diagnostic and Research Laboratory	Goleti T., Goleti Š., Softi A., Ali-Šeho A., Šabi E., Jaži A., Nicevi M., Hodži A., Terzi I.
EPI_ISL_955144	University of Sarajevo, Veterinary Faculty, Laboratory for Molecular Diagnostic and Research Laboratory	University of Sarajevo, Veterinary Faculty, Laboratory for Molecular Diagnostic and Research Laboratory	Goleti T., Goleti Š., Softi A., Ali-Šeho A., Hodži A., Šabi E., Terzi I. Jaži A., Nicevi M.
EPI_ISL_956271	Adi Husada Undaan Hospital	Institute of Tropical Disease, Universitas Airlangga	Aldise M Nastri, Jezzy R Dewantari, Rima R Prasetya, Krisnoadi Rahardjo, Irawati Marga, Gatot Soegiarto, Laksmi Wulandari, Retno A Setyoningrum, Resti Yudhawati, Yohko K Shimizu, Mitsuhiro Nishimura, Soetjipto, Yasuko Mori, Maria I Lusida, Kazufumi Shimizu
EPI_ISL_956273	Universitas Airlangga Hospital	Institute of Tropical Disease, Universitas Airlangga	Jezzy R Dewantari, Rima R Prasetya, Krisnoadi Rahardjo, Aldise M Nastri, Nasronudin, Gatot Soegiarto, Laksmi Wulandari, Resti Yudhawati, Soetjipto, Yasuko Mori, Maria I Lusida, Kazufumi Shimizu
EPI_ISL_956274	RSUD Sidoarjo	Institute of Tropical Disease, Universitas Airlangga	Rima R Prasetya, Krisnoadi Rahardjo, Aldise M Nastri, Jezzy R Dewantari, Atok Irawan, Gatot Soegiarto, Laksmi Wulandari, Resti Yudhawati, Soetjipto, Yasuko Mori, Maria I Lusida, Kazufumi Shimizu
EPI_ISL_956276	Mitra Keluarga Hospital Waru	Institute of Tropical Disease, Universitas Airlangga	Maria I Lusida, Krisnoadi Rahardjo, Aldise M Nastri, Jezzy R Dewantari, Rima R Prasetya, Christina Dian Anggraeni, Gatot Soegiarto, Laksmi Wulandari, Resti Yudhawati, Soetjipto, Yasuko Mori, Kazufumi Shimizu
EPI_ISL_956277	Paru Hospital	Institute of Tropical Disease, Universitas Airlangga	Krisnoadi Rahardjo, Aldise M Nastri, Jezzy R Dewantari, Rima R Prasetya, Dyah Retno, Gatot Soegiarto, Laksmi Wulandari, Resti Yudhawati, Soetjipto, Yasuko Mori, Maria I Lusida, Kazufumi Shimizu
EPI_ISL_956278	Siti Khodijah Hospital	Institute of Tropical Disease, Universitas Airlangga	Aldise M Nastri, Jezzy R Dewantari, Rima R Prasetya, Krisnoadi Rahardjo, Muhammad Hamdan, Gatot Soegiarto, Laksmi Wulandari, Resti Yudhawati, Soetjipto, Yasuko Mori, Maria I Lusida, Kazufumi Shimizu
EPI_ISL_956309	RSUD Sidoarjo	Institute of Tropical Disease, Universitas Airlangga	Rima R Prasetya, Krisnoadi Rahardjo, Aldise M Nastri, Jezzy R Dewantari, Atok Irawan, Gatot Soegiarto, Laksmi Wulandari, Resti Yudhawati, Yasuko Mori, Soetjipto, Kazufumi Shimizu, Maria I Lusida
EPI_ISL_956310	RSUD Dr. M. Soewandhie	Institute of Tropical Disease, Universitas Airlangga	Aldise M Nastri, Jezzy R Dewantari, Rima R Prasetya, Krisnoadi Rahardjo, Febria Rachmanita, Gatot Soegiarto, Laksmi Wulandari, Resti Yudhawati, Yasuko Mori, Soetjipto, Kazufumi Shimizu, Maria I Lusida
EPI_ISL_956311	Islamic Hospital	Institute of Tropical Disease, Universitas Airlangga	Kazufumi Shimizu, Krisnoadi Rahardjo, Aldise M Nastri, Jezzy R Dewantari, Rima R Prasetya, Dodo Anondo, Gatot Soegiarto, Laksmi Wulandari, Resti Yudhawati, Yasuko Mori, Soetjipto, Maria I Lusida
EPI_ISL_959277	National Influenza Center, Virology Department	National Influenza Center	J Yavarian,K Sadeghi, NZ Shafiei Jandaghi, V Salimi, A Nejati, N Ghavvami,F Ajaminejad and T Mokhtari Azad
EPI_ISL_959278	National Influenza Center, Virology Department	National Influenza Center	A Nejati, J Yavarian,K Sadeghi, NZ Shafiei Jandaghi, V Salimi, N Ghavvami,F Ajaminejad and T Mokhtari Azad
EPI_ISL_959281	National Influenza Center, Virology Department	National Influenza Center	NZ Shafiei Jandaghi, V Salimi, A Nejati, K Sadeghi, J Yavarian, F Ajaminejad,N Ghavvami and T Mokhtari Azad

EPI_ISL_959282	National Influenza Center, Virology Department	National Influenza Center	J Yavarian, NZ Shafiei Jandaghi, V Salimi, A Nejati, K Sadeghi, F Ajaminejad,N Ghavvami and T Mokhtari Azad
EPI_ISL_961761, EPI_ISL_961771, EPI_ISL_961780	Laboratorio de Infectología, Servicio de Infectología, Hospital Universitario Dr. José Eleuterio González - Universidad Autónoma de Nuevo León	Laboratorio de Infectología Molecular, Departamento de Bioquímica y Medicina Molecular,Facultad de Medicina - Universidad Autónoma de Nuevo León	Kame A. Galán-Huerta, María F. Herrera-Saldivar, Natalia Martínez-Acuña, Sonia A. Lozano-Sepúlveda, Daniel Arellanos-Soto, Ana M. Rivas-Estilla, Paola Bocanegra-Ibarias, Samantha M. Flores-Treviño, Elvira Garza-González, Eduardo Perez-Alba, Laura Nuzzolo-Shihadeh, Adrian Camacho-Ortiz
EPI_ISL_969509, EPI_ISL_969740	Indian Council of Medical Research-National Institute of Virology, Pune	Indian Council of Medical Research-National Institute of Virology, Maximum Containment Laboratory	Pragya D. Yadav, Anita Shete-Aich, Dimpal A. Nyayanit, Abhinendra Kumar, Savita Patil, Triparna D. Majumdar
EPI_ISL_971451	Cell culture Unit at CV-MIT belonging to HIMMV	Functional Genomic Platform UATRS-biology, CNRST	Nadia Touil, Abderrazzak Rfak, Mouhssine Hemlali, Sanaa ALAOUI-Amine, Marouane MELLOUL, Mly Abdelaziz ELALAOUI, Hicham Elannaz, Amine Idriss Lahlou, Mostafa ELOUENNASS, Khalid ENNIBI, Elmostafa EL FAHIME
EPI_ISL_977471	Instituto Adolfo Lutz - Regional de Presidente Prudente	Instituto Adolfo Lutz, Interdiciplinary Procedures Center, Strategic Laboratory	Claudio Tavares Sacchi, Claudia Regina Gonçalves, Erica Valessa Ramos Gomes, Karoline Rodrigues Campos
EPI_ISL_977472, EPI_ISL_977473, EPI_ISL_977474	Instituto Adolfo Lutz Central	Instituto Adolfo Lutz, Interdiciplinary Procedures Center, Strategic Laboratory	Claudio Tavares Sacchi, Claudia Regina Gonçalves, Erica Valessa Ramos Gomes, Karoline Rodrigues Campos
EPI_ISL_977475, EPI_ISL_977478	Instituto Adolfo Lutz - Regional de Presidente Prudente	Instituto Adolfo Lutz, Interdiciplinary Procedures Center, Strategic Laboratory	Claudio Tavares Sacchi, Claudia Regina Gonçalves, Erica Valessa Ramos Gomes, Karoline Rodrigues Campos
EPI_ISL_977479	Lab Loc - Itapecerica da Serra	Instituto Adolfo Lutz, Interdiciplinary Procedures Center, Strategic Laboratory	Claudio Tavares Sacchi, Claudia Regina Gonçalves, Erica Valessa Ramos Gomes, Karoline Rodrigues Campos
EPI_ISL_977480, EPI_ISL_977481	Instituto Adolfo Lutz - Regional de Presidente Prudente	Instituto Adolfo Lutz, Interdiciplinary Procedures Center, Strategic Laboratory	Claudio Tavares Sacchi, Claudia Regina Gonçalves, Erica Valessa Ramos Gomes, Karoline Rodrigues Campos
EPI_ISL_977483, EPI_ISL_977484	Instituto Adolfo Lutz Central	Instituto Adolfo Lutz, Interdiciplinary Procedures Center, Strategic Laboratory	Claudio Tavares Sacchi, Claudia Regina Gonçalves, Erica Valessa Ramos Gomes, Karoline Rodrigues Campos
EPI_ISL_977485	Instituto Adolfo Lutz - Regional de Presidente Prudente	Instituto Adolfo Lutz, Interdiciplinary Procedures Center, Strategic Laboratory	Claudio Tavares Sacchi, Claudia Regina Gonçalves, Erica Valessa Ramos Gomes, Karoline Rodrigues Campos
EPI_ISL_977486	Instituto Adolfo Lutz - Regional de Santo Andre	Instituto Adolfo Lutz, Interdiciplinary Procedures Center, Strategic Laboratory	Claudio Tavares Sacchi, Claudia Regina Gonçalves, Erica Valessa Ramos Gomes, Karoline Rodrigues Campos
EPI_ISL_977488	Instituto Adolfo Lutz - Regional de Presidente Prudente	Instituto Adolfo Lutz, Interdiciplinary Procedures Center, Strategic Laboratory	Claudio Tavares Sacchi, Claudia Regina Gonçalves, Erica Valessa Ramos Gomes, Karoline Rodrigues Campos
EPI_ISL_979970, EPI_ISL_979971, EPI_ISL_979972, EPI_ISL_979973	Department of Respiratory and other Viral Infections of L.V.Gromashevsky Institute of Epidemiology & Infectious Diseases NAMS of Ukrain	Department of Respiratory and other Viral Infections of L.V.Gromashevsky Institute of Epidemiology & Infectious Diseases NAMS of Ukrain, JSC "Farmak"	Alla Mironenko, Andriy Goy, Ihor Kravchuk, Ludmyla Bolotova, Larysa Radchenko, Nataliia Teteriuk
EPI_ISL_984242	Instituto Adolfo Lutz Central	Instituto Adolfo Lutz, Interdiciplinary Procedures Center, Strategic Laboratory	Claudio Tavares Sacchi, Claudia Regina Gonçalves, Erica Valessa Ramos Gomes, Karoline Rodrigues Campos
EPI_ISL_984243, EPI_ISL_984244	Instituto Adolfo Lutz - Regional de Marilia	Instituto Adolfo Lutz, Interdiciplinary Procedures Center, Strategic Laboratory	Claudio Tavares Sacchi, Claudia Regina Gonçalves, Erica Valessa Ramos Gomes, Karoline Rodrigues Campos
EPI_ISL_984246	Instituto Adolfo Lutz Central	Instituto Adolfo Lutz, Interdiciplinary Procedures Center, Strategic Laboratory	Claudio Tavares Sacchi, Claudia Regina Gonçalves, Erica Valessa Ramos Gomes, Karoline Rodrigues Campos
EPI_ISL_985170	Instituto Adolfo Lutz - Regional de Presidente Prudente	Instituto Adolfo Lutz, Interdiciplinary Procedures Center, Strategic Laboratory	Claudio Tavares Sacchi, Claudia Regina Gonçalves, Erica Valessa Ramos Gomes, Karoline Rodrigues Campos
EPI_ISL_985171, EPI_ISL_985172, EPI_ISL_985173, EPI_ISL_985174	Instituto Adolfo Lutz - Regional de Taubate	Instituto Adolfo Lutz, Interdiciplinary Procedures Center, Strategic Laboratory	Claudio Tavares Sacchi, Claudia Regina Gonçalves, Erica Valessa Ramos Gomes, Karoline Rodrigues Campos
EPI_ISL_985175, EPI_ISL_985176, EPI_ISL_985177	Instituto Adolfo Lutz Central	Instituto Adolfo Lutz, Interdiciplinary Procedures Center, Strategic Laboratory	Claudio Tavares Sacchi, Claudia Regina Gonçalves, Erica Valessa Ramos Gomes, Karoline Rodrigues Campos
EPI_ISL_985178	Lab Loc - Itapecerica da Serra	Instituto Adolfo Lutz, Interdiciplinary Procedures Center, Strategic Laboratory	Claudio Tavares Sacchi, Claudia Regina Gonçalves, Erica Valessa Ramos Gomes, Karoline Rodrigues Campos

We gratefully acknowledge the following Authors from the Originating laboratories responsible for obtaining the specimens, as well as the Submitting laboratories where the genome data were generated and shared via GISAID, on which this research is based.

All Submitters of data may be contacted directly via www.gisaid.org

Authors are sorted alphabetically.

Accession ID	Originating Laboratory	Submitting Laboratory	Authors
EPI_ISL_1001050	Centre Pasteur du Cameroun	Institut Pasteur de Dakar	Njouom Richard
EPI_ISL_1001353	hopital	National Reference Center for Viruses of Respiratory Infections, Institut Pasteur, Paris	Marion Barbet, Sylvie Behillil, Méline Bizard, Angela Brisebarre, Camille Capel, Etienne Simon-Lorière, Vincent Enouf, Maud Vanpeene, Sylvie van der Werf,Stoessel-Thouvenin Patricia
EPI_ISL_1001376, EPI_ISL_1001377, EPI_ISL_1001378, EPI_ISL_1001379, EPI_ISL_1001380	Labo Analyses Med	National Reference Center for Viruses of Respiratory Infections, Institut Pasteur, Paris	Marion Barbet, Sylvie Behillil, Méline Bizard, Angela Brisebarre, Camille Capel, Etienne Simon-Lorière, Vincent Enouf, Maud Vanpeene, Sylvie van der Werf,Besson J
EPI_ISL_1001456	Hopital	National Reference Center for Viruses of Respiratory Infections, Institut Pasteur, Paris	Marion Barbet, Sylvie Behillil, Méline Bizard, Angela Brisebarre, Camille Capel, Etienne Simon-Lorière, Vincent Enouf, Maud Vanpeene, Sylvie van der Werf,Ducancelle Alexandra
EPI_ISL_1001458	Gorgas memorial Institute for Health Studies	Gorgas memorial Institute for Health Studies	Díaz Y, Castillo D, Moreno B, Castillo M,González C, Gondola J, Moreno A, Pitti Y, Chavarria O, Franco D, Saenz L, Gaitan M, Arauz D, Martinez AA, Lopez-Verges S.
EPI_ISL_1005696	RSUD Dr. Tjitrowardojo	Genetics Working Group (Pokja Genetik) Faculty of Medicine, Public Health and Nursing Universitas Gadjah Mada (FK-KMK UGM); Disease Investigation Center Wates Ministry of Agriculture Indonesia; Department of Microbiology FK-KMK UGM; Laboratorium Diagnostik Yayasan Tahija World Mosquito Program (WMP) Yogyakarta Center for Tropical Medicine FK-KMK UGM; Integrated Research Center FK-KMK UGM; Department of Computer Science and Electronics FMIPA UGM; Balai Besar Teknik Kesehatan Lingkungan dan Pengendalian Penyakit (BBTKLPP) Yogyakarta	Gunadi, Hendra Wibawa, Marcellus, Mohamad S. Hakim, Edwin W. Daniwijaya, Ludhang P. Rizki, Endah Supriyati, Eggi Arguni, Titik Nuryastuti, Tri Wibawa, Dwi AA Nugrahaningsih, Afiahayati, Siswanto, Kristy Iskandar, Nungki Anggorowati, Irene, Indaryati, Havid Setyawan, Arry Setyowati, Chusni Mubarakh, Try Nirmala Sari, Susan Simanjaya, Alvin Santoso Kalim
EPI_ISL_1005697	RSUD Dr. Tjitrowardojo	Genetics Working Group (Pokja Genetik) Faculty of Medicine, Public Health and Nursing Universitas Gadjah Mada (FK-KMK UGM); Disease Investigation Center Wates Ministry of Agriculture Indonesia; Department of Microbiology FK-KMK UGM; Laboratorium Diagnostik Yayasan Tahija World Mosquito Program (WMP) Yogyakarta Center for Tropical Medicine FK-KMK UGM; Integrated Research Center FK-KMK UGM; Department of Computer Science and Electronics FMIPA UGM; Balai Besar Teknik Kesehatan Lingkungan dan Pengendalian Penyakit (BBTKLPP) Yogyakarta	Gunadi, Hendra Wibawa, Marcellus, Mohamad S. Hakim, Edwin W. Daniwijaya, Ludhang P. Rizki, Endah Supriyati, Eggi Arguni, Titik Nuryastuti, Tri Wibawa, Dwi AA Nugrahaningsih, Afiahayati, Siswanto, Kristy Iskandar, Nungki Anggorowati, Irene, Indaryati, Havid Setyawan, Arry Setyowati, Chusni Mubarakh, Try Nirmala Sari, Kemala Athollah, Dyah Ayu Puspitarani
EPI_ISL_1005698	RSUD Dr. Tjitrowardojo	Genetics Working Group (Pokja Genetik) Faculty of Medicine, Public Health and Nursing Universitas Gadjah Mada (FK-KMK UGM); Disease Investigation Center Wates Ministry of Agriculture Indonesia; Department of Microbiology FK-KMK UGM; Laboratorium Diagnostik Yayasan Tahija World Mosquito Program (WMP) Yogyakarta Center for Tropical Medicine FK-KMK UGM; Integrated Research Center FK-KMK UGM; Department of Computer Science and Electronics FMIPA UGM; Balai Besar Teknik Kesehatan Lingkungan dan Pengendalian Penyakit (BBTKLPP) Yogyakarta	Gunadi, Hendra Wibawa, Marcellus, Mohamad S. Hakim, Edwin W. Daniwijaya, Ludhang P. Rizki, Endah Supriyati, Eggi Arguni, Titik Nuryastuti, Tri Wibawa, Dwi AA Nugrahaningsih, Afiahayati, Siswanto, Kristy Iskandar, Nungki Anggorowati, Irene, Indaryati, Havid Setyawan, Arry Setyowati, Chusni Mubarakh, Try Nirmala Sari, Aditya Rifqi Fauzi, Fadil Fahri
EPI_ISL_1007677, EPI_ISL_1007678, EPI_ISL_1007679	Department of Internal Medicine, College of Medicine, Chosun University	Department of Internal Medicine, College of Medicine, Chosun University	Dong-Min Kim
EPI_ISL_1008703	Presidio di Brindisi Di Summa - Perrino	Istituto Zooprofilattico Sperimentale della Puglia e della Basilicata	Parisi A., Bianco A., Capozzi L., Del Sambro L., Simone D., Chironna M., Loconsole D., Sallustio A., Ridolfi D.
EPI_ISL_1010728, EPI_ISL_1010729, EPI_ISL_1010730, EPI_ISL_1010731	Department of Laboratory Medicine, National Taiwan University Hospital	Microbial Genomics Core Lab, National Taiwan University Centers of Genomic and Precision Medicine	Shiou-Hwei Yeh, You-Yu Lin, Ya-Yun Lai, Chiao-Ling Li, Shan-Chwen Chang, Pei-Jer Chen, Sui-Yuan Chang
EPI_ISL_1013094	Imelda Hospital	Imelda Hospital	Johan Frans, Hanne Valgaeren, Dagmar Obbels
EPI_ISL_1013099, EPI_ISL_1013100, EPI_ISL_1013101, EPI_ISL_1013102, EPI_ISL_1013103, EPI_ISL_1013104, EPI_ISL_1013105, EPI_ISL_1013106, EPI_ISL_1013107, EPI_ISL_1013108, EPI_ISL_1013109, EPI_ISL_1013110, EPI_ISL_1013111, EPI_ISL_1013112, EPI_ISL_1013113, EPI_ISL_1013114, EPI_ISL_1013115, EPI_ISL_1013116, EPI_ISL_1013117, EPI_ISL_1013118, EPI_ISL_1013119, EPI_ISL_1013120, EPI_ISL_1013121, EPI_ISL_1013122, EPI_ISL_1013123, EPI_ISL_1013124, EPI_ISL_1013125, EPI_ISL_1013126, EPI_ISL_1013127, EPI_ISL_1013128, EPI_ISL_1013129, EPI_ISL_1013130, EPI_ISL_1013131, EPI_ISL_1013132, EPI_ISL_1013133, EPI_ISL_1013134, EPI_ISL_1013135, EPI_ISL_1013136, EPI_ISL_1013137, EPI_ISL_1013138, EPI_ISL_1013139, EPI_ISL_1013140, EPI_ISL_1013141, EPI_ISL_1013142, EPI_ISL_1013143, EPI_ISL_1013144, EPI_ISL_1013145, EPI_ISL_1013146, EPI_ISL_1013147, EPI_ISL_1013148, EPI_ISL_1013149, EPI_ISL_1013150, EPI_ISL_1013151, EPI_ISL_1013152, EPI_ISL_1013153, EPI_ISL_1013154, EPI_ISL_1013155, EPI_ISL_1013156, EPI_ISL_1013157, EPI_ISL_1013158, EPI_ISL_1013159, EPI_ISL_1013160, EPI_ISL_1013161, EPI_ISL_1013162, EPI_ISL_1013163, EPI_ISL_1013164, EPI_ISL_1013165, EPI_ISL_1013166, EPI_ISL_1013167, EPI_ISL_1013168, EPI_ISL_1013169, EPI_ISL_1013170, EPI_ISL_1013171, EPI_ISL_1013172, EPI_ISL_1013173, EPI_ISL_1013174, EPI_ISL_1013175, EPI_ISL_1013176, EPI_ISL_1013177, EPI_ISL_1013178, EPI_ISL_1013179, EPI_ISL_1013180, EPI_ISL_1013181, EPI_ISL_1013182, EPI_ISL_1013183, EPI_ISL_1013184, EPI_ISL_1013185, EPI_ISL_1013186, EPI_ISL_1013187, EPI_ISL_1013188, EPI_ISL_1013189, EPI_ISL_1013190, EPI_ISL_1013191, EPI_ISL_1013192, EPI_ISL_1013193, EPI_ISL_1013194, EPI_ISL_1013195, EPI_ISL_1013196, EPI_ISL_1013197, EPI_ISL_1013198, EPI_ISL_1013199, EPI_ISL_1013200, EPI_ISL_1013201, EPI_ISL_1013202, EPI_ISL_1013203, EPI_ISL_1013204, EPI_ISL_1013205, EPI_ISL_1013206, EPI_ISL_1013207, EPI_ISL_1013208			
see above	CH de Mayotte	National Reference Center for Viruses of Respiratory Infections, Institut Pasteur, Paris	Marion Barbet, Sylvie Behillil, Méline Bizard, Angela Brisebarre, Camille Capel, Etienne Simon-Lorière, Vincent Enouf, Maud Vanpeene, Sylvie van der Werf,Combe Patrice
EPI_ISL_1013437	Institut Pasteur de Guadeloupe	National Reference Center for Viruses of Respiratory Infections, Institut Pasteur, Paris	Marion Barbet, Sylvie Behillil, Méline Bizard, Angela Brisebarre, Camille Capel, Etienne Simon-Lorière, Vincent Enouf, Maud Vanpeene, Sylvie van der Werf,Talarmin Antoine
EPI_ISL_1013444	Laboratoire associé au CNR des Virus Influenzae	National Reference Center for Viruses of Respiratory Infections, Institut Pasteur, Paris	Marion Barbet, Sylvie Behillil, Méline Bizard, Angela Brisebarre, Camille Capel, Etienne Simon-Lorière, Vincent Enouf, Maud Vanpeene, Sylvie van der Werf,Rousset (Guy) Dominique
EPI_ISL_1013779, EPI_ISL_1014102, EPI_ISL_1014238	Zurita & Zurita Laboratorios	Zurita & Zurita Laboratorios	Jeannete Zurita, Camilo Zurita-Salinas, Gabriela Sevillano
EPI_ISL_1014676	National Influenza Center, Virology Department	National Influenza Center	V Salimi,NZ Shafiei Jandaghi, J Yavarian, A Nejati, K Sadeghi, N Ghavvami,F Ajaminejad and T Mokhtari Azad

EPI_ISL_1014685	National Influenza Center, Virology Department	National Influenza Center	K Sadeghi, A Nejati, J Yavarian, NZ Shafiei Jandaghi, V Salimi, F Ajaminejad,N Ghavvami and T Mokhtari Azad
EPI_ISL_1014686	National Influenza Center, Virology Department	National Influenza Center	NZ Shafiei Jandaghi, V Salimi, A Nejati, K Sadeghi, J Yavarian, N Ghavvami,F Ajaminejad and T Mokhtari Azad
EPI_ISL_1016690	University of Sarajevo, Veterinary Faculty, Laboratory for Molecular Diagnostic and Research Laboratory	University of Sarajevo, Veterinary Faculty, Laboratory for Molecular Diagnostic and Research Laboratory	Goletic T., Goletic S., Softic A., Alic-Seho A., Sabic E., Terzic I., Jazic A., Nicevic M., Hodzic A.
EPI_ISL_1016822	University of Sarajevo, Veterinary Faculty, Laboratory for Molecular Diagnostic and Research Laboratory	University of Sarajevo, Veterinary Faculty, Laboratory for Molecular Diagnostic and Research Laboratory	Goletic T., Goletic S., Softic A., Alic-Seho A., Hodzic A., Sabic E., Nicevic M., Terzic I., Jazic A.
EPI_ISL_1018287, EPI_ISL_1018289, EPI_ISL_1018290, EPI_ISL_1018291, EPI_ISL_1018292, EPI_ISL_1018294, EPI_ISL_1018295, EPI_ISL_1018296, EPI_ISL_1018297, EPI_ISL_1018299, EPI_ISL_1018300, EPI_ISL_1018303, EPI_ISL_1018305, EPI_ISL_1018306, EPI_ISL_1018307, EPI_ISL_1018308, EPI_ISL_1018310, EPI_ISL_1018312, EPI_ISL_1018313, EPI_ISL_1018315, EPI_ISL_1018316, EPI_ISL_1018318, EPI_ISL_1018319, EPI_ISL_1018321, EPI_ISL_1018322, EPI_ISL_1018323, EPI_ISL_1018324, EPI_ISL_1018325, EPI_ISL_1018326, EPI_ISL_1018327, EPI_ISL_1018328, EPI_ISL_1019637, EPI_ISL_1019642, EPI_ISL_1019645, EPI_ISL_1019647, EPI_ISL_1019650, EPI_ISL_1019653, EPI_ISL_1019655, EPI_ISL_1019658, EPI_ISL_1019660, EPI_ISL_1019663, EPI_ISL_1019665, EPI_ISL_1019668, EPI_ISL_1019671, EPI_ISL_1019674, EPI_ISL_1019678, EPI_ISL_1019681, EPI_ISL_1019683, EPI_ISL_1019688, EPI_ISL_1019693, EPI_ISL_1019699, EPI_ISL_1019702, EPI_ISL_1019705, EPI_ISL_1019720, EPI_ISL_1019724, EPI_ISL_1019726, EPI_ISL_1019737, EPI_ISL_1019739, EPI_ISL_1019742, EPI_ISL_1019748, EPI_ISL_1019750, EPI_ISL_1019753, EPI_ISL_1019756, EPI_ISL_1019762, EPI_ISL_1019767, EPI_ISL_1019775, EPI_ISL_1019778, EPI_ISL_1019783, EPI_ISL_1019785, EPI_ISL_1019788, EPI_ISL_1019790, EPI_ISL_1019793, EPI_ISL_1019795, EPI_ISL_1019798, EPI_ISL_1019800, EPI_ISL_1019803, EPI_ISL_1019805, EPI_ISL_1019810, EPI_ISL_1019813, EPI_ISL_1019815, EPI_ISL_1019828, EPI_ISL_1019838, EPI_ISL_1019846, EPI_ISL_1019849, EPI_ISL_1019851, EPI_ISL_1019853, EPI_ISL_1019855, EPI_ISL_1019858, EPI_ISL_1019861, EPI_ISL_1019863, EPI_ISL_1019866, EPI_ISL_1019869, EPI_ISL_1019872, EPI_ISL_1019875, EPI_ISL_1019877, EPI_ISL_1019880, EPI_ISL_1019882, EPI_ISL_1019885, EPI_ISL_1019888, EPI_ISL_1019890, EPI_ISL_1019893, EPI_ISL_1020022, EPI_ISL_1020023, EPI_ISL_1020024, EPI_ISL_1020025, EPI_ISL_1020026, EPI_ISL_1020027, EPI_ISL_1020028, EPI_ISL_1020029, EPI_ISL_1020030, EPI_ISL_1020031, EPI_ISL_1020032, EPI_ISL_1020033, EPI_ISL_1020034, EPI_ISL_1020035, EPI_ISL_1020036, EPI_ISL_1020037, EPI_ISL_1020038, EPI_ISL_1020039, EPI_ISL_1020040, EPI_ISL_1020041, EPI_ISL_1020042, EPI_ISL_1020043, EPI_ISL_1020044, EPI_ISL_1020045, EPI_ISL_1020046, EPI_ISL_1020047, EPI_ISL_1020048, EPI_ISL_1020049, EPI_ISL_1020050, EPI_ISL_1020051, EPI_ISL_1020052, EPI_ISL_1020053, EPI_ISL_1020054, EPI_ISL_1020055, EPI_ISL_1020056, EPI_ISL_1020058, EPI_ISL_1020059, EPI_ISL_1020060, EPI_ISL_1020061, EPI_ISL_1020062, EPI_ISL_1020063, EPI_ISL_1020064, EPI_ISL_1020065, EPI_ISL_1020066, EPI_ISL_1020067, EPI_ISL_1020068, EPI_ISL_1020069, EPI_ISL_1020070, EPI_ISL_1020071, EPI_ISL_1020072, EPI_ISL_1020073, EPI_ISL_1020074, EPI_ISL_1020075, EPI_ISL_1020076, EPI_ISL_1020077, EPI_ISL_1020078, EPI_ISL_1020079, EPI_ISL_1020080, EPI_ISL_1020081, EPI_ISL_1020083, EPI_ISL_1020084, EPI_ISL_1020086, EPI_ISL_1020087, EPI_ISL_1020088, EPI_ISL_1020089, EPI_ISL_1020091, EPI_ISL_1020092, EPI_ISL_1020093, EPI_ISL_1020094, EPI_ISL_1020095, EPI_ISL_1020096, EPI_ISL_1020098, EPI_ISL_1020099, EPI_ISL_1020101, EPI_ISL_1020102, EPI_ISL_1020104, EPI_ISL_1020105, EPI_ISL_1020106, EPI_ISL_1020107, EPI_ISL_1020108, EPI_ISL_1020109, EPI_ISL_1020110, EPI_ISL_1020111, EPI_ISL_1020112, EPI_ISL_1020113, EPI_ISL_1020114, EPI_ISL_1020115, EPI_ISL_1020116, EPI_ISL_1020117, EPI_ISL_1020118, EPI_ISL_1020119, EPI_ISL_1020120			
see above	Department of Health Technology and Informatics, The Hong Kong Polytechnic University	Department of Health Technology and Informatics, The Hong Kong Polytechnic University	Gilman Kit-Hang Siu, Lam-Kwong Lee, Kenneth Siu-Sing Leung, Jake Siu-Lun Leung, Timothy Ting-Leung Ng, Chloe Toi-Mei Chan, Kingsley King-Gee Tam, Hiu-Yin Lao, Denise Sze-Hang Wong, Alan Ka-Lun Wu, Miranda Chong-Yee Yau, Yvette Wai-Man Lai, Kitty Sau-Chun Fung, Sandy Ka-Yee Chau, Barry Kin-Chung Wong, Wing-Kin To, Kristine Luk, Alex Yat-Man Ho, Tak-Lun Que, Kam-Tong Yip, Wing Cheong Yam, David Ho-Keung Shum, Shea Ping Yip
EPI_ISL_1020192	Melania Hospital, Bagor , west Java	Biosafety Level-3 Laboratory, Indonesian Institute of Sciences (LIPI)	Anik Budhi Dharmayanthi, Ade Andriani, Hariyatun, Asep M. Ridwanuloh, Syam Budi Iryanto, Eko Wahyu Putro, Gita Syahputra, Susanti, Inswasti Cahyani, Matthew W. Loose, Mohammad Ilyas, Wien Kusharyoto
EPI_ISL_1020194	UMMI Hospital, Bogor, West Java	Biosafety Level-3 Laboratory, Indonesian Institute of Sciences (LIPI)	Anggia Prasetyoputri, Isa Nuryana, Sri Swasthikawati, Sugiyono Saputra, Syam Budi Iryanto, Gita Syahputra, Ratih Asmana Ningrum
EPI_ISL_1020195	Melania Hospital, Bogor , West Java	Biosafety Level-3 Laboratory, Indonesian Institute of Sciences (LIPI)	Anggia Prasetyoputri, Erwin Hasrianda, Herjuno A. Nugroho, Nova Dhilla Yanthi, Fahrurrozi, Syam Budi Iryanto, Gita Syahputra, Ratih Asmana Ningrum
EPI_ISL_1020197	Dinkes, Bogor, West Java	Biosafety Level-3 Laboratory, Indonesian Institute of Sciences (LIPI)	Anik Budhi Dharmayanthi, Ade Andriani, Hariyatun, Asep M. Ridwanuloh, Syam Budi Iryanto, Eko Wahyu Putro, Gita Syahputra, Susanti, Inswasti Cahyani, Matthew W. Loose, Mohammad Ilyas, Wien Kusharyoto
EPI_ISL_1020198	RS Trimitra, Bogor, West java	Biosafety Level-3 Laboratory, Indonesian Institute of Sciences (LIPI)	Sri Swasthikawati, Linda Sukmarini, Idris, Masrukhin, Alfi T. Fathurahman, Eko Wahyu Putro, Ratih Asmana Ningrum, Sugiyono Saputra
EPI_ISL_1020203	Klinik Apotik Dien, Jakarta	Biosafety Level-3 Laboratory, Indonesian Institute of Sciences (LIPI)	Anik Budhi Dharmayanthi, Ade Andriani, Hariyatun, Asep M. Ridwanuloh, Syam Budi Iryanto, Eko Wahyu Putro, Gita Syahputra, Susanti, Inswasti Cahyani, Matthew W. Loose, Mohammad Ilyas, Wien Kusharyoto
EPI_ISL_1020206	Qadr Hospital, Tangerang, Banten	Biosafety Level-3 Laboratory, Indonesian Institute of Sciences (LIPI)	Anggia Prasetyoputri, Isa Nuryana, Sri Swasthikawati, Sugiyono Saputra, Syam Budi Iryanto, Eko Wahyu Putro, Gita Syahputra, Ari Sulistyo Wulandari, Ratih Asmana Ningrum
EPI_ISL_1020207	AZ Klina	AZ Klina	Dr. C. Vael
EPI_ISL_1020214, EPI_ISL_1020215, EPI_ISL_1020216, EPI_ISL_1020217, EPI_ISL_1020218, EPI_ISL_1020219, EPI_ISL_1020220, EPI_ISL_1020221, EPI_ISL_1020222, EPI_ISL_1020223, EPI_ISL_1020224, EPI_ISL_1020225, EPI_ISL_1020226, EPI_ISL_1020227, EPI_ISL_1020228, EPI_ISL_1020229, EPI_ISL_1020230, EPI_ISL_1020231, EPI_ISL_1020232, EPI_ISL_1020233, EPI_ISL_1020234, EPI_ISL_1020235, EPI_ISL_1020236, EPI_ISL_1020237, EPI_ISL_1020238, EPI_ISL_1020239, EPI_ISL_1020240, EPI_ISL_1020241, EPI_ISL_1020242, EPI_ISL_1020243, EPI_ISL_1020244, EPI_ISL_1020245, EPI_ISL_1020246, EPI_ISL_1020247, EPI_ISL_1020248, EPI_ISL_1020249, EPI_ISL_1020250, EPI_ISL_1020251, EPI_ISL_1020252, EPI_ISL_1020253, EPI_ISL_1020254, EPI_ISL_1020255, EPI_ISL_1020256, EPI_ISL_1020257, EPI_ISL_1020258, EPI_ISL_1020259, EPI_ISL_1020260, EPI_ISL_1020261, EPI_ISL_1020262, EPI_ISL_1020263, EPI_ISL_1020264, EPI_ISL_1020265, EPI_ISL_1020266, EPI_ISL_1020267, EPI_ISL_1020268, EPI_ISL_1020269, EPI_ISL_1020270, EPI_ISL_1020271, EPI_ISL_1020272, EPI_ISL_1020273, EPI_ISL_1020274, EPI_ISL_1020275, EPI_ISL_1020276, EPI_ISL_1020277, EPI_ISL_1020278, EPI_ISL_1020279, EPI_ISL_1020280, EPI_ISL_1020281, EPI_ISL_1020282, EPI_ISL_1020283, EPI_ISL_1020284			
see above	Department of Health Technology and Informatics, The Hong Kong Polytechnic University	Department of Health Technology and Informatics, The Hong Kong Polytechnic University	Gilman Kit-Hang Siu, Lam-Kwong Lee, Kenneth Siu-Sing Leung, Jake Siu-Lun Leung, Timothy Ting-Leung Ng, Chloe Toi-Mei Chan, Kingsley King-Gee Tam, Hiu-Yin Lao, Denise Sze-Hang Wong, Alan Ka-Lun Wu, Miranda Chong-Yee Yau, Yvette Wai-Man Lai, Kitty Sau-Chun Fung, Sandy Ka-Yee Chau, Barry Kin-Chung Wong, Wing-Kin To, Kristine Luk, Alex Yat-Man Ho, Tak-Lun Que, Kam-Tong Yip, Wing Cheong Yam, David Ho-Keung Shum, Shea Ping Yip
EPI_ISL_1020285, EPI_ISL_1020286, EPI_ISL_1020287, EPI_ISL_1020289, EPI_ISL_1020290, EPI_ISL_1020291, EPI_ISL_1020292, EPI_ISL_1020293	AZ Klina	AZ Klina	Dr. C. Vael
EPI_ISL_1027629, EPI_ISL_1027630, EPI_ISL_1027631, EPI_ISL_1027632, EPI_ISL_1027633, EPI_ISL_1027634, EPI_ISL_1027635, EPI_ISL_1027636	Mako Medical Laboratories	Mako Medical Laboratories	"Lauren Moon, Matthew Tugwell"
EPI_ISL_1029967	Cliniques universitaires Saint-Luc	UCLouvain/IREC/MBLG	Jean Ruelle, Lysa Pinsmaye, Benoit Kabamba Mukadi
EPI_ISL_1035889, EPI_ISL_1035890, EPI_ISL_1035891	Istituto Zooprofilattico Sperimentale del Mezzogiorno	IZSM-U.O.C. Virologia	Maurizio Viscardi, Lorena Cardillo, Sergio Brandi, Pellegrino Cerino, Massimo Zollo,Giovanna Fusco, Esterina De Carlo, Antonio Limone
EPI_ISL_1035892	Istituto Zooprofilattico Sperimentale del Mezzogiorno	IZSM-U.O.C. Virologia	Maurizio Viscardi, Lorena Cardillo, Claudio de Martinis, Giovanna Fusco, Esterina De Carlo, Antonio Limone
EPI_ISL_1035903, EPI_ISL_1035949, EPI_ISL_1035950, EPI_ISL_1035951	Outre Mer CH de Mayotte	National Reference Center for Viruses of Respiratory Infections, Institut Pasteur, Paris	Marion Barbet, Sylvie Behillil, Méline Bizard, Angela Brisebarre, Camille Capel, Etienne Simon-Lorière, Vincent Enouf, Maud Vanpeene, Sylvie van der Werf,Combe Patrice
EPI_ISL_1036213, EPI_ISL_1036214, EPI_ISL_1036218, EPI_ISL_1036219, EPI_ISL_1036220	SIESP TERAMO	Istituto Zooprofilattico Sperimentale dell'Abruzzo e Molise "G. Caporale"	Lorusso A, Marcacci M, Di Domenico M, Ancora M, Curini V, Mangone I, Rinaldi A, Scialabba S, Di Pasquale A, Cammà C, Puglia I, Calistri P, Savini G
EPI_ISL_1039008, EPI_ISL_1039009, EPI_ISL_1039010, EPI_ISL_1039011, EPI_ISL_1039157, EPI_ISL_1039158	WHO National Influenza Centre Russian Federation	WHO National Influenza Centre Russian Federation	Andrey Komissarov, Artem Fadeev, Anna Ivanova, Kseniya Komissarova, Dmitry Bazhenov, Tamila Musaeva, Maria Timofeeva, Veronika Eder, Maria Pisareva, Daria Danilenko, Ksenia Safina, Elena Nabieva, Georgii Bazykin, Dmitry Lioznov
EPI_ISL_1039160	Department of Laboratory Medicine, National Taiwan University Hospital	Microbial Genomics Core Lab, National Taiwan University Centers of Genomic and Precision Medicine	Shiou-Hwei Yeh, You-Yu Lin, Ya-Yun Lai, Chiao-Ling Li, Shan-Chwen Chang, Pei-Jer Chen, Sui-Yuan Chang
EPI_ISL_1039697	Instituto Adolfo Lutz Central	Instituto Adolfo Lutz, Interdiciplinary Procedures Center, Strategic Laboratory	Claudio Tavares Sacchi, Claudia Regina Gonçalves, Erica Valessa Ramos Gomes, Karoline Rodrigues Campos
EPI_ISL_1039698	Lab Loc - Itapecerica da Serra	Instituto Adolfo Lutz, Interdiciplinary Procedures Center, Strategic Laboratory	Claudio Tavares Sacchi, Claudia Regina Gonçalves, Erica Valessa Ramos Gomes, Karoline Rodrigues Campos
EPI_ISL_1039699	Instituto Adolfo Lutz - Regional de Taubate	Instituto Adolfo Lutz, Interdiciplinary Procedures Center, Strategic Laboratory	Claudio Tavares Sacchi, Claudia Regina Gonçalves, Erica Valessa Ramos Gomes, Karoline Rodrigues Campos
EPI_ISL_1039700, EPI_ISL_1039701	Instituto Adolfo Lutz Central	Instituto Adolfo Lutz, Interdiciplinary Procedures Center,	Claudio Tavares Sacchi, Claudia Regina Gonçalves, Erica Valessa Ramos Gomes, Karoline Rodrigues Campos

		Strategic Laboratory	
EPI_ISL_1039703	Instituto Adolfo Lutz - Regional de Taubate	Instituto Adolfo Lutz, Interdiciplinary Procedures Center, Strategic Laboratory	Claudio Tavares Sacchi, Claudia Regina Gonçalves, Erica Valessa Ramos Gomes, Karoline Rodrigues Campos
EPI_ISL_1039704	Lab Loc - Itapecerica da Serra	Instituto Adolfo Lutz, Interdiciplinary Procedures Center, Strategic Laboratory	Claudio Tavares Sacchi, Claudia Regina Gonçalves, Erica Valessa Ramos Gomes, Karoline Rodrigues Campos
EPI_ISL_1039705, EPI_ISL_1039706, EPI_ISL_1039707, EPI_ISL_1039708, EPI_ISL_1039709, EPI_ISL_1039710	Instituto Adolfo Lutz Central	Instituto Adolfo Lutz, Interdiciplinary Procedures Center, Strategic Laboratory	Claudio Tavares Sacchi, Claudia Regina Gonçalves, Erica Valessa Ramos Gomes, Karoline Rodrigues Campos
EPI_ISL_1040028, EPI_ISL_1040029	Original detection - Virology Unit, Institut Pasteur du Cambodge; Sequencing - US National Institute of Allergy and Infectious Diseases Cambodia	Virology Unit, Institut Pasteur du Cambodge	Jennifer Bohl, Sophana Chea, Sreyngim Lay, Ly Sovann, Kraing Sidonn, Yi Sengdoeurn, Chin Savuth, Chau Darapheak, Veasna Duong, Jessica Manning, Erik A Karlsson
EPI_ISL_1040825, EPI_ISL_1040826, EPI_ISL_1040827, EPI_ISL_1040828, EPI_ISL_1040829, EPI_ISL_1040830, EPI_ISL_1040831, EPI_ISL_1040832, EPI_ISL_1040833, EPI_ISL_1040834, EPI_ISL_1040835, EPI_ISL_1040836, EPI_ISL_1040837, EPI_ISL_1040838, EPI_ISL_1040839, EPI_ISL_1040840			
see above	LACEN do Mato Grosso do Sul	Instituto Adolfo Lutz, Interdiciplinary Procedures Center, Strategic Laboratory	Claudio Tavares Sacchi, Claudia Regina Gonçalves, Erica Valessa Ramos Gomes, Karoline Rodrigues Campos
EPI_ISL_1041030	Dr. Cipto Mangunkusumo (RSCM) Hospital	Faculty of Medicine, Universitas Indonesia	Rina La Distia Nora, Mei Riasanti, Rani Dwi Ningtias, Ari Fahrial Syam
EPI_ISL_1041195, EPI_ISL_1041196, EPI_ISL_1041197, EPI_ISL_1041198	Hungarian Defence Forces Military Medical Centre	National Laboratory of Virology, Szentágothai Research Centre	Endre Gábor Tóth, Balázs Somogyi, Ágnes Balázs-Nagy, Csaba Pereszlényi,Ferenc Jakab, Gábor Kemenesi
EPI_ISL_1041957, EPI_ISL_1041958	Department of Laboratory Medicine, National Taiwan University Hospital	Microbial Genomics Core Lab, National Taiwan University Centers of Genomic and Precision Medicine	Shiou-Hwei Yeh, You-Yu Lin, Ya-Yun Lai, Chiao-Ling Li, Shan-Chwen Chang, Pei-Jer Chen, Sui-Yuan Chang
EPI_ISL_1048372	Farmalab Indoutama Jakarta	National Institute of Health Research and Development	Hana Apsari Pawestri, Subangkit, Kartika Dewi Puspa, Arie Ardiansyah Nugraha, Hartanti Dian Ikawati, Krisna Nur Andriana Pangesti, Nelly Puspandari, Vivi Setiawaty.
EPI_ISL_1048406	Balai Penelitian dan Pengembangan Biomedis Papua	National Institute of Health Research and Development	Hana Apsari Pawestri, Subangkit, Kartika Dewi Puspa, Arie Ardiansyah Nugraha, Hartanti Dian Ikawati, Krisna Nur Andriana Pangesti, Nelly Puspandari, Anton Oktavianus, Hotma Sitompul, Vivi Setiawaty.
EPI_ISL_1048806, EPI_ISL_1048807, EPI_ISL_1048808, EPI_ISL_1048809, EPI_ISL_1048810, EPI_ISL_1048811, EPI_ISL_1048812, EPI_ISL_1048813	Laboratory of Clinical Microbiology, Virology and Bioemergencies - Fatebenefratelli Sacco Hospital	Virology Laboratory, Scientific Department, Army Medical Center	Valeria Micheli, Alessandro Mancon, Alberto Rizzo, Maria Rita Gismondo, Silvia Fillo, Anella Monte, Anna Anselmo, Vanessa Vera Fain, Giovanni Faggioni, Riccardo De Sanctis, Florigio Lista
EPI_ISL_1055191	Temporary Specimen Collection Centre at the AsiaWorld-Expo	Hong Kong Department of Health	Mak Gannon C.K., Lam Edman T.K., Chan Rickjason C.W., Tsang Dominic N.C.
EPI_ISL_1055192	Queen Mary Hospital	Hong Kong Department of Health	Mak Gannon C.K., Lam Edman T.K., Chan Rickjason C.W., Tsang Dominic N.C.
EPI_ISL_1055193	Prince of Wales Hospital	Hong Kong Department of Health	Mak Gannon C.K., Lam Edman T.K., Chan Rickjason C.W., Tsang Dominic N.C.
EPI_ISL_1055194, EPI_ISL_1055195	United Christian Hospital	Hong Kong Department of Health	Mak Gannon C.K., Lam Edman T.K., Chan Rickjason C.W., Tsang Dominic N.C.
EPI_ISL_1055196	Queen Mary Hospital	Hong Kong Department of Health	Mak Gannon C.K., Lam Edman T.K., Chan Rickjason C.W., Tsang Dominic N.C.
EPI_ISL_1055197, EPI_ISL_1055198	Prince of Wales Hospital	Hong Kong Department of Health	Mak Gannon C.K., Lam Edman T.K., Chan Rickjason C.W., Tsang Dominic N.C.
EPI_ISL_1055199	Princess Margaret Hospital	Hong Kong Department of Health	Mak Gannon C.K., Lam Edman T.K., Chan Rickjason C.W., Tsang Dominic N.C.
EPI_ISL_1055200	Queen Mary Hospital	Hong Kong Department of Health	Mak Gannon C.K., Lam Edman T.K., Chan Rickjason C.W., Tsang Dominic N.C.
EPI_ISL_1055201	Prince of Wales Hospital	Hong Kong Department of Health	Mak Gannon C.K., Lam Edman T.K., Chan Rickjason C.W., Tsang Dominic N.C.
EPI_ISL_1055202	Hong Kong Adventist Hospital - Tsuen Wan	Hong Kong Department of Health	Mak Gannon C.K., Lam Edman T.K., Chan Rickjason C.W., Tsang Dominic N.C.
EPI_ISL_1055203	Private medical practitioner	Hong Kong Department of Health	Mak Gannon C.K., Lam Edman T.K., Chan Rickjason C.W., Tsang Dominic N.C.
EPI_ISL_1055204	Queen Elizabeth Hospital	Hong Kong Department of Health	Mak Gannon C.K., Lam Edman T.K., Chan Rickjason C.W., Tsang Dominic N.C.
EPI_ISL_1055205	Queen Mary Hospital	Hong Kong Department of Health	Mak Gannon C.K., Lam Edman T.K., Chan Rickjason C.W., Tsang Dominic N.C.
EPI_ISL_1055206	Princess Margaret Hospital	Hong Kong Department of Health	Mak Gannon C.K., Lam Edman T.K., Chan Rickjason C.W., Tsang Dominic N.C.
EPI_ISL_1055207	Pamela Youde Nethersole Eastern Hospital	Hong Kong Department of Health	Mak Gannon C.K., Lam Edman T.K., Chan Rickjason C.W., Tsang Dominic N.C.
EPI_ISL_1055208	Private medical practitioner	Hong Kong Department of Health	Mak Gannon C.K., Lam Edman T.K., Chan Rickjason C.W., Tsang Dominic N.C.
EPI_ISL_1055209	Tuen Mun Hospital	Hong Kong Department of Health	Mak Gannon C.K., Lam Edman T.K., Chan Rickjason C.W., Tsang Dominic N.C.
EPI_ISL_1055210	Hong Kong Adventist Hospital - Tsuen Wan	Hong Kong Department of Health	Mak Gannon C.K., Lam Edman T.K., Chan Rickjason C.W., Tsang Dominic N.C.
EPI_ISL_1055211, EPI_ISL_1055212	Community Testing Centre	Hong Kong Department of Health	Mak Gannon C.K., Lam Edman T.K., Chan Rickjason C.W., Tsang Dominic N.C.
EPI_ISL_1055213	Queen Mary Hospital	Hong Kong Department of Health	Mak Gannon C.K., Lam Edman T.K., Chan Rickjason C.W., Tsang Dominic N.C.
EPI_ISL_1055214	Prince of Wales Hospital	Hong Kong Department of Health	Mak Gannon C.K., Lam Edman T.K., Chan Rickjason C.W., Tsang Dominic N.C.
EPI_ISL_1055215	Private medical practitioner	Hong Kong Department of Health	Mak Gannon C.K., Lam Edman T.K., Chan Rickjason C.W., Tsang Dominic N.C.
EPI_ISL_1055216	Community Testing Centre	Hong Kong Department of Health	Mak Gannon C.K., Lam Edman T.K., Chan Rickjason C.W., Tsang Dominic N.C.
EPI_ISL_1055217	Private medical practitioner	Hong Kong Department of Health	Mak Gannon C.K., Lam Edman T.K., Chan Rickjason C.W., Tsang Dominic N.C.
EPI_ISL_1055218	Tseung Kwan O Hospital	Hong Kong Department of Health	Mak Gannon C.K., Lam Edman T.K., Chan Rickjason C.W., Tsang Dominic N.C.
EPI_ISL_1055219	Princess Margaret Hospital	Hong Kong Department of Health	Mak Gannon C.K., Lam Edman T.K., Chan Rickjason C.W., Tsang Dominic N.C.
EPI_ISL_1055220	Community Testing Centre	Hong Kong Department of Health	Mak Gannon C.K., Lam Edman T.K., Chan Rickjason C.W., Tsang Dominic N.C.
EPI_ISL_1055221	Queen Mary Hospital	Hong Kong Department of Health	Mak Gannon C.K., Lam Edman T.K., Chan Rickjason C.W., Tsang Dominic N.C.
EPI_ISL_1055222, EPI_ISL_1055223	Queen Elizabeth Hospital	Hong Kong Department of Health	Mak Gannon C.K., Lam Edman T.K., Chan Rickjason C.W., Tsang Dominic N.C.
EPI_ISL_1055224	United Christian Hospital	Hong Kong Department of Health	Mak Gannon C.K., Lam Edman T.K., Chan Rickjason C.W., Tsang Dominic N.C.
EPI_ISL_1055225	Community Testing Centre	Hong Kong Department of Health	Mak Gannon C.K., Lam Edman T.K., Chan Rickjason C.W., Tsang Dominic N.C.
EPI_ISL_1055226	Queen Mary Hospital	Hong Kong Department of Health	Mak Gannon C.K., Lam Edman T.K., Chan Rickjason C.W., Tsang Dominic N.C.
EPI_ISL_1055227	Hong Kong Police Force	Hong Kong Department of Health	Mak Gannon C.K., Lam Edman T.K., Chan Rickjason C.W., Tsang Dominic N.C.
EPI_ISL_1055228	Queen Mary Hospital	Hong Kong Department of Health	Mak Gannon C.K., Lam Edman T.K., Chan Rickjason C.W., Tsang Dominic N.C.
EPI_ISL_1055229	Queen Elizabeth Hospital	Hong Kong Department of Health	Mak Gannon C.K., Lam Edman T.K., Chan Rickjason C.W., Tsang Dominic N.C.
EPI_ISL_1055230	Prince of Wales Hospital	Hong Kong Department of Health	Mak Gannon C.K., Lam Edman T.K., Chan Rickjason C.W., Tsang Dominic N.C.

EPI_ISL_1055231	Private medical practitioner	Hong Kong Department of Health	Mak Gannon C.K., Lam Edman T.K., Chan Rickjason C.W., Tsang Dominic N.C.
EPI_ISL_1055232	Hong Kong Baptist Hospital	Hong Kong Department of Health	Mak Gannon C.K., Lam Edman T.K., Chan Rickjason C.W., Tsang Dominic N.C.
EPI_ISL_1055233	Pamela Youde Nethersole Eastern Hospital	Hong Kong Department of Health	Mak Gannon C.K., Lam Edman T.K., Chan Rickjason C.W., Tsang Dominic N.C.
EPI_ISL_1055234	Queen Elizabeth Hospital	Hong Kong Department of Health	Mak Gannon C.K., Lam Edman T.K., Chan Rickjason C.W., Tsang Dominic N.C.
EPI_ISL_1055235	Pamela Youde Nethersole Eastern Hospital	Hong Kong Department of Health	Mak Gannon C.K., Lam Edman T.K., Chan Rickjason C.W., Tsang Dominic N.C.
EPI_ISL_1055236	Prince of Wales Hospital	Hong Kong Department of Health	Mak Gannon C.K., Lam Edman T.K., Chan Rickjason C.W., Tsang Dominic N.C.
EPI_ISL_1055237	Queen Mary Hospital	Hong Kong Department of Health	Mak Gannon C.K., Lam Edman T.K., Chan Rickjason C.W., Tsang Dominic N.C.
EPI_ISL_1055238	United Christian Hospital	Hong Kong Department of Health	Mak Gannon C.K., Lam Edman T.K., Chan Rickjason C.W., Tsang Dominic N.C.
EPI_ISL_1055239	Community Testing Centre	Hong Kong Department of Health	Mak Gannon C.K., Lam Edman T.K., Chan Rickjason C.W., Tsang Dominic N.C.
EPI_ISL_1055240	Pamela Youde Nethersole Eastern Hospital	Hong Kong Department of Health	Mak Gannon C.K., Lam Edman T.K., Chan Rickjason C.W., Tsang Dominic N.C.
EPI_ISL_1055241	United Christian Hospital	Hong Kong Department of Health	Mak Gannon C.K., Lam Edman T.K., Chan Rickjason C.W., Tsang Dominic N.C.
EPI_ISL_1055242	Queen Mary Hospital	Hong Kong Department of Health	Mak Gannon C.K., Lam Edman T.K., Chan Rickjason C.W., Tsang Dominic N.C.
EPI_ISL_1055243	Prince of Wales Hospital	Hong Kong Department of Health	Mak Gannon C.K., Lam Edman T.K., Chan Rickjason C.W., Tsang Dominic N.C.
EPI_ISL_1055244	Precious Blood Hospital	Hong Kong Department of Health	Mak Gannon C.K., Lam Edman T.K., Chan Rickjason C.W., Tsang Dominic N.C.
EPI_ISL_1055245	Queen Mary Hospital	Hong Kong Department of Health	Mak Gannon C.K., Lam Edman T.K., Chan Rickjason C.W., Tsang Dominic N.C.
EPI_ISL_1055246	Pok Oi Hospital	Hong Kong Department of Health	Mak Gannon C.K., Lam Edman T.K., Chan Rickjason C.W., Tsang Dominic N.C.
EPI_ISL_1055247	Queen Elizabeth Hospital	Hong Kong Department of Health	Mak Gannon C.K., Lam Edman T.K., Chan Rickjason C.W., Tsang Dominic N.C.
EPI_ISL_1055248	Prince of Wales Hospital	Hong Kong Department of Health	Mak Gannon C.K., Lam Edman T.K., Chan Rickjason C.W., Tsang Dominic N.C.
EPI_ISL_1055249	Caritas Medical Centre	Hong Kong Department of Health	Mak Gannon C.K., Lam Edman T.K., Chan Rickjason C.W., Tsang Dominic N.C.
EPI_ISL_1055250	Community Testing Centre	Hong Kong Department of Health	Mak Gannon C.K., Lam Edman T.K., Chan Rickjason C.W., Tsang Dominic N.C.
EPI_ISL_1055251	Princess Margaret Hospital	Hong Kong Department of Health	Mak Gannon C.K., Lam Edman T.K., Chan Rickjason C.W., Tsang Dominic N.C.
EPI_ISL_1055252, EPI_ISL_1055253	Community Testing Centre	Hong Kong Department of Health	Mak Gannon C.K., Lam Edman T.K., Chan Rickjason C.W., Tsang Dominic N.C.
EPI_ISL_1055254	Princess Margaret Hospital	Hong Kong Department of Health	Mak Gannon C.K., Lam Edman T.K., Chan Rickjason C.W., Tsang Dominic N.C.
EPI_ISL_1055255	Tuen Mun Hospital	Hong Kong Department of Health	Mak Gannon C.K., Lam Edman T.K., Chan Rickjason C.W., Tsang Dominic N.C.
EPI_ISL_1055256	Home Quarantine Taskforce	Hong Kong Department of Health	Mak Gannon C.K., Lam Edman T.K., Chan Rickjason C.W., Tsang Dominic N.C.
EPI_ISL_1055257	Pamela Youde Nethersole Eastern Hospital	Hong Kong Department of Health	Mak Gannon C.K., Lam Edman T.K., Chan Rickjason C.W., Tsang Dominic N.C.
EPI_ISL_1055258	Tuen Mun Hospital	Hong Kong Department of Health	Mak Gannon C.K., Lam Edman T.K., Chan Rickjason C.W., Tsang Dominic N.C.
EPI_ISL_1055259	United Christian Hospital	Hong Kong Department of Health	Mak Gannon C.K., Lam Edman T.K., Chan Rickjason C.W., Tsang Dominic N.C.
EPI_ISL_1055260	Caritas Medical Centre	Hong Kong Department of Health	Mak Gannon C.K., Lam Edman T.K., Chan Rickjason C.W., Tsang Dominic N.C.
EPI_ISL_1055263	Institute for Medical Research, Infectious Disease Research Centre, National Institutes of Health, Ministry of Health Malaysia	Institute for Medical Research, Infectious Disease Research Centre, National Institutes of Health, Ministry of Health Malaysia	Suppiah J, Kamel K,Moh Zawawi Z, Ramly N, Robert F, Thayan R
EPI_ISL_1055755, EPI_ISL_1055756, EPI_ISL_1055757, EPI_ISL_1055758	Microbiology Department. Complexo Hospitalario Universitario de Santiago	Microbiology Department. Complexo Hospitalario Universitario de Vigo	Microbiology Department, Complexo Hospitalario Universitario de Vigo (CHUVI). Complexo Hospitalario Universitario de Santiago (CHUS).
EPI_ISL_1055759, EPI_ISL_1055760	Vithas Lab. Hospital Vithas Vigo	Microbiology Department. Complexo Hospitalario Universitario de Vigo	Microbiology Department, Complexo Hospitalario Universitario de Vigo (CHUVI). Vithas Lab, Hospital Vithas Vigo
EPI_ISL_1055816	Ospedale SS Annunziata Chieti	Istituto Zooprofilattico Sperimentale dell'Abruzzo e Molise "G. Caporale"	Lorusso A, Marcacci M, Di Domenico M, Ancora M, Curini V, Mangone I, Rinaldi A, Delli Compagni E, Scialabba S, Di Pasquale A, Cammà C, Puglia I, Calistri P, Savini G
EPI_ISL_1055817	SIESP DIPARTIMENTO DI PREVENZIONE CHIETI	Istituto Zooprofilattico Sperimentale dell'Abruzzo e Molise "G. Caporale"	Lorusso A, Marcacci M, Di Domenico M, Ancora M, Curini V, Mangone I, Rinaldi A, Delli Compagni E, Scialabba S, Di Pasquale A, Cammà C, Puglia I, Calistri P, Savini G
EPI_ISL_1055818	DIP. PREV. AVEZZANO SERVIZIO DI IGIENE EPIDEMIOLOGIAE SANITA' PUBBLICA	Istituto Zooprofilattico Sperimentale dell'Abruzzo e Molise "G. Caporale"	Lorusso A, Marcacci M, Di Domenico M, Ancora M, Curini V, Mangone I, Rinaldi A, Delli Compagni E, Scialabba S, Di Pasquale A, Cammà C, Puglia I, Calistri P, Savini G
EPI_ISL_1055819	SIESP DIPARTIMENTO DI PREVENZIONE SULMONA	Istituto Zooprofilattico Sperimentale dell'Abruzzo e Molise "G. Caporale"	Lorusso A, Marcacci M, Di Domenico M, Ancora M, Curini V, Mangone I, Rinaldi A, Delli Compagni E, Scialabba S, Di Pasquale A, Cammà C, Puglia I, Calistri P, Savini G
EPI_ISL_1058285, EPI_ISL_1058286, EPI_ISL_1058287, EPI_ISL_1058288, EPI_ISL_1058289, EPI_ISL_1058290, EPI_ISL_1058291, EPI_ISL_1058292, EPI_ISL_1058293, EPI_ISL_1058294, EPI_ISL_1058295, EPI_ISL_1058296, EPI_ISL_1058297, EPI_ISL_1058298, EPI_ISL_1058299, EPI_ISL_1058300, EPI_ISL_1058301, EPI_ISL_1058302, EPI_ISL_1058303, EPI_ISL_1058304, EPI_ISL_1058305, EPI_ISL_1058306, EPI_ISL_1058307, EPI_ISL_1058308, EPI_ISL_1058309, EPI_ISL_1058310, EPI_ISL_1058311, EPI_ISL_1058312, EPI_ISL_1058313, EPI_ISL_1058314, EPI_ISL_1058315, EPI_ISL_1058316, EPI_ISL_1058317, EPI_ISL_1058318, EPI_ISL_1058319, EPI_ISL_1058320, EPI_ISL_1058321, EPI_ISL_1058322, EPI_ISL_1058323, EPI_ISL_1058324, EPI_ISL_1058325, EPI_ISL_1058326, EPI_ISL_1058327, EPI_ISL_1058328, EPI_ISL_1058329, EPI_ISL_1058330, EPI_ISL_1058331, EPI_ISL_1058332, EPI_ISL_1058333, EPI_ISL_1058334, EPI_ISL_1058335, EPI_ISL_1058336, EPI_ISL_1058337, EPI_ISL_1058338, EPI_ISL_1058339, EPI_ISL_1058340, EPI_ISL_1058341, EPI_ISL_1058342, EPI_ISL_1058343, EPI_ISL_1058344, EPI_ISL_1058345, EPI_ISL_1058346, EPI_ISL_1058347, EPI_ISL_1058348, EPI_ISL_1058349, EPI_ISL_1058350, EPI_ISL_1058351, EPI_ISL_1058352, EPI_ISL_1058353, EPI_ISL_1058354, EPI_ISL_1058355, EPI_ISL_1058356, EPI_ISL_1058357, EPI_ISL_1058358, EPI_ISL_1058359, EPI_ISL_1058360, EPI_ISL_1058361, EPI_ISL_1058362, EPI_ISL_1058363, EPI_ISL_1058364, EPI_ISL_1058365, EPI_ISL_1058366, EPI_ISL_1058367, EPI_ISL_1058368, EPI_ISL_1058369, EPI_ISL_1058370, EPI_ISL_1058371, EPI_ISL_1058372, EPI_ISL_1058373, EPI_ISL_1058374, EPI_ISL_1058375, EPI_ISL_1058376, EPI_ISL_1058377, EPI_ISL_1058378, EPI_ISL_1058379, EPI_ISL_1058380, EPI_ISL_1058381, EPI_ISL_1058382, EPI_ISL_1058383, EPI_ISL_1058384, EPI_ISL_1058385, EPI_ISL_1058386, EPI_ISL_1058387, EPI_ISL_1058388, EPI_ISL_1058389, EPI_ISL_1058390, EPI_ISL_1058391, EPI_ISL_1058392, EPI_ISL_1058393, EPI_ISL_1058394, EPI_ISL_1058395, EPI_ISL_1058396, EPI_ISL_1058397, EPI_ISL_1058398, EPI_ISL_1058399, EPI_ISL_1058400, EPI_ISL_1058401, EPI_ISL_1058402, EPI_ISL_1058403, EPI_ISL_1058404, EPI_ISL_1058405, EPI_ISL_1058406, EPI_ISL_1058407, EPI_ISL_1058408, EPI_ISL_1058409, EPI_ISL_1058410, EPI_ISL_1058411, EPI_ISL_1058412, EPI_ISL_1058413, EPI_ISL_1058414, EPI_ISL_1058415, EPI_ISL_1058416, EPI_ISL_1058417, EPI_ISL_1058418, EPI_ISL_1058419, EPI_ISL_1058420, EPI_ISL_1058421, EPI_ISL_1058422, EPI_ISL_1058423, EPI_ISL_1058424, EPI_ISL_1058425, EPI_ISL_1058426, EPI_ISL_1058427, EPI_ISL_1058428, EPI_ISL_1058429, EPI_ISL_1058430, EPI_ISL_1058431, EPI_ISL_1058432, EPI_ISL_1058433, EPI_ISL_1058434, EPI_ISL_1058435, EPI_ISL_1058436			
see above	Microbiology Department. Complexo Hospitalario Universitario de Vigo	Microbiology Department. Complexo Hospitalario Universitario de Vigo	Microbiology Department, Complexo Hospitalario Universitario de Vigo (CHUVI).
EPI_ISL_1059900, EPI_ISL_1059901	Malaysia Genome Institute	Malaysia Genome Institute	Mohd Noor Mat Isa, Irni Suhayu Sapian, Yusuf Muhammad Noor, Nurhezreen Md Iqbal, Mohd Faizal Abu Bakar, Enizza Kasim, Shamsidar Sopie, Siti Noraini Othman, Azrin Ahmad, Nor Azfa Johari.
EPI_ISL_1059902	DNA Laboratories Sdn. Bhd.	Malaysia Genome Institute	Mohd Noor Mat Isa, Irni Suhayu Sapian, Yusuf Muhammad Noor, Nurhezreen Md Iqbal, Mohd Faizal Abu Bakar, Enizza Kasim, Shamsidar Sopie, Siti Noraini Othman, Azrin Ahmad, Wong Yong Wee, Nor Azfa Johari.
EPI_ISL_1060359, EPI_ISL_1060360, EPI_ISL_1060361, EPI_ISL_1060362, EPI_ISL_1060363, EPI_ISL_1060364, EPI_ISL_1060365, EPI_ISL_1060366, EPI_ISL_1060367	Hopital	National Reference Center for Viruses of Respiratory Infections, Institut Pasteur, Paris	Marion Barbet, Sylvie Behillil, Méline Bizard, Angela Brisebarre, Camille Capel, Etienne Simon-Lorière, Vincent Enouf, Maud Vanpeene, Sylvie van der Werf,Fourgeaud Jacques
EPI_ISL_1060395	Labo Analyses Med	National Reference Center for Viruses of Respiratory Infections, Institut Pasteur, Paris	Marion Barbet, Sylvie Behillil, Méline Bizard, Angela Brisebarre, Camille Capel, Etienne Simon-Lorière, Vincent Enouf, Maud Vanpeene, Sylvie van der Werf,Chauvet

EPI_ISL_1060398, EPI_ISL_1060433, EPI_ISL_1060461, EPI_ISL_1060462	Hopital	National Reference Center for Viruses of Respiratory Infections, Institut Pasteur, Paris	Marion Barbet, Sylvie Behillil, Méline Bizard, Angela Brisebarre, Camille Capel, Etienne Simon-Lorière, Vincent Enouf, Maud Vanpeene, Sylvie van der Werf,Fourgeaud Jacques
EPI_ISL_1060475	Labo Analyses Med	National Reference Center for Viruses of Respiratory Infections, Institut Pasteur, Paris	Marion Barbet, Sylvie Behillil, Méline Bizard, Angela Brisebarre, Camille Capel, Etienne Simon-Lorière, Vincent Enouf, Maud Vanpeene, Sylvie van der Werf,Fiorina Jc
EPI_ISL_1060561, EPI_ISL_1060563, EPI_ISL_1060564, EPI_ISL_1060565, EPI_ISL_1060566	Hopital	National Reference Center for Viruses of Respiratory Infections, Institut Pasteur, Paris	Marion Barbet, Sylvie Behillil, Méline Bizard, Angela Brisebarre, Camille Capel, Etienne Simon-Lorière, Vincent Enouf, Maud Vanpeene, Sylvie van der Werf,Fourgeaud Jacques
EPI_ISL_1060727	Instituto de Diagnostico y Referencia Epidemiologicos (INDRE)_RNLSP	Instituto de Diagnostico y Referencia Epidemiologicos (INDRE)	Claudia Wong-Arambula, Abril Rodriguez-Maldonado, Fabiola Garces-Ayala, Adnan Araiza-Rodriguez, David Fragoso-Fonseca, Sergio Rangel-Guerrero, Mayra Jimenez-Morales, Nancy Munoz-Hernandez, Natividad Cruz-Ortiz, Tatiana Nunez-Garcia, Gisela Barrera-Badillo, Lucia Hernandez-Rivas, Irma Lopez-Martinez, Ernesto Ramirez-Gonzalez.
EPI_ISL_1061404, EPI_ISL_1061405	Queen Elizabeth Hospital	Hong Kong Children's Hospital	Herman Tse, Garnet Kwan-Yue Choi, Sally Cheuk-Ying Wong, David Christopher Lung
EPI_ISL_1061406	Kwong Wah Hospital	Hong Kong Children's Hospital	Herman Tse, Garnet Kwan-Yue Choi, Sally Cheuk-Ying Wong, David Christopher Lung
EPI_ISL_1061407	Queen Elizabeth Hospital	Hong Kong Children's Hospital	Herman Tse, Garnet Kwan-Yue Choi, Sally Cheuk-Ying Wong, David Christopher Lung
EPI_ISL_1061408	Kwong Wah Hospital	Hong Kong Children's Hospital	Herman Tse, Garnet Kwan-Yue Choi, Sally Cheuk-Ying Wong, David Christopher Lung
EPI_ISL_1061409, EPI_ISL_1061410, EPI_ISL_1061411	Queen Elizabeth Hospital	Hong Kong Children's Hospital	Herman Tse, Garnet Kwan-Yue Choi, Sally Cheuk-Ying Wong, David Christopher Lung
EPI_ISL_1063784	OSP SS ANNIUNZIATA CHIETI, PRONTO SOCCORSO	Istituto Zooprofilattico Sperimentale dell'Abruzzo e Molise "G. Caporale"	Lorusso A, Marcacci M, Di Domenico M, Ancora M, Curini V, Mangone I, Rinaldi A, Scialabba S, Di Pasquale A, Cammà C, Puglia I, Calistri P, Savini G
EPI_ISL_1078981, EPI_ISL_1078983, EPI_ISL_1078984, EPI_ISL_1078986, EPI_ISL_1078987, EPI_ISL_1078988, EPI_ISL_1078989, EPI_ISL_1078992	IAL Regional de Bauru	Instituto Adolfo Lutz, Interdiciplinary Procedures Center, Strategic Laboratory	Claudio Tavares Sacchi, Claudia Regina Gonçalves, Erica Valessa Ramos Gomes, Karoline Rodrigues Campos
EPI_ISL_1080375, EPI_ISL_1080376, EPI_ISL_1080378, EPI_ISL_1080379, EPI_ISL_1080380, EPI_ISL_1080381, EPI_ISL_1080382, EPI_ISL_1080384, EPI_ISL_1080385, EPI_ISL_1080386, EPI_ISL_1080387, EPI_ISL_1080388, EPI_ISL_1080389, EPI_ISL_1080390, EPI_ISL_1080391, EPI_ISL_1080491, EPI_ISL_1080492			
see above	Istituto Zooprofilattico Sperimentale del Mezzogiorno	Istituto Zooprofilattico Sperimentale del Mezzogiorno-U.O.C. Virologia	Maurizio Viscardi, Lorena Cardillo, Loredana Cozzolino, Pellegrino Cerino, Massimo Zollo,Giovanna Fusco, Esterina De Carlo, Antonio Limone
EPI_ISL_1080580, EPI_ISL_1080581, EPI_ISL_1080582, EPI_ISL_1080583, EPI_ISL_1080584, EPI_ISL_1080585, EPI_ISL_1080586, EPI_ISL_1080587, EPI_ISL_1080588, EPI_ISL_1080589, EPI_ISL_1080590, EPI_ISL_1080591, EPI_ISL_1080592, EPI_ISL_1080593, EPI_ISL_1080594, EPI_ISL_1080595, EPI_ISL_1080596, EPI_ISL_1080597, EPI_ISL_1080598, EPI_ISL_1080599, EPI_ISL_1080600, EPI_ISL_1080601, EPI_ISL_1080602			
see above	Istituto Zooprofilattico Sperimentale del Mezzogiorno	Istituto Zooprofilattico Sperimentale del Mezzogiorno-U.O.C. Virologia	Maurizio Viscardi, Lorena Cardillo, Loredana Cozzolino Pellegrino Cerino, Massimo Zollo,Giovanna Fusco, Esterina De Carlo, Antonio Limone
EPI_ISL_1080857, EPI_ISL_1081150, EPI_ISL_1081151, EPI_ISL_1081171, EPI_ISL_1081172, EPI_ISL_1081174, EPI_ISL_1081175, EPI_ISL_1081180, EPI_ISL_1081185, EPI_ISL_1081186, EPI_ISL_1081187, EPI_ISL_1081188, EPI_ISL_1081190, EPI_ISL_1081191			
see above	Baylor Scott & White-Temple	Baylor Scott & White-Temple	Ari Rao, Linden Morales, Kimberly Walker, Marcus Volz, Shelby Hendrickson
EPI_ISL_1081333	Institute for Medical Research, Infectious Disease Research Centre, National Institutes of Health, Ministry of Health Malaysia	Institute for Medical Research, Infectious Disease Research Centre, National Institutes of Health, Ministry of Health Malaysia	Suppiah J, Kamel K, Azizan MA, Thayan R
EPI_ISL_1081954	"Stefan S. Nicolau"Institute of Virology	"Stefan S. Nicolau"Institute of Virology	Anca Botezatu, Iulia Virginia Iancu, Coralia Bleotu, Mihaela Economescu, Laura Necula, Denisa Dragu, Lilia Matei, Ioana Pitica, Marinela Bostan, Mirela Mihaila, Laura Grecu, Camelia Sultana, Simona Ruta, Ana Iulia Neagu, Adriana Plesa, Saviana Nedeianu, Alina Nastasie, Cristina Mambet, Gabriela Anton, Carmen Cristina Diaconu.
EPI_ISL_1081958, EPI_ISL_1081959, EPI_ISL_1081961, EPI_ISL_1082252, EPI_ISL_1082290, EPI_ISL_1082291, EPI_ISL_1082294	"Stefan S. Nicolau"Institute of Virology	"Stefan S. Nicolau"Institute of Virology	Anca Botezatu, Iulia Virginia Iancu, Coralia Bleotu, Mihaela Economescu, Laura Necula, Denisa Dragu, Lilia Matei, Ioana Pitica, Marinela Bostan, Mirela Mihaila, Laura Grecu, Camelia Sultana, Simona Ruta, Ana Iulia Neagu, Adriana Plesa, Saviana Nedeianu, Alina Nastasie, Cristina Mambet,Gabriela Anton, Carmen Cristina Diaconu
EPI_ISL_1082296	"Stefan S. Nicolau"Institute of Virology	285 Mihai Bravu Ave, Bucharest, Romania	Anca Botezatu, Iulia Virginia Iancu, Coralia Bleotu, Mihaela Economescu, Laura Necula, Denisa Dragu, Lilia Matei, Ioana Pitica, Marinela Bostan, Mirela Mihaila, Laura Grecu, Camelia Sultana, Simona Ruta, Ana Iulia Neagu, Adriana Plesa, Saviana Nedeianu, Alina Nastasie, Cristina Mambet,Gabriela Anton, Carmen Cristina Diaconu
EPI_ISL_1085013	Laboratorio Biologia Molecolare Sars Cov2 - UOC Laboratorio Analisi - Servizio Medicina di Laboratorio, Ospedale "San Francesco" - ATS-ASSL Nuoro	Laboratorio Biologia Molecolare Sars Cov2 - UOC Laboratorio Analisi - Servizio Medicina di Laboratorio, Ospedale "San Francesco" - ATS-ASSL Nuoro	Piras Giovanna, Asproni Rosanna, Monne Maria Itria, Fiamma Maura, , Palmas Angelo Domenico, Lo Maglio Iana, Mameli Giuseppe
EPI_ISL_1085534, EPI_ISL_1085674, EPI_ISL_1085675	Hôpital Henri Mondor	Department of Virology, Henri Mondor University Hospital, Assistance Publique Hôpitaux de Paris, Université Paris-Est Créteil, INSERM U955	Christophe Rodriguez, Slim Fourati, Vanessa Demontant, Guillaume Gricourt, Melissa N'Debi, Alexandre Soulier, Elisabeth Trawinski, Jean-Michel Pawlotsky
EPI_ISL_1085676	University of Oregon COVID-19 MAP Laboratory	University of Oregon Genomics and Cell Characterization Core Facility (GC3F)	Douglas Turnbull, Ariana White, Jeff Bishop, Jason Carriere
EPI_ISL_1085677	University of Oregon COVID-19 MAP Laboratory	University of Oregon Genomics and Cell Characterization Core Facility (GC3F)	Douglas Turnbull, Ariana White, Jason Carriere, Jeff Bishop
EPI_ISL_1086048, EPI_ISL_1086049	IAL Regional de Bauru	Instituto Adolfo Lutz, Interdiciplinary Procedures Center, Strategic Laboratory	Claudio Tavares Sacchi, Claudia Regina Gonçalves, Erica Valessa Ramos Gomes, Karoline Rodrigues Campos, Caio Vinicius Dias Lopes
EPI_ISL_1091781	ICMT	Universidad Nacional de Colombia - Laboratorio Genómico One Health	Andres F. Cardona-Rios, Daniel O. Maldonado-Perez, Laura Silvana Perez, Karl A Ciuoderis, Idabely Betancur Ortiz, Sandra Ines Cano, Diego A. Álvarez-Díaz, Carlos Franco-Muñoz, Marcela Mercado-Reyes, Jorge E. Osorio, Juan P. Hernandez-Ortiz
EPI_ISL_1091784, EPI_ISL_1091785	LDSP	Universidad Nacional de Colombia - Laboratorio Genómico One Health	Andres F. Cardona-Rios, Daniel O. Maldonado-Perez, Laura Silvana Perez, Karl A Ciuoderis, Maria Angélica Maya, Idabely Betancur Ortiz, Sandra Ines Cano, Diego A. Álvarez-Díaz, Carlos Franco-Muñoz, Marcela Mercado-Reyes, Jorge E. Osorio, Juan P. Hernandez-Ortiz
EPI_ISL_1092005	Laboratorio de Salud Publica de Cesar	Instituto Nacional de Salud- Dirección de Investigación en Salud Pública	Katherine Laiton-Donato, Diego A. Álvarez-Díaz, Carlos Franco-Muñoz, Hector Alejandro Ruiz-Moreno, Maria T. Herrera-Sepúlveda, Diego Andrés Prada, Jhonnatan Reales-González, Sheryll Corchuelo, Julian Naizaque, Gerardo Santamaría, Magdalena Wiesner, Martha Lucia Ospina Martinez, Marcela Mercado-Reyes
EPI_ISL_1092006	Laboratorio IMAT	Instituto Nacional de Salud- Dirección de Investigación en Salud Pública	Katherine Laiton-Donato, Diego A. Álvarez-Díaz, Carlos Franco-Muñoz, Hector Alejandro Ruiz-Moreno, Maria T. Herrera-Sepúlveda, Diego Andrés Prada, Jhonnatan Reales-González, Sheryll Corchuelo, Julian Naizaque, Gerardo Santamaría, Magdalena Wiesner, Martha Lucia Ospina Martinez, Marcela Mercado-Reyes
EPI_ISL_1092007	Laboratorio Continental	Instituto Nacional de Salud- Dirección de Investigación en Salud Pública	Katherine Laiton-Donato, Diego A. Álvarez-Díaz, Carlos Franco-Muñoz, Hector Alejandro Ruiz-Moreno, Maria T. Herrera-Sepúlveda, Diego Andrés Prada, Jhonnatan Reales-González, Sheryll Corchuelo, Julian Naizaque, Gerardo Santamaría, Magdalena Wiesner, Martha Lucia Ospina Martinez, Marcela Mercado-Reyes
EPI_ISL_1096120	IAL Regional de Bauru	Instituto Adolfo Lutz, Interdiciplinary Procedures Center, Strategic Laboratory	Claudio Tavares Sacchi, Claudia Regina Gonçalves, Erica Valessa Ramos Gomes, Karoline Rodrigues Campos, Caio Vinicius Dias Lopes
EPI_ISL_1096249, EPI_ISL_1096261, EPI_ISL_1096278, EPI_ISL_1096280, EPI_ISL_1096283, EPI_ISL_1096284, EPI_ISL_1096286, EPI_ISL_1096289, EPI_ISL_1096292, EPI_ISL_1096294, EPI_ISL_1096297, EPI_ISL_1096299			

see above	Hopital	National Reference Center for Viruses of Respiratory Infections, Institut Pasteur, Paris	Marion Barbet, Sylvie Behillil, Méline Bizard, Angela Brisebarre, Camille Capel, Etienne Simon-Lorière, Vincent Enouf, Maud Vanpeene, Sylvie van der Werf,Guinard JéRome
EPI_ISL_1096331	Research	National Reference Center for Viruses of Respiratory Infections, Institut Pasteur, Paris	Marion Barbet, Sylvie Behillil, Méline Bizard, Angela Brisebarre, Camille Capel, Etienne Simon-Lorière, Vincent Enouf, Maud Vanpeene, Sylvie van der Werf,Leruez-Ville Marianne
EPI_ISL_1097313, EPI_ISL_1097314, EPI_ISL_1097315, EPI_ISL_1097316, EPI_ISL_1097317, EPI_ISL_1097321, EPI_ISL_1097322, EPI_ISL_1097323, EPI_ISL_1097324, EPI_ISL_1097325, EPI_ISL_1097326, EPI_ISL_1097327, EPI_ISL_1097328, EPI_ISL_1097329, EPI_ISL_1097330, EPI_ISL_1097331, EPI_ISL_1097332, EPI_ISL_1097333, EPI_ISL_1097334, EPI_ISL_1097335, EPI_ISL_1097336, EPI_ISL_1097337, EPI_ISL_1097338, EPI_ISL_1097339, EPI_ISL_1097340, EPI_ISL_1097341, EPI_ISL_1097342, EPI_ISL_1097343, EPI_ISL_1097344, EPI_ISL_1097345, EPI_ISL_1097346			
see above	University of Oregon COVID-19 MAP Laboratory	University of Oregon Genomics and Cell Characterization Core Facility (GC3F)	Douglas Turnbull, Ariana White, Jeff Bishop, Jason Carriere
EPI_ISL_1103557	Kurnool Medical College (KMC)	CSIR Institute of Genomics and Integrative Biology	Pallavali Roja Rani, Mohamed Imran, Juturu Vijaya Lakshmi, Bani Jolly, Abhinav Jain, Avileli Surekha, Vigneshwar Senthivel, Pulala Chandrasekhar, Mohit Kumar Divakar, Damam Srinivasulu, Rahul C Bhoyar, PR Vanaja, Vinod Scaria, Sridhar Sivasubbu
EPI_ISL_1109637	Althaia. Xarxa Assistencial Universitària de Manresa	IrsiCaixa - Can Ruti CovidSeq	Marc Noguera-Julian, Mariona Parera, Maria Casadellà, Pilar Armengol, Francesc Catala-Moll, Roger Paredes, Bonaventura Clotet Gloria Trujillo, Rafel Perez Vidal, Jaume Trape Pujol, Carolina Gonzalez Fernandez, Roger Paredes, Eulalia Grau, Bonaventura Clotet
EPI_ISL_1110291	Baylor Scott & White-Temple	Baylor Scott & White-Temple	Ari Rao, Linden Morales, Kimberly Walker, Marcus Volz, Shelby Hendrickson
EPI_ISL_1110410, EPI_ISL_1110411, EPI_ISL_1110412, EPI_ISL_1110413	Hopital	National Reference Center for Viruses of Respiratory Infections, Institut Pasteur, Paris	Marion Barbet, Sylvie Behillil, Méline Bizard, Angela Brisebarre, Camille Capel, Etienne Simon-Lorière, Vincent Enouf, Maud Vanpeene, Sylvie van der Werf,Pilorge LéA
EPI_ISL_1110429	Labo Analyses Med	National Reference Center for Viruses of Respiratory Infections, Institut Pasteur, Paris	Marion Barbet, Sylvie Behillil, Méline Bizard, Angela Brisebarre, Camille Capel, Etienne Simon-Lorière, Vincent Enouf, Maud Vanpeene, Sylvie van der Werf,Etienne Elodie
EPI_ISL_1110435, EPI_ISL_1110437, EPI_ISL_1110438, EPI_ISL_1110440	Hopital	National Reference Center for Viruses of Respiratory Infections, Institut Pasteur, Paris	Marion Barbet, Sylvie Behillil, Méline Bizard, Angela Brisebarre, Camille Capel, Etienne Simon-Lorière, Vincent Enouf, Maud Vanpeene, Sylvie van der Werf,Pilorge LéA
EPI_ISL_1110463	Labo Analyses Med	National Reference Center for Viruses of Respiratory Infections, Institut Pasteur, Paris	Marion Barbet, Sylvie Behillil, Méline Bizard, Angela Brisebarre, Camille Capel, Etienne Simon-Lorière, Vincent Enouf, Maud Vanpeene, Sylvie van der Werf,Morvan Odile
EPI_ISL_1110472	Labo Analyses Med	National Reference Center for Viruses of Respiratory Infections, Institut Pasteur, Paris	Marion Barbet, Sylvie Behillil, Méline Bizard, Angela Brisebarre, Camille Capel, Etienne Simon-Lorière, Vincent Enouf, Maud Vanpeene, Sylvie van der Werf,Etienne Elodie
EPI_ISL_1110492, EPI_ISL_1110493, EPI_ISL_1110494, EPI_ISL_1110495, EPI_ISL_1110496, EPI_ISL_1110497, EPI_ISL_1110498, EPI_ISL_1110499, EPI_ISL_1110500, EPI_ISL_1110501, EPI_ISL_1110502, EPI_ISL_1110503, EPI_ISL_1110504, EPI_ISL_1110505, EPI_ISL_1110506, EPI_ISL_1110507, EPI_ISL_1110508, EPI_ISL_1110509, EPI_ISL_1110510			
see above	Hopital	National Reference Center for Viruses of Respiratory Infections, Institut Pasteur, Paris	Marion Barbet, Sylvie Behillil, Méline Bizard, Angela Brisebarre, Camille Capel, Etienne Simon-Lorière, Vincent Enouf, Maud Vanpeene, Sylvie van der Werf,Pilorge LéA
EPI_ISL_1110952	Research	National Reference Center for Viruses of Respiratory Infections, Institut Pasteur, Paris	Marion Barbet, Sylvie Behillil, Méline Bizard, Angela Brisebarre, Camille Capel, Etienne Simon-Lorière, Vincent Enouf, Maud Vanpeene, Sylvie van der Werf,Leruez-Ville Marianne
EPI_ISL_1110967	Hopital	National Reference Center for Viruses of Respiratory Infections, Institut Pasteur, Paris	Marion Barbet, Sylvie Behillil, Méline Bizard, Angela Brisebarre, Camille Capel, Etienne Simon-Lorière, Vincent Enouf, Maud Vanpeene, Sylvie van der Werf,Pilorge LéA
EPI_ISL_1111069, EPI_ISL_1111132, EPI_ISL_1111133, EPI_ISL_1111134, EPI_ISL_1111135, EPI_ISL_1111136, EPI_ISL_1111137, EPI_ISL_1111138, EPI_ISL_1111139, EPI_ISL_1111140, EPI_ISL_1111141, EPI_ISL_1111142, EPI_ISL_1111143, EPI_ISL_1111144, EPI_ISL_1111145, EPI_ISL_1111146, EPI_ISL_1111147, EPI_ISL_1111148, EPI_ISL_1111149, EPI_ISL_1111150, EPI_ISL_1111151, EPI_ISL_1111152, EPI_ISL_1111153, EPI_ISL_1111154, EPI_ISL_1111155, EPI_ISL_1111156, EPI_ISL_1111157, EPI_ISL_1111158, EPI_ISL_1111159, EPI_ISL_1111160, EPI_ISL_1111161, EPI_ISL_1111308, EPI_ISL_1111309, EPI_ISL_1111310, EPI_ISL_1111312, EPI_ISL_1111314, EPI_ISL_1111315, EPI_ISL_1111446, EPI_ISL_1111457, EPI_ISL_1111458, EPI_ISL_1111459, EPI_ISL_1111460, EPI_ISL_1111461, EPI_ISL_1111462, EPI_ISL_1111463, EPI_ISL_1111464, EPI_ISL_1111465, EPI_ISL_1111466, EPI_ISL_1111467, EPI_ISL_1111469, EPI_ISL_1111470, EPI_ISL_1111471, EPI_ISL_1111472, EPI_ISL_1111473, EPI_ISL_1111474, EPI_ISL_1111475, EPI_ISL_1111476, EPI_ISL_1111477, EPI_ISL_1111478, EPI_ISL_1111479, EPI_ISL_1111480, EPI_ISL_1111481, EPI_ISL_1111482, EPI_ISL_1111483, EPI_ISL_1111484, EPI_ISL_1111485, EPI_ISL_1111486, EPI_ISL_1111487, EPI_ISL_1111488, EPI_ISL_1111489, EPI_ISL_1111490, EPI_ISL_1111491, EPI_ISL_1111492, EPI_ISL_1111493, EPI_ISL_1111494, EPI_ISL_1111495, EPI_ISL_1111496, EPI_ISL_1111497, EPI_ISL_1111498, EPI_ISL_1111499, EPI_ISL_1111500, EPI_ISL_1111501, EPI_ISL_1111502, EPI_ISL_1111503, EPI_ISL_1111504, EPI_ISL_1111505			
see above	Laboratorio de Referencia Nacional de Virus Respiratorio. Instituto Nacional de Salud Perú	Laboratorio de Referencia Nacional de Enteropatógenos. Instituto Nacional de Salud del Perú	Ronnie Gavilan Chavez, Junior Caro Castro, Willi Quino Sifuentes, Veronica Hurtado Vela, Iris Silva Molina, Fiorella Orellana Peralta
EPI_ISL_1112227, EPI_ISL_1112231	Istituto Zooprofilattico Sperimentale della Puglia e della Basilicata	Istituto Zooprofilattico Sperimentale della Puglia e della Basilicata	Parisi A., Bianco A., Capozzi L., Del Sambro L., Simone D., Giannico A., Ridolfi D.
EPI_ISL_1114726	Institute for Medical Research, Infectious Disease Research Centre, National Institutes of Health, Ministry of Health Malaysia	Institute for Medical Research, Infectious Disease Research Centre, National Institutes of Health, Ministry of Health Malaysia	Suppiah J, Kamel K, Azizan MA, Thayan R
EPI_ISL_1117376	Beijing Center for Disease Prevention and Control	Beijing Center for Disease Prevention and Control	Yang Pan, Shujuan Cui, Fu Li, Zhaomin Feng, Bing Lyu, Zhichao Liang, Lijuan Chen, Daitao Zhang, Quanyi Wang
EPI_ISL_1117377	Beijing Center for Disease Prevention and Control	Beijing Center for Disease Prevention and Control	Yang Pan, Fu Li, Zhaomin Feng, Shujuan Cui, Bing Lyu, Zhichao Liang, Lijuan Chen, Daitao Zhang, Quanyi Wang
EPI_ISL_1117378	Beijing Center for Disease Prevention and Control	Beijing Center for Disease Prevention and Control	Yang Pan, Zhaomin Feng, Fu Li, Shujuan Cui, Bing Lyu, Zhichao Liang, Lijuan Chen, Daitao Zhang, Quanyi Wang
EPI_ISL_1117450	Melania Hospital, Bagor , west Java	Biosafety Level-3 Laboratory, Indonesian Institute of Sciences (LIPI)	Anggia Prasetyoputri, Isa Nuryana, Sri Swasthikawati, Sugiyono Saputra, Syam Budi Iryanto, Eko Wahyu Putro, Gita Syahputra, Ari Sulistyo Wulandari, Ratih Asmana Ningrum
EPI_ISL_1117457	Klinik Apotik Dien, Jakarta	Biosafety Level-3 Laboratory, Indonesian Institute of Sciences (LIPI)	Anik Budhi Dharmayanthi, Ade Andriani, Hariyatun, Asep M. Ridwanuloh, Syam Budi Iryanto, Eko Wahyu Putro, Gita Syahputra, Susanti, Inswasti Cahyani, Matthew W. Loose, Mohammad Ilyas, Wien Kusharyoto
EPI_ISL_1117590	RS Trimitra, Bogor, West java	Biosafety Level-3 Laboratory, Indonesian Institute of Sciences (LIPI)	Anik Budhi Dharmayanthi, Ade Andriani, Hariyatun, Asep M. Ridwanuloh, Syam Budi Iryanto, Eko Wahyu Putro, Gita Syahputra, Susanti, Inswasti Cahyani, Matthew W. Loose, Mohammad Ilyas, Wien Kusharyoto
EPI_ISL_1117592	Melania Hospital, Bagor , West Java	Biosafety Level-3 Laboratory, Indonesian Institute of Sciences (LIPI)	Anik Budhi Dharmayanthi, Ade Andriani, Hariyatun, Asep M. Ridwanuloh, Syam Budi Iryanto, Eko Wahyu Putro, Gita Syahputra, Susanti, Inswasti Cahyani, Matthew W. Loose, Mohammad Ilyas, Wien Kusharyoto
EPI_ISL_1117937, EPI_ISL_1117938, EPI_ISL_1117939, EPI_ISL_1117940, EPI_ISL_1117941, EPI_ISL_1117942, EPI_ISL_1117943, EPI_ISL_1117944, EPI_ISL_1117945, EPI_ISL_1117946, EPI_ISL_1117947, EPI_ISL_1117948			
see above	Austrian Agency for Health and Food Safety (AGES)	Bergthaler laboratory, CeMM Research Center for Molecular Medicine of the Austrian Academy of Sciences	Lukas Endler, Anna Schedl, Thomas Penz, Benedikt Agerer, Maelle Le Moing, Michael Schuster, Bekir Erguner, Jan Laine, Martin Senekowitsch, Christoph Bock, Andreas Bergthaler
EPI_ISL_1117953, EPI_ISL_1117954, EPI_ISL_1117961, EPI_ISL_1117969, EPI_ISL_1117981, EPI_ISL_1117982, EPI_ISL_1117983, EPI_ISL_1117986, EPI_ISL_1117987, EPI_ISL_1117989, EPI_ISL_1117990, EPI_ISL_1118020			
see above	HG Pharma GmbH	Bergthaler laboratory, CeMM Research Center for Molecular Medicine of the Austrian Academy of Sciences	Lukas Endler, Anna Schedl, Thomas Penz, Benedikt Agerer, Maelle Le Moing, Michael Schuster, Bekir Erguner, Jan Laine, Martin Senekowitsch, Christoph Bock, Andreas Bergthaler
EPI_ISL_1118206	National Institute of Health Research and Development	National Institute of Health Research and Development	Hana Apsari Pawestri, Subangkit, Kartika Dewi Puspa, Arie Ardiansyah Nugraha, Hartanti Dian Ikawati, Krisna Nur Andriana Pangesti, Yuni Rukminiati, Ririn Ramadhany, Agustiningsih, Kindi Adam, Holy Arif Wibowo, Triyani Soekarso, Ni Ketut Susilarini, Nurika Hariastuti, Ully Alfi Nikmah, Reni Herman, Nike Susanti, Herna, Tati Febriyanti, Natalie Laurencia Kipuw, Fauzul Muna, Irene Lorinda Indalao, Nelly Puspandari, Vivi Setiawaty.
EPI_ISL_1118280	Merial Health Hospital, Jakarta	Biosafety Level-3 Laboratory, Indonesian Institute of Sciences (LIPI)	Anik Budhi Dharmayanthi, Ade Andriani, Hariyatun, Asep M. Ridwanuloh, Syam Budi Iryanto, Eko Wahyu Putro, Gita Syahputra, Susanti, Inswasti Cahyani, Matthew W. Loose, Mohammad Ilyas, Wien Kusharyoto
EPI_ISL_1118879, EPI_ISL_1118880, EPI_ISL_1118881, EPI_ISL_1118882	Labo Analyses Med	National Reference Center for Viruses of Respiratory Infections, Institut Pasteur, Paris	Marion Barbet, Sylvie Behillil, Méline Bizard, Angela Brisebarre, Camille Capel, Etienne Simon-Lorière, Vincent Enouf, Maud Vanpeene, Sylvie van der Werf,King Lisa
EPI_ISL_1121316	LACEN do Rio Grande do Sul	Instituto Adolfo Lutz, Interdiciplinary Procedures Center, Strategic Laboratory	Claudio Tavares Sacchi, Claudia Regina Gonçalves, Erica Valessa Ramos Gomes, Karoline Rodrigues Campos, Caio Vinicius Dias Lopes

EPI_ISL_1123375	IAL Regional de Santos	Instituto Adolfo Lutz, Interdiciplinary Procedures Center, Strategic Laboratory	Claudio Tavares Sacchi, Claudia Regina Gonçalves, Erica Valessa Ramos Gomes, Karoline Rodrigues Campos, Caio Vinicius Dias Lopes
EPI_ISL_1133133	LABCOVID_HCPA	LABRESIS_HCPA	Martins AF, Wink PL, Volpato F, Rosset C, de Paris F, Monteiro F, Barth AL
EPI_ISL_1138411	Hospital HIA Percy, Medical Biology	National Reference Center for Viruses of Respiratory Infections, Institut Pasteur, Paris	Marion Barbet, Sylvie Behillil, Méline Bizard, Angela Brisebarre, Camille Capel, Etienne Simon-Lorière, Vincent Enouf, Maud Vanpeene, Sylvie van der Werf,Vincent Foissaud
EPI_ISL_1138512, EPI_ISL_1138513, EPI_ISL_1138514, EPI_ISL_1138515, EPI_ISL_1138516, EPI_ISL_1138517	SA Pärnu Hospital Laboratory	1. Laboratory of Communicable Diseases (Estonia); 2. Eurofins Genomics Europe Sequencing GmbH	Liidia Dotsenko
EPI_ISL_1139059	LACEN do Mato Grosso do Sul	Instituto Adolfo Lutz, Interdiciplinary Procedures Center, Strategic Laboratory	Claudio Tavares Sacchi, Claudia Regina Gonçalves, Erica Valessa Ramos Gomes, Karoline Rodrigues Campos, Caio Vinicius Dias Lopes
EPI_ISL_1159070, EPI_ISL_1159071, EPI_ISL_1159072, EPI_ISL_1159073, EPI_ISL_1159074, EPI_ISL_1159075, EPI_ISL_1159076, EPI_ISL_1159077, EPI_ISL_1159078, EPI_ISL_1159079, EPI_ISL_1159080, EPI_ISL_1159081, EPI_ISL_1159082, EPI_ISL_1159083, EPI_ISL_1159084, EPI_ISL_1159085, EPI_ISL_1159086, EPI_ISL_1159087, EPI_ISL_1159088, EPI_ISL_1159089, EPI_ISL_1159090, EPI_ISL_1159091, EPI_ISL_1159092, EPI_ISL_1159093, EPI_ISL_1159094, EPI_ISL_1159095, EPI_ISL_1159096, EPI_ISL_1159097, EPI_ISL_1159098, EPI_ISL_1159099, EPI_ISL_1159100, EPI_ISL_1159101, EPI_ISL_1159102, EPI_ISL_1159103, EPI_ISL_1159104, EPI_ISL_1159105, EPI_ISL_1159106			
see above	Department of Health Technology and Informatics, The Hong Kong Polytechnic University	Department of Health Technology and Informatics, The Hong Kong Polytechnic University	Gilman Kit-Hang Siu, Lam-Kwong Lee, Kenneth Siu-Sing Leung, Jake Siu-Lun Leung, Timothy Ting-Leung Ng, Chloe Toi-Mei Chan, Kingsley King-Gee Tam, Hiu-Yin Lao, Denise Sze-Hang Wong, Alan Ka-Lun Wu, Miranda Chong-Yee Yau, Yvette Wai-Man Lai, Kitty Sau-Chun Fung, Sandy Ka-Yee Chau, Barry Kin-Chung Wong, Wing-Kin To, Kristine Luk, Alex Yat-Man Ho, Tak-Lun Que, Kam-Tong Yip, Wing Cheong Yam, David Ho-Keung Shum, Shea Ping Yip
EPI_ISL_1159382	Adi Husada Kapasari Hospital	Institute of Tropical Disease, Universitas Airlangga	Maria I Lusida, Krisnoadi Rahardjo, Aldise M Nastri, Jezzy R Dewantari, Rima R Prasetya, Hermanto Wijaya, Gatot Soegiarto, Laksmi Wulandari, Resti Yudhawati, Soetjipto, Yasuko Mori, Kazufumi Shimizu
EPI_ISL_1159383	Siti Khodijah Hospital	Institute of Tropical Disease, Universitas Airlangga	Aldise M Nastri, Jezzy R Dewantari, Rima R Prasetya, Krisnoadi Rahardjo, Muhammad Hamdan, Gatot Soegiarto, Laksmi Wulandari, Resti Yudhawati, Soetjipto, Yasuko Mori, Maria I Lusida, Kazufumi Shimizu
EPI_ISL_1159385	Graha Medika Maternity Hospital	Institute of Tropical Disease, Universitas Airlangga	Rima R Prasetya, Krisnoadi Rahardjo, Aldise M Nastri, Jezzy R Dewantari, Diah R Kusumawati, Gatot Soegiarto, Laksmi Wulandari, Resti Yudhawati, Soetjipto, Yasuko Mori, Maria I Lusida, Kazufumi Shimizu
EPI_ISL_1159386	Siti Khodijah Hospital	Institute of Tropical Disease, Universitas Airlangga	Krisnoadi Rahardjo, Aldise M Nastri, Jezzy R Dewantari, Rima R Prasetya, Muhammad Hamdan, Gatot Soegiarto, Laksmi Wulandari, Resti Yudhawati, Yasuko Mori, Soetjipto, Kazufumi Shimizu, Maria I Lusida
EPI_ISL_1159387	Adi Husada Kapasari Hospital	Institute of Tropical Disease, Universitas Airlangga	Maria I Lusida, Krisnoadi Rahardjo, Aldise M Nastri, Jezzy R Dewantari, Rima R Prasetya, Hermanto Wijaya, Gatot Soegiarto, Laksmi Wulandari, Resti Yudhawati, Soetjipto, Yasuko Mori, Kazufumi Shimizu
EPI_ISL_1159388	Paru Hospital	Institute of Tropical Disease, Universitas Airlangga	Aldise M Nastri, Jezzy R Dewantari, Rima R Prasetya, Krisnoadi Rahardjo, Dyah Retno, Gatot Soegiarto, Laksmi Wulandari, Resti Yudhawati, Soetjipto, Yasuko Mori, Maria I Lusida, Kazufumi Shimizu
EPI_ISL_1159389	Darmo Hospital	Institute of Tropical Disease, Universitas Airlangga	Jezzy R Dewantari, Rima R Prasetya, Krisnoadi Rahardjo, Aldise M Nastri, Sulung Budianto, Gatot Soegiarto, Laksmi Wulandari, Resti Yudhawati, Soetjipto, Yasuko Mori, Maria I Lusida, Kazufumi Shimizu
EPI_ISL_1161401, EPI_ISL_1161402, EPI_ISL_1161403, EPI_ISL_1161404, EPI_ISL_1161406, EPI_ISL_1161407, EPI_ISL_1161408, EPI_ISL_1161409, EPI_ISL_1163712, EPI_ISL_1163713, EPI_ISL_1163714, EPI_ISL_1163715, EPI_ISL_1163716			
see above	LABCOVID_HCPA	LABRESIS_HCPA	Martins AF, Wink PL, Volpato F, Rosset C, de Paris F, Monteiro F, Zavascki AP, Barth AL
EPI_ISL_1163736	LABCOVID_HCPA	LABCOVID_HCPA	Martins AF, Wink PL, Volpato F, Rosset C, de Paris F, Monteiro F, Zavascki AP, Barth AL
EPI_ISL_1163737	LABCOVID_HCPA	LABRESIS_HCPA	Martins AF, Wink PL, Volpato F, Rosset C, de Paris F, Monteiro F, Zavascki AP, Barth AL
EPI_ISL_1163827	Università degli Studi di Perugia	Istituto Zooprofilattico Sperimentale dell'Abruzzo e Molise "G. Caporale"	Mencacci A., Camilloni B., Lorusso A, Marcacci M, Di Domenico M, Ancora M, Curini V, Mangone I, Rinaldi A, Scialabba S, Di Pasquale A, Cammà C, Puglia I, Calistri P, Savini G
EPI_ISL_1164927, EPI_ISL_1164928, EPI_ISL_1164929	Genome Analysis Center, Yamanashi Central Hospital	Genome Analysis Center, Yamanashi Central Hospital	Yosuke Hirotsu
EPI_ISL_1165070	Siti Khodijah Hospital	Institute of Tropical Disease, Universitas Airlangga	Rima R Prasetya, Krisnoadi Rahardjo, Aldise M Nastri, Jezzy R Dewantari, Muhammad Hamdan, Gatot Soegiarto, Laksmi Wulandari, Resti Yudhawati, Yasuko Mori, Soetjipto, Kazufumi Shimizu, Maria I Lusida
EPI_ISL_1165071	Anwar Medika General Hospital	Institute of Tropical Disease, Universitas Airlangga	Kazufumi Shimizu, Krisnoadi Rahardjo, Aldise M Nastri, Jezzy R Dewantari, Rima R Prasetya, Nungky Taniasari, Gatot Soegiarto, Laksmi Wulandari, Resti Yudhawati, Yasuko Mori, Soetjipto, Maria I Lusida
EPI_ISL_1165073	Darus Syifa' Islamic Hospital	Institute of Tropical Disease, Universitas Airlangga	Kazufumi Shimizu, Krisnoadi Rahardjo, Aldise M Nastri, Jezzy R Dewantari, Rima R Prasetya, HM Faiz, Gatot Soegiarto, Laksmi Wulandari, Resti Yudhawati, Yasuko Mori, Soetjipto, Maria I Lusida
EPI_ISL_1165076	Rahman Rahim Hospital	Institute of Tropical Disease, Universitas Airlangga	Jezzy R Dewantari, Rima R Prasetya, Krisnoadi Rahardjo, Aldise M Nastri, Rendra E Febrianto, Gatot Soegiarto, Laksmi Wulandari, Resti Yudhawati, Yasuko Mori, Soetjipto, Kazufumi Shimizu, Maria I Lusida
EPI_ISL_1166891, EPI_ISL_1166892, EPI_ISL_1166893, EPI_ISL_1166894, EPI_ISL_1166895, EPI_ISL_1166896, EPI_ISL_1166897, EPI_ISL_1166898, EPI_ISL_1166899, EPI_ISL_1166900, EPI_ISL_1166901, EPI_ISL_1166902, EPI_ISL_1166903, EPI_ISL_1166904, EPI_ISL_1166905, EPI_ISL_1166906, EPI_ISL_1166907, EPI_ISL_1166908, EPI_ISL_1166909, EPI_ISL_1166910, EPI_ISL_1166911, EPI_ISL_1166912, EPI_ISL_1166913, EPI_ISL_1166914, EPI_ISL_1166915, EPI_ISL_1166916, EPI_ISL_1166917, EPI_ISL_1166919, EPI_ISL_1166920			
see above	Hopital	National Reference Center for Viruses of Respiratory Infections, Institut Pasteur, Paris	Marion Barbet, Sylvie Behillil, Méline Bizard, Angela Brisebarre, Camille Capel, Etienne Simon-Lorière, Vincent Enouf, Maud Vanpeene, Sylvie van der Werf,Combe Patrice
EPI_ISL_1167135, EPI_ISL_1167138, EPI_ISL_1167139, EPI_ISL_1167140, EPI_ISL_1167141, EPI_ISL_1167145, EPI_ISL_1167146, EPI_ISL_1167147, EPI_ISL_1167148, EPI_ISL_1167149, EPI_ISL_1167150, EPI_ISL_1167173, EPI_ISL_1167175, EPI_ISL_1167176, EPI_ISL_1167177			
see above	Iressef Genomics lab	L'institut de Recherche en Santé, de Surveillance Épidémiologique et de Formation (IRESSEF)	Souleymane MBOUP, Abdou PADANE, Abdoulie KANTEH ,Abdul Karim SESAY , Khadim GUEYE, Papa Alassane DIAW, Birahim Piere NDIAYE, Barada CISSE, Aminata MBOUP, Moustapha MBOW, Ndeye Coumba Toure KANE, Nafisatou LEYE, Gora LO, Ambroise AHOUIDI , Astou Gaye GAYE, Aminata DIA, Yacine DIA
EPI_ISL_1168481, EPI_ISL_1168494, EPI_ISL_1168532, EPI_ISL_1168533, EPI_ISL_1168534, EPI_ISL_1168535, EPI_ISL_1168552, EPI_ISL_1168577, EPI_ISL_1168578, EPI_ISL_1168580, EPI_ISL_1168581, EPI_ISL_1168582, EPI_ISL_1168615, EPI_ISL_1168619, EPI_ISL_1168622, EPI_ISL_1168623			
see above	Instituto de Diagnostico y Referencia Epidemiologicos INDRE_RNLSP	Instituto de Diagnostico y Referencia Epidemiologicos (INDRE)	Claudia Wong-Arambula, Abril Rodriguez-Maldonado, Vanessa Rivero-Arredondo, Ariadna Medina-Benitez, Joaquin Quiroz-Mercado,David Fragoso-Fonseca, Sergio Rangel-Guerrero, Natividad Cruz-Ortiz, Tatiana Nunez-Garcia, Gisela Barrera-Badillo, Lucia Hernandez-Rivas, Irma Lopez-Martinez, Ernesto Ramirez-Gonzalez.
EPI_ISL_1168653, EPI_ISL_1168654, EPI_ISL_1168656, EPI_ISL_1168658, EPI_ISL_1168659, EPI_ISL_1168660, EPI_ISL_1168661, EPI_ISL_1168662, EPI_ISL_1168663, EPI_ISL_1168664, EPI_ISL_1168665, EPI_ISL_1168666, EPI_ISL_1168667, EPI_ISL_1168668, EPI_ISL_1168669, EPI_ISL_1168670, EPI_ISL_1168671, EPI_ISL_1168672, EPI_ISL_1168673, EPI_ISL_1168674, EPI_ISL_1168675, EPI_ISL_1168677, EPI_ISL_1168678, EPI_ISL_1168679, EPI_ISL_1168680, EPI_ISL_1168681			
see above	Centers for Disease Control and Prevention, Dengue Branch	Centers for Disease Control and Prevention, Dengue Branch	Gilberto A. Santiago, Glenda Gonzalez, Betzabel Flores, Keyla Charriez, Gabriela Paz-Bailey, Jorge L. Munoz-Jordan
EPI_ISL_1168686	Puerto Rico Department of Health	Centers for Disease Control and Prevention, Dengue Branch	Gilberto A. Santiago, Glenda Gonzalez, Betzabel Flores, Keyla Charriez, Gabriela Paz-Bailey, Jorge L. Munoz-Jordan
EPI_ISL_1169047	RSUP Dr. Mohammad Hoesin Palembang Sumatera Selatan	National Institute of Health Research and Development	Vivi Setiawaty, Hana Apsari Pawestri, Subangkit, Kartika Dewi Puspa, Arie Ardiansyah Nugraha, Hartanti Dian Ikawati, Nelly Puspandari
EPI_ISL_1169048	BBTKLPP Banjarbaru, Kalimantan Selatan	National Institute of Health Research and Development	Vivi Setiawaty, Hana Apsari Pawestri, Subangkit, Kartika Dewi Puspa, Arie Ardiansyah Nugraha, Hartanti Dian Ikawati, Nelly Puspandari
EPI_ISL_1169919	HIV Molecular Laboratory, Ethiopian Public Health Institute	HIV Molecular Laboratory, Ethiopian Public Health Institute	Weldemariam,A.G., Vanaerschot,M., Abichu,G., Kidane,E., Damena,D., Tessema,S.K., Gutema,G., Ahyong,V., Detweiler,A., Tan,M., Neff,N., Fahsbender,E., Kalantar,K., Leta,D. and Tato,C.
EPI_ISL_1170955	National Institute of Health Research and Development	National Institute of Health Research and Development	Yuni Rukminiati, Agustiningsih, Kindi Adam, Holy Arif Wibowo, Ririn Ramadhany, Hana Apsari Pawestri, Subangkit, Kartika Dewi Puspa, Arie Ardiansyah Nugraha, Hartanti Dian Ikawati, Krisna Nur Andriana Pangesti, Ni Ketut Susilarini, Nur Ika Hariastuti, Uly Alfi Nikmah, Mursinah, Asri Febriyani, Reni Herman, Nike Susanti, Herna, Tati Febriyanti, Natalie Laurencia Kipuw, Fauzul Muna, Irene Lorinda Indalao, Aulia Rizki, Nelly Puspandari, Vivi Setiawaty

EPI_ISL_1170956	HIV Molecular Lab, Ethiopian Public Health Institute	HIV Molecular Lab	Weldemariam,A.G., Vanaerschot,M., Abichu,G., Kidane,E., Damena,D., Tessema,S.K., Gutema,G., Ahyong,V., Detweiler,A., Tan,M., Neff,N., Fahsbender,E., Kalantar,K., Leta,D. and Tato,C.
EPI_ISL_1171621	LACEN do Mato Grosso do Sul	Instituto Adolfo Lutz, Interdiciplinary Procedures Center, Strategic Laboratory	Claudio Tavares Sacchi, Claudia Regina Gonçalves, Erica Valessa Ramos Gomes, Karoline Rodrigues Campos, Caio Vinicius Dias Lopes
EPI_ISL_1171622, EPI_ISL_1171623, EPI_ISL_1171624, EPI_ISL_1171625, EPI_ISL_1171627, EPI_ISL_1171629, EPI_ISL_1171630, EPI_ISL_1171631, EPI_ISL_1171632, EPI_ISL_1171633, EPI_ISL_1171635, EPI_ISL_1171636, EPI_ISL_1171637, EPI_ISL_1171639, EPI_ISL_1171640			
see above	IAL Regional de Santos	Instituto Adolfo Lutz, Interdiciplinary Procedures Center, Strategic Laboratory	Claudio Tavares Sacchi, Claudia Regina Gonçalves, Erica Valessa Ramos Gomes, Karoline Rodrigues Campos, Caio Vinicius Dias Lopes
EPI_ISL_1171648, EPI_ISL_1171649, EPI_ISL_1171650	Centro de Saude Dr. Jose Paione em Mococa	Instituto Adolfo Lutz, Interdiciplinary Procedures Center, Strategic Laboratory	Claudio Tavares Sacchi, Claudia Regina Gonçalves, Erica Valessa Ramos Gomes, Karoline Rodrigues Campos, Caio Vinicius Dias Lopes
EPI_ISL_1171668, EPI_ISL_1171669, EPI_ISL_1171670	IAL Regional de Presidente Prudente	Instituto Adolfo Lutz, Interdiciplinary Procedures Center, Strategic Laboratory	Claudio Tavares Sacchi, Claudia Regina Gonçalves, Erica Valessa Ramos Gomes, Karoline Rodrigues Campos, Caio Vinicius Dias Lopes
EPI_ISL_1173202	Laboratorio Biologia Molecolare Sars Cov2 - UOC Laboratorio Analisi - Servizio Medicina di Laboratorio, Ospedale "San Francesco" - ATS-ASSL Nuoro	Laboratorio Specialistico Ematologia, UOC Ematologia, Ospedale "San Francesco" - ATS-ASSL Nuoro	Pira Giovanna, Rosu Valentina, Fancello Patrizia, Asproni Rosanna, Carta Franco, Serra Silvia, Piras Giovanna, Monne Maria Itria, Malune Paolo, Fiamma Maura, Palmas Angelo Domenico, Lo Maglio Iana, Mameli Giuseppe
EPI_ISL_1180250, EPI_ISL_1180252, EPI_ISL_1180253	Laboratorio Biologia Molecolare Sars Cov2 - UOC Laboratorio Analisi - Servizio Medicina di Laboratorio, Ospedale "San Francesco" - ATS-ASSL Nuoro	Laboratorio specialistico UOC Ematologia - Ospedale "San Francesco" - ATS-ASSL Nuoro	Piras Giovanna, Asproni Rosanna,,Fiamma Maura, Monne Maria Itria, Palmas Angelo Domenico, Lo Maglio Iana, Mameli Giuseppe
EPI_ISL_1180672, EPI_ISL_1180673	Institut für Pathologie, Salzkammergut Klinikum Vöcklabruck	Bergthaler laboratory, CeMM Research Center for Molecular Medicine of the Austrian Academy of Sciences	Lukas Endler, Anna Schedl, Fabian Amman, Thomas Penz, Benedikt Agerer, Maelle Le Moing, Michael Schuster, Bekir Erguner, Jan Laine, Martin Senekowitsch, Christoph Bock, Andreas Bergthaler
EPI_ISL_1180675, EPI_ISL_1180677, EPI_ISL_1180678, EPI_ISL_1180679, EPI_ISL_1180680, EPI_ISL_1180681, EPI_ISL_1180682, EPI_ISL_1180683, EPI_ISL_1180684, EPI_ISL_1180685, EPI_ISL_1180686, EPI_ISL_1180687, EPI_ISL_1180688, EPI_ISL_1180689, EPI_ISL_1180690, EPI_ISL_1180692, EPI_ISL_1180693, EPI_ISL_1180694, EPI_ISL_1180696, EPI_ISL_1180698, EPI_ISL_1180699, EPI_ISL_1180700, EPI_ISL_1180701, EPI_ISL_1180702, EPI_ISL_1180703, EPI_ISL_1180704, EPI_ISL_1180705, EPI_ISL_1180706, EPI_ISL_1180707, EPI_ISL_1180708, EPI_ISL_1180709, EPI_ISL_1180710, EPI_ISL_1180711, EPI_ISL_1180712, EPI_ISL_1180713, EPI_ISL_1180714			
see above	Public Health Authority of the Slovak Republic	Bergthaler laboratory, CeMM Research Center for Molecular Medicine of the Austrian Academy of Sciences	Lukas Endler, Anna Schedl, Fabian Amman, Thomas Penz, Benedikt Agerer, Maelle Le Moing, Michael Schuster, Bekir Erguner, Jan Laine, Martin Senekowitsch, Christoph Bock, Andreas Bergthaler
EPI_ISL_1180881, EPI_ISL_1180882, EPI_ISL_1180883, EPI_ISL_1180884	Department of Laboratory Medicine, Division of Clinical Virology, University of Medicine, Vienna	Bergthaler laboratory, CeMM Research Center for Molecular Medicine of the Austrian Academy of Sciences	Lukas Endler, Anna Schedl, Fabian Amman, Thomas Penz, Benedikt Agerer, Maelle Le Moing, Michael Schuster, Bekir Erguner, Jan Laine, Martin Senekowitsch, Christoph Bock, Andreas Bergthaler
EPI_ISL_1181360, EPI_ISL_1181381, EPI_ISL_1181382, EPI_ISL_1181383	Laboratory of Respiratory Viruses and Measles, Oswaldo Cruz Institute, FIOCRUZ	Laboratory of Respiratory Viruses and Measles, Oswaldo Cruz Institute, FIOCRUZ	Paola Resende, Luciana Appolinario, Fernando Motta, Anna Carolina Paixao, Ana Carolina Mendonca, Alice Sampaio Rocha, Renata Serrano Lopes, Marilda Siqueira on behalf of the Fiocruz COVID-19 Genomic Surveillance Network
EPI_ISL_1181434	Laboratorio Central de Saude Publica do Estado do Rio Grande do Sul (LACEN-RS)	Laboratory of Respiratory Viruses and Measles, Oswaldo Cruz Institute, FIOCRUZ	Paola Resende, Luciana Appolinario, Fernando Motta, Anna Carolina Paixao, Ana Carolina Mendonca, Alice Sampaio Rocha, Renata Serrano Lopes, Tatiana Schaffer Gregianini, Marilda Siqueira on behalf of the Fiocruz COVID-19 Genomic Surveillance Network
EPI_ISL_1181446	Laboratory of Respiratory Viruses and Measles, Oswaldo Cruz Institute, FIOCRUZ	Laboratory of Respiratory Viruses and Measles, Oswaldo Cruz Institute, FIOCRUZ	Paola Resende, Luciana Appolinario, Fernando Motta, Anna Carolina Paixao, Ana Carolina Mendonca, Alice Sampaio Rocha, Renata Serrano Lopes, Marilda Siqueira on behalf of the Fiocruz COVID-19 Genomic Surveillance Network
EPI_ISL_1181451	Laboratorio Central de Saude Publica do Estado do Rio Grande do Sul (LACEN-RS)	Laboratory of Respiratory Viruses and Measles, Oswaldo Cruz Institute, FIOCRUZ	Paola Resende, Luciana Appolinario, Fernando Motta, Anna Carolina Paixao, Ana Carolina Mendonca, Alice Sampaio Rocha, Renata Serrano Lopes, Tatiana Schaffer Gregianini, Marilda Siqueira on behalf of the Fiocruz COVID-19 Genomic Surveillance Network
EPI_ISL_1186028	Institute for Medical Research, Infectious Disease Research Centre, National Institutes of Health, Ministry of Health Malaysia	Institute for Medical Research, Infectious Disease Research Centre, National Institutes of Health, Ministry of Health Malaysia	Suppiah J, Kamel K, Mohd Zawawi Z, Ramly N, Robert F, Thayan R
EPI_ISL_1190761, EPI_ISL_1190762, EPI_ISL_1190765, EPI_ISL_1190766, EPI_ISL_1190767, EPI_ISL_1190768	CREMER(Centre de Rechercherches sur les Maladies Emergentes et Ré-émergentes)	TransVIHMI(Recherches Translationnelles sur le VIH et les Maladies Infectieuses)	Celestin Godwe, Christelle Butel, Martin Maidadi Foudi, Laetitia Serrano, Dowbiss Meta Djomsi, Nicole Vidal, Esemu Livo, Rodrigue Kamga, Marie Amougou , Eitel Mpoudi Ngole,Ahidjo Ayouba, Marcel Tongo, Martine Peeters, Eric Delaporte
EPI_ISL_1191141, EPI_ISL_1191736, EPI_ISL_1191737	Laboratorio Biologia Molecolare Sars Cov2 - UOC Laboratorio Analisi - Servizio Medicina di Laboratorio, Ospedale "San Francesco" - ATS-ASSL Nuoro	Laboratorio specialistico UOC Ematologia - Ospedale "San Francesco" - ATS-ASSL Nuoro	Piras Giovanna, Asproni Rosanna,,Fiamma Maura, Monne Maria Itria, Palmas Angelo Domenico, Lo Maglio Iana, Mameli Giuseppe
EPI_ISL_1196282	LACEN do Mato Grosso do Sul	Instituto Adolfo Lutz, Interdiciplinary Procedures Center, Strategic Laboratory	Claudio Tavares Sacchi, Claudia Regina Gonçalves, Erica Valessa Ramos Gomes, Karoline Rodrigues Campos, Caio Vinicius Dias Lopes
EPI_ISL_1196285, EPI_ISL_1196286, EPI_ISL_1196287, EPI_ISL_1196288, EPI_ISL_1196289, EPI_ISL_1196290, EPI_ISL_1196291, EPI_ISL_1196292, EPI_ISL_1196293, EPI_ISL_1196294	LACEN do Distrito Federal	Instituto Adolfo Lutz, Interdiciplinary Procedures Center, Strategic Laboratory	Claudio Tavares Sacchi, Claudia Regina Gonçalves, Erica Valessa Ramos Gomes, Karoline Rodrigues Campos, Caio Vinicius Dias Lopes
EPI_ISL_1201427, EPI_ISL_1201428, EPI_ISL_1201429, EPI_ISL_1201430	Laboratory of Genetics and Personalized Medicine, Zan Mitrev Clinic	Laboratory of Genetics and Personalized Medicine, Zan Mitrev Clinic	Kungulovski G. et al.
EPI_ISL_1201480	AZ St.Jan Brugge-Oostende	AZ SINT-JAN BRUGGE	Marijke Reynders, Patrick Descheemaeker, Laurien Hoornaert, Thomas Van Landschoot, Jorn Hellemans
EPI_ISL_1201488, EPI_ISL_1201489, EPI_ISL_1201490, EPI_ISL_1201491, EPI_ISL_1201492, EPI_ISL_1201493, EPI_ISL_1201494, EPI_ISL_1201495, EPI_ISL_1201496, EPI_ISL_1201497, EPI_ISL_1201498, EPI_ISL_1201499, EPI_ISL_1201500, EPI_ISL_1201501, EPI_ISL_1201502, EPI_ISL_1201503, EPI_ISL_1201504, EPI_ISL_1201505, EPI_ISL_1201506			
see above	Hopital	National Reference Center for Viruses of Respiratory Infections, Institut Pasteur, Paris	Marion Barbet, Sylvie Behillil, Méline Bizard, Angela Brisebarre, Camille Capel, Etienne Simon-Lorière, Vincent Enouf, Maud Vanpeene, Sylvie van der Werf,Gastli Nabil
EPI_ISL_1201535, EPI_ISL_1201536	Outre Mer	National Reference Center for Viruses of Respiratory Infections, Institut Pasteur, Paris	Marion Barbet, Sylvie Behillil, Méline Bizard, Angela Brisebarre, Camille Capel, Etienne Simon-Lorière, Vincent Enouf, Maud Vanpeene, Sylvie van der Werf,Rousset Dominique
EPI_ISL_1201894	Diagnosticos da America - DASA	Instituto Adolfo Lutz, Interdiciplinary Procedures Center, Strategic Laboratory	Claudio Tavares Sacchi, Claudia Regina Gonçalves, Erica Valessa Ramos Gomes, Karoline Rodrigues Campos, Caio Vinicius Dias Lopes
EPI_ISL_1204503	Institute for Medical Research, Infectious Disease Research Centre, National Institutes of Health, Ministry of Health Malaysia	Institute for Medical Research, Infectious Disease Research Centre, National Institutes of Health, Ministry of Health Malaysia	Suppiah J, Kamel K, Mohd Zawawi Z, Ramly N, Robert F, Thayan R
EPI_ISL_1205468	Institut de Virologie du CHU de Strasbourg	Institut de Virologie du CHU de Strasbourg	Fafi-Kremer Samira, Gallais Floriane, Gantner Pierre, Laugel Elodie, Solis Morgane, Velay Aurélie, Wendling Marie-Josée
EPI_ISL_1208949	"Scientific and Practical Center for Sanitary and Epidemiological Expertise and Monitoring" branch of the RSE on the REU "National Center for Public Health" of the Ministry of Health RK	Kazakh National Agrarian University (KazNAU); TreeGene LLP Genetic Laboratory; National Scientific Center for Especially Dangerous Infections (NSCEDI)	Beloussov Vyacheslav, Strochkov Vitaliy, Tabynov Kaissar, Sandybayev Nurlan, Tabynov Kairat, Turebekov Nurkeldy, Granica Joanna, Yerubayev Toktassyn, Yespolov Tlektes
EPI_ISL_1208951	"Scientific and Practical Center for Sanitary and Epidemiological Expertise and Monitoring" branch of the RSE on the REU "National Center for Public Health" of the Ministry of Health RK	Kazakh National Agrarian University (KazNAU); TreeGene LLP Genetic Laboratory; National Scientific Center for Especially Dangerous Infections (NSCEDI)	Tabynov Kaissar, Sandybayev Nurlan, Tabynov Kairat, Turebekov Nurkeldy, Granica Joanna, Yerubayev Toktassyn, Beloussov Vyacheslav, Strochkov Vitaliy, Yespolov Tlektes

EPI_ISL_1209243, EPI_ISL_1209244, EPI_ISL_1209245, EPI_ISL_1209246, EPI_ISL_1209247, EPI_ISL_1209248, EPI_ISL_1209249, EPI_ISL_1209250, EPI_ISL_1209251, EPI_ISL_1209252, EPI_ISL_1209253, EPI_ISL_1209254, EPI_ISL_1209255, EPI_ISL_1209256, EPI_ISL_1209258, EPI_ISL_1209259, EPI_ISL_1209260, EPI_ISL_1209261, EPI_ISL_1209262			
see above	Public Health Authority of the Slovak Republic	Bergthaler laboratory, CeMM Research Center for Molecular Medicine of the Austrian Academy of Sciences	Lukas Endler, Anna Schedl, Fabian Amman, Thomas Penz, Benedikt Agerer, Maelle Le Moing, Michael Schuster, Bekir Erguner, Jan Laine, Martin Senekowitsch, Christoph Bock, Andreas Bergthaler
EPI_ISL_1209299, EPI_ISL_1209300, EPI_ISL_1209301, EPI_ISL_1209302, EPI_ISL_1209303, EPI_ISL_1209304, EPI_ISL_1209305, EPI_ISL_1209306, EPI_ISL_1209307, EPI_ISL_1209308, EPI_ISL_1209309, EPI_ISL_1209310, EPI_ISL_1209311, EPI_ISL_1209312, EPI_ISL_1209313, EPI_ISL_1209314, EPI_ISL_1209315, EPI_ISL_1209316, EPI_ISL_1209317, EPI_ISL_1209318, EPI_ISL_1209319, EPI_ISL_1209320, EPI_ISL_1209321, EPI_ISL_1209322, EPI_ISL_1209323, EPI_ISL_1209324, EPI_ISL_1209325, EPI_ISL_1209326, EPI_ISL_1209327, EPI_ISL_1209328, EPI_ISL_1209329, EPI_ISL_1209330, EPI_ISL_1209331, EPI_ISL_1209332, EPI_ISL_1209333, EPI_ISL_1209334			
see above	Department of Laboratory Medicine, Division of Clinical Virology, University of Medicine, Vienna	Bergthaler laboratory, CeMM Research Center for Molecular Medicine of the Austrian Academy of Sciences	Lukas Endler, Anna Schedl, Fabian Amman, Thomas Penz, Benedikt Agerer, Maelle Le Moing, Michael Schuster, Bekir Erguner, Jan Laine, Martin Senekowitsch, Christoph Bock, Andreas Bergthaler
EPI_ISL_1212673, EPI_ISL_1212675, EPI_ISL_1212677, EPI_ISL_1212679, EPI_ISL_1212681	Istituto Zooprofilattico Sperimentale Umbria e Marche "Togo Rosati"	Istituto Zooprofilattico Sperimentale dell'Abruzzo e Molise "G. Caporale"	Biagetti M., Giammarioli M., Lorusso A, Marcacci M, Di Domenico M, Ancora M, Curini V, Mangone I, Rinaldi A, Scialabba S, Di Pasquale A, Cammà C, Puglia I, Calistri P, Savini G
EPI_ISL_1219707, EPI_ISL_1219710	Laboratorio Biologia Molecolare Sars Cov2 - UOC Laboratorio Analisi - Servizio Medicina di Laboratorio, Ospedale "San Francesco" - ATS-ASSL Nuoro	Laboratorio specialistico UOC Ematologia - Ospedale "San Francesco" - ATS-ASSL Nuoro	Piras Giovanna, Asproni Rosanna,,Fiamma Maura, Monne Maria Itria, Palmas Angelo Domenico, Lo Maglio Iana, Mameli Giuseppe
EPI_ISL_1219714	CICSaB/UASLP/LESP SLP	Instituto de Diagnostico y Referencia Epidemiologicos (INDRE)	Claudia Wong-Arambula, Abril Rodriguez-Maldonado, Vanessa Rivero-Arredondo, Ariadna Medina-Benitez, Joaquin Quiroz-Mercado,David Fragoso-Fonseca, Sergio Rangel-Guerrero, Andreu Comas-Garcia, Natividad Cruz-Ortiz, Tatiana Nunez-Garcia, Gisela Barrera-Badillo, Lucia Hernandez-Rivas, Irma Lopez-Martinez, Ernesto Ramirez-Gonzalez.
EPI_ISL_1219930, EPI_ISL_1219931	Hopital	National Reference Center for Viruses of Respiratory Infections, Institut Pasteur, Paris	Marion Barbet, Sylvie Behillil, Méline Bizard, Angela Brisebarre, Camille Capel, Etienne Simon-Lorière, Vincent Enouf, Maud Vanpeene, Sylvie van der Werf,Guiard Carine
EPI_ISL_1219942	Hopital	National Reference Center for Viruses of Respiratory Infections, Institut Pasteur, Paris	Marion Barbet, Sylvie Behillil, Méline Bizard, Angela Brisebarre, Camille Capel, Etienne Simon-Lorière, Vincent Enouf, Maud Vanpeene, Sylvie van der Werf,Pilorge LéA
EPI_ISL_1219961	Labo Analyses Med	National Reference Center for Viruses of Respiratory Infections, Institut Pasteur, Paris	Marion Barbet, Sylvie Behillil, Méline Bizard, Angela Brisebarre, Camille Capel, Etienne Simon-Lorière, Vincent Enouf, Maud Vanpeene, Sylvie van der Werf,Oliel Jonathan
EPI_ISL_1220057	LABORATORIO BIOLOGIA MOLECULAR IMAT SAS	Instituto Nacional de Salud- Dirección de Investigación en Salud Pública	Katherine Laiton-Donato, Carlos Franco-Muñoz, Diego A. Álvarez-Díaz, Hector Alejandro Ruiz-Moreno, Jhonnatan Reales-González, Diego Andrés Prada, Sheryll Corchuelo, Maria T. Herrera-Sepúlveda, Julian Naizaque, Gerardo Santamaría, Magdalena Wiesner, Martha Lucia Ospina Martínez, Marcela Mercado-Reyes.
EPI_ISL_1220060	FUNDACION VALLE DEL LILI	Instituto Nacional de Salud- Dirección de Investigación en Salud Pública	Katherine Laiton-Donato, Carlos Franco-Muñoz, Diego A. Álvarez-Díaz, Hector Alejandro Ruiz-Moreno, Jhonnatan Reales-González, Diego Andrés Prada, Sheryll Corchuelo, Maria T. Herrera-Sepúlveda, Julian Naizaque, Gerardo Santamaría, Magdalena Wiesner, Martha Lucia Ospina Martínez, Marcela Mercado-Reyes.
EPI_ISL_1222779	Institut de Virologie du CHU de Strasbourg	Institut de Virologie du CHU de Strasbourg	Fafi-Kremer Samira, Gallais Floriane, Gantner Pierre, Laugel Elodie, Solis Morgane, Velay Aurélie, Wendling Marie-Josée
EPI_ISL_1225156, EPI_ISL_1225157, EPI_ISL_1225158, EPI_ISL_1225160, EPI_ISL_1225161	Turkish Society of Internal Medicine	Gen Era Diagnostics Corporation Life Science Research and Molecular Diagnostics	Serhat Ünal
EPI_ISL_1229144, EPI_ISL_1229145, EPI_ISL_1229146, EPI_ISL_1229147, EPI_ISL_1229148, EPI_ISL_1229149	Laboratorio Biologia Molecolare Sars Cov2 - UOC Laboratorio Analisi - Servizio Medicina di Laboratorio, Ospedale "San Francesco" - ATS-ASSL Nuoro	Laboratorio specialistico UOC Ematologia - Ospedale "San Francesco" - ATS-ASSL Nuoro	Giovanna Piras, Rosanna Asproni, Maura Fiamma, Maria Itria Monne, Angelo Domenico Palmas, Iana Lo Maglio, Giuseppe Mameli
EPI_ISL_1233440, EPI_ISL_1233441, EPI_ISL_1233443, EPI_ISL_1233444, EPI_ISL_1233445, EPI_ISL_1233446, EPI_ISL_1233447, EPI_ISL_1233448, EPI_ISL_1233449, EPI_ISL_1233450, EPI_ISL_1233451, EPI_ISL_1233452, EPI_ISL_1233453, EPI_ISL_1233454, EPI_ISL_1233455, EPI_ISL_1233456, EPI_ISL_1233457, EPI_ISL_1233459, EPI_ISL_1233460, EPI_ISL_1233461, EPI_ISL_1233462, EPI_ISL_1233463, EPI_ISL_1233464, EPI_ISL_1233465, EPI_ISL_1233466, EPI_ISL_1233467, EPI_ISL_1233469, EPI_ISL_1233470, EPI_ISL_1233471, EPI_ISL_1233472, EPI_ISL_1233473, EPI_ISL_1233474, EPI_ISL_1233475, EPI_ISL_1233476, EPI_ISL_1233477, EPI_ISL_1233509, EPI_ISL_1233510, EPI_ISL_1233511, EPI_ISL_1233512, EPI_ISL_1233513, EPI_ISL_1233514, EPI_ISL_1233515, EPI_ISL_1233516, EPI_ISL_1233517, EPI_ISL_1233518, EPI_ISL_1233519, EPI_ISL_1233520, EPI_ISL_1233521, EPI_ISL_1233522, EPI_ISL_1233523, EPI_ISL_1233524, EPI_ISL_1233525, EPI_ISL_1233526, EPI_ISL_1233527, EPI_ISL_1233528, EPI_ISL_1233529, EPI_ISL_1233530, EPI_ISL_1233531, EPI_ISL_1233532, EPI_ISL_1233533, EPI_ISL_1233534, EPI_ISL_1233535, EPI_ISL_1233536, EPI_ISL_1233537, EPI_ISL_1233538, EPI_ISL_1233539, EPI_ISL_1233540, EPI_ISL_1233541, EPI_ISL_1233542, EPI_ISL_1233543, EPI_ISL_1233544, EPI_ISL_1233623, EPI_ISL_1233624, EPI_ISL_1233625, EPI_ISL_1233626, EPI_ISL_1233627			
see above	National Center for Infectious and Parasitic Diseases (NCIPD)	National Center for Infectious and Parasitic Diseases (NCIPD)	Alexiev et al
EPI_ISL_1235678, EPI_ISL_1235682, EPI_ISL_1235683	Baylor Scott & White-Temple	Baylor Scott & White-Temple	Ari Rao, Linden Morales, Kimberly Walker, Marcus Volz, Shelby Hendrickson, Caitlin Maloney
EPI_ISL_1238792, EPI_ISL_1238793	Ecological and Evolutionary Genomics. UGA-CINVESTAV	Unidad Universitaria de Secuenciación Masiva y Bioinformática (UUSMB). IBT-UNAM	Angelica Cibrian
EPI_ISL_1239145, EPI_ISL_1239216, EPI_ISL_1239217, EPI_ISL_1239219	National Institute of Health Research and Development	National Institute of Health Research and Development	Hana Apsari Pawestri, Subangkit, Kartika Dewi Puspa, Arie Ardiansyah Nugraha, Hartanti Dian Ikawati, Krisna Nur Andriana Pangesti, Yuni Rukminiati, Ririn Ramadhany, Agustiningsih, Kindi Adam, Holy Arif Wibowo, Triyani Soekarso, Ni Ketut Susilarini, Nurika Hariastuti, Ully Alfi Nikmah, Reni Herman, Nike Susanti, Herna, Tati Febriyanti, Natalie Laurencia Kipuw, Fauzul Muna, Irene Lorinda Indalao, Nelly Puspandari, Vivi Setiawaty.
EPI_ISL_1239220	RSUPN Dr. Cipto Mangunkusumo	National Institute of Health Research and Development	Hana Apsari Pawestri, Subangkit, Kartika Dewi Puspa, Arie Ardiansyah Nugraha, Hartanti Dian Ikawati, Krisna Nur Andriana Pangesti, Yuni Rukminiati, Ririn Ramadhany, Agustiningsih, Kindi Adam, Holy Arif Wibowo, Triyani Soekarso, Ni Ketut Susilarini, Nurika Hariastuti, Ully Alfi Nikmah, Reni Herman, Nike Susanti, Herna, Tati Febriyanti, Natalie Laurencia Kipuw, Fauzul Muna, Irene Lorinda Indalao, Nelly Puspandari, Vivi Setiawaty.
EPI_ISL_1239221, EPI_ISL_1239222	National Institute of Health Research and Development	National Institute of Health Research and Development	Hana Apsari Pawestri, Subangkit, Kartika Dewi Puspa, Arie Ardiansyah Nugraha, Hartanti Dian Ikawati, Krisna Nur Andriana Pangesti, Yuni Rukminiati, Ririn Ramadhany, Agustiningsih, Kindi Adam, Holy Arif Wibowo, Triyani Soekarso, Ni Ketut Susilarini, Nurika Hariastuti, Ully Alfi Nikmah, Reni Herman, Nike Susanti, Herna, Tati Febriyanti, Natalie Laurencia Kipuw, Fauzul Muna, Irene Lorinda Indalao, Nelly Puspandari, Vivi Setiawaty.
EPI_ISL_1250222	Clinical Microbiology Lab RSPTN Universitas Hasanuddin	Clinical Microbiology Lab RSPTN Universitas Hasanuddin	Muhammad Nasrum Massi, Rizalinda Sjahril, Gita Vita Soraya, Triwibawa, Handayani Halik, Muhammad Yusuf Usman, Rufika Shari
EPI_ISL_1251274, EPI_ISL_1251275	"Stefan S. Nicolau"Institute of Virology	"Stefan S. Nicolau"Institute of Virology	Anca Botezatu, Iulia Virginia Iancu, Coralia Bleotu, Mihaela Economescu, Laura Necula, Denisa Dragu, Lilia Matei, Ioana Pitica, Marinela Bostan, Mirela Mihaila, Laura Grecu, Camelia Sultana, Simona Ruta, Ana Iulia Neagu, Adriana Plesa, Saviana Nedeianu, Alina Nastasie, Cristina Mambet,Gabriela Anton, Carmen Cristina Diaconu
EPI_ISL_1254648	Ospedale di Venere	Istituto Zooprofilattico Sperimentale della Puglia e della Basilicata	Parisi A., Bianco A., Capozzi L., Del Sambro L., Simone D., Iacobellis M., Ridolfi D., Giannico A.
EPI_ISL_1255105	West African Centre for Cell Biology of Infectious Pathogens (WACCBIP), University of Ghana, Volta Road, Legon-Accra, Ghana	West African Centre for Cell Biology of Infectious Pathogens (WACCBIP), University of Ghana, Volta Road, Legon-Accra, Ghana	Collins M. Morang'a, Joyce M. Ngoi, Evelyn B. Quansah, Samirah Saiid, Dominic S.Y. Amuzu, Vincent Appiah, Philip M. Soglo, Vanessa Magnussen, Aisha Mohammed, Kesego Tapela, Nelson Kibinge, Abdoulaye B Diallo, Frederick Kumi-Ansah, Theophilus Odoom, Oliver D Boakye5, Emmanuella Amoako4, Abdul-Karim Abass, , Samuel Kaba Akoriyea, Frederick Tei-Maya, Lucas N. Amenga-Etego, Dam Kenneth Mibut, Yaw Bediako, Benjamin Demah Nuertey, Gordon A Awandare, Peter K Quashie, Gordon A Awandare, Yaw Bediako
EPI_ISL_1255123, EPI_ISL_1255124, EPI_ISL_1255125, EPI_ISL_1255126, EPI_ISL_1255127, EPI_ISL_1255128, EPI_ISL_1255129, EPI_ISL_1255130, EPI_ISL_1255131, EPI_ISL_1255132, EPI_ISL_1255133, EPI_ISL_1255134, EPI_ISL_1255135, EPI_ISL_1255136, EPI_ISL_1255137, EPI_ISL_1255138, EPI_ISL_1255139, EPI_ISL_1255184, EPI_ISL_1255185			
see above	West African Centre for Cell Biology of Infectious Pathogens (WACCBIP), University of Ghana, Accra, Ghana	West African Centre for Cell Biology of Infectious Pathogens (WACCBIP), University of Ghana, Volta Road, Legon-Accra, Ghana	Collins M. Morang'a, Joyce M. Ngoi, Evelyn B. Quansah, Samirah Saiid, Dominic S.Y. Amuzu, Vincent Appiah, Philip M. Soglo, Vanessa Magnussen, Aisha Mohammed, Kesego Tapela, Nelson Kibinge, Abdoulaye B Diallo, Frederick Kumi-Ansah, Theophilus Odoom, Oliver D Boakye5, Emmanuella Amoako4, Abdul-Karim Abass, , Samuel Kaba Akoriyea, Frederick Tei-Maya, Lucas N. Amenga-Etego, Dam Kenneth Mibut, Yaw Bediako, Benjamin Demah Nuertey, Gordon A Awandare, Peter K Quashie, Gordon A Awandare, Yaw Bediako

EPI_ISL_1257818, EPI_ISL_1257819, EPI_ISL_1257820, EPI_ISL_1257822	Balai Litbangkes Papua	National Institute of Health Research and Development	Hana Apsari Pawestri, Subangkit, Kartika Dewi Puspa, Arie Ardiansyah Nugraha, Hartanti Dian Ikawati, Krisna Pangesti, Nelly Puspandari, Hotma Sitompul, Antonius Octavian, Vivi Setiawaty
EPI_ISL_1257823, EPI_ISL_1257824, EPI_ISL_1257825, EPI_ISL_1257826	RSUP DR Mohammad Hoesin	National Institute of Health Research and Development	Hana Apsari Pawestri, Subangkit, Kartika Dewi Puspa, Arie Ardiansyah Nugraha, Hartanti Dian Ikawati, Krisna Pangesti, Nelly Puspandari, Eny Rahmawati, Rogaya, Vivi Setiawaty
EPI_ISL_1257832	BTKL-PP Ambon	National Institute of Health Research and Development	Vivi Setiawaty, Hana Apsari Pawestri, Subangkit, Kartika Dewi Puspa, Arie Ardiansyah Nugraha, Hartanti Dian Ikawati, Nelly Puspandari, Krisna Pangesti
EPI_ISL_1257851, EPI_ISL_1257852, EPI_ISL_1257853	BBTKLPP Banjarbaru	National Institute of Health Research and Development	Hartanti Dian Ikawati, Hana Apsari Pawestri, Subangkit, Kartika Dewi Puspa, Arie Ardiansyah Nugraha, Krisna Pangesti, Nelly Puspandari, Dewi Hermawati, Mutia Raudah, Vivi Setiawaty
EPI_ISL_1257860	Farmalab Indoutama	National Institute of Health Research and Development	Hana Apsari Pawestri, Subangkit, Kartika Dewi Puspa, Arie Ardiansyah Nugraha, Hartanti Dian Ikawati, Nelly Puspandari, Krisna Pangesti, Vivi Setiawaty
EPI_ISL_1257863	RSUP Dr. Sardjito	National Institute of Health Research and Development	Kartika Dewi Puspa, Hana Apsari Pawestri, Subangkit, Arie Ardiansyah Nugraha, Hartanti Dian Ikawati, Nelly Puspandari, Krisna Pangesti, Vivi Setiawaty
EPI_ISL_1257864	BTKL-PP Ambon	National Institute of Health Research and Development	Kartika Dewi Puspa, Hana Apsari Pawestri, Subangkit, Arie Ardiansyah Nugraha, Hartanti Dian Ikawati, Nelly Puspandari, Krisna Pangesti, Vivi Setiawaty
EPI_ISL_1257875, EPI_ISL_1257878, EPI_ISL_1257879, EPI_ISL_1257880	Centre Pasteur du Cameroun	Institut Pasteur de Dakar	Njouom Richard, Diagne Moussa Moïse, Dia Ndongo, Diallo Amadou, Sankhe Safietou, Diop Mamadou, Loucoubar Cheikh, Elisabeth Carniel, Faye Ousmane, Sall Amadou Alpha
EPI_ISL_1258423, EPI_ISL_1258424	Clinical Microbiology Lab RSPTN Universitas Hasanuddin	Clinical Microbiology Lab RSPTN Universitas Hasanuddin	Muhammad Nasrum Massi, Rizalinda Sjahril, Gita Vita Soraya, Triwibawa, Handayani, Muhammad Yusuf Usman, Rufika Shari
EPI_ISL_1258453	National Institute of Infectious Diseases-Prof. Dr. Matei Bals Molecular Diagnostics Laboratory	National Institute of Infectious Diseases-Prof. Dr. Matei Bals Molecular Diagnostics Laboratory	Corina Casangiu, Leontina Banica, Marius Surleac, Ovidiu Vlaicu, Ionelia Nicolae, Simona Paraschiv, Dan Otelea
EPI_ISL_1258463, EPI_ISL_1258464, EPI_ISL_1258465	Clinical Microbiology Lab RSPTN Universitas Hasanuddin	Clinical Microbiology Lab RSPTN Universitas Hasanuddin	Muhammad Nasrum Massi, Rizalinda Sjahril, Gita Vita Soraya, Triwibawa, Handayani Halik, Muhammad Yusuf Usman, Rufika Shari
EPI_ISL_1259297	Hopital	National Reference Center for Viruses of Respiratory Infections, Institut Pasteur, Paris	Marion Barbet, Sylvie Behillil, Méline Bizard, Angela Brisebarre, Camille Capel, Etienne Simon-Lorière, Vincent Enouf, Maud Vanpeene, Sylvie van der Werf,Dufour Mj
EPI_ISL_1259304, EPI_ISL_1259305, EPI_ISL_1259306	Hopital	National Reference Center for Viruses of Respiratory Infections, Institut Pasteur, Paris	Marion Barbet, Sylvie Behillil, Méline Bizard, Angela Brisebarre, Camille Capel, Etienne Simon-Lorière, Vincent Enouf, Maud Vanpeene, Sylvie van der Werf,Gastli Nabil
EPI_ISL_1259309	Labo Analyses Med	National Reference Center for Viruses of Respiratory Infections, Institut Pasteur, Paris	Marion Barbet, Sylvie Behillil, Méline Bizard, Angela Brisebarre, Camille Capel, Etienne Simon-Lorière, Vincent Enouf, Maud Vanpeene, Sylvie van der Werf
EPI_ISL_1260862, EPI_ISL_1260871	"Stefan S. Nicolau"Institute of Virology	"Stefan S. Nicolau"Institute of Virology	Anca Botezatu, Iulia Virginia Iancu, Coralia Bleotu, Mihaela Economescu, Laura Necula, Denisa Dragu, Lilia Matei, Ioana Pitica, Marinela Bostan, Mirela Mihaila, Laura Grecu, Camelia Sultana, Simona Ruta, Ana Iulia Neagu, Adriana Plesa, Saviana Nedeianu, Alina Nastasie, Cristina Mambet,Gabriela Anton, Carmen Cristina Diaconu
EPI_ISL_1262760, EPI_ISL_1262761, EPI_ISL_1262762	Hopital	National Reference Center for Viruses of Respiratory Infections, Institut Pasteur, Paris	Marion Barbet, Sylvie Behillil, Méline Bizard, Angela Brisebarre, Camille Capel, Etienne Simon-Lorière, Vincent Enouf, Maud Vanpeene, Sylvie van der Werf,Pilorge LéA
EPI_ISL_1262814	Labo Analyses Med	National Reference Center for Viruses of Respiratory Infections, Institut Pasteur, Paris	Marion Barbet, Sylvie Behillil, Méline Bizard, Angela Brisebarre, Camille Capel, Etienne Simon-Lorière, Vincent Enouf, Maud Vanpeene, Sylvie van der Werf,Lebaupain Aude
EPI_ISL_1262816, EPI_ISL_1262817, EPI_ISL_1262818	Hopital	National Reference Center for Viruses of Respiratory Infections, Institut Pasteur, Paris	Marion Barbet, Sylvie Behillil, Méline Bizard, Angela Brisebarre, Camille Capel, Etienne Simon-Lorière, Vincent Enouf, Maud Vanpeene, Sylvie van der Werf,Louis Mathilde
EPI_ISL_1265373	"Stefan S. Nicolau"Institute of Virology	"Stefan S. Nicolau"Institute of Virology	Anca Botezatu, Iulia Virginia Iancu, Coralia Bleotu, Mihaela Economescu, Laura Necula, Denisa Dragu, Lilia Matei, Ioana Pitica, Marinela Bostan, Mirela Mihaila, Laura Grecu, Camelia Sultana, Simona Ruta, Ana Iulia Neagu, Adriana Plesa, Saviana Nedeianu, Alina Nastasie, Cristina Mambet,Gabriela Anton, Carmen Cristina Diaconu
EPI_ISL_1273443	Department of Virology, Bangabandhu Sheikh Mujib Medical University	Bioinformatics Division, National Institute of Biotechnology (NIB)	Munira Jahan, Arittra Bhattacharjee, Raad Rahmat, S M Rashedul Islam, Tahmina Akhter, Ishtiaque Ahammad, Mohammad Uzzal Hossain, Md. Salimullah, Saif Ullah Munshi
EPI_ISL_1279948, EPI_ISL_1279950, EPI_ISL_1279951, EPI_ISL_1279952, EPI_ISL_1279953, EPI_ISL_1279954, EPI_ISL_1279956, EPI_ISL_1279957, EPI_ISL_1279958, EPI_ISL_1279959, EPI_ISL_1279960, EPI_ISL_1279962, EPI_ISL_1279963, EPI_ISL_1279964, EPI_ISL_1279966, EPI_ISL_1279967, EPI_ISL_1279968, EPI_ISL_1279970, EPI_ISL_1279973, EPI_ISL_1279974, EPI_ISL_1279975, EPI_ISL_1279976, EPI_ISL_1279977			
see above	National Institute of Infectious Diseases-Prof. Dr. Matei Bals Molecular Diagnostics Laboratory	National Institute of Infectious Diseases-Prof. Dr. Matei Bals Molecular Diagnostics Laboratory	Leontina Banica, Marius Surleac, Corina Casangiu, Petre Milu, Andreea Tudor, Simona Paraschiv, Dan Otelea
EPI_ISL_1281568, EPI_ISL_1281569, EPI_ISL_1281571, EPI_ISL_1281573	Laboratory of Extremely Dangerous Infections of Federal Budget Health Care Instute "Center of Hygiene and Epidemiology in Sakhalin region"	Group of Genomics and Postgenomic Technologies of Central Research Institute of Epidemiology	Samoilov AE, Kaptelova VV, Korneenko EV, Valdokhina AV, Bulanenko VP, Saenko SS, Arutiunian AE, Byankina MA, Golubeva AG, Speranskaya AS, Tivanova EV, Kondrasheva LV, Akimkin VG
EPI_ISL_1281680	Imelda hospital Bonheiden	Imelda hospital Bonheiden	Johan Frans, Dagmar Obbels, Hanne Valgaeren
EPI_ISL_1283923, EPI_ISL_1283924	RSIA Sayyidah	Eijkman Institute for Molecular Biology, Ministry of Research and Technology/National Agency for Research and Innovation	Edison Johar, Frilasita A Yudhaputri, Hidayat Trimarsanto, Iskandar Adnan, Lydia V. Panggalo, Sukma Oktavianthi, Willy Agustine, Safarina G Malik, Khin Saw Myint, Amin Soebandrio
EPI_ISL_1283929	RS Mitra Keluarga Kelapa Gading	Eijkman Institute for Molecular Biology, Ministry of Research and Technology/National Agency for Research and Innovation	Edison Johar, Frilasita A Yudhaputri, Hidayat Trimarsanto, Iskandar Adnan, Lydia V. Panggalo, Sukma Oktavianthi, Willy Agustine, Safarina G Malik, Khin Saw Myint, Amin Soebandrio
EPI_ISL_1283932	RS Mulya Tangerang	Eijkman Institute for Molecular Biology, Ministry of Research and Technology/National Agency for Research and Innovation	Edison Johar, Frilasita A Yudhaputri, Hidayat Trimarsanto, Iskandar Adnan, Lydia V. Panggalo, Sukma Oktavianthi, Willy Agustine, Safarina G Malik, Khin Saw Myint, Amin Soebandrio
EPI_ISL_1283933	RS Mitra Keluarga Gading Serpong	Eijkman Institute for Molecular Biology, Ministry of Research and Technology/National Agency for Research and Innovation	Edison Johar, Frilasita A Yudhaputri, Hidayat Trimarsanto, Iskandar Adnan, Lydia V. Panggalo, Sukma Oktavianthi, Willy Agustine, Safarina G Malik, Khin Saw Myint, Amin Soebandrio
EPI_ISL_1283936	RS Sentra Medika Cikarang	Eijkman Institute for Molecular Biology, Ministry of Research and Technology/National Agency for Research and Innovation	Edison Johar, Frilasita A Yudhaputri, Hidayat Trimarsanto, Iskandar Adnan, Lydia V. Panggalo, Sukma Oktavianthi, Willy Agustine, Safarina G Malik, Khin Saw Myint, Amin Soebandrio
EPI_ISL_1283938	RSUD Cileungsi	Eijkman Institute for Molecular Biology, Ministry of Research and Technology/National Agency for Research and Innovation	Frilasita A Yudhaputri, Hidayat Trimarsanto, Iskandar Adnan, Lydia V. Panggalo, Sukma Oktavianthi, Willy Agustine, Edison Johar, Safarina G Malik, Khin Saw Myint, Amin Soebandrio
EPI_ISL_1283957	RSIA Selasih Medika	Eijkman Institute for Molecular Biology, Ministry of Research and Technology/National Agency for Research and Innovation	Frilasita A Yudhaputri, Hidayat Trimarsanto, Iskandar Adnan, Lydia V. Panggalo, Sukma Oktavianthi, Willy Agustine, Edison Johar, Safarina G Malik, Khin Saw Myint, Amin Soebandrio
EPI_ISL_1283960	RS Asy-Syifaa Leuwiliang	Eijkman Institute for Molecular Biology, Ministry of Research and Technology/National Agency for Research and Innovation	Hidayat Trimarsanto, Iskandar Adnan, Lydia V. Panggalo, Sukma Oktavianthi, Willy Agustine, Edison Johar, Frilasita A Yudhaputri, Safarina G Malik, Khin Saw Myint, Amin Soebandrio
EPI_ISL_1283967	RS Bina Husada Bogor	Eijkman Institute for Molecular Biology, Ministry of Research and Technology/National Agency for Research and Innovation	Hidayat Trimarsanto, Iskandar Adnan, Lydia V. Panggalo, Sukma Oktavianthi, Willy Agustine, Edison Johar, Frilasita A Yudhaputri, Safarina G Malik, Khin Saw Myint, Amin Soebandrio

EPI_ISL_1283970	RSIA Selasih Medika	Eijkman Institute for Molecular Biology, Ministry of Research and Technology/National Agency for Research and Innovation	Hidayat Trimarsanto, Iskandar Adnan, Lydia V. Panggalo, Sukma Oktavianthi, Willy Agustine, Edison Johar, Frilasita A Yudhaputri, Safarina G Malik, Khin Saw Myint, Amin Soebandrio
EPI_ISL_1283973	RSU Bunda Mulia Cikarang	Eijkman Institute for Molecular Biology, Ministry of Research and Technology/National Agency for Research and Innovation	Hidayat Trimarsanto, Iskandar Adnan, Lydia V. Panggalo, Sukma Oktavianthi, Willy Agustine, Edison Johar, Frilasita A Yudhaputri, Safarina G Malik, Khin Saw Myint, Amin Soebandrio
EPI_ISL_1283979	RS Cendana	Eijkman Institute for Molecular Biology, Ministry of Research and Technology/National Agency for Research and Innovation	Iskandar Adnan, Lydia V. Panggalo, Sukma Oktavianthi, Willy Agustine, Edison Johar, Hidayat Trimarsanto, Frilasita A Yudhaputri, Safarina G Malik, Khin Saw Myint, Amin Soebandrio
EPI_ISL_1283987	RS Islam Sari Asih Ar-Rahmah	Eijkman Institute for Molecular Biology, Ministry of Research and Technology/National Agency for Research and Innovation	Iskandar Adnan, Lydia V. Panggalo, Sukma Oktavianthi, Willy Agustine, Edison Johar, Hidayat Trimarsanto, Frilasita A Yudhaputri, Safarina G Malik, Khin Saw Myint, Amin Soebandrio
EPI_ISL_1283988	RSUD Cileungsi	Eijkman Institute for Molecular Biology, Ministry of Research and Technology/National Agency for Research and Innovation	Iskandar Adnan, Lydia V. Panggalo, Sukma Oktavianthi, Willy Agustine, Edison Johar, Hidayat Trimarsanto, Frilasita A Yudhaputri, Safarina G Malik, Khin Saw Myint, Amin Soebandrio
EPI_ISL_1283996	RSIA Bunda Sejahtera	Eijkman Institute for Molecular Biology, Ministry of Research and Technology/National Agency for Research and Innovation	Lydia V. Panggalo, Sukma Oktavianthi, Willy Agustine, Edison Johar, Hidayat Trimarsanto, Iskandar Adnan, Frilasita A Yudhaputri, Safarina G Malik, Khin Saw Myint, Amin Soebandrio
EPI_ISL_1284000	RSU HGA Depok	Eijkman Institute for Molecular Biology, Ministry of Research and Technology/National Agency for Research and Innovation	Lydia V. Panggalo, Sukma Oktavianthi, Willy Agustine, Edison Johar, Hidayat Trimarsanto, Iskandar Adnan, Frilasita A Yudhaputri, Safarina G Malik, Khin Saw Myint, Amin Soebandrio
EPI_ISL_1284007, EPI_ISL_1284008	RS Mitra Keluarga Gading Serpong	Eijkman Institute for Molecular Biology, Ministry of Research and Technology/National Agency for Research and Innovation	Sukma Oktavianthi, Willy Agustine, Edison Johar, Hidayat Trimarsanto, Iskandar Adnan, Lydia V. Panggalo, Frilasita A Yudhaputri, Safarina G Malik, Khin Saw Myint, Amin Soebandrio
EPI_ISL_1284014	RSUD Cileungsi	Eijkman Institute for Molecular Biology, Ministry of Research and Technology/National Agency for Research and Innovation	Sukma Oktavianthi, Willy Agustine, Edison Johar, Hidayat Trimarsanto, Iskandar Adnan, Lydia V. Panggalo, Frilasita A Yudhaputri, Safarina G Malik, Khin Saw Myint, Amin Soebandrio
EPI_ISL_1284017	RS Mitra Keluarga Kalideres	Eijkman Institute for Molecular Biology, Ministry of Research and Technology/National Agency for Research and Innovation	Sukma Oktavianthi, Willy Agustine, Edison Johar, Hidayat Trimarsanto, Iskandar Adnan, Lydia V. Panggalo, Frilasita A Yudhaputri, Safarina G Malik, Khin Saw Myint, Amin Soebandrio
EPI_ISL_1284021, EPI_ISL_1284025	RS Mitra Keluarga Gading Serpong	Eijkman Institute for Molecular Biology, Ministry of Research and Technology/National Agency for Research and Innovation	Sukma Oktavianthi, Willy Agustine, Edison Johar, Hidayat Trimarsanto, Iskandar Adnan, Lydia V. Panggalo, Frilasita A Yudhaputri, Safarina G Malik, Khin Saw Myint, Amin Soebandrio
EPI_ISL_1284027	RS Sentra Medika Cikarang	Eijkman Institute for Molecular Biology, Ministry of Research and Technology/National Agency for Research and Innovation	Willy Agustine, Edison Johar, Hidayat Trimarsanto, Iskandar Adnan, Lydia V. Panggalo, Sukma Oktavianthi, Frilasita A Yudhaputri, Safarina G Malik, Khin Saw Myint, Amin Soebandrio
EPI_ISL_1284031	RS OMNI Alam Sutera	Eijkman Institute for Molecular Biology, Ministry of Research and Technology/National Agency for Research and Innovation	Willy Agustine, Edison Johar, Hidayat Trimarsanto, Iskandar Adnan, Lydia V. Panggalo, Sukma Oktavianthi, Frilasita A Yudhaputri, Safarina G Malik, Khin Saw Myint, Amin Soebandrio
EPI_ISL_1284035	RS Taman Harapan Baru	Eijkman Institute for Molecular Biology, Ministry of Research and Technology/National Agency for Research and Innovation	Willy Agustine, Edison Johar, Hidayat Trimarsanto, Iskandar Adnan, Lydia V. Panggalo, Sukma Oktavianthi, Frilasita A Yudhaputri, Safarina G Malik, Khin Saw Myint, Amin Soebandrio
EPI_ISL_1284040	RSU Azra	Eijkman Institute for Molecular Biology, Ministry of Research and Technology/National Agency for Research and Innovation	Willy Agustine, Edison Johar, Hidayat Trimarsanto, Iskandar Adnan, Lydia V. Panggalo, Sukma Oktavianthi, Frilasita A Yudhaputri, Safarina G Malik, Khin Saw Myint, Amin Soebandrio
EPI_ISL_1284044, EPI_ISL_1284048	RS Bhakti Husada Cikarang	Eijkman Institute for Molecular Biology, Ministry of Research and Technology/National Agency for Research and Innovation	Edison Johar, Frilasita A Yudhaputri, Hidayat Trimarsanto, Iskandar Adnan, Lydia V. Panggalo, Sukma Oktavianthi, Willy Agustine, Safarina G Malik, Khin Saw Myint, Amin Soebandrio
EPI_ISL_1284049	RSU Harapan Bunda	Eijkman Institute for Molecular Biology, Ministry of Research and Technology/National Agency for Research and Innovation	Edison Johar, Frilasita A Yudhaputri, Hidayat Trimarsanto, Iskandar Adnan, Lydia V. Panggalo, Sukma Oktavianthi, Willy Agustine, Safarina G Malik, Khin Saw Myint, Amin Soebandrio
EPI_ISL_1284052	RS Rumah Indonesia Sehat	Eijkman Institute for Molecular Biology, Ministry of Research and Technology/National Agency for Research and Innovation	Edison Johar, Frilasita A Yudhaputri, Hidayat Trimarsanto, Iskandar Adnan, Lydia V. Panggalo, Sukma Oktavianthi, Willy Agustine, Safarina G Malik, Khin Saw Myint, Amin Soebandrio
EPI_ISL_1284054	Metro Hospitals Cikarang	Eijkman Institute for Molecular Biology, Ministry of Research and Technology/National Agency for Research and Innovation	Edison Johar, Frilasita A Yudhaputri, Hidayat Trimarsanto, Iskandar Adnan, Lydia V. Panggalo, Sukma Oktavianthi, Willy Agustine, Safarina G Malik, Khin Saw Myint, Amin Soebandrio
EPI_ISL_1284057	RS Primaya Tangerang	Eijkman Institute for Molecular Biology, Ministry of Research and Technology/National Agency for Research and Innovation	Edison Johar, Frilasita A Yudhaputri, Hidayat Trimarsanto, Iskandar Adnan, Lydia V. Panggalo, Sukma Oktavianthi, Willy Agustine, Safarina G Malik, Khin Saw Myint, Amin Soebandrio
EPI_ISL_1284063	RS Mitra Keluarga Gading Serpong	Eijkman Institute for Molecular Biology, Ministry of Research and Technology/National Agency for Research and Innovation	Frilasita A Yudhaputri, Hidayat Trimarsanto, Iskandar Adnan, Lydia V. Panggalo, Sukma Oktavianthi, Willy Agustine, Edison Johar, Safarina G Malik, Khin Saw Myint, Amin Soebandrio
EPI_ISL_1284064	RSU HGA Depok	Eijkman Institute for Molecular Biology, Ministry of Research and Technology/National Agency for Research and Innovation	Frilasita A Yudhaputri, Hidayat Trimarsanto, Iskandar Adnan, Lydia V. Panggalo, Sukma Oktavianthi, Willy Agustine, Edison Johar, Safarina G Malik, Khin Saw Myint, Amin Soebandrio
EPI_ISL_1289960	Laboratory of Virology, Ribeirão Preto General Hospital, Ribeirão Preto Medical School, University of São Paulo	Laboratory of Oncology, Blood Center of Ribeirão Preto, Ribeirão Preto School of Medicine, University of São Paulo	CAMPOS, MR; SANTOS, A.L.P.; YAMAMOTO, A.Y.; COLLI, L.M.; FONSECA, B.A.L.; BELLISSIMO-RODRIGUES, F.
EPI_ISL_1293051	LACEN do Distrito Federal	Instituto Adolfo Lutz, Interdiciplinary Procedures Center, Strategic Laboratory	Claudio Tavares Sacchi, Claudia Regina Gonçalves, Erica Valessa Ramos Gomes, Karoline Rodrigues Campos, Caio Vinicius Dias Lopes
EPI_ISL_1293353	OLVZ Aalst	OLVZ Aalst	Astrid Holderbeke
EPI_ISL_1297194	RSUD Pasarminggu	Stem Cell Lab. Universitas Pembangunan Nasional Veteran Jakarta (UPNVJ)	Andri Pramesyanti Pramono, Yudhi Nugraha, Ria Maria Theresa, Yudi Amiarno, Megadianty Mokoginta

EPI_ISL_1299195, EPI_ISL_1299196, EPI_ISL_1299197, EPI_ISL_1299198, EPI_ISL_1299199, EPI_ISL_1299200, EPI_ISL_1299201, EPI_ISL_1299202, EPI_ISL_1299203, EPI_ISL_1299204, EPI_ISL_1299205, EPI_ISL_1299206, EPI_ISL_1299207, EPI_ISL_1299208, EPI_ISL_1299209, EPI_ISL_1299210, EPI_ISL_1299211,

EPI_ISL_1299212, EPI_ISL_1299213, EPI_ISL_1299214, EPI_ISL_1299215, EPI_ISL_1299216			
see above	Department of Virology, Bangabandhu Sheikh Mujib Medical University	Department of Virology, Bangabandhu Sheikh Mujib Medical University	Munira Jahan, Arittra Bhattacharjee, Raad Rahmat, S M Rashedul Islam, Tahmina Akhter, Ishtiaque Ahammad, Mohammad Uzzal Hossain, Md. Salimullah, Saif Ullah Munshi
EPI_ISL_1300496	RSUD Pasarminggu	Stem Cell Lab, Universitas Pembangunan Nasional Veteran Jakarta (UPNVJ)	Andri Pramesyanti Pramono, Yudhi Nugraha, Ria Maria Theresa, Yudi Amiarno, Megadianty Mokoginta
EPI_ISL_1300521	RSUD Pasarminggu	Stem Cell Lab, Universitas Pembangunan Nasional Veteran Jakarta	Andri Pramesyanti Pramono, Yudhi Nugraha, Ria Maria Theresa, Yudi Amiarno, Megadianty Mokoginta
EPI_ISL_1300535, EPI_ISL_1300540, EPI_ISL_1300644, EPI_ISL_1300660	RSUD Pasarminggu	Stem Cell Lab, Universitas Pembangunan Nasional Veteran Jakarta (UPNVJ)	Andri Pramesyanti Pramono, Yudhi Nugraha, Ria Maria Theresa, Yudi Amiarno, Megadianty Mokoginta
EPI_ISL_1301830, EPI_ISL_1301832	Outre Mer	National Reference Center for Viruses of Respiratory Infections, Institut Pasteur, Paris	Marion Barbet, Sylvie Behillil, Méline Bizard, Angela Brisebarre, Camille Capel, Louise Lefrançois, Etienne Simon-Lorière, Vincent Enouf, Maud Vanpeene, Sylvie van der Werf,Combe Patrice
EPI_ISL_1301834, EPI_ISL_1301835, EPI_ISL_1301836, EPI_ISL_1301837	Hopital	National Reference Center for Viruses of Respiratory Infections, Institut Pasteur, Paris	Marion Barbet, Sylvie Behillil, Méline Bizard, Angela Brisebarre, Camille Capel, Louise Lefrançois, Etienne Simon-Lorière, Vincent Enouf, Maud Vanpeene, Sylvie van der Werf,Leruez-Ville Marianne
EPI_ISL_1301889	Outre Mer	National Reference Center for Viruses of Respiratory Infections, Institut Pasteur, Paris	Marion Barbet, Sylvie Behillil, Méline Bizard, Angela Brisebarre, Camille Capel, Louise Lefrançois, Etienne Simon-Lorière, Vincent Enouf, Maud Vanpeene, Sylvie van der Werf,Combe Patrice
EPI_ISL_1301895	Hopital	National Reference Center for Viruses of Respiratory Infections, Institut Pasteur, Paris	Marion Barbet, Sylvie Behillil, Méline Bizard, Angela Brisebarre, Camille Capel, Louise Lefrançois, Etienne Simon-Lorière, Vincent Enouf, Maud Vanpeene, Sylvie van der Werf,Combe Patrice
EPI_ISL_1301903, EPI_ISL_1301904, EPI_ISL_1301905, EPI_ISL_1301906, EPI_ISL_1301907, EPI_ISL_1301908, EPI_ISL_1301909, EPI_ISL_1301910, EPI_ISL_1301911, EPI_ISL_1301912, EPI_ISL_1301913, EPI_ISL_1301914			
see above	Outre Mer	National Reference Center for Viruses of Respiratory Infections, Institut Pasteur, Paris	Marion Barbet, Sylvie Behillil, Méline Bizard, Angela Brisebarre, Camille Capel, Louise Lefrançois, Etienne Simon-Lorière, Vincent Enouf, Maud Vanpeene, Sylvie van der Werf,Combe Patrice
EPI_ISL_1301915	Labo Analyses Med	National Reference Center for Viruses of Respiratory Infections, Institut Pasteur, Paris	Marion Barbet, Sylvie Behillil, Méline Bizard, Angela Brisebarre, Camille Capel, Louise Lefrançois, Etienne Simon-Lorière, Vincent Enouf, Maud Vanpeene, Sylvie van der Werf,Delaval José
EPI_ISL_1301920	Hopital	National Reference Center for Viruses of Respiratory Infections, Institut Pasteur, Paris	Marion Barbet, Sylvie Behillil, Méline Bizard, Angela Brisebarre, Camille Capel, Louise Lefrançois, Etienne Simon-Lorière, Vincent Enouf, Maud Vanpeene, Sylvie van der Werf,Leruez-Ville Marianne
EPI_ISL_1301921	Hopital	National Reference Center for Viruses of Respiratory Infections, Institut Pasteur, Paris	Marion Barbet, Sylvie Behillil, Méline Bizard, Angela Brisebarre, Camille Capel, Louise Lefrançois, Etienne Simon-Lorière, Vincent Enouf, Maud Vanpeene, Sylvie van der Werf,Ducancelle Alexandra
EPI_ISL_1301924, EPI_ISL_1301925, EPI_ISL_1301926, EPI_ISL_1301927, EPI_ISL_1301928, EPI_ISL_1301929	Hopital	National Reference Center for Viruses of Respiratory Infections, Institut Pasteur, Paris	Marion Barbet, Sylvie Behillil, Méline Bizard, Angela Brisebarre, Camille Capel, Louise Lefrançois, Etienne Simon-Lorière, Vincent Enouf, Maud Vanpeene, Sylvie van der Werf,Leruez-Ville Marianne
EPI_ISL_1301981, EPI_ISL_1301982, EPI_ISL_1301983, EPI_ISL_1301984, EPI_ISL_1302090, EPI_ISL_1302094, EPI_ISL_1302098, EPI_ISL_1302099, EPI_ISL_1302100, EPI_ISL_1302101, EPI_ISL_1302102, EPI_ISL_1302103, EPI_ISL_1302104, EPI_ISL_1302105, EPI_ISL_1302106, EPI_ISL_1302107, EPI_ISL_1302108, EPI_ISL_1302109, EPI_ISL_1302122, EPI_ISL_1302123, EPI_ISL_1302124, EPI_ISL_1302125, EPI_ISL_1302126, EPI_ISL_1302127, EPI_ISL_1302128, EPI_ISL_1302129, EPI_ISL_1302130, EPI_ISL_1302131, EPI_ISL_1302132, EPI_ISL_1302133, EPI_ISL_1302134, EPI_ISL_1302135, EPI_ISL_1302136, EPI_ISL_1302137, EPI_ISL_1302138, EPI_ISL_1302139, EPI_ISL_1302140, EPI_ISL_1302141, EPI_ISL_1302142, EPI_ISL_1302143, EPI_ISL_1302144, EPI_ISL_1302145, EPI_ISL_1302146, EPI_ISL_1302147, EPI_ISL_1302148			
see above	National Center of Infectious and Parasitic Diseases	National Center of Infectious and Parasitic Diseases	Alexiev et al
EPI_ISL_1302395	Instituto Nacional de Enfermedades Respiratorias (INER)	Instituto de Biotecnología de la UNAM	Authors from IBT, IMSS, InDRE and INER (in alphabetical order): Carlos F. Arias, Santiago Ávila-Ríos, Gisela Barrera-Badillo, Eduardo Becerril-Vargas, Celia Boukadida, Natividad Cruz-Ortiz, Larissa Fernandes-Matano, Ricardo Grande, Lucia Hernandez-Rivas, Alejandra Hernández-Terán, Pavel Isa, Irma Lopez-Martinez, José Arturo Martínez-Orozco, Margarita Matías-Florentino, Fidencio Mejía-Nepomuceno, Edgar Mendieta-Condado, Mario Mújica-Sánchez, José Esteban Muñoz-Medina, Tatiana Nunez-Garcia, Luis Alberto Ochoa-Carrera, Hector Esteban Paz-Juárez, Francisco Pulido, José Ernesto Ramírez-González, Alma Rincón-Rubio, Teresita Rojas-Mendoza, Jorge Salas-Hernández, Alejandro Sanchez-Flores, Clara Esperanza Santacruz-Tinoco, Andrea Santos Coy-Arechavaleta, Blanca Taboada, Gloria Vazquez, Joel Armando Vázquez-Pérez, Jerome Jean Verleyen
EPI_ISL_1302396, EPI_ISL_1302397, EPI_ISL_1302398	Instituto de Diagnostico y Referencia Epidemiologicos InDRE_RNLSP	Instituto de Biotecnología de la UNAM	Authors from IBT, IMSS, InDRE and INER (in alphabetical order): Carlos F. Arias, Santiago Ávila-Ríos, Gisela Barrera-Badillo, Eduardo Becerril-Vargas, Celia Boukadida, Natividad Cruz-Ortiz, Larissa Fernandes-Matano, Ricardo Grande, Lucia Hernandez-Rivas, Alejandra Hernández-Terán, Pavel Isa, Irma Lopez-Martinez, José Arturo Martínez-Orozco, Margarita Matías-Florentino, Fidencio Mejía-Nepomuceno, Edgar Mendieta-Condado, Mario Mújica-Sánchez, José Esteban Muñoz-Medina, Tatiana Nunez-Garcia, Luis Alberto Ochoa-Carrera, Hector Esteban Paz-Juárez, Francisco Pulido, José Ernesto Ramírez-González, Alma Rincón-Rubio, Teresita Rojas-Mendoza, Jorge Salas-Hernández, Alejandro Sanchez-Flores, Clara Esperanza Santacruz-Tinoco, Andrea Santos Coy-Arechavaleta, Blanca Taboada, Gloria Vazquez, Joel Armando Vázquez-Pérez, Jerome Jean Verleyen
EPI_ISL_1302399, EPI_ISL_1302400	Instituto Nacional de Enfermedades Respiratorias (INER)	Instituto de Biotecnología de la UNAM	Authors from IBT, IMSS, InDRE and INER (in alphabetical order): Carlos F. Arias, Santiago Ávila-Ríos, Gisela Barrera-Badillo, Eduardo Becerril-Vargas, Celia Boukadida, Natividad Cruz-Ortiz, Larissa Fernandes-Matano, Ricardo Grande, Lucia Hernandez-Rivas, Alejandra Hernández-Terán, Pavel Isa, Irma Lopez-Martinez, José Arturo Martínez-Orozco, Margarita Matías-Florentino, Fidencio Mejía-Nepomuceno, Edgar Mendieta-Condado, Mario Mújica-Sánchez, José Esteban Muñoz-Medina, Tatiana Nunez-Garcia, Luis Alberto Ochoa-Carrera, Hector Esteban Paz-Juárez, Francisco Pulido, José Ernesto Ramírez-González, Alma Rincón-Rubio, Teresita Rojas-Mendoza, Jorge Salas-Hernández, Alejandro Sanchez-Flores, Clara Esperanza Santacruz-Tinoco, Andrea Santos Coy-Arechavaleta, Blanca Taboada, Gloria Vazquez, Joel Armando Vázquez-Pérez, Jerome Jean Verleyen
EPI_ISL_1303368	Clinica de la Costa	Instituto Nacional de Salud- Dirección de Investigación en Salud Pública	Katherine Laiton-Donato, Diego A. Álvarez-Díaz, Carlos Franco-Muñoz, Hector Alejandro Ruiz-Moreno, Maria T. Herrera-Sepúlveda, Diego Andrés Prada, Jhonnatan Reales-González, Sheryll Corchuelo, Julian Naizaque, Gerardo Santamaría, Sergio Gomez, Lisseth Pardo, Juan Camilo Martinez, Marta Lopez Blanco, Ángela Alarcon Cruz, Diana Malo, Carmen Osorio, Magdalena Wiesner, Martha Lucia Ospina Martinez, Marcela Mercado-Reyes
EPI_ISL_1303504	LACEN de Rondonia	Instituto Adolfo Lutz, Interdiciplinary Procedures Center, Strategic Laboratory	Claudio Tavares Sacchi, Claudia Regina Gonçalves, Erica Valessa Ramos Gomes, Karoline Rodrigues Campos, Caio Vinicius Dias Lopes
EPI_ISL_1303513, EPI_ISL_1303515, EPI_ISL_1303516	LACEN do Estado de Goias	Instituto Adolfo Lutz, Interdiciplinary Procedures Center, Strategic Laboratory	Claudio Tavares Sacchi, Claudia Regina Gonçalves, Erica Valessa Ramos Gomes, Karoline Rodrigues Campos, Caio Vinicius Dias Lopes
EPI_ISL_1311863	Laboratorio Biologia Molecolare Sars Cov2 - UOC Laboratorio Analisi - Servizio Medicina di Laboratorio, Ospedale "San Francesco" - ATS-ASSL Nuoro	Laboratorio specialistico UOC Ematologia - Ospedale "San Francesco" - ATS-ASSL Nuoro	Piras Giovanna, Asproni Rosanna,Fiamma Maura, Monne Maria Itria, Malune Paolo, Palmas Angelo Domenico, Lo Maglio Iana, Mameli Giuseppe
EPI_ISL_1318203, EPI_ISL_1318204, EPI_ISL_1318205, EPI_ISL_1318208, EPI_ISL_1318209, EPI_ISL_1318211, EPI_ISL_1318212	Ministry of Health Hospitals	Institute of Health and Community Medicine	Chan Chia Jui, David Perera, Ooi Mong How, Chua Hock Hin, Tonnii Sia Loong Loong, Wong Jyn Shan, Wong Kiing Aik
EPI_ISL_1324766	Laboratorio de Diagnostico de Enfermedades Emergentes y Reemergentes	Instituto de Diagnostico y Referencia Epidemiologicos (INDRE)	Claudia Wong-Arambula, Abril Rodriguez-Maldonado, Vanessa Rivero-Arredondo, Ariadna Medina-Benitez, Joaquin Quiroz-Mercado, Sergio Rangel-Guerrero,Natali Vega-Magana, Natividad Cruz-Ortiz, Tatiana Nunez-Garcia, Gisela Barrera-Badillo, Lucia Hernandez-Rivas, Irma Lopez-Martinez, Ernesto Ramirez-Gonzalez.
EPI_ISL_1335884	Department of Microbiology, Faculty of Medicine, Universitas Sumatera Utara	Faculty of Medicine, Universitas Sumatera Utara; Institute of Tropical Disease, Universitas Airlangga	Jezzy R Dewantari, Rima R Prasetya, Krisnoadi Rahardjo, Aldise M Nastri, Yasuko Mori, Soetjipto, Kazufumi Shimizu, Maria I Lusida, Meliani, R Andika D Cahyadi, Mirzan Hasibuan, Irbah R Nainggolan, Ramadhan Bestari, Franciscus Ginting, Muhammad Ichwan, R Lia Kusumawati
EPI_ISL_1335886	Sundari Hospital Medan	Faculty of Medicine, Universitas Sumatera Utara; Institute of Tropical Disease, Universitas Airlangga	Kazufumi Shimizu, Krisnoadi Rahardjo, Aldise M Nastri, Jezzy R Dewantari, Rima R Prasetya, Yasuko Mori, Soetjipto, Maria I Lusida, Meliani, R Andika D Cahyadi, Mirzan Hasibuan, Irbah R Nainggolan, Ramadhan Bestari, Franciscus Ginting, Muhammad Ichwan, R Lia Kusumawati, Inke N D Lubis
EPI_ISL_1335888	Martha Friska Multatuli Hospital Medan	Faculty of Medicine, Universitas Sumatera Utara; Institute of	Krisnoadi Rahardjo, Aldise M Nastri, Jezzy R Dewantari, Rima R Prasetya, Yasuko Mori, Soetjipto, Kazufumi Shimizu, Maria I Lusida, Meliani, R Andika D

		Tropical Disease, Universitas Airlangga	Cahyadi, Mirzan Hasibuan, Irbah R Nainggolan, Ramadhan Bestari, Franciscus Ginting, Muhammad Ichwan, R Lia Kusumawati, Inke N D Lubis
EPI_ISL_1335889	Advent Hospital Medan	Faculty of Medicine, Universitas Sumatera Utara; Institute of Tropical Disease, Universitas Airlangga	Krisnoadi Rahardjo, Aldise M Nastri, Jezzy R Dewantari, Rima R Prasetya, Yasuko Mori, Soetjipto, Kazufumi Shimizu, Maria I Lusida, Meliani, R Andika D Cahyadi, Mirzan Hasibuan, Irbah R Nainggolan, Ramadhan Bestari, Franciscus Ginting, Muhammad Ichwan, R Lia Kusumawati, Inke N D Lubis
EPI_ISL_1335890	Elisabeth Hospital Medan	Faculty of Medicine, Universitas Sumatera Utara; Institute of Tropical Disease, Universitas Airlangga	Aldise M Nastri, Jezzy R Dewantari, Rima R Prasetya, Krisnoadi Rahardjo, Yasuko Mori, Soetjipto, Kazufumi Shimizu, Maria I Lusida, Meliani, R Andika D Cahyadi, Mirzan Hasibuan, Irbah R Nainggolan, Ramadhan Bestari, Franciscus Ginting, Muhammad Ichwan, R Lia Kusumawati, Inke N D Lubis
EPI_ISL_1335891	Advent Hospital Medan	Faculty of Medicine, Universitas Sumatera Utara; Institute of Tropical Disease, Universitas Airlangga	Jezzy R Dewantari, Rima R Prasetya, Krisnoadi Rahardjo, Aldise M Nastri, Yasuko Mori, Soetjipto, Kazufumi Shimizu, Maria I Lusida, Meliani, R Andika D Cahyadi, Mirzan Hasibuan, Irbah R Nainggolan, Ramadhan Bestari, Franciscus Ginting, Muhammad Ichwan, R Lia Kusumawati, Inke N D Lubis
EPI_ISL_1335892	Department of Microbiology, Faculty of Medicine, Universitas Sumatera Utara	Faculty of Medicine, Universitas Sumatera Utara; Institute of Tropical Disease, Universitas Airlangga	Rima R Prasetya, Krisnoadi Rahardjo, Aldise M Nastri, Jezzy R Dewantari, Yasuko Mori, Soetjipto, Kazufumi Shimizu, Maria I Lusida, Meliani, R Andika D Cahyadi, Mirzan Hasibuan, Irbah R Nainggolan, Ramadhan Bestari, Franciscus Ginting, Muhammad Ichwan, R Lia Kusumawati, Inke N D Lubis
EPI_ISL_1335893	Department of Microbiology, Faculty of Medicine, Universitas Sumatera Utara	Faculty of Medicine, Universitas Sumatera Utara; Institute of Tropical Disease, Universitas Airlangga	Kazufumi Shimizu, Krisnoadi Rahardjo, Aldise M Nastri, Jezzy R Dewantari, Rima R Prasetya, Yasuko Mori, Soetjipto, Maria I Lusida, Meliani, R Andika D Cahyadi, Mirzan Hasibuan, Irbah R Nainggolan, Ramadhan Bestari, Franciscus Ginting, Muhammad Ichwan, R Lia Kusumawati, Inke N D Lubis
EPI_ISL_1335894	Department of Microbiology, Faculty of Medicine, Universitas Sumatera Utara	Faculty of Medicine, Universitas Sumatera Utara; Institute of Tropical Disease, Universitas Airlangga	Maria I Lusida, Krisnoadi Rahardjo, Aldise M Nastri, Jezzy R Dewantari, Rima R Prasetya, Yasuko Mori, Soetjipto, Kazufumi Shimizu, Meliani, R Andika D Cahyadi, Mirzan Hasibuan, Irbah R Nainggolan, Ramadhan Bestari, Franciscus Ginting, Muhammad Ichwan, R Lia Kusumawati, Inke N D Lubis
EPI_ISL_1335895	Martha Friska Multatuli Hospital Medan	Faculty of Medicine, Universitas Sumatera Utara; Institute of Tropical Disease, Universitas Airlangga	Krisnoadi Rahardjo, Aldise M Nastri, Jezzy R Dewantari, Rima R Prasetya, Yasuko Mori, Soetjipto, Kazufumi Shimizu, Maria I Lusida, Meliani, R Andika D Cahyadi, Mirzan Hasibuan, Irbah R Nainggolan, Ramadhan Bestari, Franciscus Ginting, Muhammad Ichwan, R Lia Kusumawati, Inke N D Lubis
EPI_ISL_1340754, EPI_ISL_1340755, EPI_ISL_1340756	Departamento de Virologia, Laboratorio Central de Salud Pública, Avenida Venezuela y Teniente Escurra, Asunción, Paraguay	Laboratory of Respiratory Viruses and Measles, Oswaldo Cruz Institute, FIOCRUZ	Paola Resende, Cynthia Vazquez, Luciana Appolinario, Fernando Motta, Anna Carolina Paixao, Ana Carolina Mendonca, Alice Sampaio Rocha, Renata Serrano Lopes, Marilda Siqueira on behalf of the Fiocruz COVID-19 Genomic Surveillance Network
EPI_ISL_1340782, EPI_ISL_1340783, EPI_ISL_1340784, EPI_ISL_1340785	Northwestern Memorial Hospital	Northwestern University - Ozer Lab	Ramon Lorenzo-Redondo, Lacy M. Simons, Taylor J. Dean, Chad J. Achenbach, Lawrence J. Jennings, Chao Qi, Michael G. Ison, Judd F. Hultquist, Egon A. Ozer
EPI_ISL_1346626, EPI_ISL_1346628, EPI_ISL_1346630, EPI_ISL_1346631	Center of Excellence in Clinical Virology	Research Unit of Systems Microbiology	Jiratchaya Puenpa, Pattaraporn Nimsamer, Oraphan Mayuramart, Vorthon Sawaswong, Sunchai Payungporn, Anek Mungaomklang, Ritthideach Yorsaeng, Jira Chansaenroj, Kamolthip Atsawawaranunt, Vichan Pawan, Suwanna Petto, Amornmas Kongklieng, Pakkaporn Panwijitkul, Yong Poovowan
EPI_ISL_1358316, EPI_ISL_1358317	LACEN do Mato Grosso do Sul	Instituto Adolfo Lutz, Interdiciplinary Procedures Center, Strategic Laboratory	Claudio Tavares Sacchi, Claudia Regina Gonçalves, Erica Valessa Ramos Gomes, Karoline Rodrigues Campos, Caio Vinicius Dias Lopes
EPI_ISL_1360296	CSIR-National Environmental Engineering Research Institute	CSIR-Centre for Cellular and Molecular Biology - INSACOG	Onkar Kulkarni, Lamuk Zaveri, Ara Sreenivas, Sofia Banu, Shreekant Verma, Amareshwar Vodapalli , Viswagithe S L, B Himasri, Sharath Chandra Thota, Karthik Bharadwaj Tallapaka, Krishna Khairnar, Rakesh K Mishra,Divya Tej Sowpati
EPI_ISL_1360298	CSIR-National Environmental Engineering Research Institute	CSIR-Centre for Cellular and Molecular Biology - INSACOG	Lamuk Zaveri, Ara Sreenivas, Onkar Kulkarni, Sofia Banu, Shreekant Verma,Amareshwar Vodapalli, Viswagithe S L, B Himasri, Sharath Chandra Thota, Karthik Bharadwaj Tallapaka, Krishna Khairnar, Rakesh K Mishra,Divya Tej Sowpati
EPI_ISL_1360299, EPI_ISL_1360300	CSIR-National Environmental Engineering Research Institute	CSIR-Centre for Cellular and Molecular Biology - INSACOG	Ara Sreenivas, Onkar Kulkarni, Lamuk Zaveri, Sofia Banu, Shreekant Verma, Amareshwar Vodapalli , Viswagithe S L, B Himasri, Sharath Chandra Thota, Karthik Bharadwaj Tallapaka, Krishna Khairnar, Rakesh K Mishra,Divya Tej Sowpati
EPI_ISL_1360301, EPI_ISL_1360302	CSIR-National Environmental Engineering Research Institute	CSIR-Centre for Cellular and Molecular Biology - INSACOG	Sofia Banu, Lamuk Zaveri, Ara Sreenivas, Shreekant Verma, Onkar Kulkarni, Amareshwar Vodapalli ,Viswagithe S L, B Himasri, Sharath Chandra Thota, Karthik Bharadwaj Tallapaka, Krishna Khairnar, Rakesh K Mishra,Divya Tej Sowpati
EPI_ISL_1360303, EPI_ISL_1360304	CSIR-National Environmental Engineering Research Institute	CSIR-Centre for Cellular and Molecular Biology - INSACOG	Onkar Kulkarni, Lamuk Zaveri, Ara Sreenivas, Sofia Banu, Shreekant Verma, Amareshwar Vodapalli , Viswagithe S L, B Himasri, Sharath Chandra Thota, Karthik Bharadwaj Tallapaka, Krishna Khairnar, Rakesh K Mishra,Divya Tej Sowpati
EPI_ISL_1360305	CSIR-National Environmental Engineering Research Institute	CSIR-Centre for Cellular and Molecular Biology - INSACOG	Lamuk Zaveri, Ara Sreenivas, Sofia Banu, Onkar Kulkarni, Shreekant Verma, Amareshwar Vodapalli , Viswagithe S L, B Himasri, Sharath Chandra Thota, Karthik Bharadwaj Tallapaka, Krishna Khairnar, Rakesh K Mishra,Divya Tej Sowpati
EPI_ISL_1360306	CSIR-National Environmental Engineering Research Institute	CSIR-Centre for Cellular and Molecular Biology - INSACOG	Lamuk Zaveri, Ara Sreenivas, Onkar Kulkarni, Sofia Banu, Shreekant Verma,Amareshwar Vodapalli, Viswagithe S L, B Himasri, Sharath Chandra Thota, Karthik Bharadwaj Tallapaka, Krishna Khairnar, Rakesh K Mishra,Divya Tej Sowpati
EPI_ISL_1360307, EPI_ISL_1360308	CSIR-National Environmental Engineering Research Institute	CSIR-Centre for Cellular and Molecular Biology - INSACOG	Ara Sreenivas, Onkar Kulkarni, Lamuk Zaveri, Sofia Banu, Shreekant Verma, Amareshwar Vodapalli , Viswagithe S L, B Himasri, Sharath Chandra Thota, Karthik Bharadwaj Tallapaka, Krishna Khairnar, Rakesh K Mishra,Divya Tej Sowpati
EPI_ISL_1360309, EPI_ISL_1360310	CSIR-National Environmental Engineering Research Institute	CSIR-Centre for Cellular and Molecular Biology - INSACOG	Sofia Banu, Lamuk Zaveri, Ara Sreenivas, Shreekant Verma, Onkar Kulkarni, Amareshwar Vodapalli ,Viswagithe S L, B Himasri, Sharath Chandra Thota, Karthik Bharadwaj Tallapaka, Krishna Khairnar, Rakesh K Mishra,Divya Tej Sowpati
EPI_ISL_1360311, EPI_ISL_1360312	CSIR-National Environmental Engineering Research Institute	CSIR-Centre for Cellular and Molecular Biology - INSACOG	Onkar Kulkarni, Lamuk Zaveri, Ara Sreenivas, Sofia Banu, Shreekant Verma, Amareshwar Vodapalli , Viswagithe S L, B Himasri, Sharath Chandra Thota, Karthik Bharadwaj Tallapaka, Krishna Khairnar, Rakesh K Mishra,Divya Tej Sowpati
EPI_ISL_1360313	CSIR-National Environmental Engineering Research Institute	CSIR-Centre for Cellular and Molecular Biology - INSACOG	Lamuk Zaveri, Ara Sreenivas, Sofia Banu, Onkar Kulkarni, Shreekant Verma, Amareshwar Vodapalli , Viswagithe S L, B Himasri, Sharath Chandra Thota, Karthik Bharadwaj Tallapaka, Krishna Khairnar, Rakesh K Mishra,Divya Tej Sowpati
EPI_ISL_1360314	CSIR-National Environmental Engineering Research Institute	CSIR-Centre for Cellular and Molecular Biology - INSACOG	Lamuk Zaveri, Ara Sreenivas, Onkar Kulkarni, Sofia Banu, Shreekant Verma,Amareshwar Vodapalli, Viswagithe S L, B Himasri, Sharath Chandra Thota, Karthik Bharadwaj Tallapaka, Krishna Khairnar, Rakesh K Mishra,Divya Tej Sowpati
EPI_ISL_1360315, EPI_ISL_1360316	CSIR-National Environmental Engineering Research Institute	CSIR-Centre for Cellular and Molecular Biology - INSACOG	Ara Sreenivas, Onkar Kulkarni, Lamuk Zaveri, Sofia Banu, Shreekant Verma, Amareshwar Vodapalli , Viswagithe S L, B Himasri, Sharath Chandra Thota, Karthik Bharadwaj Tallapaka, Krishna Khairnar, Rakesh K Mishra,Divya Tej Sowpati
EPI_ISL_1360317, EPI_ISL_1360318	CSIR-National Environmental Engineering Research Institute	CSIR-Centre for Cellular and Molecular Biology - INSACOG	Sofia Banu, Lamuk Zaveri, Ara Sreenivas, Shreekant Verma, Onkar Kulkarni, Amareshwar Vodapalli ,Viswagithe S L, B Himasri, Sharath Chandra Thota, Karthik Bharadwaj Tallapaka, Krishna Khairnar, Rakesh K Mishra,Divya Tej Sowpati
EPI_ISL_1360319, EPI_ISL_1360320	CSIR-National Environmental Engineering Research Institute	CSIR-Centre for Cellular and Molecular Biology - INSACOG	Onkar Kulkarni, Lamuk Zaveri, Ara Sreenivas, Sofia Banu, Shreekant Verma, Amareshwar Vodapalli , Viswagithe S L, B Himasri, Sharath Chandra Thota, Karthik Bharadwaj Tallapaka, Krishna Khairnar, Rakesh K Mishra,Divya Tej Sowpati
EPI_ISL_1360321	CSIR-National Environmental Engineering Research Institute	CSIR-Centre for Cellular and Molecular Biology - INSACOG	Lamuk Zaveri, Ara Sreenivas, Sofia Banu, Onkar Kulkarni, Shreekant Verma, Amareshwar Vodapalli , Viswagithe S L, B Himasri, Sharath Chandra Thota, Karthik Bharadwaj Tallapaka, Krishna Khairnar, Rakesh K Mishra,Divya Tej Sowpati
EPI_ISL_1360322	CSIR-National Environmental Engineering Research Institute	CSIR-Centre for Cellular and Molecular Biology - INSACOG	Lamuk Zaveri, Ara Sreenivas, Onkar Kulkarni, Sofia Banu, Shreekant Verma,Amareshwar Vodapalli, Viswagithe S L, B Himasri, Sharath Chandra Thota, Karthik Bharadwaj Tallapaka, Krishna Khairnar, Rakesh K Mishra,Divya Tej Sowpati
EPI_ISL_1360323, EPI_ISL_1360324	CSIR-National Environmental Engineering Research Institute	CSIR-Centre for Cellular and Molecular Biology - INSACOG	Ara Sreenivas, Onkar Kulkarni, Lamuk Zaveri, Sofia Banu, Shreekant Verma, Amareshwar Vodapalli , Viswagithe S L, B Himasri, Sharath Chandra Thota, Karthik Bharadwaj Tallapaka, Krishna Khairnar, Rakesh K Mishra,Divya Tej Sowpati
EPI_ISL_1360325, EPI_ISL_1360326	CSIR-National Environmental Engineering Research Institute	CSIR-Centre for Cellular and Molecular Biology - INSACOG	Sofia Banu, Lamuk Zaveri, Ara Sreenivas, Shreekant Verma, Onkar Kulkarni, Amareshwar Vodapalli ,Viswagithe S L, B Himasri, Sharath Chandra Thota, Karthik Bharadwaj Tallapaka, Krishna Khairnar, Rakesh K Mishra,Divya Tej Sowpati
EPI_ISL_1360327, EPI_ISL_1360328	CSIR-National Environmental Engineering Research Institute	CSIR-Centre for Cellular and Molecular Biology - INSACOG	Onkar Kulkarni, Lamuk Zaveri, Ara Sreenivas, Sofia Banu, Shreekant Verma, Amareshwar Vodapalli , Viswagithe S L, B Himasri, Sharath Chandra Thota, Karthik Bharadwaj Tallapaka, Krishna Khairnar, Rakesh K Mishra,Divya Tej Sowpati
EPI_ISL_1360329	CSIR-National Environmental Engineering Research Institute	CSIR-Centre for Cellular and Molecular Biology - INSACOG	Lamuk Zaveri, Ara Sreenivas, Sofia Banu, Onkar Kulkarni, Shreekant Verma, Amareshwar Vodapalli , Viswagithe S L, B Himasri, Sharath Chandra Thota, Karthik Bharadwaj Tallapaka, Krishna Khairnar, Rakesh K Mishra,Divya Tej Sowpati
EPI_ISL_1360330	CSIR-National Environmental Engineering Research Institute	CSIR-Centre for Cellular and Molecular Biology - INSACOG	Lamuk Zaveri, Ara Sreenivas, Onkar Kulkarni, Sofia Banu, Shreekant Verma,Amareshwar Vodapalli, Viswagithe S L, B Himasri, Sharath Chandra Thota, Karthik Bharadwaj Tallapaka, Krishna Khairnar, Rakesh K Mishra,Divya Tej Sowpati
EPI_ISL_1360331, EPI_ISL_1360332	CSIR-National Environmental Engineering Research Institute	CSIR-Centre for Cellular and Molecular Biology - INSACOG	Ara Sreenivas, Onkar Kulkarni, Lamuk Zaveri, Sofia Banu, Shreekant Verma, Amareshwar Vodapalli , Viswagithe S L, B Himasri, Sharath Chandra Thota, Karthik Bharadwaj Tallapaka, Krishna Khairnar, Rakesh K Mishra,Divya Tej Sowpati

EPI_ISL_1360333	CSIR-National Environmental Engineering Research Institute	CSIR-Centre for Cellular and Molecular Biology - INSACOG	Sofia Banu, Lamuk Zaveri, Ara Sreenivas, Shreekant Verma, Onkar Kulkarni, Amareshwar Vodapalli ,Viswagithe S L, B Himasri, Sharath Chandra Thota, Karthik Bharadwaj Tallapaka, Krishna Khairnar, Rakesh K Mishra,Divya Tej Sowpati
EPI_ISL_1360335, EPI_ISL_1360336	CSIR-National Environmental Engineering Research Institute	CSIR-Centre for Cellular and Molecular Biology - INSACOG	Onkar Kulkarni, Lamuk Zaveri, Ara Sreenivas, Sofia Banu, Shreekant Verma, Amareshwar Vodapalli , Viswagithe S L, B Himasri, Sharath Chandra Thota, Karthik Bharadwaj Tallapaka, Krishna Khairnar, Rakesh K Mishra,Divya Tej Sowpati
EPI_ISL_1360338	CSIR-National Environmental Engineering Research Institute	CSIR-Centre for Cellular and Molecular Biology - INSACOG	Lamuk Zaveri, Ara Sreenivas, Onkar Kulkarni, Sofia Banu, Shreekant Verma,Amareshwar Vodapalli, Viswagithe S L, B Himasri, Sharath Chandra Thota, Karthik Bharadwaj Tallapaka, Krishna Khairnar, Rakesh K Mishra,Divya Tej Sowpati
EPI_ISL_1360407, EPI_ISL_1360412	Laboratorio de Enfermedades Emergentes y Reemergentes	Laboratorio de Enfermedades Emergentes y Reemergentes	Vega-Magaña Natali, Peña-Rodriguez Marcela, Viera-Segura Oliver, García-Chagollan Mariel, Hernández-Bello Jorge, Muñoz-Valle José Francisco
EPI_ISL_1363082, EPI_ISL_1363084, EPI_ISL_1363085, EPI_ISL_1363086, EPI_ISL_1363087, EPI_ISL_1363088, EPI_ISL_1363090, EPI_ISL_1363091, EPI_ISL_1363092, EPI_ISL_1363093, EPI_ISL_1363094, EPI_ISL_1363095, EPI_ISL_1363096, EPI_ISL_1363097, EPI_ISL_1363098, EPI_ISL_1363099, EPI_ISL_1363101, EPI_ISL_1363102, EPI_ISL_1363103, EPI_ISL_1363104, EPI_ISL_1363105, EPI_ISL_1363106, EPI_ISL_1363107, EPI_ISL_1363108, EPI_ISL_1363109, EPI_ISL_1363111, EPI_ISL_1363113			
see above	Virological laboratory FBIH "The Center for Hygiene and Epidemiology in the Khabarovsk Region"	Group of Genomics and Postgenomic Technologies of Central Research Institute of Epidemiology	Samoilov AE, Kaptelova VV, Korneenko EV, Valdokhina AV, Bulanenko VP, Saenko SS, Lebedeva LA, Shubina YA, Savosina LV, Zhaleyko ZP, Lukyanov AV, Golubeva AG, Speranskaya AS, Tivanova EV, Kondrasheva LY, Akimkin VG
EPI_ISL_1363119, EPI_ISL_1363120, EPI_ISL_1363121, EPI_ISL_1363122, EPI_ISL_1363123	Borneo Medical Centre	Institute of Health and Community Medicine	Chan Chia Jui, David Perera, Ooi Mong How, Chua Hock Hin, Tonnii Sia Loong Loong, Wong Jyn Shan, Wong Kiing Aik
EPI_ISL_1363758	Departments of Intensive Care Medicine, and Clinical lab, Osaka Women's and Children's Hospital	Department of Developmental Medicine, Research Institute,	Itaru Yanagihara, Yukiko Nakura, Muneyuki Takeuchi, Shinobu Ida
EPI_ISL_1363759	Departments of Intensive Care Medicine, and Clinical lab, Osaka Women's and Children's Hospital	Department of Developmental Medicine, Research Institute, Osaka Women's and Children's Hospital	Itaru Yanagihara, Yukiko Nakura, Muneyuki Takeuchi, Shinobu Ida
EPI_ISL_1372283, EPI_ISL_1372286, EPI_ISL_1372301, EPI_ISL_1372304, EPI_ISL_1372307, EPI_ISL_1372309, EPI_ISL_1372310, EPI_ISL_1372312	WHO National Influenza Centre Russian Federation	WHO National Influenza Centre Russian Federation	Andrey Komissarov, Artem Fadeev, Anna Ivanova, Kseniya Komissarova, Dmitry Bazhenov, Tamila Musaeva, Maria Timofeeva, Veronika Eder, Maria Pisareva, Daria Danilenko, Ksenia Safina, Elena Nabieva, Georgii Bazykin, Dmitry Lioznov
EPI_ISL_1372400, EPI_ISL_1372401, EPI_ISL_1372402, EPI_ISL_1372403, EPI_ISL_1372404, EPI_ISL_1372405, EPI_ISL_1372406, EPI_ISL_1372407, EPI_ISL_1372408	Pathogenic Microorganisms Variability Laboratory	WHO National Influenza Centre Russian Federation	Andrey Komissarov, Artem Fadeev, Anna Ivanova, Kseniya Komissarova, Dmitry Bazhenov, Daria Danilenko, Ksenia Safina, Elena Nabieva, Georgii Bazykin, Nadezhda Kuznetsova, Elena Shidlovskaya, Sergey Alkhovsky, Tatyana Vishnevskaya, Elizaveta Divisenko, Alexey Shchetinin, Maria Nikiforova, Andrey Pochtovyy, Evgeny Usachev, Elena Vokalova, Maxim Rubalsky, Oleg Rubalsky, Artem Tkachuk, Vladimir Gushchin, Alexander Gintsburg, Dmitry Lioznov
EPI_ISL_1379391	Department of Molecular and Translational Medicine, Section of Microbiology, University of Brescia, ASST Spedali Civili, Brescia	Department of Molecular and Translational Medicine, Section of Microbiology, University of Brescia, ASST Spedali Civili, Brescia	Simona Fiorentini, Serena Messali, Alberto Zani, Francesca Caccuri, Arnaldo Caruso
EPI_ISL_1379435	Department of Molecular and Translational Medicine, Section of Microbiology, University of Brescia, ASST Spedali Civili, Brescia	Department of Molecular and Translational Medicine, Section of Microbiology, University of Brescia, ASST Spedali Civili, Brescia	Simona Fiorentini, Serena Messali, Alberto Zani, Erika Scaltriti, Francesca Caccuri, Arnaldo Caruso
EPI_ISL_1379733	Stanford Health Care	Stanford University School of Medicine, Clinical Virology Laboratory	Fumiko Yamamoto, Malaya K. Sahoo, Mamdouh Sibai, Daniel Solis, Michelle Vergese, ChunHong Huang, James Zehnder, and Benjamin A. Pinsky
EPI_ISL_1380060, EPI_ISL_1380061, EPI_ISL_1380062, EPI_ISL_1380063, EPI_ISL_1380064, EPI_ISL_1380065	Laboratorio Biologia Molecolare Sars Cov2 - UOC Laboratorio Analisi - Servizio Medicina di Laboratorio, Ospedale "San Francesco" - ATS-ASSL Nuoro	Laboratorio specialistico UOC Ematologia - Ospedale "San Francesco" - ATS-ASSL Nuoro	Piras Giovanna, Aspproni Rosanna,,Fiamma Maura, Monne Maria Itria, Palmas Angelo Domenico, Lo Maglio Iana, Mameli Giuseppe
EPI_ISL_1381066, EPI_ISL_1381067	LACEN do Mato Grosso do Sul	Instituto Adolfo Lutz, Interdiciplinary Procedures Center, Strategic Laboratory	Claudio Tavares Sacchi, Claudia Regina Gonçalves, Erica Valessa Ramos Gomes, Karoline Rodrigues Campos, Caio Vinicius Dias Lopes
EPI_ISL_1381146	Hospital	National Reference Center for Viruses of Respiratory Infections, Institut Pasteur, Paris	Marion Barbet, Sylvie Behillil, Méline Bizard, Angela Brisebarre, Camille Capel, Louise Lefrançois, Etienne Simon-Lorière, Vincent Enouf, Maud Vanpeene, Sylvie van der Werf,Hecquet Denise
EPI_ISL_1381161	Labo Analyses Med	National Reference Center for Viruses of Respiratory Infections, Institut Pasteur, Paris	Marion Barbet, Sylvie Behillil, Méline Bizard, Angela Brisebarre, Camille Capel, Louise Lefrançois, Etienne Simon-Lorière, Vincent Enouf, Maud Vanpeene, Sylvie van der Werf,Besson J
EPI_ISL_1381165, EPI_ISL_1381166, EPI_ISL_1381167, EPI_ISL_1381168, EPI_ISL_1381169, EPI_ISL_1381170, EPI_ISL_1381171, EPI_ISL_1381172, EPI_ISL_1381173, EPI_ISL_1381174, EPI_ISL_1381175, EPI_ISL_1381176, EPI_ISL_1381177, EPI_ISL_1381178, EPI_ISL_1381179, EPI_ISL_1381180, EPI_ISL_1381181, EPI_ISL_1381182, EPI_ISL_1381183, EPI_ISL_1381184, EPI_ISL_1381185, EPI_ISL_1381186, EPI_ISL_1381187, EPI_ISL_1381188, EPI_ISL_1381189, EPI_ISL_1381190, EPI_ISL_1381191, EPI_ISL_1381192, EPI_ISL_1381193, EPI_ISL_1381194, EPI_ISL_1381195, EPI_ISL_1381196, EPI_ISL_1381197, EPI_ISL_1381198, EPI_ISL_1381199, EPI_ISL_1381201, EPI_ISL_1381202			
see above	Hospital	National Reference Center for Viruses of Respiratory Infections, Institut Pasteur, Paris	Marion Barbet, Sylvie Behillil, Méline Bizard, Angela Brisebarre, Camille Capel, Louise Lefrançois, Etienne Simon-Lorière, Vincent Enouf, Maud Vanpeene, Sylvie van der Werf,Bressollette CéLine
EPI_ISL_1381231, EPI_ISL_1381232	Hospital	National Reference Center for Viruses of Respiratory Infections, Institut Pasteur, Paris	Marion Barbet, Sylvie Behillil, Méline Bizard, Angela Brisebarre, Camille Capel, Louise Lefrançois, Etienne Simon-Lorière, Vincent Enouf, Maud Vanpeene, Sylvie van der Werf,Pilorge LéA
EPI_ISL_1381386	Centers for Disease Control, R.O.C. (Taiwan)	Centers for Disease Control, R.O.C. (Taiwan)	Ji-Rong Yang, Yu-Chi Lin, Jung-Jung Mu, Ming-Tsan Liu
EPI_ISL_1381822, EPI_ISL_1381823, EPI_ISL_1381824, EPI_ISL_1381825, EPI_ISL_1381826, EPI_ISL_1381827	Hospital	National Reference Center for Viruses of Respiratory Infections, Institut Pasteur, Paris	Marion Barbet, Sylvie Behillil, Méline Bizard, Angela Brisebarre, Camille Capel, Louise Lefrançois, Etienne Simon-Lorière, Vincent Enouf, Maud Vanpeene, Sylvie van der Werf,Bressollette CéLine
EPI_ISL_1381831	Hospital	National Reference Center for Viruses of Respiratory Infections, Institut Pasteur, Paris	Marion Barbet, Sylvie Behillil, Méline Bizard, Angela Brisebarre, Camille Capel, Louise Lefrançois, Etienne Simon-Lorière, Vincent Enouf, Maud Vanpeene, Sylvie van der Werf,Pilorge LéA
EPI_ISL_1392889, EPI_ISL_1392890, EPI_ISL_1392891	Centrum Badawczo-Rozwojowe Invicta (Invicta sp. z o.o.)	Diagtron Laboratoria	Maciej Kosinski, Natalia Derewonko, Lukasz Rabalski, Karolina Gackowska, Celina Cybulska, Krzysztof Lukaszczuk
EPI_ISL_1396347	Laboratorio del Hospital Regional Ushuaia Gdor. Ernesto Campos	Nodo de Secuenciación Tierra del Fuego - Hospital Regional Ushuaia - Centro Austral De Investigaciones Científicas - Universidad Nacional De Tierra Del Fuego on behalf of 'Proyecto Argentino Interinstitucional de genomica de SARS-CoV-2' (PAIS Consortium)	Carina Andrea De Roccis, Gabriel Alejandro Castro, Silvana Beatriz Cáceres, Carolina Beatriz Yulan, Manuel Fabian Boutureira, Alejandro Ezequiel Rojas, Fernando Gallego, Santiago Guillermo Ceballos, Cristina Fernanda Nardi, Ivan Dario Gramundi
EPI_ISL_1400529	WHO National Influenza Centre Russian Federation	WHO National Influenza Centre Russian Federation	Andrey Komissarov, Artem Fadeev, Anna Ivanova, Kseniya Komissarova, Dmitry Bazhenov, Tamila Musaeva, Maria Timofeeva, Veronika Eder, Maria Pisareva, Daria Danilenko, Ksenia Safina, Elena Nabieva, Georgii Bazykin, Dmitry Lioznov
EPI_ISL_1400532, EPI_ISL_1400533, EPI_ISL_1400534, EPI_ISL_1400535, EPI_ISL_1400536, EPI_ISL_1400537, EPI_ISL_1400538, EPI_ISL_1400539, EPI_ISL_1400540, EPI_ISL_1400560	WHO National Influenza Centre Russian Federation	WHO National Influenza Centre Russian Federation	Andrey Komissarov, Artem Fadeev, Anna Ivanova, Kseniya Komissarova, Dmitry Bazhenov, Mikhail Bakaev, Daria Danilenko, Ksenia Safina, Elena Nabieva, Georgii Bazykin, Dmitry Lioznov
EPI_ISL_1402423, EPI_ISL_1402425	Private clinic of Biogen Med, Tashkent, Uzbekistan	Center of Genomics and bioinformatics, Bioinformatics laboratory	Mirzakamol S Ayubov, Zabardast T Buriev, Mukhammadjon H Mirzakhmedov, Abdurakhmon N Yusupov, Shukhrat E Shermatov, Ibrokhim Y Abdurakhmonov.

EPI_ISL_1404375	Klinisch Laboratorium ZNA	Klinisch Laboratorium ZNA	Verstrepen et al.
EPI_ISL_1406651	HOPITAL DE VICHY	CHU Clermont-Ferrand, service de virologie	Bisseux Maxime, Mirand Audrey, Combes Patricia, Henquell Cécile
EPI_ISL_1406681	Centre Jean Perrin	CHU Clermont-Ferrand, service de virologie	Bisseux Maxime, Mirand Audrey, Combes Patricia, Henquell Cécile
EPI_ISL_1406694	National Center of Infectious and Parasitic Diseases	National Center of Infectious and Parasitic Diseases	Alexiev et al
EPI_ISL_1416992	Outre mer	National Reference Center for Viruses of Respiratory Infections, Institut Pasteur, Paris	Marion Barbet, Sylvie Behillil, Méline Bizard,Frédéric Lemoine,Corinne Maufrais,Christophe Malabat, Angela Brisebarre, Camille Capel, Louise Lefrançois, Etienne Simon-Lorière, Vincent Enouf, Maud Vanpeene, Sylvie van der Werf,Anne Holstein
EPI_ISL_1416993, EPI_ISL_1416994	Outre mer	National Reference Center for Viruses of Respiratory Infections, Institut Pasteur, Paris	Marion Barbet, Sylvie Behillil, Méline Bizard,Frédéric Lemoine,Corinne Maufrais,Christophe Malabat, Angela Brisebarre, Camille Capel, Louise Lefrançois, Etienne Simon-Lorière, Vincent Enouf, Maud Vanpeene, Sylvie van der Werf,GréGoire Potiron
EPI_ISL_1416995	Outre mer	National Reference Center for Viruses of Respiratory Infections, Institut Pasteur, Paris	Marion Barbet, Sylvie Behillil, Méline Bizard,Frédéric Lemoine,Corinne Maufrais,Christophe Malabat, Angela Brisebarre, Camille Capel, Louise Lefrançois, Etienne Simon-Lorière, Vincent Enouf, Maud Vanpeene, Sylvie van der Werf,StéPhanie Guyomard-Rabenirina
EPI_ISL_1416996, EPI_ISL_1416997	Outre mer	National Reference Center for Viruses of Respiratory Infections, Institut Pasteur, Paris	Marion Barbet, Sylvie Behillil, Méline Bizard,Frédéric Lemoine,Corinne Maufrais,Christophe Malabat, Angela Brisebarre, Camille Capel, Louise Lefrançois, Etienne Simon-Lorière, Vincent Enouf, Maud Vanpeene, Sylvie van der Werf,Anne Holstein
EPI_ISL_1417067	Hospital	National Reference Center for Viruses of Respiratory Infections, Institut Pasteur, Paris	Marion Barbet, Sylvie Behillil, Méline Bizard,Frédéric Lemoine,Corinne Maufrais,Christophe Malabat, Angela Brisebarre, Camille Capel, Louise Lefrançois, Etienne Simon-Lorière, Vincent Enouf, Maud Vanpeene, Sylvie van der Werf,OphéLie Said-Delattre
EPI_ISL_1417069	Hospital	National Reference Center for Viruses of Respiratory Infections, Institut Pasteur, Paris	Marion Barbet, Sylvie Behillil, Méline Bizard,Frédéric Lemoine,Corinne Maufrais,Christophe Malabat, Angela Brisebarre, Camille Capel, Louise Lefrançois, Etienne Simon-Lorière, Vincent Enouf, Maud Vanpeene, Sylvie van der Werf,StéPhanie Guyomard-Rabenirina
EPI_ISL_1417070	Hospital	National Reference Center for Viruses of Respiratory Infections, Institut Pasteur, Paris	Marion Barbet, Sylvie Behillil, Méline Bizard,Frédéric Lemoine,Corinne Maufrais,Christophe Malabat, Angela Brisebarre, Camille Capel, Louise Lefrançois, Etienne Simon-Lorière, Vincent Enouf, Maud Vanpeene, Sylvie van der Werf,OphéLie Said-Delattre
EPI_ISL_1418308	National Institute of Infectious Diseases-Prof. Dr. Matei Bals Molecular Diagnostics Laboratory	National Institute of Infectious Diseases-Prof. Dr. Matei Bals Molecular Diagnostics Laboratory	Corina Casangiu, Leontina Banica, Marius Surleac, Ovidiu Vlaicu, Ionelia Nicolae, Simona Paraschiv, Dan Otelea
EPI_ISL_1424065	Institute for Medical Research, Infectious Disease Research Centre, National Institutes of Health, Ministry of Health Malaysia	Institute for Medical Research, Infectious Disease Research Centre, National Institutes of Health, Ministry of Health Malaysia	Suppiah J, Kamel K, Mohd Zawawi Z, Ramly N, Robert F, Thayan R
EPI_ISL_1435783, EPI_ISL_1435785, EPI_ISL_1435787, EPI_ISL_1435789, EPI_ISL_1435791, EPI_ISL_1435792, EPI_ISL_1435794, EPI_ISL_1435796, EPI_ISL_1435798, EPI_ISL_1435800	DNALAB Sdn. Bhd.	Malaysia Genome Institute	Mohd Noor Mat Isa, Irni Suhayu Sapian, Yusuf Muhammad Noor, Nurhezreen Md Iqbal, Mohd Faizal Abu Bakar, Enizza Kasim, Shamsidar Sopie, Siti Noraini Othman, Azrin Ahmad, Wong Yong Wee, Nor Azfa Johari.
EPI_ISL_1435802, EPI_ISL_1435804, EPI_ISL_1435806, EPI_ISL_1435808, EPI_ISL_1435810, EPI_ISL_1435812, EPI_ISL_1435814, EPI_ISL_1435816, EPI_ISL_1435817, EPI_ISL_1435820	Malaysia Genome Institute	Malaysia Genome Institute	Mohd Noor Mat Isa, Irni Suhayu Sapian, Yusuf Muhammad Noor, Nurhezreen Md Iqbal, Mohd Faizal Abu Bakar, Enizza Kasim, Shamsidar Sopie, Siti Noraini Othman, Azrin Ahmad, Nor Azfa Johari.
EPI_ISL_1437749	Institut National d'Hygiène	Unité Mixte Internationale TransVIHMI (UMI 233 IRD - U1175 INSERM - Université de Montpellier) IRD (Institut de recherche pour le développement)	Mounerou SALOU, Christelle BUTEL, Wembo A. HALATOKO,Issaka Maman, Abla A. KONOU, Amivi EHLAN, Adodo SADJI, Kokou TEGUENI,Sidonie A.M.KAGNISSODE, Akoélé SILIADIN, Alassane OURO-MEDELI, Messanh DOUFFAN,Déléma MABA,Sika DOSSIM, Améyo DORKENOO, Mireille PRINCE-DAVID,Anoumou DAGNRA,Laetitia SERRANO,Ahidjo AYOUBA,Eric DELAPORTE, Martine PEETERS
EPI_ISL_1443002	Institut National d'hygiène	"Unité Mixte Internationale TransVIHMI (UMI 233 IRD - U1175 INSERM - Université de Montpellier) IRD (Institut de recherche pour le développement)"	Mounerou SALOU, Christelle BUTEL, Wembo A. HALATOKO,Issaka Maman, Abla A. KONOU, Amivi EHLAN, Adodo SADJI, Kokou TEGUENI,Sidonie A.M.KAGNISSODE, Akoélé SILIADIN, Alassane OURO-MEDELI, Messanh DOUFFAN,Déléma MABA,Sika DOSSIM, Améyo DORKENOO, Mireille PRINCE-DAVID,Anoumou DAGNRA,Laetitia SERRANO,Ahidjo AYOUBA,Eric DELAPORTE, Martine PEETERS
EPI_ISL_1443196, EPI_ISL_1443197, EPI_ISL_1443198	Hospital Aliança	Hospital São Rafael - IDOR	Isadora Cristina de Siqueira, Aquiles Assunção Camelier, Elves A.P. Maciel, Margarida Celia L. C. Neves, Carolina Kymie Vasques Nonaka, Karoline Almeida Félix de Sousa, Victor Costa Araujo, Yasmin Santos Freitas Macêdo, Aurea Angelica Paste, Bruno Solano de Freitas Souza, Tiago Gräf
EPI_ISL_1443901, EPI_ISL_1443902	Hospital	National Reference Center for Viruses of Respiratory Infections, Institut Pasteur, Paris	Marion Barbet, Sylvie Behillil, Méline Bizard,Frédéric Lemoine,Corinne Maufrais,Christophe Malabat, Angela Brisebarre, Camille Capel, Louise Lefrançois, Etienne Simon-Lorière, Vincent Enouf, Maud Vanpeene, Sylvie van der Werf,CéLine Bressollette
EPI_ISL_1445275	Institut National d'Hygiène (INH)	Unité Mixte Internationale TransVIHMI (UMI 233 IRD - U1175 INSERM - Université de Montpellier)IRD (Institut de recherche pour le développement)	Mounerou SALOU, Christelle BUTEL, Wembo A. HALATOKO,Issaka Maman, Abla A. KONOU, Amivi EHLAN, Adodo SADJI, Kokou TEGUENI,Sidonie A.M.KAGNISSODE, Akoélé SILIADIN, Alassane OURO-MEDELI, Messanh DOUFFAN,Déléma MABA,Sika DOSSIM, Améyo DORKENOO, Mireille PRINCE-DAVID,Anoumou DAGNRA,Laetitia SERRANO,Ahidjo AYOUBA,Eric DELAPORTE, Martine PEETERS
EPI_ISL_1447339, EPI_ISL_1447341, EPI_ISL_1447343, EPI_ISL_1447348, EPI_ISL_1447349, EPI_ISL_1447350, EPI_ISL_1447353, EPI_ISL_1447354, EPI_ISL_1447355, EPI_ISL_1447356, EPI_ISL_1447357, EPI_ISL_1447358, EPI_ISL_1447359, EPI_ISL_1447360, EPI_ISL_1447361			
see above	Diagnostic and Research Center of Infectious Diseases, Medical Faculty, Andalas University	Diagnostic and Research Center of Infectious Diseases, Medical Faculty, Andalas University	Andani Eka Putra, Syafrizayanti, Ikhwan R. Sudji, Linosefa, Dede Rahman Agustian, Ayu Novita Trisnawati, Siskalil Fahma, Nia Ayuni Putri, Sekar Asri Tresnaningtyas, SM Rezvi, Fauzul Azhim, Dessy Arisanty, Gestina Aliska, Desmawati, Nita Afriani ,Syandrez Prima Putra, Juane Plantika Menra, Yolani Syaputri, Mutia Lailani
EPI_ISL_1468418	Secretaria Municipal de Saude de Birigui	Instituto Adolfo Lutz, Interdiciplinary Procedures Center, Strategic Laboratory	Claudio Tavares Sacchi, Claudia Regina Gonçalves, Erica Valessa Ramos Gomes, Karoline Rodrigues Campos, Caio Vinicius Dias Lopes
EPI_ISL_1468433, EPI_ISL_1468435, EPI_ISL_1468436, EPI_ISL_1468437, EPI_ISL_1468438, EPI_ISL_1468439, EPI_ISL_1468440, EPI_ISL_1468441	LACEN do Mato Grosso do Sul	Instituto Adolfo Lutz, Interdiciplinary Procedures Center, Strategic Laboratory	Claudio Tavares Sacchi, Claudia Regina Gonçalves, Erica Valessa Ramos Gomes, Karoline Rodrigues Campos, Caio Vinicius Dias Lopes
EPI_ISL_1469236, EPI_ISL_1469237	Diagnostic and Research Center of Infectious Diseases, Medical Faculty, Andalas University	Diagnostic and Research Center of Infectious Diseases, Medical Faculty, Andalas University	Andani Eka Putra, Syafrizayanti, Ikhwan R. Sudji, Linosefa, Dede Rahman Agustian, Ayu Novita Trisnawati, Siskalil Fahma, Nia Ayuni Putri, Sekar Asri Tresnaningtyas, SM Rezvi, Fauzul Azhim, Dessy Arisanty, Gestina Aliska, Desmawati, Nita Afriani ,Syandrez Prima Putra, Juane Plantika Menra, Yolani Syaputri, Mutia Lailani
EPI_ISL_1469279	RSUD Surakarta	National Institute of Health Research and Development	Kartika Dewi Puspa, Hana Apsari Pawestri, Subangkit, Arie Ardiansyah Nugraha, Hartanti Dian Ikawati, Nelly Puspandari, Krisna Pangesti, Vivi Setiawaty
EPI_ISL_1469290, EPI_ISL_1469291	National Institute Health Research and Development	National Institute of Health Research and Development	Nur Ika Hariastuti, Nike Susanti, Hana Apsari Pawestri, Subangkit, Kartika Dewi Puspa, Arie Ardiansyah Nugraha, Hartanti Dian Ikawati, Nelly Puspandari, Vivi Setiawaty
EPI_ISL_1469450, EPI_ISL_1469451, EPI_ISL_1469452, EPI_ISL_1469453, EPI_ISL_1469454, EPI_ISL_1469455, EPI_ISL_1469456, EPI_ISL_1469457, EPI_ISL_1469458, EPI_ISL_1469459, EPI_ISL_1469460, EPI_ISL_1469461, EPI_ISL_1469462, EPI_ISL_1469463, EPI_ISL_1469465, EPI_ISL_1469466, EPI_ISL_1469467, EPI_ISL_1469468, EPI_ISL_1469469, EPI_ISL_1469470, EPI_ISL_1469471, EPI_ISL_1469472, EPI_ISL_1469474, EPI_ISL_1469475, EPI_ISL_1469476, EPI_ISL_1469477, EPI_ISL_1469478, EPI_ISL_1469479, EPI_ISL_1469480, EPI_ISL_1469481, EPI_ISL_1469482, EPI_ISL_1469483, EPI_ISL_1469484, EPI_ISL_1469485, EPI_ISL_1469486, EPI_ISL_1469488, EPI_ISL_1469489, EPI_ISL_1469490, EPI_ISL_1469491, EPI_ISL_1469492			
see above	Center for Laboratory Medicine St. Gallen	Center for Laboratory Medicine St. Gallen	Yannick Gerth
EPI_ISL_1469549	FUNDACAO HOSPITALAR DE SAPUCAIA DO SUL	Epiclin	Fernando Hayashi Sant'Anna, Ana Paula Muterle, Janira Prichula, Juliana Comerlato, Carolina Comerlato, Eliana Márcia Da Ros Wendland
EPI_ISL_1472367, EPI_ISL_1472368, EPI_ISL_1472369, EPI_ISL_1472370, EPI_ISL_1472371	Department of Pathology, National Institute of Infectious Diseases	Department of Pathology, National Institute of Infectious Diseases	Tsuyoshi Sekizuka, Kentaro Itokawa, Rina Tanaka, Masanori Hashino, Makoto Kuroda, Harutaka Katano, Tadaki Suzuki
EPI_ISL_1477044	Private clinic of Biogen Med, Tashkent, Uzbekistan	Center of Genomics and bioinformatics, Bioinformatics laboratory	Mirzakamol S Ayubov, Zabardast T Buriev, Mukhammadjon H Mirzakhmedov, Abdurakhmon N Yusupov, Shukhrat E Shermatov, Ibrokhim Y Abdurakhmonov.

EPI_ISL_1478754	Microbiology Department. Complexo Hospitalario Universitario de Vigo	Microbiology Department. Complexo Hospitalario Universitario de Vigo	Alfaia N, Alonso I, Alvarez M, Cabrera JJ, Carballo R, Cores O, Cortizo S, del-Campo V, Martinez L, Mediero G, Perez S, Potel C, Regueiro B, Rey S, Vasallo FJ
EPI_ISL_1483319, EPI_ISL_1483320	Microbiology Department. Complexo Hospitalario Universitario de Vigo	Microbiology Department. Complexo Hospitalario Universitario de Vigo	Alfaya N, Alonso I, Alvarez M, Cabrera JJ, Carballo R, Cores O, Cortizo S, del-Campo V, Martinez L, Mediero G, Perez S, Potel C, Regueiro B, Rey S, Vasallo FJ
EPI_ISL_1491614, EPI_ISL_1491617, EPI_ISL_1491628, EPI_ISL_1491629, EPI_ISL_1491630, EPI_ISL_1491631, EPI_ISL_1491632, EPI_ISL_1491633, EPI_ISL_1491634, EPI_ISL_1491635, EPI_ISL_1491636, EPI_ISL_1491637, EPI_ISL_1491638, EPI_ISL_1491639, EPI_ISL_1491640, EPI_ISL_1491641, EPI_ISL_1491642, EPI_ISL_1491643, EPI_ISL_1491644, EPI_ISL_1491645, EPI_ISL_1491646, EPI_ISL_1491647, EPI_ISL_1491648, EPI_ISL_1491649, EPI_ISL_1491650, EPI_ISL_1491651, EPI_ISL_1491652, EPI_ISL_1491653, EPI_ISL_1491654, EPI_ISL_1491655, EPI_ISL_1491656, EPI_ISL_1491657, EPI_ISL_1491658, EPI_ISL_1491659, EPI_ISL_1491660, EPI_ISL_1491662, EPI_ISL_1491663, EPI_ISL_1491664, EPI_ISL_1491665, EPI_ISL_1491666, EPI_ISL_1491667, EPI_ISL_1491668, EPI_ISL_1491669, EPI_ISL_1491670			
see above	Pathogenic Microorganisms Variability Laboratory	WHO National Influenza Centre Russian Federation	Andrey Komissarov, Artem Fadeev, Anna Ivanova, Kseniya Komissarova, Alexey Masharsky, Maria Baturova, Dmitry Bazhenov, Daria Danilenko, Ksenia Safina, Elena Nabieva, Georgii Bazykin, Nadezhda Kuznetsova, Elena Shidlovskaya, Sergey Alkhovsky, Tatyana Vishnevskaya, Elizaveta Divisenko, Alexey Shchetinin, Maria Nikiforova, Andrey Pochtovyy, Evgeny Usachev, Elena Vokalova, Maxim Rubalsky, Oleg Rubalsky, Artem Tkachuk, Vladimir Gushchin, Alexander Gintsburg, Dmitry Lioznov
EPI_ISL_1493597, EPI_ISL_1493598, EPI_ISL_1493599, EPI_ISL_1493600	LACEN do Estado de Rondonia	Instituto Adolfo Lutz, Interdiciplinary Procedures Center, Strategic Laboratory	Claudio Tavares Sacchi, Claudia Regina Gonçalves, Erica Valessa Ramos Gomes, Karoline Rodrigues Campos, Caio Vinicius Dias Lopes
EPI_ISL_1494728, EPI_ISL_1494729, EPI_ISL_1494730	Centro de Investigación en Ciencias de la Salud y Biomedicina, U.A.S.L.P.	Centro de Investigación en Ciencias de la Salud y Biomedicina, U.A.S.L.P.	MD PhD Sofía Bernal Silva
EPI_ISL_1500960, EPI_ISL_1500961, EPI_ISL_1500962, EPI_ISL_1500963, EPI_ISL_1500964, EPI_ISL_1500965, EPI_ISL_1500966, EPI_ISL_1500967, EPI_ISL_1500968, EPI_ISL_1500969, EPI_ISL_1500970, EPI_ISL_1500971, EPI_ISL_1500994, EPI_ISL_1500995, EPI_ISL_1500996, EPI_ISL_1500997, EPI_ISL_1500998, EPI_ISL_1500999, EPI_ISL_1501000, EPI_ISL_1501001, EPI_ISL_1501002, EPI_ISL_1501003, EPI_ISL_1501218			
see above	HEGP - Laboratoire de Virologie	HEGP - Laboratoire de Virologie	David Veyer
EPI_ISL_1502814, EPI_ISL_1502816, EPI_ISL_1502817	Infectious Diseases Department , Instituto Nacional de Ciencias Medicas y Nutrición	Instituto Nacional de Ciencias Medicas y Nutricion Infectious Diseases	Guillermo M. Ruiz-Palacios, Pilar Ramos Cervantes, Violeta Ibarra Gonzalez, Fernando Ledesma Barrientos, Miriam Arciniega Fuentes , Fernando Arteaga Cabello, Diana Paola Davalos Martinez, Luis Alberto Garcia Andrade, Luz Elena Cervantes Villar
EPI_ISL_1502887, EPI_ISL_1502888, EPI_ISL_1502889, EPI_ISL_1502890, EPI_ISL_1502891, EPI_ISL_1502892, EPI_ISL_1502893, EPI_ISL_1502894, EPI_ISL_1502895, EPI_ISL_1502896, EPI_ISL_1502897, EPI_ISL_1502898, EPI_ISL_1502899, EPI_ISL_1502900, EPI_ISL_1502901, EPI_ISL_1502903, EPI_ISL_1502905, EPI_ISL_1502907, EPI_ISL_1502908, EPI_ISL_1502911, EPI_ISL_1502912, EPI_ISL_1502913, EPI_ISL_1502915, EPI_ISL_1502917, EPI_ISL_1502919, EPI_ISL_1502921, EPI_ISL_1502923, EPI_ISL_1502925, EPI_ISL_1502928, EPI_ISL_1502929, EPI_ISL_1502930, EPI_ISL_1502931, EPI_ISL_1502933, EPI_ISL_1502934, EPI_ISL_1502935, EPI_ISL_1502936, EPI_ISL_1502937, EPI_ISL_1502939, EPI_ISL_1502941, EPI_ISL_1502943, EPI_ISL_1502944, EPI_ISL_1502946, EPI_ISL_1502947, EPI_ISL_1502949, EPI_ISL_1502951, EPI_ISL_1502954, EPI_ISL_1502955, EPI_ISL_1502956, EPI_ISL_1502957, EPI_ISL_1502959, EPI_ISL_1502961, EPI_ISL_1502963, EPI_ISL_1502966, EPI_ISL_1502967, EPI_ISL_1502968, EPI_ISL_1502970, EPI_ISL_1502972, EPI_ISL_1502978, EPI_ISL_1502983, EPI_ISL_1502984, EPI_ISL_1502985, EPI_ISL_1502986, EPI_ISL_1502987, EPI_ISL_1502988, EPI_ISL_1502989, EPI_ISL_1502990, EPI_ISL_1502991, EPI_ISL_1502992, EPI_ISL_1502993, EPI_ISL_1502994, EPI_ISL_1502995, EPI_ISL_1502996, EPI_ISL_1502998, EPI_ISL_1502999, EPI_ISL_1503003			
see above	Gorgas Memorial Laboratory of Health Studies	Gorgas Memorial Laboratory of Health Studies	Gonzalez Claudia, Leyda Abrego, Moreno Ambar, Oris Chavarria, Jessica Gondola, Marlenne Castillo, Ortiz Alma, Castillo Jorge, Moreno Brechla, Franco Danilo, Lopez-Verges Sandra, Martinez Alexander
EPI_ISL_1508848	Medical Genetics Laboratory, Regional Centre of Medical Genetics, Emergency County Hospital Craiova	Medical Genetics Laboratory, Regional Centre of Medical Genetics, Emergency County Hospital Craiova	Anca-Lelia (Riza) Costache, Ioana Streata, Stefania Dorobantu, Adina Dragos, Mihai Cucu, Ana-Maria Buga, Razvan Plesea, Andrei Pirvu, Elena Plesea, Monica Cara, Mihai Ioana
EPI_ISL_1508953	Institut National d'hygiène	Unité Mixte Internationale TransVIHMI (UMI 233 IRD - U1175 INSERM - Université de Montpellier) IRD (Institut de recherche pour le développement)	Mounerou SALOU, Christelle BUTEL, Wembo A. HALATOKO, Amivi EHLAN, Abla A. KONOU, Issaka Maman,Syntyche DEVATCHAGNI, Adodo SADJI, Kokou TEGUENI,Koku AGBODEKA, Sidonie A.M.KAGNISSODE, Akoélé SILIADIN, Alassane OURO-MEDELI, Messanh DOUFFAN,Déléma MABA,Sika DOSSIM, Améyo DORKENOO, Mireille PRINCE-DAVID,Anoumou DAGNRA, Laetitia SERRANO, Ahidjo AYOUBA, Eric DELAPORTE, Martine PEETERS
EPI_ISL_1508956	Laboratoire Biolim/FSS/UL	Unité Mixte Internationale TransVIHMI (UMI 233 IRD - U1175 INSERM - Université de Montpellier) IRD (Institut de recherche pour le développement)	Mounerou SALOU, Christelle BUTEL, Wembo A. HALATOKO, Amivi EHLAN, Abla A. KONOU, Issaka Maman, Syntyche DEVATCHAGNI, Adodo SADJI, Kokou TEGUENI,Koku AGBODEKA, Sidonie A.M.KAGNISSODE, Akoélé SILIADIN, Alassane OURO-MEDELI, Messanh DOUFFAN,Déléma MABA,Sika DOSSIM, Améyo DORKENOO, Mireille PRINCE-DAVID,Anoumou DAGNRA, Laetitia SERRANO,Ahidjo AYOUBA, Eric DELAPORTE, Martine PEETERS
EPI_ISL_1508958	Institut National d'hygiène	Unité Mixte Internationale TransVIHMI (UMI 233 IRD - U1175 INSERM - Université de Montpellier) IRD (Institut de recherche pour le développement)	Mounerou SALOU, Christelle BUTEL, Wembo A. HALATOKO, Amivi EHLAN, Abla A. KONOU, Issaka Maman,Syntyche DEVATCHAGNI, Adodo SADJI, Kokou TEGUENI,Koku AGBODEKA, Sidonie A.M.KAGNISSODE, Akoélé SILIADIN, Alassane OURO-MEDELI, Messanh DOUFFAN,Déléma MABA,Sika DOSSIM, Améyo DORKENOO, Mireille PRINCE-DAVID,Anoumou DAGNRA, Laetitia SERRANO,Ahidjo AYOUBA, Eric DELAPORTE, Martine PEETERS
EPI_ISL_1508960	Institut National d'hygiène	Unité Mixte Internationale TransVIHMI (UMI 233 IRD - U1175 INSERM - Université de Montpellier) IRD (Institut de recherche pour le développement)	Mounerou SALOU, Christelle BUTEL, Wembo A. HALATOKO, Amivi EHLAN, Abla A. KONOU, Issaka Maman, Syntyche DEVATCHAGNI, Adodo SADJI, Kokou TEGUENI,Koku AGBODEKA, Sidonie A.M.KAGNISSODE, Akoélé SILIADIN, Alassane OURO-MEDELI, Messanh DOUFFAN,Déléma MABA,Sika DOSSIM, Améyo DORKENOO, Mireille PRINCE-DAVID,Anoumou DAGNRA, Laetitia SERRANO,Ahidjo AYOUBA, Eric DELAPORTE, Martine PEETERS
EPI_ISL_1510669, EPI_ISL_1510670, EPI_ISL_1510671, EPI_ISL_1510672, EPI_ISL_1510673, EPI_ISL_1510674, EPI_ISL_1510675, EPI_ISL_1510676, EPI_ISL_1510677, EPI_ISL_1510678, EPI_ISL_1510679, EPI_ISL_1510680, EPI_ISL_1510681, EPI_ISL_1510682, EPI_ISL_1510683, EPI_ISL_1510684, EPI_ISL_1510685, EPI_ISL_1510686			
see above	University of Rome Tor Vergata: Departm Experim Medicine Chair of Virology	University of Rome Tor Vergata: Departm Experim Medicine Chair of Virology	Maria Concetta Bellocchi
EPI_ISL_1511400, EPI_ISL_1511401, EPI_ISL_1511402	Hospital General de México, Medicina Genómica	Hospital General de México, Medicina Genómica	Ana Laura Sánchez-Sandoval, María Eligia Juárez-Torres, Mariana Villalba-De la Peña, Siddhartha Ríos-Zaragoza, Guadalupe Mercedes Lucía Guerrero-Avendaño, Eira Valeria Barrón-Palma
EPI_ISL_1516898	Hospital	National Reference Center for Viruses of Respiratory Infections, Institut Pasteur, Paris	Marion Barbet, Sylvie Behillil, Méline Bizard, Angela Brisebarre, Camille Capel,Frédéric Lemoine,Corinne Maufrais,Christophe Malabat,Pierre Lechat, Louise Lefrançois, Etienne Simon-Lorière, Vincent Enouf, Maud Vanpeene, Sylvie van der Werf,FrançOise Jouble
EPI_ISL_1517037, EPI_ISL_1517038, EPI_ISL_1517040, EPI_ISL_1517042, EPI_ISL_1517044, EPI_ISL_1517048, EPI_ISL_1517050, EPI_ISL_1517053	Hospital	National Reference Center for Viruses of Respiratory Infections, Institut Pasteur, Paris	Marion Barbet, Sylvie Behillil, Méline Bizard, Angela Brisebarre, Camille Capel,Frédéric Lemoine,Corinne Maufrais,Christophe Malabat, Louise Lefrançois, Etienne Simon-Lorière, Vincent Enouf, Maud Vanpeene, Sylvie van der Werf,LéA Pilorge
EPI_ISL_1517085, EPI_ISL_1517088	Labo Analyses Med	National Reference Center for Viruses of Respiratory Infections, Institut Pasteur, Paris	Marion Barbet, Sylvie Behillil, Méline Bizard, Angela Brisebarre, Camille Capel,Frédéric Lemoine,Corinne Maufrais,Christophe Malabat, Louise Lefrançois, Etienne Simon-Lorière, Vincent Enouf, Maud Vanpeene, Sylvie van der Werf,J Besson
EPI_ISL_1517198	Labo Analyses Med	National Reference Center for Viruses of Respiratory Infections, Institut Pasteur, Paris	Marion Barbet, Sylvie Behillil, Méline Bizard, Angela Brisebarre, Camille Capel,Frédéric Lemoine,Corinne Maufrais,Christophe Malabat, Louise Lefrançois, Etienne Simon-Lorière, Vincent Enouf, Maud Vanpeene, Sylvie van der Werf,Nabil Gastli
EPI_ISL_1517387	Inciensa, Instituto Costarricense de Investigación y Enseñanza en Nutrición y Salud	Inciensa, Instituto Costarricense de Investigación y Enseñanza en Nutrición y Salud	Cristian Peréz-Corrales, Valeria Peralta-Barquero & Patricia Salas
EPI_ISL_1517388, EPI_ISL_1517389	Inciensa, Instituto Costarricense de Investigación y Enseñanza en Nutrición y Salud	Inciensa, Instituto Costarricense de Investigación y Enseñanza en Nutrición y Salud	Cristian Peréz-Corrales & Valeria Peralta-Barquero
EPI_ISL_1517390	Inciensa, Instituto Costarricense de Investigación y Enseñanza en Nutrición y Salud	Inciensa, Instituto Costarricense de Investigación y Enseñanza en Nutrición y Salud	Cristian Peréz-Corrales, Valeria Peralta-Barquero & Blanco-Delgado C
EPI_ISL_1517391, EPI_ISL_1517393, EPI_ISL_1517394, EPI_ISL_1517395, EPI_ISL_1517396	Inciensa, Instituto Costarricense de Investigación y Enseñanza en Nutrición y Salud	Inciensa, Instituto Costarricense de Investigación y Enseñanza en Nutrición y Salud	Cristian Peréz-Corrales & Valeria Peralta-Barquero
EPI_ISL_1517397	Inciensa, Instituto Costarricense de Investigación y Enseñanza en Nutrición y Salud	Inciensa, Instituto Costarricense de Investigación y Enseñanza en Nutrición y Salud	Cristian Peréz-Corrales, Valeria Peralta-Barquero & Patricia Salas
EPI_ISL_1517398, EPI_ISL_1517399, EPI_ISL_1517400	Inciensa, Instituto Costarricense de Investigación y Enseñanza en Nutrición y Salud	Inciensa, Instituto Costarricense de Investigación y Enseñanza en Nutrición y Salud	Cristian Peréz-Corrales & Valeria Peralta-Barquero
EPI_ISL_1517411	Inciensa, Instituto Costarricense de Investigación y Enseñanza en Nutrición y Salud	Inciensa, Instituto Costarricense de Investigación y Enseñanza en Nutrición y Salud	Cristian Peréz-Corrales, Valeria Peralta-Barquero & Kellerman-Montero K
EPI_ISL_1517412, EPI_ISL_1517413,	Inciensa, Instituto Costarricense de Investigación y	Inciensa, Instituto Costarricense de Investigación y	Cristian Peréz-Corrales, Valeria Peralta-Barquero & Salas-Abarca P

EPI_ISL_1517414	Enseñanza en Nutrición y Salud	Enseñanza en Nutrición y Salud	
EPI_ISL_1517415, EPI_ISL_1517416, EPI_ISL_1517417, EPI_ISL_1517418, EPI_ISL_1517419	Inciensa, Instituto Costarricense de Investigación y Enseñanza en Nutrición y Salud	Inciensa, Instituto Costarricense de Investigación y Enseñanza en Nutrición y Salud	Cristian Peréz-Corrales, Valeria Peralta-Barquero & César Cerdas Quesada
EPI_ISL_1517420, EPI_ISL_1517421, EPI_ISL_1517422, EPI_ISL_1517423, EPI_ISL_1517424	Inciensa, Instituto Costarricense de Investigación y Enseñanza en Nutrición y Salud	Inciensa, Instituto Costarricense de Investigación y Enseñanza en Nutrición y Salud	Cristian Peréz-Corrales, Valeria Peralta-Barquero & Gallegos-Carrillo B
EPI_ISL_1517425, EPI_ISL_1517426	Inciensa, Instituto Costarricense de Investigación y Enseñanza en Nutrición y Salud	Inciensa, Instituto Costarricense de Investigación y Enseñanza en Nutrición y Salud	Cristian Peréz-Corrales, Valeria Peralta-Barquero & Salas-Abarca P
EPI_ISL_1517427, EPI_ISL_1517428	Inciensa, Instituto Costarricense de Investigación y Enseñanza en Nutrición y Salud	Inciensa, Instituto Costarricense de Investigación y Enseñanza en Nutrición y Salud	Cristian Peréz-Corrales, Valeria Peralta-Barquero & Kellerman-Montero K
EPI_ISL_1520107	LACEN do Estado de Rondonia	Instituto Adolfo Lutz, Interdiciplinary Procedures Center, Strategic Laboratory	Claudio Tavares Sacchi, Claudia Regina Gonçalves, Erica Valessa Ramos Gomes, Karoline Rodrigues Campos, Caio Vinicius Dias Lopes
EPI_ISL_1524332, EPI_ISL_1524344	Biology Department, College of Science, Al Muthanna University and Public Health Laboratory, Al-Muthanna Health Directorate	Department of Virology, Faculty of Medicine, University of Helsinki, Helsinki, Finland	Nihad Al-Rashedi, Hussein Alburkat, Murad Munahi, Alaa Hameed, Ali Jasim, Olli Vapalahti, Tarja Sironen,Teemu Smura
EPI_ISL_1526784	Hospital	National Reference Center for Viruses of Respiratory Infections, Institut Pasteur, Paris	Marion Barbet, Sylvie Behillil, Méline Bizard, Angela Brisebarre, Camille Capel,Frédéric Lemoine,Corinne Maufrais,Christophe Malabat, Louise Lefrançois, Etienne Simon-Lorière, Vincent Enouf, Maud Vanpeene, Sylvie van der Werf,LéA Pilorge
EPI_ISL_1527364, EPI_ISL_1527365, EPI_ISL_1527366, EPI_ISL_1527367, EPI_ISL_1527368, EPI_ISL_1527370, EPI_ISL_1527371, EPI_ISL_1527372	Molecular diagnostic laboratory of Federal Budget Institution of Science "Central Research Institute of Epidemiology" of The Federal Service on Customers' Rights Protection and Human Well-being Surveillance	Group of Genomics and Postgenomic Technologies of Central Research Institute of Epidemilology	Samoilov AE, Kaptelova VV, Korneenko EV, Saenko SS, Shipulina OY, Speranskaya AS, Tivanova EV, Kondrasheva LY, Akimkin VG
EPI_ISL_1527390, EPI_ISL_1527391, EPI_ISL_1527392, EPI_ISL_1527394, EPI_ISL_1527395, EPI_ISL_1527396, EPI_ISL_1527397, EPI_ISL_1527398	Laboratory of Extremely Dangerous Infections of Federal Budget Health Care Instute "Center of Hygiene and Epidemiology in Sakhalin region"	Group of Genomics and Postgenomic Technologies of Central Research Institute of Epidemiology	Samoilov AE, Kaptelova VV, Korneenko EV, Valdokhina AV, Bulanenko VP, Saenko SS, Arutiunian AE, Byankina MA, Zagorodnov YL, Golubeva AG, Speranskaya AS, Tivanova EV, Kondrasheva LY, Akimkin VG
EPI_ISL_1528224, EPI_ISL_1528225	Labo Analyses Med	National Reference Center for Viruses of Respiratory Infections, Institut Pasteur, Paris	Marion Barbet, Sylvie Behillil, Frédéric Lemoine, Corinne Maufrais, Christophe Malabat, Méline Bizard, Angela Brisebarre, Camille Capel, Louise Lefrançois, Etienne Simon-Lorière, Vincent Enouf, Maud Vanpeene, Sylvie van der Werf,Sarah Schillinger
EPI_ISL_1528240	Hospital	National Reference Center for Viruses of Respiratory Infections, Institut Pasteur, Paris	Marion Barbet, Sylvie Behillil, Frédéric Lemoine, Corinne Maufrais, Christophe Malabat, Méline Bizard, Angela Brisebarre, Camille Capel, Louise Lefrançois, Etienne Simon-Lorière, Vincent Enouf, Maud Vanpeene, Sylvie van der Werf,Eric Farfour
EPI_ISL_1532291, EPI_ISL_1532292, EPI_ISL_1532293, EPI_ISL_1532294	Oman-National Influenza Center	Biotechnology & OMICs Laboratory	Aisha Al-Amri, Ahmed Al Harrasi, Intisar Al-Shukri, Amal Al-Maani, Sajjad Asaf, Bilal Hussain, Samiya Al-Zadjali, Ahmed N Al-Rawahi, Saqib Bilal, Abdul Latif Khan, Samira Al-Mahruqi, Ahmed Al-Rawahi, Hanan Al-Kindi, Amina Al-Jardani.
EPI_ISL_1532295, EPI_ISL_1532296, EPI_ISL_1532297, EPI_ISL_1532298, EPI_ISL_1532299, EPI_ISL_1532300	Oman-National Influenza Center	Biotechnology & OMICs Laboratory	Abdul Latif Khan, Aisha Al-Amri, Intisar Al-Shukri, Amal Al-Maani, Sajjad Asaf, Bilal Hussain, Samiya Al-Zadjali, Ahmed N Al-Rawahi, Saqib Bilal, Samira Al-Mahruqi, Ahmed Al-Rawahi, Hanan Al-Kindi, Amina Al-Jardani, Ahmed Al Harrasi.
EPI_ISL_1532301	Oman-National Influenza Center	Biotechnology & OMICs Laboratory	Ahmed Al Harrasi, Aisha Al-Amri, Intisar Al-Shukri, Amal Al-Maani, Sajjad Asaf, Bilal Hussain, Samiya Al-Zadjali, Ahmed N Al-Rawahi, Saqib Bilal, Abdul Latif Khan, Samira Al-Mahruqi, Ahmed Al-Rawahi, Hanan Al-Kindi, Amina Al-Jardani.
EPI_ISL_1532304	Oman-National Influenza Center	Biotechnology & OMICs Laboratory	Abdul Latif Khan, Aisha Al-Amri, Intisar Al-Shukri, Amal Al-Maani, Sajjad Asaf, Bilal Hussain, Samiya Al-Zadjali, Ahmed N Al-Rawahi, Saqib Bilal, Samira Al-Mahruqi, Ahmed Al-Rawahi, Hanan Al-Kindi, Amina Al-Jardani, Ahmed Al Harrasi.
EPI_ISL_1532793, EPI_ISL_1532795	Microbiology Department. Complexo Hospitalario Universitario de Vigo	Microbiology Department. Complexo Hospitalario Universitario de Vigo	Alfaya N, Alonso I, Alvarez M, Cabrera JJ, Carballo R, Cores O, Cortizo S, del-Campo V, Martinez L, Mediero G, Perez S, Potel C, Regueiro B, Rey S, Vasallo FJ
EPI_ISL_1533034, EPI_ISL_1533035, EPI_ISL_1533038, EPI_ISL_1533039, EPI_ISL_1533041, EPI_ISL_1533042, EPI_ISL_1533043, EPI_ISL_1533045, EPI_ISL_1533047, EPI_ISL_1533048, EPI_ISL_1533049, EPI_ISL_1533050, EPI_ISL_1533051, EPI_ISL_1533052, EPI_ISL_1533053, EPI_ISL_1533054, EPI_ISL_1533055, EPI_ISL_1533056, EPI_ISL_1533057, EPI_ISL_1533059, EPI_ISL_1533060, EPI_ISL_1533062, EPI_ISL_1533063, EPI_ISL_1533064, EPI_ISL_1533067, EPI_ISL_1533068, EPI_ISL_1533070, EPI_ISL_1533071, EPI_ISL_1533072, EPI_ISL_1533073, EPI_ISL_1533074, EPI_ISL_1533075, EPI_ISL_1533076, EPI_ISL_1533078, EPI_ISL_1533079, EPI_ISL_1533080, EPI_ISL_1533081, EPI_ISL_1533083, EPI_ISL_1533084, EPI_ISL_1533085, EPI_ISL_1533086, EPI_ISL_1533087, EPI_ISL_1533088, EPI_ISL_1533090, EPI_ISL_1533091, EPI_ISL_1533092, EPI_ISL_1533093, EPI_ISL_1533095, EPI_ISL_1533096, EPI_ISL_1533097, EPI_ISL_1533098, EPI_ISL_1533100, EPI_ISL_1533101			
see above	Diagnostic and Research Center of Infectious Diseases, Medical Faculty, Andalas University	Diagnostic and Research Center of Infectious Diseases, Medical Faculty, Andalas University	Andani Eka Putra, Syafrizayanti, Ikhwan R. Sudji, Linosefa, Dede Rahman Agustian, Ayu Novita Trisnawati, Siskalil Fahma, Nia Ayuni Putri, Sekar Asri Tresnaningtyas, SM Rezvi, Fauzul Azhim, Dessy Arisanty, Gestina Aliska, Desmawati, Nita Afriani ,Syandrez Prima Putra, Juane Plantika Menra, Yolani Syaputri, Mutia Lailani
EPI_ISL_1540448	Labo Analyses Med	National Reference Center for Viruses of Respiratory Infections, Institut Pasteur, Paris	Marion Barbet, Sylvie Behillil, Frédéric Lemoine, Corinne Maufrais, Christophe Malabat,Adrien Pain, Méline Bizard, Angela Brisebarre, Camille Capel, Louise Lefrançois, Etienne Simon-Lorière, Vincent Enouf, Maud Vanpeene, Sylvie van der Werf,Sarah Schillinger
EPI_ISL_1544118	Supratech Micropath Laboratory Research Institute Pvt Ltd, Ahmedabad	Gujarat Biotechnology Research Centre	Mahendra Parikh,Zuber Saiyed,Ramesh Pandit,Janvi Raval,Zarna Patel,Nitin Savaliya,Dinesh Kumar,Twinkle Soni,Sonal Sharma ,Umang Mishra,Nitesh Shah,Chaitanya Joshi,Madhvi Joshi
EPI_ISL_1544119	Supratech Micropath Laboratory Research Institute Pvt Ltd, Ahmedabad	Gujarat Biotechnology Research Centre	Mahendra Parikh,Ramesh Pandit,Janvi Raval,Zarna Patel,Nitin Savaliya,Dinesh Kumar,Twinkle Soni,Sonal Sharma ,Zuber Saiyed,Umang Mishra,Nitesh Shah,Chaitanya Joshi,Madhvi Joshi
EPI_ISL_1544120	Supratech Micropath Laboratory Research Institute Pvt Ltd, Ahmedabad	Gujarat Biotechnology Research Centre	Mahendra Parikh,Janvi Raval,Zarna Patel,Nitin Savaliya,Dinesh Kumar,Twinkle Soni,Sonal Sharma ,Zuber Saiyed,Ramesh Pandit,Umang Mishra,Nitesh Shah,Chaitanya Joshi,Madhvi Joshi
EPI_ISL_1544121	Supratech Micropath Laboratory Research Institute Pvt Ltd, Ahmedabad	Gujarat Biotechnology Research Centre	Zarna Patel,Nitin Savaliya,Dinesh Kumar,Twinkle Soni,Sonal Sharma ,Zuber Saiyed,Ramesh Pandit,Janvi Raval,Mahendra Parikh,Umang Mishra,Nitesh Shah,Chaitanya Joshi,Madhvi Joshi
EPI_ISL_1544122	Supratech Micropath Laboratory Research Institute Pvt Ltd, Ahmedabad	Gujarat Biotechnology Research Centre	Nitin Savaliya,Dinesh Kumar,Twinkle Soni,Sonal Sharma ,Zuber Saiyed,Ramesh Pandit,Janvi Raval,Zarna Patel,Mahendra Parikh,Umang Mishra,Nitesh Shah,Chaitanya Joshi,Madhvi Joshi
EPI_ISL_1544123	Supratech Micropath Laboratory Research Institute Pvt Ltd, Ahmedabad	Gujarat Biotechnology Research Centre	Dinesh Kumar,Twinkle Soni,Sonal Sharma ,Zuber Saiyed,Ramesh Pandit,Janvi Raval,Zarna Patel,Nitin Savaliya,Mahendra Parikh,Umang Mishra,Nitesh Shah,Chaitanya Joshi,Madhvi Joshi
EPI_ISL_1544124	Supratech Micropath Laboratory Research Institute Pvt Ltd, Ahmedabad	Gujarat Biotechnology Research Centre	Twinkle Soni,Sonal Sharma ,Zuber Saiyed,Ramesh Pandit,Janvi Raval,Zarna Patel,Nitin Savaliya,Dinesh Kumar,Mahendra Parikh,Umang Mishra,Nitesh Shah,Chaitanya Joshi,Madhvi Joshi
EPI_ISL_1544125	Supratech Micropath Laboratory Research Institute Pvt Ltd, Ahmedabad	Gujarat Biotechnology Research Centre	Sonal Sharma ,Zuber Saiyed,Ramesh Pandit,Janvi Raval,Zarna Patel,Nitin Savaliya,Dinesh Kumar,Twinkle Soni,Mahendra Parikh,Umang Mishra,Nitesh Shah,Chaitanya Joshi,Madhvi Joshi
EPI_ISL_1544126	Supratech Micropath Laboratory Research Institute Pvt Ltd, Ahmedabad	Gujarat Biotechnology Research Centre	Zuber Saiyed,Ramesh Pandit,Janvi Raval,Zarna Patel,Nitin Savaliya,Dinesh Kumar,Twinkle Soni,Sonal Sharma ,Mahendra Parikh,Umang Mishra,Nitesh Shah,Chaitanya Joshi,Madhvi Joshi
EPI_ISL_1544127	Supratech Micropath Laboratory Research Institute Pvt Ltd, Ahmedabad	Gujarat Biotechnology Research Centre	Ramesh Pandit,Janvi Raval,Zarna Patel,Nitin Savaliya,Dinesh Kumar,Twinkle Soni,Sonal Sharma ,Zuber Saiyed,Mahendra Parikh,Umang Mishra,Nitesh Shah,Chaitanya Joshi,Madhvi Joshi
EPI_ISL_1544128	Supratech Micropath Laboratory Research Institute Pvt Ltd, Ahmedabad	Gujarat Biotechnology Research Centre	Janvi Raval,Zarna Patel,Nitin Savaliya,Dinesh Kumar,Twinkle Soni,Sonal Sharma ,Zuber Saiyed,Ramesh Pandit,Mahendra Parikh,Umang Mishra,Nitesh Shah,Chaitanya Joshi,Madhvi Joshi

EPI_ISL_1544129	Supratech Micropath Laboratory Research Institute Pvt Ltd, Ahmedabad	Gujarat Biotechnology Research Centre	Zarna Patel,Nitin Savaliya,Dinesh Kumar,Twinkle Soni,Sonal Sharma ,Zuber Saiyed,Ramesh Pandit,Janvi Raval,Mahendra Parikh,Umang Mishra,Nitesh Shah,Chaitanya Joshi,Madhvi Joshi
EPI_ISL_1544130	Supratech Micropath Laboratory Research Institute Pvt Ltd, Ahmedabad	Gujarat Biotechnology Research Centre	Nitin Savaliya,Dinesh Kumar,Twinkle Soni,Sonal Sharma ,Zuber Saiyed,Ramesh Pandit,Janvi Raval,Zarna Patel,Mahendra Parikh,Umang Mishra,Nitesh Shah,Chaitanya Joshi,Madhvi Joshi
EPI_ISL_1544131	Supratech Micropath Laboratory Research Institute Pvt Ltd, Ahmedabad	Gujarat Biotechnology Research Centre	Dinesh Kumar,Twinkle Soni,Sonal Sharma ,Zuber Saiyed,Ramesh Pandit,Janvi Raval,Zarna Patel,Nitin Savaliya,Mahendra Parikh,Umang Mishra,Nitesh Shah,Chaitanya Joshi,Madhvi Joshi
EPI_ISL_1544132	Supratech Micropath Laboratory Research Institute Pvt Ltd, Ahmedabad	Gujarat Biotechnology Research Centre	Twinkle Soni,Sonal Sharma ,Zuber Saiyed,Ramesh Pandit,Janvi Raval,Zarna Patel,Nitin Savaliya,Dinesh Kumar,Mahendra Parikh,Umang Mishra,Nitesh Shah,Chaitanya Joshi,Madhvi Joshi
EPI_ISL_1544135	Supratech Micropath Laboratory Research Institute Pvt Ltd, Ahmedabad	Gujarat Biotechnology Research Centre	Ramesh Pandit,Janvi Raval,Zarna Patel,Nitin Savaliya,Dinesh Kumar,Twinkle Soni,Sonal Sharma ,Zuber Saiyed,Mahendra Parikh,Umang Mishra,Nitesh Shah,Chaitanya Joshi,Madhvi Joshi
EPI_ISL_1544136	Supratech Micropath Laboratory Research Institute Pvt Ltd, Ahmedabad	Gujarat Biotechnology Research Centre	Janvi Raval,Zarna Patel,Nitin Savaliya,Dinesh Kumar,Twinkle Soni,Sonal Sharma ,Zuber Saiyed,Ramesh Pandit,Mahendra Parikh,Umang Mishra,Nitesh Shah,Chaitanya Joshi,Madhvi Joshi
EPI_ISL_1544137	Supratech Micropath Laboratory Research Institute Pvt Ltd, Ahmedabad	Gujarat Biotechnology Research Centre	Zarna Patel,Nitin Savaliya,Dinesh Kumar,Twinkle Soni,Sonal Sharma ,Zuber Saiyed,Ramesh Pandit,Janvi Raval,Mahendra Parikh,Umang Mishra,Nitesh Shah,Chaitanya Joshi,Madhvi Joshi
EPI_ISL_1544138	Supratech Micropath Laboratory Research Institute Pvt Ltd, Ahmedabad	Gujarat Biotechnology Research Centre	Nitin Savaliya,Dinesh Kumar,Twinkle Soni,Sonal Sharma ,Zuber Saiyed,Ramesh Pandit,Janvi Raval,Zarna Patel,Mahendra Parikh,Umang Mishra,Nitesh Shah,Chaitanya Joshi,Madhvi Joshi
EPI_ISL_1544139	Supratech Micropath Laboratory Research Institute Pvt Ltd, Ahmedabad	Gujarat Biotechnology Research Centre	Dinesh Kumar,Twinkle Soni,Sonal Sharma ,Zuber Saiyed,Ramesh Pandit,Janvi Raval,Zarna Patel,Nitin Savaliya,Mahendra Parikh,Umang Mishra,Nitesh Shah,Chaitanya Joshi,Madhvi Joshi
EPI_ISL_1544140	Supratech Micropath Laboratory Research Institute Pvt Ltd, Ahmedabad	Gujarat Biotechnology Research Centre	Twinkle Soni,Sonal Sharma ,Zuber Saiyed,Ramesh Pandit,Janvi Raval,Zarna Patel,Nitin Savaliya,Dinesh Kumar,Mahendra Parikh,Umang Mishra,Nitesh Shah,Chaitanya Joshi,Madhvi Joshi
EPI_ISL_1544141	Supratech Micropath Laboratory Research Institute Pvt Ltd, Ahmedabad	Gujarat Biotechnology Research Centre	Sonal Sharma ,Zuber Saiyed,Ramesh Pandit,Janvi Raval,Zarna Patel,Nitin Savaliya,Dinesh Kumar,Twinkle Soni,Mahendra Parikh,Umang Mishra,Nitesh Shah,Chaitanya Joshi,Madhvi Joshi
EPI_ISL_1544142	SSG Medical College, Baroda	Gujarat Biotechnology Research Centre	Tanuja B. Javadekar,Zuber Saiyed,Ramesh Pandit,Janvi Raval,Zarna Patel,Nitin Savaliya,Dinesh Kumar,Twinkle Soni,Sonal Sharma ,Umang Mishra,Nitesh Shah,Chaitanya Joshi,Madhvi Joshi
EPI_ISL_1544143	SSG Medical College, Baroda	Gujarat Biotechnology Research Centre	Tanuja B. Javadekar,Ramesh Pandit,Janvi Raval,Zarna Patel,Nitin Savaliya,Dinesh Kumar,Twinkle Soni,Sonal Sharma ,Zuber Saiyed,Umang Mishra,Nitesh Shah,Chaitanya Joshi,Madhvi Joshi
EPI_ISL_1544144	SSG Medical College, Baroda	Gujarat Biotechnology Research Centre	Tanuja B. Javadekar,Janvi Raval,Zarna Patel,Nitin Savaliya,Dinesh Kumar,Twinkle Soni,Sonal Sharma ,Zuber Saiyed,Ramesh Pandit,Umang Mishra,Nitesh Shah,Chaitanya Joshi,Madhvi Joshi
EPI_ISL_1544145	SSG Medical College, Baroda	Gujarat Biotechnology Research Centre	Zarna Patel,Nitin Savaliya,Dinesh Kumar,Twinkle Soni,Sonal Sharma ,Zuber Saiyed,Ramesh Pandit,Janvi Raval,Tanuja B. Javadekar,Umang Mishra,Nitesh Shah,Chaitanya Joshi,Madhvi Joshi
EPI_ISL_1544146	SSG Medical College, Baroda	Gujarat Biotechnology Research Centre	Nitin Savaliya,Dinesh Kumar,Twinkle Soni,Sonal Sharma ,Zuber Saiyed,Ramesh Pandit,Janvi Raval,Zarna Patel,Tanuja B. Javadekar,Umang Mishra,Nitesh Shah,Chaitanya Joshi,Madhvi Joshi
EPI_ISL_1544147	SSG Medical College, Baroda	Gujarat Biotechnology Research Centre	Dinesh Kumar,Twinkle Soni,Sonal Sharma ,Zuber Saiyed,Ramesh Pandit,Janvi Raval,Zarna Patel,Nitin Savaliya,Tanuja B. Javadekar,Umang Mishra,Nitesh Shah,Chaitanya Joshi,Madhvi Joshi
EPI_ISL_1544148	SSG Medical College, Baroda	Gujarat Biotechnology Research Centre	Twinkle Soni,Sonal Sharma ,Zuber Saiyed,Ramesh Pandit,Janvi Raval,Zarna Patel,Nitin Savaliya,Dinesh Kumar,Tanuja B. Javadekar,Umang Mishra,Nitesh Shah,Chaitanya Joshi,Madhvi Joshi
EPI_ISL_1544149	SSG Medical College, Baroda	Gujarat Biotechnology Research Centre	Sonal Sharma ,Zuber Saiyed,Ramesh Pandit,Janvi Raval,Zarna Patel,Nitin Savaliya,Dinesh Kumar,Twinkle Soni,Tanuja B. Javadekar,Umang Mishra,Nitesh Shah,Chaitanya Joshi,Madhvi Joshi
EPI_ISL_1544150	SSG Medical College, Baroda	Gujarat Biotechnology Research Centre	Zuber Saiyed,Ramesh Pandit,Janvi Raval,Zarna Patel,Nitin Savaliya,Dinesh Kumar,Twinkle Soni,Sonal Sharma ,Tanuja B. Javadekar,Umang Mishra,Nitesh Shah,Chaitanya Joshi,Madhvi Joshi
EPI_ISL_1544151	SSG Medical College, Baroda	Gujarat Biotechnology Research Centre	Ramesh Pandit,Janvi Raval,Zarna Patel,Nitin Savaliya,Dinesh Kumar,Twinkle Soni,Sonal Sharma ,Zuber Saiyed,Tanuja B. Javadekar,Umang Mishra,Nitesh Shah,Chaitanya Joshi,Madhvi Joshi
EPI_ISL_1544152	SSG Medical College, Baroda	Gujarat Biotechnology Research Centre	Janvi Raval,Zarna Patel,Nitin Savaliya,Dinesh Kumar,Twinkle Soni,Sonal Sharma ,Zuber Saiyed,Ramesh Pandit,Tanuja B. Javadekar,Umang Mishra,Nitesh Shah,Chaitanya Joshi,Madhvi Joshi
EPI_ISL_1544153	SSG Medical College, Baroda	Gujarat Biotechnology Research Centre	Zarna Patel,Nitin Savaliya,Dinesh Kumar,Twinkle Soni,Sonal Sharma ,Zuber Saiyed,Ramesh Pandit,Janvi Raval,Tanuja B. Javadekar,Umang Mishra,Nitesh Shah,Chaitanya Joshi,Madhvi Joshi
EPI_ISL_1544154	SSG Medical College, Baroda	Gujarat Biotechnology Research Centre	Nitin Savaliya,Dinesh Kumar,Twinkle Soni,Sonal Sharma ,Zuber Saiyed,Ramesh Pandit,Janvi Raval,Zarna Patel,Tanuja B. Javadekar,Umang Mishra,Nitesh Shah,Chaitanya Joshi,Madhvi Joshi
EPI_ISL_1544155	SSG Medical College, Baroda	Gujarat Biotechnology Research Centre	Dinesh Kumar,Twinkle Soni,Sonal Sharma ,Zuber Saiyed,Ramesh Pandit,Janvi Raval,Zarna Patel,Nitin Savaliya,Tanuja B. Javadekar,Umang Mishra,Nitesh Shah,Chaitanya Joshi,Madhvi Joshi
EPI_ISL_1544156	SSG Medical College, Baroda	Gujarat Biotechnology Research Centre	Twinkle Soni,Sonal Sharma ,Zuber Saiyed,Ramesh Pandit,Janvi Raval,Zarna Patel,Nitin Savaliya,Dinesh Kumar,Tanuja B. Javadekar,Umang Mishra,Nitesh Shah,Chaitanya Joshi,Madhvi Joshi
EPI_ISL_1544157	SSG Medical College, Baroda	Gujarat Biotechnology Research Centre	Sonal Sharma ,Zuber Saiyed,Ramesh Pandit,Janvi Raval,Zarna Patel,Nitin Savaliya,Dinesh Kumar,Twinkle Soni,Tanuja B. Javadekar,Umang Mishra,Nitesh Shah,Chaitanya Joshi,Madhvi Joshi
EPI_ISL_1544158	SSG Medical College, Baroda	Gujarat Biotechnology Research Centre	Zuber Saiyed,Ramesh Pandit,Janvi Raval,Zarna Patel,Nitin Savaliya,Dinesh Kumar,Twinkle Soni,Sonal Sharma ,Tanuja B. Javadekar,Umang Mishra,Nitesh Shah,Chaitanya Joshi,Madhvi Joshi
EPI_ISL_1544159	SSG Medical College, Baroda	Gujarat Biotechnology Research Centre	Ramesh Pandit,Janvi Raval,Zarna Patel,Nitin Savaliya,Dinesh Kumar,Twinkle Soni,Sonal Sharma ,Zuber Saiyed,Tanuja B. Javadekar,Umang Mishra,Nitesh Shah,Chaitanya Joshi,Madhvi Joshi
EPI_ISL_1544160	SSG Medical College, Baroda	Gujarat Biotechnology Research Centre	Janvi Raval,Zarna Patel,Nitin Savaliya,Dinesh Kumar,Twinkle Soni,Sonal Sharma ,Zuber Saiyed,Ramesh Pandit,Tanuja B. Javadekar,Umang Mishra,Nitesh Shah,Chaitanya Joshi,Madhvi Joshi
EPI_ISL_1544161	SSG Medical College, Baroda	Gujarat Biotechnology Research Centre	Zarna Patel,Nitin Savaliya,Dinesh Kumar,Twinkle Soni,Sonal Sharma ,Zuber Saiyed,Ramesh Pandit,Janvi Raval,Tanuja B. Javadekar,Umang Mishra,Nitesh Shah,Chaitanya Joshi,Madhvi Joshi
EPI_ISL_1544162	SSG Medical College, Baroda	Gujarat Biotechnology Research Centre	Nitin Savaliya,Dinesh Kumar,Twinkle Soni,Sonal Sharma ,Zuber Saiyed,Ramesh Pandit,Janvi Raval,Zarna Patel,Tanuja B. Javadekar,Umang Mishra,Nitesh Shah,Chaitanya Joshi,Madhvi Joshi
EPI_ISL_1544163	SSG Medical College, Baroda	Gujarat Biotechnology Research Centre	Dinesh Kumar,Twinkle Soni,Sonal Sharma ,Zuber Saiyed,Ramesh Pandit,Janvi Raval,Zarna Patel,Nitin Savaliya,Tanuja B. Javadekar,Umang Mishra,Nitesh Shah,Chaitanya Joshi,Madhvi Joshi
EPI_ISL_1544164	SSG Medical College, Baroda	Gujarat Biotechnology Research Centre	Twinkle Soni,Sonal Sharma ,Zuber Saiyed,Ramesh Pandit,Janvi Raval,Zarna Patel,Nitin Savaliya,Dinesh Kumar,Tanuja B. Javadekar,Umang Mishra,Nitesh Shah,Chaitanya Joshi,Madhvi Joshi
EPI_ISL_1544165	SSG Medical College, Baroda	Gujarat Biotechnology Research Centre	Sonal Sharma ,Zuber Saiyed,Ramesh Pandit,Janvi Raval,Zarna Patel,Nitin Savaliya,Dinesh Kumar,Twinkle Soni,Tanuja B. Javadekar,Umang Mishra,Nitesh Shah,Chaitanya Joshi,Madhvi Joshi

EPI_ISL_1544166	Supratech Micropath Laboratory Research Institute Pvt Ltd, Ahmedabad	Gujarat Biotechnology Research Centre	Zuber Saiyed,Ramesh Pandit,Janvi Raval,Zarna Patel,Nitin Savaliya,Dinesh Kumar,Twinkle Soni,Sonal Sharma ,Mahendra Parikh,Umang Mishra,Nitesh Shah,Chaitanya Joshi,Madhvi Joshi
EPI_ISL_1544167	Supratech Micropath Laboratory Research Institute Pvt Ltd, Ahmedabad	Gujarat Biotechnology Research Centre	Ramesh Pandit,Janvi Raval,Zarna Patel,Nitin Savaliya,Dinesh Kumar,Twinkle Soni,Sonal Sharma ,Zuber Saiyed,Mahendra Parikh,Umang Mishra,Nitesh Shah,Chaitanya Joshi,Madhvi Joshi
EPI_ISL_1544168	Supratech Micropath Laboratory Research Institute Pvt Ltd, Ahmedabad	Gujarat Biotechnology Research Centre	Janvi Raval,Zarna Patel,Nitin Savaliya,Dinesh Kumar,Twinkle Soni,Sonal Sharma ,Zuber Saiyed,Ramesh Pandit,Mahendra Parikh,Umang Mishra,Nitesh Shah,Chaitanya Joshi,Madhvi Joshi
EPI_ISL_1544169	Supratech Micropath Laboratory Research Institute Pvt Ltd, Ahmedabad	Gujarat Biotechnology Research Centre	Zarna Patel,Nitin Savaliya,Dinesh Kumar,Twinkle Soni,Sonal Sharma ,Zuber Saiyed,Ramesh Pandit,Janvi Raval,Mahendra Parikh,Umang Mishra,Nitesh Shah,Chaitanya Joshi,Madhvi Joshi
EPI_ISL_1544170	SSG Medical College, Baroda	Gujarat Biotechnology Research Centre	Nitin Savaliya,Dinesh Kumar,Twinkle Soni,Sonal Sharma ,Zuber Saiyed,Ramesh Pandit,Janvi Raval,Zarna Patel,Tanuja B. Javadekar,Umang Mishra,Nitesh Shah,Chaitanya Joshi,Madhvi Joshi
EPI_ISL_1544171	SSG Medical College, Baroda	Gujarat Biotechnology Research Centre	Dinesh Kumar,Twinkle Soni,Sonal Sharma ,Zuber Saiyed,Ramesh Pandit,Janvi Raval,Zarna Patel,Nitin Savaliya,Tanuja B. Javadekar,Umang Mishra,Nitesh Shah,Chaitanya Joshi,Madhvi Joshi
EPI_ISL_1544172	SSG Medical College, Baroda	Gujarat Biotechnology Research Centre	Twinkle Soni,Sonal Sharma ,Zuber Saiyed,Ramesh Pandit,Janvi Raval,Zarna Patel,Nitin Savaliya,Dinesh Kumar,Tanuja B. Javadekar,Umang Mishra,Nitesh Shah,Chaitanya Joshi,Madhvi Joshi
EPI_ISL_1544173	SSG Medical College, Baroda	Gujarat Biotechnology Research Centre	Sonal Sharma ,Zuber Saiyed,Ramesh Pandit,Janvi Raval,Zarna Patel,Nitin Savaliya,Dinesh Kumar,Twinkle Soni,Tanuja B. Javadekar,Umang Mishra,Nitesh Shah,Chaitanya Joshi,Madhvi Joshi
EPI_ISL_1544174	SSG Medical College, Baroda	Gujarat Biotechnology Research Centre	Zuber Saiyed,Ramesh Pandit,Janvi Raval,Zarna Patel,Nitin Savaliya,Dinesh Kumar,Twinkle Soni,Sonal Sharma ,Tanuja B. Javadekar,Umang Mishra,Nitesh Shah,Chaitanya Joshi,Madhvi Joshi
EPI_ISL_1544175	SSG Medical College, Baroda	Gujarat Biotechnology Research Centre	Ramesh Pandit,Janvi Raval,Zarna Patel,Nitin Savaliya,Dinesh Kumar,Twinkle Soni,Sonal Sharma ,Zuber Saiyed,Tanuja B. Javadekar,Umang Mishra,Nitesh Shah,Chaitanya Joshi,Madhvi Joshi
EPI_ISL_1544176	SSG Medical College, Baroda	Gujarat Biotechnology Research Centre	Janvi Raval,Zarna Patel,Nitin Savaliya,Dinesh Kumar,Twinkle Soni,Sonal Sharma ,Zuber Saiyed,Ramesh Pandit,Tanuja B. Javadekar,Umang Mishra,Nitesh Shah,Chaitanya Joshi,Madhvi Joshi
EPI_ISL_1544177	SSG Medical College, Baroda	Gujarat Biotechnology Research Centre	Zarna Patel,Nitin Savaliya,Dinesh Kumar,Twinkle Soni,Sonal Sharma ,Zuber Saiyed,Ramesh Pandit,Janvi Raval,Tanuja B. Javadekar,Umang Mishra,Nitesh Shah,Chaitanya Joshi,Madhvi Joshi
EPI_ISL_1544178	SSG Medical College, Baroda	Gujarat Biotechnology Research Centre	Nitin Savaliya,Dinesh Kumar,Twinkle Soni,Sonal Sharma ,Zuber Saiyed,Ramesh Pandit,Janvi Raval,Zarna Patel,Tanuja B. Javadekar,Umang Mishra,Nitesh Shah,Chaitanya Joshi,Madhvi Joshi
EPI_ISL_1544179	Government Medical College (GMC), Surat	Gujarat Biotechnology Research Centre	Neeta Khandelwal,Dinesh Kumar,Twinkle Soni,Sonal Sharma ,Zuber Saiyed,Ramesh Pandit,Janvi Raval,Zarna Patel,Nitin Savaliya,Umang Mishra,Nitesh Shah,Chaitanya Joshi,Madhvi Joshi
EPI_ISL_1544180	Government Medical College (GMC), Surat	Gujarat Biotechnology Research Centre	Neeta Khandelwal,Twinkle Soni,Sonal Sharma ,Zuber Saiyed,Ramesh Pandit,Janvi Raval,Zarna Patel,Nitin Savaliya,Dinesh Kumar,Umang Mishra,Nitesh Shah,Chaitanya Joshi,Madhvi Joshi
EPI_ISL_1544181	Government Medical College (GMC), Surat	Gujarat Biotechnology Research Centre	Neeta Khandelwal,Sonal Sharma ,Zuber Saiyed,Ramesh Pandit,Janvi Raval,Zarna Patel,Nitin Savaliya,Dinesh Kumar,Twinkle Soni,Umang Mishra,Nitesh Shah,Chaitanya Joshi,Madhvi Joshi
EPI_ISL_1544182	Government Medical College (GMC), Surat	Gujarat Biotechnology Research Centre	Zuber Saiyed,Ramesh Pandit,Janvi Raval,Zarna Patel,Nitin Savaliya,Dinesh Kumar,Twinkle Soni,Sonal Sharma ,Neeta Khandelwal,Umang Mishra,Nitesh Shah,Chaitanya Joshi,Madhvi Joshi
EPI_ISL_1544183	Government Medical College (GMC), Surat	Gujarat Biotechnology Research Centre	Ramesh Pandit,Janvi Raval,Zarna Patel,Nitin Savaliya,Dinesh Kumar,Twinkle Soni,Sonal Sharma ,Zuber Saiyed,Neeta Khandelwal,Umang Mishra,Nitesh Shah,Chaitanya Joshi,Madhvi Joshi
EPI_ISL_1544184	Government Medical College (GMC), Surat	Gujarat Biotechnology Research Centre	Janvi Raval,Zarna Patel,Nitin Savaliya,Dinesh Kumar,Twinkle Soni,Sonal Sharma ,Zuber Saiyed,Ramesh Pandit,Neeta Khandelwal,Umang Mishra,Nitesh Shah,Chaitanya Joshi,Madhvi Joshi
EPI_ISL_1544185	Government Medical College (GMC), Surat	Gujarat Biotechnology Research Centre	Zarna Patel,Nitin Savaliya,Dinesh Kumar,Twinkle Soni,Sonal Sharma ,Zuber Saiyed,Ramesh Pandit,Janvi Raval,Neeta Khandelwal,Umang Mishra,Nitesh Shah,Chaitanya Joshi,Madhvi Joshi
EPI_ISL_1544186	Government Medical College (GMC), Surat	Gujarat Biotechnology Research Centre	Nitin Savaliya,Dinesh Kumar,Twinkle Soni,Sonal Sharma ,Zuber Saiyed,Ramesh Pandit,Janvi Raval,Zarna Patel,Neeta Khandelwal,Umang Mishra,Nitesh Shah,Chaitanya Joshi,Madhvi Joshi
EPI_ISL_1544187	Government Medical College (GMC), Surat	Gujarat Biotechnology Research Centre	Dinesh Kumar,Twinkle Soni,Sonal Sharma ,Zuber Saiyed,Ramesh Pandit,Janvi Raval,Zarna Patel,Nitin Savaliya,Neeta Khandelwal,Umang Mishra,Nitesh Shah,Chaitanya Joshi,Madhvi Joshi
EPI_ISL_1544188	Government Medical College (GMC), Surat	Gujarat Biotechnology Research Centre	Twinkle Soni,Sonal Sharma ,Zuber Saiyed,Ramesh Pandit,Janvi Raval,Zarna Patel,Nitin Savaliya,Dinesh Kumar,Neeta Khandelwal,Umang Mishra,Nitesh Shah,Chaitanya Joshi,Madhvi Joshi
EPI_ISL_1544189	Government Medical College (GMC), Surat	Gujarat Biotechnology Research Centre	Sonal Sharma ,Zuber Saiyed,Ramesh Pandit,Janvi Raval,Zarna Patel,Nitin Savaliya,Dinesh Kumar,Twinkle Soni,Neeta Khandelwal,Umang Mishra,Nitesh Shah,Chaitanya Joshi,Madhvi Joshi
EPI_ISL_1544190	Government Medical College (GMC), Surat	Gujarat Biotechnology Research Centre	Zuber Saiyed,Ramesh Pandit,Janvi Raval,Zarna Patel,Nitin Savaliya,Dinesh Kumar,Twinkle Soni,Sonal Sharma ,Neeta Khandelwal,Umang Mishra,Nitesh Shah,Chaitanya Joshi,Madhvi Joshi
EPI_ISL_1544191	Government Medical College (GMC), Surat	Gujarat Biotechnology Research Centre	Ramesh Pandit,Janvi Raval,Zarna Patel,Nitin Savaliya,Dinesh Kumar,Twinkle Soni,Sonal Sharma ,Zuber Saiyed,Neeta Khandelwal,Umang Mishra,Nitesh Shah,Chaitanya Joshi,Madhvi Joshi
EPI_ISL_1544192	Government Medical College (GMC), Surat	Gujarat Biotechnology Research Centre	Janvi Raval,Zarna Patel,Nitin Savaliya,Dinesh Kumar,Twinkle Soni,Sonal Sharma ,Zuber Saiyed,Ramesh Pandit,Neeta Khandelwal,Umang Mishra,Nitesh Shah,Chaitanya Joshi,Madhvi Joshi
EPI_ISL_1544193	Government Medical College (GMC), Surat	Gujarat Biotechnology Research Centre	Zarna Patel,Nitin Savaliya,Dinesh Kumar,Twinkle Soni,Sonal Sharma ,Zuber Saiyed,Ramesh Pandit,Janvi Raval,Neeta Khandelwal,Umang Mishra,Nitesh Shah,Chaitanya Joshi,Madhvi Joshi
EPI_ISL_1544194	Government Medical College (GMC), Surat	Gujarat Biotechnology Research Centre	Nitin Savaliya,Dinesh Kumar,Twinkle Soni,Sonal Sharma ,Zuber Saiyed,Ramesh Pandit,Janvi Raval,Zarna Patel,Neeta Khandelwal,Umang Mishra,Nitesh Shah,Chaitanya Joshi,Madhvi Joshi
EPI_ISL_1544195	Government Medical College (GMC), Surat	Gujarat Biotechnology Research Centre	Dinesh Kumar,Twinkle Soni,Sonal Sharma ,Zuber Saiyed,Ramesh Pandit,Janvi Raval,Zarna Patel,Nitin Savaliya,Neeta Khandelwal,Umang Mishra,Nitesh Shah,Chaitanya Joshi,Madhvi Joshi
EPI_ISL_1548045, EPI_ISL_1548046, EPI_ISL_1548047, EPI_ISL_1548048	University of Rome Tor Vergata: Departm Experim Medicine Chair of Virology	University of Rome Tor Vergata: Departm Experim Medicine Chair of Virology	Maria Concetta Bellocchi, Luca Carioti, Maria Botticelli, Lorenzo Piermatteo, Francesca Ceccherini-Silberstein
EPI_ISL_1575297, EPI_ISL_1575436	Laboratoire Biolim/FSS/UL	Unité Mixte Internationale TransVIHMI (UMI 233 IRD - U1175 INSERM - Université de Montpellier) IRD (Institut de recherche pour le développement)	Mounerou SALOU, Christelle BUTEL, Wembo A. HALATOKO, Amivi EHLAN, Abla A. KONOU, Issaka Maman, Syntyche DEVATCHAGNI, Adodo SADJI, Kokou TEGUENI,Koku AGBODEKA, Sidonie A.M.KAGNISSODE, Akoélé SILIADIN, Alassane OURO-MEDELI, Messanh DOUFFAN,Déléma MABA,Sika DOSSIM, Améyo DORKENOO, Mireille PRINCE-DAVID,Anoumou DAGNRA, Laetitia SERRANO,Ahidjo AYOUBA, Eric DELAPORTE, Martine PEETERS
EPI_ISL_1576835, EPI_ISL_1577026, EPI_ISL_1577182, EPI_ISL_1577390, EPI_ISL_1582589, EPI_ISL_1582590	Universidad Industrial de Santander (Laboratorio Central de Investigaciones - Clínica Chicamocha).	Universidad Industrial de Santander.	Francisco Martinez-Perez, Cristian E. Cadena-Caballero, Diego Rueda-Plata, Carolina S. Torres-Jiménez, Lizeth J. Forero-Buitrago, Erika Lizarazo-Gutiérrez, Carlos Barrios-Hernández, Lina M. Vera-Cala.
EPI_ISL_1582639, EPI_ISL_1582640, EPI_ISL_1582641	Research	National Reference Center for Viruses of Respiratory Infections, Institut Pasteur, Paris	Marion Barbet, Sylvie Behillil, Frédéric Lemoine, Corinne Maufrais, Christophe Malabat,Gael Millot, Méline Bizard, Angela Brisebarre, Camille Capel, Louise Lefrançois, Etienne Simon-Lorière, Vincent Enouf, Maud Vanpeene, Sylvie van der Werf,Marianne Leruez-Ville
EPI_ISL_1582643, EPI_ISL_1582644,	Research	National Reference Center for Viruses of Respiratory	Marion Barbet, Sylvie Behillil, Frédéric Lemoine, Corinne Maufrais, Christophe Malabat, Méline Bizard, Angela Brisebarre, Camille Capel, Louise Lefrançois,

EPI_ISL_1582645, EPI_ISL_1582646		Infections, Institut Pasteur, Paris	Etienne Simon-Lorière, Vincent Enouf, Maud Vanpeene, Sylvie van der Werf,Marianne Leruez-Ville
EPI_ISL_1582647	Research	National Reference Center for Viruses of Respiratory Infections, Institut Pasteur, Paris	Marion Barbet, Sylvie Behillil, Frédéric Lemoine, Corinne Maufrais, Christophe Malabat,Gael Millot, Méline Bizard, Angela Brisebarre, Camille Capel, Louise Lefrançois, Etienne Simon-Lorière, Vincent Enouf, Maud Vanpeene, Sylvie van der Werf,Marianne Leruez-Ville
EPI_ISL_1582648, EPI_ISL_1582649	Research	National Reference Center for Viruses of Respiratory Infections, Institut Pasteur, Paris	Marion Barbet, Sylvie Behillil, Frédéric Lemoine, Corinne Maufrais, Christophe Malabat, Méline Bizard, Angela Brisebarre, Camille Capel, Louise Lefrançois, Etienne Simon-Lorière, Vincent Enouf, Maud Vanpeene, Sylvie van der Werf,Marianne Leruez-Ville
EPI_ISL_1582650	Research	National Reference Center for Viruses of Respiratory Infections, Institut Pasteur, Paris	Marion Barbet, Sylvie Behillil, Frédéric Lemoine, Corinne Maufrais, Christophe Malabat,Gael Millot, Méline Bizard, Angela Brisebarre, Camille Capel, Louise Lefrançois, Etienne Simon-Lorière, Vincent Enouf, Maud Vanpeene, Sylvie van der Werf,Marianne Leruez-Ville
EPI_ISL_1582651, EPI_ISL_1582652	Research	National Reference Center for Viruses of Respiratory Infections, Institut Pasteur, Paris	Marion Barbet, Sylvie Behillil, Frédéric Lemoine, Corinne Maufrais, Christophe Malabat, Méline Bizard, Angela Brisebarre, Camille Capel, Louise Lefrançois, Etienne Simon-Lorière, Vincent Enouf, Maud Vanpeene, Sylvie van der Werf,Marianne Leruez-Ville
EPI_ISL_1582653	Research	National Reference Center for Viruses of Respiratory Infections, Institut Pasteur, Paris	Marion Barbet, Sylvie Behillil, Frédéric Lemoine, Corinne Maufrais, Christophe Malabat,Pierre Lechat, Méline Bizard, Angela Brisebarre, Camille Capel, Louise Lefrançois, Etienne Simon-Lorière, Vincent Enouf, Maud Vanpeene, Sylvie van der Werf,Marianne Leruez-Ville
EPI_ISL_1582654, EPI_ISL_1582655, EPI_ISL_1582656	Research	National Reference Center for Viruses of Respiratory Infections, Institut Pasteur, Paris	Marion Barbet, Sylvie Behillil, Frédéric Lemoine, Corinne Maufrais, Christophe Malabat, Méline Bizard, Angela Brisebarre, Camille Capel, Louise Lefrançois, Etienne Simon-Lorière, Vincent Enouf, Maud Vanpeene, Sylvie van der Werf,Marianne Leruez-Ville
EPI_ISL_1582756	Hospital	National Reference Center for Viruses of Respiratory Infections, Institut Pasteur, Paris	Marion Barbet, Sylvie Behillil, Frédéric Lemoine, Corinne Maufrais, Christophe Malabat,Pierre Lechat, Méline Bizard, Angela Brisebarre, Camille Capel, Louise Lefrançois, Etienne Simon-Lorière, Vincent Enouf, Maud Vanpeene, Sylvie van der Werf,Franck Griscelli
EPI_ISL_1583053	Labo Analyses Med	National Reference Center for Viruses of Respiratory Infections, Institut Pasteur, Paris	Marion Barbet, Sylvie Behillil, Frédéric Lemoine, Corinne Maufrais, Christophe Malabat, Méline Bizard, Angela Brisebarre, Camille Capel, Louise Lefrançois, Etienne Simon-Lorière, Vincent Enouf, Maud Vanpeene, Sylvie van der Werf,GréGoire Potiron
EPI_ISL_1583074, EPI_ISL_1583078	Labo analyses Med	National Reference Center for Viruses of Respiratory Infections, Institut Pasteur, Paris	Marion Barbet, Sylvie Behillil, Frédéric Lemoine, Corinne Maufrais, Christophe Malabat, Méline Bizard, Angela Brisebarre, Camille Capel, Louise Lefrançois, Etienne Simon-Lorière, Vincent Enouf, Maud Vanpeene, Sylvie van der Werf,Anne Holstein
EPI_ISL_1589116, EPI_ISL_1589117, EPI_ISL_1589118, EPI_ISL_1589119, EPI_ISL_1589127, EPI_ISL_1589128, EPI_ISL_1589129, EPI_ISL_1589130, EPI_ISL_1589131, EPI_ISL_1589132, EPI_ISL_1589133, EPI_ISL_1589134, EPI_ISL_1589135, EPI_ISL_1589136, EPI_ISL_1589137, EPI_ISL_1589138			
see above	Department of Medical, Surgical, and Health Sciences, University of Trieste and Institute for Maternal and Child Health "IRCCS Burlo Garofolo"	Department of Biomedical, Surgical and Dental Sciences; University of Milan	Ferrante Pasquale, Delbue Serena, Signorini Lucia, Dolci Maria, Comar Manola, Campisciano Giuseppina, Cason Carolina, Ticozzi Rosalia
EPI_ISL_1591101, EPI_ISL_1591102, EPI_ISL_1591103, EPI_ISL_1591104	Queen Astrid Military Hospital	Institute of Tropical Medicine	Philippe Selhorst
EPI_ISL_1594431	Labo Analyses Med	National Reference Center for Viruses of Respiratory Infections, Institut Pasteur, Paris	Marion Barbet, Sylvie Behillil, Frédéric Lemoine, Corinne Maufrais, Christophe Malabat, Adrien Pain, Méline Bizard, Angela Brisebarre, Camille Capel, Louise Lefrançois, Etienne Simon-Lorière, Vincent Enouf, Maud Vanpeene, Sylvie van der Werf,J Besson
EPI_ISL_1595731	Microbiology Department. Complexo Hospitalario Universitario de Vigo	Microbiology Department. Complexo Hospitalario Universitario de Vigo	Alfaya N, Alonso I, Alvarez M, Cabrera JJ, Carballo R, Cores O, Cortizo S, del-Campo V, Martinez L, Mediero G, Perez S, Potel C, Regueiro B, Rey S, Vasallo FJ
EPI_ISL_1620172, EPI_ISL_1620173, EPI_ISL_1620174	MRCG at LSHTM Genomics lab	MRCG at LSHTM Genomics lab	Abdul Karim sesay, Abdoulie Kanteh, Jarra Manneh, Mariama Kujabi, Bakary Sanyang
EPI_ISL_1622360, EPI_ISL_1622361	RS Mitra Keluarga Gading Serpong	Eijkman Institute for Molecular Biology, Ministry of Research and Technology/National Agency for Research and Innovation	Willy Agustine, Hidayat Trimarsanto, Lydia V. Panggalo, Iskandar Adnan, Sukma Oktavianthi, Edison Johar, Frilasita A Yudhaputri, Muhammad Rezki Rasyak, Safarina G Malik, Khin Saw Myint, Amin Soebandrio
EPI_ISL_1622362	RS Sentra Medika Cikarang	Eijkman Institute for Molecular Biology, Ministry of Research and Technology/National Agency for Research and Innovation	Willy Agustine, Hidayat Trimarsanto, Lydia V. Panggalo, Iskandar Adnan, Sukma Oktavianthi, Edison Johar, Frilasita A Yudhaputri, Muhammad Rezki Rasyak, Safarina G Malik, Khin Saw Myint, Amin Soebandrio
EPI_ISL_1622363, EPI_ISL_1622364	RS Sari Asih Ciledug	Eijkman Institute for Molecular Biology, Ministry of Research and Technology/National Agency for Research and Innovation	Willy Agustine, Hidayat Trimarsanto, Lydia V. Panggalo, Iskandar Adnan, Sukma Oktavianthi, Edison Johar, Frilasita A Yudhaputri, Muhammad Rezki Rasyak, Safarina G Malik, Khin Saw Myint, Amin Soebandrio
EPI_ISL_1622365	RS Bhakti Husada Cikarang	Eijkman Institute for Molecular Biology, Ministry of Research and Technology/National Agency for Research and Innovation	Willy Agustine, Hidayat Trimarsanto, Lydia V. Panggalo, Iskandar Adnan, Sukma Oktavianthi, Edison Johar, Frilasita A Yudhaputri, Muhammad Rezki Rasyak, Safarina G Malik, Khin Saw Myint, Amin Soebandrio
EPI_ISL_1622366	RSUD Cileungsi	Eijkman Institute for Molecular Biology, Ministry of Research and Technology/National Agency for Research and Innovation	Willy Agustine, Hidayat Trimarsanto, Lydia V. Panggalo, Iskandar Adnan, Sukma Oktavianthi, Edison Johar, Frilasita A Yudhaputri, Muhammad Rezki Rasyak, Safarina G Malik, Khin Saw Myint, Amin Soebandrio
EPI_ISL_1622367	RS Harapan Bunda	Eijkman Institute for Molecular Biology, Ministry of Research and Technology/National Agency for Research and Innovation	Willy Agustine, Hidayat Trimarsanto, Lydia V. Panggalo, Iskandar Adnan, Sukma Oktavianthi, Edison Johar, Frilasita A Yudhaputri, Muhammad Rezki Rasyak, Safarina G Malik, Khin Saw Myint, Amin Soebandrio
EPI_ISL_1622368	RSJ Dr. Soeharto Heerdjan	Eijkman Institute for Molecular Biology, Ministry of Research and Technology/National Agency for Research and Innovation	Willy Agustine, Hidayat Trimarsanto, Lydia V. Panggalo, Iskandar Adnan, Sukma Oktavianthi, Edison Johar, Frilasita A Yudhaputri, Muhammad Rezki Rasyak, Safarina G Malik, Khin Saw Myint, Amin Soebandrio
EPI_ISL_1622369	RS Sumber Waras	Eijkman Institute for Molecular Biology, Ministry of Research and Technology/National Agency for Research and Innovation	Willy Agustine, Hidayat Trimarsanto, Lydia V. Panggalo, Iskandar Adnan, Sukma Oktavianthi, Edison Johar, Frilasita A Yudhaputri, Muhammad Rezki Rasyak, Safarina G Malik, Khin Saw Myint, Amin Soebandrio
EPI_ISL_1622370	RSIA Selasih Medika	Eijkman Institute for Molecular Biology, Ministry of Research and Technology/National Agency for Research and Innovation	Willy Agustine, Hidayat Trimarsanto, Lydia V. Panggalo, Iskandar Adnan, Sukma Oktavianthi, Edison Johar, Frilasita A Yudhaputri, Muhammad Rezki Rasyak, Safarina G Malik, Khin Saw Myint, Amin Soebandrio
EPI_ISL_1622371	RS Mitra Keluarga Gading Serpong	Eijkman Institute for Molecular Biology, Ministry of Research and Technology/National Agency for Research and Innovation	Willy Agustine, Hidayat Trimarsanto, Lydia V. Panggalo, Iskandar Adnan, Sukma Oktavianthi, Edison Johar, Frilasita A Yudhaputri, Muhammad Rezki Rasyak, Safarina G Malik, Khin Saw Myint, Amin Soebandrio
EPI_ISL_1622372	RS Mitra Keluarga Gading Serpong	Eijkman Institute for Molecular Biology, Ministry of Research and Technology/National Agency for Research and Innovation	Hidayat Trimarsanto, Lydia V. Panggalo, Iskandar Adnan, Sukma Oktavianthi, Edison Johar, Frilasita A Yudhaputri, Muhammad Rezki Rasyak, Willy Agustine, Safarina G Malik, Khin Saw Myint, Amin Soebandrio
EPI_ISL_1622373	RS Mary Cileungsi Hijau	Eijkman Institute for Molecular Biology, Ministry of Research and Technology/National Agency for Research and Innovation	Hidayat Trimarsanto, Lydia V. Panggalo, Iskandar Adnan, Sukma Oktavianthi, Edison Johar, Frilasita A Yudhaputri, Muhammad Rezki Rasyak, Willy Agustine, Safarina G Malik, Khin Saw Myint, Amin Soebandrio
EPI_ISL_1622374	RS Sentra Medika Cikarang	Eijkman Institute for Molecular Biology, Ministry of Research and Technology/National Agency for Research and Innovation	Hidayat Trimarsanto, Lydia V. Panggalo, Iskandar Adnan, Sukma Oktavianthi, Edison Johar, Frilasita A Yudhaputri, Muhammad Rezki Rasyak, Willy Agustine, Safarina G Malik, Khin Saw Myint, Amin Soebandrio
EPI_ISL_1622375	RS Taman Harapan Baru	Eijkman Institute for Molecular Biology, Ministry of Research and Technology/National Agency for Research and Innovation	Hidayat Trimarsanto, Lydia V. Panggalo, Iskandar Adnan, Sukma Oktavianthi, Edison Johar, Frilasita A Yudhaputri, Muhammad Rezki Rasyak, Willy Agustine, Safarina G Malik, Khin Saw Myint, Amin Soebandrio

EPI_ISL_1622376, EPI_ISL_1622377	RSAB Harapan Kita	Eijkman Institute for Molecular Biology, Ministry of Research and Technology/National Agency for Research and Innovation	Hidayat Trimarsanto, Lydia V. Panggalo, Iskandar Adnan, Sukma Oktavianthi, Edison Johar, Frilasita A Yudhaputri, Muhammad Rezki Rasyak, Willy Agustine, Safarina G Malik, Khin Saw Myint, Amin Soebandrio
EPI_ISL_1622378	RSIA Bun	Eijkman Institute for Molecular Biology, Ministry of Research and Technology/National Agency for Research and Innovation	Hidayat Trimarsanto, Lydia V. Panggalo, Iskandar Adnan, Sukma Oktavianthi, Edison Johar, Frilasita A Yudhaputri, Muhammad Rezki Rasyak, Willy Agustine, Safarina G Malik, Khin Saw Myint, Amin Soebandrio
EPI_ISL_1622379, EPI_ISL_1622380	RS Mitra Keluarga Gading Serpong	Eijkman Institute for Molecular Biology, Ministry of Research and Technology/National Agency for Research and Innovation	Hidayat Trimarsanto, Lydia V. Panggalo, Iskandar Adnan, Sukma Oktavianthi, Edison Johar, Frilasita A Yudhaputri, Muhammad Rezki Rasyak, Willy Agustine, Safarina G Malik, Khin Saw Myint, Amin Soebandrio
EPI_ISL_1622388	RS Mitra Keluarga Gading Serpong	Eijkman Institute for Molecular Biology, Ministry of Research and Technology/National Agency for Research and Innovation	Lydia V. Panggalo, Iskandar Adnan, Sukma Oktavianthi, Edison Johar, Frilasita A Yudhaputri, Muhammad Rezki Rasyak, Willy Agustine, Hidayat Trimarsanto, Safarina G Malik, Khin Saw Myint, Amin Soebandrio
EPI_ISL_1623687	National Platform bis UMONS/Jolimont	National Platform bis UMONS/Jolimont	François Dufrasne, Guillaume Bayon-Vicente, Gautier Detry, Ruddy Wattiez
EPI_ISL_1626009	Instituto Adolfo Lutz - Regional de Rio Claro	Instituto Adolfo Lutz, Interdiciplinary Procedures Center, Strategic Laboratory	Claudio Tavares Sacchi, Claudia Regina Gonçalves, Erica Valessa Ramos Gomes, Karoline Rodrigues Campos, Caio Vinicius Dias Lopes, Leonardo Jose Tadeu de Araujo, Katia Correa de Oliveira Santos
EPI_ISL_1626490, EPI_ISL_1626493, EPI_ISL_1626499, EPI_ISL_1626500, EPI_ISL_1626501, EPI_ISL_1626502, EPI_ISL_1626514, EPI_ISL_1626516, EPI_ISL_1626519, EPI_ISL_1626521, EPI_ISL_1626522, EPI_ISL_1626523, EPI_ISL_1626524, EPI_ISL_1626527			
see above	NSTU COVID-19 Diagnostic Center	NSU Genome Research Institute (NGRI)	Maqsud Hossain, Tahrima Saiha Huq, Aura Rahman, Md. Aminul Islam, Syeda Naushin Tabassum, Kazi Nadim Hasan, Abdul Khaleque, Abdus Sadique, Mohammad Salim Hossain, Newaz Mohammed Bahadur, Firoz Ahmed, Hasan Mahmud Reza
EPI_ISL_1628345, EPI_ISL_1628346	Instituto Adolfo Lutz - Regional de Rio Claro	Instituto Adolfo Lutz, Interdiciplinary Procedures Center, Strategic Laboratory	Claudio Tavares Sacchi, Claudia Regina Gonçalves, Erica Valessa Ramos Gomes, Karoline Rodrigues Campos, Caio Vinicius Dias Lopes, Leonardo Jose Tadeu de Araujo, Katia Correa de Oliveira Santos
EPI_ISL_1628474	Universidad Industrial de Santander (Laboratorio Central de Investigaciones - Clínica Chicamocha).	Universidad Industrial de Santander.	Francisco Martinez-Perez, Cristian E. Cadena-Caballero, Diego Rueda-Plata, Carolina S. Torres-Jiménez, Lizeth J. Forero-Buitrago, Erika Lizarazo-Gutiérrez, Carlos Barrios-Hernández, Lina M. Vera-Cala.
EPI_ISL_1628499	Universidad Industrial de Santander (Laboratorio Central de Investigaciones - Clínica Chicamocha).	Universidad Industrial de Santander	Francisco Martinez-Perez, Cristian E. Cadena-Caballero, Diego Rueda-Plata, Carolina S. Torres-Jiménez, Lizeth J. Forero-Buitrago, Erika Lizarazo-Gutiérrez, Carlos Barrios-Hernández, Lina M. Vera-Cala.
EPI_ISL_1629382, EPI_ISL_1629387, EPI_ISL_1629388, EPI_ISL_1629397	CHU	UMR PIMIT	Dr David A Wilkinson, Dr Patrick Mavingui, Dr Camille Lebarbenchon, Magali Turpin
EPI_ISL_1629710	Universidad Industrial de Santander (Laboratorio Central de Investigaciones - Clínica Chicamocha).	Universidad Industrial de Santander.	Francisco Martinez-Perez, Cristian E. Cadena-Caballero, Diego Rueda-Plata, Carolina S. Torres-Jiménez, Lizeth J. Forero-Buitrago, Erika Lizarazo-Gutiérrez, Carlos Barrios-Hernández, Lina M. Vera-Cala.
EPI_ISL_1629720	ICMT	Universidad Nacional de Colombia - Laboratorio Genómico One Health	Maria Stella López, Andres F. Cardona-Rios, Daniel O. Maldonado-Perez, Simón Villegas Velásquez, Laura Silvana Perez, Karl A Ciuoderis, Idabely Betancur Ortiz, Sandra Ines Cano, Eliana Patricia Calvo Tapiero, Diego A. Álvarez-Díaz, Carlos Franco-Muñoz, Hector Alejandro Ruiz-Moreno, Marcela Mercado-Reyes, Jorge E. Osorio, Juan P. Hernandez-Ortiz
EPI_ISL_1629724, EPI_ISL_1629725	SOMER	Universidad Nacional de Colombia - Laboratorio Genómico One Health	Simón Villegas Velásquez, Andres F. Cardona-Rios, Daniel O. Maldonado-Perez, Maria Stella López, Laura Silvana Perez, Karl A Ciuoderis, Idabely Betancur Ortiz, Sandra Ines Cano, Eliana Patricia Calvo Tapiero, Diego A. Álvarez-Díaz, Carlos Franco-Muñoz, Hector Alejandro Ruiz-Moreno, Marcela Mercado-Reyes, Jorge E. Osorio, Juan P. Hernandez-Ortiz
EPI_ISL_1629729	UDEA	Universidad Nacional de Colombia - Laboratorio Genómico One Health	Daniel O. Maldonado-Perez, Andres F. Cardona-Rios, Maria Stella López, Simón Villegas Velásquez, Laura Silvana Perez, Karl A Ciuoderis, Idabely Betancur Ortiz, Sandra Ines Cano, Eliana Patricia Calvo Tapiero, Diego A. Álvarez-Díaz, Carlos Franco-Muñoz, Hector Alejandro Ruiz-Moreno, Marcela Mercado-Reyes, Jorge E. Osorio, Juan P. Hernandez-Ortiz
EPI_ISL_1629734	SURA	Universidad Nacional de Colombia - Laboratorio Genómico One Health	Laura Silvana Perez, Andres F. Cardona-Rios, Daniel O. Maldonado-Perez, Maria Stella López, Simón Villegas Velásquez, Karl A Ciuoderis, Idabely Betancur Ortiz, Sandra Ines Cano, Eliana Patricia Calvo Tapiero, Diego A. Álvarez-Díaz, Carlos Franco-Muñoz, Hector Alejandro Ruiz-Moreno, Marcela Mercado-Reyes, Jorge E. Osorio, Juan P. Hernandez-Ortiz
EPI_ISL_1629744, EPI_ISL_1629745	LABORATORIO ECHAVARRIA	Universidad Nacional de Colombia - Laboratorio Genómico One Health	Andres F. Cardona-Rios, Daniel O. Maldonado-Perez, Maria Stella López, Simón Villegas Velásquez, Laura Silvana Perez, Karl A Ciuoderis, Idabely Betancur Ortiz, Sandra Ines Cano, Eliana Patricia Calvo Tapiero, Diego A. Álvarez-Díaz, Carlos Franco-Muñoz, Hector Alejandro Ruiz-Moreno, Marcela Mercado-Reyes, Jorge E. Osorio, Juan P. Hernandez-Ortiz
EPI_ISL_1629752	SYNLAB	Universidad Nacional de Colombia - Laboratorio Genómico One Health	Andres F. Cardona-Rios, Daniel O. Maldonado-Perez, Maria Stella López, Simón Villegas Velásquez, Laura Silvana Perez, Karl A Ciuoderis, Idabely Betancur Ortiz, Sandra Ines Cano, Eliana Patricia Calvo Tapiero, Diego A. Álvarez-Díaz, Carlos Franco-Muñoz, Hector Alejandro Ruiz-Moreno, Marcela Mercado-Reyes, Jorge E. Osorio, Juan P. Hernandez-Ortiz
EPI_ISL_1629753, EPI_ISL_1629754, EPI_ISL_1629755, EPI_ISL_1629757, EPI_ISL_1629759	H.U SAN VICENTE FUNDACION	Universidad Nacional de Colombia - Laboratorio Genómico One Health	Andres F. Cardona-Rios, Daniel O. Maldonado-Perez, Maria Stella López, Simón Villegas Velásquez, Laura Silvana Perez, Karl A Ciuoderis, Idabely Betancur Ortiz, Sandra Ines Cano, Eliana Patricia Calvo Tapiero, Diego A. Álvarez-Díaz, Carlos Franco-Muñoz, Hector Alejandro Ruiz-Moreno, Marcela Mercado-Reyes, Jorge E. Osorio, Juan P. Hernandez-Ortiz
EPI_ISL_1629760, EPI_ISL_1629761	LABMÉDICO	Universidad Nacional de Colombia - Laboratorio Genómico One Health	Andres F. Cardona-Rios, Daniel O. Maldonado-Perez, Maria Stella López, Simón Villegas Velásquez, Laura Silvana Perez, Karl A Ciuoderis, Idabely Betancur Ortiz, Sandra Ines Cano, Eliana Patricia Calvo Tapiero, Diego A. Álvarez-Díaz, Carlos Franco-Muñoz, Hector Alejandro Ruiz-Moreno, Marcela Mercado-Reyes, Jorge E. Osorio, Juan P. Hernandez-Ortiz
EPI_ISL_1629769, EPI_ISL_1629770, EPI_ISL_1629772, EPI_ISL_1629776, EPI_ISL_1629778	Hospital Carlos Alberto Seguín Escobedo - EsSalud	Laboratorio de Genómica Microbiana, Universidad Peruana Cayetano Heredia	Lenin Maturrano, Pablo Tsukayama, Alejandra Dávila-Barclay, Guillermo Salvatierra, Luis González, Pedro E. Romero, Janet Huancachoque, Pool Marcos, Fernando Sánchez Fragoso, Lin Zevallos Cuarite
EPI_ISL_1630158	Laboratório de Microbiologia Molecular - Universidade FEEVALE	Laboratório de Microbiologia Molecular - Universidade FEEVALE	Alana Witt Hansen, Fágner Henrique Heldt, Fernando Rosado Spilki, Flávio Silveira, Juliana Schons Gularte, Juliane Deise Fleck, Mariana Soares da Silva, Meriane Demoliner, Matheus Nunes Weber, Paula Rodrigues de Almeida, Micheli Filippi.
EPI_ISL_1647425, EPI_ISL_1647426, EPI_ISL_1647446, EPI_ISL_1647447, EPI_ISL_1647448, EPI_ISL_1647449, EPI_ISL_1647450, EPI_ISL_1647451, EPI_ISL_1647452	Indian Council of Medical Research-National Institute of Virology, Microbial Containment Complex	Indian Council of Medical Research-National Institute of Virology, Microbial Containment Complex	Pragya D Yadav
EPI_ISL_1652049, EPI_ISL_1652050, EPI_ISL_1652051, EPI_ISL_1652052, EPI_ISL_1652053, EPI_ISL_1652054, EPI_ISL_1652057, EPI_ISL_1652058, EPI_ISL_1652061, EPI_ISL_1652062, EPI_ISL_1652064, EPI_ISL_1652066, EPI_ISL_1652067, EPI_ISL_1652068, EPI_ISL_1652071, EPI_ISL_1652072, EPI_ISL_1652073			
see above	Integrated Biorepository of H3Africa Uganda - IBRH3AU	Molecular Biology Laboratory	Savannah Mwesigwa, Eric Katagirya, Gerald Mboowa, David Patrick Kateete, Misaki Wayengera, Emmanuel Nasinghe, Ashaba Fred Katabazi, Edgar Kigozi, Samuel Kirimunda, Rogers Kamulegeya, Moses Luutu, Lwanga Newton, Nsubuga Gideon, Bernard Ssentalo Bagaya, Sarah Stanley, Moses Joloba
EPI_ISL_1653804, EPI_ISL_1653805, EPI_ISL_1653807, EPI_ISL_1653809, EPI_ISL_1653811, EPI_ISL_1653812	Southern Nevada Public Health Laboratory	Southern Nevada Public Health Laboratory	Michael Picker
EPI_ISL_1654202, EPI_ISL_1654204, EPI_ISL_1654205	Research	National Reference Center for Viruses of Respiratory Infections, Institut Pasteur, Paris	Marion Barbet, Sylvie Behillil, Frédéric Lemoine, Corinne Maufrais, Christophe Malabat, Damien Mornico, Méline Bizard, Angela Brisebarre, Camille Capel, Louise Lefrançois, Etienne Simon-Lorière, Vincent Enouf, Maud Vanpeene, Sylvie van der Werf,Marianne Leruez-Ville
EPI_ISL_1655075	Fujian Center for Disease Control and Prevention	Fujian Center for Disease Control and Prevention	Lin Qi, Huang Zhimiao, Weng Yuwei
EPI_ISL_1661250, EPI_ISL_1661251, EPI_ISL_1661252	Laboratorio de Ecologia de Doencas Transmissiveis na Amazonia, Instituto Leonidas e Maria Deane - Fiocruz	Laboratorio de Ecologia de Doencas Transmissiveis na Amazonia, Instituto Leonidas e Maria Deane - Fiocruz	Valdinete Nascimento, Victor Souza, André Corado, Fernanda Nascimento, George Silva, Ágatha Costa, Debora Duarte, Karina Pessoa, Matilde Mejía, Luciana Gonçalves, Maria Júlia Brandão, Michele Jesus, Felipe Naveca

	Amazonia	Amazonia	
EPI_ISL_1663017	Fujian Center for Disease Control and Prevention	Fujian Center for Disease Control and Prevention	Lin Qi, Huang Zhimiao, Weng Yuwei
EPI_ISL_1673261, EPI_ISL_1673262	LABORATORIO LAS AMERICAS	Universidad Nacional de Colombia - Laboratorio Genómico One Health	Maria Stella López, Andres F. Cardona-Rios, Daniel O. Maldonado-Perez, Simón Villegas Velásquez, Lina M. Hurtado, Laura Silvana Perez, Karl A Ciuoderis, Idabely Betancur Ortiz, Sandra Ines Cano, Eliana Patricia Calvo Tapiero, Diego A. Álvarez-Díaz, Carlos Franco-Muñoz, Hector Alejandro Ruiz-Moreno, Marcela Mercado-Reyes, Jorge E. Osorio, Juan P. Hernandez-Ortiz
EPI_ISL_1675009	Labo Analyses Med	National Reference Center for Viruses of Respiratory Infections, Institut Pasteur, Paris	Marion Barbet, Sylvie Behillil, Méline Bizard, Angela Brisebarre, Camille Capel, Vincent Enouf, Louise Lefrançois, Frédéric Lemoine, Christophe Malabat, Corinne Maufrais, Etienne Simon-Lorière, Maud Vanpeene, Sylvie Van der Werf ,Brieuc Gestin
EPI_ISL_1675033	Hospital	National Reference Center for Viruses of Respiratory Infections, Institut Pasteur, Paris	Marion Barbet, Sylvie Behillil, Méline Bizard, Angela Brisebarre, Camille Capel, Vincent Enouf, Louise Lefrançois, Frédéric Lemoine, Christophe Malabat, Corinne Maufrais, Etienne Simon-Lorière, Maud Vanpeene, Sylvie Van der Werf ,Alexandra Ducancelle
EPI_ISL_1708512	Collection of WHO National Influenza Center, N.F. Gamaleya National Research Center for Epidemiology and Microbiology of the Ministry of Health of the Russian Federation	Laboratory of genome analysis, N.F. Gamaleya National Research Center for Epidemiology and Microbiology of the Ministry of Health of the Russian Federation	Olga Voronina, Ekaterina Aksenova, Natalia Ryzhova, Marina Kunda, Kirill Krasnoslobodtsev, Elena Burtseva, Denis Logunov
EPI_ISL_1708513, EPI_ISL_1708514, EPI_ISL_1708515, EPI_ISL_1708516, EPI_ISL_1708519	State collection of viruses, N.F. Gamaleya National Research Center for Epidemiology and Microbiology of the Ministry of Health of the Russian Federation	Laboratory of genome analysis, N.F. Gamaleya National Research Center for Epidemiology and Microbiology of the Ministry of Health of the Russian Federation	Olga Voronina, Ekaterina Aksenova, Natalia Ryzhova, Marina Kunda, Inna Dolzhikova, Andrey Botikov, Daria Grousova, Anna Kovyrshina, Denis Logunov
EPI_ISL_1710849, EPI_ISL_1710850, EPI_ISL_1710851, EPI_ISL_1710852, EPI_ISL_1710853, EPI_ISL_1710854	Pathogenic Microorganisms Variability Laboratory	Pathogenic Microorganisms Variability Laboratory	Alexey Shchetinin, Maria Nikiforova, Andrei Siniavin, Andrey Pochtovyy, Evgeny Usachev, Elena Shidlovskaya, Elizaveta Divisenko, Nadezhda Kuznetsova, Valeria Bacalin, Olga Burgasova, Ludmila Kolobukhina, Svetlana Smetanina, Denis Logunov, Vladimir Gushchin, Alexander Gintsburg
EPI_ISL_1714798	Institute for Medical Research, Infectious Disease Research Centre, National Institutes of Health, Ministry of Health Malaysia	Institute for Medical Research, Infectious Disease Research Centre, National Institutes of Health, Ministry of Health Malaysia	Suppiah J, Kamel K, Azizan MA, Thayan R
EPI_ISL_1731043	Collection of WHO National Influenza Center, N.F. Gamaleya National Research Center for Epidemiology and Microbiology of the Ministry of Health of the Russian Federation	N.F. Gamaleya Research Center for Epidemiology and Microbiology	Olga Voronina, Ekaterina Aksenova, Natalia Ryzhova, Marina Kunda, Kirill Krasnoslobodtsev, Elena Burtseva, Denis Logunov
EPI_ISL_1731044, EPI_ISL_1731045	State collection of viruses, N.F. Gamaleya National Research Center for Epidemiology and Microbiology of the Ministry of Health of the Russian Federation	N.F. Gamaleya Research Center for Epidemiology and Microbiology	Olga Voronina, Ekaterina Aksenova, Natalia Ryzhova, Marina Kunda, Inna Dolzhikova, Andrey Botikov, Daria Grousova, Anna Kovyrshina, Denis Logunov
EPI_ISL_1731067, EPI_ISL_1731068	Molecular diagnostic laboratory of Federal Budget Institution of Science "Central Research Institute of Epidemiology" of The Federal Service on Customers' Rights Protection and Human Well-being Surveillance	Group of Genomics and Postgenomic Technologies of Central Research Institute of Epidemilology	Samoilov AE, Kaptelova VV, Korneenko EV, Saenko SS, Shipulina OY, Speranskaya AS, Tivanova EV, Kondrasheva LY, Akimkin VG
EPI_ISL_1731069, EPI_ISL_1731070, EPI_ISL_1731071, EPI_ISL_1731072, EPI_ISL_1731073, EPI_ISL_1731074, EPI_ISL_1731075, EPI_ISL_1731076, EPI_ISL_1731078, EPI_ISL_1731079	Laboratory of Extremely Dangerous Infections of Federal Budget Health Care Instute "Center of Hygiene and Epidemiology in Sakhalin region"	Group of Genomics and Postgenomic Technologies of Central Research Institute of Epidemiology	Samoilov AE, Kaptelova VV, Korneenko EV, Valdokhina AV, Bulanenko VP, Saenko SS, Arutiunian AE, Byankina MA, Zagorodnov YL, Golubeva AG, Speranskaya AS, Tivanova EV, Kondrasheva LY, Akimkin VG
EPI_ISL_1752475, EPI_ISL_1752510	LHUB-ULB	LHUB-ULB	Charlotte Michel, Olivier Vandenberg, Marie Hallin
EPI_ISL_1752592, EPI_ISL_1752593, EPI_ISL_1752594, EPI_ISL_1752595, EPI_ISL_1752596, EPI_ISL_1752597, EPI_ISL_1752598, EPI_ISL_1752599, EPI_ISL_1752600, EPI_ISL_1752601, EPI_ISL_1752602, EPI_ISL_1752603, EPI_ISL_1752604, EPI_ISL_1752605, EPI_ISL_1752606, EPI_ISL_1752607, EPI_ISL_1752608, EPI_ISL_1752609, EPI_ISL_1752610, EPI_ISL_1752611, EPI_ISL_1752612, EPI_ISL_1752613, EPI_ISL_1752614, EPI_ISL_1752615, EPI_ISL_1752616, EPI_ISL_1752617, EPI_ISL_1752618, EPI_ISL_1752619, EPI_ISL_1752620, EPI_ISL_1752632, EPI_ISL_1752634, EPI_ISL_1752635			
see above	Showa University PCR Center	Showa University PCR Center	Fumihiro Ishikawa, Hideto Oyamada, Yuko Udaka, Keiko Ishino, Yuji Kiuchi
EPI_ISL_1753847	Servicio Microbiología Hospital La Paz	Servicio Microbiología Hospital La Paz	Fernando Lázaro, Rubén Cáceres, Jesús Mingorance Cruz, Elie Dahdouh
EPI_ISL_1760210, EPI_ISL_1760211, EPI_ISL_1760212, EPI_ISL_1760213, EPI_ISL_1760214, EPI_ISL_1760215, EPI_ISL_1760216, EPI_ISL_1760217, EPI_ISL_1760218, EPI_ISL_1760219, EPI_ISL_1760220, EPI_ISL_1760221, EPI_ISL_1760222, EPI_ISL_1760223, EPI_ISL_1760224, EPI_ISL_1760225, EPI_ISL_1760226, EPI_ISL_1760227, EPI_ISL_1760228, EPI_ISL_1760229, EPI_ISL_1760230, EPI_ISL_1760231, EPI_ISL_1760232, EPI_ISL_1760233, EPI_ISL_1760234, EPI_ISL_1760235, EPI_ISL_1760241, EPI_ISL_1760242			
see above	Centers for Disease Control and Prevention, Dengue Branch	Centers for Disease Control and Prevention, Dengue Branch	Gilberto A. Santiago, Glenda Gonzalez, Betzabel Flores, Keyla Charriez, Gabriela Paz-Bailey, Jorge L. Munoz-Jordan
EPI_ISL_1760615, EPI_ISL_1760616, EPI_ISL_1760617, EPI_ISL_1760618, EPI_ISL_1760619, EPI_ISL_1760621, EPI_ISL_1760623, EPI_ISL_1760624, EPI_ISL_1760625, EPI_ISL_1760626, EPI_ISL_1760627, EPI_ISL_1760628, EPI_ISL_1760629, EPI_ISL_1760631, EPI_ISL_1760632, EPI_ISL_1760633, EPI_ISL_1760634, EPI_ISL_1760635, EPI_ISL_1760636, EPI_ISL_1760637, EPI_ISL_1760638, EPI_ISL_1760639, EPI_ISL_1760640, EPI_ISL_1760641, EPI_ISL_1760642, EPI_ISL_1760643, EPI_ISL_1760644, EPI_ISL_1760645, EPI_ISL_1760646			
see above	Clinical specimen	Centre de Recherche et de Formation en Infectiologie Guinée	Alpha Kabinet KEITA, Haby DIALLO, Abdoul Karim SOUMAH, Abdoulaye TOURE, Thibaut Armel Cherif GNIMADI, Joel KOIVOGUI, Jean-louis MONEMOU, Moriba POVOGUI, Mamadou Saliou SOW, Mamadou Bhoye KEITA, Penda Malhado DIALLO, Alimou CAMARA, Kaba KOUROUMA, Mandiou DIAKITE, Mamadou Saliou BAH, Sakoba KEITA, Bouna Yatassaye, Christelle BUTEL, Laetitia SERRANO, Ahidjo AYOUBA, Eric DELAPORTE, Martine PEETERS
EPI_ISL_1761310, EPI_ISL_1761311, EPI_ISL_1761312, EPI_ISL_1761313, EPI_ISL_1761314, EPI_ISL_1761315, EPI_ISL_1761316, EPI_ISL_1761317, EPI_ISL_1761318, EPI_ISL_1761319, EPI_ISL_1761320, EPI_ISL_1761321, EPI_ISL_1761323, EPI_ISL_1761324, EPI_ISL_1761325, EPI_ISL_1761326, EPI_ISL_1761327, EPI_ISL_1761328, EPI_ISL_1761329, EPI_ISL_1761330, EPI_ISL_1761331, EPI_ISL_1761332, EPI_ISL_1761333, EPI_ISL_1761334, EPI_ISL_1761335, EPI_ISL_1761336, EPI_ISL_1761337, EPI_ISL_1761338, EPI_ISL_1761339, EPI_ISL_1761340, EPI_ISL_1761363			
see above	Tokyo Medical and Dental University	Tokyo Medical and Dental University	Kousuke Tanimoto, Yukie Tanaka, Yoko Nukui, Akinori Kimura, Hiroaki Takeuchi
EPI_ISL_1761587	Hospital de Palamós. Serveis de Salut Integral Baix Empordà (SSIBE)	IrsiCaixa	Marc Noguera-Julian, Mariona Parera, Maria Casadellà, Pilar Armengol, Francesc Catala-Moll, Roger Paredes, Bonaventura Clotet Martin-Urda Diez-Canseco, Anabel; Toda Savall, Mº de la Roca; Marchena Romero, Jose Andres; Rodriguez Jareño, Mari Cruz; Erick Fernando Mayer Arispe; Claver Belver, Natalia
EPI_ISL_1785071, EPI_ISL_1785072, EPI_ISL_1785074, EPI_ISL_1785075, EPI_ISL_1785076, EPI_ISL_1785077, EPI_ISL_1785078, EPI_ISL_1785079, EPI_ISL_1785080	Infectious Agents & Hygiene	GIMAP and Virpath teams-CIRI	Sylvie Pillet, Thomas Bourlet, Julien Fouret, Thomas Julien, Victoria Dulière, Olivier Terrier, Andrés Pizzorno, Manuel Rosa-Calatrava, Bruno Pozzetto, Stéphane Paul
EPI_ISL_1789114	Siti Khodijah Hospital	Institute of Tropical Disease, Universitas Airlangga	Jezzy R Dewantari, Rima R Prasetya, Krisnoadi Rahardjo, Aldise M Nastri, Muhammad Hamdan, Gatot Soegiarto, Laksmi Wulandari, Resti Yudhawati, Soetjipto, Yasuko Mori, Maria I Lusida, Kazufumi Shimizu
EPI_ISL_1789116	Anwar Medika General Hospital	Institute of Tropical Disease, Universitas Airlangga	Maria I Lusida, Krisnoadi Rahardjo, Aldise M Nastri, Jezzy R Dewantari, Rima R Prasetya, Nungky Taniasari, Gatot Soegiarto, Laksmi Wulandari, Resti Yudhawati, Soetjipto, Yasuko Mori, Kazufumi Shimizu
EPI_ISL_1789117	Anwar Medika General Hospital	Institute of Tropical Disease, Universitas Airlangga	Krisnoadi Rahardjo, Aldise M Nastri, Jezzy R Dewantari, Rima R Prasetya, Muhammad Hamdan, Gatot Soegiarto, Laksmi Wulandari, Resti Yudhawati, Yasuko Mori, Soetjipto, Kazufumi Shimizu, Maria I Lusida
EPI_ISL_1790080, EPI_ISL_1790082, EPI_ISL_1790084, EPI_ISL_1790086	Centre Pasteur Annexe de Garoua	Institut Pasteur de Dakar	Njouom Richard, Diagne Moussa Moïse, Dia Ndongo, Diallo Amadou, Sankhe Safietou, Diop Mamadou, Ndiaye Ndack, Loucoubar Cheikh, Carniel Elisabeth, Faye Ousmane, Sall Amadou Alpha
EPI_ISL_1795053	Anwar Medika General Hospital	Institute of Tropical Disease, Universitas Airlangga	Aldise M Nastri, Jezzy R Dewantari, Rima R Prasetya, Krisnoadi Rahardjo, Nungky Taniasari, Gatot Soegiarto, Laksmi Wulandari, Resti Yudhawati, Yasuko Mori, Soetjipto, Kazufumi Shimizu, Maria I Lusida
EPI_ISL_1795054	Darus Syifa' Islamic Hospital	Institute of Tropical Disease, Universitas Airlangga	Krisnoadi Rahardjo, Aldise M Nastri, Jezzy R Dewantari, Rima R Prasetya, HM Faiz, Gatot Soegiarto, Laksmi Wulandari, Resti Yudhawati, Yasuko Mori, Soetjipto, Kazufumi Shimizu, Maria I Lusida

EPI_ISL_1795055	Muhammadiyah Hospital	Institute of Tropical Disease, Universitas Airlangga	Rima R Prasetya, Krisnoadi Rahardjo, Aldise M Nastri, Jezzy R Dewantari, Imam Suyuthi, Gatot Soegiarto, Laksmi Wulandari, Resti Yudhawati, Yasuko Mori, Soetjipto, Kazufumi Shimizu, Maria I Lusida
EPI_ISL_1795056	Siloam Hospital	Institute of Tropical Disease, Universitas Airlangga	Kazufumi Shimizu, Krisnoadi Rahardjo, Aldise M Nastri, Jezzy R Dewantari, Rima R Prasetya, Maria M Padmidewi, Gatot Soegiarto, Laksmi Wulandari, Retno A Setyoningrum, Resti Yudhawati, Yohko K Shimizu, Mitsuhiro Nishimura, Yasuko Mori, Soetjipto, Maria I Lusida
EPI_ISL_1795057	Muhammadiyah Hospital	Institute of Tropical Disease, Universitas Airlangga	Jezzy R Dewantari, Rima R Prasetya, Krisnoadi Rahardjo, Aldise M Nastri, Imam Suyuthi, Gatot Soegiarto, Laksmi Wulandari, Resti Yudhawati, Yasuko Mori, Soetjipto, Kazufumi Shimizu, Maria I Lusida
EPI_ISL_1795059	Citra Medika Hospital	Institute of Tropical Disease, Universitas Airlangga	Aldise M Nastri, Jezzy R Dewantari, Rima R Prasetya, Krisnoadi Rahardjo, Wahidah Rachmaniyah, Gatot Soegiarto, Laksmi Wulandari, Resti Yudhawati, Yasuko Mori, Soetjipto, Kazufumi Shimizu, Maria I Lusida
EPI_ISL_1805697	National Center for Communicable Diseases (NCCD) National Influenza Center	National Centre for Disease Control (NCDC) National Influenza Center	Naranzul Ts,Bayasgalan N,Khishigmunkh Ch,Seiichiro F,Hideka M,Mina N,Shinji W,Ankhbayar S,Tsogzolmaa G,Darmaa B,Battur L,Nymadawa P
EPI_ISL_1805716, EPI_ISL_1805717, EPI_ISL_1805718, EPI_ISL_1805740, EPI_ISL_1805741, EPI_ISL_1805744, EPI_ISL_1805745, EPI_ISL_1805787, EPI_ISL_1805789, EPI_ISL_1805790	National Center for Communicable Diseases (NCCD) National Influenza Center	National Center for Communicable Diseases (NCCD) National Influenza Center	Naranzul Ts,Bayasgalan N,Khishigmunkh Ch,Seiichiro F,Hideka M,Mina N,Shinji W,Ankhbayar S,Tsogzolmaa G,Darmaa B,Battur L,Nymadawa P
EPI_ISL_1821208	Hospital de Base de Bauru Hospital de Base Setima Regiao	Instituto Adolfo Lutz, Interdiciplinary Procedures Center, Strategic Laboratory	Claudio Tavares Sacchi, Claudia Regina Gonçalves, Erica Valessa Ramos Gomes, Karoline Rodrigues Campos, Caio Vinicius Dias Lopes, Leonardo Jose Tadeu de Araujo
EPI_ISL_1823412	Indiana Animal Disease Diagnostic Laboratory	Carpi Laboratory - Purdue University	Jack Dorman, Ilinca I Ciubotariu, Nicole M Perry, Jobin J Kattoor, Lev Gorenstein, Abebe A Fola, G Kenitra Hendrix, Rebecca P Wilkes, Giovanna Carpi
EPI_ISL_1824616	RSUD Bahteramas Kendari	National Institute of Health Research and Development	Hana Apsari Pawestri, Subangkit, Kartika Dewi Puspa, Arie Ardiansyah Nugraha, Hartanti Dian Ikawati, Krisna Pangesti, Nelly Puspandari, Vivi Setiawaty
EPI_ISL_1859356, EPI_ISL_1859367, EPI_ISL_1859368, EPI_ISL_1859376, EPI_ISL_1859378, EPI_ISL_1859382, EPI_ISL_1859384, EPI_ISL_1859385, EPI_ISL_1859401, EPI_ISL_1859406	Kazan Federal University, Academic Consortium	FEDERAL RESEARCH AND CLINICAL CENTER OF PHYSICAL-CHEMICAL MEDICINE OF FEDERAL MEDICAL BIOLOGICAL AGENCY, Academic Consortium	Vladislav Babenko, Ramiz Bakhtyev, Vladimir Baklaushev, Larisa Balykova, Pavel Bashkirov, Anna Blagonravova, Daria Boldyreva, Dmitry Fedorov, Ilshat Gafurov, Raushaniya Gaifullina, Yulia Galeeva, Elena Galova, Vadim Govorun, Elena Ilina, Konstantin Ivanov, Daria Kharlampieva, Polina Khromova, Ksenia Klimina, Konstantin Kolontarev, Nadezhda Kolyshkina, Andrey Koritsky, Vyacheslav Kuropatkin, Vasily Lazarev, Alexander Manolov, Valentin Manuvera, Daria Matyushkina, Ekaterina Moskaleva, Varvara Musarova, Oleg Ogarkov, Elizaveta Orlova, Alexander Pavlenko, Alla Petrova, Natalia Pozhenko, Dmitry Pushkar, Alexander Rumyantsev, Sergey Rumyantsev, Vladimir Rumyantsev, Lyubov Rychkova, Alexander Samoilov, Irina Shirokova, Vyacheslav Sinkov, Svetlana Solovieva, Polina Tikhonova, Galina Trifonova, Alexander Troitsky, Alexander Tulichev, Yuri Udalov, Anna Varizhuk, Alexander Vasiliev, Vladimir Veselovsky, Alexey Volnukhin, Gaukhar Yusubalieva
EPI_ISL_1859410	Federal State Budgetary Educational Institution of Higher Education «Privolzhsky Research Medical University» of the Ministry of Health of the Russian Federation, Academic Consortium	FEDERAL RESEARCH AND CLINICAL CENTER OF PHYSICAL-CHEMICAL MEDICINE OF FEDERAL MEDICAL BIOLOGICAL AGENCY, Academic Consortium	Vladislav Babenko, Ramiz Bakhtyev, Vladimir Baklaushev, Larisa Balykova, Pavel Bashkirov, Anna Blagonravova, Daria Boldyreva, Dmitry Fedorov, Ilshat Gafurov, Raushaniya Gaifullina, Yulia Galeeva, Elena Galova, Vadim Govorun, Elena Ilina, Konstantin Ivanov, Daria Kharlampieva, Polina Khromova, Ksenia Klimina, Konstantin Kolontarev, Nadezhda Kolyshkina, Andrey Koritsky, Vyacheslav Kuropatkin, Vasily Lazarev, Alexander Manolov, Valentin Manuvera, Daria Matyushkina, Ekaterina Moskaleva, Varvara Musarova, Oleg Ogarkov, Elizaveta Orlova, Alexander Pavlenko, Alla Petrova, Natalia Pozhenko, Dmitry Pushkar, Alexander Rumyantsev, Sergey Rumyantsev, Vladimir Rumyantsev, Lyubov Rychkova, Alexander Samoilov, Irina Shirokova, Vyacheslav Sinkov, Svetlana Solovieva, Polina Tikhonova, Galina Trifonova, Alexander Troitsky, Alexander Tulichev, Yuri Udalov, Anna Varizhuk, Alexander Vasiliev, Vladimir Veselovsky, Alexey Volnukhin, Gaukhar Yusubalieva
EPI_ISL_1859424, EPI_ISL_1859427, EPI_ISL_1859433, EPI_ISL_1859475, EPI_ISL_1859481	Kazan Federal University, Academic Consortium	FEDERAL RESEARCH AND CLINICAL CENTER OF PHYSICAL-CHEMICAL MEDICINE OF FEDERAL MEDICAL BIOLOGICAL AGENCY, Academic Consortium	Vladislav Babenko, Ramiz Bakhtyev, Vladimir Baklaushev, Larisa Balykova, Pavel Bashkirov, Anna Blagonravova, Daria Boldyreva, Dmitry Fedorov, Ilshat Gafurov, Raushaniya Gaifullina, Yulia Galeeva, Elena Galova, Vadim Govorun, Elena Ilina, Konstantin Ivanov, Daria Kharlampieva, Polina Khromova, Ksenia Klimina, Konstantin Kolontarev, Nadezhda Kolyshkina, Andrey Koritsky, Vyacheslav Kuropatkin, Vasily Lazarev, Alexander Manolov, Valentin Manuvera, Daria Matyushkina, Ekaterina Moskaleva, Varvara Musarova, Oleg Ogarkov, Elizaveta Orlova, Alexander Pavlenko, Alla Petrova, Natalia Pozhenko, Dmitry Pushkar, Alexander Rumyantsev, Sergey Rumyantsev, Vladimir Rumyantsev, Lyubov Rychkova, Alexander Samoilov, Irina Shirokova, Vyacheslav Sinkov, Svetlana Solovieva, Polina Tikhonova, Galina Trifonova, Alexander Troitsky, Alexander Tulichev, Yuri Udalov, Anna Varizhuk, Alexander Vasiliev, Vladimir Veselovsky, Alexey Volnukhin, Gaukhar Yusubalieva
EPI_ISL_1859498	City Clinical Hospital named after S.I. Spasokukotsky of Moscow Healthcare Department, Academic Consortium	FEDERAL RESEARCH AND CLINICAL CENTER OF PHYSICAL-CHEMICAL MEDICINE OF FEDERAL MEDICAL BIOLOGICAL AGENCY, Academic Consortium	Vladislav Babenko, Ramiz Bakhtyev, Vladimir Baklaushev, Larisa Balykova, Pavel Bashkirov, Anna Blagonravova, Daria Boldyreva, Dmitry Fedorov, Ilshat Gafurov, Raushaniya Gaifullina, Yulia Galeeva, Elena Galova, Vadim Govorun, Elena Ilina, Konstantin Ivanov, Daria Kharlampieva, Polina Khromova, Ksenia Klimina, Konstantin Kolontarev, Nadezhda Kolyshkina, Andrey Koritsky, Vyacheslav Kuropatkin, Vasily Lazarev, Alexander Manolov, Valentin Manuvera, Daria Matyushkina, Ekaterina Moskaleva, Varvara Musarova, Oleg Ogarkov, Elizaveta Orlova, Alexander Pavlenko, Alla Petrova, Natalia Pozhenko, Dmitry Pushkar, Alexander Rumyantsev, Sergey Rumyantsev, Vladimir Rumyantsev, Lyubov Rychkova, Alexander Samoilov, Irina Shirokova, Vyacheslav Sinkov, Svetlana Solovieva, Polina Tikhonova, Galina Trifonova, Alexander Troitsky, Alexander Tulichev, Yuri Udalov, Anna Varizhuk, Alexander Vasiliev, Vladimir Veselovsky, Alexey Volnukhin, Gaukhar Yusubalieva
EPI_ISL_1859513, EPI_ISL_1859530, EPI_ISL_1859547, EPI_ISL_1859562	Kazan Federal University, Academic Consortium	FEDERAL RESEARCH AND CLINICAL CENTER OF PHYSICAL-CHEMICAL MEDICINE OF FEDERAL MEDICAL BIOLOGICAL AGENCY, Academic Consortium	Vladislav Babenko, Ramiz Bakhtyev, Vladimir Baklaushev, Larisa Balykova, Pavel Bashkirov, Anna Blagonravova, Daria Boldyreva, Dmitry Fedorov, Ilshat Gafurov, Raushaniya Gaifullina, Yulia Galeeva, Elena Galova, Vadim Govorun, Elena Ilina, Konstantin Ivanov, Daria Kharlampieva, Polina Khromova, Ksenia Klimina, Konstantin Kolontarev, Nadezhda Kolyshkina, Andrey Koritsky, Vyacheslav Kuropatkin, Vasily Lazarev, Alexander Manolov, Valentin Manuvera, Daria Matyushkina, Ekaterina Moskaleva, Varvara Musarova, Oleg Ogarkov, Elizaveta Orlova, Alexander Pavlenko, Alla Petrova, Natalia Pozhenko, Dmitry Pushkar, Alexander Rumyantsev, Sergey Rumyantsev, Vladimir Rumyantsev, Lyubov Rychkova, Alexander Samoilov, Irina Shirokova, Vyacheslav Sinkov, Svetlana Solovieva, Polina Tikhonova, Galina Trifonova, Alexander Troitsky, Alexander Tulichev, Yuri Udalov, Anna Varizhuk, Alexander Vasiliev, Vladimir Veselovsky, Alexey Volnukhin, Gaukhar Yusubalieva
EPI_ISL_1859572	City Clinical Hospital named after S.I. Spasokukotsky of Moscow Healthcare Department, Academic Consortium	FEDERAL RESEARCH AND CLINICAL CENTER OF PHYSICAL-CHEMICAL MEDICINE OF FEDERAL MEDICAL BIOLOGICAL AGENCY, Academic Consortium	Vladislav Babenko, Ramiz Bakhtyev, Vladimir Baklaushev, Larisa Balykova, Pavel Bashkirov, Anna Blagonravova, Daria Boldyreva, Dmitry Fedorov, Ilshat Gafurov, Raushaniya Gaifullina, Yulia Galeeva, Elena Galova, Vadim Govorun, Elena Ilina, Konstantin Ivanov, Daria Kharlampieva, Polina Khromova, Ksenia Klimina, Konstantin Kolontarev, Nadezhda Kolyshkina, Andrey Koritsky, Vyacheslav Kuropatkin, Vasily Lazarev, Alexander Manolov, Valentin Manuvera, Daria Matyushkina, Ekaterina Moskaleva, Varvara Musarova, Oleg Ogarkov, Elizaveta Orlova, Alexander Pavlenko, Alla Petrova, Natalia Pozhenko, Dmitry Pushkar, Alexander Rumyantsev, Sergey Rumyantsev, Vladimir Rumyantsev, Lyubov Rychkova, Alexander Samoilov, Irina Shirokova, Vyacheslav Sinkov, Svetlana Solovieva, Polina Tikhonova, Galina Trifonova, Alexander Troitsky, Alexander Tulichev, Yuri Udalov, Anna Varizhuk, Alexander Vasiliev, Vladimir Veselovsky, Alexey Volnukhin, Gaukhar Yusubalieva
EPI_ISL_1859600, EPI_ISL_1859602, EPI_ISL_1859626	Kazan Federal University, Academic Consortium	FEDERAL RESEARCH AND CLINICAL CENTER OF PHYSICAL-CHEMICAL MEDICINE OF FEDERAL MEDICAL BIOLOGICAL AGENCY, Academic Consortium	Vladislav Babenko, Ramiz Bakhtyev, Vladimir Baklaushev, Larisa Balykova, Pavel Bashkirov, Anna Blagonravova, Daria Boldyreva, Dmitry Fedorov, Ilshat Gafurov, Raushaniya Gaifullina, Yulia Galeeva, Elena Galova, Vadim Govorun, Elena Ilina, Konstantin Ivanov, Daria Kharlampieva, Polina Khromova, Ksenia Klimina, Konstantin Kolontarev, Nadezhda Kolyshkina, Andrey Koritsky, Vyacheslav Kuropatkin, Vasily Lazarev, Alexander Manolov, Valentin Manuvera, Daria Matyushkina, Ekaterina Moskaleva, Varvara Musarova, Oleg Ogarkov, Elizaveta Orlova, Alexander Pavlenko, Alla Petrova, Natalia Pozhenko, Dmitry Pushkar, Alexander Rumyantsev, Sergey Rumyantsev, Vladimir Rumyantsev, Lyubov Rychkova, Alexander Samoilov, Irina Shirokova, Vyacheslav Sinkov, Svetlana Solovieva, Polina Tikhonova, Galina Trifonova, Alexander Troitsky, Alexander Tulichev, Yuri Udalov, Anna Varizhuk, Alexander Vasiliev, Vladimir Veselovsky, Alexey Volnukhin, Gaukhar Yusubalieva
EPI_ISL_1859654	FEDERAL RESEARCH AND CLINICAL CENTER OF PHYSICAL-CHEMICAL MEDICINE OF FEDERAL MEDICAL BIOLOGICAL AGENCY, Academic Consortium	FEDERAL RESEARCH AND CLINICAL CENTER OF PHYSICAL-CHEMICAL MEDICINE OF FEDERAL MEDICAL BIOLOGICAL AGENCY, Academic Consortium	Vladislav Babenko, Ramiz Bakhtyev, Vladimir Baklaushev, Larisa Balykova, Pavel Bashkirov, Anna Blagonravova, Daria Boldyreva, Dmitry Fedorov, Ilshat Gafurov, Raushaniya Gaifullina, Yulia Galeeva, Elena Galova, Vadim Govorun, Elena Ilina, Konstantin Ivanov, Daria Kharlampieva, Polina Khromova, Ksenia Klimina, Konstantin Kolontarev, Nadezhda Kolyshkina, Andrey Koritsky, Vyacheslav Kuropatkin, Vasily Lazarev, Alexander Manolov, Valentin Manuvera, Daria Matyushkina, Ekaterina Moskaleva, Varvara Musarova, Oleg Ogarkov, Elizaveta Orlova, Alexander Pavlenko, Alla Petrova, Natalia Pozhenko, Dmitry Pushkar, Alexander Rumyantsev, Sergey Rumyantsev, Vladimir Rumyantsev, Lyubov Rychkova, Alexander Samoilov, Irina Shirokova, Vyacheslav Sinkov, Svetlana Solovieva, Polina Tikhonova, Galina Trifonova, Alexander Troitsky, Alexander Tulichev, Yuri Udalov, Anna Varizhuk, Alexander Vasiliev, Vladimir Veselovsky, Alexey Volnukhin, Gaukhar Yusubalieva
EPI_ISL_1859673, EPI_ISL_1859675, EPI_ISL_1859677, EPI_ISL_1859681, EPI_ISL_1859683, EPI_ISL_1859687,	Kazan Federal University, Academic Consortium	FEDERAL RESEARCH AND CLINICAL CENTER OF PHYSICAL-CHEMICAL MEDICINE OF FEDERAL MEDICAL BIOLOGICAL AGENCY, Academic Consortium	Vladislav Babenko, Ramiz Bakhtyev, Vladimir Baklaushev, Larisa Balykova, Pavel Bashkirov, Anna Blagonravova, Daria Boldyreva, Dmitry Fedorov, Ilshat Gafurov, Raushaniya Gaifullina, Yulia Galeeva, Elena Galova, Vadim Govorun, Elena Ilina, Konstantin Ivanov, Daria Kharlampieva, Polina Khromova, Ksenia Klimina, Konstantin Kolontarev, Nadezhda Kolyshkina, Andrey Koritsky, Vyacheslav Kuropatkin, Vasily Lazarev, Alexander Manolov, Valentin Manuvera, Daria

EPI_ISL_1859689, EPI_ISL_1859696, EPI_ISL_1859721			Matyushkina, Ekaterina Moskaleva, Varvara Musarova, Oleg Ogarkov, Elizaveta Orlova, Alexander Pavlenko, Alla Petrova, Natalia Pozhenko, Dmitry Pushkar, Alexander Rumyantsev, Sergey Rumyantsev, Vladimir Rumyantsev, Lyubov Rychkova, Alexander Samoilov, Irina Shirokova, Vyacheslav Sinkov, Svetlana Solovieva, Polina Tikhonova, Galina Trifonova, Alexander Troitsky, Alexander Tulichev, Yuri Udalov, Anna Varizhuk, Alexander Vasiliev, Vladimir Veselovsky, Alexey Volnukhin, Gaukhar Yusubalieva
EPI_ISL_1859727	City Clinical Hospital named after S.I. Spasokukotsky of Moscow Healthcare Department, Academic Consortium	FEDERAL RESEARCH AND CLINICAL CENTER OF PHYSICAL-CHEMICAL MEDICINE OF FEDERAL MEDICAL BIOLOGICAL AGENCY, Academic Consortium	Vladislav Babenko, Ramiz Bakhtyev, Vladimir Baklaushev, Larisa Balykova, Pavel Bashkirov, Anna Blagonravova, Daria Boldyreva, Dmitry Fedorov, Ilshat Gafurov, Raushaniya Gaifullina, Yulia Galeeva, Elena Galova, Vadim Govorun, Elena Ilina, Konstantin Ivanov, Daria Kharlampieva, Polina Khromova, Ksenia Klimina, Konstantin Kolontarev, Nadezhda Kolyshkina, Andrey Koritsky, Vyacheslav Kuropatkin, Vasily Lazarev, Alexander Manolov, Valentin Manuvera, Daria Matyushkina, Ekaterina Moskaleva, Varvara Musarova, Oleg Ogarkov, Elizaveta Orlova, Alexander Pavlenko, Alla Petrova, Natalia Pozhenko, Dmitry Pushkar, Alexander Rumyantsev, Sergey Rumyantsev, Vladimir Rumyantsev, Lyubov Rychkova, Alexander Samoilov, Irina Shirokova, Vyacheslav Sinkov, Svetlana Solovieva, Polina Tikhonova, Galina Trifonova, Alexander Troitsky, Alexander Tulichev, Yuri Udalov, Anna Varizhuk, Alexander Vasiliev, Vladimir Veselovsky, Alexey Volnukhin, Gaukhar Yusubalieva
EPI_ISL_1859729, EPI_ISL_1859733, EPI_ISL_1859738, EPI_ISL_1859746, EPI_ISL_1859764	Kazan Federal University, Academic Consortium	FEDERAL RESEARCH AND CLINICAL CENTER OF PHYSICAL-CHEMICAL MEDICINE OF FEDERAL MEDICAL BIOLOGICAL AGENCY, Academic Consortium	Vladislav Babenko, Ramiz Bakhtyev, Vladimir Baklaushev, Larisa Balykova, Pavel Bashkirov, Anna Blagonravova, Daria Boldyreva, Dmitry Fedorov, Ilshat Gafurov, Raushaniya Gaifullina, Yulia Galeeva, Elena Galova, Vadim Govorun, Elena Ilina, Konstantin Ivanov, Daria Kharlampieva, Polina Khromova, Ksenia Klimina, Konstantin Kolontarev, Nadezhda Kolyshkina, Andrey Koritsky, Vyacheslav Kuropatkin, Vasily Lazarev, Alexander Manolov, Valentin Manuvera, Daria Matyushkina, Ekaterina Moskaleva, Varvara Musarova, Oleg Ogarkov, Elizaveta Orlova, Alexander Pavlenko, Alla Petrova, Natalia Pozhenko, Dmitry Pushkar, Alexander Rumyantsev, Sergey Rumyantsev, Vladimir Rumyantsev, Lyubov Rychkova, Alexander Samoilov, Irina Shirokova, Vyacheslav Sinkov, Svetlana Solovieva, Polina Tikhonova, Galina Trifonova, Alexander Troitsky, Alexander Tulichev, Yuri Udalov, Anna Varizhuk, Alexander Vasiliev, Vladimir Veselovsky, Alexey Volnukhin, Gaukhar Yusubalieva
EPI_ISL_1893362, EPI_ISL_1893366, EPI_ISL_1893368, EPI_ISL_1893370, EPI_ISL_1893372, EPI_ISL_1893374, EPI_ISL_1893375, EPI_ISL_1893376, EPI_ISL_1893377, EPI_ISL_1893382, EPI_ISL_1893437, EPI_ISL_1893439, EPI_ISL_1893442, EPI_ISL_1893449, EPI_ISL_1893451, EPI_ISL_1893453, EPI_ISL_1893455, EPI_ISL_1893460, EPI_ISL_1893462, EPI_ISL_1893469, EPI_ISL_1893473, EPI_ISL_1893475, EPI_ISL_1893478, EPI_ISL_1893480, EPI_ISL_1893487, EPI_ISL_1893489, EPI_ISL_1893490, EPI_ISL_1893493, EPI_ISL_1893495, EPI_ISL_1893497, EPI_ISL_1893499, EPI_ISL_1893507, EPI_ISL_1893508, EPI_ISL_1893531, EPI_ISL_1893533, EPI_ISL_1893534, EPI_ISL_1893537, EPI_ISL_1893539, EPI_ISL_1893541, EPI_ISL_1893543, EPI_ISL_1893545, EPI_ISL_1893547, EPI_ISL_1893549, EPI_ISL_1893552, EPI_ISL_1893553, EPI_ISL_1893555, EPI_ISL_1893557, EPI_ISL_1893592, EPI_ISL_1893599, EPI_ISL_1893602, EPI_ISL_1893603, EPI_ISL_1893606, EPI_ISL_1893608, EPI_ISL_1893610			
see above	IPK	Respiratory Infections Laboratory	Lissette Perez, Yahisel Tejero, Yanet Pintos, Daylin Mederos, Odalys Valdes, Mirtha Elisa Aguado, Liena Ponce
EPI_ISL_1910399	Centre Hospitalier Universitaire Clermont-Ferrand	CHU Clermont-Ferrand, service de virologie	Bisseux Maxime, Mirand Audrey, Combes Patricia, Henquell Cécile
EPI_ISL_1910400	Centre Jean Perrin	CHU Clermont-Ferrand, service de virologie	Bisseux Maxime, Mirand Audrey, Combes Patricia, Henquell Cécile
EPI_ISL_1910401, EPI_ISL_1910402	Centre Hospitalier Universitaire Clermont-Ferrand	CHU Clermont-Ferrand, service de virologie	Bisseux Maxime, Mirand Audrey, Combes Patricia, Henquell Cécile
EPI_ISL_1910403, EPI_ISL_1910404, EPI_ISL_1910405, EPI_ISL_1910406, EPI_ISL_1910407, EPI_ISL_1910408, EPI_ISL_1910409, EPI_ISL_1910410, EPI_ISL_1910411, EPI_ISL_1910412, EPI_ISL_1910413, EPI_ISL_1910414, EPI_ISL_1910415			
see above	Centre Hospitalier Billom	CHU Clermont-Ferrand, service de virologie	Bisseux Maxime, Mirand Audrey, Combes Patricia, Henquell Cécile
EPI_ISL_1910416	Centre Hospitalier Paul Ardier	CHU Clermont-Ferrand, service de virologie	Bisseux Maxime, Mirand Audrey, Combes Patricia, Henquell Cécile
EPI_ISL_1910417	HOPITAL DE VICHY	CHU Clermont-Ferrand, service de virologie	Bisseux Maxime, Mirand Audrey, Combes Patricia, Henquell Cécile
EPI_ISL_1910418	Centre Hospitalier Universitaire Clermont-Ferrand	CHU Clermont-Ferrand, service de virologie	Bisseux Maxime, Mirand Audrey, Combes Patricia, Henquell Cécile
EPI_ISL_1910419, EPI_ISL_1910420	Centre Hospitalier Billom	CHU Clermont-Ferrand, service de virologie	Bisseux Maxime, Mirand Audrey, Combes Patricia, Henquell Cécile
EPI_ISL_1910421	Centre Hospitalier Universitaire Clermont-Ferrand	CHU Clermont-Ferrand, service de virologie	Bisseux Maxime, Mirand Audrey, Combes Patricia, Henquell Cécile
EPI_ISL_1910422	Centre Hospitalier Billom	CHU Clermont-Ferrand, service de virologie	Bisseux Maxime, Mirand Audrey, Combes Patricia, Henquell Cécile
EPI_ISL_1910423	Centre Hospitalier Universitaire Clermont-Ferrand	CHU Clermont-Ferrand, service de virologie	Bisseux Maxime, Mirand Audrey, Combes Patricia, Henquell Cécile
EPI_ISL_1910424, EPI_ISL_1910425	Centre Hospitalier Billom	CHU Clermont-Ferrand, service de virologie	Bisseux Maxime, Mirand Audrey, Combes Patricia, Henquell Cécile
EPI_ISL_1910426, EPI_ISL_1910427, EPI_ISL_1910428, EPI_ISL_1910429, EPI_ISL_1910430, EPI_ISL_1910431, EPI_ISL_1910432, EPI_ISL_1910433, EPI_ISL_1910434, EPI_ISL_1910435	Centre Hospitalier Universitaire Clermont-Ferrand	CHU Clermont-Ferrand, service de virologie	Bisseux Maxime, Mirand Audrey, Combes Patricia, Henquell Cécile
EPI_ISL_1910436	Centre Hospitalier Paul Ardier	CHU Clermont-Ferrand, service de virologie	Bisseux Maxime, Mirand Audrey, Combes Patricia, Henquell Cécile
EPI_ISL_1910437, EPI_ISL_1910438, EPI_ISL_1910439, EPI_ISL_1910440, EPI_ISL_1910441, EPI_ISL_1910442, EPI_ISL_1910443, EPI_ISL_1910446, EPI_ISL_1910447, EPI_ISL_1910448, EPI_ISL_1910449, EPI_ISL_1910450			
see above	Centre Hospitalier Universitaire Clermont-Ferrand	CHU Clermont-Ferrand, service de virologie	Bisseux Maxime, Mirand Audrey, Combes Patricia, Henquell Cécile
EPI_ISL_1911013, EPI_ISL_1911014, EPI_ISL_1911015, EPI_ISL_1911016, EPI_ISL_1911017, EPI_ISL_1911018, EPI_ISL_1911019, EPI_ISL_1911020, EPI_ISL_1911021, EPI_ISL_1911022, EPI_ISL_1911023, EPI_ISL_1911024, EPI_ISL_1911025, EPI_ISL_1911026, EPI_ISL_1911027, EPI_ISL_1911028, EPI_ISL_1911029, EPI_ISL_1911030, EPI_ISL_1911031, EPI_ISL_1911032, EPI_ISL_1911034, EPI_ISL_1911035, EPI_ISL_1911036, EPI_ISL_1911037, EPI_ISL_1911038, EPI_ISL_1911039, EPI_ISL_1911040, EPI_ISL_1911041, EPI_ISL_1911042, EPI_ISL_1911043, EPI_ISL_1911044, EPI_ISL_1911045, EPI_ISL_1911047, EPI_ISL_1911048, EPI_ISL_1911049, EPI_ISL_1911050, EPI_ISL_1911051, EPI_ISL_1911052, EPI_ISL_1911053, EPI_ISL_1911054, EPI_ISL_1911055, EPI_ISL_1911056, EPI_ISL_1911057, EPI_ISL_1911058, EPI_ISL_1911059, EPI_ISL_1911060, EPI_ISL_1911061, EPI_ISL_1911062, EPI_ISL_1911063, EPI_ISL_1911064, EPI_ISL_1911065, EPI_ISL_1911066, EPI_ISL_1911067, EPI_ISL_1911068, EPI_ISL_1911069, EPI_ISL_1911070, EPI_ISL_1911071, EPI_ISL_1911073, EPI_ISL_1911074, EPI_ISL_1911075, EPI_ISL_1911076, EPI_ISL_1911077, EPI_ISL_1911078, EPI_ISL_1911079, EPI_ISL_1911080, EPI_ISL_1911081			
see above	The Ohio State University Applied Microbiology Services Laboratory	The Ohio State University Applied Microbiology Services Laboratory	Seth A. Faith PhD
EPI_ISL_1915141	Hospital	National Reference Center for Viruses of Respiratory Infections, Institut Pasteur, Paris	Marion Barbet, Sylvie Behillil, Méline Bizard, Angela Brisebarre, Camille Capel, Vincent Enouf, Louise Lefrançois, Frédéric Lemoine, Christophe Malabat, Corinne Maufrais, Emmanuelle Permal, Etienne Simon-Lorière, Maud Vanpeene, Sylvie Van der Werf ,Farida Moreau Benaoudia
EPI_ISL_1915189	Labo Analyses Med	National Reference Center for Viruses of Respiratory Infections, Institut Pasteur, Paris	Marion Barbet, Sylvie Behillil, Méline Bizard, Angela Brisebarre, Camille Capel, Vincent Enouf, Louise Lefrançois, Frédéric Lemoine, Christophe Malabat, Corinne Maufrais, Emmanuelle Permal, Etienne Simon-Lorière, Maud Vanpeene, Sylvie Van der Werf ,Anne Grattard
EPI_ISL_1915604	The Ohio State University Applied Microbiology Services Laboratory	The Ohio State University Applied Microbiology Services Laboratory	Seth A. Faith PhD
EPI_ISL_1916277, EPI_ISL_1916278, EPI_ISL_1916280, EPI_ISL_1916281, EPI_ISL_1916290, EPI_ISL_1916293, EPI_ISL_1916296, EPI_ISL_1916305, EPI_ISL_1916306	CSIR-National Environmental Engineering Research Institute	CSIR-Centre for Cellular and Molecular Biology - INSACOG	Sofia Banu, Onkar Kulkarni, Lamuk Zaveri, Ara Sreenivas, Shreekant Verma, Amareshwar Vodapalli , Viswagithe S L, B Himasri, Sharath Chandra Thota, Karthik Bharadwaj Tallapaka, Krishna Khairnar, Rakesh K Mishra,Divya Tej Sowpati
EPI_ISL_1916312, EPI_ISL_1916313, EPI_ISL_1916370, EPI_ISL_1916371, EPI_ISL_1916374, EPI_ISL_1916382, EPI_ISL_1916383, EPI_ISL_1916387, EPI_ISL_1916395	CSIR-National Environmental Engineering Research Institute	CSIR-Centre for Cellular and Molecular Biology - INSACOG	Lamuk Zaveri, Sofia Banu, Onkar Kulkarni, Ara Sreenivas, Shreekant Verma, Amareshwar Vodapalli , Viswagithe S L, B Himasri, Sharath Chandra Thota, Karthik Bharadwaj Tallapaka, Krishna Khairnar, Rakesh K Mishra,Divya Tej Sowpati
EPI_ISL_1916396, EPI_ISL_1916399	CSIR-National Environmental Engineering Research Institute	CSIR-Centre for Cellular and Molecular Biology - INSACOG	Onkar Kulkarni, Lamuk Zaveri, Ara Sreenivas, Sofia Banu, Shreekant Verma, Amareshwar Vodapalli , Viswagithe S L, B Himasri, Sharath Chandra Thota, Karthik Bharadwaj Tallapaka, Krishna Khairnar, Rakesh K Mishra,Divya Tej Sowpati
EPI_ISL_1916491, EPI_ISL_1916492, EPI_ISL_1916493, EPI_ISL_1916494, EPI_ISL_1916495, EPI_ISL_1916496, EPI_ISL_1916497, EPI_ISL_1916498	CSIR-National Environmental Engineering Research Institute	CSIR-Centre for Cellular and Molecular Biology - INSACOG	Ara Sreenivas, Onkar Kulkarni, Lamuk Zaveri, Sofia Banu, Shreekant Verma, Amareshwar Vodapalli , Viswagithe S L, B Himasri, Sharath Chandra Thota, Karthik Bharadwaj Tallapaka, Krishna Khairnar, Rakesh K Mishra,Divya Tej Sowpati

EPI_ISL_1965722	3. Medizinische Abteilung, Hanusch Krankenhaus	Bergthaler laboratory, CeMM Research Center for Molecular Medicine of the Austrian Academy of Sciences	Lukas Endler, Anna Schedl, Fabian Amman, Petr Triska, Thomas Penz, Benedikt Agerer, Maelle Le Moing, Michael Schuster, Bekir Erguner, Jan Laine, Martin Senekowitsch, Christoph Bock, Andreas Bergthaler
EPI_ISL_1970625, EPI_ISL_1970626	CSIR-National Environmental Engineering Research Institute	CSIR-Centre for Cellular and Molecular Biology - INSACOG	Sofia Banu, Onkar Kulkarni, Lamuk Zaveri, Ara Sreenivas, Shreekant Verma, Amareshwar Vodapalli , Viswagithe S L, B Himasri, Sharath Chandra Thota, Karthik Bharadwaj Tallapaka, Krishna Khairnar, Rakesh K Mishra,Divya Tej Sowpati
EPI_ISL_2001116	VRDL, Goverment Medical College (GMC), Bhavnagar	Gujarat Biotechnology Research Centre	Dinesh Kumar,Twinkle Soni,Sonal Sharma ,Zuber Saiyed,Ramesh Pandit,Janvi Raval,Zarna Patel,Nitin Savaliya,Kairavi Jwalantbhai Desai,Umang Mishra,Nitesh Shah,Chaitanya Joshi,Madhvi Joshi
EPI_ISL_2001119	VRDL, Goverment Medical College (GMC), Bhavnagar	Gujarat Biotechnology Research Centre	Zuber Saiyed,Ramesh Pandit,Janvi Raval,Zarna Patel,Nitin Savaliya,Dinesh Kumar,Twinkle Soni,Sonal Sharma ,Kairavi Jwalantbhai Desai,Umang Mishra,Nitesh Shah,Chaitanya Joshi,Madhvi Joshi
EPI_ISL_2001120	VRDL, Goverment Medical College (GMC), Bhavnagar	Gujarat Biotechnology Research Centre	Ramesh Pandit,Janvi Raval,Zarna Patel,Nitin Savaliya,Dinesh Kumar,Twinkle Soni,Sonal Sharma ,Zuber Saiyed,Kairavi Jwalantbhai Desai,Umang Mishra,Nitesh Shah,Chaitanya Joshi,Madhvi Joshi
EPI_ISL_2001149	VRDL, Goverment Medical College (GMC), Bhavnagar	Gujarat Biotechnology Research Centre	Zuber Saiyed,Ramesh Pandit,Janvi Raval,Zarna Patel,Nitin Savaliya,Dinesh Kumar,Twinkle Soni,Sonal Sharma ,Kairavi Jwalantbhai Desai,Umang Mishra,Nitesh Shah,Chaitanya Joshi,Madhvi Joshi
EPI_ISL_2001150	VRDL, Goverment Medical College (GMC), Bhavnagar	Gujarat Biotechnology Research Centre	Ramesh Pandit,Janvi Raval,Zarna Patel,Nitin Savaliya,Dinesh Kumar,Twinkle Soni,Sonal Sharma ,Zuber Saiyed,Kairavi Jwalantbhai Desai,Umang Mishra,Nitesh Shah,Chaitanya Joshi,Madhvi Joshi
EPI_ISL_2003113	Hospital Municipal Dr Mario Gatti Campinas	Instituto Adolfo Lutz, Interdiciplinary Procedures Center, Strategic Laboratory	Claudio Tavares Sacchi, Claudia Regina Gonçalves, Erica Valessa Ramos Gomes, Karoline Rodrigues Campos, Caio Vinicius Dias Lopes, Leonardo Jose Tadeu de Araujo
EPI_ISL_2003153, EPI_ISL_2003154, EPI_ISL_2003155, EPI_ISL_2003156, EPI_ISL_2003157, EPI_ISL_2003158, EPI_ISL_2003159, EPI_ISL_2003160, EPI_ISL_2003161	Instituto Adolfo Lutz Central	Instituto Adolfo Lutz, Interdiciplinary Procedures Center, Strategic Laboratory	Claudio Tavares Sacchi, Claudia Regina Gonçalves, Erica Valessa Ramos Gomes, Karoline Rodrigues Campos, Caio Vinicius Dias Lopes, Leonardo Jose Tadeu de Araujo
EPI_ISL_2009174, EPI_ISL_2009175, EPI_ISL_2009178, EPI_ISL_2009179, EPI_ISL_2009180, EPI_ISL_2009181, EPI_ISL_2009182, EPI_ISL_2009183, EPI_ISL_2009184, EPI_ISL_2009185	MSK Microbiology Lab	MSK Microbiology Lab	Esther Babady, Tracy McMillen, Krupa Jani
EPI_ISL_2009232, EPI_ISL_2009233, EPI_ISL_2009234, EPI_ISL_2009305, EPI_ISL_2009530, EPI_ISL_2009595	National Public Health Laboratory Malaysia	National Public Health Laboratory Malaysia	Noorliza MN, JL Tan, Chong CK, Norhayati R, Yukie C, Norazimah T, Rehan SAB, Selvanesan S, Hannah PYP, NurulAina MCA, W.NurAfiza WMA Zirwatul AA, Hani MH, Nurulsyahida I, Aziyati O, Ushananthiny R, Kamal HKZ, Mohd AY, YF Ngeow
EPI_ISL_2013043	RS Sentra Medika Cikarang	Eijkman Institute for Molecular Biology, National Agency for Research and Innovation	Edison Johar, Frilasita A Yudhaputri, Muhammad Rezki Rasyak, Willy Agustine, Hidayat Trimarsanto, Lydia V. Panggalo, Iskandar Adnan, Sukma Oktavianthi, Safarina G Malik, Khin Saw Myint, Amin Soebandrio
EPI_ISL_2013044	RS Sentra Medika Cikarang	Eijkman Institute for Molecular Biology, National Agency for Research and Innovation	Willy Agustine, Hidayat Trimarsanto, Lydia V. Panggalo, Iskandar Adnan, Sukma Oktavianthi, Edison Johar, Frilasita A Yudhaputri, Muhammad Rezki Rasyak, Safarina G Malik, Khin Saw Myint, Amin Soebandrio
EPI_ISL_2013045	RS Mitra Keluarga Cikarang	Eijkman Institute for Molecular Biology, National Agency for Research and Innovation	Edison Johar, Frilasita A Yudhaputri, Muhammad Rezki Rasyak, Willy Agustine, Hidayat Trimarsanto, Lydia V. Panggalo, Iskandar Adnan, Sukma Oktavianthi, Safarina G Malik, Khin Saw Myint, Amin Soebandrio
EPI_ISL_2013046	RSUD Cileungsi	Eijkman Institute for Molecular Biology, National Agency for Research and Innovation	Lydia V. Panggalo, Iskandar Adnan, Sukma Oktavianthi, Edison Johar, Frilasita A Yudhaputri, Muhammad Rezki Rasyak, Willy Agustine, Hidayat Trimarsanto, Safarina G Malik, Khin Saw Myint, Amin Soebandrio
EPI_ISL_2013047	RS Mitra Keluarga Kelapa Gading	Eijkman Institute for Molecular Biology, National Agency for Research and Innovation	Hidayat Trimarsanto, Lydia V. Panggalo, Iskandar Adnan, Sukma Oktavianthi, Edison Johar, Frilasita A Yudhaputri, Muhammad Rezki Rasyak, Willy Agustine, Safarina G Malik, Khin Saw Myint, Amin Soebandrio
EPI_ISL_2013326	IU-Cerrahpasa, Cerrahpasa School of Medicine, COVID-19 Lab	IU-Cerrahpasa, Cerrahpasa School of Medicine, COVID-19 Lab	Mert Kuskucu, Yesim Tuyji Tok, Kenan Midilli
EPI_ISL_2013509	RS Harapan Bunda	Eijkman Institute for Molecular Biology, National Agency for Research and Innovation	Lydia V. Panggalo, Iskandar Adnan, Sukma Oktavianthi, Edison Johar, Frilasita A Yudhaputri, Muhammad Rezki Rasyak, Willy Agustine, Hidayat Trimarsanto, Safarina G Malik, Khin Saw Myint, Amin Soebandrio
EPI_ISL_2015096, EPI_ISL_2015097, EPI_ISL_2015098, EPI_ISL_2015099, EPI_ISL_2015100, EPI_ISL_2015103, EPI_ISL_2015104, EPI_ISL_2015116, EPI_ISL_2015117, EPI_ISL_2015121, EPI_ISL_2015122, EPI_ISL_2015125, EPI_ISL_2015139, EPI_ISL_2015140, EPI_ISL_2015142, EPI_ISL_2015143			
see above	IU-Cerrahpasa, Cerrahpasa School of Medicine, COVID-19 Lab	IU-Cerrahpasa, Cerrahpasa School of Medicine, COVID-19 Lab	Mert Kuskucu, Yesim Tuyji Tok, Kenan Midilli
EPI_ISL_2020241	Middlemore Hospital	Institute of Environmental Science and Research (ESR) WHO National Influenza Centre	Nikki Freed, Paula scholes, Susan Lin, Xiaoyun Ren, Matt Storey, Muhammad Faisal, Jing Wang, Hermes Perez, Anja Werno, Antje van der Linden, Arlo Upton, Chris Mansell, David Hammer, Dragana Drinkovic, Gary McAuliffe, Hana Sofia Andersson, James Ussher, Jill Sherwood, Josh Freeman, Julia Howard, Juliet Elvy, Mary DeAlmeida, Matt Blakiston, Matthew Rogers, Max Bloomfield, Michael Addidle, Michelle Balm, Sally Roberts, Sarah Jefferies, Sharmini Muttaiyah, Susan Morpeth, Susan Taylor, Timothy Blackmore, Vani Sathyendran, Veronica Playle, Virginia Hope, Erasmus Smit, Lauren Jelly, Joep de Ligt, Olin Silander
EPI_ISL_2029607	Hospital	National Reference Center for Viruses of Respiratory Infections, Institut Pasteur, Paris	Marion Barbet, Sylvie Behillil, Méline Bizard, Angela Brisebarre, Camille Capel, Vincent Enouf, Louise Lefrançois, Frédéric Lemoine, Christophe Malabat, Corinne Maufrais, Amaury Vaysse, Etienne Simon-Lorière, Maud Vanpeene, Sylvie Van der Werf ,CéLine Bressollette
EPI_ISL_2029618, EPI_ISL_2029624	Labo Analyses Med	National Reference Center for Viruses of Respiratory Infections, Institut Pasteur, Paris	Marion Barbet, Sylvie Behillil, Méline Bizard, Angela Brisebarre, Camille Capel, Vincent Enouf, Louise Lefrançois, Frédéric Lemoine, Christophe Malabat, Corinne Maufrais, Etienne Simon-Lorière, Maud Vanpeene, Sylvie Van der Werf ,Jean-Christophe Denis
EPI_ISL_2029723	Labo Analyses Med	National Reference Center for Viruses of Respiratory Infections, Institut Pasteur, Paris	Marion Barbet, Sylvie Behillil, Méline Bizard, Angela Brisebarre, Camille Capel, Vincent Enouf, Louise Lefrançois, Frédéric Lemoine, Christophe Malabat, Corinne Maufrais, Etienne Simon-Lorière, Maud Vanpeene, Sylvie Van der Werf
EPI_ISL_2029793	Labo Analyses Med	National Reference Center for Viruses of Respiratory Infections, Institut Pasteur, Paris	Marion Barbet, Sylvie Behillil, Méline Bizard, Angela Brisebarre, Camille Capel, Vincent Enouf, Louise Lefrançois, Frédéric Lemoine, Christophe Malabat, Corinne Maufrais, Etienne Simon-Lorière, Maud Vanpeene, Sylvie Van der Werf ,Jean-Christophe Denis
EPI_ISL_2029796	Labo Analyses Med	National Reference Center for Viruses of Respiratory Infections, Institut Pasteur, Paris	Marion Barbet, Sylvie Behillil, Méline Bizard, Angela Brisebarre, Camille Capel, Vincent Enouf, Louise Lefrançois, Frédéric Lemoine, Christophe Malabat, Corinne Maufrais, Emmanuelle Permal, Etienne Simon-Lorière, Maud Vanpeene, Sylvie Van der Werf ,StéPhane Romand
EPI_ISL_2029862	Labo Analyses Med	National Reference Center for Viruses of Respiratory Infections, Institut Pasteur, Paris	Marion Barbet, Sylvie Behillil, Méline Bizard, Angela Brisebarre, Camille Capel, Vincent Enouf, Louise Lefrançois, Frédéric Lemoine, Christophe Malabat, Corinne Maufrais, Etienne Simon-Lorière, Maud Vanpeene, Sylvie Van der Werf ,Jean-Christophe Denis
EPI_ISL_2029903	Labo Analyses Med	National Reference Center for Viruses of Respiratory Infections, Institut Pasteur, Paris	Marion Barbet, Sylvie Behillil, Méline Bizard, Angela Brisebarre, Camille Capel, Vincent Enouf, Louise Lefrançois, Frédéric Lemoine, Christophe Malabat, Corinne Maufrais, Emmanuelle Permal, Etienne Simon-Lorière, Maud Vanpeene, Sylvie Van der Werf ,StéPhane Romand
EPI_ISL_2029974	Labo Analyses Med	National Reference Center for Viruses of Respiratory Infections, Institut Pasteur, Paris	Marion Barbet, Sylvie Behillil, Méline Bizard, Angela Brisebarre, Camille Capel, Vincent Enouf, Louise Lefrançois, Frédéric Lemoine, Christophe Malabat, Corinne Maufrais, Etienne Simon-Lorière, Maud Vanpeene, Sylvie Van der Werf ,Jean-Christophe Denis
EPI_ISL_2030109, EPI_ISL_2030171	Hospital	National Reference Center for Viruses of Respiratory Infections, Institut Pasteur, Paris	Marion Barbet, Sylvie Behillil, Méline Bizard, Angela Brisebarre, Camille Capel, Vincent Enouf, Louise Lefrançois, Frédéric Lemoine, Christophe Malabat, Corinne Maufrais, Etienne Simon-Lorière, Maud Vanpeene, Sylvie Van der Werf ,Ali Si-Mohammed
EPI_ISL_2081877, EPI_ISL_2081879, EPI_ISL_2081880, EPI_ISL_2081881, EPI_ISL_2081882, EPI_ISL_2081883, EPI_ISL_2081884, EPI_ISL_2081885, EPI_ISL_2081888, EPI_ISL_2081889, EPI_ISL_2081890, EPI_ISL_2081891, EPI_ISL_2081892, EPI_ISL_2081893, EPI_ISL_2081894, EPI_ISL_2081895, EPI_ISL_2081896, EPI_ISL_2081897, EPI_ISL_2081898, EPI_ISL_2081899, EPI_ISL_2081900, EPI_ISL_2081901, EPI_ISL_2081903, EPI_ISL_2081904, EPI_ISL_2081906, EPI_ISL_2081907, EPI_ISL_2081908, EPI_ISL_2081911, EPI_ISL_2081913, EPI_ISL_2081914, EPI_ISL_2081916, EPI_ISL_2081921, EPI_ISL_2081931, EPI_ISL_2081932,			

EPI_ISL_2081933, EPI_ISL_2081934, EPI_ISL_2081935, EPI_ISL_2081936, EPI_ISL_2081938, EPI_ISL_2081939, EPI_ISL_2081940, EPI_ISL_2081941, EPI_ISL_2081942, EPI_ISL_2081943, EPI_ISL_2081944, EPI_ISL_2081945, EPI_ISL_2081946, EPI_ISL_2081947, EPI_ISL_2081948, EPI_ISL_2081958, EPI_ISL_2081959, EPI_ISL_2081960, EPI_ISL_2081961, EPI_ISL_2081962, EPI_ISL_2081963, EPI_ISL_2082013, EPI_ISL_2082014, EPI_ISL_2082015, EPI_ISL_2082016, EPI_ISL_2082017, EPI_ISL_2082018, EPI_ISL_2082019, EPI_ISL_2082020, EPI_ISL_2082021, EPI_ISL_2082022, EPI_ISL_2082023, EPI_ISL_2082024, EPI_ISL_2082030, EPI_ISL_2082031			
see above	National Center of Infectious and Parasitic Diseases	National Center of Infectious and Parasitic Diseases	Alexiev, Ivanov, Korsun, Stoitsova, Philipova, Dimitrova, Grigorova L., Hristova, Donchev, Stoykov, Trifonova, Dobrinov, Grigorova I., Kantardjiev
EPI_ISL_2086187, EPI_ISL_2086192	CQRC_QUALITY CONTROL CHEMICAL BIOLOGICAL RISK_AOOR Villa Sofia Cervello Palermo	CQRC_QUALITY CONTROL CHEMICAL BIOLOGICAL RISK_AOOR Villa Sofia Cervello Palermo	Di Gaudio, F., Contino, F., Brunacci, G..
EPI_ISL_2086202, EPI_ISL_2086207, EPI_ISL_2086210	CQRC_QUALITY CONTROL CHEMICAL BIOLOGICAL RISK_AOOR Villa Sofia Cervello Palermo	CQRC_QUALITY CONTROL CHEMICAL BIOLOGICAL RISK_AOOR Villa Sofia Cervello Palermo	Di Gaudio, F., Brunacci, G., Contino, F.
EPI_ISL_2086268	Laboratory of Molecular Biology of Flavivirus, Oswaldo Cruz Institute, FIOCRUZ	Laboratory of Molecular Biology of Flavivirus, Oswaldo Cruz Institute, FIOCRUZ	Fernando Lopez Tort, Ieda Ribeiro, Lidiane Menezes, Alexandre dos Santos, Marta Pereira, Myrna Bonaldo
EPI_ISL_2098849, EPI_ISL_2098851, EPI_ISL_2098852, EPI_ISL_2098853, EPI_ISL_2098854, EPI_ISL_2098857, EPI_ISL_2098859	CQRC_QUALITY CONTROL CHEMICAL BIOLOGICAL RISK_AOOR Villa Sofia Cervello Palermo	CQRC_QUALITY CONTROL CHEMICAL BIOLOGICAL RISK_AOOR Villa Sofia Cervello Palermo	Di Gaudio, F., Contino, F., Brunacci, G.
EPI_ISL_2098860, EPI_ISL_2098862, EPI_ISL_2098863, EPI_ISL_2098864, EPI_ISL_2098865, EPI_ISL_2098866, EPI_ISL_2098867, EPI_ISL_2098868, EPI_ISL_2098869, EPI_ISL_2098871	CQRC_QUALITY CONTROL CHEMICAL BIOLOGICAL RISK_AOOR Villa Sofia Cervello Palermo	CQRC_QUALITY CONTROL CHEMICAL BIOLOGICAL RISK_AOOR Villa Sofia Cervello Palermo	Di Gaudio, F., Brunacci G., Contino, F.
EPI_ISL_2098948	Laboratorio Biologia Molecolare Sars Cov2 - UOC Laboratorio Analisi - Servizio Medicina di Laboratorio, Ospedale "San Francesco" - ATS-ASSL Nuoro	Laboratorio specialistico UOC Ematologia - Ospedale "San Francesco" - ATS-ASSL Nuoro	Piras Giovanna, Asproni Rosanna,Paolo Malune,Monne Maria Itria, Palmas Angelo Domenico, Fiamma Maura, Garau Maria Cristina, Mameli Giuseppe
EPI_ISL_2099949, EPI_ISL_2099950, EPI_ISL_2099951, EPI_ISL_2099952, EPI_ISL_2099953	CQRC_QUALITY CONTROL CHEMICAL BIOLOGICAL RISK_AOOR Villa Sofia Cervello Palermo	CQRC_QUALITY CONTROL CHEMICAL BIOLOGICAL RISK_AOOR Villa Sofia Cervello Palermo	Di Gaudio, F., Contino, F., Brunacci, G.
EPI_ISL_2099955	CQRC_QUALITY CONTROL CHEMICAL BIOLOGICAL RISK_AOOR Villa Sofia Cervello Palermo	CQRC_QUALITY CONTROL CHEMICAL BIOLOGICAL RISK_AOOR Villa Sofia Cervello Palermo	Di Gaudio, F., Contino, F., Gasparro G., Rinaudo G., Brunacci, G.
EPI_ISL_2099956, EPI_ISL_2099958, EPI_ISL_2099959, EPI_ISL_2099960, EPI_ISL_2099961, EPI_ISL_2099962, EPI_ISL_2099963, EPI_ISL_2099964	CQRC_QUALITY CONTROL CHEMICAL BIOLOGICAL RISK_AOOR Villa Sofia Cervello Palermo	CQRC_QUALITY CONTROL CHEMICAL BIOLOGICAL RISK_AOOR Villa Sofia Cervello Palermo	Di Gaudio, Brunacci, G., F. Contino, F.
EPI_ISL_2099965	CQRC_QUALITY CONTROL CHEMICAL BIOLOGICAL RISK_AOOR Villa Sofia Cervello Palermo	CQRC_QUALITY CONTROL CHEMICAL BIOLOGICAL RISK_AOOR Villa Sofia Cervello Palermo	Di Gaudio, F., Contino, F., Gasparro G., Rinaudo G., Brunacci, G.
EPI_ISL_2100701, EPI_ISL_2100702, EPI_ISL_2100703, EPI_ISL_2100704, EPI_ISL_2100705, EPI_ISL_2100706, EPI_ISL_2100707, EPI_ISL_2100708, EPI_ISL_2100710, EPI_ISL_2100711, EPI_ISL_2100712, EPI_ISL_2100713, EPI_ISL_2100714, EPI_ISL_2100715, EPI_ISL_2100716, EPI_ISL_2100717, EPI_ISL_2100718, EPI_ISL_2100719, EPI_ISL_2100720, EPI_ISL_2100721, EPI_ISL_2100722, EPI_ISL_2100723, EPI_ISL_2100724, EPI_ISL_2100725, EPI_ISL_2100726, EPI_ISL_2100727, EPI_ISL_2100728, EPI_ISL_2100729, EPI_ISL_2100730, EPI_ISL_2100731, EPI_ISL_2100732, EPI_ISL_2100733, EPI_ISL_2100734, EPI_ISL_2100735, EPI_ISL_2100736, EPI_ISL_2100737, EPI_ISL_2100738, EPI_ISL_2100739, EPI_ISL_2100740, EPI_ISL_2100741, EPI_ISL_2100742, EPI_ISL_2100744, EPI_ISL_2100745, EPI_ISL_2100746, EPI_ISL_2100747, EPI_ISL_2100748, EPI_ISL_2100749, EPI_ISL_2100750, EPI_ISL_2100751, EPI_ISL_2100752, EPI_ISL_2100753, EPI_ISL_2100754, EPI_ISL_2100755, EPI_ISL_2100756, EPI_ISL_2100757, EPI_ISL_2100758, EPI_ISL_2100759, EPI_ISL_2100760, EPI_ISL_2100761, EPI_ISL_2100762, EPI_ISL_2100763, EPI_ISL_2100764, EPI_ISL_2100765, EPI_ISL_2100766, EPI_ISL_2100767, EPI_ISL_2100768, EPI_ISL_2100769, EPI_ISL_2100770, EPI_ISL_2100771, EPI_ISL_2100772, EPI_ISL_2100773, EPI_ISL_2100775, EPI_ISL_2100776, EPI_ISL_2100777, EPI_ISL_2100778, EPI_ISL_2100779, EPI_ISL_2100780, EPI_ISL_2100781, EPI_ISL_2100782, EPI_ISL_2100783, EPI_ISL_2100784, EPI_ISL_2100785, EPI_ISL_2100786, EPI_ISL_2100789, EPI_ISL_2100791, EPI_ISL_2100792, EPI_ISL_2100794, EPI_ISL_2100796, EPI_ISL_2100797, EPI_ISL_2100798, EPI_ISL_2100799, EPI_ISL_2100800, EPI_ISL_2100801, EPI_ISL_2100802, EPI_ISL_2100804, EPI_ISL_2100805, EPI_ISL_2100806, EPI_ISL_2100807, EPI_ISL_2100808, EPI_ISL_2100810, EPI_ISL_2100811, EPI_ISL_2100812, EPI_ISL_2100813, EPI_ISL_2100814, EPI_ISL_2100816, EPI_ISL_2100818, EPI_ISL_2100819, EPI_ISL_2100820, EPI_ISL_2100822, EPI_ISL_2100824, EPI_ISL_2100825, EPI_ISL_2100826, EPI_ISL_2100827, EPI_ISL_2100828, EPI_ISL_2100830, EPI_ISL_2100831, EPI_ISL_2100832, EPI_ISL_2100833, EPI_ISL_2100834, EPI_ISL_2100835, EPI_ISL_2100836, EPI_ISL_2100839, EPI_ISL_2100840, EPI_ISL_2100841, EPI_ISL_2100842, EPI_ISL_2100843, EPI_ISL_2100845, EPI_ISL_2100846, EPI_ISL_2100847, EPI_ISL_2100848, EPI_ISL_2100849, EPI_ISL_2100850, EPI_ISL_2100851, EPI_ISL_2100852, EPI_ISL_2100854, EPI_ISL_2100855, EPI_ISL_2100857, EPI_ISL_2100858, EPI_ISL_2100859, EPI_ISL_2100860, EPI_ISL_2100861, EPI_ISL_2100862, EPI_ISL_2100865, EPI_ISL_2100867, EPI_ISL_2100868, EPI_ISL_2100869, EPI_ISL_2100883, EPI_ISL_2100884, EPI_ISL_2100885, EPI_ISL_2100886, EPI_ISL_2100887, EPI_ISL_2100888, EPI_ISL_2100889, EPI_ISL_2100890			
see above	The Ohio State University Applied Microbiology Services Laboratory	The Ohio State University Applied Microbiology Services Laboratory	Seth A. Faith PhD
EPI_ISL_2101022	Molecular Biology Laboratory, Faculty Medicine and Health Sciences, Warmadewa University	Eijkman Institute for Molecular Biology, National Agency for Research and Innovation; Molecular Biology Laboratory, Faculty Medicine and Health Sciences, Warmadewa University	Frilasita A Yudhaputri, Muhammad Rezki Rasyak, Willy Agustine, Hidayat Trimarsanto, Lydia V. Panggalo, Iskandar Adnan, Sukma Oktavianthi, Lidwina Priliani, Edison Johar, Sri Masyeni, Erly Sintya, Safarina G Malik, Khin Saw Myint, Amin Soebandrio
EPI_ISL_2101023	Molecular Biology Laboratory, Faculty Medicine and Health Sciences, Warmadewa University	Eijkman Institute for Molecular Biology, National Agency for Research and Innovation; Molecular Biology Laboratory, Faculty Medicine and Health Sciences, Warmadewa University	Muhammad Rezki Rasyak, Willy Agustine, Hidayat Trimarsanto, Lydia V. Panggalo, Iskandar Adnan, Sukma Oktavianthi, Lidwina Priliani, Edison Johar, Frilasita A Yudhaputri, Sri Masyeni, Erly Sintya, Safarina G Malik, Khin Saw Myint, Amin Soebandrio
EPI_ISL_2101024	Molecular Biology Laboratory, Faculty Medicine and Health Sciences, Warmadewa University	Eijkman Institute for Molecular Biology, National Agency for Research and Innovation; Molecular Biology Laboratory, Faculty Medicine and Health Sciences, Warmadewa University	Willy Agustine, Hidayat Trimarsanto, Lydia V. Panggalo, Iskandar Adnan, Sukma Oktavianthi, Lidwina Priliani, Edison Johar, Frilasita A Yudhaputri, Muhammad Rezki Rasyak, Sri Masyeni, Erly Sintya, Safarina G Malik, Khin Saw Myint, Amin Soebandrio
EPI_ISL_2101025	Molecular Biology Laboratory, Faculty Medicine and Health Sciences, Warmadewa University	Eijkman Institute for Molecular Biology, National Agency for Research and Innovation; Molecular Biology Laboratory, Faculty Medicine and Health Sciences, Warmadewa University	Hidayat Trimarsanto, Lydia V. Panggalo, Iskandar Adnan, Sukma Oktavianthi, Lidwina Priliani, Edison Johar, Frilasita A Yudhaputri, Muhammad Rezki Rasyak, Willy Agustine, Sri Masyeni, Erly Sintya, Safarina G Malik, Khin Saw Myint, Amin Soebandrio
EPI_ISL_2101026	Molecular Biology Laboratory, Faculty Medicine and Health Sciences, Warmadewa University	Eijkman Institute for Molecular Biology, National Agency for Research and Innovation; Molecular Biology Laboratory, Faculty Medicine and Health Sciences, Warmadewa University	Willy Agustine, Hidayat Trimarsanto, Lydia V. Panggalo, Iskandar Adnan, Sukma Oktavianthi, Lidwina Priliani, Edison Johar, Frilasita A Yudhaputri, Muhammad Rezki Rasyak, Sri Masyeni, Erly Sintya, Safarina G Malik, Khin Saw Myint, Amin Soebandrio
EPI_ISL_2101027	Molecular Biology Laboratory, Faculty Medicine and Health Sciences, Warmadewa University	Eijkman Institute for Molecular Biology, National Agency for Research and Innovation; Molecular Biology Laboratory, Faculty Medicine and Health Sciences, Warmadewa University	Iskandar Adnan, Sukma Oktavianthi, Lidwina Priliani, Edison Johar, Frilasita A Yudhaputri, Muhammad Rezki Rasyak, Willy Agustine, Hidayat Trimarsanto, Lydia V. Panggalo, Sri Masyeni, Erly Sintya, Safarina G Malik, Khin Saw Myint, Amin Soebandrio
EPI_ISL_2101028	Molecular Biology Laboratory, Faculty Medicine and Health Sciences, Warmadewa University	Eijkman Institute for Molecular Biology, National Agency for Research and Innovation; Molecular Biology Laboratory, Faculty Medicine and Health Sciences, Warmadewa University	Edison Johar, Frilasita A Yudhaputri, Muhammad Rezki Rasyak, Willy Agustine, Hidayat Trimarsanto, Lydia V. Panggalo, Iskandar Adnan, Sukma Oktavianthi, Lidwina Priliani, Sri Masyeni, Erly Sintya, Safarina G Malik, Khin Saw Myint, Amin Soebandrio
EPI_ISL_2101029	Molecular Biology Laboratory, Faculty Medicine and Health Sciences, Warmadewa University	Eijkman Institute for Molecular Biology, National Agency for Research and Innovation; Molecular Biology Laboratory, Faculty Medicine and Health Sciences, Warmadewa University	Hidayat Trimarsanto, Lydia V. Panggalo, Iskandar Adnan, Sukma Oktavianthi, Lidwina Priliani, Edison Johar, Frilasita A Yudhaputri, Muhammad Rezki Rasyak, Willy Agustine, Sri Masyeni, Erly Sintya, Safarina G Malik, Khin Saw Myint, Amin Soebandrio

EPI_ISL_2101030	Molecular Biology Laboratory, Faculty Medicine and Health Sciences, Warmadewa University	Eijkman Institute for Molecular Biology, National Agency for Research and Innovation; Molecular Biology Laboratory, Faculty Medicine and Health Sciences, Warmadewa University	Sukma Oktavianthi, Lidwina Priliani, Edison Johar, Frilasita A Yudhaputri, Muhammad Rezki Rasyak, Willy Agustine, Hidayat Trimarsanto, Lydia V. Panggalo, Iskandar Adnan, Sri Masyeni, Erly Sintya, Safarina G Malik, Khin Saw Myint, Amin Soebandrio
EPI_ISL_2101031	Molecular Biology Laboratory, Faculty Medicine and Health Sciences, Warmadewa University	Eijkman Institute for Molecular Biology, National Agency for Research and Innovation; Molecular Biology Laboratory, Faculty Medicine and Health Sciences, Warmadewa University	Frilasita A Yudhaputri, Muhammad Rezki Rasyak, Willy Agustine, Hidayat Trimarsanto, Lydia V. Panggalo, Iskandar Adnan, Sukma Oktavianthi, Lidwina Priliani, Edison Johar, Sri Masyeni, Erly Sintya, Safarina G Malik, Khin Saw Myint, Amin Soebandrio
EPI_ISL_2101032	Molecular Biology Laboratory, Faculty Medicine and Health Sciences, Warmadewa University	Eijkman Institute for Molecular Biology, National Agency for Research and Innovation; Molecular Biology Laboratory, Faculty Medicine and Health Sciences, Warmadewa University	Lydia V. Panggalo, Iskandar Adnan, Sukma Oktavianthi, Lidwina Priliani, Edison Johar, Frilasita A Yudhaputri, Muhammad Rezki Rasyak, Willy Agustine, Hidayat Trimarsanto, Sri Masyeni, Erly Sintya, Safarina G Malik, Khin Saw Myint, Amin Soebandrio
EPI_ISL_2101034	Molecular Biology Laboratory, Faculty Medicine and Health Sciences, Warmadewa University	Eijkman Institute for Molecular Biology, National Agency for Research and Innovation; Molecular Biology Laboratory, Faculty Medicine and Health Sciences, Warmadewa University	Willy Agustine, Hidayat Trimarsanto, Lydia V. Panggalo, Iskandar Adnan, Sukma Oktavianthi, Lidwina Priliani, Edison Johar, Frilasita A Yudhaputri, Muhammad Rezki Rasyak, Sri Masyeni, Erly Sintya, Safarina G Malik, Khin Saw Myint, Amin Soebandrio
EPI_ISL_2101035, EPI_ISL_2101036, EPI_ISL_2101037	Molecular Biology Laboratory, Faculty Medicine and Health Sciences, Warmadewa University	Eijkman Institute for Molecular Biology, National Agency for Research and Innovation; Molecular Biology Laboratory, Faculty Medicine and Health Sciences, Warmadewa University	Lydia V. Panggalo, Iskandar Adnan, Sukma Oktavianthi, Lidwina Priliani, Edison Johar, Frilasita A Yudhaputri, Muhammad Rezki Rasyak, Willy Agustine, Hidayat Trimarsanto, Sri Masyeni, Erly Sintya, Safarina G Malik, Khin Saw Myint, Amin Soebandrio
EPI_ISL_2101038	Molecular Biology Laboratory, Faculty Medicine and Health Sciences, Warmadewa University	Eijkman Institute for Molecular Biology, National Agency for Research and Innovation; Molecular Biology Laboratory, Faculty Medicine and Health Sciences, Warmadewa University	Iskandar Adnan, Sukma Oktavianthi, Lidwina Priliani, Edison Johar, Frilasita A Yudhaputri, Muhammad Rezki Rasyak, Willy Agustine, Hidayat Trimarsanto, Lydia V. Panggalo, Sri Masyeni, Erly Sintya, Safarina G Malik, Khin Saw Myint, Amin Soebandrio
EPI_ISL_2101039	Molecular Biology Laboratory, Faculty Medicine and Health Sciences, Warmadewa University	Eijkman Institute for Molecular Biology, National Agency for Research and Innovation; Molecular Biology Laboratory, Faculty Medicine and Health Sciences, Warmadewa University	Edison Johar, Frilasita A Yudhaputri, Muhammad Rezki Rasyak, Willy Agustine, Hidayat Trimarsanto, Lydia V. Panggalo, Iskandar Adnan, Sukma Oktavianthi, Lidwina Priliani, Sri Masyeni, Erly Sintya, Safarina G Malik, Khin Saw Myint, Amin Soebandrio
EPI_ISL_2101040	Molecular Biology Laboratory, Faculty Medicine and Health Sciences, Warmadewa University	Eijkman Institute for Molecular Biology, National Agency for Research and Innovation; Molecular Biology Laboratory, Faculty Medicine and Health Sciences, Warmadewa University	Hidayat Trimarsanto, Lydia V. Panggalo, Iskandar Adnan, Sukma Oktavianthi, Lidwina Priliani, Edison Johar, Frilasita A Yudhaputri, Muhammad Rezki Rasyak, Willy Agustine, Sri Masyeni, Erly Sintya, Safarina G Malik, Khin Saw Myint, Amin Soebandrio
EPI_ISL_2101041	Molecular Biology Laboratory, Faculty Medicine and Health Sciences, Warmadewa University	Eijkman Institute for Molecular Biology, National Agency for Research and Innovation; Molecular Biology Laboratory, Faculty Medicine and Health Sciences, Warmadewa University	Sukma Oktavianthi, Lidwina Priliani, Edison Johar, Frilasita A Yudhaputri, Muhammad Rezki Rasyak, Willy Agustine, Hidayat Trimarsanto, Lydia V. Panggalo, Iskandar Adnan, Sri Masyeni, Erly Sintya, Safarina G Malik, Khin Saw Myint, Amin Soebandrio
EPI_ISL_2101042	Molecular Biology Laboratory, Faculty Medicine and Health Sciences, Warmadewa University	Eijkman Institute for Molecular Biology, National Agency for Research and Innovation; Molecular Biology Laboratory, Faculty Medicine and Health Sciences, Warmadewa University	Willy Agustine, Hidayat Trimarsanto, Lydia V. Panggalo, Iskandar Adnan, Sukma Oktavianthi, Lidwina Priliani, Edison Johar, Frilasita A Yudhaputri, Muhammad Rezki Rasyak, Sri Masyeni, Erly Sintya, Safarina G Malik, Khin Saw Myint, Amin Soebandrio
EPI_ISL_2101043	Molecular Biology Laboratory, Faculty Medicine and Health Sciences, Warmadewa University	Eijkman Institute for Molecular Biology, National Agency for Research and Innovation; Molecular Biology Laboratory, Faculty Medicine and Health Sciences, Warmadewa University	Hidayat Trimarsanto, Lydia V. Panggalo, Iskandar Adnan, Sukma Oktavianthi, Lidwina Priliani, Edison Johar, Frilasita A Yudhaputri, Muhammad Rezki Rasyak, Willy Agustine, Sri Masyeni, Erly Sintya, Safarina G Malik, Khin Saw Myint, Amin Soebandrio
EPI_ISL_2101044	Molecular Biology Laboratory, Faculty Medicine and Health Sciences, Warmadewa University	Eijkman Institute for Molecular Biology, National Agency for Research and Innovation; Molecular Biology Laboratory, Faculty Medicine and Health Sciences, Warmadewa University	Lydia V. Panggalo, Iskandar Adnan, Sukma Oktavianthi, Lidwina Priliani, Edison Johar, Frilasita A Yudhaputri, Muhammad Rezki Rasyak, Willy Agustine, Hidayat Trimarsanto, Sri Masyeni, Erly Sintya, Safarina G Malik, Khin Saw Myint, Amin Soebandrio
EPI_ISL_2101045	Molecular Biology Laboratory, Faculty Medicine and Health Sciences, Warmadewa University	Eijkman Institute for Molecular Biology, National Agency for Research and Innovation; Molecular Biology Laboratory, Faculty Medicine and Health Sciences, Warmadewa University	Iskandar Adnan, Sukma Oktavianthi, Lidwina Priliani, Edison Johar, Frilasita A Yudhaputri, Muhammad Rezki Rasyak, Willy Agustine, Hidayat Trimarsanto, Lydia V. Panggalo, Sri Masyeni, Erly Sintya, Safarina G Malik, Khin Saw Myint, Amin Soebandrio
EPI_ISL_2101046	Molecular Biology Laboratory, Faculty Medicine and Health Sciences, Warmadewa University	Eijkman Institute for Molecular Biology, National Agency for Research and Innovation; Molecular Biology Laboratory, Faculty Medicine and Health Sciences, Warmadewa University	Sukma Oktavianthi, Lidwina Priliani, Edison Johar, Frilasita A Yudhaputri, Muhammad Rezki Rasyak, Willy Agustine, Hidayat Trimarsanto, Lydia V. Panggalo, Iskandar Adnan, Sri Masyeni, Erly Sintya, Safarina G Malik, Khin Saw Myint, Amin Soebandrio
EPI_ISL_2101047	Molecular Biology Laboratory, Faculty Medicine and Health Sciences, Warmadewa University	Eijkman Institute for Molecular Biology, National Agency for Research and Innovation; Molecular Biology Laboratory, Faculty Medicine and Health Sciences, Warmadewa University	Lidwina Priliani, Edison Johar, Frilasita A Yudhaputri, Muhammad Rezki Rasyak, Willy Agustine, Hidayat Trimarsanto, Lydia V. Panggalo, Iskandar Adnan, Sukma Oktavianthi, Sri Masyeni, Erly Sintya, Safarina G Malik, Khin Saw Myint, Amin Soebandrio
EPI_ISL_2101048	Molecular Biology Laboratory, Faculty Medicine and Health Sciences, Warmadewa University	Eijkman Institute for Molecular Biology, National Agency for Research and Innovation; Molecular Biology Laboratory, Faculty Medicine and Health Sciences, Warmadewa University	Edison Johar, Frilasita A Yudhaputri, Muhammad Rezki Rasyak, Willy Agustine, Hidayat Trimarsanto, Lydia V. Panggalo, Iskandar Adnan, Sukma Oktavianthi, Lidwina Priliani, Sri Masyeni, Erly Sintya, Safarina G Malik, Khin Saw Myint, Amin Soebandrio
EPI_ISL_2101049	Molecular Biology Laboratory, Faculty Medicine and Health Sciences, Warmadewa University	Eijkman Institute for Molecular Biology, National Agency for Research and Innovation; Molecular Biology Laboratory, Faculty Medicine and Health Sciences, Warmadewa University	Frilasita A Yudhaputri, Muhammad Rezki Rasyak, Willy Agustine, Hidayat Trimarsanto, Lydia V. Panggalo, Iskandar Adnan, Sukma Oktavianthi, Lidwina Priliani, Edison Johar, Sri Masyeni, Erly Sintya, Safarina G Malik, Khin Saw Myint, Amin Soebandrio
EPI_ISL_2101050	Molecular Biology Laboratory, Faculty Medicine and Health Sciences, Warmadewa University	Eijkman Institute for Molecular Biology, National Agency for Research and Innovation; Molecular Biology Laboratory, Faculty Medicine and Health Sciences, Warmadewa University	Willy Agustine, Hidayat Trimarsanto, Lydia V. Panggalo, Iskandar Adnan, Sukma Oktavianthi, Lidwina Priliani, Edison Johar, Frilasita A Yudhaputri, Muhammad Rezki Rasyak, Sri Masyeni, Erly Sintya, Safarina G Malik, Khin Saw Myint, Amin Soebandrio
EPI_ISL_2101051	Molecular Biology Laboratory, Faculty Medicine and Health Sciences, Warmadewa University	Eijkman Institute for Molecular Biology, National Agency for Research and Innovation; Molecular Biology Laboratory, Faculty Medicine and Health Sciences, Warmadewa University	Hidayat Trimarsanto, Lydia V. Panggalo, Iskandar Adnan, Sukma Oktavianthi, Lidwina Priliani, Edison Johar, Frilasita A Yudhaputri, Muhammad Rezki Rasyak, Willy Agustine, Sri Masyeni, Erly Sintya, Safarina G Malik, Khin Saw Myint, Amin Soebandrio
EPI_ISL_2101052	Molecular Biology Laboratory, Faculty Medicine and Health Sciences, Warmadewa University	Eijkman Institute for Molecular Biology, National Agency for Research and Innovation; Molecular Biology Laboratory,	Lydia V. Panggalo, Iskandar Adnan, Sukma Oktavianthi, Lidwina Priliani, Edison Johar, Frilasita A Yudhaputri, Muhammad Rezki Rasyak, Willy Agustine, Hidayat Trimarsanto, Sri Masyeni, Erly Sintya, Safarina G Malik, Khin Saw Myint, Amin Soebandrio

		Faculty Medicine and Health Sciences, Warmadewa University	
EPI_ISL_2101053	Molecular Biology Laboratory, Faculty Medicine and Health Sciences, Warmadewa University	Eijkman Institute for Molecular Biology, National Agency for Research and Innovation; Molecular Biology Laboratory, Faculty Medicine and Health Sciences, Warmadewa University	Iskandar Adnan, Sukma Oktavianthi, Lidwina Priliani, Edison Johar, Frilasita A Yudhaputri, Muhammad Rezki Rasyak, Willy Agustine, Hidayat Trimarsanto, Lydia V. Panggalo, Sri Masyeni, Erly Sintya, Safarina G Malik, Khin Saw Myint, Amin Soebandrio
EPI_ISL_2101054	Molecular Biology Laboratory, Faculty Medicine and Health Sciences, Warmadewa University	Eijkman Institute for Molecular Biology, National Agency for Research and Innovation; Molecular Biology Laboratory, Faculty Medicine and Health Sciences, Warmadewa University	Sukma Oktavianthi, Lidwina Priliani, Edison Johar, Frilasita A Yudhaputri, Muhammad Rezki Rasyak, Willy Agustine, Hidayat Trimarsanto, Lydia V. Panggalo, Iskandar Adnan, Sri Masyeni, Erly Sintya, Safarina G Malik, Khin Saw Myint, Amin Soebandrio
EPI_ISL_2101055	Molecular Biology Laboratory, Faculty Medicine and Health Sciences, Warmadewa University	Eijkman Institute for Molecular Biology, National Agency for Research and Innovation; Molecular Biology Laboratory, Faculty Medicine and Health Sciences, Warmadewa University	Lidwina Priliani, Edison Johar, Frilasita A Yudhaputri, Muhammad Rezki Rasyak, Willy Agustine, Hidayat Trimarsanto, Lydia V. Panggalo, Iskandar Adnan, Sukma Oktavianthi, Sri Masyeni, Erly Sintya, Safarina G Malik, Khin Saw Myint, Amin Soebandrio
EPI_ISL_2101056	Molecular Biology Laboratory, Faculty Medicine and Health Sciences, Warmadewa University	Eijkman Institute for Molecular Biology, National Agency for Research and Innovation; Molecular Biology Laboratory, Faculty Medicine and Health Sciences, Warmadewa University	Edison Johar, Frilasita A Yudhaputri, Muhammad Rezki Rasyak, Willy Agustine, Hidayat Trimarsanto, Lydia V. Panggalo, Iskandar Adnan, Sukma Oktavianthi, Lidwina Priliani, Sri Masyeni, Erly Sintya, Safarina G Malik, Khin Saw Myint, Amin Soebandrio
EPI_ISL_2101057	Molecular Biology Laboratory, Faculty Medicine and Health Sciences, Warmadewa University	Eijkman Institute for Molecular Biology, National Agency for Research and Innovation; Molecular Biology Laboratory, Faculty Medicine and Health Sciences, Warmadewa University	Frilasita A Yudhaputri, Muhammad Rezki Rasyak, Willy Agustine, Hidayat Trimarsanto, Lydia V. Panggalo, Iskandar Adnan, Sukma Oktavianthi, Lidwina Priliani, Edison Johar, Sri Masyeni, Erly Sintya, Safarina G Malik, Khin Saw Myint, Amin Soebandrio
EPI_ISL_2101058	Molecular Biology Laboratory, Faculty Medicine and Health Sciences, Warmadewa University	Eijkman Institute for Molecular Biology, National Agency for Research and Innovation; Molecular Biology Laboratory, Faculty Medicine and Health Sciences, Warmadewa University	Willy Agustine, Hidayat Trimarsanto, Lydia V. Panggalo, Iskandar Adnan, Sukma Oktavianthi, Lidwina Priliani, Edison Johar, Frilasita A Yudhaputri, Muhammad Rezki Rasyak, Sri Masyeni, Erly Sintya, Safarina G Malik, Khin Saw Myint, Amin Soebandrio
EPI_ISL_2101059	Molecular Biology Laboratory, Faculty Medicine and Health Sciences, Warmadewa University	Eijkman Institute for Molecular Biology, National Agency for Research and Innovation; Molecular Biology Laboratory, Faculty Medicine and Health Sciences, Warmadewa University	Hidayat Trimarsanto, Lydia V. Panggalo, Iskandar Adnan, Sukma Oktavianthi, Lidwina Priliani, Edison Johar, Frilasita A Yudhaputri, Muhammad Rezki Rasyak, Willy Agustine, Sri Masyeni, Erly Sintya, Safarina G Malik, Khin Saw Myint, Amin Soebandrio
EPI_ISL_2101060	Molecular Biology Laboratory, Faculty Medicine and Health Sciences, Warmadewa University	Eijkman Institute for Molecular Biology, National Agency for Research and Innovation; Molecular Biology Laboratory, Faculty Medicine and Health Sciences, Warmadewa University	Lydia V. Panggalo, Iskandar Adnan, Sukma Oktavianthi, Lidwina Priliani, Edison Johar, Frilasita A Yudhaputri, Muhammad Rezki Rasyak, Willy Agustine, Hidayat Trimarsanto, Sri Masyeni, Erly Sintya, Safarina G Malik, Khin Saw Myint, Amin Soebandrio
EPI_ISL_2101061	Molecular Biology Laboratory, Faculty Medicine and Health Sciences, Warmadewa University	Eijkman Institute for Molecular Biology, National Agency for Research and Innovation; Molecular Biology Laboratory, Faculty Medicine and Health Sciences, Warmadewa University	Iskandar Adnan, Sukma Oktavianthi, Lidwina Priliani, Edison Johar, Frilasita A Yudhaputri, Muhammad Rezki Rasyak, Willy Agustine, Hidayat Trimarsanto, Lydia V. Panggalo, Sri Masyeni, Erly Sintya, Safarina G Malik, Khin Saw Myint, Amin Soebandrio
EPI_ISL_2101170	Molecular Biology Laboratory, Faculty Medicine and Health Sciences, Warmadewa University	Eijkman Institute for Molecular Biology, National Agency for Research and Innovation; Molecular Biology Laboratory, Faculty Medicine and Health Sciences, Warmadewa University	Lidwina Priliani, Edison Johar, Frilasita A Yudhaputri, Muhammad Rezki Rasyak, Willy Agustine, Hidayat Trimarsanto, Lydia V. Panggalo, Iskandar Adnan, Sukma Oktavianthi, Sri Masyeni, Erly Sintya, Safarina G Malik, Khin Saw Myint, Amin Soebandrio
EPI_ISL_2101171	Molecular Biology Laboratory, Faculty Medicine and Health Sciences, Warmadewa University	Eijkman Institute for Molecular Biology, National Agency for Research and Innovation; Molecular Biology Laboratory, Faculty Medicine and Health Sciences, Warmadewa University	Edison Johar, Frilasita A Yudhaputri, Muhammad Rezki Rasyak, Willy Agustine, Hidayat Trimarsanto, Lydia V. Panggalo, Iskandar Adnan, Sukma Oktavianthi, Lidwina Priliani, Sri Masyeni, Erly Sintya, Safarina G Malik, Khin Saw Myint, Amin Soebandrio
EPI_ISL_2101172	Molecular Biology Laboratory, Faculty Medicine and Health Sciences, Warmadewa University	Eijkman Institute for Molecular Biology, National Agency for Research and Innovation; Molecular Biology Laboratory, Faculty Medicine and Health Sciences, Warmadewa University	Iskandar Adnan, Sukma Oktavianthi, Lidwina Priliani, Edison Johar, Frilasita A Yudhaputri, Muhammad Rezki Rasyak, Willy Agustine, Hidayat Trimarsanto, Lydia V. Panggalo, Sri Masyeni, Erly Sintya, Safarina G Malik, Khin Saw Myint, Amin Soebandrio
EPI_ISL_2101173	Molecular Biology Laboratory, Faculty Medicine and Health Sciences, Warmadewa University	Eijkman Institute for Molecular Biology, National Agency for Research and Innovation; Molecular Biology Laboratory, Faculty Medicine and Health Sciences, Warmadewa University	Sukma Oktavianthi, Lidwina Priliani, Edison Johar, Frilasita A Yudhaputri, Muhammad Rezki Rasyak, Willy Agustine, Hidayat Trimarsanto, Lydia V. Panggalo, Iskandar Adnan, Sri Masyeni, Erly Sintya, Safarina G Malik, Khin Saw Myint, Amin Soebandrio
EPI_ISL_2101174	Molecular Biology Laboratory, Faculty Medicine and Health Sciences, Warmadewa University	Eijkman Institute for Molecular Biology, National Agency for Research and Innovation; Molecular Biology Laboratory, Faculty Medicine and Health Sciences, Warmadewa University	Lidwina Priliani, Edison Johar, Frilasita A Yudhaputri, Muhammad Rezki Rasyak, Willy Agustine, Hidayat Trimarsanto, Lydia V. Panggalo, Iskandar Adnan, Sukma Oktavianthi, Sri Masyeni, Erly Sintya, Safarina G Malik, Khin Saw Myint, Amin Soebandrio
EPI_ISL_2101175	Molecular Biology Laboratory, Faculty Medicine and Health Sciences, Warmadewa University	Eijkman Institute for Molecular Biology, National Agency for Research and Innovation; Molecular Biology Laboratory, Faculty Medicine and Health Sciences, Warmadewa University	Edison Johar, Frilasita A Yudhaputri, Muhammad Rezki Rasyak, Willy Agustine, Hidayat Trimarsanto, Lydia V. Panggalo, Iskandar Adnan, Sukma Oktavianthi, Lidwina Priliani, Sri Masyeni, Erly Sintya, Safarina G Malik, Khin Saw Myint, Amin Soebandrio
EPI_ISL_2101176	Molecular Biology Laboratory, Faculty Medicine and Health Sciences, Warmadewa University	Eijkman Institute for Molecular Biology, National Agency for Research and Innovation; Molecular Biology Laboratory, Faculty Medicine and Health Sciences, Warmadewa University	Frilasita A Yudhaputri, Muhammad Rezki Rasyak, Willy Agustine, Hidayat Trimarsanto, Lydia V. Panggalo, Iskandar Adnan, Sukma Oktavianthi, Lidwina Priliani, Edison Johar, Sri Masyeni, Erly Sintya, Safarina G Malik, Khin Saw Myint, Amin Soebandrio
EPI_ISL_2101177	Molecular Biology Laboratory, Faculty Medicine and Health Sciences, Warmadewa University	Eijkman Institute for Molecular Biology, National Agency for Research and Innovation; Molecular Biology Laboratory, Faculty Medicine and Health Sciences, Warmadewa University	Willy Agustine, Hidayat Trimarsanto, Lydia V. Panggalo, Iskandar Adnan, Sukma Oktavianthi, Lidwina Priliani, Edison Johar, Frilasita A Yudhaputri, Muhammad Rezki Rasyak, Sri Masyeni, Erly Sintya, Safarina G Malik, Khin Saw Myint, Amin Soebandrio
EPI_ISL_2101178	Molecular Biology Laboratory, Faculty Medicine and Health Sciences, Warmadewa University	Eijkman Institute for Molecular Biology, National Agency for Research and Innovation; Molecular Biology Laboratory, Faculty Medicine and Health Sciences, Warmadewa University	Hidayat Trimarsanto, Lydia V. Panggalo, Iskandar Adnan, Sukma Oktavianthi, Lidwina Priliani, Edison Johar, Frilasita A Yudhaputri, Muhammad Rezki Rasyak, Willy Agustine, Sri Masyeni, Erly Sintya, Safarina G Malik, Khin Saw Myint, Amin Soebandrio
EPI_ISL_2101179	Molecular Biology Laboratory, Faculty Medicine and Health Sciences, Warmadewa University	Eijkman Institute for Molecular Biology, National Agency for Research and Innovation; Molecular Biology Laboratory, Faculty Medicine and Health Sciences, Warmadewa University	Lydia V. Panggalo, Iskandar Adnan, Sukma Oktavianthi, Lidwina Priliani, Edison Johar, Frilasita A Yudhaputri, Muhammad Rezki Rasyak, Willy Agustine, Hidayat Trimarsanto, Sri Masyeni, Erly Sintya, Safarina G Malik, Khin Saw Myint, Amin Soebandrio

EPI_ISL_2101180	Molecular Biology Laboratory, Faculty Medicine and Health Sciences, Warmadewa University	Eijkman Institute for Molecular Biology, National Agency for Research and Innovation; Molecular Biology Laboratory, Faculty Medicine and Health Sciences, Warmadewa University	Sukma Oktavianthi, Lidwina Priliani, Edison Johar, Frilasita A Yudhaputri, Muhammad Rezki Rasyak, Willy Agustine, Hidayat Trimarsanto, Lydia V. Panggalo, Iskandar Adnan, Sri Masyeni, Erly Sintya, Safarina G Malik, Khin Saw Myint, Amin Soebandrio
EPI_ISL_2101181	Molecular Biology Laboratory, Faculty Medicine and Health Sciences, Warmadewa University	Eijkman Institute for Molecular Biology, National Agency for Research and Innovation; Molecular Biology Laboratory, Faculty Medicine and Health Sciences, Warmadewa University	Lidwina Priliani, Edison Johar, Frilasita A Yudhaputri, Muhammad Rezki Rasyak, Willy Agustine, Hidayat Trimarsanto, Lydia V. Panggalo, Iskandar Adnan, Sukma Oktavianthi, Sri Masyeni, Erly Sintya, Safarina G Malik, Khin Saw Myint, Amin Soebandrio
EPI_ISL_2101182	Molecular Biology Laboratory, Faculty Medicine and Health Sciences, Warmadewa University	Eijkman Institute for Molecular Biology, National Agency for Research and Innovation; Molecular Biology Laboratory, Faculty Medicine and Health Sciences, Warmadewa University	Edison Johar, Frilasita A Yudhaputri, Muhammad Rezki Rasyak, Willy Agustine, Hidayat Trimarsanto, Lydia V. Panggalo, Iskandar Adnan, Sukma Oktavianthi, Lidwina Priliani, Sri Masyeni, Erly Sintya, Safarina G Malik, Khin Saw Myint, Amin Soebandrio
EPI_ISL_2101183	Molecular Biology Laboratory, Faculty Medicine and Health Sciences, Warmadewa University	Eijkman Institute for Molecular Biology, National Agency for Research and Innovation; Molecular Biology Laboratory, Faculty Medicine and Health Sciences, Warmadewa University	Frilasita A Yudhaputri, Muhammad Rezki Rasyak, Willy Agustine, Hidayat Trimarsanto, Lydia V. Panggalo, Iskandar Adnan, Sukma Oktavianthi, Lidwina Priliani, Edison Johar, Sri Masyeni, Erly Sintya, Safarina G Malik, Khin Saw Myint, Amin Soebandrio
EPI_ISL_2101184	Molecular Biology Laboratory, Faculty Medicine and Health Sciences, Warmadewa University	Eijkman Institute for Molecular Biology, National Agency for Research and Innovation; Molecular Biology Laboratory, Faculty Medicine and Health Sciences, Warmadewa University	Willy Agustine, Hidayat Trimarsanto, Lydia V. Panggalo, Iskandar Adnan, Sukma Oktavianthi, Lidwina Priliani, Edison Johar, Frilasita A Yudhaputri, Muhammad Rezki Rasyak, Sri Masyeni, Erly Sintya, Safarina G Malik, Khin Saw Myint, Amin Soebandrio
EPI_ISL_2101185	Molecular Biology Laboratory, Faculty Medicine and Health Sciences, Warmadewa University	Eijkman Institute for Molecular Biology, National Agency for Research and Innovation; Molecular Biology Laboratory, Faculty Medicine and Health Sciences, Warmadewa University	Hidayat Trimarsanto, Lydia V. Panggalo, Iskandar Adnan, Sukma Oktavianthi, Lidwina Priliani, Edison Johar, Frilasita A Yudhaputri, Muhammad Rezki Rasyak, Willy Agustine, Sri Masyeni, Erly Sintya, Safarina G Malik, Khin Saw Myint, Amin Soebandrio
EPI_ISL_2101186	Molecular Biology Laboratory, Faculty Medicine and Health Sciences, Warmadewa University	Eijkman Institute for Molecular Biology, National Agency for Research and Innovation; Molecular Biology Laboratory, Faculty Medicine and Health Sciences, Warmadewa University	Lydia V. Panggalo, Iskandar Adnan, Sukma Oktavianthi, Lidwina Priliani, Edison Johar, Frilasita A Yudhaputri, Muhammad Rezki Rasyak, Willy Agustine, Hidayat Trimarsanto, Sri Masyeni, Erly Sintya, Safarina G Malik, Khin Saw Myint, Amin Soebandrio
EPI_ISL_2101187	Molecular Biology Laboratory, Faculty Medicine and Health Sciences, Warmadewa University	Eijkman Institute for Molecular Biology, National Agency for Research and Innovation; Molecular Biology Laboratory, Faculty Medicine and Health Sciences, Warmadewa University	Iskandar Adnan, Sukma Oktavianthi, Lidwina Priliani, Edison Johar, Frilasita A Yudhaputri, Muhammad Rezki Rasyak, Willy Agustine, Hidayat Trimarsanto, Lydia V. Panggalo, Sri Masyeni, Erly Sintya, Safarina G Malik, Khin Saw Myint, Amin Soebandrio
EPI_ISL_2101188	Molecular Biology Laboratory, Faculty Medicine and Health Sciences, Warmadewa University	Eijkman Institute for Molecular Biology, National Agency for Research and Innovation; Molecular Biology Laboratory, Faculty Medicine and Health Sciences, Warmadewa University	Sukma Oktavianthi, Lidwina Priliani, Edison Johar, Frilasita A Yudhaputri, Muhammad Rezki Rasyak, Willy Agustine, Hidayat Trimarsanto, Lydia V. Panggalo, Iskandar Adnan, Sri Masyeni, Erly Sintya, Safarina G Malik, Khin Saw Myint, Amin Soebandrio
EPI_ISL_2101189	Molecular Biology Laboratory, Faculty Medicine and Health Sciences, Warmadewa University	Eijkman Institute for Molecular Biology, National Agency for Research and Innovation; Molecular Biology Laboratory, Faculty Medicine and Health Sciences, Warmadewa University	Lidwina Priliani, Edison Johar, Frilasita A Yudhaputri, Muhammad Rezki Rasyak, Willy Agustine, Hidayat Trimarsanto, Lydia V. Panggalo, Iskandar Adnan, Sukma Oktavianthi, Sri Masyeni, Erly Sintya, Safarina G Malik, Khin Saw Myint, Amin Soebandrio
EPI_ISL_2101190	Molecular Biology Laboratory, Faculty Medicine and Health Sciences, Warmadewa University	Eijkman Institute for Molecular Biology, National Agency for Research and Innovation; Molecular Biology Laboratory, Faculty Medicine and Health Sciences, Warmadewa University	Frilasita A Yudhaputri, Muhammad Rezki Rasyak, Willy Agustine, Hidayat Trimarsanto, Lydia V. Panggalo, Iskandar Adnan, Sukma Oktavianthi, Lidwina Priliani, Edison Johar, Sri Masyeni, Erly Sintya, Safarina G Malik, Khin Saw Myint, Amin Soebandrio
EPI_ISL_2101191	Molecular Biology Laboratory, Faculty Medicine and Health Sciences, Warmadewa University	Eijkman Institute for Molecular Biology, National Agency for Research and Innovation; Molecular Biology Laboratory, Faculty Medicine and Health Sciences, Warmadewa University	Willy Agustine, Hidayat Trimarsanto, Lydia V. Panggalo, Iskandar Adnan, Sukma Oktavianthi, Lidwina Priliani, Edison Johar, Frilasita A Yudhaputri, Muhammad Rezki Rasyak, Sri Masyeni, Erly Sintya, Safarina G Malik, Khin Saw Myint, Amin Soebandrio
EPI_ISL_2101192	Molecular Biology Laboratory, Faculty Medicine and Health Sciences, Warmadewa University	Eijkman Institute for Molecular Biology, National Agency for Research and Innovation; Molecular Biology Laboratory, Faculty Medicine and Health Sciences, Warmadewa University	Hidayat Trimarsanto, Lydia V. Panggalo, Iskandar Adnan, Sukma Oktavianthi, Lidwina Priliani, Edison Johar, Frilasita A Yudhaputri, Muhammad Rezki Rasyak, Willy Agustine, Sri Masyeni, Erly Sintya, Safarina G Malik, Khin Saw Myint, Amin Soebandrio
EPI_ISL_2101193	Molecular Biology Laboratory, Faculty Medicine and Health Sciences, Warmadewa University	Eijkman Institute for Molecular Biology, National Agency for Research and Innovation; Molecular Biology Laboratory, Faculty Medicine and Health Sciences, Warmadewa University	Lydia V. Panggalo, Iskandar Adnan, Sukma Oktavianthi, Lidwina Priliani, Edison Johar, Frilasita A Yudhaputri, Muhammad Rezki Rasyak, Willy Agustine, Hidayat Trimarsanto, Sri Masyeni, Erly Sintya, Safarina G Malik, Khin Saw Myint, Amin Soebandrio
EPI_ISL_2101194	Molecular Biology Laboratory, Faculty Medicine and Health Sciences, Warmadewa University	Eijkman Institute for Molecular Biology, National Agency for Research and Innovation; Molecular Biology Laboratory, Faculty Medicine and Health Sciences, Warmadewa University	Iskandar Adnan, Sukma Oktavianthi, Lidwina Priliani, Edison Johar, Frilasita A Yudhaputri, Muhammad Rezki Rasyak, Willy Agustine, Hidayat Trimarsanto, Lydia V. Panggalo, Sri Masyeni, Erly Sintya, Safarina G Malik, Khin Saw Myint, Amin Soebandrio
EPI_ISL_2101195	Molecular Biology Laboratory, Faculty Medicine and Health Sciences, Warmadewa University	Eijkman Institute for Molecular Biology, National Agency for Research and Innovation; Molecular Biology Laboratory, Faculty Medicine and Health Sciences, Warmadewa University	Sukma Oktavianthi, Lidwina Priliani, Edison Johar, Frilasita A Yudhaputri, Muhammad Rezki Rasyak, Willy Agustine, Hidayat Trimarsanto, Lydia V. Panggalo, Iskandar Adnan, Sri Masyeni, Erly Sintya, Safarina G Malik, Khin Saw Myint, Amin Soebandrio
EPI_ISL_2101196	Molecular Biology Laboratory, Faculty Medicine and Health Sciences, Warmadewa University	Eijkman Institute for Molecular Biology, National Agency for Research and Innovation; Molecular Biology Laboratory, Faculty Medicine and Health Sciences, Warmadewa University	Lidwina Priliani, Edison Johar, Frilasita A Yudhaputri, Muhammad Rezki Rasyak, Willy Agustine, Hidayat Trimarsanto, Lydia V. Panggalo, Iskandar Adnan, Sukma Oktavianthi, Sri Masyeni, Erly Sintya, Safarina G Malik, Khin Saw Myint, Amin Soebandrio
EPI_ISL_2101197	Molecular Biology Laboratory, Faculty Medicine and Health Sciences, Warmadewa University	Eijkman Institute for Molecular Biology, National Agency for Research and Innovation; Molecular Biology Laboratory, Faculty Medicine and Health Sciences, Warmadewa University	Edison Johar, Frilasita A Yudhaputri, Muhammad Rezki Rasyak, Willy Agustine, Hidayat Trimarsanto, Lydia V. Panggalo, Iskandar Adnan, Sukma Oktavianthi, Lidwina Priliani, Sri Masyeni, Erly Sintya, Safarina G Malik, Khin Saw Myint, Amin Soebandrio
EPI_ISL_2101198	Molecular Biology Laboratory, Faculty Medicine and Health Sciences, Warmadewa University	Eijkman Institute for Molecular Biology, National Agency for Research and Innovation; Molecular Biology Laboratory, Faculty Medicine and Health Sciences, Warmadewa University	Frilasita A Yudhaputri, Muhammad Rezki Rasyak, Willy Agustine, Hidayat Trimarsanto, Lydia V. Panggalo, Iskandar Adnan, Sukma Oktavianthi, Lidwina Priliani, Edison Johar, Sri Masyeni, Erly Sintya, Safarina G Malik, Khin Saw Myint, Amin Soebandrio
EPI_ISL_2101199	Molecular Biology Laboratory, Faculty Medicine and Health Sciences, Warmadewa University	Eijkman Institute for Molecular Biology, National Agency for Research and Innovation; Molecular Biology Laboratory,	Willy Agustine, Hidayat Trimarsanto, Lydia V. Panggalo, Iskandar Adnan, Sukma Oktavianthi, Lidwina Priliani, Edison Johar, Frilasita A Yudhaputri, Muhammad Rezki Rasyak, Sri Masyeni, Erly Sintya, Safarina G Malik, Khin Saw Myint, Amin Soebandrio

		Faculty Medicine and Health Sciences, Warmadewa University	
EPI_ISL_2101200	Molecular Biology Laboratory, Faculty Medicine and Health Sciences, Warmadewa University	Eijkman Institute for Molecular Biology, National Agency for Research and Innovation; Molecular Biology Laboratory, Faculty Medicine and Health Sciences, Warmadewa University	Lydia V. Panggalo, Iskandar Adnan, Sukma Oktavianthi, Lidwina Priliani, Edison Johar, Frilasita A Yudhaputri, Muhammad Rezki Rasyak, Willy Agustine, Hidayat Trimarsanto, Sri Masyeni, Erly Sintya, Safarina G Malik, Khin Saw Myint, Amin Soebandrio
EPI_ISL_2101202	Molecular Biology Laboratory, Faculty Medicine and Health Sciences, Warmadewa University	Eijkman Institute for Molecular Biology, National Agency for Research and Innovation; Molecular Biology Laboratory, Faculty Medicine and Health Sciences, Warmadewa University	Iskandar Adnan, Sukma Oktavianthi, Lidwina Priliani, Edison Johar, Frilasita A Yudhaputri, Muhammad Rezki Rasyak, Willy Agustine, Hidayat Trimarsanto, Lydia V. Panggalo, Sri Masyeni, Erly Sintya, Safarina G Malik, Khin Saw Myint, Amin Soebandrio
EPI_ISL_2101203	Molecular Biology Laboratory, Faculty Medicine and Health Sciences, Warmadewa University	Eijkman Institute for Molecular Biology, National Agency for Research and Innovation; Molecular Biology Laboratory, Faculty Medicine and Health Sciences, Warmadewa University	Sukma Oktavianthi, Lidwina Priliani, Edison Johar, Frilasita A Yudhaputri, Muhammad Rezki Rasyak, Willy Agustine, Hidayat Trimarsanto, Lydia V. Panggalo, Iskandar Adnan, Sri Masyeni, Erly Sintya, Safarina G Malik, Khin Saw Myint, Amin Soebandrio
EPI_ISL_2101204	Molecular Biology Laboratory, Faculty Medicine and Health Sciences, Warmadewa University	Eijkman Institute for Molecular Biology, National Agency for Research and Innovation; Molecular Biology Laboratory, Faculty Medicine and Health Sciences, Warmadewa University	Lidwina Priliani, Edison Johar, Frilasita A Yudhaputri, Muhammad Rezki Rasyak, Willy Agustine, Hidayat Trimarsanto, Lydia V. Panggalo, Iskandar Adnan, Sukma Oktavianthi, Sri Masyeni, Erly Sintya, Safarina G Malik, Khin Saw Myint, Amin Soebandrio
EPI_ISL_2101205	Molecular Biology Laboratory, Faculty Medicine and Health Sciences, Warmadewa University	Eijkman Institute for Molecular Biology, National Agency for Research and Innovation; Molecular Biology Laboratory, Faculty Medicine and Health Sciences, Warmadewa University	Edison Johar, Frilasita A Yudhaputri, Muhammad Rezki Rasyak, Willy Agustine, Hidayat Trimarsanto, Lydia V. Panggalo, Iskandar Adnan, Sukma Oktavianthi, Lidwina Priliani, Sri Masyeni, Erly Sintya, Safarina G Malik, Khin Saw Myint, Amin Soebandrio
EPI_ISL_2101206	Molecular Biology Laboratory, Faculty Medicine and Health Sciences, Warmadewa University	Eijkman Institute for Molecular Biology, National Agency for Research and Innovation; Molecular Biology Laboratory, Faculty Medicine and Health Sciences, Warmadewa University	Frilasita A Yudhaputri, Muhammad Rezki Rasyak, Willy Agustine, Hidayat Trimarsanto, Lydia V. Panggalo, Iskandar Adnan, Sukma Oktavianthi, Lidwina Priliani, Edison Johar, Sri Masyeni, Erly Sintya, Safarina G Malik, Khin Saw Myint, Amin Soebandrio
EPI_ISL_2101207	Molecular Biology Laboratory, Faculty Medicine and Health Sciences, Warmadewa University	Eijkman Institute for Molecular Biology, National Agency for Research and Innovation; Molecular Biology Laboratory, Faculty Medicine and Health Sciences, Warmadewa University	Willy Agustine, Hidayat Trimarsanto, Lydia V. Panggalo, Iskandar Adnan, Sukma Oktavianthi, Lidwina Priliani, Edison Johar, Frilasita A Yudhaputri, Muhammad Rezki Rasyak, Sri Masyeni, Erly Sintya, Safarina G Malik, Khin Saw Myint, Amin Soebandrio
EPI_ISL_2101208	Molecular Biology Laboratory, Faculty Medicine and Health Sciences, Warmadewa University	Eijkman Institute for Molecular Biology, National Agency for Research and Innovation; Molecular Biology Laboratory, Faculty Medicine and Health Sciences, Warmadewa University	Hidayat Trimarsanto, Lydia V. Panggalo, Iskandar Adnan, Sukma Oktavianthi, Lidwina Priliani, Edison Johar, Frilasita A Yudhaputri, Muhammad Rezki Rasyak, Willy Agustine, Sri Masyeni, Erly Sintya, Safarina G Malik, Khin Saw Myint, Amin Soebandrio
EPI_ISL_2101209	Molecular Biology Laboratory, Faculty Medicine and Health Sciences, Warmadewa University	Eijkman Institute for Molecular Biology, National Agency for Research and Innovation; Molecular Biology Laboratory, Faculty Medicine and Health Sciences, Warmadewa University	Lydia V. Panggalo, Iskandar Adnan, Sukma Oktavianthi, Lidwina Priliani, Edison Johar, Frilasita A Yudhaputri, Muhammad Rezki Rasyak, Willy Agustine, Hidayat Trimarsanto, Sri Masyeni, Erly Sintya, Safarina G Malik, Khin Saw Myint, Amin Soebandrio
EPI_ISL_2101210	Molecular Biology Laboratory, Faculty Medicine and Health Sciences, Warmadewa University	Eijkman Institute for Molecular Biology, National Agency for Research and Innovation; Molecular Biology Laboratory, Faculty Medicine and Health Sciences, Warmadewa University	Iskandar Adnan, Sukma Oktavianthi, Lidwina Priliani, Edison Johar, Frilasita A Yudhaputri, Muhammad Rezki Rasyak, Willy Agustine, Hidayat Trimarsanto, Lydia V. Panggalo, Sri Masyeni, Erly Sintya, Safarina G Malik, Khin Saw Myint, Amin Soebandrio
EPI_ISL_2101211	Molecular Biology Laboratory, Faculty Medicine and Health Sciences, Warmadewa University	Eijkman Institute for Molecular Biology, National Agency for Research and Innovation; Molecular Biology Laboratory, Faculty Medicine and Health Sciences, Warmadewa University	Lidwina Priliani, Edison Johar, Frilasita A Yudhaputri, Muhammad Rezki Rasyak, Willy Agustine, Hidayat Trimarsanto, Lydia V. Panggalo, Iskandar Adnan, Sukma Oktavianthi, Sri Masyeni, Erly Sintya, Safarina G Malik, Khin Saw Myint, Amin Soebandrio
EPI_ISL_2101212	Molecular Biology Laboratory, Faculty Medicine and Health Sciences, Warmadewa University	Eijkman Institute for Molecular Biology, National Agency for Research and Innovation; Molecular Biology Laboratory, Faculty Medicine and Health Sciences, Warmadewa University	Edison Johar, Frilasita A Yudhaputri, Muhammad Rezki Rasyak, Willy Agustine, Hidayat Trimarsanto, Lydia V. Panggalo, Iskandar Adnan, Sukma Oktavianthi, Lidwina Priliani, Sri Masyeni, Erly Sintya, Safarina G Malik, Khin Saw Myint, Amin Soebandrio
EPI_ISL_2101213	Molecular Biology Laboratory, Faculty Medicine and Health Sciences, Warmadewa University	Eijkman Institute for Molecular Biology, National Agency for Research and Innovation; Molecular Biology Laboratory, Faculty Medicine and Health Sciences, Warmadewa University	Frilasita A Yudhaputri, Muhammad Rezki Rasyak, Willy Agustine, Hidayat Trimarsanto, Lydia V. Panggalo, Iskandar Adnan, Sukma Oktavianthi, Lidwina Priliani, Edison Johar, Sri Masyeni, Erly Sintya, Safarina G Malik, Khin Saw Myint, Amin Soebandrio
EPI_ISL_2101214	Molecular Biology Laboratory, Faculty Medicine and Health Sciences, Warmadewa University	Eijkman Institute for Molecular Biology, National Agency for Research and Innovation; Molecular Biology Laboratory, Faculty Medicine and Health Sciences, Warmadewa University	Willy Agustine, Hidayat Trimarsanto, Lydia V. Panggalo, Iskandar Adnan, Sukma Oktavianthi, Lidwina Priliani, Edison Johar, Frilasita A Yudhaputri, Muhammad Rezki Rasyak, Sri Masyeni, Erly Sintya, Safarina G Malik, Khin Saw Myint, Amin Soebandrio
EPI_ISL_2101215	Molecular Biology Laboratory, Faculty Medicine and Health Sciences, Warmadewa University	Eijkman Institute for Molecular Biology, National Agency for Research and Innovation; Molecular Biology Laboratory, Faculty Medicine and Health Sciences, Warmadewa University	Hidayat Trimarsanto, Lydia V. Panggalo, Iskandar Adnan, Sukma Oktavianthi, Lidwina Priliani, Edison Johar, Frilasita A Yudhaputri, Muhammad Rezki Rasyak, Willy Agustine, Sri Masyeni, Erly Sintya, Safarina G Malik, Khin Saw Myint, Amin Soebandrio
EPI_ISL_2101236	RSUD Klungkung	Eijkman Institute for Molecular Biology, National Agency for Research and Innovation; Molecular Biology Laboratory, Faculty Medicine and Health Sciences, Warmadewa University	Frilasita A Yudhaputri, Muhammad Rezki Rasyak, Willy Agustine, Hidayat Trimarsanto, Lydia V. Panggalo, Iskandar Adnan, Sukma Oktavianthi, Lidwina Priliani, Edison Johar, Sri Masyeni, Erly Sintya, I Komang Parwata, Safarina G Malik, Khin Saw Myint, Amin Soebandrio
EPI_ISL_2101237	RSUD Klungkung	Eijkman Institute for Molecular Biology, National Agency for Research and Innovation; Molecular Biology Laboratory, Faculty Medicine and Health Sciences, Warmadewa University	Willy Agustine, Hidayat Trimarsanto, Lydia V. Panggalo, Iskandar Adnan, Sukma Oktavianthi, Lidwina Priliani, Edison Johar, Frilasita A Yudhaputri, Muhammad Rezki Rasyak, Sri Masyeni, Erly Sintya, I Komang Parwata, Safarina G Malik, Khin Saw Myint, Amin Soebandrio
EPI_ISL_2101238	RSUD Klungkung	Eijkman Institute for Molecular Biology, National Agency for Research and Innovation; Molecular Biology Laboratory, Faculty Medicine and Health Sciences, Warmadewa University	Iskandar Adnan, Sukma Oktavianthi, Lidwina Priliani, Edison Johar, Frilasita A Yudhaputri, Muhammad Rezki Rasyak, Willy Agustine, Hidayat Trimarsanto, Lydia V. Panggalo, Sri Masyeni, Erly Sintya, I Komang Parwata, Safarina G Malik, Khin Saw Myint, Amin Soebandrio
EPI_ISL_2101239	RSUD Klungkung	Eijkman Institute for Molecular Biology, National Agency for Research and Innovation; Molecular Biology Laboratory, Faculty Medicine and Health Sciences, Warmadewa University	Lidwina Priliani, Edison Johar, Frilasita A Yudhaputri, Muhammad Rezki Rasyak, Willy Agustine, Hidayat Trimarsanto, Lydia V. Panggalo, Iskandar Adnan, Sukma Oktavianthi, Sri Masyeni, Erly Sintya, I Komang Parwata, Safarina G Malik, Khin Saw Myint, Amin Soebandrio

EPI_ISL_2101240	RSUD Klungkung	Eijkman Institute for Molecular Biology, National Agency for Research and Innovation; Molecular Biology Laboratory, Faculty Medicine and Health Sciences, Warmadewa University	Edison Johar, Frilasita A Yudhaputri, Muhammad Rezki Rasyak, Willy Agustine, Hidayat Trimarsanto, Lydia V. Panggalo, Iskandar Adnan, Sukma Oktavianthi, Lidwina Priliani, Sri Masyeni, Erly Sintya, I Komang Parwata, Safarina G Malik, Khin Saw Myint, Amin Soebandrio
EPI_ISL_2101241	RSUD Klungkung	Eijkman Institute for Molecular Biology, National Agency for Research and Innovation; Molecular Biology Laboratory, Faculty Medicine and Health Sciences, Warmadewa University	Frilasita A Yudhaputri, Muhammad Rezki Rasyak, Willy Agustine, Hidayat Trimarsanto, Lydia V. Panggalo, Iskandar Adnan, Sukma Oktavianthi, Lidwina Priliani, Edison Johar, Sri Masyeni, Erly Sintya, I Komang Parwata, Safarina G Malik, Khin Saw Myint, Amin Soebandrio
EPI_ISL_2101242	RSUD Klungkung	Eijkman Institute for Molecular Biology, National Agency for Research and Innovation; Molecular Biology Laboratory, Faculty Medicine and Health Sciences, Warmadewa University	Muhammad Rezki Rasyak, Willy Agustine, Hidayat Trimarsanto, Lydia V. Panggalo, Iskandar Adnan, Sukma Oktavianthi, Lidwina Priliani, Edison Johar, Frilasita A Yudhaputri, Sri Masyeni, Erly Sintya, I Komang Parwata, Safarina G Malik, Khin Saw Myint, Amin Soebandrio
EPI_ISL_2101243	RSUD Klungkung	Eijkman Institute for Molecular Biology, National Agency for Research and Innovation; Molecular Biology Laboratory, Faculty Medicine and Health Sciences, Warmadewa University	Willy Agustine, Hidayat Trimarsanto, Lydia V. Panggalo, Iskandar Adnan, Sukma Oktavianthi, Lidwina Priliani, Edison Johar, Frilasita A Yudhaputri, Muhammad Rezki Rasyak, Sri Masyeni, Erly Sintya, I Komang Parwata, Safarina G Malik, Khin Saw Myint, Amin Soebandrio
EPI_ISL_2101244	RSUD Klungkung	Eijkman Institute for Molecular Biology, National Agency for Research and Innovation; Molecular Biology Laboratory, Faculty Medicine and Health Sciences, Warmadewa University	Lydia V. Panggalo, Iskandar Adnan, Sukma Oktavianthi, Lidwina Priliani, Edison Johar, Frilasita A Yudhaputri, Muhammad Rezki Rasyak, Willy Agustine, Hidayat Trimarsanto, Sri Masyeni, Erly Sintya, I Komang Parwata, Safarina G Malik, Khin Saw Myint, Amin Soebandrio
EPI_ISL_2101245	RSUD Klungkung	Eijkman Institute for Molecular Biology, National Agency for Research and Innovation; Molecular Biology Laboratory, Faculty Medicine and Health Sciences, Warmadewa University	Iskandar Adnan, Sukma Oktavianthi, Lidwina Priliani, Edison Johar, Frilasita A Yudhaputri, Muhammad Rezki Rasyak, Willy Agustine, Hidayat Trimarsanto, Lydia V. Panggalo, Sri Masyeni, Erly Sintya, I Komang Parwata, Safarina G Malik, Khin Saw Myint, Amin Soebandrio
EPI_ISL_2101256	RSUD Mangusada	Eijkman Institute for Molecular Biology, National Agency for Research and Innovation; Molecular Biology Laboratory, Faculty Medicine and Health Sciences, Warmadewa University	Edison Johar, Frilasita A Yudhaputri, Muhammad Rezki Rasyak, Willy Agustine, Hidayat Trimarsanto, Lydia V. Panggalo, Iskandar Adnan, Sukma Oktavianthi, Lidwina Priliani, Sri Masyeni, Erly Sintya, Ida Ayu Wayan Mahayani, Safarina G Malik, Khin Saw Myint, Amin Soebandrio
EPI_ISL_2101257	RSUD Mangusada	Eijkman Institute for Molecular Biology, National Agency for Research and Innovation; Molecular Biology Laboratory, Faculty Medicine and Health Sciences, Warmadewa University	Frilasita A Yudhaputri, Muhammad Rezki Rasyak, Willy Agustine, Hidayat Trimarsanto, Lydia V. Panggalo, Iskandar Adnan, Sukma Oktavianthi, Lidwina Priliani, Edison Johar, Sri Masyeni, Erly Sintya, Ida Ayu Wayan Mahayani, Safarina G Malik, Khin Saw Myint, Amin Soebandrio
EPI_ISL_2101258	RSUD Mangusada	Eijkman Institute for Molecular Biology, National Agency for Research and Innovation; Molecular Biology Laboratory, Faculty Medicine and Health Sciences, Warmadewa University	Muhammad Rezki Rasyak, Willy Agustine, Hidayat Trimarsanto, Lydia V. Panggalo, Iskandar Adnan, Sukma Oktavianthi, Lidwina Priliani, Edison Johar, Frilasita A Yudhaputri, Sri Masyeni, Erly Sintya, Ida Ayu Wayan Mahayani, Safarina G Malik, Khin Saw Myint, Amin Soebandrio
EPI_ISL_2101259	RSUD Mangusada	Eijkman Institute for Molecular Biology, National Agency for Research and Innovation; Molecular Biology Laboratory, Faculty Medicine and Health Sciences, Warmadewa University	Willy Agustine, Hidayat Trimarsanto, Lydia V. Panggalo, Iskandar Adnan, Sukma Oktavianthi, Lidwina Priliani, Edison Johar, Frilasita A Yudhaputri, Muhammad Rezki Rasyak, Sri Masyeni, Erly Sintya, Ida Ayu Wayan Mahayani, Safarina G Malik, Khin Saw Myint, Amin Soebandrio
EPI_ISL_2101260	RSUD Mangusada	Eijkman Institute for Molecular Biology, National Agency for Research and Innovation; Molecular Biology Laboratory, Faculty Medicine and Health Sciences, Warmadewa University	Hidayat Trimarsanto, Lydia V. Panggalo, Iskandar Adnan, Sukma Oktavianthi, Lidwina Priliani, Edison Johar, Frilasita A Yudhaputri, Muhammad Rezki Rasyak, Willy Agustine, Sri Masyeni, Erly Sintya, Ida Ayu Wayan Mahayani, Safarina G Malik, Khin Saw Myint, Amin Soebandrio
EPI_ISL_2101261	RSUD Mangusada	Eijkman Institute for Molecular Biology, National Agency for Research and Innovation; Molecular Biology Laboratory, Faculty Medicine and Health Sciences, Warmadewa University	Lydia V. Panggalo, Iskandar Adnan, Sukma Oktavianthi, Lidwina Priliani, Edison Johar, Frilasita A Yudhaputri, Muhammad Rezki Rasyak, Willy Agustine, Hidayat Trimarsanto, Sri Masyeni, Erly Sintya, Ida Ayu Wayan Mahayani, Safarina G Malik, Khin Saw Myint, Amin Soebandrio
EPI_ISL_2101262	RSUD Mangusada	Eijkman Institute for Molecular Biology, National Agency for Research and Innovation; Molecular Biology Laboratory, Faculty Medicine and Health Sciences, Warmadewa University	Iskandar Adnan, Sukma Oktavianthi, Lidwina Priliani, Edison Johar, Frilasita A Yudhaputri, Muhammad Rezki Rasyak, Willy Agustine, Hidayat Trimarsanto, Lydia V. Panggalo, Sri Masyeni, Erly Sintya, Ida Ayu Wayan Mahayani, Safarina G Malik, Khin Saw Myint, Amin Soebandrio
EPI_ISL_2101263	RSUD Mangusada	Eijkman Institute for Molecular Biology, National Agency for Research and Innovation; Molecular Biology Laboratory, Faculty Medicine and Health Sciences, Warmadewa University	Sukma Oktavianthi, Lidwina Priliani, Edison Johar, Frilasita A Yudhaputri, Muhammad Rezki Rasyak, Willy Agustine, Hidayat Trimarsanto, Lydia V. Panggalo, Iskandar Adnan, Sri Masyeni, Erly Sintya, Ida Ayu Wayan Mahayani, Safarina G Malik, Khin Saw Myint, Amin Soebandrio
EPI_ISL_2101264	RSUD Mangusada	Eijkman Institute for Molecular Biology, National Agency for Research and Innovation; Molecular Biology Laboratory, Faculty Medicine and Health Sciences, Warmadewa University	Lidwina Priliani, Edison Johar, Frilasita A Yudhaputri, Muhammad Rezki Rasyak, Willy Agustine, Hidayat Trimarsanto, Lydia V. Panggalo, Iskandar Adnan, Sukma Oktavianthi, Sri Masyeni, Erly Sintya, Ida Ayu Wayan Mahayani, Safarina G Malik, Khin Saw Myint, Amin Soebandrio
EPI_ISL_2101265	RSUD Mangusada	Eijkman Institute for Molecular Biology, National Agency for Research and Innovation; Molecular Biology Laboratory, Faculty Medicine and Health Sciences, Warmadewa University	Edison Johar, Frilasita A Yudhaputri, Muhammad Rezki Rasyak, Willy Agustine, Hidayat Trimarsanto, Lydia V. Panggalo, Iskandar Adnan, Sukma Oktavianthi, Lidwina Priliani, Sri Masyeni, Erly Sintya, Ida Ayu Wayan Mahayani, Safarina G Malik, Khin Saw Myint, Amin Soebandrio
EPI_ISL_2101266	RSUD Mangusada	Eijkman Institute for Molecular Biology, National Agency for Research and Innovation; Molecular Biology Laboratory, Faculty Medicine and Health Sciences, Warmadewa University	Frilasita A Yudhaputri, Muhammad Rezki Rasyak, Willy Agustine, Hidayat Trimarsanto, Lydia V. Panggalo, Iskandar Adnan, Sukma Oktavianthi, Lidwina Priliani, Edison Johar, Sri Masyeni, Erly Sintya, Ida Ayu Wayan Mahayani, Safarina G Malik, Khin Saw Myint, Amin Soebandrio
EPI_ISL_2101267	RSUD Mangusada	Eijkman Institute for Molecular Biology, National Agency for Research and Innovation; Molecular Biology Laboratory, Faculty Medicine and Health Sciences, Warmadewa University	Muhammad Rezki Rasyak, Willy Agustine, Hidayat Trimarsanto, Lydia V. Panggalo, Iskandar Adnan, Sukma Oktavianthi, Lidwina Priliani, Edison Johar, Frilasita A Yudhaputri, Sri Masyeni, Erly Sintya, Ida Ayu Wayan Mahayani, Safarina G Malik, Khin Saw Myint, Amin Soebandrio
EPI_ISL_2101268	RSUD Mangusada	Eijkman Institute for Molecular Biology, National Agency for Research and Innovation; Molecular Biology Laboratory, Faculty Medicine and Health Sciences, Warmadewa University	Willy Agustine, Hidayat Trimarsanto, Lydia V. Panggalo, Iskandar Adnan, Sukma Oktavianthi, Lidwina Priliani, Edison Johar, Frilasita A Yudhaputri, Muhammad Rezki Rasyak, Sri Masyeni, Erly Sintya, Ida Ayu Wayan Mahayani, Safarina G Malik, Khin Saw Myint, Amin Soebandrio
EPI_ISL_2101269	RSUD Mangusada	Eijkman Institute for Molecular Biology, National Agency for Research and Innovation; Molecular Biology Laboratory,	Hidayat Trimarsanto, Lydia V. Panggalo, Iskandar Adnan, Sukma Oktavianthi, Lidwina Priliani, Edison Johar, Frilasita A Yudhaputri, Muhammad Rezki Rasyak, Willy Agustine, Sri Masyeni, Erly Sintya, Ida Ayu Wayan Mahayani, Safarina G Malik, Khin Saw Myint, Amin Soebandrio

		Faculty Medicine and Health Sciences, Warmadewa University	
EPI_ISL_2101270	RSUD Mangusada	Eijkman Institute for Molecular Biology, National Agency for Research and Innovation; Molecular Biology Laboratory, Faculty Medicine and Health Sciences, Warmadewa University	Lydia V. Panggalo, Iskandar Adnan, Sukma Oktavianthi, Lidwina Priliani, Edison Johar, Frilasita A Yudhaputri, Muhammad Rezki Rasyak, Willy Agustine, Hidayat Trimarsanto, Sri Masyeni, Erly Sintya, Ida Ayu Wayan Mahayani, Safarina G Malik, Khin Saw Myint, Amin Soebandrio
EPI_ISL_2101271	RSUD Mangusada	Eijkman Institute for Molecular Biology, National Agency for Research and Innovation; Molecular Biology Laboratory, Faculty Medicine and Health Sciences, Warmadewa University	Iskandar Adnan, Sukma Oktavianthi, Lidwina Priliani, Edison Johar, Frilasita A Yudhaputri, Muhammad Rezki Rasyak, Willy Agustine, Hidayat Trimarsanto, Lydia V. Panggalo, Sri Masyeni, Erly Sintya, Ida Ayu Wayan Mahayani, Safarina G Malik, Khin Saw Myint, Amin Soebandrio
EPI_ISL_2101272	RSUD Mangusada	Eijkman Institute for Molecular Biology, National Agency for Research and Innovation; Molecular Biology Laboratory, Faculty Medicine and Health Sciences, Warmadewa University	Sukma Oktavianthi, Lidwina Priliani, Edison Johar, Frilasita A Yudhaputri, Muhammad Rezki Rasyak, Willy Agustine, Hidayat Trimarsanto, Lydia V. Panggalo, Iskandar Adnan, Sri Masyeni, Erly Sintya, Ida Ayu Wayan Mahayani, Safarina G Malik, Khin Saw Myint, Amin Soebandrio
EPI_ISL_2101273	RSUD Mangusada	Eijkman Institute for Molecular Biology, National Agency for Research and Innovation; Molecular Biology Laboratory, Faculty Medicine and Health Sciences, Warmadewa University	Lidwina Priliani, Edison Johar, Frilasita A Yudhaputri, Muhammad Rezki Rasyak, Willy Agustine, Hidayat Trimarsanto, Lydia V. Panggalo, Iskandar Adnan, Sukma Oktavianthi, Sri Masyeni, Erly Sintya, Ida Ayu Wayan Mahayani, Safarina G Malik, Khin Saw Myint, Amin Soebandrio
EPI_ISL_2101274	RSUD Mangusada	Eijkman Institute for Molecular Biology, National Agency for Research and Innovation; Molecular Biology Laboratory, Faculty Medicine and Health Sciences, Warmadewa University	Edison Johar, Frilasita A Yudhaputri, Muhammad Rezki Rasyak, Willy Agustine, Hidayat Trimarsanto, Lydia V. Panggalo, Iskandar Adnan, Sukma Oktavianthi, Lidwina Priliani, Sri Masyeni, Erly Sintya, Ida Ayu Wayan Mahayani, Safarina G Malik, Khin Saw Myint, Amin Soebandrio
EPI_ISL_2101275	RSUD Mangusada	Eijkman Institute for Molecular Biology, National Agency for Research and Innovation; Molecular Biology Laboratory, Faculty Medicine and Health Sciences, Warmadewa University	Frilasita A Yudhaputri, Muhammad Rezki Rasyak, Willy Agustine, Hidayat Trimarsanto, Lydia V. Panggalo, Iskandar Adnan, Sukma Oktavianthi, Lidwina Priliani, Edison Johar, Sri Masyeni, Erly Sintya, Ida Ayu Wayan Mahayani, Safarina G Malik, Khin Saw Myint, Amin Soebandrio
EPI_ISL_2101276	RSUD Mangusada	Eijkman Institute for Molecular Biology, National Agency for Research and Innovation; Molecular Biology Laboratory, Faculty Medicine and Health Sciences, Warmadewa University	Muhammad Rezki Rasyak, Willy Agustine, Hidayat Trimarsanto, Lydia V. Panggalo, Iskandar Adnan, Sukma Oktavianthi, Lidwina Priliani, Edison Johar, Frilasita A Yudhaputri, Sri Masyeni, Erly Sintya, Ida Ayu Wayan Mahayani, Safarina G Malik, Khin Saw Myint, Amin Soebandrio
EPI_ISL_2101277	RSUD Mangusada	Eijkman Institute for Molecular Biology, National Agency for Research and Innovation; Molecular Biology Laboratory, Faculty Medicine and Health Sciences, Warmadewa University	Willy Agustine, Hidayat Trimarsanto, Lydia V. Panggalo, Iskandar Adnan, Sukma Oktavianthi, Lidwina Priliani, Edison Johar, Frilasita A Yudhaputri, Muhammad Rezki Rasyak, Sri Masyeni, Erly Sintya, Ida Ayu Wayan Mahayani, Safarina G Malik, Khin Saw Myint, Amin Soebandrio
EPI_ISL_2101278	RSUD Mangusada	Eijkman Institute for Molecular Biology, National Agency for Research and Innovation; Molecular Biology Laboratory, Faculty Medicine and Health Sciences, Warmadewa University	Hidayat Trimarsanto, Lydia V. Panggalo, Iskandar Adnan, Sukma Oktavianthi, Lidwina Priliani, Edison Johar, Frilasita A Yudhaputri, Muhammad Rezki Rasyak, Willy Agustine, Sri Masyeni, Erly Sintya, Ida Ayu Wayan Mahayani, Safarina G Malik, Khin Saw Myint, Amin Soebandrio
EPI_ISL_2101279	RSUD Mangusada	Eijkman Institute for Molecular Biology, National Agency for Research and Innovation; Molecular Biology Laboratory, Faculty Medicine and Health Sciences, Warmadewa University	Lydia V. Panggalo, Iskandar Adnan, Sukma Oktavianthi, Lidwina Priliani, Edison Johar, Frilasita A Yudhaputri, Muhammad Rezki Rasyak, Willy Agustine, Hidayat Trimarsanto, Sri Masyeni, Erly Sintya, Ida Ayu Wayan Mahayani, Safarina G Malik, Khin Saw Myint, Amin Soebandrio
EPI_ISL_2101280	RSUD Mangusada	Eijkman Institute for Molecular Biology, National Agency for Research and Innovation; Molecular Biology Laboratory, Faculty Medicine and Health Sciences, Warmadewa University	Iskandar Adnan, Sukma Oktavianthi, Lidwina Priliani, Edison Johar, Frilasita A Yudhaputri, Muhammad Rezki Rasyak, Willy Agustine, Hidayat Trimarsanto, Lydia V. Panggalo, Sri Masyeni, Erly Sintya, Ida Ayu Wayan Mahayani, Safarina G Malik, Khin Saw Myint, Amin Soebandrio
EPI_ISL_2101281	RSUD Mangusada	Eijkman Institute for Molecular Biology, National Agency for Research and Innovation; Molecular Biology Laboratory, Faculty Medicine and Health Sciences, Warmadewa University	Sukma Oktavianthi, Lidwina Priliani, Edison Johar, Frilasita A Yudhaputri, Muhammad Rezki Rasyak, Willy Agustine, Hidayat Trimarsanto, Lydia V. Panggalo, Iskandar Adnan, Sri Masyeni, Erly Sintya, Ida Ayu Wayan Mahayani, Safarina G Malik, Khin Saw Myint, Amin Soebandrio
EPI_ISL_2101282	RSUD Mangusada	Eijkman Institute for Molecular Biology, National Agency for Research and Innovation; Molecular Biology Laboratory, Faculty Medicine and Health Sciences, Warmadewa University	Lidwina Priliani, Edison Johar, Frilasita A Yudhaputri, Muhammad Rezki Rasyak, Willy Agustine, Hidayat Trimarsanto, Lydia V. Panggalo, Iskandar Adnan, Sukma Oktavianthi, Sri Masyeni, Erly Sintya, Ida Ayu Wayan Mahayani, Safarina G Malik, Khin Saw Myint, Amin Soebandrio
EPI_ISL_2101283	RSUD Mangusada	Eijkman Institute for Molecular Biology, National Agency for Research and Innovation; Molecular Biology Laboratory, Faculty Medicine and Health Sciences, Warmadewa University	Edison Johar, Frilasita A Yudhaputri, Muhammad Rezki Rasyak, Willy Agustine, Hidayat Trimarsanto, Lydia V. Panggalo, Iskandar Adnan, Sukma Oktavianthi, Lidwina Priliani, Sri Masyeni, Erly Sintya, Ida Ayu Wayan Mahayani, Safarina G Malik, Khin Saw Myint, Amin Soebandrio
EPI_ISL_2101284	RSUD Mangusada	Eijkman Institute for Molecular Biology, National Agency for Research and Innovation; Molecular Biology Laboratory, Faculty Medicine and Health Sciences, Warmadewa University	Frilasita A Yudhaputri, Muhammad Rezki Rasyak, Willy Agustine, Hidayat Trimarsanto, Lydia V. Panggalo, Iskandar Adnan, Sukma Oktavianthi, Lidwina Priliani, Edison Johar, Sri Masyeni, Erly Sintya, Ida Ayu Wayan Mahayani, Safarina G Malik, Khin Saw Myint, Amin Soebandrio
EPI_ISL_2101285	RSUD Mangusada	Eijkman Institute for Molecular Biology, National Agency for Research and Innovation; Molecular Biology Laboratory, Faculty Medicine and Health Sciences, Warmadewa University	Muhammad Rezki Rasyak, Willy Agustine, Hidayat Trimarsanto, Lydia V. Panggalo, Iskandar Adnan, Sukma Oktavianthi, Lidwina Priliani, Edison Johar, Frilasita A Yudhaputri, Sri Masyeni, Erly Sintya, Ida Ayu Wayan Mahayani, Safarina G Malik, Khin Saw Myint, Amin Soebandrio
EPI_ISL_2101286	RSUD Mangusada	Eijkman Institute for Molecular Biology, National Agency for Research and Innovation; Molecular Biology Laboratory, Faculty Medicine and Health Sciences, Warmadewa University	Willy Agustine, Hidayat Trimarsanto, Lydia V. Panggalo, Iskandar Adnan, Sukma Oktavianthi, Lidwina Priliani, Edison Johar, Frilasita A Yudhaputri, Muhammad Rezki Rasyak, Sri Masyeni, Erly Sintya, Ida Ayu Wayan Mahayani, Safarina G Malik, Khin Saw Myint, Amin Soebandrio
EPI_ISL_2101287	RSUD Mangusada	Eijkman Institute for Molecular Biology, National Agency for Research and Innovation; Molecular Biology Laboratory, Faculty Medicine and Health Sciences, Warmadewa University	Hidayat Trimarsanto, Lydia V. Panggalo, Iskandar Adnan, Sukma Oktavianthi, Lidwina Priliani, Edison Johar, Frilasita A Yudhaputri, Muhammad Rezki Rasyak, Willy Agustine, Sri Masyeni, Erly Sintya, Ida Ayu Wayan Mahayani, Safarina G Malik, Khin Saw Myint, Amin Soebandrio
EPI_ISL_2101288	RSUD Mangusada	Eijkman Institute for Molecular Biology, National Agency for Research and Innovation; Molecular Biology Laboratory, Faculty Medicine and Health Sciences, Warmadewa University	Lydia V. Panggalo, Iskandar Adnan, Sukma Oktavianthi, Lidwina Priliani, Edison Johar, Frilasita A Yudhaputri, Muhammad Rezki Rasyak, Willy Agustine, Hidayat Trimarsanto, Sri Masyeni, Erly Sintya, Ida Ayu Wayan Mahayani, Safarina G Malik, Khin Saw Myint, Amin Soebandrio

EPI_ISL_2101290	RSUD Mangusada	Eijkman Institute for Molecular Biology, National Agency for Research and Innovation; Molecular Biology Laboratory, Faculty Medicine and Health Sciences, Warmadewa University	Iskandar Adnan, Sukma Oktavianthi, Lidwina Priliani, Edison Johar, Frilasita A Yudhaputri, Muhammad Rezki Rasyak, Willy Agustine, Hidayat Trimarsanto, Lydia V. Panggalo, Sri Masyeni, Erly Sintya, Ida Ayu Wayan Mahayani, Safarina G Malik, Khin Saw Myint, Amin Soebandrio
EPI_ISL_2101291	RSUD Mangusada	Eijkman Institute for Molecular Biology, National Agency for Research and Innovation; Molecular Biology Laboratory, Faculty Medicine and Health Sciences, Warmadewa University	Sukma Oktavianthi, Lidwina Priliani, Edison Johar, Frilasita A Yudhaputri, Muhammad Rezki Rasyak, Willy Agustine, Hidayat Trimarsanto, Lydia V. Panggalo, Iskandar Adnan, Sri Masyeni, Erly Sintya, Ida Ayu Wayan Mahayani, Safarina G Malik, Khin Saw Myint, Amin Soebandrio
EPI_ISL_2105218, EPI_ISL_2105225, EPI_ISL_2105226	Cagayan Valley Medical Center Molecular Laboratory	Philippine Genome Center	Francis A. Tablizo, Kenneth M. Kim, Carlo M. Lapid, Marc Jerrone R. Castro, Maria Sofia L. Yangzon, Benedict A. Maralit, Marc Edsel C. Ayes, Eva Maria Cutiongco-de la Paz, Alethea R. de Guzman, Jan Michael C. Yap, Jo-Hannah S. Llames, Sheila Mae M. Araiza, Kris P. Punayan, Irish Coleen A. Asin, Candice Francheska B. Tambaoan, Asia Louisa U. Chong, Karol Sophia Agape R. Padilla, Rianna Patricia S. Cruz, El King D. Morado, Joshua Gregor A. Dizon, Razel Nikka M. Hao, Arianne A. Zamora, Devon Ray Pacial, Juan Antonio R. Magalang, Marissa Alejandria, Celia Carlos, Anna Ong-Lim, Edsel Maurice Salvaña, John Q. Wong, Jaime C. Montoya, Maria Rosario Singh-Vergeire and Cynthia P. Saloma
EPI_ISL_2105232	South Super Highway Molecular Diagnostic Laboratory	Philippine Genome Center	Francis A. Tablizo, Kenneth M. Kim, Carlo M. Lapid, Marc Jerrone R. Castro, Maria Sofia L. Yangzon, Benedict A. Maralit, Marc Edsel C. Ayes, Eva Maria Cutiongco-de la Paz, Alethea R. de Guzman, Jan Michael C. Yap, Jo-Hannah S. Llames, Sheila Mae M. Araiza, Kris P. Punayan, Irish Coleen A. Asin, Candice Francheska B. Tambaoan, Asia Louisa U. Chong, Karol Sophia Agape R. Padilla, Rianna Patricia S. Cruz, El King D. Morado, Joshua Gregor A. Dizon, Razel Nikka M. Hao, Arianne A. Zamora, Devon Ray Pacial, Juan Antonio R. Magalang, Marissa Alejandria, Celia Carlos, Anna Ong-Lim, Edsel Maurice Salvaña, John Q. Wong, Jaime C. Montoya, Maria Rosario Singh-Vergeire and Cynthia P. Saloma
EPI_ISL_2105242, EPI_ISL_2105245, EPI_ISL_2105253	Cagayan Valley Medical Center Molecular Laboratory	Philippine Genome Center	Francis A. Tablizo, Kenneth M. Kim, Carlo M. Lapid, Marc Jerrone R. Castro, Maria Sofia L. Yangzon, Benedict A. Maralit, Marc Edsel C. Ayes, Eva Maria Cutiongco-de la Paz, Alethea R. de Guzman, Jan Michael C. Yap, Jo-Hannah S. Llames, Sheila Mae M. Araiza, Kris P. Punayan, Irish Coleen A. Asin, Candice Francheska B. Tambaoan, Asia Louisa U. Chong, Karol Sophia Agape R. Padilla, Rianna Patricia S. Cruz, El King D. Morado, Joshua Gregor A. Dizon, Razel Nikka M. Hao, Arianne A. Zamora, Devon Ray Pacial, Juan Antonio R. Magalang, Marissa Alejandria, Celia Carlos, Anna Ong-Lim, Edsel Maurice Salvaña, John Q. Wong, Jaime C. Montoya, Maria Rosario Singh-Vergeire and Cynthia P. Saloma
EPI_ISL_2105264, EPI_ISL_2105266	Southern Philippines Medical Center (SPMC)	Philippine Genome Center	Francis A. Tablizo, Kenneth M. Kim, Carlo M. Lapid, Marc Jerrone R. Castro, Maria Sofia L. Yangzon, Benedict A. Maralit, Marc Edsel C. Ayes, Eva Maria Cutiongco-de la Paz, Alethea R. de Guzman, Jan Michael C. Yap, Jo-Hannah S. Llames, Sheila Mae M. Araiza, Kris P. Punayan, Irish Coleen A. Asin, Candice Francheska B. Tambaoan, Asia Louisa U. Chong, Karol Sophia Agape R. Padilla, Rianna Patricia S. Cruz, El King D. Morado, Joshua Gregor A. Dizon, Razel Nikka M. Hao, Arianne A. Zamora, Devon Ray Pacial, Juan Antonio R. Magalang, Marissa Alejandria, Celia Carlos, Anna Ong-Lim, Edsel Maurice Salvaña, John Q. Wong, Jaime C. Montoya, Maria Rosario Singh-Vergeire and Cynthia P. Saloma
EPI_ISL_2105276	Cagayan Valley Medical Center Molecular Laboratory	Philippine Genome Center	Francis A. Tablizo, Kenneth M. Kim, Carlo M. Lapid, Marc Jerrone R. Castro, Maria Sofia L. Yangzon, Benedict A. Maralit, Marc Edsel C. Ayes, Eva Maria Cutiongco-de la Paz, Alethea R. de Guzman, Jan Michael C. Yap, Jo-Hannah S. Llames, Sheila Mae M. Araiza, Kris P. Punayan, Irish Coleen A. Asin, Candice Francheska B. Tambaoan, Asia Louisa U. Chong, Karol Sophia Agape R. Padilla, Rianna Patricia S. Cruz, El King D. Morado, Joshua Gregor A. Dizon, Razel Nikka M. Hao, Arianne A. Zamora, Devon Ray Pacial, Juan Antonio R. Magalang, Marissa Alejandria, Celia Carlos, Anna Ong-Lim, Edsel Maurice Salvaña, John Q. Wong, Jaime C. Montoya, Maria Rosario Singh-Vergeire and Cynthia P. Saloma
EPI_ISL_2105280	The Lord's Grace Medical and Industrial Clinic	Philippine Genome Center	Francis A. Tablizo, Kenneth M. Kim, Carlo M. Lapid, Marc Jerrone R. Castro, Maria Sofia L. Yangzon, Benedict A. Maralit, Marc Edsel C. Ayes, Eva Maria Cutiongco-de la Paz, Alethea R. de Guzman, Jan Michael C. Yap, Jo-Hannah S. Llames, Sheila Mae M. Araiza, Kris P. Punayan, Irish Coleen A. Asin, Candice Francheska B. Tambaoan, Asia Louisa U. Chong, Karol Sophia Agape R. Padilla, Rianna Patricia S. Cruz, El King D. Morado, Joshua Gregor A. Dizon, Razel Nikka M. Hao, Arianne A. Zamora, Devon Ray Pacial, Juan Antonio R. Magalang, Marissa Alejandria, Celia Carlos, Anna Ong-Lim, Edsel Maurice Salvaña, John Q. Wong, Jaime C. Montoya, Maria Rosario Singh-Vergeire and Cynthia P. Saloma
EPI_ISL_2105282	Cagayan Valley Medical Center Molecular Laboratory	Philippine Genome Center	Francis A. Tablizo, Kenneth M. Kim, Carlo M. Lapid, Marc Jerrone R. Castro, Maria Sofia L. Yangzon, Benedict A. Maralit, Marc Edsel C. Ayes, Eva Maria Cutiongco-de la Paz, Alethea R. de Guzman, Jan Michael C. Yap, Jo-Hannah S. Llames, Sheila Mae M. Araiza, Kris P. Punayan, Irish Coleen A. Asin, Candice Francheska B. Tambaoan, Asia Louisa U. Chong, Karol Sophia Agape R. Padilla, Rianna Patricia S. Cruz, El King D. Morado, Joshua Gregor A. Dizon, Razel Nikka M. Hao, Arianne A. Zamora, Devon Ray Pacial, Juan Antonio R. Magalang, Marissa Alejandria, Celia Carlos, Anna Ong-Lim, Edsel Maurice Salvaña, John Q. Wong, Jaime C. Montoya, Maria Rosario Singh-Vergeire and Cynthia P. Saloma
EPI_ISL_2105289	Research Institute for Tropical Medicine, Inc. (RITM)	Philippine Genome Center	Francis A. Tablizo, Kenneth M. Kim, Carlo M. Lapid, Marc Jerrone R. Castro, Maria Sofia L. Yangzon, Benedict A. Maralit, Marc Edsel C. Ayes, Eva Maria Cutiongco-de la Paz, Alethea R. de Guzman, Jan Michael C. Yap, Jo-Hannah S. Llames, Sheila Mae M. Araiza, Kris P. Punayan, Irish Coleen A. Asin, Candice Francheska B. Tambaoan, Asia Louisa U. Chong, Karol Sophia Agape R. Padilla, Rianna Patricia S. Cruz, El King D. Morado, Joshua Gregor A. Dizon, Razel Nikka M. Hao, Arianne A. Zamora, Devon Ray Pacial, Juan Antonio R. Magalang, Marissa Alejandria, Celia Carlos, Anna Ong-Lim, Edsel Maurice Salvaña, John Q. Wong, Jaime C. Montoya, Maria Rosario Singh-Vergeire and Cynthia P. Saloma
EPI_ISL_2105291	Cagayan Valley Medical Center Molecular Laboratory	Philippine Genome Center	Francis A. Tablizo, Kenneth M. Kim, Carlo M. Lapid, Marc Jerrone R. Castro, Maria Sofia L. Yangzon, Benedict A. Maralit, Marc Edsel C. Ayes, Eva Maria Cutiongco-de la Paz, Alethea R. de Guzman, Jan Michael C. Yap, Jo-Hannah S. Llames, Sheila Mae M. Araiza, Kris P. Punayan, Irish Coleen A. Asin, Candice Francheska B. Tambaoan, Asia Louisa U. Chong, Karol Sophia Agape R. Padilla, Rianna Patricia S. Cruz, El King D. Morado, Joshua Gregor A. Dizon, Razel Nikka M. Hao, Arianne A. Zamora, Devon Ray Pacial, Juan Antonio R. Magalang, Marissa Alejandria, Celia Carlos, Anna Ong-Lim, Edsel Maurice Salvaña, John Q. Wong, Jaime C. Montoya, Maria Rosario Singh-Vergeire and Cynthia P. Saloma
EPI_ISL_2105294, EPI_ISL_2105300, EPI_ISL_2105302, EPI_ISL_2105303	Southern Philippines Medical Center (SPMC-SNL-MBL)	Philippine Genome Center	Francis A. Tablizo, Kenneth M. Kim, Carlo M. Lapid, Marc Jerrone R. Castro, Maria Sofia L. Yangzon, Benedict A. Maralit, Marc Edsel C. Ayes, Eva Maria Cutiongco-de la Paz, Alethea R. de Guzman, Jan Michael C. Yap, Jo-Hannah S. Llames, Sheila Mae M. Araiza, Kris P. Punayan, Irish Coleen A. Asin, Candice Francheska B. Tambaoan, Asia Louisa U. Chong, Karol Sophia Agape R. Padilla, Rianna Patricia S. Cruz, El King D. Morado, Joshua Gregor A. Dizon, Razel Nikka M. Hao, Arianne A. Zamora, Devon Ray Pacial, Juan Antonio R. Magalang, Marissa Alejandria, Celia Carlos, Anna Ong-Lim, Edsel Maurice Salvaña, John Q. Wong, Jaime C. Montoya, Maria Rosario Singh-Vergeire and Cynthia P. Saloma
EPI_ISL_2105306	Dr. Jorge P. Royeca Hospital	Philippine Genome Center	Francis A. Tablizo, Kenneth M. Kim, Carlo M. Lapid, Marc Jerrone R. Castro, Maria Sofia L. Yangzon, Benedict A. Maralit, Marc Edsel C. Ayes, Eva Maria Cutiongco-de la Paz, Alethea R. de Guzman, Jan Michael C. Yap, Jo-Hannah S. Llames, Sheila Mae M. Araiza, Kris P. Punayan, Irish Coleen A. Asin, Candice Francheska B. Tambaoan, Asia Louisa U. Chong, Karol Sophia Agape R. Padilla, Rianna Patricia S. Cruz, El King D. Morado, Joshua Gregor A. Dizon, Razel Nikka M. Hao, Arianne A. Zamora, Devon Ray Pacial, Juan Antonio R. Magalang, Marissa Alejandria, Celia Carlos, Anna Ong-Lim, Edsel Maurice Salvaña, John Q. Wong, Jaime C. Montoya, Maria Rosario Singh-Vergeire and Cynthia P. Saloma
EPI_ISL_2105309, EPI_ISL_2105316, EPI_ISL_2105321, EPI_ISL_2105324, EPI_ISL_2105336	Southern Philippines Medical Center (SPMC-SNL-MBL)	Philippine Genome Center	Francis A. Tablizo, Kenneth M. Kim, Carlo M. Lapid, Marc Jerrone R. Castro, Maria Sofia L. Yangzon, Benedict A. Maralit, Marc Edsel C. Ayes, Eva Maria Cutiongco-de la Paz, Alethea R. de Guzman, Jan Michael C. Yap, Jo-Hannah S. Llames, Sheila Mae M. Araiza, Kris P. Punayan, Irish Coleen A. Asin, Candice Francheska B. Tambaoan, Asia Louisa U. Chong, Karol Sophia Agape R. Padilla, Rianna Patricia S. Cruz, El King D. Morado, Joshua Gregor A. Dizon, Razel Nikka M. Hao, Arianne A. Zamora, Devon Ray Pacial, Juan Antonio R. Magalang, Marissa Alejandria, Celia Carlos, Anna Ong-Lim, Edsel Maurice Salvaña, John Q. Wong, Jaime C. Montoya, Maria Rosario Singh-Vergeire and Cynthia P. Saloma
EPI_ISL_2105337	Research Institute for Tropical Medicine, Inc. (RITM)	Philippine Genome Center	Francis A. Tablizo, Kenneth M. Kim, Carlo M. Lapid, Marc Jerrone R. Castro, Maria Sofia L. Yangzon, Benedict A. Maralit, Marc Edsel C. Ayes, Eva Maria Cutiongco-de la Paz, Alethea R. de Guzman, Jan Michael C. Yap, Jo-Hannah S. Llames, Sheila Mae M. Araiza, Kris P. Punayan, Irish Coleen A. Asin, Candice Francheska B. Tambaoan, Asia Louisa U. Chong, Karol Sophia Agape R. Padilla, Rianna Patricia S. Cruz, El King D. Morado, Joshua Gregor A. Dizon, Razel Nikka M. Hao, Arianne A. Zamora, Devon Ray Pacial, Juan Antonio R. Magalang, Marissa Alejandria, Celia Carlos, Anna Ong-Lim, Edsel Maurice Salvaña, John Q. Wong, Jaime C. Montoya, Maria Rosario Singh-Vergeire and Cynthia P. Saloma
EPI_ISL_2105344, EPI_ISL_2105346, EPI_ISL_2105347, EPI_ISL_2105348, EPI_ISL_2105349, EPI_ISL_2105351, EPI_ISL_2105358	Southern Philippines Medical Center (SPMC-SNL-MBL)	Philippine Genome Center	Francis A. Tablizo, Kenneth M. Kim, Carlo M. Lapid, Marc Jerrone R. Castro, Maria Sofia L. Yangzon, Benedict A. Maralit, Marc Edsel C. Ayes, Eva Maria Cutiongco-de la Paz, Alethea R. de Guzman, Jan Michael C. Yap, Jo-Hannah S. Llames, Sheila Mae M. Araiza, Kris P. Punayan, Irish Coleen A. Asin, Candice Francheska B. Tambaoan, Asia Louisa U. Chong, Karol Sophia Agape R. Padilla, Rianna Patricia S. Cruz, El King D. Morado, Joshua Gregor A. Dizon, Razel Nikka M. Hao, Arianne A. Zamora, Devon Ray Pacial, Juan Antonio R. Magalang, Marissa Alejandria, Celia Carlos, Anna Ong-Lim, Edsel Maurice Salvaña, John Q. Wong, Jaime C. Montoya, Maria Rosario Singh-Vergeire and Cynthia P. Saloma
EPI_ISL_2105360	Dr. Jorge P. Royeca Hospital	Philippine Genome Center	Francis A. Tablizo, Kenneth M. Kim, Carlo M. Lapid, Marc Jerrone R. Castro, Maria Sofia L. Yangzon, Benedict A. Maralit, Marc Edsel C. Ayes, Eva Maria

			Cutiongco-de la Paz, Alethea R. de Guzman, Jan Michael C. Yap, Jo-Hannah S. Llames, Sheila Mae M. Araiza, Kris P. Punayan, Irish Coleen A. Asin, Candice Francheska B. Tambaoan, Asia Louisa U. Chong, Karol Sophia Agape R. Padilla, Rianna Patricia S. Cruz, El King D. Morado, Joshua Gregor A. Dizon, Razel Nikka M. Hao, Arianne A. Zamora, Devon Ray Pacial, Juan Antonio R. Magalang, Marissa Alejandria, Celia Carlos, Anna Ong-Lim, Edsel Maurice Salvaña, John Q. Wong, Jaime C. Montoya, Maria Rosario Singh-Vergeire and Cynthia P. Saloma
EPI_ISL_2105362	Southern Philippines Medical Center (SPMC-SNL-MBL)	Philippine Genome Center	Francis A. Tablizo, Kenneth M. Kim, Carlo M. Lapid, Marc Jerrone R. Castro, Maria Sofia L. Yangzon, Benedict A. Maralit, Marc Edsel C. Ayes, Eva Maria Cutiongco-de la Paz, Alethea R. de Guzman, Jan Michael C. Yap, Jo-Hannah S. Llames, Sheila Mae M. Araiza, Kris P. Punayan, Irish Coleen A. Asin, Candice Francheska B. Tambaoan, Asia Louisa U. Chong, Karol Sophia Agape R. Padilla, Rianna Patricia S. Cruz, El King D. Morado, Joshua Gregor A. Dizon, Razel Nikka M. Hao, Arianne A. Zamora, Devon Ray Pacial, Juan Antonio R. Magalang, Marissa Alejandria, Celia Carlos, Anna Ong-Lim, Edsel Maurice Salvaña, John Q. Wong, Jaime C. Montoya, Maria Rosario Singh-Vergeire and Cynthia P. Saloma
EPI_ISL_2105365	Dr. Jorge P. Royeca Hospital	Philippine Genome Center	Francis A. Tablizo, Kenneth M. Kim, Carlo M. Lapid, Marc Jerrone R. Castro, Maria Sofia L. Yangzon, Benedict A. Maralit, Marc Edsel C. Ayes, Eva Maria Cutiongco-de la Paz, Alethea R. de Guzman, Jan Michael C. Yap, Jo-Hannah S. Llames, Sheila Mae M. Araiza, Kris P. Punayan, Irish Coleen A. Asin, Candice Francheska B. Tambaoan, Asia Louisa U. Chong, Karol Sophia Agape R. Padilla, Rianna Patricia S. Cruz, El King D. Morado, Joshua Gregor A. Dizon, Razel Nikka M. Hao, Arianne A. Zamora, Devon Ray Pacial, Juan Antonio R. Magalang, Marissa Alejandria, Celia Carlos, Anna Ong-Lim, Edsel Maurice Salvaña, John Q. Wong, Jaime C. Montoya, Maria Rosario Singh-Vergeire and Cynthia P. Saloma
EPI_ISL_2105366, EPI_ISL_2105374	Southern Philippines Medical Center (SPMC-SNL-MBL)	Philippine Genome Center	Francis A. Tablizo, Kenneth M. Kim, Carlo M. Lapid, Marc Jerrone R. Castro, Maria Sofia L. Yangzon, Benedict A. Maralit, Marc Edsel C. Ayes, Eva Maria Cutiongco-de la Paz, Alethea R. de Guzman, Jan Michael C. Yap, Jo-Hannah S. Llames, Sheila Mae M. Araiza, Kris P. Punayan, Irish Coleen A. Asin, Candice Francheska B. Tambaoan, Asia Louisa U. Chong, Karol Sophia Agape R. Padilla, Rianna Patricia S. Cruz, El King D. Morado, Joshua Gregor A. Dizon, Razel Nikka M. Hao, Arianne A. Zamora, Devon Ray Pacial, Juan Antonio R. Magalang, Marissa Alejandria, Celia Carlos, Anna Ong-Lim, Edsel Maurice Salvaña, John Q. Wong, Jaime C. Montoya, Maria Rosario Singh-Vergeire and Cynthia P. Saloma
EPI_ISL_2105377	Safeguard DNA Diagnostic, Inc.	Philippine Genome Center	Francis A. Tablizo, Kenneth M. Kim, Carlo M. Lapid, Marc Jerrone R. Castro, Maria Sofia L. Yangzon, Benedict A. Maralit, Marc Edsel C. Ayes, Eva Maria Cutiongco-de la Paz, Alethea R. de Guzman, Jan Michael C. Yap, Jo-Hannah S. Llames, Sheila Mae M. Araiza, Kris P. Punayan, Irish Coleen A. Asin, Candice Francheska B. Tambaoan, Asia Louisa U. Chong, Karol Sophia Agape R. Padilla, Rianna Patricia S. Cruz, El King D. Morado, Joshua Gregor A. Dizon, Razel Nikka M. Hao, Arianne A. Zamora, Devon Ray Pacial, Juan Antonio R. Magalang, Marissa Alejandria, Celia Carlos, Anna Ong-Lim, Edsel Maurice Salvaña, John Q. Wong, Jaime C. Montoya, Maria Rosario Singh-Vergeire and Cynthia P. Saloma
EPI_ISL_2105384, EPI_ISL_2105386, EPI_ISL_2105387, EPI_ISL_2105389, EPI_ISL_2105390, EPI_ISL_2105402	Southern Philippines Medical Center (SPMC-SNL-MBL)	Philippine Genome Center	Francis A. Tablizo, Kenneth M. Kim, Carlo M. Lapid, Marc Jerrone R. Castro, Maria Sofia L. Yangzon, Benedict A. Maralit, Marc Edsel C. Ayes, Eva Maria Cutiongco-de la Paz, Alethea R. de Guzman, Jan Michael C. Yap, Jo-Hannah S. Llames, Sheila Mae M. Araiza, Kris P. Punayan, Irish Coleen A. Asin, Candice Francheska B. Tambaoan, Asia Louisa U. Chong, Karol Sophia Agape R. Padilla, Rianna Patricia S. Cruz, El King D. Morado, Joshua Gregor A. Dizon, Razel Nikka M. Hao, Arianne A. Zamora, Devon Ray Pacial, Juan Antonio R. Magalang, Marissa Alejandria, Celia Carlos, Anna Ong-Lim, Edsel Maurice Salvaña, John Q. Wong, Jaime C. Montoya, Maria Rosario Singh-Vergeire and Cynthia P. Saloma
EPI_ISL_2105404	Negros Oriental Provincial Hospital	Philippine Genome Center	Francis A. Tablizo, Kenneth M. Kim, Carlo M. Lapid, Marc Jerrone R. Castro, Maria Sofia L. Yangzon, Benedict A. Maralit, Marc Edsel C. Ayes, Eva Maria Cutiongco-de la Paz, Alethea R. de Guzman, Jan Michael C. Yap, Jo-Hannah S. Llames, Sheila Mae M. Araiza, Kris P. Punayan, Irish Coleen A. Asin, Candice Francheska B. Tambaoan, Asia Louisa U. Chong, Karol Sophia Agape R. Padilla, Rianna Patricia S. Cruz, El King D. Morado, Joshua Gregor A. Dizon, Razel Nikka M. Hao, Arianne A. Zamora, Devon Ray Pacial, Juan Antonio R. Magalang, Marissa Alejandria, Celia Carlos, Anna Ong-Lim, Edsel Maurice Salvaña, John Q. Wong, Jaime C. Montoya, Maria Rosario Singh-Vergeire and Cynthia P. Saloma
EPI_ISL_2105409	Cagayan Valley Medical Center Molecular Laboratory	Philippine Genome Center	Francis A. Tablizo, Kenneth M. Kim, Carlo M. Lapid, Marc Jerrone R. Castro, Maria Sofia L. Yangzon, Benedict A. Maralit, Marc Edsel C. Ayes, Eva Maria Cutiongco-de la Paz, Alethea R. de Guzman, Jan Michael C. Yap, Jo-Hannah S. Llames, Sheila Mae M. Araiza, Kris P. Punayan, Irish Coleen A. Asin, Candice Francheska B. Tambaoan, Asia Louisa U. Chong, Karol Sophia Agape R. Padilla, Rianna Patricia S. Cruz, El King D. Morado, Joshua Gregor A. Dizon, Razel Nikka M. Hao, Arianne A. Zamora, Devon Ray Pacial, Juan Antonio R. Magalang, Marissa Alejandria, Celia Carlos, Anna Ong-Lim, Edsel Maurice Salvaña, John Q. Wong, Jaime C. Montoya, Maria Rosario Singh-Vergeire and Cynthia P. Saloma
EPI_ISL_2105412, EPI_ISL_2105416	Negros Oriental Provincial Hospital	Philippine Genome Center	Francis A. Tablizo, Kenneth M. Kim, Carlo M. Lapid, Marc Jerrone R. Castro, Maria Sofia L. Yangzon, Benedict A. Maralit, Marc Edsel C. Ayes, Eva Maria Cutiongco-de la Paz, Alethea R. de Guzman, Jan Michael C. Yap, Jo-Hannah S. Llames, Sheila Mae M. Araiza, Kris P. Punayan, Irish Coleen A. Asin, Candice Francheska B. Tambaoan, Asia Louisa U. Chong, Karol Sophia Agape R. Padilla, Rianna Patricia S. Cruz, El King D. Morado, Joshua Gregor A. Dizon, Razel Nikka M. Hao, Arianne A. Zamora, Devon Ray Pacial, Juan Antonio R. Magalang, Marissa Alejandria, Celia Carlos, Anna Ong-Lim, Edsel Maurice Salvaña, John Q. Wong, Jaime C. Montoya, Maria Rosario Singh-Vergeire and Cynthia P. Saloma
EPI_ISL_2105418	Research Institute for Tropical Medicine, Inc. (RITM)	Philippine Genome Center	Francis A. Tablizo, Kenneth M. Kim, Carlo M. Lapid, Marc Jerrone R. Castro, Maria Sofia L. Yangzon, Benedict A. Maralit, Marc Edsel C. Ayes, Eva Maria Cutiongco-de la Paz, Alethea R. de Guzman, Jan Michael C. Yap, Jo-Hannah S. Llames, Sheila Mae M. Araiza, Kris P. Punayan, Irish Coleen A. Asin, Candice Francheska B. Tambaoan, Asia Louisa U. Chong, Karol Sophia Agape R. Padilla, Rianna Patricia S. Cruz, El King D. Morado, Joshua Gregor A. Dizon, Razel Nikka M. Hao, Arianne A. Zamora, Devon Ray Pacial, Juan Antonio R. Magalang, Marissa Alejandria, Celia Carlos, Anna Ong-Lim, Edsel Maurice Salvaña, John Q. Wong, Jaime C. Montoya, Maria Rosario Singh-Vergeire and Cynthia P. Saloma
EPI_ISL_2105419, EPI_ISL_2105422, EPI_ISL_2105423	Negros Oriental Provincial Hospital	Philippine Genome Center	Francis A. Tablizo, Kenneth M. Kim, Carlo M. Lapid, Marc Jerrone R. Castro, Maria Sofia L. Yangzon, Benedict A. Maralit, Marc Edsel C. Ayes, Eva Maria Cutiongco-de la Paz, Alethea R. de Guzman, Jan Michael C. Yap, Jo-Hannah S. Llames, Sheila Mae M. Araiza, Kris P. Punayan, Irish Coleen A. Asin, Candice Francheska B. Tambaoan, Asia Louisa U. Chong, Karol Sophia Agape R. Padilla, Rianna Patricia S. Cruz, El King D. Morado, Joshua Gregor A. Dizon, Razel Nikka M. Hao, Arianne A. Zamora, Devon Ray Pacial, Juan Antonio R. Magalang, Marissa Alejandria, Celia Carlos, Anna Ong-Lim, Edsel Maurice Salvaña, John Q. Wong, Jaime C. Montoya, Maria Rosario Singh-Vergeire and Cynthia P. Saloma
EPI_ISL_2105425	Southern Philippines Medical Center (SPMC-SNL-MBL)	Philippine Genome Center	Francis A. Tablizo, Kenneth M. Kim, Carlo M. Lapid, Marc Jerrone R. Castro, Maria Sofia L. Yangzon, Benedict A. Maralit, Marc Edsel C. Ayes, Eva Maria Cutiongco-de la Paz, Alethea R. de Guzman, Jan Michael C. Yap, Jo-Hannah S. Llames, Sheila Mae M. Araiza, Kris P. Punayan, Irish Coleen A. Asin, Candice Francheska B. Tambaoan, Asia Louisa U. Chong, Karol Sophia Agape R. Padilla, Rianna Patricia S. Cruz, El King D. Morado, Joshua Gregor A. Dizon, Razel Nikka M. Hao, Arianne A. Zamora, Devon Ray Pacial, Juan Antonio R. Magalang, Marissa Alejandria, Celia Carlos, Anna Ong-Lim, Edsel Maurice Salvaña, John Q. Wong, Jaime C. Montoya, Maria Rosario Singh-Vergeire and Cynthia P. Saloma
EPI_ISL_2105427, EPI_ISL_2105431	Negros Oriental Provincial Hospital	Philippine Genome Center	Francis A. Tablizo, Kenneth M. Kim, Carlo M. Lapid, Marc Jerrone R. Castro, Maria Sofia L. Yangzon, Benedict A. Maralit, Marc Edsel C. Ayes, Eva Maria Cutiongco-de la Paz, Alethea R. de Guzman, Jan Michael C. Yap, Jo-Hannah S. Llames, Sheila Mae M. Araiza, Kris P. Punayan, Irish Coleen A. Asin, Candice Francheska B. Tambaoan, Asia Louisa U. Chong, Karol Sophia Agape R. Padilla, Rianna Patricia S. Cruz, El King D. Morado, Joshua Gregor A. Dizon, Razel Nikka M. Hao, Arianne A. Zamora, Devon Ray Pacial, Juan Antonio R. Magalang, Marissa Alejandria, Celia Carlos, Anna Ong-Lim, Edsel Maurice Salvaña, John Q. Wong, Jaime C. Montoya, Maria Rosario Singh-Vergeire and Cynthia P. Saloma
EPI_ISL_2105433	Dr. Jorge P. Royeca Hospital	Philippine Genome Center	Francis A. Tablizo, Kenneth M. Kim, Carlo M. Lapid, Marc Jerrone R. Castro, Maria Sofia L. Yangzon, Benedict A. Maralit, Marc Edsel C. Ayes, Eva Maria Cutiongco-de la Paz, Alethea R. de Guzman, Jan Michael C. Yap, Jo-Hannah S. Llames, Sheila Mae M. Araiza, Kris P. Punayan, Irish Coleen A. Asin, Candice Francheska B. Tambaoan, Asia Louisa U. Chong, Karol Sophia Agape R. Padilla, Rianna Patricia S. Cruz, El King D. Morado, Joshua Gregor A. Dizon, Razel Nikka M. Hao, Arianne A. Zamora, Devon Ray Pacial, Juan Antonio R. Magalang, Marissa Alejandria, Celia Carlos, Anna Ong-Lim, Edsel Maurice Salvaña, John Q. Wong, Jaime C. Montoya, Maria Rosario Singh-Vergeire and Cynthia P. Saloma
EPI_ISL_2105435	Negros Oriental Provincial Hospital	Philippine Genome Center	Francis A. Tablizo, Kenneth M. Kim, Carlo M. Lapid, Marc Jerrone R. Castro, Maria Sofia L. Yangzon, Benedict A. Maralit, Marc Edsel C. Ayes, Eva Maria Cutiongco-de la Paz, Alethea R. de Guzman, Jan Michael C. Yap, Jo-Hannah S. Llames, Sheila Mae M. Araiza, Kris P. Punayan, Irish Coleen A. Asin, Candice Francheska B. Tambaoan, Asia Louisa U. Chong, Karol Sophia Agape R. Padilla, Rianna Patricia S. Cruz, El King D. Morado, Joshua Gregor A. Dizon, Razel Nikka M. Hao, Arianne A. Zamora, Devon Ray Pacial, Juan Antonio R. Magalang, Marissa Alejandria, Celia Carlos, Anna Ong-Lim, Edsel Maurice Salvaña, John Q. Wong, Jaime C. Montoya, Maria Rosario Singh-Vergeire and Cynthia P. Saloma
EPI_ISL_2105448	Cagayan Valley Medical Center Molecular Laboratory	Philippine Genome Center	Francis A. Tablizo, Kenneth M. Kim, Carlo M. Lapid, Marc Jerrone R. Castro, Maria Sofia L. Yangzon, Benedict A. Maralit, Marc Edsel C. Ayes, Eva Maria Cutiongco-de la Paz, Alethea R. de Guzman, Jan Michael C. Yap, Jo-Hannah S. Llames, Sheila Mae M. Araiza, Kris P. Punayan, Irish Coleen A. Asin, Candice Francheska B. Tambaoan, Asia Louisa U. Chong, Karol Sophia Agape R. Padilla, Rianna Patricia S. Cruz, El King D. Morado, Joshua Gregor A. Dizon, Razel Nikka M. Hao, Arianne A. Zamora, Devon Ray Pacial, Juan Antonio R. Magalang, Marissa Alejandria, Celia Carlos, Anna Ong-Lim, Edsel Maurice Salvaña, John Q. Wong, Jaime C. Montoya, Maria Rosario Singh-Vergeire and Cynthia P. Saloma

EPI_ISL_2105450, EPI_ISL_2105451, EPI_ISL_2105452	Negros Oriental Provincial Hospital	Philippine Genome Center	Francis A. Tablizo, Kenneth M. Kim, Carlo M. Lapid, Marc Jerrone R. Castro, Maria Sofia L. Yangzon, Benedict A. Maralit, Marc Edsel C. Ayes, Eva Maria Cutiongco-de la Paz, Alethea R. de Guzman, Jan Michael C. Yap, Jo-Hannah S. Llames, Sheila Mae M. Araiza, Kris P. Punayan, Irish Coleen A. Asin, Candice Francheska B. Tambaoan, Asia Louisa U. Chong, Karol Sophia Agape R. Padilla, Rianna Patricia S. Cruz, El King D. Morado, Joshua Gregor A. Dizon, Razel Nikka M. Hao, Arianne A. Zamora, Devon Ray Pacial, Juan Antonio R. Magalang, Marissa Alejandria, Celia Carlos, Anna Ong-Lim, Edsel Maurice Salvaña, John Q. Wong, Jaime C. Montoya, Maria Rosario Singh-Vergeire and Cynthia P. Saloma
EPI_ISL_2105453	Cagayan Valley Medical Center Molecular Laboratory	Philippine Genome Center	Francis A. Tablizo, Kenneth M. Kim, Carlo M. Lapid, Marc Jerrone R. Castro, Maria Sofia L. Yangzon, Benedict A. Maralit, Marc Edsel C. Ayes, Eva Maria Cutiongco-de la Paz, Alethea R. de Guzman, Jan Michael C. Yap, Jo-Hannah S. Llames, Sheila Mae M. Araiza, Kris P. Punayan, Irish Coleen A. Asin, Candice Francheska B. Tambaoan, Asia Louisa U. Chong, Karol Sophia Agape R. Padilla, Rianna Patricia S. Cruz, El King D. Morado, Joshua Gregor A. Dizon, Razel Nikka M. Hao, Arianne A. Zamora, Devon Ray Pacial, Juan Antonio R. Magalang, Marissa Alejandria, Celia Carlos, Anna Ong-Lim, Edsel Maurice Salvaña, John Q. Wong, Jaime C. Montoya, Maria Rosario Singh-Vergeire and Cynthia P. Saloma
EPI_ISL_2105460	Negros Oriental Provincial Hospital	Philippine Genome Center	Francis A. Tablizo, Kenneth M. Kim, Carlo M. Lapid, Marc Jerrone R. Castro, Maria Sofia L. Yangzon, Benedict A. Maralit, Marc Edsel C. Ayes, Eva Maria Cutiongco-de la Paz, Alethea R. de Guzman, Jan Michael C. Yap, Jo-Hannah S. Llames, Sheila Mae M. Araiza, Kris P. Punayan, Irish Coleen A. Asin, Candice Francheska B. Tambaoan, Asia Louisa U. Chong, Karol Sophia Agape R. Padilla, Rianna Patricia S. Cruz, El King D. Morado, Joshua Gregor A. Dizon, Razel Nikka M. Hao, Arianne A. Zamora, Devon Ray Pacial, Juan Antonio R. Magalang, Marissa Alejandria, Celia Carlos, Anna Ong-Lim, Edsel Maurice Salvaña, John Q. Wong, Jaime C. Montoya, Maria Rosario Singh-Vergeire and Cynthia P. Saloma
EPI_ISL_2105471, EPI_ISL_2105483, EPI_ISL_2105502	Governor Celestino Gallares Memorial Hospital	Philippine Genome Center	Francis A. Tablizo, Kenneth M. Kim, Carlo M. Lapid, Marc Jerrone R. Castro, Maria Sofia L. Yangzon, Benedict A. Maralit, Marc Edsel C. Ayes, Eva Maria Cutiongco-de la Paz, Alethea R. de Guzman, Jan Michael C. Yap, Jo-Hannah S. Llames, Sheila Mae M. Araiza, Kris P. Punayan, Irish Coleen A. Asin, Candice Francheska B. Tambaoan, Asia Louisa U. Chong, Karol Sophia Agape R. Padilla, Rianna Patricia S. Cruz, El King D. Morado, Joshua Gregor A. Dizon, Razel Nikka M. Hao, Arianne A. Zamora, Devon Ray Pacial, Juan Antonio R. Magalang, Marissa Alejandria, Celia Carlos, Anna Ong-Lim, Edsel Maurice Salvaña, John Q. Wong, Jaime C. Montoya, Maria Rosario Singh-Vergeire and Cynthia P. Saloma
EPI_ISL_2105513, EPI_ISL_2105518, EPI_ISL_2105520	Army General Hospital Molecular Laboratory	Philippine Genome Center	Francis A. Tablizo, Kenneth M. Kim, Carlo M. Lapid, Marc Jerrone R. Castro, Maria Sofia L. Yangzon, Benedict A. Maralit, Marc Edsel C. Ayes, Eva Maria Cutiongco-de la Paz, Alethea R. de Guzman, Jan Michael C. Yap, Jo-Hannah S. Llames, Sheila Mae M. Araiza, Kris P. Punayan, Irish Coleen A. Asin, Candice Francheska B. Tambaoan, Asia Louisa U. Chong, Karol Sophia Agape R. Padilla, Rianna Patricia S. Cruz, El King D. Morado, Joshua Gregor A. Dizon, Razel Nikka M. Hao, Arianne A. Zamora, Devon Ray Pacial, Juan Antonio R. Magalang, Marissa Alejandria, Celia Carlos, Anna Ong-Lim, Edsel Maurice Salvaña, John Q. Wong, Jaime C. Montoya, Maria Rosario Singh-Vergeire and Cynthia P. Saloma
EPI_ISL_2105555	Governor Celestino Gallares Memorial Hospital	Philippine Genome Center	Francis A. Tablizo, Kenneth M. Kim, Carlo M. Lapid, Marc Jerrone R. Castro, Maria Sofia L. Yangzon, Benedict A. Maralit, Marc Edsel C. Ayes, Eva Maria Cutiongco-de la Paz, Alethea R. de Guzman, Jan Michael C. Yap, Jo-Hannah S. Llames, Sheila Mae M. Araiza, Kris P. Punayan, Irish Coleen A. Asin, Candice Francheska B. Tambaoan, Asia Louisa U. Chong, Karol Sophia Agape R. Padilla, Rianna Patricia S. Cruz, El King D. Morado, Joshua Gregor A. Dizon, Razel Nikka M. Hao, Arianne A. Zamora, Devon Ray Pacial, Juan Antonio R. Magalang, Marissa Alejandria, Celia Carlos, Anna Ong-Lim, Edsel Maurice Salvaña, John Q. Wong, Jaime C. Montoya, Maria Rosario Singh-Vergeire and Cynthia P. Saloma
EPI_ISL_2107800, EPI_ISL_2107802	National Center of Infectious and Parasitic Diseases	National Center of Infectious and Parasitic Diseases	Alexiev, Ivanov, Korsun, Stoitsova, Philipova, Dimitrova, Grigorova L., Hristova, Donchev, Stoykov, Trifonova, Dobrinov, Grigorova I., Kantardjiev
EPI_ISL_2131619, EPI_ISL_2131803, EPI_ISL_2131835, EPI_ISL_2131836, EPI_ISL_2131837, EPI_ISL_2131840	Laboratoire de Virologie - CHU François Mitterrand - Dijon - France	INSERM U1231 - Equipe GAD	Ali SI-MOHAMMED, Yannis DUFFOURD, Hélène GIRAUDON, Lionel PIROTH
EPI_ISL_2150999	Laboratório de Biologia Molecular Jean Piaget	MRCG at LSHTM, Genomics lab	Aladje Balde, Abdul Karim Sesay, Abdoulie Kanteh, Bakary Sanyang, Simão Tchuda Bióté, Rui Inndi, Adul Candé, Faatu Cassama, Milanca Agostinho Cá, Rei José Pereira, Erica Luis Maria Magalhães, Aicha Balde, Bubacar Delgado Pinto Embaló, Edmira Maria da Costa, Paulina Joãozinho da Costa Jarra Manneh, Mariama Kujabi, Dabiri Damilari, Sainabou Laye Ndure
EPI_ISL_2154027	Dr. Jorge P. Royeca Hospital	Philippine Genome Center	Francis A. Tablizo, Kenneth M. Kim, Carlo M. Lapid, Marc Jerrone R. Castro, Maria Sofia L. Yangzon, Benedict A. Maralit, Marc Edsel C. Ayes, Eva Maria Cutiongco-de la Paz, Alethea R. de Guzman, Jan Michael C. Yap, Jo-Hannah S. Llames, Sheila Mae M. Araiza, Kris P. Punayan, Irish Coleen A. Asin, Candice Francheska B. Tambaoan, Asia Louisa U. Chong, Karol Sophia Agape R. Padilla, Rianna Patricia S. Cruz, El King D. Morado, Joshua Gregor A. Dizon, Razel Nikka M. Hao, Arianne A. Zamora, Devon Ray Pacial, Juan Antonio R. Magalang, Marissa Alejandria, Celia Carlos, Anna Ong-Lim, Edsel Maurice Salvaña, John Q. Wong, Jaime C. Montoya, Maria Rosario Singh-Vergeire and Cynthia P. Saloma
EPI_ISL_2154028	Philippine Red Cross Molecular Laboratory	Philippine Genome Center	Francis A. Tablizo, Kenneth M. Kim, Carlo M. Lapid, Marc Jerrone R. Castro, Maria Sofia L. Yangzon, Benedict A. Maralit, Marc Edsel C. Ayes, Eva Maria Cutiongco-de la Paz, Alethea R. de Guzman, Jan Michael C. Yap, Jo-Hannah S. Llames, Sheila Mae M. Araiza, Kris P. Punayan, Irish Coleen A. Asin, Candice Francheska B. Tambaoan, Asia Louisa U. Chong, Karol Sophia Agape R. Padilla, Rianna Patricia S. Cruz, El King D. Morado, Joshua Gregor A. Dizon, Razel Nikka M. Hao, Arianne A. Zamora, Devon Ray Pacial, Juan Antonio R. Magalang, Marissa Alejandria, Celia Carlos, Anna Ong-Lim, Edsel Maurice Salvaña, John Q. Wong, Jaime C. Montoya, Maria Rosario Singh-Vergeire and Cynthia P. Saloma
EPI_ISL_2154029, EPI_ISL_2154030	Baguio General Hospital Medical Center (BGHMC)	Philippine Genome Center	Francis A. Tablizo, Kenneth M. Kim, Carlo M. Lapid, Marc Jerrone R. Castro, Maria Sofia L. Yangzon, Benedict A. Maralit, Marc Edsel C. Ayes, Eva Maria Cutiongco-de la Paz, Alethea R. de Guzman, Jan Michael C. Yap, Jo-Hannah S. Llames, Sheila Mae M. Araiza, Kris P. Punayan, Irish Coleen A. Asin, Candice Francheska B. Tambaoan, Asia Louisa U. Chong, Karol Sophia Agape R. Padilla, Rianna Patricia S. Cruz, El King D. Morado, Joshua Gregor A. Dizon, Razel Nikka M. Hao, Arianne A. Zamora, Devon Ray Pacial, Juan Antonio R. Magalang, Marissa Alejandria, Celia Carlos, Anna Ong-Lim, Edsel Maurice Salvaña, John Q. Wong, Jaime C. Montoya, Maria Rosario Singh-Vergeire and Cynthia P. Saloma
EPI_ISL_2154031	Army General Hospital Molecular Laboratory	Philippine Genome Center	Francis A. Tablizo, Kenneth M. Kim, Carlo M. Lapid, Marc Jerrone R. Castro, Maria Sofia L. Yangzon, Benedict A. Maralit, Marc Edsel C. Ayes, Eva Maria Cutiongco-de la Paz, Alethea R. de Guzman, Jan Michael C. Yap, Jo-Hannah S. Llames, Sheila Mae M. Araiza, Kris P. Punayan, Irish Coleen A. Asin, Candice Francheska B. Tambaoan, Asia Louisa U. Chong, Karol Sophia Agape R. Padilla, Rianna Patricia S. Cruz, El King D. Morado, Joshua Gregor A. Dizon, Razel Nikka M. Hao, Arianne A. Zamora, Devon Ray Pacial, Juan Antonio R. Magalang, Marissa Alejandria, Celia Carlos, Anna Ong-Lim, Edsel Maurice Salvaña, John Q. Wong, Jaime C. Montoya, Maria Rosario Singh-Vergeire and Cynthia P. Saloma
EPI_ISL_2154032, EPI_ISL_2154033	Baguio General Hospital Medical Center (BGHMC)	Philippine Genome Center	Francis A. Tablizo, Kenneth M. Kim, Carlo M. Lapid, Marc Jerrone R. Castro, Maria Sofia L. Yangzon, Benedict A. Maralit, Marc Edsel C. Ayes, Eva Maria Cutiongco-de la Paz, Alethea R. de Guzman, Jan Michael C. Yap, Jo-Hannah S. Llames, Sheila Mae M. Araiza, Kris P. Punayan, Irish Coleen A. Asin, Candice Francheska B. Tambaoan, Asia Louisa U. Chong, Karol Sophia Agape R. Padilla, Rianna Patricia S. Cruz, El King D. Morado, Joshua Gregor A. Dizon, Razel Nikka M. Hao, Arianne A. Zamora, Devon Ray Pacial, Juan Antonio R. Magalang, Marissa Alejandria, Celia Carlos, Anna Ong-Lim, Edsel Maurice Salvaña, John Q. Wong, Jaime C. Montoya, Maria Rosario Singh-Vergeire and Cynthia P. Saloma
EPI_ISL_2154034	Army General Hospital Molecular Laboratory	Philippine Genome Center	Francis A. Tablizo, Kenneth M. Kim, Carlo M. Lapid, Marc Jerrone R. Castro, Maria Sofia L. Yangzon, Benedict A. Maralit, Marc Edsel C. Ayes, Eva Maria Cutiongco-de la Paz, Alethea R. de Guzman, Jan Michael C. Yap, Jo-Hannah S. Llames, Sheila Mae M. Araiza, Kris P. Punayan, Irish Coleen A. Asin, Candice Francheska B. Tambaoan, Asia Louisa U. Chong, Karol Sophia Agape R. Padilla, Rianna Patricia S. Cruz, El King D. Morado, Joshua Gregor A. Dizon, Razel Nikka M. Hao, Arianne A. Zamora, Devon Ray Pacial, Juan Antonio R. Magalang, Marissa Alejandria, Celia Carlos, Anna Ong-Lim, Edsel Maurice Salvaña, John Q. Wong, Jaime C. Montoya, Maria Rosario Singh-Vergeire and Cynthia P. Saloma
EPI_ISL_2154035, EPI_ISL_2154036, EPI_ISL_2154037	Baguio General Hospital Medical Center (BGHMC)	Philippine Genome Center	Francis A. Tablizo, Kenneth M. Kim, Carlo M. Lapid, Marc Jerrone R. Castro, Maria Sofia L. Yangzon, Benedict A. Maralit, Marc Edsel C. Ayes, Eva Maria Cutiongco-de la Paz, Alethea R. de Guzman, Jan Michael C. Yap, Jo-Hannah S. Llames, Sheila Mae M. Araiza, Kris P. Punayan, Irish Coleen A. Asin, Candice Francheska B. Tambaoan, Asia Louisa U. Chong, Karol Sophia Agape R. Padilla, Rianna Patricia S. Cruz, El King D. Morado, Joshua Gregor A. Dizon, Razel Nikka M. Hao, Arianne A. Zamora, Devon Ray Pacial, Juan Antonio R. Magalang, Marissa Alejandria, Celia Carlos, Anna Ong-Lim, Edsel Maurice Salvaña, John Q. Wong, Jaime C. Montoya, Maria Rosario Singh-Vergeire and Cynthia P. Saloma
EPI_ISL_2154038	St. Luke's Medical Center- QC	Philippine Genome Center	Francis A. Tablizo, Kenneth M. Kim, Carlo M. Lapid, Marc Jerrone R. Castro, Maria Sofia L. Yangzon, Benedict A. Maralit, Marc Edsel C. Ayes, Eva Maria Cutiongco-de la Paz, Alethea R. de Guzman, Jan Michael C. Yap, Jo-Hannah S. Llames, Sheila Mae M. Araiza, Kris P. Punayan, Irish Coleen A. Asin, Candice Francheska B. Tambaoan, Asia Louisa U. Chong, Karol Sophia Agape R. Padilla, Rianna Patricia S. Cruz, El King D. Morado, Joshua Gregor A. Dizon, Razel Nikka M. Hao, Arianne A. Zamora, Devon Ray Pacial, Juan Antonio R. Magalang, Marissa Alejandria, Celia Carlos, Anna Ong-Lim, Edsel Maurice Salvaña, John Q. Wong, Jaime C. Montoya, Maria Rosario Singh-Vergeire and Cynthia P. Saloma
EPI_ISL_2154039, EPI_ISL_2154040	Baguio General Hospital Medical Center (BGHMC)	Philippine Genome Center	Francis A. Tablizo, Kenneth M. Kim, Carlo M. Lapid, Marc Jerrone R. Castro, Maria Sofia L. Yangzon, Benedict A. Maralit, Marc Edsel C. Ayes, Eva Maria Cutiongco-de la Paz, Alethea R. de Guzman, Jan Michael C. Yap, Jo-Hannah S. Llames, Sheila Mae M. Araiza, Kris P. Punayan, Irish Coleen A. Asin, Candice

			Francheska B. Tambaoan, Asia Louisa U. Chong, Karol Sophia Agape R. Padilla, Rianna Patricia S. Cruz, El King D. Morado, Joshua Gregor A. Dizon, Razel Nikka M. Hao, Arianne A. Zamora, Devon Ray Pacial, Juan Antonio R. Magalang, Marissa Alejandria, Celia Carlos, Anna Ong-Lim, Edsel Maurice Salvaña, John Q. Wong, Jaime C. Montoya, Maria Rosario Singh-Vergeire and Cynthia P. Saloma
EPI_ISL_2154041	Army General Hospital Molecular Laboratory	Philippine Genome Center	Francis A. Tablizo, Kenneth M. Kim, Carlo M. Lapid, Marc Jerrone R. Castro, Maria Sofia L. Yangzon, Benedict A. Maralit, Marc Edsel C. Ayes, Eva Maria Cutiongco-de la Paz, Alethea R. de Guzman, Jan Michael C. Yap, Jo-Hannah S. Llames, Sheila Mae M. Araiza, Kris P. Punayan, Irish Coleen A. Asin, Candice Francheska B. Tambaoan, Asia Louisa U. Chong, Karol Sophia Agape R. Padilla, Rianna Patricia S. Cruz, El King D. Morado, Joshua Gregor A. Dizon, Razel Nikka M. Hao, Arianne A. Zamora, Devon Ray Pacial, Juan Antonio R. Magalang, Marissa Alejandria, Celia Carlos, Anna Ong-Lim, Edsel Maurice Salvaña, John Q. Wong, Jaime C. Montoya, Maria Rosario Singh-Vergeire and Cynthia P. Saloma
EPI_ISL_2154042, EPI_ISL_2154043	Baguio General Hospital Medical Center (BGHMC)	Philippine Genome Center	Francis A. Tablizo, Kenneth M. Kim, Carlo M. Lapid, Marc Jerrone R. Castro, Maria Sofia L. Yangzon, Benedict A. Maralit, Marc Edsel C. Ayes, Eva Maria Cutiongco-de la Paz, Alethea R. de Guzman, Jan Michael C. Yap, Jo-Hannah S. Llames, Sheila Mae M. Araiza, Kris P. Punayan, Irish Coleen A. Asin, Candice Francheska B. Tambaoan, Asia Louisa U. Chong, Karol Sophia Agape R. Padilla, Rianna Patricia S. Cruz, El King D. Morado, Joshua Gregor A. Dizon, Razel Nikka M. Hao, Arianne A. Zamora, Devon Ray Pacial, Juan Antonio R. Magalang, Marissa Alejandria, Celia Carlos, Anna Ong-Lim, Edsel Maurice Salvaña, John Q. Wong, Jaime C. Montoya, Maria Rosario Singh-Vergeire and Cynthia P. Saloma
EPI_ISL_2154395	Research Institute for Tropical Medicine, Inc. (RITM)	Philippine Genome Center	Francis A. Tablizo, Kenneth M. Kim, Carlo M. Lapid, Marc Jerrone R. Castro, Maria Sofia L. Yangzon, Benedict A. Maralit, Marc Edsel C. Ayes, Eva Maria Cutiongco-de la Paz, Alethea R. de Guzman, Jan Michael C. Yap, Jo-Hannah S. Llames, Sheila Mae M. Araiza, Kris P. Punayan, Irish Coleen A. Asin, Candice Francheska B. Tambaoan, Asia Louisa U. Chong, Karol Sophia Agape R. Padilla, Rianna Patricia S. Cruz, El King D. Morado, Joshua Gregor A. Dizon, Razel Nikka M. Hao, Arianne A. Zamora, Devon Ray Pacial, Juan Antonio R. Magalang, Marissa Alejandria, Celia Carlos, Anna Ong-Lim, Edsel Maurice Salvaña, John Q. Wong, Jaime C. Montoya, Maria Rosario Singh-Vergeire and Cynthia P. Saloma
EPI_ISL_2154415, EPI_ISL_2154419, EPI_ISL_2154422, EPI_ISL_2154424, EPI_ISL_2154427, EPI_ISL_2154431, EPI_ISL_2154459, EPI_ISL_2154472, EPI_ISL_2154478	Cagayan Valley Medical Center Molecular Laboratory	Philippine Genome Center	Francis A. Tablizo, Kenneth M. Kim, Carlo M. Lapid, Marc Jerrone R. Castro, Maria Sofia L. Yangzon, Benedict A. Maralit, Marc Edsel C. Ayes, Eva Maria Cutiongco-de la Paz, Alethea R. de Guzman, Jan Michael C. Yap, Jo-Hannah S. Llames, Sheila Mae M. Araiza, Kris P. Punayan, Irish Coleen A. Asin, Candice Francheska B. Tambaoan, Asia Louisa U. Chong, Karol Sophia Agape R. Padilla, Rianna Patricia S. Cruz, El King D. Morado, Joshua Gregor A. Dizon, Razel Nikka M. Hao, Arianne A. Zamora, Devon Ray Pacial, Juan Antonio R. Magalang, Marissa Alejandria, Celia Carlos, Anna Ong-Lim, Edsel Maurice Salvaña, John Q. Wong, Jaime C. Montoya, Maria Rosario Singh-Vergeire and Cynthia P. Saloma
EPI_ISL_2154490	Cardinal Santos Medical Center	Philippine Genome Center	Francis A. Tablizo, Kenneth M. Kim, Carlo M. Lapid, Marc Jerrone R. Castro, Maria Sofia L. Yangzon, Benedict A. Maralit, Marc Edsel C. Ayes, Eva Maria Cutiongco-de la Paz, Alethea R. de Guzman, Jan Michael C. Yap, Jo-Hannah S. Llames, Sheila Mae M. Araiza, Kris P. Punayan, Irish Coleen A. Asin, Candice Francheska B. Tambaoan, Asia Louisa U. Chong, Karol Sophia Agape R. Padilla, Rianna Patricia S. Cruz, El King D. Morado, Joshua Gregor A. Dizon, Razel Nikka M. Hao, Arianne A. Zamora, Devon Ray Pacial, Juan Antonio R. Magalang, Marissa Alejandria, Celia Carlos, Anna Ong-Lim, Edsel Maurice Salvaña, John Q. Wong, Jaime C. Montoya, Maria Rosario Singh-Vergeire and Cynthia P. Saloma
EPI_ISL_2154497, EPI_ISL_2154499, EPI_ISL_2154501, EPI_ISL_2154505	Cagayan Valley Medical Center Molecular Laboratory	Philippine Genome Center	Francis A. Tablizo, Kenneth M. Kim, Carlo M. Lapid, Marc Jerrone R. Castro, Maria Sofia L. Yangzon, Benedict A. Maralit, Marc Edsel C. Ayes, Eva Maria Cutiongco-de la Paz, Alethea R. de Guzman, Jan Michael C. Yap, Jo-Hannah S. Llames, Sheila Mae M. Araiza, Kris P. Punayan, Irish Coleen A. Asin, Candice Francheska B. Tambaoan, Asia Louisa U. Chong, Karol Sophia Agape R. Padilla, Rianna Patricia S. Cruz, El King D. Morado, Joshua Gregor A. Dizon, Razel Nikka M. Hao, Arianne A. Zamora, Devon Ray Pacial, Juan Antonio R. Magalang, Marissa Alejandria, Celia Carlos, Anna Ong-Lim, Edsel Maurice Salvaña, John Q. Wong, Jaime C. Montoya, Maria Rosario Singh-Vergeire and Cynthia P. Saloma
EPI_ISL_2154547	Southern Isabela Medical Center Molecular Diagnostic & Research Laboratory (SIMC - MDRL)	Philippine Genome Center	Francis A. Tablizo, Kenneth M. Kim, Carlo M. Lapid, Marc Jerrone R. Castro, Maria Sofia L. Yangzon, Benedict A. Maralit, Marc Edsel C. Ayes, Eva Maria Cutiongco-de la Paz, Alethea R. de Guzman, Jan Michael C. Yap, Jo-Hannah S. Llames, Sheila Mae M. Araiza, Kris P. Punayan, Irish Coleen A. Asin, Candice Francheska B. Tambaoan, Asia Louisa U. Chong, Karol Sophia Agape R. Padilla, Rianna Patricia S. Cruz, El King D. Morado, Joshua Gregor A. Dizon, Razel Nikka M. Hao, Arianne A. Zamora, Devon Ray Pacial, Juan Antonio R. Magalang, Marissa Alejandria, Celia Carlos, Anna Ong-Lim, Edsel Maurice Salvaña, John Q. Wong, Jaime C. Montoya, Maria Rosario Singh-Vergeire and Cynthia P. Saloma
EPI_ISL_2154559	Philippine Red Cross Molecular Laboratory	Philippine Genome Center	Francis A. Tablizo, Kenneth M. Kim, Carlo M. Lapid, Marc Jerrone R. Castro, Maria Sofia L. Yangzon, Benedict A. Maralit, Marc Edsel C. Ayes, Eva Maria Cutiongco-de la Paz, Alethea R. de Guzman, Jan Michael C. Yap, Jo-Hannah S. Llames, Sheila Mae M. Araiza, Kris P. Punayan, Irish Coleen A. Asin, Candice Francheska B. Tambaoan, Asia Louisa U. Chong, Karol Sophia Agape R. Padilla, Rianna Patricia S. Cruz, El King D. Morado, Joshua Gregor A. Dizon, Razel Nikka M. Hao, Arianne A. Zamora, Devon Ray Pacial, Juan Antonio R. Magalang, Marissa Alejandria, Celia Carlos, Anna Ong-Lim, Edsel Maurice Salvaña, John Q. Wong, Jaime C. Montoya, Maria Rosario Singh-Vergeire and Cynthia P. Saloma
EPI_ISL_2154584	Cagayan Valley Medical Center Molecular Laboratory	Philippine Genome Center	Francis A. Tablizo, Kenneth M. Kim, Carlo M. Lapid, Marc Jerrone R. Castro, Maria Sofia L. Yangzon, Benedict A. Maralit, Marc Edsel C. Ayes, Eva Maria Cutiongco-de la Paz, Alethea R. de Guzman, Jan Michael C. Yap, Jo-Hannah S. Llames, Sheila Mae M. Araiza, Kris P. Punayan, Irish Coleen A. Asin, Candice Francheska B. Tambaoan, Asia Louisa U. Chong, Karol Sophia Agape R. Padilla, Rianna Patricia S. Cruz, El King D. Morado, Joshua Gregor A. Dizon, Razel Nikka M. Hao, Arianne A. Zamora, Devon Ray Pacial, Juan Antonio R. Magalang, Marissa Alejandria, Celia Carlos, Anna Ong-Lim, Edsel Maurice Salvaña, John Q. Wong, Jaime C. Montoya, Maria Rosario Singh-Vergeire and Cynthia P. Saloma
EPI_ISL_2154597	Research Institute for Tropical Medicine, Inc. (RITM)	Philippine Genome Center	Francis A. Tablizo, Kenneth M. Kim, Carlo M. Lapid, Marc Jerrone R. Castro, Maria Sofia L. Yangzon, Benedict A. Maralit, Marc Edsel C. Ayes, Eva Maria Cutiongco-de la Paz, Alethea R. de Guzman, Jan Michael C. Yap, Jo-Hannah S. Llames, Sheila Mae M. Araiza, Kris P. Punayan, Irish Coleen A. Asin, Candice Francheska B. Tambaoan, Asia Louisa U. Chong, Karol Sophia Agape R. Padilla, Rianna Patricia S. Cruz, El King D. Morado, Joshua Gregor A. Dizon, Razel Nikka M. Hao, Arianne A. Zamora, Devon Ray Pacial, Juan Antonio R. Magalang, Marissa Alejandria, Celia Carlos, Anna Ong-Lim, Edsel Maurice Salvaña, John Q. Wong, Jaime C. Montoya, Maria Rosario Singh-Vergeire and Cynthia P. Saloma
EPI_ISL_2154612	Detoxicare Molecular Diagnostics Laboratory	Philippine Genome Center	Francis A. Tablizo, Kenneth M. Kim, Carlo M. Lapid, Marc Jerrone R. Castro, Maria Sofia L. Yangzon, Benedict A. Maralit, Marc Edsel C. Ayes, Eva Maria Cutiongco-de la Paz, Alethea R. de Guzman, Jan Michael C. Yap, Jo-Hannah S. Llames, Sheila Mae M. Araiza, Kris P. Punayan, Irish Coleen A. Asin, Candice Francheska B. Tambaoan, Asia Louisa U. Chong, Karol Sophia Agape R. Padilla, Rianna Patricia S. Cruz, El King D. Morado, Joshua Gregor A. Dizon, Razel Nikka M. Hao, Arianne A. Zamora, Devon Ray Pacial, Juan Antonio R. Magalang, Marissa Alejandria, Celia Carlos, Anna Ong-Lim, Edsel Maurice Salvaña, John Q. Wong, Jaime C. Montoya, Maria Rosario Singh-Vergeire and Cynthia P. Saloma
EPI_ISL_2154613	Cagayan Valley Medical Center Molecular Laboratory	Philippine Genome Center	Francis A. Tablizo, Kenneth M. Kim, Carlo M. Lapid, Marc Jerrone R. Castro, Maria Sofia L. Yangzon, Benedict A. Maralit, Marc Edsel C. Ayes, Eva Maria Cutiongco-de la Paz, Alethea R. de Guzman, Jan Michael C. Yap, Jo-Hannah S. Llames, Sheila Mae M. Araiza, Kris P. Punayan, Irish Coleen A. Asin, Candice Francheska B. Tambaoan, Asia Louisa U. Chong, Karol Sophia Agape R. Padilla, Rianna Patricia S. Cruz, El King D. Morado, Joshua Gregor A. Dizon, Razel Nikka M. Hao, Arianne A. Zamora, Devon Ray Pacial, Juan Antonio R. Magalang, Marissa Alejandria, Celia Carlos, Anna Ong-Lim, Edsel Maurice Salvaña, John Q. Wong, Jaime C. Montoya, Maria Rosario Singh-Vergeire and Cynthia P. Saloma
EPI_ISL_2154632	South Super Highway Molecular Diagnostic Laboratory	Philippine Genome Center	Francis A. Tablizo, Kenneth M. Kim, Carlo M. Lapid, Marc Jerrone R. Castro, Maria Sofia L. Yangzon, Benedict A. Maralit, Marc Edsel C. Ayes, Eva Maria Cutiongco-de la Paz, Alethea R. de Guzman, Jan Michael C. Yap, Jo-Hannah S. Llames, Sheila Mae M. Araiza, Kris P. Punayan, Irish Coleen A. Asin, Candice Francheska B. Tambaoan, Asia Louisa U. Chong, Karol Sophia Agape R. Padilla, Rianna Patricia S. Cruz, El King D. Morado, Joshua Gregor A. Dizon, Razel Nikka M. Hao, Arianne A. Zamora, Devon Ray Pacial, Juan Antonio R. Magalang, Marissa Alejandria, Celia Carlos, Anna Ong-Lim, Edsel Maurice Salvaña, John Q. Wong, Jaime C. Montoya, Maria Rosario Singh-Vergeire and Cynthia P. Saloma
EPI_ISL_2154650	Research Institute for Tropical Medicine, Inc. (RITM)	Philippine Genome Center	Francis A. Tablizo, Kenneth M. Kim, Carlo M. Lapid, Marc Jerrone R. Castro, Maria Sofia L. Yangzon, Benedict A. Maralit, Marc Edsel C. Ayes, Eva Maria Cutiongco-de la Paz, Alethea R. de Guzman, Jan Michael C. Yap, Jo-Hannah S. Llames, Sheila Mae M. Araiza, Kris P. Punayan, Irish Coleen A. Asin, Candice Francheska B. Tambaoan, Asia Louisa U. Chong, Karol Sophia Agape R. Padilla, Rianna Patricia S. Cruz, El King D. Morado, Joshua Gregor A. Dizon, Razel Nikka M. Hao, Arianne A. Zamora, Devon Ray Pacial, Juan Antonio R. Magalang, Marissa Alejandria, Celia Carlos, Anna Ong-Lim, Edsel Maurice Salvaña, John Q. Wong, Jaime C. Montoya, Maria Rosario Singh-Vergeire and Cynthia P. Saloma
EPI_ISL_2154662	Philippine Red Cross Molecular Laboratory	Philippine Genome Center	Francis A. Tablizo, Kenneth M. Kim, Carlo M. Lapid, Marc Jerrone R. Castro, Maria Sofia L. Yangzon, Benedict A. Maralit, Marc Edsel C. Ayes, Eva Maria Cutiongco-de la Paz, Alethea R. de Guzman, Jan Michael C. Yap, Jo-Hannah S. Llames, Sheila Mae M. Araiza, Kris P. Punayan, Irish Coleen A. Asin, Candice Francheska B. Tambaoan, Asia Louisa U. Chong, Karol Sophia Agape R. Padilla, Rianna Patricia S. Cruz, El King D. Morado, Joshua Gregor A. Dizon, Razel Nikka M. Hao, Arianne A. Zamora, Devon Ray Pacial, Juan Antonio R. Magalang, Marissa Alejandria, Celia Carlos, Anna Ong-Lim, Edsel Maurice Salvaña, John Q. Wong, Jaime C. Montoya, Maria Rosario Singh-Vergeire and Cynthia P. Saloma
EPI_ISL_2154684, EPI_ISL_2154687,	Cagayan Valley Medical Center Molecular Laboratory	Philippine Genome Center	Francis A. Tablizo, Kenneth M. Kim, Carlo M. Lapid, Marc Jerrone R. Castro, Maria Sofia L. Yangzon, Benedict A. Maralit, Marc Edsel C. Ayes, Eva Maria

EPI_ISL_2154688			Cutiongco-de la Paz, Alethea R. de Guzman, Jan Michael C. Yap, Jo-Hannah S. Llames, Sheila Mae M. Araiza, Kris P. Punayan, Irish Coleen A. Asin, Candice Francheska B. Tambaoan, Asia Louisa U. Chong, Karol Sophia Agape R. Padilla, Rianna Patricia S. Cruz, El King D. Morado, Joshua Gregor A. Dizon, Razel Nikka M. Hao, Arianne A. Zamora, Devon Ray Pacial, Juan Antonio R. Magalang, Marissa Alejandria, Celia Carlos, Anna Ong-Lim, Edsel Maurice Salvaña, John Q. Wong, Jaime C. Montoya, Maria Rosario Singh-Vergeire and Cynthia P. Saloma
EPI_ISL_2154702	Health Delivery Systems, Inc.	Philippine Genome Center	Francis A. Tablizo, Kenneth M. Kim, Carlo M. Lapid, Marc Jerrone R. Castro, Maria Sofia L. Yangzon, Benedict A. Maralit, Marc Edsel C. Ayes, Eva Maria Cutiongco-de la Paz, Alethea R. de Guzman, Jan Michael C. Yap, Jo-Hannah S. Llames, Sheila Mae M. Araiza, Kris P. Punayan, Irish Coleen A. Asin, Candice Francheska B. Tambaoan, Asia Louisa U. Chong, Karol Sophia Agape R. Padilla, Rianna Patricia S. Cruz, El King D. Morado, Joshua Gregor A. Dizon, Razel Nikka M. Hao, Arianne A. Zamora, Devon Ray Pacial, Juan Antonio R. Magalang, Marissa Alejandria, Celia Carlos, Anna Ong-Lim, Edsel Maurice Salvaña, John Q. Wong, Jaime C. Montoya, Maria Rosario Singh-Vergeire and Cynthia P. Saloma
EPI_ISL_2154704	Cagayan Valley Medical Center Molecular Laboratory	Philippine Genome Center	Francis A. Tablizo, Kenneth M. Kim, Carlo M. Lapid, Marc Jerrone R. Castro, Maria Sofia L. Yangzon, Benedict A. Maralit, Marc Edsel C. Ayes, Eva Maria Cutiongco-de la Paz, Alethea R. de Guzman, Jan Michael C. Yap, Jo-Hannah S. Llames, Sheila Mae M. Araiza, Kris P. Punayan, Irish Coleen A. Asin, Candice Francheska B. Tambaoan, Asia Louisa U. Chong, Karol Sophia Agape R. Padilla, Rianna Patricia S. Cruz, El King D. Morado, Joshua Gregor A. Dizon, Razel Nikka M. Hao, Arianne A. Zamora, Devon Ray Pacial, Juan Antonio R. Magalang, Marissa Alejandria, Celia Carlos, Anna Ong-Lim, Edsel Maurice Salvaña, John Q. Wong, Jaime C. Montoya, Maria Rosario Singh-Vergeire and Cynthia P. Saloma
EPI_ISL_2154712	South Super Highway Molecular Diagnostic Laboratory	Philippine Genome Center	Francis A. Tablizo, Kenneth M. Kim, Carlo M. Lapid, Marc Jerrone R. Castro, Maria Sofia L. Yangzon, Benedict A. Maralit, Marc Edsel C. Ayes, Eva Maria Cutiongco-de la Paz, Alethea R. de Guzman, Jan Michael C. Yap, Jo-Hannah S. Llames, Sheila Mae M. Araiza, Kris P. Punayan, Irish Coleen A. Asin, Candice Francheska B. Tambaoan, Asia Louisa U. Chong, Karol Sophia Agape R. Padilla, Rianna Patricia S. Cruz, El King D. Morado, Joshua Gregor A. Dizon, Razel Nikka M. Hao, Arianne A. Zamora, Devon Ray Pacial, Juan Antonio R. Magalang, Marissa Alejandria, Celia Carlos, Anna Ong-Lim, Edsel Maurice Salvaña, John Q. Wong, Jaime C. Montoya, Maria Rosario Singh-Vergeire and Cynthia P. Saloma
EPI_ISL_2154730, EPI_ISL_2154733, EPI_ISL_2154771, EPI_ISL_2154783, EPI_ISL_2154787	Cagayan Valley Medical Center Molecular Laboratory	Philippine Genome Center	Francis A. Tablizo, Kenneth M. Kim, Carlo M. Lapid, Marc Jerrone R. Castro, Maria Sofia L. Yangzon, Benedict A. Maralit, Marc Edsel C. Ayes, Eva Maria Cutiongco-de la Paz, Alethea R. de Guzman, Jan Michael C. Yap, Jo-Hannah S. Llames, Sheila Mae M. Araiza, Kris P. Punayan, Irish Coleen A. Asin, Candice Francheska B. Tambaoan, Asia Louisa U. Chong, Karol Sophia Agape R. Padilla, Rianna Patricia S. Cruz, El King D. Morado, Joshua Gregor A. Dizon, Razel Nikka M. Hao, Arianne A. Zamora, Devon Ray Pacial, Juan Antonio R. Magalang, Marissa Alejandria, Celia Carlos, Anna Ong-Lim, Edsel Maurice Salvaña, John Q. Wong, Jaime C. Montoya, Maria Rosario Singh-Vergeire and Cynthia P. Saloma
EPI_ISL_2154794	Research Institute for Tropical Medicine, Inc. (RITM)	Philippine Genome Center	Francis A. Tablizo, Kenneth M. Kim, Carlo M. Lapid, Marc Jerrone R. Castro, Maria Sofia L. Yangzon, Benedict A. Maralit, Marc Edsel C. Ayes, Eva Maria Cutiongco-de la Paz, Alethea R. de Guzman, Jan Michael C. Yap, Jo-Hannah S. Llames, Sheila Mae M. Araiza, Kris P. Punayan, Irish Coleen A. Asin, Candice Francheska B. Tambaoan, Asia Louisa U. Chong, Karol Sophia Agape R. Padilla, Rianna Patricia S. Cruz, El King D. Morado, Joshua Gregor A. Dizon, Razel Nikka M. Hao, Arianne A. Zamora, Devon Ray Pacial, Juan Antonio R. Magalang, Marissa Alejandria, Celia Carlos, Anna Ong-Lim, Edsel Maurice Salvaña, John Q. Wong, Jaime C. Montoya, Maria Rosario Singh-Vergeire and Cynthia P. Saloma
EPI_ISL_2154808	South Super Highway Molecular Diagnostic Laboratory	Philippine Genome Center	Francis A. Tablizo, Kenneth M. Kim, Carlo M. Lapid, Marc Jerrone R. Castro, Maria Sofia L. Yangzon, Benedict A. Maralit, Marc Edsel C. Ayes, Eva Maria Cutiongco-de la Paz, Alethea R. de Guzman, Jan Michael C. Yap, Jo-Hannah S. Llames, Sheila Mae M. Araiza, Kris P. Punayan, Irish Coleen A. Asin, Candice Francheska B. Tambaoan, Asia Louisa U. Chong, Karol Sophia Agape R. Padilla, Rianna Patricia S. Cruz, El King D. Morado, Joshua Gregor A. Dizon, Razel Nikka M. Hao, Arianne A. Zamora, Devon Ray Pacial, Juan Antonio R. Magalang, Marissa Alejandria, Celia Carlos, Anna Ong-Lim, Edsel Maurice Salvaña, John Q. Wong, Jaime C. Montoya, Maria Rosario Singh-Vergeire and Cynthia P. Saloma
EPI_ISL_2154814, EPI_ISL_2154823, EPI_ISL_2154825	Cagayan Valley Medical Center Molecular Laboratory	Philippine Genome Center	Francis A. Tablizo, Kenneth M. Kim, Carlo M. Lapid, Marc Jerrone R. Castro, Maria Sofia L. Yangzon, Benedict A. Maralit, Marc Edsel C. Ayes, Eva Maria Cutiongco-de la Paz, Alethea R. de Guzman, Jan Michael C. Yap, Jo-Hannah S. Llames, Sheila Mae M. Araiza, Kris P. Punayan, Irish Coleen A. Asin, Candice Francheska B. Tambaoan, Asia Louisa U. Chong, Karol Sophia Agape R. Padilla, Rianna Patricia S. Cruz, El King D. Morado, Joshua Gregor A. Dizon, Razel Nikka M. Hao, Arianne A. Zamora, Devon Ray Pacial, Juan Antonio R. Magalang, Marissa Alejandria, Celia Carlos, Anna Ong-Lim, Edsel Maurice Salvaña, John Q. Wong, Jaime C. Montoya, Maria Rosario Singh-Vergeire and Cynthia P. Saloma
EPI_ISL_2154835	Research Institute for Tropical Medicine, Inc. (RITM)	Philippine Genome Center	Francis A. Tablizo, Kenneth M. Kim, Carlo M. Lapid, Marc Jerrone R. Castro, Maria Sofia L. Yangzon, Benedict A. Maralit, Marc Edsel C. Ayes, Eva Maria Cutiongco-de la Paz, Alethea R. de Guzman, Jan Michael C. Yap, Jo-Hannah S. Llames, Sheila Mae M. Araiza, Kris P. Punayan, Irish Coleen A. Asin, Candice Francheska B. Tambaoan, Asia Louisa U. Chong, Karol Sophia Agape R. Padilla, Rianna Patricia S. Cruz, El King D. Morado, Joshua Gregor A. Dizon, Razel Nikka M. Hao, Arianne A. Zamora, Devon Ray Pacial, Juan Antonio R. Magalang, Marissa Alejandria, Celia Carlos, Anna Ong-Lim, Edsel Maurice Salvaña, John Q. Wong, Jaime C. Montoya, Maria Rosario Singh-Vergeire and Cynthia P. Saloma
EPI_ISL_2154838, EPI_ISL_2154844, EPI_ISL_2154861, EPI_ISL_2154866, EPI_ISL_2154874	Cagayan Valley Medical Center Molecular Laboratory	Philippine Genome Center	Francis A. Tablizo, Kenneth M. Kim, Carlo M. Lapid, Marc Jerrone R. Castro, Maria Sofia L. Yangzon, Benedict A. Maralit, Marc Edsel C. Ayes, Eva Maria Cutiongco-de la Paz, Alethea R. de Guzman, Jan Michael C. Yap, Jo-Hannah S. Llames, Sheila Mae M. Araiza, Kris P. Punayan, Irish Coleen A. Asin, Candice Francheska B. Tambaoan, Asia Louisa U. Chong, Karol Sophia Agape R. Padilla, Rianna Patricia S. Cruz, El King D. Morado, Joshua Gregor A. Dizon, Razel Nikka M. Hao, Arianne A. Zamora, Devon Ray Pacial, Juan Antonio R. Magalang, Marissa Alejandria, Celia Carlos, Anna Ong-Lim, Edsel Maurice Salvaña, John Q. Wong, Jaime C. Montoya, Maria Rosario Singh-Vergeire and Cynthia P. Saloma
EPI_ISL_2154879	Baguio General Hospital Medical Center (BGHMC)	Philippine Genome Center	Francis A. Tablizo, Kenneth M. Kim, Carlo M. Lapid, Marc Jerrone R. Castro, Maria Sofia L. Yangzon, Benedict A. Maralit, Marc Edsel C. Ayes, Eva Maria Cutiongco-de la Paz, Alethea R. de Guzman, Jan Michael C. Yap, Jo-Hannah S. Llames, Sheila Mae M. Araiza, Kris P. Punayan, Irish Coleen A. Asin, Candice Francheska B. Tambaoan, Asia Louisa U. Chong, Karol Sophia Agape R. Padilla, Rianna Patricia S. Cruz, El King D. Morado, Joshua Gregor A. Dizon, Razel Nikka M. Hao, Arianne A. Zamora, Devon Ray Pacial, Juan Antonio R. Magalang, Marissa Alejandria, Celia Carlos, Anna Ong-Lim, Edsel Maurice Salvaña, John Q. Wong, Jaime C. Montoya, Maria Rosario Singh-Vergeire and Cynthia P. Saloma
EPI_ISL_2154880	Cagayan Valley Medical Center Molecular Laboratory	Philippine Genome Center	Francis A. Tablizo, Kenneth M. Kim, Carlo M. Lapid, Marc Jerrone R. Castro, Maria Sofia L. Yangzon, Benedict A. Maralit, Marc Edsel C. Ayes, Eva Maria Cutiongco-de la Paz, Alethea R. de Guzman, Jan Michael C. Yap, Jo-Hannah S. Llames, Sheila Mae M. Araiza, Kris P. Punayan, Irish Coleen A. Asin, Candice Francheska B. Tambaoan, Asia Louisa U. Chong, Karol Sophia Agape R. Padilla, Rianna Patricia S. Cruz, El King D. Morado, Joshua Gregor A. Dizon, Razel Nikka M. Hao, Arianne A. Zamora, Devon Ray Pacial, Juan Antonio R. Magalang, Marissa Alejandria, Celia Carlos, Anna Ong-Lim, Edsel Maurice Salvaña, John Q. Wong, Jaime C. Montoya, Maria Rosario Singh-Vergeire and Cynthia P. Saloma
EPI_ISL_2154883	Baguio General Hospital Medical Center (BGHMC)	Philippine Genome Center	Francis A. Tablizo, Kenneth M. Kim, Carlo M. Lapid, Marc Jerrone R. Castro, Maria Sofia L. Yangzon, Benedict A. Maralit, Marc Edsel C. Ayes, Eva Maria Cutiongco-de la Paz, Alethea R. de Guzman, Jan Michael C. Yap, Jo-Hannah S. Llames, Sheila Mae M. Araiza, Kris P. Punayan, Irish Coleen A. Asin, Candice Francheska B. Tambaoan, Asia Louisa U. Chong, Karol Sophia Agape R. Padilla, Rianna Patricia S. Cruz, El King D. Morado, Joshua Gregor A. Dizon, Razel Nikka M. Hao, Arianne A. Zamora, Devon Ray Pacial, Juan Antonio R. Magalang, Marissa Alejandria, Celia Carlos, Anna Ong-Lim, Edsel Maurice Salvaña, John Q. Wong, Jaime C. Montoya, Maria Rosario Singh-Vergeire and Cynthia P. Saloma
EPI_ISL_2154894	Detoxicare Molecular Diagnostics Laboratory	Philippine Genome Center	Francis A. Tablizo, Kenneth M. Kim, Carlo M. Lapid, Marc Jerrone R. Castro, Maria Sofia L. Yangzon, Benedict A. Maralit, Marc Edsel C. Ayes, Eva Maria Cutiongco-de la Paz, Alethea R. de Guzman, Jan Michael C. Yap, Jo-Hannah S. Llames, Sheila Mae M. Araiza, Kris P. Punayan, Irish Coleen A. Asin, Candice Francheska B. Tambaoan, Asia Louisa U. Chong, Karol Sophia Agape R. Padilla, Rianna Patricia S. Cruz, El King D. Morado, Joshua Gregor A. Dizon, Razel Nikka M. Hao, Arianne A. Zamora, Devon Ray Pacial, Juan Antonio R. Magalang, Marissa Alejandria, Celia Carlos, Anna Ong-Lim, Edsel Maurice Salvaña, John Q. Wong, Jaime C. Montoya, Maria Rosario Singh-Vergeire and Cynthia P. Saloma
EPI_ISL_2154896	Cagayan Valley Medical Center Molecular Laboratory	Philippine Genome Center	Francis A. Tablizo, Kenneth M. Kim, Carlo M. Lapid, Marc Jerrone R. Castro, Maria Sofia L. Yangzon, Benedict A. Maralit, Marc Edsel C. Ayes, Eva Maria Cutiongco-de la Paz, Alethea R. de Guzman, Jan Michael C. Yap, Jo-Hannah S. Llames, Sheila Mae M. Araiza, Kris P. Punayan, Irish Coleen A. Asin, Candice Francheska B. Tambaoan, Asia Louisa U. Chong, Karol Sophia Agape R. Padilla, Rianna Patricia S. Cruz, El King D. Morado, Joshua Gregor A. Dizon, Razel Nikka M. Hao, Arianne A. Zamora, Devon Ray Pacial, Juan Antonio R. Magalang, Marissa Alejandria, Celia Carlos, Anna Ong-Lim, Edsel Maurice Salvaña, John Q. Wong, Jaime C. Montoya, Maria Rosario Singh-Vergeire and Cynthia P. Saloma
EPI_ISL_2154903, EPI_ISL_2154908, EPI_ISL_2155013	Baguio General Hospital Medical Center (BGHMC)	Philippine Genome Center	Francis A. Tablizo, Kenneth M. Kim, Carlo M. Lapid, Marc Jerrone R. Castro, Maria Sofia L. Yangzon, Benedict A. Maralit, Marc Edsel C. Ayes, Eva Maria Cutiongco-de la Paz, Alethea R. de Guzman, Jan Michael C. Yap, Jo-Hannah S. Llames, Sheila Mae M. Araiza, Kris P. Punayan, Irish Coleen A. Asin, Candice Francheska B. Tambaoan, Asia Louisa U. Chong, Karol Sophia Agape R. Padilla, Rianna Patricia S. Cruz, El King D. Morado, Joshua Gregor A. Dizon, Razel Nikka M. Hao, Arianne A. Zamora, Devon Ray Pacial, Juan Antonio R. Magalang, Marissa Alejandria, Celia Carlos, Anna Ong-Lim, Edsel Maurice Salvaña, John Q. Wong, Jaime C. Montoya, Maria Rosario Singh-Vergeire and Cynthia P. Saloma

EPI_ISL_2155055, EPI_ISL_2155056	Research Institute for Tropical Medicine, Inc. (RITM)	Philippine Genome Center	Francis A. Tablizo, Kenneth M. Kim, Carlo M. Lapid, Marc Jerrone R. Castro, Maria Sofia L. Yangzon, Benedict A. Maralit, Marc Edsel C. Ayes, Eva Maria Cutiongco-de la Paz, Alethea R. de Guzman, Jan Michael C. Yap, Jo-Hannah S. Llames, Sheila Mae M. Araiza, Kris P. Punayan, Irish Coleen A. Asin, Candice Francheska B. Tambaoan, Asia Louisa U. Chong, Karol Sophia Agape R. Padilla, Rianna Patricia S. Cruz, El King D. Morado, Joshua Gregor A. Dizon, Razel Nikka M. Hao, Arianne A. Zamora, Devon Ray Pacial, Juan Antonio R. Magalang, Marissa Alejandria, Celia Carlos, Anna Ong-Lim, Edsel Maurice Salvaña, John Q. Wong, Jaime C. Montoya, Maria Rosario Singh-Vergeire and Cynthia P. Saloma
EPI_ISL_2155059	Philippine Red Cross - Port Area	Philippine Genome Center	Francis A. Tablizo, Kenneth M. Kim, Carlo M. Lapid, Marc Jerrone R. Castro, Maria Sofia L. Yangzon, Benedict A. Maralit, Marc Edsel C. Ayes, Eva Maria Cutiongco-de la Paz, Alethea R. de Guzman, Jan Michael C. Yap, Jo-Hannah S. Llames, Sheila Mae M. Araiza, Kris P. Punayan, Irish Coleen A. Asin, Candice Francheska B. Tambaoan, Asia Louisa U. Chong, Karol Sophia Agape R. Padilla, Rianna Patricia S. Cruz, El King D. Morado, Joshua Gregor A. Dizon, Razel Nikka M. Hao, Arianne A. Zamora, Devon Ray Pacial, Juan Antonio R. Magalang, Marissa Alejandria, Celia Carlos, Anna Ong-Lim, Edsel Maurice Salvaña, John Q. Wong, Jaime C. Montoya, Maria Rosario Singh-Vergeire and Cynthia P. Saloma
EPI_ISL_2155060	Research Institute for Tropical Medicine, Inc. (RITM)	Philippine Genome Center	Francis A. Tablizo, Kenneth M. Kim, Carlo M. Lapid, Marc Jerrone R. Castro, Maria Sofia L. Yangzon, Benedict A. Maralit, Marc Edsel C. Ayes, Eva Maria Cutiongco-de la Paz, Alethea R. de Guzman, Jan Michael C. Yap, Jo-Hannah S. Llames, Sheila Mae M. Araiza, Kris P. Punayan, Irish Coleen A. Asin, Candice Francheska B. Tambaoan, Asia Louisa U. Chong, Karol Sophia Agape R. Padilla, Rianna Patricia S. Cruz, El King D. Morado, Joshua Gregor A. Dizon, Razel Nikka M. Hao, Arianne A. Zamora, Devon Ray Pacial, Juan Antonio R. Magalang, Marissa Alejandria, Celia Carlos, Anna Ong-Lim, Edsel Maurice Salvaña, John Q. Wong, Jaime C. Montoya, Maria Rosario Singh-Vergeire and Cynthia P. Saloma
EPI_ISL_2155069	Laguna Provincial Hospital - San Pablo District Hospital	Philippine Genome Center	Francis A. Tablizo, Kenneth M. Kim, Carlo M. Lapid, Marc Jerrone R. Castro, Maria Sofia L. Yangzon, Benedict A. Maralit, Marc Edsel C. Ayes, Eva Maria Cutiongco-de la Paz, Alethea R. de Guzman, Jan Michael C. Yap, Jo-Hannah S. Llames, Sheila Mae M. Araiza, Kris P. Punayan, Irish Coleen A. Asin, Candice Francheska B. Tambaoan, Asia Louisa U. Chong, Karol Sophia Agape R. Padilla, Rianna Patricia S. Cruz, El King D. Morado, Joshua Gregor A. Dizon, Razel Nikka M. Hao, Arianne A. Zamora, Devon Ray Pacial, Juan Antonio R. Magalang, Marissa Alejandria, Celia Carlos, Anna Ong-Lim, Edsel Maurice Salvaña, John Q. Wong, Jaime C. Montoya, Maria Rosario Singh-Vergeire and Cynthia P. Saloma
EPI_ISL_2155072	Research Institute for Tropical Medicine, Inc. (RITM)	Philippine Genome Center	Francis A. Tablizo, Kenneth M. Kim, Carlo M. Lapid, Marc Jerrone R. Castro, Maria Sofia L. Yangzon, Benedict A. Maralit, Marc Edsel C. Ayes, Eva Maria Cutiongco-de la Paz, Alethea R. de Guzman, Jan Michael C. Yap, Jo-Hannah S. Llames, Sheila Mae M. Araiza, Kris P. Punayan, Irish Coleen A. Asin, Candice Francheska B. Tambaoan, Asia Louisa U. Chong, Karol Sophia Agape R. Padilla, Rianna Patricia S. Cruz, El King D. Morado, Joshua Gregor A. Dizon, Razel Nikka M. Hao, Arianne A. Zamora, Devon Ray Pacial, Juan Antonio R. Magalang, Marissa Alejandria, Celia Carlos, Anna Ong-Lim, Edsel Maurice Salvaña, John Q. Wong, Jaime C. Montoya, Maria Rosario Singh-Vergeire and Cynthia P. Saloma
EPI_ISL_2155073	Qualimed Hospital Sta. Rosa	Philippine Genome Center	Francis A. Tablizo, Kenneth M. Kim, Carlo M. Lapid, Marc Jerrone R. Castro, Maria Sofia L. Yangzon, Benedict A. Maralit, Marc Edsel C. Ayes, Eva Maria Cutiongco-de la Paz, Alethea R. de Guzman, Jan Michael C. Yap, Jo-Hannah S. Llames, Sheila Mae M. Araiza, Kris P. Punayan, Irish Coleen A. Asin, Candice Francheska B. Tambaoan, Asia Louisa U. Chong, Karol Sophia Agape R. Padilla, Rianna Patricia S. Cruz, El King D. Morado, Joshua Gregor A. Dizon, Razel Nikka M. Hao, Arianne A. Zamora, Devon Ray Pacial, Juan Antonio R. Magalang, Marissa Alejandria, Celia Carlos, Anna Ong-Lim, Edsel Maurice Salvaña, John Q. Wong, Jaime C. Montoya, Maria Rosario Singh-Vergeire and Cynthia P. Saloma
EPI_ISL_2155079	Research Institute for Tropical Medicine, Inc. (RITM)	Philippine Genome Center	Francis A. Tablizo, Kenneth M. Kim, Carlo M. Lapid, Marc Jerrone R. Castro, Maria Sofia L. Yangzon, Benedict A. Maralit, Marc Edsel C. Ayes, Eva Maria Cutiongco-de la Paz, Alethea R. de Guzman, Jan Michael C. Yap, Jo-Hannah S. Llames, Sheila Mae M. Araiza, Kris P. Punayan, Irish Coleen A. Asin, Candice Francheska B. Tambaoan, Asia Louisa U. Chong, Karol Sophia Agape R. Padilla, Rianna Patricia S. Cruz, El King D. Morado, Joshua Gregor A. Dizon, Razel Nikka M. Hao, Arianne A. Zamora, Devon Ray Pacial, Juan Antonio R. Magalang, Marissa Alejandria, Celia Carlos, Anna Ong-Lim, Edsel Maurice Salvaña, John Q. Wong, Jaime C. Montoya, Maria Rosario Singh-Vergeire and Cynthia P. Saloma
EPI_ISL_2155080	Southern Isabela Medical Center Molecular Diagnostic & Research Laboratory (SIMC - MDRL)	Philippine Genome Center	Francis A. Tablizo, Kenneth M. Kim, Carlo M. Lapid, Marc Jerrone R. Castro, Maria Sofia L. Yangzon, Benedict A. Maralit, Marc Edsel C. Ayes, Eva Maria Cutiongco-de la Paz, Alethea R. de Guzman, Jan Michael C. Yap, Jo-Hannah S. Llames, Sheila Mae M. Araiza, Kris P. Punayan, Irish Coleen A. Asin, Candice Francheska B. Tambaoan, Asia Louisa U. Chong, Karol Sophia Agape R. Padilla, Rianna Patricia S. Cruz, El King D. Morado, Joshua Gregor A. Dizon, Razel Nikka M. Hao, Arianne A. Zamora, Devon Ray Pacial, Juan Antonio R. Magalang, Marissa Alejandria, Celia Carlos, Anna Ong-Lim, Edsel Maurice Salvaña, John Q. Wong, Jaime C. Montoya, Maria Rosario Singh-Vergeire and Cynthia P. Saloma
EPI_ISL_2155084	Qualimed Hospital Sta. Rosa	Philippine Genome Center	Francis A. Tablizo, Kenneth M. Kim, Carlo M. Lapid, Marc Jerrone R. Castro, Maria Sofia L. Yangzon, Benedict A. Maralit, Marc Edsel C. Ayes, Eva Maria Cutiongco-de la Paz, Alethea R. de Guzman, Jan Michael C. Yap, Jo-Hannah S. Llames, Sheila Mae M. Araiza, Kris P. Punayan, Irish Coleen A. Asin, Candice Francheska B. Tambaoan, Asia Louisa U. Chong, Karol Sophia Agape R. Padilla, Rianna Patricia S. Cruz, El King D. Morado, Joshua Gregor A. Dizon, Razel Nikka M. Hao, Arianne A. Zamora, Devon Ray Pacial, Juan Antonio R. Magalang, Marissa Alejandria, Celia Carlos, Anna Ong-Lim, Edsel Maurice Salvaña, John Q. Wong, Jaime C. Montoya, Maria Rosario Singh-Vergeire and Cynthia P. Saloma
EPI_ISL_2155095, EPI_ISL_2155099, EPI_ISL_2155102	Research Institute for Tropical Medicine, Inc. (RITM)	Philippine Genome Center	Francis A. Tablizo, Kenneth M. Kim, Carlo M. Lapid, Marc Jerrone R. Castro, Maria Sofia L. Yangzon, Benedict A. Maralit, Marc Edsel C. Ayes, Eva Maria Cutiongco-de la Paz, Alethea R. de Guzman, Jan Michael C. Yap, Jo-Hannah S. Llames, Sheila Mae M. Araiza, Kris P. Punayan, Irish Coleen A. Asin, Candice Francheska B. Tambaoan, Asia Louisa U. Chong, Karol Sophia Agape R. Padilla, Rianna Patricia S. Cruz, El King D. Morado, Joshua Gregor A. Dizon, Razel Nikka M. Hao, Arianne A. Zamora, Devon Ray Pacial, Juan Antonio R. Magalang, Marissa Alejandria, Celia Carlos, Anna Ong-Lim, Edsel Maurice Salvaña, John Q. Wong, Jaime C. Montoya, Maria Rosario Singh-Vergeire and Cynthia P. Saloma
EPI_ISL_2155110	Philippine Red Cross - Port Area	Philippine Genome Center	Francis A. Tablizo, Kenneth M. Kim, Carlo M. Lapid, Marc Jerrone R. Castro, Maria Sofia L. Yangzon, Benedict A. Maralit, Marc Edsel C. Ayes, Eva Maria Cutiongco-de la Paz, Alethea R. de Guzman, Jan Michael C. Yap, Jo-Hannah S. Llames, Sheila Mae M. Araiza, Kris P. Punayan, Irish Coleen A. Asin, Candice Francheska B. Tambaoan, Asia Louisa U. Chong, Karol Sophia Agape R. Padilla, Rianna Patricia S. Cruz, El King D. Morado, Joshua Gregor A. Dizon, Razel Nikka M. Hao, Arianne A. Zamora, Devon Ray Pacial, Juan Antonio R. Magalang, Marissa Alejandria, Celia Carlos, Anna Ong-Lim, Edsel Maurice Salvaña, John Q. Wong, Jaime C. Montoya, Maria Rosario Singh-Vergeire and Cynthia P. Saloma
EPI_ISL_2155111, EPI_ISL_2155116, EPI_ISL_2155120, EPI_ISL_2155122	Research Institute for Tropical Medicine, Inc. (RITM)	Philippine Genome Center	Francis A. Tablizo, Kenneth M. Kim, Carlo M. Lapid, Marc Jerrone R. Castro, Maria Sofia L. Yangzon, Benedict A. Maralit, Marc Edsel C. Ayes, Eva Maria Cutiongco-de la Paz, Alethea R. de Guzman, Jan Michael C. Yap, Jo-Hannah S. Llames, Sheila Mae M. Araiza, Kris P. Punayan, Irish Coleen A. Asin, Candice Francheska B. Tambaoan, Asia Louisa U. Chong, Karol Sophia Agape R. Padilla, Rianna Patricia S. Cruz, El King D. Morado, Joshua Gregor A. Dizon, Razel Nikka M. Hao, Arianne A. Zamora, Devon Ray Pacial, Juan Antonio R. Magalang, Marissa Alejandria, Celia Carlos, Anna Ong-Lim, Edsel Maurice Salvaña, John Q. Wong, Jaime C. Montoya, Maria Rosario Singh-Vergeire and Cynthia P. Saloma
EPI_ISL_2155123	Cagayan Valley Medical Center Molecular Laboratory	Philippine Genome Center	Francis A. Tablizo, Kenneth M. Kim, Carlo M. Lapid, Marc Jerrone R. Castro, Maria Sofia L. Yangzon, Benedict A. Maralit, Marc Edsel C. Ayes, Eva Maria Cutiongco-de la Paz, Alethea R. de Guzman, Jan Michael C. Yap, Jo-Hannah S. Llames, Sheila Mae M. Araiza, Kris P. Punayan, Irish Coleen A. Asin, Candice Francheska B. Tambaoan, Asia Louisa U. Chong, Karol Sophia Agape R. Padilla, Rianna Patricia S. Cruz, El King D. Morado, Joshua Gregor A. Dizon, Razel Nikka M. Hao, Arianne A. Zamora, Devon Ray Pacial, Juan Antonio R. Magalang, Marissa Alejandria, Celia Carlos, Anna Ong-Lim, Edsel Maurice Salvaña, John Q. Wong, Jaime C. Montoya, Maria Rosario Singh-Vergeire and Cynthia P. Saloma
EPI_ISL_2155124	Research Institute for Tropical Medicine, Inc. (RITM)	Philippine Genome Center	Francis A. Tablizo, Kenneth M. Kim, Carlo M. Lapid, Marc Jerrone R. Castro, Maria Sofia L. Yangzon, Benedict A. Maralit, Marc Edsel C. Ayes, Eva Maria Cutiongco-de la Paz, Alethea R. de Guzman, Jan Michael C. Yap, Jo-Hannah S. Llames, Sheila Mae M. Araiza, Kris P. Punayan, Irish Coleen A. Asin, Candice Francheska B. Tambaoan, Asia Louisa U. Chong, Karol Sophia Agape R. Padilla, Rianna Patricia S. Cruz, El King D. Morado, Joshua Gregor A. Dizon, Razel Nikka M. Hao, Arianne A. Zamora, Devon Ray Pacial, Juan Antonio R. Magalang, Marissa Alejandria, Celia Carlos, Anna Ong-Lim, Edsel Maurice Salvaña, John Q. Wong, Jaime C. Montoya, Maria Rosario Singh-Vergeire and Cynthia P. Saloma
EPI_ISL_2155137	Cagayan Valley Medical Center Molecular Laboratory	Philippine Genome Center	Francis A. Tablizo, Kenneth M. Kim, Carlo M. Lapid, Marc Jerrone R. Castro, Maria Sofia L. Yangzon, Benedict A. Maralit, Marc Edsel C. Ayes, Eva Maria Cutiongco-de la Paz, Alethea R. de Guzman, Jan Michael C. Yap, Jo-Hannah S. Llames, Sheila Mae M. Araiza, Kris P. Punayan, Irish Coleen A. Asin, Candice Francheska B. Tambaoan, Asia Louisa U. Chong, Karol Sophia Agape R. Padilla, Rianna Patricia S. Cruz, El King D. Morado, Joshua Gregor A. Dizon, Razel Nikka M. Hao, Arianne A. Zamora, Devon Ray Pacial, Juan Antonio R. Magalang, Marissa Alejandria, Celia Carlos, Anna Ong-Lim, Edsel Maurice Salvaña, John Q. Wong, Jaime C. Montoya, Maria Rosario Singh-Vergeire and Cynthia P. Saloma
EPI_ISL_2155141	SAN PABLO CITY HEALTH OFFICE	Philippine Genome Center	Francis A. Tablizo, Kenneth M. Kim, Carlo M. Lapid, Marc Jerrone R. Castro, Maria Sofia L. Yangzon, Benedict A. Maralit, Marc Edsel C. Ayes, Eva Maria Cutiongco-de la Paz, Alethea R. de Guzman, Jan Michael C. Yap, Jo-Hannah S. Llames, Sheila Mae M. Araiza, Kris P. Punayan, Irish Coleen A. Asin, Candice Francheska B. Tambaoan, Asia Louisa U. Chong, Karol Sophia Agape R. Padilla, Rianna Patricia S. Cruz, El King D. Morado, Joshua Gregor A. Dizon, Razel Nikka M. Hao, Arianne A. Zamora, Devon Ray Pacial, Juan Antonio R. Magalang, Marissa Alejandria, Celia Carlos, Anna Ong-Lim, Edsel Maurice Salvaña, John Q. Wong, Jaime C. Montoya, Maria Rosario Singh-Vergeire and Cynthia P. Saloma

EPI_ISL_2155143	Research Institute for Tropical Medicine, Inc. (RITM)	Philippine Genome Center	Francis A. Tablizo, Kenneth M. Kim, Carlo M. Lapid, Marc Jerrone R. Castro, Maria Sofia L. Yangzon, Benedict A. Maralit, Marc Edsel C. Ayes, Eva Maria Cutiongco-de la Paz, Alethea R. de Guzman, Jan Michael C. Yap, Jo-Hannah S. Llames, Sheila Mae M. Araiza, Kris P. Punayan, Irish Coleen A. Asin, Candice Francheska B. Tambaoan, Asia Louisa U. Chong, Karol Sophia Agape R. Padilla, Rianna Patricia S. Cruz, El King D. Morado, Joshua Gregor A. Dizon, Razel Nikka M. Hao, Arianne A. Zamora, Devon Ray Pacial, Juan Antonio R. Magalang, Marissa Alejandria, Celia Carlos, Anna Ong-Lim, Edsel Maurice Salvaña, John Q. Wong, Jaime C. Montoya, Maria Rosario Singh-Vergeire and Cynthia P. Saloma
EPI_ISL_2155144	Cagayan Valley Medical Center Molecular Laboratory	Philippine Genome Center	Francis A. Tablizo, Kenneth M. Kim, Carlo M. Lapid, Marc Jerrone R. Castro, Maria Sofia L. Yangzon, Benedict A. Maralit, Marc Edsel C. Ayes, Eva Maria Cutiongco-de la Paz, Alethea R. de Guzman, Jan Michael C. Yap, Jo-Hannah S. Llames, Sheila Mae M. Araiza, Kris P. Punayan, Irish Coleen A. Asin, Candice Francheska B. Tambaoan, Asia Louisa U. Chong, Karol Sophia Agape R. Padilla, Rianna Patricia S. Cruz, El King D. Morado, Joshua Gregor A. Dizon, Razel Nikka M. Hao, Arianne A. Zamora, Devon Ray Pacial, Juan Antonio R. Magalang, Marissa Alejandria, Celia Carlos, Anna Ong-Lim, Edsel Maurice Salvaña, John Q. Wong, Jaime C. Montoya, Maria Rosario Singh-Vergeire and Cynthia P. Saloma
EPI_ISL_2155147	Philippine Red Cross - Port Area	Philippine Genome Center	Francis A. Tablizo, Kenneth M. Kim, Carlo M. Lapid, Marc Jerrone R. Castro, Maria Sofia L. Yangzon, Benedict A. Maralit, Marc Edsel C. Ayes, Eva Maria Cutiongco-de la Paz, Alethea R. de Guzman, Jan Michael C. Yap, Jo-Hannah S. Llames, Sheila Mae M. Araiza, Kris P. Punayan, Irish Coleen A. Asin, Candice Francheska B. Tambaoan, Asia Louisa U. Chong, Karol Sophia Agape R. Padilla, Rianna Patricia S. Cruz, El King D. Morado, Joshua Gregor A. Dizon, Razel Nikka M. Hao, Arianne A. Zamora, Devon Ray Pacial, Juan Antonio R. Magalang, Marissa Alejandria, Celia Carlos, Anna Ong-Lim, Edsel Maurice Salvaña, John Q. Wong, Jaime C. Montoya, Maria Rosario Singh-Vergeire and Cynthia P. Saloma
EPI_ISL_2155149, EPI_ISL_2155152	Research Institute for Tropical Medicine, Inc. (RITM)	Philippine Genome Center	Francis A. Tablizo, Kenneth M. Kim, Carlo M. Lapid, Marc Jerrone R. Castro, Maria Sofia L. Yangzon, Benedict A. Maralit, Marc Edsel C. Ayes, Eva Maria Cutiongco-de la Paz, Alethea R. de Guzman, Jan Michael C. Yap, Jo-Hannah S. Llames, Sheila Mae M. Araiza, Kris P. Punayan, Irish Coleen A. Asin, Candice Francheska B. Tambaoan, Asia Louisa U. Chong, Karol Sophia Agape R. Padilla, Rianna Patricia S. Cruz, El King D. Morado, Joshua Gregor A. Dizon, Razel Nikka M. Hao, Arianne A. Zamora, Devon Ray Pacial, Juan Antonio R. Magalang, Marissa Alejandria, Celia Carlos, Anna Ong-Lim, Edsel Maurice Salvaña, John Q. Wong, Jaime C. Montoya, Maria Rosario Singh-Vergeire and Cynthia P. Saloma
EPI_ISL_2155155	Philippine Children's Medical Center	Philippine Genome Center	Francis A. Tablizo, Kenneth M. Kim, Carlo M. Lapid, Marc Jerrone R. Castro, Maria Sofia L. Yangzon, Benedict A. Maralit, Marc Edsel C. Ayes, Eva Maria Cutiongco-de la Paz, Alethea R. de Guzman, Jan Michael C. Yap, Jo-Hannah S. Llames, Sheila Mae M. Araiza, Kris P. Punayan, Irish Coleen A. Asin, Candice Francheska B. Tambaoan, Asia Louisa U. Chong, Karol Sophia Agape R. Padilla, Rianna Patricia S. Cruz, El King D. Morado, Joshua Gregor A. Dizon, Razel Nikka M. Hao, Arianne A. Zamora, Devon Ray Pacial, Juan Antonio R. Magalang, Marissa Alejandria, Celia Carlos, Anna Ong-Lim, Edsel Maurice Salvaña, John Q. Wong, Jaime C. Montoya, Maria Rosario Singh-Vergeire and Cynthia P. Saloma
EPI_ISL_2155156	Philippine Red Cross - Port Area	Philippine Genome Center	Francis A. Tablizo, Kenneth M. Kim, Carlo M. Lapid, Marc Jerrone R. Castro, Maria Sofia L. Yangzon, Benedict A. Maralit, Marc Edsel C. Ayes, Eva Maria Cutiongco-de la Paz, Alethea R. de Guzman, Jan Michael C. Yap, Jo-Hannah S. Llames, Sheila Mae M. Araiza, Kris P. Punayan, Irish Coleen A. Asin, Candice Francheska B. Tambaoan, Asia Louisa U. Chong, Karol Sophia Agape R. Padilla, Rianna Patricia S. Cruz, El King D. Morado, Joshua Gregor A. Dizon, Razel Nikka M. Hao, Arianne A. Zamora, Devon Ray Pacial, Juan Antonio R. Magalang, Marissa Alejandria, Celia Carlos, Anna Ong-Lim, Edsel Maurice Salvaña, John Q. Wong, Jaime C. Montoya, Maria Rosario Singh-Vergeire and Cynthia P. Saloma
EPI_ISL_2155160	Laguna Provincial Hospital - San Pablo District Hospital	Philippine Genome Center	Francis A. Tablizo, Kenneth M. Kim, Carlo M. Lapid, Marc Jerrone R. Castro, Maria Sofia L. Yangzon, Benedict A. Maralit, Marc Edsel C. Ayes, Eva Maria Cutiongco-de la Paz, Alethea R. de Guzman, Jan Michael C. Yap, Jo-Hannah S. Llames, Sheila Mae M. Araiza, Kris P. Punayan, Irish Coleen A. Asin, Candice Francheska B. Tambaoan, Asia Louisa U. Chong, Karol Sophia Agape R. Padilla, Rianna Patricia S. Cruz, El King D. Morado, Joshua Gregor A. Dizon, Razel Nikka M. Hao, Arianne A. Zamora, Devon Ray Pacial, Juan Antonio R. Magalang, Marissa Alejandria, Celia Carlos, Anna Ong-Lim, Edsel Maurice Salvaña, John Q. Wong, Jaime C. Montoya, Maria Rosario Singh-Vergeire and Cynthia P. Saloma
EPI_ISL_2155162, EPI_ISL_2155164, EPI_ISL_2155167	Research Institute for Tropical Medicine, Inc. (RITM)	Philippine Genome Center	Francis A. Tablizo, Kenneth M. Kim, Carlo M. Lapid, Marc Jerrone R. Castro, Maria Sofia L. Yangzon, Benedict A. Maralit, Marc Edsel C. Ayes, Eva Maria Cutiongco-de la Paz, Alethea R. de Guzman, Jan Michael C. Yap, Jo-Hannah S. Llames, Sheila Mae M. Araiza, Kris P. Punayan, Irish Coleen A. Asin, Candice Francheska B. Tambaoan, Asia Louisa U. Chong, Karol Sophia Agape R. Padilla, Rianna Patricia S. Cruz, El King D. Morado, Joshua Gregor A. Dizon, Razel Nikka M. Hao, Arianne A. Zamora, Devon Ray Pacial, Juan Antonio R. Magalang, Marissa Alejandria, Celia Carlos, Anna Ong-Lim, Edsel Maurice Salvaña, John Q. Wong, Jaime C. Montoya, Maria Rosario Singh-Vergeire and Cynthia P. Saloma
EPI_ISL_2155168	Qualimed Hospital Sta. Rosa	Philippine Genome Center	Francis A. Tablizo, Kenneth M. Kim, Carlo M. Lapid, Marc Jerrone R. Castro, Maria Sofia L. Yangzon, Benedict A. Maralit, Marc Edsel C. Ayes, Eva Maria Cutiongco-de la Paz, Alethea R. de Guzman, Jan Michael C. Yap, Jo-Hannah S. Llames, Sheila Mae M. Araiza, Kris P. Punayan, Irish Coleen A. Asin, Candice Francheska B. Tambaoan, Asia Louisa U. Chong, Karol Sophia Agape R. Padilla, Rianna Patricia S. Cruz, El King D. Morado, Joshua Gregor A. Dizon, Razel Nikka M. Hao, Arianne A. Zamora, Devon Ray Pacial, Juan Antonio R. Magalang, Marissa Alejandria, Celia Carlos, Anna Ong-Lim, Edsel Maurice Salvaña, John Q. Wong, Jaime C. Montoya, Maria Rosario Singh-Vergeire and Cynthia P. Saloma
EPI_ISL_2155177, EPI_ISL_2155178, EPI_ISL_2155180	Research Institute for Tropical Medicine, Inc. (RITM)	Philippine Genome Center	Francis A. Tablizo, Kenneth M. Kim, Carlo M. Lapid, Marc Jerrone R. Castro, Maria Sofia L. Yangzon, Benedict A. Maralit, Marc Edsel C. Ayes, Eva Maria Cutiongco-de la Paz, Alethea R. de Guzman, Jan Michael C. Yap, Jo-Hannah S. Llames, Sheila Mae M. Araiza, Kris P. Punayan, Irish Coleen A. Asin, Candice Francheska B. Tambaoan, Asia Louisa U. Chong, Karol Sophia Agape R. Padilla, Rianna Patricia S. Cruz, El King D. Morado, Joshua Gregor A. Dizon, Razel Nikka M. Hao, Arianne A. Zamora, Devon Ray Pacial, Juan Antonio R. Magalang, Marissa Alejandria, Celia Carlos, Anna Ong-Lim, Edsel Maurice Salvaña, John Q. Wong, Jaime C. Montoya, Maria Rosario Singh-Vergeire and Cynthia P. Saloma
EPI_ISL_2155186	Qualimed Hospital Sta. Rosa	Philippine Genome Center	Francis A. Tablizo, Kenneth M. Kim, Carlo M. Lapid, Marc Jerrone R. Castro, Maria Sofia L. Yangzon, Benedict A. Maralit, Marc Edsel C. Ayes, Eva Maria Cutiongco-de la Paz, Alethea R. de Guzman, Jan Michael C. Yap, Jo-Hannah S. Llames, Sheila Mae M. Araiza, Kris P. Punayan, Irish Coleen A. Asin, Candice Francheska B. Tambaoan, Asia Louisa U. Chong, Karol Sophia Agape R. Padilla, Rianna Patricia S. Cruz, El King D. Morado, Joshua Gregor A. Dizon, Razel Nikka M. Hao, Arianne A. Zamora, Devon Ray Pacial, Juan Antonio R. Magalang, Marissa Alejandria, Celia Carlos, Anna Ong-Lim, Edsel Maurice Salvaña, John Q. Wong, Jaime C. Montoya, Maria Rosario Singh-Vergeire and Cynthia P. Saloma
EPI_ISL_2155188	Laguna Provincial Hospital - San Pablo District Hospital	Philippine Genome Center	Francis A. Tablizo, Kenneth M. Kim, Carlo M. Lapid, Marc Jerrone R. Castro, Maria Sofia L. Yangzon, Benedict A. Maralit, Marc Edsel C. Ayes, Eva Maria Cutiongco-de la Paz, Alethea R. de Guzman, Jan Michael C. Yap, Jo-Hannah S. Llames, Sheila Mae M. Araiza, Kris P. Punayan, Irish Coleen A. Asin, Candice Francheska B. Tambaoan, Asia Louisa U. Chong, Karol Sophia Agape R. Padilla, Rianna Patricia S. Cruz, El King D. Morado, Joshua Gregor A. Dizon, Razel Nikka M. Hao, Arianne A. Zamora, Devon Ray Pacial, Juan Antonio R. Magalang, Marissa Alejandria, Celia Carlos, Anna Ong-Lim, Edsel Maurice Salvaña, John Q. Wong, Jaime C. Montoya, Maria Rosario Singh-Vergeire and Cynthia P. Saloma
EPI_ISL_2155191	Cagayan Valley Medical Center Molecular Laboratory	Philippine Genome Center	Francis A. Tablizo, Kenneth M. Kim, Carlo M. Lapid, Marc Jerrone R. Castro, Maria Sofia L. Yangzon, Benedict A. Maralit, Marc Edsel C. Ayes, Eva Maria Cutiongco-de la Paz, Alethea R. de Guzman, Jan Michael C. Yap, Jo-Hannah S. Llames, Sheila Mae M. Araiza, Kris P. Punayan, Irish Coleen A. Asin, Candice Francheska B. Tambaoan, Asia Louisa U. Chong, Karol Sophia Agape R. Padilla, Rianna Patricia S. Cruz, El King D. Morado, Joshua Gregor A. Dizon, Razel Nikka M. Hao, Arianne A. Zamora, Devon Ray Pacial, Juan Antonio R. Magalang, Marissa Alejandria, Celia Carlos, Anna Ong-Lim, Edsel Maurice Salvaña, John Q. Wong, Jaime C. Montoya, Maria Rosario Singh-Vergeire and Cynthia P. Saloma
EPI_ISL_2155192, EPI_ISL_2155197	Research Institute for Tropical Medicine, Inc. (RITM)	Philippine Genome Center	Francis A. Tablizo, Kenneth M. Kim, Carlo M. Lapid, Marc Jerrone R. Castro, Maria Sofia L. Yangzon, Benedict A. Maralit, Marc Edsel C. Ayes, Eva Maria Cutiongco-de la Paz, Alethea R. de Guzman, Jan Michael C. Yap, Jo-Hannah S. Llames, Sheila Mae M. Araiza, Kris P. Punayan, Irish Coleen A. Asin, Candice Francheska B. Tambaoan, Asia Louisa U. Chong, Karol Sophia Agape R. Padilla, Rianna Patricia S. Cruz, El King D. Morado, Joshua Gregor A. Dizon, Razel Nikka M. Hao, Arianne A. Zamora, Devon Ray Pacial, Juan Antonio R. Magalang, Marissa Alejandria, Celia Carlos, Anna Ong-Lim, Edsel Maurice Salvaña, John Q. Wong, Jaime C. Montoya, Maria Rosario Singh-Vergeire and Cynthia P. Saloma
EPI_ISL_2155199	Laguna Provincial Hospital - San Pablo District Hospital	Philippine Genome Center	Francis A. Tablizo, Kenneth M. Kim, Carlo M. Lapid, Marc Jerrone R. Castro, Maria Sofia L. Yangzon, Benedict A. Maralit, Marc Edsel C. Ayes, Eva Maria Cutiongco-de la Paz, Alethea R. de Guzman, Jan Michael C. Yap, Jo-Hannah S. Llames, Sheila Mae M. Araiza, Kris P. Punayan, Irish Coleen A. Asin, Candice Francheska B. Tambaoan, Asia Louisa U. Chong, Karol Sophia Agape R. Padilla, Rianna Patricia S. Cruz, El King D. Morado, Joshua Gregor A. Dizon, Razel Nikka M. Hao, Arianne A. Zamora, Devon Ray Pacial, Juan Antonio R. Magalang, Marissa Alejandria, Celia Carlos, Anna Ong-Lim, Edsel Maurice Salvaña, John Q. Wong, Jaime C. Montoya, Maria Rosario Singh-Vergeire and Cynthia P. Saloma
EPI_ISL_2155203	Philippine Red Cross - Port Area	Philippine Genome Center	Francis A. Tablizo, Kenneth M. Kim, Carlo M. Lapid, Marc Jerrone R. Castro, Maria Sofia L. Yangzon, Benedict A. Maralit, Marc Edsel C. Ayes, Eva Maria Cutiongco-de la Paz, Alethea R. de Guzman, Jan Michael C. Yap, Jo-Hannah S. Llames, Sheila Mae M. Araiza, Kris P. Punayan, Irish Coleen A. Asin, Candice Francheska B. Tambaoan, Asia Louisa U. Chong, Karol Sophia Agape R. Padilla, Rianna Patricia S. Cruz, El King D. Morado, Joshua Gregor A. Dizon, Razel Nikka M. Hao, Arianne A. Zamora, Devon Ray Pacial, Juan Antonio R. Magalang, Marissa Alejandria, Celia Carlos, Anna Ong-Lim, Edsel Maurice Salvaña, John Q. Wong, Jaime C. Montoya, Maria Rosario Singh-Vergeire and Cynthia P. Saloma

EPI_ISL_2155206	Research Institute for Tropical Medicine, Inc. (RITM)	Philippine Genome Center	Francis A. Tablizo, Kenneth M. Kim, Carlo M. Lapid, Marc Jerrone R. Castro, Maria Sofia L. Yangzon, Benedict A. Maralit, Marc Edsel C. Ayes, Eva Maria Cutiongco-de la Paz, Alethea R. de Guzman, Jan Michael C. Yap, Jo-Hannah S. Llames, Sheila Mae M. Araiza, Kris P. Punayan, Irish Coleen A. Asin, Candice Francheska B. Tambaoan, Asia Louisa U. Chong, Karol Sophia Agape R. Padilla, Rianna Patricia S. Cruz, El King D. Morado, Joshua Gregor A. Dizon, Razel Nikka M. Hao, Arianne A. Zamora, Devon Ray Pacial, Juan Antonio R. Magalang, Marissa Alejandria, Celia Carlos, Anna Ong-Lim, Edsel Maurice Salvaña, John Q. Wong, Jaime C. Montoya, Maria Rosario Singh-Vergeire and Cynthia P. Saloma
EPI_ISL_2155207	Qualimed Hospital Sta. Rosa	Philippine Genome Center	Francis A. Tablizo, Kenneth M. Kim, Carlo M. Lapid, Marc Jerrone R. Castro, Maria Sofia L. Yangzon, Benedict A. Maralit, Marc Edsel C. Ayes, Eva Maria Cutiongco-de la Paz, Alethea R. de Guzman, Jan Michael C. Yap, Jo-Hannah S. Llames, Sheila Mae M. Araiza, Kris P. Punayan, Irish Coleen A. Asin, Candice Francheska B. Tambaoan, Asia Louisa U. Chong, Karol Sophia Agape R. Padilla, Rianna Patricia S. Cruz, El King D. Morado, Joshua Gregor A. Dizon, Razel Nikka M. Hao, Arianne A. Zamora, Devon Ray Pacial, Juan Antonio R. Magalang, Marissa Alejandria, Celia Carlos, Anna Ong-Lim, Edsel Maurice Salvaña, John Q. Wong, Jaime C. Montoya, Maria Rosario Singh-Vergeire and Cynthia P. Saloma
EPI_ISL_2155210, EPI_ISL_2155221, EPI_ISL_2155223	Research Institute for Tropical Medicine, Inc. (RITM)	Philippine Genome Center	Francis A. Tablizo, Kenneth M. Kim, Carlo M. Lapid, Marc Jerrone R. Castro, Maria Sofia L. Yangzon, Benedict A. Maralit, Marc Edsel C. Ayes, Eva Maria Cutiongco-de la Paz, Alethea R. de Guzman, Jan Michael C. Yap, Jo-Hannah S. Llames, Sheila Mae M. Araiza, Kris P. Punayan, Irish Coleen A. Asin, Candice Francheska B. Tambaoan, Asia Louisa U. Chong, Karol Sophia Agape R. Padilla, Rianna Patricia S. Cruz, El King D. Morado, Joshua Gregor A. Dizon, Razel Nikka M. Hao, Arianne A. Zamora, Devon Ray Pacial, Juan Antonio R. Magalang, Marissa Alejandria, Celia Carlos, Anna Ong-Lim, Edsel Maurice Salvaña, John Q. Wong, Jaime C. Montoya, Maria Rosario Singh-Vergeire and Cynthia P. Saloma
EPI_ISL_2155225	Philippine Red Cross - Port Area	Philippine Genome Center	Francis A. Tablizo, Kenneth M. Kim, Carlo M. Lapid, Marc Jerrone R. Castro, Maria Sofia L. Yangzon, Benedict A. Maralit, Marc Edsel C. Ayes, Eva Maria Cutiongco-de la Paz, Alethea R. de Guzman, Jan Michael C. Yap, Jo-Hannah S. Llames, Sheila Mae M. Araiza, Kris P. Punayan, Irish Coleen A. Asin, Candice Francheska B. Tambaoan, Asia Louisa U. Chong, Karol Sophia Agape R. Padilla, Rianna Patricia S. Cruz, El King D. Morado, Joshua Gregor A. Dizon, Razel Nikka M. Hao, Arianne A. Zamora, Devon Ray Pacial, Juan Antonio R. Magalang, Marissa Alejandria, Celia Carlos, Anna Ong-Lim, Edsel Maurice Salvaña, John Q. Wong, Jaime C. Montoya, Maria Rosario Singh-Vergeire and Cynthia P. Saloma
EPI_ISL_2155227	Research Institute for Tropical Medicine, Inc. (RITM)	Philippine Genome Center	Francis A. Tablizo, Kenneth M. Kim, Carlo M. Lapid, Marc Jerrone R. Castro, Maria Sofia L. Yangzon, Benedict A. Maralit, Marc Edsel C. Ayes, Eva Maria Cutiongco-de la Paz, Alethea R. de Guzman, Jan Michael C. Yap, Jo-Hannah S. Llames, Sheila Mae M. Araiza, Kris P. Punayan, Irish Coleen A. Asin, Candice Francheska B. Tambaoan, Asia Louisa U. Chong, Karol Sophia Agape R. Padilla, Rianna Patricia S. Cruz, El King D. Morado, Joshua Gregor A. Dizon, Razel Nikka M. Hao, Arianne A. Zamora, Devon Ray Pacial, Juan Antonio R. Magalang, Marissa Alejandria, Celia Carlos, Anna Ong-Lim, Edsel Maurice Salvaña, John Q. Wong, Jaime C. Montoya, Maria Rosario Singh-Vergeire and Cynthia P. Saloma
EPI_ISL_2155237	Philippine Red Cross - Port Area	Philippine Genome Center	Francis A. Tablizo, Kenneth M. Kim, Carlo M. Lapid, Marc Jerrone R. Castro, Maria Sofia L. Yangzon, Benedict A. Maralit, Marc Edsel C. Ayes, Eva Maria Cutiongco-de la Paz, Alethea R. de Guzman, Jan Michael C. Yap, Jo-Hannah S. Llames, Sheila Mae M. Araiza, Kris P. Punayan, Irish Coleen A. Asin, Candice Francheska B. Tambaoan, Asia Louisa U. Chong, Karol Sophia Agape R. Padilla, Rianna Patricia S. Cruz, El King D. Morado, Joshua Gregor A. Dizon, Razel Nikka M. Hao, Arianne A. Zamora, Devon Ray Pacial, Juan Antonio R. Magalang, Marissa Alejandria, Celia Carlos, Anna Ong-Lim, Edsel Maurice Salvaña, John Q. Wong, Jaime C. Montoya, Maria Rosario Singh-Vergeire and Cynthia P. Saloma
EPI_ISL_2155238	Cagayan Valley Medical Center Molecular Laboratory	Philippine Genome Center	Francis A. Tablizo, Kenneth M. Kim, Carlo M. Lapid, Marc Jerrone R. Castro, Maria Sofia L. Yangzon, Benedict A. Maralit, Marc Edsel C. Ayes, Eva Maria Cutiongco-de la Paz, Alethea R. de Guzman, Jan Michael C. Yap, Jo-Hannah S. Llames, Sheila Mae M. Araiza, Kris P. Punayan, Irish Coleen A. Asin, Candice Francheska B. Tambaoan, Asia Louisa U. Chong, Karol Sophia Agape R. Padilla, Rianna Patricia S. Cruz, El King D. Morado, Joshua Gregor A. Dizon, Razel Nikka M. Hao, Arianne A. Zamora, Devon Ray Pacial, Juan Antonio R. Magalang, Marissa Alejandria, Celia Carlos, Anna Ong-Lim, Edsel Maurice Salvaña, John Q. Wong, Jaime C. Montoya, Maria Rosario Singh-Vergeire and Cynthia P. Saloma
EPI_ISL_2155241	Research Institute for Tropical Medicine, Inc. (RITM)	Philippine Genome Center	Francis A. Tablizo, Kenneth M. Kim, Carlo M. Lapid, Marc Jerrone R. Castro, Maria Sofia L. Yangzon, Benedict A. Maralit, Marc Edsel C. Ayes, Eva Maria Cutiongco-de la Paz, Alethea R. de Guzman, Jan Michael C. Yap, Jo-Hannah S. Llames, Sheila Mae M. Araiza, Kris P. Punayan, Irish Coleen A. Asin, Candice Francheska B. Tambaoan, Asia Louisa U. Chong, Karol Sophia Agape R. Padilla, Rianna Patricia S. Cruz, El King D. Morado, Joshua Gregor A. Dizon, Razel Nikka M. Hao, Arianne A. Zamora, Devon Ray Pacial, Juan Antonio R. Magalang, Marissa Alejandria, Celia Carlos, Anna Ong-Lim, Edsel Maurice Salvaña, John Q. Wong, Jaime C. Montoya, Maria Rosario Singh-Vergeire and Cynthia P. Saloma
EPI_ISL_2155243	Qualimed Hospital Sta. Rosa	Philippine Genome Center	Francis A. Tablizo, Kenneth M. Kim, Carlo M. Lapid, Marc Jerrone R. Castro, Maria Sofia L. Yangzon, Benedict A. Maralit, Marc Edsel C. Ayes, Eva Maria Cutiongco-de la Paz, Alethea R. de Guzman, Jan Michael C. Yap, Jo-Hannah S. Llames, Sheila Mae M. Araiza, Kris P. Punayan, Irish Coleen A. Asin, Candice Francheska B. Tambaoan, Asia Louisa U. Chong, Karol Sophia Agape R. Padilla, Rianna Patricia S. Cruz, El King D. Morado, Joshua Gregor A. Dizon, Razel Nikka M. Hao, Arianne A. Zamora, Devon Ray Pacial, Juan Antonio R. Magalang, Marissa Alejandria, Celia Carlos, Anna Ong-Lim, Edsel Maurice Salvaña, John Q. Wong, Jaime C. Montoya, Maria Rosario Singh-Vergeire and Cynthia P. Saloma
EPI_ISL_2155244, EPI_ISL_2155248	Research Institute for Tropical Medicine, Inc. (RITM)	Philippine Genome Center	Francis A. Tablizo, Kenneth M. Kim, Carlo M. Lapid, Marc Jerrone R. Castro, Maria Sofia L. Yangzon, Benedict A. Maralit, Marc Edsel C. Ayes, Eva Maria Cutiongco-de la Paz, Alethea R. de Guzman, Jan Michael C. Yap, Jo-Hannah S. Llames, Sheila Mae M. Araiza, Kris P. Punayan, Irish Coleen A. Asin, Candice Francheska B. Tambaoan, Asia Louisa U. Chong, Karol Sophia Agape R. Padilla, Rianna Patricia S. Cruz, El King D. Morado, Joshua Gregor A. Dizon, Razel Nikka M. Hao, Arianne A. Zamora, Devon Ray Pacial, Juan Antonio R. Magalang, Marissa Alejandria, Celia Carlos, Anna Ong-Lim, Edsel Maurice Salvaña, John Q. Wong, Jaime C. Montoya, Maria Rosario Singh-Vergeire and Cynthia P. Saloma
EPI_ISL_2155254, EPI_ISL_2155260	Philippine Red Cross - Port Area	Philippine Genome Center	Francis A. Tablizo, Kenneth M. Kim, Carlo M. Lapid, Marc Jerrone R. Castro, Maria Sofia L. Yangzon, Benedict A. Maralit, Marc Edsel C. Ayes, Eva Maria Cutiongco-de la Paz, Alethea R. de Guzman, Jan Michael C. Yap, Jo-Hannah S. Llames, Sheila Mae M. Araiza, Kris P. Punayan, Irish Coleen A. Asin, Candice Francheska B. Tambaoan, Asia Louisa U. Chong, Karol Sophia Agape R. Padilla, Rianna Patricia S. Cruz, El King D. Morado, Joshua Gregor A. Dizon, Razel Nikka M. Hao, Arianne A. Zamora, Devon Ray Pacial, Juan Antonio R. Magalang, Marissa Alejandria, Celia Carlos, Anna Ong-Lim, Edsel Maurice Salvaña, John Q. Wong, Jaime C. Montoya, Maria Rosario Singh-Vergeire and Cynthia P. Saloma
EPI_ISL_2155261, EPI_ISL_2155266	Research Institute for Tropical Medicine, Inc. (RITM)	Philippine Genome Center	Francis A. Tablizo, Kenneth M. Kim, Carlo M. Lapid, Marc Jerrone R. Castro, Maria Sofia L. Yangzon, Benedict A. Maralit, Marc Edsel C. Ayes, Eva Maria Cutiongco-de la Paz, Alethea R. de Guzman, Jan Michael C. Yap, Jo-Hannah S. Llames, Sheila Mae M. Araiza, Kris P. Punayan, Irish Coleen A. Asin, Candice Francheska B. Tambaoan, Asia Louisa U. Chong, Karol Sophia Agape R. Padilla, Rianna Patricia S. Cruz, El King D. Morado, Joshua Gregor A. Dizon, Razel Nikka M. Hao, Arianne A. Zamora, Devon Ray Pacial, Juan Antonio R. Magalang, Marissa Alejandria, Celia Carlos, Anna Ong-Lim, Edsel Maurice Salvaña, John Q. Wong, Jaime C. Montoya, Maria Rosario Singh-Vergeire and Cynthia P. Saloma
EPI_ISL_2155271	Laguna Provincial Hospital - San Pablo District Hospital	Philippine Genome Center	Francis A. Tablizo, Kenneth M. Kim, Carlo M. Lapid, Marc Jerrone R. Castro, Maria Sofia L. Yangzon, Benedict A. Maralit, Marc Edsel C. Ayes, Eva Maria Cutiongco-de la Paz, Alethea R. de Guzman, Jan Michael C. Yap, Jo-Hannah S. Llames, Sheila Mae M. Araiza, Kris P. Punayan, Irish Coleen A. Asin, Candice Francheska B. Tambaoan, Asia Louisa U. Chong, Karol Sophia Agape R. Padilla, Rianna Patricia S. Cruz, El King D. Morado, Joshua Gregor A. Dizon, Razel Nikka M. Hao, Arianne A. Zamora, Devon Ray Pacial, Juan Antonio R. Magalang, Marissa Alejandria, Celia Carlos, Anna Ong-Lim, Edsel Maurice Salvaña, John Q. Wong, Jaime C. Montoya, Maria Rosario Singh-Vergeire and Cynthia P. Saloma
EPI_ISL_2155273, EPI_ISL_2155281, EPI_ISL_2155287	Research Institute for Tropical Medicine, Inc. (RITM)	Philippine Genome Center	Francis A. Tablizo, Kenneth M. Kim, Carlo M. Lapid, Marc Jerrone R. Castro, Maria Sofia L. Yangzon, Benedict A. Maralit, Marc Edsel C. Ayes, Eva Maria Cutiongco-de la Paz, Alethea R. de Guzman, Jan Michael C. Yap, Jo-Hannah S. Llames, Sheila Mae M. Araiza, Kris P. Punayan, Irish Coleen A. Asin, Candice Francheska B. Tambaoan, Asia Louisa U. Chong, Karol Sophia Agape R. Padilla, Rianna Patricia S. Cruz, El King D. Morado, Joshua Gregor A. Dizon, Razel Nikka M. Hao, Arianne A. Zamora, Devon Ray Pacial, Juan Antonio R. Magalang, Marissa Alejandria, Celia Carlos, Anna Ong-Lim, Edsel Maurice Salvaña, John Q. Wong, Jaime C. Montoya, Maria Rosario Singh-Vergeire and Cynthia P. Saloma
EPI_ISL_2155294	Philippine Red Cross - Port Area	Philippine Genome Center	Francis A. Tablizo, Kenneth M. Kim, Carlo M. Lapid, Marc Jerrone R. Castro, Maria Sofia L. Yangzon, Benedict A. Maralit, Marc Edsel C. Ayes, Eva Maria Cutiongco-de la Paz, Alethea R. de Guzman, Jan Michael C. Yap, Jo-Hannah S. Llames, Sheila Mae M. Araiza, Kris P. Punayan, Irish Coleen A. Asin, Candice Francheska B. Tambaoan, Asia Louisa U. Chong, Karol Sophia Agape R. Padilla, Rianna Patricia S. Cruz, El King D. Morado, Joshua Gregor A. Dizon, Razel Nikka M. Hao, Arianne A. Zamora, Devon Ray Pacial, Juan Antonio R. Magalang, Marissa Alejandria, Celia Carlos, Anna Ong-Lim, Edsel Maurice Salvaña, John Q. Wong, Jaime C. Montoya, Maria Rosario Singh-Vergeire and Cynthia P. Saloma
EPI_ISL_2155296	Research Institute for Tropical Medicine, Inc. (RITM)	Philippine Genome Center	Francis A. Tablizo, Kenneth M. Kim, Carlo M. Lapid, Marc Jerrone R. Castro, Maria Sofia L. Yangzon, Benedict A. Maralit, Marc Edsel C. Ayes, Eva Maria Cutiongco-de la Paz, Alethea R. de Guzman, Jan Michael C. Yap, Jo-Hannah S. Llames, Sheila Mae M. Araiza, Kris P. Punayan, Irish Coleen A. Asin, Candice Francheska B. Tambaoan, Asia Louisa U. Chong, Karol Sophia Agape R. Padilla, Rianna Patricia S. Cruz, El King D. Morado, Joshua Gregor A. Dizon, Razel Nikka M. Hao, Arianne A. Zamora, Devon Ray Pacial, Juan Antonio R. Magalang, Marissa Alejandria, Celia Carlos, Anna Ong-Lim, Edsel Maurice Salvaña, John Q. Wong, Jaime C. Montoya, Maria Rosario Singh-Vergeire and Cynthia P. Saloma

EPI_ISL_2155302	Philippine Children's Medical Center	Philippine Genome Center	Francis A. Tablizo, Kenneth M. Kim, Carlo M. Lapid, Marc Jerrone R. Castro, Maria Sofia L. Yangzon, Benedict A. Maralit, Marc Edsel C. Ayes, Eva Maria Cutiongco-de la Paz, Alethea R. de Guzman, Jan Michael C. Yap, Jo-Hannah S. Llames, Sheila Mae M. Araiza, Kris P. Punayan, Irish Coleen A. Asin, Candice Francheska B. Tambaoan, Asia Louisa U. Chong, Karol Sophia Agape R. Padilla, Rianna Patricia S. Cruz, El King D. Morado, Joshua Gregor A. Dizon, Razel Nikka M. Hao, Arianne A. Zamora, Devon Ray Pacial, Juan Antonio R. Magalang, Marissa Alejandria, Celia Carlos, Anna Ong-Lim, Edsel Maurice Salvaña, John Q. Wong, Jaime C. Montoya, Maria Rosario Singh-Vergeire and Cynthia P. Saloma
EPI_ISL_2155303	Philippine Red Cross - National Blood Center	Philippine Genome Center	Francis A. Tablizo, Kenneth M. Kim, Carlo M. Lapid, Marc Jerrone R. Castro, Maria Sofia L. Yangzon, Benedict A. Maralit, Marc Edsel C. Ayes, Eva Maria Cutiongco-de la Paz, Alethea R. de Guzman, Jan Michael C. Yap, Jo-Hannah S. Llames, Sheila Mae M. Araiza, Kris P. Punayan, Irish Coleen A. Asin, Candice Francheska B. Tambaoan, Asia Louisa U. Chong, Karol Sophia Agape R. Padilla, Rianna Patricia S. Cruz, El King D. Morado, Joshua Gregor A. Dizon, Razel Nikka M. Hao, Arianne A. Zamora, Devon Ray Pacial, Juan Antonio R. Magalang, Marissa Alejandria, Celia Carlos, Anna Ong-Lim, Edsel Maurice Salvaña, John Q. Wong, Jaime C. Montoya, Maria Rosario Singh-Vergeire and Cynthia P. Saloma
EPI_ISL_2155307, EPI_ISL_2155312, EPI_ISL_2155314, EPI_ISL_2155322	Research Institute for Tropical Medicine, Inc. (RITM)	Philippine Genome Center	Francis A. Tablizo, Kenneth M. Kim, Carlo M. Lapid, Marc Jerrone R. Castro, Maria Sofia L. Yangzon, Benedict A. Maralit, Marc Edsel C. Ayes, Eva Maria Cutiongco-de la Paz, Alethea R. de Guzman, Jan Michael C. Yap, Jo-Hannah S. Llames, Sheila Mae M. Araiza, Kris P. Punayan, Irish Coleen A. Asin, Candice Francheska B. Tambaoan, Asia Louisa U. Chong, Karol Sophia Agape R. Padilla, Rianna Patricia S. Cruz, El King D. Morado, Joshua Gregor A. Dizon, Razel Nikka M. Hao, Arianne A. Zamora, Devon Ray Pacial, Juan Antonio R. Magalang, Marissa Alejandria, Celia Carlos, Anna Ong-Lim, Edsel Maurice Salvaña, John Q. Wong, Jaime C. Montoya, Maria Rosario Singh-Vergeire and Cynthia P. Saloma
EPI_ISL_2155325	Philippine Children's Medical Center	Philippine Genome Center	Francis A. Tablizo, Kenneth M. Kim, Carlo M. Lapid, Marc Jerrone R. Castro, Maria Sofia L. Yangzon, Benedict A. Maralit, Marc Edsel C. Ayes, Eva Maria Cutiongco-de la Paz, Alethea R. de Guzman, Jan Michael C. Yap, Jo-Hannah S. Llames, Sheila Mae M. Araiza, Kris P. Punayan, Irish Coleen A. Asin, Candice Francheska B. Tambaoan, Asia Louisa U. Chong, Karol Sophia Agape R. Padilla, Rianna Patricia S. Cruz, El King D. Morado, Joshua Gregor A. Dizon, Razel Nikka M. Hao, Arianne A. Zamora, Devon Ray Pacial, Juan Antonio R. Magalang, Marissa Alejandria, Celia Carlos, Anna Ong-Lim, Edsel Maurice Salvaña, John Q. Wong, Jaime C. Montoya, Maria Rosario Singh-Vergeire and Cynthia P. Saloma
EPI_ISL_2155326, EPI_ISL_2155332, EPI_ISL_2155334, EPI_ISL_2155335	Research Institute for Tropical Medicine, Inc. (RITM)	Philippine Genome Center	Francis A. Tablizo, Kenneth M. Kim, Carlo M. Lapid, Marc Jerrone R. Castro, Maria Sofia L. Yangzon, Benedict A. Maralit, Marc Edsel C. Ayes, Eva Maria Cutiongco-de la Paz, Alethea R. de Guzman, Jan Michael C. Yap, Jo-Hannah S. Llames, Sheila Mae M. Araiza, Kris P. Punayan, Irish Coleen A. Asin, Candice Francheska B. Tambaoan, Asia Louisa U. Chong, Karol Sophia Agape R. Padilla, Rianna Patricia S. Cruz, El King D. Morado, Joshua Gregor A. Dizon, Razel Nikka M. Hao, Arianne A. Zamora, Devon Ray Pacial, Juan Antonio R. Magalang, Marissa Alejandria, Celia Carlos, Anna Ong-Lim, Edsel Maurice Salvaña, John Q. Wong, Jaime C. Montoya, Maria Rosario Singh-Vergeire and Cynthia P. Saloma
EPI_ISL_2155337	Cagayan Valley Medical Center Molecular Laboratory	Philippine Genome Center	Francis A. Tablizo, Kenneth M. Kim, Carlo M. Lapid, Marc Jerrone R. Castro, Maria Sofia L. Yangzon, Benedict A. Maralit, Marc Edsel C. Ayes, Eva Maria Cutiongco-de la Paz, Alethea R. de Guzman, Jan Michael C. Yap, Jo-Hannah S. Llames, Sheila Mae M. Araiza, Kris P. Punayan, Irish Coleen A. Asin, Candice Francheska B. Tambaoan, Asia Louisa U. Chong, Karol Sophia Agape R. Padilla, Rianna Patricia S. Cruz, El King D. Morado, Joshua Gregor A. Dizon, Razel Nikka M. Hao, Arianne A. Zamora, Devon Ray Pacial, Juan Antonio R. Magalang, Marissa Alejandria, Celia Carlos, Anna Ong-Lim, Edsel Maurice Salvaña, John Q. Wong, Jaime C. Montoya, Maria Rosario Singh-Vergeire and Cynthia P. Saloma
EPI_ISL_2155340	Research Institute for Tropical Medicine, Inc. (RITM)	Philippine Genome Center	Francis A. Tablizo, Kenneth M. Kim, Carlo M. Lapid, Marc Jerrone R. Castro, Maria Sofia L. Yangzon, Benedict A. Maralit, Marc Edsel C. Ayes, Eva Maria Cutiongco-de la Paz, Alethea R. de Guzman, Jan Michael C. Yap, Jo-Hannah S. Llames, Sheila Mae M. Araiza, Kris P. Punayan, Irish Coleen A. Asin, Candice Francheska B. Tambaoan, Asia Louisa U. Chong, Karol Sophia Agape R. Padilla, Rianna Patricia S. Cruz, El King D. Morado, Joshua Gregor A. Dizon, Razel Nikka M. Hao, Arianne A. Zamora, Devon Ray Pacial, Juan Antonio R. Magalang, Marissa Alejandria, Celia Carlos, Anna Ong-Lim, Edsel Maurice Salvaña, John Q. Wong, Jaime C. Montoya, Maria Rosario Singh-Vergeire and Cynthia P. Saloma
EPI_ISL_2155344	Qualimed Hospital Sta. Rosa	Philippine Genome Center	Francis A. Tablizo, Kenneth M. Kim, Carlo M. Lapid, Marc Jerrone R. Castro, Maria Sofia L. Yangzon, Benedict A. Maralit, Marc Edsel C. Ayes, Eva Maria Cutiongco-de la Paz, Alethea R. de Guzman, Jan Michael C. Yap, Jo-Hannah S. Llames, Sheila Mae M. Araiza, Kris P. Punayan, Irish Coleen A. Asin, Candice Francheska B. Tambaoan, Asia Louisa U. Chong, Karol Sophia Agape R. Padilla, Rianna Patricia S. Cruz, El King D. Morado, Joshua Gregor A. Dizon, Razel Nikka M. Hao, Arianne A. Zamora, Devon Ray Pacial, Juan Antonio R. Magalang, Marissa Alejandria, Celia Carlos, Anna Ong-Lim, Edsel Maurice Salvaña, John Q. Wong, Jaime C. Montoya, Maria Rosario Singh-Vergeire and Cynthia P. Saloma
EPI_ISL_2155347	Research Institute for Tropical Medicine, Inc. (RITM)	Philippine Genome Center	Francis A. Tablizo, Kenneth M. Kim, Carlo M. Lapid, Marc Jerrone R. Castro, Maria Sofia L. Yangzon, Benedict A. Maralit, Marc Edsel C. Ayes, Eva Maria Cutiongco-de la Paz, Alethea R. de Guzman, Jan Michael C. Yap, Jo-Hannah S. Llames, Sheila Mae M. Araiza, Kris P. Punayan, Irish Coleen A. Asin, Candice Francheska B. Tambaoan, Asia Louisa U. Chong, Karol Sophia Agape R. Padilla, Rianna Patricia S. Cruz, El King D. Morado, Joshua Gregor A. Dizon, Razel Nikka M. Hao, Arianne A. Zamora, Devon Ray Pacial, Juan Antonio R. Magalang, Marissa Alejandria, Celia Carlos, Anna Ong-Lim, Edsel Maurice Salvaña, John Q. Wong, Jaime C. Montoya, Maria Rosario Singh-Vergeire and Cynthia P. Saloma
EPI_ISL_2155348	Philippine Children's Medical Center	Philippine Genome Center	Francis A. Tablizo, Kenneth M. Kim, Carlo M. Lapid, Marc Jerrone R. Castro, Maria Sofia L. Yangzon, Benedict A. Maralit, Marc Edsel C. Ayes, Eva Maria Cutiongco-de la Paz, Alethea R. de Guzman, Jan Michael C. Yap, Jo-Hannah S. Llames, Sheila Mae M. Araiza, Kris P. Punayan, Irish Coleen A. Asin, Candice Francheska B. Tambaoan, Asia Louisa U. Chong, Karol Sophia Agape R. Padilla, Rianna Patricia S. Cruz, El King D. Morado, Joshua Gregor A. Dizon, Razel Nikka M. Hao, Arianne A. Zamora, Devon Ray Pacial, Juan Antonio R. Magalang, Marissa Alejandria, Celia Carlos, Anna Ong-Lim, Edsel Maurice Salvaña, John Q. Wong, Jaime C. Montoya, Maria Rosario Singh-Vergeire and Cynthia P. Saloma
EPI_ISL_2155349	Philippine Red Cross - Port Area	Philippine Genome Center	Francis A. Tablizo, Kenneth M. Kim, Carlo M. Lapid, Marc Jerrone R. Castro, Maria Sofia L. Yangzon, Benedict A. Maralit, Marc Edsel C. Ayes, Eva Maria Cutiongco-de la Paz, Alethea R. de Guzman, Jan Michael C. Yap, Jo-Hannah S. Llames, Sheila Mae M. Araiza, Kris P. Punayan, Irish Coleen A. Asin, Candice Francheska B. Tambaoan, Asia Louisa U. Chong, Karol Sophia Agape R. Padilla, Rianna Patricia S. Cruz, El King D. Morado, Joshua Gregor A. Dizon, Razel Nikka M. Hao, Arianne A. Zamora, Devon Ray Pacial, Juan Antonio R. Magalang, Marissa Alejandria, Celia Carlos, Anna Ong-Lim, Edsel Maurice Salvaña, John Q. Wong, Jaime C. Montoya, Maria Rosario Singh-Vergeire and Cynthia P. Saloma
EPI_ISL_2155350	Research Institute for Tropical Medicine, Inc. (RITM)	Philippine Genome Center	Francis A. Tablizo, Kenneth M. Kim, Carlo M. Lapid, Marc Jerrone R. Castro, Maria Sofia L. Yangzon, Benedict A. Maralit, Marc Edsel C. Ayes, Eva Maria Cutiongco-de la Paz, Alethea R. de Guzman, Jan Michael C. Yap, Jo-Hannah S. Llames, Sheila Mae M. Araiza, Kris P. Punayan, Irish Coleen A. Asin, Candice Francheska B. Tambaoan, Asia Louisa U. Chong, Karol Sophia Agape R. Padilla, Rianna Patricia S. Cruz, El King D. Morado, Joshua Gregor A. Dizon, Razel Nikka M. Hao, Arianne A. Zamora, Devon Ray Pacial, Juan Antonio R. Magalang, Marissa Alejandria, Celia Carlos, Anna Ong-Lim, Edsel Maurice Salvaña, John Q. Wong, Jaime C. Montoya, Maria Rosario Singh-Vergeire and Cynthia P. Saloma
EPI_ISL_2155357	Cagayan Valley Medical Center Molecular Laboratory	Philippine Genome Center	Francis A. Tablizo, Kenneth M. Kim, Carlo M. Lapid, Marc Jerrone R. Castro, Maria Sofia L. Yangzon, Benedict A. Maralit, Marc Edsel C. Ayes, Eva Maria Cutiongco-de la Paz, Alethea R. de Guzman, Jan Michael C. Yap, Jo-Hannah S. Llames, Sheila Mae M. Araiza, Kris P. Punayan, Irish Coleen A. Asin, Candice Francheska B. Tambaoan, Asia Louisa U. Chong, Karol Sophia Agape R. Padilla, Rianna Patricia S. Cruz, El King D. Morado, Joshua Gregor A. Dizon, Razel Nikka M. Hao, Arianne A. Zamora, Devon Ray Pacial, Juan Antonio R. Magalang, Marissa Alejandria, Celia Carlos, Anna Ong-Lim, Edsel Maurice Salvaña, John Q. Wong, Jaime C. Montoya, Maria Rosario Singh-Vergeire and Cynthia P. Saloma
EPI_ISL_2155412, EPI_ISL_2155414	Fatima University Medical Center Antipolo Corp.	Philippine Genome Center	Francis A. Tablizo, Kenneth M. Kim, Carlo M. Lapid, Marc Jerrone R. Castro, Maria Sofia L. Yangzon, Benedict A. Maralit, Marc Edsel C. Ayes, Eva Maria Cutiongco-de la Paz, Alethea R. de Guzman, Jan Michael C. Yap, Jo-Hannah S. Llames, Sheila Mae M. Araiza, Kris P. Punayan, Irish Coleen A. Asin, Candice Francheska B. Tambaoan, Asia Louisa U. Chong, Karol Sophia Agape R. Padilla, Rianna Patricia S. Cruz, El King D. Morado, Joshua Gregor A. Dizon, Razel Nikka M. Hao, Arianne A. Zamora, Devon Ray Pacial, Juan Antonio R. Magalang, Marissa Alejandria, Celia Carlos, Anna Ong-Lim, Edsel Maurice Salvaña, John Q. Wong, Jaime C. Montoya, Maria Rosario Singh-Vergeire and Cynthia P. Saloma
EPI_ISL_2155438, EPI_ISL_2155446, EPI_ISL_2155447, EPI_ISL_2155450, EPI_ISL_2155452, EPI_ISL_2155458	Philippine Red Cross - National Blood Center	Philippine Genome Center	Francis A. Tablizo, Kenneth M. Kim, Carlo M. Lapid, Marc Jerrone R. Castro, Maria Sofia L. Yangzon, Benedict A. Maralit, Marc Edsel C. Ayes, Eva Maria Cutiongco-de la Paz, Alethea R. de Guzman, Jan Michael C. Yap, Jo-Hannah S. Llames, Sheila Mae M. Araiza, Kris P. Punayan, Irish Coleen A. Asin, Candice Francheska B. Tambaoan, Asia Louisa U. Chong, Karol Sophia Agape R. Padilla, Rianna Patricia S. Cruz, El King D. Morado, Joshua Gregor A. Dizon, Razel Nikka M. Hao, Arianne A. Zamora, Devon Ray Pacial, Juan Antonio R. Magalang, Marissa Alejandria, Celia Carlos, Anna Ong-Lim, Edsel Maurice Salvaña, John Q. Wong, Jaime C. Montoya, Maria Rosario Singh-Vergeire and Cynthia P. Saloma
EPI_ISL_2155525, EPI_ISL_2155527, EPI_ISL_2155531, EPI_ISL_2155532, EPI_ISL_2155554, EPI_ISL_2155555, EPI_ISL_2155561, EPI_ISL_2155569, EPI_ISL_2155570, EPI_ISL_2155577, EPI_ISL_2155579, EPI_ISL_2155580, EPI_ISL_2155581			
see above	UPLB Covid-19 Molecular Diagnostic Laboratory	Philippine Genome Center	Francis A. Tablizo, Kenneth M. Kim, Carlo M. Lapid, Marc Jerrone R. Castro, Maria Sofia L. Yangzon, Benedict A. Maralit, Marc Edsel C. Ayes, Eva Maria Cutiongco-de la Paz, Alethea R. de Guzman, Jan Michael C. Yap, Jo-Hannah S. Llames, Sheila Mae M. Araiza, Kris P. Punayan, Irish Coleen A. Asin, Candice Francheska B. Tambaoan, Asia Louisa U. Chong, Karol Sophia Agape R. Padilla, Rianna Patricia S. Cruz, El King D. Morado, Joshua Gregor A. Dizon, Razel

			Nikka M. Hao, Arianne A. Zamora, Devon Ray Pacial, Juan Antonio R. Magalang, Marissa Alejandria, Celia Carlos, Anna Ong-Lim, Edsel Maurice Salvaña, John Q. Wong, Jaime C. Montoya, Maria Rosario Singh-Vergeire and Cynthia P. Saloma
EPI_ISL_2155611, EPI_ISL_2155614, EPI_ISL_2155616, EPI_ISL_2155618, EPI_ISL_2155619, EPI_ISL_2155620, EPI_ISL_2155626, EPI_ISL_2155628, EPI_ISL_2155631, EPI_ISL_2155632, EPI_ISL_2155634, EPI_ISL_2155637			
see above	Research Institute for Tropical Medicine, Inc. (RITM)	Philippine Genome Center	Francis A. Tablizo, Kenneth M. Kim, Carlo M. Lapid, Marc Jerrone R. Castro, Maria Sofia L. Yangzon, Benedict A. Maralit, Marc Edsel C. Ayes, Eva Maria Cutiongco-de la Paz, Alethea R. de Guzman, Jan Michael C. Yap, Jo-Hannah S. Llames, Sheila Mae M. Araiza, Kris P. Punayan, Irish Coleen A. Asin, Candice Francheska B. Tambaoan, Asia Louisa U. Chong, Karol Sophia Agape R. Padilla, Rianna Patricia S. Cruz, El King D. Morado, Joshua Gregor A. Dizon, Razel Nikka M. Hao, Arianne A. Zamora, Devon Ray Pacial, Juan Antonio R. Magalang, Marissa Alejandria, Celia Carlos, Anna Ong-Lim, Edsel Maurice Salvaña, John Q. Wong, Jaime C. Montoya, Maria Rosario Singh-Vergeire and Cynthia P. Saloma
EPI_ISL_2155657	Ilocos Training and Regional Medical Center	Philippine Genome Center	Francis A. Tablizo, Kenneth M. Kim, Carlo M. Lapid, Marc Jerrone R. Castro, Maria Sofia L. Yangzon, Benedict A. Maralit, Marc Edsel C. Ayes, Eva Maria Cutiongco-de la Paz, Alethea R. de Guzman, Jan Michael C. Yap, Jo-Hannah S. Llames, Sheila Mae M. Araiza, Kris P. Punayan, Irish Coleen A. Asin, Candice Francheska B. Tambaoan, Asia Louisa U. Chong, Karol Sophia Agape R. Padilla, Rianna Patricia S. Cruz, El King D. Morado, Joshua Gregor A. Dizon, Razel Nikka M. Hao, Arianne A. Zamora, Devon Ray Pacial, Juan Antonio R. Magalang, Marissa Alejandria, Celia Carlos, Anna Ong-Lim, Edsel Maurice Salvaña, John Q. Wong, Jaime C. Montoya, Maria Rosario Singh-Vergeire and Cynthia P. Saloma
EPI_ISL_2155705	Cagayan Valley Medical Center Molecular Laboratory	Philippine Genome Center	Francis A. Tablizo, Kenneth M. Kim, Carlo M. Lapid, Marc Jerrone R. Castro, Maria Sofia L. Yangzon, Benedict A. Maralit, Marc Edsel C. Ayes, Eva Maria Cutiongco-de la Paz, Alethea R. de Guzman, Jan Michael C. Yap, Jo-Hannah S. Llames, Sheila Mae M. Araiza, Kris P. Punayan, Irish Coleen A. Asin, Candice Francheska B. Tambaoan, Asia Louisa U. Chong, Karol Sophia Agape R. Padilla, Rianna Patricia S. Cruz, El King D. Morado, Joshua Gregor A. Dizon, Razel Nikka M. Hao, Arianne A. Zamora, Devon Ray Pacial, Juan Antonio R. Magalang, Marissa Alejandria, Celia Carlos, Anna Ong-Lim, Edsel Maurice Salvaña, John Q. Wong, Jaime C. Montoya, Maria Rosario Singh-Vergeire and Cynthia P. Saloma
EPI_ISL_2155733, EPI_ISL_2155753	Philippine Red Cross Logistics and Multipurpose Center	Philippine Genome Center	Francis A. Tablizo, Kenneth M. Kim, Carlo M. Lapid, Marc Jerrone R. Castro, Maria Sofia L. Yangzon, Benedict A. Maralit, Marc Edsel C. Ayes, Eva Maria Cutiongco-de la Paz, Alethea R. de Guzman, Jan Michael C. Yap, Jo-Hannah S. Llames, Sheila Mae M. Araiza, Kris P. Punayan, Irish Coleen A. Asin, Candice Francheska B. Tambaoan, Asia Louisa U. Chong, Karol Sophia Agape R. Padilla, Rianna Patricia S. Cruz, El King D. Morado, Joshua Gregor A. Dizon, Razel Nikka M. Hao, Arianne A. Zamora, Devon Ray Pacial, Juan Antonio R. Magalang, Marissa Alejandria, Celia Carlos, Anna Ong-Lim, Edsel Maurice Salvaña, John Q. Wong, Jaime C. Montoya, Maria Rosario Singh-Vergeire and Cynthia P. Saloma
EPI_ISL_2155756, EPI_ISL_2155758, EPI_ISL_2155759	First Aide Diagnostic Center	Philippine Genome Center	Francis A. Tablizo, Kenneth M. Kim, Carlo M. Lapid, Marc Jerrone R. Castro, Maria Sofia L. Yangzon, Benedict A. Maralit, Marc Edsel C. Ayes, Eva Maria Cutiongco-de la Paz, Alethea R. de Guzman, Jan Michael C. Yap, Jo-Hannah S. Llames, Sheila Mae M. Araiza, Kris P. Punayan, Irish Coleen A. Asin, Candice Francheska B. Tambaoan, Asia Louisa U. Chong, Karol Sophia Agape R. Padilla, Rianna Patricia S. Cruz, El King D. Morado, Joshua Gregor A. Dizon, Razel Nikka M. Hao, Arianne A. Zamora, Devon Ray Pacial, Juan Antonio R. Magalang, Marissa Alejandria, Celia Carlos, Anna Ong-Lim, Edsel Maurice Salvaña, John Q. Wong, Jaime C. Montoya, Maria Rosario Singh-Vergeire and Cynthia P. Saloma
EPI_ISL_2155760, EPI_ISL_2155761	Qualimed Hospital Sta. Rosa	Philippine Genome Center	Francis A. Tablizo, Kenneth M. Kim, Carlo M. Lapid, Marc Jerrone R. Castro, Maria Sofia L. Yangzon, Benedict A. Maralit, Marc Edsel C. Ayes, Eva Maria Cutiongco-de la Paz, Alethea R. de Guzman, Jan Michael C. Yap, Jo-Hannah S. Llames, Sheila Mae M. Araiza, Kris P. Punayan, Irish Coleen A. Asin, Candice Francheska B. Tambaoan, Asia Louisa U. Chong, Karol Sophia Agape R. Padilla, Rianna Patricia S. Cruz, El King D. Morado, Joshua Gregor A. Dizon, Razel Nikka M. Hao, Arianne A. Zamora, Devon Ray Pacial, Juan Antonio R. Magalang, Marissa Alejandria, Celia Carlos, Anna Ong-Lim, Edsel Maurice Salvaña, John Q. Wong, Jaime C. Montoya, Maria Rosario Singh-Vergeire and Cynthia P. Saloma
EPI_ISL_2155767, EPI_ISL_2155768, EPI_ISL_2155770, EPI_ISL_2155771, EPI_ISL_2155774, EPI_ISL_2155775, EPI_ISL_2155779, EPI_ISL_2155780, EPI_ISL_2155782	Philippine Red Cross - Port Area	Philippine Genome Center	Francis A. Tablizo, Kenneth M. Kim, Carlo M. Lapid, Marc Jerrone R. Castro, Maria Sofia L. Yangzon, Benedict A. Maralit, Marc Edsel C. Ayes, Eva Maria Cutiongco-de la Paz, Alethea R. de Guzman, Jan Michael C. Yap, Jo-Hannah S. Llames, Sheila Mae M. Araiza, Kris P. Punayan, Irish Coleen A. Asin, Candice Francheska B. Tambaoan, Asia Louisa U. Chong, Karol Sophia Agape R. Padilla, Rianna Patricia S. Cruz, El King D. Morado, Joshua Gregor A. Dizon, Razel Nikka M. Hao, Arianne A. Zamora, Devon Ray Pacial, Juan Antonio R. Magalang, Marissa Alejandria, Celia Carlos, Anna Ong-Lim, Edsel Maurice Salvaña, John Q. Wong, Jaime C. Montoya, Maria Rosario Singh-Vergeire and Cynthia P. Saloma
EPI_ISL_2155803, EPI_ISL_2155805, EPI_ISL_2155808	Safeguard DNA Diagnostic, Inc.	Philippine Genome Center	Francis A. Tablizo, Kenneth M. Kim, Carlo M. Lapid, Marc Jerrone R. Castro, Maria Sofia L. Yangzon, Benedict A. Maralit, Marc Edsel C. Ayes, Eva Maria Cutiongco-de la Paz, Alethea R. de Guzman, Jan Michael C. Yap, Jo-Hannah S. Llames, Sheila Mae M. Araiza, Kris P. Punayan, Irish Coleen A. Asin, Candice Francheska B. Tambaoan, Asia Louisa U. Chong, Karol Sophia Agape R. Padilla, Rianna Patricia S. Cruz, El King D. Morado, Joshua Gregor A. Dizon, Razel Nikka M. Hao, Arianne A. Zamora, Devon Ray Pacial, Juan Antonio R. Magalang, Marissa Alejandria, Celia Carlos, Anna Ong-Lim, Edsel Maurice Salvaña, John Q. Wong, Jaime C. Montoya, Maria Rosario Singh-Vergeire and Cynthia P. Saloma
EPI_ISL_2155816	Southern Isabela Medical Center Molecular Diagnostic & Research Laboratory (SIMC - MDRL)	Philippine Genome Center	Francis A. Tablizo, Kenneth M. Kim, Carlo M. Lapid, Marc Jerrone R. Castro, Maria Sofia L. Yangzon, Benedict A. Maralit, Marc Edsel C. Ayes, Eva Maria Cutiongco-de la Paz, Alethea R. de Guzman, Jan Michael C. Yap, Jo-Hannah S. Llames, Sheila Mae M. Araiza, Kris P. Punayan, Irish Coleen A. Asin, Candice Francheska B. Tambaoan, Asia Louisa U. Chong, Karol Sophia Agape R. Padilla, Rianna Patricia S. Cruz, El King D. Morado, Joshua Gregor A. Dizon, Razel Nikka M. Hao, Arianne A. Zamora, Devon Ray Pacial, Juan Antonio R. Magalang, Marissa Alejandria, Celia Carlos, Anna Ong-Lim, Edsel Maurice Salvaña, John Q. Wong, Jaime C. Montoya, Maria Rosario Singh-Vergeire and Cynthia P. Saloma
EPI_ISL_2156120	Manila Doctors Hospital	Philippine Genome Center	Francis A. Tablizo, Kenneth M. Kim, Carlo M. Lapid, Marc Jerrone R. Castro, Maria Sofia L. Yangzon, Benedict A. Maralit, Marc Edsel C. Ayes, Eva Maria Cutiongco-de la Paz, Alethea R. de Guzman, Jan Michael C. Yap, Jo-Hannah S. Llames, Sheila Mae M. Araiza, Kris P. Punayan, Irish Coleen A. Asin, Candice Francheska B. Tambaoan, Asia Louisa U. Chong, Karol Sophia Agape R. Padilla, Rianna Patricia S. Cruz, El King D. Morado, Joshua Gregor A. Dizon, Razel Nikka M. Hao, Arianne A. Zamora, Devon Ray Pacial, Juan Antonio R. Magalang, Marissa Alejandria, Celia Carlos, Anna Ong-Lim, Edsel Maurice Salvaña, John Q. Wong, Jaime C. Montoya, Maria Rosario Singh-Vergeire and Cynthia P. Saloma
EPI_ISL_2156131	Baguio General Hospital Medical Center (BGHMC)	Philippine Genome Center	Francis A. Tablizo, Kenneth M. Kim, Carlo M. Lapid, Marc Jerrone R. Castro, Maria Sofia L. Yangzon, Benedict A. Maralit, Marc Edsel C. Ayes, Eva Maria Cutiongco-de la Paz, Alethea R. de Guzman, Jan Michael C. Yap, Jo-Hannah S. Llames, Sheila Mae M. Araiza, Kris P. Punayan, Irish Coleen A. Asin, Candice Francheska B. Tambaoan, Asia Louisa U. Chong, Karol Sophia Agape R. Padilla, Rianna Patricia S. Cruz, El King D. Morado, Joshua Gregor A. Dizon, Razel Nikka M. Hao, Arianne A. Zamora, Devon Ray Pacial, Juan Antonio R. Magalang, Marissa Alejandria, Celia Carlos, Anna Ong-Lim, Edsel Maurice Salvaña, John Q. Wong, Jaime C. Montoya, Maria Rosario Singh-Vergeire and Cynthia P. Saloma
EPI_ISL_2156170, EPI_ISL_2156171, EPI_ISL_2156175, EPI_ISL_2156180, EPI_ISL_2156182, EPI_ISL_2156183	Research Institute for Tropical Medicine, Inc. (RITM)	Philippine Genome Center	Francis A. Tablizo, Kenneth M. Kim, Carlo M. Lapid, Marc Jerrone R. Castro, Maria Sofia L. Yangzon, Benedict A. Maralit, Marc Edsel C. Ayes, Eva Maria Cutiongco-de la Paz, Alethea R. de Guzman, Jan Michael C. Yap, Jo-Hannah S. Llames, Sheila Mae M. Araiza, Kris P. Punayan, Irish Coleen A. Asin, Candice Francheska B. Tambaoan, Asia Louisa U. Chong, Karol Sophia Agape R. Padilla, Rianna Patricia S. Cruz, El King D. Morado, Joshua Gregor A. Dizon, Razel Nikka M. Hao, Arianne A. Zamora, Devon Ray Pacial, Juan Antonio R. Magalang, Marissa Alejandria, Celia Carlos, Anna Ong-Lim, Edsel Maurice Salvaña, John Q. Wong, Jaime C. Montoya, Maria Rosario Singh-Vergeire and Cynthia P. Saloma
EPI_ISL_2156207, EPI_ISL_2156209, EPI_ISL_2156212, EPI_ISL_2156213, EPI_ISL_2156215, EPI_ISL_2156216	Cagayan Valley Medical Center Molecular Laboratory	Philippine Genome Center	Francis A. Tablizo, Kenneth M. Kim, Carlo M. Lapid, Marc Jerrone R. Castro, Maria Sofia L. Yangzon, Benedict A. Maralit, Marc Edsel C. Ayes, Eva Maria Cutiongco-de la Paz, Alethea R. de Guzman, Jan Michael C. Yap, Jo-Hannah S. Llames, Sheila Mae M. Araiza, Kris P. Punayan, Irish Coleen A. Asin, Candice Francheska B. Tambaoan, Asia Louisa U. Chong, Karol Sophia Agape R. Padilla, Rianna Patricia S. Cruz, El King D. Morado, Joshua Gregor A. Dizon, Razel Nikka M. Hao, Arianne A. Zamora, Devon Ray Pacial, Juan Antonio R. Magalang, Marissa Alejandria, Celia Carlos, Anna Ong-Lim, Edsel Maurice Salvaña, John Q. Wong, Jaime C. Montoya, Maria Rosario Singh-Vergeire and Cynthia P. Saloma
EPI_ISL_2156617	Research Institute for Tropical Medicine, Inc. (RITM)	Philippine Genome Center	Francis A. Tablizo, Kenneth M. Kim, Carlo M. Lapid, Marc Jerrone R. Castro, Maria Sofia L. Yangzon, Elcid Aaron R. Pangilinan, Benedict A. Maralit, Marc Edsel C. Ayes, Eva Maria Cutiongco-de la Paz, Alethea R. de Guzman, Jan Michael C. Yap, Jo-Hannah S. Llames, Sheila Mae M. Araiza, Kris P. Punayan, Irish Coleen A. Asin, Candice Francheska B. Tambaoan, Asia Louisa U. Chong, Karol Sophia Agape R. Padilla, Rianna Patricia S. Cruz, El King D. Morado, Joshua Gregor A. Dizon, Razel Nikka M. Hao, Arianne A. Zamora, Devon Ray Pacial, Juan Antonio R. Magalang, Marissa Alejandria, Celia Carlos, Anna Ong-Lim, Edsel Maurice Salvaña, John Q. Wong, Jaime C. Montoya, Maria Rosario Singh-Vergeire and Cynthia P. Saloma
EPI_ISL_2156623	Baguio General Hospital Medical Center (BGHMC)	Philippine Genome Center	Francis A. Tablizo, Kenneth M. Kim, Carlo M. Lapid, Marc Jerrone R. Castro, Maria Sofia L. Yangzon, Elcid Aaron R. Pangilinan, Benedict A. Maralit, Marc Edsel C. Ayes, Eva Maria Cutiongco-de la Paz, Alethea R. de Guzman, Jan Michael C. Yap, Jo-Hannah S. Llames, Sheila Mae M. Araiza, Kris P. Punayan, Irish Coleen A. Asin, Candice Francheska B. Tambaoan, Asia Louisa U. Chong, Karol Sophia Agape R. Padilla, Rianna Patricia S. Cruz, El King D. Morado, Joshua Gregor A. Dizon, Razel Nikka M. Hao, Arianne A. Zamora, Devon Ray Pacial, Juan Antonio R. Magalang, Marissa Alejandria, Celia Carlos, Anna Ong-Lim, Edsel Maurice Salvaña, John Q. Wong, Jaime C. Montoya, Maria Rosario Singh-Vergeire and Cynthia P. Saloma
EPI_ISL_2156624	University of the East Ramon Magsaysay Memorial Medical	Philippine Genome Center	Francis A. Tablizo, Kenneth M. Kim, Carlo M. Lapid, Marc Jerrone R. Castro, Maria Sofia L. Yangzon, Elcid Aaron R. Pangilinan, Benedict A. Maralit, Marc

	Center (UERM)		Edsel C. Ayes, Eva Maria Cutiongco-de la Paz, Alethea R. de Guzman, Jan Michael C. Yap, Jo-Hannah S. Llames, Sheila Mae M. Araiza, Kris P. Punayan, Irish Coleen A. Asin, Candice Francheska B. Tambaoan, Asia Louisa U. Chong, Karol Sophia Agape R. Padilla, Rianna Patricia S. Cruz, El King D. Morado, Joshua Gregor A. Dizon, Razel Nikka M. Hao, Arianne A. Zamora, Devon Ray Pacial, Juan Antonio R. Magalang, Marissa Alejandria, Celia Carlos, Anna Ong-Lim, Edsel Maurice Salvaña, John Q. Wong, Jaime C. Montoya, Maria Rosario Singh-Vergeire and Cynthia P. Saloma
EPI_ISL_2156625	Cagayan Valley Medical Center Molecular Laboratory	Philippine Genome Center	Francis A. Tablizo, Kenneth M. Kim, Carlo M. Lapid, Marc Jerrone R. Castro, Maria Sofia L. Yangzon, Elcid Aaron R. Pangilinan, Benedict A. Maralit, Marc Edsel C. Ayes, Eva Maria Cutiongco-de la Paz, Alethea R. de Guzman, Jan Michael C. Yap, Jo-Hannah S. Llames, Sheila Mae M. Araiza, Kris P. Punayan, Irish Coleen A. Asin, Candice Francheska B. Tambaoan, Asia Louisa U. Chong, Karol Sophia Agape R. Padilla, Rianna Patricia S. Cruz, El King D. Morado, Joshua Gregor A. Dizon, Razel Nikka M. Hao, Arianne A. Zamora, Devon Ray Pacial, Juan Antonio R. Magalang, Marissa Alejandria, Celia Carlos, Anna Ong-Lim, Edsel Maurice Salvaña, John Q. Wong, Jaime C. Montoya, Maria Rosario Singh-Vergeire and Cynthia P. Saloma
EPI_ISL_2156629	Baguio General Hospital Medical Center (BGHMC)	Philippine Genome Center	Francis A. Tablizo, Kenneth M. Kim, Carlo M. Lapid, Marc Jerrone R. Castro, Maria Sofia L. Yangzon, Elcid Aaron R. Pangilinan, Benedict A. Maralit, Marc Edsel C. Ayes, Eva Maria Cutiongco-de la Paz, Alethea R. de Guzman, Jan Michael C. Yap, Jo-Hannah S. Llames, Sheila Mae M. Araiza, Kris P. Punayan, Irish Coleen A. Asin, Candice Francheska B. Tambaoan, Asia Louisa U. Chong, Karol Sophia Agape R. Padilla, Rianna Patricia S. Cruz, El King D. Morado, Joshua Gregor A. Dizon, Razel Nikka M. Hao, Arianne A. Zamora, Devon Ray Pacial, Juan Antonio R. Magalang, Marissa Alejandria, Celia Carlos, Anna Ong-Lim, Edsel Maurice Salvaña, John Q. Wong, Jaime C. Montoya, Maria Rosario Singh-Vergeire and Cynthia P. Saloma
EPI_ISL_2156631, EPI_ISL_2156634, EPI_ISL_2156635	Research Institute for Tropical Medicine, Inc. (RITM)	Philippine Genome Center	Francis A. Tablizo, Kenneth M. Kim, Carlo M. Lapid, Marc Jerrone R. Castro, Maria Sofia L. Yangzon, Elcid Aaron R. Pangilinan, Benedict A. Maralit, Marc Edsel C. Ayes, Eva Maria Cutiongco-de la Paz, Alethea R. de Guzman, Jan Michael C. Yap, Jo-Hannah S. Llames, Sheila Mae M. Araiza, Kris P. Punayan, Irish Coleen A. Asin, Candice Francheska B. Tambaoan, Asia Louisa U. Chong, Karol Sophia Agape R. Padilla, Rianna Patricia S. Cruz, El King D. Morado, Joshua Gregor A. Dizon, Razel Nikka M. Hao, Arianne A. Zamora, Devon Ray Pacial, Juan Antonio R. Magalang, Marissa Alejandria, Celia Carlos, Anna Ong-Lim, Edsel Maurice Salvaña, John Q. Wong, Jaime C. Montoya, Maria Rosario Singh-Vergeire and Cynthia P. Saloma
EPI_ISL_2156637	Laguna Provincial Hospital - San Pablo District Hospital	Philippine Genome Center	Francis A. Tablizo, Kenneth M. Kim, Carlo M. Lapid, Marc Jerrone R. Castro, Maria Sofia L. Yangzon, Elcid Aaron R. Pangilinan, Benedict A. Maralit, Marc Edsel C. Ayes, Eva Maria Cutiongco-de la Paz, Alethea R. de Guzman, Jan Michael C. Yap, Jo-Hannah S. Llames, Sheila Mae M. Araiza, Kris P. Punayan, Irish Coleen A. Asin, Candice Francheska B. Tambaoan, Asia Louisa U. Chong, Karol Sophia Agape R. Padilla, Rianna Patricia S. Cruz, El King D. Morado, Joshua Gregor A. Dizon, Razel Nikka M. Hao, Arianne A. Zamora, Devon Ray Pacial, Juan Antonio R. Magalang, Marissa Alejandria, Celia Carlos, Anna Ong-Lim, Edsel Maurice Salvaña, John Q. Wong, Jaime C. Montoya, Maria Rosario Singh-Vergeire and Cynthia P. Saloma
EPI_ISL_2156638, EPI_ISL_2156639, EPI_ISL_2156643, EPI_ISL_2156645, EPI_ISL_2156647, EPI_ISL_2156648, EPI_ISL_2156649	Research Institute for Tropical Medicine, Inc. (RITM)	Philippine Genome Center	Francis A. Tablizo, Kenneth M. Kim, Carlo M. Lapid, Marc Jerrone R. Castro, Maria Sofia L. Yangzon, Elcid Aaron R. Pangilinan, Benedict A. Maralit, Marc Edsel C. Ayes, Eva Maria Cutiongco-de la Paz, Alethea R. de Guzman, Jan Michael C. Yap, Jo-Hannah S. Llames, Sheila Mae M. Araiza, Kris P. Punayan, Irish Coleen A. Asin, Candice Francheska B. Tambaoan, Asia Louisa U. Chong, Karol Sophia Agape R. Padilla, Rianna Patricia S. Cruz, El King D. Morado, Joshua Gregor A. Dizon, Razel Nikka M. Hao, Arianne A. Zamora, Devon Ray Pacial, Juan Antonio R. Magalang, Marissa Alejandria, Celia Carlos, Anna Ong-Lim, Edsel Maurice Salvaña, John Q. Wong, Jaime C. Montoya, Maria Rosario Singh-Vergeire and Cynthia P. Saloma
EPI_ISL_2156650, EPI_ISL_2156652	Cagayan Valley Medical Center Molecular Laboratory	Philippine Genome Center	Francis A. Tablizo, Kenneth M. Kim, Carlo M. Lapid, Marc Jerrone R. Castro, Maria Sofia L. Yangzon, Elcid Aaron R. Pangilinan, Benedict A. Maralit, Marc Edsel C. Ayes, Eva Maria Cutiongco-de la Paz, Alethea R. de Guzman, Jan Michael C. Yap, Jo-Hannah S. Llames, Sheila Mae M. Araiza, Kris P. Punayan, Irish Coleen A. Asin, Candice Francheska B. Tambaoan, Asia Louisa U. Chong, Karol Sophia Agape R. Padilla, Rianna Patricia S. Cruz, El King D. Morado, Joshua Gregor A. Dizon, Razel Nikka M. Hao, Arianne A. Zamora, Devon Ray Pacial, Juan Antonio R. Magalang, Marissa Alejandria, Celia Carlos, Anna Ong-Lim, Edsel Maurice Salvaña, John Q. Wong, Jaime C. Montoya, Maria Rosario Singh-Vergeire and Cynthia P. Saloma
EPI_ISL_2156657, EPI_ISL_2156664, EPI_ISL_2156665	Research Institute for Tropical Medicine, Inc. (RITM)	Philippine Genome Center	Francis A. Tablizo, Kenneth M. Kim, Carlo M. Lapid, Marc Jerrone R. Castro, Maria Sofia L. Yangzon, Elcid Aaron R. Pangilinan, Benedict A. Maralit, Marc Edsel C. Ayes, Eva Maria Cutiongco-de la Paz, Alethea R. de Guzman, Jan Michael C. Yap, Jo-Hannah S. Llames, Sheila Mae M. Araiza, Kris P. Punayan, Irish Coleen A. Asin, Candice Francheska B. Tambaoan, Asia Louisa U. Chong, Karol Sophia Agape R. Padilla, Rianna Patricia S. Cruz, El King D. Morado, Joshua Gregor A. Dizon, Razel Nikka M. Hao, Arianne A. Zamora, Devon Ray Pacial, Juan Antonio R. Magalang, Marissa Alejandria, Celia Carlos, Anna Ong-Lim, Edsel Maurice Salvaña, John Q. Wong, Jaime C. Montoya, Maria Rosario Singh-Vergeire and Cynthia P. Saloma
EPI_ISL_2156666, EPI_ISL_2156668	Southern Isabela Medical Center Molecular Diagnostic & Research Laboratory (SIMC - MDRL)	Philippine Genome Center	Francis A. Tablizo, Kenneth M. Kim, Carlo M. Lapid, Marc Jerrone R. Castro, Maria Sofia L. Yangzon, Elcid Aaron R. Pangilinan, Benedict A. Maralit, Marc Edsel C. Ayes, Eva Maria Cutiongco-de la Paz, Alethea R. de Guzman, Jan Michael C. Yap, Jo-Hannah S. Llames, Sheila Mae M. Araiza, Kris P. Punayan, Irish Coleen A. Asin, Candice Francheska B. Tambaoan, Asia Louisa U. Chong, Karol Sophia Agape R. Padilla, Rianna Patricia S. Cruz, El King D. Morado, Joshua Gregor A. Dizon, Razel Nikka M. Hao, Arianne A. Zamora, Devon Ray Pacial, Juan Antonio R. Magalang, Marissa Alejandria, Celia Carlos, Anna Ong-Lim, Edsel Maurice Salvaña, John Q. Wong, Jaime C. Montoya, Maria Rosario Singh-Vergeire and Cynthia P. Saloma
EPI_ISL_2156670, EPI_ISL_2156674, EPI_ISL_2156675	Research Institute for Tropical Medicine, Inc. (RITM)	Philippine Genome Center	Francis A. Tablizo, Kenneth M. Kim, Carlo M. Lapid, Marc Jerrone R. Castro, Maria Sofia L. Yangzon, Elcid Aaron R. Pangilinan, Benedict A. Maralit, Marc Edsel C. Ayes, Eva Maria Cutiongco-de la Paz, Alethea R. de Guzman, Jan Michael C. Yap, Jo-Hannah S. Llames, Sheila Mae M. Araiza, Kris P. Punayan, Irish Coleen A. Asin, Candice Francheska B. Tambaoan, Asia Louisa U. Chong, Karol Sophia Agape R. Padilla, Rianna Patricia S. Cruz, El King D. Morado, Joshua Gregor A. Dizon, Razel Nikka M. Hao, Arianne A. Zamora, Devon Ray Pacial, Juan Antonio R. Magalang, Marissa Alejandria, Celia Carlos, Anna Ong-Lim, Edsel Maurice Salvaña, John Q. Wong, Jaime C. Montoya, Maria Rosario Singh-Vergeire and Cynthia P. Saloma
EPI_ISL_2156677, EPI_ISL_2156678	Southern Isabela Medical Center Molecular Diagnostic & Research Laboratory (SIMC - MDRL)	Philippine Genome Center	Francis A. Tablizo, Kenneth M. Kim, Carlo M. Lapid, Marc Jerrone R. Castro, Maria Sofia L. Yangzon, Elcid Aaron R. Pangilinan, Benedict A. Maralit, Marc Edsel C. Ayes, Eva Maria Cutiongco-de la Paz, Alethea R. de Guzman, Jan Michael C. Yap, Jo-Hannah S. Llames, Sheila Mae M. Araiza, Kris P. Punayan, Irish Coleen A. Asin, Candice Francheska B. Tambaoan, Asia Louisa U. Chong, Karol Sophia Agape R. Padilla, Rianna Patricia S. Cruz, El King D. Morado, Joshua Gregor A. Dizon, Razel Nikka M. Hao, Arianne A. Zamora, Devon Ray Pacial, Juan Antonio R. Magalang, Marissa Alejandria, Celia Carlos, Anna Ong-Lim, Edsel Maurice Salvaña, John Q. Wong, Jaime C. Montoya, Maria Rosario Singh-Vergeire and Cynthia P. Saloma
EPI_ISL_2156679	Cagayan Valley Medical Center Molecular Laboratory	Philippine Genome Center	Francis A. Tablizo, Kenneth M. Kim, Carlo M. Lapid, Marc Jerrone R. Castro, Maria Sofia L. Yangzon, Elcid Aaron R. Pangilinan, Benedict A. Maralit, Marc Edsel C. Ayes, Eva Maria Cutiongco-de la Paz, Alethea R. de Guzman, Jan Michael C. Yap, Jo-Hannah S. Llames, Sheila Mae M. Araiza, Kris P. Punayan, Irish Coleen A. Asin, Candice Francheska B. Tambaoan, Asia Louisa U. Chong, Karol Sophia Agape R. Padilla, Rianna Patricia S. Cruz, El King D. Morado, Joshua Gregor A. Dizon, Razel Nikka M. Hao, Arianne A. Zamora, Devon Ray Pacial, Juan Antonio R. Magalang, Marissa Alejandria, Celia Carlos, Anna Ong-Lim, Edsel Maurice Salvaña, John Q. Wong, Jaime C. Montoya, Maria Rosario Singh-Vergeire and Cynthia P. Saloma
EPI_ISL_2156681	Research Institute for Tropical Medicine, Inc. (RITM)	Philippine Genome Center	Francis A. Tablizo, Kenneth M. Kim, Carlo M. Lapid, Marc Jerrone R. Castro, Maria Sofia L. Yangzon, Elcid Aaron R. Pangilinan, Benedict A. Maralit, Marc Edsel C. Ayes, Eva Maria Cutiongco-de la Paz, Alethea R. de Guzman, Jan Michael C. Yap, Jo-Hannah S. Llames, Sheila Mae M. Araiza, Kris P. Punayan, Irish Coleen A. Asin, Candice Francheska B. Tambaoan, Asia Louisa U. Chong, Karol Sophia Agape R. Padilla, Rianna Patricia S. Cruz, El King D. Morado, Joshua Gregor A. Dizon, Razel Nikka M. Hao, Arianne A. Zamora, Devon Ray Pacial, Juan Antonio R. Magalang, Marissa Alejandria, Celia Carlos, Anna Ong-Lim, Edsel Maurice Salvaña, John Q. Wong, Jaime C. Montoya, Maria Rosario Singh-Vergeire and Cynthia P. Saloma
EPI_ISL_2156682	Cagayan Valley Medical Center Molecular Laboratory	Philippine Genome Center	Francis A. Tablizo, Kenneth M. Kim, Carlo M. Lapid, Marc Jerrone R. Castro, Maria Sofia L. Yangzon, Elcid Aaron R. Pangilinan, Benedict A. Maralit, Marc Edsel C. Ayes, Eva Maria Cutiongco-de la Paz, Alethea R. de Guzman, Jan Michael C. Yap, Jo-Hannah S. Llames, Sheila Mae M. Araiza, Kris P. Punayan, Irish Coleen A. Asin, Candice Francheska B. Tambaoan, Asia Louisa U. Chong, Karol Sophia Agape R. Padilla, Rianna Patricia S. Cruz, El King D. Morado, Joshua Gregor A. Dizon, Razel Nikka M. Hao, Arianne A. Zamora, Devon Ray Pacial, Juan Antonio R. Magalang, Marissa Alejandria, Celia Carlos, Anna Ong-Lim, Edsel Maurice Salvaña, John Q. Wong, Jaime C. Montoya, Maria Rosario Singh-Vergeire and Cynthia P. Saloma
EPI_ISL_2156687, EPI_ISL_2156688, EPI_ISL_2156693	Southern Isabela Medical Center Molecular Diagnostic & Research Laboratory (SIMC - MDRL)	Philippine Genome Center	Francis A. Tablizo, Kenneth M. Kim, Carlo M. Lapid, Marc Jerrone R. Castro, Maria Sofia L. Yangzon, Elcid Aaron R. Pangilinan, Benedict A. Maralit, Marc Edsel C. Ayes, Eva Maria Cutiongco-de la Paz, Alethea R. de Guzman, Jan Michael C. Yap, Jo-Hannah S. Llames, Sheila Mae M. Araiza, Kris P. Punayan, Irish Coleen A. Asin, Candice Francheska B. Tambaoan, Asia Louisa U. Chong, Karol Sophia Agape R. Padilla, Rianna Patricia S. Cruz, El King D. Morado, Joshua Gregor A. Dizon, Razel Nikka M. Hao, Arianne A. Zamora, Devon Ray Pacial, Juan Antonio R. Magalang, Marissa Alejandria, Celia Carlos, Anna Ong-Lim, Edsel Maurice Salvaña, John Q. Wong, Jaime C. Montoya, Maria Rosario Singh-Vergeire and Cynthia P. Saloma
EPI_ISL_2156697	Manila Doctors Hospital	Philippine Genome Center	Francis A. Tablizo, Kenneth M. Kim, Carlo M. Lapid, Marc Jerrone R. Castro, Maria Sofia L. Yangzon, Elcid Aaron R. Pangilinan, Benedict A. Maralit, Marc Edsel C. Ayes, Eva Maria Cutiongco-de la Paz, Alethea R. de Guzman, Jan Michael C. Yap, Jo-Hannah S. Llames, Sheila Mae M. Araiza, Kris P. Punayan, Irish Coleen A. Asin, Candice Francheska B. Tambaoan, Asia Louisa U. Chong, Karol Sophia Agape R. Padilla, Rianna Patricia S. Cruz, El King D. Morado, Joshua Gregor A. Dizon, Razel Nikka M. Hao, Arianne A. Zamora, Devon Ray Pacial, Juan Antonio R. Magalang, Marissa Alejandria, Celia Carlos, Anna Ong-Lim, Edsel Maurice Salvaña, John Q. Wong, Jaime C. Montoya, Maria Rosario Singh-Vergeire and Cynthia P. Saloma

EPI_ISL_2156698, EPI_ISL_2156699	Research Institute for Tropical Medicine, Inc. (RITM)	Philippine Genome Center	Francis A. Tablizo, Kenneth M. Kim, Carlo M. Lapid, Marc Jerrone R. Castro, Maria Sofia L. Yangzon, Elcid Aaron R. Pangilinan, Benedict A. Maralit, Marc Edsel C. Ayes, Eva Maria Cutiongco-de la Paz, Alethea R. de Guzman, Jan Michael C. Yap, Jo-Hannah S. Llames, Sheila Mae M. Araiza, Kris P. Punayan, Irish Coleen A. Asin, Candice Francheska B. Tambaoan, Asia Louisa U. Chong, Karol Sophia Agape R. Padilla, Rianna Patricia S. Cruz, El King D. Morado, Joshua Gregor A. Dizon, Razel Nikka M. Hao, Arianne A. Zamora, Devon Ray Pacial, Juan Antonio R. Magalang, Marissa Alejandria, Celia Carlos, Anna Ong-Lim, Edsel Maurice Salvaña, John Q. Wong, Jaime C. Montoya, Maria Rosario Singh-Vergeire and Cynthia P. Saloma
EPI_ISL_2156700, EPI_ISL_2156705	Cagayan Valley Medical Center Molecular Laboratory	Philippine Genome Center	Francis A. Tablizo, Kenneth M. Kim, Carlo M. Lapid, Marc Jerrone R. Castro, Maria Sofia L. Yangzon, Elcid Aaron R. Pangilinan, Benedict A. Maralit, Marc Edsel C. Ayes, Eva Maria Cutiongco-de la Paz, Alethea R. de Guzman, Jan Michael C. Yap, Jo-Hannah S. Llames, Sheila Mae M. Araiza, Kris P. Punayan, Irish Coleen A. Asin, Candice Francheska B. Tambaoan, Asia Louisa U. Chong, Karol Sophia Agape R. Padilla, Rianna Patricia S. Cruz, El King D. Morado, Joshua Gregor A. Dizon, Razel Nikka M. Hao, Arianne A. Zamora, Devon Ray Pacial, Juan Antonio R. Magalang, Marissa Alejandria, Celia Carlos, Anna Ong-Lim, Edsel Maurice Salvaña, John Q. Wong, Jaime C. Montoya, Maria Rosario Singh-Vergeire and Cynthia P. Saloma
EPI_ISL_2156707, EPI_ISL_2156709	Research Institute for Tropical Medicine, Inc. (RITM)	Philippine Genome Center	Francis A. Tablizo, Kenneth M. Kim, Carlo M. Lapid, Marc Jerrone R. Castro, Maria Sofia L. Yangzon, Elcid Aaron R. Pangilinan, Benedict A. Maralit, Marc Edsel C. Ayes, Eva Maria Cutiongco-de la Paz, Alethea R. de Guzman, Jan Michael C. Yap, Jo-Hannah S. Llames, Sheila Mae M. Araiza, Kris P. Punayan, Irish Coleen A. Asin, Candice Francheska B. Tambaoan, Asia Louisa U. Chong, Karol Sophia Agape R. Padilla, Rianna Patricia S. Cruz, El King D. Morado, Joshua Gregor A. Dizon, Razel Nikka M. Hao, Arianne A. Zamora, Devon Ray Pacial, Juan Antonio R. Magalang, Marissa Alejandria, Celia Carlos, Anna Ong-Lim, Edsel Maurice Salvaña, John Q. Wong, Jaime C. Montoya, Maria Rosario Singh-Vergeire and Cynthia P. Saloma
EPI_ISL_2156711	UPLB Covid-19 Molecular Diagnostic Laboratory	Philippine Genome Center	Francis A. Tablizo, Kenneth M. Kim, Carlo M. Lapid, Marc Jerrone R. Castro, Maria Sofia L. Yangzon, Elcid Aaron R. Pangilinan, Benedict A. Maralit, Marc Edsel C. Ayes, Eva Maria Cutiongco-de la Paz, Alethea R. de Guzman, Jan Michael C. Yap, Jo-Hannah S. Llames, Sheila Mae M. Araiza, Kris P. Punayan, Irish Coleen A. Asin, Candice Francheska B. Tambaoan, Asia Louisa U. Chong, Karol Sophia Agape R. Padilla, Rianna Patricia S. Cruz, El King D. Morado, Joshua Gregor A. Dizon, Razel Nikka M. Hao, Arianne A. Zamora, Devon Ray Pacial, Juan Antonio R. Magalang, Marissa Alejandria, Celia Carlos, Anna Ong-Lim, Edsel Maurice Salvaña, John Q. Wong, Jaime C. Montoya, Maria Rosario Singh-Vergeire and Cynthia P. Saloma
EPI_ISL_2156712	Cagayan Valley Medical Center Molecular Laboratory	Philippine Genome Center	Francis A. Tablizo, Kenneth M. Kim, Carlo M. Lapid, Marc Jerrone R. Castro, Maria Sofia L. Yangzon, Elcid Aaron R. Pangilinan, Benedict A. Maralit, Marc Edsel C. Ayes, Eva Maria Cutiongco-de la Paz, Alethea R. de Guzman, Jan Michael C. Yap, Jo-Hannah S. Llames, Sheila Mae M. Araiza, Kris P. Punayan, Irish Coleen A. Asin, Candice Francheska B. Tambaoan, Asia Louisa U. Chong, Karol Sophia Agape R. Padilla, Rianna Patricia S. Cruz, El King D. Morado, Joshua Gregor A. Dizon, Razel Nikka M. Hao, Arianne A. Zamora, Devon Ray Pacial, Juan Antonio R. Magalang, Marissa Alejandria, Celia Carlos, Anna Ong-Lim, Edsel Maurice Salvaña, John Q. Wong, Jaime C. Montoya, Maria Rosario Singh-Vergeire and Cynthia P. Saloma
EPI_ISL_2156716, EPI_ISL_2156721, EPI_ISL_2156726, EPI_ISL_2156728	Research Institute for Tropical Medicine, Inc. (RITM)	Philippine Genome Center	Francis A. Tablizo, Kenneth M. Kim, Carlo M. Lapid, Marc Jerrone R. Castro, Maria Sofia L. Yangzon, Elcid Aaron R. Pangilinan, Benedict A. Maralit, Marc Edsel C. Ayes, Eva Maria Cutiongco-de la Paz, Alethea R. de Guzman, Jan Michael C. Yap, Jo-Hannah S. Llames, Sheila Mae M. Araiza, Kris P. Punayan, Irish Coleen A. Asin, Candice Francheska B. Tambaoan, Asia Louisa U. Chong, Karol Sophia Agape R. Padilla, Rianna Patricia S. Cruz, El King D. Morado, Joshua Gregor A. Dizon, Razel Nikka M. Hao, Arianne A. Zamora, Devon Ray Pacial, Juan Antonio R. Magalang, Marissa Alejandria, Celia Carlos, Anna Ong-Lim, Edsel Maurice Salvaña, John Q. Wong, Jaime C. Montoya, Maria Rosario Singh-Vergeire and Cynthia P. Saloma
EPI_ISL_2156729, EPI_ISL_2156732, EPI_ISL_2156733, EPI_ISL_2156734	Cagayan Valley Medical Center Molecular Laboratory	Philippine Genome Center	Francis A. Tablizo, Kenneth M. Kim, Carlo M. Lapid, Marc Jerrone R. Castro, Maria Sofia L. Yangzon, Elcid Aaron R. Pangilinan, Benedict A. Maralit, Marc Edsel C. Ayes, Eva Maria Cutiongco-de la Paz, Alethea R. de Guzman, Jan Michael C. Yap, Jo-Hannah S. Llames, Sheila Mae M. Araiza, Kris P. Punayan, Irish Coleen A. Asin, Candice Francheska B. Tambaoan, Asia Louisa U. Chong, Karol Sophia Agape R. Padilla, Rianna Patricia S. Cruz, El King D. Morado, Joshua Gregor A. Dizon, Razel Nikka M. Hao, Arianne A. Zamora, Devon Ray Pacial, Juan Antonio R. Magalang, Marissa Alejandria, Celia Carlos, Anna Ong-Lim, Edsel Maurice Salvaña, John Q. Wong, Jaime C. Montoya, Maria Rosario Singh-Vergeire and Cynthia P. Saloma
EPI_ISL_2156736, EPI_ISL_2156740, EPI_ISL_2156753	Research Institute for Tropical Medicine, Inc. (RITM)	Philippine Genome Center	Francis A. Tablizo, Kenneth M. Kim, Carlo M. Lapid, Marc Jerrone R. Castro, Maria Sofia L. Yangzon, Elcid Aaron R. Pangilinan, Benedict A. Maralit, Marc Edsel C. Ayes, Eva Maria Cutiongco-de la Paz, Alethea R. de Guzman, Jan Michael C. Yap, Jo-Hannah S. Llames, Sheila Mae M. Araiza, Kris P. Punayan, Irish Coleen A. Asin, Candice Francheska B. Tambaoan, Asia Louisa U. Chong, Karol Sophia Agape R. Padilla, Rianna Patricia S. Cruz, El King D. Morado, Joshua Gregor A. Dizon, Razel Nikka M. Hao, Arianne A. Zamora, Devon Ray Pacial, Juan Antonio R. Magalang, Marissa Alejandria, Celia Carlos, Anna Ong-Lim, Edsel Maurice Salvaña, John Q. Wong, Jaime C. Montoya, Maria Rosario Singh-Vergeire and Cynthia P. Saloma
EPI_ISL_2156754, EPI_ISL_2156757	Cagayan Valley Medical Center Molecular Laboratory	Philippine Genome Center	Francis A. Tablizo, Kenneth M. Kim, Carlo M. Lapid, Marc Jerrone R. Castro, Maria Sofia L. Yangzon, Elcid Aaron R. Pangilinan, Benedict A. Maralit, Marc Edsel C. Ayes, Eva Maria Cutiongco-de la Paz, Alethea R. de Guzman, Jan Michael C. Yap, Jo-Hannah S. Llames, Sheila Mae M. Araiza, Kris P. Punayan, Irish Coleen A. Asin, Candice Francheska B. Tambaoan, Asia Louisa U. Chong, Karol Sophia Agape R. Padilla, Rianna Patricia S. Cruz, El King D. Morado, Joshua Gregor A. Dizon, Razel Nikka M. Hao, Arianne A. Zamora, Devon Ray Pacial, Juan Antonio R. Magalang, Marissa Alejandria, Celia Carlos, Anna Ong-Lim, Edsel Maurice Salvaña, John Q. Wong, Jaime C. Montoya, Maria Rosario Singh-Vergeire and Cynthia P. Saloma
EPI_ISL_2156760	University of the Philippines National Institutes of Health (UP NIH)	Philippine Genome Center	Francis A. Tablizo, Kenneth M. Kim, Carlo M. Lapid, Marc Jerrone R. Castro, Maria Sofia L. Yangzon, Elcid Aaron R. Pangilinan, Benedict A. Maralit, Marc Edsel C. Ayes, Eva Maria Cutiongco-de la Paz, Alethea R. de Guzman, Jan Michael C. Yap, Jo-Hannah S. Llames, Sheila Mae M. Araiza, Kris P. Punayan, Irish Coleen A. Asin, Candice Francheska B. Tambaoan, Asia Louisa U. Chong, Karol Sophia Agape R. Padilla, Rianna Patricia S. Cruz, El King D. Morado, Joshua Gregor A. Dizon, Razel Nikka M. Hao, Arianne A. Zamora, Devon Ray Pacial, Juan Antonio R. Magalang, Marissa Alejandria, Celia Carlos, Anna Ong-Lim, Edsel Maurice Salvaña, John Q. Wong, Jaime C. Montoya, Maria Rosario Singh-Vergeire and Cynthia P. Saloma
EPI_ISL_2156763	Cagayan Valley Medical Center Molecular Laboratory	Philippine Genome Center	Francis A. Tablizo, Kenneth M. Kim, Carlo M. Lapid, Marc Jerrone R. Castro, Maria Sofia L. Yangzon, Elcid Aaron R. Pangilinan, Benedict A. Maralit, Marc Edsel C. Ayes, Eva Maria Cutiongco-de la Paz, Alethea R. de Guzman, Jan Michael C. Yap, Jo-Hannah S. Llames, Sheila Mae M. Araiza, Kris P. Punayan, Irish Coleen A. Asin, Candice Francheska B. Tambaoan, Asia Louisa U. Chong, Karol Sophia Agape R. Padilla, Rianna Patricia S. Cruz, El King D. Morado, Joshua Gregor A. Dizon, Razel Nikka M. Hao, Arianne A. Zamora, Devon Ray Pacial, Juan Antonio R. Magalang, Marissa Alejandria, Celia Carlos, Anna Ong-Lim, Edsel Maurice Salvaña, John Q. Wong, Jaime C. Montoya, Maria Rosario Singh-Vergeire and Cynthia P. Saloma
EPI_ISL_2156766	University of the Philippines National Institutes of Health (UP NIH)	Philippine Genome Center	Francis A. Tablizo, Kenneth M. Kim, Carlo M. Lapid, Marc Jerrone R. Castro, Maria Sofia L. Yangzon, Elcid Aaron R. Pangilinan, Benedict A. Maralit, Marc Edsel C. Ayes, Eva Maria Cutiongco-de la Paz, Alethea R. de Guzman, Jan Michael C. Yap, Jo-Hannah S. Llames, Sheila Mae M. Araiza, Kris P. Punayan, Irish Coleen A. Asin, Candice Francheska B. Tambaoan, Asia Louisa U. Chong, Karol Sophia Agape R. Padilla, Rianna Patricia S. Cruz, El King D. Morado, Joshua Gregor A. Dizon, Razel Nikka M. Hao, Arianne A. Zamora, Devon Ray Pacial, Juan Antonio R. Magalang, Marissa Alejandria, Celia Carlos, Anna Ong-Lim, Edsel Maurice Salvaña, John Q. Wong, Jaime C. Montoya, Maria Rosario Singh-Vergeire and Cynthia P. Saloma
EPI_ISL_2157345, EPI_ISL_2157346, EPI_ISL_2157577	Red - Regional de Vigilancia Genómica del COVID-19	Laboratory of Respiratory Viruses and Measles, Oswaldo Cruz Institute, FIOCRUZ	Paola Resende, Mitzi Castro, Claudia Díaz, Sandra Paola Paz, Luciana Appolinario, Fernando Motta, Anna Carolina Paixao, Ana Carolina Mendonca, Alice Sampaio Rocha, Taina Venas, Eilisa Cavalcante Pereira, Renata Serrano Lopes, Marilda Siqueira on behalf of the Fiocruz COVID-19 Genomic Surveillance Network
EPI_ISL_2171041	Dhulikhel Hospital, Kathmandu University Hospital	Molecular and Genomics Research Lab, Dhulikhel Hospital, Kathmandu University Hospital	Rajeev Shrestha, Nishan Katuwal, Navin Adhikari, Manu Vanaerschot, Dipesh Tamrakar, Meghnath Dhimal, Pradip Gyanwali, Saroj Bhattarai, Surendra Kumar Madhup
EPI_ISL_2171266	Janamaitri Hospital	Molecular and Genomics Research Lab, Dhulikhel Hospital, Kathmandu University Hospital	Rajeev Shrestha, Nishan Katuwal, Navin Adhikari, Dipesh Tamrakar, Meghnath Dhimal, Pradip Gyanwali, Saroj Bhattarai, Surendra Kumar Madhup
EPI_ISL_2178470	Hospital	National Reference Center for Viruses of Respiratory Infections, Institut Pasteur, Paris	Marion Barbet, Sylvie Behillil, Méline Bizard, Angela Brisebarre, Camille Capel, Vincent Enouf, Louise Lefrançois, Frédéric Lemoine, Christophe Malabat, Corinne Maufrais, Pierre Lechat, Etienne Simon-Lorière, Maud Vanpeene, Sylvie Van der Werf ,Laura Djamdjian
EPI_ISL_2188323	Hospital	National Reference Center for Viruses of Respiratory Infections, Institut Pasteur, Paris	Marion Barbet, Sylvie Behillil, Méline Bizard, Angela Brisebarre, Camille Capel, Vincent Enouf, Louise Lefrançois, Frédéric Lemoine, Christophe Malabat, Corinne Maufrais, Etienne Simon-Lorière, Maud Vanpeene, Sylvie Van der Werf ,Alexandra Ducancelle
EPI_ISL_2188437, EPI_ISL_2188438	Labo Analyses Med	National Reference Center for Viruses of Respiratory Infections, Institut Pasteur, Paris	Marion Barbet, Sylvie Behillil, Méline Bizard, Angela Brisebarre, Camille Capel, Vincent Enouf, Louise Lefrançois, Frédéric Lemoine, Christophe Malabat, Corinne Maufrais, Etienne Simon-Lorière, Maud Vanpeene, Sylvie Van der Werf ,M.-J. Blanc-Galiby
EPI_ISL_2202673, EPI_ISL_2202675, EPI_ISL_2202678	The Ohio State University Applied Microbiology Services Laboratory	The Ohio State University Applied Microbiology Services Laboratory	Seth A. Faith PhD
EPI_ISL_2214905	Centre Hospitalier Universitaire Clermont-Ferrand	CHU Clermont-Ferrand, service de virologie	Bisseux Maxime, Mirand Audrey, Combes Patricia, Henquell Cécile
EPI_ISL_2214914	EHPAD	CHU Clermont-Ferrand, service de virologie	Bisseux Maxime, Mirand Audrey, Combes Patricia, Henquell Cécile

EPI_ISL_2226217, EPI_ISL_2226218, EPI_ISL_2226219, EPI_ISL_2226220, EPI_ISL_2226221, EPI_ISL_2226222, EPI_ISL_2226223, EPI_ISL_2226225, EPI_ISL_2226226, EPI_ISL_2226227, EPI_ISL_2226228			
see above	Department of Internal Medicine, College of Medicine, Chosun University	Department of Internal Medicine, College of Medicine, Chosun University	Dong-Min Kim
EPI_ISL_2226264, EPI_ISL_2226325, EPI_ISL_2226329, EPI_ISL_2226336, EPI_ISL_2226339, EPI_ISL_2226340	BIO86 CHAUMONT	CHU Poitiers	Caroline MICHAUD, Maxime PICHON, Manon PRAT, Valentin BON-BARET, Birama N'DIAYE, Magali GARCIA, Agnes BEBY-DEFAUX, Nicolas LEVEQUE
EPI_ISL_2227286	Veterinary institute in Zvolen, Slovakia	Veterinary institute in Zvolen	Dirbáková Z., Sujová S., Mokryšová S., Tinák M., Mojžiš M.
EPI_ISL_2227287	Veterinary institute in Zvolen, Pod Drahami 918, 960 86 Zvolen Slovakia	Veterinary institute in Zvolen, Pod Drahami 918, 960 86 Zvolen Slovakia	Dirbáková Z., Sujová S., Mokryšová S., Tinák M., Mojžiš M.
EPI_ISL_2227288	Veterinary institute in Zvolen Slovakia	Veterinary institute in Zvolen Slovakia	Dirbáková Z., Sujová S., Mokryšová S., Tinák M., Mojžiš M.
EPI_ISL_2231152, EPI_ISL_2231153	Centro de Diagnostico COVID-19 UABC Tijuana	Andersen lab at Scripps Research	SEARCH Alliance San Diego with Idanya Rubi Serafin Higuera, Manuel Sanchez Alavez, Jorge Luis Jimenez Niebla, German Ibarra, Jonathan Vincent Baena, Oscar Efren Zazueta Fierro
EPI_ISL_2231154	Sharp HealthCare Laboratory	Andersen lab at Scripps Research	SEARCH Alliance San Diego with Aaron Harding, Jacquelyn Berumen, Cathy Woerle, Liam McGinnis, Art Mendoza, Omid Bakhtar
EPI_ISL_2231171, EPI_ISL_2231172	Centro de Diagnostico COVID-19 UABC Tijuana	Andersen lab at Scripps Research	SEARCH Alliance San Diego with Idanya Rubi Serafin Higuera, Manuel Sanchez Alavez, Jorge Luis Jimenez Niebla, German Ibarra, Jonathan Vincent Baena, Oscar Efren Zazueta Fierro
EPI_ISL_2231173	San Diego County Public Health Laboratory	Andersen lab at Scripps Research	SEARCH Alliance San Diego with Ashleigh Murphy, Jovan Shephard, Brett Austin
EPI_ISL_2231174	Centro de Diagnostico COVID-19 UABC Tijuana	Andersen lab at Scripps Research	SEARCH Alliance San Diego with Idanya Rubi Serafin Higuera, Manuel Sanchez Alavez, Jorge Luis Jimenez Niebla, German Ibarra, Jonathan Vincent Baena, Oscar Efren Zazueta Fierro
EPI_ISL_2231175, EPI_ISL_2231186, EPI_ISL_2231188, EPI_ISL_2231189	Sharp HealthCare Laboratory	Andersen lab at Scripps Research	SEARCH Alliance San Diego with Aaron Harding, Jacquelyn Berumen, Cathy Woerle, Liam McGinnis, Art Mendoza, Omid Bakhtar
EPI_ISL_2231190, EPI_ISL_2231191, EPI_ISL_2231192	Centro de Diagnostico COVID-19 UABC Tijuana	Andersen lab at Scripps Research	SEARCH Alliance San Diego with Idanya Rubi Serafin Higuera, Manuel Sanchez Alavez, Jorge Luis Jimenez Niebla, German Ibarra, Jonathan Vincent Baena, Oscar Efren Zazueta Fierro
EPI_ISL_2231193	San Diego County Public Health Laboratory	Andersen lab at Scripps Research	SEARCH Alliance San Diego with Ashleigh Murphy, Jovan Shephard, Brett Austin
EPI_ISL_2231194, EPI_ISL_2231196, EPI_ISL_2231197	Sharp HealthCare Laboratory	Andersen lab at Scripps Research	SEARCH Alliance San Diego with Aaron Harding, Jacquelyn Berumen, Cathy Woerle, Liam McGinnis, Art Mendoza, Omid Bakhtar
EPI_ISL_2231199, EPI_ISL_2231200	Centro de Diagnostico COVID-19 UABC Tijuana	Andersen lab at Scripps Research	SEARCH Alliance San Diego with Idanya Rubi Serafin Higuera, Manuel Sanchez Alavez, Jorge Luis Jimenez Niebla, German Ibarra, Jonathan Vincent Baena, Oscar Efren Zazueta Fierro
EPI_ISL_2231201, EPI_ISL_2231202, EPI_ISL_2231203, EPI_ISL_2231204, EPI_ISL_2231207, EPI_ISL_2231208, EPI_ISL_2231209, EPI_ISL_2231210, EPI_ISL_2231211, EPI_ISL_2231212, EPI_ISL_2231213, EPI_ISL_2231214, EPI_ISL_2231215, EPI_ISL_2231216, EPI_ISL_2231217, EPI_ISL_2231218, EPI_ISL_2231219, EPI_ISL_2231220, EPI_ISL_2231221, EPI_ISL_2231222, EPI_ISL_2231223, EPI_ISL_2231224, EPI_ISL_2231225, EPI_ISL_2231226, EPI_ISL_2231227, EPI_ISL_2231228, EPI_ISL_2231229, EPI_ISL_2231230, EPI_ISL_2231231, EPI_ISL_2231232, EPI_ISL_2231233, EPI_ISL_2231234, EPI_ISL_2231235, EPI_ISL_2231236, EPI_ISL_2231237, EPI_ISL_2231238, EPI_ISL_2231239, EPI_ISL_2231240, EPI_ISL_2231241, EPI_ISL_2231242, EPI_ISL_2231243, EPI_ISL_2231244, EPI_ISL_2231245, EPI_ISL_2231246, EPI_ISL_2231247, EPI_ISL_2231248, EPI_ISL_2231249, EPI_ISL_2231250, EPI_ISL_2231251, EPI_ISL_2231252, EPI_ISL_2231253, EPI_ISL_2231254, EPI_ISL_2231255, EPI_ISL_2231256, EPI_ISL_2231257, EPI_ISL_2231258, EPI_ISL_2231259, EPI_ISL_2231260, EPI_ISL_2231261, EPI_ISL_2231262, EPI_ISL_2231263, EPI_ISL_2231264, EPI_ISL_2231265, EPI_ISL_2231266, EPI_ISL_2231267, EPI_ISL_2231268, EPI_ISL_2231269			
see above	San Diego County Public Health Laboratory	Andersen lab at Scripps Research	SEARCH Alliance San Diego with Ashleigh Murphy, Jovan Shephard, Brett Austin
EPI_ISL_2231270, EPI_ISL_2231271, EPI_ISL_2231272, EPI_ISL_2231273, EPI_ISL_2231274, EPI_ISL_2231275, EPI_ISL_2231276, EPI_ISL_2231277, EPI_ISL_2231278, EPI_ISL_2231279, EPI_ISL_2231280, EPI_ISL_2231281, EPI_ISL_2231282, EPI_ISL_2231283, EPI_ISL_2231284, EPI_ISL_2231285, EPI_ISL_2231286, EPI_ISL_2231287, EPI_ISL_2231288, EPI_ISL_2231289, EPI_ISL_2231290, EPI_ISL_2231291, EPI_ISL_2231292, EPI_ISL_2231293, EPI_ISL_2231294, EPI_ISL_2231295, EPI_ISL_2231296, EPI_ISL_2231297, EPI_ISL_2231298, EPI_ISL_2231299, EPI_ISL_2231300, EPI_ISL_2231301, EPI_ISL_2231302, EPI_ISL_2231303, EPI_ISL_2231304, EPI_ISL_2231305, EPI_ISL_2231306, EPI_ISL_2231307, EPI_ISL_2231308, EPI_ISL_2231309, EPI_ISL_2231310, EPI_ISL_2231311, EPI_ISL_2231312, EPI_ISL_2231313, EPI_ISL_2231314, EPI_ISL_2231315, EPI_ISL_2231316, EPI_ISL_2231317			
see above	Sharp HealthCare Laboratory	Andersen lab at Scripps Research	SEARCH Alliance San Diego with Aaron Harding, Jacquelyn Berumen, Cathy Woerle, Liam McGinnis, Art Mendoza, Omid Bakhtar
EPI_ISL_2231321, EPI_ISL_2231322, EPI_ISL_2231323, EPI_ISL_2231324, EPI_ISL_2231325, EPI_ISL_2231326, EPI_ISL_2231327, EPI_ISL_2231328, EPI_ISL_2231329, EPI_ISL_2231330, EPI_ISL_2231331, EPI_ISL_2231332, EPI_ISL_2231333, EPI_ISL_2231334, EPI_ISL_2231335, EPI_ISL_2231336, EPI_ISL_2231337, EPI_ISL_2231338, EPI_ISL_2231339, EPI_ISL_2231340, EPI_ISL_2231341, EPI_ISL_2231342, EPI_ISL_2231343, EPI_ISL_2231344, EPI_ISL_2231345, EPI_ISL_2231346, EPI_ISL_2231347, EPI_ISL_2231348, EPI_ISL_2231349, EPI_ISL_2231350, EPI_ISL_2231351, EPI_ISL_2231352, EPI_ISL_2231353, EPI_ISL_2231354, EPI_ISL_2231355, EPI_ISL_2231356, EPI_ISL_2231357, EPI_ISL_2231358, EPI_ISL_2231359, EPI_ISL_2231360, EPI_ISL_2231361, EPI_ISL_2231362, EPI_ISL_2231363, EPI_ISL_2231364, EPI_ISL_2231365, EPI_ISL_2231366, EPI_ISL_2231367, EPI_ISL_2231368, EPI_ISL_2231369, EPI_ISL_2231370, EPI_ISL_2231371, EPI_ISL_2231372, EPI_ISL_2231373, EPI_ISL_2231374, EPI_ISL_2231375, EPI_ISL_2231376, EPI_ISL_2231377, EPI_ISL_2231378, EPI_ISL_2231379, EPI_ISL_2231380, EPI_ISL_2231381, EPI_ISL_2231382, EPI_ISL_2231383, EPI_ISL_2231384, EPI_ISL_2231385, EPI_ISL_2231386, EPI_ISL_2231387, EPI_ISL_2231388, EPI_ISL_2231389, EPI_ISL_2231390, EPI_ISL_2231391, EPI_ISL_2231392, EPI_ISL_2231393, EPI_ISL_2231394, EPI_ISL_2231395, EPI_ISL_2231396, EPI_ISL_2231397, EPI_ISL_2231398, EPI_ISL_2231399, EPI_ISL_2231400, EPI_ISL_2231401, EPI_ISL_2231402, EPI_ISL_2231403, EPI_ISL_2231404, EPI_ISL_2231405, EPI_ISL_2231406, EPI_ISL_2231407, EPI_ISL_2231408, EPI_ISL_2231409, EPI_ISL_2231410, EPI_ISL_2231411, EPI_ISL_2231412, EPI_ISL_2231413, EPI_ISL_2231414, EPI_ISL_2231415, EPI_ISL_2231416, EPI_ISL_2231417, EPI_ISL_2231418, EPI_ISL_2231419, EPI_ISL_2231420, EPI_ISL_2231421, EPI_ISL_2231422, EPI_ISL_2231423, EPI_ISL_2231424, EPI_ISL_2231425, EPI_ISL_2231426, EPI_ISL_2231427, EPI_ISL_2231428, EPI_ISL_2231429, EPI_ISL_2231430, EPI_ISL_2231431, EPI_ISL_2231432, EPI_ISL_2231433, EPI_ISL_2231434			
see above	Centro de Diagnostico COVID-19 UABC Tijuana	Andersen lab at Scripps Research	SEARCH Alliance San Diego with Idanya Rubi Serafin Higuera, Manuel Sanchez Alavez, Jorge Luis Jimenez Niebla, German Ibarra, Jonathan Vincent Baena, Oscar Efren Zazueta Fierro
EPI_ISL_2231436, EPI_ISL_2231437, EPI_ISL_2231438, EPI_ISL_2231439, EPI_ISL_2231440, EPI_ISL_2231441, EPI_ISL_2231443, EPI_ISL_2231444, EPI_ISL_2231445, EPI_ISL_2231446, EPI_ISL_2231447, EPI_ISL_2231448, EPI_ISL_2231449, EPI_ISL_2231450, EPI_ISL_2231451, EPI_ISL_2231452, EPI_ISL_2231453, EPI_ISL_2231454, EPI_ISL_2231455, EPI_ISL_2231456, EPI_ISL_2231457, EPI_ISL_2231458, EPI_ISL_2231459, EPI_ISL_2231460, EPI_ISL_2231461, EPI_ISL_2231462, EPI_ISL_2231463, EPI_ISL_2231464, EPI_ISL_2231465			
see above	San Diego County Public Health Laboratory	Andersen lab at Scripps Research	SEARCH Alliance San Diego with Ashleigh Murphy, Jovan Shephard, Brett Austin
EPI_ISL_2231467	Centro de Diagnostico COVID-19 UABC Tijuana	Andersen lab at Scripps Research	SEARCH Alliance San Diego with Idanya Rubi Serafin Higuera, Manuel Sanchez Alavez, Jorge Luis Jimenez Niebla, German Ibarra, Jonathan Vincent Baena, Oscar Efren Zazueta Fierro
EPI_ISL_2231468, EPI_ISL_2231469, EPI_ISL_2231470, EPI_ISL_2231471, EPI_ISL_2231473, EPI_ISL_2231474, EPI_ISL_2231477, EPI_ISL_2231480, EPI_ISL_2231481, EPI_ISL_2231482, EPI_ISL_2231483, EPI_ISL_2231484, EPI_ISL_2231487, EPI_ISL_2231488, EPI_ISL_2231489, EPI_ISL_2231490, EPI_ISL_2231491, EPI_ISL_2231492, EPI_ISL_2231493, EPI_ISL_2231495, EPI_ISL_2231496, EPI_ISL_2231497, EPI_ISL_2231500, EPI_ISL_2231501, EPI_ISL_2231504, EPI_ISL_2231505, EPI_ISL_2231506, EPI_ISL_2231507			
see above	San Diego County Public Health Laboratory	Andersen lab at Scripps Research	SEARCH Alliance San Diego with Ashleigh Murphy, Jovan Shephard, Brett Austin
EPI_ISL_2231508	Centro de Diagnostico COVID-19 UABC Tijuana	Andersen lab at Scripps Research	SEARCH Alliance San Diego with Idanya Rubi Serafin Higuera, Manuel Sanchez Alavez, Jorge Luis Jimenez Niebla, German Ibarra, Jonathan Vincent Baena, Oscar Efren Zazueta Fierro
EPI_ISL_2231510, EPI_ISL_2231511, EPI_ISL_2231512, EPI_ISL_2231513, EPI_ISL_2231514, EPI_ISL_2231518, EPI_ISL_2231519, EPI_ISL_2231520, EPI_ISL_2231521, EPI_ISL_2231522	San Diego County Public Health Laboratory	Andersen lab at Scripps Research	SEARCH Alliance San Diego with Ashleigh Murphy, Jovan Shephard, Brett Austin
EPI_ISL_2231528, EPI_ISL_2231529, EPI_ISL_2231531, EPI_ISL_2231532, EPI_ISL_2231533, EPI_ISL_2231534, EPI_ISL_2231535, EPI_ISL_2231536, EPI_ISL_2231537, EPI_ISL_2231538, EPI_ISL_2231539, EPI_ISL_2231540, EPI_ISL_2231541, EPI_ISL_2231542, EPI_ISL_2231543, EPI_ISL_2231544, EPI_ISL_2231545, EPI_ISL_2231546, EPI_ISL_2231547, EPI_ISL_2231548, EPI_ISL_2231549, EPI_ISL_2231550, EPI_ISL_2231551, EPI_ISL_2231552, EPI_ISL_2231553, EPI_ISL_2231554, EPI_ISL_2231555, EPI_ISL_2231556, EPI_ISL_2231557, EPI_ISL_2231558, EPI_ISL_2231559, EPI_ISL_2231560, EPI_ISL_2231561, EPI_ISL_2231562, EPI_ISL_2231563, EPI_ISL_2231564			
see above	Sharp HealthCare Laboratory	Andersen lab at Scripps Research	SEARCH Alliance San Diego with Aaron Harding, Jacquelyn Berumen, Cathy Woerle, Liam McGinnis, Art Mendoza, Omid Bakhtar
EPI_ISL_2231883, EPI_ISL_2231884, EPI_ISL_2231885, EPI_ISL_2231886, EPI_ISL_2231887, EPI_ISL_2231888, EPI_ISL_2231889, EPI_ISL_2231890	CQRC_QUALITY CONTROL CHEMICAL BIOLOGICAL RISK_AOOR Villa Sofia Cervello Palermo	CQRC_QUALITY CONTROL CHEMICAL BIOLOGICAL RISK_AOOR Villa Sofia Cervello Palermo	Di Gaudio, F., Contino, F., Brunacci, G.
EPI_ISL_2231891, EPI_ISL_2231892, EPI_ISL_2231893	CQRC_QUALITY CONTROL CHEMICAL BIOLOGICAL RISK_AOOR Villa Sofia Cervello Palermo	CQRC_QUALITY CONTROL CHEMICAL BIOLOGICAL RISK_AOOR Villa Sofia Cervello Palermo	Di Gaudio, F., Brunacci, G., Contino, F.
EPI_ISL_2231894	CQRC_QUALITY CONTROL CHEMICAL BIOLOGICAL	CQRC_QUALITY CONTROL CHEMICAL BIOLOGICAL	Di Gaudio, F., Contino, F., Brunacci, G.

	RISK_AOOR Villa Sofia Cervello Palermo	RISK_AOOR Villa Sofia Cervello Palermo	
EPI_ISL_2231899	CQRC_QUALITY CONTROL CHEMICAL BIOLOGICAL RISK_AOOR Villa Sofia Cervello Palermo	CQRC_QUALITY CONTROL CHEMICAL BIOLOGICAL RISK_AOOR Villa Sofia Cervello Palermo	Di Gaudio, F., Contino, F., Gasparro O., Rinaudo G., Brunacci, G.
EPI_ISL_2231900, EPI_ISL_2231901, EPI_ISL_2231902, EPI_ISL_2231903, EPI_ISL_2231904	CQRC_QUALITY CONTROL CHEMICAL BIOLOGICAL RISK_AOOR Villa Sofia Cervello Palermo	CQRC_QUALITY CONTROL CHEMICAL BIOLOGICAL RISK_AOOR Villa Sofia Cervello Palermo	Di Gaudio, F., Brunacci, G., Contino, F.
EPI_ISL_2240801	Laboratorio Biologia Molecolare Sars Cov2 - UOC Laboratorio Analisi - Servizio Medicina di Laboratorio, Ospedale "San Francesco" - ATS-ASSL Nuoro	Laboratorio specialistico UOC Ematologia - Ospedale "San Francesco" - ATS-ASSL Nuoro	Piras Giovanna, Asproni Rosanna,,Fiamma Maura, Monne Maria Itria, Palmas Angelo Domenico, Lo Maglio Iana, Mameli Giuseppe
EPI_ISL_2253214, EPI_ISL_2254130, EPI_ISL_2254496	Veterinary institute in Zvolen Slovakia	Veterinary institute in Zvolen Slovakia	Dirbáková Z., Sujová S., Mokryšová S., Tinák M., Mojžiš M
EPI_ISL_2258200	Laboratorio Biologia Molecolare Sars Cov2 - UOC Laboratorio Analisi - Servizio Medicina di Laboratorio, Ospedale "San Francesco" - ATS-ASSL Nuoro	Laboratorio specialistico UOC Ematologia - Ospedale "San Francesco" - ATS-ASSL Nuoro	Piras Giovanna, Asproni Rosanna,,Fiamma Maura, Monne Maria Itria, Palmas Angelo Domenico, Lo Maglio Iana, Mameli Giuseppe
EPI_ISL_2271905, EPI_ISL_2271910, EPI_ISL_2271911	Hospital General de México, Medicina Genómica	Hospital General de México, Medicina Genómica	Ana Laura Sánchez-Sandoval, María Eligia Juárez-Torres, Mariana Villalba-De la Peña, Siddhartha Ríos-Zaragoza, Guadalupe Mercedes Lucía Guerrero-Avendaño, Erika Anabel Alvarado-Silva, Eira Valeria Barrón-Palma
EPI_ISL_2272477, EPI_ISL_2272479, EPI_ISL_2272480, EPI_ISL_2272481, EPI_ISL_2272482, EPI_ISL_2272483, EPI_ISL_2272484, EPI_ISL_2272485, EPI_ISL_2272486, EPI_ISL_2272487, EPI_ISL_2272488, EPI_ISL_2272489, EPI_ISL_2272490, EPI_ISL_2272491, EPI_ISL_2272492, EPI_ISL_2272493, EPI_ISL_2272494, EPI_ISL_2272495, EPI_ISL_2272496			
see above	Indian Council of Medical Research-National Institute of Virology, Microbial Containment Complex	Indian Council of Medical Research-National Institute of Virology, Microbial Containment Complex	Pragya D Yadav
EPI_ISL_2279498	Institut National d'Hygiène (INH)	Unité Mixte Internationale TransVIHMI (UMI 233 IRD - U1175 INSERM - Université de Montpellier)IRD (Institut de recherche pour le développement)	Mounerou SALOU, Christelle BUTEL, Wembo A. HALATOKO,Issaka Maman, Abla A. KONOU, Amivi EHLAN, Adodo SADJI, Kokou TEGUENI,Sidonie A.M.KAGNISSODE, Akoélé SILIADIN, Alassane OURO-MEDELI, Messanh DOUFFAN,Déléma MABA,Sika DOSSIM, Améyo DORKENOO, Mireille PRINCE-DAVID,Anoumou DAGNRA,Laetitia SERRANO,Ahidjo AYOUBA,Eric DELAPORTE, Martine PEETERS
EPI_ISL_2283768, EPI_ISL_2283769, EPI_ISL_2283770, EPI_ISL_2283771, EPI_ISL_2283772, EPI_ISL_2283773, EPI_ISL_2283774, EPI_ISL_2283775, EPI_ISL_2283776, EPI_ISL_2283777, EPI_ISL_2283778, EPI_ISL_2283779, EPI_ISL_2283780, EPI_ISL_2283781, EPI_ISL_2283782, EPI_ISL_2283783, EPI_ISL_2283784, EPI_ISL_2283785, EPI_ISL_2283786, EPI_ISL_2283787, EPI_ISL_2283788, EPI_ISL_2283789, EPI_ISL_2283790, EPI_ISL_2283791, EPI_ISL_2283792, EPI_ISL_2283793, EPI_ISL_2283794, EPI_ISL_2283795, EPI_ISL_2283796, EPI_ISL_2283797, EPI_ISL_2283798, EPI_ISL_2283799, EPI_ISL_2283800, EPI_ISL_2283801, EPI_ISL_2283802, EPI_ISL_2283803, EPI_ISL_2283804, EPI_ISL_2283807, EPI_ISL_2283808, EPI_ISL_2283809, EPI_ISL_2283810			
see above	Genome Analysis Center, Yamanashi Central Hospital	Genome Analysis Center, Yamanashi Central Hospital	Yosuke Hirotsu
EPI_ISL_2284874	Siti Khodijah Hospital	Institute of Tropical Disease, Universitas Airlangga	Kazufumi Shimizu, Krisnoadi Rahardjo, Aldise M Nastri, Jezzy R Dewantari, Rima R Prasetya, Muhammad Hamdan, Gatot Soegiarto, Laksmi Wulandari, Resti Yudhawati, Yasuko Mori, Soetjipto, Maria I Lusida
EPI_ISL_2285106, EPI_ISL_2285150	Microbiology Department. Complexo Hospitalario Universitario de Vigo	Microbiology Department. Complexo Hospitalario Universitario de Vigo	Alfaya N, Alonso I, Alvarez M, Cabrera JJ, Carballo R, Cores O, Cortizo S, del-Campo V, Martinez L, Mediero G, Perez S, Potel C, Regueiro B, Rey S, Vasallo FJ
EPI_ISL_2285723, EPI_ISL_2285732	Department of Pathology, National Institute of Infectious Diseases	Department of Pathology, National Institute of Infectious Diseases	Harutaka Katano, Takayuki Kanno, Tadaki Suzuki
EPI_ISL_2293282	Labo Analyses Med	National Reference Center for Viruses of Respiratory Infections, Institut Pasteur, Paris	Marion Barbet, Sylvie Behillil, Méline Bizard, Angela Brisebarre, Camille Capel, Vincent Enouf, Louise Lefrançois, Frédéric Lemoine, Christophe Malabat, Corinne Maufrais, Etienne Simon-Lorière, Maud Vanpeene, Sylvie Van der Werf ,Caroline Malderet
EPI_ISL_2293421, EPI_ISL_2293422	Research	National Reference Center for Viruses of Respiratory Infections, Institut Pasteur, Paris	Marion Barbet, Sylvie Behillil, Méline Bizard, Angela Brisebarre, Camille Capel, Vincent Enouf, Louise Lefrançois, Frédéric Lemoine, Christophe Malabat, Corinne Maufrais, Etienne Simon-Lorière, Maud Vanpeene, Sylvie Van der Werf ,Marianne Leruez-Ville
EPI_ISL_2296063	LESP Hidalgo	Instituto de Diagnostico y Referencia Epidemiologicos (INDRE)	Claudia Wong-Arambula, Abril Rodriguez-Maldonado, Vanessa Rivero-Arredondo, Ariadna Medina-Benitez, Joaquin Quiroz-Mercado, Sergio Rangel-Guerrero, Natividad Cruz-Ortiz, Tatiana Nunez-Garcia, Gisela Barrera-Badillo, Lucia Hernandez-Rivas, Irma Lopez-Martinez, Ernesto Ramirez-Gonzalez.
EPI_ISL_2304298, EPI_ISL_2304299, EPI_ISL_2304300, EPI_ISL_2304301, EPI_ISL_2304302, EPI_ISL_2304303, EPI_ISL_2307325, EPI_ISL_2307327, EPI_ISL_2307328, EPI_ISL_2307331, EPI_ISL_2307332, EPI_ISL_2307333, EPI_ISL_2307334, EPI_ISL_2307335, EPI_ISL_2307336, EPI_ISL_2307337, EPI_ISL_2307349			
see above	Genome Analysis Center, Yamanashi Central Hospital	Genome Analysis Center, Yamanashi Central Hospital	Yosuke Hirotsu
EPI_ISL_2308271, EPI_ISL_2308272, EPI_ISL_2308273, EPI_ISL_2308274, EPI_ISL_2308275, EPI_ISL_2308276	Indian Council of Medical Research-National Institute of Virology, Microbial Containment Complex	Indian Council of Medical Research-National Institute of Virology, Microbial Containment Complex	Pragya D Yadav
EPI_ISL_2321226, EPI_ISL_2321230, EPI_ISL_2322453, EPI_ISL_2322454	Genome Analysis Center, Yamanashi Central Hospital	Genome Analysis Center, Yamanashi Central Hospital	Yosuke Hirotsu
EPI_ISL_2322758, EPI_ISL_2322761	Collection of WHO National Influenza Center, N.F. Gamaleya National Research Center for Epidemiology and Microbiology of the Ministry of Health of the Russian Federation	Laboratory of genome analysis, N.F. Gamaleya National Research Center for Epidemiology and Microbiology of the Ministry of Health of the Russian Federation	Olga Voronina, Ekaterina Aksenova, Natalia Ryzhova, Marina Kunda, Kirill Krasnoslobodtsev, Elena Burtseva, Denis Logunov
EPI_ISL_2324194	Austrian Agency for Health and Food Safety (AGES)	Bergthaler laboratory, CeMM Research Center for Molecular Medicine of the Austrian Academy of Sciences	Lukas Endler, Anna Schedl, Fabian Amman, Petr Triska, Thomas Penz, Benedikt Agerer, Maelle Le Moing, Michael Schuster, Bekir Erguner, Jan Laine, Martin Senekowitsch, Christoph Bock, Andreas Bergthaler
EPI_ISL_2324258, EPI_ISL_2324259, EPI_ISL_2324260, EPI_ISL_2324261	Institut für Virologie am Department für Hygiene, Mikrobiologie und Public Health	Bergthaler laboratory, CeMM Research Center for Molecular Medicine of the Austrian Academy of Sciences	Lukas Endler, Anna Schedl, Fabian Amman, Petr Triska, Thomas Penz, Benedikt Agerer, Maelle Le Moing, Michael Schuster, Bekir Erguner, Jan Laine, Martin Senekowitsch, Christoph Bock, Andreas Bergthaler
EPI_ISL_2339875, EPI_ISL_2339903, EPI_ISL_2339904	Centro de Estudio de Enfermedades Autoinmunes (CREA), Universidad del Rosario, Bogota, Colombia	Centro de Investigaciones en Microbiología y Biotecnología-UR (CIMBIUR), Facultad de Ciencias Naturales, Universidad del Rosario, Bogotá, Colombia	Juan-Manuel Anaya, Gustavo Salguero, Juan Esteban Gallo, Carolina Ramírez-Santana, Sergio Castañeda, Nathalia Ballesteros, Marina Muñoz, Luz H. Patiño, Juan David Ramírez
EPI_ISL_2342585, EPI_ISL_2342586, EPI_ISL_2342587, EPI_ISL_2342588, EPI_ISL_2342589, EPI_ISL_2342590, EPI_ISL_2342591, EPI_ISL_2342592, EPI_ISL_2342593, EPI_ISL_2342594, EPI_ISL_2342595, EPI_ISL_2342596, EPI_ISL_2342597, EPI_ISL_2342598, EPI_ISL_2342599, EPI_ISL_2342600			
see above	Malaysia Genome Institute	Malaysia Genome Institute	Mohd Noor Mat Isa, Irni Suhayu Sapian, Yusuf Muhammad Noor, Nurhezreen Md Iqbal, Mohd Faizal Abu Bakar, Enizza Kasim, Shamsidar Sopie, Siti Noraini Othman, Azrin Ahmad, Nor Azfa Johari.
EPI_ISL_2343010	LESP Hidalgo	Instituto de Diagnostico y Referencia Epidemiologicos (INDRE)	Claudia Wong-Arambula, Abril Rodriguez-Maldonado, Vanessa Rivero-Arredondo, Ariadna Medina-Benitez, Joaquin Quiroz-Mercado, Sergio Rangel-Guerrero, Natividad Cruz-Ortiz, Tatiana Nunez-Garcia, Gisela Barrera-Badillo, Lucia Hernandez-Rivas, Irma Lopez-Martinez, Ernesto Ramirez-Gonzalez.
EPI_ISL_2350786	Translational Health Science and Technology Institute -ESIC medical college and hospital, Faridabad	THSTI Bioassay laboratory	Naseem Ahmed Khan, Saurabh Kumar, Neha Jha, Anish Maitra, Partha Chattopadhyay, Anil Kumar Pandey, Guruprasad R Medigeshi, Rajesh Pandey
EPI_ISL_2364489, EPI_ISL_2364490, EPI_ISL_2364500, EPI_ISL_2364501, EPI_ISL_2364502, EPI_ISL_2364503, EPI_ISL_2364509	Genome Analysis Center, Yamanashi Central Hospital	Genome Analysis Center, Yamanashi Central Hospital	Yosuke Hirotsu
EPI_ISL_2364568	RS Medirossa Cikarang	Eijkman Institute for Molecular Biology, National Agency for Research and Innovation	Willy Agustine, Hidayat Trimarsanto, Lydia V. Panggalo, Iskandar Adnan, Sukma Oktavianthi, Lidwina Priliani, Edison Johar, Frilasita A Yudhaputri, Muhammad Rezki Rasyak, Safarina G Malik, Khin Saw Myint, Amin Soebandrio
EPI_ISL_2364569, EPI_ISL_2364570, EPI_ISL_2364571	RS Sentra Medika Cikarang	Eijkman Institute for Molecular Biology, National Agency for Research and Innovation	Lydia V. Panggalo, Iskandar Adnan, Sukma Oktavianthi, Lidwina Priliani, Edison Johar, Frilasita A Yudhaputri, Muhammad Rezki Rasyak, Willy Agustine, Hidayat Trimarsanto, Safarina G Malik, Khin Saw Myint, Amin Soebandrio

EPI_ISL_2364572	RS Sentra Medika Cikarang	Eijkman Institute for Molecular Biology, National Agency for Research and Innovation	Iskandar Adnan, Sukma Oktavianthi, Lidwina Priliani, Edison Johar, Frilasita A Yudhaputri, Muhammad Rezki Rasyak, Willy Agustine, Hidayat Trimarsanto, Lydia V. Panggalo, Safarina G Malik, Khin Saw Myint, Amin Soebandrio
EPI_ISL_2364573	RS Bhakti Husada Cikarang	Eijkman Institute for Molecular Biology, National Agency for Research and Innovation	Iskandar Adnan, Sukma Oktavianthi, Lidwina Priliani, Edison Johar, Frilasita A Yudhaputri, Muhammad Rezki Rasyak, Willy Agustine, Hidayat Trimarsanto, Lydia V. Panggalo, Safarina G Malik, Khin Saw Myint, Amin Soebandrio
EPI_ISL_2364574	RS Bhakti Husada Cikarang	Eijkman Institute for Molecular Biology, National Agency for Research and Innovation	Sukma Oktavianthi, Lidwina Priliani, Edison Johar, Frilasita A Yudhaputri, Muhammad Rezki Rasyak, Willy Agustine, Hidayat Trimarsanto, Lydia V. Panggalo, Iskandar Adnan, Safarina G Malik, Khin Saw Myint, Amin Soebandrio
EPI_ISL_2364575	RS Masmitra	Eijkman Institute for Molecular Biology, National Agency for Research and Innovation	Iskandar Adnan, Sukma Oktavianthi, Lidwina Priliani, Edison Johar, Frilasita A Yudhaputri, Muhammad Rezki Rasyak, Willy Agustine, Hidayat Trimarsanto, Lydia V. Panggalo, Safarina G Malik, Khin Saw Myint, Amin Soebandrio
EPI_ISL_2364576	RS Harapan Bunda	Eijkman Institute for Molecular Biology, National Agency for Research and Innovation	Hidayat Trimarsanto, Lydia V. Panggalo, Iskandar Adnan, Sukma Oktavianthi, Lidwina Priliani, Edison Johar, Frilasita A Yudhaputri, Muhammad Rezki Rasyak, Willy Agustine, Safarina G Malik, Khin Saw Myint, Amin Soebandrio
EPI_ISL_2364577	RS Permata Bekasi	Eijkman Institute for Molecular Biology, National Agency for Research and Innovation	Sukma Oktavianthi, Lidwina Priliani, Edison Johar, Frilasita A Yudhaputri, Muhammad Rezki Rasyak, Willy Agustine, Hidayat Trimarsanto, Lydia V. Panggalo, Iskandar Adnan, Safarina G Malik, Khin Saw Myint, Amin Soebandrio
EPI_ISL_2364578	RS Asy-Syifaa Leuwiliang	Eijkman Institute for Molecular Biology, National Agency for Research and Innovation	Sukma Oktavianthi, Lidwina Priliani, Edison Johar, Frilasita A Yudhaputri, Muhammad Rezki Rasyak, Willy Agustine, Hidayat Trimarsanto, Lydia V. Panggalo, Iskandar Adnan, Safarina G Malik, Khin Saw Myint, Amin Soebandrio
EPI_ISL_2364579	RS Mitra Keluarga Cibubur	Eijkman Institute for Molecular Biology, National Agency for Research and Innovation	Lidwina Priliani, Edison Johar, Frilasita A Yudhaputri, Muhammad Rezki Rasyak, Willy Agustine, Hidayat Trimarsanto, Lydia V. Panggalo, Iskandar Adnan, Sukma Oktavianthi, Safarina G Malik, Khin Saw Myint, Amin Soebandrio
EPI_ISL_2364580	RSUD Cileungsi	Eijkman Institute for Molecular Biology, National Agency for Research and Innovation	Edison Johar, Frilasita A Yudhaputri, Muhammad Rezki Rasyak, Willy Agustine, Hidayat Trimarsanto, Lydia V. Panggalo, Iskandar Adnan, Sukma Oktavianthi, Lidwina Priliani, Safarina G Malik, Khin Saw Myint, Amin Soebandrio
EPI_ISL_2364581	RS Sentra Medika Cikarang	Eijkman Institute for Molecular Biology, National Agency for Research and Innovation	Iskandar Adnan, Sukma Oktavianthi, Lidwina Priliani, Edison Johar, Frilasita A Yudhaputri, Muhammad Rezki Rasyak, Willy Agustine, Hidayat Trimarsanto, Lydia V. Panggalo, Safarina G Malik, Khin Saw Myint, Amin Soebandrio
EPI_ISL_2364582	RS Sentra Medika Cikarang	Eijkman Institute for Molecular Biology, National Agency for Research and Innovation	Frilasita A Yudhaputri, Muhammad Rezki Rasyak, Willy Agustine, Hidayat Trimarsanto, Lydia V. Panggalo, Iskandar Adnan, Sukma Oktavianthi, Lidwina Priliani, Edison Johar, Safarina G Malik, Khin Saw Myint, Amin Soebandrio
EPI_ISL_2364583	RSJ Dr. Soeharto Heerdjan	Eijkman Institute for Molecular Biology, National Agency for Research and Innovation	Muhammad Rezki Rasyak, Willy Agustine, Hidayat Trimarsanto, Lydia V. Panggalo, Iskandar Adnan, Sukma Oktavianthi, Lidwina Priliani, Edison Johar, Frilasita A Yudhaputri, Safarina G Malik, Khin Saw Myint, Amin Soebandrio
EPI_ISL_2364584	Ciputra Hospital Citra Raya Tangerang	Eijkman Institute for Molecular Biology, National Agency for Research and Innovation	Edison Johar, Frilasita A Yudhaputri, Muhammad Rezki Rasyak, Willy Agustine, Hidayat Trimarsanto, Lydia V. Panggalo, Iskandar Adnan, Sukma Oktavianthi, Lidwina Priliani, Safarina G Malik, Khin Saw Myint, Amin Soebandrio
EPI_ISL_2364585	RSUD Cileungsi	Eijkman Institute for Molecular Biology, National Agency for Research and Innovation	Frilasita A Yudhaputri, Muhammad Rezki Rasyak, Willy Agustine, Hidayat Trimarsanto, Lydia V. Panggalo, Iskandar Adnan, Sukma Oktavianthi, Lidwina Priliani, Edison Johar, Safarina G Malik, Khin Saw Myint, Amin Soebandrio
EPI_ISL_2364586	RS Graha Juanda	Eijkman Institute for Molecular Biology, National Agency for Research and Innovation	Muhammad Rezki Rasyak, Willy Agustine, Hidayat Trimarsanto, Lydia V. Panggalo, Iskandar Adnan, Sukma Oktavianthi, Lidwina Priliani, Edison Johar, Frilasita A Yudhaputri, Safarina G Malik, Khin Saw Myint, Amin Soebandrio
EPI_ISL_2364588	RS Mitra Keluarga Gading Serpong	Eijkman Institute for Molecular Biology, National Agency for Research and Innovation	Hidayat Trimarsanto, Lydia V. Panggalo, Iskandar Adnan, Sukma Oktavianthi, Lidwina Priliani, Edison Johar, Frilasita A Yudhaputri, Muhammad Rezki Rasyak, Willy Agustine, Safarina G Malik, Khin Saw Myint, Amin Soebandrio
EPI_ISL_2364590	RS Bhakti Husada Cikarang	Eijkman Institute for Molecular Biology, National Agency for Research and Innovation	Lidwina Priliani, Edison Johar, Frilasita A Yudhaputri, Muhammad Rezki Rasyak, Willy Agustine, Hidayat Trimarsanto, Lydia V. Panggalo, Iskandar Adnan, Sukma Oktavianthi, Safarina G Malik, Khin Saw Myint, Amin Soebandrio
EPI_ISL_2364591	RS Graha Juanda	Eijkman Institute for Molecular Biology, National Agency for Research and Innovation	Edison Johar, Frilasita A Yudhaputri, Muhammad Rezki Rasyak, Willy Agustine, Hidayat Trimarsanto, Lydia V. Panggalo, Iskandar Adnan, Sukma Oktavianthi, Lidwina Priliani, Safarina G Malik, Khin Saw Myint, Amin Soebandrio
EPI_ISL_2364592	RS Mitra Keluarga Gading Serpong	Eijkman Institute for Molecular Biology, National Agency for Research and Innovation	Frilasita A Yudhaputri, Muhammad Rezki Rasyak, Willy Agustine, Hidayat Trimarsanto, Lydia V. Panggalo, Iskandar Adnan, Sukma Oktavianthi, Lidwina Priliani, Edison Johar, Safarina G Malik, Khin Saw Myint, Amin Soebandrio
EPI_ISL_2364594	RSUD Cileungsi	Eijkman Institute for Molecular Biology, National Agency for Research and Innovation	Muhammad Rezki Rasyak, Willy Agustine, Hidayat Trimarsanto, Lydia V. Panggalo, Iskandar Adnan, Sukma Oktavianthi, Lidwina Priliani, Edison Johar, Frilasita A Yudhaputri, Safarina G Malik, Khin Saw Myint, Amin Soebandrio
EPI_ISL_2364596	RS Mitra Keluarga Kelapa Gading	Eijkman Institute for Molecular Biology, National Agency for Research and Innovation	Hidayat Trimarsanto, Lydia V. Panggalo, Iskandar Adnan, Sukma Oktavianthi, Lidwina Priliani, Edison Johar, Frilasita A Yudhaputri, Muhammad Rezki Rasyak, Willy Agustine, Safarina G Malik, Khin Saw Myint, Amin Soebandrio
EPI_ISL_2364597	RS Permata Cibubur	Eijkman Institute for Molecular Biology, National Agency for Research and Innovation	Edison Johar, Frilasita A Yudhaputri, Muhammad Rezki Rasyak, Willy Agustine, Hidayat Trimarsanto, Lydia V. Panggalo, Iskandar Adnan, Sukma Oktavianthi, Lidwina Priliani, Safarina G Malik, Khin Saw Myint, Amin Soebandrio
EPI_ISL_2364598	RSIA Bunda Sejahtera	Eijkman Institute for Molecular Biology, National Agency for Research and Innovation	Muhammad Rezki Rasyak, Willy Agustine, Hidayat Trimarsanto, Lydia V. Panggalo, Iskandar Adnan, Sukma Oktavianthi, Lidwina Priliani, Edison Johar, Frilasita A Yudhaputri, Safarina G Malik, Khin Saw Myint, Amin Soebandrio
EPI_ISL_2364600	RS Mitra Keluarga Cibubur	Eijkman Institute for Molecular Biology, National Agency for Research and Innovation	Willy Agustine, Hidayat Trimarsanto, Lydia V. Panggalo, Iskandar Adnan, Sukma Oktavianthi, Lidwina Priliani, Edison Johar, Frilasita A Yudhaputri, Muhammad Rezki Rasyak, Safarina G Malik, Khin Saw Myint, Amin Soebandrio
EPI_ISL_2364601	RS Mary Cileungsi Hijau	Eijkman Institute for Molecular Biology, National Agency for Research and Innovation	Hidayat Trimarsanto, Lydia V. Panggalo, Iskandar Adnan, Sukma Oktavianthi, Lidwina Priliani, Edison Johar, Frilasita A Yudhaputri, Muhammad Rezki Rasyak, Willy Agustine, Safarina G Malik, Khin Saw Myint, Amin Soebandrio
EPI_ISL_2364602	RS Sumber Waras	Eijkman Institute for Molecular Biology, National Agency for Research and Innovation	Iskandar Adnan, Sukma Oktavianthi, Lidwina Priliani, Edison Johar, Frilasita A Yudhaputri, Muhammad Rezki Rasyak, Willy Agustine, Hidayat Trimarsanto, Lydia V. Panggalo, Safarina G Malik, Khin Saw Myint, Amin Soebandrio
EPI_ISL_2364603	RS Mitra Keluarga Gading Serpong	Eijkman Institute for Molecular Biology, National Agency for Research and Innovation	Sukma Oktavianthi, Lidwina Priliani, Edison Johar, Frilasita A Yudhaputri, Muhammad Rezki Rasyak, Willy Agustine, Hidayat Trimarsanto, Lydia V. Panggalo, Iskandar Adnan, Safarina G Malik, Khin Saw Myint, Amin Soebandrio
EPI_ISL_2364605	RS Islam Sari Asih Ar-Rahmah	Eijkman Institute for Molecular Biology, National Agency for Research and Innovation	Willy Agustine, Hidayat Trimarsanto, Lydia V. Panggalo, Iskandar Adnan, Sukma Oktavianthi, Lidwina Priliani, Edison Johar, Frilasita A Yudhaputri, Muhammad Rezki Rasyak, Safarina G Malik, Khin Saw Myint, Amin Soebandrio
EPI_ISL_2364607	RS Medika Dramaga	Eijkman Institute for Molecular Biology, National Agency for Research and Innovation	Lydia V. Panggalo, Iskandar Adnan, Sukma Oktavianthi, Lidwina Priliani, Edison Johar, Frilasita A Yudhaputri, Muhammad Rezki Rasyak, Willy Agustine, Hidayat Trimarsanto, Safarina G Malik, Khin Saw Myint, Amin Soebandrio
EPI_ISL_2364608	RS Harapan Bunda	Eijkman Institute for Molecular Biology, National Agency for Research and Innovation	Iskandar Adnan, Sukma Oktavianthi, Lidwina Priliani, Edison Johar, Frilasita A Yudhaputri, Muhammad Rezki Rasyak, Willy Agustine, Hidayat Trimarsanto, Lydia V. Panggalo, Safarina G Malik, Khin Saw Myint, Amin Soebandrio
EPI_ISL_2364609	RS Harapan Bunda	Eijkman Institute for Molecular Biology, National Agency for Research and Innovation	Frilasita A Yudhaputri, Muhammad Rezki Rasyak, Willy Agustine, Hidayat Trimarsanto, Lydia V. Panggalo, Iskandar Adnan, Sukma Oktavianthi, Lidwina Priliani, Edison Johar, Safarina G Malik, Khin Saw Myint, Amin Soebandrio
EPI_ISL_2364610	RS Mitra Keluarga Kalideres	Eijkman Institute for Molecular Biology, National Agency for Research and Innovation	Muhammad Rezki Rasyak, Willy Agustine, Hidayat Trimarsanto, Lydia V. Panggalo, Iskandar Adnan, Sukma Oktavianthi, Lidwina Priliani, Edison Johar, Frilasita A Yudhaputri, Safarina G Malik, Khin Saw Myint, Amin Soebandrio
EPI_ISL_2364611	RSUD Cileungsi	Eijkman Institute for Molecular Biology, National Agency for Research and Innovation	Hidayat Trimarsanto, Lydia V. Panggalo, Iskandar Adnan, Sukma Oktavianthi, Lidwina Priliani, Edison Johar, Frilasita A Yudhaputri, Muhammad Rezki Rasyak, Willy Agustine, Safarina G Malik, Khin Saw Myint, Amin Soebandrio
EPI_ISL_2364612	RSUD Cileungsi	Eijkman Institute for Molecular Biology, National Agency for Research and Innovation	Sukma Oktavianthi, Lidwina Priliani, Edison Johar, Frilasita A Yudhaputri, Muhammad Rezki Rasyak, Willy Agustine, Hidayat Trimarsanto, Lydia V. Panggalo, Iskandar Adnan, Safarina G Malik, Khin Saw Myint, Amin Soebandrio
EPI_ISL_2364613	RSUD Cileungsi	Eijkman Institute for Molecular Biology, National Agency for Research and Innovation	Frilasita A Yudhaputri, Muhammad Rezki Rasyak, Willy Agustine, Hidayat Trimarsanto, Lydia V. Panggalo, Iskandar Adnan, Sukma Oktavianthi, Lidwina Priliani, Edison Johar, Safarina G Malik, Khin Saw Myint, Amin Soebandrio

EPI_ISL_2364615	RS Sentra Medika Cibinong	Eijkman Institute for Molecular Biology, National Agency for Research and Innovation	Lidwina Priliani, Edison Johar, Frilasita A Yudhaputri, Muhammad Rezki Rasyak, Willy Agustine, Hidayat Trimarsanto, Lydia V. Panggalo, Iskandar Adnan, Sukma Oktavianthi, Safarina G Malik, Khin Saw Myint, Amin Soebandrio
EPI_ISL_2364616	RS Mitra Keluarga Gading Serpong	Eijkman Institute for Molecular Biology, National Agency for Research and Innovation	Muhammad Rezki Rasyak, Willy Agustine, Hidayat Trimarsanto, Lydia V. Panggalo, Iskandar Adnan, Sukma Oktavianthi, Lidwina Priliani, Edison Johar, Frilasita A Yudhaputri, Safarina G Malik, Khin Saw Myint, Amin Soebandrio
EPI_ISL_2364617	RS Mitra Keluarga Gading Serpong	Eijkman Institute for Molecular Biology, National Agency for Research and Innovation	Hidayat Trimarsanto, Lydia V. Panggalo, Iskandar Adnan, Sukma Oktavianthi, Lidwina Priliani, Edison Johar, Frilasita A Yudhaputri, Muhammad Rezki Rasyak, Willy Agustine, Safarina G Malik, Khin Saw Myint, Amin Soebandrio
EPI_ISL_2364618	RS Mitra Keluarga Kelapa Gading	Eijkman Institute for Molecular Biology, National Agency for Research and Innovation	Lydia V. Panggalo, Iskandar Adnan, Sukma Oktavianthi, Lidwina Priliani, Edison Johar, Frilasita A Yudhaputri, Muhammad Rezki Rasyak, Willy Agustine, Hidayat Trimarsanto, Safarina G Malik, Khin Saw Myint, Amin Soebandrio
EPI_ISL_2364619	RS Mitra Keluarga Kelapa Gading	Eijkman Institute for Molecular Biology, National Agency for Research and Innovation	Iskandar Adnan, Sukma Oktavianthi, Lidwina Priliani, Edison Johar, Frilasita A Yudhaputri, Muhammad Rezki Rasyak, Willy Agustine, Hidayat Trimarsanto, Lydia V. Panggalo, Safarina G Malik, Khin Saw Myint, Amin Soebandrio
EPI_ISL_2364621	RS Sentra Medika Cikarang	Eijkman Institute for Molecular Biology, National Agency for Research and Innovation	Sukma Oktavianthi, Lidwina Priliani, Edison Johar, Frilasita A Yudhaputri, Muhammad Rezki Rasyak, Willy Agustine, Hidayat Trimarsanto, Lydia V. Panggalo, Iskandar Adnan, Safarina G Malik, Khin Saw Myint, Amin Soebandrio
EPI_ISL_2364624	RS Sentra Medika Cikarang	Eijkman Institute for Molecular Biology, National Agency for Research and Innovation	Lidwina Priliani, Edison Johar, Frilasita A Yudhaputri, Muhammad Rezki Rasyak, Willy Agustine, Hidayat Trimarsanto, Lydia V. Panggalo, Iskandar Adnan, Sukma Oktavianthi, Safarina G Malik, Khin Saw Myint, Amin Soebandrio
EPI_ISL_2364626	RS Sentra Medika Cikarang	Eijkman Institute for Molecular Biology, National Agency for Research and Innovation	Edison Johar, Frilasita A Yudhaputri, Muhammad Rezki Rasyak, Willy Agustine, Hidayat Trimarsanto, Lydia V. Panggalo, Iskandar Adnan, Sukma Oktavianthi, Lidwina Priliani, Safarina G Malik, Khin Saw Myint, Amin Soebandrio
EPI_ISL_2364629	RS Bhakti Husada Cikarang	Eijkman Institute for Molecular Biology, National Agency for Research and Innovation	Frilasita A Yudhaputri, Muhammad Rezki Rasyak, Willy Agustine, Hidayat Trimarsanto, Lydia V. Panggalo, Iskandar Adnan, Sukma Oktavianthi, Lidwina Priliani, Edison Johar, Safarina G Malik, Khin Saw Myint, Amin Soebandrio
EPI_ISL_2364632	RS Mitra Keluarga Bekasi Barat	Eijkman Institute for Molecular Biology, National Agency for Research and Innovation	Edison Johar, Frilasita A Yudhaputri, Muhammad Rezki Rasyak, Willy Agustine, Hidayat Trimarsanto, Lydia V. Panggalo, Iskandar Adnan, Sukma Oktavianthi, Lidwina Priliani, Safarina G Malik, Khin Saw Myint, Amin Soebandrio
EPI_ISL_2364635	RS Harapan Bunda	Eijkman Institute for Molecular Biology, National Agency for Research and Innovation	Iskandar Adnan, Sukma Oktavianthi, Lidwina Priliani, Edison Johar, Frilasita A Yudhaputri, Muhammad Rezki Rasyak, Willy Agustine, Hidayat Trimarsanto, Lydia V. Panggalo, Safarina G Malik, Khin Saw Myint, Amin Soebandrio
EPI_ISL_2364638	RS Mary Cileungsi Hijau	Eijkman Institute for Molecular Biology, National Agency for Research and Innovation	Willy Agustine, Hidayat Trimarsanto, Lydia V. Panggalo, Iskandar Adnan, Sukma Oktavianthi, Lidwina Priliani, Edison Johar, Frilasita A Yudhaputri, Muhammad Rezki Rasyak, Safarina G Malik, Khin Saw Myint, Amin Soebandrio
EPI_ISL_2364641	RS Mary Cileungsi Hijau	Eijkman Institute for Molecular Biology, National Agency for Research and Innovation	Frilasita A Yudhaputri, Muhammad Rezki Rasyak, Willy Agustine, Hidayat Trimarsanto, Lydia V. Panggalo, Iskandar Adnan, Sukma Oktavianthi, Lidwina Priliani, Edison Johar, Safarina G Malik, Khin Saw Myint, Amin Soebandrio
EPI_ISL_2364644	RSUD Cileungsi	Eijkman Institute for Molecular Biology, National Agency for Research and Innovation	Sukma Oktavianthi, Lidwina Priliani, Edison Johar, Frilasita A Yudhaputri, Muhammad Rezki Rasyak, Willy Agustine, Hidayat Trimarsanto, Lydia V. Panggalo, Iskandar Adnan, Safarina G Malik, Khin Saw Myint, Amin Soebandrio
EPI_ISL_2364646	RS Permata Cibubur	Eijkman Institute for Molecular Biology, National Agency for Research and Innovation	Lidwina Priliani, Edison Johar, Frilasita A Yudhaputri, Muhammad Rezki Rasyak, Willy Agustine, Hidayat Trimarsanto, Lydia V. Panggalo, Iskandar Adnan, Sukma Oktavianthi, Safarina G Malik, Khin Saw Myint, Amin Soebandrio
EPI_ISL_2364650	RS Permata Cibubur	Eijkman Institute for Molecular Biology, National Agency for Research and Innovation	Edison Johar, Frilasita A Yudhaputri, Muhammad Rezki Rasyak, Willy Agustine, Hidayat Trimarsanto, Lydia V. Panggalo, Iskandar Adnan, Sukma Oktavianthi, Lidwina Priliani, Safarina G Malik, Khin Saw Myint, Amin Soebandrio
EPI_ISL_2364653	RSU HGA Depok	Eijkman Institute for Molecular Biology, National Agency for Research and Innovation	Willy Agustine, Hidayat Trimarsanto, Lydia V. Panggalo, Iskandar Adnan, Sukma Oktavianthi, Lidwina Priliani, Edison Johar, Frilasita A Yudhaputri, Muhammad Rezki Rasyak, Safarina G Malik, Khin Saw Myint, Amin Soebandrio
EPI_ISL_2364656	RSIA Assyifa	Eijkman Institute for Molecular Biology, National Agency for Research and Innovation	Hidayat Trimarsanto, Lydia V. Panggalo, Iskandar Adnan, Sukma Oktavianthi, Lidwina Priliani, Edison Johar, Frilasita A Yudhaputri, Muhammad Rezki Rasyak, Willy Agustine, Safarina G Malik, Khin Saw Myint, Amin Soebandrio
EPI_ISL_2364659	RS Medirossa Cikarang	Eijkman Institute for Molecular Biology, National Agency for Research and Innovation	Hidayat Trimarsanto, Lydia V. Panggalo, Iskandar Adnan, Sukma Oktavianthi, Lidwina Priliani, Edison Johar, Frilasita A Yudhaputri, Muhammad Rezki Rasyak, Willy Agustine, Safarina G Malik, Khin Saw Myint, Amin Soebandrio
EPI_ISL_2364663	RS Medirossa Cikarang	Eijkman Institute for Molecular Biology, National Agency for Research and Innovation	Frilasita A Yudhaputri, Muhammad Rezki Rasyak, Willy Agustine, Hidayat Trimarsanto, Lydia V. Panggalo, Iskandar Adnan, Sukma Oktavianthi, Lidwina Priliani, Edison Johar, Safarina G Malik, Khin Saw Myint, Amin Soebandrio
EPI_ISL_2364666	RSIA PKU Muhammadiyah	Eijkman Institute for Molecular Biology, National Agency for Research and Innovation	Willy Agustine, Hidayat Trimarsanto, Lydia V. Panggalo, Iskandar Adnan, Sukma Oktavianthi, Lidwina Priliani, Edison Johar, Frilasita A Yudhaputri, Muhammad Rezki Rasyak, Safarina G Malik, Khin Saw Myint, Amin Soebandrio
EPI_ISL_2364669	RSIA PKU Muhammadiyah	Eijkman Institute for Molecular Biology, National Agency for Research and Innovation	Muhammad Rezki Rasyak, Willy Agustine, Hidayat Trimarsanto, Lydia V. Panggalo, Iskandar Adnan, Sukma Oktavianthi, Lidwina Priliani, Edison Johar, Frilasita A Yudhaputri, Safarina G Malik, Khin Saw Myint, Amin Soebandrio
EPI_ISL_2365884, EPI_ISL_2365896	Microbiology Department. Complexo Hospitalario Universitario de Vigo	Microbiology Department. Complexo Hospitalario Universitario de Vigo	Alfaya N, Alonso I, Alvarez M, Cabrera JJ, Carballo R, Cores O, Cortizo S, del-Campo V, Martinez L, Mediero G, Perez S, Potel C, Regueiro B, Rey S, Vasallo FJ
EPI_ISL_2374159	Research	National Reference Center for Viruses of Respiratory Infections, Institut Pasteur, Paris	Marion Barbet, Sylvie Behillil, Méline Bizard, Angela Brisebarre, Camille Capel, Vincent Enouf, Louise Lefrançois, Frédéric Lemoine, Christophe Malabat, Corinne Maufrais, Etienne Simon-Lorière, Maud Vanpeene, Sylvie Van der Werf ,Marianne Leruez-Ville
EPI_ISL_2374160	Research	National Reference Center for Viruses of Respiratory Infections, Institut Pasteur, Paris	Marion Barbet, Sylvie Behillil, Méline Bizard, Angela Brisebarre, Camille Capel, Vincent Enouf, Louise Lefrançois, Frédéric Lemoine, Christophe Malabat, Corinne Maufrais, Emmanuelle Permal, Etienne Simon-Lorière, Maud Vanpeene, Sylvie Van der Werf ,Marianne Leruez-Ville
EPI_ISL_2374161	Research	National Reference Center for Viruses of Respiratory Infections, Institut Pasteur, Paris	Marion Barbet, Sylvie Behillil, Méline Bizard, Angela Brisebarre, Camille Capel, Vincent Enouf, Louise Lefrançois, Frédéric Lemoine, Christophe Malabat, Corinne Maufrais, Etienne Simon-Lorière, Maud Vanpeene, Sylvie Van der Werf ,Marianne Leruez-Ville
EPI_ISL_2375848, EPI_ISL_2375849	Programa de Oncovirologia, Instituto Nacional de Câncer	Programa de Oncovirologia, Instituto Nacional de Câncer	Livia R. Goes, Juliana D. Siqueira, Marianne M. Garrido, Brunna M. Alves, Ana Cristina P. M. Pereira, Claudia Cicala, James Arthos, João P.B. Viola, Marcelo A. Soares
EPI_ISL_2383317	The teaching Baghdad hospital	PCR lab	Abdulhussein Thaer, Fadhil Hula
EPI_ISL_2384369	Cayman Islands Forensic Science Lab	Cayman Islands Forensic Science Lab	Jonathan Smellie, Karla Montes, Brittany Balcewich, Allen Grolla, Sarah Medina
EPI_ISL_2398296, EPI_ISL_2399459, EPI_ISL_2399460, EPI_ISL_2399461, EPI_ISL_2399462	Centre for Human Virology and Genomics, Microbiology Department, Nigerian Institute of Medical Research	Central Research Laboratory, Nigerian Institute of Medical Research	Chika Kingsley Onwuamah, Ayorinde Babatunde James, Joseph Ojonugwa Shaibu, Rahaman A. Ahmed, Azuka Patrick Okwuraiwe, Sola Ajibaye, Sharon Abimbola, Olufemi Samuel Amoo, Muinah Adenike Fowora, Phasha-Muchemenye Mmatshepho, Joy Ayoola, Nyam Itse Yusuf, Grace Oni, Yusuf Jimoh, Josiah Ayoola Isong, Uyi Emokpae, Ngozi Mirabel Otuonye, Rosemary Ajuma Audu, Bamidele Iwalokun, Babatunde Lawal Salako
EPI_ISL_2402534	OLVZ Aalst	OLVZ Aalst	Astrid Holderbeke
EPI_ISL_2422576, EPI_ISL_2422580, EPI_ISL_2422584, EPI_ISL_2422587, EPI_ISL_2422590, EPI_ISL_2422592, EPI_ISL_2422596, EPI_ISL_2422599, EPI_ISL_2422606, EPI_ISL_2422613, EPI_ISL_2422616, EPI_ISL_2422620, EPI_ISL_2422623, EPI_ISL_2422626, EPI_ISL_2422629, EPI_ISL_2422632, EPI_ISL_2422633, EPI_ISL_2422634, EPI_ISL_2422635, EPI_ISL_2422638, EPI_ISL_2422640, EPI_ISL_2422641, EPI_ISL_2422642, EPI_ISL_2422643, EPI_ISL_2422644, EPI_ISL_2422645, EPI_ISL_2422647, EPI_ISL_2422648, EPI_ISL_2422649, EPI_ISL_2422651, EPI_ISL_2422656, EPI_ISL_2422657, EPI_ISL_2422661, EPI_ISL_2422662, EPI_ISL_2422664, EPI_ISL_2422665, EPI_ISL_2422667			
see above	San Diego County Public Health Laboratory	Andersen lab at Scripps Research	SEARCH Alliance San Diego with Ashleigh Murphy, Jovan Shephard, Brett Austin
EPI_ISL_2422669, EPI_ISL_2422670, EPI_ISL_2422671, EPI_ISL_2422672, EPI_ISL_2422674, EPI_ISL_2422675, EPI_ISL_2422676, EPI_ISL_2422677, EPI_ISL_2422678, EPI_ISL_2422679, EPI_ISL_2422680, EPI_ISL_2422681, EPI_ISL_2422682, EPI_ISL_2422683, EPI_ISL_2422684, EPI_ISL_2422685, EPI_ISL_2422686, EPI_ISL_2422687, EPI_ISL_2422688, EPI_ISL_2422689, EPI_ISL_2422690, EPI_ISL_2422691, EPI_ISL_2422692, EPI_ISL_2422693, EPI_ISL_2422694, EPI_ISL_2422695, EPI_ISL_2422696, EPI_ISL_2422697, EPI_ISL_2422698, EPI_ISL_2422699, EPI_ISL_2422700, EPI_ISL_2422701, EPI_ISL_2422702, EPI_ISL_2422703, EPI_ISL_2422704, EPI_ISL_2422705, EPI_ISL_2422706, EPI_ISL_2422707, EPI_ISL_2422708, EPI_ISL_2422709, EPI_ISL_2422710, EPI_ISL_2422711, EPI_ISL_2422712, EPI_ISL_2422713, EPI_ISL_2422714, EPI_ISL_2422715, EPI_ISL_2422716, EPI_ISL_2422717, EPI_ISL_2422718, EPI_ISL_2422719			
see above	Sharp HealthCare Laboratory	Andersen lab at Scripps Research	SEARCH Alliance San Diego with Aaron Harding, Jacquelyn Berumen, Cathy Woerle, Liam McGinnis, Art Mendoza, Omid Bakhtar
EPI_ISL_2422720, EPI_ISL_2422722, EPI_ISL_2422723, EPI_ISL_2422724, EPI_ISL_2422726, EPI_ISL_2422727, EPI_ISL_2422728, EPI_ISL_2422729, EPI_ISL_2422730, EPI_ISL_2422731, EPI_ISL_2422733, EPI_ISL_2422734, EPI_ISL_2422737, EPI_ISL_2422738, EPI_ISL_2422739, EPI_ISL_2422740, EPI_ISL_2422742, EPI_ISL_2422743, EPI_ISL_2422744, EPI_ISL_2422745, EPI_ISL_2422747, EPI_ISL_2422748, EPI_ISL_2422749, EPI_ISL_2422750, EPI_ISL_2422751, EPI_ISL_2422752, EPI_ISL_2422753, EPI_ISL_2422754, EPI_ISL_2422755, EPI_ISL_2422757, EPI_ISL_2422758, EPI_ISL_2422759, EPI_ISL_2422760, EPI_ISL_2422763,			

EPI_ISL_2422764, EPI_ISL_2422765, EPI_ISL_2422766, EPI_ISL_2422767, EPI_ISL_2422768, EPI_ISL_2422769, EPI_ISL_2422770, EPI_ISL_2422771, EPI_ISL_2422772, EPI_ISL_2422774, EPI_ISL_2422776, EPI_ISL_2422778, EPI_ISL_2422779, EPI_ISL_2422780, EPI_ISL_2422781, EPI_ISL_2422782			
see above	San Diego County Public Health Laboratory	Andersen lab at Scripps Research	SEARCH Alliance San Diego with Ashleigh Murphy, Jovan Shephard, Brett Austin
EPI_ISL_2422783, EPI_ISL_2422784, EPI_ISL_2422785, EPI_ISL_2422786, EPI_ISL_2422787, EPI_ISL_2422788, EPI_ISL_2422789, EPI_ISL_2422790, EPI_ISL_2422791, EPI_ISL_2422792, EPI_ISL_2422793, EPI_ISL_2422794, EPI_ISL_2422795, EPI_ISL_2422796, EPI_ISL_2422797, EPI_ISL_2422798, EPI_ISL_2422799, EPI_ISL_2422800, EPI_ISL_2422801, EPI_ISL_2422802, EPI_ISL_2422803, EPI_ISL_2422804, EPI_ISL_2422805, EPI_ISL_2422806, EPI_ISL_2422807, EPI_ISL_2422808, EPI_ISL_2422809, EPI_ISL_2422810, EPI_ISL_2422811, EPI_ISL_2422812, EPI_ISL_2422813, EPI_ISL_2422814, EPI_ISL_2422828, EPI_ISL_2422829, EPI_ISL_2422830, EPI_ISL_2422834, EPI_ISL_2422835, EPI_ISL_2422836, EPI_ISL_2422837, EPI_ISL_2422838, EPI_ISL_2422839, EPI_ISL_2422840, EPI_ISL_2422841, EPI_ISL_2422842, EPI_ISL_2422843, EPI_ISL_2422844, EPI_ISL_2422845, EPI_ISL_2422846, EPI_ISL_2422847, EPI_ISL_2422848, EPI_ISL_2422849, EPI_ISL_2422850, EPI_ISL_2422851, EPI_ISL_2422852, EPI_ISL_2422853, EPI_ISL_2422854, EPI_ISL_2422855, EPI_ISL_2422856, EPI_ISL_2422857, EPI_ISL_2422858, EPI_ISL_2422859, EPI_ISL_2422860, EPI_ISL_2422861, EPI_ISL_2422862, EPI_ISL_2422863, EPI_ISL_2422864, EPI_ISL_2422865, EPI_ISL_2422866, EPI_ISL_2422867, EPI_ISL_2422868, EPI_ISL_2422869, EPI_ISL_2422870, EPI_ISL_2422871, EPI_ISL_2422872, EPI_ISL_2422873, EPI_ISL_2422874, EPI_ISL_2422875, EPI_ISL_2422876, EPI_ISL_2422877, EPI_ISL_2422878, EPI_ISL_2422879, EPI_ISL_2422880, EPI_ISL_2422881, EPI_ISL_2422882, EPI_ISL_2422883, EPI_ISL_2422884, EPI_ISL_2422885, EPI_ISL_2422886, EPI_ISL_2422887, EPI_ISL_2422888, EPI_ISL_2422889, EPI_ISL_2422890, EPI_ISL_2422891, EPI_ISL_2422892, EPI_ISL_2422893, EPI_ISL_2422894, EPI_ISL_2422895, EPI_ISL_2422896			
see above	Sharp HealthCare Laboratory	Andersen lab at Scripps Research	SEARCH Alliance San Diego with Aaron Harding, Jacquelyn Berumen, Cathy Woerle, Liam McGinnis, Art Mendoza, Omid Bakhtar
EPI_ISL_2422897, EPI_ISL_2422898, EPI_ISL_2422899, EPI_ISL_2422900, EPI_ISL_2422901, EPI_ISL_2422902, EPI_ISL_2422903, EPI_ISL_2422905, EPI_ISL_2422906, EPI_ISL_2422907, EPI_ISL_2422908, EPI_ISL_2422909, EPI_ISL_2422910, EPI_ISL_2422911, EPI_ISL_2422912, EPI_ISL_2422913, EPI_ISL_2422914, EPI_ISL_2422915, EPI_ISL_2422916, EPI_ISL_2422917, EPI_ISL_2422918, EPI_ISL_2422919, EPI_ISL_2422920, EPI_ISL_2422921, EPI_ISL_2422922, EPI_ISL_2422923, EPI_ISL_2422924, EPI_ISL_2422925, EPI_ISL_2422926, EPI_ISL_2422927, EPI_ISL_2422928, EPI_ISL_2422929, EPI_ISL_2422930, EPI_ISL_2422931, EPI_ISL_2422932, EPI_ISL_2422933, EPI_ISL_2422934, EPI_ISL_2422935, EPI_ISL_2422936, EPI_ISL_2422937, EPI_ISL_2422938, EPI_ISL_2422939, EPI_ISL_2422940, EPI_ISL_2422941, EPI_ISL_2422942, EPI_ISL_2422943, EPI_ISL_2422944, EPI_ISL_2422945, EPI_ISL_2422946, EPI_ISL_2422947, EPI_ISL_2422948, EPI_ISL_2422949, EPI_ISL_2422950, EPI_ISL_2422951, EPI_ISL_2422952, EPI_ISL_2422953, EPI_ISL_2422954, EPI_ISL_2422955, EPI_ISL_2422956, EPI_ISL_2422957, EPI_ISL_2422958, EPI_ISL_2422959, EPI_ISL_2422960, EPI_ISL_2422961, EPI_ISL_2422962, EPI_ISL_2422963, EPI_ISL_2422964, EPI_ISL_2422965, EPI_ISL_2422966, EPI_ISL_2422967, EPI_ISL_2422970, EPI_ISL_2422971, EPI_ISL_2422972, EPI_ISL_2422973, EPI_ISL_2422974, EPI_ISL_2422975			
see above	San Diego County Public Health Laboratory	Andersen lab at Scripps Research	SEARCH Alliance San Diego with Ashleigh Murphy, Jovan Shephard, Brett Austin
EPI_ISL_2426898, EPI_ISL_2426899, EPI_ISL_2426900, EPI_ISL_2426901, EPI_ISL_2426902, EPI_ISL_2426903, EPI_ISL_2426904, EPI_ISL_2426905, EPI_ISL_2426906, EPI_ISL_2426907, EPI_ISL_2426908, EPI_ISL_2426909, EPI_ISL_2426910, EPI_ISL_2426911, EPI_ISL_2426912, EPI_ISL_2426913, EPI_ISL_2426914, EPI_ISL_2426915, EPI_ISL_2426916, EPI_ISL_2426917, EPI_ISL_2426918, EPI_ISL_2426919, EPI_ISL_2426920, EPI_ISL_2426921, EPI_ISL_2426922, EPI_ISL_2426923, EPI_ISL_2426924, EPI_ISL_2426925, EPI_ISL_2426926, EPI_ISL_2426927, EPI_ISL_2426928, EPI_ISL_2426929, EPI_ISL_2426930, EPI_ISL_2426931, EPI_ISL_2426932, EPI_ISL_2426933, EPI_ISL_2426934, EPI_ISL_2426935, EPI_ISL_2426936, EPI_ISL_2426937, EPI_ISL_2426938, EPI_ISL_2426939, EPI_ISL_2426940, EPI_ISL_2426942, EPI_ISL_2426943			
see above	Centers for Disease Control and Prevention, Dengue Branch	Centers for Disease Control and Prevention, Dengue Branch	Gilberto A. Santiago, Glenda Gonzalez, Betzabel Flores, Keyla Charriez, Gabriela Paz-Bailey, Jorge L. Munoz-Jordan
EPI_ISL_2438892	Motol University Hospital	Charles University, Faculty of Science, BIOCEV, OMICS Genomics	Vladimír Hampl, Štpánka Hrdá, Ruth Tachezy, Blanka Hamplová, Sebastian Cristian Treitli, Zoltán Füssy, Magdalena Janáová, Ingrid Poláková, Jana Šmahelová, Jíí Novák
EPI_ISL_2443546	Lboratorio Central de Saude Publica do Estado do Parana (LACEN/PR)	Laboratory of Respiratory Viruses and Measles, Oswaldo Cruz Institute, FIOCRUZ	Paola Resende, Luciana Appolinario, Fernando Motta, Anna Carolina Paixao, Ana Carolina Mendonca, Alice Sampaio Rocha, Taina Venas, Elisa Cavalcante Pereira, Renata Serrano Lopes, Irina Riediger, Marilda Siqueira on behalf of the Fiocruz COVID-19 Genomic Surveillance Network
EPI_ISL_2448646, EPI_ISL_2448647, EPI_ISL_2448648, EPI_ISL_2448649, EPI_ISL_2448650, EPI_ISL_2448651, EPI_ISL_2448652, EPI_ISL_2448654, EPI_ISL_2448679, EPI_ISL_2448680, EPI_ISL_2448681			
see above	Molecular diagnostic laboratory of Federal Budget Institution of Science "Central Research Institute of Epidemiology" of The Federal Service on Customers' Rights Protection and Human Well-being Surveillance	Group of Genomics and Postgenomic Technologies of Central Research Institute of Epidemiology	Samoilov A.E., Kaptelova V.V., Korneenko E.V., Valdohina A.V., Bulanenko V.P., Saenko S.S., Sinitsyn S.O., Golubeva A.G., Speranskaya A.S., Tivanova E.V., Shipulina O.Y., Akimkin V.G.
EPI_ISL_2455237	CICESE-BSL3	CICESE-BSL3	Cervantes Karla, Gonzalez-Sanchez Ricardo, Martinez Miguel, Galindo Clara and Licea Alexei
EPI_ISL_2455244	CICESE-BSL3	CICESE-BSL3	Karla Cervantes, Ricardo Gonzalez, Miguel Martinez, Clara Galindo and Alexei Licea
EPI_ISL_2458066	Retrovirus Laboratory Adolfo Lutz Institute_Sao Paulo	Retrovirus Laboratory Adolfo Lutz Institute	Gabriela Bastos Cabral, Giselle I S Lopez-Lopes, Cintia Ahagon, Audrey Cilli, Paula Morena Guimaraes, Igor Mohamed Hussein, Luís Brigido
EPI_ISL_2462895	Hospital	National Reference Center for Viruses of Respiratory Infections, Institut Pasteur, Paris	Marion Barbet, Sylvie Behillil, Méline Bizard, Angela Brisebarre, Camille Capel, Vincent Enouf, Louise Lefrançois, Frédéric Lemoine, Christophe Malabat, Corinne Maufrais, Etienne Simon-Lorière, Maud Vanpeene, Sylvie Van der Werf ,Olivia Raulin
EPI_ISL_2467872, EPI_ISL_2467873, EPI_ISL_2467874, EPI_ISL_2467875, EPI_ISL_2467876, EPI_ISL_2467877, EPI_ISL_2467878, EPI_ISL_2467879	Rockford Ambulatory Surgery Center	BioInfoExperts LLC	Sarah E. Hagarty, David J Nolan, Rebecca Rose, Susanna L Lamers, Ed Yavitz
EPI_ISL_2484218, EPI_ISL_2484219	University of Brescia - Dept. of Molecular and Translational Medicine	Istituto Zooprofilattico Sperimentale della Lombardia e dell'Emilia Romagna (IZSLER), Risk Analysis and Genomic Epidemiology Unit	Caruso Arnaldo, Fiorentini Simona, Caccuri Francesca, Messali Serena, Zani Alberto, Marina Morganti, Erika Scaltriti, Stefano Pongolini
EPI_ISL_2485171, EPI_ISL_2485172, EPI_ISL_2485173, EPI_ISL_2485174, EPI_ISL_2485176, EPI_ISL_2485180, EPI_ISL_2485181, EPI_ISL_2485188, EPI_ISL_2485189, EPI_ISL_2485190, EPI_ISL_2485191, EPI_ISL_2485194, EPI_ISL_2485195, EPI_ISL_2485197, EPI_ISL_2485202, EPI_ISL_2485203, EPI_ISL_2485204, EPI_ISL_2485207, EPI_ISL_2485208, EPI_ISL_2485210, EPI_ISL_2485214, EPI_ISL_2485215, EPI_ISL_2485220, EPI_ISL_2485222, EPI_ISL_2485223, EPI_ISL_2485224, EPI_ISL_2485225			
see above	ILV Kärnten	Bergthaler laboratory, CeMM Research Center for Molecular Medicine of the Austrian Academy of Sciences	Lukas Endler, Anna Schedl, Fabian Amman, Petr Triska, Thomas Penz, Benedikt Agerer, Maelle Le Moing, Michael Schuster, Bekir Erguner, Jan Laine, Martin Senekowitsch, Christoph Bock, Andreas Bergthaler
EPI_ISL_2491665, EPI_ISL_2491666, EPI_ISL_2491668	Centre Hospitalier Universitaire Clermont-Ferrand	CHU Clermont-Ferrand, service de virologie	Bisseux Maxime, Mirand Audrey, Combes Patricia, Henquell Cécile
EPI_ISL_2491669	Centre De Prelevement COVID RIOM	CHU Clermont-Ferrand, service de virologie	Bisseux Maxime, Mirand Audrey, Combes Patricia, Henquell Cécile
EPI_ISL_2491670	Centre Hospitalier Universitaire Clermont-Ferrand	CHU Clermont-Ferrand, service de virologie	Bisseux Maxime, Mirand Audrey, Combes Patricia, Henquell Cécile
EPI_ISL_2495717, EPI_ISL_2495718, EPI_ISL_2495719, EPI_ISL_2495720	MB-Cadham Provincial laboratory	National Microbiology Laboratory (NML)	Anna Majer, Shari Tyson, Grace Seo, Philip Mabon, Elsie Grudeski, Rhiannon Huzarewich, Russell Mandes, Anneliese Landgraff, Jennifer Tanner, Natalie Knox, Morag Graham, Gary Van Domselaar, Paul Van Caeseele, Jared Bullard, David Alexander, Kerry Dust, Nathalie Bastien, Yan Li, Timothy Booth, Darian Hole, Madison Chapel, Kirsten Biggar, CanCOGeN's metadata curation team, Public Health Agency of Canada CanCOGeN team
EPI_ISL_2501088, EPI_ISL_2501089	Centre for Human Virology and Genomics, Microbiology Department, Nigerian Institute of Medical Research	Central Research Laboratory, Nigerian Institute of Medical Research	Chika Kingsley Onwuamah, Ayorinde Babatunde James, Joseph Ojonugwa Shaibu, Rahaman A. Ahmed, Azuka Patrick Okwuraiwe, Sola Ajibaye, Sharon Abimbola, Olufemi Samuel Amoo, Muinah Adenike Fowora, Phasha-Muchemenye Mmatshepho, Joy Ayoola, Nyam Itse Yusuf, Grace Oni, Yusuf Jimoh, Josiah Ayoola Isong, Uyi Emokpae, Ngozi Mirabel Otuonye, Rosemary Ajuma Audu, Bamidele Iwalokun, Babatunde Lawal Salako
EPI_ISL_2501312, EPI_ISL_2501348, EPI_ISL_2501349, EPI_ISL_2501350, EPI_ISL_2501351, EPI_ISL_2501352, EPI_ISL_2501353, EPI_ISL_2501354, EPI_ISL_2501355, EPI_ISL_2501356, EPI_ISL_2501357, EPI_ISL_2501358, EPI_ISL_2501359, EPI_ISL_2501360, EPI_ISL_2501361, EPI_ISL_2501362, EPI_ISL_2501379, EPI_ISL_2501380, EPI_ISL_2501381, EPI_ISL_2501382, EPI_ISL_2501383, EPI_ISL_2501384, EPI_ISL_2501385, EPI_ISL_2501386, EPI_ISL_2501387, EPI_ISL_2501388, EPI_ISL_2501389, EPI_ISL_2501390, EPI_ISL_2501391, EPI_ISL_2501392, EPI_ISL_2501393			
see above	Microbiology Department. Complexo Hospitalario Universitario de Vigo	Microbiology Department. Complexo Hospitalario Universitario de Vigo	Alfaya N, Alonso I, Alvarez M, Cabrera JJ, Carballo R, Cores O, Cortizo S, del-Campo V, Martinez L, Mediero G, Perez S, Potel C, Regueiro B, Rey S, Vasallo FJ
EPI_ISL_2502019, EPI_ISL_2502026	Centre for Human Virology and Genomics, Microbiology Department, Nigerian Institute of Medical Research	Central Research Laboratory, Nigerian Institute of Medical Research	Chika Kingsley Onwuamah, Ayorinde Babatunde James, Joseph Ojonugwa Shaibu, Rahaman A. Ahmed, Azuka Patrick Okwuraiwe, Sola Ajibaye, Sharon Abimbola, Olufemi Samuel Amoo, Muinah Adenike Fowora, Phasha-Muchemenye Mmatshepho, Joy Ayoola, Nyam Itse Yusuf, Grace Oni, Yusuf Jimoh, Josiah Ayoola Isong, Uyi Emokpae, Ngozi Mirabel Otuonye, Rosemary Ajuma Audu, Bamidele Iwalokun, Babatunde Lawal Salako
EPI_ISL_2502639, EPI_ISL_2502641, EPI_ISL_2502642, EPI_ISL_2502645, EPI_ISL_2502648, EPI_ISL_2502650, EPI_ISL_2502651, EPI_ISL_2502652, EPI_ISL_2502653, EPI_ISL_2502654, EPI_ISL_2502655			
see above	All India Institute of Medical Sciences Delhi Hospital	Virology Laboratory, AIIMS Delhi	Aashish Choudhary, Sumedha Bagga, Lata Rani, Dibyabhaba Pradhan, Pooja Pandey, Jyoti Jethani, Shivram Dhakad, Nazneen Arif, Deepankar Srigyan, Megha Brijwal, Lalit Dar, Manish Soneja, Rakesh Lodha, Puneet Kaur, Ritu Gupta, Subrata Sinha, Chitra Sarkar, Randeep Guleria
EPI_ISL_2532369	Labo Analyses Med	National Reference Center for Viruses of Respiratory Infections, Institut Pasteur, Paris	Marion Barbet, Sylvie Behillil, Méline Bizard, Angela Brisebarre, Camille Capel, Vincent Enouf, Louise Lefrançois, Frédéric Lemoine, Christophe Malabat, Corinne Maufrais, Etienne Simon-Lorière, Maud Vanpeene, Sylvie Van der Werf ,Hervé Dupont
EPI_ISL_2532467	Labo Analyses Med	National Reference Center for Viruses of Respiratory Infections, Institut Pasteur, Paris	Marion Barbet, Sylvie Behillil, Méline Bizard, Angela Brisebarre, Camille Capel, Vincent Enouf, Louise Lefrançois, Frédéric Lemoine, Christophe Malabat, Corinne Maufrais, Adrien Pain, Etienne Simon-Lorière, Maud Vanpeene, Sylvie Van der Werf
EPI_ISL_2532470	Labo Analyses Med	National Reference Center for Viruses of Respiratory Infections, Institut Pasteur, Paris	Marion Barbet, Sylvie Behillil, Méline Bizard, Angela Brisebarre, Camille Capel, Vincent Enouf, Louise Lefrançois, Frédéric Lemoine, Christophe Malabat, Corinne Maufrais, Etienne Simon-Lorière, Maud Vanpeene, Sylvie Van der Werf
EPI_ISL_2532676	IZSM	IZSM-U.O.C. Virologia	Maurizio Viscardi, Lorena Cardillo, Claudio de Martinis, Giovanna Fusco, Esterina De Carlo, Antonio Limone

EPI_ISL_2535801	National Influenza Centre	National Influenza Centre	William K. Ampofo, Michael Marks, Ivy A. Asante, Sharon Hsu, Benjamiin B. Lindsey, Benjamin H. Foulkes, Mildred Adusei-Poku, Linda Boatemaa, Lorreta Kwasah, Joseph Oliver-Commey, Ernest Asiedu, Franklin Asiedu-Bekoe, Gordon Awandare, Joyce Ngoi, Dennis Laryea, Mathew D. Parker, Thushan I de Silva,
EPI_ISL_2544854, EPI_ISL_2544855, EPI_ISL_2544879, EPI_ISL_2544880, EPI_ISL_2544883, EPI_ISL_2544884, EPI_ISL_2544885, EPI_ISL_2544886, EPI_ISL_2544888, EPI_ISL_2544890, EPI_ISL_2544892, EPI_ISL_2544893, EPI_ISL_2544894, EPI_ISL_2544895, EPI_ISL_2544898			
see above	Laboratorio de Pesquisa em Virologia, FAMERP, SJRP	Laboratorio de Pesquisa em Virologia, FAMERP, SJRP	Cecília Artico Banho; Lívia Sacchetto; Guilherme Campos; Fábio Sossai Possebon; Leila Sabrina Ullmann; Cíntia Bittar; Helena Lage Ferreira; Jorge A. Petroli Marchesi; Maisa C. Pereira Parra; Marília Moraes; Paula Rahal; Paulo Inacio da Costa; João Pessoa Araújo Jr.; Maurício L. Nogueira.
EPI_ISL_2566185, EPI_ISL_2566187, EPI_ISL_2566190, EPI_ISL_2603239, EPI_ISL_2603240, EPI_ISL_2603241, EPI_ISL_2603245, EPI_ISL_2603246	Microbiology Department. Complexo Hospitalario Universitario de Vigo	Microbiology Department. Complexo Hospitalario Universitario de Vigo	Alfaya N, Alonso I, Alvarez M, Cabrera JJ, Carballo R, Cores O, Cortizo S, del-Campo V, Martinez L, Mediero G, Perez S, Potel C, Regueiro B, Rey S, Vasallo FJ
EPI_ISL_2612601, EPI_ISL_2612612	Labo Analyses Med	National Reference Center for Viruses of Respiratory Infections, Institut Pasteur, Paris	Marion Barbet, Sylvie Behillil, Méline Bizard, Angela Brisebarre, Camille Capel, Vincent Enouf, Louise Lefrançois, Frédéric Lemoine, Christophe Malabat, Corinne Maufrais, Etienne Simon-Lorière, Maud Vanpeene, Sylvie Van der Werf ,Pierre-Yves Leonard
EPI_ISL_2614351	Labortorio Central de Saude Publica do Estado do Rio de Janeiro (LACEN/RJ)	Laboratory of Respiratory Viruses and Measles, Oswaldo Cruz Institute, FIOCRUZ	Paola Resende, Luciana Appolinario, Fernando Motta, Anna Carolina Paixao, Ana Carolina Mendonca, Alice Sampaio Rocha, Taina Venas, Elisa Cavalcante Pereira, Renata Serrano Lopes, Andrea Cony Cavalcanti, Marilda Siqueira on behalf of the Fiocruz COVID-19 Genomic Surveillance Network
EPI_ISL_2614513, EPI_ISL_2614514, EPI_ISL_2614515, EPI_ISL_2614516, EPI_ISL_2614517	Instituto Adolfo Lutz Central	Instituto Adolfo Lutz, Interdiciplinary Procedures Center, Strategic Laboratory	Claudio Tavares Sacchi, Claudia Regina Gonçalves, Erica Valessa Ramos Gomes, Karoline Rodrigues Campos, Caio Vinicius Dias Lopes, Leonardo Jose Tadeu de Araujo
EPI_ISL_2614540	IdenGene Medicina Diagnostica SA	Instituto Adolfo Lutz, Interdiciplinary Procedures Center, Strategic Laboratory	Claudio Tavares Sacchi, Claudia Regina Gonçalves, Erica Valessa Ramos Gomes, Karoline Rodrigues Campos, Caio Vinicius Dias Lopes, Leonardo Jose Tadeu de Araujo
EPI_ISL_2614541	LACEN do Estado de Goias	Instituto Adolfo Lutz, Interdiciplinary Procedures Center, Strategic Laboratory	Claudio Tavares Sacchi, Claudia Regina Gonçalves, Erica Valessa Ramos Gomes, Karoline Rodrigues Campos, Caio Vinicius Dias Lopes, Leonardo Jose Tadeu de Araujo
EPI_ISL_2617086	Africa_CDC - Malawi P4 S2	CERI, Centre for Epidemic Response and Innvoation, Stellenbosch University and KRISP, KZN Research Innovation and Sequencing Platform, UKZN.	Mvula B, Chilima B, Chiwaula M, Mwangomba W, Panja L, Kasambara W, Auld A, Kim L, Kampira E, Kaba M, Wadonda N, Maida A, Giandhari J, Pillay S, Naidoo Y, Lessells R, Emmanuel SJ, Tegally H, Wilkinson E, de Oliveira T
EPI_ISL_2617876, EPI_ISL_2617877, EPI_ISL_2617878, EPI_ISL_2617879	LABCOVID_HCPA	LABRESIS_HCPA	Wink PL, Martins AF, Volpato F, Monteiro F, Zavascki AP, Barth AL
EPI_ISL_2627738	Belo Horizonte center-south emergency care unit - UPA-BH	Laboratório de Virologia Clínica e Molecular	Erick Gustavo Dorlass, Karine Lima Lourenço, Rubens Daniel Miserani Magalhães, Hugo Sato, Alex Fiorini, Renata Peixoto, Helena Perez Coelho, Ana Paula Salles Fernandes, Bruna Larotonda Telezynski, Guilherme Pereira Scagion, Tatiana Ometto, Luciano Matsumiya Thomazelli, Danielle Bruna Leal Oliveira, Edison Luiz Durigon, Flavio Fonseca e Santuza Teixeira
EPI_ISL_2627985, EPI_ISL_2627986, EPI_ISL_2627987	Hospital	National Reference Center for Viruses of Respiratory Infections, Institut Pasteur, Paris	Marion Barbet, Sylvie Behillil, Méline Bizard, Angela Brisebarre, Camille Capel, Vincent Enouf, Louise Lefrançois, Frédéric Lemoine, Christophe Malabat, Corinne Maufrais, Etienne Simon-Lorière, Maud Vanpeene, Sylvie Van der Werf ,CéCile Farrugia
EPI_ISL_2629070	HEGP - Laboratoire de Virologie	HEGP - Laboratoire de Virologie	David Veyer, Sandrine Imbeaud, Julien Puech, Hélène Péré, Maxime Wack, Nicolas Robillard
EPI_ISL_2637801	U.O. Igiene, Ospedale Policlinico San Martino	U.O. Igiene, Ospedale Policlinico San Martino	Bruzzone Bianca, Caligiuri Patrizia, De Pace Vanessa, Domnich Alexander, Orsi Andrea, Ricucci Valentina, Lai Alessia, Bergna Annalisa, Ventura Francesco, Della Ventura Carla, Zehender Gianguglielmo, Icardi Giancarlo on behalf of SARS-CoV-2 ITALIAN RESEARCH ENTERPRISE-(SCIRE) Collaborative Group
EPI_ISL_2645531	Laboratorio Central de Saude Publica do Estado do Espirito Santo (LACEN/ES)	Laboratory of Respiratory Viruses and Measles, Oswaldo Cruz Institute, FIOCRUZ	Paola Resende, Luciana Appolinario, Fernando Motta, Anna Carolina Paixao, Ana Carolina Mendonca, Alice Sampaio Rocha, Taina Venas, Eilisa Cavalcante Pereira, Renata Serrano Lopes, Rodrigo Ribeiro Rodrigues, Marilda Siqueira on behalf of the Fiocruz COVID-19 Genomic Surveillance Network
EPI_ISL_2645639	Laboratorio Central de Saude Publica do Estado de Alagoas (LACEN/AL)	Laboratory of Respiratory Viruses and Measles, Oswaldo Cruz Institute, FIOCRUZ	Paola Resende, Luciana Appolinario, Fernando Motta, Anna Carolina Paixao, Ana Carolina Mendonca, Alice Sampaio Rocha, Taina Venas, Elisa Cavalcante Pereira, Renata Serrano Lopes, Anderson Brandao Leite, Marilda Siqueira on behalf of the Fiocruz COVID-19 Genomic Surveillance Network
EPI_ISL_2652117	AZ Klina	AZ Klina	Carl Vael - Lynsey Berckmans
EPI_ISL_2652123	Molecular diagnostic laboratory of Federal Budget Institution of Science "Central Research Institute of Epidemiology" of The Federal Service on Customers' Rights Protection and Human Well-being Surveillance	Group of Genomics and Postgenomic Technologies of Central Research Institute of Epidemiology	Samoilov A.E., Kaptelova V.V., Korneenko E.V., Valdohina A.V., Bulanenko V.P., Saenko S.S., Golubeva A.G., Lebedeva L.A., Shubina Y.A., Savosina L.V., Zhaleiko Z.P., Lukyanov A.V., Speranskaya A.S., Tivanova E.V., Kondrasheva L.V., Akimkin V.G.
EPI_ISL_2652124	Molecular diagnostic laboratory of Federal Budget Institution of Science "Central Research Institute of Epidemiology" of The Federal Service on Customers' Rights Protection and Human Well-being Surveillance	Group of Genomics and Postgenomic Technologies of Central Research Institute of Epidemiology	Samoilov A.E., Kaptelova V.V., Korneenko E.V., Valdohina A.V., Bulanenko V.P., Saenko S.S., Sinitsyn S.O., Golubeva A.G., Speranskaya A.S., Tivanova E.V., Shipulina O.Y., Akimkin V.G.
EPI_ISL_2652133, EPI_ISL_2652134	Molecular diagnostic laboratory of Federal Budget Institution of Science "Central Research Institute of Epidemiology" of The Federal Service on Customers' Rights Protection and Human Well-being Surveillance	Group of Genomics and Postgenomic Technologies of Central Research Institute of Epidemiology	Samoilov A.E., Kaptelova V.V., Korneenko E.V., Valdohina A.V., Bulanenko V.P., Saenko S.S., Golubeva A.G., Lebedeva L.A., Shubina Y.A., Savosina L.V., Zhaleiko Z.P., Lukyanov A.V., Speranskaya A.S., Tivanova E.V., Kondrasheva L.V., Akimkin V.G.
EPI_ISL_2652135, EPI_ISL_2652136	Molecular diagnostic laboratory of Federal Budget Institution of Science "Central Research Institute of Epidemiology" of The Federal Service on Customers' Rights Protection and Human Well-being Surveillance	Group of Genomics and Postgenomic Technologies of Central Research Institute of Epidemiology	Samoilov A.E., Kaptelova V.V., Korneenko E.V., Valdohina A.V., Bulanenko V.P., Saenko S.S., Sinitsyn S.O., Golubeva A.G., Speranskaya A.S., Tivanova E.V., Shipulina O.Y., Akimkin V.G.
EPI_ISL_2660452	Laboratory of Respiratory Viruses and Measles, Oswaldo Cruz Institute, FIOCRUZ	Laboratory of Respiratory Viruses and Measles, Oswaldo Cruz Institute, FIOCRUZ	Paola Resende, Luciana Appolinario, Fernando Motta, Anna Carolina Paixao, Ana Carolina Mendonca, Alice Sampaio Rocha, Taina Venas, Elisa Cavalcante Pereira, Renata Serrano Lopes, Marilda Siqueira on behalf of the Fiocruz COVID-19 Genomic Surveillance Network
EPI_ISL_2661782, EPI_ISL_2661783, EPI_ISL_2661784, EPI_ISL_2661786, EPI_ISL_2661788, EPI_ISL_2661794, EPI_ISL_2661801, EPI_ISL_2661803, EPI_ISL_2661804, EPI_ISL_2661811, EPI_ISL_2661813, EPI_ISL_2661823, EPI_ISL_2661824, EPI_ISL_2661825, EPI_ISL_2661826, EPI_ISL_2661827			
see above	Laboratorio Central de Saude Publica do Estado do Rio Grande do Sul (LACEN-RS)	Laboratory of Respiratory Viruses and Measles, Oswaldo Cruz Institute, FIOCRUZ	Paola Resende, Luciana Appolinario, Fernando Motta, Anna Carolina Paixao, Ana Carolina Mendonca, Alice Sampaio Rocha, Taina Venas, Elisa Cavalcante Pereira, Renata Serrano Lopes, Tatiana Schaffer Gregianini, Richard Salvato, Marilda Siqueira on behalf of the Fiocruz COVID-19 Genomic Surveillance Network
EPI_ISL_2663115, EPI_ISL_2663117, EPI_ISL_2663118, EPI_ISL_2663120, EPI_ISL_2663121, EPI_ISL_2663123, EPI_ISL_2663162, EPI_ISL_2663163, EPI_ISL_2663165, EPI_ISL_2663166, EPI_ISL_2663168, EPI_ISL_2663171, EPI_ISL_2663173, EPI_ISL_2663174, EPI_ISL_2663179, EPI_ISL_2663181, EPI_ISL_2663182, EPI_ISL_2663184, EPI_ISL_2663185, EPI_ISL_2663187, EPI_ISL_2663188, EPI_ISL_2663190			
see above	Ochsner Health	Bioinfoexperts, LLC	Judy Crabtree, Amy Feehan, Rebecca Rose, David J. Nolan, Tessa LaFleur, Julia-Garcia-Diaz, Lucio Miele, Susanna L. Lamers
EPI_ISL_2677079	Labortorio Central de Saude Publica do Estado de Santa Catarina (LACEN/SC)	Laboratory of Respiratory Viruses and Measles, Oswaldo Cruz Institute, FIOCRUZ	Paola Resende, Luciana Appolinario, Fernando Motta, Anna Carolina Paixao, Ana Carolina Mendonca, Alice Sampaio Rocha, Taina Venas, Elisa Cavalcante Pereira, Renata Serrano Lopes, Darcita Buerger Rovaris, Sandra Bianchini Fernandes, Marilda Siqueira on behalf of the Fiocruz COVID-19 Genomic Surveillance Network
EPI_ISL_2688267, EPI_ISL_2688277	Centers for Disease Control and Prevention, Dengue Branch	Centers for Disease Control and Prevention, Dengue Branch	Gilberto A. Santiago, Glenda Gonzalez, Betzabel Flores, Keyla Charriez, Gabriela Paz-Bailey, Jorge L. Munoz-Jordan
EPI_ISL_2689147, EPI_ISL_2689148	MSK Microbiology Lab	MSK Microbiology Lab	Esther Babady, Tracy McMillen, Krupa Jani
EPI_ISL_2690129, EPI_ISL_2690130, EPI_ISL_2690131, EPI_ISL_2690132, EPI_ISL_2690151, EPI_ISL_2690152, EPI_ISL_2690153, EPI_ISL_2690154, EPI_ISL_2690155, EPI_ISL_2690156, EPI_ISL_2690158, EPI_ISL_2690159, EPI_ISL_2690160, EPI_ISL_2690162, EPI_ISL_2690163, EPI_ISL_2690164, EPI_ISL_2690165, EPI_ISL_2690166, EPI_ISL_2690167, EPI_ISL_2690173, EPI_ISL_2690176, EPI_ISL_2690177, EPI_ISL_2690185, EPI_ISL_2690186, EPI_ISL_2690187, EPI_ISL_2690188, EPI_ISL_2690189, EPI_ISL_2690190			
see above	Ochsner Health	Bioinfoexperts, LLC	Judy Crabtree, Amy Feehan, Rebecca Rose, David J. Nolan, Tessa LaFleur, Julia-Garcia-Diaz, Lucio Miele, Susanna L. Lamers

EPI_ISL_2690433, EPI_ISL_2690434, EPI_ISL_2690444, EPI_ISL_2690572, EPI_ISL_2690576, EPI_ISL_2690580, EPI_ISL_2690595, EPI_ISL_2690596, EPI_ISL_2690602, EPI_ISL_2690608, EPI_ISL_2690609, EPI_ISL_2690611, EPI_ISL_2690612, EPI_ISL_2690614, EPI_ISL_2690621, EPI_ISL_2690624, EPI_ISL_2691003			
see above	MSK Microbiology Lab	MSK Microbiology Lab	Esther Babady, Tracy McMillen, Krupa Jani
EPI_ISL_2692207	Clinical Microbiology Laboratory, Faculty of Medicine, Universitas Indonesia	Clinical Microbiology Laboratory, Faculty of Medicine, Universitas Indonesia	Ibrahim F, Natasha A, Yasmon A, Fitrhiyah, Saharman YR, Karuniawati A, Sudarmono P.
EPI_ISL_2692886, EPI_ISL_2692887	Clinical Microbiology Lab RSPTN Universitas Hasanuddin	Clinical Microbiology Lab RSPTN Universitas Hasanuddin	Muhammad Nasrum Massi, Handayani Halik, Rizalinda Sjahril, Gita Vita Soraya, Triwibawa, Muhammad Yusuf Usman, Rufika Shari
EPI_ISL_2696943, EPI_ISL_2696944	All India Institute of Medical Sciences Delhi Hospital	Virology Laboratory, AIIMS Delhi	Aashish Choudhary, Sumedha Bagga, Lata Rani, Dibyabhaba Pradhan, Pooja Pandey, Jyoti Jethani, Shivram Dhakad, Nazneen Arif, Deepankar Srigyan, Megha Brijwal, Lalit Dar, Manish Soneja, Rakesh Lodha, Puneet Kaur, Ritu Gupta, Subrata Sinha, Chitra Sarkar, Randeep Guleria
EPI_ISL_2710012, EPI_ISL_2710013, EPI_ISL_2710014, EPI_ISL_2710015, EPI_ISL_2710024, EPI_ISL_2710026	Ochsner Health	Bioinfoexperts, LLC	Judy Crabtree, Amy Feehan, Rebecca Rose, David J. Nolan, Tessa LaFleur, Julia-Garcia-Diaz, Lucio Miele, Susanna L. Lamers
EPI_ISL_2713080, EPI_ISL_2713081, EPI_ISL_2713082, EPI_ISL_2713084, EPI_ISL_2713085, EPI_ISL_2713086, EPI_ISL_2713087, EPI_ISL_2713089, EPI_ISL_2713090, EPI_ISL_2713092, EPI_ISL_2713094, EPI_ISL_2713098, EPI_ISL_2713099, EPI_ISL_2713114, EPI_ISL_2713115, EPI_ISL_2713120			
see above	Department of Health Technology and Informatics, The Hong Kong Polytechnic University	Department of Health Technology and Informatics, The Hong Kong Polytechnic University	Gilman Kit-Hang Siu, Lam-Kwong Lee, Kenneth Siu-Sing Leung, Jake Siu-Lun Leung, Wing-Hei Lo, Timothy Ting-Leung Ng, Chloe Toi-Mei Chan, Hoi-Ching Jim, Ivan Tak-Fai Wong, Kingsley King-Gee Tam, Hiu-Yin Lao, Denise Sze-Hang Wong, Alan Ka-Lun Wu, Miranda Chong-Yee Yau, Yvette Wai-Man Lai, Kitty Sau-Chun Fung, Sandy Ka-Yee Chau, Barry Kin-Chung Wong, Wing-Kin To, Kristine Luk, Alex Yat-Man Ho, Tak-Lun Que, Kam-Tong Yip, Wing Cheong Yam, David Ho-Keung Shum, Shea Ping Yip
EPI_ISL_2727895, EPI_ISL_2727896, EPI_ISL_2727897, EPI_ISL_2727898	All India Institute of Medical Sciences Delhi Hospital	Virology Laboratory, AIIMS Delhi	Aashish Choudhary, Sumedha Bagga, Lata Rani, Dibyabhaba Pradhan, Pooja Pandey, Jyoti Jethani, Shivram Dhakad, Nazneen Arif, Deepankar Srigyan, Megha Brijwal, Lalit Dar, Manish Soneja, Rakesh Lodha, Puneet Kaur, Ritu Gupta, Subrata Sinha, Chitra Sarkar, Randeep Guleria
EPI_ISL_2728580	Laboratorio de Infectologia y Virologia Molecular	Laboratory of Molecular Virology, School of Medicine, Pontificia Universidad Catolica de Chile	Catalina Pardo-Roa, Constanza Martinez-Valdevenito, Jennifer Angulo, Tamara Garcia-Salum, Estefany Poblete, Maria Jose Avendano, Leonardo I. Almonacid, Ana Maria Contreras, Carlos Palma, Jorge Levican, Eileen Serrano, Constanza Maldonado, M. Belen Leyton, Erick Salinas, Andres E. Munoz-Marcos, Francisco Melo, Marcela Ferres, Rafael A. Medina
EPI_ISL_2756453	Instituto Adolfo Lutz Central	Instituto Adolfo Lutz, Interdiciplinary Procedures Center, Strategic Laboratory	Claudio Tavares Sacchi, Claudia Regina Gonçalves, Erica Valessa Ramos Gomes, Karoline Rodrigues Campos, Caio Vinicius Dias Lopes, Leonardo Jose Tadeu de Araujo
EPI_ISL_2779355, EPI_ISL_2779356, EPI_ISL_2779357, EPI_ISL_2779358, EPI_ISL_2779359, EPI_ISL_2779360, EPI_ISL_2779361, EPI_ISL_2779362, EPI_ISL_2779363, EPI_ISL_2779364	KEMRI/DEID Surveillance Site (Mtongwe Navy Base Hospital)	USAMRD-A, Basic Science Laboratory	Gathii Kimita, Josphat Nyataya, Esther Omuseni, Faith Sigei, Alan Lemtudo, Eric Muthanje, Brian Andika, Rehema Liyai, Rachel Githii, Clement Masakwe, Stephen Ochola, George Awinda, Carol Kifude, Beth Mutai, John Waitumbi
EPI_ISL_2779365, EPI_ISL_2779366, EPI_ISL_2779367, EPI_ISL_2779368, EPI_ISL_2779369, EPI_ISL_2779370	Jaramogi Oginga Odinga Teaching and Referral Hospital	USAMRD-A, Basic Science Laboratory	Gathii Kimita, Josphat Nyataya, Esther Omuseni, Faith Sigei, Alan Lemtudo, Eric Muthanje, Brian Andika, Rehema Liyai, Rachel Githii, Clement Masakwe, Stephen Ochola, George Awinda, Carol Kifude, Beth Mutai, John Waitumbi
EPI_ISL_2779374, EPI_ISL_2779375	KEMRI/DEID Surveillance Site (Lamu County Hospital)	USAMRD-A, Basic Science Laboratory	Gathii Kimita, Josphat Nyataya, Esther Omuseni, Faith Sigei, Alan Lemtudo, Eric Muthanje, Brian Andika, Rehema Liyai, Rachel Githii, Clement Masakwe, Stephen Ochola, George Awinda, Carol Kifude, Beth Mutai, John Waitumbi
EPI_ISL_2779382, EPI_ISL_2779386	Jaramogi Oginga Odinga Teaching and Referral Hospital	USAMRD-A, Basic Science Laboratory	Gathii Kimita, Josphat Nyataya, Esther Omuseni, Faith Sigei, Alan Lemtudo, Eric Muthanje, Brian Andika, Rehema Liyai, Rachel Githii, Clement Masakwe, Stephen Ochola, George Awinda, Carol Kifude, Beth Mutai, John Waitumbi
EPI_ISL_2779387, EPI_ISL_2779396, EPI_ISL_2779397	Busia County Referral Hospital	USAMRD-A, Basic Science Laboratory	Gathii Kimita, Josphat Nyataya, Esther Omuseni, Faith Sigei, Alan Lemtudo, Eric Muthanje, Brian Andika, Rehema Liyai, Rachel Githii, Clement Masakwe, Stephen Ochola, George Awinda, Carol Kifude, Beth Mutai, John Waitumbi
EPI_ISL_2779419	Port Health Clinic, Busia border point	USAMRD-A, Basic Science Laboratory	Gathii Kimita, Josphat Nyataya, Esther Omuseni, Faith Sigei, Alan Lemtudo, Eric Muthanje, Brian Andika, Rehema Liyai, Rachel Githii, Clement Masakwe, Stephen Ochola, George Awinda, Carol Kifude, Beth Mutai, John Waitumbi
EPI_ISL_2779422, EPI_ISL_2779423	GK Prison Busia (Matayo)Matayos Prison	USAMRD-A, Basic Science Laboratory	Gathii Kimita, Josphat Nyataya, Esther Omuseni, Faith Sigei, Alan Lemtudo, Eric Muthanje, Brian Andika, Rehema Liyai, Rachel Githii, Clement Masakwe, Stephen Ochola, George Awinda, Carol Kifude, Beth Mutai, John Waitumbi
EPI_ISL_2779456, EPI_ISL_2779457, EPI_ISL_2779458, EPI_ISL_2779459, EPI_ISL_2779460, EPI_ISL_2779461, EPI_ISL_2779463, EPI_ISL_2779464, EPI_ISL_2779465	KCRH KEMRI/DEID Surveillance Site	USAMRD-A, Basic Science Laboratory	Gathii Kimita, Josphat Nyataya, Esther Omuseni, Faith Sigei, Alan Lemtudo, Eric Muthanje, Brian Andika, Rehema Liyai, Rachel Githii, Clement Masakwe, Stephen Ochola, George Awinda, Carol Kifude, Beth Mutai, John Waitumbi
EPI_ISL_2779477, EPI_ISL_2779478, EPI_ISL_2779479, EPI_ISL_2779480, EPI_ISL_2779491	MOH-K	USAMRD-A, Basic Science Laboratory	Gathii Kimita, Josphat Nyataya, Esther Omuseni, Faith Sigei, Alan Lemtudo, Eric Muthanje, Brian Andika, Rehema Liyai, Rachel Githii, Clement Masakwe, Stephen Ochola, George Awinda, Carol Kifude, Beth Mutai, John Waitumbi
EPI_ISL_2779493	Port Health Clinic, Malaba border point	USAMRD-A, Basic Science Laboratory	Gathii Kimita, Josphat Nyataya, Esther Omuseni, Faith Sigei, Alan Lemtudo, Eric Muthanje, Brian Andika, Rehema Liyai, Rachel Githii, Clement Masakwe, Stephen Ochola, George Awinda, Carol Kifude, Beth Mutai, John Waitumbi
EPI_ISL_2779526, EPI_ISL_2779527, EPI_ISL_2779528, EPI_ISL_2779529, EPI_ISL_2779530, EPI_ISL_2779531, EPI_ISL_2779532, EPI_ISL_2779533, EPI_ISL_2779534	MOH-Q	USAMRD-A, Basic Science Laboratory	Gathii Kimita, Josphat Nyataya, Esther Omuseni, Faith Sigei, Alan Lemtudo, Eric Muthanje, Brian Andika, Rehema Liyai, Rachel Githii, Clement Masakwe, Stephen Ochola, George Awinda, Carol Kifude, Beth Mutai, John Waitumbi
EPI_ISL_2779535	PSTC	USAMRD-A, Basic Science Laboratory	Gathii Kimita, Josphat Nyataya, Esther Omuseni, Faith Sigei, Alan Lemtudo, Eric Muthanje, Brian Andika, Rehema Liyai, Rachel Githii, Clement Masakwe, Stephen Ochola, George Awinda, Carol Kifude, Beth Mutai, John Waitumbi
EPI_ISL_2779544	KCRH KEMRI/DEID Surveillance Site	USAMRD-A, Basic Science Laboratory	Gathii Kimita, Josphat Nyataya, Esther Omuseni, Faith Sigei, Alan Lemtudo, Eric Muthanje, Brian Andika, Rehema Liyai, Rachel Githii, Clement Masakwe, Stephen Ochola, George Awinda, Carol Kifude, Beth Mutai, John Waitumbi
EPI_ISL_2779545	Port Health Clinic, Malaba border point	USAMRD-A, Basic Science Laboratory	Gathii Kimita, Josphat Nyataya, Esther Omuseni, Faith Sigei, Alan Lemtudo, Eric Muthanje, Brian Andika, Rehema Liyai, Rachel Githii, Clement Masakwe, Stephen Ochola, George Awinda, Carol Kifude, Beth Mutai, John Waitumbi
EPI_ISL_2779546, EPI_ISL_2779547, EPI_ISL_2779548	KCRH KEMRI/DEID Surveillance Site	USAMRD-A, Basic Science Laboratory	Gathii Kimita, Josphat Nyataya, Esther Omuseni, Faith Sigei, Alan Lemtudo, Eric Muthanje, Brian Andika, Rehema Liyai, Rachel Githii, Clement Masakwe, Stephen Ochola, George Awinda, Carol Kifude, Beth Mutai, John Waitumbi
EPI_ISL_2789820, EPI_ISL_2789821, EPI_ISL_2789822	Centre Hospitalier Universitaire Clermont-Ferrand	CHU Clermont-Ferrand, service de virologie	Bisseux Maxime, Mirand Audrey, Combes Patricia, Henquell Cécile
EPI_ISL_2789838	Centre Hospitalier RIOM	CHU Clermont-Ferrand, service de virologie	Bisseux Maxime, Mirand Audrey, Combes Patricia, Henquell Cécile
EPI_ISL_2789839, EPI_ISL_2789840, EPI_ISL_2789841, EPI_ISL_2789842, EPI_ISL_2789843, EPI_ISL_2789844, EPI_ISL_2789845, EPI_ISL_2789846, EPI_ISL_2789847, EPI_ISL_2789848, EPI_ISL_2789849, EPI_ISL_2789850, EPI_ISL_2789851, EPI_ISL_2789852, EPI_ISL_2789853, EPI_ISL_2789854, EPI_ISL_2789855, EPI_ISL_2789856, EPI_ISL_2789857, EPI_ISL_2789858, EPI_ISL_2789859, EPI_ISL_2789860, EPI_ISL_2789861, EPI_ISL_2789862, EPI_ISL_2789863, EPI_ISL_2789864, EPI_ISL_2789865, EPI_ISL_2789866, EPI_ISL_2789867, EPI_ISL_2789868, EPI_ISL_2789869, EPI_ISL_2789870, EPI_ISL_2789871, EPI_ISL_2789872, EPI_ISL_2789873, EPI_ISL_2789874, EPI_ISL_2789875			
see above	Centre Hospitalier Universitaire Clermont-Ferrand	CHU Clermont-Ferrand, service de virologie	Bisseux Maxime, Mirand Audrey, Combes Patricia, Henquell Cécile
EPI_ISL_2790860, EPI_ISL_2790861	Hospital	National Reference Center for Viruses of Respiratory Infections, Institut Pasteur, Paris	Marion Barbet, Sylvie Behillil, Méline Bizard, Angela Brisebarre, Camille Capel, Vincent Enouf, Louise Lefrançois, Frédéric Lemoine, Christophe Malabat, Corinne Maufrais, Emmanuelle Permal, Etienne Simon-Lorière, Maud Vanpeene, Sylvie Van der Werf ,Pascale Martres

EPI_ISL_2790873, EPI_ISL_2790874, EPI_ISL_2790875, EPI_ISL_2790876, EPI_ISL_2790877, EPI_ISL_2790878, EPI_ISL_2790879, EPI_ISL_2790880, EPI_ISL_2790883, EPI_ISL_2790884, EPI_ISL_2790885, EPI_ISL_2790886, EPI_ISL_2790887			
see above	Labo Analyses Med	National Reference Center for Viruses of Respiratory Infections, Institut Pasteur, Paris	Marion Barbet, Sylvie Behillil, Méline Bizard, Angela Brisebarre, Camille Capel, Vincent Enouf, Louise Lefrançois, Frédéric Lemoine, Christophe Malabat, Corinne Maufrais, Emmanuelle Permal, Etienne Simon-Lorière, Maud Vanpeene, Sylvie Van der Werf ,StéPhane Romand
EPI_ISL_2790888	Medecine sentinel network	National Reference Center for Viruses of Respiratory Infections, Institut Pasteur, Paris	Marion Barbet, Sylvie Behillil, Méline Bizard, Angela Brisebarre, Camille Capel, Vincent Enouf, Louise Lefrançois, Frédéric Lemoine, Christophe Malabat, Corinne Maufrais, Emmanuelle Permal, Etienne Simon-Lorière, Maud Vanpeene, Sylvie Van der Werf ,Anne Garrigues
EPI_ISL_2790902, EPI_ISL_2790903	Hospital	National Reference Center for Viruses of Respiratory Infections, Institut Pasteur, Paris	Marion Barbet, Sylvie Behillil, Méline Bizard, Angela Brisebarre, Camille Capel, Vincent Enouf, Louise Lefrançois, Frédéric Lemoine, Christophe Malabat, Corinne Maufrais, Emmanuelle Permal, Etienne Simon-Lorière, Maud Vanpeene, Sylvie Van der Werf ,GisèLe Lagathu
EPI_ISL_2790905	Medecine sentinel network	National Reference Center for Viruses of Respiratory Infections, Institut Pasteur, Paris	Marion Barbet, Sylvie Behillil, Méline Bizard, Angela Brisebarre, Camille Capel, Vincent Enouf, Louise Lefrançois, Frédéric Lemoine, Christophe Malabat, Corinne Maufrais, Emmanuelle Permal, Etienne Simon-Lorière, Maud Vanpeene, Sylvie Van der Werf ,Sophie Langlais
EPI_ISL_2795658	LESP Hidalgo	LESP Hidalgo GENE2LIFE	HERRERA-GOMEZ CONNIE ALEJANDRA; CASTILLO-ORTEGA LAURA SOFIA; GARCIA-REYES MELITO; VIGUERAS-MORALES YAJAIRA SULIM; GARCIA-GONZALEZ OCTAVIO; VILLAFUENTES-TELLEZ HECTOR ALBERTO
EPI_ISL_2820471, EPI_ISL_2820473	The Hong Kong Polytechnic University - Molecular Diagnostic Laboratory	The Hong Kong Polytechnic University - Molecular Diagnostic Laboratory	Hin Fung Tsang, Allen Chi Shing Yu, Aldrin Kay Yuen Yim, Wai Ming Stanley Leung, Jiachi Chiou, Yin Kwan Evelyn Wong, Dominic Ngai Chong Tsang, Peter and SO, Samuel Chun Lap Lo,Wing Tak Wong, Sze Chuen Cesar Wong
EPI_ISL_2835003, EPI_ISL_2835004	Laboratoire Central Analyse Médicale mohammedia	Functional Genomic Platform_UATRS_CNRST_Rabat	Hajjaji Mohammed, Oumaima Ait Si Mohammed, Mouhcine Gardoul, Marouane Melloul, Sanaa Alaoui-Amine, Mouhssine Hemlali, Taha Chouati Omar Askander, Belayachi Lamiae, Hassan Aguenaou, Hasnae Benkirane, Jaafar Heikel, Amina Barkat, Nadia Touil, Elmostafa El fahime
EPI_ISL_2835490, EPI_ISL_2835491, EPI_ISL_2835492, EPI_ISL_2835493, EPI_ISL_2835494, EPI_ISL_2835495, EPI_ISL_2835496, EPI_ISL_2835497, EPI_ISL_2835499, EPI_ISL_2835500, EPI_ISL_2835502, EPI_ISL_2835503, EPI_ISL_2835504, EPI_ISL_2835505, EPI_ISL_2835506, EPI_ISL_2835507, EPI_ISL_2835508, EPI_ISL_2835509, EPI_ISL_2835510			
see above	Instituto de Diagnostico y Referencia Epidemiologicos (InDRE)	Andersen lab at Scripps Research	SEARCH Alliance with Gisela Barrera Badillo, Jose Ernesto Ramirez Gonzalez, Maria Natividad Cruz Ortiz, Nervain Benjamin Contreras Gonzalez, Tatiana Ernestina Nunez Garcia, Dayanira Sarith Arellano Suarez, Irma Lopez Martinez, Rita Flores Leon, Beatriz Olivares Flores
EPI_ISL_2835846, EPI_ISL_2835847, EPI_ISL_2835848	Hospital	National Reference Center for Viruses of Respiratory Infections, Institut Pasteur, Paris	Marion Barbet, Sylvie Behillil, Méline Bizard, Angela Brisebarre, Camille Capel, Vincent Enouf, Louise Lefrançois, Frédéric Lemoine, Christophe Malabat, Corinne Maufrais, Emmanuelle Permal, Etienne Simon-Lorière, Maud Vanpeene, Sylvie Van der Werf ,Evelyne Schvoerer
EPI_ISL_2836970, EPI_ISL_2836971, EPI_ISL_2837247, EPI_ISL_2838053, EPI_ISL_2838054, EPI_ISL_2838055, EPI_ISL_2838353	Department of Microbiology and Virology, School of Medicine, Mazandaran University of Medical Sciences, Sari, Iran	Genetics Research Center, University of Social Welfare and Rehabilitation Sciences	Zohreh Fattahi, Marzieh Mohseni, Kimia Kahrizi, Mohammad Reza Haghshenas, Hossein Najmabadi.
EPI_ISL_2839238, EPI_ISL_2839240, EPI_ISL_2839243, EPI_ISL_2839245, EPI_ISL_2839247, EPI_ISL_2839249, EPI_ISL_2839251, EPI_ISL_2839255, EPI_ISL_2839259, EPI_ISL_2839261, EPI_ISL_2839268, EPI_ISL_2839270, EPI_ISL_2839272, EPI_ISL_2839274, EPI_ISL_2839276, EPI_ISL_2839278, EPI_ISL_2839280, EPI_ISL_2839282, EPI_ISL_2839284, EPI_ISL_2839286, EPI_ISL_2839287, EPI_ISL_2839289, EPI_ISL_2839291, EPI_ISL_2839299, EPI_ISL_2839301, EPI_ISL_2839303, EPI_ISL_2839305, EPI_ISL_2839309, EPI_ISL_2839311, EPI_ISL_2839315, EPI_ISL_2839319, EPI_ISL_2839321, EPI_ISL_2839323, EPI_ISL_2839325, EPI_ISL_2839327, EPI_ISL_2839329, EPI_ISL_2839331, EPI_ISL_2839335, EPI_ISL_2839336, EPI_ISL_2839340, EPI_ISL_2839342, EPI_ISL_2839344, EPI_ISL_2839346, EPI_ISL_2839348, EPI_ISL_2839352, EPI_ISL_2839353, EPI_ISL_2839359, EPI_ISL_2839361, EPI_ISL_2839367, EPI_ISL_2839371, EPI_ISL_2839373, EPI_ISL_2839377, EPI_ISL_2839380, EPI_ISL_2839382			
see above	Instituto Nacional de Enfermedades Respiratorias (INER)	Centro de Investigación en Enfermedades Infecciosas (CIENI), Instituto Nacional de Enfermedades Respiratorias (INER)	Consorcio Mexicano de Vigilancia Genómica (CoViGen-Mex). Authors (in alphabetical order): Julio Elias Alvarado-Yaah, Carlos F. Arias, Santiago Ávila-Ríos, Eduardo Becerril-Vargas, Víctor Hugo Borja-Aburto, Celia Boukadida, Cristóbal Cháidez-Quiróz, Juan Bautista Chale-Dzul, Ricardo Ciria Merce, Andreu Comas-García, Célida Duque Molina, Julissa Enciso-Ibarra, José Antonio Enciso-Moreno, Gloria Elena Espinosa-Ayala, Fernando Fontove-Herrera, Daniel Fregoso-Rueda, Victor Eduardo Garcia-Arias, Alejandra Garcia-Gasca, Bruno Gomez-Gil, Jean Pierre González, Irvin González-López, Concepción Grajales-Muñiz, Ricardo Grande, Rosa María Gutierrez Rios, Alejandra Hernández-Terán, Alfredo Herrera-Estrella, Carla Ivón Herrera-Najera, Pavel Isa, Daniel Lira Morales, Susana Lopez, Antonio Loza Román, Brenda Irasema Maldonado-Meza, Bernardo Martínez-Miguel, José Arturo Martínez-Orozco, Célida Martínez- Rodríguez, Margarita Matías-Florentino, Fidencio Mejía-Nepomuceno, María Guadalupe de Jesús Mireles-Rivera, Gloria María Molina-Salinas, Hector Montoya-Fuentes, Mario Müjica-Sánchez, José Esteban Muñoz-Medina, José de Jesús Nuñez-Contreras, Alicia Ocaña-Mondragón, Luis Alberto Ochoa-Carrera, Hector Esteban Paz-Juárez, Marissa Pérez-García, Francisco Pulido, Helen Haydee Fernanda Ramírez-Plascencia, Jorge Salas-Hernández, Angel Gustavo Salas-Lais, Alejandro Sanchez-Flores, Clara Esperanza Santacruz-Tinoco, María Guadalupe Santiago-Mauricio, Selene Zárate, Nelly Sélem-Mojica, Blanca Taboada, Kathia Elizabeth Tapia-Diaz, Gloria Vazquez, Joel Armando Vázquez-Pérez.
EPI_ISL_2860777, EPI_ISL_2860778, EPI_ISL_2860779, EPI_ISL_2860780, EPI_ISL_2860782, EPI_ISL_2860783, EPI_ISL_2860787, EPI_ISL_2860788	Department of Health Technology and Informatics, The Hong Kong Polytechnic University	Department of Health Technology and Informatics, The Hong Kong Polytechnic University	Gilman Kit-Hang Siu, Lam-Kwong Lee, Kenneth Siu-Sing Leung, Jake Siu-Lun Leung, Wing-Hei Lo, Timothy Ting-Leung Ng, Chloe Toi-Mei Chan, Hoi-Ching Jim, Ivan Tak-Fai Wong, Kingsley King-Gee Tam, Hiu-Yin Lao, Denise Sze-Hang Wong, Alan Ka-Lun Wu, Miranda Chong-Yee Yau, Yvette Wai-Man Lai, Kitty Sau-Chun Fung, Sandy Ka-Yee Chau, Barry Kin-Chung Wong, Wing-Kin To, Kristine Luk, Alex Yat-Man Ho, Tak-Lun Que, Kam-Tong Yip, Wing Cheong Yam, David Ho-Keung Shum, Shea Ping Yip
EPI_ISL_2864604, EPI_ISL_2864606	Ga East Hospital	UHAS COVID-19 Lab	Kwabena O. Duedu, Jones Gyamfi, Reuben Ayivor-Djanie, John O. Gyapong and the UHAS COVID-19 Lab Team
EPI_ISL_568577	The National Institute of Public Health	State Veterinary Institute Prague	Nagy, A; Jirincova, H; Novakova, L; Trnka,D; Vecerova, J
EPI_ISL_574431	Hospital IESS Babahoyo	Institute of Microbiology, Universidad San Francisco de Quito	Belén Prado-Vivar, Sully Márquez, Juan José Guadalupe, Monica Becerra-Wong, Fernanda Zurita, Bernardo Gutiérrez, Francisco Cordova, Ninfa Henriquez, Killen Briones-Zamora, Killen Briones-Claudette, Verónica Barragán, Patricio Rojas-Silva, Gabriel Trueba, Michelle Grunauer, Paúl Cárdenas
EPI_ISL_576383	RSUD Budi Rahayu Kota Magelang	Genetics Working Group (Pokja Genetik) Faculty of Medicine, Public Health and Nursing Universitas Gadjah Mada (FK-KMK UGM); Disease Investigation Center Wates Ministry of Agriculture Indonesia; Department of Microbiology FK-KMK UGM; Laboratorium Diagnostik Yayasan Tahija World Mosquito Program (WMP) Yogyakarta Center for Tropical Medicine FK-KMK UGM; Integrated Research Center FK-KMK UGM; Department of Computer Science and Electronics FMIPA UGM; Balai Besar Teknik Kesehatan Lingkungan dan Pengendalian Penyakit (BBTKLPP) Yogyakarta	Gunadi, Hendra Wibawa, Marcellus, Mohamad S. Hakim, Edwin W. Daniwijaya, Ludhang P. Rizki, Endah Supriyati, Eggi Arguni, Titik Nuryastuti, Tri Wibawa, Dwi AA Nugrahaningsih, Afiahayati, Siswanto, Kristy Iskandar, Nungki Anggorowati, Irene, Indaryati, Havid Setyawan, Ari Meliyanti, Merliana Sari Situmeang, Audric Kenny Tedja, Aditya Rifqi Fauzi
EPI_ISL_577630	The National Institute of Public Health	State Veterinary Institute Prague	Nagy,A;Jirincova,H;Novakova,L;Trnka,D;Vecerova,J
EPI_ISL_582128, EPI_ISL_582129, EPI_ISL_582130, EPI_ISL_582131, EPI_ISL_582132	Antwerp University Hospital	Institute of Tropical Medicine	Philippe Selhorst, Colin Anthony
EPI_ISL_582659, EPI_ISL_582662, EPI_ISL_582663, EPI_ISL_582674, EPI_ISL_582675, EPI_ISL_582679, EPI_ISL_582680, EPI_ISL_582681, EPI_ISL_582683, EPI_ISL_582688	Sheikh Khalifa Medical City	Molecular/Surveillance lab Sheikh Khalifa Medical City	Amirtharaj Francis, Sajeed Abdul, Hala Imambaccus, Sahar Almarzooqi, Hiba Saud, Stefan Weber
EPI_ISL_583893	Singapore General Hospital	Department of Microbiology	Nurdyana Abdul Rahman, Kun Lee Lim, Chenhao Li, Sui Sin Goh, Kenneth Xin Long Chan, Kian Sing Chan, Lynette Oon, Kern Rei Chng, Niranjan Nagarajan, Karrie Ko
EPI_ISL_584082	The National Institute of Public Health	State Veterinary Institute Prague	Nagy,A;Jirincova,H;Novakova,L;Trnka,D;Vecerova,J
[truncated: 867,298 more chars]
